# Evidence update on the respiratory health effects of vaping e-cigarettes: A systematic review and meta-analysis

**DOI:** 10.18332/tid/209954

**Published:** 2025-11-19

**Authors:** Anasua Kundu, Anna Feore, Nada Abu-Zarour, Sherald Sanchez, Megan Sutton, Kyran Sachdeva, Siddharth Seth, Robert Schwartz, Michael Chaiton

**Affiliations:** 1 Institute of Medical Science, University of Toronto, Toronto, Canada; 2 School of Medicine, University College Cork, Cork, Ireland; 3 Peel Public Health, Toronto, Canada; 4 Department of Medical Sciences, Western University, Toronto, Canada; 5 Department of Health Sciences, Queen’s University, Toronto, Canada; 6 Centre for Addiction and Mental Health, Toronto, Canada; 7 Dalla Lana School of Public Health, University of Toronto, Canada

**Keywords:** electronic cigarette, respiratory, lung, asthma, COPD

## Abstract

**INTRODUCTION:**

In this review, we aimed to explore whether nicotine e-cigarette or vaping product use impact respiratory health.

**METHODS:**

We searched CINAHL, Embase, MEDLINE, PsycINFO, PubMed and Cochrane library databases initially in January 2023 and updated the search in January 2024. We included peer-reviewed human, animal, cell/*in vitro* original studies published between July 2021 and December 2023 but excluded qualitative studies. Three types of e-cigarette exposure were examined: acute, short-to-medium term, and long-term.

**RESULTS:**

We included 119 studies in the main analysis, and 5 in meta-analysis. Over half of the studies had low risk of bias. Non-smoker current vapers had higher incident risk of respiratory symptoms (relative risk, RR=1.90; 95% CI: 1.28–2.83) but statistically non-significant risk of chronic obstructive pulmonary disease (COPD) (RR=2.53; 95% CI: 0.96–6.67) compared to never users. They also had lower incident risk of respiratory symptoms compared to non-vaper current smokers (RR=0.75; 95% CI: 0.64–0.89) and dual users (dual use vs vaping, RR=1.26; 95% CI: 1.03–1.55). Dual users had higher risk of incidence of respiratory symptoms and prevalence of COPD compared to never users (RR=2.53; 95% CI: 1.44–4.45 and RR=3.86; 95% CI: 1.49–10.02, respectively), and the risk was statistically similar to non-vaper current smokers (RR=0.97; 95% CI: 0.84–1.14 and RR=1.15; 95% CI: 1.00–1.33, respectively). All meta-analysis findings were of ‘very low’ to ‘low’ certainty evidence. Of the studies not included in meta-analysis, we found ‘moderate’ certainty evidence of higher risk of respiratory symptoms, COPD, asthma, lung inflammation and damage in non-smoker current vapers compared to non-users, inconsistent findings on the risk of COVID-19 and other respiratory infections, and no significant association with e-cigarette associated lung injury.

**CONCLUSIONS:**

E-cigarettes are associated with harms to the respiratory system. Further longitudinal research with special attention to measuring effects in different e-cigarette user populations are warranted.

## INTRODUCTION

In recent years, the rising popularity of e-cigarettes has sparked the need for more research on the health impacts of vaping. In 2022, 20% of Canadian young adults aged 20–24 years reported having vaped in the past 30 days, which is up from 17% in 2021 and 13% in 2020^1^. Those aged ≥25 years were less likely to have vaped in the past 30 days, given that only 15% people of this age group reported ever using e-cigarettes in their lifetime^1^. With an expanding market and rising prevalence of e-cigarette use among young people, understanding the potential risk is crucial.

E-cigarettes have been found to deliver fine and ultrafine particles in flavored e-liquid, and trace metals from the heated coil into the lungs^2-6^. These toxicants, in addition to the nicotine component, have potentially damaging effects on the respiratory system^5,7^. In recent years, several reviews have been published which showed that e-cigarette use might increase risk of several respiratory conditions including asthma, chronic obstructive pulmonary diseases (COPD), Coronavirus Disease 2019 (COVID-19), and pulmonary inflammation^2,5,8,9^. However, these reviews were often limited by lack of meta-analysis, focusing on a specific type of study design, biomarkers, or toxicological analysis of e-cigarette contents. As the research on the health effects of vaping are rapidly emerging, there is a need for conducting an updated review. Our review sought to amalgamate the available evidence to provide insights into both the immediate and long-term impacts of e-cigarette exposure on respiratory health. The research question for our review was: ‘Does nicotine e-cigarette or vaping product use (active and passive/second hand use) impact respiratory health?’. We also explored whether these impacts varied in magnitude by different population subgroups by vaping and smoking status and sociodemographic conditions such as age groups, sex, gender, sexual orientation, race, ethnicity, indigenous identity, pregnant/postpartum, education level, individual or household income, employment status, and occupation.

## METHODS

This review was conducted as a part of the ‘Vaping and Electronic Cigarette Toxicity Overview and Recommendations (VECTOR)’ project aimed to evaluate various health risks (i.e. cardiovascular, respiratory, cancer, dependence) of vaping e-cigarettes in different population groups based on their vaping and smoking status^10-12^. The protocol of this project was prospectively registered on PROSPERO (CRD42023385632) and we followed the Preferred Reporting Items for Systematic Reviews and Meta-Analyses (PRISMA) guideline^13^ and guideline for reporting animal evidence^14^.

### Information sources and search strategy

We searched the following databases: CINAHL, Embase, MEDLINE, PsycINFO, PubMed and Cochrane library. As the National Academics of Sciences, Engineering, and Medicine review, 2018^3^ and the McNeill et al.^2^ review included studies on respiratory health effects of e-cigarette use published since the inception of the databases up to June 2021, we limited our search for studies published between July 2021 and December 2023 to avoid duplication with those reviews. The literature search was conducted under the VECTOR project with an initial search done in 31 January 2023 and updating it in 2 January 2024. The detail database search strategies are presented as Supplementary file Material 1. The McNeill et al.^2^ review did not conduct any sociodemographic factor-based subgroup analysis, so we reviewed the 427 original studies that were included in that review to assess their eligibility for subgroup analysis. We did not conduct any manual or grey literature search for this review.

### Eligibility and study selection process

We included studies based on following inclusion criteria: 1) population – human, animal, and cell/*in vitro* (i.e. human or animal); 2) intervention/exposure – exposed to nicotine e-cigarettes (active or second hand); 3) comparators – exposed to either cigarettes, other tobacco products, cannabis vaping products, placebo or no exposure; 4) outcomes – any respiratory health effects; and 5) study designs – any peer-reviewed studies including observational and experimental studies except qualitative studies and literature reviews. Additional criteria were studies published in English or French language due to our expertise in these two languages and availability of most of the publications in English language. We excluded studies evaluating respiratory health effects resulting from cannabis vaping, heated tobacco products or other tobacco products use. As we have assessed risk of cancer separately in the VECTOR project, we excluded studies on lung cancer from this review, but stated the findings on lung cancer in another article^10^. Similarly studies on other health effects of e-cigarette use (i.e. cardiovascular, other cancers, vaping dependence)^10-12^ were excluded as part of the VECTOR project during the full-text screening process.

All search results collected from the electronic databases were imported to the Covidence workflow platform where duplicate articles were removed automatically. We also removed any duplicate articles which were missed by Covidence manually. Study selection process was conducted in the Covidence and full texts of each articles following title and abstract screening were uploaded there. At least two reviewers independently screened each title and abstract followed by full text reviews of the remaining articles in accordance with the inclusion and exclusion criteria. Disagreements were resolved by one reviewer upon discussion with or guidance from other reviewers.

### Data extraction and management

A custom-made data extraction form was developed which mainly included general characteristics of the included studies (author and year, funding source, conflict of interest), population characteristics (sample size and demographics), type and duration of exposure, intervention/exposure characteristics (definition and sample size of comparison groups), health condition/outcome assessed, reversibility of health effects, study findings, subgroup types, sample size of the subgroups and findings, and risk of bias ratings for each study. While one reviewer conducted the data extraction, another reviewer checked for accuracy of the extracted data. Disagreements were resolved by discussion between the reviewers. In accordance with the McNeill et al.^2^ review, we assessed the health effects of 3 different types of e-cigarette exposure: acute (one-off exposure to 7 days), short- to medium-term (8 days to 12 months), and long-term exposure (more than 12 months). No exposure type was determined for cross-sectional studies due to design limitations. For consistency, we categorized the comparison groups according to their frequency of exposure. For example, using the definition of current use as using the respective product in past 30 days, we categorized e-cigarette users as non-smoker current vapers, never smoker current vapers, former smoker current vapers, and dual users. Similarly, we established other categories like non-vaper current smokers, never vaper current smokers, former vaper current smokers, non-users, and never users. If a study met the eligibility criteria for meta-analysis, we extracted the relevant sample size and event rates for each comparison groups. Data extraction sheets are presented as Supplementary file Tables S2–S4.

### Quality assessment

Two independent reviewers used the different risk of bias assessment tools for quality assessment of individual studies. Any disagreements between the reviewers were resolved by discussion. For non-randomized experimental studies and longitudinal observational studies, we used the Cochrane risk of bias tools – Risk of Bias in Non-randomized Studies of Interventions tool (ROBINS-I)^15^ and Risk of Bias in Non-randomized Studies- of Exposure (ROBINS-E) tool^16^, respectively, and rated the studies as having as ‘low’, ‘moderate’, ‘serious’ and ‘critical’ risk of bias. For cross-sectional, case reports and case series, we used the Joanna Briggs Institute (JBI) critical appraisal tool^17^ except for the studies that involved biomarker-based cross-sectional assessment where we used the BIOCROSS risk of bias tool^18^. Following the approach of previous research^19,20^, a study was considered to have a ‘low’, ‘moderate’ or ‘high’ risk of bias if the total score was ≥70%, 50–70%, <50%, respectively, in the JBI critical appraisal tools^17^ or 13–20, 7–12, and ≤6, respectively, in the BIOCROSS tool^18^. Additionally, we used the Office of Health assessment and Translation (OHAT) tool^21^ and the Systematic Review Center for Laboratory animal Experimentation (SYRCLE) tool^22^ for the cell/*in vitro* and animal studies, respectively. Studies were considered ‘low’, ‘moderate’, and ‘high’ risk of bias if the majority, half and minority criteria were met respectively^23^.

### Data synthesis

We conducted meta-analyses of mainly human observational studies to compare risk of incidence of respiratory symptoms and prevalence of COPD in non-smoker current vapers and dual users. We excluded any case studies or case series, studies that did not have any clear definition of the exposure received, and studies where outcome was assessed in current vapers instead of non-smoker current vapers. Cell/*in vitro* and animal studies were also excluded from meta-analysis due to wide heterogeneity between studies. We attempted to obtain missing data from two studies^24,25^ for inclusion in the meta-analysis but failed to get any response from the authors and eventually excluded these studies from meta-analysis. The detail reasons for exclusion in the meta-analysis, relevant extracted data, and reviewers’ information are provided in Supplementary file Table S4. We used random effects models for meta-analysis according to the Cochrane guidelines and calculated the risk ratio (RR) with 95% CI with p-value for binary outcomes^26^. We considered results statistically significant when p<0.05 or 95% CI did not cross the null value. Heterogeneity in the dataset was measured by τ^2^ and χ^2^ tests, and I^2^ statistic, and we used restricted maximum likelihood (REML)^27^ to measure heterogeneity in τ^2^. Heterogeneity in the dataset was determined as low, moderate and high, when the I^2^ statistic value was <25%, 25–50% and >50%, respectively^28^. Additionally, we used Doi plot and Luis Furuya Kanamuri (LFK index)^29^ for identifying small-study effects and publication bias. LFK index value of -1 to 1 was considered as no risk of publication bias, while LFK value of -1 to -2 or 1 to 2 as minor risk of publication bias and LFK value of <-2 or >2 as major risk of publication bias^29^. Due to the low number of studies (n=2) included in each meta-analysis, we could not conduct sensitivity analysis or subgroup analysis. We used R statistics (version 4.3.0) for the meta-analyses.

For the studies that were not included in meta-analysis, we followed synthesis without meta-analysis (SWiM)^30^ and narrative data synthesis approach^31^ to present our findings. Studies were grouped as mainly observational studies (i.e. longitudinal observational and cross-sectional studies) and experimental studies (i.e. human, cell/*in vitro*, and animal studies). Harvest plots^30^ were used to compare risk of different outcomes between various comparison groups (i.e. non-smoker current vapers, non-users, dual users, non-vaper current smokers). A ‘higher’ or ‘lower’ risk of an outcome was determined when the effect was statistically significant (p<0.05 or 95% CI did not cross the null value), otherwise it was considered as having ‘similar’ risk. As the studies included in the sociodemographic factor-based subgroup analysis did not meet the eligibility criteria for meta-analysis, we used narrative synthesis approach to present our findings with the exception of using harvest plots for demonstrating sex-based subgroup differences.

### Certainty assessment

We used Grading of Recommendations Assessment, Development and Evaluation (GRADE) approach^32^ for assessing certainty of our meta-analysis evidence and the Confidence in Evidence from Reviews of Qualitative Research (GRADE-CERQual) approach^33^ for evidence that were found mainly from the harvest plots. We avoided GRADE-CERQual assessment if the findings were based on only one study, and the risk of outcome was not assessed in non-smoker current vapers or dual users. For each finding, two independent reviewers conducted their assessment separately and provided an overall certainty of very low, low, moderate, or high. Any disagreement was resolved by discussion between reviewers.

## RESULTS

### Study selection

**Figure 1:**
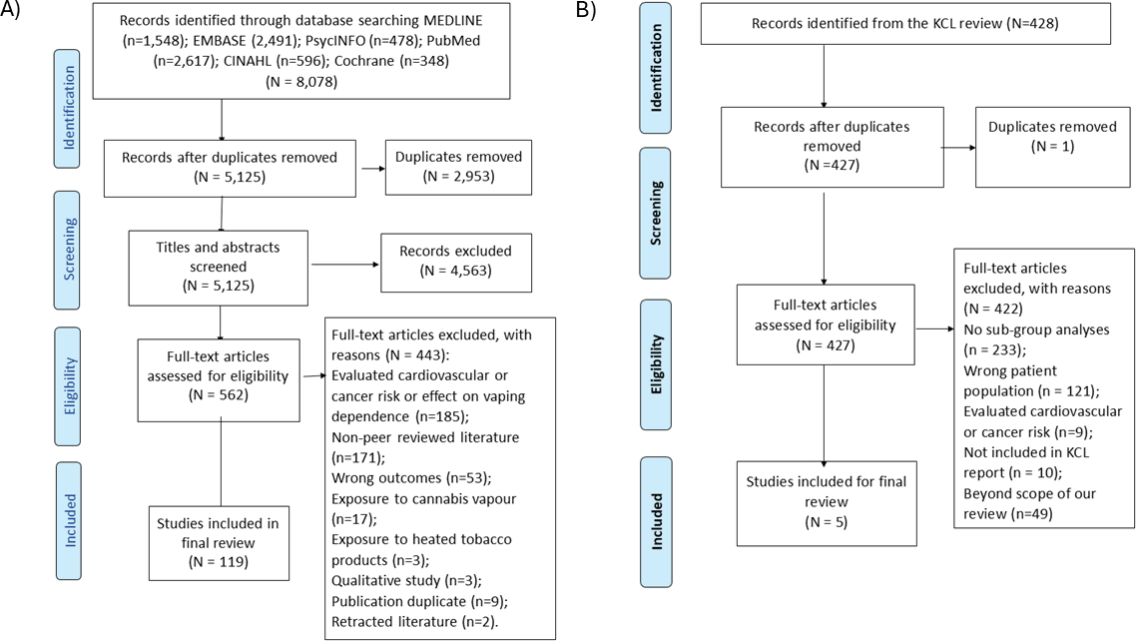
PRISMA flow diagram showing study selection process for: A) main analysis; and B) from the McNeill et al.^2^ review for the sociodemographic factor-based subgroup analysis

As part of the VECTOR systematic review, we retrieved a total of 8078 articles from the databases. After removing 2953 duplicates, we screened titles and abstract of 5125 articles, of which 562 articles were selected for full text screening. Following removal of 443 articles for various reasons (Figure 1), finally 119 articles^24,25,34-36, s37-150^ [Supplementary file Material 8, references 37–150] were selected for inclusion in this study. Additionally, from the 427 studies of the McNeill et al.^2^ review, following removal of 422 studies, we selected 5 studies^s151-155^ for including in the subgroup analysis (Figure 1).

### Study characteristics

Of the 119 studies^24,25,34-36, s37-150^ included in the main analysis, 26.1% (n=31) evaluated effects of acute exposure, 32.8% (n=39) assessed short-to-medium term exposure and 10.9% (n=13) examined long-term exposure (Table 1). Almost all of the acute exposures were examined by the human non-randomized experimental studies, cell/*in vitro* and animal experimental studies, whereas all longitudinal observational studies looked into short-to-medium term and long-term exposures. We categorized the outcomes into 8 main categories: respiratory symptoms, COPD, asthma, impact on lung function, lung inflammation and damage, COVID-19 and other respiratory infections, e-cigarette or vaping associated lung injury (EVALI) and other lung conditions, and lung development *in utero*; of which lung inflammation and damage is the most commonly investigated outcome (n=43; 36.1%). We did not find any study examining passive or secondhand exposure. A total of 18 studies were included in the subgroup analysis, of which 88.2% (n=15) were in sex, and 17.6% (n=3) were in age-based analysis (Table 1). Characteristics of the individual studies are presented in the Supplementary file Material 2 and 3.

**Table 1:** Summary statistics of included studies published between July 2021 and December 2023 examining respiratory effects of vaping e-cigarettes (N=119)

*Characteristics*	*Number of studies (%)*
**Outcomes/health condition(s)**	
Respiratory symptoms	10 (8.4)
COPD	18 (15.1)
Asthma	19 (16.0)
Impact on lung function	14 (11.8)
Lung inflammation and damage	43 (36.1)
COVID-19 and other respiratory infections	23 (19.3)
EVALI and other lung conditions	24 (20.2)
Lung development *in utero*	7 (5.9)
**Country**	
USA	75 (63.0)
Canada	5 (4.2)
European countries	16 (13.4)
Other	23 (19.3)
**Type of exposure**	
Acute	31 (26.1)
Short-to-medium	39 (32.8)
Long-term	13 (10.9)
**Study design**	
Non-randomized experimental	3 (2.5)
Longitudinal observational	12 (10.1)
Cross-sectional	29 (24.4)
Case reports/case series	24 (20.2)
*In vitro*/cell studies	14 (11.8)
Animal studies	38 (31.9)
**Participants** (human studies only, N=68)	
<100	34 (50)
100–1000	9 (13.2)
1001–10000	7 (10.3)
>10000	18 (26.5)
**Age of the participants** (years) (human studies only, N=68)	
≤18	17 (25)
>18	58 (85.3)
**Risk of bias**	
Low	62 (52.1)
Moderate/some concerns	31 (26)
High/serious/critical	26 (21.8)
**Association with tobacco companies**	
Yes	5 (4.2)
No	94 (79)
Not specified	20 (16.8)
**Subgroup analysis** (N=17)^a^	
Age	3 (17.6)
Sex	15 (88.2)
Race/ethnicity	1 (5.9)
Sexual orientation	1 (5.9)

a Subgroup analysis included studies from both this review (n=12) and the McNeill et al.^2^ review (n=5). EVALI: e-cigarette or vaping associated lung injury. COPD: chronic obstructive pulmonary disease.

### Quality assessment

Of the 119 studies, 52.1% (n=63) had low risk of bias (Table 1, Supplementary file Material 4). Of the 26 studies that had high risk of bias, one was a non-randomized experimental study examining effect on lung function^s100^, another was a longitudinal study examining incidence and prevalence of asthma^s44^, and others were cell/*in vitro* and animal studies^36, s38,41,42,47,56,59,60,64,73,83,88,101,108,111,118,119,125,128,129,132,138,148,149^. Five studies were either funded by or had any association with tobacco companies^s47,109,123,138,144^, while the association status could not be determined for 20 studies^s43,51,53,68,69,74,80,84,85,93,105,114,120,125,126,130,132-134,136^.

### Respiratory symptoms

**Figure 2:**
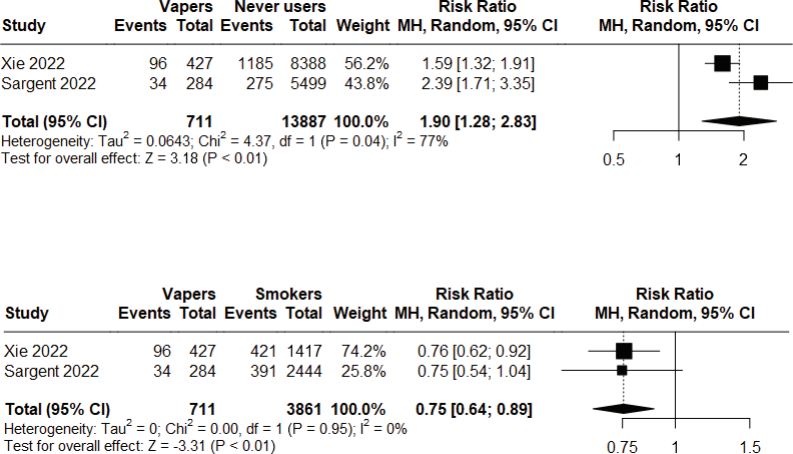
Meta-analysis comparing incidence of respiratory symptoms among non-smoker current vapers vs never users (top graph) and non-smoker current vapers vs non-vaper current smokers (bottom graph) in observational studies

**Figure 3:**
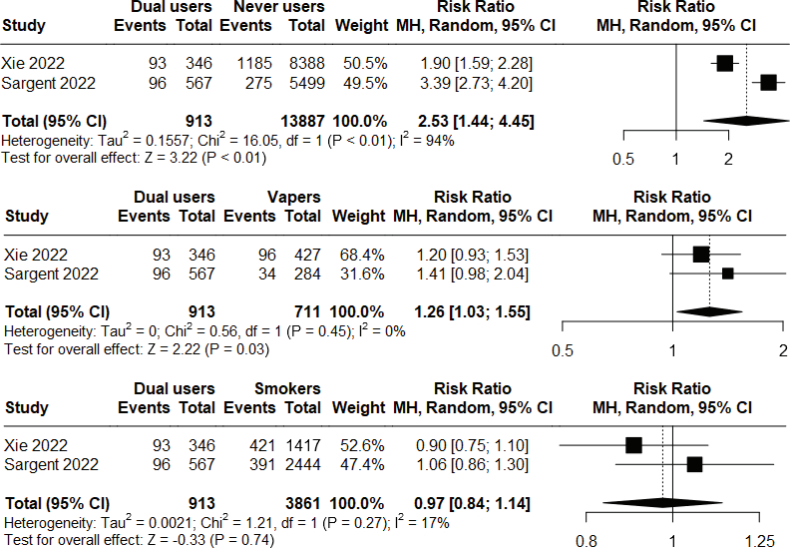
Meta-analysis comparing incidence of respiratory symptoms among dual users vs never users (top graph), dual users vs non-smoker current vapers (middle graph), and dual users vs non-vaper current smokers (bottom graph) in observational studies

Two longitudinal observational studies^s116,143^ of low to moderate risk of bias were included in a meta-analysis to assess the incident risk of respiratory symptoms (cough, phlegm, wheezing, or shortness of breath) following at least short-to-medium term exposure. Non-smoker current vapers had significantly higher incident risk compared to never users (RR=1.90; 95% CI: 1.28–2.83; n=14598), but significantly lower incident risk compared to non-vaper current smokers (RR=0.75; 95% CI: 0.64–0.89; n=4572). Heterogeneity was high (I^2^=77%) in the first comparison, but low (I^2^=0%) in the second comparison (Figure 2). Additionally, we found that dual users had significantly higher incidence risk compared to both never users (RR=2.53; 95% CI: 1.44–4.45; n=14800) and non-smoker current vapers (RR=1.26; 95% CI: 1.03–1.55; n=1624), but the risk was not statistically significant when compared with non-vaper current smokers (RR=0.97; 95% CI: 0.84–1.14; n=4774). Heterogeneity was high (I^2^=94%) in the first comparison, but low (I^2^=0%, I^2^=17%, respectively) in the latter two comparisons (Figure 3). Major risk of publication bias was detected in the Doi plots (LFK index 2.98, -2.84, 2.02, 2.49, respectively) for the comparison of: 1) non-smoker current vapers with never users and non-vaper current smokers; and 2) comparison of dual users with never users and non-smoker current vapers indicating small study effects. The comparison between dual users and non-vaper current smokers had minor risk of publication bias (LFK index 1.79) (Supplementary file Material 5).

**Figure 4:**
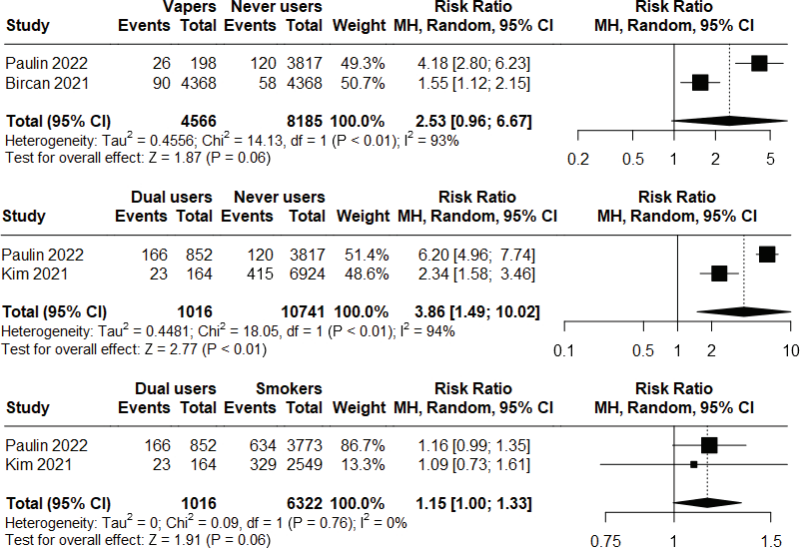
Meta-analysis comparing prevalence of COPD among non-smoker current vapers vs never users (top graph), dual users vs never users (middle graph) and dual users vs non-vaper current smokers (bottom graph) in observational studies

Among the other observational studies that were not included in the meta-analysis^s45,49,50,52,86,96,123,137^, 2 studiess^50,96^ detected higher risk of respiratory symptoms in non-smoker current vapers compared to non-users (Figure 4). One of these 2 studies was a longitudinal observational study assessing the incident risk of respiratory symptoms following long-term exposures^96^. The same study found higher risk in dual users compared to non-users^s96^, while another study found lower risk in non-smoker current vapers compared to non-vaper current smokers^s123^. Rest of the studies were too heterogenous in terms of their population and findings^s45,49,52,86,137^, limiting comparability

### COPD

One longitudinal observational study and two cross-sectional studies^s43,81,106^ of low risk of bias were included in meta-analyses to compare prevalence of COPD. Non-smoker current vapers have statistically non-significant risk (RR=2.53; 95% CI: 0.96–6.67; n=12751) of prevalence of COPD compared to never users. However, dual users were found to have significantly higher risk (RR=3.86; 95% CI: 1.49–10.02; n=11487) compared to never users, but no statistically significant difference was seen when compared to non-vaper current smokers (RR=1.15; 95% CI: 1.00–1.33; N=7338) (Figure 4). Heterogeneity was high (I^2^=93–94%) for the first two analyses, but low (I^2^=0%) for the last one. Major risk of publication bias was detected in the Doi plots (LFK index 2.08, -2.92, -3.76, respectively) for all three meta-analyses (Supplementary file Material 5).

Harvest plots of observational studies^24, s54,92,106,142^ revealed that the majority of the studies found higher risk of COPD in non-smoker current vapers^24, s92,106^ and dual users^24, s106,142^ compared to non-users (Supplementary file Figure 1). Additionally, harvest plots of the experimental studies^s60,64,112,113,145,148,150^ showed similar findings with all studies except one^s148^ reporting higher risk of COPD following acute and short-to-medium term exposure to e-cigarettes compared to non-use (Supplementary file Figure 2). However, all evidence on experimental studies were cell/*in vitro* or animal based. On the other hand, one observational study found similar incident risk of COPD between non-smoker current vapers and non-vaper current smokers following long-term exposure^s106^ (Supplementary file Figure 1). Similarly, one cellular experimental study detected no statistically significant differences in risk of COPD following acute exposure to e-cigarettes compared to cigarettes^s64^, indicating similar response (Supplementary file Figure 2). The population and findings in other observational studies^s37,43,52,58,65,137^ were too heterogenous to reach any conclusion.

### Asthma

No meta-analysis could be conducted to assess risk of asthma from e-cigarette use. However, harvest plots of observational studies^24,25, s85,92,135,141,142^ showed that the majority of the studies detected higher risk of asthma or asthma severity in non-smoker current vapers compared to non-users^s92,135,141,142^, but no significant higher risk in dual users compared to non-users^24,25, s85,141^ (Supplementary file Figure 1). On the other hand, harvest plots of experimental studies showed that all studies detected higher risk of asthma following acute and short-to-medium term exposure of e-cigarettes compared to non-use^s64,89,128,129^ (Supplementary file Figure 2). Of the experimental studies, one was a non-randomized human experimental study assessing biomarker-based evidence of increased susceptibility to asthma following acute exposure to e-cigarettes^s89^. Additionally, one of the animal experimental studies found similar risk of asthma following acute exposure to e-cigarettes compared to cigarettes^s64^. The rest of the studies were too heterogenous in population and findings^s37,43,44,49,58,65,86,137^ to allow for meaningful comparison.

### Impact on lung function

We could not conduct any meta-analysis to assess the impact on lung function following e-cigarette exposure. Two animal experimental studies did not find any significant evidence of impairment of lung function (e.g. forced expiratory volume in the first second/forced vital capacity ratio) following acute exposure to e-cigarettes compared to non-use^s115,144^ (Supplementary file Figure 2). On the other hand, although two human non-randomized experimental studies did not find any significant risk^s89,100^, four animal experimental studies reported higher risk of impairment of lung function following short-to-medium term exposure^s113,119,148,150^. Additionally, one animal experimental study found similar risk of impairment of lung function following short-tomedium term exposure to e-cigarettes compared to cigarettes^s144^. The rest of the studies were heterogeneous in their studied population and findings^s52,55,57,76,81,86^.

### Lung inflammation and damage

No meta-analysis could be conducted to assess the risk of lung inflammation and damage from e-cigarette use. Harvest plots of observational studies^s107,110,122,139,146^ showed that all studies except ones^146^ found higher risk of lung inflammation and damage in non-smoker current vapers compared to non-users (Supplementary file Figure 1). All studies were cross-sectional in study design. Similarly, harvest plots of experimental studies^35, s39-42,47,55,56,59,60,63,66,70,73, 75,83,88,89,93,94,97,101,113,115,117,119,121,127,131,132,138,140,144,148,149^ showed that all studies except three^s47,75,144^ found higher risk of lung inflammation and damage following acute and short-to-medium term exposure to e-cigarettes compared to non-use (Supplementary file Figure 2). Among the experimental studies, two were human non-randomized experimental studiess^89,117^, and rest of the studies were cell/*in vitro* and animal studies. Additionally, one observational study reported higher risk in non-smoker current vapers compared to non-vaper current smokers^s139^ (Supplementary file Figure 1), while two animal experimental studies reported similar risk^s42,113^, one study reported lower risks^144^, and one study found higher risk^s55^ following acute and short-to-medium term exposure of e-cigarettes compared to cigarettes^s42,55,113^ (Supplementary file Figure 2). Other studies were too heterogenous in their study population^s38,57,125^.

### COVID-19 and other respiratory infections

No meta-analysis was conducted to assess the risk of COVID-19 and other respiratory infections. Among the observational studies found on these outcomes, four studies reported higher risk of COVID-19 or other respiratory infections in non-smoker current vapers compared to non-users^s72,78,95,107^, while 4 other studies found no such significant risk^s62,79,109,147^ (Supplementary file Figure 1). Two studies in the latter group were longitudinal observational studies^s62,147^. On the other hand, all experimental studies^s42,64,88,108,111,112,118,149^ showed higher risk of respiratory infections following acute and short-to-medium term exposure to e-cigarettes compared to non-use, although all were cell/*in vitro* and animal studies (Supplementary file Figure 2). Additionally, one observational study found similar risk in dual users compared to non-users^s95^, while another observational study showed lower risk in non-smoker current vapers compared to non-vaper current smokers^s78^ (Supplementary file Figure 1). Similarly, one animal experimental study reported similar risk, while another animal study found lower risk following e-cigarette exposure compared to cigarette exposure^s42,64^ (Supplementary file Figure 2). Other studies were heterogenous in their study population^s37,65,91^ to allow comparison.

### EVALI and other lung conditions

Of the five case series^s68,69,82,134,136^ and thirteen case reports^s48,51,53,61,67,71,74,80,87,99,105,114,126^ looking into EVALI, one case series^s68^ and five case reports^s51,53,61,67,126^ reported presence of EVALI in exclusive nicotine vapers, following mostly short-to-medium term exposure. The rest of the cases were mostly dual vapers of tetrahydrocannabinol (THC) and nicotine or exclusive THC vapers. Additionally, we found three cases of acute eosinophilic pneumonia^34,s120,133^, one case of diffuse alveolar hemorrhage^s84^, one case of pulmonary Langerhans cell histiocytosis^s90^, and one case of sarcoidosis^s130^ among e-cigarette users. No meta-analysis or harvest plots were used to assess the risk of EVALI in nicotine e-cigarette users.

### Lung development *in utero*

We found seven animal experimental studies that reported impaired lung development or lung function in the offsprings exposed to nicotine e-cigarettes *in utero*^36, s46,98,102-104,124^ (Supplementary file Figure 2). These changes included reduced lung weight and impaired mucociliary clearance, emphysematous changes, lung fibrosis, respiratory inflammation, and changes in gene expression. Only one animal experimental study compared lung development *in utero* between exposure to e-cigarettes and cigarettes, and found lower risk of impact following e-cigarette exposure^s124^. We did not conduct any meta-analysis to assess the risk of impaired lung development following e-cigarette exposure *in utero*.

### Sociodemographic factor-based subgroup findings

Supplementary file Material 6 depicts the distribution of studies comparing different respiratory effects based on sex subgroups. As evident, there were inconsistent findings on sex-based differences in COPD and asthma, impact on lung function, and lung development *in utero*. One longitudinal observational study reported higher risk of asthma in female current vapers compared to male current vapers^s135^, while another cross-sectional study found lower risk of COPD in female dual users compared to male dual users^s81^. Similarly, while one animal experimental study^s113^ reported higher risk of COPD, another animal study^s128^ found lower risk of asthma in female mice compared to male mice. Additionally, one animal study reported higher risk of impact on lung function in female mice compared to male mice, while another animal study found no such differences^s113,148^. In case of lung development *in utero*, one animal study reported higher risk of impaired lung development in female offsprings compared to male offsprings, one animal study found the opposite effect, and two other animal studies found mixed findings^36, s46,98,102^.

Among the experimental studies examining sex-based differences in lung inflammation and damage^s113,148,151,154,155^, the majority of the studies found no sex-based differences^s113,148,151^ (Supplementary file Material 6). In case of COVID-19, two animal experimental studies^s152,153^ found lower risk in females compared to males, while one cross-sectional study reported mixed findings^s91^. Among the three cross-sectional studies examining age-based differences, one study reported higher odds of prevalence of COPD in older dual users (≥65 years) compared to younger (40–64 years) dual users^s81^, one study found higher risk of increased asthma severity in older (≥60 years) current vapers compared to younger (18–39 years) current vapers^s128^, and another study found higher odds of prevalence of COVID-19 in older people compared to younger people (average age of the population was 20.3 ± 1.5 years)^s91^. The third study also examined race, and sexual orientation-based differences in prevalence of COVID-19 and reported higher risk in non-Hispanic Whites compared to others, and no significant differences between heterosexual and sexual minority people^s91^. No meta-analysis could be conducted to assess any sociodemographic factor-based differences for different respiratory outcomes.

### Certainty of evidence

Supplementary file Material 7 summarizes the evidence profile on different components of the GRADE and GRADE-CERQual certainty assessment for each individual findings and Table 2 presents the summary of these assessments. Overall, all meta-analysis findings were rated as ‘very low’ to ‘low’ certainty evidence, while harvest plot findings were rated as ‘very low’ to ‘moderate’ certainty evidence. None of the findings was rated as having ‘high’ certainty evidence.

**Table 2:** GRADE and GRADE-CERQual summary of findings on certainty of the evidence based on the studies included in main analysis and subgroup analysis (N=88)

*Summary of findings*	*Number of studies*	*Study design*	*Explanations of certainty assessment*	*Certainty*
**GRADE assessment**				
Incident risk of respiratory symptoms: Non-smoker current vapers had higher risk compared to never users	2	Observational	Serious risk of bias; serious inconsistency; major risk of publication bias; small number of studies	⨁◯◯◯ Very low
Incident risk of respiratory symptoms: Non-smoker current vapers had lower risk compared to non-vaper current smokers	2	Observational	Serious risk of bias; major risk of publication bias; small number of studies	⨁⨁◯◯ Low
Incident risk of respiratory symptoms: Dual users had higher risk compared to never users	2	Observational	Serious risk of bias; serious inconsistency; major risk of publication bias; small number of studies	⨁◯◯◯ Very low
Incident risk of respiratory symptoms: Dual users had higher risk compared to non-smoker current vapers	2	Observational	Serious risk of bias; major risk of publication bias; small number of studies	⨁⨁◯◯ Low
Incident risk of respiratory symptoms: Dual users had similar risk compared to non-vaper current smokers	2	Observational	Serious risk of bias; serious imprecision; minor risk of publication bias; small number of studies	⨁◯◯◯ Very low
Prevalent risk of COPD: Non-smoker current vapers had statistically non-significant risk compared to never users	2	Observational	Serious inconsistency; serious imprecision; major risk of publication bias; small number of studies	⨁◯◯◯ Very low
Prevalent risk of COPD: Dual users had higher risk compared to never users	2	Observational	Serious inconsistency; major risk of publication bias; small number of studies	⨁⨁◯◯ Low
Prevalent risk of COPD: Dual users had similar risk compared to non-vaper current smokers	2	Observational	Serious imprecision; major risk of publication bias; small number of studies	⨁⨁◯◯ Low
**GRADE-CERQual assessment**				
Respiratory symptoms: higher risk in non-smoker current vapers compared to non-users	2	Observational	Serious concerns on adequacy of data	Moderate
COPD: Higher risk in non-smoker current vapers and dual users compared to non-users	5	Observational	Minor concerns on adequacy of data	Moderate
COPD: Higher risk following acute and short-to-medium term exposure to e-cigarettes compared to non-use	7	Experimental (cell/*in vitro*, animal)	Serious methodological limitations; and moderate concerns on relevance; moderate concerns on relevance	Low
Asthma: Higher risk among non-smoker current vapers compared to non-users, but no significant risk in dual users compared to non-users	7	Observational	Minor methodological limitations	Moderate
Asthma: Higher risk following acute and short-to-medium term exposure to e-cigarettes compared to non-use	4	Experimental (human, cell/*in vitro*, and animal)	Serious methodological limitations; minor concerns on adequacy of data; and moderate concerns on relevance	Very low
Impact on lung function: No significant risk following acute exposure, but higher risk following short-to-medium term exposure to e-cigarettes compared to non-use	8	Experimental (human and animal)	Serious methodological limitations, and moderate concerns on relevance	Low
Lung inflammation and damage: Higher risk in non-smoker current vapers compared to non-users	5	Observational	Minor methodological limitations, and minor concerns on adequacy of data	Moderate
Lung inflammation and damage: Higher risk following acute and short-to-medium term exposure to e-cigarettes compared to non-use, and similar risk following short-to-medium term exposure to e-cigarettes compared to cigarettes	35	Experimental (human, cell/*in vitro*, and animal)	Serious methodological limitations, moderate concerns on relevance	Low
COVID-19 and Respiratory infections: Inconsistent findings on risk among non-smoker current vapers compared to non-users	8	Observational	Serious methodological limitations	Moderate
COVID-19 and Respiratory infections: Higher risk following acute and short-to-medium term exposure to e-cigarettes compared to non-use, but inconsistent findings on risk between e-cigarette exposure and cigarette exposure	8	Experimental (cell/*in vitro*, animal)	Serious methodological limitations; moderate concerns on relevance	Low
E-cigarette or vaping associated lung injury (EVALI): is mostly associated with cannabis vaping, not nicotine vaping	18	Observational	Moderate methodological limitations, moderate concerns on relevance	Moderate
Lung development *in utero*: Higher risk of impact following exposure to e-cigarettes compared to nonuse	7	Experimental (animal)	Serious methodological limitations, moderate concerns on relevance	Low
Sex-based subgroup differences: Inconsistent findings for impact on asthma and COPD, impact on lung function, and lung development *in utero*	9	Experimental (animal); observational	Serious methodological limitations, moderate concerns on relevance	Low
Sex-based subgroup differences: No sex-based differences in lung inflammation and damage and lower risk of COVID-19 in females compared to males	8	Experimental (animal); observational	Serious methodological limitations, moderate concerns on relevance	Low

EVALI: e-cigarette or vaping associated lung injury. COPD: chronic obstructive pulmonary disease. GRADE: Grading of Recommendations Assessment, Development and Evaluation. GRADE-CERQual: Confidence in Evidence from Reviews of Qualitative Research.

## DISCUSSION

This systematic review and meta-analysis highlighted several important findings on the respiratory health impacts of e-cigarette use. First, both meta-analysis and harvest plot findings showed that non-smoker current vapers had significantly higher incident risk of respiratory symptoms compared to never users and non-users, respectively. Nevertheless, the risk level was found to be lower than that seen among non-vaper current smokers. Additionally, dual users were found to have significantly higher incident risk of respiratory symptoms compared to both never users and non-smoker current vapers, and the risk level was similar to that seen among non-vaper current smokers. Although these findings ranged from ‘very low’ to ‘moderate’ certainty evidence, they were consistent with previous reviews^3, s156^.

Second, although non-smoker current vapers were not found to have any statistically significant increase in the risk of prevalence of COPD compared to never users in meta-analysis, harvest plot findings suggested they had higher risk compared to non-users. The later finding was rated as having ‘moderate’ certainty evidence and matches with previous meta-analyses^9, s157^. Additionally, evidence from the experimental studies showed higher risk of COPD following both acute and short-to-medium term exposure to e-cigarettes compared to non-use. The discrepancy between the meta-analysis and harvest plot findings appears to stem from the limited number of studies included in the meta-analysis. Both meta-analysis and harvest plot findings demonstrated that dual users had higher risk of prevalence of COPD compared to never users and non-users, respectively, where the harvest plot finding has ‘moderate’ certainty evidence. Moreover, the risk level was found to be similar to that seen among non-vaper current smokers. These findings question the safety of e-cigarette use as a smoking cessation aid and underscore the need for further investigation into the association.

Third, we found ‘moderate’ certainty evidence that non-smoker current vapers had higher risk of asthma or asthma severity compared to non-users. Similarly, experimental study findings revealed that acute and short-to-medium term exposure to e-cigarettes increases risk of asthma compared to non-use. These findings matches with previous reviews^s158,159^. Although dual users seemed to have no such significant risk, it contradicts a previous meta-analysis^s157^, hence, this association should be further investigated. Additionally, there was higher risk of impairment of lung function following short-to-medium term exposure to e-cigarettes compared to non-use (Supplementary file Figure 2). However, no such risk was found following acute exposure, which matches findings from previous reviews^2, s160^.

Fourth, we found ‘moderate’ certainty evidence that non-smoker current vapers had higher risk of lung inflammation and damage compared to non-users. Similarly, there was substantial number of experimental studies suggesting that both acute and short-to-medium term exposure to e-cigarettes can induce significant lung inflammation and damage compared to non-use, and the risk level was similar to that of cigarettes following short-to-medium term exposure. Although it was ‘low’ certainty evidence, the findings matches previous review^s161^.

Fifth, there were inconsistent findings on risk of COVID-19 and respiratory infections among non-smoker current vapers compared to non-users, but higher risk was seen following both acute and short-to-medium term exposure to e-cigarettes compared to non-use in the experimental studies. Hence, more human longitudinal research is needed to further investigate this association.

Sixth, consistent with previous evidence^s162,163^, we found ‘moderate’ certainty evidence that EVALI is mostly associated with cannabis vaping, rather than nicotine vaping. However, future longitudinal observational studies are needed to reach a definitive conclusion. Additionally, animal study findings suggest that exposure to e-cigarettes *in utero* increases risk of impaired lung development and function in offsprings, which definitely needs to be further investigated though future human research.

Finally, our findings on sex-based subgroup analysis revealed no significant sex differences in the risk of lung inflammation and damage, but lower risk of COVID-19 in females compared to males. As the findings on other sex-based differences were inconsistent and findings on other sociodemographic factor-based subgroup analysis were insufficient to reach any conclusion, further longitudinal investigations are warranted.

We observed several methodological challenges and research gaps in the included studies, which need further attention. For example, several studies defined their population as current vapers^s49,65,77,85,100^, without clearly distinguishing amongst non-smoker current vapers, former smoker current vapers and dual users. It might be inappropriate and misinformative to the audience to indicate risk of respiratory effects in current vapers, whereas this effect might be from smoking cigarettes rather than e-cigarettes. In addition, while it is important to examine effect of e-cigarette exposure among non-smoker current vapers, it is equally important to understand the magnitude of effects in former smoker current vapers and dual users compared to non-vaper current smokers. There was significant lack of evidence on long-term respiratory effects of e-cigarette use, highlighting the need for prospective longitudinal observational research to assess these effects.

### Limitations

Our review has several limitations. First, most of our findings were rated as ‘very low’ and ‘low’ certainty evidence with a few ‘moderate’ certainty evidence (Table 2). Hence, these findings should be interpreted with caution. The reasons behind having ‘very low’ and ‘low’ certainty evidence were mainly availability of low number and quality of studies, and lack of enough human evidence. Therefore, it highlights the need for more longitudinal observational research and rigorously designed randomized controlled trails to further investigate our findings. Second, our meta-analysis findings are limited by the number of studies, high heterogeneity in the data, lack of robust estimators, and minor to major risk of publication bias. These limitations present a considerable barrier to the generalizability of our results. To strengthen our synthesis, we complemented the meta-analysis with a SWiM approach and visualized findings using harvest plots. Nevertheless, in respect of previous evidence^3, s156,164^, it is worth exploring the risk of incidence and prevalence of COPD and respiratory symptoms among different e-cigarette user populations and updating our findings by adding new data from future research. Third, we included studies that were published following the McNeill et al.^2^ review to avoid duplication with previous reviews^2,3^. Therefore, our findings should be interpreted alongside theirs. A future umbrella review can be conducted to easily incorporate studies from previous reviews alongside those from our review. Fourth, there are some methodological differences between the McNeill et al.^2^ review and our review. For example, the McNeill et al.^2^ review did not include any self-reported data, hence did not report findings on the incidence or prevalence of any respiratory conditions. However, we thought this information were important to understand population trends and included studies with self-reported data in our review. Moreover, the McNeill et al.^2^ review mostly defined non-use as not smoking or vaping in the past 6 months, and daily or almost daily use as current use. We defined our comparison groups differently to reflect the definition of current use (use in the past 30 days) that was used in the majority of the studies. Hence, these differences should be kept in mind while comparing our findings with the McNeill et al.^2^ review. Finally, other limitations of this review include the absence of grey literature search, multiple comparisons and outcomes that may inflate positive results, potential residual confounding in included studies, and the inability to establish causal relationships. Additionally, we did not assess the impact of dual vaping of THC and nicotine on respiratory outcomes beyond EVALI. Future research should address these limitations to strengthen the evidence base.

## CONCLUSIONS

We found evidence of significant harmful respiratory health effects from use of e-cigarettes such as increased risk of respiratory symptoms, COPD, asthma, impact on lung function, and respiratory inflammation. However, most of our findings were severely limited by having ‘very low’ and ‘low’ certainty evidence, number of studies, and methodological quality. Hence, there is a need for future research to update the evidence and investigate further respiratory risks of e-cigarette exposure by conducting rigorously designed clinical trials and longitudinal studies.

## Supplementary Material



## Data Availability

The data supporting this research can be found in the Supplementary file.
