# Supplementary material for: Evidence update on the respiratory health effects of vaping e-cigarettes: A systematic review and meta-analysis
Source: Tob Induc Dis. 2025 Nov 19;23:10.18332/tid/209954. doi: 10.18332/tid/209954 (PMC12628669; doi:10.18332/tid/209954)
Supplement: Supplementary file 1 [file TID-23-177-s1.pdf]

Supplementary Materials

Evidence update on the respiratory health effects of vaping e-cigarettes: A systematic review and meta-analysis.

Supplementary Figures: 1-2

Supplementary Material 1. Database search strategies

Supplementary Material 2. Summary of studies assessing risk of respiratory health outcomes from exposure to e-cigarettes (n=119).

Supplementary Material 3. Summary of subgroup findings from the 2022 McNeill et al. review assessing risk of respiratory health outcomes (n=5).

Supplementary Material 4. Quality assessment findings

Supplementary Material 5. Publication bias assessment of studies included in the meta-analyses.

Supplementary Material 6. Harvest plots showing distribution of studies assessing sex-based differences in risk of asthma and COPD, impact on lung function, lung inflammation and damage, COVID-19, and lung development in utero among females compared to males (individual bar in the plot represents a single study) (n=15).

Supplementary Material 7. GRADE and GRADE-CERQual evidence profile to assess certainty or confidence in the body of evidence.

Supplementary Material 8. References 37-164.

Supplementary Material 9: Data _retrieved from literature search_ N_5_125_

Supplementary Figure 1. Harvest plots showing distribution of observational studies assessing risk of different outcomes (respiratory symptoms, COPD, asthma, lung inflammation and damage, COVID-19 and respiratory infections) between non-smoker current vapers vs non-users, non-smoker current vapers vs non-vaper current smokers, and dual users vs non-users (individual bar in the plot represents a single study) (n=24).

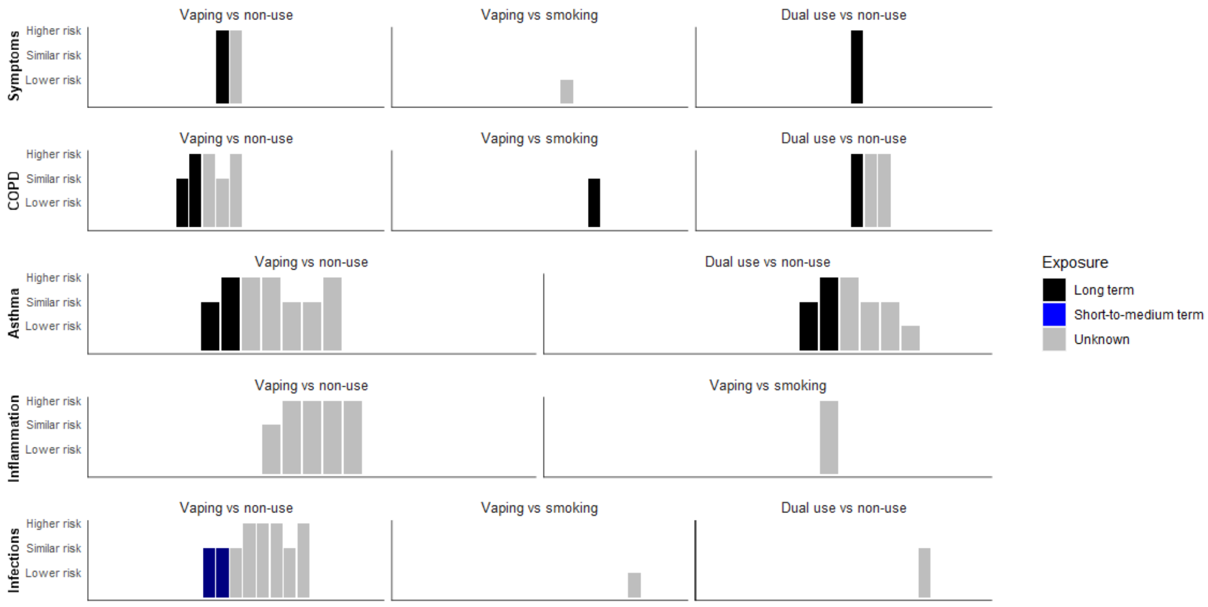

Supplementary Figure 2. Harvest plots showing distribution of experimental studies assessing risk of different outcomes (COPD, asthma, impact on lung function, lung inflammation and damage, COVID-19 and respiratory infections, and lung development in utero) between vaping vs non-use and vaping vs smoking (individual bar in the plot represents a single study) (n=52).

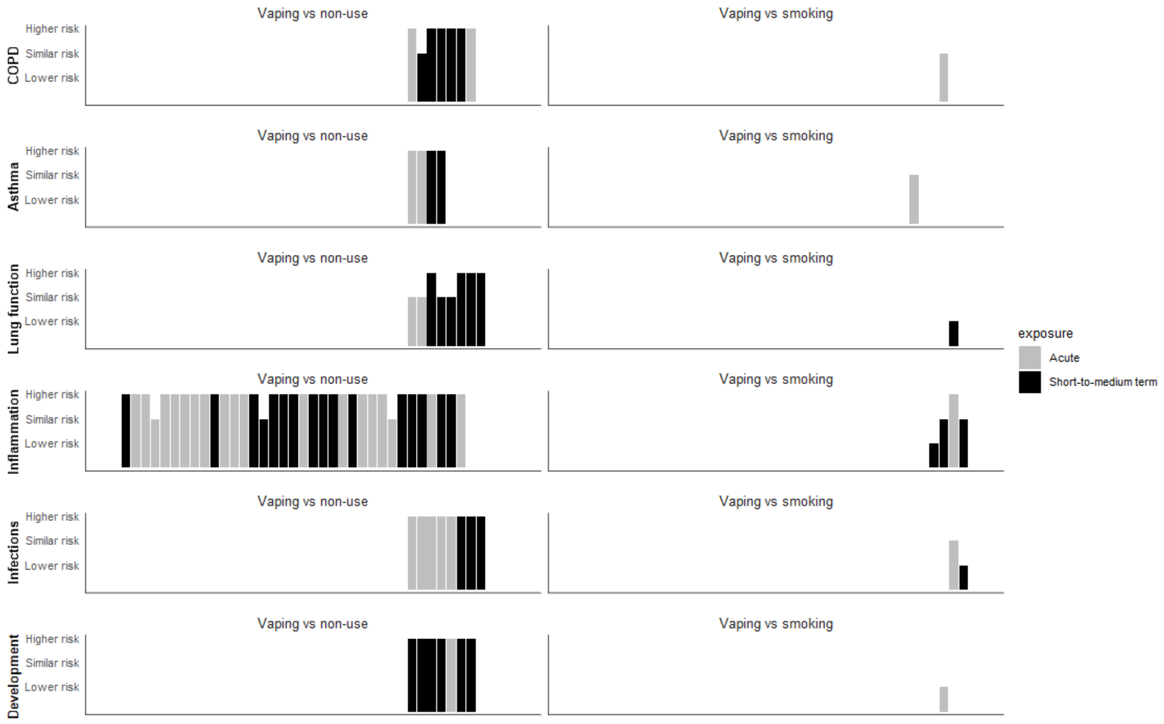

Supplementary Material 1. Database search strategies

Date	Serial	Searches	Results
**MEDLINE**			
Jan 31, 2023	1.	1. exp Electronic Nicotine Delivery Systems/<br>2. exp Vaping/<br>3. e-cig*.tw,kw.<br>4. electronic cig*.tw,kw.<br>5. (ENDS and nicotine).tw,kw.<br>6. electronic nicotine delivery system*.tw,kw.<br>7. vaping.tw,kw.<br>8. vape*.tw,kw.<br>9. (nicotine and (vapor* or vapouris*)).tw,kw.<br>10. 1 or 2 or 3 or 4 or 5 or 6 or 7 or 8 or 9<br>11. exp Lung/<br>12. ((respiratory or lung) adj2 effect*).tw,kw.<br>13. exp Cardiovascular Diseases/<br>14. ((heart or cardiac or cardiovascular or vascular) adj2 effect*).tw,kw.<br>15. exp Neoplasms/<br>16. (neoplas* or cancer* or carcinoma* or malignan* or tumor*).tw,kw.<br>17. 11 or 12 or 13 or 14 or 15 or 16<br>18. 10 and 17<br>19. limit 18 to yr="2021 -Current"	359
Jan 31, 2023	2.	1. exp Electronic Nicotine Delivery Systems/<br>2. exp Vaping/<br>3. exp "Tobacco Use Disorder"/<br>4. (e-cig* or (electronic adj2 cig*) or ENDS or vaping or vape*).tw,kw.<br>5. ((e-cig* or vaping or nicotine) adj2 (dependen* or addict*)).tw,kw.	891

		6. 1 or 2 or 4<br>7. 3 or 5<br>8. 6 and 7<br>9. limit 8 to yr="2017 -Current"	
Jan 02, 2024	3.	Updated search 1<br>limit 18 to dt=20230201-20231231	152
Jan 02, 2024	4.	Updated search 2<br>limit 8 to dt=20230201-20231231	146
**EMBASE**			
Jan 31, 2023	1.2	1. exp Electronic Nicotine Delivery Systems/<br>2. exp Vaping/<br>3. e-cig*.tw,kw.<br>4. electronic cig*.tw,kw.<br>5. (ENDS and nicotine).tw,kw.<br>6. electronic nicotine delivery system*.tw,kw.<br>7. vaping.tw,kw.<br>8. vape*.tw,kw.<br>9. (nicotine and (vapor* or vapouris*)).tw,kw.<br>10. 1 or 2 or 3 or 4 or 5 or 6 or 7 or 8 or 9<br>11. exp Lung/<br>12. ((respiratory or lung) adj2 effect*).tw,kw.<br>13. exp Cardiovascular Diseases/<br>14. ((heart or cardiac or cardiovascular or vascular) adj2 effect*).tw,kw.<br>15. exp Neoplasms/<br>16. (neoplas* or cancer* or carcinoma* or malignan* or tumor*).tw,kw.<br>17. 11 or 12 or 13 or 14 or 15 or 16<br>18. 10 and 17<br>19. limit 18 to yr="2021 -Current"	907
Jan 31, 2023	2.	1. exp Electronic Nicotine Delivery Systems/	1362

		2. exp Vaping/<br>3. exp "Tobacco Use Disorder"/<br>4. (e-cig* or (electronic adj2 cig*) or ENDS or vaping or vape*).tw,kw.<br>5. ((e-cig* or vaping or nicotine) adj2 (dependen* or addict*)).tw,kw.<br>6. 1 or 2 or 4<br>7. 3 or 5<br>8. 6 and 7<br>9. limit 8 to yr="2017 -Current"	
Jan 02, 2024	3.	Updated search 1<br>limit 18 to dd=20230201-20231231	170
Jan 02, 2024	4.	Updated search 2<br>limit 8 to dd=20230201-20231231	52
**PsycINFO**			
Jan 31, 2023	1.	1. exp Electronic Cigarettes/<br>2. e-cig*.tw.<br>3. electronic cig*.tw.<br>4. (ENDS and nicotine).tw.<br>5. electronic nicotine delivery system*.tw.<br>6. vaping.tw.<br>7. vape*.tw.<br>8. (nicotine and (vapor* or vapouris*)).tw.<br>9. 1 or 2 or 3 or 4 or 5 or 6 or 7 or 8<br>10 exp lung disorders/<br>11 ((respiratory or lung) adj2 effect*).tw.<br>12 exp Cardiovascular Disorders/<br>13 ((heart or cardiac or cardiovascular or vascular) adj2 effect*).tw.<br>14 exp Neoplasms/<br>15 (neoplas* or cancer* or carcinoma* or malignan* or tumor*).tw.<br>16 10 or 11 or 12 or 13 or 14 or 15	49

		17 9 and 16<br>18 limit 17 to yr="2021 -Current"	
Jan 31, 2023	2,	1. exp Electronic Cigarettes/<br>2. "tobacco use disorder"/<br>3. (e-cig* or (electronic adj2 cig*) or ENDS or vaping or vape*).tw.<br>4. ((e-cig* or vaping or nicotine) adj2 (dependen* or addict*)).tw.<br>5. 1 or 3<br>6. 2 or 4<br>7. 5 and 6<br>8. limit 7 to yr="2017 -Current"	336
Jan 02, 2024	3.	Updated search 1<br>limit 17 to up =20230201-20231231	28
Jan 02, 2024	4.	Updated search 2<br>limit 7 to up =20230201-20231231	65
**CINAHL**			
Jan 31, 2023	1.	S1. (MH "Electronic Cigarettes")<br>S2. (MH "Vaping")<br>S3. TI (e-cig* or (electronic N2 cig*) or ENDS or vaping or vape*)<br>S4. S1 OR S2 OR S3<br>S5. (MH "Lung Diseases")<br>S6. TI ((respiratory or lung) N2 effect*)<br>S7. (MH "Cardiovascular Diseases")<br>S8. TI ((heart or cardiac or cardiovascular or vascular) N2 effect*)<br>S9. (MH "Neoplasms")<br>S10. TI (neoplas* or cancer* or carcinoma* or malignan* or tumor*)<br>S11. S5 OR S6 OR S7 OR S8 OR S9 OR S10<br>S12. S4 AND S11<br>S13. S12 ((Limiters - Published Date: 20210701-20221231)	339
Jan 31, 2023	2.	S1. (MH "Electronic Cigarettes")<br>S2. (MH "Vaping")<br>S3. TI (e-cig* or (electronic N2 cig*) or ENDS or vaping or vape*)<br>S4. S1 OR S2 OR S3<br>S5. TI (e-cig* or vaping or nicotine) N2 (dependen* or addict*)<br>S6. S4 AND S5<br>S7.S6 (Limiters - Published Date: 20170101-20221231)	73
Jan 02, 2024	3	Updated search 1<br>S14. S12 ((Limiters - Published Date: 20230201-20231231)	171

Jan 02, 2024	4.	Updated search 2<br>S8. S6 ((Limiters - Published Date: 20230201-20231231)	13
**PubMED**			
Jan 31, 2023	1.	1. "electronic nicotine delivery systems"[MeSH Terms] OR ("electronic"[All Fields] AND "nicotine"[All Fields] AND "delivery"[All Fields] AND "systems"[All Fields]) OR "electronic nicotine delivery systems"[All Fields] OR ("electronic"[All Fields] AND "cigarettes"[All Fields]) OR "electronic cigarettes"[All Fields]<br>2. "vaped"[All Fields] OR "vaping"[MeSH Terms] OR "vaping"[All Fields] OR "vapes"[All Fields]<br>3. #1 OR #2<br>4. "lung diseases"[MeSH Terms] OR ("lung"[All Fields] AND "diseases"[All Fields]) OR "lung diseases"[All Fields]<br>5. "cardiovascular diseases"[MeSH Terms] OR ("cardiovascular"[All Fields] AND "diseases"[All Fields]) OR "cardiovascular diseases"[All Fields]<br>6. "cancer s"[All Fields] OR "cancerated"[All Fields] OR "canceration"[All Fields] OR "cancerization"[All Fields] OR "cancerized"[All Fields] OR "cancerous"[All Fields] OR "neoplasms"[MeSH Terms] OR "neoplasms"[All Fields] OR "cancer"[All Fields] OR "cancers"[All Fields]<br>7. #4 OR #5 OR #6<br>8. #3 AND #7<br>9. #8 Filters: from 2021/7/1 - 2022/11/28	937
Jan 31, 2023	2.	1. "electronic nicotine delivery systems"[MeSH Terms] OR ("electronic"[All Fields] AND "nicotine"[All Fields] AND "delivery"[All Fields] AND "systems"[All Fields]) OR "electronic nicotine delivery systems"[All Fields] OR ("electronic"[All Fields] AND "cigarettes"[All Fields]) OR "electronic cigarettes"[All Fields]<br>2. "vaped"[All Fields] OR "vaping"[MeSH Terms] OR "vaping"[All Fields] OR "vapes"[All Fields]<br>3. #1 OR #2<br>4. "tobacco use disorder"[MeSH Terms] OR ("tobacco"[All Fields] AND "disorder"[All Fields]) OR "tobacco use disorder"[All Fields] OR ("nicotine"[All Fields] AND "dependence"[All Fields]) OR "nicotine dependence"[All Fields]<br>5. #3 AND #4<br>6. #5 Filters: from 2017/1/1 - 2022/11/28	998
Jan 02, 2024	3.	Updated search 1<br>9. #8 Filters: from 2023/02/01 - 2023/12/31	520
Jan 02, 2024	4.	Updated search 2<br>7. #5 Filters: from 2023/02/01 - 2023/12/31	162
**Cochrane**			
Jan 31, 2023	1.	#1 [mh "Electronic Nicotine Delivery Systems"]<br>#2 [mh vaping]<br>#3 (e-cig* or (electronic NEAR/2 cig*) or ENDS or vaping or vape*):ti,ab,kw<br>#4 [mh "lung diseases"]<br>#5 ((respiratory or lung) NEAR/2 effect*):ti,ab,kw<br>#6 [mh "Cardiovascular Diseases"]<br>#7 ((heart or cardiac or cardiovascular or vascular) NEAR/2 effect*):ti,ab,kw<br>#8 [mh Neoplasms]<br>#9 (neoplas* or cancer* or carcinoma* or malignan* or tumor*):ti,ab,kw<br>#10 #1 or #2 or #3<br>#11 #4 or #5 or #6 or #7 or #8 or #9<br>#12 #10 and #11 with Cochrane Library publication date Between Jul 2021 and Nov 2022	92
Jan 31, 2023	2.	#1 [mh "Electronic Nicotine Delivery Systems"]<br>#2 [mh vaping]	185

		#3 (e-cig* or (electronic NEAR/2 cig*) or ENDS or vaping or vape*):ti,ab,kw<br>#4 [mh "Tobacco Use Disorder"]<br>#5 ((e-cig* or vaping or nicotine) NEAR/2 (dependen* or addict*)):ti,ab,kw<br>#6 #1 or #2 or #3<br>#7 #4 or #5<br>#8 #6 and #7 with Cochrane Library publication date Between Jan 2017 and Nov 2022	
Jan 02, 2024	3.	Updated search 1<br>#12 #10 and #11 with Cochrane Library publication date Between Feb 2023 and Dec 2023	51
Jan 02, 2024	4.	Updated search 2<br>#8 #6 and #7 with Cochrane Library publication date Between Feb 2023 and Dec 20231	20

Supplementary Material 2. Summary of studies assessing risk of respiratory health outcomes from exposure to e-cigarettes (n=119).

Author and year; Country	Funding source; conflict of interest	Type of exposure (exposure length/follow-up duration)	Total Participants (participant characteristics)	Intervention/exposure	Health condition/outcome; Reversibility of health effect	Study findings	Subgroup characteristics	Subgroup findings	Risk of bias/critical appraisal
**Non-Randomised Experimental Study**									
Nyilas et al., 2022;[100] Switzerland	Independent funding organization(s); some (No pro-tobacco association)	Acute (Single exposure, <24 hrs.)	N=44; mean age 41 (SD 12) years; 63.6% male.	Healthy Control (never smokers): n=10.<br>Former Smokers: n=9, recent quitters for max 6 months, no current EC use.<br>Current vapers: n=13; included dual users (n=5), participants were daily vapers, 31% used flavoured EC.<br>Current smokers: n=12,included dual users (n=2), participants were daily smokers.<br><br>Participants were instructed to smoke or vape ad lib using their own products.	Impairment of Lung function; not measured	fMRI:<br>Current vapers had significant increase in perfusion after exposure ($p = 0.01$). NS changes in functional ventilation (RFV) among current smokers and vapers.<br><br>PFT:<br>NS changes in FEV1 and FVC. NS changes in PFT after vaping.<br><br>Interpretation: Acute exposure to EC caused an increase in lung perfusion, but no effect on lung ventilation.	N/A	N/A	Serious
Sayed et al., 2021;[117] US	Independent funding organization(s); some (No pro-tobacco association)	Short to medium term (2 weeks)	N=33; mean age =21 years; 67% males, 33% females; 3% AA, 55% Asian, 36% Caucasian, 6% Mixed.	Nonusers: n= 13; were healthy people not using e-cigarettes and cigarettes.<br><br>EC users: n= 20; used 0.5-1mL e-liquid/day or 3.5-7 mL/wk for >6mo at baseline; instructed to reduce vaping over 2 weeks; included non-smoker current vapers (n=15), dual users (n=5).	Respiratory inflammation; Not measured	Saliva levels of inflammatory markers:<br>EC users had significantly lower levels of IL-1 receptor antagonist ($p < 0.0001$) compared to non-users.<br><br>Sputum levels of inflammatory markers:<br>EC users had significantly higher IL-1Ra and growth-regulated oncogene (GRO) levels ($p < 0.01$ and $p < 0.05$ respectively) compared to non-users.	N/A	N/A	Moderate

						Interpretation: Short-medium term exposure of EC aerosols induced inflammation in the airways.			
Majek et al., 2023;[89] UK	None; None	Acute (single exposure)	N=160; 18-30 years old; 53.1% male, 46.9% female.	EC (n=40): participants were non-smoker current vapers; instructed to vape non-flavored EC containing 12 mg/ml nicotine for 5 min.<br>Cig (n=40): participants were non-vaper current smokers; instructed to smoke one Cig- non-flavored containing 0.5 mg nicotine per Cig.<br>HTP (n=40): participants were exclusive HTP users; Instructed to use one non-flavored tobacco stick of IQOS 2.4<br>Control (n=40): participants never smoked or vaped in their lifetime, instructed to simulate smoking for 5 min.<br><br>Participants were instructed to not use their products for 6 hrs. before the intervention.	Impairment of lung function, Asthma, Respiratory inflammation; Reversible	FeNO:<br>Significantly decreased 5 min post-exposure compared to baseline ($p<0.01$) in EC group. NS difference was seen between baseline and 30-min post-exposure measures in EC group.<br>FeNO significantly increased in 30 min post-exposure compared to 5-min post-exposure measures in EC group.<br><br>Temperature of exhaled air-indicating inflammation:<br>Significantly increased 5 min and 30 min post-exposure compared to baseline ($p<0.01$) in EC group.<br><br>FEV1/FVC (%):<br>NS difference was found between baseline and post-exposure in EC group.<br><br>Interpretation: Acute exposure to EC increased susceptibility to asthma, and was associated with respiratory inflammation, NS effect was seen on lung function.	N/A	N/A	Moderate
**Longitudinal observational studies**									
Gao et al., 2022;[62] UK	Independent funding organization(s); some (No pro-tobacco association)	Short to medium term (4 months)	N=7,869,534; mean age 48.2 years (SD 18.6); 52.2% male, 47.8% female; 68.1% White, 1.2% Caribbean, 2.5% Black African,	Never smokers: n=4,718,435;<br>Former smokers: n=1,748,966;<br>Current smokers: n=1,402,133;<br>Current vapers:	COVID-19-associated severity; Not measured (death-irreversible).	Hospital records of COVID-19 hospitalization, ICU admission, and death:<br>NS difference was seen between current vapers and non-smokers.<br>NS difference was seen	N/A	N/A	Some concerns

			1.0% Chinese, 3.9% Other.	n=69,047; Never smoker current vapers: n=3251; Former smoker current vapers: n=35267; Dual users: n=30529. <br>No definitive definition of groups provided.		between former smoker current vapers vs former smokers, and dual users vs non-vaper current smokers. <br>Interpretation: NS association between EC use and COVID-19 related severity was seen.			
Young-Wolff et al., 2022;[147] US	Independent funding organization(s); some (No pro-tobacco association)	Short to medium term (10 months)	N=74,853; mean age 27.6 (4.8) years; 83.3% females, 36% White, 27.6% Hispanic, 23% Asian/Pacific Islander, 7.6% Black, 5.8% Other race; all were never smokers.	Never users: n=72728; Never used either products in their lifetime; <br>Never smoker current vapers: n=1233; Never smoked Cigs, but used currently ECs, even sometimes. <br>Never smoker former vapers: n=892; Never smoked Cigs, and formerly used ECs.	COVID-19 infection; Reversible	COVID positive PCR test: NS difference in COVID-19 infection risk between groups. <br>Interpretation: EC use was not associated with risk of COVID-19 infection among never smokers.	N/A	N/A	Some concerns
Xie et al., 2022;[143] US	Independent funding organization(s); none	Long-term (3 years)	N=6,378 (unique adults), N= 13,083 (total observations); 18-24 years; 49.0% Female, 51% male; 70.2% White, 14.7% Black, 15.1% Other racial groups.	Never vapers (n=10,205), Former Vapers (n=2,080), Current Vapes (n=798). <br>Never users (n=8,388): used <100 Cigs in their lifetime and never used ECs. Never smoker former vapers (n=1,140): used <100 Cigs in their lifetime and used EC previously. Never smoker current vapers (n=312): used <100 Cigs in their lifetime and used EC everyday or some days.	Respiratory symptoms (wheezing, dry cough); Reversible	Self-reported any respiratory symptoms, self-reported wheezing in the chest: Former vapers and current vapers had significantly higher odds compared to never vapers ($p<0.05$ for all). Never smoker former vapers and never smoker current vapers had significantly higher odds compared to never users ($p<0.05$ for all). Never-smoker current vapers, and current dual users had significantly higher odds compared to non-users/never smokers ($p<0.05$ for all); with the highest effect seen among dual users. NS difference between former smoker current vapers and	N/A	N/A	Some concerns

				Former smoker current vapers (n=115): Used >100 Cigs in their lifetime, but not currently, used EC everyday or some days.<br><br>Non users: used neither products currently.<br>Non-smoker current vapers (n=427): Currently used only EC everyday or some days.<br>Non vaper current smokers (n=1,417): Currently used only Cigs everyday or some days.<br>Current dual users (n=346): Used both products everyday or some days.		non-users/never smokers.<br><br>Wheezing during exercise:<br>NS difference between never vapers, former vapers and current vapers.<br>Never smoker current vapers had significantly higher risk compared to never users (aOR 2.41).<br>Never-smoker current vapers, and current dual users had significantly higher odds compared to non-users/never smokers ($p<0.05$ for all); with the highest effect seen among dual users.<br>NS difference between former smoker current vapers and non-users/never smokers.<br><br>Self-reported night time dry cough:<br>NS difference between never vapers and current vapers.<br>NS difference between never smoker current vapers or never smoker former vapers and never users.<br>Dual users had significantly higher odds compared to non-users/never smokers ($p<0.05$);<br>NS difference between Never-smoker current vapers or former smoker current vapers and non-users/never smokers.<br><br>Interpretation: Never smoker current vapers and dual users had significantly higher risk of respiratory symptoms compared to never users or non-users.			
Paulin et al., 2022;[106]	Independent funding	Long-term (4 years)	N=13,752 for prevalence study at	Never users: n=3817; never used any tobacco	COPD; Irreversible	Prevalence of COPD:<br>Non-smoker current vapers,	N/A	N/A	Low

US	organization(s); none		baseline (cross-sectional), N=6,945 for incidence study (cohort): mean age 58.1 (SE 0.1) years; 47.2% males, 71.4% NH White, 11.4% Hispanic.	products;<br>Former tobacco users: n=2173; currently did not use any tobacco products, but previously used.<br>Non-smoker current vapers: n=198; Used only ECs in past 30 days.<br>Non vaper current smokers: n=3773; Used only Cigs in past 30 days.<br>exclusive cigar users: n=391; Used only cigars in past 30 days.<br>exclusive smokeless/snus users: n=482; Used only smokeless tobacco in past 30 days.<br>Current dual users: n=852; used both Cig and EC in past 30 days.<br>Current polycombusted tobacco users: n=619; Used Cigs and other combustibles in past 30 days<br>Current polycombusted and non-combusted users: n=461; Used ECs, Cigs and other combustibles in past 30 days		dual users, and polycombusted+noncombusted users had significantly higher odds (p<0.001 for all) compared to never users.<br>NS difference between non-smoker current vapers or dual users and non-vaper current smokers.<br><br>Incidence of COPD:<br>Non-smoker current vapers and dual users had significantly higher odds (p<0.001) compared to never users.<br>NS difference between polycombusted+noncombusted users and never users<br>NS difference between non-smoker current vapers or dual users and non-vaper current smokers.<br><br>Interpretation: non-smoker current vapers and dual users had significantly higher prevalent and incident risk of COPD compared to never users, which was similar to that seen among non-vaper current smokers.			
Sargent et al., 2022;[116] US	Independent funding organization(s); some (No pro-tobacco association)	Short- to medium-term; and long-term (1 year)	N=16,295; 18-55+ years old; 50.6% females, 49.4% males; 65.6% White, 11.3% Black, 15% Hispanic, 8.1% Other race.	Never users (N=5,888): Never used any tobacco products.<br>Non-smoker current vapers (N=327): Used only ECs in past 30 days.<br>Non vaper current smokers (N=3230):	Respiratory symptoms; Not measured	ISAAC core wheezing module for functionally important respiratory symptoms:<br>Dual users, and combustible + non-combustible users had significantly higher odds (p<0.001 for all) compared to never users.<br>NS difference between non-	N/A	N/A	Low

				Used only Cigs in past 30 days.<br>Exclusive cigar users (n=548): Used only cigars in past 30 days.<br>Exclusive SLT users (n=450): Used only smokeless tobacco in past 30 days.<br>Exclusive hookah users (n=321): Used only hookah in past 30 days.<br>Current dual users (N=792): used both Cig and EC in past 30 days.<br>Polycombustible users (n=1266): Used Cigs and other combustibles in past 30 days<br>Combustible + non-combustible users (n=1118): Used ECs, Cigs and other combustibles in past 30 days		smoker current vapers and never users.<br><br>Worsening of respiratory symptoms or becoming symptomatic over time:<br>Non-smoker current vapers had a significant association with worsening symptoms at a threshold of ≥2 ($p<0.05$) compared to never users, but not at a symptom threshold of ≥3, which was seen among non vaper current smokers.<br>Dual users, and combustible + non-combustible users had significant higher risk at both worsening symptoms at a threshold of ≥2 or ≥3 compared to never users ($p<0.05$ for all).<br><br>Improvement of respiratory symptoms or becoming asymptomatic over time:<br>Only non-smoker current vapers had higher likelihood of improving symptoms at a threshold of ≥3 ($p<0.05$) compared to never users.<br><br>Interpretation: Dual users and combustible + non-combustible users had significantly higher risk of respiratory symptoms, symptoms worsening compared to never users. Non-smoker current vapers only had significant risk of worsening of symptoms compared to never users.			
Goldberg Scott et al., 2023;[65] US	Independent funding organization(s);	Long-term/Prospective (1.4-2 yrs);	N=119,593; 18+ years age, 22% >70 years; 60% female,	Never vapers (n=112396): Never used ECs.	COPD, asthma, pneumonia, influenza; reversible	Prevalence of COPD:<br>Former and current vapers had significantly higher odds	N/A	N/A	Low

	none	Retrospective	40% male; Heterosexual (n=109,222), gay or lesbian (n=8023) and other sexual orientation (n=1218); 70% NH White, 10% Hispanic, 10% NH Asian, 6% NH Black, 2% American Indian/Alaska Native, 2% NH Native Hawaiian/Other race/ethnicity; used both prospective and retrospective EHR data.	Former vapers (n=5603): Used ECs in the past year, but not in past 30 days.<br>Current vapers (n=1594): Used ECs in past 30 days; included dual users, non-smoker current vapers	and irreversible	compared to never vapers ($p<0.05$ for all).<br><br>Prevalence of asthma:<br>Former vapers had significantly higher odds compared to never vapers ($p<0.05$). NS difference between current vapers and never vapers.<br><br>Incidence of pneumonia and influenza:<br>NS difference between groups.<br><br>Interpretation: current vapers had significantly higher prevalent risk of COPD than never vapers. NS risk of asthma, or incidence of pneumonia and influenza was seen among current vapers.			
Kang et al., 2021;[77] South Korea	Independent funding organization(s); none	Long-term (retrospective); 6 years	N=28,950; >19 years; male (n-9,388); none were former smokers; pooled data over 3 waves (6 years).	Current vapers (n=578, 2%): used EC in the past 30 days.<br>Non-vapers (n=28,372, 98%): did not use EC in past 30 days.	Acute severe pneumonia; Not measured	Hospital admission data of acute severe pneumonia:<br>Current vapers had 0 incidence, NS risk among current vapers.	N/A	N/A	Some concerns
Brunette et al., 2023;[44] US	Independent funding organization(s); none	Long-term; 3 years	N=10,267; 18-38 years old; 49.6% male, 50.4% female; 58.3% White, 11.9% Black, 9.8% other race, 19.9% Hispanic; none of participants were diagnosed with COPD or other non-asthma respiratory diseases	At baseline,<br>Current vapers (n=1,323): Used EC in past 30 days,<br><br>Non-vapers (n=8,944): Did not use EC in past 30 days,<br><br>Current smokers (n= 3,708): Smoked cigarette in past 30 days,<br><br>Non-smokers (n=6,559): Did not smoke cigarette in past	Asthma, asthma severity; reversible	Prevalence and incidence of asthma:<br>NS difference in asthma prevalence and incidence were seen between non-vapers and current vapers. However, similar findings were also seen among non-smokers vs current smokers.<br><br>Prevalence and incidence of severity of asthma in asthma control test (ACT) score:<br>NS difference in incidence were seen between non-vapers and current vapers.	N/A	N/A	High

				30 days.		Interpretation: Long-term exposure to EC was not associated with risk of asthma development or asthma severity.			
Cook et al., 2023;[54] US	Independent funding organization(s); none	Long-term (6 years)	N=9,681; ≥ 40 years age; 47% male, 53% female; 70.4% NH White, 11.4% Hispanic, 11.2% NH Black, 6% NH others.	At baseline,<br>Current vapers (n=359): Used EC everyday or some days,<br><br>Non-vapers (n=9,502): Did not use EC regularly,<br><br>Current smokers (n= 3,387): Currently smoked cigarettes everyday or some days,<br><br>Former smokers (n=1,962): Not currently smoking cigarettes, but smoked ≥100 cigarettes in their lifetime,<br><br>Never-smokers (n=4,512): Did not smoke or smoked <100 cigarettes in lifetime.	COPD; irreversible	Self-reported incidence of COPD in past 12 months: Current vapers had significantly (p<0.001) higher odds of incident COPD compared to non-vapers. However, smoking acts as a confounders and NS risk was seen when model was adjusted for smoking status (higher risk among former and current smokers) and cigarette pack years.<br><br>Incidence: NS risk of development of COPD was found on long-term exposure to EC when the influence of smoking cigarettes was taken into consideration.	N/A	N/A	Low
Mukerjee et al., 2023;[96] US	Independent funding organization(s); none	Long-term (6 years)	N=17,517; 12-17 years age; 51.5% male, 48.5% female; 53% NH White, 12.9% NH Black, 24.1% Hispanic, 10% other race/ethnicity.	At baseline,<br>Non-smoker current vapers (1.3-3.9%): Used only EC not Cig in past 30 days,<br><br>Non-vaper current smokers (1-2%): Used only Cig not EC in past 30 days,<br><br>Dual users (0.6-0.9%): Used both Cig and EC in past 30 days,	Respiratory symptoms (chronic cough measured with bronchitis and pneumonia); reversible	Self-reported incidence of respiratory symptoms in past 12 months:<br>Both non-smoker current vapers and dual users had significantly higher incident risk (IRR 1.5, p<0.05 and IRR 2.4, p<0.001 respectively) compared to non-users.<br><br>Interpretation: Long-term exposure to EC and dual use were significantly associated with increased risk of respiratory symptoms.	N/A	N/A	Low

				Non-users (94.2-96.2%): Did not smoke or vape in lifetime or past 30 days.					
Patel et al., 2023;[25] US	Independent funding organization(s); none	Long-term (6 years)	N=9,141; 12-17 yrs age at baseline, 50.4% were 15-17 yrs. old; 50.2% female, 49.8% male; 55.3% NH White, 22.7% Hispanic, 13.4% NH Black, 8.6% other race/ethnicity.	At baseline,<br>Non-users (n=8,590): Did not use wither Cig or EC in their lifetime or in past 30 days.<br><br>Non-smoker current vapers (n=142): Used EC exclusively in past 30 days.<br><br>Non-vaper current smokers (n=283): Smoked Cig exclusively in past 30 days.<br><br>Dual users (n=126): used both Cig and EC in past 30 days.	Asthma; reversible	Self-reported incidence of asthma in past 12 months:<br>NS risk was seen in non-smoker current vapers and dual users compared to non-users.<br><br>Interpretation: Long-term exposure to EC was not associated with risk of developing asthma in either non-smoker current vapers or dual users.	N/A	N/A	Some concerns
To et al., 2023;[135] Canada	Independent funding organization(s); none	Long-Term (3-years)	N=2700; aged 15-30 years;	Current vapers (n=505): used EC in the past 30 days; 34.65% female and 65.35% male; 74.26% were dual users.<br><br>Non-users (n=2195): Did not use EC in past 30 days; 37.36% female and 62.64% male; 70.39% were daily smokers.<br>Both groups were matched using a propensity score.<br><br>Never smoker current vapers (n=75),<br>Former smoker current vapers (n=55),	Asthma, asthma severity; reversible	Self-reported prevalence of asthma:<br>NS difference between groups was seen.<br><br>Self-reported incidence of asthma related health service use in past 12 months:<br>Never smoker current vapers and dual users had significantly higher odds ($p<0.05$, $p<0.0001$ respectively) compared to non-user never smokers.<br><br>Interpretation: Long-term use of EC was associated with higher probability of asthma-related health service use among never smoker current vapers and dual users.	Sex:<br>Current vapers: 34.65% female and 65.35% male;<br><br>Non-users: 37.36% female and 62.64% male	Self-reported incidence of asthma attack in past 12 months:<br>Female current vapers had significantly higher odds ($p=0.005$) compared to male non-users.<br>NS difference was seen in male current vapers and male non-users.<br><br>Self-reported	Low

				Dual users (n=375): Used both Cig and EC in past 30 days, Non-vaper current smoker (n=1545), Non-vaper never smokers (n=365).				incidence of asthma related health service use in past 12 months: Female current vapers had significantly higher odds (p<0.001) compared to male non-users. NS difference was seen in male current vapers and male non-users.<br><br>Interpretation: Long-term EC use was associated with significant asthma severity in female current vapers, but not in male current vapers.	
**Cross-sectional studies**									
Varella et al., 2022;[137] US	None; none	N/A	N=18,079; 18-44 years old; males (n=8691) , White (n=11496), Black (n=2214) , Hispanic (n=3002), other (n=1367)	Current daily vapers: (n=448): Used EC everyday. Current frequent vapers: (n=753): Used EC some days. Former vapers: (n=4,261): Had quit EC. Never vapers: (12,559): Did not use EC in their lifetime.	Asthma, COPD, Respiratory symptoms (cough, phlegm, or shortness of breath); Reversible and non-reversible.	Prevalence of asthma: Significantly higher among current frequent vapers (p<0.001).<br><br>Prevalence of COPD: Significantly higher among current frequent vapers (p<0.001).<br><br>Self-reported respiratory symptoms:	N/A	N/A	Low (7/8)

						Significantly increased in current frequent vapers and former vapers (p<0.05 and 0=0.009 respectively) compared to never vapers. NS difference between current daily vapers and never vapers.<br><br>Interpretation: Asthma and COPD was significantly prevalent among frequent EC users. Current vapers and former vapers had significantly higher prevalence of respiratory symptoms compared to never vapers.			
Ying et al., 2022;[146] US	Independent funding organization(s); some (No pro-tobacco association)	N/A	N=28; 21-30 years old; male (n=19), female (n=9); White (n=24), AA/Asian (n=4)	Never smokers: n=10; used <100 cigarettes lifetime and no cigarette/EC use in past 1 year.<br><br>Non-smoker current vapers: n=10; on average used 13.4mg/ml nicotine containing EC 7.1 ml/day, 147.5 puff/day for at least 6 months.<br><br>Non vaper current smokers: n=8; on average smoked for 7.9 years, 18.1 cigarettes/day for at least 6 months.	Alteration in lung microbiota (associated with pulmonary inflammation); Not measured	BAL samples -meta transcriptome profiling: Non-smoker current vapers had NS changes in the lung microbiome and transcriptional load compared with never-smokers. Twelve bacteria species differed between smokers vs vapers, with decrease in Neisseria sp. KEM232 and Curvibacter sp. AEP1-3 were observed in vapers.<br><br>Interpretation: EC use did not significantly affect lung microbiota- indicating NS association with respiratory inflammation.	N/A	N/A	Low (14/20)
Kim and Kang, 2021;[81] South Korea	Independent funding organization(s); none	N/A	N=12,919; 40+ years old.	Never-smokers: n=6924; never smoked an EC and had not smoked an EC in the past 30 days, had never smoked or smoked <100 cigarettes in their lifetime.	Impairment of lung function; COPD; Non-reversible	PFT:<br>FEV1, predicted FEV1% were significantly lower and FVC was significantly higher in dual users (p<0.001, p<0.001, p=0.007 respectively) than others.<br>NS difference in predicted FVC%.	Age groups: Middle aged (40-64 years) (n=9896) and Older adults ≥65 years (n=3032)<br><br>Sex:	Age groups: The odds of COPD was significantly higher among older adults compared to middle aged among current	Low (15/20)

				Non vaper former smokers: n=3282; smoked >100 cigarettes in their lifetime but did not smoke currently and did not use an e-cigarette in the past 30 days were categorized as former smokers.<br><br>Non vaper current smokers: n=2549; >100 cigarettes in their lifetime, smoke currently, and did not use an e-cigarette.<br><br>Dual users: n=164; those who smoke currently, have smoked >100 cigarettes in their lifetime, and used an e-cigarette in the past 30 days.		FEV1/FVC% was significantly lower in dual users ($p<0.001$) than others.<br><br>Prevalence of COPD:<br>Dual users had significantly higher odds of COPD ($p<0.001$) compared to never smokers.<br><br>Interpretation: Dual users had significantly lower FEV1/FVC ratio than non-vaper current, former or never smokers. Dual users also had significantly higher prevalent risk of COPD.	Male (n=6596): Never smokers (n=1165), current dual users (n=155)<br><br>Females (n=6323): Never smokers (n=5759), current dual users (n=9)	dual users ($p<0.05$).<br><br>Sex:<br>Male dual users had significantly higher odds of COPD ($p<0.001$) compared to male never smokers.<br>NS difference was seen in female groups.<br><br>Age and sex combined:<br>The odds of COPD was significantly higher among male older adults compared to male middle aged for all groups ($p<0.05$).<br>NS difference was seen in older or middle aged female groups who were dual users.	
Mori et al., 2022;[92] US	Independent funding organization(s); none	N/A	N=84; 21-30 years old; mean age 25.7 years; female 45.2%; White 77.4%, Non-white 22.6%.	Never users: n=43, had smoked <100 cigarettes in their lifetime, and also did not use either products for at least 1 year.<br><br>Non-smoker current	Susceptibility to respiratory diseases (asthma, COPD); Not measured	mtCN and mtDNA mutations in lung epithelium from bronchoscopy:<br>Non-smoker current vapers were in between non-vaper current smokers and never users in terms of mtCN and mtDNA mutations. NS	N/A	N/A	Low (15/20)

				vapers: n=15, used nicotine-containing EC daily for at least 1 year and had not smoked a cigarette for >6 months<br><br>Non vaper current smokers: n=26, were daily smokers who smoked >10 cigarettes per day for >6 months and did not use EC for at least 1 year.		difference in point mutation in the D-loop at nucleotide position T16519C between groups.<br><br>Inflammatory markers:<br>IL-2 and IL-4 significantly increased in only non-smoker current vapers (FDR=0.065 for both) compared to never users.<br><br>Gene expression:<br>Genes associated with respiratory disease cases (asthma, COPD) and genes associated with cellular movement, inflammation, metabolism, and airway hyperresponsiveness increased in non-smoker current vapers compared to healthy controls.<br><br>Interpretation: Non-smoker current vapers had higher susceptibility to respiratory diseases like Asthma and COPD.			
Merianos et al., 2022;[91] US	Independent funding organization(s); none	N/A	N=756; 18-24 years old; 50.4% males, 49.6% females; NH white (61.6%), NH Black (8.1%), NH other/multiracial (14.8%), Hispanic (15.5%); all were current EC users.	Non-smoker current vapers (n=405, 53.6%): Used only EC in past 30 days.<br><br>Dual users of cigarette and EC (n=154, 20.4%): Used both Cig and EC in past 30 days.<br><br>Dual users of cigar and EC (n=25, 4.6%): Used both cigar and EC in past 30 days.<br><br>Poly tobacco users (n=162, 21.4%): Used EC, Cig and cigars in	COVID-19; Reversible	Self-reported symptoms of COVID-19 ≥1:<br>Current dual users of cigarettes and EC and current polytobacco users had significantly higher risk compared to non-smoker current vapers (p= 0.035 and p<0.001 respectively).<br><br>Self-reported COVID-19 diagnosis:<br>Current dual users of cigarettes and EC and current polytobacco users had significantly higher risk compared to non-smoker current vapers (p= 0.035 and	Age: Average age: 20.3 (SD 1.5 years).<br><br>Sex: Male (n=381), female (n=375)<br><br>Race/ethnicity: NH white (n=466), NH Black (n=61), NH other/multiracial (n=112), Hispanic (n=117).	Age: Older participants had higher risk of COVID-19 symptoms (AOR=1.31, p=0.001).<br><br>Sex: Females had higher risk of COVID-19 symptoms (AOR=1.85, p=0.022) but lower risk of COVID-19 diagnosis (APR= 0.55,	Low (6/8)

				past 30 days.		p<0.001 respectively).<br><br>Interpretation: Dual users had significantly higher risk of COVID-19 and COVID-19 related symptoms than non-smoker current vapers.	Sexual orientation: Heterosexual (n=605), LGBTQ (n=151).	p=0.010) compared to males.<br><br>Race/ethnicity: NH other /multiracial had lower risk of COVID-19 diagnosis compared to NH White (APR= 0.40, p=0.014).<br><br>Sexual orientation: NS difference in COVID-19 symptoms, testing and diagnosis between heterosexual and LGBTQ.	
Kelesidis et al., 2022;[79] US	Independent funding organization(s); none	N/A	N=33; mean age 24 years; males (n=19)	Current smokers: n=9; currently smoked Cigs,<br><br>Non-smoker current vapers: n=12 (7 were former smokers who had quit more than 2 years before the study)<br><br>Non-users: n=12; Not using either products currently.<br><br>Age, sex, race, and educational level did not differ among groups.	COVID-19; Not measured	Key COVD-19 proteins ACE2, TMPRSS2, and ADAM17 levels in CD45+ T cells detection in peripheral blood:<br>NS difference between current vapers and non-smokers.<br><br>Furin MFI:<br>Significantly increased (1.15-fold, p < .05) in current vapers compared to non-smokers.<br><br>Interpretation: Non-smoker current vapers had no significant risk of COVID-19 compared to non-users.	N/A	N/A	Moderate (7/20)
Wills et al., 2022;[142] US	Independent funding organization(s);	N/A	N=21,494; 18-59 years old; 54.2% females; 56.4% NH	Never vapers: n=96,005; did not use EC in their lifetime.	Asthma and COPD; Reversible and irreversible	Self-reported asthma:<br>Former vapers, current nondaily vapers had higher	N/A	N/A	Low (6/8)

	none		White, 12.8% NH Black, 22.2% Hispanic, 7% NH Asian, 1.5% NH multiracial.	Former vapers: n=31,784; Used EC in their lifetime, but not currently.<br><br>Current nondaily vapers: n=4,845; Used EC some days currently.<br><br>Current daily vapers: n=3744; used EC everyday currently;<br><br>Never smokers: n=128,336; never smoked <100 Cigs in their lifetime.<br><br>Former smokers: n=40,728; smoked >100 Cigs in their lifetime, but not currently.<br><br>Current nondaily smokers: n=9681; smoke Cigs some days currently.<br><br>Current daily smokers: n=24,677; smoked Cigs everyday currently.		risk compared to never vapers (p<0.0001; p<0.001 respectively). Non-smoker former vapers, non-smoker current nondaily vapers, non-smoker current daily vapers and dual users had higher risk compared to never users (p<0.0001; p<0.001; p<0.01; p<0.0001 respectively).<br>NS difference between current daily vapers vs never vapers.<br><br>Self-reported COPD:<br>Former vapers, current nondaily vapers and current daily vapers had higher risk compared to never vapers (p<0.0001; p<0.001; p<0.0001 respectively). Non-smoker former vapers, and dual users (non-daily and daily vapers and smokers) had higher risk compared to never users (p<0.0001; p<0.0001 respectively).<br>NS difference between non-smoker current nondaily vapers or daily vapers vs never users.<br><br>Interpretation: Non-smoker current vapers, non-smoker former vapers and dual users had significantly higher prevalent risk of asthma, while dual users had significantly higher prevalent risk of COPD.			
Cordova et al., 2022;[24] US	Independent funding organization(s); none	N/A	N=26,072; 18+ years age, 70.4% 35+ years old; 48% male, 52% female; 65.1% White, 11.7% Black,	Non-smoker current vapers (n=621): used EC some day or every day.<br><br>Non-vaper current	Asthma, bronchitis and COPD; Reversible and irreversible	Self-reported diagnosis of asthma in past 12 months:<br>NS risk was seen in non-smoker current vapers and dual users compared to never users.	N/A	N/A	Low (6/8)

			15.5% Hispanic, 7.8% other/multiracial. (pooled data over 4 years).	smokers (n=5776): smoked cigarette some day or every day<br><br>Dual users (n=806): Used both cigarette and EC some day or every day.<br><br>Never users of any product (n=10211).		Self-reported diagnosis of COPD in past 12 months: Significantly increased risk was seen among all groups- non-smoker current vapers ($p<0.05$) and dual users ($p<0.05$) compared to never users.<br><br>Self-reported diagnosis of bronchitis in past 12 months: Significantly increased risk was seen among dual users compared to never users ($p<0.05$). NS difference was seen between non-smoker current vapers and never users.<br><br>Interpretation: Non-smoker current vapers and dual users had significantly higher prevalent risk of COPD and dual users had higher prevalent risk of bronchitis compared to never users, but NS risk of asthma was seen.			
Chaffee et al., 2021;[49] US	Independent funding organization(s); none	N/A	N=10,483; 13-21 years age; 45.2% male, 54.4% female; 32.3% NH White, 46.6% Hispanic, 21.2% other race/ethnicity	Non-vapers (n=7,783): Did not use in past 30 days<br><br>Current vapers (n=2,448): Used EC in past 30 days; most commonly used pod type device; categorized into:<br>Low frequency vapers (used EC 1-5 days in past 30 days),<br>High frequency vapers (used EC 6-30 days in past 30 days).	Asthma exacerbation, respiratory symptoms (shortness of breath and bronchitis symptoms); Reversible	Asthma exacerbation: Multiple device users had higher risk compared to pods only users (aOR 1.64). NS difference between low or high frequency current vapers.<br><br>Shortness of breath: Compared to non-users, high frequency current vapers had higher odds of shortness of breath (aOR 1.68) and bronchitis symptoms (aOR 1.56). NS risk was seen in low frequency current vapers.<br><br>Interpretation: High frequency EC use was associated with	N/A	N/A	Low (6/8)

						high prevalent risk of bronchitis symptoms and shortness of breath, while multiple device EC users had higher prevalent risk of asthma exacerbation.			
Davis et al., 2022;[57] US	Independent funding organization(s); none	N/A	N=41; mean age 26.3-33.5 years; female (n=20), male (n=21); White (n=21), AA (n=12), Asian (n=3), Hispanic (n=3), Other (n=2).	Never users (n=17): Never used either products in their lifetime.<br><br>Smokers (n=13): were daily smokers; used on average 12.6 ± 6.8 cigarettes per day with history of 9.0 ± 5 pack years.<br><br>Vapers (n=11): were daily users; vaped 50.5 ± 92.0 puffs/per day, e-liquid consumption 5.2 ± 11.8 ml/day; were either former smoker current vapers or never smoker current vapers or dual users.	Impairment of lung function; Pulmonary inflammation; Not measured	FEV1, FVC:<br>NS difference in either FEV1 or FVC between groups.<br><br>Inflammatory cells in BALF:<br>NS difference in cell counts between never users, smokers and vapers.<br><br>iNOS expression:<br>Vapers had a significant increase in iNOS-positive alveolar macrophages compared to the other groups ($p<0.05$).<br><br>Gene expression:<br>Vapers alveolar macrophages had greater proportion of downregulated genes than smokers and never users. IL-19, IL-24, CXCL12, IL-6, MUC1 and CD70 downregulated, and EBI3, CCL15, and IL-17F , IL-3, IL-17F, TNFRSF13 and IL1RL2 were upregulated among vapers.<br><br>Interpretation: Vaping EC was associated with unique changes to host defence and can increase susceptibility to lung inflammation. NS difference in lung function observed between current vapers and current smokers.	N/A	N/A	Low (13/20)
Chaiton et al., 2023;[50]	Independent funding	N/A	N=3,082; 16-25 years age; 80.6%	Never vapers (n=1554, 50.4%);	Respiratory symptoms	Self-reported respiratory symptoms:	N/A	N/A	Low (6/8)

Canada	organization(s); none		females; 73.7% White; (n-396) had a diagnosis of asthma	Ever vapers (n=323, 10.5%);<br>Current vapers (n=1205, 39%);<br>Daily dual users (42, 1.4%);<br>Never users (n= 825, 26.8%);<br>Ever users (n=1303, 42.3%);<br>Current smokers (n=92, 3.0%).<br><br>Mean no. of year vaped by EC users 1.7-2.4 years	(shortness of breath, cough, phlegm, wheezing, recurrent infection); Not measured	Each additional puff year increased the risk of respiratory symptoms significantly ($p<0.05$) among never smokers/ never users, but not in current smokers.<br>Current vapers using pod-style devices had significantly higher risk of respiratory symptoms ($p<0.01$). NS risk was seen among cig-a-like device users.<br><br>Interpretation: Vaping can increase risk of respiratory symptoms among never smokers.			
Wetherill et al., 2023;[139] US	Independent funding organization(s); some (No pro-tobacco association)	N/A	N=15; mean age 27-35 years.	Non-smoker current vapers (n=5): vaped average 7 ± 4 times/day.<br>Non-smoker current smokers (n=5): smoked average 8 ± 4 cigarettes/day.<br>Never users (cig and e-cig) (n=5): did not use either product in their lifetime.	Pulmonary inflammation; Not measured	NOS expression in 18F-NOS PET:<br>Non-smoker current vapers had higher 18F-NOS non-displaceable binding potential values than cigarette smokers ($p= 0.03$) and healthy never users ($p= 0.01$).<br>NS difference in 18F-NOS lung tissue delivery and iNOS distribution volume between groups.<br><br>Interpretation: Non-smoker current vapers had greater pulmonary inflammation than non-vaper current smokers and never users.	N/A	N/A	Moderate (11/20)
Lee et al., 2021;[86] Australia	Independent funding organization(s); some (No pro-tobacco association)	N/A	N=519; 45.2-55.3 years old, male (n=213)	Ever vapers (n=46): 7% were non-smokers, 30% former smokers and 63% current smokers.<br>Never vapers (n=473): 56% were non-smokers, 36% former smokers and 12% current smokers.	Impairment of lung function, Respiratory symptoms, Asthma; Not measured	Self-reported symptoms:<br>Ever vapers had had higher prevalence of self-reported asthma (63% vs 42%, $p=0.007$), higher risk of chest tightness in the last 12 months ($p=0.017$) compared to never vapers.<br>NS association with wheeze, breathlessness, dyspnea, upper	N/A	N/A	Low (7/8)

						respiratory tract infection, chronic cough, and chronic phlegm.<br><br>Spirometry:<br>NS difference in asthma severity, spirometric COPD and nasal allergies/hay fever between ever and never vapers. NS difference in spirometric findings between ever vapers and never vapers.<br><br>Forced oscillation technique (FOT):<br>Differences in reactance and area under the reactance curve were found significant among ever vapers than never vapers ($p<0.05$).<br><br>Interpretation: Ever vaping was associated with increased prevalence of asthma and chest tightness. NS impact on lung function was observed among ever vapers compared to never vapers.			
Joshi et al., 2021;[76] Canada	Independent funding organization(s); none	N/A	N=44,817; 45-85 years old; Male (n=21,879, 47.8%), female (n=22,937, 52.2%); European ethnicity (n= 42,722, 95.2%), non-European (n=2,095)	Ever vapers: n=2,913, Never vapers: 41,904, 15+ pack year smokers: n=63,049, 0-14 pack year smokers: n= 13,500, Never smokers: n= 21,015.	Impairment of lung function; Not measured	Obstructive (FEV1/FVC < age dependent lower limit of normal), restrictive (FEV1/FVC> lower limit of normal):<br>Ever vaping (without ever smoking) significantly increased risk of obstructive lung function (aOR 2.10) compared to never vaping. But, NS association of ever vaping with restrictive lung function was found<br>Ever vaping with 15+ pack year smoking significantly increased risk of obstructive lung function compared to	N/A	N/A	Low (13/20)

						never vaping (aOR 2.40), but, had NS risk on restrictive lung function compared to never vaping.<br>Ever vaping with 0-14 pack year smoking had NS risk of obstructive and restrictive lung function compared to never vaping.<br><br>Interpretation: Ever vaping was associated with increased prevalent risk of obstructive lung function among never smokers. NS risk of restrictive lung function was seen.			
Hickman et al., 2022;[72] US	Independent funding organization(s); none	N/A	N = 103; mean=26.5; male = 55 (53.4%); white=72 (69.9%), black=19 (18.4%), Asian/Pacific Islander=7 (6.8%), mixed/other=5 (4.9%), Hispanic=10 (9.7%)	Nonusers: n = 28<br><br>Non-vaper current smokers: n = 21,<br><br>Non-smoker current vapers: n = 54, were current daily users (10–20 puffs per day) and did not report the use of tobacco products other than EC in the past 3 months or a greater than 10 pack-year history of smoking Cigs. Were categorized into:<br>3rd gen EC users: using primarily vape pens, box mods, or similar devices that contain freebase nicotine<br>4th gen EC users: using primarily JUUL or other low-powered e-cigarettes that contain nicotine salts.	Respiratory infection; Reversible	Sputum cytology:<br>Non-smokers current vapers had significantly increased lymphocytes per mg than nonusers (p=0.018), 3rd-gen users had significantly more macrophages per mg of sputum (p<0.012) than smokers<br><br>Inflammatory biomarkers:<br>4th gen users had Increased bronchial cells/mg than non-users and 3rd gen users (p<0.004), reduced concentrations of CRP, IFN-gamma, MCP-1, and uteroglobin in 4th gen e-cig users vs nonusers (but didn't reach statistical significance), sICAM1 and sVCAM1 significantly lower in 4th gen e-cig users compared to nonusers, decreased concentrations of TARC and VEGF in 4th gen users, increased concentrations of IL-10, MMP9, Eotaxin-3 in 4th gen users, increased Tie2 and	N/A	N/A	Low (15/20)

						MPO in 3rd gen users<br><br>Interpretations: Non-smoker current vapers had dysregulated immune homeostasis, increasing susceptibility to respiratory infection, highest effect was seen in 4th gen EC users.			
Polosa et al., 2021;[109] Italy	Independent funding organization(s); many (pro-tobacco association)	N/A	N=159; Median=32 years old; female n=79	Never smokers: n=40, smoked less than 100 cigarettes in their lifetime.<br><br>Current smokers: n=39, smoked ⩾10 cigarettes per day.<br><br>Former Smokers: n=40, did not smoke for at least 3–6months.<br><br>Current exclusive HTP users: n=20, exclusively used HTPs daily and not smoking for at least 3–6months.<br><br>Non-smoker current vapers: n=20, exclusively used ECs daily and not smoking for at least 3–6months.	Respiratory infection; Reversible	Mucociliary clearance:<br>NS difference between never smokers, and non-smoker current vapers.<br><br>Interpretation: Non-smoker current vapers have normal mucociliary clearance, hence less susceptibility to respiratory infection.	N/A	N/A	Low (13/20)
Payton et al., 2022;[107] US	Independent funding organization(s); none	N/A	N=44; 18 to 50 years; 57.1% male, 63.6% White, 22.7% Black 9.1%, Asian, 4.5% Other"	Non-smokers (n=14),<br><br>Current smokers (n=13): smoked a mean of 12 cigarettes/day,<br><br>Current vapers (n=17): Vaped a mean of 82 puffs/day with average nicotine content of 12 mg/mL.	Respiratory infection, respiratory inflammation; Not measured	Cytokine clusters in airway samples:<br>Two cytokine clusters that were related to airway immunity and inflammation were modified significantly ($p<0.05$) among current vapers compared to non-smokers. There were minimal changes at the individual cytokine level.	N/A	N/A	Low (13/20)

						Interpretation: Current vapers are highly susceptible to respiratory infections and inflammation compared to non-smokers.			
Bircan et al., 2021;[43] US	Not specified; none	N/A	N= 8736, 18-24 years, 34.4% female, 65.5% male, 64.10% NH White, 9.2% NH Black, 7.9% NH other race.	Never vapers: n= 4368, never used EC in their lifetime,<br><br>Current vapers: n= 4368; used EC everyday or some days.<br><br>Both groups were demographically matched.	Asthma, COPD, ACOS; Non-reversible	Self-reported prevalence of asthma, COPD, and ACOS: Compared with never vapers, current vapers had significantly higher odds of self-reported ACOS (OR=2.27), asthma (OR=1.26) and COPD (OR=1.44).<br><br>Interpretation: Current vapers had significantly higher prevalent risk of ACOS, asthma and COPD compared to never vapers.	N/A	N/A	Low (6/8)
Lee and Shin, 2023;[85] US	Not specified; none	N/A	N=218,911; 18-29 years age; 47.4% male, 52.6% female, 14.1% Hispanic, 85.9% NH, 79.1% White, 10.9% Black.	Current vapers: n=3,668, used EC everyday or some days,<br><br>Non-vapers: n=215243, did not use ECs currently,<br><br>Current smokers: n=17,229, definition not provided,<br><br>Former smokers: n=28,883, definition not provided,<br><br>Non-smokers: n=172,799, definition not provided.	Asthma severity; Reversible	Self-reported asthma attack or episode in past 12 months: Current Vapers had significantly higher odds of asthma episode/attack ($p<0.05$) compared to non-vapers.<br>Dual users and former smoker current vapers had significantly lower odds of asthma episode/attack ($p<0.05$ for both) compared to non-smokers.<br><br>Self-reported ER visits due to asthma in past 12 months: NS risk was seen any groups compared to non-smokers.<br><br>Interpretation: Current vapers had significantly higher prevalent risk of asthma attack, but no such effects was seen in dual users or former smoker current vapers.	Age groups: 18-29 years: n=38972, 30-39 years: n=36020, 40-49 years: n=34884, 50-59 years: n=38713, 60+ years: n=70322.	Asthma attack Age 18-29: current vapers had higher risk of asthma episode/attack ($p<0.05$) and ER visit due to asthma ($p<0.05$) compared to current vapers of 60+ years.<br><br>Age 30-39: current vapers had higher risk of ER visit due to asthma ($p<0.05$) and NS difference in asthma episode/attack compared to current vapers of 60+ years.	Low (6/8)

								NS difference in asthma episode/attack and ER visits due to asthma were observed between current vapers of age 40-49 or age 50-59 and current vapers of age 60+.	
Austin-Datta et al., 2023;[37] US	Independent funding organization; none	N/A	N=7,253; ≥18 years age; 36.9% male, 63.1% female; 48.4% Black/AA, 44.4% White, 7.2% other races, 92% non-Hispanic, 8% Hispanic.	Never vapers (n=6076): Never used EC in lifetime.<br><br>Ever vapers (n=1,177): Used EC in their lifetime.	Asthma, COPD, respiratory infection (pneumonia, sinus infection); reversible and irreversible	Asthma:<br>Odds of ever use was NS in those with asthma compared to those not having any asthma.<br><br>COPD:<br>Compared to those not having COPD, those with COPD had significantly higher odds of being ever vapers (aOR 2.0).<br><br>Respiratory infection:<br>Compared to those not having any other respiratory infection, those with respiratory infection had significantly higher odds of being ever vapers (aOR 1.3).<br><br>Interpretation: Ever vapers had higher probability of prevalence of COPD and respiratory infection, but no higher prevalent risk detected for asthma.	N/A	N/A	Moderate (5/8)
Dirisanala et al., 2023;[58] US	None; none	N/A	N = 178,300; >18 years age; 47.63% male, 52.37% female; 35.72% NH White, 20.6% NH	Ever vapers (*N* = 7756): Used EC in their lifetime.<br><br>Ever smokers (*N* =	Asthma, COPD; reversible; irreversible	Prevalence of asthma<br>Ever vapers and ever dual users had significantly higher odds compared to never users (p= 0.0001, p<0.0001	N/A	N/A	Low (8/8)

			Black, 13.02% NH Asian, 15.1% Hispanic American, 11.29% other Hispanic, 4.26% multiracial.	48,625): Smoked >100 Cigs in their lifetime or currently smoked Cig.<br><br>Ever dual users (*N* = 23,444): Ever used both Cig and EC.<br><br>Never users (*N* = 98,475): Did not use EC or Cig in their lifetime.		respectively).<br><br>Prevalence of COPD:<br>Ever vapers and ever dual users had significantly higher odds compared to never users (p<0.0001 for both).<br><br>Interpretation: Ever vapers and ever dual users had higher probability of prevalent asthma and COPD compared to never users.			
Chandra et al., 2023;[52] US	Independent funding organizations; some (no pro-tobacco association)	N/A	N=94; 45–80 years old with > 10 pack-years of current or prior smoking; 44.7% male, 55.3% female; 67% Caucasian, 33% AA.	Non-menthol flavoured EC users (n=69),<br><br>Menthol flavoured EC users (n=25)<br><br>NS difference in age, sex, race, and current smoking between groups	Impairment of lung function, respiratory symptoms, COPD; reversible and irreversible	FEV1, %predicted:<br>Non-menthol flavored EC users had significantly higher FEV1 compared to menthol flavored EC users (p=0.05).<br><br>FEV1/FVC, bronchodilator response, spirometric stage:<br>NS difference between groups was seen.<br><br>% emphysema, % gas trapping (Chest CT):<br>NS difference between groups was seen.<br><br>MMRC dyspnea score and St George's Respiratory Questionnaire (respiratory symptoms):<br>NS difference between groups was seen.<br><br>Interpretation: Menthol and non-menthol flavored EC users did not differ by impact on lung function, obstructive changes in lung, and respiratory symptoms.	N/A	N/A	Low (14/20)
Moyers et al., 2023;[95] US	Independent funding organizations;	N/A	N=29,482; mean age 48.61 (SD 18.79) years;	Non-users (n=24,319): Dis not use either EC or Cig currently,	COVID-19 Infection and Severity; reversible	Self-reported COVID-19 infection:<br>Non-smoker current vapers	N/A	N/A	Low (7/8)

	some (no pro-tobacco association)		48.42% male, 51.59% female; 63.17% NH White, 16.8% Hispanic, 11.48% NH Black, 5.88% NH Asian, 1.35% NH American Indian/Alaskan Native, and 1.32% other race/ethnicity.	Non-smoker current vapers (n=758): Used EC everyday or some days,<br><br>Non-vaper current smokers (n=3,049): Used Cig everyday or some days,<br><br>Dual users (n=351): Used both Cig and EC everyday or some days.		had significantly higher odds (aOR 1.30) compared to non-users.<br>NS difference found between dual users and non-users.<br><br>Self-reported COVID-19 severity:<br>NS difference between different severity status between groups.<br><br>Interpretation: Non-smoker current vapers had significantly higher probability of COVID-19 infection, but no effect of EC exposure on infection severity was found.			
Williams et al., 2023;[141] US	Independent funding organization; none	N/A	N=150,634; Mean age was 16.2 years (SD=1.1); 51.7% female; 53.5% Hispanic, 20.7% NH White, 12.5% NH Asian, 2.7% NH Black, 2.4% NH other race and 8.2% NH Multiracial.	Never Users (N=95,359): Did not use any tobacco products in lifetime,<br><br>Non-smoker current vapers (N=7,441): Used only EC in past 30 days.<br><br>Non-vaper current smokers (N=698): Used only Cig in past 30 days.<br><br>Dual users (N=565): Used both Cig and EC in past 30 days.	Asthma, asthma severity; reversible	Self-reported prevalence of lifetime diagnosis of asthma:<br>Non-smoker current vapers had significantly higher odds ($p<0.01$) and dual users had significantly lower odds ($p<0.05$) of lifetime asthma compared to never users.<br><br>Self-reported asthma attack in past 12 months:<br>NS difference was seen between never users and non-smoker current vapers or dual users.<br><br>Interpretation: Non-smoker current vapers had higher prevalent risk of asthma, while NS risk of asthma severity was seen among them or dual users.	N/A	N/A	Low (7/8)
Kelesidis et al., 2023;[78] US	Independent funding organization; none	N/A	N=104; mean age 18-27 years; male (n=67), female (37); White (n= 63), Asian (n= 24),	Nonusers (N=45): Did not use either Cig or EC in past 1 year.<br><br>Non-smoker current	COVID-19 infection; Reversible	Plasma levels of ACE2, Ang 1–7, Ang II, and IL-6 (needed for viral entry):<br>NS differences found between groups.	N/A	N/A	Moderate (11/20)

			AA (n= 5), Hispanic (n= 8), Native Hawaiian (n= 2).	vapers (N=30): Used EC for >1 years, but did not use Cig.<br><br>Current smokers (N=29): Smoked Cig for >1 year.		Plasma levels of ADAM17 shedding activity (predict morbidity):<br>Non-smoker current vapers had significantly lower levels compared to current smokers ($p<0.001$).<br><br>Plasma levels of Furin (protease that increase affinity of the SARS-CoV-2 spike protein for ACE2):<br>Non-smoker current vapers had significantly increased levels compared to nonusers ($p<0.001$) and current smokers ($p=0.037$).<br><br>Interpretation: Non-smoker current vapers might have increased risk of COVID-19 infection than non-users and current smokers, but the risk of severity is lower than current smokers.			
Shields et al., 2023;[122] US	Independent funding organizations; some (no pro-tobacco association)	N/A	N=28; 21-30 years age; male (n=19), female (n=9); White (n=24), AA/Asian (n=4).	Nonusers (N=10): Smoked less than 100 Cigs in their lifetime and had not smoked or vaped in the last 1 year.<br><br>Non-smoker current vapers (N=10): Used EC daily for ≥6 months and had not smoked a cigarette for ≥1 year.<br><br>Non-vaper current smokers (N=8): Smoked ≥10 cigarettes/day for at least 6 months and had not used EC within 1	Pulmonary inflammation; Not measured	Inflammatory cell count in BALF:<br>Undifferentiated M0 macrophages were significantly increased and M2 macrophages significantly decreased in non-smoker current vapers compared to nonusers ($p<0.01$) but the level was lower than non-vaper current smokers.<br><br>Inflammatory Gene Expression Analysis:<br>There were 19, and 1 significantly differentially expressed inflammatory genes (DEG) between non-vaper current smokers/non-smoker	N/A	N/A	Low (14/20)

				year.		current vapers, and non-smoker current vapers/non-users respectively.<br><br>Interpretation: Non-smoker current vapers showed significant pulmonary inflammatory changes compared to non-users.			
Buu et al., 2023;[45] UK	Independent funding organization; None	N/A	N = 686; mean age ~19 years; all used EC at least once per week in the last 4 weeks	Frequent cannabis users (n = 247): used cannabis daily, almost daily or weekly, mean age 19.70; 46.2% male; 74.1% White.<br><br>Infrequent/non-cannabis users (n = 439): Used cannabis monthly or less than monthly, mean age 19.89; 50.3% male; 67.7% White.	Respiratory symptoms; not measured	Respiratory symptoms at American Thoracic Society Questionnaire (ATSQ): Compared to infrequent/non marijuana users, frequent users had significantly higher frequencies of coughing 1st thing in the morning (p=0.02), cough frequently throughout the day (p=0.008), wheezing (p=0.017), shortness of breath during exercise/walking upstairs (p=0.050), phlegm/mucous when coughing (p<0.001), pain or tightness in the chest (p=0.037), and got very tired in a short time (p=0.002).<br><br>Interpretation: Prevalence of respiratory symptoms were higher among current vapers who concurrently used cannabis frequently.	N/A	N/A	Low (6/8)
Shiffman et al., 2023;[123] UK	JUUL Labs, many (pro-tobacco association)	N/A	N=264; mean age ~ 45-years; 42.4% Male; 87.9% White.	Former smoker current vapers (N= 140): former smokers who switched completely to JUUL 5.0% nicotine ENDS for ≥ 6 months, and did not use cigarette or OTPs.<br><br>Non-vaper current smokers (N=124):	Respiratory symptoms; not measured	Respiratory symptom experience scale (RESS): Former smoker current vapers had significantly lower RESS score compared to non-vaper current smokers (p<0.0001).<br><br>Interpretation: Former smoker current vapers had significantly lower prevalence of respiratory symptoms compared to non-vaper current	N/A	N/A	Low (15/20)

				Smoked ≥10 cigarettes per day for ≥10 years and had not used other tobacco or nicotine-containing products in the past 30 days.		smokers.			
Pozuelos et al., 2022;[110] US	Independent funding organization(s); none	N/A	N=9; Age <50 years; 100% female, 100% White	NS (n=3): undetectable urine cotinine at <0.25 ng/ml.<br><br>EC (n=3): urine cotinine levels of at least 400 ng/ml<br><br>CS (n=3): urine cotinine levels of at least 400 ng/ml.<br><br>RNA was extracted from nasal epithelial biopsy from each group.	Respiratory inflammation and damage; Not measured	Gene expression profiles associated with EC use are not equivalent to those from non-smokers. EC use may interfere with airway epithelium recovery by promoting increased oxidative stress, inhibition of ciliogenesis, and maintaining an inflammatory response. So, long-term use of ECs may contribute to the progression of several respiratory disease.<br><br>Interpretation: EC exposure may lead to increased susceptibility to respiratory inflammation and damage.	N/A	N/A	Low (15/20)
**Case reports / Case series**									
Kligerman et al., 2021;[82] US	None; none	Not specified for nicotine vapers (N/A)	N=160; 15-68 years old; mean age 28.2 years; male (n=127, 79.4%), female (n=33, 20.6%); vaped at least 12 months (n=61).	THC vapers (n=77, 48.1%): Mean age 29.4 ± 10.9 years,<br>Nicotine vapers (n=15, 9.4%): mean age 33.6 ± 13.9 years,<br>THC + Nicotine vapers (n=68, 42.5%): mean age 25.5 ± 10.3 years.	EVALI; Not measured	CT chest:<br>THC + nicotine vapers had significantly higher number of moderate severity EVALI (n=41) than THC vapers (n=28) and nicotine vapers (n=6) (P<0.05).<br>NS difference between groups in terms of mild or severe severity of EVALI.<br>NS difference between groups in terms of parenchymal attenuation (including ground glass opacity), parenchymal sparring, centrilobular nodules, septal thickening, lymphadenopathy and pleural effusions.	N/A	N/A	Low (7/10)

						NS difference between groups for risk of diffuse alveolar damage (DAD) pattern, acute eosinophilic pneumonia (AEP)-like pattern, diffuse alveolar haemorrhage (DAH) pattern, parenchymal organizing pneumonia (OP) pattern and % of lung involvement.			
Tanz et al., 2021;[134] US	Not specified; none	Not specified, possible short-to medium term (90 days)	N=78; 13-72 years, 63% male; 15% had pre-existing respiratory disease. Analytic sample (N=20).	N=20 were interviewed.<br>E-liquid samples collected N=96 from 10 cases.	EVALI; Not measured	Most of the cases were used THC vaping products (n= 16); of them exclusive THC users were n=3; daily users were n=12.<br>Nicotine vaping was reported by n=14; of them exclusive nicotine users were n=2, daily users were n=12.<br>CBD vapers were n=6; of them exclusive CBD users n=0.<br>THC and Nicotine vapers were n=10<br><br>E-liquid laboratory test: THC/CBD/cannabinol: n=74 samples.<br>Nicotine: n=7 samples.<br>Vitamin E acetate: n=54 samples, all also contained THC.<br>Tarpenes: n= 54, Glycerol: n=17, Menthol: n=5,Arsenic: n=1, Barium: n=96, Cadmium: n=96, Lead n=96, Thalium n=3, Uranium n=2 samples.	N/A	N/A	Low (7/10)
Gupta et al., 2022;[68] US	Not specified; some (no pro-tobacco association)	Not specified (probably short-medium term- at least 3 months)	N=14; 16-45 years; female (n=4, 29%) male (n=10, 71%)	Exclusive Nicotine vapers (n=5): Male (n=3), female (n=2), age 16-39 years.<br>Nicotine + THC vapers (n=4): Male (n=3), female (n=1), age 16-27 years.	EVALI; Not measured	Clinical feature: (n=14) had respiratory symptoms (chest pain, cough, or shortness of breath) and >50% had gastrointestinal symptoms (nausea, vomiting, abdominal pain, and diarrhea). Other symptoms- fever, tachycardia,	N/A	N/A	Moderate (5/10)

				Exclusive non-nicotine vapers (n=1): Male (n=1), 45 years.<br>Exclusive cannabis vapers (n=3): Male (n=2), female (n=1), age 16-24 years.<br>Unknown vapers (n=1): Male (n=1), 19 years.		tachypnea, and/or hypoxemia.<br><br>CXR: (n=13) bilateral pulmonary infiltrate, (n=3) pneumothorax. One of the pneumothorax case was exclusive THC users.<br><br>Primary infection screening: Negative.<br><br>Primary echocardiogram: (n=7) had normal ventricular function, (n=5) had ventricular dysfunction.			
Helfgott et al., 2022;[71] US	Independent funding organization(s); none	Long-term (Case 1: 1 year, Case 2: 2-5 years)	N=2, 17 years old male.	Case 1 - Vaped every other day for 1 year (nicotine + occasional THC)<br>Case 2 - Vaped daily for 5 years (Nicotine + THC)<br><br>Smoking history: not specified	EVALI; Not measured	Clinical feature:<br>Fever, cough, shortness of breath, hypoxaemia, tachycardia, SpO2 91% for case 1, 84% for case 2.<br><br>CXR: bilateral infiltrates.<br><br>Chest CT: Multifocal opacities throughout the lung (Case 2 has no chest CT report).<br><br>Infectious screening: negative.	N/A	N/A	Low (6/8)
Guarino et al., 2022;[67] Italy	None; none	Long-term (5 years)	N=1; 59 years female; had a previous 40 pack-year smoking history; vaped tobacco flavoured EC with nicotine content 20 mg/ml, PG/VG ratio 50:50, 8 hours/day for every day for 5 years.	N/A	EVALI; Not measured	Clinical feature: progressive dyspnea on exertion and daily cough, basal bilateral reduction of breath sound, no crackles, SpO2 95%.<br><br>HRCT chest: Consolidation in middle lobe with non-calcific bilateral nodules. After 18 months, halo sign (RHS), patchy ground glass opacity (GGO), pleuro-parenchymal bands, and indeed perilobular pattern, suggestive of organizing pneumonia (OP).<br><br>BALF cytology: 95%	N/A	N/A	Moderate (5/8)

						macrophages, accumulation of lipids in the cytoplasm of the alveolar macrophages.<br><br>FEV1 = 1.39, FVC = 2.3 L, FEV1/FVC = 58.47%, indicating obstructive ventilatory defect<br><br>Infection screening: negative			
Pan et al., 2022;[105] Taiwan	Not specified; none	Long-term (4 years)	N=1; 15 years old male; used e-cigarettes for 4 years and smoked cigarettes for weeks	N/A	EVALI; Reversible	Clinical feature: abdominal pain, productive cough, dyspnea, SpO2 88% on room air.<br><br>CXR and CT chest: bilateral multiple infiltrates with sparing of left upper lobe. Day 8 CXR showed no infiltrative nodules.<br><br>Bronchoscopy: hypervascularity of mucosa in bronchus, bronchial trees<br><br>BALF: no pathogen/malignant cells detected.	N/A	N/A	Moderate (4/8)
Hoekstra et al., 2021;[74] US	Not specified; none	Not specified (N/A)	N=1; 17 years old male, used JUUL pods and cannabis every 2-3 days. History of smoking not specified.	N/A	EVALI; Not measured	Clinical feature: acute hypoxaemia.<br><br>BALF: negative lipid index, grossly bloody fluid, return of 217,500 RBCs/mm3.<br><br>Carbon monoxide diffusing capacity (DLCO): markedly elevated DLCO 201% predicted<br><br>Infection screening: negative	N/A	N/A	Low (6/8)
Chan et al., 2021;[51] Australia	Not specified; none	Short to medium term (7 months)	N=1; 15 years old female; vaped 5% Nicotine EC 300puffs every 3 weeks, smoked	N/A	EVALI; Not measured	Clinical feature: Febrile, dysuria, urinary frequency, back pain, vomiting, rigors, HR 120-140 bpm, BP 110/80 mm of Hg, RR 20 breaths/min,	N/A	N/A	Moderate (5/8)

			cigarette for last 7 months, used cannabis through waterpipes for last 4 months.			SpO2 92-95% dropping to 87%.<br><br>CXR: Bilateral pulmonary infiltrates that were worse in the lower zones.<br><br>CT chest: Extensive pulmonary infiltrates in the lung bases with bilateral effusions.<br><br>Urine drug screen: Positive for cannabinoids and benzodiazepines.<br><br>Infection screening: Negative.<br><br>E-liquid analysis: Contained glycerol, nicotine, flavouring agents ethyl-maltol and menthol. THC or vitamin E acetate was not detected.			
McCormick et al., 2022;[90] US	None; none	Not specified (N/A)	N=1; 44 year old female; was former smoker current daily vaper.	N/A	Pulmonary Langerhans cell histiocytosis (PLCH); Reversible	Clinical feature: Left chest wall pain<br><br>CT Chest: numerous, small, and bilateral nodules with ground-glass appearance. The largest nodule was in the right upper lung lobe.<br><br>PFT: unremarkable.<br><br>Infection screening: negative.<br><br>Lung histopathology: PLCH in the upper and lower lobes, with CD1a staining highlighting the aggregates of Langerhans cells.	N/A	N/A	Moderate (5/8)
Casamento Tumeo et al., 2022;[48] Italy	None; none	Not specified (N/A)	N=1; 15 years old Caucasian female; concurrent smoking (10 cigarettes/day);	N/A	EVALI; Not measured	Clinical feature: Severe dyspnea with SatO2 75%, HR 120 bpm, bilateral wheezes with prolonged expiration on	N/A	N/A	Low (7/8)

			was former vaper current smoker (last e-cigarette use 1 month prior to hospital admission).			lung auscultation.<br>Chest CT: a central ground glass pattern with peripheral sparing.<br>Total IgE: High, 419 kU/l<br>PFT: restrictive pattern, no bronchodilator reversibility.<br>Bronchial exhaled (eNO): Low, 3 ppb (normal reference value:<20 ppb) and nasal eNO was slightly reduced.<br>The 6 minute walking test (6MWT): showed slightly reduced functionality.			
Triantafyllou et al., 2021;[136] US	Not specified; none	Not specified (N/A)	N=41; median age 21 years; 78% males, 22% females; 83% White, 10% Black; 29% had underlying lung disease, specifically asthma, chronic obstructive pulmonary disease, or cystic fibrosis; history of smoking and THC vaping not specified.	N/A	Long-term outcome of EVALI; Reversible	Mortality:<br>2 patients with substantial underlying medical comorbidities died after hospitalization, with a 1-year all-cause mortality rate of 4.9%.<br><br>Radiological imaging:<br>18 (44%) of 41 patients had 1 year follow-up radiographical imaging. 12 (67%) of these 18 patients had complete resolution and four (22%) had improvement in initial radiographical abnormalities. Two (11%) patients showed focal pulmonary scarring.<br><br>PFT:<br>12 (29%) of 41 patients had pulmonary function testing performed after initial hospital admission; nine (75%) of these 12 patients had abnormal results (restrictive ventilatory defect, mixed obstructive/restrictive defect).	N/A	N/A	Low (8/10)
Smith et al., 2022;[126] UK	Not specified; none	Acute/Single exposure	N=1; 14 years old healthy female; first time vaped 48 hrs.	N/A	EVALI; Reversible	Clinical feature: cough, chest discomfort, abdominal pain, and rigors, needed ventilation.	N/A	N/A	Low (6/8)

			prior to symptom onset; urine drug screen: negative for THC; e-liquid composition not specified.			CXR: diffuse interstitial markings with bilateral patchy nodular infiltrates, progressing to bilateral consolidation and pleural effusions.<br>CT chest: centrilobular nodules consistent with diffuse alveolar damage, intra-lobular septal thickening, and moderate bilateral pleural effusions.<br>BALF analysis: RBC/blood positive.<br>PFT: restrictive picture with FEV1 and FVC of 1.77 L (55% predicted) and 2.07 L (57%).<br><br>Findings reversed within 2 months.			
Collins et al., 2022;[53] UK	Not specified; none	Short to medium term (3 months)	N=1; 18 years male; used mainly nicotine containing vaping products, occasionally with cannabidiol (CBD); smoking status not specified	N/A	EVALI; Not measured	Clinical feature: progressive dyspnea and cough, severe respiratory failure requiring intubation.<br>Chest CT: Widespread "tree-in-bud" nodularity with more confluent apical lesions and lower lobe consolidation.<br>BAL: 70% fat-laden macrophages on oil-red O staining and less than 1% eosinophils, but was negative for infectious causes.	N/A	N/A	Moderate (5/8)
Khan et al 2022;[80] UK	Not specified; none	Short to medium term (Few months)	N=1; age in 40s; female; had past medical history of asthma; was a moderate alcohol drinker: type of EC and smoking history not specified.	N/A	EVALI; Reversible	Clinical feature: Fever, cough, shortness of breath and confusion, bilateral crackles.<br>CXR: Showed widespread, bilateral pulmonary infiltrates, consisting of a mixture of ill-defined and nodular opacities.<br>CT Chest: Patchy bilateral lung consolidation involving all lobes along with enlarged bilateral hilar and mediastinal nodes.	N/A	N/A	Moderate (5/8)

						After 2 weeks, CT chest was clear.<br>Infection screening and culture: negative.			
Roman et al., 2021;[114] US	Not specified; none	Not specified (N/A)	N=1; 31 years old male; history of smoking 10 cigarettes/day for 18 years and quit smoking 11 months ago; vaped cannabis oils and nicotine 3-5 times/day; recurrent history of hospital admission for pneumonia.	N/A	EVALI; Reversible	Clinical feature: Fever, cough, shortness of breath, chest tightness, hypoxia with SpO2 of 85% on room air.<br>CXR: bilateral generalized infiltrates.<br>CT chest: Extensive bilateral alveolar infiltrates with peripheral sparing, septal thickening with crazy paving pattern.<br>Lung biopsy: cryptogenic organizing pneumonia (COP).<br>Infection screening: negative.	N/A	N/A	Low (6/8)
Takigawa et al., 2022;[133] Japan	Not specified; none	Acute (4 days)	N=1; 18 years old male; used a non-nicotine non tar EC; was not a smoker (never smoker current vaper); no history of bronchial asthma	N/A	Vaping associated acute eosinophilic pneumonia (AEP); Reversible	Clinical feature: Fever, dyspnea, anorexia, tachycardia, SpO2 94% on 2 L/min, Coarse bilateral lung crackles.<br>CXR and CT scan: Thickening of the bronchovascular bundles and interlobular septa in both lungs. In right upper lobe, a granular shadow, nodules and peripheral ground-glass opacity, right mild pleural effusion.<br>BALF analysis (confirmatory): 64% eosinophils and 18% lymphocytes.<br>PFT: FEV1 1.56 L (low), VC 1.9L (low), PEF 2.79 L/min (low), DLCO 12.03 ml/min/mmHg (low).	N/A	N/A	Low (6/8)
Soybel et al., 2022;[130] US	Not specified; none	Short-to-medium-term (1 year)	N=1, 50 year old white female; smoked 20 Cigs/day for >30 yrs.	N/A	Sarcoidosis in lung; Non-reversible	Clinical feature: Resting pulse oximeter found O2 saturation at 94%; with ambulation, O2 saturation levels fell to 80% after 1-min of walking, necessitating 2 L of nasal cannula to rebound to a pulse	N/A	N/A	Low (8/8)

						oximeter reading of 96%.<br>CTA scan: diffuse ground-glass opacities, scattered pulmonary nodules, and hilar (1.8 cm) and subcarinal (1.7 cm) lymphadenopathy. Prior CTA of the chest 3 years prior (obtained due to a motor vehicle accident), there were no signs of lymphadenopathy or ground-glass opacities.<br>Diagnosis:<br>Extensive differential for pulmonary non-necrotizing granulomatous inflammation. Considering the patient's pathological findings, cell count findings on BAL, and imaging, sarcoidosis was diagnosed. Assuring a documentation of e-cigarette usage and its potential correlation, association, and/or causation of an established pulmonary illness should be on the minds of all healthcare professionals.			
Kubbara et al., 2021;[84] US	Not specified; none	Long-term (10 years)	N=1; 67 year old male; 10 year vaping history (nicotine used 6mg/mL; 0.6% on daily basis), vaped until the night prior to elective lobectomy for lung cancer.	N/A	Diffuse alveolar haemorrhage (DAH) from H. influenzae pneumonia.; Reversible	Clinical feature: Respiratory rate 28 (tachypnea), and SpO2 was 90% despite 100% oxygen at a rate of 55 L/min, reduced air entry bilaterally with biphasic crepitations and wheezing.<br>CXR: g diffuse alveolar infiltrates suggestive of alveolar haemorrhage.<br>PT, APTT and platelet count: Normal<br>CBC: Hb was chronically reduced at 120g/L.<br>BALF analysis: progressive increase in blood-tinged return, confirming DAH diagnosis. Gram stain of the	N/A	N/A	Moderate (5/8)

						BALF showed few Gram-positive cocci; the final culture report showed heavy growth of H. influenzae.<br>Chest CT: bilateral patchy infiltrates in keeping with alveolar haemorrhage.			
Norman et al., 2023;[99] Spain	Independent funding organization(s); none	Not specified (N/A)	N=1; 33 years old male; vaper of electronic e cigarettes for unknown period of time, changed brands of e cigarettes used recently, did not use OTPs or cannabis.	N/A	EVALI; reversible	Clinical feature: progressive shortness of breath, 89% SpO2, fever.<br><br>Chest X ray: bilateral pulmonary infiltrates.<br><br>CT chest: patchy ground glass opacities and interstitial lung involvement<br><br>High peripheral eosinophil count: peak at 2000 cells/ul<br><br>Negative for viral, bacterial/atypical and fungal pathogens in nasopharyngeal samples, sputum, BAL, blood, serum	N/A	N/A	Low (7/8)
Harry-Hernandez et al., 2023;[69] US	Not specified; not specified	Not specified (vaped within 90 days of symptom onset)	N=21; 16-68 years old; 57.1% male, 42.9% female; 76.2 % White, 23.8% Black.	THC only vapers (n=14, 66.7%),<br>Nicotine only vapers (n=4, 19.0%),<br>THC + Nicotine vapers (n=2, 9.5%).	EVALI; Not measured	CT chest: diffuse lung injury of predominantly ground glass opacities (GGO), consolidation, or mix of GGO + consolidation.<br><br>Interpretation: Prevalence of EVALI was lower among nicotine EC users.	N/A	N/A	Low (8/10)
Sezgin et al., 2023;[120] Turkey	Not specified; None	Short-to-medium-term (2 months)	N=1; 55 years old female; had a 30-packs/year smoking history but had switched to E-cigarettes around 2 months earlier, had pneumonia 8 months earlier.	N/A	Acute eosinophilic pneumonia (AEP); Reversible	Clinical Features: Shortness of breath, dry cough, fever.<br><br>Chest X ray: Revealed bilaterally-dispersed infiltration.<br><br>CT-chest: Nodular-shaped focal consolidations and blurry glass densities, accompanied	N/A	N/A	Low (7/8)

						by interstitial thickening were observed in both lung parenchyma.<br><br>PFT: FEV_1 100%, FEV_1/FVC 78%.<br><br>BALF: 80% eosinophil was detected.			
Gaba et al., 2023;[61] India	None; None	Short-to-medium-term (4 months)	N=1; not specified (in 20's), male; smoked 3-4 Cigs per day for the last 2 years, vaped with nicotine ECs for the past 4 months, no history of cannabis use.	N/A	EVALI; Reversible	Clinical Features: Shortness of breath, Chest examination had bilateral equal air entry with no added sounds.<br><br>CXR: Symmetrical diffuse hazy alveolar opacities, most apparently in bilateral lower zones<br><br>CT pulmonary angiogram: No evidence of pulmonary embolism, there was ground-glass attenuation in the lung window, which was symmetrical, diffuse, and centrilobular within the middle and lower pulmonary lobes.<br><br>H1N1 and COVID-19: negative	N/A	N/A	Low (7/8)
Lucas et al., 2023;[87] Brazil	None; None	Short-to-medium-term (9 months)	N=1; 43 years old Male, had been using a Juul 5% flavored EC at a rate of 1 cartridge every 4 days, twice a day, for at least 9 months, no history of smoking Cigs.	N/A	EVALI; reversible	Clinical Features: Fever, chills, coughing yellowish phlegm, dyspnea, and wheezing.<br><br>COVID-19: negative<br><br>CXR: Diffusely distributed pulmonary micronodules<br><br>CT chest: Diffuse pulmonary nodules of centrilobular distribution predominantly in the upper lobes.	N/A	N/A	Low (7/8)

Alsaid et al., 2023;[34] Saudi Arabia	None; none	Short-to-medium term (20 days)	N=1; 17 years old female; non-smoker; recently started vaping; wasn't on any medications	N/A	Acute eosinophilic pneumonia (AEP); reversible	Clinical feature: Sudden onset of shortness of breath, left sided chest pain, fever.<br><br>BALF analysis: High eosinophilic count (84%)<br><br>CBC: WBC count normal with 14% eosinophils, otherwise normal differential count<br>CXR: bilateral lower lung zones airspace opacities with subtle left-sided pleural effusion.<br>CT Chest: Bilateral ground glass opacities in periphery.<br><br>COVID-19 negative.	N/A	N/A	Low (6/8)
**Cell/*in vitro* studies**									
Wick et al., 2022;[140] US	Independent funding organization(s); some COI (No pro-tobacco association)	Acute; 3 days	Human alveolar AT2 cells<br><br>5 male donors (age at least 36 years; n=3 Hispanic Latino; n=3 were non-smokers)	Control: unexposed cells.<br>PG/VG: Exposed to 50:50 LG:VG mixture for 20 min and 60 min over 3 days.<br>Nic-EC 20 min: exposed to mint flavoured JUUL pod containing 0.7 mL of 5% nicotine salt e-liquid or ~59 mg/mL aerosol over 20 min/day for 3 days.<br>Nic-EC 60 min: exposed to mint flavoured JUUL pod containing 0.7 mL of 5% nicotine salt e-liquid or ~59 mg/mL aerosol over 60 min/day for 3 days.<br><br>Power of JUUL pod was1.5 ohms and	Respiratory inflammation and damage; Not measured	Lactate dehydrogenase (LDH) and multiplex protein analysis: There was a small but significant increase in the LDH concentration in AT2 cells after 20 or 60min of exposure to VG/PG aerosols. NS difference between JUUL exposed AT2 cells and control.<br><br>Inflammatory markers: Significant increase in MCP-1 after 60 min of daily Juul exposure as compared with 20 min ($p<0.05$), but there was NS difference between 60 min and control conditions.<br><br>Gene expression: Individual genes upregulated by Juul exposure included several involved in the response to oxidative and metabolic stress and fatty acid metabolism and	N/A	N/A	Moderate

				power of 9.4 W<br>All groups infected with H1N1 PR8 strain after 3 days exposure and incubated for 24-48 hrs.		biotransformation. Individual genes downregulated by Juul exposure are crucial to the innate immune response.<br><br>Interpretation: Juul exposure may increase respiratory inflammation while reducing innate immune signaling capacity.			
Caruso et al., 2021;[47] Italy	Independent funding organization(s); many COI	Acute; Single exposure	Human bronchial epithelial cells (NCI-H292)	Whole smoke:<br>Exposed to 2, 5, 10, 12, 15, 20, 25, or 30 puffs of cigarette smoke or vapour delivered at 35 ml puff volume, 2 s duration, and every 60s. Cigarette vapour exposure was achieved by positioning a Cambridge filter pad in line.<br><br>Nic-EC Exposed to 10 puffs from Vype ePen 3 e ("Master Blend" flavour with 18 mg/mL nicotine) and 25 puffs from Vype eStick ("Toasted Tobacco" flavour and 18 mg/mL nicotine). EC aerosol was delivered at 5 mL puff volume, 2s duration, every 30 s with 1 s button pre-activation for each puff and held at a 45° angle (mouthpiece up) to reflect consumer use.<br><br>HTPs: Exposed to 8 puffs from Neostick (glo PRO) and to 7	Respiratory inflammation and damage; Not measured	Cytotoxicity assay:<br>No toxicity to lung cells from EC aerosol.<br><br>Cell viability assay:<br>NS effect was seen in exposure to EC aerosol.<br><br>Overall: nicotine EC exposure did not cause cytotoxicity.	N/A	N/A	High

				puffs from Heets (IQOS 3 DUO).					
Komura et al., 2022;[83] Japan	Independent funding organization(s); none	Acute; 4 hours	Primary human small airway epithelial cells (SAECs) from healthy donors and COPD-SAECs from COPD patients	Control SAECs (PG:Gly 0:0)<br>PG-treated SAECs (1-4% PG)<br>VD-treated SAECs (1-4% VG)<br>COPD-PG (1-4% PG) treated SAECs	Respiratory inflammation and damage; Not measured	Cell proliferation and cell viability:<br>SAECs exposed to PG significantly inhibited proliferation (1%, PG, p=0.021; 2-4% PG, p<0.0001) and decreased cell viability (1-4% PG, p<0.0001) in a concentration-dependent manner. 2-4% PG affected cell proliferation more significantly than 2-4% VG. Cell viability as measured by CCK-8 decreased significantly after PG exposure in a concentration-dependent manner. 2-4% VG also decreased cell viability, but to a lesser extent than 2-4% PG. Cell proliferation and cell viability were significantly greater in COPD-SAECs. Cell surface area of COPD-SAECs was significantly decreased following exposure with 2% PG. Furthermore, COPD-SAECs showed significant inhibition of cell viability based on CCK-8 with 1%-3% PG.<br><br>LDH release:<br>Increased significantly with 3-4% PG exposure only in a concentration-dependent manner (3% PG, p=0.0055; 4% PG, p<0.0001).<br><br>Interpretation: 4% PG can cause significant airway epithelial damage.	N/A	N/A	High
Schaunaman et al.,	Independent funding	Acute; 3 days	SAEC (Small airway epithelial	Nic-EC + Influenza A Virus (IAV): SAECs	Respiratory infection; Not	Transepithelial electrical resistance (TEER) to measure	N/A	N/A	High

2022;[118] US	organization(s); none		cells) from human donors (n=6; 18-57 years old; 83.33% males, n=5 were never smokers, n=1 was former smokers).	exposed to 'Virginia Tobacco' 3% nicotine (35mg/ml) and PG:VG = 30:60 given by 15 puffs over 15 min, 1/day for 3 days. After 3 days, SAECs were exposed to IAV infection for 3 days.<br><br>SAECs + Influenza A Virus (IAV) alone: SAECs were exposed to IAV infection for 3 days.	measured	epithelial barrier function/integrity: Significantly reduced in both groups with NS difference between them.<br><br>Inflammatory markers and gene expression: IL-8, CXCL10 and IL-8 mRNA expression were significantly increased in SAECs +JUUL + IAV compared to SAECs + IAV alone (p=0.013, p=0.019 and p=0.032 respectively). CXCL10 mRNA expression increased in SAECs + IAV group, with no additional effect noted in the SAECs + JUUL + IAV. A significant increase of IFN-β protein and mRNA expression, MX1 expression were seen in SAECs + JUUL + IAV group compared to SAECs + IAV infection alone (p=0.027).<br><br>Overall: EC exposure amplifies human distal airway pro-inflammatory response to IAV infection.			
Wang et al., 2021;[138] China	Independent funding organization(s); many (association with vape industry)	Acute; single exposure	Human lung epithelial cells (BEAS-2B)	DMSO (control): treated with 0.1% DMSO<br>Nic-EC condensate (ECSC): (10 µg/ml); prepared from 40 mg/g nicotine menthol flavoured EC.<br>Cigarette smoke condensate (CSC): (0-32 µg/ml) prepared from 10 mg tar and 1 mg nicotine per cigarette.	Respiratory damage; Not measured	Cell viability by CCK-8 assay and morphology: IC50 value was more than 32 µg/ml (nicotine contained) for ECSC. NS change in cell morphology with ECSC exposure.<br><br>Inflammatory cytokines On exposure to 10µg/ml of ECSC, the mRNA levels of inflammatory cytokines IL-1α (p<0.001), IL- 1β (p<0.001), TNF-α (p<0.001) increased	N/A	N/A	High

						significantly.<br><br>Apoptosis:<br>NS changes was seen in ECSC compared to control.<br><br>Gene expression:<br>ECSC weakly affected the genes regulation. Compared to ECSC, CSC could induce cell cycle arrest at the S/G2/M phase on BEAS-2B cells.<br><br>Overall: Nicotine EC exposure caused inflammatory reaction but NS cytotoxic effects.			
Baldovinos et al., 2022;[38] US	Independent funding organization(s); none	Acute; Single exposure	Bronchial epithelial cells (BEAS-2B) and adenocarcinomic alveolar epithelial cell A549.	Individual exposure to triethyl citrate: Exposed to only e-liquid ingredient- diluent (triethyl citrate)<br><br>Individual exposure to limonene: Exposed to only e-liquid ingredient- terpene (Limonene)<br><br>Binary exposure to 97:3 v/v triethyl citrate/limonene: Exposed to e-liquid ingredient- diluent (triethyl citrate) and terpene (Limonene) at 97% and 3% concentration<br><br>Binary exposure to 80:20 v/v triethyl citrate/limonene: Exposed to e-liquid ingredient- diluent (triethyl citrate) and terpene (Limonene) at	Lung damage; Not measured	LC50 values using Methyltetrazolium salt (MTS) assay:<br>Individual exposure to limonene was found to be more cytotoxic than triethyl citrate in both BEAS-2B and A549 cells.<br><br>Binary exposure to 97:3 v/v or 80:20 v/v triethyl citrate/limonene mixture were less cytotoxic than individual exposure to either limonene and triethyl citrate. Increased concentration of limonene, meaning 80:20 v/v triethyl citrate/limonene was more cytotoxic than 97:3 v/v triethyl citrate/limonene.<br><br>Interpretation: lung cytotoxicity is mainly mediated by terpene (limonene) in e-liquid.	N/A	N/A	High

				80% and 20% concentration					
Sinha et al., 2022;[125] US	Not specified; none	Acute; Single exposure	Human bronchial epithelial cell line (BEAS-2B), NCI-H460 cells (lung cancer cells), and human monocyte-macrophage cell line (THP-1)	Control: PG:VG -60:40 (0mg/ml nicotine)<br>Positive Control: cigarette smoke extract<br>Smoothol Flavoured "menthol" (0mg/ml nicotine)<br>Nic-EC (6mg/ml nicotine): delivered with menthol or without menthol<br>Nic-EC (12mg/ml nicotine): delivered with menthol or without menthol<br>Flavors: French Vanilla, Vanilla Custard, Rainbow Candy, Vanilla	Respiratory inflammation; not measured	BEAS-2B cell growth:<br>was significantly reduced for Nic-EC + menthol exposure in a dose-dependent manner, however, NIC-EC alone did not have an appreciable impact on BEAS-2B cells.<br><br>Flavour analysis in e-liquid: Rainbow Candy and 'Vanilla' showed a moderate decrease in BEAS-2B cell growth compared to PG:VG alone. Compared to positive control, 1% aerosolized e-liquids were much more cytotoxic.<br><br>Inflammatory markers in THP-1 derived macrophages: Compared to unflavoured PG/VG, menthol flavoured e-liquid significantly reduced TNF-B levels ($p<0.05$), moderately decreased IL-8 levels ($p>0.05$), significantly reduced IL-6 levels ($p<0.05$) and reduced MCP-1 levels ($p<0.05$). When combining all four inflammatory response markers (TNF- α, IL-8, IL-6, and MCP-1) there was a significant reduction in combined inflammatory response by 1% aerosolized nic-EC + menthol exposure.<br><br>Interpretation: Flavored nicotine EC exposure induced higher cytotoxic effects in lung cells compared to nicotine only EC. Flavored EC exposure was associated with reduced inflammatory	N/A	N/A	High

						reaction.			
Morris et al., 2021;[93] US	Not specified; none	Acute (Single exposure)	Human bronchial epithelial cells (BEAS-2B) and both naïve and activated macrophages (THP-1) cell lines	Control: BEAS-2B and THP-1 were exposed to 1% PG/VG 50:50 vehicle for 4 and 24 hrs.<br>30 flavouring chemicals: BEAS-2B and THP-1 were exposed to 10uM, 100uM and 1000uM of 30 flavouring chemicals for 4 and 24 hrs.	Pulmonary inflammation; Not measured	Cell viability:<br>At 1000 ÂµM, the greatest reductions in viability were seen with decanal, hexanal, nonanal, cinnamaldehyde, eugenol, vanillin, alpha-pinene, and limonene.<br>Effect at 24 hr was greater than 4 hr.<br><br>Inflammatory cytokine release: Naive THP-1 cells produced significantly elevated levels of IL-1Î², IL-8, and TNF-Î± when exposed to ethyl maltol and hexanal. Activated THP-1 cells released increased IL-1Î² and TNF-Î± when exposed to ethyl maltol.<br>Effect at 24 hr was greater than 4 hr.<br><br>Interpretation: Flavoured EC exposure induced significant cytotoxicity and inflammation.	N/A	N/A	Low
Ghosh et al., 2023;[64] US	Independent funding organization(s); none	Acute (6hrs and 2hrs)	Human bronchial epithelial culture (HBEC) and Human peripheral blood neutrophils (HPBN)	Experiment 1: HBEC exposed for 6hrs to BALF from smokers, vapers, and non-smokers<br><br>Experiment 2: HPBN was exposed for 2hrs to one of the 5 exposures below and treated HPBN was added to HBEC:<br>Control,<br>Juul-menthol e-liquid 0%,<br>Juul-Menthol e-liquid 1%,<br>Juul-Menthol e-liquid	Airway dehydration (cystic fibrosis, COPD, asthma)	Epithelial Sodium channel (ENaC) activity and airway surface liquid (ASL) height: ENaC was significantly increased and ASK height was significantly decreased on exposure to vapers BALF compared to control. This effect was similar to the effect seen on exposure to smokers BALF. NS difference was found between exposure to non-smokers BALF and control. ENaC cleavage was found on exposure to vapers BALF and E-liquid-treated HPBN ($p<0.05$).	N/A	N/A	High

				3%,<br>Juul - Blank.		Interpretation: EC causes similar airway dehydration to smoking which can have implications for COPD, asthma, and cystic fibrosis.			
Raduka et al., 2023;[111] US	Independent funding organization(s); none	Acute; 24hrs	Human bronchial epithelial cells (16HBE), normal human bronchial epithelial cells (NHBE)	Both 16HBE and NHBE were exposed to 6 types of aerosol:<br>Control: Exposed to air,<br>Nic-Ec: Exposed to unflavored 36 mg/ml nicotine EC aerosol,<br>Menthol+Nic-EC Exposed to menthol flavored 36 mg/ml nicotine EC aerosol,<br>Cinnamon+Nic-EC: Exposed to cinnamon flavored 36 mg/ml nicotine EC aerosol.<br><br>And 2 types of EC extract:<br>2.5% PG/VG: exposed to 50%/50% PG/VG extract without nicotine,<br>Multiple concentrations of Nic-EC extract: Exposed to 0.5-10 mM concentration of nicotine with 2.5% PG/VG extract.<br><br>All EC aerosol exposure was prepared from e-liquid of 50%/50% PG/VG and aerosol exposure was given in 4 puffs/min lasting for 3s, with a volume of 100 mL, for 24 hrs. EC extract	Respiratory infection; not measured	Airway epithelium barrier integrity measured by Transepithelial electrical resistance (TEER):<br>All Nic-EC extract (0.5-10mM concentration) and 2.5% PG/VG induced significant reduction ($p<0.0001$) in TEER compared to control.<br>Menthol + Nic-EC and Cinnamon + nic-EC induced significant reduction ($p<0.05$) in TEER compared to Nic-EC exposure.<br><br>Airway epithelium barrier dysfunction measured by apical junctional complexes (AJC):<br>2.5% PG/VG induced significant disruption of AJC ($p<0.0001$) compared to control. NS difference was seen between Nic-EC extracts and control.<br>Menthol + Nic-EC and Cinnamon + nic-EC induced significant disruption of AJC compared to control.<br><br>RSV induced airway damage: significantly increased ($p<0.05$) following pre-exposure with Nic-EC aerosol compared to non-exposed RSV infected cells.<br><br>Interpretation: Nicotine containing EC exposure disrupted the airway epithelial	N/A	N/A	High

				exposure was given directly to cell lines for 24 hrs.		barrier and exacerbated RSV-induced damage			
Begum et al., 2023;[41] US	Independent funding organization(s); none	Acute (24hrs)	Human alveolar epithelial cells (A549) cells	Control: Exposed to filtered air,<br>PG/VG: Exposed to non-nicotine tobacco-flavored (TF) EC vapor condensate (ECVC) containing 35%/75% PG/VG,<br>Nic-EC: Exposed to tobacco-flavored (TF) EC vapor condensate (ECVC) containing 35%/75% PG/VG with nicotine (6 mg/ml).<br><br>Exposure was given as 3s/puff interrupted by 20s of air.	Respiratory inflammation; not measured	Inflammatory markers: IFN-γ, TNF-α, IL-6, IL-8, CCL2, CCL5, inducible proteasome subunits (LMP7/PSMB8, LMP2/PSMB9, MECL1/PSMB10) levels significantly increased ($p<0.01$) in both PG/VG and Nic-EC exposure compared to control.<br><br>Interpretation: EC exposure both with and without nicotine caused significant pulmonary inflammatory reaction.	N/A	N/A	High
Phandthong et al., 2023;[108] US	Independent funding organization; none	Acute (24hrs)	Human bronchial epithelial (BEAS-2B) cells	3 types of exposure given: submerged treatments, aerosol exposure in cloud chamber, aerosol exposure via Cultex exposure system<br><br>In Submerged treatments, e-liquid was directly applied to cell culture medium, exposure was given as: Control (air), Nicotine (either 0.03% or 0.3%), PG/VG (either 0.5% PG/VG (30:70), 0.5% PG/VG + 0.03% nicotine, or 0.5% PG/VG + 0.3% nicotine), and 0.5% JUUL.	COVID-19 infection; not measured	Angiotensin converting enzyme 2 (ACE2) levels-SARS-CoV-2 receptor:<br>In submerged treatments and Cutex exposure system, Nicotine and PG/VG + Nicotine significantly increased ACE2 levels ($p<0.05$) compared to control. NS effect was seen in cloud chamber and JUUL exposure.<br><br>Transmembrane serine protease 2 (TMPRSS2) activity- enzyme essential for viral entry:<br>Significantly increased in all 3 types of treatment in exposure to Nicotine, PG/VG + Nicotine, and JUUL compared to control. Significant increase ($p<0.05$) was also noted in PG/VG + Nicotine, and JUUL	N/A	N/A	High

				In aerosol exposure in cloud chamber, exposure was given as: Control (PBS), Nicotine (either 0.03% or 0.3%).<br><br>In aerosol exposure via Cultex exposure system, exposure was given as:<br>Control (air), PG/VG, PG/VG + Nic in either 6mg/ml or 60 mg/ml nicotine, and JUUL.		compared to PG/VG alone.<br><br>Interpretation: Acute exposure to nicotine containing EC increased susceptibility to COVID-19 infection.			
Rasmussen et al., 2023;[112] US	Independent funding organizations; some (no pro-tobacco association)	Acute (single exposure)	Human bronchial epithelial (HBEC) cells from donors with wild type cystic fibrosis transmembrane conductance regulator (CFTR) and no history of respiratory disease and using Cig or EC.	Control: Exposed to air<br>PG/VG: Exposed to EC aerosols from a 1:1 mixture of PG/VG,<br>Nic-EC: Exposed to 1.8% nicotine containing PG/VG EC aerosol.<br><br>Exposure was given as 8-s puffs of 35 mL/min for 10 min.	Respiratory infection (Cystic fibrosis), COPD; irreversible	CFTR and epithelial sodium channel (ENaC)-mediated ion transport:<br>was significantly reduced in Nic-EC exposure compared to control ($p<0.05$).<br>NS difference was seen between PG/VG and control.<br><br>Interpretation: Nicotine EC exposure might increase susceptibility to respiratory infection and COPD, but not non-nicotine EC exposure.	N/A	N/A	Low
Effah et al., 2023;[59] UK	Independent funding organization; none	Acute (single exposure)	Human bronchial epithelial cells (HBEC-3KT)	Control: media only control,<br>PG/VG alone: Exposed to non-flavored 60%/40% PG/VG containing e-liquids at 0.25%, 0.5% and 1% (v/v),<br>Flavored PG/VG: Exposed to 15categories of flavored 60%/40% PG/VG containing e-liquids at 0.25%, 0.5% and 1% (v/v).	Respiratory inflammation; not measured	Cell count:<br>was significantly reduced in Cinnamon, hazelnut, and vanilla tobacco flavored PG/VG exposure compared to PG/VG alone and control ($p<0.0001$) in a dose-dependent manner.<br><br>Cell viability:<br>was significantly reduced in Cinnamon flavored PG/VG exposure compared to PG/VG alone and control ($p<0.0001$) in a dose-dependent manner.	N/A	N/A	High

						LDH release:<br>Significantly increased in Cinnamon flavored PG/VG exposure compared to PG/VG alone and control ($p<0.0001$) in a dose-dependent manner.<br><br>Interpretation: Cinnamon, hazelnut and vanilla tobacco flavored non-nicotine EC exposure increased cytotoxicity and respiratory inflammation.			
**Animal studies**									
Zhang et al., 2023;[148] China	Independent funding organization(s); none	Short to medium term (10 weeks)	N=50; 6 weeks old C57BL/6 mice; Female n=25, male n=25	Control: n=5 males, n=5 females; exposed to filtered clean air for 2h per day.<br><br>Nic-EC low dose: n=5 males, n=5 females; 5% nicotine, and 5% benzoic acid for 1 h per day.<br><br>Nic-EC: n=5 males, n=5 females; 5% nicotine, and 5% benzoic acid for 2 h per day.<br><br>C-cig low dose: n=5 males, n=5 females; 1R6F cigarette smoke for 1 h per day.<br><br>C-cig high dose: n=5 males, n=5 females; 1R6F cigarette smoke for 2 h per day.	Impaired lung function, COPD, pulmonary inflammation measured	Spirometry parameters:<br>All spirometry parameters except FEV0.1 and FVC changes were significant in EC high dose compared to control group ($p<0.05$).<br>EC low dose had significant changes compared to control ($p<0.05$) only in TV and MVV.<br>NS difference in changes between C-cig low dose and EC low dose.<br><br>Inflammatory cell infiltration in lung:<br>Increased alveolar macrophages, neutrophils and 8-OHdG (oxidative lesions) ($p<0.05$) was seen in EC high dose. EC low dose had increased alveolar macrophages.<br><br>Lung and small airway morphometry:<br>EC high dose had increased airway wall thickness, but not mean linear intercept or alveolar airway size; and no	Sex: Male mice (n=25), female mice (n=25)	NS difference in between male mice and female mice.	High

						airway remodelling was seen in EC low dose.<br><br>Interpretation: High dose nicotine e-cigarette exposure lead to respiratory inflammation and impaired lung function compared to controls, but no significant airway remodelling, particularly obstructive changes were seen.			
Goto et al., 2022;[66] US	Independent funding organization(s); none	Acute (single exposure)	N=48; 12-14 weeks old C57BL/6J male mice	Control: exposed to air,<br><br>0-puff aged coil: Exposed to non-nicotine EC aerosol with a new coil,<br><br>900-puff aged coil: Exposed to non-nicotine EC aerosol with a 900 puff aged coil,<br><br>1800-puff aged coil: Exposed to non-nicotine EC aerosol with a 1800 puff aged coil.<br><br>*E-liquid was a mixture of PG/VG 50:50 ratio, no nicotine or flavour added. Puffs were given at 2 puffs/min, 100 ml/puff for 1 hour.	Lung inflammation and damage; not measured	Lung tissue histology: Exposure to 1800 puff aged coil caused significant acute lung injury ($p<0.001$), increase in lung wet/dry ratio ($p<0.0001$), increase in lung inflammatory cytokine levels-IL6 and MIP-2 ($p<0.001$) and mRNA expression of IL-6, TNF-α, IL-1β, MIP-2 ($P<0.0001$) compared to control, 0 puff and 900 puff aged coil.<br><br>Respiratory rate and SpO2: A significant decrease ($p<0.001$) was observed in 1800 puff aged coil exposure compared to control and 0 puff aged coil exposure.<br><br>Interpretation: Acute e-cigarette exposure caused acute lung injury as well as increased inflammatory cytokine levels. The 1800 puff-aged device caused more of a decrease in lung function measures than the younger device (900 puffs).	N/A	N/A	Moderate
Zhang et al., 2021;[149] US	Independent funding organization(s);	Acute (3 days)	N=77; 6–8-week-old C57-BL/6J male and female	Experiment 1:<br>Nic-EC +KP (n=4): Received intranasal	Respiratory inflammation, respiratory	Lung wet/dry ratio: Increased lung wet/dry ratio in Vape +KP compared to KP	N/A	N/A	High

	none		mice	2mg/ml nicotine e-liquid for 3 days followed by infected with K. pneumonia on 4th day.<br>PG/VG + KP (n=4): Received intranasal PG:VG 50:50 without nicotine e-liquid for 3 days followed by infected with K. pneumonia on 4th day.<br>KP only (n=4): Were infected with K. pneumonia<br>Control (n=4): Received intranasal saline for 3 days without being infected with K. pneumonia.<br><br>Experiment 2:<br>Pyr6+ Nic-EC treated (n=6): Received intranasal Ca2+ channel antagonist Pyr6 (10 µl) 30 min prior intranasal 2mg/ml nicotine e-liquid for 3 days.<br>Pyr10 + Nic-EC treated (n=6): Received intranasal Ca2+ channel antagonist Pyr10 (10 µl) 30 min prior intranasal 2mg/ml nicotine e-liquid for 3 days.<br>2-APB + Nic-EC treated (n=6): Received intranasal Ca2+ channel antagonist 2-APB (10 µl) 30 min prior intranasal 2mg/ml nicotine e-liquid for 3	infection; Not measured	only ($p<0.05$).<br>NS difference between PG/VG + KP and KP only.<br><br>Inflammatory cytokine analysis in BALF:<br>Increased TNF-α, IL-6 and Mip-1α and , IL-17 levels in Vape +KP and PG/VG + KP compared to control ($P<0.01$).<br>Decreased IL-6 and IFN-γ levels in Pyr6+ vape treated, Pyr10 + vape treated, 2-APB + vape treated groups compared to control + vape treated group ($p<0.05$) with highest effect seen in 2-APB + vape treated for IL-6.<br><br>BALF cytology:<br>Increased alveolar macrophages (AM) and decreased interstitial macrophages (IM) Pyr6+ vape treated, Pyr10 + vape treated, 2-APB + vape treated groups compared to control+ vape treated group.<br>Neutrophil population increased in control+ vape treated group infected with K. Pneumoniae, but not in Pyr6 or Pyr10 or 2-APB + vape treated groups infected with K. Pneumoniae.<br><br>Interpretation: Acute vaping exacerbates microbial pneumonia due to calcium (Ca2+) dysregulation and increases respiratory inflammation.			

				days.<br>Control + vape treated (n=6): Received no drug treatment only intranasal 2mg/ml nicotine e-liquid for 3 days.<br>Mock (n=6): Received no drug treatment and no vaping.<br><br>KP: Klebsiella pneumoniae					
Daou et al., 2021;[56] Lebanon	Independent funding organization(s); none	Acute (7 days)	N=64; 4 months old FVB-Tg mice (diabetic and non-diabetic) female mice	Diabetic + EC (n=8), Non-diabetic + EC (n=8): Exposed to 18mg/ml nicotine content EC aerosol for 3 hrs., two times daily, total 6 hrs/day.<br><br>Diabetic + HTP (n=8), Non-diabetic + HTP (n=8): Exposed to IQOS, for 3 hrs., two times daily, total 6 hrs/day.<br><br>Diabetic + CS (n=8), Non-diabetic + CS (n=8): Exposed to 3R4F cigarettes, 9.4 mg tar, and 0.726 mg nicotine per cigarette for 3 hrs., two times daily, total 6 hrs/day.<br><br>Diabetic + Control (n=8), Non-diabetic + Control (n=8): Exposed to air.	Respiratory inflammation and damage; Not measured	Lung histopathology, wet/dry lung ratio"<br>Increased edema and inflammatory cells in lung tissue in non-diabetic mice exposed to EC compared to controls ($p<0.05$).<br><br>Albumin in BALF:<br>NS effect on EC exposure.<br><br>Gene expression of inflammatory mediators:<br>Significant increase in TNF-α, IL-6 and IL-1β was observed with diabetic and non-diabetic mice exposed to EC compared to controls ($p<0.01$).<br><br>Interpretation: Diabetes (and other comorbidities) may exacerbate the effects of nicotine-containing e-cigarettes. EC exposure resulted in respiratory inflammation and damage.	N/A	N/A	High
Esquer et al., 2022;[60] US	Independent funding organization(s); none	Short to medium term (9 weeks)	N=20; 4-wk-old male and female C57BL/6J mice	Control (n=10): mice not exposed to vape<br><br>PG/VG (n=10): mice	Respiratory inflammation, COPD; Not measured	Lung parenchyma and airways morphology and histology:<br>Average free distance between alveolar walls significantly	N/A	N/A	High

				exposed to Peach Ice 70VG/30PG (ORGNX) flavoured vape juice<br><br>*delivered as e-vapour from JUUL pens in whole- body exposure chambers for 4h/day, 5 d/week for 9 wks.		increased in vape, p<0.05.<br>Bronchiole wall thickness significantly increased in vape, P<0.01<br>elastic fiber disorganization in airways in vape increased collagen deposition surrounding bronchioles, vasculature, parenchyma in vape lungs.<br><br>Inflammatory activity in lung: CD11b, CD11c cell count significantly increased in vape, p<0.01<br>Inflammatory cytokine expression of IL 6, IL 1, box protein 1 increased in vaped lung.<br><br>Transcriptome profiling: Significant changes to gene families coding for xenobiotic response, glycolipid metabolic processes, and oxidative stress. E Cadherin levels significantly increased in vaped lungs, P<0.01.<br><br>Interpretation: Profound pathological changes to upper airway (emphysematous changes), lung tissue architecture, and cellular structure are evident following EC aerosol exposure.			
Yang et al., 2022;[144] China	Independent funding organization(s); many COI (association with vape industry)	Short to medium term (10 weeks)	N=32; 8 weeks old male C57BL/6 mice.	Control (air-exposed),<br><br>Nic-EC low-dose group (ECAL): exposed to nicotine 6 mg/kg EC aerosol for 1 h per day, 5 consecutive days per week for 10 weeks.	Impairment of lung function, pulmonary inflammation; Not measured	Lung function:<br>Enhanced pause (Penh) was significantly decreased after ECA exposure compared to CS exposure (p<0.05).<br>NS changes in respiratory rate, 50% exhalation force (EF50), and ventilation per minute (MV) were observed after	N/A	N/A	Low

				Nic-EC high-dose group (ECAH): exposed to nicotine 12 mg/kg EC aerosol for 2 h per day, 5 consecutive days per week for 10 weeks.<br><br>CS-exposed group (CS): exposed to nicotine 6 mg/kg cigarette smoke for 1 h per day, 5 consecutive days per week for 10 weeks.		ECA exposure.<br><br>Inflammatory responses:<br>NS changes in KC and G-CSF levels were seen after ECA exposure. Compared with ECA exposure, KC was elevated nearly 0.5-fold and C-GCF was increased more than 4fold after CS exposure ($p<0.05$).<br><br>Proteomic analysis:<br>Greater overall protein changes by CS than that of ECA, with more severe inflammatory network perturbations ($p<0.05$). Protein–protein interactions (PPI) showed that ECA significantly changed ribosome and complement system-related proteins in mouse lung tissue.<br><br>Interpretation: EC exposure was associated with significantly lower impact on lung function and lung morphology and inflammation than cigarette exposure.			
Onyenwoke et al., 2022;[101] US	Independent funding organization(s); none	Short to medium exposure (5 weeks).	Mice (C57-BL/6J).	Mice:<br>Vehicle control: 50:50 PG/VG<br>Nic-EC (50:50 PG/VG +33mg/mL nicotine)<br>Nic-EC "Mint" (3% nicotine)<br>Control (Saline 10uL)<br>Exposure was given as intranasal delivery into lungs once daily for 5 weeks. In each group - after 5 weeks, half of the mice were sedated	Respiratory inflammation; Not measured	Mice BALF: A notable and statistically significant increase in IL-6 was observed with the e-liquid-treated and MHV infected mice ($p<0.05$), most prominent effect seen in 'mint' exposure ($p<0.01$).<br><br>Interpretation: E-liquid exposure alone results in pro-inflammatory lung responses in the C57BL/6J model.	N/A	N/A	High

				and infected with MHV-A59 (coronavirus) while others were euthanized for BAL.					
Getiye et al., 2022;[63] US	Independent funding organization(s); none	Short to medium term (6 months)	N=39; 2 months old male, C57BL/6 wild type (WT), caspase recruitment domain-containing protein 9 (CARD9)-/-, and NOD-like receptor family pyrin domain-containing 3 (NLRP3)-/- mice	Control: Air exposed.<br><br>Nic-EC with heat (EwH): Exposed to 6 mg/ml nicotine, PG/VG 7.5%:17.5%, raspberry- flavoured EC aerosol by heating the e-liquid for 3 h/day, 5 days/week for 6 months.<br><br>Nic-EC without heat (EwoH): Exposed to 6 mg/ml nicotine, PG/VG 7.5%:17.5%, raspberry- flavoured EC aerosol without heating the e-liquid for 3 h/day, 5 days/week for 6 months.	Respiratory inflammation; Not measured	Immunofluorescence staining of CD68+ macrophages: Increased in both EwH and EwoH exposure compared to control (p<0.01).<br><br>Plasma cytokines and chemokines:<br>Significantly increased level of IL-1α±, IL-6 (p<0.001), G-CSF, MIP-1α±, Eotaxin compared to control in WT mice on EwH exposure.<br>Significantly decreased level of IL-1α± (p<0.0001), IL-1β, IL-10, IFN-gamma (p<0.001), TNFα± (p<0.001), G-CSF, MIP-1α± (p<0.001), MIP-1β (p<0.001), RANTES, Eotaxin compared to control in CARD9-/- mice.<br>Significantly activated TGF-β1/SMAD2/3/α±-SMA fibrosis signalling in the lungs of the WT mice but not in the RV, CARD9-/- and NLRP3-/- mice.<br><br>Interpretation: EC aerosol exposure following EwH or EwoH induced pulmonary inflammation and remodelling.	N/A	N/A	Low
Moshensky et al., 2022;[94] US	Independent funding organization(s); none	Short to medium term (4-12 weeks)	6-8 weeks old female C57BL/6 mice	Randomized to-<br>Air (control) (room air only)<br>Nic-EC JUUL Mint (5% nicotinic salts in mint flavour)<br>Nic-EC JUUL Mango (5% nicotinic salts in	Respiratory inflammation; Not measured	Lung inflammatory gene expression: Exposure to JUUL Mango aerosols led to 155 significant gene expression changes, while Mint JUUL aerosols led to 74. The genes whose expression was most significantly altered included	N/A	N/A	Low

				mango flavour)<br><br>Exposure was given 20 mins at a time, three times daily, for a total of 60 min per day, for 4-12 weeks; harvest occurred 30 minutes after the last exposure.		GTPases (nicotine), mucins (mint flavour), CCL6 (mango flavour), and TGF-β receptors (nicotine and mango flavour).<br><br>Mechanic scan for physiology: NS changes in lung physiology at 1 or 3 months.<br><br>Interpretation: JUUL exposure increased changes in lung inflammatory gene expression.			
Shi et al., 2022a;[121] US	Independent funding organization(s); none	Acute (single exposure)	N=80; 5 months old Sprague-Dawley rats; 40% male, 50% female.	Control: (n=16) exposed to pure air<br>PG/VG SS high power (n=16): Stainless steel was used as heating element, in a high powered ENDS (70 W)<br>PG/VG Stainless steel (n=16): Stainless steel was used as heating element in a low powered ENDS (45 W)<br>PG/VG NC high power (n=16): Nickel chromium was used as heating element, in a high powered ENDS (70 W)<br>PG/VG Nickel-chromium (n=16): Nickel chromium was used as heating element, in a low powered ENDS (45 W)<br><br>In all EC exposure, aerosol was produced from 50%/50% PG/VG tobacco flavored e-liquid.<br>Exposure was given for 4 hrs.	Respiratory inflammation; Not measured	Lung histology:<br>EC groups had accumulation of inflammatory cells (mainly macrophages) in bronchial lumen, near the pleura, and within the alveolar space. The numbers of inflammatory cells per field in the lung parenchyma were significantly greater in the rats exposed to EC using SS or NC heating element compared to the air control group ($P < 0.05$).<br><br>Inflammatory gene expression: IL-1β, IL-6, IL-10, IFN-γ and endothelin-1 gene expression were significantly increased in SS EC exposure at 70W power (SS70) vs control. In NC70 group, IL-6, endothelin-1 and ACE2 gene expression were significantly up-regulated compared to control<br><br>Interpretation: Vaping caused significant lung inflammation compared to control and the effect was significantly higher with stainless steel or nickel cadmium heating element or high power device.	N/A	N/A	Moderate

Been et al., 2023;[39] Canada	Independent funding organization(s); none	Short to medium term (4 weeks)	8-12 weeks old male and female C57BL/6J mice	Control group: Exposed to air.<br><br>Nic-EC JUUL group: exposed to 59mg/ml nicotine, mango flavoured JUUL product<br><br>PG/VG group: Exposed to 30:70 ratio of PG/VG e-liquid<br><br>Exposure was given as 1 puff/minute, 78 ml puff volume, 2.4s puff duration, 20 min exposures per day for 4 weeks (equivalent to 3 years in human)	Respiratory inflammation; Not measured	BALF:<br>Significant increase in neutrophil population in both JUUL and PG/VG groups compared to control group. NS change was seen in macrophages.<br>NS change in BAL cytokines (G-CSF, IL_2, IL-15, IP-10, MIG, MIP-2, VEGF) was seen in either groups compared to control.<br><br>Lung tissue:<br>NS change in inflammatory cells (macrophage, neutrophil, lymphocytes, CD8+ T4 cells) in lung tissue in either JUUL or PG/VG group.<br><br>Gene expression:<br>Significant increase in RNA expression of Ace2 and antioxidant genes Nqo1 and Sod2. in both groups and IL6 in JUUL group.<br><br>Proteomic and transcriptomic analysis: Significant changes in numerous biological pathways including neutrophil degranulation, PPAR signalling, and xenobiotic metabolism in both groups.<br><br>Interpretation: JUUL exposure, both nicotine and non-nicotine, caused significant pulmonary inflammation.	N/A	N/A	Moderate
Orzabal et al., 2021;[102] US	Independent funding organization(s); none	Short to medium term (16 days)	N=18; 6-8 weeks old pregnant Sprague Dawley rats.	Control (n=6): pair-fed control group exposed to room air;<br>PG/VG (n=6): a pair-fed group exposed to e-	Lung development In Utero; Irreversible	AA profiling in foetal lung: The AAs altered in the both male and female foetal lungs of EC-Nic group compared to control were aspartate (↑),	Sex: male and female rats foetal lung	Same as study findings of entire population.	Moderate

				cig aerosols without nicotine, 80:20 PG/VG 3hrs per day, 5 days per week from gestation day (GD) 5-20. n=6<br><br>Nic-EC (n=6): a group exposed to e-cig aerosols containing nicotine, 80:20 PG/VG and either 5% (50 mg/mL) nicotine during acclimatization or 10% (100 mg/mL) nicotine for 3hrs per day, 5 days per week from gestation day (GD) 5-20.n=6		glutamate (↑), asparagine (↑), threonine (↑), citrulline (↑), valine (↑), isoleucine (↑), and ornithine (↑) ($p<0.05$); for only male foetal lungs arginine (↑), methionine (↑), leucine (↑) ($p<0.05$); and for only female foetal lungs was glutamine (↑) ($p<0.05$).<br>The only AA found to be significantly different in foetal lungs of EC-base compared to control was threonine (↑) ($P<0.05$) for both male and female foetal lungs and alanine (↑) ($P<0.05$) for only female foetal lungs.<br>The only AA found to be significantly different in male and female foetal lungs of EC-Nic compared to EC-base was ornithine (↑) ($p<0.05$).<br><br>Interpretation: Gestational EC exposure can cause significant biological changes in foetal lung.			
Alzoubi et al., 2022;[35] Jordan	Independent funding organization(s); none	Short to medium term; 1, 2 or 4 weeks	N=24; 6-8 weeks male mice (Balb/c)	Control (room air),<br><br>PG/VG (80:20 PG:VG): for 3 hours per day, 5 days per week from gestational day (GD) 5-20, excluding GDs 9,10,16, and 17.<br><br>*Nic-EC (80:20 PG:VG + 5 or 10 mg/ml nicotine): 3 hours per day, 5 days per week from gestational day (GD) 5-20, excluding GDs 9,10,16, and 17.	Respiratory inflammation; Not measured	Inflammatory cells and markers:<br>Significant increase in total inflammatory cells, eosinophils, macrophages and TNFα after 1,2, and 4 week exposure.<br>Significant decrease in IL-10 and increase in neutrophils and basophils after 1 week exposure.<br><br>Interpretation: Vaping can cause significant lung inflammation.	N/A	N/A	Moderate

				*EC aerosol was given by 0.4 ohm Mesh Z1 coil attached to a 65 W power source at 1 sec puff of ~ 42 mL every 20 seconds to give a total of 540 puffs per episode.					
Orzabal et al., 2022;[103] US	Independent funding organization(s); none	Short to medium term (12 days)	N=36 (18 foetal and 18 neonatal) offspring of Sprague Dawley rats<br><br>Control (air)<br>PG/VG (80:20)<br>Nic-EC	Control (room air)<br>PG/VG (80:20)<br>Nic-EC	Lung development in utero; Irreversible	RNA assessment:<br>Significant alterations to the transcriptome of EC-Nic and EC indicate that chemical constituents other than nicotine in EC aerosols may have a negative effect on foetal lung gene expression.984 genes were significantly downregulated and 2322 genes that were significantly upregulated in the EC-Nic group compared to control.7 genes were significantly downregulated and 41 genes were significantly upregulated in the EC group compared to control.<br><br>Neonatal lung morphology:<br>Fixed lung weight was significantly decreased in the EC-Nic group compared to control (p=0.004) and EC (p=0.01) groups.<br>Mean linear intercept (MLI) in the EC-Nic group was significantly increased compared to control (p=0.009) meaning increased free space in lung parenchyma. Radial alveolar count (RAC) in the EC-Nic group was significantly decreased compared to Control (p=0.002) and EC (p=0.019) groups.	N/A	N/A	Low

						Morphologic changes in the EC-Nic group are consistent with an emphysematic phenotype corresponding to fewer and larger distal air spaces.<br><br>Neonatal pulmonary mechanics: Area of the pressure volume loop on lung function assessment was lower among EC and EC-Nic group ($p<0.05$). NS difference in resistance, compliance, elastance between groups.<br><br>Interpretation: prenatal EC exposure may result in altered lung function, emphysematous changes or development of lung diseases later in life.			
Aslaner et al., 2022;[36] US	Independent funding organization(s); none	Short-to-medium term (entire gestation- 18-20 days)	N=~10-12; 5 months parent mice	Control group: In-utero exposure to filtered air for entire gestation.<br><br>Nicotine EC exposed group: In-utero exposure to EC vapour with 2% Nic and 50:50 PG:VG ratio for entire gestation.<br><br>Non-nicotine EC exposed group: In-utero exposure to e cigarette vapour with 50:50 PG:VG ratio for entire gestation.	Lung development in utero; irreversible	PFT:<br>Significantly decreased lung compliance on PFT among male mice in nicotine EC exposed group and in both male and female mice in non-nicotine Ec exposed group.<br>In non-nicotine EC exposed group, decrease in FEV0.1 in female mice compared to control was observed.<br><br>Lung histology:<br>Significantly increased collagen deposition/ increased stiffness was seen around the vessels/airways and in alveolar tissue in both groups among males and females.<br>Compared to control, significant goblet hyperplasia was observed in female mice in nicotine EC exposed group and in both male and females	Sex: male and female mice	Same as study findings of entire population.	High

						in non-nicotine EC exposed groups.<br><br>Interpretation: EC exposure increases risk of lung dysfunction, pulmonary fibrosis, goblet cell hyperplasia.			
Hassan and El-Wafaey, 2022;[70] Egypt	None; none	Short-to-medium term (4 weeks)	N=20; adult male Wistar albino rat	Control Group: n=10, exposed to fresh air; EC group: n=10. exposed to 1 ml/day e-liquid; exposure was given as 11 cycles within 1 hr. (17 s puff (6s on, 5s off, 6s on) followed by 20 min stop) a day for 5 consecutive days a week for 4 consecutive weeks	Respiratory inflammation and lung damage; Not measured	Lung weight:<br>EC group had significantly decreased lungs weights ($P < 0.005$) than controls.<br><br>Lung histopathology:<br>Increase in lung injury degree as oedema, hyperaemia, thickened inter-alveolar septum, cellular infiltration, loss of alveolar epithelium and haemorrhage ($p < 0.0001$).<br><br>Interpretation: Exposure to e-liquid with nicotine induced significant lung damage.	N/A	N/A	Low
Hinds et al., 2022;[73] US	Independent funding organization(s); none	Acute (2 days)	N=48; 6-8 weeks old male Lakeview Golden (LKG) Syrian hamsters (Mesocricetus auratus)	Control: (n=16) exposed to room air;<br><br>PG/VG + 0% nicotine: (n=16).<br><br>PG/VG + 2.5% nicotine: (n=16).	Respiratory inflammation; Not measured	Gene expression:<br>In the lung, nicotine-dependent increases in the expression of genes involved in the renin-angiotensin pathway [angiotensin-converting enzyme (ACE),ACE2], coagulation (tissue factor,Serpine-1), extracellular matrix remodelling (MMP-2,MMP-9), type 1 inflammation (IL-1b,TNF-a,andCXCL-10),fibrosis (TGF-bandSerpine-1), oxidative stress response (SOD-2), neutrophil extracellular traps release (ELANE), and vasculogenesis and angiogenesis (VEGF-A) were found.	N/A	N/A	High

						Interpretation: Acute exposure to e-cigarette increased susceptibility to respiratory inflammation.			
Suryadinata et al., 2021;[132] Indonesia	Not specified; none	Short to medium term (30 days)	Male Wistar rats	Control group: only food without any exposure to e-cigarette aerosol.<br>Experimental group: was given food and exposed to e-cigarette aerosol for two minutes daily during the 4-week period of the experiment.	Respiratory inflammation; Not measured	In the experiment group, the number of positive cells from malondialdehyde, IL-8, IL-10 and the MMP-8 matrix were significantly higher than those in the control group. Positive cells of type-2 collagen in the control group were significantly higher than those in the experiment group. T-test results exhibited a difference in the number of alveolar macrophages between both groups (P <0.001), where the experiment group (53.26 ± 0.93) had a greater number of positive cells compared to the control group. The results of the correlation test uncovered a strong association between malondialdehyde, IL-8, IL-10, matrix MMP-8, type-2 collagen and alveolar macrophages present in lung tissue (r >0.80).<br><br>Interpretation: The results showed that exposure to e-cigarette smoke caused an increase in free radicals, triggered an inflammatory process and degraded the type-2 collagen present in the lung tissue.	N/A	N/A	High
Been et al., 2022;[40] Canada	Independent funding organization(s); none	Acute (3 days)	Male and female C57BL/6J mice; 8-12 weeks old	Mango flavoured JUUL: exposed to mango flavoured 59 mg/ml nicotine JUUL puff.<br><br>Mint flavoured JUUL:	Respiratory inflammation; Not measured	Inflammatory markers and cells:<br>Significant increase of lung mRNA expression of IL13 and IL10 (except mint flavoured JUUL) compared to control. No significant change in lung	N/A	N/A	Moderate

				exposed to mint flavoured 59 mg/ml nicotine JUUL puff.<br><br>Virginia tobacco flavoured JUUL: exposed to Virginia tobacco flavoured 59 mg/ml nicotine JUUL puff.<br><br>PG/VG: exposed to control liquid made of 30:70 ratio of PG and VG<br><br>Control: Exposed to air.<br><br>Mice were exposed for 20 mins per day for 3 consecutive days. Each puff regime involved 1 (light exposure) or 4 puffs (moderate exposure) per minute with a 78 mL puff volume, 2.4 second puff duration and 3 hours between exposures.		mRNA expression for IL1β, IL6, TNF-α, IL4, IL5 and Ptgs2.<br>At 4 puffs/min exposure, Significant increase in neutrophils by mint flavoured JUUL compared to control, PG/VG and other flavoured JUUL, Significant increase in lung mRNA expression (TNF-α and IL13) by mango flavoured JUUL compared to control.<br><br>PG/VG group: Significant increase in lung mRNA expression (IL13) at 1 puff/min exposure compared to control.<br><br>Interpretation: Inhalation of flavoured JUUL e-cigarette, both with nicotine or without nicotine, results in lung inflammation			
Da Silva et al., 2022;[55] Brazil	Independent funding organization(s); none	Acute (5 days)	n=45 C57BL/6 mice; 11-12 weeks old, 100% male mice	CS (n=15): Exposed to 6 commercial full flavour filtered Virginia cigarettes.<br>EC (n=15): Exposed to Blu brand disposable EC with nicotine 24 mg per cartridge.<br>Control (n=15): Exposed to ambient air .<br><br>Exposure were given as 40 puffs/ per	Respiratory inflammation; Not measured	Lung function:<br>Respiratory rate was significantly higher than CS (p<0.002), NS difference in tidal volume, minute ventilation between EC and CS or control.<br><br>Inflammatory cells and markers in BALF:<br>Significant increase in leukocytes and macrophages in BALF compared to control (p<0.0001), significant	N/A	N/A	Moderate

				exposure, 120 puffs/day, for 5 consecutive days		increase in neutrophil count in BALF compared to control and CS (p=0.01), NS difference in lymphocyte count between groups. Significant increase in inflammatory markers- IL-17, CCL5, protein carbonyl compared to control ($p<0.05$).<br><br>Interpretation:<br>Acute exposure to EC promoted more acute inflammation compared to CS in male mice.			
Cahill et al., 2022;[46] US	Independent funding organization(s); none	Short to medium term (20 days)	BALB/c dams offspring mice,	Air: exposed air in utero + saline at 8-11 weeks.<br>JUUL+ Saline: Exposed to mint flavoured JUUL containing 5% nicotine, 55 ml puff volume, 0.15 mg/puff in utero + saline at 8-11 weeks.<br>Air+ HDM: Exposed to air in utero + HDM extract at 8-11 weeks.<br>JUUL + HDM: Exposed to mint flavoured JUUL containing 5% nicotine, 55 ml puff volume, 0.15 mg/puff in utero + HDM extract at 8-11 weeks.<br><br>JUUL composition: PG 100 0 Î¼g/puff, VG 300 0 Î¼g/puff, benzoic acid >40 0 Î¼g/puff.<br><br>Exposure was given as	Lung development in utero; Irreversible	At 11 weeks of age, JUUL +HDM exposed mice exhibited pulmonary inflammation when compared to their respective air +HDM controls. Additionally, the JUUL +HDM exposure dysregulated several genes associated with allergies and asthma.<br><br>Interpretation: In-utero JUUL exposure impairs lung development, and heightens the effects of allergic airway responses in adult offspring.	Sex: male and female offspring.	Female offspring exposed to JUUL + HDM:<br>Higher macrophage count in BALF compared to male offspring.<br>Significantly higher total lung inflammation score ($p<0.01$) compared to JUUL+ saline group, but not seen in male offspring	Moderate

				1 hr/day for 20 consecutive days.					
Su et al., 2023;[131] Taiwan	None; none	Acute (5 days)	8-12 weeks old male C57BL/6 mice	VG + PBS (phosphate buffered saline)<br>VG + LPS (lipopolysaccharide) (LPS added on day 5)<br>PBS + LPS (LPS added on day 5)<br>LPS only<br>VG + p38 inhibitor<br>LPS + p38 inhibitor<br>VG + LPS + p38 inhibitor<br>PBS only- control	Lung inflammation; Not measured	Lung histopathology:<br>VG significantly increased histopathological and fibrotic changes which indicates lung damage in both the VG group and VG+LPS group.<br><br>Inflammatory markers:<br>VG administration resulted in upregulation of neutrophil markers as well as upregulation of the expression of transforming growth factor-β (TGF-β), a central mediator of fibrogenesis, in the lungs of both VG and VG+LPS groups. VG enhanced the expression of adhesion molecules [very late antigen 4 (VLA-4) and vascular cell adhesion molecule 1 (VCAM-1)] and increased activation of p38 mitogen-activated protein kinase (p38 MAPK) to prompt neutrophil recruitment in the lungs of mice.<br><br>Intraperitoneal administration of a p38 inhibitor:<br>Attenuated histopathological changes significantly as well as VG-induced upregulation in expression of Ly6G, MPO, VLA-4, VCAM-1, TGF-β, and collagen-1 in mice with ALI.<br><br>Interpretation: VG enhanced the inflammatory response in mice lung.	N/A	N/A	Low
Silva-Ribeiro et al., 2023;[124]	Independent funding organization(s);	Acute (48 hrs)	Chick (Gallus gallus) embryonic lungs	Control medium.<br>EC exposed medium: 200 mg e-liquid (16	Lung development in utero; Irreversible	Morphological analysis of chick lung explants: EC aerosol impairs chick lung	N/A	N/A	Moderate

Portugal	none			mg/ml nicotine, <40% VG and <60% PG, menthol flavoured) delivered, on average 17-18 puffs.<br>Conventional cigarette exposed medium: 35mL puff at a time, allowed to contact medium for 1 min, repeated until cigarette (0.8 mg nicotine, 10 mg Tar; 10 mg carbon monoxide /cigarette) fully delivered (average 7-8 puffs)<br><br>Chick embryonic lungs were cultured in either of the 3 mediums for 48 hrs.		explant growth. A 10 % statistically significant decrease in the total area and perimeter was detected between EC aerosol and controls. Smoke-exposed explants showed a statistically significant decrease in all morphometric parameters compared to controls [15-30 %] and e-cig aerosol-exposed explants [12-34 %].<br><br>Inflammatory markers:<br>A statistically significant increase in TNF-α levels was detected in the EC aerosol and smoke-exposed explants compared to controls (9x and 7x times larger, respectively).<br><br>Overall: EC aerosol impairs lung growth and promotes lung inflammation. However, its impact on early lung growth seems less detrimental than conventional cigarette smoke.			
Husari et al., 2023;[75] Lebanon	Independent funding organization(s); none	Acute (7 days)	N=56; C57BL/6 mice	Control: Exposed to air,<br>CS: Exposed to 3R4F Cig smoke,<br>EC: Exposed to 18 mg/ml nicotine containing EC aerosol,<br>HTP: Exposed to IQOS,<br>EC + CS: exposed to both EC aerosol and Cig smoke,<br>HTP + CS: Exposed to both IQOS and Cig smoke,<br>HTP + EC: Exposed to both IQOS and EC	Respiratory inflammation; not measured	Lung wet to dry ratio (W/D): NS difference was seen between EC and control or EC+CS and control.<br><br>Albumin leak in BALF: Significantly increased in EC +CS exposure compared to control ($p<0.05$). NS effect was seen in EC group.<br><br>IL-1β, IL-6, and TNF-a gene expression in lung tissue: Significantly increased in EC +CS exposure compared to control ($p<0.05$, $p<0.001$, and $p<0.0001$ respectively). NS	N/A	N/A	Low

				aerosol.<br><br>EC aerosol exposure was given as 4s puff duration and the inter-puff interval was 14s generating a volume of 80 ml per puff.		effect was seen in EC group.<br><br>Histological examination of lung tissue:<br>Inflammatory cells infiltration, collagen deposits, ROS production and cell death significantly increased in EC+CS exposure. NS effect was seen in EC group.<br><br>Interpretation: Dual use of Cig and EC increased respiratory inflammation, while NS such effect was seen in only EC exposure.			
Bhat et al., 2023;[42] US	Independent Funding organization(s); some (no pro-tobacco association)	Short to medium (8 weeks and 12 weeks)	N=150; 8-weeks old C57BL/6NCr mice	EC (n=40): Exposed to nicotine containing EC aerosol,<br>HTP (n=40): Exposed to HTP aerosol,<br>Cig (n=40): Exposed to Cig smoke,<br>Control (n=30): Exposed to air.<br><br>Half of the mice were exposed for 8 weeks, while rest of them were vaccinated against a respiratory pathogen and exposed for 12 weeks followed by challenged with a bacterial pathogen.<br>EC aerosol exposure was given as 0.5 ml puff volume, 11 puffs per cluster, 20 min interpuff cluster interval and 13 puff clusters over 5h/day, 5 days a week.<br>EC, HTP and Cig had equal nicotine content.	Respiratory inflammation, respiratory infection; not measured	Immune cell infiltrates in the lungs:<br>Total cell infiltrates, B cells, T cells, and pro-inflammatory IL17A+ T cells significantly increased in EC exposure ($p<0.001$) compared to control, equivalent to that achieved following Cig exposure. The number of neutrophil in EC exposure was significantly lower compared to that of Cig exposure ($p<0.0001$).<br><br>Inflammatory markers in BALF:<br>Several cytokines and chemokines including IL-6, IL-2, VEGF, Eotaxin, MIP-2 significantly increased in EC exposure ($p<0.001$) compared to control, almost equivalent to that achieved following Cig exposure.<br><br>Lung damage:<br>Total BAL protein, BAL albumin level significantly	N/A	N/A	High

						increased in EC exposure ($p<0.0001$) compared to control, although the effect was lower than that of Cig exposure ($p<0.0001$).<br><br>Immunity following bacterial infection challenge:<br>Was significantly impaired in EC exposure ($p<0.05$) compared to control, but to a lesser extent than Cig exposure ($p<0.0001$).<br><br>Interpretation: EC exposure increased respiratory inflammation and susceptibility to bacterial infection.			
Ozekin et al., 2023;[104] US	Independent funding organization(s); none	Short to medium term (18.5 days)	Wildtype(C57BL/6) and Kcnj2 KO/+ mice; all offsprings were male mice	Control: Exposed to air;<br>EC: Pregnant mice were exposed to 2.4% freebase nicotine containing EC aerosol during gestation (total 96mg of nicotine over 4 hrs. each day)	Lung development in utero; irreversible	Fetal lung development:<br>EC exposure in wildtype mice had significantly reduced airspace area compared to controls ($p<0.001$).<br><br>Embryonic lung gene expression:<br>Embryonic lung gene expression changes mimicked transcriptional changes observed in adult mouse lungs exposed to Cig smoke. Significant reduction in genes that control ciliogenesis and ciliated cell formation in EC exposure in wildtype mice lungs were noted.<br><br>Interpretation: Intrauterine EC exposure disrupted fetal lung development and mucociliary clearance leading to susceptibility to respiratory infection.	N/A	N/A	Moderate

Maishan et al., 2023;[88] US	Independent funding organization(s); none	Short to medium (9 days)	8–12 weeks old C57BL/6 mice	Control: Exposed to air<br>PG/VG: Exposed to non-nicotine PG/VG 1:1 containing EC aerosol,<br>Nic-EC: Exposed to 36mg/mL nicotine and PG/VG 1:1 containing EC aerosol.<br><br>EC exposure was given as 80 ml puff volume, 10 puffs over 1 min followed by 110 puffs over 1 hr per day for 9 consecutive days. Some mice were challenged by influenza virus after 9 days exposure.	Pulmonary inflammation, respiratory infection; not measured	Inflammatory markers in BALF:<br>Significant increases in CXCL1,MCP-1, and IL-17A ($p<0.05$ for all) in the Nic-EC exposure group compared to PG/VG and control group.<br><br>Inflammatory markers in post-infection with influenza:<br>Both Nic-EC and PG/VG groups had significantly higher levels ($p<0.05$ for all) of IFN-g, TNF-α, IL-1b, IL-6, IL-17A, and MCP-1 compared to control group at 7 days post-infection.<br><br>Lung barrier interruption in post-infection with influenza:<br>Total protein and mucin concentration in BALF significantly increased ($p<0.05$ for all) in Nic-EC exposure compared to control and often PG/VG at 7 days post-infection.<br><br>Clearance of influenza virus:<br>Lung gene expression and number of virions in lung tissue in influenza-infected mice exposed to Nic-EC was significantly higher than PG/VG and control groups ($p<0.05$ for all) indicating impaired clearance of influenza virus.<br><br>Interpretation: Nicotine-containing EC exposure caused pulmonary inflammation, worsened viral infection and increased immune responses to influenza	N/A	N/A	High

						infection.			
Roxlau et al., 2023;[115] Germany	Independent funding organization(s); none	Short to medium term (8 months)	Wild-type C57BL/6 J mice	Control: Exposed to filtered air,<br>EC: exposed to nicotine free EC vapour prepared from 60%/30% PG/VG containing e-liquid.<br>EC+ Nic: exposed to EC vapour prepared from 60%/30% PG/VG and 18mg/mL nicotine containing e-liquid.<br><br>EC vapor was given as 6 hr/day, 5 days/week for 8 months.	Pulmonary inflammation and impaired lung function; not measured	Inflammatory cells in BALF: Neutrophil and lymphocytes counts significantly increased in EC + Nic (p<0.001) exposure compared to control. NS difference was seen between EC and control.<br><br>Inflammatory markers in BALF:<br>Cytokines (e.g., IL-2, IL-5, IL-16, IL-13, IL-33), chemokines, MMP-9, MMP-12, ExMAS, and rAMS significantly increased in EC + Nic (p<0.001) exposure compared to control. NS difference was seen between EC and control.<br><br>Lung function:<br>Airspace significantly increased in EC + Nic exposure compared to control (p<0.05). NS effect was seen on compliance, resistance, volume, density, and inspiratory capacity.<br><br>Interpretation: EC exposure with nicotine was significantly associated with pulmonary inflammation, NS effect on lung function was observed.	N/A	N/A	Low
Zhao et al., 2023;[150] China	Independent funding organization(s); none	Short to medium term (8 weeks)	N=60; Male C57BL/6 mice	Control: Exposed to air,<br>Cig: exposed to Marlboro Cigs (0.8 mg of nicotine),<br>Nic-EC: Exposed to 12 mg/mL nicotine and PG/VG 30:70 containing EC aerosol,<br>PG/VG: Exposed to	Impact on lung function, COPD; not measured	Lung function:<br>Peak inspiratory flow (PIF), peak expiratory flow (PEF), expiratory flow 50 (EF50), airway resistance, and inspiratory capacity were significantly decreased in both PG/VG and Nic-EC exposure groups (p < .05) compared to control. Static compliance also	N/A	N/A	Moderate

				PG/VG 30:70 containing EC aerosol.<br><br>Exposure was given for 0.34ml/EC exposure, 30 mins per sessions, 3 sessions/day for 8 weeks.		significantly increased in PG/VG and Nic-EC exposure ($p < .05$ or $p < .01$) compared to control.<br><br>Histological analysis - Structural changes:<br>Enlarged alveolar size with many ruptured alveoli and thickened bronchiolar wall accompanied by inflammatory cell infiltration was noted in both PG/VG and Nic-EC exposure.<br><br>Inflammatory markers gene expression in lung tissue:<br>IL-6 and TNF-α mRNA expression were upregulated in Nic-EC exposure compared to control, Cig and PG/VG exposure ($p<0.01$ for all).<br><br>Interpretation: EC exposure, both nicotine and non-nicotine, impacted lung function and caused significant morphological changes in lung, corresponding to COPD.			
Rasmussen et al., 2023;[112] US	Independent funding organizations; some (no pro-tobacco association)	Short to medium term (6 weeks)	Sprague Dawley rats: >8 weeks old, sex matched, with wild type CFTR expression<br><br>Ferrets: >22 weeks old, both male and female, with wild type CFTR expression	Control: Exposed to air<br>PG/VG: Exposed to EC aerosols from a 1:1 mixture of PG/VG,<br>Nic-EC: Exposed to 1.8% nicotine containing PG/VG EC aerosol.<br><br>Exposure was given as two 1 hr. sessions separated by 2-3 hrs. interval per day for 6 weeks.	Respiratory infection (cystic fibrosis), COPD; not measured	CFTR and epithelial sodium channel (ENaC)-mediated ion transport:<br>was significantly reduced in Nic-EC exposure compared to control ($p<0.05$).<br>NS difference was seen between PG/VG and control.<br><br>Mucociliary clearance and airway surface hydration:<br>was significantly reduced in Nic-EC exposure compared to control ($p<0.05$).<br>NS difference was seen between PG/VG and control.	N/A	N/A	Low

						Interpretation: Nicotine containing EC exposure increased risk of respiratory infections in cystic fibrosis and susceptibility to COPD.			
Muthumalage and Rahman, 2023;[97] US	Independent funding organization(s); none	Acute (3 days)	N=16-20; 8-10 week old male and female e C57BL/6J and BALB/cJ mice	Control: Exposed to air,<br>PG/VG: Exposed EC aerosol containing PG/VG at 1:1 ratio,<br>Menthol 0mg. Nic-EC: Exposed to 0mg/ml menthol flavored EC aerosol,<br>Menthol 6mg. Nic-EC: Exposed to 6mg/ml menthol flavored EC aerosol,<br>Tobacco 0mg. Nic-EC: Exposed to 0mg/ml tobacco flavored EC aerosol,<br>Tobacco 6mg. Nic-EC: Exposed to 6mgg/ml tobacco flavored EC aerosol.<br><br>EC exposure was given by 3rd generation ENDS, Brand A and B, device 2 puffs/min (70 ml puff volume,<br>2 s puff duration) for 2 hrs for 3 consecutive days.	Respiratory inflammation; not measured	Differential count in BALF: Both brand Menthol 0 mg Nic-EC and PG/VG exposure caused significantly (p<0.05 at least) increased neutrophils, macrophage and differential T-lymphocyte influx and induced significant immunosuppression (decreased CD8+ T cells) compared to control. Brand A Tobacco 0mg. Nic-EC and 6 mg. Nic-EC caused only significant immunosuppression compared to control.<br><br>Inflammatory markers in BALF:<br>Significantly increased Eotaxin, IL-6, and RANTES levels (p<0.05) were observed in Tobacco 6 mg. Nic-EC group, and sometimes in menthol 0mg. Nic-EC group compared to control- indicating allergic inflammatory response.<br><br>Interpretation: Acute exposure to flavored EC caused significant respiratory inflammation, immunosuppression, and allergic inflammatory response.	N/A	N/A	Low
Noël et al., 2023;[98] US	Independent funding organization(s);	Short-to-medium term (20 days)	N=28; Both male and female offspring mice	Control: Was exposed to filtered air in utero,	Lung development in utero; irreversible	Lung transcriptomic responses at birth:<br>Significantly regulated 88	Sex: male and female offspring mice	Same as study findings of entire	Low

	none			Nic-EC: exposed to vanilla flavored EC aerosols in utero containing 18 mg/ml nicotine, and 50/50 PG/VG.<br><br>Exposure was given throughout gestation. Some offsprings were sacrificed at birth. Some male mice were treated with wither house dust mites (HDM) or saline at 4 weeks of age and sacrificed at 7 weeks of age.		genes in males and 65 genes in females were found in Nic-EC offsprings. These genes were involved in CD28 signaling in T helper cells, regulation of immune responses, and oxidative stress responses.<br><br>HDM-induced asthma at 7 weeks:<br>Tidal volume significantly decreased (p<0.05) and perbronchial, alveoli, and perivascular inflammation significantly increased (p<0.05) in male mice in Nic-EC group exposed to HDM compared to control group exposed to HDM- indicating HDM induced asthma exacerbation.<br><br>Interpretation: In-utero exposure to nicotine containing EC might increase risk of asthma exacerbation in offsprings.		population.	
Song et al., 2023;[128] and Song et al; 2023a;[129] US	Independent funding organization(s); none	Short-to-medium term (2 weeks, 3 months)	8-10 week age C57/BL6J mice, both male and female	Asthmatic mice: Mice were sensitized and challenged with a DRA (dust mite).<br>Naive mice: Mice were not sensitized with DRA<br><br>Both groups of mice were exposed to following:<br>Control: some were exposed daily to filtered air for 2 weeks and some for 3months,<br>PG/VG: Exposed daily to EC aerosol	Asthma severity; reversible	mRNA expression in lung tissue:<br>At 2 weeks, mRNA associated with asthmatic lung inflammation significantly increased (p<0.05) in asthmatic Nic-EC group compared to naive Nic-EC group. NS difference was seen between naive Nic-EC and naive control groups.<br>At 3 months, Different gene expression patterns were profoundly found in both asthmatic PG/VG and asthmatic Nic-EC group, including genes involved in	Sex:<br>Male mice (n=10): control (n=3) + others (n=7)<br>Female mice (n=10): control (n=3) + others (n=7)	Mitochondrial DNA (mtDNA) content and mitochondrial transcription factor A (TFAM):<br>At 2 weeks, significant reduction (p<0.05) was seen in naive PG/VG+Nic group compared to naive control	High

				containing 30:70 PG/VG for 3 months, Nic-EC: exposed daily to EC aerosol containing 2% (20 mg/ml) nicotine and 30:70 PG/VG, some for 2 weeks and some for 3 months.<br><br>EC puff duration was 3 s, puff interval was 1 min, and the puff volume was 70 mL, exposure was given for 4 hrs/day.		mitochondrial dysfunction, oxidative phosphorylation, and p21-activated kinase (PAK) signalling.<br><br>Inflammatory markers in BALF:<br>At 2 weeks, IL-12, MPO, TARC/CCL17, MDC/CCL22 significantly increased ($p<0.05$) in asthmatic Nic-EC group compared to naive Nic-EC group. NS difference was seen between naive Nic-EC and naive control groups.<br><br>mtDNA content and mitochondrial transcription factor A (TFAM):<br>At 2 weeks, significant reduction ($p<0.05$) was seen in asthmatic Nic-EC group compared to naive Nic-EC group in both male and female mice.<br>At 3 months, significant increase ($p<0.05$) in mtDNA copy numbers in both asthmatic PG/VG and asthmatic Nic-EC group was seen compared to asthmatic control group, significant decrease ($p<0.05$) was seen in naive PG/VG and naive Nic-EC group compared to naive control group.<br><br>Periodic acid-schiff (PAS) positive cells in lung tissue:<br>At 3 months, significant increase ($p<0.05$) was seen in asthmatic Nic-EC group compared to asthmatic control and asthmatic PG/VG group.		group in male mice, but not in female mice<br><br>Interpretation: EC exposure caused higher asthma severity in male mice than female mice.	

						Interpretation: Short-to-medium term exposure to nicotine-containing EC could increase asthma severity, more profoundly in asthmatic mice compared to non-asthmatic mice.			
Scieszka et al., 2023;[119] US	Independent funding organization(s); none	Short-to-medium term (8 weeks)	N=48; 8-20 week old female C57/BL6J mice	Control (n=16): Exposed to air,<br>PG/VG (n=16): Exposed to PG/VG 50%/50% containing EC aerosol,<br>PG/VG + phytol (n=16): Exposed to PG/VG 50%/50% with 1% Phytol containing EC aerosol (Phytol is a diterpene alcohol that has been found in commercial ENDS products)<br><br>Exposure was given as 2 h/day, 5 days/week for 8 weeks	Impact on lung function and Respiratory Inflammation; not measured	Lung function:<br>NS changes after PG/VG exposure alone but PG/VG + phytol exposure significantly increased quasistatic compliance ($p < 0.01$) compared to control.<br>NS difference in airways resistance or dynamic compliance found between groups.<br><br>Inflammatory markers and cells in lung:<br>PG/VG + phytol exposure was associated with upregulation of multiple genes, including genes that regulate lung function, and several inflammatory genes including IL-1b. NS changes in t-lymphocytes was found between groups..<br><br>Interpretation: Phytol containing EC exposure impacted lung function and was associated with pulmonary inflammation. However, only PG/VG containing EC exposure did not have the same effect.	N/A	N/A	High
Rodriguez-Herrera et al., 2023;[113] Brazil	Independent funding organizations; None	Short to medium (60 days)	N=48; C57BL mice; 50% male and 50% female; Aged between 8-10 weeks	Control (n=16): Exposed to air,<br><br>Cig (n=16): Exposed to 12 full flavor filtered Virginia Marlboro	Impact on lung function; respiratory inflammation, COPD; not measured	Lung function:<br>Respiratory rate, and minute ventilation significantly higher ($p<0.05$) following EC exposure compared to control in both male and female and	Sex: Male (n=24), Female (n=24)	Same as study findings of entire population.	Low

				commercial cigarettes (0.9 mg of nicotine per cigarette).<br><br>EC (n=16): Exposed to Vype eTank Pro 2 EC aerosol containing 35/65 PG/VG and 6 mg/ml nicotine.<br><br>Exposure was given as 240 puffs per day for 60 days.		compared in to Cig exposure in female. In male mice, significantly higher tidal volume in EC exposure was also noted compared to control ($p<0.01$).<br><br>Inflammatory cells in BALF: Significant increase ($p<0.01$) in total cell count and differential counts were observed in EC exposure compared to control, in both male and female mice. The effect was similar or often higher than Cig exposure ($p<0.01$).<br><br>Inflammatory markers of BALF:<br>Significant increase ($p=0.01$) in IL-6 in both sex, IL-13 in female mice, and IL-15 in male mice was noted in EC exposure compared to control. NS difference was seen between EC and Cig exposure.<br><br>Lung Parenchyma:<br>Increased alveolar air space and septal destruction was significantly higher ($p<0.01$) in EC exposure compared to control in both sex and compared to also Cig exposure in female mice.<br><br>Interpretation: EC exposure impaired lung function compared to control. EC exposure also caused emphysematous changes in lung in both male and female mice. The effect was often similar or higher than smoking			

						cigarettes and female mice seemed more susceptible than male mice.			
Snoderly et al., 2023;[127] US	Independent funding organizations; None	Acute (3 days)	N=10; Aged 12-16 weeks old female BALB/cJ mice	Control (n=5): exposed to air<br><br>PG/VG (n=5): Exposed to nicotine free PG/VG 1.1 EC aerosol.<br><br>Exposure was given as 1L/min flow rate for 5s once every minute for 2.5 hr daily for 3 days.	Respiratory inflammation; Reversible	Inflammatory cells in pulmonary microvasculature: Significant increase ($p<0.05$) in neutrophil count was observed up to 48 hrs. of PG/VG exposure.<br><br>Inflammatory cells in BALF: NS changes was observed up to 48 hrs. of PG/VG exposure.<br><br>MPO concentration in BALF: NS changes was observed up to 48 hrs. of PG/VG exposure.<br><br>Interpretation: Non-nicotine EC exposure was associated with minimal inflammatory changes in lung parenchyma.	N/A	N/A	Low
Yanina et al., 2023;[145] Russia	Independent funding organizations; None	Acute (single exposure)	N=12; male Wistar Rats	Control (n=6): exposed to air<br><br>PG/VG (n=6): Exposed to nicotine free 40%/60% PG/VG EC aerosol.<br><br>Exposure was given at a rate of 0.15L/min for 10 mins.	COPD; not measured	Histological examination of lung: Areas of emphysematous expansion in the alveoli, thickening of the alveolar septa, and the phenomenon of immersion liquid permeation was observed in PG/VG exposure.<br><br>3D optical coherence tomography images: A decrease in the volume of air in the lung tissue, optical clearing and the expansion of the alveoli were observed in PG/VG exposure compared to control.<br><br>Interpretation: Non-nicotine EC exposure was associated with emphysematous changes in lung.	N/A	N/A	Low

Abbreviations: AA, African American; ACh, Acetylcholine; ACOS, asthma-COPD overlap syndrome; BALF, Bronchoalveolar lavage fluid; CI, confidence interval ; COPD, chronic obstructive pulmonary disease; CT, computed tomography; CXR, chest X-ray; EC, e-cigarette; ENDS, electronic nicotine delivery system; EHR, electronic health record; EVALI, e-cigarette or vaping associated lung injury; FEV1, Forced expiratory volume in 1 sec; FeNO, Fractional exhaled nitric oxide; fMRI, functional magnetic resonance imaging; FVC, Forced vital capacity; Gly, glycerine; HTP, heated tobacco products; iNOS, inducible nitric oxide synthase; LCI, lung clearance index; LFT, lung function test; mtCN, Mitochondrial DNA copy numbers; N/A, not applicable; NH, Non-Hispanic; NOS, nitric oxide synthase; NRT, nicotine replacement therapy; NS, not significant; OR, odds ratio; PFT, pulmonary function test; PG, propylene glycol; ROS, reactive oxygen species; RR, respiratory rate; SpO2, oxygen saturation; US, The United States; UK, The United Kingdom; VG, vegetable glycerin.

Supplementary Material 3. Summary of subgroup findings from the 2022 McNeill et al. review assessing risk of respiratory health outcomes (n=5).

Author(s), Year; Country	Sub-group category	Exposure	Sub-group description	Number of participants in sub-groups	Intervention/ exposure	Health Outcome (reversibility)	Study Findings	Risk of Bias
**Human: sex-based**								
Ghosh et. al, 2019;[151] US	Sex	N/A	Male and Female	N= 42	Non-smokers: no use in 6 months; male (n=14), female (n=10).<br><br>Smokers: only cigarette use in 6 months; male (n=8), female (n=6).<br><br>Vapers: former or never smokers; male (n=10), female (n=4).	Respiratory Inflammation (not measured)	Inflammatory markers in BALF:<br>NS difference in increase in NE (neutrophil elastase), MMP-2, and MMP-9 proteases was seen between males and females.<br><br>Interpretation: No sex-based differences in respiratory inflammation was seen among EC users.	Low (14/20)
**Animal: sex-based**								
Naidu et al., 2021;[153] US	Sex	Short-to-medium term; 21 days	Male and Female (BALB/c mice)	5 male, 5 female	Control: air<br>PG/VG<br>Nic-EC (18mg/mL nicotine)<br><br>Mice were exposed for a total of 30 minutes, 2x a day for 21 days.<br>.	COVID-19 infection (not measured)	ACE2 expression in Lungs:<br>Exposure to e-cigarette significantly increased lung ACE-2 protein expression in a nicotine-dependent manner in male but not in female mice and significant increase in ACE-2 mRNA in a nicotine-dependent manner in male but not in female mice.<br><br>Interpretation: Nicotine EC exposure was associated with increased susceptibility to COVID-19 infection in male mice, but not in female mice.	Not specified
Wang et. al., 2020;[155] US	Sex	Short-to-medium term; 30 days	Male and Female Mice<br><br>wild-type (WT), nAChRŒ±7 knockout (KO), and lung epithelial cell-specific KO (nAChRŒ±7 CreCC10) mice	6-10 Males<br>6-10 Females	Control: air<br>PG/VG<br>Nic-EC (25mg/mL)<br><br>Whole body exposure for 2 h/day, 5 days/week, for 30 days.	Respiratory inflammation; COVID-19 infection (not measured)	p50 and p105 (immune-related protein gene) expression:<br>-Nic-EC WT female showed upregulation of both genes compared to control and PG/VG (P< 0.05). NS differences in male mice groups<br><br>ACE2 protein expression:<br>Nic-EC WT female showed upregulation of ACE2 (P< 0.01 at least) compared to control and PG/VG. In males, the PG/VG exposure significantly decreased the ACE2 protein level in WT compared to the control (P<0.05)	Not specified

							Interpretation: Nicotine EC exposure was associated with increased susceptibility to respiratory inflammation and COVID-19 infection in female mice, but not in male mice.	
Wang et. al., 2019;[154] US	Sex	Acute; 3 days	Male and Female mice	3 Male 3 Female	Control: air<br>PG/VG<br>Nic-EC (25mg/mL)<br><br>Whole body exposure for 2 hours/day for 3 consecutive days	Respiratory inflammation; not measured	Inflammatory cell counts:<br>Significant increases in T-lymphocytes and increased neutrophil counts in PG/VG group in females compared to control ($P < 0.01, P < 0.05$), whereas NS effect was seen in males. Significantly increased MPO activity in the males and females in PG/VG alone exposed group compared to control ($P < 0.01$). MPO activity was significantly lower in the Nic-EC exposed group compared to PG/VG in both males and females ($P < 0.01$).<br><br>Gene expression:<br>Females showed significant ADRP and PPARγ upregulation in both PG/VG and Nic-EC groups and CTNN1B upregulation in Nic-EC group compared to the control ($P<0.05$).<br>Males showed only significant ADRP upregulation in PG/VG group, and CTNN1B upregulation in both PG/VG and Nic-EC groups compared to control ($P<0.05$).<br><br>Interpretation: Both male and female mice showed significant respiratory inflammation following both nicotine and non-nicotine EC exposure, higher effects was seen following nicotine EC exposure and slightly higher effects in females.	Not specified
Lallai, Manca, & Fowler, 2021;[152] US	Sex	Acute; 5 days	Male and Female (mice)	N=	Control: air<br>PG/VG<br>Nic-EC (7.5mg/mL)<br><br><br>1hr/day for 5 days. One puff every five min for a total of 12 puffs per hr session; each puff	COVID-19 infection (not measured)	ACE2 mRNA and protein expression:<br>A significant increase in ACE2 mRNA was found in male mice (, p =0.0005, Control vs. Nicotine p =0.0012, Vehicle vs. Nicotine p =0.0032), NS change in females compared to control.<br><br>ACE2 positive cells (density):	N/A

					administration allowed for ~40 s of vapor exposure		Nicotine vapor inhalation in males induced a statistically significant increase in the density of ACE2 positive cells (p <0.0001, Control vs. Nicotine p =0.0007, Vehicle vs. Nicotine p =0.0001), NS change in female mice.<br><br>a7 subunit (involved in immune and inflammatory responses) expression in lungs: NS differences were found between males and females.<br><br>Interpretation: Nicotine EC exposure was associated with increased susceptibility to COVID-19 infection compared to control in male mice, but not in female mice.	

Abbreviations: COVID-19, Coronavirus Disease 2019; EC, e-cigarette; OR, odds ratio; CI, confidence interval; NS, not significant; N/A, not applicable; PG, propylene glycol; VG, vegetable glycerin; US, the United States; UK, the United Kingdom.

Supplementary Material 4. Quality assessment findings

Table 1. Risk of bias assessment of non-randomized experimental studies with the Risk of Bias in Non-randomized Studies of Interventions tool (ROBINS-I) and longitudinal observational studies with the Risk of Bias in Non-randomized Studies- of Exposure (ROBINS-E) tool.

Author and year	Overall based on B2/B3/B4	Domain 1	Domain 2	Domain 3	Domain 4	Domain 5	Domain 6	Domain 7	Overall
**Non-randomized experimental studies**									
Nyilas et al., 2022;[100] Switzerland	Not applicable	Serious	Low	Low	Low	Low	Moderate	Low	**Serious**
Sayed et al., 2021;[117] US	Not applicable	Moderate	Low	Low	Moderate	Low	Moderate	Low	**Moderate**
Majek et al., 2023;[89] UK	Not applicable	Moderate	Low	Low	Low	Low	Moderate	Low	**Moderate**
**Longitudinal observational studies**									
Gao et al., 2022;[62] UK	Needs further assessment	Low	Low	Low	Low	Some concerns	Low	Low	**Some concerns**
Young-Wolff et al., 2022;[147] US	Needs further assessment	Low	Low	Low	Low	Some concerns	Low	Low	**Some concerns**
Xie et al., 2022;[143] US	Needs further assessment	Low	Low	Low	Low	Some concerns	Low	Low	**Some concerns**
Paulin et al., 2022;[106] US	Needs further assessment	Low	Low	Low	Low	Low	Low	Low	**Low**
Sargent et al., 2022;[116] US	Needs further assessment	Low	Low	Low	Low	Low	Low	Low	**Low**
Goldberg Scott et al., 2023;[65] US	Needs further assessment	Low	Low	Low	Low	Low	Low	Low	**Low**
Kang et al., 2021;[77] South Korea	Needs further assessment	Some concerns	Low	Low	Low	Low	Low	Low	**Some concerns**
Brunette et al., 2023;[44] US	Needs further assessment	High	Low	Low	Low	Some concerns	Low	Low	**High**
Cook et al., 2023;[54] US	Needs further assessment	Low	Low	Low	Low	Low	Low	Low	**Low**
Mukerjee et al., 2023;[96] US	Needs further assessment	Low	Low	Low	Low	Low	Low	Low	**Low**
Patel et al., 2023;[25] US	Needs further assessment	Low	Low	Low	Low	Some concerns	Low	Low	**Some concerns**
To et al., 2023;[135] Canada	Needs further assessment	Low	Low	Low	Low	Low	Low	Low	**Low**

Table 2. Risk of bias assessment of biomarker-based cross-sectional studies with the BIOCROSS tool.

Author and year	Item 1	Item 2	Item 3	Item 4	Item 5	Item 6	Item 7	Item 8	Item 9	Item 10	Overall
Ying et al., 2022;[146] US	2	1	1	1	1	2	2	2	1	1	**Low (14/20)**
Kim and Kang, 2021;[81] South Korea	2	2	1	1	2	2	2	1	1	1	**Low (15/20)**
Mori et al., 2022;[92] US	2	2	1	1	2	2	2	1	1	1	**Low (15/20)**
Kelesidis et al., 2022;[79] US	2	0	0	0	1	2	1	1	0	0	**Moderate (7/20)**
Davis et al., 2022;[57] US	2	1	0	1	1	2	1	2	1	2	**Low (13/20)**
Wetherill et al., 2023;[139] US	2	1	1	1	1	2	0	1	1	1	**Moderate (11/20)**
Joshi et al., 2021;[76] Canada	2	2	1	1	1	2	1	1	1	1	**Low (13/20)**
Hickman et al., 2022;[72] US	2	2	1	1	2	2	2	1	1	1	**Low (15/20)**
Polosa et al., 2021;[109] Italy	2	2	1	1	1	2	1	1	1	1	**Low (13/20)**
Payton et al., 2022;[107] US	2	1	1	1	1	2	2	1	1	1	**Low (13/20)**
Chandra et al., 2023;[52] US	2	2	2	1	2	2	1	1	0	1	**Low (14/20)**
Kelesidis et al., 2023;[78] US	2	1	1	1	1	2	0	1	1	1	**Moderate (11/20)**
Shields et al., 2023;[122] US	2	1	1	1	2	2	2	1	1	1	**Low (14/20)**
Shiffman et al., 2023;[123] UK	2	2	1	1	2	2	2	1	1	1	**Low (15/20)**
Pozuelos et al., 2022;[110] US	2	2	1	1	2	2	2	1	1	1	**Low (15/20)**

Abbreviation: NA= not applicable.

Table 3. Risk of bias assessment of non-biomarker based cross-sectional studies, case reports, and case series with the Joanna Briggs Institute (JBI) critical appraisal tools.

Author and year	Item 1	Item 2	Item 3	Item 4	Item 5	Item 6	Item 7	Item 8	Item 9	Item 10	Overall
**Cross-sectional studies**											
Varella et al., 2022;[137] US	Y	Y	N	Y	Y	Y	Y	Y	NA	NA	**Low (7/8)**
Merianos et al., 2022;[91] US	Y	Y	N	Y	Y	Y	N	Y	NA	NA	**Low (6/8)**
Wills et al., 2022;[142] US	Y	Y	N	Y	Y	Y	N	Y	NA	NA	**Low (6/8)**
Cordova et al., 2022;[24] US	Y	Y	N	Y	Y	Y	N	Y	NA	NA	**Low (6/8)**
Chaffee et al., 2021;[49] US	Y	Y	N	Y	Y	Y	N	Y	NA	NA	**Low (6/8)**
Chaiton et al., 2023;[50] Canada	Y	Y	N	Y	Y	Y	N	Y	NA	NA	**Low (6/8)**
Lee et al., 2021;[86] Australia	Y	Y	N	Y	Y	Y	Y	Y	NA	NA	**Low (7/8)**
Bircan et al., 2021;[43] US	Y	Y	N	Y	Y	Y	N	Y	NA	NA	**Low (6/8)**
Lee and Shin, 2023;[85] US	Y	Y	N	Y	Y	Y	N	Y	NA	NA	**Low (6/8)**
Austin-Datta et al., 2023;[37] US	Y	Y	N	N	Y	Y	N	Y	NA	NA	**Moderate (5/8)**
Dirisanala et al., 2023;[58] US	Y	Y	Y	Y	Y	Y	Y	Y	NA	NA	**Low (8/8)**
Moyers et al., 2023;[95] US	Y	N	Y	Y	Y	Y	Y	Y	NA	NA	**Low (7/8)**
Williams et al., 2023;[141] US	Y	Y	N	Y	Y	Y	Y	Y	NA	NA	**Low (7/8)**
Buu et al., 2023;[45] UK	Y	Y	N	Y	Y	Y	N	Y	NA	NA	**Low (6/8)**
**Case reports**											
Helfgott et al., 2022;[71] US	N	Y	Y	Y	Y	Y	N	Y	NA	NA	**Low (6/8)**
Guarino et al., 2022;[67] Italy	N	N	Y	Y	Y	Y	N	Y	NA	NA	**Moderate (5/8)**
Pan et al., 2022;[105] Taiwan	N	N	Y	Y	Y	N	N	Y	NA	NA	**Moderate (4/8)**
Hoekstra et al., 2021;[74] US	N	N	Y	Y	Y	Y	Y	Y	NA	NA	**Low (6/8)**
Chan et al., 2021;[51] Australia	N	Y	Y	Y	Y	N	N	Y	NA	NA	**Moderate (5/8)**

McCormick et al., 2022;[90] US	N	N	Y	Y	Y	Y	Y	N	NA	NA	**Moderate (5/8)**
Casamento Tumeo et al., 2022;[48] Italy	Y	N	Y	Y	Y	Y	Y	Y	NA	NA	**Low (7/8)**
Smith et al., 2022;[126] UK	N	Y	Y	Y	Y	Y	N	Y	NA	NA	**Low (6/8)**
Collins et al., 2022;[53] UK	N	N	Y	Y	Y	N	Y	Y	NA	NA	**Moderate (5/8)**
Khan et al 2022;[80] UK	N	N	Y	Y	Y	Y	N	Y	NA	NA	**Moderate (5/8)**
Roman et al., 2021;[114] US	N	Y	Y	Y	Y	Y	N	Y	NA	NA	**Low (6/8)**
Takigawa et al., 2022;[133] Japan	N	Y	Y	Y	Y	Y	N	Y	NA	NA	**Low (6/8)**
Soybel et al., 2022;[130] US	Y	Y	Y	Y	Y	Y	Y	Y	NA	NA	**Low (8/8)**
Kubbara et al., 2021;[84] US	N	N	Y	Y	Y	Y	N	Y	NA	NA	**Moderate (5/8)**
Norman et al., 2023;[99] Spain	Y	Y	Y	Y	Y	Y	N	Y	NA	NA	**Low (7/8)**
Sezgin et al., 2023;[120] Turkey	Y	N	Y	Y	Y	Y	Y	Y	NA	NA	**Low (7/8)**
Gaba et al., 2023;[61] India	Y	Y	Y	Y	Y	Y	N	Y	NA	NA	**Low (7/8)**
Lucas et al., 2023;[87] Brazil	Y	N	Y	Y	Y	Y	Y	Y	NA	NA	**Low (7/8)**
Alsaid et al., 2023;[34] Saudi Arabia	N	Y	Y	Y	Y	Y	N	Y	NA	NA	**Low (6/8)**
**Case series**											
Kligerman et al., 2021;[82] US	Y	Y	Y	Y	N	N	N	Y	Y	Y	**Low (7/10)**
Tanz et al., 2021;[134] US	Y	Y	Y	Y	N	Y	Y	N	N	Y	**Low (7/10)**
Gupta et al., 2022;[68] US	Y	Y	Y	N	N	N	N	Y	N	Y	**Moderate (5/10)**
Triantafyllou et al., 2021;[136] US	Y	Y	Y	Y	N	N	Y	Y	Y	Y	**Low (8/10)**
Harry-Hernandez et al., 2023;[69] US	Y	Y	Y	Y	N	Y	N	Y	Y	Y	**Low (8/10)**

Abbreviation: Y= yes, N=No, UC= unclear, NA= not applicable.

Table 4. Risk of bias assessment of animal studies with the Office of Health Assessment and Translation (OHAT) tool.

Author and year	Item 1	Item 2	Item 3	Item 4	Item 5	Item 6	Item 7	Item 8	Item 9	Overall
Wick et al., 2022;[126] US	++	-	++	-	-	++	--	++	++	**Moderate**
Caruso et al., 2021;[33] Italy	--	--	++	--	-	++	--	++	++	**High**
Komura et al., 2022;[69] Japan	--	--	++	--	-	++	--	++	++	**High**
Schaunaman et al., 2022;[104] US	--	--	++	--	-	++	--	++	++	**High**
Wang et al., 2021;[124] China	--	--	++	--	-	++	--	++	++	**High**
Baldovinos et al., 2022;[24] US	--	--	++	--	-	++	--	++	++	**High**
Sinha et al., 2022;[111] US	--	--	++	--	-	++	--	++	++	**High**
Morris et al., 2021;[79] US	++	++	++	++	-	++	++	++	++	**Low**
Ghosh et al., 2023;[50] US	--	--	++	--	-	++	--	++	++	**High**
Raduka et al., 2023;[97] US	--	--	++	--	-	++	--	++	++	**High**
Begum et al., 2023;[27] US	--	--	++	--	-	++	--	++	++	**High**
Phandthong et al., 2023;[94] US	--	--	++	--	-	++	--	++	++	**High**
Rasmussen et al., 2023;[98] US	-	++	++	++	-	++	++	++	++	**Low**
Effah et al., 2023;[45] UK	--	--	++	--	-	++	--	++	++	**High**

Note: ++ is definitely low, + is probably low, - is probably high, -- is definitely high.

Table 5. Risk of bias assessment of animal studies with the Systematic Review Center for Laboratory animal Experimentation (SYRCLE) tool.

Author and year	Item 1	Item 2	Item 3	Item 4	Item 5	Item 6	Item 7	Item 8	Item 9	Item 10	Overall
Zhang et al., 2023;[148] China	No	Yes	No	No	No	No	No	Yes	Yes	Yes	**High**
Goto et al., 2022;[66] US	Yes	Yes	No	Yes	No	Unclear	No	Yes	Yes	Yes	**Moderate**
Zhang et al., 2021;[149] US	No	No	No	No	No	No	No	Yes	Yes	Yes	**High**
Daou et al., 2021;[56] Lebanon	No	Yes	Unclear	No	No	No	No	Yes	Yes	Yes	**High**
Esquer et al., 2022;[60] US	No	Yes	No	No	No	No	No	Yes	Yes	Yes	**High**
Yang et al., 2022;[144] China	Yes	Yes	Unclear	Yes	Yes	Unclear	Yes	Yes	Yes	Yes	**Low**
Onyenwoke et al., 2022;[101] US	No	No	No	No	No	No	Yes	Yes	Yes	Yes	**High**
Getiye et al., 2022;[63] US	Yes	Yes	Unclear	Yes	Unclear	Yes	Yes	Yes	Yes	Yes	**Low**
Moshensky et al., 2022;[94] US	Yes	Yes	Unclear	Yes	Yes	Yes	Yes	Yes	Yes	Yes	**Low**
Shi et al., 2022a;[121] US	Yes	Unclear	Unclear	Yes	No	No	No	Yes	Yes	Yes	**Moderate**
Been et al., 2023;[39] Canada	Yes	Unclear	Yes	Yes	No	No	No	Yes	Yes	Yes	**Moderate**
Orzabal et al., 2021;[102] US	Yes	Yes	Unclear	Yes	No	No	No	Yes	Yes	Yes	**Moderate**
Alzoubi et al., 2022;[35] Jordan	Yes	Yes	Unclear	Yes	No	No	No	Yes	Yes	Yes	**Moderate**
Orzabal et al., 2022;[103] US	Yes	Yes	Unclear	Yes	Yes	Yes	Yes	Yes	Yes	Yes	**Low**
Aslaner et al., 2022;[36] US	No	Unclear	No	No	No	No	No	Yes	Yes	Yes	**High**
Hassan and El-Wafaey, 2022;[70] Egypt	Yes	Yes	Unclear	Yes	Yes	Yes	Yes	Yes	Yes	Yes	**Low**
Hinds et al., 2022;[73] US	No	Yes	No	No	No	No	No	Yes	Yes	Yes	**High**
Suryadinata et al., 2021;[132] Indonesia	No	Unclear	No	No	No	No	No	Yes	Yes	Yes	**High**
Been et al., 2022;[40] Canada	Yes	Unclear	Unclear	Yes	No	No	No	Yes	Yes	Yes	**Moderate**

Da Silva et al., 2022;[55] Brazil	Yes	No	Unclear	Yes	No	No	No	Yes	Yes	Yes	**Moderate**
Cahill et al., 2022;[46] US	Unclear	Unclear	Yes	Unclear	Yes	Unclear	Yes	Yes	Yes	Yes	**Moderate**
Su et al., 2023;[131] Taiwan	Unclear	Yes	Yes	Unclear	Yes	Yes	Yes	Yes	Yes	Yes	**Low**
Silva-Ribeiro et al., 2023;[124] Portugal	Yes	Yes	Unclear	Yes	No	No	No	Yes	Yes	Yes	**Moderate**
Husari et al., 2023;[75] Lebanon	Unclear	Yes	Yes	Unclear	Yes	Unclear	Yes	Yes	Yes	Yes	**Low**
Bhat et al., 2023;[42] US	No	Unclear	No	No	No	No	No	Yes	Yes	Yes	**High**
Ozekin et al., 2023;[104] US	No	Unclear	No	No	Yes	No	Yes	Yes	Yes	Yes	**Moderate**
Maishan et al., 2023;[88] US	No	Unclear	No	No	No	No	No	Yes	Yes	Yes	**High**
Roxlau et al., 2023;[115] Germany	Yes	Yes	Unclear	Yes	No	Yes	No	Yes	Yes	Yes	**Low**
Zhao et al., 2023;[150] China	Yes	Yes	Unclear	Yes	No	No	No	Yes	Yes	Yes	**Moderate**
Rasmussen et al., 2023;[112] US	Yes	Yes	Unclear	Yes	Yes	Unclear	Yes	Yes	Yes	Yes	**Low**
Muthumalage and Rahman, 2023;[97] US	Unclear	Yes	Yes	Unclear	Yes	Unclear	Yes	Yes	Yes	Yes	**Low**
Noël et al., 2023;[98] US	Unclear	Yes	Yes	Unclear	Yes	Unclear	Yes	Yes	Yes	Yes	**Low**
Song et al., 2023;[128] US	No	No	No	No	No	No	No	Yes	Yes	Yes	**High**
Song et al; 2023a;[129] US	No	Yes	No	No	No	No	No	Yes	Yes	Yes	**High**
Scieszka et al., 2023;[119] US	No	Yes	No	No	No	No	No	Yes	Yes	Yes	**High**
Rodriguez-Herrera et al., 2023;[113] Brazil	Yes	Yes	Unclear	Yes	Yes	Yes	Yes	Yes	Yes	Yes	**Low**
Snoderly et al., 2023;[127] US	Yes	Yes	Unclear	Yes	No	Yes	No	Yes	Yes	Yes	**Low**
Yanina et al., 2023;[145] Russia	Unclear	Yes	Yes	Unclear	Yes	Unclear	Yes	Yes	Yes	Yes	**Low**

Supplementary Material 5. Publication bias assessment of studies included in the meta-analyses.

Fig 1. Doi plots for studies examining incidence of respiratory symptoms among non-smoker current vapers vs never users and non-vaper current smokers (upper row) and among dual users vs never users, non-smoker current vapers, and non-vaper current smokers (middle and bottom rows).

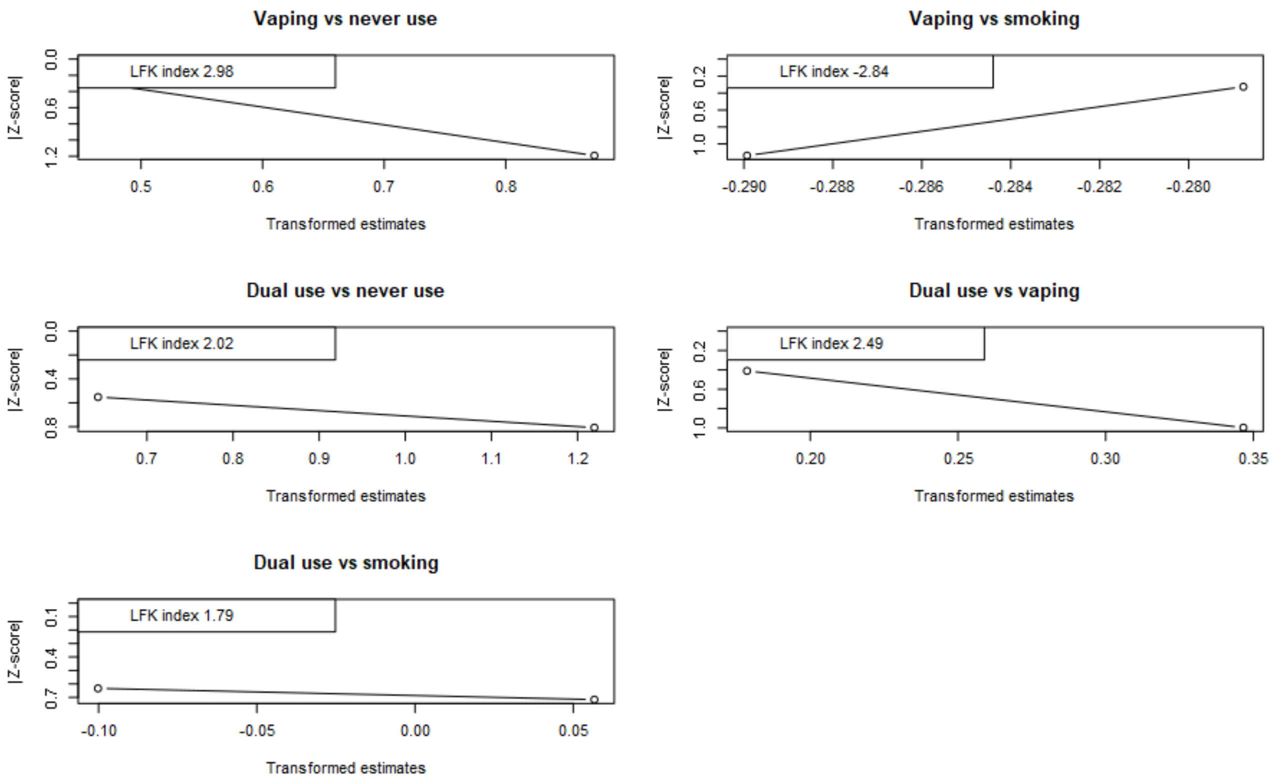

Fig 2. Doi plots for studies examining prevalence of COPD among non-smoker current vapers vs never users (Upper left) and dual users vs never users (upper right) or non-vaper current smokers (bottom left).

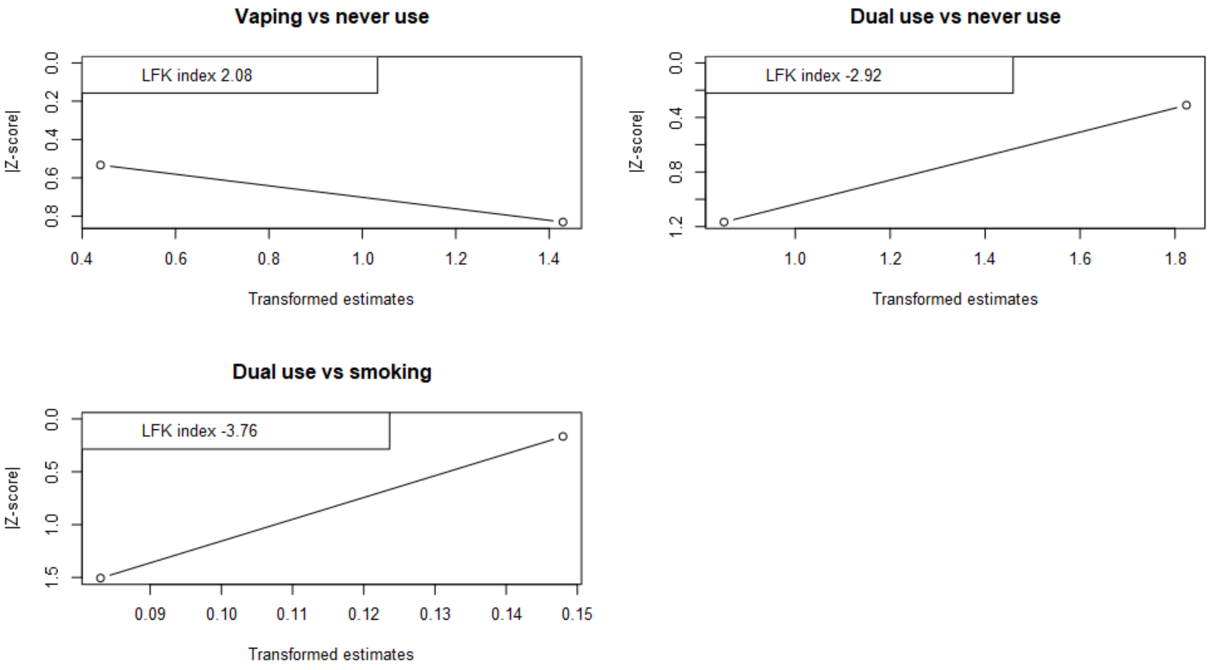

Supplementary Material 6. Harvest plots showing distribution of studies assessing sex-based differences in risk of asthma and COPD, impact on lung function, lung inflammation and damage, COVID-19, and lung development in utero among females compared to males (individual bar in the plot represents a single study) (n=15).

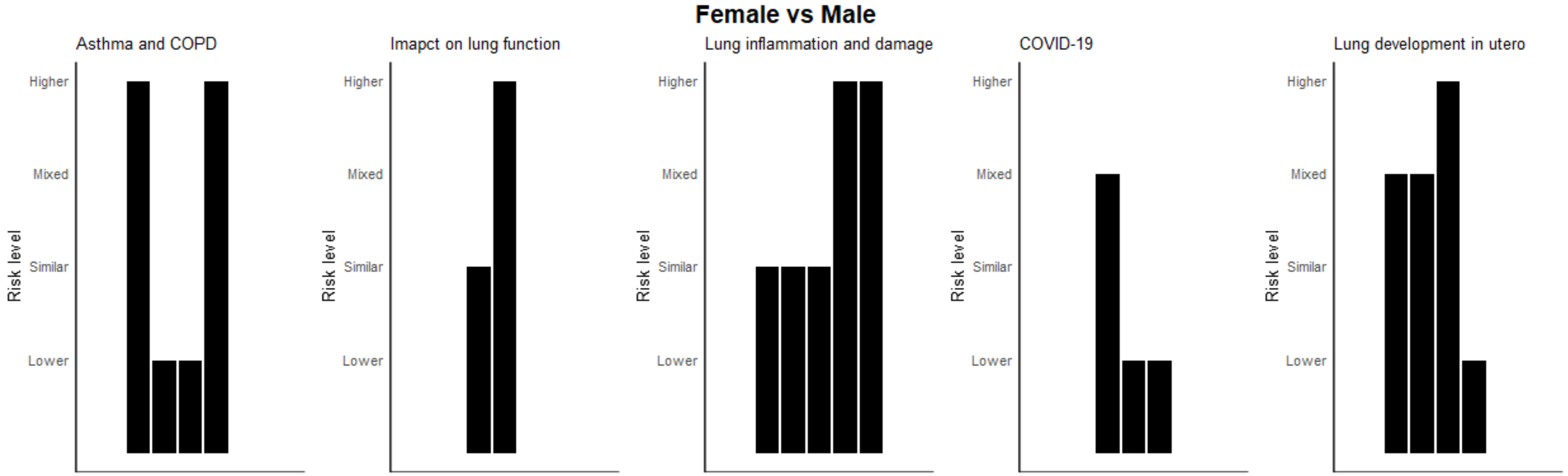

Supplementary Material 7. GRADE and GRADE-CERQual evidence profile to assess certainty or confidence in the body of evidence.

**Meta-analysis findings**	**GRADE Certainty assessment**								**Certainty**
**Summary**	**No. of studies**	**Studies**	**Study design**	**Risk of bias**	**Inconsistency**	**Indirectness**	**Imprecision**	**Other considerations**	
Incident risk of respiratory symptoms: Non-smoker current vapers had higher risk compared to never users	2	[116,143]	Observational	Serious	Serious	Not serious	Not serious	Major risk of publication bias; small number of studies	⊕◯◯◯ Very low
Incident risk of respiratory symptoms: Non-smoker current vapers had lower risk compared to non-vaper current smokers.	2	[116,143]	Observational	Serious	Not serious	Not serious	Not serious	Major risk of publication bias; small number of studies	⊕⊕◯◯ Low
Incident risk of respiratory symptoms: Dual users had higher risk compared to never users	2	[116,143]	Observational	Serious	Serious	Not serious	Not serious	Major risk of publication bias; small number of studies	⊕◯◯◯ Very low
Incident risk of respiratory symptoms: Dual users had higher risk compared to non-smoker current vapers	2	[116,143]	Observational	Serious	Not serious	Not serious	Not serious	Major risk of publication bias; small number of studies	⊕⊕◯◯ Low
Incident risk of respiratory symptoms: Dual users had similar risk compared to non-vaper current smokers	2	[116,143]	Observational	Serious	Not serious	Not serious	Serious	Minor risk of publication bias; small number of studies	⊕◯◯◯ Very low
Prevalent risk of COPD: Non-smoker current vapers had statistically non-significant risk compared to never users	2	[43,106]	Observational	Not serious	Serious	Not serious	Serious	Major risk of publication bias; small number of studies	⊕◯◯◯ Very low
Prevalent risk of COPD: Dual users had higher risk compared to never users	2	[81,106]	Observational	Not serious	Serious	Not serious	Not serious	Major risk of publication bias; small number of studies	⊕⊕◯◯ Low
Prevalent risk of COPD: Dual users had similar risk compared to non-vaper current smokers	2	[81,106]	Observational	Not serious	Not serious	Not serious	Serious	Major risk of publication bias; small number of studies	⊕⊕◯◯ Low
**Narrative review findings**	**GRADE-CERQual Certainty assessment**								
**Summary**	**No. of studies**	**Studies**	**Study design**	**Methodological limitations**	**Coherence**	**Adequacy**	**Relevance**	**Explanation of CERQual assessment**	**Certainty**
Respiratory symptoms: higher risk in non-smoker current vapers	2	[50,96]	Observational	No/very minor	No/very minor concerns	Serious concerns	No/very minor	Serious concerns on adequacy of data	Moderate

compared to non-users				concerns		(No. of studies are very low)	concerns		
COPD: Higher risk in non-smoker current vapers and dual users compared to non-users	5	[24,54,92,106,142]	Observational	No/very minor concerns	No/very minor concerns	Minor concerns (No. of studies are low)	No/very minor concerns	Minor concerns on adequacy of data	Moderate
COPD: Higher risk following acute and short-to-medium term exposure to e-cigarettes compared to non-use	7	[60,64,112,113,145,148,150]	Experimental (cell/*in vitro*, animal)	Serious concerns (3 studies had high risk of bias)	No/very minor concerns	No/very minor concerns	Moderate concerns (Evidence is based on cell and animal studies)	Serious methodological limitations; and moderate concerns on relevance; moderate concerns on relevance	Low
Asthma: Higher risk among non-smoker current vapers compared to non-users, but no significant risk in dual users compared to non-users	13	[24,25,85,92,135,141,142]	Observational	Minor concerns (1 study with moderate risk of bias)	No/very minor concerns	No/very minor concerns	No/very minor concerns	Minor methodological limitations	Moderate
Asthma: Higher risk following acute and short-to-medium term exposure to e-cigarettes compared to non-use	4	[64,89,128,129]	Experimental (human, cell/*in vitro*, and animal)	Serious concerns (3 studies had high risk of bias)	No/very minor concerns	Minor concerns (number of studies were low)	Moderate concerns (Evidence is based on cell and animal studies)	Serious methodological limitations; minor concerns on adequacy of data; and moderate concerns on relevance	Very low
Impact on lung function: No significant risk following acute exposure, but higher risk following short-to-medium term exposure to e-cigarettes compared to non-use	8	[89,100,113,115,119,144,148,150]	Experimental (human and animal)	Serious concerns (2 studies had high risk of bias, and 1 study had pro-tobacco association)	No/very minor concerns	No/very minor concerns	Moderate concerns (Evidence is based on mostly animal studies)	Serious methodological limitations, and moderate concerns on relevance	low
Lung inflammation and damage: Higher risk in non-smoker current vapers compared to non-users.	5	[107,110,122,139,146]	Observational	Minor concerns (One study had moderate	No/very minor concerns	Minor concerns (No. of studies are low)	No/very minor concerns	Minor methodological limitations, and minor concerns on adequacy of data	Moderate

				risk of bias)					
Lung inflammation and damage: Higher risk following acute and short-to-medium term exposure to e-cigarettes compared to non-use, and similar risk following short-to-medium term exposure to e-cigarettes compared to cigarettes.	35	[35,39–42,47,55,56,59,60,63,66,70,73,75,83,88,89,93,94,97,101,113,115,117,119,121,127,131,132,138,140,144,148,149]	Experimental (human, cell/*in vitro*, and animal)		No/very minor concerns	No/very minor concerns	Moderate concerns (Evidence is based on 2 human non-randomized experimental, cell, and animal studies)	Serious methodological limitations, moderate concerns on relevance	Low
COVID-19 and Respiratory infections: Inconsistent findings on risk among non-smoker current vapers compared to non-users.	8	[62,72,78,79,95,107,109,147]	Observational	Serious concerns (4 studies had moderate risk of bias and 1 study had pro-tobacco association)	No/very minor concerns	No/very minor concerns	No/very minor concerns	Serious methodological limitations	Moderate
COVID-19 and Respiratory infections: Higher risk following acute and short-to-medium term exposure to e-cigarettes compared to non-use, but inconsistent findings on risk between e-cigarette exposure and cigarette exposure.	8	[42,64,88,108,111,112,118,149]	Experimental (cell/*in vitro*, animal)	Serious concerns (7 studies had high risk of bias)	No/very minor concerns	No/very minor concerns	Moderate concerns (Evidence is based on mainly cell and animal studies)	Serious methodological limitations; moderate concerns on relevance	Low
E-cigarette or vaping associated lung injury (EVALI): is mostly associated with cannabis vaping, not nicotine vaping	18	[48,51,53,61,67–69,71,74,80,82,87,99,105,114,126,134,136]	Observational	Moderate concerns (7 studies had moderate risk of bias)	No/very minor concerns	No/very minor concerns	Moderate concerns (Evidence is based on case reports and case series)	Moderate methodological limitations, moderate concerns on relevance	Moderate
Lung development in utero: Higher	7	[36,46,98	Experimental	Serious	No/very minor	No/very	Moderate	Serious methodological limitations,	Low

risk of impact following exposure to e-cigarettes compared to non-use		,102–104,124]	(animal)	concerns (1 study had high risk and 4 studies had moderate risk of bias)	concerns	minor concerns	concerns (Evidence is based on mainly animal studies)	moderate concerns on relevance	
Sex-based subgroup differences: Inconsistent findings for impact on asthma and COPD, impact on lung function, and lung development in utero.	9	[36,46,81,98,102,113,128,135,148]	Experimental (animal); observational	Serious concerns (At least 3 studies had high risk of bias)	No/very minor concerns	No/very minor concerns	Moderate concerns (Evidence is based on mainly animal studies)	Serious methodological limitations, moderate concerns on relevance	Low
Sex-based subgroup differences: No sex-based differences in lung inflammation and damage and lower risk of COVID-19 in females compared to males.	8	[91,113,148,151–155]	Experimental (animal); observational	Serious concerns (At least 1 study had high risk of bias)	No/very minor concerns	No/very minor concerns	Moderate concerns (Evidence is based on mainly animal studies)	Serious methodological limitations, moderate concerns on relevance	Low

Note: While using GRADE certainty assessment, if one study had moderate risk of bias, it was considered as having serious concerns; inconsistency is based on heterogeneity (I^2 and Q-test); indirectness on whether sample represents population; imprecision is based on whether 95% CI for individual studies crossed the threshold and whether the sample size was adequate.

Supplementary Material 8. References 37-164.

37. Austin-Datta RJ, Chaudhari PV, Cheng TYD, Klarenberg G, Striley CW, Cottler LB. Electronic Nicotine Delivery Systems (ENDS) use Among Members of a Community Engagement Program. *J Community Health*. 2023;48(2):338-346. doi:10.1007/s10900-022-01169-2

38. Baldovinos Y, Archer A, Salamanca J, Strongin RM, Sayes CM. Chemical Interactions and Cytotoxicity of Terpene and Diluent Vaping Ingredients. *Chem Res Toxicol*. 2023;36(4):589-597. doi:10.1021/acs.chemrestox.2c00218

39. Been T, Alakhtar B, Traboulsi H, et al. Chronic low-level JUUL aerosol exposure causes pulmonary immunologic, transcriptomic, and proteomic changes. *FASEB J*. 2023;37(2). doi:10.1096/fj.202201392R

40. Been T, Traboulsi H, Paoli S, et al. Differential impact of JUUL flavors on pulmonary immune modulation and oxidative stress responses in male and female mice. *Arch Toxicol*. 2022;96(6):1783-1798. doi:10.1007/s00204-022-03269-3

41. Begum R, Thota S, Batra S. Interplay between proteasome function and inflammatory responses in e-cig vapor condensate-challenged lung epithelial cells. *Arch Toxicol*. 2023;97(8):2193-2208. doi:10.1007/s00204-023-03504-5

42. Bhat TA, Kalathil SG, Leigh N, Hutson A, Goniewicz ML, Thanavala YM. Do alternative tobacco products induce less adverse respiratory risk than cigarettes? *Respir Res*. 2023;24(1):261. doi:10.1186/s12931-023-02568-2

43. Bircan E, Bezirhan U, Porter A, Fagan P, Orloff MS. Electronic cigarette use and its association with asthma, chronic obstructive pulmonary disease (COPD) and asthma-COPD overlap syndrome among never cigarette smokers. *Tob Induc Dis*. 2021;19(April). doi:10.18332/TID/132833

44. Brunette MF, Halenar MJ, Edwards KC, et al. Association between tobacco product use and asthma among US adults from the Population Assessment of Tobacco and Health (PATH) Study waves 2–4. *BMJ Open Respir Res*. 2023;10(1):e001187. doi:10.1136/bmjresp-2021-001187

45. Buu A, Yang JJ, Ou TS, Kyung Nam J, Suh G, Lin HC. An ecological momentary assessment study to examine covariates and effects of concurrent and simultaneous use of electronic cigarettes and marijuana among college students. *Addict Behav*. 2023;141:107662. doi:10.1016/j.addbeh.2023.107662

46. Cahill KM, Johnson TK, Perveen Z, et al. In utero exposures to mint-flavored JUUL aerosol impair lung development and aggravate house dust mite-induced asthma in adult offspring mice. *Toxicology*. 2022;477. doi:10.1016/j.tox.2022.153272

47. Caruso M, Emma R, Distefano A, et al. Electronic nicotine delivery systems exhibit reduced bronchial epithelial cells toxicity compared to cigarette: the Replica Project. *Sci Rep*. 2021;11(1). doi:10.1038/s41598-021-03310-y

48. Casamento Tumeo C, Schiavino A, Paglietti MG, et al. E-cigarette or Vaping product use Associated Lung Injury (EVALI) in a 15 year old female patient – case report. *Ital J Pediatr*. 2022;48(1). doi:10.1186/s13052-022-01314-6

49. Chaffee BW, Barrington-Trimis J, Liu F, et al. E-cigarette use and adverse respiratory symptoms among adolescents and Young adults in the United States. *Prev Med*. 2021;153. doi:10.1016/j.ypmed.2021.106766

50. Chaiton M, Pienkowski M, Musani I, et al. Smoking, e-cigarettes and the effect on respiratory symptoms among a population sample of youth: Retrospective cohort study. *Tob Induc Dis*. 2023;21. doi:10.18332/tid/156839

51. Chan BS, Kiss A, McIntosh N, Sheppeard V, Dawson AH. E-cigarette or vaping product use-associated lung injury in an adolescent. *Med J Aust*. 2021;215(7):313-314.e1. doi:10.5694/mja2.51244

52. Chandra D, Bogdanoff RF, Bowler RP, Benam KH. Electronic cigarette menthol flavoring is associated with increased inhaled micro and sub-micron particles and worse lung function in combustion cigarette smokers. *Respir Res*. 2023;24(1):108. doi:10.1186/s12931-023-02410-9

53. Collins PD, Meadows CIS, Lams BEA, Agarwal S, Wyncoll DLA. Diffuse "Tree-in-Bud" Pattern on High-Resolution Computed Tomography in Severe Vaping-induced Lung Injury. *Am J Respir Crit Care Med*. 2022;206(4):501-502. doi:10.1164/rccm.202112-2666IM

54. Cook SF, Hirschtick JL, Fleischer NL, et al. Cigarettes, ENDS Use, and Chronic Obstructive Pulmonary Disease Incidence: A Prospective Longitudinal Study. *Am J Prev Med*. 2023;65(2):173-181. doi:10.1016/j.amepre.2023.01.038

55. Da Silva PF, De Matos NA, Ramos CDO, et al. Acute Outcomes of Cigarette Smoke and Electronic Cigarette Aerosol Inhalation in a Murine Model. *BioMed Res Int*. 2022;2022. doi:10.1155/2022/9938179

56. Daou MAZ, Shihadeh A, Hashem Y, et al. Role of diabetes in lung injury from acute exposure to electronic cigarette, heated tobacco product, and combustible cigarette aerosols in an animal model. *PLoS ONE*. 2021;16(8 August). doi:10.1371/journal.pone.0255876

57. Davis ES, Ghosh A, Coakley RD, et al. Chronic E-Cigarette Exposure Alters Human Alveolar Macrophage Morphology and Gene Expression. *Nicotine Tob Res*. 2022;24(3):395-399. doi:10.1093/ntr/ntab186

58. Dirisanala S, Laller S, Ganti N, et al. E-cigarette use and prevalence of lung diseases among the U.S. population: a NHANES survey. *J Investig Med*. 2023;71(6):613-622. doi:10.1177/10815589231167357

59. Effah F, Elzein A, Taiwo B, Baines D, Bailey A, Marczylo T. In Vitro high-throughput toxicological assessment of E-cigarette flavors on human bronchial epithelial cells and the potential involvement of TRPA1 in cinnamon flavor-induced toxicity. *Toxicology*. 2023;496:153617. doi:10.1016/j.tox.2023.153617

60. Esquer C, Echeagaray O, Firouzi F, et al. Fundamentals of vaping-associated pulmonary injury leading to severe respiratory distress. *Life Sci Alliance*. 2022;5(2). doi:10.26508/LSA.202101246

61. Gaba M, Kumar N, Arumugam P, Dewan A. Vape-associated lung injury in immediate postoperative period: an upcoming perioperative respiratory risk factor. *BMJ Case Rep*. 2023;16(7):e255250. doi:10.1136/bcr-2023-255250

62. Gao M, Aveyard P, Lindson N, et al. Association between smoking, e-cigarette use and severe COVID-19: A cohort study. *Int J Epidemiol*. 2022;51(4):1062-1072. doi:10.1093/ije/dyac028

63. Getiye Y, Peterson MR, Phillips BD, Carrillo D, Bisha B, He G. E-cigarette exposure with or without heating the e-liquid induces differential remodeling in the lungs and right heart of mice. *J Mol Cell Cardiol*. 2022;168:83-95. doi:10.1016/j.yjmcc.2022.04.014

64. Ghosh A, Coakley RD, Alexis NE, Tarran R. Vaping-Induced Proteolysis Causes Airway Surface Dehydration. *Int J Mol Sci*. 2023;24(20):15348. doi:10.3390/ijms242015348

65. Goldberg Scott S, Feigelson HS, Powers JD, et al. Demographic, Clinical, and Behavioral Factors Associated With Electronic Nicotine Delivery Systems Use in a Large Cohort in the United States. *Tob Use Insights*. 2023;16:1179173X2211348-1179173X2211348. doi:10.1177/1179173x221134855

66. Goto S, Grange RMH, Pinciroli R, et al. Electronic cigarette vaping with aged coils causes acute lung injury in mice. *Arch Toxicol*. 2022;96(12):3363-3371. doi:10.1007/s00204-022-03388-x

67. Guarino C, Pedicelli I, Perna F, et al. E-cigarette, or vaping, product use associated lung injury (EVALI): new scenarios for physicians and radiologists. *Monaldi Arch Chest Dis*. 2022;92(3). doi:10.4081/monaldi.2021.1962

68. Gupta VS, Hayes D, Hsu SC, et al. Extracorporeal Life Support for Respiratory Failure in Patients With Electronic Cigarette or Vaping Product Use–Associated Lung Injury. *Crit Care Med*. 2022;50(2):E173-E182. doi:10.1097/CCM.0000000000005299

69. Harry-Hernandez S, Thiboutot J, Wahidi MM, et al. Bronchoalveolar Lavage (BAL) and Pathologic Assessment of Electronic Cigarette or Vaping Product Use-associated Lung Injury (EVALI): The EVALI-BAL Study, A Multicenter Cohort. *J Bronchol Interv Pulmonol*. 2023;30(2):144-154. doi:10.1097/LBR.0000000000000890

70. Hassan NH, El-Wafaey DI. Histopathological scoring system role in evaluation of electronic cigarette's impact on respiratory pathway in albino rat: Biochemical, histo-morphometric and ultrastructural study. *Tissue Cell*. 2022;79. doi:10.1016/j.tice.2022.101945

71. Helfgott D, Capozzoli G, Madray J, et al. E-cigarette or vaping product use associated lung injury (EVALI) in the time of COVID-19: A clinical dilemma. *Pediatr Pulmonol*. 2022;57(3):623-630. doi:10.1002/ppul.25804

72. Hickman E, Payton A, Duffney P, et al. Biomarkers of Airway Immune Homeostasis Differ Significantly with Generation of E-Cigarettes. *Am J Respir Crit Care Med*. 2022;206(10):1248-1258. doi:10.1164/rccm.202202-0373OC

73. Hinds DM, Nick HJ, Vallin TM, et al. Acute vaping in a golden Syrian hamster causes inflammatory response transcriptomic changes. *Am J Physiol - Lung Cell Mol Physiol*. 2022;323(5):L525-L535. doi:10.1152/ajplung.00162.2022

74. Hoekstra NE, Dannull KA, Weinman JP, Liptzin DR, Hinds DM. Vaping and diffuse alveolar hemorrhage: All EVALI is not created equal. *Pediatr Pulmonol*. 2021;56(12):4057-4059. doi:10.1002/ppul.25675

75. Husari A, El-Harakeh M, Shihadeh A, et al. The Substitution of Fifty Percent of Combustible Tobacco Smoke Exposure With Either Electronic Cigarettes or Heated tobacco Products Did Not Attenuate Acute Lung Injury in an Animal Model. *Nicotine Tob Res*. 2023;25(7):1361-1368. doi:10.1093/ntr/ntad045

76. Joshi D, Duong M, Kirkland S, Raina P. Impact of electronic cigarette ever use on lung function in adults aged 45-85: A cross-sectional analysis from the Canadian Longitudinal Study on Aging. *BMJ Open*. 2021;11(10). doi:10.1136/bmjopen-2021-051519

77. Kang HS, Kim JY, Park HJ, et al. E-cigarette-associated Severe Pneumonia in Korea Using Data Linkage between the Korea National Health and Nutrition Examination Survey (KNHANES, 2013–2019) and the National Health Insurance Service (NHIS) Claims Database. *J Korean Med Sci*. 2021;36(48). doi:10.3346/jkms.2021.36.e331

78. Kelesidis T, Sharma M, Satta S, et al. Ectodomain shedding of proteins important for SARS-CoV-2 pathogenesis in plasma of tobacco cigarette smokers compared to electronic cigarette vapers: a cross-sectional study. *J Mol Med*. 2023;101(3):327-335. doi:10.1007/s00109-023-02286-8

79. Kelesidis T, Zhang Y, Tran E, Sosa G, Middlekauff HR. Instigators of COVID-19 in Immune Cells Are Increased in Tobacco Cigarette Smokers and Electronic Cigarette Vapers Compared with Nonsmokers. *Nicotine Tob Res*. 2022;24(3):413-415. doi:10.1093/ntr/ntab168

80. Khan T, Huda AB, Al-Jibury M, Tin Z. A case of acute lung injury due to an e-cigarette. *Clin Med J R Coll Physicians Lond*. 2022;22:S16-S17. doi:10.7861/clinmed.22-4-s16

81. Kim T, Kang J. Association between dual use of e-cigarette and cigarette and chronic obstructive pulmonary disease: an analysis of a nationwide representative sample from 2013 to 2018. *BMC Pulm Med*. 2021;21(1). doi:10.1186/s12890-021-01590-8

82. Kligerman SJ, Kay FU, Raptis CA, et al. CT Findings and Patterns of e-Cigarette or Vaping Product Use-Associated Lung Injury: A Multicenter Cohort of 160 Cases. *Chest*. 2021;160(4):1492-1511. doi:10.1016/j.chest.2021.04.054

83. Komura M, Sato T, Yoshikawa H, et al. Propylene glycol, a component of electronic cigarette liquid, damages epithelial cells in human small airways. *Respir Res*. 2022;23(1). doi:10.1186/s12931-022-02142-2

84. Kubbara A, Hawari F, Johnkoski J. Diffuse alveolar haemorrhage secondary to haemophilus influenzae in a vaping patient. *BMJ Case Rep*. 2021;14(6). doi:10.1136/bcr-2021-242701

85. Lee SY, Shin J. Association between Electronic Cigarettes Use and Asthma in the United States: Data from the National Health Interview Survey 2016–2019. *Yonsei Med J*. 2023;64(1):54-65. doi:10.3349/ymj.2022.0292

86. Lee WK, Smith CL, Gao CX, et al. Are e-cigarette use and vaping associated with increased respiratory symptoms and poorer lung function in a population exposed to smoke from a coal mine fire? *Respirology*. 2021;26(10):974-981. doi:10.1111/resp.14113

87. Lucas LGDCS, Acha LFM, Lucas VS, Capone D. A 43-Year-Old Brazilian Man with Acute Impairment of Lung Function and Pulmonary Nodules with Features of Electronic Cigarette or Vaping Product Use-Associated Lung Injury (EVALI). *Am J Case Rep*. 2023;24. doi:10.12659/AJCR.939365

88. Maishan M, Sarma A, Chun LF, et al. Aerosolized nicotine from e-cigarettes alters gene expression, increases lung protein permeability, and impairs viral clearance in murine influenza infection. *Front Immunol*. 2023;14:1076772. doi:10.3389/fimmu.2023.1076772

89. Majek P, Jankowski M, Brożek GM. Acute health effects of heated tobacco products: comparative analysis with traditional cigarettes and electronic cigarettes in young adults. *ERJ Open Res*. 2023;9(3):00595-02022. doi:10.1183/23120541.00595-2022

90. Mccormick W, Baykara Y, Siddique A, Van Truong L, Corbett M, Hacking SM. Lung Findings in a Patient with a History of Nicotine Vaping and Cannabis Smoking. *R I Med J*. Published online 2022:36-40.

91. Merianos AL, Russell AM, Mahabee-Gittens EM, Barry AE, Yang M, Lin HC. Assessment of Exclusive, Dual, and Polytobacco E-Cigarette Use and COVID-19 Outcomes Among College Students. *Am J Health Promot*. 2022;36(3):421-428. doi:10.1177/08901171211055904

92. Mori KM, Mcelroy JP, Weng DY, et al. Lung mitochondrial DNA copy number, inflammatory biomarkers, gene transcription and gene methylation in vapers and smokers. Published online 2022. doi:10.1016/j

93. Morris AM, Leonard SS, Fowles JR, Boots TE, Mnatsakanova A, Attfield KR. Effects of E-cigarette flavoring chemicals on human macrophages and bronchial epithelial cells. *Int J Environ Res Public Health*. 2021;18(21). doi:10.3390/ijerph182111107

94. Moshensky A, Brand CS, Alhaddad H, et al. Effects of mango and mint pod-based e-cigarette aerosol inhalation on inflammatory states of the brain, lung, heart, and colon in mice. *eLife*. 2022;11. doi:10.7554/eLife.67621

95. Moyers SA, Hartwell M, Chiaf A, Greiner B, Oliver JA, Croff JM. Associations of Combustible Cigarette, Electronic Cigarette, and Dual Use With COVID Infection and Severity in the U.S.: A Cross-sectional Analysis of the 2021 National Health Information Survey. *Tob Use Insights*. 2023;16:1179173X2311796. doi:10.1177/1179173X231179675

96. Mukerjee R, Hirschtick JL, Arciniega LZ, et al. ENDS, Cigarettes, and Respiratory Illness: Longitudinal Associations Among U.S. Youth. *Am J Prev Med*. Published online December 2023:S0749379723005032. doi:10.1016/j.amepre.2023.12.005

97. Muthumalage T, Rahman I. Pulmonary immune response regulation, genotoxicity, and metabolic reprogramming by menthol- and tobacco-flavored e-cigarette exposures in mice. *Toxicol Sci*. 2023;193(2):146-165. doi:10.1093/toxsci/kfad033

98. Noël A, Yilmaz S, Farrow T, Schexnayder M, Eickelberg O, Jelesijevic T. Sex-Specific Alterations of the Lung Transcriptome at Birth in Mouse Offspring Prenatally Exposed to Vanilla-Flavored E-Cigarette Aerosols and Enhanced Susceptibility to Asthma. *Int J Environ Res Public Health*. 2023;20(4):3710. doi:10.3390/ijerph20043710

99. Norman FF, Diaz J, Martin-Davila P, et al. Bilateral pulmonary infiltrates in a traveller from Saudi Arabia with probable electronic cigarette or vaping associated lung injury (EVALI). *J Travel Med*. 2023;30(8):taad133. doi:10.1093/jtm/taad133

100. Nyilas S, Bauman G, Korten I, et al. MRI Shows Lung Perfusion Changes after Vaping and Smoking. *Radiology*. 2022;304(1):195-204. doi:10.1148/radiol.211327

101. Onyenwoke RU, Leung TC, Huang X, et al. An assessment of vaping-induced inflammation and toxicity: A feasibility study using a 2-stage zebrafish and mouse platform. *Food Chem Toxicol*. 2022;163. doi:10.1016/j.fct.2022.112923

102. Orzabal MR, Naik VD, Lee J, Wu G, Ramadoss J. Impact of gestational electronic cigarette vaping on amino acid signature profile in the pregnant mother and the fetus. *Metab Open*. 2021;11:100107-100107. doi:10.1016/j.metop.2021.100107

103. ORZABAL MR, NAIK VD, LEE J, et al. Impact of E-cig aerosol vaping on fetal and neonatal respiratory development and function. *Transl Res*. 2022;246:102-114. doi:10.1016/j.trsl.2022.03.009

104. Ozekin YH, Saal ML, Pineda RH, et al. Intrauterine exposure to nicotine through maternal vaping disrupts embryonic lung and skeletal development via the Kcnj2 potassium channel. *Dev Biol*. 2023;501:111-123. doi:10.1016/j.ydbio.2023.06.002

105. Pan HH, Tsao TF, Tsao SM, Sun HL, Lue KH. Electronic cigarette vaping product use is associated with lung injury in a 15-year-old adolescent. *Pediatr Neonatol*. 2022;63(6):651-652. doi:10.1016/j.pedneo.2022.05.006

106. Paulin LM, Halenar MJ, Edwards KC, et al. Association of tobacco product use with chronic obstructive pulmonary disease (COPD) prevalence and incidence in Waves 1 through 5 (2013–2019) of the Population Assessment of Tobacco and Health (PATH) Study. *Respir Res*. 2022;23(1). doi:10.1186/s12931-022-02197-1

107. Payton AD, Perryman AN, Hoffman JR, et al. Cytokine signature clusters as a tool to compare changes associated with tobacco product use in upper and lower airway samples. *Am J Physiol - Lung Cell Mol Physiol*. 2022;322(5):L722-L736. doi:10.1152/ajplung.00299.2021

108. Phandthong R, Wong M, Song A, Martinez T, Talbot P. New insights into how popular electronic cigarette aerosols and aerosol constituents affect SARS-CoV-2 infection of human bronchial epithelial cells. *Sci Rep*. 2023;13(1):5807. doi:10.1038/s41598-023-31592-x

109. Polosa R, Emma R, Cibella F, et al. Impact of exclusive e-cigarettes and heated tobacco products use on muco-ciliary clearance. *Ther Adv Chronic Dis*. 2021;12. doi:10.1177/20406223211035267

110. Pozuelos GL, Kagda M, Rubin MA, Goniewicz ML, Girke T, Talbot P. Transcriptomic Evidence That Switching from Tobacco to Electronic Cigarettes Does Not Reverse Damage to the Respiratory Epithelium. *Toxics*. 2022;10(7). doi:10.3390/toxics10070370

111. Raduka A, Gao N, Chatburn RL, Rezaee F. Electronic cigarette exposure disrupts airway epithelial barrier function and exacerbates viral infection. *Am J Physiol-Lung Cell Mol Physiol*. 2023;325(5):L580-L593. doi:10.1152/ajplung.00135.2023

112. Rasmussen LW, Stanford D, LaFontaine J, Allen AD, Raju SV. Nicotine aerosols diminish airway CFTR function and mucociliary clearance. *Am J Physiol-Lung Cell Mol Physiol*. 2023;324(5):L557-L570. doi:10.1152/ajplung.00453.2021

113. Rodriguez-Herrera AJ, De Souza ABF, Castro TDF, et al. Long-term e-cigarette aerosol exposure causes pulmonary emphysema in adult female and male mice. *Regul Toxicol Pharmacol*. 2023;142:105412. doi:10.1016/j.yrtph.2023.105412

114. Roman S, Millet C, Geris S, Manickam R, Mechineni A. Crazy vaping and crazy-paving, a case of E-Cigarette/Vaping-Associated Lung Injury (EVALI) with chest CT showing crazy-paving pattern. *Radiol Case Rep*. 2021;16(11):3208-3212. doi:10.1016/j.radcr.2021.07.058

115. Roxlau ET, Pak O, Hadzic S, et al. Nicotine promotes e-cigarette vapour-induced lung inflammation and structural alterations. *Eur Respir J*. 2023;61(6):2200951. doi:10.1183/13993003.00951-2022

116. Sargent JD, Halenar MJ, Edwards KC, et al. Tobacco Use and Respiratory Symptoms Among Adults: Findings From the Longitudinal Population Assessment of Tobacco and Health (PATH) Study 2014-2016. *Nicotine Tob Res*. 2022;24(10):1607-1618. doi:10.1093/ntr/ntac080

117. Sayed IM, Masso-Silva JA, Mittal A, et al. Inflammatory phenotype modulation in the respiratory tract and systemic circulation of e-cigarette users: a pilot study. *Am J Physiol - Lung Cell Mol Physiol*. 2021;321(6):L1134-L1146. doi:10.1152/ajplung.00363.2021

118. Schaunaman N, Crue T, Cervantes D, et al. Electronic cigarette vapor exposure exaggerates the pro-inflammatory response during influenza A viral infection in human distal airway epithelium. *Arch Toxicol*. 2022;96(8):2319-2328. doi:10.1007/s00204-022-03305-2

119. Scieszka DP, Garland D, Hunter R, et al. Multi-omic assessment shows dysregulation of pulmonary and systemic immunity to e-cigarette exposure. *Respir Res*. 2023;24(1):138. doi:10.1186/s12931-023-02441-2

120. Sezgin ME, Duman B, Colak M, Özdemir C, Dalar L. Acute Eosinophilic Pneumonia Associated with E-Cigarettes: A Case Report. *Respir Case Rep*. 2023;12(3):105-108. doi:10.5505/respircase.2023.16023

121. Shi J, Dai W, Chavez J, et al. One Acute Exposure to E-Cigarette Smoke Using Various Heating Elements and Power Levels Induces Pulmonary Inflammation. *Cardiol Res*. 2022;13(6):323-332. doi:10.14740/CR1425

122. Shields PG, Ying KL, Brasky TM, et al. A Pilot Cross-Sectional Study of Immunological and Microbiome Profiling Reveals Distinct Inflammatory Profiles for Smokers, Electronic Cigarette Users, and Never-Smokers. *Microorganisms*. 2023;11(6):1405. doi:10.3390/microorganisms11061405

123. Shiffman S, Oliveri DR, Goldenson NI, Liang Q, Black RA, Mishra S. Comparing Adult Smokers Who Switched to JUUL versus Continuing Smokers: Biomarkers of Exposure and of Potential Harm and Respiratory Symptoms. *Nicotine Tob Res*. Published online October 14, 2023:ntad197. doi:10.1093/ntr/ntad197

124. Silva-Ribeiro T, Coelho E, Genisheva Z, et al. Comparative study of e-cigarette aerosol and cigarette smoke effect on ex vivo embryonic chick lung explants. *Toxicol Lett*. 2023;376:13-19. doi:10.1016/j.toxlet.2023.01.002

125. Sinha I, Goel R, Bitzer ZT, Trushin N, Liao J, Sinha R. Evaluating electronic cigarette cytotoxicity and inflammatory responses in vitro. *Tob Induc Dis*. 2022;20(5). doi:10.18332/tid/147200

126. Smith JM, Smedley M, Kansra S, Kulkarni H. Vaping induced lung injury in a 14-year-old girl. *Pediatr Pulmonol*. 2022;57(1):320-321. doi:10.1002/ppul.25705

127. Snoderly HT, Alkhadrawi H, Panchal DM, et al. Short-term exposure of female BALB/cJ mice to e-cigarette aerosol promotes neutrophil recruitment and enhances neutrophil-platelet aggregation in pulmonary microvasculature. *J Toxicol Environ Health A*. 2023;86(8):246-262. doi:10.1080/15287394.2023.2184738

128. Song MA, Kim JY, Gorr MW, et al. Sex-specific lung inflammation and mitochondrial damage in a model of electronic cigarette exposure in asthma. *Am J Physiol-Lung Cell Mol Physiol*. 2023;325(5):L568-L579. doi:10.1152/ajplung.00033.2023

129. Song MA, Wold LE, Aslaner DM, et al. Long-Term Impact of Daily E-cigarette Exposure on the Lungs of Asthmatic Mice. *Nicotine Tob Res*. 2023;25(12):1904-1908. doi:10.1093/ntr/ntad100

130. Soybel A, DeJaco V, Ellison-Barnes A, Galiatsatos P. Sarcoidosis Associated With Electronic Cigarette Use in an Adult: A Case Report. *J Med Cases*. 2022;13(3):95-98. doi:10.14740/jmc3887

131. Su VYF, Chen WC, Yu WK, Wu HH, Chen H, Yang KY. The main e-cigarette component vegetable glycerin enhances neutrophil migration and fibrosis in endotoxin-induced lung injury via p38 MAPK activation. *Respir Res*. 2023;24(1). doi:10.1186/s12931-022-02307-z

132. Suryadinata RV, Wirjatmadi B. The molecular pathways of lung damage by e-cigarettes in male wistar rats. *Sultan Qaboos Univ Med J*. 2021;21(3):436-441. doi:10.18295/squmj.4.2021.003

133. Takigawa Y, Sato K, Inoue A, et al. Acute eosinophilic pneumonia caused by nicotine-free vaping in an adolescent patient: A case report. *Respirol Case Rep*. 2022;10(6). doi:10.1002/rcr2.961

134. Tanz LJ, Christensen A, Knuth KB, et al. Characteristics of an Outbreak of E-cigarette, or Vaping, Product Use-Associated Lung Injury-North Carolina, 2019. *N C Med J*. 2021;82(6):384-392. doi:10.18043/ncm.82.6.384

135. To T, Borkhoff CM, Chow CW, et al. Vaping and Health Service Use: A Canadian Health Survey and Health Administrative Data Study. *Ann Am Thorac Soc*. 2023;20(6):815-824. doi:10.1513/AnnalsATS.202207-578OC

136. Triantafyllou GA, Tiberio PJ, Zou RH, et al. Long-term outcomes of EVALI: a 1-year retrospective study. *Lancet Respir Med*. 2021;9(12):e112-e113. doi:10.1016/S2213-2600(21)00415-X

137. Varella MH, Andrade OA, Shaffer SM, et al. E-cigarette use and respiratory symptoms in residents of the United States: A BRFSS report. *PLoS ONE*. 2022;17(12 December). doi:10.1371/journal.pone.0269760

138. Wang L, Wang Y, Chen J, et al. Comparison of biological and transcriptomic effects of conventional cigarette and electronic cigarette smoke exposure at toxicological dose in BEAS-2B cells. *Ecotoxicol Environ Saf*. 2021;222. doi:10.1016/j.ecoenv.2021.112472

139. Wetherill RR, Doot RK, Young AJ, et al. Molecular Imaging of Pulmonary Inflammation in Users of Electronic and Combustible Cigarettes: A Pilot Study. *J Nucl Med*. 2023;64(5):797-802. doi:10.2967/jnumed.122.264529

140. Wick KD, Fang X, Maishan M, et al. Impact of e-cigarette aerosol on primary human alveolar epithelial type 2 cells. *Am J Physiol - Lung Cell Mol Physiol*. 2022;323(2):L152-L164. doi:10.1152/ajplung.00503.2021

141. Williams RJ, Wills TA, Choi K, Pagano I. Associations for subgroups of E-cigarette, cigarette, and cannabis use with asthma in a population sample of California adolescents. *Addict Behav*. 2023;145:107777. doi:10.1016/j.addbeh.2023.107777

142. Wills TA, Choi K, Pokhrel P, Pagano I. Tests for confounding with cigarette smoking in the association of E-cigarette use with respiratory disorder: 2020 National-Sample Data. *Prev Med*. 2022;161. doi:10.1016/j.ypmed.2022.107137

143. Xie W, Tackett AP, Berlowitz JB, et al. Association of Electronic Cigarette Use with Respiratory Symptom Development among U.S. Young Adults. *Am J Respir Crit Care Med*. 2022;205(11):1320-1329. doi:10.1164/rccm.202107-1718OC

144. Yang W, Yang X, Jiang L, et al. Combined biological effects and lung proteomics analysis in mice reveal different toxic impacts of electronic cigarette aerosol and combustible cigarette smoke on the respiratory system. *Arch Toxicol*. 2022;96(12):3331-3347. doi:10.1007/s00204-022-03378-z

145. Yanina IYu, Genin VD, Genina EA, et al. Multimodal Diagnostics of Changes in Rat Lungs after Vaping. *Diagnostics*. 2023;13(21):3340. doi:10.3390/diagnostics13213340

146. Ying KL, Brasky TM, Freudenheim JL, et al. Saliva and Lung Microbiome Associations with Electronic Cigarette Use and Smoking. *Cancer Prev Res (Phila Pa)*. 2022;15(7):435-446. doi:10.1158/1940-6207.CAPR-21-0601

147. Young-Wolff KC, Slama NE, Alexeeff SE, Prochaska JJ, Fogelberg R, Sakoda LC. Electronic cigarette use and risk of COVID-19 among young adults without a history of cigarette smoking. *Prev Med*. 2022;162. doi:10.1016/j.ypmed.2022.107151

148. Zhang J, Cheng H, Xue M, et al. Effects of chronic electronic cigarettes exposure in inducing respiratory function decline and pulmonary tissue injury – A direct comparison to combustible cigarettes. *Ecotoxicol Environ Saf*. 2023;249. doi:10.1016/j.ecoenv.2022.114426

149. Zhang R, Jones MM, Parker D, et al. Acute vaping exacerbates microbial pneumonia due to calcium (Ca2+) dysregulation. *PLoS ONE*. 2021;16(8 August). doi:10.1371/journal.pone.0256166

150. Zhao HZ, Guo ZW, Wang ZL, et al. A comparative study of the effects of electronic cigarette and traditional cigarette on the pulmonary functions of C57BL/6 male mice. *Nicotine Tob Res Off J Soc Res Nicotine Tob*. Published online August 3, 2023:ntad139. doi:10.1093/ntr/ntad139

151. Ghosh A, Coakley RD, Ghio AJ, et al. Chronic E-cigarette use increases neutrophil elastase and matrix metalloprotease levels in the lung. *Am J Respir Crit Care Med*. 2019;200(11):1392-1401. doi:10.1164/rccm.201903-0615OC

152. Lallai V, Manca L, Fowler CD. E-cigarette vape and lung ACE2 expression: Implications for coronavirus vulnerability. *Environ Toxicol Pharmacol*. 2021;86. doi:10.1016/j.etap.2021.103656

153. Naidu V, Zeki AA, Sharma P. Sex differences in the induction of angiotensin converting enzyme 2 (ACE-2) in mouse lungs after e-cigarette vapor exposure and its relevance to COVID-19. *J Investig Med*. 2021;69(5):954-961. doi:10.1136/jim-2020-001768

154. Wang Q, Ahmad Khan N, Muthumalage T, et al. Dysregulated repair and inflammatory responses by e-cigarette-derived inhaled nicotine and humectant propylene glycol in a sex-dependent manner in mouse lung. *FASEB BioAdvances*. 2019;1(10):609-623. doi:10.1096/fba.2019-00048

155. Wang Q, Sundar IK, Li D, et al. E-cigarette-induced pulmonary inflammation and dysregulated repair are mediated by nAChR α7 receptor: Role of nAChR α7 in SARS-CoV-2 Covid-19 ACE2 receptor regulation. *Respir Res*. 2020;21(1). doi:10.1186/s12931-020-01396-y

156. Alqahtani MM, Alenezi FK, Almeshari MA, et al. E-cigarette use and respiratory symptoms in adults: A systematic review and meta-analysis. *Tob Induc Dis*. 2023;21(December):1-14. doi:10.18332/tid/174660

157. Glantz S, Jeffers A, Winickoff JP. Nicotine Addiction and Intensity of e-Cigarette Use by Adolescents in the US, 2014 to 2021. *JAMA Netw Open*. 2022;5(11):e2240671-e2240671. doi:10.1001/jamanetworkopen.2022.40671

158. Li X, Zhang Y, Zhang R, Chen F, Shao L, Zhang L. Association Between E-Cigarettes and Asthma in Adolescents: A Systematic Review and Meta-Analysis. *Am J Prev Med*. 2022;62(6):953-960. doi:10.1016/j.amepre.2022.01.015

159. Chand BR, Hosseinzadeh H. Association between e-cigarette use and asthma: a systematic review and meta-analysis. *J Asthma Off J Assoc Care Asthma*. 2022;59(9):1722-1731. doi:10.1080/02770903.2021.1971703

160. Honeycutt L, Huerne K, Miller A, et al. A systematic review of the effects of e-cigarette use on lung function. *Npj Prim Care Respir Med 2022 321*. 2022;32(1):1-7. doi:10.1038/s41533-022-00311-w

161. Merecz-Sadowska A, Sitarek P, Zielinska-Blizniewska H, et al. A Summary of In Vitro and In Vivo Studies Evaluating the Impact of E-Cigarette Exposure on Living Organisms and the Environment. *Int J Mol Sci*. 2020;21(2):652. doi:10.3390/ijms21020652

162. Blount BC, Karwowski MP, Shields PG, et al. Vitamin E Acetate in Bronchoalveolar-Lavage Fluid Associated with EVALI. *N Engl J Med*. 2020;382(8):697-705. doi:10.1056/NEJMoa1916433

163. Jonas AM, Raj R. Vaping-Related Acute Parenchymal Lung Injury: A Systematic Review. *Chest*. 2020;158(4):1555-1565. doi:10.1016/j.chest.2020.03.085

164. Brook J, Turner M. How safe are e-cigarettes for patients with COPD? A systematic review. *Pract Nurs*. 2022;33(3):105-111. doi:10.12968/pnur.2022.33.3.105

Supplementary Material 9: Data _retrieved from literature search_ N_5_125_

No. of studies	Reference of articles	Included	Reasons for exclusion	Stage of exclusion
1	Alsaid AH, Elfaki A, Alkhouzaie MT, Alghamdi RA. A rare case of acute eosinophilic pneumonia induced by vaping-associated lung injury: a case report. BMC Pulm Med. 2023;23: 291. doi:10.1186/s12890-023-02581-7	Yes	Not applicable	Not applicable
2	Alzoubi KH, Khabour OF, Al-Sawalha NA, Karaoghlanian N, Shihadeh A, Eissenberg T. Time course of changes in inflammatory and oxidative biomarkers in lung tissue of mice induced by exposure to electronic cigarette aerosol. Toxicol Rep. 2022;9: 1484–1490. doi:10.1016/j.toxrep.2022.07.001	Yes	Not applicable	Not applicable
3	Aslaner DM, Alghothani O, Saldana TA, Ezell KG, Yallourakis MD, MacKenzie DM, et al. E-cigarette vapor exposure in utero causes long-term pulmonary effects in offspring. Am J Physiol - Lung Cell Mol Physiol. 2022;323: L676–L682. doi:10.1152/ajplung.00233.2022	Yes	Not applicable	Not applicable
4	Austin-Datta RJ, Chaudhari PV, Cheng T-YD, Klarenberg G, Striley CW, Cottler LB. Electronic Nicotine Delivery Systems (ENDS) use Among Members of a Community Engagement Program. J Community Health. 2023;48: 338–346. doi:10.1007/s10900-022-01169-2	Yes	Not applicable	Not applicable
5	Baldovinos Y, Archer A, Salamanca J, Strongin RM, Sayes CM. Chemical Interactions and Cytotoxicity of Terpene and Diluent Vaping Ingredients. Chem Res Toxicol. 2023;36: 589–597. doi:10.1021/acs.chemrestox.2c00218	Yes	Not applicable	Not applicable
6	Been T, Alakhtar B, Traboulsi H, Tsering T, Bartolomucci A, Heimbach N, et al. Chronic low-level JUUL aerosol exposure causes pulmonary immunologic, transcriptomic, and proteomic changes. FASEB J. 2023;37. doi:10.1096/fj.202201392R	Yes	Not applicable	Not applicable
7	Been T, Traboulsi H, Paoli S, Alakhtar B, Mann KK, Eidelman DH, et al. Differential impact of JUUL flavors on pulmonary immune modulation and oxidative stress responses in male and female mice. Arch Toxicol. 2022;96: 1783–1798. doi:10.1007/s00204-022-03269-3	Yes	Not applicable	Not applicable
8	Begum R, Thota S, Batra S. Interplay between proteasome function and inflammatory responses in e-cig vapor condensate-challenged lung epithelial cells. Arch Toxicol. 2023;97: 2193–2208. doi:10.1007/s00204-023-03504-5	Yes	Not applicable	Not applicable
9	Bhat TA, Kalathil SG, Leigh N, Hutson A, Goniewicz ML, Thanavala YM. Do alternative tobacco products induce less adverse respiratory risk than cigarettes? Respir Res. 2023;24: 261. doi:10.1186/s12931-023-02568-2	Yes	Not applicable	Not applicable
10	Bircan E, Bezirhan U, Porter A, Fagan P, Orloff MS. Electronic cigarette use and its association with asthma, chronic obstructive pulmonary disease (COPD) and asthma-COPD overlap syndrome among never cigarette smokers. Tob Induc Dis. 2021;19. doi:10.18332/TID/132833	Yes	Not applicable	Not applicable
11	Brunette MF, Halenar MJ, Edwards KC, Taylor KA, Emond JA, Tanski SE, et al. Association between tobacco product use and asthma among US adults from the Population Assessment of Tobacco and Health (PATH) Study waves 2–4. BMJ Open Respir Res. 2023;10: e001187. doi:10.1136/bmjresp-2021-001187	Yes	Not applicable	Not applicable
12	Buu A, Yang JJ, Ou T-S, Kyung Nam J, Suh G, Lin H-C. An ecological momentary assessment study to examine covariates and effects of concurrent and simultaneous use of electronic cigarettes and marijuana among college students. Addict Behav. 2023;141: 107662. doi:10.1016/j.addbeh.2023.107662	Yes	Not applicable	Not applicable
13	Cahill KM, Johnson TK, Perveen Z, Schexnayder M, Xiao R, Heffernan LM, et al. In utero exposures to mint-flavored JUUL aerosol impair lung development and aggravate house dust mite-induced asthma in adult offspring mice. Toxicology. 2022;477. doi:10.1016/j.tox.2022.153272	Yes	Not applicable	Not applicable
14	Caruso M, Emma R, Distefano A, Rust S, Poulas K, Zadjali F, et al. Electronic nicotine delivery systems exhibit reduced bronchial epithelial cells toxicity compared to cigarette: the Replica Project. Sci Rep. 2021;11. doi:10.1038/s41598-021-03310-y	Yes	Not applicable	Not applicable
15	Casamento Tumeo C, Schiavino A, Paglietti MG, Petreschi F, Ottavianelli A, Onofri A, et al. E-cigarette or Vaping product use Associated Lung Injury (EVALI) in a 15 year old female patient – case report. Ital J Pediatr. 2022;48. doi:10.1186/s13052-022-01314-6	Yes	Not applicable	Not applicable
16	Chaffee BW, Barrington-Trimis J, Liu F, Wu R, McConnell R, Krishnan-Sarin S, et al. E-cigarette use and adverse respiratory symptoms among adolescents and Young adults in the United States. Prev Med. 2021;153. doi:10.1016/j.ypmed.2021.106766	Yes	Not applicable	Not applicable
17	Chaiton M, Pienkowski M, Musani I, Bondy SJ, Cohen JE, Dubray J, et al. Smoking, e-cigarettes and the effect on respiratory symptoms among a population sample of youth: Retrospective cohort study. Tob Induc Dis. 2023;21. doi:10.18332/tid/156839	Yes	Not applicable	Not applicable
18	Chan BS, Kiss A, McIntosh N, Sheppeard V, Dawson AH. E-cigarette or vaping product use-associated lung injury in an adolescent. Med J Aust. 2021;215: 313-314.e1. doi:10.5694/mja2.51244	Yes	Not applicable	Not applicable
19	Chandra D, Bogdanoff RF, Bowler RP, Benam KH. Electronic cigarette menthol flavoring is associated with increased inhaled micro and sub-micron particles and worse lung function in combustion cigarette smokers. Respir Res. 2023;24: 108. doi:10.1186/s12931-023-02410-9	Yes	Not applicable	Not applicable
20	Collins PD, Meadows CIS, Lams BEA, Agarwal S, Wyncoll DLA. Diffuse "Tree-in-Bud" Pattern on High-Resolution Computed Tomography in Severe Vaping-induced Lung Injury. Am J Respir Crit Care Med. 2022;206: 501–502. doi:10.1164/rccm.202112-2666IM	Yes	Not applicable	Not applicable
21	Cook SF, Hirschtick JL, Fleischer NL, Arenberg DA, Barnes GD, Levy DT, et al. Cigarettes, ENDS Use, and Chronic Obstructive Pulmonary Disease Incidence: A Prospective Longitudinal Study. Am J Prev Med. 2023;65: 173–181. doi:10.1016/j.amepre.2023.01.038	Yes	Not applicable	Not applicable
22	Cordova J, Pfeiffer RM, Choi K, Grana Mayne R, Baker L, Bachand J, et al. Tobacco use profiles by respiratory disorder status for adults in the wave 1-wave 4 population assessment of tobacco and health (PATH) study. Prev Med Rep. 2022;30. doi:10.1016/j.pmedr.2022.102016	Yes	Not applicable	Not applicable
23	Da Silva PF, De Matos NA, Ramos CDO, Castro TDF, Araújo NPDS, De Souza ABF, et al. Acute Outcomes of Cigarette Smoke and Electronic Cigarette Aerosol Inhalation in a Murine Model. BioMed Res Int. 2022;2022. doi:10.1155/2022/9938179	Yes	Not applicable	Not applicable
24	Daou MAZ, Shihadeh A, Hashem Y, Bitar H, Kassir A, El-Harakeh M, et al. Role of diabetes in lung injury from acute exposure to electronic cigarette, heated tobacco product, and combustible cigarette aerosols in an animal model. PLoS ONE. 2021;16. doi:10.1371/journal.pone.0255876	Yes	Not applicable	Not applicable
25	Davis ES, Ghosh A, Coakley RD, Wrennall JA, Lubamba BA, Rowell TR, et al. Chronic E-Cigarette Exposure Alters Human Alveolar Macrophage Morphology and Gene Expression. Nicotine Tob Res. 2022;24: 395–399. doi:10.1093/ntr/ntab186	Yes	Not applicable	Not applicable
26	Dirisanala S, Laller S, Ganti N, Taj S, Patel N, Singh Arora K, et al. E-cigarette use and prevalence of lung diseases among the U.S. population: a NHANES survey. J Investig Med. 2023;71: 613–622. doi:10.1177/10815589231167357	Yes	Not applicable	Not applicable
27	Effah F, Elzein A, Taiwo B, Baines D, Bailey A, Marczylo T. In Vitro high-throughput toxicological assessment of E-cigarette flavors on human bronchial epithelial cells and the potential involvement of TRPA1 in cinnamon flavor-induced toxicity. Toxicology. 2023;496: 153617. doi:10.1016/j.tox.2023.153617	Yes	Not applicable	Not applicable
28	Esquer C, Echeagaray O, Firouzi F, Savko C, Shain G, Bose P, et al. Fundamentals of vaping-associated pulmonary injury leading to severe respiratory distress. Life Sci Alliance. 2022;5. doi:10.26508/LSA.202101246	Yes	Not applicable	Not applicable
29	Gaba M, Kumar N, Arumugam P, Dewan A. Vape-associated lung injury in immediate postoperative period: an upcoming perioperative respiratory risk factor. BMJ Case Rep. 2023;16: e255250. doi:10.1136/bcr-2023-255250	Yes	Not applicable	Not applicable
30	Gao M, Aveyard P, Lindson N, Hartmann-Boyce J, Watkinson P, Young D, et al. Association between smoking, e-cigarette use and severe COVID-19: A cohort study. Int J Epidemiol. 2022;51: 1062–1072. doi:10.1093/ije/dyac028	Yes	Not applicable	Not applicable
31	Getiye Y, Peterson MR, Phillips BD, Carrillo D, Bisha B, He G. E-cigarette exposure with or without heating the e-liquid induces differential remodeling in the lungs and right heart of mice. J Mol Cell Cardiol. 2022;168: 83–95. doi:10.1016/j.yjmcc.2022.04.014	Yes	Not applicable	Not applicable
32	Ghosh A, Coakley RD, Alexis NE, Tarran R. Vaping-Induced Proteolysis Causes Airway Surface Dehydration. Int J Mol Sci. 2023;24: 15348. doi:10.3390/ijms242015348	Yes	Not applicable	Not applicable

33	Goldberg Scott S, Feigelson HS, Powers JD, Clennin MN, Lyons JA, Gray MT, et al. Demographic, Clinical, and Behavioral Factors Associated With Electronic Nicotine Delivery Systems Use in a Large Cohort in the United States. Tob Use Insights. 2023;16: 1179173X2211348-1179173X2211348. doi:10.1177/1179173x221134855	Yes	Not applicable	Not applicable
34	Goto S, Grange RMH, Pinciroli R, Rosales IA, Li R, Boerboom SL, et al. Electronic cigarette vaping with aged coils causes acute lung injury in mice. Arch Toxicol. 2022;96: 3363–3371. doi:10.1007/s00204-022-03388-x	Yes	Not applicable	Not applicable
35	Guarino C, Pedicelli I, Perna F, Di Spirito V, Fiorentino G, Procaccini F, et al. E-cigarette, or vaping, product use associated lung injury (EVALI): new scenarios for physicians and radiologists. Monaldi Arch Chest Dis. 2022;92. doi:10.4081/monaldi.2021.1962	Yes	Not applicable	Not applicable
36	Gupta VS, Hayes D, Hsu SC, Tonna JE, Rycus PT, Bridges BC, et al. Extracorporeal Life Support for Respiratory Failure in Patients With Electronic Cigarette or Vaping Product Use–Associated Lung Injury. Crit Care Med. 2022;50: E173–E182. doi:10.1097/CCM.0000000000005299	Yes	Not applicable	Not applicable
37	Harry-Hernandez S, Thiboutot J, Wahidi MM, Giovacchini CX, De Cardenas J, Meldrum C, et al. Bronchoalveolar Lavage (BAL) and Pathologic Assessment of Electronic Cigarette or Vaping Product Use-associated Lung Injury (EVALI): The EVALI-BAL Study, A Multicenter Cohort. J Bronchol Interv Pulmonol. 2023;30: 144–154. doi:10.1097/LBR.0000000000000890	Yes	Not applicable	Not applicable
38	Hassan NH, El-Wafaey DI. Histopathological scoring system role in evaluation of electronic cigarette's impact on respiratory pathway in albino rat: Biochemical, histo-morphometric and ultrastructural study. Tissue Cell. 2022;79. doi:10.1016/j.tice.2022.101945	Yes	Not applicable	Not applicable
39	Helfgott D, Capozzoli G, Madray J, Baig A, Uppaluri L, Gaur S, et al. E-cigarette or vaping product use associated lung injury (EVALI) in the time of COVID-19: A clinical dilemma. Pediatr Pulmonol. 2022;57: 623–630. doi:10.1002/ppul.25804	Yes	Not applicable	Not applicable
40	Hickman E, Payton A, Duffney P, Wells H, Ceppe AS, Brocke S, et al. Biomarkers of Airway Immune Homeostasis Differ Significantly with Generation of E-Cigarettes. Am J Respir Crit Care Med. 2022;206: 1248–1258. doi:10.1164/rccm.202202-0373OC	Yes	Not applicable	Not applicable
41	Hinds DM, Nick HJ, Vallin TM, Bloomquist LA, Christeson S, Bratcher PE, et al. Acute vaping in a golden Syrian hamster causes inflammatory response transcriptomic changes. Am J Physiol - Lung Cell Mol Physiol. 2022;323: L525–L535. doi:10.1152/ajplung.00162.2022	Yes	Not applicable	Not applicable
42	Hoekstra NE, Dannull KA, Weinman JP, Liptzin DR, Hinds DM. Vaping and diffuse alveolar hemorrhage: All EVALI is not created equal. Pediatr Pulmonol. 2021;56: 4057–4059. doi:10.1002/ppul.25675	Yes	Not applicable	Not applicable
43	Husari A, El-Harakeh M, Shihadeh A, Daou MAZ, Bitar H, Karaoghlanian N, et al. The Substitution of Fifty Percent of Combustible Tobacco Smoke Exposure With Either Electronic Cigarettes or Heated tobacco Products Did Not Attenuate Acute Lung Injury in an Animal Model. Nicotine Tob Res. 2023;25: 1361–1368. doi:10.1093/ntr/ntad045	Yes	Not applicable	Not applicable
44	Joshi D, Duong M, Kirkland S, Raina P. Impact of electronic cigarette ever use on lung function in adults aged 45-85: A cross-sectional analysis from the Canadian Longitudinal Study on Aging. BMJ Open. 2021;11. doi:10.1136/bmjopen-2021-051519	Yes	Not applicable	Not applicable
45	Kang HS, Kim JY, Park HJ, Jung JW, Choi HS, Park JS, et al. E-cigarette-associated Severe Pneumonia in Korea Using Data Linkage between the Korea National Health and Nutrition Examination Survey (KNHANES, 2013–2019) and the National Health Insurance Service (NHIS) Claims Database. J Korean Med Sci. 2021;36. doi:10.3346/jkms.2021.36.e331	Yes	Not applicable	Not applicable
46	Kelesidis T, Sharma M, Satta S, Tran E, Gupta R, Araujo JA, et al. Ectodomain shedding of proteins important for SARS-CoV-2 pathogenesis in plasma of tobacco cigarette smokers compared to electronic cigarette vapers: a cross-sectional study. J Mol Med. 2023;101: 327–335. doi:10.1007/s00109-023-02286-8	Yes	Not applicable	Not applicable
47	Kelesidis T, Zhang Y, Tran E, Sosa G, Middlekauff HR. Instigators of COVID-19 in Immune Cells Are Increased in Tobacco Cigarette Smokers and Electronic Cigarette Vapers Compared with Nonsmokers. Nicotine Tob Res. 2022;24: 413–415. doi:10.1093/ntr/ntab168	Yes	Not applicable	Not applicable
48	Khan T, Huda AB, Al-Jibury M, Tin Z. A case of acute lung injury due to an e-cigarette. Clin Med J R Coll Physicians Lond. 2022;22: S16–S17. doi:10.7861/clinmed.22-4-s16	Yes	Not applicable	Not applicable
49	Kim T, Kang J. Association between dual use of e-cigarette and cigarette and chronic obstructive pulmonary disease: an analysis of a nationwide representative sample from 2013 to 2018. BMC Pulm Med. 2021;21. doi:10.1186/s12890-021-01590-8	Yes	Not applicable	Not applicable
50	Kligerman SJ, Kay FU, Raptis CA, Henry TS, Sechrist JW, Walker CM, et al. CT Findings and Patterns of e-Cigarette or Vaping Product Use-Associated Lung Injury: A Multicenter Cohort of 160 Cases. Chest. 2021;160: 1492–1511. doi:10.1016/j.chest.2021.04.054	Yes	Not applicable	Not applicable
51	Komura M, Sato T, Yoshikawa H, Nitta NA, Suzuki Y, Koike K, et al. Propylene glycol, a component of electronic cigarette liquid, damages epithelial cells in human small airways. Respir Res. 2022;23. doi:10.1186/s12931-022-02142-2	Yes	Not applicable	Not applicable
52	Kubbara A, Hawari F, Johnkoski J. Diffuse alveolar haemorrhage secondary to haemophilus influenzae in a vaping patient. BMJ Case Rep. 2021;14. doi:10.1136/bcr-2021-242701	Yes	Not applicable	Not applicable
53	Lee SY, Shin J. Association between Electronic Cigarettes Use and Asthma in the United States: Data from the National Health Interview Survey 2016–2019. Yonsei Med J. 2023;64: 54–65. doi:10.3349/ymj.2022.0292	Yes	Not applicable	Not applicable
54	Lee WK, Smith CL, Gao CX, Borg BM, Nilsen K, Brown D, et al. Are e-cigarette use and vaping associated with increased respiratory symptoms and poorer lung function in a population exposed to smoke from a coal mine fire? Respirology. 2021;26: 974–981. doi:10.1111/resp.14113	Yes	Not applicable	Not applicable
55	Lucas LGDCS, Acha LFM, Lucas VS, Capone D. A 43-Year-Old Brazilian Man with Acute Impairment of Lung Function and Pulmonary Nodules with Features of Electronic Cigarette or Vaping Product Use-Associated Lung Injury (EVALI). Am J Case Rep. 2023;24. doi:10.12659/AJCR.939365	Yes	Not applicable	Not applicable
56	Maishan M, Sarma A, Chun LF, Caldera S, Fang X, Abbott J, et al. Aerosolized nicotine from e-cigarettes alters gene expression, increases lung protein permeability, and impairs viral clearance in murine influenza infection. Front Immunol. 2023;14: 1076772. doi:10.3389/fimmu.2023.1076772	Yes	Not applicable	Not applicable
57	Majek P, Jankowski M, Brożek GM. Acute health effects of heated tobacco products: comparative analysis with traditional cigarettes and electronic cigarettes in young adults. ERJ Open Res. 2023;9: 00595–02022. doi:10.1183/23120541.00595-2022	Yes	Not applicable	Not applicable
58	Mccormick W, Baykara Y, Siddique A, Van Truong L, Corbett M, Hacking SM. Lung Findings in a Patient with a History of Nicotine Vaping and Cannabis Smoking. R I Med J. 2022; 36–40.	Yes	Not applicable	Not applicable
59	Merianos AL, Russell AM, Mahabee-Gittens EM, Barry AE, Yang M, Lin HC. Assessment of Exclusive, Dual, and Polytobacco E-Cigarette Use and COVID-19 Outcomes Among College Students. Am J Health Promot. 2022;36: 421–428. doi:10.1177/08901171211055904	Yes	Not applicable	Not applicable
60	Mori KM, Mcelroy JP, Weng DY, Chung S, Fadda P, Reisinger SA, et al. Lung mitochondrial DNA copy number, inflammatory biomarkers, gene transcription and gene methylation in vapers and smokers. 2022. doi:10.1016/j	Yes	Not applicable	Not applicable
61	Morris AM, Leonard SS, Fowles JR, Boots TE, Mnatsakanova A, Attfield KR. Effects of E-cigarette flavoring chemicals on human macrophages and bronchial epithelial cells. Int J Environ Res Public Health. 2021;18. doi:10.3390/ijerph182111107	Yes	Not applicable	Not applicable
62	Moshensky A, Brand CS, Alhaddad H, Shin J, Masso-Silva JA, Advani I, et al. Effects of mango and mint pod-based e-cigarette aerosol inhalation on inflammatory states of the brain, lung, heart, and colon in mice. eLife. 2022;11. doi:10.7554/eLife.67621	Yes	Not applicable	Not applicable
63	Moyers SA, Hartwell M, Chiaf A, Greiner B, Oliver JA, Croff JM. Associations of Combustible Cigarette, Electronic Cigarette, and Dual Use With COVID Infection and Severity in the U.S.: A Cross-sectional Analysis of the 2021 National Health Information Survey. Tob Use Insights. 2023;16: 1179173X2311796. doi:10.1177/1179173X231179675	Yes	Not applicable	Not applicable

64	Mukerjee R, Hirschtick JL, Arciniega LZ, Xie Y, Barnes GD, Arenberg DA, et al. ENDS, Cigarettes, and Respiratory Illness: Longitudinal Associations Among U.S. Youth. Am J Prev Med. 2023; S0749379723005032. doi:10.1016/j.amepre.2023.12.005	Yes	Not applicable	Not applicable
65	Muthumalage T, Rahman I. Pulmonary immune response regulation, genotoxicity, and metabolic reprogramming by menthol- and tobacco-flavored e-cigarette exposures in mice. Toxicol Sci. 2023;193: 146–165. doi:10.1093/toxsci/kfad033	Yes	Not applicable	Not applicable
66	Noël A, Yilmaz S, Farrow T, Schexnayder M, Eickelberg O, Jelesijevic T. Sex-Specific Alterations of the Lung Transcriptome at Birth in Mouse Offspring Prenatally Exposed to Vanilla-Flavored E-Cigarette Aerosols and Enhanced Susceptibility to Asthma. Int J Environ Res Public Health. 2023;20: 3710. doi:10.3390/ijerph20043710	Yes	Not applicable	Not applicable
67	Norman FF, Diaz J, Martin-Davila P, Tato M, García-San Miguel L, Gil E, et al. Bilateral pulmonary infiltrates in a traveller from Saudi Arabia with probable electronic cigarette or vaping associated lung injury (EVALI). J Travel Med. 2023;30: taad133. doi:10.1093/jtm/taad133	Yes	Not applicable	Not applicable
68	Nyilas S, Bauman G, Korten I, Pusterla O, Singer F, Ith M, et al. MRI Shows Lung Perfusion Changes after Vaping and Smoking. Radiology. 2022;304: 195–204. doi:10.1148/radiol.211327	Yes	Not applicable	Not applicable
69	Onyenwoke RU, Leung TC, Huang X, Parker DJ, Shipman JG, Alhadyan SK, et al. An assessment of vaping-induced inflammation and toxicity: A feasibility study using a 2-stage zebrafish and mouse platform. Food Chem Toxicol. 2022;163. doi:10.1016/j.fct.2022.112923	Yes	Not applicable	Not applicable
70	Orzabal MR, Naik VD, Lee J, Wu G, Ramadoss J. Impact of gestational electronic cigarette vaping on amino acid signature profile in the pregnant mother and the fetus. Metab Open. 2021;11: 100107–100107. doi:10.1016/j.metop.2021.100107	Yes	Not applicable	Not applicable
71	ORZABAL MR, NAIK VD, LEE J, HILLHOUSE AE, BRASHEAR WA, THREADGILL DW, et al. Impact of E-cig aerosol vaping on fetal and neonatal respiratory development and function. Transl Res. 2022;246: 102–114. doi:10.1016/j.trsl.2022.03.009	Yes	Not applicable	Not applicable
72	Ozekin YH, Saal ML, Pineda RH, Moehn K, Ordonez-Erives MA, Delgado Figueroa MF, et al. Intrauterine exposure to nicotine through maternal vaping disrupts embryonic lung and skeletal development via the Kcnj2 potassium channel. Dev Biol. 2023;501: 111–123. doi:10.1016/j.ydbio.2023.06.002	Yes	Not applicable	Not applicable
73	Pan HH, Tsao TF, Tsao SM, Sun HL, Lue KH. Electronic cigarette vaping product use is associated with lung injury in a 15-year-old adolescent. Pediatr Neonatol. 2022;63: 651–652. doi:10.1016/j.pedneo.2022.05.006	Yes	Not applicable	Not applicable
74	Patel A, Cook S, Mattingly DT, Barnes GD, Arenberg DA, Levy DT, et al. Longitudinal Association Between Exclusive and Dual Use of Cigarettes and Electronic Nicotine Delivery Systems and Asthma Among U.S. Adolescents. J Adolesc Health. 2023;73: 437–444. doi:10.1016/j.jadohealth.2023.04.009	Yes	Not applicable	Not applicable
75	Paulin LM, Halenar MJ, Edwards KC, Lauten K, Stanton CA, Taylor K, et al. Association of tobacco product use with chronic obstructive pulmonary disease (COPD) prevalence and incidence in Waves 1 through 5 (2013–2019) of the Population Assessment of Tobacco and Health (PATH) Study. Respir Res. 2022;23. doi:10.1186/s12931-022-02197-1	Yes	Not applicable	Not applicable
76	Payton AD, Perryman AN, Hoffman JR, Avula V, Wells H, Robinette C, et al. Cytokine signature clusters as a tool to compare changes associated with tobacco product use in upper and lower airway samples. Am J Physiol - Lung Cell Mol Physiol. 2022;322: L722–L736. doi:10.1152/ajplung.00299.2021	Yes	Not applicable	Not applicable
77	Phandthong R, Wong M, Song A, Martinez T, Talbot P. New insights into how popular electronic cigarette aerosols and aerosol constituents affect SARS-CoV-2 infection of human bronchial epithelial cells. Sci Rep. 2023;13: 5807. doi:10.1038/s41598-023-31592-x	Yes	Not applicable	Not applicable
78	Polosa R, Emma R, Cibella F, Caruso M, Conte G, Benfatto F, et al. Impact of exclusive e-cigarettes and heated tobacco products use on muco-ciliary clearance. Ther Adv Chronic Dis. 2021;12. doi:10.1177/20406223211035267	Yes	Not applicable	Not applicable
79	Pozuelos GL, Kagda M, Rubin MA, Goniewicz ML, Girke T, Talbot P. Transcriptomic Evidence That Switching from Tobacco to Electronic Cigarettes Does Not Reverse Damage to the Respiratory Epithelium. Toxics. 2022;10. doi:10.3390/toxics10070370	Yes	Not applicable	Not applicable
80	Raduka A, Gao N, Chatburn RL, Rezaee F. Electronic cigarette exposure disrupts airway epithelial barrier function and exacerbates viral infection. Am J Physiol-Lung Cell Mol Physiol. 2023;325: L580–L593. doi:10.1152/ajplung.00135.2023	Yes	Not applicable	Not applicable
81	Rasmussen LW, Stanford D, LaFontaine J, Allen AD, Raju SV. Nicotine aerosols diminish airway CFTR function and mucociliary clearance. Am J Physiol-Lung Cell Mol Physiol. 2023;324: L557–L570. doi:10.1152/ajplung.00453.2021	Yes	Not applicable	Not applicable
82	Rodriguez-Herrera AJ, De Souza ABF, Castro TDF, Machado-Junior PA, Marcano-Gomez EC, Menezes TP, et al. Long-term e-cigarette aerosol exposure causes pulmonary emphysema in adult female and male mice. Regul Toxicol Pharmacol. 2023;142: 105412. doi:10.1016/j.yrtph.2023.105412	Yes	Not applicable	Not applicable
83	Roman S, Millet C, Geris S, Manickam R, Mechineni A. Crazy vaping and crazy-paving, a case of E-Cigarette/Vaping-Associated Lung Injury (EVALI) with chest CT showing crazy-paving pattern. Radiol Case Rep. 2021;16: 3208–3212. doi:10.1016/j.radcr.2021.07.058	Yes	Not applicable	Not applicable
84	Roxlau ET, Pak O, Hadzic S, Garcia-Castro CF, Gredic M, Wu C-Y, et al. Nicotine promotes e-cigarette vapour-induced lung inflammation and structural alterations. Eur Respir J. 2023;61: 2200951. doi:10.1183/13993003.00951-2022	Yes	Not applicable	Not applicable
85	Sargent JD, Halenar MJ, Edwards KC, Woloshin S, Schwartz L, Emond J, et al. Tobacco Use and Respiratory Symptoms Among Adults: Findings From the Longitudinal Population Assessment of Tobacco and Health (PATH) Study 2014-2016. Nicotine Tob Res. 2022;24: 1607–1618. doi:10.1093/ntr/ntac080	Yes	Not applicable	Not applicable
86	Sayed IM, Masso-Silva JA, Mittal A, Patel A, Lin E, Moshensky A, et al. Inflammatory phenotype modulation in the respiratory tract and systemic circulation of e-cigarette users: a pilot study. Am J Physiol - Lung Cell Mol Physiol. 2021;321: L1134–L1146. doi:10.1152/ajplung.00363.2021	Yes	Not applicable	Not applicable
87	Schaunaman N, Crue T, Cervantes D, Schweitzer K, Robbins H, Day BJ, et al. Electronic cigarette vapor exposure exaggerates the pro-inflammatory response during influenza A viral infection in human distal airway epithelium. Arch Toxicol. 2022;96: 2319–2328. doi:10.1007/s00204-022-03305-2	Yes	Not applicable	Not applicable
88	Scieszka DP, Garland D, Hunter R, Herbert G, Lucas S, Jin Y, et al. Multi-omic assessment shows dysregulation of pulmonary and systemic immunity to e-cigarette exposure. Respir Res. 2023;24: 138. doi:10.1186/s12931-023-02441-2	Yes	Not applicable	Not applicable
89	Sezgin ME, Duman B, Colak M, Özdemir C, Dalar L. Acute Eosinophilic Pneumonia Associated with E-Cigarettes: A Case Report. Respir Case Rep. 2023;12: 105–108. doi:10.5505/respircase.2023.16023	Yes	Not applicable	Not applicable
90	Shi J, Dai W, Chavez J, Carreno J, Zhao L, Kleinman MT, et al. One Acute Exposure to E-Cigarette Smoke Using Various Heating Elements and Power Levels Induces Pulmonary Inflammation. Cardiol Res. 2022;13: 323–332. doi:10.14740/CR1425	Yes	Not applicable	Not applicable
91	Shields PG, Ying KL, Brasky TM, Freudenheim JL, Li Z, McElroy JP, et al. A Pilot Cross-Sectional Study of Immunological and Microbiome Profiling Reveals Distinct Inflammatory Profiles for Smokers, Electronic Cigarette Users, and Never-Smokers. Microorganisms. 2023;11: 1405. doi:10.3390/microorganisms11061405	Yes	Not applicable	Not applicable
92	Shiffman S, Oliveri DR, Goldenson NI, Liang Q, Black RA, Mishra S. Comparing Adult Smokers Who Switched to JUUL versus Continuing Smokers: Biomarkers of Exposure and of Potential Harm and Respiratory Symptoms. Nicotine Tob Res. 2023; ntad197. doi:10.1093/ntr/ntad197	Yes	Not applicable	Not applicable
93	Silva-Ribeiro T, Coelho E, Genisheva Z, Oliveira JM, Correia-Pinto J, Sampaio P, et al. Comparative study of e-cigarette aerosol and cigarette smoke effect on ex vivo embryonic chick lung explants. Toxicol Lett. 2023;376: 13–19. doi:10.1016/j.toxlet.2023.01.002	Yes	Not applicable	Not applicable
94	Sinha I, Goel R, Bitzer ZT, Trushin N, Liao J, Sinha R. Evaluating electronic cigarette cytotoxicity and inflammatory responses in vitro. Tob Induc Dis. 2022;20. doi:10.18332/tid/147200	Yes	Not applicable	Not applicable
95	Smith JM, Smedley M, Kansra S, Kulkarni H. Vaping induced lung injury in a 14-year-old girl. Pediatr Pulmonol. 2022;57: 320–321. doi:10.1002/ppul.25705	Yes	Not applicable	Not applicable

96	Snoderly HT, Alkhadrawi H, Panchal DM, Weaver KL, Vito JN, Freshwater KA, et al. Short-term exposure of female BALB/cJ mice to e-cigarette aerosol promotes neutrophil recruitment and enhances neutrophil-platelet aggregation in pulmonary microvasculature. J Toxicol Environ Health A. 2023;86: 246–262. doi:10.1080/15287394.2023.2184738	Yes	Not applicable	Not applicable
97	Song M-A, Kim JY, Gorr MW, Miller RA, Karpurapu M, Nguyen J, et al. Sex-specific lung inflammation and mitochondrial damage in a model of electronic cigarette exposure in asthma. Am J Physiol-Lung Cell Mol Physiol. 2023;325: L568–L579. doi:10.1152/ajplung.00033.2023	Yes	Not applicable	Not applicable
98	Song M-A, Wold LE, Aslaner DM, Archer KJ, Patel D, Jeon H, et al. Long-Term Impact of Daily E-cigarette Exposure on the Lungs of Asthmatic Mice. Nicotine Tob Res. 2023;25: 1904–1908. doi:10.1093/ntr/ntad100	Yes	Not applicable	Not applicable
99	Soybel A, DeJaco V, Ellison-Barnes A, Galiatsatos P. Sarcoidosis Associated With Electronic Cigarette Use in an Adult: A Case Report. J Med Cases. 2022;13: 95–98. doi:10.14740/jmc3887	Yes	Not applicable	Not applicable
100	Su VYF, Chen WC, Yu WK, Wu HH, Chen H, Yang KY. The main e-cigarette component vegetable glycerin enhances neutrophil migration and fibrosis in endotoxin-induced lung injury via p38 MAPK activation. Respir Res. 2023;24. doi:10.1186/s12931-022-02307-z	Yes	Not applicable	Not applicable
101	Suryadinata RV, Wirjatmadi B. The molecular pathways of lung damage by e-cigarettes in male wistar rats. Sultan Qaboos Univ Med J. 2021;21: 436–441. doi:10.18295/squmj.4.2021.003	Yes	Not applicable	Not applicable
102	Takigawa Y, Sato K, Inoue A, Nagae M, Inoue T, Onishi K, et al. Acute eosinophilic pneumonia caused by nicotine-free vaping in an adolescent patient: A case report. Respirol Case Rep. 2022;10. doi:10.1002/rcr2.961	Yes	Not applicable	Not applicable
103	Tanz LJ, Christensen A, Knuth KB, Hoffman MN, Dandeneau D, Koehler K, et al. Characteristics of an Outbreak of E-cigarette, or Vaping, Product Use-Associated Lung Injury-North Carolina, 2019. N C Med J. 2021;82: 384–392. doi:10.18043/ncm.82.6.384	Yes	Not applicable	Not applicable
104	To T, Borkhoff CM, Chow C-W, Moraes TJ, Schwartz R, Vozoris N, et al. Vaping and Health Service Use: A Canadian Health Survey and Health Administrative Data Study. Ann Am Thorac Soc. 2023;20: 815–824. doi:10.1513/AnnalsATS.202207-578OC	Yes	Not applicable	Not applicable
105	Triantafyllou GA, Tiberio PJ, Zou RH, Lynch MJ, Kreit JW, McVerry BJ, et al. Long-term outcomes of EVALI: a 1-year retrospective study. Lancet Respir Med. 2021;9: e112–e113. doi:10.1016/S2213-2600(21)00415-X	Yes	Not applicable	Not applicable
106	Varella MH, Andrade OA, Shaffer SM, Castro G, Rodriguez P, Barengo NC, et al. E-cigarette use and respiratory symptoms in residents of the United States: A BRFSS report. PLoS ONE. 2022;17. doi:10.1371/journal.pone.0269760	Yes	Not applicable	Not applicable
107	Wang L, Wang Y, Chen J, Yang XM, Jiang XT, Liu P, et al. Comparison of biological and transcriptomic effects of conventional cigarette and electronic cigarette smoke exposure at toxicological dose in BEAS-2B cells. Ecotoxicol Environ Saf. 2021;222. doi:10.1016/j.ecoenv.2021.112472	Yes	Not applicable	Not applicable
108	Wetherill RR, Doot RK, Young AJ, Lee H, Schubert EK, Wiers CE, et al. Molecular Imaging of Pulmonary Inflammation in Users of Electronic and Combustible Cigarettes: A Pilot Study. J Nucl Med. 2023;64: 797–802. doi:10.2967/jnumed.122.264529	Yes	Not applicable	Not applicable
109	Wick KD, Fang X, Maishan M, Matsumoto S, Spottiswoode N, Sarma A, et al. Impact of e-cigarette aerosol on primary human alveolar epithelial type 2 cells. Am J Physiol - Lung Cell Mol Physiol. 2022;323: L152–L164. doi:10.1152/ajplung.00503.2021	Yes	Not applicable	Not applicable
110	Williams RJ, Wills TA, Choi K, Pagano I. Associations for subgroups of E-cigarette, cigarette, and cannabis use with asthma in a population sample of California adolescents. Addict Behav. 2023;145: 107777. doi:10.1016/j.addbeh.2023.107777	Yes	Not applicable	Not applicable
111	Wills TA, Choi K, Pokhrel P, Pagano I. Tests for confounding with cigarette smoking in the association of E-cigarette use with respiratory disorder: 2020 National-Sample Data. Prev Med. 2022;161. doi:10.1016/j.ypmed.2022.107137	Yes	Not applicable	Not applicable
112	Xie W, Tackett AP, Berlowitz JB, Harlow AF, Kathuria H, Galiatsatos P, et al. Association of Electronic Cigarette Use with Respiratory Symptom Development among U.S. Young Adults. Am J Respir Crit Care Med. 2022;205: 1320–1329. doi:10.1164/rccm.202107-1718OC	Yes	Not applicable	Not applicable
113	Yang W, Yang X, Jiang L, Song H, Huang G, Duan K, et al. Combined biological effects and lung proteomics analysis in mice reveal different toxic impacts of electronic cigarette aerosol and combustible cigarette smoke on the respiratory system. Arch Toxicol. 2022;96: 3331–3347. doi:10.1007/s00204-022-03378-z	Yes	Not applicable	Not applicable
114	Yanina IYu, Genin VD, Genina EA, Mudrak DA, Navolokin NA, Bucharskaya AB, et al. Multimodal Diagnostics of Changes in Rat Lungs after Vaping. Diagnostics. 2023;13: 3340. doi:10.3390/diagnostics13213340	Yes	Not applicable	Not applicable
115	Ying KL, Brasky TM, Freudenheim JL, McElroy JP, Nickerson QA, Song MA, et al. Saliva and Lung Microbiome Associations with Electronic Cigarette Use and Smoking. Cancer Prev Res (Phila Pa). 2022;15: 435–446. doi:10.1158/1940-6207.CAPR-21-0601	Yes	Not applicable	Not applicable
116	Young-Wolff KC, Slama NE, Alexeeff SE, Prochaska JJ, Fogelberg R, Sakoda LC. Electronic cigarette use and risk of COVID-19 among young adults without a history of cigarette smoking. Prev Med. 2022;162. doi:10.1016/j.ypmed.2022.107151	Yes	Not applicable	Not applicable
117	Zhang J, Cheng H, Xue M, Xiong Y, Zhu Y, Björkegren JLM, et al. Effects of chronic electronic cigarettes exposure in inducing respiratory function decline and pulmonary tissue injury – A direct comparison to combustible cigarettes. Ecotoxicol Environ Saf. 2023;249. doi:10.1016/j.ecoenv.2022.114426	Yes	Not applicable	Not applicable
118	Zhang R, Jones MM, Parker D, Dornsife RE, Wymer N, Onyenwoke RU, et al. Acute vaping exacerbates microbial pneumonia due to calcium (Ca2+) dysregulation. PLoS ONE. 2021;16. doi:10.1371/journal.pone.0256166	Yes	Not applicable	Not applicable
119	Zhao H-Z, Guo Z-W, Wang Z-L, Wang C, Luo X-Y, Han N-N, et al. A comparative study of the effects of electronic cigarette and traditional cigarette on the pulmonary functions of C57BL/6 male mice. Nicotine Tob Res Off J Soc Res Nicotine Tob. 2023; ntad139. doi:10.1093/ntr/ntad139	Yes	Not applicable	Not applicable
120	Hirschtick JL, Cook S, Patel A, Barnes GD, Arenberg D, Bondarenko I, et al. Longitudinal Associations between Exclusive and Dual Use of Electronic Nicotine Delivery Systems and Cigarettes and Self-Reported Incident Diagnosed Cardiovascular Disease among Adults. Nicotine Tob Res. 2023;25: 386–394. doi:10.1093/ntr/ntac182	No	Evaluated cardiovascular effects of e-cigarette use	Full text screening
121	Klonizakis M, Gumber A, McIntosh E, Brose LS. Medium- and longer-term cardiovascular effects of e-cigarettes in adults making a stop-smoking attempt: a randomized controlled trial. BMC Med. 2022;20. doi:10.1186/s12916-022-02451-9	No	Evaluated cardiovascular effects of e-cigarette use	Full text screening
122	Klonizakis M, Gumber A, McIntosh E, Brose LS. Short-term cardiovascular effects of e-cigarettes in adults making a stop-smoking attempt: A randomized controlled trial. Biology. 2021;10. doi:10.3390/biology10111208	No	Evaluated cardiovascular effects of e-cigarette use	Full text screening
123	MacLean RR, Gueorguieva R, DeVito EE, Peltier MR, Parida S, Sofuoglu M. The Effects of Inhaled Flavors on Intravenous Nicotine. Exp Clin Psychopharmacol. 2021;29: 615–624. doi:10.1037/pha0000394	No	Evaluated cardiovascular effects of e-cigarette use	Full text screening
124	Gueorguieva R, Schwartz EKC, MacLean RR, DeVito EE, Eid T, Wu R, et al. Plasma Menthol Glucuronide as a Biomarker for the Behavioral Effects of Menthol and Nicotine in Humans. Front Pharmacol. 2022;13. doi:10.3389/fphar.2022.844824	No	Evaluated cardiovascular effects of e-cigarette use	Full text screening
125	Dimitriadis K, Narkiewicz K, Leontsinis I, Konstantinidis D, Mihas C, Andrikou I, et al. Acute Effects of Electronic and Tobacco Cigarette Smoking on Sympathetic Nerve Activity and Blood Pressure in Humans. Int J Environ Res Public Health. 2022;19. doi:10.3390/ijerph19063237	No	Evaluated cardiovascular effects of e-cigarette use	Full text screening
126	Felicione NJ, Douglas AE, Joseph McClernon F, Blank MD. Preliminary Evaluation of Short-Term Abstinence Effects Among Never-Smoking Experienced Users of Modern Electronic Cigarettes. Nicotine Tob Res. 2022;24: 1125–1129. doi:10.1093/ntr/ntab252	No	Evaluated cardiovascular effects of e-cigarette use	Full text screening
127	Shahin K, West SL, Brenner IKM. Acute Cardiovascular Effects of Vaping Compared to Cigarette Smoking in Young Adults. McGill J Med. 2022;20. doi:10.26443/mjm.v20i1.861	No	Evaluated cardiovascular	Full text screening

			effects of e-cigarette use	
128	Majid S, Weisbrod RM, Fetterman JL, Keith RJ, Rizvi SHM, Zhou Y, et al. Pod-based e-liquids impair human vascular endothelial cell function. PLoS ONE. 2023;18. doi:10.1371/journal.pone.0280674	No	Evaluated cardiovascular effects of e-cigarette use	Full text screening
129	Lorkiewicz P, Keith R, Lynch J, Jin L, Theis W, Krivokhizhina T, et al. Electronic Cigarette Solvents, JUUL E-Liquids, and Biomarkers of Exposure: In Vivo Evidence for Acrolein and Glycidol in E-Cig-Derived Aerosols. Chem Res Toxicol. 2022;35: 283–292. doi:10.1021/acs.chemrestox.1c00328	No	Evaluated cardiovascular effects of e-cigarette use	Full text screening
130	Mahoney MC, Rivard C, Kimmel HL, Hammad HT, Sharma E, Halenar MJ, et al. Cardiovascular Outcomes among Combustible-Tobacco and Electronic Nicotine Delivery System (ENDS) Users in Waves 1 through 5 of the Population Assessment of Tobacco and Health (PATH) Study, 2013–2019. Int J Environ Res Public Health. 2022;19. doi:10.3390/ijerph19074137	No	Evaluated cardiovascular effects of e-cigarette use	Full text screening
131	Berlowitz JB, Xie W, Harlow AF, Hamburg NM, Blaha MJ, Bhatnagar A, et al. E-Cigarette Use and Risk of Cardiovascular Disease: A Longitudinal Analysis of the PATH Study (2013-2019). Circulation. 2022;145: 1557–1559. doi:10.1161/CIRCULATIONAHA.121.057369	No	Evaluated cardiovascular effects of e-cigarette use	Full text screening
132	Mueller SD, Britton GR, James GD, Stewart Fahs P. Vaping behaviour patterns and daily blood pressure and heart rate variation: a brief report. Ann Hum Biol. 2021;48: 535–539. doi:10.1080/03014460.2021.2010803	No	Evaluated cardiovascular effects of e-cigarette use	Full text screening
133	Shi H, Leventhal AM, Wen Q, Ossip DJ, Li D. Sex Differences in the Association of E-cigarette and Cigarette Use and Dual Use with Self-Reported Hypertension Incidence in US Adults. Nicotine Tob Res. 2022;XX: 1–8. doi:10.1093/ntr/ntac170	No	Evaluated cardiovascular effects of e-cigarette use	Full text screening
134	Bricknell RAT, Ducaud C, Figueroa A, Schwarzman LS, Rodriguez P, Castro G, et al. An association between electronic nicotine delivery systems use and a history of stroke using the 2016 behavioral risk factor surveillance system. Med U S. 2021;100. doi:10.1097/MD.0000000000027180	No	Evaluated cardiovascular effects of e-cigarette use	Full text screening
135	Okafor CN, Okafor N, Kaliszewski C, Wang L. Association between electronic cigarette and combustible cigarette use with cardiometabolic risk biomarkers among U.S. adults. Ann Epidemiol. 2022;71: 44–50. doi:10.1016/j.annepidem.2022.02.002	No	Evaluated cardiovascular effects of e-cigarette use	Full text screening
136	Critcher CR, Siegel M. Re-examining the Association Between E-Cigarette Use and Myocardial Infarction: A Cautionary Tale. Am J Prev Med. 2021;61: 474–482. doi:10.1016/j.amepre.2021.05.003	No	Evaluated cardiovascular effects of e-cigarette use	Full text screening
137	Kim SY, Jeong SH, Joo HJ, Park M, Park E-C, Kim JH, et al. High prevalence of hypertension among smokers of conventional and e-cigarette: Using the nationally representative community dwelling survey. Front Public Health. 2022;10. doi:10.3389/fpubh.2022.919585	No	Evaluated cardiovascular effects of e-cigarette use	Full text screening
138	Liu X, Yuan Z, Ji Y. The association between electronic cigarettes, sleep duration, and the adverse cardiovascular outcomes: Findings from behavioral risk factor surveillance system, 2020. Front Cardiovasc Med. 2022;9: 909383. doi:10.3389/fcvm.2022.909383	No	Evaluated cardiovascular effects of e-cigarette use	Full text screening
139	Falk GE, Hayrettin Okut PhD, Mohinder R. Vindhyal M.Ed. MD, Elizabeth Ablah MPH PhD. Hypertension and Cardiovascular Diseases among Electronic and Combustible Cigarette Users. Kans J Med. 2022;15: 226–230. doi:10.17161/kjm.vol15.16752	No	Evaluated cardiovascular effects of e-cigarette use	Full text screening
140	Mohammadi L, Han DD, Xu F, Huang A, Derakhshandeh R, Rao P, et al. Chronic E-Cigarette Use Impairs Endothelial Function on the Physiological and Cellular Levels. Arterioscler Thromb Vasc Biol. 2022;42: 1333–1350. doi:10.1161/ATVBAHA.121.317749	No	Evaluated cardiovascular effects of e-cigarette use	Full text screening
141	Patel U, Patel N, Khurana M, Parulekar A, Patel A, Ortiz JF, et al. Effect Comparison of E-Cigarette and Traditional Smoking and Association with Stroke—A Cross-Sectional Study of NHANES. Neurol Int. 2022;14: 441–452. doi:10.3390/neurolint14020037	No	Evaluated cardiovascular effects of e-cigarette use	Full text screening
142	Metzen D, M'Pembele R, Zako S, Mourikis P, Helten C, Zikeli D, et al. Platelet reactivity is higher in e-cigarette vaping as compared to traditional smoking. Int J Cardiol. 2021;343: 146–148. doi:10.1016/j.ijcard.2021.09.005	No	Evaluated cardiovascular effects of e-cigarette use	Full text screening
143	Podzolkov VI, Bragina AE, Druzhinina NA, Mohammadi LN. E-cigarette smoking (vaping) and markers of vascular wall damage in young subjects without cardiovascular disease. Ration Pharmacother Cardiol. 2021;17: 521–527. doi:10.20996/1819-6446-2021-08-04	No	Evaluated cardiovascular effects of e-cigarette use	Full text screening
144	Amraotkar AR, Owolabi US, Malovichko MV, Majid S, Weisbrod RM, Benjamin EJ, et al. Association of electronic cigarette use with circulating angiogenic cell levels in healthy young adults: Evidence for chronic systemic injury. Vasc Med U K. 2023;28: 18–27. doi:10.1177/1358863X221126205	No	Evaluated cardiovascular effects of e-cigarette use	Full text screening
145	Glenski TA, Dorris CE, Patel GM, Taylor CM, Doyle NM. Vaping Associated Cardiac Arrest at School in a Teenager with Anomalous Left Coronary Artery. Mo Med. 2021;118: 450–452.	No	Evaluated cardiovascular effects of e-cigarette use	Full text screening
146	Grech AK, Keating DT, Garner DJ, Naughton MT. A case of extreme carboxyhaemoglominemia due to vaping. Respirol Case Rep. 2022;10. doi:10.1002/rcr2.942	No	Evaluated cardiovascular effects of e-cigarette use	Full text screening
147	Le HHT, Liu C wei, Denaro P, Jousma J, Shao NY, Rahman I, et al. Genome-wide differential expression profiling of lncRNAs and mRNAs in human induced pluripotent stem cell-derived endothelial cells exposed to e-cigarette extract. Stem Cell Res Ther. 2021;12. doi:10.1186/s13287-021-02654-6	No	Evaluated cardiovascular effects of e-cigarette use	Full text screening
148	Mulorz J, Spin JM, Mulorz P, Wagenhäuser MU, Deng A, Mattern K, et al. E-cigarette exposure augments murine abdominal aortic aneurysm development: role of Chil1. Cardiovasc Res. 2022;00: 1–12. doi:10.1093/cvr/cvac173	No	Evaluated cardiovascular effects of e-cigarette use	Full text screening
149	Caruso M, Emma R, Distefano A, Rust S, Poulas K, Giordano A, et al. Comparative assessment of electronic nicotine delivery systems aerosol and cigarette smoke on endothelial cell migration: The Replica Project. Drug Test Anal. 2022;15: 1164–1174. doi:10.1002/dta.3349	No	Evaluated cardiovascular effects of e-cigarette use	Full text screening
150	Michon M, Mercier C, Petit C, Leclerc L, Bertoletti L, Pourchez J, et al. In Vitro Biological Effects of E-Cigarette on the Cardiovascular System—Pro-Inflammatory Response Enhanced by the Presence of the Cinnamon Flavor. Toxics. 2022;10. doi:10.3390/toxics10120784	No	Evaluated cardiovascular effects of e-cigarette use	Full text screening
151	Liu C-W, Huong H, Le T, Denaro P, Dai Z, Shao N-Y, et al. E-cigarettes induce dysregulation of autophagy leading to endothelial dysfunction in pulmonary arterial hypertension. Stem Cells. 2023;41: 328–340. doi:10.1093/stmcls/sxad004/6987575	No	Evaluated cardiovascular effects of e-cigarette use	Full text screening
152	Mills A, Dakhlallah D, Robinson M, Kirk A, Llavina S, Boyd JW, et al. Short-term effects of electronic cigarettes on cerebrovascular function: A time course study. Exp Physiol. 2022;107: 994–1006. doi:10.1113/EP090341	No	Evaluated cardiovascular effects of e-cigarette use	Full text screening
153	El-Mahdy MA, Ewees MG, Eid MS, Mahgoup EM, Khaleel SA, Zweier JL. Electronic cigarette exposure causes vascular endothelial dysfunction due to NADPH oxidase activation and eNOS uncoupling. Am J Physiol - Heart Circ Physiol. 2022;322: H549–H567. doi:10.1152/ajpheart.00460.2021	No	Evaluated cardiovascular effects of e-cigarette use	Full text screening
154	Sifat AE, Archie SR, Nozohouri S, Villalba H, Zhang Y, Sharma S, et al. Short-term exposure to JUUL electronic cigarettes can worsen ischemic stroke outcome. Fluids Barriers CNS. 2022;19. doi:10.1186/s12987-022-00371-7	No	Evaluated cardiovascular effects of e-cigarette use	Full text screening

155	Fried ND, Whitehead A, Lazartigues E, Yue X, Gardner JD. Ovarian hormones do not mediate protection against pulmonary hypertension and right ventricular remodeling in female mice exposed to chronic, inhaled nicotine. Am J Physiol - Heart Circ Physiol. 2022;323: H941–H948. doi:10.1152/ajpheart.00467.2022	No	Evaluated cardiovascular effects of e-cigarette use	Full text screening
156	Carll AP, Arab C, Salatini R, Miles MD, Nystoriak MA, Fulghum KL, et al. E-cigarettes and their lone constituents induce cardiac arrhythmia and conduction defects in mice. Nat Commun. 2022;13. doi:10.1038/s41467-022-33203-1	No	Evaluated cardiovascular effects of e-cigarette use	Full text screening
157	Fried ND, Oakes JM, Whitehead AK, Lazartigues E, Yue X, Gardner JD. Nicotine and novel tobacco products drive adverse cardiac remodeling and dysfunction in preclinical studies. Front Cardiovasc Med. 2022;9: 993617. doi:10.3389/fcvm.2022.993617	No	Evaluated cardiovascular effects of e-cigarette use	Full text screening
158	Rao P, Han DD, Tan K, Mohammadi L, Derakhshandeh R, Navabzadeh M, et al. Comparable Impairment of Vascular Endothelial Function by a Wide Range of Electronic Nicotine Delivery Devices. Nicotine Tob Res. 2022;24: 1055–1062. doi:10.1093/ntr/ntac019	No	Evaluated cardiovascular effects of e-cigarette use	Full text screening
159	Piechowski JM, Bagatto B. Cardiovascular function during early development is suppressed by cinnamon flavored, nicotine-free, electronic cigarette vapor. Birth Defects Res. 2021;113: 1215–1223. doi:10.1002/bdr2.1951	No	Evaluated cardiovascular effects of e-cigarette use	Full text screening
160	Yu X, Zeng X, Xiao F, Chen R, Sinharoy P, Gross ER. E-cigarette aerosol exacerbates cardiovascular oxidative stress in mice with an inactive aldehyde dehydrogenase 2 enzyme. Redox Biol. 2022;54. doi:10.1016/j.redox.2022.102369	No	Evaluated cardiovascular effects of e-cigarette use	Full text screening
161	Whitehead AK, Fried ND, Li Z, Neelamegam K, Pearson CS, LaPenna KB, et al. Alpha7 nicotinic acetylcholine receptor mediates chronic nicotine inhalation-induced cardiopulmonary dysfunction. Clin Sci. 2022;136: 973–987. doi:10.1042/CS20220083	No	Evaluated cardiovascular effects of e-cigarette use	Full text screening
162	Belkin S, Benthien J, Axt PN, Mohr T, Mortensen K, Weckmann M, et al. Impact of Heated Tobacco Products, E-Cigarettes, and Cigarettes on Inflammation and Endothelial Dysfunction. Int J Mol Sci. 2023;24: 9432. doi:10.3390/ijms24119432	No	Evaluated cardiovascular effects of e-cigarette use	Full text screening
163	Cook S, Hirschtick JL, Barnes G, Arenberg D, Bondarenko I, Patel A, et al. Time-varying association between cigarette and ENDS use on incident hypertension among US adults: a prospective longitudinal study. BMJ Open. 2023;13: e062297. doi:10.1136/bmjopen-2022-062297	No	Evaluated cardiovascular effects of e-cigarette use	Full text screening
164	April-Sanders AK, Daviglus ML, Lee UJ, Perreira KM, Kaplan RC, Blaha MJ, et al. Prevalence of electronic cigarette use and its determinants in us persons of Hispanic/Latino background: The Hispanic community health study / study of Latinos (HCHS/SOL). Am J Med Open. 2023;9: 100029. doi:10.1016/j.ajmo.2022.100029	No	Evaluated cardiovascular effects of e-cigarette use	Full text screening
165	Halstead KM, Wetzel EM, Cho JL, Stanhewicz AE. Sex Differences in Oxidative Stress–Mediated Reductions in Microvascular Endothelial Function in Young Adult e-Cigarette Users. Hypertension. 2023;80: 2641–2649. doi:10.1161/HYPERTENSIONAHA.123.21684	No	Evaluated cardiovascular effects of e-cigarette use	Full text screening
166	Boakye E, Uddin SMI, Osuji N, Meinert J, Obisesan OH, Mirbolouk M, et al. Examining the association of habitual e-cigarette use withinflammation and endothelial dysfunction in young adults:The VAPORS-Endothelial function study. Tob Induc Dis. 2023;21: 1–11. doi:10.18332/tid/162327	No	Evaluated cardiovascular effects of e-cigarette use	Full text screening
167	Kelesidis T, Sharma M, Sharma E, Ruedisueli I, Tran E, Middlekauff HR. Chronic Electronic Cigarette Use and Atherosclerosis Risk in Young People: A Cross-Sectional Study—Brief Report. Arterioscler Thromb Vasc Biol. 2023;43: 1713–1718. doi:10.1161/ATVBAHA.123.319172	No	Evaluated cardiovascular effects of e-cigarette use	Full text screening
168	Shi J, Xiong L, Guo J, Yang Y. The association between combustible/electronic cigarette use and stroke based on national health and nutrition examination survey. BMC Public Health. 2023;23: 697. doi:10.1186/s12889-023-15371-x	No	Evaluated cardiovascular effects of e-cigarette use	Full text screening
169	Chhor M, Tulpar E, Nguyen T, Cranfield CG, Gorrie CA, Chan YL, et al. E-Cigarette Aerosol Condensate Leads to Impaired Coronary Endothelial Cell Health and Restricted Angiogenesis. Int J Mol Sci. 2023;24: 6378. doi:10.3390/ijms24076378	No	Evaluated cardiovascular effects of e-cigarette use	Full text screening
170	Dai W, Shi J, Siddarth P, Zhao L, Carreno J, Kleinman MT, et al. Effects of Electronic Cigarette Exposure on Myocardial Infarction and No-Reflow, and Cardiac Function in a Rat Model. J Cardiovasc Pharmacol Ther. 2023;28: 107424842311559. doi:10.1177/10742484231155992	No	Evaluated cardiovascular effects of e-cigarette use	Full text screening
171	Whitehead AK, Li Z, LaPenna KB, Abbes N, Sharp TE, Lefer DJ, et al. Cardiovascular dysfunction induced by combined exposure to nicotine inhalation and high-fat diet. Am J Physiol-Heart Circ Physiol. 2024;326: H278–H290. doi:10.1152/ajpheart.00474.2023	No	Evaluated cardiovascular effects of e-cigarette use	Full text screening
172	Wölkart G, Kollau A, Russwurm M, Koesling D, Schrammel A, Mayer B. Varied effects of tobacco smoke and e-cigarette vapor suggest that nicotine does not affect endothelium-dependent relaxation and nitric oxide signaling. Sci Rep. 2023;13: 15833. doi:10.1038/s41598-023-42750-6	No	Evaluated cardiovascular effects of e-cigarette use	Full text screening
173	Han DD, Rao P, Qiu H, Navabzadeh M, Wang X, Goyal N, et al. Impairment of Endothelial Function by Cigarette Smoke and e-Cigarette Aerosol Requires RAGE. Arterioscler Thromb Vasc Biol. 2023;43: 2369–2371. doi:10.1161/ATVBAHA.123.319514	No	Evaluated cardiovascular effects of e-cigarette use	Full text screening
174	Ballenberger M, Vojnic M, Indaram M, Machnicki S, Harshan M, Novoselac AV, et al. A 33-Year-Old Man With Chest Pain. Chest. 2022;161: e43–e49. doi:10.1016/j.chest.2021.08.069	No	Evaluated cancer risk of e-cigarette use	Full text screening
175	Catto JWF, Rogers Z, Downing A, Mason SJ, Jubber I, Bottomley S, et al. Lifestyle Factors in Patients with Bladder Cancer: A Contemporary Picture of Tobacco Smoking, Electronic Cigarette Use, Body Mass Index, and Levels of Physical Activity. Eur Urol Focus. 2023;9: 974–982. doi:10.1016/j.euf.2023.04.003	No	Evaluated cancer risk of e-cigarette use	Full text screening
176	Chen M, Carmella SG, Lindgren BR, Luo X, Ikuemonisan J, Niesen B, et al. Increased Levels of the Acrolein Metabolite 3-Hydroxypropyl Mercapturic Acid in the Urine of e-Cigarette Users. Chem Res Toxicol. 2023;36: 583–588. doi:10.1021/acs.chemrestox.2c00145	No	Evaluated cancer risk of e-cigarette use	Full text screening
177	De Lima JM, Macedo CCS, Barbosa GV, Castellano LRC, Hier MP, Alaoui-Jamali MA, et al. E-liquid alters oral epithelial cell function to promote epithelial to mesenchymal transition and invasiveness in preclinical oral squamous cell carcinoma. Sci Rep. 2023;13: 3330. doi:10.1038/s41598-023-30016-0	No	Evaluated cancer risk of e-cigarette use	Full text screening
178	Dugan K, Breit S, Okut H, Ablah E. Electronic Cigarette Use and the Diagnosis of Nonmelanoma Skin Cancer Among United States Adults. Cureus. 13: e19053. doi:10.7759/cureus.19053	No	Evaluated cancer risk of e-cigarette use	Full text screening
179	Guo J, Ikuemonisan J, Hatsukami DK, Hecht SS. Liquid Chromatography-Nanoelectrospray Ionization-High-Resolution Tandem Mass Spectrometry Analysis of Apurinic/Apyrimidinic Sites in Oral Cell DNA of Cigarette Smokers, e-Cigarette Users, and Nonsmokers. Chem Res Toxicol. 2021;34: 2540–2548. doi:10.1021/acs.chemrestox.1c00308	No	Evaluated cancer risk of e-cigarette use	Full text screening
180	Jeon J, Zhang Q, Chepaitis PS, Greenwald R, Black M, Wright C. Toxicological Assessment of Particulate and Metal Hazards Associated with Vaping Frequency and Device Age. Toxics. 2023;11: 155. doi:10.3390/toxics11020155	No	Evaluated cancer risk of e-cigarette use	Full text screening
181	Kamal NM, Shams NS. The impact of tobacco smoking and electronic cigarette vaping on salivary biomarkers. A comparative study. Saudi Dent J. 2022;34: 404–409. doi:10.1016/j.sdentj.2022.05.003	No	Evaluated cancer risk of e-cigarette use	Full text screening
182	Kim J, Keegan TH. Characterizing risky alcohol use, cigarette smoking, e-cigarette use, and physical inactivity among cancer survivors in the USA—a cross-sectional study. J Cancer Surviv. 2023;17: 1799–1812. doi:10.1007/s11764-022-01245-5	No	Evaluated cancer risk of e-cigarette use	Full text screening
183	Kwon HJ, Oh YT, Park S, Kim SS, Park J, Yin J, et al. Analysis of electric cigarette liquid effect on mouse brain tumor growth through EGFR and ERK activation. Di Paola R, editor. PLOS ONE. 2021;16: e0256730. doi:10.1371/journal.pone.0256730	No	Evaluated cancer risk of e-cigarette use	Full text screening

184	Molony RD, Wu C-H, Lee Y-F. E-liquid exposure induces bladder cancer cells to release extracellular vesicles that promote non-malignant urothelial cell transformation. Sci Rep. 2023;13: 142. doi:10.1038/s41598-022-27165-z	No	Evaluated cancer risk of e-cigarette use	Full text screening
185	Platel A, Dusautoir R, Kervoaze G, Dourdin G, Gateau E, Talahari S, et al. Comparison of the in vivo genotoxicity of electronic and conventional cigarettes aerosols after subacute, subchronic and chronic exposures. J Hazard Mater. 2022;423: 127246. doi:10.1016/j.jhazmat.2021.127246	No	Evaluated cancer risk of e-cigarette use	Full text screening
186	Richmond RC, Sillero-Rejon C, Khouja JN, Prince C, Board A, Sharp G, et al. Investigating the DNA methylation profile of e-cigarette use. Clin Epigenetics. 2021;13: 183. doi:10.1186/s13148-021-01174-7	No	Evaluated cancer risk of e-cigarette use	Full text screening
187	Robin HP, Trudeau CN, Robbins AJ, Chung EJ, Rahman E, Strickland OLG, et al. Inflammation and Invasion in Oral Squamous Cell Carcinoma Cells Exposed to Electronic Cigarette Vapor Extract. Front Oncol. 2022;12: 917862. doi:10.3389/fonc.2022.917862	No	Evaluated cancer risk of e-cigarette use	Full text screening
188	Song M-A, Mori KM, McElroy JP, Freudenheim JL, Weng DY, Reisinger SA, et al. Accelerated epigenetic age, inflammation, and gene expression in lung: comparisons of smokers and vapers with non-smokers. Clin Epigenetics. 2023;15: 160. doi:10.1186/s13148-023-01577-8	No	Evaluated cancer risk of e-cigarette use	Full text screening
189	Sun Q, Chen D, Raja A, Grunig G, Zelikoff J, Jin C. Downregulation of Stem-Loop Binding Protein by Nicotine via α7-Nicotinic Acetylcholine Receptor and Its Role in Nicotine-Induced Cell Transformation. Toxicol Sci. 2022;189: 186–202. doi:10.1093/toxsci/kfac080	No	Evaluated cancer risk of e-cigarette use	Full text screening
190	Tellez CS, Grimes MJ, Juri DE, Do K, Willink R, Dye WW, et al. Flavored E-cigarette product aerosols induce transformation of human bronchial epithelial cells. Lung Cancer. 2023;179: 107180. doi:10.1016/j.lungcan.2023.107180	No	Evaluated cancer risk of e-cigarette use	Full text screening
191	Trifunovic S, Smiljanić K, Sickmann A, Solari FA, Kolarevic S, Divac Rankov A, et al. Electronic cigarette liquids impair metabolic cooperation and alter proteomic profiles in V79 cells. Respir Res. 2022;23: 191. doi:10.1186/s12931-022-02102-w	No	Evaluated cancer risk of e-cigarette use	Full text screening
192	Tsai JC, Saad OA, Magesh S, Xu J, Lee AC, Li WT, et al. Tobacco Smoke and Electronic Cigarette Vapor Alter Enhancer RNA Expression That Can Regulate the Pathogenesis of Lung Squamous Cell Carcinoma. Cancers. 2021;13: 4225. doi:10.3390/cancers13164225	No	Evaluated cancer risk of e-cigarette use	Full text screening
193	Wang W, Zeng R, Liu M, Chen M, Wei S, Li B, et al. Exosome proteomics study of the effects of traditional cigarettes and electronic cigarettes on human bronchial epithelial cells. Toxicol In Vitro. 2022;86: 105516. doi:10.1016/j.tiv.2022.105516	No	Evaluated cancer risk of e-cigarette use	Full text screening
194	Wharram CE, Kyko JM, Ruterbusch JJ, Beebe-Dimmer JL, Schwartz AG, Cote ML. Use of electronic cigarettes among African American cancer survivors. Cancer. 2023;129: 3334–3345. doi:10.1002/cncr.34933	No	Evaluated cancer risk of e-cigarette use	Full text screening
195	Zarcone G, Lenski M, Martinez T, Talahari S, Simonin O, Garçon G, et al. Impact of Electronic Cigarettes, Heated Tobacco Products and Conventional Cigarettes on the Generation of Oxidative Stress and Genetic and Epigenetic Lesions in Human Bronchial Epithelial BEAS-2B Cells. Toxics. 2023;11: 847. doi:10.3390/toxics11100847	No	Evaluated cancer risk of e-cigarette use	Full text screening
196	Rayner RE, Makena P, Liu G, Prasad GL, Cormet-Boyaka E. Differential gene expression of 3D primary human airway cultures exposed to cigarette smoke and electronic nicotine delivery system (ENDS) preparations. BMC Med Genomics. 2022;15: 76. doi:10.1186/s12920-022-01215-x	No	Evaluated cancer risk of e-cigarette use	Full text screening
197	Rayner RE, Wellmerling J, Makena P, Zhao J, Prasad GL, Cormet-Boyaka E. Transcriptomic Response of Primary Human Bronchial Cells to Repeated Exposures of Cigarette and ENDS Preparations. Cell Biochem Biophys. 2022;80: 217–228. doi:10.1007/s12013-021-01042-4	No	Evaluated cancer risk of e-cigarette use	Full text screening
198	Park H-R, Vallarino J, O'Sullivan M, Wirth C, Panganiban RA, Webb G, et al. Electronic cigarette smoke reduces ribosomal protein gene expression to impair protein synthesis in primary human airway epithelial cells. Sci Rep. 2021;11: 17517. doi:10.1038/s41598-021-97013-z	No	Evaluated cancer risk of e-cigarette use	Full text screening
199	Noël A, Yilmaz S, Farrow T, Schexnayder M, Eickelberg O, Jelesijevic T. Sex-Specific Alterations of the Lung Transcriptome at Birth in Mouse Offspring Prenatally Exposed to Vanilla-Flavored E-Cigarette Aerosols and Enhanced Susceptibility to Asthma. Int J Environ Res Public Health. 2023;20: 3710. doi:10.3390/ijerph20043710	No	Evaluated cancer risk of e-cigarette use	Full text screening
200	Caruso M, Emma R, Distefano A, Rust S, Poulas K, Giordano A, et al. Comparative assessment of electronic nicotine delivery systems aerosol and cigarette smoke on endothelial cell migration: The Replica Project. Drug Test Anal. 2022;15: 1164–1174. doi:10.1002/dta.3349	No	Evaluated cancer risk of e-cigarette use	Full text screening
201	Yogeswaran S, Shaikh SBi, Manevski M, Chand HS, Rahman I. The role of synthetic coolants, WS-3 and WS-23, in modulating E-cigarette-induced reactive oxygen species (ROS) in lung epithelial cells. Toxicol Rep. 2022;9: 1700–1709. doi:10.1016/j.toxrep.2022.08.007	No	Evaluated cancer risk of e-cigarette use	Full text screening
202	Hammond D, Reid JL, Rynard VL, et al. Indicators of dependence and efforts to quit vaping and smoking among youth in Canada, England and the USA. *Tob Control*. Published online February 1, 2021. doi:10.1136/tobaccocontrol-2020-056269	No	Evaluated e-cigarette dependence	Full text screening
203	Kaplan B, Alrumaih F, Breland A, Eissenberg T, Cohen JE. A comparison of product dependence among cigarette only, ENDS only, and dual users: Findings from Wave 3 (2015–2016) of the PATH study. *Drug Alcohol Depend*. 2020;217. doi:10.1016/j.drugalcdep.2020.108347	No	Evaluated e-cigarette dependence	Full text screening
204	Jankowski M, Krzystanek M, Zejda JE, et al. E-cigarettes are more addictive than traditional cigarettes—A study in highly educated young people. *Int J Environ Res Public Health*. 2019;16(13). doi:10.3390/ijerph16132279	No	Evaluated e-cigarette dependence	Full text screening
205	Huh Y, Lee CM, Cho HJ. Comparison of nicotine dependence between single and multiple tobacco product users among South Korean adults. *Tob Induc Dis*. 2022;20(February). doi:10.18332/tid/145899	No	Evaluated e-cigarette dependence	Full text screening
206	Liu G, Wasserman E, Kong L, Foulds J. A comparison of nicotine dependence among exclusive E-cigarette and cigarette users in the PATH study. *Prev Med*. 2017;104:86-91.	No	Evaluated e-cigarette dependence	Full text screening
207	Vogel EA, Ramo DE, Rubinstein ML. Prevalence and correlates of adolescents' e-cigarette use frequency and dependence. *Drug Alcohol Depend*. 2018;188:109-112.	No	Evaluated e-cigarette dependence	Full text screening
208	Palmer AM, Sawyer LE, Brandon TH. Distinct influences of nicotine and sensorimotor stimuli on reducing cravings to smoke and vape among dual users. *Addict Behav*. 2021;122. doi:10.1016/j.addbeh.2021.107051	No	Evaluated e-cigarette dependence	Full text screening
209	Yingst J, Wang X, Lopez AA, et al. Changes in Nicotine Dependence Among Smokers Using Electronic Cigarettes to Reduce Cigarette Smoking in a Randomized Controlled Trial. *Nicotine Tob Res*. 2022;25(3):372-378.	No	Evaluated e-cigarette dependence	Full text screening
210	St Helen G, Nardone N, Addo N, et al. Differences in nicotine intake and effects from electronic and combustible cigarettes among dual users. *Addiction*. 2020;115(4):757-767.	No	Evaluated e-cigarette dependence	Full text screening
211	Harvanko AM, St Helen G, Nardone N, Addo N, Benowitz NL. Twenty-four-hour subjective and pharmacological effects of ad-libitum electronic and combustible cigarette use among dual users. *Addiction*. 2020;115(6):1149-1159.	No	Evaluated e-cigarette dependence	Full text screening
212	Rüther T, Hagedorn D, Schiela K, Schettgen T, Osiander-Fuchs H, Schober W. Nicotine delivery efficiency of first- and second-generation e-cigarettes and its impact on relief of craving during the acute phase of use. *Int J Hyg Environ Health*. 2018;221(2):191-198.	No	Evaluated e-cigarette dependence	Full text screening
213	Hughes JR, Peters EN, Callas PW, et al. Withdrawal Symptoms from E-Cigarette Abstinence among Former Smokers: A Pre-Post Clinical Trial. *Nicotine Tob Res*. 2020;22(5):734-739.	No	Evaluated e-cigarette dependence	Full text screening
214	Yingst JM, Foulds J, Veldheer S, et al. Nicotine absorption during electronic cigarette use among regular users. *PLoS One*. 2019;14(7). doi:10.1371/journal.pone.0220300	No	Evaluated e-cigarette dependence	Full text screening
215	Rose JE, Behm FM, Willette PN, Botts TL, Botts DR. Using varenicline in combination with electronic nicotine delivery systems (ENDS). *Drug Alcohol Depend*. 2023;251:110916.	No	Evaluated e-cigarette dependence	Full text screening

216	Loukas A, Marti CN, Harrell MB, Pasch KE, Wilkinson AV. Electronic nicotine delivery systems (ENDS) dependence among Texas Young Adults, 2014-2019: Increasing trajectory after the 2017 surge in vape pod popularity. *Drug Alcohol Depend*. 2022;241. doi:10.1016/j.drugalcdep.2022.109700	No	Evaluated e-cigarette dependence	Full text screening
217	Creamer ML, Case K, Loukas A, Cooper M, Perry CL. Patterns of sustained e-cigarette use in a sample of young adults. *Addict Behav*. 2019;92:28-31.	No	Evaluated e-cigarette dependence	Full text screening
218	Strong DR, Leas E, Noble M, et al. Predictive validity of the adult tobacco dependence index: Findings from waves 1 and 2 of the Population Assessment of Tobacco and Health (PATH) study. *Drug Alcohol Depend*. 2020;214. doi:10.1016/j.drugalcdep.2020.108134	No	Evaluated e-cigarette dependence	Full text screening
219	Du P, Fan T, Yingst J, et al. Changes in E-Cigarette Use Behaviors and Dependence in Long-term E-Cigarette Users. *Am J Prev Med*. 2019;57(3):374-383.	No	Evaluated e-cigarette dependence	Full text screening
220	Snell LM, Barnes AJ, Nicksic &nicole E. *ALongitudinal Analysis of Nicotine Dependence and Transitions From Dual Use of Cigarettes and Electronic Cigarettes: Evidence From Waves1-3 of the PATH Study*.; 2020.	No	Evaluated e-cigarette dependence	Full text screening
221	Vogel EA, Prochaska JJ, Ramo DE, Andres J, Rubinstein ML. Adolescents' E-Cigarette Use: Increases in Frequency, Dependence, and Nicotine Exposure Over 12 Months. *J Adolesc Health Care*. 2019;64(6):770-775.	No	Evaluated e-cigarette dependence	Full text screening
222	Vogel EA, Cho J, McConnell RS, Barrington-Trimis JL, Leventhal AM. Prevalence of Electronic Cigarette Dependence among Youth and Its Association with Future Use. *JAMA Network Open*. 2020;3(2). doi:10.1001/jamanetworkopen.2019.21513	No	Evaluated e-cigarette dependence	Full text screening
223	Rest EC, Mermelstein RJ, Hedeker D. Nicotine dependence in dual users of cigarettes and E-Cigarettes: Common and distinct elements. *Nicotine Tob Res*. 2021;23(4):662-668.	No	Evaluated e-cigarette dependence	Full text screening
224	Etter JF. An 8-year longitudinal study of long-term, continuous users of electronic cigarettes. *Addict Behav*. 2024;149:107891.	No	Evaluated e-cigarette dependence	Full text screening
225	Boyd CJ, McCabe SE, Evans-Polce RJ, Voepel-Lewis T, Shuman C, Veliz P. Adolescents' Nicotine/Tobacco Dependency Symptoms Using 4 Waves of PATH Data. *Health Behav Policy Rev*. 2022;9(4):980-995.	No	Evaluated e-cigarette dependence	Full text screening
226	Do EK, Aarvig K, Donovan EM, Barrington-Trimis JL, Vallone DM, Hair EC. E-cigarette Device Type, Source, and Use Behaviors of Youth and Young Adults: Findings from the Truth Longitudinal Cohort (2020–2021). *Subst Use Misuse*. 2023;58(6):796-803.	No	Evaluated e-cigarette dependence	Full text screening
227	Shiffman S, Goldenson NI. Changes in dependence over one year among US adults who smoke cigarettes and switched completely or partially to use of the JUUL-brand electronic nicotine delivery system. *Drug Alcohol Depend Rep*. 2023;6:100137.	No	Evaluated e-cigarette dependence	Full text screening
228	Pienkowski M, Chaiton M, Bondy SJ, et al. Milestones in the natural course of the onset of e-cigarette dependence among adolescents and young adults: Retrospective study. *Addict Behav*. 2024;148:107846.	No	Evaluated e-cigarette dependence	Full text screening
229	Leventhal A, Dai H, Barrington-Trimis J, Sussman S. Ice' flavoured e-cigarette use among young adults. *Tob Control*. 2021;32(1):114-117.	No	Evaluated e-cigarette dependence	Full text screening
230	Hammond D, Reid JL, Burkhalter R, et al. Trends in e-cigarette brands, devices and the nicotine profile of products used by youth in England, Canada and the USA: 2017-2019. *Tob Control*. 2023;32(1):19-29.	No	Evaluated e-cigarette dependence	Full text screening
231	Tackett AP, Hébert ET, Smith CE, et al. Youth use of e-cigarettes: Does dependence vary by device type? *Addict Behav*. 2021;119:106918.	No	Evaluated e-cigarette dependence	Full text screening
232	Sargent JD, Stoolmiller M, Dai H, et al. First E-Cigarette Flavor and Device Type Used: Associations with Vaping Persistence, Frequency, and Dependence in Young Adults. *Nicotine Tob Res*. 2022;24(3):380-387.	No	Evaluated e-cigarette dependence	Full text screening
233	Parks MJ, Fleischer NL, Patrick ME. Increased nicotine vaping due to the COVID-19 pandemic among US young adults: Associations with nicotine dependence, vaping frequency, and reasons for use. *Prev Med* . 2022;159. doi:10.1016/j.ypmed.2022.107059	No	Evaluated e-cigarette dependence	Full text screening
234	Do EK, O'Connor K, Perks SN, et al. E-cigarette device and liquid characteristics and E-cigarette dependence: A pilot study of pod-based and disposable E-cigarette users. *Addict Behav*. 2022;124. doi:10.1016/j.addbeh.2021.107117	No	Evaluated e-cigarette dependence	Full text screening
235	Douglas AE, Childers MG, Romm KF, Felicione NJ, Ozga JE, Blank MD. Device features and user behaviors as predictors of dependence among never-smoking electronic cigarette users: PATH Wave 4. *Addict Behav*. 2021;125. doi:10.1016/j.addbeh.2021.107161	No	Evaluated e-cigarette dependence	Full text screening
236	Hoyt DL, Kinsler BE, Otto MW, Garey L, Mayorga NA, Zvolensky MJ. An investigation of racial and ethnic differences in e-cigarette beliefs and use characteristics. *J Ethn Subst Abuse*. 2022;21(2):387-401.	No	Evaluated e-cigarette dependence	Full text screening
237	Jackson SE, Brown J, Jarvis MJ. Dependence on nicotine in US high school students in the context of changing patterns of tobacco product use. *Addiction*. 2021;116(7):1859-1870.	No	Evaluated e-cigarette dependence	Full text screening
238	Winburn C, Ofei-Dodoo S. Nicotine Dependence from Electronic Cigarettes Use and Depressive Symptoms Among Adolescents. *Kans Med*. 2023;16(1):1-4.	No	Evaluated e-cigarette dependence	Full text screening
239	Choi HJ, Yu M, Sacco P. Racial and ethnic differences in patterns of adolescent tobacco users: A latent class analysis. *Child Youth Serv Rev*. 2018;84:86-93.	No	Evaluated e-cigarette dependence	Full text screening
240	Zvolensky MJ, Mayorga NA, Garey L. Main and Interactive Effects of e-Cigarette Use Health Literacy and Anxiety Sensitivity in Terms of e-Cigarette Perceptions and Dependence. *Cognit Ther Res*. 2019;43(1):121-130.	No	Evaluated e-cigarette dependence	Full text screening
241	Borland R, Murray K, Gravely S, et al. A new classification system for describing concurrent use of nicotine vaping products alongside cigarettes (so-called 'dual use'): findings from the ITC-4 Country Smoking and Vaping wave 1 Survey. *Addiction*. 2019;114(S1):24-34.	No	Evaluated e-cigarette dependence	Full text screening
242	Wade NE, Courtney KE, Doran N, et al. Young Adult E-Cigarette and Combustible Tobacco Users Attitudes, Substance Use Behaviors, Mental Health, and Neurocognitive Performance. *Brain Sciences*. 2022;12(7). doi:10.3390/brainsci12070889	No	Evaluated e-cigarette dependence	Full text screening
243	Sung HY, Wang Y, Yao T, Lightwood J, Max W. Polytobacco use and nicotine dependence symptoms among US adults, 2012-2014. *Nicotine Tob Res*. 2018;20:S88-S98.	No	Evaluated e-cigarette dependence	Full text screening
244	Camara-Medeiros A, Diemert L, O'Connor S, Schwartz R, Eissenberg T, Cohen JE. Perceived addiction to vaping among youth and young adult regular vapers. *Tob Control*. 2021;30(3):273-278.	No	Evaluated e-cigarette dependence	Full text screening
245	Kim JY, Kang HS, Jung JW, et al. Nicotine dependence and stress susceptibility in E-cigarette smokers: The Korea national health and nutrition examination survey 2013–2017. *Tuberc Respir Dis* . 2021;84(2):159-166.	No	Evaluated e-cigarette dependence	Full text screening
246	Pamungkasningsih SW, Taufik FF, Samoedro E, Andarini S, Susanto AD. Urinary Cotinine and Nicotine Dependence Levels in Regular Male Electronic Cigarette Users. *Eurasian Journal of Medicine*. 2021;53(3):168-173.	No	Evaluated e-cigarette dependence	Full text screening
247	Temourian AA, Song AV, Halliday DM, Gonzalez M, Epperson AE. Why do smokers use e-cigarettes? A study on reasons among dual users. *Preventive Medicine Reports*. 2022;29. doi:10.1016/j.pmedr.2022.101924	No	Evaluated e-cigarette dependence	Full text screening
248	Douglas AE, Felicione NJ, Childers MG, Soule EK, Blank MD. Predictors of electronic cigarette dependence among non-smoking electronic cigarette users: User behavior and device characteristics. *Addict Behav*. 2023;137. doi:10.1016/j.addbeh.2022.107500	No	Evaluated e-cigarette dependence	Full text screening
249	Fearon IM, Seltzer RGN, Houser TL, et al. Examination of the impact of myblu electronic nicotine delivery system e-liquid nicotine strength on self-reported measures of dependence. *Drug Test Anal*. Published online 2022. doi:10.1002/dta.3335	No	Evaluated e-cigarette dependence	Full text screening

250	Mohd Radzi NA, Saub R, Yusof ZYM, Dahlui M, Sujak SL. Nicotine dependence among adolescents single and dual cigarette users. *Children*. 2021;8(2). doi:10.3390/children8020144	No	Evaluated e-cigarette dependence	Full text screening
251	Carroll DM, Wagener TL, Thompson DM, et al. Electronic nicotine delivery system use behaviour and loss of autonomy among American Indians: Results from an observational study. *BMJ Open*. 2017;7(12). doi:10.1136/bmjopen-2017-018469	No	Evaluated e-cigarette dependence	Full text screening
252	Chaffee BW, Halpern-Felsher B, Jacob P, St. Helen G. Biomarkers of nicotine exposure correlate with the Hooked on Nicotine Checklist among adolescents in California, United States. *Addict Behav*. 2022;128. doi:10.1016/j.addbeh.2022.107235	No	Evaluated e-cigarette dependence	Full text screening
253	Perry RN, Girgulis JP, Harrison SL, Barrett SP. Dual users' perceptions of the addictive properties of cigarettes versus E-cigarettes. *Addict Behav*. 2023;136. doi:10.1016/j.addbeh.2022.107483	No	Evaluated e-cigarette dependence	Full text screening
254	Chavez JF, Smit T, Olofsson H, Mayorga NA, Garey L, Zvolensky MJ. Substance Use among Exclusive Electronic Cigarette Users and Dual Combustible Cigarette Users: Extending Work to Adult Users. *Subst Use Misuse*. 2021;56(6):888-896.	No	Evaluated e-cigarette dependence	Full text screening
255	Vogel EA, Prochaska JJ, Rubinstein ML. Measuring e-cigarette addiction among adolescents. *Tob Control*. 2020;29(3):258-262.	No	Evaluated e-cigarette dependence	Full text screening
256	Saran SK, Salinas KZ, Foulds J, et al. A Comparison of Vaping Behavior, Perceptions, and Dependence among Individuals Who Vape Nicotine, Cannabis, or Both. *Int J Environ Res Public Health*. 2022;19(16). doi:10.3390/ijerph191610392	No	Evaluated e-cigarette dependence	Full text screening
257	Hughes JR, Callas PW. Prevalence of withdrawal symptoms from electronic cigarette cessation: A cross-sectional analysis of the US Population Assessment of Tobacco and Health. *Addict Behav*. 2019;91:234-237.	No	Evaluated e-cigarette dependence	Full text screening
258	Leventhal AM, Dai H, Barrington-Trimis JL, Tackett AP, Pedersen ER, Tran DD. Disposable E-Cigarette Use Prevalence, Correlates, and Associations with Previous Tobacco Product Use in Young Adults. *Nicotine Tob Res*. 2022;24(3):372-379.	No	Evaluated e-cigarette dependence	Full text screening
259	Strong DR, Pearson J, Ehlke S, et al. Indicators of dependence for different types of tobacco product users: Descriptive findings from Wave 1 (2013–2014) of the Population Assessment of Tobacco and Health (PATH) study. *Drug Alcohol Depend*. 2017;178:257-266.	No	Evaluated e-cigarette dependence	Full text screening
260	Odani S, Armour B, Agaku IT. Flavored Tobacco Product Use and Its Association with Indicators of Tobacco Dependence among US Adults, 2014-2015. *Nicotine Tob Res*. 2020;22(6):1004-1015.	No	Evaluated e-cigarette dependence	Full text screening
261	Morean ME, Krishnan-Sarin S, S. O'Malley S. Assessing nicotine dependence in adolescent E-cigarette users: The 4-item Patient-Reported Outcomes Measurement Information System (PROMIS) Nicotine Dependence Item Bank for electronic cigarettes. *Drug Alcohol Depend*. 2018;188:60-63.	No	Evaluated e-cigarette dependence	Full text screening
262	Piper ME, Baker TB, Mermelstein R, Benowitz N, Jorenby DE. Relations among cigarette dependence, e-cigarette dependence, and key dependence criteria among dual users of combustible and e-cigarettes. *Psychol Addict Behav*. 2022;36(1):100-108.	No	Evaluated e-cigarette dependence	Full text screening
263	Glantz S, Jeffers A, Winickoff JP. Nicotine Addiction and Intensity of e-Cigarette Use by Adolescents in the US, 2014 to 2021. *JAMA network open*. 2022;5(11):e2240671-e2240671.	No	Evaluated e-cigarette dependence	Full text screening
264	Roh EJ, Chen-Sankey JC, Wang MQ. Electronic nicotine delivery system (ENDS) use patterns and its associations with cigarette smoking and nicotine addiction among Asian Americans: Findings from the national adult tobacco survey (NATS) 2013-2014. *J Ethn Subst Abuse*. 2022;21(1):253-271.	No	Evaluated e-cigarette dependence	Full text screening
265	Morean M, Krishnan-Sarin S, O'Malley SS. Comparing cigarette and e-cigarette dependence and predicting frequency of smoking and e-cigarette use in dual-users of cigarettes and e-cigarettes. *Addict Behav*. 2018;87:92-96.	No	Evaluated e-cigarette dependence	Full text screening
266	Rhoades DA, Comiford AL, Dvorak JD, et al. Vaping patterns, nicotine dependence and reasons for vaping among American Indian dual users of cigarettes and electronic cigarettes. *BMC Public Health*. 2019;19(1). doi:10.1186/s12889-019-7523-5	No	Evaluated e-cigarette dependence	Full text screening
267	Case KR, Mantey DS, Creamer MR, Harrell MB, Kelder SH, Perry CL. E-cigarette- specific symptoms of nicotine dependence among Texas adolescents. *Addict Behav*. 2018;84:57-61.	No	Evaluated e-cigarette dependence	Full text screening
268	Do EK, O'Connor K, Kreslake JM, Friedrichsen SC, Vallone DM, Hair EC. Influence of Flavors and Nicotine Concentration on Nicotine Dependence in Adolescent and Young Adult E-Cigarette Users. *Subst Use Misuse*. 2022;57(4):632-639.	No	Evaluated e-cigarette dependence	Full text screening
269	Lin C, Gaiha SM, Halpern-Felsher B. Nicotine Dependence from Different E-Cigarette Devices and Combustible Cigarettes among US Adolescent and Young Adult Users. *Int J Environ Res Public Health*. 2022;19(10). doi:10.3390/ijerph19105846	No	Evaluated e-cigarette dependence	Full text screening
270	Case KR, Hinds JT, Creamer MLR, Loukas A, Perry CL. Who is JUULing and Why? An Examination of Young Adult Electronic Nicotine Delivery Systems Users. *J Adolesc Health Care*. 2020;66(1):48-55.	No	Evaluated e-cigarette dependence	Full text screening
271	Boykan R, Goniewicz ML, Messina CR. Evidence of Nicotine Dependence in Adolescents Who Use Juul and Similar Pod Devices. *Int J Environ Res Public Health*. 2019;16(12). doi:10.3390/ijerph16122135	No	Evaluated e-cigarette dependence	Full text screening
272	Harvanko AM, McCubbin AK, Ashford KB, Kelly TH. Electronic cigarette liquid and device parameters and aerosol characteristics: A survey of regular users. *Addict Behav*. 2018;84:201-206.	No	Evaluated e-cigarette dependence	Full text screening
273	Leavens ELS, Smith TT, Natale N, Carpenter MJ. Electronic cigarette dependence and demand among pod mod users as a function of smoking status. *Psychol Addict Behav*. 2020;34(7):804-810.	No	Evaluated e-cigarette dependence	Full text screening
274	Shiffman S, Sembower MA. Dependence on e-cigarettes and cigarettes in a cross-sectional study of US adults. *Addiction*. 2020;115(10):1924-1931.	No	Evaluated e-cigarette dependence	Full text screening
275	Azagba S, Shan L, Latham K. Adolescent Dual Use Classification and Its Association With Nicotine Dependence and Quit Intentions. *J Adolesc Health Care*. 2019;65(2):195-201.	No	Evaluated e-cigarette dependence	Full text screening
276	Johnson JM, Muilenburg JL, Rathbun SL, Yu X, Naeher LP, Wang JS. Elevated Nicotine Dependence Scores among Electronic Cigarette Users at an Electronic Cigarette Convention. *J Community Health*. 2018;43(1):164-174.	No	Evaluated e-cigarette dependence	Full text screening
277	Rycroft N, Hogarth L, MacKillop J, Dawkins L. Vapers exhibit similar subjective nicotine dependence but lower nicotine reinforcing value compared to smokers. *Addict Behav*. 2021;115. doi:10.1016/j.addbeh.2020.106737	No	Evaluated e-cigarette dependence	Full text screening
278	Rudasingwa G, Kim Y, Lee C, Lee J, Kim S, Kim S. Comparison of nicotine dependence and biomarker levels among traditional cigarette, heat-not-burn cigarette, and liquid e-cigarette users: Results from the think study. *Int J Environ Res Public Health*. 2021;18(9). doi:10.3390/ijerph18094777	No	Evaluated e-cigarette dependence	Full text screening
279	Browne M, Todd DG. Then and now: Consumption and dependence in e-cigarette users who formerly smoked cigarettes. *Addict Behav*. 2018;76:113-121.	No	Evaluated e-cigarette dependence	Full text screening
280	Mantey DS, Case KR, Omega-Njemnobi O, Springer AE, Kelder SH. Use frequency and symptoms of nicotine dependence among adolescent E-cigarette users: Comparison of JUUL and Non-JUUL users. *Drug Alcohol Depend*. 2021;228. doi:10.1016/j.drugalcdep.2021.109078	No	Evaluated e-cigarette dependence	Full text screening
281	Zaidi AB, Shaikh S. Nicotine dependence and intention to quit among electronic, conventional and dual cigarette users in Karachi. *J Pak Med Assoc*. 2022;72(9):1766-1770.	No	Evaluated e-cigarette dependence	Full text screening
282	Yingst J, Foulds J, Hobkirk AL. Dependence and Use Characteristics of Adult JUUL Electronic Cigarette Users. *Subst Use Misuse*. 2021;56(1):61-66.	No	Evaluated e-cigarette dependence	Full text screening

283	Kechter A, Cho J, Miech RA, Barrington-Trimis JL, Leventhal AM. Nicotine dependence symptoms in U.S. youth who use JUUL E-cigarettes. *Drug Alcohol Depend*. 2021;227:108941.	No	Evaluated e-cigarette dependence	Full text screening
284	Dobbs PD, Hodges EJ, Dunlap CM, Cheney MK. Addiction vs. dependence: A mixed methods analysis of young adult JUUL users. *Addict Behav*. 2020;107:106402.	No	Evaluated e-cigarette dependence	Full text screening
285	Adjei A, Chen B, Mantey DS, Wilkinson AV, Harrell MB. Symptoms of nicotine dependence by e-cigarette and cigarette use behavior and brand: A population-based, nationally representative cross-sectional study. *Drug Alcohol Depend*. 2023;255:111059.	No	Evaluated e-cigarette dependence	Full text screening
286	Cristol BA, Clendennen SL, Hébert ET, Harrell MB. Nicotine dependence among young adults: Comparing exclusive ENDS users to exclusive cigarette smokers. *Addict Behav*. 2024;149:107897.	No	Evaluated e-cigarette dependence	Full text screening
287	Strong DR, Glasser AM, Leas EC, et al. Indicators of Tobacco Dependence Among Youth: Findings From Wave 1 (2013–2014) of the Population Assessment of Tobacco and Health Study. *Nicotine Tob Res*. 2023;25(9):1565-1574.	No	Evaluated e-cigarette dependence	Full text screening
288	Alshaibani M, Alajmi M, Alabduljalil N, et al. Prevalence of use, perceptions of harm and addictiveness, and dependence of electronic cigarettes among adults in Kuwait: A cross-sectional study. *Tob Induc Dis*. 2023;21:90.	No	Evaluated e-cigarette dependence	Full text screening
289	Parms TA, Valverde R, Park-Lee E, et al. Symptoms of tobacco dependence among middle and high school tobacco users - Data from the 2019-2020 National Youth Tobacco Survey. *Addict Behav*. 2023;137:107537.	No	Evaluated e-cigarette dependence	Full text screening
290	Sreeramareddy CT, Shroff SM, Gunjal S. Nicotine dependence and associated factors among persons who use electronic e-cigarettes in Malaysia - an online survey. *Subst Abuse Treat Prev Policy*. 2023;18(1):51.	No	Evaluated e-cigarette dependence	Full text screening
291	Lohner V, McNeill A, Schneider S, et al. Understanding perceived addiction to and addictiveness of electronic cigarettes among electronic cigarette users: a cross-sectional analysis of the International Tobacco Control Smoking and Vaping (ITC 4CV) England Survey. *Addiction*. 2023;118(7):1359-1369.	No	Evaluated e-cigarette dependence	Full text screening
292	Tashakkori NA, Park-Lee E, Roh EJ, Christensen CH. Multiple Tobacco Product Use Among Youth E-Cigarette Users: National Youth Tobacco Survey, 2020. *J Adolesc Health*. 2023;73(4):769-775.	No	Evaluated e-cigarette dependence	Full text screening
293	Shiffman S, Oliveri DR, Goldenson NI, Liang Q, Black RA, Mishra S. Comparing Adult Smokers Who Switched to JUUL versus Continuing Smokers: Biomarkers of Exposure and of Potential Harm and Respiratory Symptoms. *Nicotine Tob Res*. Published online October 14, 2023:ntad197.	No	Evaluated e-cigarette dependence	Full text screening
294	Bagdas D, Harris L, Addy NA. Chronic oral nicotine exposure decreases aversive taste of nicotine, increases nicotine withdrawal and reinstatement, but cherry flavor does not alter nicotine's effects in adolescent rats. *Neurosci Lett*. 2023;793. doi:10.1016/j.neulet.2022.137008	No	Evaluated e-cigarette dependence	Full text screening
295	Cooper SY, Willis CV, Richardson MR, et al. Chemical Flavorants in Vaping Products Alter Neurobiology in a Sex-Dependent Manner to Promote Vaping-Related Behaviors. *Journal of Neuroscience*. 2023;43(8):1360-1374.	No	Evaluated e-cigarette dependence	Full text screening
296	Smith LC, Kallupi M, Tieu L, et al. Validation of a nicotine vapor self-administration model in rats with relevance to electronic cigarette use. *Neuropsychopharmacology*. 2020;45(11):1909-1919.	No	Evaluated e-cigarette dependence	Full text screening
297	Zhu M, Rogers NG, Jahad JV, Herman MA. Sex Differences in the Impact of Electronic Nicotine Vapor on Corticotropin-Releasing Factor Receptor 1 Neurons in the Mouse Ventral Tegmental Area. *J Neurosci*. 2023;43(17):3081-3093.	No	Evaluated e-cigarette dependence	Full text screening
298	Kallupi M, de Guglielmo G, Larrosa E, George O. Exposure to passive nicotine vapor in male adolescent rats produces a withdrawal-like state and facilitates nicotine self-administration during adulthood. *Eur Neuropsychopharmacol*. 2019;29(11):1227-1234.	No	Evaluated e-cigarette dependence	Full text screening
299	Montanari C, Kelley LK, Kerr TM, Cole M, Gilpin NW. Nicotine e-cigarette vapor inhalation effects on nicotine & cotinine plasma levels and somatic withdrawal signs in adult male Wistar rats. *Psychopharmacology* . 2020;237(3):613-625.	No	Evaluated e-cigarette dependence	Full text screening
300	Alasmari F, Crotty Alexander LE, Hammad AM, Bojanowski CM, Moshensky A, Sari Y. Effects of Chronic Inhalation of Electronic Cigarette Vapor Containing Nicotine on Neurotransmitters in the Frontal Cortex and Striatum of C57BL/6 Mice. *Front Pharmacol*. 2019;10(JULY). doi:10.3389/fphar.2019.00885	No	Evaluated e-cigarette dependence	Full text screening
301	Alasmari F, Crotty Alexander LE, Hammad AM, et al. E-cigarette aerosols containing nicotine modulate nicotinic acetylcholine receptors and astroglial glutamate transporters in mesocorticolimbic brain regions of chronically exposed mice. *Chem Biol Interact*. 2021;333. doi:10.1016/j.cbi.2020.109308	No	Evaluated e-cigarette dependence	Full text screening
302	Harris AC, Muelken P, Smethells JR, Krueger M, Le LS. Similar precipitated withdrawal effects on intracranial self-stimulation during chronic infusion of an e-cigarette liquid or nicotine alone. *Pharmacol Biochem Behav*. 2017;161:1-5.	No	Evaluated e-cigarette dependence	Full text screening
303	Harris AC, Muelken P, Alcheva A, Stepanov I, LeSage MG. Cigarette Smoke Extract, but Not Electronic Cigarette Aerosol Extract, Inhibits Monoamine Oxidase in vitro and Produces Greater Acute Aversive/Anhedonic Effects Than Nicotine Alone on Intracranial Self-Stimulation in Rats. *Front Neurosci*. 2022;16. doi:10.3389/fnins.2022.868088	No	Evaluated e-cigarette dependence	Full text screening
304	Martínez M, Espinoza VE, Garcia V, et al. Withdrawal from repeated nicotine vapor exposure increases somatic signs of physical dependence, anxiety-like behavior, and brain reward thresholds in adult male rats. *Neuropharmacology*. 2023;240:109681.	No	Evaluated e-cigarette dependence	Full text screening
305	Abdelmaseih R., Abdelmasih R., Hasan S., Patel J., Abusaada K. Epiglotitis related to e-cigarette smoking -a case report. Am J Respir Crit Care Med. 2021;203. doi:10.1164/ajrccm-conference.2021.203.1_MeetingAbstracts.A3011	No	Non-peer reviewed literature	Full text screening
306	Addicott MA, Sutfin EL, Reynolds LM, Donny EC, Matich EK, Hsu PC. Biochemical validation of self-reported electronic nicotine delivery system and tobacco heaviness of use. Exp Clin Psychopharmacol. 2022. doi:10.1037/pha0000604	No	Wrong outcomes	Full text screening
307	Advani I.N., Du M., Moshensky A., Shin J., Gunge D., Javier C.J., et al. Vaping induced metabolite signatures driven by device type, e-liquid, and sex. Am J Respir Crit Care Med. 2021;203. doi:10.1164/ajrccm-conference.2021.203.1_MeetingAbstracts.A3096	No	Non-peer reviewed literature	Full text screening
308	Ahmed H., Ismayl M., Palicherla A., May J., Thirumalareddy J. Vaping Associated Sudden Cardiac Arrest in a Young Healthy Patient. Circulation. 2023;148. doi:10.1161/circ.148.suppl_1.16037	No	Non-peer reviewed literature	Full text screening
309	Alavi R., Dai W., Arechavala R.J., Kleinman M.T., Kloner R.A., Pahlevan N.M. Detection of the Effect of Nicotine Delivered by E-Cigarettes or Standard Cigarettes on Cardiovascular System From a Carotid Waveform Using a Physics-Based Machine Learning Approach. Circulation. 2022;146. doi:10.1161/circ.146.suppl_1.12922	No	Non-peer reviewed literature	Full text screening
310	Ali S., McClafferty A., Misra A. ST ELEVATION MYOCARDIAL INFARCTION FOLLOWING VAPING-ASSOCIATED PULMONARY INJURY. J Am Coll Cardiol. 2021;77: 2516. doi:10.1016/S0735-1097%2821%2903871-7	No	Wrong outcomes	Full text screening
311	Alkhadrawi H., Snoderly H., Panchal D.M., Bennewitz M., Weaver K. Acute Nicotine-Free E-cigarette Vapor Exposure Modulates Immune Response in Murine Lung Vasculature. Mol Imaging Biol. 2022;24: S386–S387. doi:10.1007/s11307-022-01794-2	No	Non-peer reviewed literature	Full text screening
312	Almutairi RM, Al-Dubai SAR, Almutairi MM, Alharbi HH, Alshlahi HM, Albadrani MS. The degree of nicotine dependence among users of different tobacco and nicotine products in Madinah City, Saudi Arabia. J Fam Community Med. 2023;30: 30–36. doi:10.4103/jfcm.jfcm_181_22	No	Wrong outcomes	Full text screening
313	ALSIBAI T., ABID MEMON H., BANGASH B., MARAGIRI S. A CASE OF SEVERE HYPOXEMIA DUE TO LUNG INJURY FROM VAPING. Chest. 2023;164: A4694–A4695. doi:10.1016/j.chest.2023.07.3042	No	Non-peer reviewed literature	Full text screening
314	Amraotkar A.R., Owolabi U.S., Malovichko M., Benjamin E.J., Fetterman J.L., Hirsch G.A., et al. Association of electronic nicotine delivery systems use with circulating angiogenic cell levels. Circulation. 2021;144. doi:10.1161/circ.144.suppl-1.11398	No	Non-peer reviewed literature	Full text screening

315	Arambulo J.L., Espinoza-Derout J., Hasan K., Jordan M.C., Lao C.J., Wilson J., et al. CARDIAC TRANSCRIPTOME ANALYSIS of the ELECTRONIC CIGARETTE-INDUCED CARDIAC DYSFUNCTION MOUSE MODEL TREATED with ACIPIMOX. J Investig Med. 2021;70: 223. doi:10.1136/jim-2022-WRMC.221	No	Non-peer reviewed literature	Full text screening
316	Arjun S., Sayedy N., Mehta V., Akella J., Iqbal J. Rare Case of Rapid Empyema Caused by Streptococcus Anginosus Group. Am J Respir Crit Care Med. 2022;205. doi:10.1164/ajrccm-conference.2022.205.1_MeetingAbstracts.A4205	No	Non-peer reviewed literature	Full text screening
317	Ashford K.B., Chavan N.R., Wiggins A., Barnett J., McCubbin A., Ducas L., et al. Prenatal electronic cigarette, dual use and nicotine dependency. Am J Obstet Gynecol. 2018;218: S431–S432.	No	Non-peer reviewed literature	Full text screening
318	Ashford KB, Chavan NR, Wiggins A, Barnett J, McCubbin A, Ducas L, et al. 717: Prenatal electronic cigarette, dual use and nicotine dependency. Am J Obstet Gynecol. 2018;218: S431–S432. doi:10.1016/j.ajog.2017.11.248	No	Publication duplicate	Full text screening
319	Ashraf O., Nasrullah A., Karna R., Alhajhusain A. Vaping associated spontaneous pneumothorax - A case series of an enigmatic entity! Respir Med Case Rep. 2021;34: 101535. doi:10.1016/j.rmcr.2021.101535	No	Evaluated effects of cannabis vapour exposure only	Full text screening
320	Astudillo C, Garcia-Perdomo HA. Electronic cigarette: is it a risk factor for the development of bladder cancer?. Actas Urol Esp. 2023;47: 327–328. doi:10.1016/j.acuroe.2023.04.007	No	Non-peer reviewed literature	Full text screening
321	Atarere J.O., Glover Q., Orhurhu V., Delungahawatta T., Osman M., Thompson C.C. RELATIONSHIP BETWEEN E-CIGARETTE USE AND COLORECTAL CANCER SCREENING AMONG U.S. ADULTS. Gastroenterology. 2023;164: S-323. doi:10.1016/S0016-5085%2823%2901730-4	No	Non-peer reviewed literature	Full text screening
322	AYAZ AHMED M., HOSSAIN ESHAN S., ISHA S., SUN C., MEDINA INOJOSA J.O.S.E., HUSSEIN S.A.M.I., et al. VAPING: A RISKY VALSALVA MANEUVER? A CASE OF VAPING OR ELECTRONIC CIGARETTE-INDUCED SPONTANEOUS PNEUMOMEDIASTINUM. Chest. 2023;164: A6363–A6364. doi:10.1016/j.chest.2023.07.4099	No	Non-peer reviewed literature	Full text screening
323	Aziz-Ur-rahman, Mohamed M.H.N., Mahmood S., Balaraman A.K., Baig M.A.I. Development and assessment of modified glover nilsson vaping behavioural questionnaire among malaysian electronic cigarettes users. Curr Trends Biotechnol Pharm. 2020;14: 32–37. doi:10.5530/ctbp.2020.4s.4	No	Wrong outcomes	Full text screening
324	Baker MM, Procter TD, Belzak L, Ogunnaike-Cooke S. Vaping-associated lung illness (VALI) in Canada: a descriptive analysis of VALI cases reported from September 2019 to December 2020. Health Promot Chronic Dis Prev Can Res Policy Pract. 2022;42: 37–44. doi:10.24095/hpcdp.42.1.06	No	Wrong outcomes	Full text screening
325	Bandela M., Lee J., Dudek S. E-CIGARETTE CONTENT ALTERS MITOCHONDRIAL AND BIOPHYSICAL PROPERTIES OF LUNG ENDOTHELIUM. J Investig Med. 2022;70: 1557. doi:10.1136/jim-2022-MW.1	No	Non-peer reviewed literature	Full text screening
326	Basma H., Lowes B.D., Dhar K., Qiu F. Both electronic and regular cigarettes induce transcriptional changes in IPS-derived cardiomyocytes. Circulation. 2021;144. doi:10.1161/circ.144.suppl_1.12948	No	Non-peer reviewed literature	Full text screening
327	Behrooz L., Xie W., Goghari A., Robertson R.M., Bhatnagar A., Stokes A., et al. Electronic Cigarette Use and Chest Pain Report in US Adults. Circulation. 2022;146. doi:10.1161/circ.146.suppl_1.10088	No	Non-peer reviewed literature	Full text screening
328	Bell R.L., McAuley D.F., Shyamsundar M., O'Kane C.M., Dombrowski Y. E-cigarette vapour from base components propylene glycol and vegetable glycerine inhibits the inflammatory response in macrophages and epithelial cells. bioRxiv. 2022. doi:10.1101/2022.03.11.483808	No	Non-peer reviewed literature	Full text screening
329	Berenguer C, Pereira JAM, Câmara JS. Urinary volatomic profile of traditional tobacco smokers and electronic cigarettes users as a strategy to unveil potential health issues. J Sep Sci. 2022;45: 582–593. doi:10.1002/jssc.202100671	No	Wrong outcomes	Full text screening
330	Bertani AL, Tanni SE, Godoy I. Dual and Poly Use of Tobacco Products in a Sample of Pregnant Smokers: A Cross-sectional Study. Matern Child Health J. 2023;27: 1616–1620. doi:10.1007/s10995-023-03698-1	No	Wrong outcomes	Full text screening
331	Blagev DP, Callahan SJ, Harris D, Collingridge DS, Hopkins RO, Eve JR, et al. Prospectively Assessed Long-Term Outcomes of Patients with E-Cigarette- or Vaping-associated Lung Injury. Ann Am Thorac Soc. 2022;19: 1892–1899. doi:10.1513/AnnalsATS.202201-049OC	No	Wrong outcomes	Full text screening
332	Bliton K., Oates G. Vaping and vaping exposure in children, adolescents, and young adults with cystic fibrosis: Prevalence and outcomes. J Cyst Fibros. 2022;21: S34–S35. doi:10.1016/S1569-1993%2822%2900747-0	No	Non-peer reviewed literature	Full text screening
333	BOPPANA H.K., NIU C., GENESE F., S SONDHI D. DYSKERATOSIS CONGENITA: A RARE CAUSE OF USUAL INTERSTITIAL PNEUMONIA. Chest. 2022;162: A2140. doi:10.1016/j.chest.2022.08.1767	No	Non-peer reviewed literature	Full text screening
334	Borja T., Ilahi Z., Sangan S., Romanos-Sirakis E. A CASE OF EVALI PRESENTING WITH COAGULOPATHY IN THE SETTING OF A RECENT COVID-19 INFECTION. Pediatr Blood Cancer. 2022;69: S211–S212. doi:10.1002/pbc.29735	No	Non-peer reviewed literature	Full text screening
335	Bors L.A., Szabo E., Orsolits B., Merkely B., Apati A., Molnar A.A., et al. Assessment of cardiovascular effects of nicotine in using human induced pluripotent stem cell-derived endothelial cells from identical twins. Cardiovasc Res. 2022;118: i168. doi:10.1093/cvr/cvac066.155	No	Wrong outcomes	Full text screening
336	BOWKER W.E., IUSIM S. VAPING-INDUCED ACUTE EOSINOPHILIC PNEUMONIA. Chest. 2023;164: A3215. doi:10.1016/j.chest.2023.07.2105	No	Non-peer reviewed literature	Full text screening
337	Boyd CJ, Veliz P, Evans-Polce R, Eisman AB, Esteban McCabe S. Latent class trajectories: U.S. adolescents' nicotine use and its association with nicotine dependence. Addict Behav Rep. 2020;12: 100303. doi:10.1016/j.abrep.2020.100303	No	Wrong outcomes	Full text screening
338	Bozier J., Wang B., de Vries M., Brandsma C.-A., van den Berge M., Timens W., et al. E-cigarette vapour induces cellular senescence in primary lung fibroblasts and may contribute to lung pathology. bioRxiv. 2023. doi:10.1101/2023.08.30.555286	No	Non-peer reviewed literature	Full text screening
339	Brandon T, Martinez U, Simmons V, Meltzer L, Sutton S, Drobes D, et al. Dual use of combustible and electronic cigarettes: patterns and associations between products. 2018;16: 90-. doi:10.18332/tid/83791	No	Wrong outcomes	Full text screening
340	Bremmer MP, Campbell AM, Xia K, Tarran R, Girdler SS, Hendershot CS. Effects of nicotine content and preferred flavor on subjective responses to e-cigarettes: A randomized, placebo-controlled laboratory study. Nicotine Tob Res Off J Soc Res Nicotine Tob. 2023. doi:10.1093/ntr/ntad143	No	Wrong outcomes	Full text screening
341	Brobst D., Manyanga J., Georgescu C., Wren J., Queimado L. Exposure to E-cigarette aerosol reduces the expression of Toll-like receptor 3 in lung epithelial cells. Cancer Immunol Res. 2021;9. doi:10.1158/2326-6074.TUMIMM20-PO033	No	Non-peer reviewed literature	Full text screening
342	Buu A, Cai Z, Li R, Wong SW, Lin HC, Su WC, et al. Validating E-Cigarette Dependence Scales Based on Dynamic Patterns of Vaping Behaviors. Nicotine Tob Res. 2021;23: 1484–1489. doi:10.1093/ntr/ntab050	No	Wrong outcomes	Full text screening
343	Buu A, Hu YH, Piper ME, Lin HC. The association between e-cigarette use characteristics and combustible cigarette consumption and dependence symptoms: Results from a national longitudinal study. Addict Behav. 2018;84: 69–74. doi:10.1016/j.addbeh.2018.03.035	No	Wrong outcomes	Full text screening
344	Buu A, Tong Z, Cai Z, Li R, Yang J, Jorenby DE, et al. Subtypes of dual users of combustible and electronic cigarettes: longitudinal changes in product use and dependence symptomatology. Nicotine Tob Res. 2022. doi:10.1093/ntr/ntac151	No	Wrong outcomes	Full text screening
345	Buu A, Tong Z, Cai Z, Li R, Yang JJ, Jorenby DE, et al. Subtypes of dual users of combustible and electronic cigarettes: Longitudinal changes in product use and dependence symptomatology. Nicotine Tob Res. 2023;25: 438–443. doi:10.1093/ntr/ntac151	No	Publication duplicate	Full text screening
346	Camenga DR, Haeny AM, Krishnan-Sarin S, O'Malley SS, Bold KW. Pilot Ecological Momentary Assessment Study of Subjective and Contextual Factors Surrounding E-Cigarette and Combustible Tobacco Product Use among Young Adults. Int J Env Res Public Health. 2021;18. doi:10.3390/ijerph182111005	No	Wrong outcomes	Full text screening

347	Canchola A, Ahmed CMS, Chen K, Chen JY, Lin YH. Formation of Redox-Active Duroquinone from Vaping of Vitamin E Acetate Contributes to Oxidative Lung Injury. Chem Res Toxicol. 2022;35: 254–264. doi:10.1021/acs.chemrestox.1c00309	No	Evaluated effect of cannabis vapour	Full text screening
348	Cano Aguirre MDP, Esperanza Barrios A, Martínez Muñiz F, Alonso Viteri S, Muñiz González F, Segoviano Mateo R, et al. Hemoptysis induced by vaping. Arch Bronconeumol. 2021;57: 505–506. doi:10.1016/j.arbr.2021.05.006	No	Non-peer reviewed literature	Full text screening
349	Carmel B., CANIZARES-OTERO M., Srinivasan V., Vazquez Saad H. A SINGLE ODDITY: EVALI OF THE UNILATERAL LUNG. Chest. 2021;160: A2447. doi:10.1016/j.chest.2021.07.2114	No	Non-peer reviewed literature	Full text screening
350	Caruso M, Emma R, Distefano A, Rust S, Poulas K, Giordano A, et al. Comparative assessment of electronic nicotine delivery systems aerosol and cigarette smoke on endothelial cell migration: The Replica Project. Drug Test Anal. 2023;15: 1164–1174. doi:10.1002/dta.3349	No	Publication duplicate	Full text screening
351	Castaldi P. Blood transcriptomic and proteomic biomarkers of electronic nicotine delivery systems use in the copdgene study. Am J Respir Crit Care Med. 2021;203. doi:10.1164/ajrccm-conference.2021.203.1_MeetingAbstracts.A3097	No	Non-peer reviewed literature	Full text screening
352	Chaiton M, Pienkowski M, Musani I, Bondy SJ, Cohen JE, Dubray J, et al. Smoking, e-cigarettes and the effect on respiratory symptoms among a population sample of youth: Retrospective cohort study. Tob Induc Dis. 2023;21: 08. doi:10.18332/tid/156839	No	Publication duplicate	Full text screening
353	Chandna S., Shah M., Bhasin S., Aftab G., Watts A. A CONFOUNDING CASE OF SUPERIOR VENA CAVA SYNDROME. Chest. 2021;160: A1598. doi:10.1016/j.chest.2021.07.1460	No	Non-peer reviewed literature	Full text screening
354	Chidharla A, Agarwal K, Abdelwahed S, Bhandari R, Singh A, Rabbani R, et al. Cancer Prevalence in E-Cigarette Users: A Retrospective Cross-Sectional NHANES Study. World J Oncol. 2022;13: 20–26. doi:10.14740/wjon1438	No	Retracted literature	Full text screening
355	Chitteti R., Zuniga-Hertz J.P., Masso-Silva J.A., Crotty Alexander L.E., Shin J., Patel H.H., et al. Plasma Mediators in E Cigarette Users Inhibit SARS-CoV-2 Pseudovirus Infection. Am J Respir Crit Care Med. 2022;205. doi:10.1164/ajrccm-conference.2022.205.1_MeetingAbstracts.A1282	No	Non-peer reviewed literature	Full text screening
356	Choi S, Lee K, Park SM. Combined Associations of Changes in Noncombustible Nicotine or Tobacco Product and Combustible Cigarette Use Habits With Subsequent Short-Term Cardiovascular Disease Risk Among South Korean Men: A Nationwide Cohort Study. Circulation. 2021;144: 1528–1538. doi:10.1161/CIRCULATIONAHA.121.054967	No	Evaluated effects of heated tobacco products exposure	Full text screening
357	Chua T.H., Takano A., Yao Y.J., Chow S.Y., Devanand A., Tay C.K. Autoimmune pulmonary alveolar proteinosis with a history of vaping and vitamin E-positive bronchoalveolar lavage. Respirol Case Rep. 2021;9: e0864. doi:10.1002/rcr2.864	No	Evaluated effects of cannabis vapour exposure only	Full text screening
358	Clendennen SL, Case KR, Sumbe A, Mantey DS, Mason EJ, Harrell MB. Stress, Dependence, and COVID-19-related Changes in Past 30-day Marijuana, Electronic Cigarette, and Cigarette Use among Youth and Young Adults. Tob Use Insights. 2021;14: 1179173X211067439. doi:10.1177/1179173X211067439	No	Evaluated effects of cannabis vapour exposure only	Full text screening
359	Colaco B., Arunthari V. Vaping causes lung injury. Respirology. 2023;28: 174–175. doi:10.1111/resp.14433	No	Non-peer reviewed literature	Full text screening
360	Creed K., Siddiqui S., Wichmann A., Test V. VAPING-ASSOCIATED LUNG INJURY COMPLICATED BY SEVERE ARDS AND PNEUMOTHORAX. Chest. 2021;160: A2448. doi:10.1016/j.chest.2021.07.2115	No	Non-peer reviewed literature	Full text screening
361	D. HILTON J., RAMAN S.M. DIFFUSE ALVEOLAR HEMORRHAGE FROM ELECTRONIC CIGARETTE USE: A RARE MANIFESTATION OF AN INCREASINGLY COMMON DISEASE. Chest. 2023;164: A3162. doi:10.1016/j.chest.2023.07.2074	No	Non-peer reviewed literature	Full text screening
362	Dai W., Shi J., Carreno J., Kleinman M.T., Herman D., Johnson R., et al. The Effect of Chronic Standard Cigarette Smoke Exposure Compared to Chronic Electronic Cigarette Exposure With or Without Nicotine on Blood Pressure. Circulation. 2023;148. doi:10.1161/circ.148.suppl_1.13349	No	Non-peer reviewed literature	Full text screening
363	Dai W., Shi J., Carreno J., Kleinman M.T., Herman D., Johnson R., et al. The Effect of Electronic Cigarette Exposure on Flow Mediated Vasodilation and LV Remodeling in a Chronic Myocardial Infarction Model. Circulation. 2023;148. doi:10.1161/circ.148.suppl_1.13361	No	Non-peer reviewed literature	Full text screening
364	Dai W., Shi J., Zhao L., Carreno J., Kleinman M.T., Herman D., et al. Differences in Infarct Size and No-Reflow by Sex in Young Adult Rats in an Electronic Cigarette Exposure Study. Circulation. 2022;146. doi:10.1161/circ.146.suppl_1.13315	No	Non-peer reviewed literature	Full text screening
365	Dai W., Shi J., Zhao L., Carreno J., Kleinman M.T., Herman D., et al. Impact of Chronic Electronic Cigarette Vapor Exposure on the Recovery of Cardiovascular Function in Experimental Rat Myocardial Ischemia/Reperfusion Model. Circulation. 2022;146. doi:10.1161/circ.146.suppl_1.13324	No	Non-peer reviewed literature	Full text screening
366	De La Garza R, Shuman S, Yammine L, Yoon J, Salas R, Holst M. A Pilot Study of E-Cigarette Naïve Cigarette Smokers and the Effects on Craving After Acute Exposure to E-Cigarettes in the Laboratory. 2019;28: 361-366. doi:10.1111/ajad.12895	No	Wrong outcomes	Full text screening
367	Dimitriadis K., Narkiewicz K., Leontsinis I., Konstantinidis D., Mihas C., Andrikou I., et al. Sympathetic nerve activity changes following acute exposures to electronic and tobacco cigarette smoking in humans. Eur Heart J. 2021;42: 2596. doi:10.1093/eurheartj/ehab724.2596	No	Non-peer reviewed literature	Full text screening
368	Djordjevic I., Kuntic M., Oelze M., Munzel T., Daiber A. Impact of E-cigarette vapor condensate on NADPH oxidase activity in cultured endothelial or macrophage cells - comparison with effects of acrolein. Free Radic Biol Med. 2022;180: s73. doi:10.1016/j.freeradbiomed.2021.12.169	No	Non-peer reviewed literature	Full text screening
369	Djordjevic I., Kuntic M., Oelze M., Munzel T., Daiber A. [YIA] Changes in NADPH oxidase activity in E-cigarette vapor condensate exposed cultured cells and the role of acrolein. Free Radic Biol Med. 2022;189: 15. doi:10.1016/j.freeradbiomed.2022.06.074	No	Non-peer reviewed literature	Full text screening
370	Dowd AN, Motschman CA, Tiffany ST. Development and Validation of the Questionnaire of Vaping Craving. Nicotine Tob Res. 2019;21: 63–70. doi:10.1093/ntr/nty046	No	Wrong outcomes	Full text screening
371	Duc H.L., Xuan S.N.H. Cerebral Infarction and Association with Substance Use Disorders - a Nationwide Study. Cerebrovasc Dis. 2022;51: 124–125. doi:10.1159/000528018	No	Non-peer reviewed literature	Full text screening
372	Dugas EN, Sylvestre MP, O'Loughlin J. Type of e-liquid vaped, poly-nicotine use and nicotine dependence symptoms in young adult e-cigarette users: a descriptive study. BMC Public Health. 2020;20: 922. doi:10.1186/s12889-020-09056-y	No	Evaluated effects of cannabis vapour exposure only	Full text screening
373	Durmus N., Grunig G., Raja A., Goriainova V., Joung H., Chalupa D., et al. Long-Term Toxicity of E-Cigarette Whole Body Aerosol Exposure Using Cardiovascular Health and Pulmonary Changes in Mice as Persistent Outcomes. Am J Respir Crit Care Med. 2022;205. doi:10.1164/ajrccm-conference.2022.205.1_MeetingAbstracts.A1149	No	Non-peer reviewed literature	Full text screening
374	Dweik A., Dharmarpandi G., Al-Bayati M., Hassan F.A., Waqas R., Walker J. Vaping induced platelet dysfunction. Am J Med Sci. 2023;365: S116. doi:10.1016/S0002-9629%2823%2900224-0	No	Non-peer reviewed literature	Full text screening
375	Elmasry O., Chae S.Y., Ding J. A Young Man With E-Cigarette or Vaping-Associated Lung Injury. Consultant. 2022;62: 8–11. doi:10.25270/con.2021.04.00015	No	Non-peer reviewed literature	Full text screening
376	Estifanos Y., Peterson M.R., Rice T., He G. Electronic cigarette vaping with or without heating induces differential pathological remodeling in the lungs and right heart. Circulation. 2021;144. doi:10.1161/circ.144.suppl-1.11365	No	Non-peer reviewed literature	Full text screening
377	Fagerstrom K. A Comparison of Dependence across Different Types of Nicotine Containing Products and Coffee. Int J Env Res Public Health. 2018;15. doi:10.3390/ijerph15081609	No	Wrong outcomes	Full text screening

378	Fearon IM, Seltzer RGN, Houser TL, Tope A, Cahours X, Verron T, et al. Examination of the impact of myblu electronic nicotine delivery system e-liquid nicotine strength on self-reported measures of dependence. Drug Test Anal. 2023;15: 1270–1280. doi:10.1002/dta.3335	No	Publication duplicate	Full text screening
379	Field W., Ahmed N., Hasan Choudhury N., Patel D. VAPING, THE NEW CIGARETTE: TWO CASE REPORTS OF VAPING-ASSOCIATED LUNG INJURY. Chest. 2021;160: A1742. doi:10.1016/j.chest.2021.07.1586	No	Non-peer reviewed literature	Full text screening
380	Flashner B., Kanarek D.J., O'Donnell W.J., Soskis A., Ziehr D.R., Frank A.J., et al. Chronic Bronchiolocentric Fibrosis from E-Cigarette Use. Am J Respir Crit Care Med. 2022;205. doi:10.1164/ajrccm-conference.2022.205.1_MeetingAbstracts.A1345	No	Non-peer reviewed literature	Full text screening
381	Ganapathy V., Manyanga J., Queimado L. Differential expression of antioxidant and detoxifying enzymes induced by e-cigarette aerosol. Cancer Res. 2021;81. doi:10.1158/1538-7445.AM2021-2021	No	Non-peer reviewed literature	Full text screening
382	Garey L, Mayorga NA, Peraza N, Smit T, Nizio P, Otto MW, et al. Distinguishing Characteristics of E-Cigarette Users Who Attempt and Fail to Quit: Dependence, Perceptions, and Affective Vulnerability. J Stud Alcohol Drugs. 2019;80: 134–140.	No	Wrong outcomes	Full text screening
383	Garrido Márquez I, Sánchez Torrente A, Pérez Cuenca E. Pulmonary Disease Due to the Use of Electronic Cigarettes (EVALI): About a Case. Arch Bronconeumol. 2023;59: 324–325. doi:10.1016/j.arbres.2022.12.009	No	Evaluated effects of cannabis vapour exposure only	Full text screening
384	George O., Kallupi M., Tieu L., Jaquish A., Barr J., Su Y., et al. Vaping electronic cigarettes with nicotine produces addiction-like behaviors and prolonged pulmonary abnormalities in rats. Neuropsychopharmacology. 2019;44: 370. doi:10.1038/s41386-019-0546-x	No	Non-peer reviewed literature	Full text screening
385	Giner P., Mendez I. Exposure to nicotine using a model of electronic vapor inhalation increases risky choice and beta2 nicotinic receptor subunit gene expression in rats. Neuropsychopharmacology. 2021;46: 453. doi:10.1038/s41386-021-01238-5	No	Non-peer reviewed literature	Full text screening
386	Girish G, Xiang B, Hsu LL. A 21-Year-Old Woman with Sickle Cell Disease and Vaso-Occlusive Pain Associated with Using an Electronic Nicotine Dispensing System (E-Cigarette or Vape) - a Case Report. Am J Case Rep. 2023;24: e941268. doi:10.12659/AJCR.941268	No	Wrong outcomes	Full text screening
387	GODUGUCHINTA V., ELSAYED N., SHAFRANYUK P., AKHTER O.M.A.R. A RARE JUUL: AN INTERESTING CASE OF LEGIONELLA PNEUMONIA FROM VAPING. Chest. 2023;164: A6401. doi:10.1016/j.chest.2023.07.4126	No	Non-peer reviewed literature	Full text screening
388	Golob S., Winston L., Manson D.K., Fedyna S. E-cigarette or vaping-product associated lung injury complicated by spontaneous pneumothoraces in the setting of covid-19 pandemic. Am J Respir Crit Care Med. 2021;203. doi:10.1164/ajrccm-conference.2021.203.1_MeetingAbstracts.A1993	No	Non-peer reviewed literature	Full text screening
389	Gonuguntla V., Soni P., Yizhak K. Pneumomediastinum in vaping induced lung injury. Am J Respir Crit Care Med. 2021;203. doi:10.1164/ajrccm-conference.2021.203.1_MeetingAbstracts.A3013	No	Non-peer reviewed literature	Full text screening
390	Gonzalez M.R., Weiss L., Boucek M. E-cigarette or Vaping Product Use Associated Lung Injury (EVALI) in Teenager After Reported COVID-19 Infection. Pediatrics. 2022;149. Available: https://publications.aap.org/pediatrics/article/149/1MeetingAbstractsFebruary2022/739/186224/E-cigarette-or-Vaping-Product-Use-Associated-Lung	No	Non-peer reviewed literature	Full text screening
391	González-Roz A, MacKillop J. No evidence of differences in smoking levels, nicotine dependence, carbon monoxide or motivational indices between cigarette smokers and cigarette + e-cigarette dual users in two samples. Addict Behav. 2021;112: 106543. doi:10.1016/j.addbeh.2020.106543	No	Wrong outcomes	Full text screening
392	Gregory A., Xu Z., Pratte K., Berman S., Lu R., Suryadevara R., et al. Electronic nicotine delivery systems use is associated with multi-omic biomarkers and prospective cardiopulmonary health outcomes. medRxiv. 2022. doi:10.1101/2022.09.19.22280093	No	Non-peer reviewed literature	Full text screening
393	Gupta N., Condiles N., Ahmed B., Liu S. A Complex Case of Acute Hypoxemic Respiratory Failure in 2020. Am J Respir Crit Care Med. 2021;203. doi:10.1164/ajrccm-conference.2021.TP100	No	Non-peer reviewed literature	Full text screening
394	Gutierrez J., Hirth J., Zoorob R. ASSOCIATION OF HIGH SENSITIVITY C-REACTIVE PROTEIN LEVELS WITH ELECTRONIC CIGARETTE USE COMPARED TO TRADITIONAL CIGARETTE, DUAL USERS, AND NON- SMOKERS AMONG US ADULTS. J Gen Intern Med. 2023;38: S159. doi:10.1007/s11606-023-08226-z	No	Non-peer reviewed literature	Full text screening
395	Hajek P, Pittaccio K, Pesola F, Myers Smith K, Phillips-Waller A, Przulj D. Nicotine delivery and users' reactions to Juul compared with cigarettes and other e-cigarette products. Addiction. 2020;115: 1141–1148. doi:10.1111/add.14936	No	Wrong outcomes	Full text screening
396	Hamid K., Perinkulam Sathyanarayanan S., Hamza M., Helberg S.B. Vaping, is it a safer alternative then smoking? Am J Respir Crit Care Med. 2021;203. doi:10.1164/ajrccm-conference.2021.203.1_MeetingAbstracts.A3015	No	Non-peer reviewed literature	Full text screening
397	Hamilton M., Suliman M., Ezeh E., Akhigbe E. VAPING-INDUCED ALVEOLAR RUPTURE LEADING TO PNEUMOMEDIASTINUM IN A YOUNG FEMALE. Chest. 2021;160: A1294. doi:10.1016/j.chest.2021.07.1183	No	Non-peer reviewed literature	Full text screening
398	Han D.D., Xu F., Derakhshandeh R., Huang A., Whitlatch A., Keith R.J., et al. Inhibition of receptor for advanced glycation end products (RAGE) reduces e-cigarette-induced increase in endothelial permeability. Circulation. 2021;144. doi:10.1161/circ.144.suppl-1.9929	No	Non-peer reviewed literature	Full text screening
399	Hayes D Jr, Board A, Calfee CS, Ellington S, Pollack LA, Kathuria H, et al. Pulmonary and Critical Care Considerations for e-Cigarette, or Vaping, Product Use-Associated Lung Injury. Chest. 2022;162: 256–264. doi:10.1016/j.chest.2022.02.039	No	Wrong outcomes	Full text screening
400	He M., Qing K., Tustison N., Myc L.A., MacLeod J., Nunoo-Asare R., et al. Probing early-stage pulmonary pathophysiology in young healthy ecigarettes users using hyperpolarized 129Xe MRI. Am J Respir Crit Care Med. 2021;203. doi:10.1164/ajrccm-conference.2021.203.1_MeetingAbstracts.A1113	No	Non-peer reviewed literature	Full text screening
401	Helgertz S, Kingsbury J. Teens less susceptible to vaping when restricted to tobacco-flavored e-cigarettes: Implications for flavored tobacco policies. Nicotine Tob Res. 2023;25: 991–995. doi:10.1093/ntr/ntac272	No	Wrong outcomes	Full text screening
402	Herriges M.J., Pinkhasov R., Shapiro O., Jacob J.M., Basnet A., Bratslavsky G., et al. E-cigarette use and the risk of bladder and lung cancer. J Clin Oncol. 2022;40. doi:10.1200/JCO.2022.40.6_suppl.443	No	Non-peer reviewed literature	Full text screening
403	Hickman E., Duffney P., Brocke S., Bailey A., Wells H., Robinette C., et al. Differences in airway biomarkers of inflammation and lung injury in pod and previous generation e-cigarette users. Am J Respir Crit Care Med. 2021;203. doi:10.1164/ajrccm-conference.2021.203.1_MeetingAbstracts.A3103	No	Non-peer reviewed literature	Full text screening
404	Hinds D., Nick H.J., Vallin T., White C.W., Bosco-Lauth A. Effect of Vaping on Lung Inflammation and SARS-CoV-2 Infection in a Hamster Model. Am J Respir Crit Care Med. 2022;205. doi:10.1164/ajrccm-conference.2022.205.1_MeetingAbstracts.A1151	No	Non-peer reviewed literature	Full text screening
405	Ho D., Towns B., Baskerville W., Jenkins J., Green R., Gillis A., et al. Electronic nicotine delivery systems (ENDS) use among heavy drinking smokers. Alcohol Clin Exp Res. 2020;44: 118A. doi:10.1111/acer.14356	No	Non-peer reviewed literature	Full text screening
406	Ho M., Ho M.S., Librach C. iPSC: NEW STRATEGIES TO HEAL A BROKEN HEART: HUMAN UMBILICAL CORD PERIVASCULAR CELLS ALLEVIATE VAPING-INDUCED CARDIOTOXICITY VIA PARACRINE MECHANISMS. Cytotherapy. 2022;24: S149–S150. doi:10.1016/S1465-3249%2822%2900396-6	No	Non-peer reviewed literature	Full text screening
407	Ho M., Librach C. Acute exposure to vaping e-liquid induces multiple cardiotoxic effects on fetal-like human induced pluripotent stem cell-derived cardiomyocytes and epicardial cells. Circulation. 2021;144. doi:10.1161/circ.144.suppl-1.9810	No	Non-peer reviewed literature	Full text screening
408	Ho M., Librach C. Not Just Smoke: The Cytotoxicity and Adverse Consequences of ELiquid Flavors on Human Induced Pluripotent Stem Cell-Derived Lung Epithelium and Cardiomyocytes. Am J Respir Crit Care Med. 2022;205. doi:10.1164/ajrccm-conference.2022.205.1_MeetingAbstracts.A3263	No	Non-peer reviewed literature	Full text screening

409	Ho M.S., Ho M., Librach C. Exosomes/EVs: ENDOTHELIAL CELL-PERICYTE CO-CULTURE DERIVED EXTRACELLULAR VESICLES MITIGATE APOPTOSIS AND PATHOLOGICAL METABOLIC REPROGRAMMING OF CARDIOMYOCYTES EXPOSED TO VAPING LIQUID. Cytotherapy. 2023;25: S110. doi:10.1016/S1465-3249%2823%2900336-5	No	Non-peer reviewed literature	Full text screening
410	HOCHFELD U.R.I., MOHAMMED A.S.N.A., HONG S.H., RAHMAN W., MINOCK M., OMIDVARI K. E-CIGARETTE USE AS AN INDEPENDENT RISK FACTOR FOR SEVERE SARS-COV-2 INFECTION. Chest. 2023;164: A4851–A4852. doi:10.1016/j.chest.2023.07.3143	No	Non-peer reviewed literature	Full text screening
411	Hoekstra N.E., Hinds D., Dannull K.A., Liptzin D. Vaping and diffuse alveolar hemorrhage: All e-cigarette, or vaping, product use associated lung injury (evali) is not created equal. Am J Respir Crit Care Med. 2021;203. doi:10.1164/ajrccm-conference.2021.203.1_MeetingAbstracts.A3205	No	Non-peer reviewed literature	Full text screening
412	Holt LJ, Ginley MK, Pingeon C, Feinn R. Primed for positive perceptions? Applying the acquired preparedness model to explain college students' e-cigarette use and dependence. J Am Coll Health. 2022; 1–11. doi:10.1080/07448481.2022.2089846	No	Wrong outcomes	Full text screening
413	Hughes JR, Peters EN, Callas PW, Peasley-Miklus C, Oga E, Etter J-F, et al. Withdrawal Symptoms From E-Cigarette Abstinence Among Adult Never-Smokers: A Pilot Experimental Study. Nicotine Tob Res Off J Soc Res Nicotine Tob. 2020;22: 740–746. doi:10.1093/ntr/ntz169	No	Publication duplicate	Full text screening
414	Hughey C.M., Piasecki T.M., Korcarz C.E., Hansen K.M., Ott N.R., Tattersall M.C., et al. Differences in Treadmill Exercise Stress Testing Parameters Among Electronic Cigarette Vapers, Combustible Cigarette Smokers, and Controls: The Clues Study. Circulation. 2022;146. doi:10.1161/circ.146.suppl_1.10218	No	Non-peer reviewed literature	Full text screening
415	Huh J, Yu S, Galimov A, Meza LR, Galstyan E, Medel D, et al. Hypothetical flavour ban and intention to vape among vape shop customers: the role of flavour preference and e-cigarette dependence. Tob Control. 2023;32: 110–113. doi:10.1136/tobaccocontrol-2020-056321	No	Wrong outcomes	Full text screening
416	Hussein O., Patil S., Mansour M., Saad B. Community-Acquired Pseudomonas Pneumonia Attributed to Electronic Cigarette Vaping in a Young Patient with COVID. Am J Respir Crit Care Med. 2022;205. doi:10.1164/ajrccm-conference.2022.205.1_MeetingAbstracts.A4563	No	Non-peer reviewed literature	Full text screening
417	Iftikhar A., Patel M., Cheema M., Adial A. Vaping Induce Pneumonitis: Missed in Pandemic. Am J Respir Crit Care Med. 2022;205. doi:10.1164/ajrccm-conference.2022.205.1_MeetingAbstracts.A1400	No	Non-peer reviewed literature	Full text screening
418	Ishler KJ, Flocke SA, Albert EL, Trapl E, Gunzler D. Cigarillo and multiple tobacco product use and nicotine dependence in adolescents and young adults. Addict Behav. 2020;111: 106537. doi:10.1016/j.addbeh.2020.106537	No	Wrong outcomes	Full text screening
419	Jabba S.V., Diaz A.N., Caceres A.I., Erythropel H.C., Kumar V., Varghese S., et al. Cellular Respiratory Toxicity of Novel Flavor-Solvent Adducts in Electronic Cigarettes. Am J Respir Crit Care Med. 2021;203. doi:10.1164/ajrccm-conference.2021.TP109	No	Non-peer reviewed literature	Full text screening
420	Jagadeesan C.T., Giclas H., Varda B., Baskaran J. Evere primary pulmonary coccidioidomycosis in an immunocompetent patient with heavy electronic cigarette use. J Gen Intern Med. 2021;36: S353. doi:10.1007/s11606-021-06830-5	No	Non-peer reviewed literature	Full text screening
421	Jankowski M., Brozek G., Krzystanek M., Lawson J., Zejda J. Nicotine dependence levels among e-cigarettes users. Eur Respir J. 2019;54. doi:10.1183/13993003.congress-2019.PA4465	No	Non-peer reviewed literature	Full text screening
422	Jasper AE, Faniyi AA, Davis LC, Grudzinska FS, Halston R, Hazeldine J, et al. E-cigarette vapor renders neutrophils dysfunctional due to filamentous actin accumulation. J Allergy Clin Immunol. 2023. doi:10.1016/j.jaci.2023.08.025	No	Wrong outcomes	Full text screening
423	Ji X. Electronic Cigarettes Smoking Induces Ferroptosis in a Pre-Clinical Model of COPD. Am J Respir Crit Care Med. 2022;205. doi:10.1164/ajrccm-conference.2022.205.1_MeetingAbstracts.A3267	No	Non-peer reviewed literature	Full text screening
424	Jiang E.X., Sangapalaarachchi D.N., LoIacono N.J., Ilievski V., Hawkins D., Oelsner E., et al. E-cigarette Use and Markers of Endothelial Function in Diverse Young Adults From New York City: The VapeScan Study. Circulation. 2022;146. doi:10.1161/circ.146.suppl_1.9921	No	Non-peer reviewed literature	Full text screening
425	Jin L., Richardson A., Fryar L., Lorkiewicz P., Riggs D., Srivastava S., et al. Electronic Cigarette-Derived Aerosols Induce Endothelial Dysfunction: Role of Aldehydes and Transient Receptor Potential Ankyrin-1 in Mice. Circulation. 2022;146. doi:10.1161/circ.146.suppl_1.13745	No	Non-peer reviewed literature	Full text screening
426	Jin L., Riggs D., Richardson A., Lynch J., Fryar L., Theis W., et al. Role of Aldehydes, Transient Receptor Potential Ankyrin 1, and Catecholamines in Electronic Cigarette-Induced Endothelial Dysfunction in Mice. Circulation. 2023;148. doi:10.1161/circ.148.suppl_1.17126	No	Non-peer reviewed literature	Full text screening
427	John WS, Grover K, Greenblatt LH, Schwartz RP, Wu LT. E-Cigarette Use Among Adult Primary Care Patients: Results from a Multisite Study. J Gen Intern Med. 2020;35: 268–275. doi:10.1007/s11606-019-05488-4	No	Wrong outcomes	Full text screening
428	Jones DM, Guy MC, Fairman BJ, Soule E, Eissenberg T, Fagan P. Nicotine Dependence among Current Cigarette Smokers Who Use E-Cigarettes and Cannabis. Subst Use Misuse. 2023;58: 618–628. doi:10.1080/10826084.2023.2177961	No	Wrong outcomes	Full text screening
429	Kastratovic N, Markovic V, Harrell CR, Arsenijevic A, Stojanovic MD, Djonov V, et al. Effects of combustible cigarettes and electronic nicotine delivery systems on the development and progression of chronic lung inflammation in mice. Nicotine Tob Res. 2023. doi:10.1093/ntr/ntad235	No	Evaluated effects of heated tobacco products exposure	Full text screening
430	KHOSA J.K., SPECHT L. WHEN THE VAPORS PART: A CLOUDY CASE OF DIFFUSE ALVEOLAR HEMORRHAGE. Chest. 2022;162: A2134. doi:10.1016/j.chest.2022.08.1762	No	Non-peer reviewed literature	Full text screening
431	Kika N., Patel N., Saleh A. SPONTANEOUS PNEUMOTHORAX SECONDARY TO VAPING. Chest. 2021;160: A1364. doi:10.1016/j.chest.2021.07.1247	No	Non-peer reviewed literature	Full text screening
432	Klibanoff-Dombrowski K., Simeone S., Chigurupati N., Nguyen S., Parikh R. From fashion trend to life support: A severe case of evali requiring ecmo. Am J Respir Crit Care Med. 2021;203. doi:10.1164/ajrccm-conference.2021.203.1_MeetingAbstracts.A2453	No	Non-peer reviewed literature	Full text screening
433	Kopsombut G, Ajjegowda A, Livingston F, Epelman M, Brown B, Werk L, et al. Clinical Findings in Adolescents Hospitalized With EVALI; Novel Report on Coagulopathy. Hosp Pediatr. 2022;12: 229–240. doi:10.1542/hpeds.2021-006059	No	Evaluated effects of cannabis vapour exposure only	Full text screening
434	Kunadharaju R., Salemme R., Tappeta K., Monegro A. VAPING-ASSOCIATED ASTHMA EXACERBATION PRESENTING AS SPONTANEOUS PNEUMOMEDIASTINUM. Chest. 2021;160: A1763. doi:10.1016/j.chest.2021.07.1606	No	Non-peer reviewed literature	Full text screening
435	Kupelian C., Vijayan V., Kallas H., Kim A. YET ANOTHER COMPLICATION OF E-CIGARETTE OR VAPING PRODUCT USE: PULMONARY ASPERGILLOSIS. J Investig Med. 2021;69: 248. doi:10.1136/jim-2021-WRMC.328	No	Evaluated effects of cannabis vapour exposure only	Full text screening
436	Lao C.J., Espinoza-Derout J., Hasan K., Jordan M.C., Wilson J., Molina J., et al. ACIPIMOX PREVENTS CARDIAC DYSFUNCTION INDUCED by ELECTRONIC CIGARETTES in MICE. J Investig Med. 2021;70: 231. doi:10.1136/jim-2022-WRMC.238	No	Non-peer reviewed literature	Full text screening
437	Lau S., Risher J. Association Between Use of Electronic Cigarettes and Stroke. J Am Coll Surg. 2021;233: e96. doi:10.1016/j.jamcollsurg.2021.08.256	No	Non-peer reviewed literature	Full text screening
438	Le H., Liu C.-W., Denaro P., Shao N.-Y., Ong S.-G., Lee W.H. Identification of differentially expressed long non-coding RNAs in human-induced pluripotent stem cell derived endothelial cells triggered by E-cigarette exposure. Circulation. 2021;144. doi:10.1161/circ.144.suppl_1.12461	No	Non-peer reviewed literature	Full text screening
439	Leavens ELS, Lambart L, Diaz FJ, Wagener TL, Ahluwalia JS, Benowitz N, et al. Nicotine delivery and changes in withdrawal and craving during acute electronic cigarette, heated tobacco product, and cigarette use among a sample of Black and White people who smoke. Nicotine Tob Res. 2023. doi:10.1093/ntr/ntad247	No	Wrong outcomes	Full text screening

440	Levine A., Alalawi L., Hussein Y., Sussman R. AN INTERESTING CASE OF E-CIGARETTE OR VAPING PRODUCT USE-ASSOCIATED LUNG INJURY. Chest. 2021;160: A1680. doi:10.1016/j.chest.2021.07.1528	No	Non-peer reviewed literature	Full text screening
441	Liu C.-W., Le H., Denaro P., Ong S.-G., Lee W.H. E-cigarettes induce dysregulation of autophagy leading to endothelial dysfunction in an ipsc model of pulmonary arterial hypertension. Circulation. 2021;144. doi:10.1161/circ.144.suppl_1.11407	No	Non-peer reviewed literature	Full text screening
442	Liu Y, Shen Z, Zhao C, Gao Y. Urine proteomic analysis of the rat e-cigarette model. PeerJ. 2023;11: e16041. doi:10.7717/peerj.16041	No	Wrong outcomes	Full text screening
443	Ljujic M., Trifunovic S., Smiljanic K., Solari F., Sickmann A., Divac Rankov A. Electronic cigarette liquids impair protein synthesis and alter proteomic profiles in V79 cells. Eur Respir J. 2022;60. doi:10.1183/13993003.congress-2022.1934	No	Non-peer reviewed literature	Full text screening
444	Luchnikova T., Mikhailovskii A., Prikhodko O. The impact of electronic smoking systems on the bronchopulmonary system. Respirology. 2021;26: 81. doi:10.1111/resp.14150	No	Non-peer reviewed literature	Full text screening
445	Lyytinen G., Bryndal A., Anesater E., Antoniewicz L., Blomberg A., Wallen H., et al. Electronic cigarettes containing nicotine increase thrombotic activity and impair microcirculation. Eur Respir J. 2021;58. doi:10.1183/13993003.congress-2021.OA2576	No	Non-peer reviewed literature	Full text screening
446	Majid S., Fetterman J.L., Weisbrod R.M., Behrooz L., Stokes A., Blaha M.J., et al. Effects of Pod-Based Electronic Cigarette Use on Vascular Health and Relation to Volatile Organic Compound Exposure in Young Adults. medRxiv. 2022. doi:10.1101/2022.12.16.22283590	No	Non-peer reviewed literature	Full text screening
447	Majid S., Weisbrod R., Feng B., Robertson R.M., Bhatnagar A., Conklin D., et al. The effects of pod-based e-liquids on vascular endothelial cell function. FASEB J. 2021;35. doi:10.1096/fasebj.2021.35.S1.02523	No	Non-peer reviewed literature	Full text screening
448	Majid S., Weisbrod R.M., Fetterman J.L., Keith R.J., Robertson R.M., Bhatnagar A., et al. Pod-based e-liquids induce vascular endothelial cell dysfunction. Circulation. 2021;144. doi:10.1161/circ.144.suppl_1.10999	No	Non-peer reviewed literature	Full text screening
449	Majid S.A., Fetterman J.L., Weisbrod R.M., Stokes A., Blaha M.J., Srivastava S., et al. Association of Volatile Organic Compound Levels With Pod-Based Electronic Cigarette-Induced Changes in Vascular Function of Young Adults. Circulation. 2022;146. doi:10.1161/circ.146.suppl_1.15569	No	Non-peer reviewed literature	Full text screening
450	Maloney SF, Breland A, Soule EK, Hiler M, Ramôa C, Lipato T, et al. Abuse liability assessment of an electronic cigarette in combustible cigarette smokers. Exp Clin Psychopharmacol. 2019;27: 443–454. doi:10.1037/pha0000261	No	Wrong outcomes	Full text screening
451	Manning K., Garey L., Mayorga N.A., Nizio P., Zvolensky M.J. Predicting electronic cigarette dependence and perceived barriers for electronic cigarette cessation: examining the roles of fatigue severity and emotion dysregulation. Fatigue Biomed Health Behav. 2020;8: 11–23. doi:10.1080/21641846.2020.1714111	No	Wrong outcomes	Full text screening
452	MANSOUR C., MOHAMMAD J., KUTSCHE W., KIRK J., CLARKSON D., SCROGGIE D. VAPING AND VATS: A CASE OF BILATERAL PNEUMOTHORACES IN A HEALTHY YOUNG FEMALE. Chest. 2023;164: A6399–A6400. doi:10.1016/j.chest.2023.07.4125	No	Non-peer reviewed literature	Full text screening
453	Marano M., Menchini L., Khalil Ramla M., Perdichizzi S., Villani A., Zampini G., et al. It is not always COVID-19: a case of respiratory failure from lung damage associated with electronic cigarettes (EVALI). Clin Toxicol. 2022;60: 5. doi:10.1080/15563650.2022.2054576	No	Non-peer reviewed literature	Full text screening
454	Martinez M., Uribe K., Garcia V., Lira O., Matos-Ocasio F., Negishi K., et al. Withdrawal from repeated nicotine vapor exposure increases somatic signs of physical dependence, anxiety-like behavior, and brain reward thresholds in adult male rats. bioRxiv. 2022. doi:10.1101/2022.01.08.475467	No	Non-peer reviewed literature	Full text screening
455	Martínez Ú, Martínez-Loredo V, Simmons VN, Meltzer LR, Drobes DJ, Brandon KO, et al. How Does Smoking and Nicotine Dependence Change After Onset of Vaping? A Retrospective Analysis of Dual Users. Nicotine Tob Res. 2020;22: 764–770. doi:10.1093/ntr/ntz043	No	Wrong outcomes	Full text screening
456	Masonbrink A, Richardson T, Hall M, Catley D, Wilson K. Trends in Incidence of Nicotine Use Disorder Among Adolescents in the Pediatric Hospital, 2012-2019. Hosp Pediatr. 2021;11: 25–29. doi:10.1542/hpeds.2020-0183	No	Wrong outcomes	Full text screening
457	Matsumoto S, Traber MG, Leonard SW, Choi J, Fang X, Maishan M, et al. Aerosolized vitamin E acetate causes oxidative injury in mice and in alveolar macrophages. Am J Physiol Lung Cell Mol Physiol. 2022;322: L771–L783. doi:10.1152/ajplung.00482.2021	No	Evaluated effects of cannabis vapour exposure only	Full text screening
458	Mattingly DT, Cook S, Hirschtick JL, Patel A, Arenberg DA, Barnes GD, et al. Longitudinal associations between exclusive, dual, and polytobacco use and asthma among US youth. Prev Med. 2023;171: 107512. doi:10.1016/j.ypmed.2023.107512	No	Publication duplicate	Full text screening
459	McCabe AJ, Fitzgerald N, Striley C, Cottler L. E-cigarette Use Among Community-Recruited Adults with a History of Asthma in North Central Florida. J Community Health. 2023;48: 1010–1014. doi:10.1007/s10900-023-01256-y	No	Wrong outcomes	Full text screening
460	McCubbin A, Wiggins A, Barnett J, Ashford K. Perceptions, Characteristics, and Behaviors of Cigarette and Electronic Cigarette Use among Pregnant Smokers. Womens Health Issues. 2020;30: 221–229. doi:10.1016/j.whi.2020.03.006	No	Wrong outcomes	Full text screening
461	Mena J.E.M., Hsu H.-W., Arechavala R.J., Hasen I., Ting A., Bliss B., et al. Inflammatory gene expression changes and immune system depression in rats exposed to a single 4-HR exposure to electronic cigarette vapor. Circulation. 2021;144. doi:10.1161/circ.144.suppl-1.12450	No	Non-peer reviewed literature	Full text screening
462	Mikhail S., Savko C., Bose P., Sussman M. Vaping and Cardiopulmonary Fitness: Consequences of Aerobic Exercise. J Biol Chem. 2023;299: S474. doi:10.1016/j.jbc.2023.103892	No	Non-peer reviewed literature	Full text screening
463	Millan Cotto H., Matz J., Farra Y., Bellini C., Oakes J.M. Characterization of Parenchymal Structural and Functional Changes Due to Chronic JUUL Electronic Cigarette Aerosol Exposure in Apoe- /-Mice. Am J Respir Crit Care Med. 2022;205. doi:10.1164/ajrccm-conference.2022.205.1_MeetingAbstracts.A3649	No	Non-peer reviewed literature	Full text screening
464	Mohammadi L., Han D.D., Fang X., Xu F., Derakhshandeh R., Hellman J., et al. Increased pulmonary microvascular endothelial permeability by e-cigarette aerosol: The pathway from lung to peripheral blood vessels. Circulation. 2021;144. doi:10.1161/circ.144.suppl-1.11496	No	Non-peer reviewed literature	Full text screening
465	Molony R., Wu C.-H., Lee Y.-F. Cigarette smoke and e-liquid exposure drive the stress-induced production of pro-oncogenic extracellular vesicles by bladder cancer cells. J Urol. 2021;206: e659. doi:10.1097/JU.0000000000002047.10	No	Non-peer reviewed literature	Full text screening
466	Molony R., Wu C.-H., Lee Y.-F. E-LIQUID EXPOSURE INDUCES THE RELEASE OF PRO-ONCOGENIC EXTRACELLULAR VESICLES FROM BLADDER CANCER CELLS THAT PROMOTE THE MALIGNANT TRANSFORMATION OF RECIPIENT UROTHELIAL CELLS. J Urol. 2023;209: e184. doi:10.1097/JU.0000000000003234.06	No	Non-peer reviewed literature	Full text screening
467	Mori K.M., McElroy J.P., Weng D.Y., Chung S., Reisinger S.A., Ying K.L., et al. Lung mitochondrial DNA copy number variations: E-cig users, smokers, and never-smokers. Cancer Res. 2021;81. doi:10.1158/1538-7445.AM2021-751	No	Non-peer reviewed literature	Full text screening
468	Moussa C., Mahmoud N., Rouis H., Khattab A., Zendah I., Maalej S. E-cigarette dependence in former smoker: A Tunisian survey. Tunis Med. 2023;101: 580–584.	No	Non-peer reviewed literature	Full text screening
469	Mueller D., barounis D., Fonseca X. VAPING CAN BREAK YOUR HEART. Chest. 2021;160: A977. doi:10.1016/j.chest.2021.07.910	No	Non-peer reviewed literature	Full text screening
470	Mulorz J, Spin JM, Mulorz P, Wagenhäuser MU, Deng A, Mattern K, et al. E-cigarette exposure augments murine abdominal aortic aneurysm development: role of Chil1. Cardiovasc Res. 2023;119: 867–878. doi:10.1093/cvr/cvac173	No	Publication duplicate	Full text screening
471	Nardone N, Helen GS, Addo N, Meighan S, Benowitz NL. JUUL electronic cigarettes: Nicotine exposure and the user experience. Drug Alcohol Depend. 2019;203: 83–87. doi:10.1016/j.drugalcdep.2019.05.019	No	Qualitative study	Full text screening

472	Ng DQ, Ritt-Olson A, Freyer DR, Miller KA, Thomas SM, Milam J, et al. Substance Use Among Young Adult Survivors of Childhood Cancer With Cognitive Impairment: An Analysis of the Project Forward Cohort. JCO Oncol Pract. 2022; OP2200458. doi:10.1200/OP.22.00458	No	Evaluated effects of cannabis vapour exposure only	Full text screening
473	Nguyen M., Thompson G. A rare case of vaping-induced spontaneous pneumomediastinum. Respirology. 2023;28: 245. doi:10.1111/resp.14460	No	Non-peer reviewed literature	Full text screening
474	Nguyen S., Harb A., Luna R., Arriaga K., Marshall J., Abraham A., et al. Electronic Nicotine Delivery Systems Elevate Oxidative Stress and Accelerate Fibrosis Within the Mouse Corpora Cavernosa. J Sex Med. 2021;18: S24. doi:10.1016/j.jsxm.2021.01.112	No	Non-peer reviewed literature	Full text screening
475	Ni F., Ogura T., Lin W. Electrophysiological assessment of sensory irritation induced by flavors in electronic cigarette liquids in the mouse nasal respiratory epithelium. Chem Senses. 2019;44: e13. doi:10.1093/chemse/bjz035	No	Non-peer reviewed literature	Full text screening
476	Noel A., Johnson T., Penn A. Altered lung function is an early sensitive indicator of lung damage following 6 months of electronic-cigarette aerosol exposure in adult male mice. Am J Respir Crit Care Med. 2021;203. doi:10.1164/ajrccm-conference.2021.203.1_MeetingAbstracts.A3102	No	Non-peer reviewed literature	Full text screening
477	NWAY N.W.A.Y., GHIMIRE B., CHEN V., CHOWDHURY T., SUBEDI S., G. PATA R. A RARE CASE OF SPONTANEOUS PNEUMOMEDIASTINUM SECONDARY TO VAPING. Chest. 2023;164: A3499–A3500. doi:10.1016/j.chest.2023.07.2273	No	Non-peer reviewed literature	Full text screening
478	Osuji N., Uddin S.M.I., Obisesan O.H., Osei A., Erhabor J., Boakye E., et al. ASSOCIATION BETWEEN SOLE E-CIGARETTE USE AND ENDOTHELIAL FUNCTION: THE VAPORSENDOTHELIAL FUNCTION STUDY (VAPORS-E). J Am Coll Cardiol. 2022;79: 1600. doi:10.1016/S0735-1097%2822%2902591-8	No	Non-peer reviewed literature	Full text screening
479	PARK C., RODRIGUEZ CORDERO A., JEE AH RHEE C. THE FORGOTTEN LUNG DISEASE OF YOUNG ADULTS: EVALI STRIKES BACK. Chest. 2023;164: A3397. doi:10.1016/j.chest.2023.07.2209	No	Non-peer reviewed literature	Full text screening
480	Park K., Advani I.N., Masso Silva J.A., Pham J., Crotty Alexander L.E. The Effects of E-cigarette Use, Cigarette Smoke and Dual-Use of Both on Murine Susceptibility to Streptococcal Pneumonia. Am J Respir Crit Care Med. 2022;205. doi:10.1164/ajrccm-conference.2022.205.1_MeetingAbstracts.A1210	No	Non-peer reviewed literature	Full text screening
481	Parms T.A., Valverde R., Park-Lee E., Graham-Glover B.S., Cunningham C.S., Sawdey M.D., et al. Symptoms of tobacco dependence among middle and high school tobacco users - Data from the 2019-2020 National Youth Tobacco Survey. Addict Behav. 2022;137: 107537. doi:10.1016/j.addbeh.2022.107537	No	Publication duplicate	Full text screening
482	PATEL G.I.N.A., HARISH V.K., BAPAT N.E.H.A., SLACK D.F. AN UNUSUAL PRESENTATION OF ANTI-GBM DISEASE. Chest. 2023;164: A5643–A5644. doi:10.1016/j.chest.2023.07.3643	No	Non-peer reviewed literature	Full text screening
483	Perez Gandara B., Perez Perez J.L., Goldenberg H.A., Bramante A., Foronjy R.F., Abdoulaye D., et al. Nicotine in e-cigarettes dysregulates interferon beta, tumor necrosis factor alpha, and matrix metalloproteinase 12 expression, without effecting respiratory syncytial virus virulence in mice. Am J Respir Crit Care Med. 2021;203. doi:10.1164/ajrccm-conference.2021.203.1_MeetingAbstracts.A3104	No	Non-peer reviewed literature	Full text screening
484	Phandthong R., Wong M., Song A., Martinez T., Talbot P. Does Vaping Increase the Likelihood of SARS-CoV-2 Infection? Paradoxically Yes and No. bioRxiv. 2022. doi:10.1101/2022.09.09.507373	No	Non-peer reviewed literature	Full text screening
485	Phandthong R., Wong M., Song A., Martinez T., Talbot P. New Insights into How JUULTM Electronic Cigarette Aerosols and Aerosol Constituents Affect SARS-CoV-2 Infection of Human Bronchial Epithelial Cells. bioRxiv. 2022. doi:10.1101/2022.08.23.505031	No	Non-peer reviewed literature	Full text screening
486	Pienkowski M, Chaiton M, Dubray J, Schwartz R. E-Cigarette Dependence in Youth. Nicotine Tob Res. 2022;24: 1089–1094. doi:10.1093/ntr/ntab268	No	Wrong outcomes	Full text screening
487	Piper ME, Baker TB, Benowitz NL, Smith SS, Jorenby DE. E-cigarette Dependence Measures in Dual Users: Reliability and Relations With Dependence Criteria and E-cigarette Cessation. Nicotine Tob Res. 2020;22: 756–763. doi:10.1093/ntr/ntz040	No	Wrong outcomes	Full text screening
488	Poindexter M.E., Li X., Pinkerton K.E. Biological Effects of Progressive Exposure to Vapor from a 3rd Generation E-Cigarette Device. Am J Respir Crit Care Med. 2021;203. doi:10.1164/ajrccm-conference.2021.TP109	No	Non-peer reviewed literature	Full text screening
489	Polisetty L., Amoah K., Singh Rahi M., Wolff A. A CASE OF SEVERE HYPOXEMIA AND HEMOPTYSIS SECONDARY TO E-CIGARETTE AND VAPING PRODUCT USE-ASSOCIATED LUNG INJURY IN COVID-19 ERA. Chest. 2021;160: A1174. doi:10.1016/j.chest.2021.07.1077	No	Non-peer reviewed literature	Full text screening
490	Polosa R, Morjaria JB, Prosperini U, Busà B, Pennisi A, Gussoni G, et al. Health outcomes in COPD smokers using heated tobacco products: a 3-year follow-up. Intern Emerg Med. 2021;16: 687–696. doi:10.1007/s11739-021-02674-3	No	Evaluated effects of heated tobacco products exposure	Full text screening
491	Price SN, Palmer AM, Fucito LM, Graboyes EM, Baker NL, Rojewski AM, et al. Tobacco use and cancer-related symptom burden: Analysis of the US Population Assessment of Tobacco and Health Study. Cancer. 2023;129: 2385–2394. doi:10.1002/cncr.34746	No	Wrong outcomes	Full text screening
492	Pruitt S.E., McKee S. A can't-miss diagnosis at an unusual age: An adolescent withgranulomatosis with polyangiitis. Pediatrics. 2021;147: 611. doi:10.1542/peds.147.3-MeetingAbstract.611-a	No	Non-peer reviewed literature	Full text screening
493	PURI C., SINGH S., SLACK D. VAPING-INDUCED PLEURAL EFFUSIONS. Chest. 2022;162: A1381. doi:10.1016/j.chest.2022.08.1166	No	Non-peer reviewed literature	Full text screening
494	Puthumana R., Ahmad H., Gohar A. EVALI IN THE ERA OF COVID-19. Chest. 2021;160: A2385. doi:10.1016/j.chest.2021.07.2063	No	Evaluated effects of cannabis vapour exposure only	Full text screening
495	Putri N.L.P.D.A., Wiryawan N., Aryadana W., Widiana I.G.R., Arijana I.G.K.N., Dewangga I.M.S.Y. The effects of electric cigarette smoke and conventional cigarette smoke exposure on the increase of atherogenic marker: Leptin receptor (mRNAObR) and aortic calcification in mice. Eur Heart J Suppl. 2021;23: F14. doi:10.1093/eurheartjsupp/suab122.066	No	Non-peer reviewed literature	Full text screening
496	Qiu H., Zhang H., Derakhshandeh R., Wang X., Wilson E., Rao P., et al. Increased susceptibility to arrhythmias, hypertension, and hypertrophy in a rat model of exposure to electronic, heat-not-burn, or conventional cigarettes, or marijuana. Circulation. 2021;144. doi:10.1161/circ.144.suppl_1.9922	No	Non-peer reviewed literature	Full text screening
497	Rahman AU, Mohamed MHN, Jamshed S, Mahmood S, Iftikhar Baig MA. The Development and Assessment of Modified Fagerstrom Test for Nicotine Dependence Scale among Malaysian Single Electronic Cigarette Users. J Pharm Bioallied Sci. 2020;12: S671–S675. doi:10.4103/jpbs.JPBS_245_19	No	Wrong outcomes	Full text screening
498	Ramakrishnan R., Murray C., Roca Garcia M.R. Teenager with history of vaping diagnosed with e-cigarette or vaping product useassociated lung injury (Evali). J Investig Med. 2021;69: 471. doi:10.1136/jim-2021-SRMC.134	No	Non-peer reviewed literature	Full text screening
499	Ramalingam A., Kucera C., Merchant M., Bhatnagar A., Carll A.P. E-Cigarettes Induce Persistent Cardiac Autonomic Dysfunction and Molecular Signatures of Dilated and Arrhythmogenic Cardiomyopathy. Circulation. 2023;148. doi:10.1161/circ.148.suppl_1.19102	No	Non-peer reviewed literature	Full text screening
500	Ramanathan G., Johnson R., Chen J.H., Huang A.J.-E., Bliss B., Kleinman M.T., et al. Cigarette smoke and e-cigarette aerosols lead to clonal expansion of tet2-/- and dnmt3ar878h cells in vivo. Blood. 2021;138: 2167. doi:10.1182/blood-2021-151322	No	Non-peer reviewed literature	Full text screening
501	Raol K., Pothuru S., Altamimi A., Raheem A., Rakholiya J., Jain S., et al. E-CIGARETTE SMOKING IS LINKED WITH CARDIOVASCULAR DISORDERS. Chest. 2021;160: A2459–A2460. doi:10.1016/j.chest.2021.07.2125	No	Non-peer reviewed literature	Full text screening
502	Rapp JL, Alpert N, Flores RM, Taioli E. Serum cotinine levels and nicotine addiction potential of e-cigarettes: an NHANES analysis. Carcinogenesis. 2020;41: 1454–1459. doi:10.1093/carcin/bgaa015	No	Wrong outcomes	Full text screening

503	Reddy A, Jenssen BP, Chidambaram A, Yehya N, Lindell RB. Characterizing e-cigarette vaping-associated lung injury in the pediatric intensive care unit. Pediatr Pulmonol. 2021;56: 162–170. doi:10.1002/ppul.25086	No	Evaluated effects of cannabis vapour exposure only	Full text screening
504	Reed BW, Doran N, Courtney KE. Associations between nicotine product use and craving among stable daily and non-daily users. Addict Behav. 2023;146: 107803. doi:10.1016/j.addbeh.2023.107803	No	Wrong outcomes	Full text screening
505	REYNOLDS C., SEIFERT S.J., REIMER V. AN UNUSUAL CASE OF PULMONARY MYCOBACTERIUM ABSCESSUS ASSOCIATED WITH VAPING IN A YOUNG IMMUNOCOMPETENT FEMALE. Chest. 2023;164: A1378–A1379. doi:10.1016/j.chest.2023.07.963	No	Non-peer reviewed literature	Full text screening
506	Ross C.S., Pae V., Henehan E.R., Zhang T.C., Siegel M.B. Vaping Flavors Associated With Vaping Dependence Symptoms Among Adolescents: A Pilot Study Using Vape Factor Fast Find (VF3). J Adolesc Health. 2019;64: S79. doi:10.1016/j.jadohealth.2018.10.170	No	Non-peer reviewed literature	Full text screening
507	Royer C.M., Miller L.A. E-Cigarette Vaping Causes Lasting Suppression of Ciliated Primary Tracheobronchial Epithelia. Am J Respir Crit Care Med. 2021;203. doi:10.1164/ajrccm-conference.2021.TP109	No	Non-peer reviewed literature	Full text screening
508	Ruran HB, Maciag MC, Murphy SE, Phipatanakul W, Hauptman M. Cross-sectional study of urinary biomarkers of environmental tobacco and e-cigarette exposure and asthma morbidity. Ann Allergy Asthma Immunol. 2022;129: 378–380. doi:10.1016/j.anai.2022.06.001	No	Non-peer reviewed literature	Full text screening
509	Saha P., Mukherjee I., Jain S., Zeki A.A., Naidu V.G.M., Sharma P. Effect of Dual IQOS and Cigarette Smoke Exposure on Airway Epithelial Cell Stress Pathways and Inflammation: Implications for Lung Health and Respiratory Disease Pathogenesis. Am J Respir Crit Care Med. 2022;205. doi:10.1164/ajrccm-conference.2022.205.1_MeetingAbstracts.A3266	No	Non-peer reviewed literature	Full text screening
510	SALAM K.B., PRABHAKAR A.P., AGARWAL A.J.A.Y. GOING UP IN SMOKE: E-CIGARETTE-ASSOCIATED CARDIAC ARRHYTHMIA. Chest. 2023;164: A6402. doi:10.1016/j.chest.2023.07.4127	No	Non-peer reviewed literature	Full text screening
511	Sana M., Hagblom F., Hand M., Pezzino A., Wen X. Maternal Cigarette and Electronic Cigarette Use before Pregnancy Associated with the Risk of Hypertension and Gestational Hypertension. Obstet Gynecol. 2023;141: 83S. doi:10.1097/01.AOG.0000930992.06174.be	No	Non-peer reviewed literature	Full text screening
512	SCHUH S., BAJWA S., TUN AUNG T.U.N., BOYD T., REED A., WINTERTON B.M., et al. PERICARDITIS AS A MANIFESTATION OF E-CIGARETTE- OR VAPING-ASSOCIATED LUNG ILLNESS. Chest. 2023;164: A6252–A6253. doi:10.1016/j.chest.2023.07.4022	No	Non-peer reviewed literature	Full text screening
513	SCHUH S., M PATIL S., B CHAIRMAN D. DIFFUSE ALVEOLAR HEMORRHAGE AS A FATAL PRESENTATION OF E-CIGARETTE OR VAPING-ASSOCIATED LUNG ILLNESS. Chest. 2022;162: A1176. doi:10.1016/j.chest.2022.08.938	No	Non-peer reviewed literature	Full text screening
514	Scieszka D, Byrum SD, Mackintosh SG, Madison M, Knight J, Campen MJ, et al. Subchronic Electronic Cigarette Exposures Have Overlapping Protein Biomarkers with Chronic Obstructive Pulmonary Disease and Idiopathic Pulmonary Fibrosis. Am J Respir Cell Mol Biol. 2022;67: 503–506. doi:10.1165/rcmb.2021-0482LE	No	Non-peer reviewed literature	Full text screening
515	Seeliger B., Pape T., Horn P., Stahl K., Schmidt J.J., Welte T., et al. Exposure to nicotine containing e-cigarette vapor leads to endothelial barrier dysfunction and increased susceptibility to LPS in-vitro. Eur Respir J. 2022;60. doi:10.1183/13993003.congress-2022.2327	No	Non-peer reviewed literature	Full text screening
516	Seneviratne C.P., Tewari P., Hernandez N., Koltz B., Safi F. Evali masquerading as COVID-19: A case report. Am J Respir Crit Care Med. 2021;203. doi:10.1164/ajrccm-conference.2021.203.1_MeetingAbstracts.A2437	No	Non-peer reviewed literature	Full text screening
517	Shafie-Khorassani F, Piper ME, Jorenby DE, Baker TB, Benowitz NL, Hayes-Birchler T, et al. Associations of demographics, dependence, and biomarkers with transitions in tobacco product use in a cohort of cigarette users and dual users of cigarettes and e-cigarettes. Nicotine Tob Res. 2023;25: 462–469. doi:10.1093/ntr/ntac207	No	Wrong outcomes	Full text screening
518	Sharapova S, Reyes-Guzman C, Singh T, Phillips E, Marynak KL, Agaku I. Age of tobacco use initiation and association with current use and nicotine dependence among US middle and high school students, 2014-2016. Tob Control. 2020;29: 49–54. doi:10.1136/tobaccocontrol-2018-054593	No	Wrong outcomes	Full text screening
519	Shi H, Leventhal AM, Wen Q, Ossip DJ, Li D. Sex Differences in the Association of E-cigarette and Cigarette Use and Dual Use With Self-Reported Hypertension Incidence in US Adults. Nicotine Tob Res. 2023;25: 478–485. doi:10.1093/ntr/ntac170	No	Publication duplicate	Full text screening
520	Shi J, Fu R, Hamilton H, Chaiton M. A machine learning approach to predict e-cigarette use and dependence among Ontario youth. Health Promot Chronic Prev Can. 2022;42: 21–28. doi:10.24095/hpcdp.42.1.04	No	Wrong outcomes	Full text screening
521	Shi J., Dai W., Carreno J., Kleinman M.T., Herman D., Johnson R., et al. The Effect of Electronic Cigarette Exposure With Nicotine on Expression of Inflammatory Genes and Blood Parameters in a Chronic Myocardial Infarction Model. Circulation. 2023;148. doi:10.1161/circ.148.suppl_1.14730	No	Non-peer reviewed literature	Full text screening
522	Shi J., Dai W., Chavez J.N., Carreno J., Zhao L., Kleinman M.T., et al. One acute exposure to e-cigarette smoke using various heating elements and power levels induces pulmonary inflammation. Circulation. 2021;144. doi:10.1161/circ.144.suppl-1.10299	No	Non-peer reviewed literature	Full text screening
523	Siddiqui S., Gorti A. A case of e-cigarette/vapingassociated lung injury in the era of COVID-19. Crit Care Med. 2022;50: 140. doi:10.1097/01.ccm.0000807556.18501.7d	No	Non-peer reviewed literature	Full text screening
524	Simon P, Buta E, Jackson A, Camenga DR, Kong G, Morean ME, et al. The first nicotine product tried is associated with current multiple nicotine product use and nicotine dependence among a nationally representative sample of U.S. youths. Prev Med. 2023;169: 107437. doi:10.1016/j.ypmed.2023.107437	No	Wrong outcomes	Full text screening
525	Snoderly H., Bennewitz M. Acute Exposure to E-Cigarette Vapor Promotes Neutrophil-Platelet Aggregation in Murine Pulmonary Microvasculature. FASEB J. 2021;35. doi:10.1096/fasebj.2021.35.S1.05039	No	Non-peer reviewed literature	Full text screening
526	Sobierajski T, Surma S, Romanczyk M, Banach M, Oparil S. Knowledge of Primary Care Patients Living in the Urban Areas about Risk Factors of Arterial Hypertension. Int J Environ Res Public Health. 2023;20. doi:10.3390/ijerph20021250	No	Wrong outcomes	Full text screening
527	Soji-Ayoade D., Ndebbio U., Kadariya K., Kyei-Nimako E. EARLY SECONDARY SPONTANEOUS PNEUMOTHORAX IN A CAUCASIAN FEMALE WITH PAST PRIMARY SPONTANEOUS PNEUMOTHORAX. Chest. 2021;160: A1698. doi:10.1016/j.chest.2021.07.1545	No	Non-peer reviewed literature	Full text screening
528	Soule EK, Lee JGL, Egan KL, Bode KM, Desrosiers AC, Guy MC, et al. "I cannot live without my vape": Electronic cigarette user-identified indicators of vaping dependence. Drug Alcohol Depend. 2020;209: 107886. doi:10.1016/j.drugalcdep.2020.107886	No	Qualitative study	Full text screening
529	Stamm P., Mihalikova D., Czarnowski A., Kuntic M., Kuntic I., Hahad O., et al. The cardiovascular effects of alternative tobacco products and the role of phagocytic NADPH oxidase (NOX-2). Free Radic Biol Med. 2023;201: 37. doi:10.1016/j.freeradbiomed.2023.03.152	No	Non-peer reviewed literature	Full text screening
530	Stein J, Kay HE, Sites J, Pirzadeh A, Joyner BLJ, Darville T, et al. Electronic cigarette, or vaping, product use-associated lung injury (EVALI) in a patient with testicular cancer: A case report. Tumori. 2023; 3008916231172806. doi:10.1177/03008916231172806	No	Evaluated effects of cannabis vapour exposure only	Full text screening
531	Stiles M.F., Campbell L.R., Jin T., Graff D.W., Fant R.V., Henningfield J.E. Assessment of the abuse liability of three menthol Vuse Solo electronic cigarettes relative to combustible cigarettes and nicotine gum. Psychopharmacology (Berl). 2018;235: 2077–2086. doi:10.1007/s00213-018-4904-x	No	Wrong outcomes	Full text screening
532	Strong D.R., Pierce J.P., White M., Stone M.D., Abrams D.B., Glasser A.M., et al. Changes in Tobacco Dependence and Association with Onset and Progression of Use by Product Type from Wave 1 to Wave 3 of the Population Assessment of Tobacco and Health (PATH) Study. Nicotine Tob Res Off J Soc Res Nicotine Tob. 2022. doi:10.1093/ntr/ntac167	No	Retracted literature	Full text screening

533	Sussman M.A., Esquer C., Savko C., Shain G., Echeagaray O.E., Firouzi F., et al. Fundamentals of Vaping-Associated Pulmonary Injury Leading to Severe Respiratory Distress. Circ Res. 2021;129: e243. doi:10.1161/RES.0000000000000520	No	Publication duplicate	Full text screening
534	Sussman M.A., Savko C., Bose P., Rokaw S., Rieder A., Alani T., et al. Vaping and Cardiopulmonary Fitness: Consequences of Aerobic Exercise. Circ Res. 2022;131: e177. doi:10.1161/RES.0000000000000584	No	Non-peer reviewed literature	Full text screening
535	Szafran D, Gorig T, Vollstadt-Klein S, Grundinger N, Mons U, Lohner V, et al. Addictive Potential of e-Cigarettes as Reported in e-Cigarette Online Forums: Netnographic Analysis of Subjective Experiences. J Med Internet Res. 2023;25: e41669. doi:10.2196/41669	No	Qualitative study	Full text screening
536	Tackett A.P., Cho J., Whitted L., Harlow A., Vogel E., Cwalina S., et al. Examining Respiratory Symptoms Among Young Adults Using "Ice"- Hybrid E-Cigarette Flavors. Am J Respir Crit Care Med. 2022;205. doi:10.1164/ajrccm-conference.2022.205.1_MeetingAbstracts.A3856	No	Non-peer reviewed literature	Full text screening
537	Tattersall M.C., Hughey C.M., Piasecki T.M., Korcarz C.E., Hansen K., Fiore M.C., et al. Acute Effects of Nicotine-Containing Product Challenges on Cardiovascular and Autonomic Function Among Electronic Cigarette Vapers, Combustible Cigarette Smokers, and Controls: The CLUES Study. Circulation. 2022;146. doi:10.1161/circ.146.suppl_1.11674	No	Non-peer reviewed literature	Full text screening
538	Theriault P.-O., Carignan L., Lechasseur A., Tremblay F., Pineault M., Beaulieu M.-J., et al. Impact of vaping on tumor growth and metabolism. Can J Respir Crit Care Sleep Med. 2023;7: 31–32. doi:10.1080/24745332.2023.2214070	No	Non-peer reviewed literature	Full text screening
539	THOMAS S.H.O.N., CHANG J., FITTON K. A RARE CASE OF COMBINED SMALL-CELL LUNG CARCINOMA. Chest. 2023;164: A4336. doi:10.1016/j.chest.2023.07.2823	No	Non-peer reviewed literature	Full text screening
540	Torrez W.V.V., Grimaldo M.T., Clowers M.J., Yuan B., Del Aguila Soto S., Bouchelkia I., et al. Comparative effects of combustible cigarette versus electronic cigarette exposures on K-ras mutant lung cancer. Cancer Res. 2023;83. doi:10.1158/1538-7445.AM2023-649	No	Non-peer reviewed literature	Full text screening
541	Tremblay F., Pineault M., Theriault P.-O., Milad N., Lechasseur A., Routhier J., et al. Signs of Dendritic Cell Activation in the Lungs of Mice Exposed to Aerosols of Flavored Vaping Liquid. J Immunol. 2023;210. doi:10.4049/jimmunol.210.Supp.221.07	No	Non-peer reviewed literature	Full text screening
542	Tremblay F., Pineault M., Theriault P.-O., Milad N., Lechasseur A., Routhier J., et al. Signs of dendritic cell activation in the lungs of mice exposed to aerosols of flavored vaping liquid. Can J Respir Crit Care Sleep Med. 2023;7: 33. doi:10.1080/24745332.2023.2214070	No	Publication duplicate	Full text screening
543	TUBIG C., LIMPIN M.E. COMPARISON OF RESPIRATORY SYMPTOMS AMONG ADULTS WITH VS WITHOUT VAPING, SMOKING, AND DUAL USE: A CROSS-SECTIONAL STUDY IN A CALL CENTER IN QUEZON CITY. Chest. 2022;162: A2613. doi:10.1016/j.chest.2022.08.2135	No	Non-peer reviewed literature	Full text screening
544	Tucker MR, Laugesen M, Bullen C, Grace RC. Predicting Short-Term Uptake of Electronic Cigarettes: Effects of Nicotine, Subjective Effects, and Simulated Demand. Nicotine Tob Res. 2018;20: 1265–1271. doi:10.1093/ntr/ntx269	No	Wrong outcomes	Full text screening
545	Upadhyay P. A Stroke of Bad Luck. Pediatrics. 2022;149. Available: https://publications.aap.org/pediatrics/article/149/1MeetingAbstractsFebruary2022/736/186376/A-Stroke-of-Bad-Luck	No	Non-peer reviewed literature	Full text screening
546	Valentine GW, Hefner K, Jatlow PI, Rosenheck RA, Gueorguieva R, Sofuoglu M. Impact of E-cigarettes on Smoking and Related Outcomes in Veteran Smokers With Psychiatric Comorbidity. J Dual Diagn. 2018;14: 2–13. doi:10.1080/15504263.2017.1384877	No	Wrong outcomes	Full text screening
547	Veliz P.T., Evans-Polce R., McCabe S.E., McCabe V.V., Boyd C.J. Longitudinal changes in US adolescents' e-cigarette use and tobacco use disorder symptoms. J Addict Med. 2019;13: E11–E12. doi:10.1097/ADM.0000000000000548	No	Non-peer reviewed literature	Full text screening
548	VINAN VEGA M.N., YINGCHONCHAROEN P., NUGENT K.M., THONGPIYA J., EVANS A. SEVERE ACUTE RESPIRATORY DISTRESS SYNDROME SECONDARY TO UNFORTUNATE TRIAD: VAPING-INDUCED LUNG INJURY, RHINOVIRUS, AND MRSA PNEUMONIA. Chest. 2023;164: A1926–A1927. doi:10.1016/j.chest.2023.07.1324	No	Evaluated effects of cannabis vapour exposure only	Full text screening
549	Voos N, Kaiser L, Mahoney MC, Bradizza CM, Kozlowski LT, Benowitz NL, et al. Randomized within-subject trial to evaluate smokers' initial perceptions, subjective effects and nicotine delivery across six vaporized nicotine products. Addiction. 2019;114: 1236–1248. doi:10.1111/add.14602	No	Wrong outcomes	Full text screening
550	Wainer P, Decoud A, Migliore D, Taglapietra V, Gnocchi C, Lopez Macchi G, et al. [Vaping-induced lung injury]. Med B Aires. 2022;82: 151–153.	No	Evaluated effects of cannabis vapour exposure only	Full text screening
551	Wan Puteh SE, Mohd Ismail N, Md Isa Z, Ban AY-L. Exhaled Carbon Monoxide Level and Practices among Tobacco and Nicotine Adult Users in Klang Valley, Malaysia. Int J Environ Res Public Health. 2023;20. doi:10.3390/ijerph20054443	No	Wrong outcomes	Full text screening
552	Wang X., Qiu H., Han D.D., Tan K., Derakhshandeh R., Springer M.L. Adverse effects of smoking/vaping of several types of Tobacco products and Marijuana on cardiac function and platelet aggregation. Circulation. 2021;144. doi:10.1161/circ.144.suppl_1.9869	No	Non-peer reviewed literature	Full text screening
553	Warren KJ, Beck EM, Callahan SJ, Helms MN, Middleton E, Maddock S, et al. Alveolar macrophages from EVALI patients and e-cigarette users: a story of shifting phenotype. Respir Res. 2023;24: 162. doi:10.1186/s12931-023-02455-w	No	Evaluated effects of cannabis vapour exposure only	Full text screening
554	Wasserman E., Perosevic N. A CASE OF SEVERE E-CIGARETTE OR VAPING PRODUCT USE-ASSOCIATED LUNG INJURY IN THE TIME OF COVID-19. Chest. 2021;160: A1204. doi:10.1016/j.chest.2021.07.1106	No	Non-peer reviewed literature	Full text screening
555	Wen X, Xia T, Li R, Qiu H, Yu B, Zhang Y, et al. Trends in Electronic Cigarette Use Among US Adults With a History of Cardiovascular Disease. JAMA Netw Open. 2023;6: e2328962. doi:10.1001/jamanetworkopen.2023.28962	No	Wrong outcomes	Full text screening
556	West JC, Burt KB, Klemperer EM, Chen HL, Villanti AC. Latent Classes of Perceived Addictiveness Predict Marijuana, Alcohol, and Tobacco Use in Youth and Young Adults. Subst Use Misuse. 2023;58: 454–464. doi:10.1080/10826084.2023.2167497	No	Wrong outcomes	Full text screening
557	Wick K.D., Fang X., Simoneau C., Maishan M., Matsumoto S., Spottiswoode N., et al. Impact of Popular E Cigarette Pods on Human Alveolar Epithelial Type 2 Cells: Implications for Long Term Use. Am J Respir Crit Care Med. 2022;205. doi:10.1164/ajrccm-conference.2022.205.1_MeetingAbstracts.A3265	No	Non-peer reviewed literature	Full text screening
558	Wickramasinghe S., Haider Y., Ebaid S. Vaping induced non-specific interstitial pneumonia - A rarely described entity. Respirology. 2021;26: 496. doi:10.1111/resp.14150	No	Non-peer reviewed literature	Full text screening
559	Yingst J, Foulds J, Veldheer S, Cobb CO, Yen MS, Hrabovsky S, et al. Measurement of Electronic Cigarette Frequency of Use Among Smokers Participating in a Randomized Controlled Trial. Nicotine Tob Res. 2020;22: 699–704. doi:10.1093/ntr/nty233	No	Wrong outcomes	Full text screening
560	Zambrano I., Manocha U., Gong W., Tsai Y.-H., Zhu M., Herman M.A., et al. A murine MODEL of e-cigarettes exposure to evaluate the carcinogenic effects of vaping on the bladder urothelium. J Clin Oncol. 2022;40. doi:10.1200/JCO.2022.40.6_suppl.566	No	Non-peer reviewed literature	Full text screening
561	Zambrano I., Manocha U., Gong W., Tsai Y.-H., Zhu M., Herman M., et al. E-CIGARETTE EXPOSURE IN MICE PROMOTES CHANGES IN DNA METHYLATION OF THE BLADDER UROTHELIUM. J Urol. 2022;207: e912–e913. doi:10.1097/JU.0000000000002630.08	No	Publication duplicate	Full text screening
562	Zhuikova E., Durrant P., Macauley E., Goss H., Goldsmith N., Ioannides C., et al. Monitoring the transition from cigarette smoking to electronic cigarette use: Nicotine intake, psychometric, and clinical outcomes. Br J Clin Pharmacol. 2019;85: 1627–1628. doi:10.1111/bcp.13937	No	Non-peer reviewed literature	Full text screening

563	Barnes JM, Ali M. E-Cigarette or Vaping Associated Lung Injury: Evolving Threat to Healthy Teens. Med. 2021;74: 318–321.	No	Publication before July 2021	Title and abstract screening
564	Bowler RP, Hansel NN, Jacobson S, Graham Barr R, Make BJ, Han MK, et al. Electronic Cigarette Use in US Adults at Risk for or with COPD: Analysis from Two Observational Cohorts. J Gen Intern Med. 2017;32: 1315–1322. doi:10.1007/s11606-017-4150-7	No	Publication before July 2021	Title and abstract screening
565	Chidharla A., Rabbani R., Agarwal K., Abdelwahed S., Bhandari R., Manaktala P.S., et al. 1825P Prevalence of cancer among e-cigarette smokers compared to non-smokers: A retrospective cross-sectional survey study of NHANES-CDC. Ann Oncol. 2021;32: S1236. doi:10.1016/j.annonc.2021.08.713	No	Non-peer reviewed literature	Title and abstract screening
566	Hasan K.M., Munoz A., Tumoyan H., Parveen M., Espinoza-Derout J., Shao X.M., et al. Adverse effects of fetal exposure of electronic-cigarettes and high-fat diet on male neonatal hearts. Exp Mol Pathol. 2021;118: 104573. doi:10.1016/j.yexmp.2020.104573	No	Publication before July 2021	Title and abstract screening
567	Kirshenbaum AP, Hughes JR. Reinforcement enhancement by nicotine: A novel abuse-liability assessment of e-cigarettes in young adults. Exp Clin Psychopharmacol. 2022;30: 959–972. doi:10.1037/pha0000496	No	Publication before July 2021	Title and abstract screening
568	Lallai V, Manca L, Fowler CD. E-cigarette vape and lung ACE2 expression: Implications for coronavirus vulnerability. Env Toxicol Pharmacol. 2021;86: 103656. doi:10.1016/j.etap.2021.103656	No	Publication before July 2021	Title and abstract screening
569	Majmundar A, Allem JP, Cruz TB, Unger JB, Pentz MA. Monitoring Health Effects of Vaping Discussed on Twitter in 2018 and 2019. J Gen Intern Med. 2022;37: 673–675. doi:10.1007/s11606-021-06705-9	No	Publication before July 2021	Title and abstract screening
570	McClelland ML, Sesoko CS, MacDonald DA, Davis LM, McClelland SC. The Immediate Physiological Effects of E-Cigarette Use and Exposure to Secondhand E-Cigarette Vapor. Respir Care. 2021;66: 943–950. doi:10.4187/respcare.08596	No	Publication before July 2021	Title and abstract screening
571	O'Farrell HE, Brown R, Brown Z, Milijevic B, Ristovski ZD, Bowman RV, et al. E-cigarettes induce toxicity comparable to tobacco cigarettes in airway epithelium from patients with COPD. Toxicol Vitro. 2021;75: 105204. doi:10.1016/j.tiv.2021.105204	No	Publication before July 2021	Title and abstract screening
572	Reddy KP, Schwamm E, Kalkhoran S, Noubary F, Walensky RP, Rigotti NA. Respiratory Symptom Incidence among People Using Electronic Cigarettes, Combustible Tobacco, or Both. Am J Respir Crit Care Med. 2021;204: 231–234. doi:10.1164/rccm.202012-4441LE	No	Publication before July 2021	Title and abstract screening
573	Sahota A, Naidu S, Jacobi A, Giannarelli C, Woodward M, Fayad ZA, et al. Atherosclerosis inflammation and burden in young adult smokers and vapers measured by PET/MR. Atherosclerosis. 2021;325: 110–116. doi:10.1016/j.atherosclerosis.2021.03.021	No	Publication before July 2021	Title and abstract screening
574	Schneller LM, Quiñones Tavárez Z, Goniewicz ML, Xie Z, McIntosh S, Rahman I, et al. Cross-Sectional Association Between Exclusive and Concurrent Use of Cigarettes, ENDS, and Cigars, the Three Most Popular Tobacco Products, and Wheezing Symptoms Among U.S. Adults. Nicotine Tob Res. 2020;22: S76–S84. doi:10.1093/ntr/ntaa199	No	Publication before July 2021	Title and abstract screening
575	Ho M., Librach C. Smoke Without Fire: E-Liquid Exposure Impairs Cross-Talk Between Human Induced Pluripotent Stem Cell (hiPSC)-Derived Vascular Cell Types Via a Paracrine Mechanism. Am J Respir Crit Care Med. 2022;205. doi:10.1164/ajrccm-conference.2022.205.1_MeetingAbstracts.A5470	No	Not meeting inclusion criteria	Title and abstract screening
576	Zarcone G., Lenski M., Martinez T., Talahari S., Simonin O., Garcon G., et al. OS01-02 Impact of heated tobacco product, electronic cigarette and conventional cigarette emissions on the generation of oxidative stress, genetic and epigenetic lesions in human bronchial epithelial cells. Toxicol Lett. 2023;384: S58–S59. doi:10.1016/S0378-4274%2823%2900412-5	No	Not meeting inclusion criteria	Title and abstract screening
577	Abadi MH, Shamblen SR, Thompson K, Lipperman-Kreda S, Grube J, Richard BO, et al. Socio-temporal contextual and community factors associated with daily exclusive ENDS use and dual use with tobacco cigarettes among adolescent vapers: an ecological momentary assessment study. BMC Public Health. 2022;22: 2289. doi:10.1186/s12889-022-14787-1	No	Not meeting inclusion criteria	Title and abstract screening
578	Abbadi A., Alnahar J., Zoghoul S., Bsoul A., Alarood S., Al-Mistarehi A.-H., et al. Waterpipe Nicotine Dependence and Depressive Symptoms among Adolescent Waterpipe and Dual Users. J Environ Public Health. 2020;2020: 2364571. doi:10.1155/2020/2364571	No	Not meeting inclusion criteria	Title and abstract screening
579	Abbasi J. The American Heart Association Takes on Vaping. JAMA - J Am Med Assoc. 2020;323: 205–206. doi:10.1001/jama.2019.20781	No	Not meeting inclusion criteria	Title and abstract screening
580	Abbasi Y, Hout MV, Faragalla M, Itani L. Knowledge and Use of Electronic Cigarettes in Young Adults in the United Arab Emirates, Particularly during the COVID-19 Pandemic. Int J Env Res Public Health. 2022;19. doi:10.3390/ijerph19137828	No	Not meeting inclusion criteria	Title and abstract screening
581	Abbott AJ, Reibel YG, Arnett MC, Marka N, Drake MA. Oral and Systemic Health Implications of Electronic Cigarette Usage as Compared to Conventional Tobacco Cigarettes: A review of the literature. J Dent Hyg JDH. 2023;97: 21–35.	No	Not meeting inclusion criteria	Title and abstract screening
582	Abd-Elsayed A, Rupp A, D'Souza RS, Hussain N, Milam AJ, Strand N, et al. Interventional Pain Physician Burnout During the COVID-19 Pandemic: A Survey from the American Society of Pain and Neuroscience. Curr Pain Headache Rep. 2023;27: 259–267. doi:10.1007/s11916-023-01121-6	No	Not meeting inclusion criteria	Title and abstract screening
583	Abdallah B, Lee H, Weerakoon SM, Messiah SE, Harrell MB, Rao DR. Clinical manifestations of EVALI in adolescents before and during the COVID-19 pandemic. Pediatr Pulmonol. 2023;58: 949–958. doi:10.1002/ppul.26283	No	Not meeting inclusion criteria	Title and abstract screening
584	Abdallah B., Maple K., Rao D.R. Clinical presentation, management and short-term outcomes in adolescents hospitalized with electronic cigarette, or vaping, product-use associated lung injury (evali). Am J Respir Crit Care Med. 2021;203. doi:10.1164/ajrccm-conference.2021.203.1_MeetingAbstracts.A3198	No	Not meeting inclusion criteria	Title and abstract screening
585	Abdallah B., Rao D.R. Lung Disease Severity and Steroid Dosing in Adolescents with ECigarette, or Vaping Product, Use Associated Lung Injury (EVALI). Am J Respir Crit Care Med. 2022;205. doi:10.1164/ajrccm-conference.2022.205.1_MeetingAbstracts.A3857	No	Not meeting inclusion criteria	Title and abstract screening
586	Abdayem P., Veron L., Garcia G., Berkane K., Pradere P., Pudlarz T., et al. 1734P Early impact of a personalized lung cancer interception program for heavy smokers. Ann Oncol. 2023;34: S942. doi:10.1016/j.annonc.2023.09.2688	No	Not meeting inclusion criteria	Title and abstract screening
587	Abdelnabi M, Eshak N, Almaghraby A, Saleh Y, Gerges F, Ahmed A. Usefulness of statins in end-stage renal disease. Bayl Univ Med Cent Proc. 2021;34: 361–363. doi:10.1080/08998280.2021.1874774	No	Not meeting inclusion criteria	Title and abstract screening
588	Abdelsalam M., Tanami S.A., Naguib T. CANNABIS-INDUCED SUPERIOR MESENTRIC VEIN THROMBOSIS; AN UNUSUAL ETIOLOGY. J Gen Intern Med. 2023;38: S436–S437. doi:10.1007/s11606-023-08226-z	No	Not meeting inclusion criteria	Title and abstract screening
589	Abelia XA, Lesmana R, Goenawan H, Abdulah R, Barliana MI. Comparison impact of cigarettes and e-cigs as lung cancer risk inductor: a narrative review. Eur Rev Med Pharmacol Sci. 2023;27: 6301–6318. doi:10.26355/eurrev_202307_32990	No	Not meeting inclusion criteria	Title and abstract screening
590	Abellard A., Pappalardo A.A. Overview of severe asthma, with emphasis on pediatric patients: A review for practitioners. J Investig Med. 2021;69: 1297–1309. doi:10.1136/jim-2020-001752	No	Not meeting inclusion criteria	Title and abstract screening
591	Abeyesundere N, Osborn CC, Pike Moore S, Suratkal JP, Kaur M, Otieno C, et al. Substitution of flavored cigarillos with menthol cigarettes among young adults in the U.S. Drug Alcohol Depend. 2022;239: 109603. doi:10.1016/j.drugalcdep.2022.109603	No	Not meeting inclusion criteria	Title and abstract screening

592	Abi Nehme AM, Lou X, Yan X, Lee JH, Salloum RG. Transition to smoking cessation among dual cigarette and e-cigarette users in the population assessment of tobacco and health study, Waves 3 and 4 (2015-2017). Addict Behav. 2022;129: 107284. doi:10.1016/j.addbeh.2022.107284	No	Not meeting inclusion criteria	Title and abstract screening
593	Abo K., Wilson A. Human induced pluripotent stem cell-derived alveolar type 2 cells mature at air-liquid interface and respond to airborne stimuli. Eur Respir J. 2022;60. doi:10.1183/13993003.congress-2022.4648	No	Not meeting inclusion criteria	Title and abstract screening
594	Abo KM, Sainz de Aja J, Lindstrom-Vautrin J, Alysandratos KD, Richards A, Garcia-de-Alba C, et al. Air-liquid interface culture promotes maturation and allows environmental exposure of pluripotent stem cell-derived alveolar epithelium. JCI Insight. 2022;7. doi:10.1172/jci.insight.155589	No	Not meeting inclusion criteria	Title and abstract screening
595	Abou-Assali O., Chidipi B., Reiser M., Chang M., Calcul L., Noujaim S.F. BS-454195-4 MITOCHONDRIAL REMODELING MEDIATES CARDIAC ELECTROPHYSIOLOGICAL INJURY IN INHALATION EXPOSURE TO FLAVORED ELECTRONIC NICOTINE DELIVERY SYSTEMS. Heart Rhythm. 2023;20: S8. doi:10.1016/j.hrthm.2023.03.073	No	Not meeting inclusion criteria	Title and abstract screening
596	Abouassali O, Chang M, Chidipi B, Martinez JL, Reiser M, Kanithi M, et al. In vitro and in vivo cardiac toxicity of flavored electronic nicotine delivery systems. Am J Physiol Heart Circ Physiol. 2021;320: H133–H143. doi:10.1152/ajpheart.00283.2020	No	Not meeting inclusion criteria	Title and abstract screening
597	Abram A, Zeller M. The U.S. Food and Drug Administration's New Plan to Combat Tobacco's Staggering Public Health Toll. Ann Am Thorac Soc. 2019;16: 40–43. doi:10.1513/AnnalsATS.201807-474PS	No	Not meeting inclusion criteria	Title and abstract screening
598	Abrams D.B., Glasser A.M., Villanti A.C., Pearson J.L., Rose S., Niaura R.S. Re: Disregarding the impact of nicotine on the developing brain when evaluating costs and benefits of noncombustible nicotine products. Prev Med. 2019;120: 158–159. doi:10.1016/j.ypmed.2018.12.027	No	Not meeting inclusion criteria	Title and abstract screening
599	Abrams DB, Glasser AM, Villanti AC, Pearson JL, Rose S, Niaura RS. Managing nicotine without smoke to save lives now: Evidence for harm minimization. Prev Med. 2018;117: 88–97. doi:10.1016/j.ypmed.2018.06.010	No	Not meeting inclusion criteria	Title and abstract screening
600	ACTRN12617000288325. The use of a visualisation intervention to improve recovery behaviours following colorectal and gynaecological oncology surgery. 2017. Available: https://www.cochranelibrary.com/central/doi/10.1002/central/CN-02440012/full	No	Not meeting inclusion criteria	Title and abstract screening
601	ACTRN12617000849392. The QuitNic Study: a pilot study of electronic nicotine devices for smoking cessation with drug and alcohol clients. 2017. Available: https://www.cochranelibrary.com/central/doi/10.1002/central/CN-02443155/full	No	Not meeting inclusion criteria	Title and abstract screening
602	ACTRN12621000511831. Smokers', vapers', and non-users' reactions to vaping cues presented in virtual reality. 2021. Available: https://www.cochranelibrary.com/central/doi/10.1002/central/CN-02437748/full	No	Not meeting inclusion criteria	Title and abstract screening
603	ACTRN12621001349831. A phase I trial to study the effect of food on orally dosed IMG-7289 (Bomedemstat) in healthy adult volunteers. 2021. Available: https://www.cochranelibrary.com/central/doi/10.1002/central/CN-02348983/full	No	Not meeting inclusion criteria	Title and abstract screening
604	ACTRN12621001752853. The Sun-D Trial: the effect of high SPF sunscreen application on vitamin D. 2021. Available: https://www.cochranelibrary.com/central/doi/10.1002/central/CN-02379309/full	No	Not meeting inclusion criteria	Title and abstract screening
605	ACTRN12622000581763. Effects of tobacco health warnings and television advertisements to counter reassurance-based tobacco marketing among Australian smokers. 2022. Available: https://www.cochranelibrary.com/central/doi/10.1002/central/CN-02408137/full	No	Not meeting inclusion criteria	Title and abstract screening
606	ACTRN12622000614796. Randomised Study assessing reconstruction of the urinary tract via robotic (intracorporeal) or open (extracorporeal) method during removal of the bladder. 2022. Available: https://www.cochranelibrary.com/central/doi/10.1002/central/CN-02408160/full	No	Not meeting inclusion criteria	Title and abstract screening
607	ACTRN12622000643774. A Phase 1, Randomised, Double-Blind, Placebo-Controlled, First in Human Study of the Safety, Tolerability and Pharmacokinetics of AI-071 in Healthy Volunteers. 2022. Available: https://www.cochranelibrary.com/central/doi/10.1002/central/CN-02408179/full	No	Not meeting inclusion criteria	Title and abstract screening
608	ACTRN12622000755730. Australian Immunity Trial (Trial A): the effect of milk proteins on immune function in healthy young adults. 2022. Available: https://www.cochranelibrary.com/central/doi/10.1002/central/CN-02408239/full	No	Not meeting inclusion criteria	Title and abstract screening
609	ACTRN12622000757718. Australian Immunity Trial (Trial B): the effect of milk proteins on immune function in healthy young adults. 2022. Available: https://www.cochranelibrary.com/central/doi/10.1002/central/CN-02408241/full	No	Not meeting inclusion criteria	Title and abstract screening
610	ACTRN12622001199707. Using real-time feedback of patient-reported outcome measures to direct delivery of standard-of-care therapies in relapsed multiple myeloma. 2022. Available: https://www.cochranelibrary.com/central/doi/10.1002/central/CN-02460858/full	No	Not meeting inclusion criteria	Title and abstract screening
611	ACTRN12622001265763. A Phase 1, Placebo-Controlled, Single/Multiple Ascending Dose Study of DNTH103 Safety, Tolerability, Pharmacokinetics and Pharmacodynamics Following Intravenous and Subcutaneous Administration in Healthy Volunteers. 2022. Available: https://www.cochranelibrary.com/central/doi/10.1002/central/CN-02465906/full	No	Not meeting inclusion criteria	Title and abstract screening
612	ACTRN12623000022662. The OurFutures Vaping Program: a cluster randomised controlled trial to evaluate the efficacy of a school-based eHealth intervention to prevent e-cigarette use among adolescents. 2023. Available: https://www.cochranelibrary.com/central/doi/10.1002/central/CN-02515191/full	No	Not meeting inclusion criteria	Title and abstract screening
613	ACTRN12623000117617. A Multi-Centre, Randomized, Double-Blind, Placebo-Controlled, Single Ascending Dose Phase 1 Study to Evaluate the Safety, Tolerability and Pharmacokinetics of OLX72021 in Healthy Males with Androgenetic Alopecia. 2023. Available: https://www.cochranelibrary.com/central/doi/10.1002/central/CN-02520427/full	No	Not meeting inclusion criteria	Title and abstract screening
614	ACTRN12623000296639. A Phase 1, Randomised, Double-Blind, Placebo-Controlled, First-in-Human Study of the Safety, Tolerability and Pharmacokinetics of AXN-001 in Healthy Volunteers. 2023. Available: https://www.cochranelibrary.com/central/doi/10.1002/central/CN-02536465/full	No	Not meeting inclusion criteria	Title and abstract screening
615	ACTRN12623000440628. A Study of ELVN-002 in Healthy Adult Volunteers: part A. 2023. Available: https://www.cochranelibrary.com/central/doi/10.1002/central/CN-02554213/full	No	Not meeting inclusion criteria	Title and abstract screening
616	ACTRN12623000526673. A Randomised Controlled Trial of Cannabidiol (CBD) for the treatment of Cannabis Use Disorder (CUD). 2023. Available: https://www.cochranelibrary.com/central/doi/10.1002/central/CN-02563480/full	No	Not meeting inclusion criteria	Title and abstract screening
617	Adams M., Effertz T. Taxation of Deliberately Raised Addictive Potential in Cigarettes and New Nicotine Products. Sucht. 2020;66: 155–159. doi:10.1024/0939-5911/a000657	No	Not meeting inclusion criteria	Title and abstract screening
618	Adams ZW, Kwon E, Aalsma MC, Zapolski TCB, Dir A, Hulvershorn LA. Treatment of Adolescent e-Cigarette Use: Limitations of Existing Nicotine Use Disorder Treatment and Future Directions for e-Cigarette Use Cessation. J Am Acad Child Adolesc Psychiatry. 2021;60: 14–16. doi:10.1016/j.jaac.2020.07.007	No	Not meeting inclusion criteria	Title and abstract screening
619	Aday A.W., Matsushita K. Epidemiology of Peripheral Artery Disease and Polyvascular Disease. Circ Res. 2021;128: 1818–1832. doi:10.1161/CIRCRESAHA.121.318535	No	Not meeting inclusion criteria	Title and abstract screening
620	Adcock IM, Bhatt SP, Balkissoon R, Wise RA. The Use of Inhaled Corticosteroids for Patients with COPD Who Continue to Smoke Cigarettes: An Evaluation of Current Practice. Am J Med. 2022;135: 302–312. doi:10.1016/j.amjmed.2021.09.006	No	Not meeting inclusion criteria	Title and abstract screening

621	Addicott MA, Sutfin EL, Reynolds LM, Donny EC, Matich EK, Hsu PC. Biochemical validation of self-reported electronic nicotine delivery system and tobacco heaviness of use. Exp Clin Psychopharmacol. 2023;31: 715–723. doi:10.1037/pha0000604	No	Not meeting inclusion criteria	Title and abstract screening
622	Adelman WP. Dual Use and Dual Truths: Categorizing the Adolescent Electronic Cigarette User. J Adolesc Health. 2019;65: 167–168. doi:10.1016/j.jadohealth.2019.05.009	No	Not meeting inclusion criteria	Title and abstract screening
623	Adouard V, Menecier P, Chapalain F, Gelly B, Tamboloni JC. [Smoking prevalence among nursing students and nursing assistants in Mâcon: 2021 survey and evolution over 13 years]. Rev Mal Respir. 2022;39: 328–333. doi:10.1016/j.rmr.2022.02.058	No	Not meeting inclusion criteria	Title and abstract screening
624	Adriaens K, Van Gucht D, Van Lommel S, Baeyens F. Vaping during the COVID-19 lockdown period in Belgium. BMC Public Health. 2021;21: 1613. doi:10.1186/s12889-021-11637-4	No	Not meeting inclusion criteria	Title and abstract screening
625	Adriaens K., Van Gucht D., Baeyens F. Differences between dual users and switchers center around vaping behavior and its experiences rather than beliefs and attitudes. Int J Environ Res Public Health. 2018;15: 12. doi:10.3390/ijerph15010012	No	Not meeting inclusion criteria	Title and abstract screening
626	Adriaens K, Van Gucht D, Baeyens F. Differences between Dual Users and Switchers Center around Vaping Behavior and Its Experiences Rather than Beliefs and Attitudes. Int J Environ Res Public Health. 2017;15. doi:10.3390/ijerph15010012	No	Not meeting inclusion criteria	Title and abstract screening
627	ADUSUMILLI S., FRANCO R.E.N.E. SMOKING KILLS! SO CAN VAPING SAVE YOUR LIFE? Chest. 2023;164: A6397. doi:10.1016/j.chest.2023.07.4123	No	Not meeting inclusion criteria	Title and abstract screening
628	Adyanthaya S., Mehra I., Kundu S., Thomas B., Rao K., Westerman J. Heat injury: Heatstroke: Is ice the cause? Crit Care Med. 2022;50: 424. doi:10.1097/01.ccm.0000809756.55487.74	No	Not meeting inclusion criteria	Title and abstract screening
629	Adzrago D., Odame E., Dada O.M., Meyer C., Nriagu V., Paul T., et al. Effects of Covid-19 on Cardiometabolic Outcomes (diabetes, Hypertension, and Obesity). Circulation. 2022;145. doi:10.1161/circ.145.suppl_1.P171	No	Not meeting inclusion criteria	Title and abstract screening
630	Afolabi F, Rao DR. E-cigarettes and asthma in adolescents. Curr Opin Allergy Clin Immunol. 2023;23: 137–143. doi:10.1097/ACI.0000000000000891	No	Not meeting inclusion criteria	Title and abstract screening
631	Afolalu EF, Spies E, Bacso A, Clerc E, Abetz-Webb L, Gallot S, et al. Impact of tobacco and/or nicotine products on health and functioning: a scoping review and findings from the preparatory phase of the development of a new self-report measure. Harm Reduct J. 2021;18: 79. doi:10.1186/s12954-021-00526-z	No	Not meeting inclusion criteria	Title and abstract screening
632	Agaku I, Odani S, Vardavas C, Neff L. Self-Identified Tobacco Use and Harm Perceptions Among US Youth. Pediatrics. 2018;141. doi:10.1542/peds.2017-3523	No	Not meeting inclusion criteria	Title and abstract screening
633	Agaku IT, Egbe CO, Ayo-Yusuf OA. Circumvention of COVID-19-related restrictions on tobacco sales by the e-cigarette industry in South Africa and comparative analyses of heated tobacco product vs combustible cigarette volume sales during 2018-2020. Prev Med. 2021;148: 106526. doi:10.1016/j.ypmed.2021.106526	No	Not meeting inclusion criteria	Title and abstract screening
634	Agarwal S, Singh V, Sangwan A, Mishra S, Singh VP. Refractory cancer pain in young child at end-of-life: Can we alleviate the suffering? 2022. p. 268. Available: http://myaccess.library.utoronto.ca/login?url=https://search.ebscohost.com/login.aspx?direct=true&db=cin20&AN=158678577&site=ehost-live	No	Not meeting inclusion criteria	Title and abstract screening
635	Agbonlahor O, Mattingly DT, Rai J, Hart JL, McLeish AC, Walker KL. Differences in health care provider screening for tobacco use among youth in the United States: The National Youth Tobacco Survey, 2021. Prev Med. 2023;175: 107718. doi:10.1016/j.ypmed.2023.107718	No	Not meeting inclusion criteria	Title and abstract screening
636	Aghababaie M, Suresh V, McGlashan S, Tawhai M, Burrowes K. In silico prediction of e-cigarette aerosol particle transport and deposition within the airways. Annu Int Conf IEEE Eng Med Biol Soc IEEE Eng Med Biol Soc Annu Int Conf. 2023;2023: 1–4. doi:10.1109/EMBC40787.2023.10340944	No	Not meeting inclusion criteria	Title and abstract screening
637	Aglago EK, Mayén A-L, Knaze V, Freisling H, Fedirko V, Hughes DJ, et al. Dietary Advanced Glycation End-Products and Colorectal Cancer Risk in the European Prospective Investigation into Cancer and Nutrition (EPIC) Study. Nutrients. 2021;13: 3132–3132. doi:10.3390/nu13093132	No	Not meeting inclusion criteria	Title and abstract screening
638	Agoramoorthy G. Legal and health dilemmas challenging India's e-cigarette endorsement. Cancer. 2017;123: 3197. doi:10.1002/cncr.30836	No	Not meeting inclusion criteria	Title and abstract screening
639	Agraval H, Crue T, Schaunaman N, Numata M, Day BJ, Chu HW. Electronic Cigarette Exposure Increases the Severity of Influenza a Virus Infection via TRAIL Dysregulation in Human Precision-Cut Lung Slices. Int J Mol Sci. 2023;24. doi:10.3390/ijms24054295	No	Not meeting inclusion criteria	Title and abstract screening
640	Agulto A., Das K. Non-rheumatological cause of biopsy-proven pulmonaryvasculitis in ulcerative colitis. Pediatrics. 2021;147: 635. doi:10.1542/peds.147.3-MeetingAbstract.635-a	No	Not meeting inclusion criteria	Title and abstract screening
641	Aherrera A., Aravindakshan A., Jarmul S., Olmedo P., Chen R., Cohen J.E., et al. E-cigarette use behaviors and device characteristics of daily exclusive e-cigarette users in Maryland: Implications for product toxicity. Tob Induc Dis. 2021;18: 1–10. doi:10.18332/tid/128319	No	Not meeting inclusion criteria	Title and abstract screening
642	Ahmed AR, Etchey B, Ahmed M. Explosions, Burn Injuries and Adverse Health Effects of Electronic Nicotine Delivery Systems: A Review of Current Regulations and Future Perspectives. J Pharm Pharm Sci Publ Can Soc Pharm Sci Soc Can Sci Pharm. 2021;24: 462–474. doi:10.18433/jpps32242	No	Not meeting inclusion criteria	Title and abstract screening
643	Ahmed A, Shapiro D, Su J, Nelson LP. Vaping Cannabis Butane Hash Oil Leads to Severe Acute Respiratory Distress Syndrome-A Case of EVALI in a Teenager With Hypertrophic Cardiomyopathy. J Intensive Care Med. 2021;36: 617–621. doi:10.1177/0885066620941004	No	Not meeting inclusion criteria	Title and abstract screening
644	Ahuja NA, Kedia SK, Jiang Y, Ward KD, Pichon LC, Dillon PJ, et al. Factors Associated with E-Cigarette Quit Intention Among Adolescents in the United States. Subst Use Misuse. 2022;57: 2074–2084. doi:10.1080/10826084.2022.2130000	No	Not meeting inclusion criteria	Title and abstract screening
645	Ahuja NA, Kedia SK, Regmi S, Dillon PJ. Disparities in E-Cigarette Harm and Addiction Perceptions Among Adolescents in the United States: a Systematic Review of the Literature. J Racial Ethn Health Disparities. 2023. doi:10.1007/s40615-023-01553-1	No	Not meeting inclusion criteria	Title and abstract screening
646	Ahuja N, Kedia SK, Dillon PJ, Jiang Y, Yu X. Perception and intention of using less harmful and less addictive hypothetical modified risk tobacco products among never tobacco users in the united states. Addict Behav. 2021;122: 107016. doi:10.1016/j.addbeh.2021.107016	No	Not meeting inclusion criteria	Title and abstract screening
647	Ainscough TS, Mitchell A, Hewitt C, Horspool M, Stewart P, Ker S, et al. Investigating Changes in Patients' Smoking Behavior, Tobacco Dependence, and Motivation to Stop Smoking Following a "Smoke-Free" Mental Health Inpatient Stay: Results From a Longitudinal Survey in England. Nicotine Tob Res. 2021;23: 1010–1018. doi:10.1093/ntr/ntaa258	No	Not meeting inclusion criteria	Title and abstract screening
648	Akbar SA, Tomko RL, Salazar CA, Squeglia LM, McClure EA. Tobacco and cannabis co-use and interrelatedness among adults. Addict Behav. 2019;90: 354–361. doi:10.1016/j.addbeh.2018.11.036	No	Not meeting inclusion criteria	Title and abstract screening
649	Akbarpour E, Sadjadi A, Derakhshan MH, Roshandel G, Alimohammadian M. Gastric Cancer in Iran: An Overview of Risk Factors and Preventive Measures. Arch Iran Med AIM. 2021;24: 556–567. doi:10.34172/aim.2021.79	No	Not meeting inclusion criteria	Title and abstract screening

650	Akinola LS, Rahman Y, Ondo O, Cobb CO, Holt AK, Peace MR, et al. Impact of tobacco flavoring on oral nicotine consumption in C57BL/6J mice. Drug Alcohol Depend. 2022;241: 109685. doi:10.1016/j.drugalcdep.2022.109685	No	Not meeting inclusion criteria	Title and abstract screening
651	Akinyemi OA, Lipscombe CA, Nasef KE, Aaron S, Ranjit A. Disparities in Outcomes Following Ovarian Cancer: An Analysis of the Surveillance, Epidemiology, and End Results (SEER) Database (1998-2016) [A313]. Obstet Gynecol. 2022;139: 90S-90S. doi:10.1097/01.AOG.0000825572.48793.95	No	Not meeting inclusion criteria	Title and abstract screening
652	Akre C, Suris J-C. Adolescents and young adults' perceptions of electronic cigarettes as a gateway to smoking: a qualitative study in Switzerland. Health Educ Res. 2017;32: 448–454. doi:10.1093/her/cyx054	No	Not meeting inclusion criteria	Title and abstract screening
653	Al Hadidi S. The Use of Modified Surrogate End Points in Cancer Clinical Trials. Cancer Invest. 2022;40: 14–16. doi:10.1080/07357907.2021.2006683	No	Not meeting inclusion criteria	Title and abstract screening
654	Al-Hamdani M, Hopkins DB, Hardardottir A, Davidson M. Perceptions and Experiences of Vaping Among Youth and Young Adult E-Cigarette Users: Considering Age, Gender, and Tobacco Use. J Adolesc Health Off Publ Soc Adolesc Med. 2021;68: 787–793. doi:10.1016/j.jadohealth.2020.08.004	No	Not meeting inclusion criteria	Title and abstract screening
655	Al-Hamdani M, Manly E. Smoking cessation or initiation: The paradox of vaping. Prev Med Rep. 2021;22: 101363. doi:10.1016/j.pmedr.2021.101363	No	Not meeting inclusion criteria	Title and abstract screening
656	Al-Khlaiwi T, Alshammari H, Habib SS, Alobaid R, Alrumaih L, Almojel A, et al. High prevalence of lack of knowledge and unhealthy lifestyle practices regarding premature coronary artery disease and its risk factors among the Saudi population. BMC Public Health. 2023;23: 908. doi:10.1186/s12889-023-15834-1	No	Not meeting inclusion criteria	Title and abstract screening
657	Al-Mandhari A, Hammerich A, El-Awa F, Bettcher D, Mandil A. Full implementation of the WHO Framework Convention on Tobacco Control in the Eastern Mediterranean Region is the responsibility of all. East Mediterr Health J Rev Sante Mediterr Orient Al-Majallah Al-Sihhiyah Li-Sharq Al-Mutawassit. 2020;26: 4–5. doi:10.26719/2020.26.1.4	No	Not meeting inclusion criteria	Title and abstract screening
658	Al-Rabi KH, Maakoseh M, Jaddoua S, Salah S, Alta'ani O, Yasin A, et al. Trends of using immune checkpoint inhibitors near end of life in limited resources setting: Experience of single cancer center in the MENA region. J Clin Oncol. 41: 6566–6566. doi:10.1200/JCO.2023.41.16_suppl.6566	No	Not meeting inclusion criteria	Title and abstract screening
659	Alade O, Folayan MO, Adeniyi A, Adeyemo YI, Oyapero A, Olatosi OO, et al. Differences in Oral Lesions Associated with Tobacco Smoking, E-Cigarette Use and COVID-19 Infection among Adolescents and Young People in Nigeria. Int J Env Res Public Health. 2022;19. doi:10.3390/ijerph191710509	No	Not meeting inclusion criteria	Title and abstract screening
660	Alalwan MA, Singer JM, Roberts ME. Factors Associated with Quit Interest and Quit Attempts among Young Adult JUUL Users. Int J Env Res Public Health. 2022;19. doi:10.3390/ijerph19031403	No	Not meeting inclusion criteria	Title and abstract screening
661	Alanazi AMM, Alqahtani MM, Pavela G, Ford EW, Leventhal AM, Hendricks PS. Mental Health and the Association between Asthma and E-cigarette Use among Young Adults in The United States: A Mediation Analysis. Int J Environ Res Public Health. 2020;17. doi:10.3390/ijerph17238799	No	Not meeting inclusion criteria	Title and abstract screening
662	Alanazi AMM, Alqahtani MM, Lein DH, Ford EW. The relationship between asthma diagnosis and E-Cigarette use among youth and young adults: the mediation effects of anxiety, depression, and impulsivity and the moderation effects of substance use. J Asthma. 2022;59: 682–690. doi:10.1080/02770903.2021.1879849	No	Not meeting inclusion criteria	Title and abstract screening
663	Alanazi AMM, Alqahtani MM, Wells JM, Lein DH Jr, Hendricks PS. Outcome expectancies and resistance self-efficacy mediate the relationship between asthma diagnosis and e-cigarette use among youth and young adults. J Asthma. 2023;60: 1088–1096. doi:10.1080/02770903.2022.2132955	No	Not meeting inclusion criteria	Title and abstract screening
664	Alarabi AB, Lozano PA, Khasawneh FT, Alshbool FZ. The effect of emerging tobacco related products and their toxic constituents on thrombosis. Life Sci. 2022;290: 120255. doi:10.1016/j.lfs.2021.120255	No	Not meeting inclusion criteria	Title and abstract screening
665	Alasmari F, Alotibi FM, Alqahtani F, Alshammari TK, Kadi AA, Alghamdi AM, et al. Effects of Chronic Inhalation of Electronic Cigarette Vapor Containing Nicotine on Neurobehaviors and Pre/Postsynaptic Neuron Markers. Toxics. 2022;10. doi:10.3390/toxics10060338	No	Not meeting inclusion criteria	Title and abstract screening
666	Alasmari F, Crotty Alexander LE, Drummond CA, Sari Y. A computerized exposure system for animal models to optimize nicotine delivery into the brain through inhalation of electronic cigarette vapors or cigarette smoke. Saudi Pharm J. 2018;26: 622–628. doi:10.1016/j.jsps.2018.02.031	No	Not meeting inclusion criteria	Title and abstract screening
667	Alasmari F, Crotty Alexander LE, Nelson JA, Schiefer IT, Breen E, Drummond CA, et al. Effects of chronic inhalation of electronic cigarettes containing nicotine on glial glutamate transporters and α-7 nicotinic acetylcholine receptor in female CD-1 mice. Prog Neuropsychopharmacol Biol Psychiatry. 2017;77: 1–8. doi:10.1016/j.pnpbp.2017.03.017	No	Not meeting inclusion criteria	Title and abstract screening
668	Alasmari FF. Chronic exposure to electronic cigarette vapor-containing nicotine and co-exposure to alcohol and nicotine: Effects on glial glutamate transporters, nicotinic receptors and neurotransmitters. Diss Abstr Int Sect B Sci Eng. 2021;82: No-Specified.	No	Not meeting inclusion criteria	Title and abstract screening
669	Albarrak DA, Alotaibi AB, Alotaibi RF, Alramadhan SH, Bin Muhanna AI, Aldehan AM, et al. The Association Between Nicotine Dependence and Mental Health in the General Population of Saudi Arabia: A Cross-Sectional Analytical Study. Int J Gen Med. 2023;16: 5801–5815. doi:10.2147/IJGM.S429609	No	Not meeting inclusion criteria	Title and abstract screening
670	Alber JM, Conover S, Marts E, Ganjooi K, Grossman S. Examining e-cigarette perspectives before and after the EVALI peak in cases. Addict Behav. 2021;119: 106939. doi:10.1016/j.addbeh.2021.106939	No	Not meeting inclusion criteria	Title and abstract screening
671	Alberca G., Alberca R. E-cigarettes in the COVID-19 era. Ann Thorac Med. 2021;16: 219–220. doi:10.4103/atm.atm_62_21	No	Not meeting inclusion criteria	Title and abstract screening
672	AlDukhail S, Bahdila D. Self-perception of health and physical activity among adults before and amidst the COVID-19 pandemic: United States, 2019-2020. Prev Med. 2022;158: 107037. doi:10.1016/j.ypmed.2022.107037	No	Not meeting inclusion criteria	Title and abstract screening
673	Alex Mason W., Patwardhan I., Fleming C.B., Stevens A.L., James T.D., Nelson J.M., et al. Associations of childhood executive control with adolescent cigarette and E-cigarette use: Tests of moderation by poverty level. Addict Behav. 2021;119: 106923. doi:10.1016/j.addbeh.2021.106923	No	Not meeting inclusion criteria	Title and abstract screening
674	Alexander A., Honan R., Molina A., Rahman A.K.M.F., Walley S.C. Tobacco screening and use in hospitalized adolescents at a Children's Hospital. Hosp Pediatr. 2021;11: 605–612. doi:10.1542/hpeds.2020-002311	No	Not meeting inclusion criteria	Title and abstract screening
675	Alexander A.E., Honan R., Adolfo M., Lauren W.A., Duemler A., Walley S.C. Tobacco Screening in Inpatient Adolescents on Admission. Pediatrics. 2020;146: 482–483. doi:10.1542/peds.146.1_MeetingAbstract.482	No	Not meeting inclusion criteria	Title and abstract screening
676	Alexander AC, Ward KD, Forde DR, Stockton M, Read MC. Do current smokers use more cigarettes and become more dependent on nicotine because of psychological distress after a natural disaster? Addict Behav. 2019;93: 129–134. doi:10.1016/j.addbeh.2019.01.030	No	Not meeting inclusion criteria	Title and abstract screening
677	Alexander JP, Williams P, Lee YO. Youth who use e-cigarettes regularly: A qualitative study of behavior, attitudes, and familial norms. Prev Med Rep. 2019;13: 93–97. doi:10.1016/j.pmedr.2018.11.011	No	Not meeting inclusion criteria	Title and abstract screening
678	AlHarthi A, Alasmari F, AlSharari SD, Alrasheed NM, Alshammari MA, Alshammari TK. Investigating Behavioral and Neuronal Changes in Adolescent Mice Following Prenatal Exposure to Electronic Cigarette (E-Cigarette) Vapor Containing Nicotine. Brain Sci. 2023;13. doi:10.3390/brainsci13101417	No	Not meeting inclusion criteria	Title and abstract screening

679	Ali FRM, Rice K, Fang X, Xu X. Tobacco 21 policies in California and Hawaii and sales of cigarette packs: a difference-in-differences analysis. Tob Control. 2020;29: 588–592. doi:10.1136/tobaccocontrol-2019-055031	No	Not meeting inclusion criteria	Title and abstract screening
680	Ali FRM, Al-Shawaf M, Wang TW, King BA. U.S. Adults' Attitudes Toward Lowering Nicotine Levels in Cigarettes. Am J Prev Med. 2019;57: 403–407. doi:10.1016/j.amepre.2019.04.016	No	Not meeting inclusion criteria	Title and abstract screening
681	Ali FRM, Seidenberg AB, Crane E, Seaman E, Tynan MA, Marynak K. E-cigarette Unit Sales by Product and Flavor Type, and Top-Selling Brands, United States, 2020-2022. MMWR Morb Mortal Wkly Rep. 2023;72: 672–677. doi:10.15585/mmwr.mm7225a1	No	Not meeting inclusion criteria	Title and abstract screening
682	Alkan ŞB, Artaç M, Aksoy F, Belviranlı MM, Gürbilek M, Çizmecioğlu HA, et al. Are dietary and serum advanced glycation end-products related to inflammation and oxidation biomarkers in breast cancer patients: a follow-up study. Support Care Cancer. 2023;31: 1–13. doi:10.1007/s00520-023-07772-w	No	Not meeting inclusion criteria	Title and abstract screening
683	Alkhlaif Y, Shelton KL. Assessment of Abuse-Related Discriminative Stimulus Effects of Nicotine Aerosol in Rodents. J Pharmacol Exp Ther. 2023;385: 171–179. doi:10.1124/jpet.122.001520	No	Not meeting inclusion criteria	Title and abstract screening
684	Allagbé I, Le Faou AL, Thomas D, Airagnes G, Limosin F, Chagué F, et al. Tobacco-related cardiovascular risk in women: New issues and therapeutic perspectives. Arch Cardiovasc Dis. 2021;114: 694–706. doi:10.1016/j.acvd.2021.06.013	No	Not meeting inclusion criteria	Title and abstract screening
685	Allagbé I, Zeller M, Thomas D, Airagnes G, Limosin F, Boussadi A, et al. Cardiovascular Risk Among Patients Who Smoke: Risk Profiles and Differences by Sex. Am J Prev Med. 2022;63: 800–808. doi:10.1016/j.amepre.2022.04.028	No	Not meeting inclusion criteria	Title and abstract screening
686	Allagbe I., Marianne Z., Thomas D., Limosin F., Chague F., Le Faou A.L. Identification of factors associated with smoking cessation in patients with coronary artery diseases. Findings from a large nationwide cohort of smoking cessation services. Eur Respir J. 2022;60: 2322. doi:10.1093/eurheartj/ehac544.2322	No	Not meeting inclusion criteria	Title and abstract screening
687	Allagbe I., Zeller M., Thomas D., Limosin F., Chague F., Le Faou A.-L. Identification of factors associated with smoking cessation in patients with coronary artery diseases. Findings from a large nationwide cohort of smoking cessation services. Arch Cardiovasc Dis Suppl. 2023;15: 129. doi:10.1016/j.acvdsp.2022.10.249	No	Not meeting inclusion criteria	Title and abstract screening
688	Allem J-P, Dormanesh A, Majmundar A, Unger JB, Kirkpatrick MG, Choube A, et al. Topics of Nicotine-Related Discussions on Twitter: Infoveillance Study. J Med Internet Res. 2021;23: e25579. doi:10.2196/25579	No	Not meeting inclusion criteria	Title and abstract screening
689	Allem JP, Dormanesh A, Majmundar A, Rivera V, Chu M, Unger JB, et al. Leading Topics in Twitter Discourse on JUUL and Puff Bar Products: Content Analysis. J Med Internet Res. 2021;23: e26510. doi:10.2196/26510	No	Not meeting inclusion criteria	Title and abstract screening
690	Allen AM, Friedrichsen SC, Petersen N, Allen SS. Subjective response to intranasal nicotine administration in oral contraceptive users and naturally-cycling women. Addict Behav. 2019;98: 106043. doi:10.1016/j.addbeh.2019.106043	No	Not meeting inclusion criteria	Title and abstract screening
691	Allen JL, Wiginton K, Nakarmi J, Golman M, Amick BC 3rd. Physical Activity as a Mediator for Health Outcomes Among Sexual Minority Adults. LGBT Health. 2023;10: 158–168. doi:10.1089/lgbt.2021.0344	No	Not meeting inclusion criteria	Title and abstract screening
692	Allen MJ, Dunn N, Guan T, Harrington J, Walpole E. End-of-life intravenous chemotherapy administration patterns in the treatment of Queensland lung and pancreas cancer patients: a 10-year retrospective analysis. Intern Med J. 2022;52: 623–632. doi:10.1111/imj.15107	No	Not meeting inclusion criteria	Title and abstract screening
693	Allison D.R., Mubarak M., Sharma N., Rao D.S. Kratom (Mitragyna speciosa)-Induced Hepatitis. ACG Case Rep J. 2022;9: E00715. doi:10.14309/crj.0000000000000715	No	Not meeting inclusion criteria	Title and abstract screening
694	Almeda J., Chinnaiyan V., Andl C. E-cigarette vape promotes cell cycle progression and inflammation in 3D pre-clinical oral spheroid models. Cancer Res. 2023;83. doi:10.1158/1538-7445.AM2023-1437	No	Not meeting inclusion criteria	Title and abstract screening
695	Almeida L.M., Silva R.P.D., Santos A.T.C.D., Andrade J.D., Suarez M.C. Mists, vapors and other illusory volatilities of electronic cigarettes. Cad Saude Publica. 2017;33: e00139615. doi:10.1590/0102-311X00139615	No	Not meeting inclusion criteria	Title and abstract screening
696	Almeida LM, Silva RPD, Santos ATCD, Andrade JD, Suarez MC. Mists, vapors and other illusory volatilities of electronic cigarettes. Cad Saude Publica. 2017;33Suppl 3: e00139615. doi:10.1590/0102-311X00139615	No	Not meeting inclusion criteria	Title and abstract screening
697	Almeida-da-Silva CLC, Matshik Dakafay H, O'Brien K, Montierth D, Xiao N, Ojcius DM. Effects of electronic cigarette aerosol exposure on oral and systemic health. Biomed J. 2021;44: 252–259. doi:10.1016/j.bj.2020.07.003	No	Not meeting inclusion criteria	Title and abstract screening
698	ALMNAJAM M., Homsi M. UNUSUAL CAUSE OF TACHYCARDIA-INDUCED CARDIOMYOPATHY. Heart Rhythm. 2023;20: S208–S209. doi:10.1016/j.hrthm.2023.03.615	No	Not meeting inclusion criteria	Title and abstract screening
699	AlMulla A, Mamtani R, Cheema S, Maisonneuve P, Daghfal J, Kouyoumjian S. The association between tobacco use and COVID-19 in Qatar. Prev Med Rep. 2022;28: 101832. doi:10.1016/j.pmedr.2022.101832	No	Not meeting inclusion criteria	Title and abstract screening
700	AlMulla A., Mamtani R., Cheema S., Maisonneuve P., BaSuhai J.A., Mahmoud G., et al. Epidemiology of tobacco use in Qatar: Prevalence and its associated factors. PLoS ONE. 2021;16: e0250065. doi:10.1371/journal.pone.0250065	No	Not meeting inclusion criteria	Title and abstract screening
701	Almutham A, Altami M, Sharaf F, AlAraj A. E-cigarette use among medical students at Qassim University: Knowledge, perception, and prevalence. J Fam Med Prim Care. 2019;8: 2921–2926. doi:10.4103/jfmpc.jfmpc_567_19	No	Not meeting inclusion criteria	Title and abstract screening
702	Alnasser AHA, Al-Tawfiq JA, Kheimi RMA, Alibrahim RMS, Albanawi NAH, Almeshal AKA, et al. Gender Differences in Smoking Attitude among Saudi Medical Students. Asian Pac J Cancer Prev. 2022;23: 2089–2093. doi:10.31557/APJCP.2022.23.6.2089	No	Not meeting inclusion criteria	Title and abstract screening
703	Alomari MA, Khabour OF, Alzoubi KH, Maikano AB. The impact of COVID-19 pandemic on tobacco use: A population-based study. PLoS One. 2023;18: e0287375. doi:10.1371/journal.pone.0287375	No	Not meeting inclusion criteria	Title and abstract screening
704	Alqahtani JS, Aldhahir AM, Oyelade T, Alghamdi SM, Almamary AS. Smoking cessation during COVID-19: the top to-do list. NPJ Prim Care Respir Med. 2021;31: 22. doi:10.1038/s41533-021-00238-8	No	Not meeting inclusion criteria	Title and abstract screening
705	Alqahtani JS, Aldhahir AM, Siraj RA, Alqarni AA, AlDraiwiesh IA, AlAnazi AF, et al. A nationwide survey of public COPD knowledge and awareness in Saudi Arabia: A population-based survey of 15,000 adults. PLoS One. 2023;18: e0287565. doi:10.1371/journal.pone.0287565	No	Not meeting inclusion criteria	Title and abstract screening
706	Alqahtani MM, Alenezi FK, Almeshari MA, Alanazi AM, Taleb ZB, Kalan MEE, et al. E-cigarette use and respiratory symptoms in adults: A systematic review and meta-analysis. Tob Induc Dis. 2023;21: 168. doi:10.18332/tid/174660	No	Not meeting inclusion criteria	Title and abstract screening
707	Alqahtani MM, Pavela G, Lein DH Jr, Vilcassim R, Hendricks PS. The Influence of Mental Health and Respiratory Symptoms on the Association Between Chronic Lung Disease and E-Cigarette Use in Adults in the United States. Respir Care. 2022;67: 814–822. doi:10.4187/respcare.09579	No	Not meeting inclusion criteria	Title and abstract screening

708	Alqahtani MM, Alanazi AMM, Dransfield MT, Wells JM, Lein Jr. DH, Hendricks PS. Relationship Between Chronic Lung Disease Diagnosis and Susceptibility to E-Cigarette Use in Adults. Respir Care. 2023;68: 658–668. doi:10.4187/respcare.10071	No	Not meeting inclusion criteria	Title and abstract screening
709	Alqarni AA, Brand OJ, Pasini A, Alahmari M, Alghamdi A, Pang L. Imbalanced prostanoid release mediates cigarette smoke-induced human pulmonary artery cell proliferation. Respir Res. 2022;23: 136. doi:10.1186/s12931-022-02056-z	No	Not meeting inclusion criteria	Title and abstract screening
710	ALQudah MA, ALFaqih MA, Hamouri S, Al-Shaikh AF, Haddad HK, Al-Quran WY, et al. Epidemiology and histopathological classification of lung cancer: A study from Jordan, retrospective observational study. Ann Med Surg 2012. 2021;65: 102330. doi:10.1016/j.amsu.2021.102330	No	Not meeting inclusion criteria	Title and abstract screening
711	ALRJOOB M., ALKHATIB A.L.A.A., KHAN M., KHAN A.A., WEINER S.M., AL-ALWAN A. A CASE OF RECURRENT NECROTIZING PNEUMONIA SECONDARY TO SELECTIVE IGM DEFICIENCY IN A YOUNG MALE. Chest. 2023;164: A1518–A1519. doi:10.1016/j.chest.2023.07.1044	No	Not meeting inclusion criteria	Title and abstract screening
712	Alrushaid S., Alessa T. Intravenous Iodinated Contrast Induced Thyrotoxic Periodic Paralysis: A Case Report. Case Rep Endocrinol. 2022;2022: 3615312. doi:10.1155/2022/3615312	No	Not meeting inclusion criteria	Title and abstract screening
713	Althobaiti YS, Alzahrani MA, Alsharif NA, Alrobaie NS, Alsaab HO, Uddin MN. The Possible Relationship between the Abuse of Tobacco, Opioid, or Alcohol with COVID-19. Healthc Basel. 2020;9. doi:10.3390/healthcare9010002	No	Not meeting inclusion criteria	Title and abstract screening
714	Aly AS, Mamikutty R, Marhazlinda J. Association between Harmful and Addictive Perceptions of E-Cigarettes and E-Cigarette Use among Adolescents and Youth-A Systematic Review and Meta-Analysis. Child Basel Switz. 2022;9. doi:10.3390/children9111678	No	Not meeting inclusion criteria	Title and abstract screening
715	Alzahrani AAH. Prevalence and social determinants associated with oral cancer in Al-Baha Region of Saudi Arabia: A seven-year retrospective cohort hospital-based study. J Fam Med Prim Care. 2023;12: 2292–2298. doi:10.4103/jfmpc.jfmpc_489_23	No	Not meeting inclusion criteria	Title and abstract screening
716	Alzahrani SH, Alghamdi RA, Almutairi AM, Alghamdi AA, Aljuhani AA, ALbalawi AH. Knowledge and Attitudes Among Medical Students Toward the Clinical Usage of e-Cigarettes: A Cross-Sectional Study in a University Hospital in Saudi Arabia. Risk Manag Healthc Policy. 2021;14: 1969–1984. doi:10.2147/RMHP.S302309	No	Not meeting inclusion criteria	Title and abstract screening
717	Alzahrani Z., Zaidi S.F., Alsolami H., Bashrahil B., Alghamdi N., Nooh M., et al. Electronic cigarettes consumption and associated factors among general population in Western Saudi Arabia. J Public Health Res. 2022;11: 2346. doi:10.4081/jphr.2021.2346	No	Not meeting inclusion criteria	Title and abstract screening
718	Amalia B, Fu M, Tigova O, Ballbè M, Castellano Y, Semple S, et al. Environmental and individual exposure to secondhand aerosol of electronic cigarettes in confined spaces: Results from the TackSHS Project(†). Indoor Air. 2021;31: 1601–1613. doi:10.1111/ina.12841	No	Not meeting inclusion criteria	Title and abstract screening
719	Amalia B, Fu M, Tigova O, Ballbè M, Paniello B, Castellano Y, et al. Exposure to secondhand aerosol from electronic cigarettes at homes: A real-life study in four European countries. Sci Total Env. 2022; 158668. doi:10.1016/j.scitotenv.2022.158668	No	Not meeting inclusion criteria	Title and abstract screening
720	Amaral AL, Lwaleed BA, Andrade SA. Electronic nicotine delivery systems (ENDS): a strategy for smoking cessation or a new risk factor for oral health?. Evid Based Dent. 2023. doi:10.1038/s41432-023-00929-w	No	Not meeting inclusion criteria	Title and abstract screening
721	Amato M.S., Bottcher M.M., Cha S., Jacobs M.A., Pearson J.L., Graham A.L. "It's really addictive and I'm trapped:" A qualitative analysis of the reasons for quitting vaping among treatment-seeking young people. Addict Behav. 2021;112: 106599. doi:10.1016/j.addbeh.2020.106599	No	Not meeting inclusion criteria	Title and abstract screening
722	Ambrosino N., Bertella E. Lifestyle interventions in prevention and comprehensive management of COPD. Breathe. 2018;14: 186–194. doi:10.1183/20734735.018618	No	Not meeting inclusion criteria	Title and abstract screening
723	Amerio A, Stival C, Bosetti C, Carreras G, Fanucchi T, Gorini G, et al. Cannabis use in repeated representative cross-sectional studies on Italian adults after the COVID-19 pandemic. J Psychiatr Res. 2023;164: 382–388. doi:10.1016/j.jpsychires.2023.06.041	No	Not meeting inclusion criteria	Title and abstract screening
724	Amin S, Kawamoto CT, Pokhrel P. Exploring the ChatGPT platform with scenario-specific prompts for vaping cessation. Tob Control. 2023. doi:10.1136/tc-2023-058009	No	Not meeting inclusion criteria	Title and abstract screening
725	Amirahmadi R, Childress J, Patel S, Wagner L-A. Electric cigarette-related lung injury and cardiovascular insult. BMJ Case Rep. 2021;14. doi:10.1136/bcr-2020-238352	No	Not meeting inclusion criteria	Title and abstract screening
726	Amjad M., Hamid Z., Patel Y., Raheja V., Ochieng P.O. Drug-Induced Lung Disease: Who Is the Culprit? Am J Respir Crit Care Med. 2022;205. doi:10.1164/ajrccm-conference.2022.205.1_MeetingAbstracts.A1105	No	Not meeting inclusion criteria	Title and abstract screening
727	Ammar L, Tindle HA, Miller AM, Adgent MA, Nian H, Ryckman KK, et al. Electronic cigarette use during pregnancy and the risk of adverse birth outcomes: A cross-sectional surveillance study of the US Pregnancy Risk Assessment Monitoring System (PRAMS) population. PloS One. 2023;18: e0287348. doi:10.1371/journal.pone.0287348	No	Not meeting inclusion criteria	Title and abstract screening
728	Amoah K., Gunasekaran K., Rahi M.S., Buscher M. A CASE OF ACUTE LUNG INJURY SUPERIMPOSED ON CHRONIC RESPIRATORY BRONCHIOLITIS-INTERSTITIAL LUNG DISEASE. Chest. 2020;158: A761. doi:10.1016/j.chest.2020.08.710	No	Not meeting inclusion criteria	Title and abstract screening
729	An A, Alio AP, Epstein RM, Seplaki C. Key components of goals of care conversations on determining end-of-life care for advanced cancer patients. J Clin Oncol. 41: 12130–12130. doi:10.1200/JCO.2023.41.16_suppl.12130	No	Not meeting inclusion criteria	Title and abstract screening
730	Anand R, Sarode G, Sengupta N, Sarode SC. Smoke and Mirrors: Unmasking E-Cigarette Awareness Among Indian Youth. Asian Pac J Cancer Prev. 2023;24: 3305–3306. doi:10.31557/APJCP.2023.24.10.3305	No	Not meeting inclusion criteria	Title and abstract screening
731	Ananth P. Reenvisioning End-of-Life Care Quality Measurement for Adolescents and Young Adults With Cancer—Novel Patient-Centered Indicators and Approaches. JAMA Netw Open. 2021;4: e2122323–e2122323. doi:10.1001/jamanetworkopen.2021.22323	No	Not meeting inclusion criteria	Title and abstract screening
732	Ananth P, Lindsay M, Mun S, De Oliveira S, Pitafi S, Gross C, et al. Parent Priorities in End-of-Life Care for Children with Cancer (CO202C). J Pain Symptom Manage. 2022;63: 1064–1064. doi:10.1016/j.jpainsymman.2022.04.011	No	Not meeting inclusion criteria	Title and abstract screening
733	Ananth P, Lindsay M, Mun S, McCollum S, Shabanova V, de Oliveira S, et al. Parent Priorities in End-of-Life Care for Children With Cancer. JAMA Netw Open. 2023;6: e2313503–e2313503. doi:10.1001/jamanetworkopen.2023.13503	No	Not meeting inclusion criteria	Title and abstract screening
734	Ananth P, Lindsay M, Nye R, Mun S, Feudtner C, Wolfe J. End-of-life care quality for children with cancer who receive palliative care. Pediatr Blood Cancer. 2022;69: 1–9. doi:10.1002/pbc.29841	No	Not meeting inclusion criteria	Title and abstract screening
735	Ananth P, Mun S, Lindsay M, De Oliveira S, Pitafi S, Ma X, et al. Parent Priorities in End-of-Life Care for Children with Cancer. J Pain Symptom Manage. 2022;63: 885–885. doi:10.1016/j.jpainsymman.2022.02.087	No	Not meeting inclusion criteria	Title and abstract screening
736	Ananth P, Mun S, Reffat N, Li R, Sedghi T, Avery M, et al. A Stakeholder-Driven Qualitative Study to Define High Quality End-of-Life Care for Children With Cancer. J Pain Symptom Manage. 2021;62: 492–502. doi:10.1016/j.jpainsymman.2021.01.134	No	Not meeting inclusion criteria	Title and abstract screening

737	Ananth P, Wolfe J, Johnston EE. Charting a path to high-quality end-of-life care for children with cancer. Cancer 0008543X. 2022;128: 3586–3592. doi:10.1002/cncr.34419	No	Not meeting inclusion criteria	Title and abstract screening
738	Ananthakrishnan A.N., Kaplan G.G., Bernstein C.N., Burke K.E., Lochhead P.J., Sasson A.N., et al. Lifestyle, behaviour, and environmental modification for the management of patients with inflammatory bowel diseases: an International Organization for Study of Inflammatory Bowel Diseases consensus. Lancet Gastroenterol Hepatol. 2022;7: 666–678. doi:10.1016/S2468-1253%2822%2900021-8	No	Not meeting inclusion criteria	Title and abstract screening
739	Anderson Goodell EM, Nordeck C, Finan PH, Vandrey R, Dunn KE, Thrul J. Feasibility and acceptability of using smartphone-based EMA to assess patterns of prescription opioid and medical cannabis use among individuals with chronic pain. Internet Interv. 2021;26: 100460. doi:10.1016/j.invent.2021.100460	No	Not meeting inclusion criteria	Title and abstract screening
740	Anderson K.R., Villafranco N., Cameron L.H., Schallert E.K., Joshi-Patel A., Arrington A., et al. A 16-year-old boy with cough and fever in the era of COVID-19. Pediatrics. 2021;147: e2020008235. doi:10.1542/PEDS.2020-008235	No	Not meeting inclusion criteria	Title and abstract screening
741	Anderson P., Gual A., Rehm J. Reducing the health risks derived from exposure to addictive substances. Curr Opin Psychiatry. 2018;31: 333–341. doi:10.1097/YCO.0000000000000432	No	Not meeting inclusion criteria	Title and abstract screening
742	Anderson S., Atkins P., Backman P., Cipolla D., Clark A., Daviskas E., et al. Inhaled Medicines: Past, Present, and Future. Pharmacol Rev. 2022;74: 50–118. doi:10.1124/PHARMREV.120.000108	No	Not meeting inclusion criteria	Title and abstract screening
743	Andler R, Guignard R, Spilka S, Le Nezet O, Pasquereau A, Richard J-B, et al. [Smoking and vaping in France]. Rev Mal Respir. 2018;35: 673–685. doi:10.1016/j.rmr.2018.01.008	No	Not meeting inclusion criteria	Title and abstract screening
744	Andreas M, Grundinger N, Wolber N, Szafran D, Mons U, Vollstadt-Klein S, et al. Subjective experiences of the addictive potential of e-cigarettes: Results from focus group discussions. Addict Res Theory. 2023; No-Specified. doi:10.1080/16066359.2023.2288831	No	Not meeting inclusion criteria	Title and abstract screening
745	Andreas S., Pankow W. Smoking cessation - Achievable and effective. Dtsch Med Wochenschr. 2021;146: 748–751. doi:10.1055/a-1259-8353	No	Not meeting inclusion criteria	Title and abstract screening
746	Andreas S, Kotz D, Batra A, Hellmann A, Muhlig S, Nowak D, et al. [Smoking Cessation in Patients with COPD]. Pneumol Stuttg Ger. 2023;77: 206–219. doi:10.1055/a-2020-4284	No	Not meeting inclusion criteria	Title and abstract screening
747	Andrews JC, Mays D, Netemeyer RG, Burton S, Kees J. Effects of E-Cigarette Health Warnings and Modified Risk Ad Claims on Adolescent E-Cigarette Craving and Susceptibility. Nicotine Tob Res Off J Soc Res Nicotine Tob. 2019;21: 792–798. doi:10.1093/ntr/nty076	No	Not meeting inclusion criteria	Title and abstract screening
748	Andrews J, Mays D, Netemeyer R, Burton S, Kees J. Effects of E-cigarette health warnings and modified risk Ad claims on adolescent E-cigarette craving and susceptibility. 2018;21: 792-798. doi:10.1093/ntr/nty076	No	Not meeting inclusion criteria	Title and abstract screening
749	Andrus MR. E-Cigarette, or Vaping, Product Use-Associated Lung Injury (EVALI) Continues: An Opportunity for Pharmacist Intervention. Ann Pharmacother. 2023; 10600280231193770. doi:10.1177/10600280231193770	No	Not meeting inclusion criteria	Title and abstract screening
750	Anguita R, Brennan N, Ramsden CM, Mehat M, Keegan D, Cahill R, et al. Patient generated aerosol in the context of ophthalmic surgery. Eur J Ophthalmol. 2022;32: 2445–2451. doi:10.1177/11206721211037823	No	Not meeting inclusion criteria	Title and abstract screening
751	Anic GM, Sawdey MD, Jamal A, Trivers KF. Frequency of Use Among Middle and High School Student Tobacco Product Users - United States, 2015-2017. MMWR Morb Mortal Wkly Rep. 2018;67: 1353–1357. doi:10.15585/mmwr.mm6749a1	No	Not meeting inclusion criteria	Title and abstract screening
752	Anic GM, Rostron BL, Hammad HT, van Bemmel DM, Del Valle-Pinero AY, Christensen CH, et al. Changes in Biomarkers of Tobacco Exposure among Cigarette Smokers Transitioning to ENDS Use: The Population Assessment of Tobacco and Health Study, 2013-2015. Int J Env Res Public Health. 2022;19. doi:10.3390/ijerph19031462	No	Not meeting inclusion criteria	Title and abstract screening
753	Annechino R., Antin T.M.J. Truth telling about tobacco and nicotine. Int J Environ Res Public Health. 2019;16: 530. doi:10.3390/ijerph16040530	No	Not meeting inclusion criteria	Title and abstract screening
754	Anonymous. Nicotine Addiction and E-cigarettes. J Addict Nurs. 2017;28: 229–230. doi:10.1097/JAN.0000000000000201	No	Not meeting inclusion criteria	Title and abstract screening
755	Anonymous. Correction to: Changes in Nicotine Dependence Among Smokers Using Electronic Cigarettes to Reduce Cigarette Smoking in a Randomized Controlled Trial. Nicotine Tob Res Off J Soc Res Nicotine Tob. 2023. doi:10.1093/ntr/ntad229	No	Not meeting inclusion criteria	Title and abstract screening
756	Anonymous. AMB warns against the use of electronic nicotine delivery devices: Electronic and heated cigarettes. Rev Assoc Med Bras. 2017;63: 825–826. doi:10.1590/1806-9282.63.10.825	No	Not meeting inclusion criteria	Title and abstract screening
757	Anonymous. Foundation funding to prevent Smoking. Health Aff (Millwood). 2017;36: 1515–1515. doi:10.1377/hlthaff.2017.0815	No	Not meeting inclusion criteria	Title and abstract screening
758	Anonymous. To vape or not to vape. Aust J Pharm. 2017;98: 16–18.	No	Not meeting inclusion criteria	Title and abstract screening
759	Anonymous. Why do i smoke and why do i keep smoking? Am J Respir Crit Care Med. 2017;196: 7–8.	No	Not meeting inclusion criteria	Title and abstract screening
760	Anonymous. The debate over e-cigarettes demands stronger evidence of their value. Nature. 2019;570: 415. doi:10.1038/d41586-019-01785-4	No	Not meeting inclusion criteria	Title and abstract screening
761	Anonymous. Erratum regarding missing Declaration of Competing Interest statements in previously published articles (Contemporary Clinical Trials Communications (2019) 16, (S2451865419302236), (10.1016/j.conctc.2019.100461)). Contemp Clin Trials Commun. 2020;20: 100689. doi:10.1016/j.conctc.2020.100689	No	Not meeting inclusion criteria	Title and abstract screening
762	Anonymous. 23rd ISAM Congress. J Aerosol Med Pulm Drug Deliv. 2021;34.	No	Not meeting inclusion criteria	Title and abstract screening
763	Anonymous. CME exam: An Adolescent with Respiratory Distress. J Allergy Clin Immunol Pract. 2021;9: 4505–4506. doi:10.1016/j.jaip.2021.10.031	No	Not meeting inclusion criteria	Title and abstract screening
764	Anonymous. Symposia, Case Conference, and Medical Update. Am J Addict. 2021;30: 226–232. doi:10.1111/ajad.13174	No	Not meeting inclusion criteria	Title and abstract screening
765	Anonymous. 42nd International Congress of the European Association of Poisons Centres and Clinical Toxicologists. Clin Toxicol. 2022;60.	No	Not meeting inclusion criteria	Title and abstract screening

766	Anonymous. Abstracts from The Aerosol Society Drug Delivery to the Lungs 32. J Aerosol Med Pulm Drug Deliv. 2022;35.	No	Not meeting inclusion criteria	Title and abstract screening
767	Anonymous. Retraction: "Cancer Prevalence in E-Cigarette Users: A Retrospective Cross-Sectional NHANES Study", (World J Oncol., (2022), 13(1), (20-26), (10.14740/wjon1438)). World J Oncol. 2022;13: 417–417. doi:10.14740/WJON1438R	No	Not meeting inclusion criteria	Title and abstract screening
768	Anonymous. 8th International Pharmaceutical Conference of Al-Zaytoonah University, ZIPC 2022. Jordan J Pharm Sci. 2023;16.	No	Not meeting inclusion criteria	Title and abstract screening
769	Anonymous. 17th Meeting of the Academy AIPMB and 1st National Congress SPPMB. Med Oral Patol Oral Cirugia Bucal. 2023;28.	No	Not meeting inclusion criteria	Title and abstract screening
770	Anonymous. Abstracts from the International Symposium "Signal Transduction at the Blood-Brain Barriers" 2022. Fluids Barriers CNS. 2023;20.	No	Not meeting inclusion criteria	Title and abstract screening
771	Anonymous. Erratum regarding missing patient consent statements in previously published articles (Radiology Case Reports (2021) 16(9) (2530-2533), (S1930043321003885), (10.1016/j.radcr.2021.06.011)). Radiol Case Rep. 2023. doi:10.1016/j.radcr.2023.01.019	No	Not meeting inclusion criteria	Title and abstract screening
772	Antin TMJ, Hess C, Kaner E, Lipperman-Kreda S, Annechino R, Hunt G. Pathways of Nicotine Product Use: A Qualitative Study of Youth and Young Adults in California. Nicotine Tob Res. 2020;22: 722–727. doi:10.1093/ntr/ntz028	No	Not meeting inclusion criteria	Title and abstract screening
773	Antin TMJ, Hunt G, Kaner E, Lipperman-Kreda S. Youth perspectives on concurrent smoking and vaping: Implications for tobacco control. Int J Drug Policy. 2019;66: 57–63. doi:10.1016/j.drugpo.2019.01.018	No	Not meeting inclusion criteria	Title and abstract screening
774	Antwi GO, Lohrmann DK, Jayawardene W, Chow A, Obeng CS, Sayegh AM. Associations between Cigarette Smoking and Health-Related Quality of Life in Adult Survivors of Adolescent and Young Adult Cancer. J Cancer Educ. 2022;37: 508–516. doi:10.1007/s13187-020-01837-8	No	Not meeting inclusion criteria	Title and abstract screening
775	Antwi GO, Rhodes DL. Association between E-cigarette use and chronic obstructive pulmonary disease in non-asthmatic adults in the USA. J Public Health Oxf. 2022;44: 158–164. doi:10.1093/pubmed/fdaa229	No	Not meeting inclusion criteria	Title and abstract screening
776	Antwi GO, Rhodes DL. Association between e-cigarette use and depression in US cancer survivors: a cross-sectional study. J Cancer Surviv. 2022. doi:10.1007/s11764-022-01176-1	No	Not meeting inclusion criteria	Title and abstract screening
777	Antwi GO, Rhodes DL. Association between e-cigarette use and depression in US cancer survivors: a cross-sectional study. J Cancer Surviv. 2023;17: 1452–1460. doi:10.1007/s11764-022-01176-1	No	Not meeting inclusion criteria	Title and abstract screening
778	Aoyanagi M, Shindo Y, Takahashi K. General Ward Nurses' Self-Efficacy, Ethical Behavior, and Practice of Discharge Planning for End-Stage Cancer Patients: Path Analysis. Healthc 2227-9032. 2022;10: 1161–1161. doi:10.3390/healthcare10071161	No	Not meeting inclusion criteria	Title and abstract screening
779	April-Sanders A.K., Daviglus M.L., Lee U.J., Perreira K., Pirzada A., Bandiera F., et al. Prevalence of electronic cigarette use and its determinants in US persons of hispanic heritage: The hispanic community health study / study of Latinos (HCHS/SOL). Circulation. 2021;144. doi:10.1161/circ.144.suppl_1.10690	No	Not meeting inclusion criteria	Title and abstract screening
780	Archie S.R., Sifat A.E., Villalba H., Sharma S., Zhang Y., Abbruscato T. North American Travel Grant Recipient: Potential Postnatal Neurotoxicity Mediated by Maternal E-Cigarette Exposure. Int J Toxicol. 2023;42: 78. doi:10.1177/10915818221143638	No	Not meeting inclusion criteria	Title and abstract screening
781	Arezo S, Naavaal S, Garrett C, Wright MS, Sheppard VB, Preston MA. Implementation of a Cancer Education Program in Rural Counties with the Lowest HPV Vaccination Rates and Health Rankings. J Health Res. 2024;38: 88–93. doi:10.56808/2586-940x.1057	No	Not meeting inclusion criteria	Title and abstract screening
782	Ariel I. Effect of electronic cigarette messages on young-adult behavioral dispositions towards use. Diss Abstr Int Sect B Sci Eng. 2018;78: No-Specified.	No	Not meeting inclusion criteria	Title and abstract screening
783	Arnaud N., Holtmann M., Melchers P., Klein M., Schimansky G., Kromer T., et al. Use of Electronic Cigarettes (e-cigs) and e-Shishas by Children and Adolescents: Evidence Paper of the Joint Addiction Commission of the German Societies and Professional Associations of Child and Adolescent Psychiatry and Psychotherapy. Z Kinder Jugendpsychiatr Psychother. 2022;50: 121–132. doi:10.1024/1422-4917/a000831	No	Not meeting inclusion criteria	Title and abstract screening
784	Arnaud N, Holtmann M, Melchers P, Klein M, Schimansky G, Kromer T, et al. [Use of Electronic Cigarettes (e-cigs) and e-Shishas by Children and Adolescents: Evidence Paper of the Joint Addiction Commission of the German Societies and Professional Associations of Child and Adolescent Psychiatry and Psychotherapy]. Z Kinder Jugendpsychiatr Psychother. 2021;50: 121–132. doi:10.1024/1422-4917/a000831	No	Not meeting inclusion criteria	Title and abstract screening
785	Arnaud N, Wartberg L, Simon-Kutscher K, Thomasius R. Prevalence of substance use disorders and associations with mindfulness, impulsive personality traits and psychopathological symptoms in a representative sample of adolescents in Germany. Eur Child Adolesc Psychiatry. 2023. doi:10.1007/s00787-023-02173-0	No	Not meeting inclusion criteria	Title and abstract screening
786	Arreola-Ramírez JL, Vargas MH, Carbajal V, Alquicira-Mireles J, Montaño M, Ramos-Abraham C, et al. Mesenchymal stem cells attenuate the proinflammatory cytokine pattern in a guinea pig model of chronic cigarette smoke exposure. Cytokine. 2023;162: 156104. doi:10.1016/j.cyto.2022.156104	No	Not meeting inclusion criteria	Title and abstract screening
787	Arshad H, Jackson SE, Kock L, Ide-Walters C, Tattan-Birch H. What drives public perceptions of e-cigarettes? A mixed-methods study exploring reasons behind adults' perceptions of e-cigarettes in Northern England. Drug Alcohol Depend. 2023;245: 109806. doi:10.1016/j.drugalcdep.2023.109806	No	Not meeting inclusion criteria	Title and abstract screening
788	Arthur RA, Dos Santos Bezerra R, Ximenez JPB, Merlin BL, de Andrade Morraye R, Neto JV, et al. Microbiome and oral squamous cell carcinoma: a possible interplay on iron metabolism and its impact on tumor microenvironment. Braz J Microbiol. 2021;52: 1287–1302. doi:10.1007/s42770-021-00491-6	No	Not meeting inclusion criteria	Title and abstract screening
789	Arvin MC, Jin XT, Yan Y, Wang Y, Ramsey MD, Kim VJ, et al. Chronic Nicotine Exposure Alters the Neurophysiology of Habenulo-Interpeduncular Circuitry. J Neurosci. 2019;39: 4268–4281. doi:10.1523/JNEUROSCI.2816-18.2019	No	Not meeting inclusion criteria	Title and abstract screening
790	Asano A, Sakai S, Seki N, Koyama Y. Development and Psychometric Evaluation of a Japanese Version of Newly Graduated Nurses' Difficulties with End-of-Life Care for Cancer Patients (NDEC Scale). Nurs Rep. 12: 637–647. doi:10.3390/nursrep12030063	No	Not meeting inclusion criteria	Title and abstract screening
791	Asare S, Majmundar A, Xue Z, Jemal A, Nargis N. Association of Comprehensive Menthol Flavor Ban With Current Cigarette Smoking in Massachusetts From 2017 to 2021. JAMA Intern Med. 2023;183: 383–386. doi:10.1001/jamainternmed.2022.6743	No	Not meeting inclusion criteria	Title and abstract screening
792	Asaumi K, Oki M, Murakami Y. Timely Identification of Patients With Cancer and Family Caregivers in Need of End-of-Life Discussions by Home-Visit Nurses in Japan: A Qualitative Descriptive Study. Glob Qual Nurs Res. 2023; 1–10. doi:10.1177/23333936221146048	No	Not meeting inclusion criteria	Title and abstract screening
793	Asfar T, Jebai R, Li W, Oluwole OJ, Ferdous T, Gautam P, et al. Risk and safety profile of electronic nicotine delivery systems (ENDS): an umbrella review to inform ENDS health communication strategies. Tob Control. 2022. doi:10.1136/tc-2022-057495	No	Not meeting inclusion criteria	Title and abstract screening
794	Asfar T, Livingstone-Banks J, Ward KD, Eissenberg T, Oluwole O, Bursac Z, et al. Interventions for waterpipe smoking cessation. Cochrane Database Syst Rev. 2023;6: CD005549. doi:10.1002/14651858.CD005549.pub4	No	Not meeting inclusion criteria	Title and abstract screening

795	Asfar T, Oluwole OJ, Pan Y, Casas A, Hernandez Garayua AM, Schmidt M, et al. Youth Exposure and Response to the FDA Health Warning Label on Electronic Cigarettes Packaging: Policy Implications. Nicotine Tob Res Off J Soc Res Nicotine Tob. 2023. doi:10.1093/ntr/ntad175	No	Not meeting inclusion criteria	Title and abstract screening
796	Asfour V, Nikolopoulos K, Digesu G, Emery S, Khan Z. Modified autologous fascial sling technique ('sling on a string') for stress incontinence. 2021. doi:10.1007/s00192-021-04815-w	No	Not meeting inclusion criteria	Title and abstract screening
797	Asgharian B., Price O., Creel A., Chesnutt J., Schroeter J., Fallica J., et al. Simulation Modeling of Air and Droplet Temperatures in the Human Respiratory Tract for Inhaled Tobacco Products. Ann Biomed Eng. 2022. doi:10.1007/s10439-022-03082-0	No	Not meeting inclusion criteria	Title and abstract screening
798	Ashing K., Presant C., Yeung S., Macalintal J., Tiep B., Sandoval A., et al. Spatial and descriptive analysis of smoke and vape shop locations focusing on a cancer center neighboring catchment area. Cancer Epidemiol Biomarkers Prev. 2022;31. doi:10.1158/1538-7755.DISP21-PO-273	No	Not meeting inclusion criteria	Title and abstract screening
799	Ashing KT, Song G, Tiep B, Presant C, Obodo U, Macalintal J, et al. Does neighborhood or residence influence continued smoking among cancer patients: a spatial-ecological and descriptive analyses brief report. Cancer Causes Control CCC. 2022. doi:10.1007/s10552-022-01655-x	No	Not meeting inclusion criteria	Title and abstract screening
800	Ashing KT, Song G, O'Connor T, Obodo U, Abuan F, Dawson CT, et al. Spatial and Descriptive Analysis of Smoke and Vape Shop Locations Focusing on A Cancer Center Neighboring Catchment Area. Pap Appl Geogr. 2022;8: 61–71. doi:10.1080/23754931.2021.1947354	No	Not meeting inclusion criteria	Title and abstract screening
801	Ashing KT, Song G, Tiep B, Presant C, Obodo U, Macalintal J, et al. Does neighborhood or residence influence continued smoking among cancer patients: a spatial-ecological and descriptive analyses brief report. Cancer Causes Control. 2023;34: 389–398. doi:10.1007/s10552-022-01655-x	No	Not meeting inclusion criteria	Title and abstract screening
802	Ashley DL, Spears CA, Weaver SR, Huang J, Eriksen MP. E-cigarettes: How can they help smokers quit without addicting a new generation?. Prev Med. 2020;140: 106145. doi:10.1016/j.ypmed.2020.106145	No	Not meeting inclusion criteria	Title and abstract screening
803	Ashraf MT, Shaikh A, Khan MKS, Uddin N, Kashif MAB, Rizvi SHA, et al. Association between e-cigarette use and myocardial infarction: a systematic review and meta-analysis. Egypt Heart J. 2023;75: 97. doi:10.1186/s43044-023-00426-6	No	Not meeting inclusion criteria	Title and abstract screening
804	Ashton J. Framing the question: electronic cigarettes and harm reduction. J R Soc Med. 2019;112: 485–486. doi:10.1177/0141076819885806	No	Not meeting inclusion criteria	Title and abstract screening
805	ASKAN G, ERBARUT SEVEN I, OZKAN N, EREN F. Are histomorphologic changes in the fimbrial ends more to blame for primary epithelial ovarian carcinomas than initially thought? Marmara Med J. 2022;35: 23–30. doi:10.5472/marumj.1056169	No	Not meeting inclusion criteria	Title and abstract screening
806	Aso S, Hayashi N, Sekimoto G, Nakayama N, Tamura K, Yamamoto C, et al. Association between temporary discharge from the inpatient palliative care unit and achievement of good death in end-of-life cancer patients: A nationwide survey of bereaved family members. Jpn J Nurs Sci. 2022;19: 1–10. doi:10.1111/jjns.12474	No	Not meeting inclusion criteria	Title and abstract screening
807	Assi HI, Meouchy P, El Mahmoud A, Massouh A, Bou Zerdan M, Alameh I, et al. A Survey on the Knowledge, Attitudes, and Practices of Lebanese Physicians Regarding Air Pollution. Int J Env Res Public Health. 2022;19. doi:10.3390/ijerph19137907	No	Not meeting inclusion criteria	Title and abstract screening
808	Atuegwu NC, Perez MF, Oncken C, Thacker S, Mead EL, Mortensen EM. Association between Regular Electronic Nicotine Product Use and Self-reported Periodontal Disease Status: Population Assessment of Tobacco and Health Survey. Int J Env Res Public Health. 2019;16. doi:10.3390/ijerph16071263	No	Not meeting inclusion criteria	Title and abstract screening
809	Auclair J, Sanchez S, Chrusciel J, Hannetel L, Frasca M, Economos G, et al. Duration of palliative care involvement and immunotherapy treatment near the end of life among patients with cancer who died in-hospital. Support Care Cancer. 2022;30: 4997–5006. doi:10.1007/s00520-022-06901-1	No	Not meeting inclusion criteria	Title and abstract screening
810	Audrain-McGovern J., Rodriguez D., Pianin S., Testa S. Conjoint developmental trajectories of adolescent e-cigarette and combustible cigarette use. Pediatrics. 2021;148: e2021051828. doi:10.1542/peds.2021-051828	No	Not meeting inclusion criteria	Title and abstract screening
811	Auer R, Diethelm P, Berthet A. Heating Tobacco Sticks Instead of Combusting Conventional Cigarettes and Future Heart Attacks: Still Smoke, and Risk. Circulation. 2021;144: 1539–1542. doi:10.1161/CIRCULATIONAHA.121.056959	No	Not meeting inclusion criteria	Title and abstract screening
812	Auschwitz E, Almeda J, Andl CD. Mechanisms of E-Cigarette Vape-Induced Epithelial Cell Damage. Cells. 2023;12. doi:10.3390/cells12212552	No	Not meeting inclusion criteria	Title and abstract screening
813	Austin-Datta RJ, Chaudhari PV, Cheng TD, Klarenberg G, Striley CW, Cottler LB. Electronic Nicotine Delivery Systems (ENDS) use Among Members of a Community Engagement Program. J Community Health. 2022. doi:10.1007/s10900-022-01169-2	No	Not meeting inclusion criteria	Title and abstract screening
814	Avelar AJ, Akers AT, Baumgard ZJ, Cooper SY, Casinelli GP, Henderson BJ. Why flavored vape products may be attractive: Green apple tobacco flavor elicits reward-related behavior, upregulates nAChRs on VTA dopamine neurons, and alters midbrain dopamine and GABA neuron function. Neuropharmacology. 2019;158: 107729. doi:10.1016/j.neuropharm.2019.107729	No	Not meeting inclusion criteria	Title and abstract screening
815	Avelar AJ, Cooper SY, Wright TD, Wright SK, Richardson MR, Henderson BJ. Morphine Exposure Reduces Nicotine-Induced Upregulation of Nicotinic Receptors and Decreases Volitional Nicotine Intake in a Mouse Model. Nicotine Tob Res Off J Soc Res Nicotine Tob. 2022;24: 1161–1168. doi:10.1093/ntr/ntac002	No	Not meeting inclusion criteria	Title and abstract screening
816	Avery RJ, Kalaji M, Niederdeppe J, Mathios A, Dorf M, Byrne S, et al. Perceived threat and fear responses to e-cigarette warning label messages: Results from 16 focus groups with U.S. youth and adults. PloS One. 2023;18: e0286806. doi:10.1371/journal.pone.0286806	No	Not meeting inclusion criteria	Title and abstract screening
817	Aveyard P, Arnott D, Johnson KC. Should we recommend e-cigarettes to help smokers quit? BMJ. 2018;361: k1759. doi:10.1136/bmj.k1759	No	Not meeting inclusion criteria	Title and abstract screening
818	Avishai A, Ribisl KM, Sheeran P. Realizing the Tobacco Endgame: Understanding and mobilizing public support for banning combustible cigarette sales in the United States. Soc Sci Med. 2023;327: 115939. doi:10.1016/j.socscimed.2023.115939	No	Not meeting inclusion criteria	Title and abstract screening
819	Awad K, Mohammed M, Martin SS, Banach M. Association between electronic nicotine delivery systems use and risk of stroke: a meta-analysis of 1,024,401 participants. Arch Med Sci. 2023;19: 1538–1540. doi:10.5114/aoms/171473	No	Not meeting inclusion criteria	Title and abstract screening
820	Ayaz T, Fredrickson S, O'Mary K, Panchbhavi MA, Panchbhavi VK. Differences in cancer amputee survival based on marital status: an analysis of the Surveillance, Epidemiology, and End Results (SEER) database. J Psychosoc Oncol. 2022;40: 203–214. doi:10.1080/07347332.2021.1887429	No	Not meeting inclusion criteria	Title and abstract screening
821	Aycock CA, Wang XQ, Williams JB, Fahey MC, Talcott GW, Klesges RC, et al. Motives for using electronic nicotine delivery systems (ENDS) as a cessation tool are associated with tobacco abstinence at 1-year follow-up: A prospective investigation among young adults in the United States Air Force. Prev Med Rep. 2023;35: 102399. doi:10.1016/j.pmedr.2023.102399	No	Not meeting inclusion criteria	Title and abstract screening
822	Ayers JW, Leas EC, Dredze M, Caputi TL, Zhu SH, Cohen JE. Did Philip Morris International use the e-cigarette, or vaping, product use associated lung injury (EVALI) outbreak to market IQOS heated tobacco? Tob Control. 2023;32: 131–132. doi:10.1136/tobaccocontrol-2021-056661	No	Not meeting inclusion criteria	Title and abstract screening
823	Azagba S., Shan L. Tobacco craving, nicotine dependence, and quit intentions among lgb and non-lgb high school students: A quasi-experimental analysis. Int J Environ Res Public Health. 2021;18: 9000. doi:10.3390/ijerph18179000	No	Not meeting inclusion criteria	Title and abstract screening

824	Azagba S, Manzione LC, Shan L. Response to “Adolescent tobacco use in the current era of multiple products for smoking”. J Adolesc Health. 2020;66: 259. doi:10.1016/j.jadohealth.2019.11.296https://dx.doi.org/10.1016/j.jadohealth.2019.11.296	No	Not meeting inclusion criteria	Title and abstract screening
825	AZEEM A.H.A.D., STALLER A.R. INHALED ANESTHETIC-INDUCED DIFFUSE ALVEOLAR HEMORRHAGE. Chest. 2023;164: A88. doi:10.1016/j.chest.2023.07.110	No	Not meeting inclusion criteria	Title and abstract screening
826	Azeem N, Sarfraz Z, Sarfraz A, Hange N, Sarfraz M, Cherrez-Ojeda I. Vaping and smokeless tobacco control in South Asia: A policy review. Ann Med Surg Lond. 2022;81: 104285. doi:10.1016/j.amsu.2022.104285	No	Not meeting inclusion criteria	Title and abstract screening
827	Baca-Atlas M., Mounsey A., Goldstein A.O. Electronic cigarettes: More questions than answers. Am Fam Physician. 2019;100: 600–601.	No	Not meeting inclusion criteria	Title and abstract screening
828	Bachan M., Khan Z., Menon A. When COVID Goes Undetected. Am J Respir Crit Care Med. 2021;203. doi:10.1164/ajrccm-conference.2021.TP100	No	Not meeting inclusion criteria	Title and abstract screening
829	Bacle A., Le Dare B., Vigneau C. Lessons for the clinical nephrologist: unexplained tacrolimus overdose in a kidney transplant patient. J Nephrol. 2022;35: 1911–1913. doi:10.1007/s40620-022-01297-x	No	Not meeting inclusion criteria	Title and abstract screening
830	Badayai ARA, Wahab S, Basri NA, Essau CA. Nicotine and e-cigarettes addiction. Adolesc Addict Epidemiol Assess Treat 2nd Ed. 2020; 137–157. doi:10.1016/B978-0-12-818626-8.00005-0https://dx.doi.org/10.1016/B978-0-12-818626-8.00005-0	No	Not meeting inclusion criteria	Title and abstract screening
831	Bade AN, Gendelman HE, Boska MD, Liu Y. MEMRI is a biomarker defining nicotine-specific neuronal responses in subregions of the rodent brain. Am J Transl Res. 2017;9: 601–610.	No	Not meeting inclusion criteria	Title and abstract screening
832	Baer C.M., Khoussine M.A., Dobbs P.D. Tobacco prevention education for middle school and high school educators. Health Educ J. 2021;80: 16–27. doi:10.1177/0017896920950344	No	Not meeting inclusion criteria	Title and abstract screening
833	Bafunno D., Galetta D., Catino A., De Leo A., D’Alonzo M.G., Longo V., et al. EP01.01-12 Binge-Watching Frequency and Tobacco Use in Adolescents: Data from “Blaam Smoke-Free Movie” Program. J Thorac Oncol. 2023;18: S418. doi:10.1016/j.jtho.2023.09.751	No	Not meeting inclusion criteria	Title and abstract screening
834	Bagale K, Paudel S, Cagle H, Sigel E, Kulkarni R. Electronic Cigarette (E-Cigarette) Vapor Exposure Alters the Streptococcus pneumoniae Transcriptome in a Nicotine-Dependent Manner without Affecting Pneumococcal Virulence. Appl Environ Microbiol. 2020;86. doi:10.1128/AEM.02125-19	No	Not meeting inclusion criteria	Title and abstract screening
835	Bagdas D, Kebede N, Zepei AM, Harris L, Minanov K, Picciotto MR, et al. Animal Models to Investigate the Impact of Flavors on Nicotine Addiction and Dependence. Curr Neuropharmacol. 2022;20: 2175–2201. doi:10.2174/1570159X20666220524120231	No	Not meeting inclusion criteria	Title and abstract screening
836	Baggett TP, Yaqubi A, Berkowitz SA, Kalkhoran SM, McGlave C, Chang Y, et al. Subsistence difficulties are associated with more barriers to quitting and worse abstinence outcomes among homeless smokers: evidence from two studies in Boston, Massachusetts. BMC Public Health. 2018;18: 463. doi:10.1186/s12889-018-5375-z	No	Not meeting inclusion criteria	Title and abstract screening
837	Bahar- Ozdemir Y, Kaya S, Babacan NA-, Al T, Albayrak E, Coskun N, et al. Can assessment of disease burden and quality of life based on mobility level in patients with end-stage cancer provide an insight into unmet needs? An exploratory cross-sectional study. Philadelphia, Pennsylvania: Taylor & Francis Ltd; 2023 pp. 1141–1151. Available: http://myaccess.library.utoronto.ca/login?url=https://search.ebscohost.com/login.aspx?direct=true&db=cin20&AN=163915353&site=ehost-live	No	Not meeting inclusion criteria	Title and abstract screening
838	Bähler C, Näpflin M, Scherer M, Blozik E. Continuity of care and treatment intensity at the end of life in Swiss cancer patients. Eur J Public Health. 2023;33: 396–402. doi:10.1093/eurpub/ckad047	No	Not meeting inclusion criteria	Title and abstract screening
839	Bailey T., Reed M. PULMONARY MANIFESTIONS OF INFLAMMATORY BOWEL DISEASE. Chest. 2021;160: A1686. doi:10.1016/j.chest.2021.07.1534	No	Not meeting inclusion criteria	Title and abstract screening
840	Bains S., Garmany R., Gao X., Lopes Neves R.A., Bos J.M., Tester D., et al. PO-704-02 VAPING-ASSOCIATED SUDDEN DEATH IN THE YOUNG. Heart Rhythm. 2022;19: S450. doi:10.1016/j.hrthm.2022.03.1065	No	Not meeting inclusion criteria	Title and abstract screening
841	Bakaloudi DR, Evripidou K, Siargkas A, Breda J, Chourdakis M. Impact of COVID-19 lockdown on smoking and vaping: systematic review and meta-analysis. Public Health. 2023;218: 160–172. doi:10.1016/j.puhe.2023.02.007	No	Not meeting inclusion criteria	Title and abstract screening
842	Baker AN. The role of flavor in electronic cigarette use and appeal. Diss Abstr Int Sect B Sci Eng. 2022;83: No-Specified.	No	Not meeting inclusion criteria	Title and abstract screening
843	Baker L, Foxhall M, Hulbert J, Lewis-Norman C, Allison E, Irwin R. Life beyond cancer: exploring the value of end-of-treatment clinics for teenagers and young adults. Cancer Nurs Pract. 2021;20: 30–35. doi:10.7748/cnp.2021.e1751	No	Not meeting inclusion criteria	Title and abstract screening
844	Balakrishnan K., Jackler R.K., Brenner M.J., Collar R.M. Do You Juul? Vaping and related emerging public health threats in otolaryngology. Otolaryngol Head Neck Surg. 2020;163: P37. doi:10.1177/0194599820934779	No	Not meeting inclusion criteria	Title and abstract screening
845	Balakrishnan K., Jackler R.K., Brenner M.J., Collar R.M. From E-Cig to Puff Bar: Otolaryngology and the vaping epidemic. Otolaryngol Head Neck Surg. 2021;165: P16. doi:10.1177/01945998211030919a	No	Not meeting inclusion criteria	Title and abstract screening
846	Balante J, van den Broek D, White K. Mixed-methods systematic review: Cultural attitudes, beliefs and practices of internationally educated nurses towards end-of-life care in the context of cancer. J Adv Nurs John Wiley Sons Inc. 2021;77: 3618–3629. doi:10.1111/jan.14814	No	Not meeting inclusion criteria	Title and abstract screening
847	Balaraman A.K., Aziz-Ur-rahman, Xuan Y.W., Keshavarzi F., Farrukh M.J. Smoking Cessation Practitioners Views Towards Safety and Effectiveness of Electronic Cigarettes in Klang Valley, Malaysia: A Qualitative Study Approach. Curr Trends Biotechnol Pharm. 2022;16: 77–88. doi:10.5530/ctbp.2022.1.8	No	Not meeting inclusion criteria	Title and abstract screening
848	Balboni TA, Rades D. Palliative Radiation Oncology: Personalized Approaches to Radiotherapeutic Technologies, Quality of Life, and End-of-life Cancer Care. Semin Radiat Oncol. 2023;33: 91–92. doi:10.1016/j.semradonc.2023.01.001	No	Not meeting inclusion criteria	Title and abstract screening
849	Baldassarri SR. Electronic Cigarettes: Past, Present, and Future: What Clinicians Need to Know. Clin Chest Med. 2020;41: 797–807. doi:10.1016/j.ccm.2020.08.018	No	Not meeting inclusion criteria	Title and abstract screening
850	Baldassarri SR, Bernstein SL, Chupp GL, Slade MD, Fucito LM, Toll BA. Electronic cigarettes for adults with tobacco dependence enrolled in a tobacco treatment program: A pilot study. Addict Behav. 2018;80: 1–5. doi:10.1016/j.addbeh.2017.11.033	No	Not meeting inclusion criteria	Title and abstract screening
851	Baldassarri SR, Fiellin DA, Savage ME, Madden LM, Beitel M, Dhingra LK, et al. Electronic cigarette and tobacco use in individuals entering methadone or buprenorphine treatment. Drug Alcohol Depend. 2019;197: 37–41. doi:10.1016/j.drugalcdep.2018.12.012	No	Not meeting inclusion criteria	Title and abstract screening

852	Baldassarri SR, Hillmer AT, Anderson JM, Jatlow P, Nabulsi N, Labaree D, et al. Use of Electronic Cigarettes Leads to Significant Beta2-Nicotinic Acetylcholine Receptor Occupancy: Evidence From a PET Imaging Study. Nicotine Tob Res. 2018;20: 425–433. doi:10.1093/ntr/ntx091	No	Not meeting inclusion criteria	Title and abstract screening
853	Balfour DJK, Benowitz NL, Colby SM, Hatsukami DK, Lando HA, Leischow SJ, et al. Balancing Consideration of the Risks and Benefits of E-Cigarettes. Am J Public Health. 2021;111: 1661–1672. doi:10.2105/AJPH.2021.306416	No	Not meeting inclusion criteria	Title and abstract screening
854	Ballal S, Yadav MP, Satapathy S, Raju S, Tripathi M, Damle NA, et al. Long-term survival outcomes of salvage [225Ac]Ac-PSMA-617 targeted alpha therapy in patients with PSMA-expressing end-stage metastatic castration-resistant prostate cancer: a real-world study. Eur J Nucl Med Mol Imaging. 2023;50: 3777–3789. doi:10.1007/s00259-023-06340-y	No	Not meeting inclusion criteria	Title and abstract screening
855	Ballbè M, Fu M, Masana G, Pérez-Ortuño R, Gual A, Gil F, et al. Passive exposure to electronic cigarette aerosol in pregnancy: A case study of a family. Env Res. 2023;216: 114490. doi:10.1016/j.envres.2022.114490	No	Not meeting inclusion criteria	Title and abstract screening
856	Balmes J.R. Vaping-induced acute lung injury: An epidemic that could have been prevented. Am J Respir Crit Care Med. 2019;200: 1342–1344. doi:10.1164/rccm.201910-1903ED	No	Not meeting inclusion criteria	Title and abstract screening
857	Balozian P, Al Armashi AR, Boustany A, Palaparty P. Overall survival trends of papillary thyroid cancer: A Surveillance, Epidemiology, and End Results (SEER) population-based data (2002-2016). J Clin Oncol. 41: e18108–e18108. doi:10.1200/JCO.2023.41.16_suppl.e18108	No	Not meeting inclusion criteria	Title and abstract screening
858	Bals R, Boyd J, Esposito S, Foronjy R, Hiemstra PS, Jiménez-Ruiz CA, et al. Electronic cigarettes: a task force report from the European Respiratory Society. Eur Respir J. 2019;53. doi:10.1183/13993003.01151-2018	No	Not meeting inclusion criteria	Title and abstract screening
859	Balwicki L, Smith D, Balwicka-Szczyrba M, Gawron M, Sobczak A, Goniewicz ML. Youth Access to Electronic Cigarettes in an Unrestricted Market: A Cross-Sectional Study from Poland. Int J Env Res Public Health. 2018;15. doi:10.3390/ijerph15071465	No	Not meeting inclusion criteria	Title and abstract screening
860	Bamunuarachchi K., Ragatha R.K., Anwar M., Russell P., Ekeowa U., Long M. A Case of Exogenous Lipoid Pneumonia Caused by Inhalation of Baby Oil. Am J Respir Crit Care Med. 2022;205. doi:10.1164/ajrccm-conference.2022.205.1_MeetingAbstracts.A4213	No	Not meeting inclusion criteria	Title and abstract screening
861	Ban M, Chen J. Fabrication of plane-type axon guidance substrates by applying diamond-like carbon thin film deposition. Sci Rep. 2023;13: 8489. doi:10.1038/s41598-023-35528-3	No	Not meeting inclusion criteria	Title and abstract screening
862	Bandara N.A. Adolescents' electronic cigarette use. Pediatrics. 2019;143: e0193328. doi:10.1542/peds.2018-3151	No	Not meeting inclusion criteria	Title and abstract screening
863	Bandara N.A., Herath J., Mehrnoush V. Addressing e-cigarette health claims made on social media amidst the COVID-19 pandemic. World J Pediatr. 2021;17: 3–5. doi:10.1007/s12519-021-00410-7	No	Not meeting inclusion criteria	Title and abstract screening
864	Bandara NA, Mehrnoush V. E-Cigarettes, Incentives, and Drugs for Smoking Cessation. N Engl J Med. 2018;379: 991. doi:10.1056/NEJMc1809349	No	Not meeting inclusion criteria	Title and abstract screening
865	Bandara NA, Zhou XR, Alhamam A, Black PC, St-Laurent M-P. The genitourinary impacts of electronic cigarette use: a systematic review of the literature. World J Urol. 2023;41: 2637–2646. doi:10.1007/s00345-023-04546-1	No	Not meeting inclusion criteria	Title and abstract screening
866	Bandela M., Letsiou E., Singla S., Natarajan V., Dudek S. Cigarette or E-cigarette content alters autophagy and permeability of lung endothelium. FASEB J. 2021;35. doi:10.1096/fasebj.2021.35.S1.03191	No	Not meeting inclusion criteria	Title and abstract screening
867	Bandela M., Letsiou E., Singla S., Natarajan V., Dudek S.M. Cortactin Dysfunction Mediates Cigarette Smoke-Induced Lung Endothelial Apoptosis. Am J Respir Crit Care Med. 2021;203. doi:10.1164/ajrccm-conference.2021.TP121	No	Not meeting inclusion criteria	Title and abstract screening
868	Bandela M, Letsiou E, Natarajan V, Ware LB, Garcia JGN, Singla S, et al. Cortactin Modulates Lung Endothelial Apoptosis Induced by Cigarette Smoke. Cells. 2021;10. doi:10.3390/cells10112869	No	Not meeting inclusion criteria	Title and abstract screening
869	Bandi P, Asare S, Majmundar A, Nargis N, Jemal A, Fedewa SA. Relative Harm Perceptions of E-Cigarettes Versus Cigarettes, U.S. Adults, 2018-2020. Am J Prev Med. 2022;63: 186–194. doi:10.1016/j.amepre.2022.03.019	No	Not meeting inclusion criteria	Title and abstract screening
870	Bandi P, Star J, Minihan AK, Patel M, Nargis N, Jemal A. Changes in E-Cigarette Use Among U.S. Adults, 2019-2021. Am J Prev Med. 2023;65: 322–326. doi:10.1016/j.amepre.2023.02.026	No	Not meeting inclusion criteria	Title and abstract screening
871	Banks E., Yazidjoglou A., Brown S., Joshy G., Martin M. SMOKING CESSATION AND E-CIGARETTES. Aust N Z J Psychiatry. 2022;56: 16. doi:10.1177/00048674221088686	No	Not meeting inclusion criteria	Title and abstract screening
872	Banks E, Yazidjoglou A, Brown S, Nguyen M, Martin M, Beckwith K, et al. Electronic cigarettes and health outcomes: umbrella and systematic review of the global evidence. Med J Aust. 2023;218: 267–275. doi:10.5694/mja2.51890	No	Not meeting inclusion criteria	Title and abstract screening
873	Bansal-Travers M, Price SM, Bold KW, Villanti AC, Barnes A, Chansky M, et al. Common Measures to Evaluate Flavored Tobacco Products: Recommendations from the Tobacco Centers for Regulatory Science (TCORS) Flavored Tobacco Products Measurement Subcommittee. Nicotine Tob Res. 2022. doi:10.1093/ntr/ntac171	No	Not meeting inclusion criteria	Title and abstract screening
874	Bansal-Travers M, Price SM, Bold KW, Villanti AC, Barnes A, Chansky M, et al. Common Measures to Evaluate Flavored Tobacco Products: Recommendations From the Tobacco Centers for Regulatory Science (TCORS) Flavored Tobacco Products Measurement Subcommittee. Nicotine Tob Res. 2023;25: 159–163. doi:10.1093/ntr/ntac171	No	Not meeting inclusion criteria	Title and abstract screening
875	Bansal-Travers M, Rivard C, Silveira ML, Kimmel H, Poonai K, Bernat JK, et al. Factors associated with changes in flavored tobacco products used: Findings from wave 2 and wave 3 (2014-2016) of the population assessment of tobacco and health (PATH) study. Addict Behav. 2022;130: 107290. doi:10.1016/j.addbeh.2022.107290	No	Not meeting inclusion criteria	Title and abstract screening
876	Bao Y, Yang X, Men Y, Kang J, Sun X, Zhao M, et al. Postoperative radiotherapy improves survival of patients with ypN2 non-small cell lung cancer after neoadjuvant chemotherapy followed by surgery – A propensity score matching study of the Surveillance, Epidemiology, and End Results database. Thorac Cancer. 2022;13: 404–411. doi:10.1111/1759-7714.14273	No	Not meeting inclusion criteria	Title and abstract screening
877	Baptista M, Barata J, Craveiro AP, Guimarães S, Valente MJ. A 30-Year-Old Man With Cough, Dyspnea, and Upper Lobe Opacities. Chest. 2022;161: e359–e363. doi:10.1016/j.chest.2021.12.671	No	Not meeting inclusion criteria	Title and abstract screening
878	Bar-Sela G, Tur-Sinai A, Givon-Schaham N, Bentur N. Advance Care Planning and Attainment of Cancer Patients' End-of-Life Preferences: Relatives' Perspective. Am J Hosp Palliat Med. 2023;40: 322–328. doi:10.1177/10499091221099921	No	Not meeting inclusion criteria	Title and abstract screening
879	Baraona LK, Lovelace D, Daniels JL, McDaniel L. Tobacco Harms, Nicotine Pharmacology, and Pharmacologic Tobacco Cessation Interventions for Women. J Midwifery Womens Health. 2017;62: 253–269. doi:10.1111/jmwh.12616	No	Not meeting inclusion criteria	Title and abstract screening
880	Barat E, Vanbergue B, Leguillon R, Chenailler C, Arrii M, Curado J, et al. [Tobacco and surgery: A survey of health professionals' practices and knowledge. A role for the community pharmacist]. Ann Pharm Fr. 2023;81: 1082–1089. doi:10.1016/j.pharma.2023.07.005	No	Not meeting inclusion criteria	Title and abstract screening

881	Bares CB, Dick DM, Kendler KS. Nicotine dependence, internalizing symptoms, mood variability and daily tobacco use among young adult smokers. Addict Behav. 2018;83: 87–94. doi:10.1016/j.addbeh.2017.09.004	No	Not meeting inclusion criteria	Title and abstract screening
882	Barkat SS, Tellier SM, Eloma AS. Varenicline for cessation from nicotine-containing electronic cigarettes. Am J Health Syst Pharm. 2019;76: 1894–1895. doi:10.1093/ajhp/zxz218	No	Not meeting inclusion criteria	Title and abstract screening
883	Barker HE, Czaplicki L, Cui Y, Shen R, Nian Q, Xie M, et al. Exposure to and appeal of tobacco ads and displays in China: a qualitative exploration of Chinese youth perceptions. Nicotine Tob Res Off J Soc Res Nicotine Tob. 2023. doi:10.1093/ntr/ntad192	No	Not meeting inclusion criteria	Title and abstract screening
884	Barnes M.N., Eller Z.M., Layman I., Rizwan M., Sumon M.R., Poddar V. Case Report: Severe Empyema Thoracis: A Rare Complication of Mycobacterium Fortuitum. Am J Respir Crit Care Med. 2021;203. doi:10.1164/ajrccm-conference.2021.TP99	No	Not meeting inclusion criteria	Title and abstract screening
885	Barnoya J, Monzon D, Pinetta J, Grilo G, Cohen JE. New tobacco products, old advertising strategies: point-of-sale advertising in Guatemala. Tob Control. 2021;30: 591–593. doi:10.1136/tobaccocontrol-2020-055681	No	Not meeting inclusion criteria	Title and abstract screening
886	Barrett R. Adverse-event management and reporting for electronic cigarettes (e-cigarettes). Eur J Hosp Pharm. 2019;26: 2–3. doi:10.1136/ejhpharm-2018-001747	No	Not meeting inclusion criteria	Title and abstract screening
887	BARRINGER A., THORPE K.I.R.K., WAKWE W., BHAMRAH P.S., BHAMRAH M., RAVI S. CATAMENIAL PNEUMOTHORAX REVISITED: A CASE REPORT. Chest. 2023;164: A3726–A3727. doi:10.1016/j.chest.2023.07.2424	No	Not meeting inclusion criteria	Title and abstract screening
888	Barrington-Trimis J.L., Liu F., Unger J.B., Alonzo T., Cruz T.B., Urman R., et al. Evaluating the predictive value of measures of susceptibility to tobacco and alternative tobacco products. Addict Behav. 2019;96: 50–55. doi:10.1016/j.addbeh.2019.03.017	No	Not meeting inclusion criteria	Title and abstract screening
889	Barrington-Trimis JL, Leventhal AM. Adolescents' Use of "Pod Mod" E-Cigarettes - Urgent Concerns. N Engl J Med. 2018;379: 1099–1102. doi:10.1056/NEJMp1805758	No	Not meeting inclusion criteria	Title and abstract screening
890	Barry A.R. Do the benefits of electronic cigarettes outweigh the risks?: The "cON" side. Can J Hosp Pharm. 2018;71: 45–47.	No	Not meeting inclusion criteria	Title and abstract screening
891	Barton A.K. E-cigarettes: informing the conversation with patients. Prescriber. 2021;32: 21–27. doi:10.1002/psb.1917	No	Not meeting inclusion criteria	Title and abstract screening
892	Barton K, Bogetz J, Trowbridge A, Taylor M, Kingsley J, Walsh C, et al. "It's the Thing I Don't Want to Talk About" to "You Have to Talk About Dying if You Have Cancer": Adolescent and Young Adult and Parent Perspectives on End-of-Life Discussions (RP416). J Pain Symptom Manage. 2022;63: 1091–1092. doi:10.1016/j.jpainsymman.2022.04.057	No	Not meeting inclusion criteria	Title and abstract screening
893	Bartsch A.-L., Carlsen L.M., Harter M., Brutt A.L., Buchholz A. A cross-sectional survey of factors associated with the uptake of smoking cessation aids among smokers and ex-smokers. J Subst Use. 2018;23: 597–602. doi:10.1080/14659891.2018.1459906	No	Not meeting inclusion criteria	Title and abstract screening
894	Bartsch P, Delvaux M, Englebert E, Beaupain MH, Louis R. [E-cigarette and smoking cessation : current situation in Belgium]. Rev Med Liege. 2017;72: 14–19.	No	Not meeting inclusion criteria	Title and abstract screening
895	Barua R.S., Rigotti N.A., Benowitz N.L., Cummings K.M., Jazayeri M.-A., Morris P.B., et al. 2018 ACC Expert Consensus Decision Pathway on Tobacco Cessation Treatment: A Report of the American College of Cardiology Task Force on Clinical Expert Consensus Documents. J Am Coll Cardiol. 2018;72: 3332–3365. doi:10.1016/j.jacc.2018.10.027	No	Not meeting inclusion criteria	Title and abstract screening
896	Barufaldi LA, Guerra RL, Albuquerque RCR, Nascimento AD, Chança RD, Souza MC, et al. [Risk of initiation to smoking with the use of electronic cigarettes: systematic review and meta-analysis]. Cien Saude Colet. 2021;26: 6089–6103. doi:10.1590/1413-812320212612.35032020	No	Not meeting inclusion criteria	Title and abstract screening
897	Basnayake TL, Valery PC, Carson P, De Ieso PB. Treatment and outcomes for indigenous and non-indigenous lung cancer patients in the Top End of the Northern Territory. Intern Med J. 2021;51: 1081–1091. doi:10.1111/imj.14961	No	Not meeting inclusion criteria	Title and abstract screening
898	Bassil N.K., Ohanian M.L.K., Bou Saba T.G. Nicotine Use Disorder in Older Adults. Clin Geriatr Med. 2022;38: 119–131. doi:10.1016/j.cger.2021.07.008	No	Not meeting inclusion criteria	Title and abstract screening
899	Bastian L.A., Driscoll M., DeRycke E., Edmond S., Mattocks K., Goulet J., et al. Pain and smoking study (PASS): A comparative effectiveness trial of smoking cessation counseling for veterans with chronic pain. Contemp Clin Trials Commun. 2021;23: 100839. doi:10.1016/j.conctc.2021.100839	No	Not meeting inclusion criteria	Title and abstract screening
900	Bates CD. POINT: e-Cigarette Use for Harm Reduction in Tobacco Use Disorder? Yes. Chest. 2021;160: 807–809. doi:10.1016/j.chest.2021.04.046	No	Not meeting inclusion criteria	Title and abstract screening
901	Bates JHT, Hamlington KL, Garrison G, Kinsey CM. Prediction of lung cancer risk based on age and smoking history. Comput Methods Programs Biomed. 2022;216: 106660. doi:10.1016/j.cmpb.2022.106660	No	Not meeting inclusion criteria	Title and abstract screening
902	Batra A., Kiefer F., Andreas S., Gohlke H., Klein M., Kotz D., et al. S3 Guideline "Smoking and Tobacco Dependence: Screening, Diagnosis, and Treatment" - Short Version. Eur Addict Res. 2022;28: 382–400. doi:10.1159/000525265	No	Not meeting inclusion criteria	Title and abstract screening
903	Battiato S, Caponnetto P, Leotta R, Marotta G, Midolo A, Ortis A, et al. Development and User Evaluation of a Food-recognition app (FoodRec): Experimental Data and Qualitative Analysis. Health Psychol Res. 2023;11: 70401. doi:10.52965/001c.70401	No	Not meeting inclusion criteria	Title and abstract screening
904	Bauer-Kemeny C., Kreuter M. Drug support in smoking cessation. Atemwegs- Lungenkrankh. 2019;45: 342–349. doi:10.5414/ATX02356	No	Not meeting inclusion criteria	Title and abstract screening
905	Bauer-Kemeny C., Kreuter M. Addictive inhalants-A challenge for the lungs. Pneumologe. 2022;19: 49–59. doi:10.1007/s10405-021-00428-8	No	Not meeting inclusion criteria	Title and abstract screening
906	Baum J, Zickler D, Bolbrinker J, Richter R, Braicu EI, Grabowski J, et al. Olaparib in an ovarian cancer patient with end-stage renal disease and hemodialysis. Cancer Chemother Pharmacol. 2023;91: 325–330. doi:10.1007/s00280-023-04514-x	No	Not meeting inclusion criteria	Title and abstract screening
907	Baum LVM, Rosenblum R, Scarborough B, Smith CB. Evaluating end-of-life chemotherapy for solid tumor and hematologic malignancy patients. Prog Palliat Care. 2021;29: 337–341. doi:10.1080/09699260.2021.1872138	No	Not meeting inclusion criteria	Title and abstract screening
908	Baumlin N., Kim M., Chung S., Whitney P., Sabater J.R., Abraham W.M., et al. E-liquid with nicotine causes mucociliary dysfunction via TRPA1 receptors in a novel large animal model of exposure. Am J Respir Crit Care Med. 2019;199. Available: https://www.atsjournals.org/doi/abs/10.1164/ajrccm-conference.2019.199.1_MeetingAbstracts.A4188	No	Not meeting inclusion criteria	Title and abstract screening
909	Bavel NV, Lai P, Loebenberg R, Prenner EJ. Cholesterol enhances the negative impact of vaping additives on lung surfactant model systems. Nanomed. 2022;17: 2231–2243. doi:10.2217/nnm-2022-0232	No	Not meeting inclusion criteria	Title and abstract screening

910	Baxter K. TRUST YOUR INSTINCTS: DIAGNOSTIC DIFFICULITIES OF ADVANVED LUNG CANCER IN A YOUNG MALE. Chest. 2021;160: A1509. doi:10.1016/j.chest.2021.07.1379	No	Not meeting inclusion criteria	Title and abstract screening
911	Beard E, Brown J, Jackson SE, Tattan-Birch H, Shahab L. Differences between ethnic groups in self-reported use of e-cigarettes and nicotine replacement therapy for cutting down and temporary abstinence: a cross-sectional population-level survey in England. Addiction. 2021;116: 2476–2485. doi:10.1111/add.15431	No	Not meeting inclusion criteria	Title and abstract screening
912	Beard E, Brown J, Shahab L. Association of quarterly prevalence of e-cigarette use with ever regular smoking among young adults in England: a time-series analysis between 2007 and 2018. Addiction. 2022;117: 2283–2293. doi:10.1111/add.15838	No	Not meeting inclusion criteria	Title and abstract screening
913	Beard E, Jackson SE, West R, Kuipers MAG, Brown J. Trends in Attempts to Quit Smoking in England Since 2007: A Time Series Analysis of a Range of Population-Level Influences. Nicotine Tob Res Off J Soc Res Nicotine Tob. 2020;22: 1476–1483. doi:10.1093/ntr/ntz141	No	Not meeting inclusion criteria	Title and abstract screening
914	Beasley MB. Acute lung injury-from cannabis to COVID. Mod Pathol. 2022;35: 1–7. doi:10.1038/s41379-021-00915-6	No	Not meeting inclusion criteria	Title and abstract screening
915	Beatrice F, Massaro G. Exhaled Carbon Monoxide Levels in Forty Resistant to Cessation Male Smokers after Six Months of Full Switch to Electronic Cigarettes (e-Cigs) or to A Tobacco Heating Systems (THS). Int J Environ Res Public Health. 2019;16. doi:10.3390/ijerph16203916	No	Not meeting inclusion criteria	Title and abstract screening
916	Beaudet M-É, Lacasse Y, Labbé C. Palliative Systemic Therapy Given near the End of Life for Metastatic Non-Small Cell Lung Cancer. Curr Oncol. 2022;29: 1316–1325. doi:10.3390/curroncol29030112	No	Not meeting inclusion criteria	Title and abstract screening
917	Becerra BJ, Arias D, Becerra MB. Sex-Specific Association between Environmental Tobacco Smoke Exposure and Asthma Severity among Adults with Current Asthma. Int J Env Res Public Health. 2022;19. doi:10.3390/ijerph19095036	No	Not meeting inclusion criteria	Title and abstract screening
918	Bechthold A, Azuero A, Pisu M, Pierce J, Williams G, Taylor R, et al. The Project ENABLE Cornerstone randomized controlled trial: study protocol for a lay navigator-led, early palliative care coaching intervention for African American and rural-dwelling advanced cancer family caregivers. 2022;23. doi:10.1186/s13063-022-06305-w	No	Not meeting inclusion criteria	Title and abstract screening
919	Beck DC, Boyd CJ, Evans-Polce R, McCabe SE, Veliz PT. An examination of how e-cigarette/cigarette use during adolescence is associated with future use during the third trimester of pregnancy. Subst Abus. 2022;43: 344–348. doi:10.1080/08897077.2021.1941519	No	Not meeting inclusion criteria	Title and abstract screening
920	Becker T.D., Arnold M.K., Ro V., Martin L., Rice T.R. Systematic Review of Electronic Cigarette Use (Vaping) and Mental Health Comorbidity among Adolescents and Young Adults. Nicotine Tob Res. 2021;23: 415–425. doi:10.1093/ntr/ntaa171	No	Not meeting inclusion criteria	Title and abstract screening
921	Bedi MK, Bedi DK, Ledgerwood DM. Gender Differences in Reasons for Using Electronic Cigarettes: A Systematic Review. Nicotine Tob Res. 2022;24: 1355–1362. doi:10.1093/ntr/ntac108	No	Not meeting inclusion criteria	Title and abstract screening
922	Bednarczuk N., Williams E.E., Dassios T., Greenough A. Nicotine replacement therapy and e-cigarettes in pregnancy and infant respiratory outcomes. Early Hum Dev. 2022;164: 105509. doi:10.1016/j.earlhumdev.2021.105509	No	Not meeting inclusion criteria	Title and abstract screening
923	Begh R, Aveyard P. Rebuttal from Rachna Begh and Paul Aveyard. J Physiol. 2020;598: 3057. doi:10.1113/JP280092	No	Not meeting inclusion criteria	Title and abstract screening
924	Begh R., Aveyard P. CrossTalk proposal: The benefits of e-cigarettes outweigh the harms. J Physiol. 2020;598: 3049–3051. doi:10.1113/JP279270	No	Not meeting inclusion criteria	Title and abstract screening
925	Begum R., Batra S., Obuya J., Thota S. Urolithins Rescue E-Cigarette Condensate Induced DAMPs Mediated Signaling. Curr Dev Nutr. 2023;7: 101463. doi:10.1016/j.cdnut.2023.101463	No	Not meeting inclusion criteria	Title and abstract screening
926	Behl D. PPD01.01 Patterns of Use of Medical Cannabis in Lung Cancer and Other Cancer Patients. J Thorac Oncol. 2023;18: e5. doi:10.1016/j.jtho.2022.09.017	No	Not meeting inclusion criteria	Title and abstract screening
927	Beklen A., Yildirim B.G., Mimaroglu M., Yavuz M.B. The impact of smoking on oral health and patient assessment of tobacco cessation support from turkish dentists. Tob Induc Dis. 2021;19: A1. doi:10.18332/TID/136418	No	Not meeting inclusion criteria	Title and abstract screening
928	Belfleur L, Sonavane M, Hernandez A, Gassman NR, Migaud ME. Solution Chemistry of Dihydroxyacetone and Synthesis of Monomeric Dihydroxyacetone. Chem Res Toxicol. 2022;35: 616–625. doi:10.1021/acs.chemrestox.1c00403	No	Not meeting inclusion criteria	Title and abstract screening
929	Bell S., Dean J., Gilks C., Boyd M.A., Fitzgerald L., Mutch A., et al. Tobacco harm reduction with vaporised nicotine (THRiVe): The study protocol of an uncontrolled feasibility study of novel nicotine replacement products among people living with HIV who smoke. Int J Environ Res Public Health. 2017;14: 799. doi:10.3390/ijerph14070799	No	Not meeting inclusion criteria	Title and abstract screening
930	Bellenguez Y., Chevalier F., Duhaut P., Colpart E., Dernoncourt A. Smoking cessation in secondary prevention of acute coronary syndrome: The role of the electronic cigarette. Ann Cardiol Angeiol (Paris). 2019;68: 39–48. doi:10.1016/j.ancard.2018.08.029	No	Not meeting inclusion criteria	Title and abstract screening
931	Bellisario A, Bourbeau K, Crespo DA, DeLuzio N, Ferro A, Sanchez A, et al. An Observational Study of Vaping Knowledge and Perceptions in a Sample of U.S. Adults. Cureus. 2020;12: e8800. doi:10.7759/cureus.8800	No	Not meeting inclusion criteria	Title and abstract screening
932	Belsare P, Senyurek VY, Imtiaz MH, Betts J, Motschman CA, Dowd AN, et al. Analyzing Impact of Mouthpiece-based Puff Topography Devices on Smoking Behavior using Wearable Sensors. Annu Int Conf IEEE Eng Med Biol Soc. 2022;2022: 1787–1791. doi:10.1109/EMBC48229.2022.9871589	No	Not meeting inclusion criteria	Title and abstract screening
933	Ben Taleb Z., Kalan M.E., Bahelah R., Boateng G.O., Rahman M., Alshbool F.Z. Vaping while high: Factors associated with vaping marijuana among youth in the United States. Drug Alcohol Depend. 2020;217: 108290. doi:10.1016/j.drugalcdep.2020.108290	No	Not meeting inclusion criteria	Title and abstract screening
934	Benavides-Vasquez J, Shen C, McDonald A, Wang L. Effect of residence in nonmetropolitan counties on stage and tumor size at diagnosis in patients with breast cancer: A Surveillance, Epidemiology, and End Results analysis. J Rural Health. 2023;39: 408–415. doi:10.1111/jrh.12727	No	Not meeting inclusion criteria	Title and abstract screening
935	Benites AC, Arantes de Oliveira-Cardoso É, dos Santos MA. Spirituality in Brazilian family caregivers of patients with cancer from the end-of-life care to bereavement. Death Stud. 2023;47: 249–258. doi:10.1080/07481187.2022.2051095	No	Not meeting inclusion criteria	Title and abstract screening
936	Benites AC, Rodin G, de Oliveira-Cardoso ÉA, dos Santos MA. "You begin to give more value in life, in minutes, in seconds": spiritual and existential experiences of family caregivers of patients with advanced cancer receiving end-of-life care in Brazil. Support Care Cancer. 2022;30: 2631–2638. doi:10.1007/s00520-021-06712-w	No	Not meeting inclusion criteria	Title and abstract screening
937	Benmarhnia T., Pierce J.P., Leas E., White M.M., Strong D.R., Noble M.L., et al. Can E-Cigarettes and Pharmaceutical AIDS Increase Smoking Cessation and Reduce Cigarette Consumption? Findings from a Nationally Representative Cohort of American Smokers. Am J Epidemiol. 2018;187: 2397–2404. doi:10.1093/aje/kwy129	No	Not meeting inclusion criteria	Title and abstract screening
938	Bennet TJ, Randhawa A, Hua J, Cheung KC. Airway-On-A-Chip: Designs and Applications for Lung Repair and Disease. Cells. 2021;10. doi:10.3390/cells10071602	No	Not meeting inclusion criteria	Title and abstract screening

939	Bennett B, Romm KF, Berg CJ. Changes in cigarette and e-cigarette use among US young adults from before to during the COVID-19 pandemic: News exposure and risk perceptions as potential predictors. Tob Prev Cessat. 2022;8: 18. doi:10.18332/tpc/148245	No	Not meeting inclusion criteria	Title and abstract screening
940	Bennett M, Speer J, Taylor N, Alexander T. Changes in E-cigarette Use Among Youth and Young Adults During the COVID-19 Pandemic: Insights Into Risk Perceptions and Reasons for Changing Use Behavior. Nicotine Tob Res. 2023;25: 350–355. doi:10.1093/ntr/ntac136	No	Not meeting inclusion criteria	Title and abstract screening
941	Bennett WD, Clapp PW, Holbrook LT, Zeman KL. Respiratory Tract Deposition of E-Cigarette Particles. Compr Physiol. 2022;12: 3823–3832. doi:10.1002/cphy.c210038	No	Not meeting inclusion criteria	Title and abstract screening
942	Benowitz N.L. The Central Role of pH in the Clinical Pharmacology of Nicotine: Implications for Abuse Liability, Cigarette Harm Reduction and FDA Regulation. Clin Pharmacol Ther. 2022;111: 1004–1006. doi:10.1002/cpt.2555	No	Not meeting inclusion criteria	Title and abstract screening
943	Benowitz N.L., St.Helen G., Liakoni E. Clinical Pharmacology of Electronic Nicotine Delivery Systems (ENDS): Implications for Benefits and Risks in the Promotion of the Combusted Tobacco Endgame. J Clin Pharmacol. 2021;61: S18–S36. doi:10.1002/jcph.1915	No	Not meeting inclusion criteria	Title and abstract screening
944	Benowitz NL, Donny EC, Hatsukami DK. Reduced nicotine content cigarettes, e-cigarettes and the cigarette end game. Addict Abingdon Engl. 2017;112: 6–7. doi:10.1111/add.13534	No	Not meeting inclusion criteria	Title and abstract screening
945	Benowitz NL. Comprehensive Nicotine Regulation to End the Combustible Tobacco Epidemic. Ann Intern Med. 2017;167: 736–737. doi:10.7326/M17-2071	No	Not meeting inclusion criteria	Title and abstract screening
946	Benowitz NL. E-cigarettes and dual nicotine replacement therapy for smoking cessation. Lancet Respir Med. 2020;8: 7–8. doi:10.1016/S2213-2600(19)30308-X	No	Not meeting inclusion criteria	Title and abstract screening
947	Benowitz NL, Goniewicz ML, Halpern-Felsher B, Krishnan-Sarin S, Ling PM, O'Connor RJ, et al. Tobacco product use and the risks of SARS-CoV-2 infection and COVID-19: current understanding and recommendations for future research. Lancet Respir Med. 2022;10: 900–915. doi:10.1016/S2213-2600(22)00182-5	No	Not meeting inclusion criteria	Title and abstract screening
948	Benowitz NL, Havel C, Jacob P, O'Shea DF, Wu D, Fowles J. Vaping THC-O Acetate: Potential for Another EVALI Epidemic. J Med Toxicol. 2023;19: 37–39. doi:10.1007/s13181-022-00921-3	No	Not meeting inclusion criteria	Title and abstract screening
949	Benowitz NL, Henningfield JE. Nicotine Reduction Strategy: State of the science and challenges to tobacco control policy and FDA tobacco product regulation. Prev Med. 2018;117: 5–7. doi:10.1016/j.ypmed.2018.06.012	No	Not meeting inclusion criteria	Title and abstract screening
950	Benowitz NL, Liakoni E. Tobacco use disorder and cardiovascular health. Addiction. 2022;117: 1128–1138. doi:10.1111/add.15703	No	Not meeting inclusion criteria	Title and abstract screening
951	Benowitz NL, St Helen G, Liakoni E. Clinical Pharmacology of Electronic Nicotine Delivery Systems (ENDS): Implications for Benefits and Risks in the Promotion of the Combusted Tobacco Endgame. J Clin Pharmacol. 2021;61 Suppl 2: S18–S36. doi:10.1002/jcph.1915	No	Not meeting inclusion criteria	Title and abstract screening
952	Benson R, Hu M, Chen AT, Zhu SH, Conway M. Examining Cannabis, Tobacco, and Vaping Discourse on Reddit: An Exploratory Approach Using Natural Language Processing. Front Public Health. 2021;9: 738513. doi:10.3389/fpubh.2021.738513	No	Not meeting inclusion criteria	Title and abstract screening
953	Bentivegna K, Atuegwu NC, Oncken C, DiFranza JR, Mortensen EM. Electronic Cigarettes Associated With Incident and Polysubstance Use Among Youth. J Adolesc Health. 2021;68: 123–129. doi:10.1016/j.jadohealth.2020.05.026	No	Not meeting inclusion criteria	Title and abstract screening
954	Bentivegna K, Goniewicz ML, Waldman RA. Letter in Reply: Promoting accurate public health messages about electronic cigarettes: E-cigs contain carcinogens. J Am Acad Dermatol. 2023;88: e37. doi:10.1016/j.jaad.2019.11.054	No	Not meeting inclusion criteria	Title and abstract screening
955	Benyo SE, Bruinsma TJ, Drda E, Brady-Olympia J, Hicks SD, Boehmer S, et al. Risk Factors and Medical Symptoms Associated With Electronic Vapor Product Use Among Adolescents and Young Adults. Clin Pediatr (Phila). 2021;60: 279–289. doi:10.1177/00099228211009681	No	Not meeting inclusion criteria	Title and abstract screening
956	Berg C.J., Haardorfer R., Payne J.B., Getachew B., Vu M., Guttentag A., et al. Ecological momentary assessment of various tobacco product use among young adults. Addict Behav. 2019;92: 38–46. doi:10.1016/j.addbeh.2018.12.014	No	Not meeting inclusion criteria	Title and abstract screening
957	Berg CJ, Payne J, Henriksen L, Cavazos-Rehg P, Getachew B, Schauer GL, et al. Reasons for Marijuana and Tobacco Co-use Among Young Adults: A Mixed Methods Scale Development Study. Subst Use Misuse. 2018;53: 357–369. doi:10.1080/10826084.2017.1327978	No	Not meeting inclusion criteria	Title and abstract screening
958	Berg CJ, Callanan R, Johnson TO, Schliecher NC, Sussman S, Wagener TL, et al. Vape shop and consumer activity during COVID-19 non-essential business closures in the USA. Tob Control. 2021;30: e41–e44. doi:10.1136/tobaccocontrol-2020-056171	No	Not meeting inclusion criteria	Title and abstract screening
959	Berg CJ, Krishnan N, Graham AL, Abroms LC. A synthesis of the literature to inform vaping cessation interventions for young adults. Addict Behav. 2021;119: 106898. doi:10.1016/j.addbeh.2021.106898	No	Not meeting inclusion criteria	Title and abstract screening
960	Berg CJ, Melena A, Wittman FD, Robles T, Henriksen L. The Reshaping of the E-Cigarette Retail Environment: Its Evolution and Public Health Concerns. Int J Env Res Public Health. 2022;19. doi:10.3390/ijerph19148518	No	Not meeting inclusion criteria	Title and abstract screening
961	Berg CJ, Romm KF, Bar-Zeev Y, Abroms LC, Klinkhammer K, Wysota CN, et al. IQOS marketing strategies in the USA before and after US FDA modified risk tobacco product authorisation. Tob Control. 2023;32: 418–427. doi:10.1136/tobaccocontrol-2021-056819	No	Not meeting inclusion criteria	Title and abstract screening
962	Berg CJ, Romm KF, Barker DC, Schleicher N, Johnson TO, Wang Y, et al. Changes in the Point-of-Sale Among Vape Shops in Six U.S. Metropolitan Areas Over Time, 2018-2021. Nicotine Tob Res. 2023;25: 1369–1377. doi:10.1093/ntr/ntad046	No	Not meeting inclusion criteria	Title and abstract screening
963	Berg CJ, Romm KF, Patterson B, Wysota CN. Heated Tobacco Product Awareness, Use, and Perceptions in a Sample of Young Adults in the United States. Nicotine Tob Res. 2021;23: 1967–1971. doi:10.1093/ntr/ntab058	No	Not meeting inclusion criteria	Title and abstract screening
964	Bergen A.W., Do E.K., Chen L.-S., David S.P. Tobacco genomics: Complexity and translational challenges. Nicotine Tob Res. 2018;21: 705–706. doi:10.1093/ntr/ntz033	No	Not meeting inclusion criteria	Title and abstract screening
965	Bergström H, Helde Frankling M, Klasson C, Lövgren Sandblom A, Diczfalusy U, Björkhem-Bergman L. CYP3A Activity in End-of-Life Cancer Patients Measured by 4β-Hydroxycholesterol/cholesterol Ratio, in Men and Women. Cancers. 2021;13: 4689. doi:10.3390/cancers13184689	No	Not meeting inclusion criteria	Title and abstract screening
966	Berlin I. Risk of COVID-19 and smoking. Heart. 2021;107: 170. doi:10.1136/heartjnl-2020-318311	No	Not meeting inclusion criteria	Title and abstract screening
967	Berlin I., Dautzenberg B., Lehmann B., Palmyre J., Liegey E., De Rycke Y., et al. Randomised, placebo-controlled, double-blind, double-dummy, multicentre trial comparing electronic cigarettes with nicotine to varenicline and to electronic cigarettes without nicotine: The ECSMOKE trial protocol. BMJ Open. 2019;9: e028832. doi:10.1136/bmjopen-2018-028832	No	Not meeting inclusion criteria	Title and abstract screening

968	Bernat D, Gasquet N, Wilson KO, Porter L, Choi K. Electronic Cigarette Harm and Benefit Perceptions and Use Among Youth. Am J Prev Med. 2018;55: 361–367. doi:10.1016/j.amepre.2018.04.043	No	Not meeting inclusion criteria	Title and abstract screening
969	Bernat JK, Ferrer RA, Margolis KA, Blake KD. US adult tobacco users' absolute harm perceptions of traditional and alternative tobacco products, information-seeking behaviors, and (mis)beliefs about chemicals in tobacco products. Addict Behav. 2017;71: 38–45. doi:10.1016/j.addbeh.2017.02.027	No	Not meeting inclusion criteria	Title and abstract screening
970	Bernstein MH, Oueidat K, Wasserman P, Agarwal S, Baird GL, Sokolovsky A, et al. Electronic Cigarettes for Smoking Cessation: The Gap Between Behavior in Smokers and Medical Education. Cureus. 2022;14: e29603. doi:10.7759/cureus.29603	No	Not meeting inclusion criteria	Title and abstract screening
971	Bernstein SL, Dziura J, Weiss J, Brooks AH, Miller T, Vickerman KA, et al. Successful Optimization of Tobacco Dependence Treatment in the Emergency Department: A Randomized Controlled Trial Using the Multiphase Optimization Strategy. Ann Emerg Med. 2023;81: 209–221. doi:10.1016/j.annemergmed.2022.08.018	No	Not meeting inclusion criteria	Title and abstract screening
972	Berridge V, Hall W, Taylor S, Gartner C, Morphett K. A first pass, using pre-history and contemporary history, at understanding why Australia and England have such different policies towards electronic nicotine delivery systems, 1970s-c. 2018. Addiction. 2021;116: 2577–2585. doi:10.1111/add.15391	No	Not meeting inclusion criteria	Title and abstract screening
973	Berridge V. History and the future of addiction. Int J Drug Policy. 2021;94: 103054. doi:10.1016/j.drugpo.2020.103054	No	Not meeting inclusion criteria	Title and abstract screening
974	Berry C, Burton S. Reduced-Risk Warnings Versus the US FDA-Mandated Addiction Warning: the Effects of E-Cigarette Warning Variations on Health Risk Perceptions. 2019;21: 979-984. doi:10.1093/ntr/nty177	No	Not meeting inclusion criteria	Title and abstract screening
975	Berry C., Burton S., Howlett E. Are cigarette smokers', e-cigarette users', and dual users' health-risk beliefs and responses to advertising influenced by addiction warnings and product type? Nicotine Tob Res. 2017;19: 1185–1191. doi:10.1093/ntr/ntx075	No	Not meeting inclusion criteria	Title and abstract screening
976	Berry C, Burton S, Howlett E. The impact of e-cigarette addiction warnings and health-related claims on consumers' risk beliefs and use intentions. J Public Policy Mark. 2017;36: 54–69. doi:10.1509/jppm.15.024https://dx.doi.org/10.1509/jppm.15.024	No	Not meeting inclusion criteria	Title and abstract screening
977	Bertoni N, Cavalcante TM, Souza MC, Szklo AS. Prevalence of electronic nicotine delivery systems and waterpipe use in Brazil: where are we going? Rev Bras Epidemiol. 2021;24: e210007. doi:10.1590/1980-549720210007.supl.2	No	Not meeting inclusion criteria	Title and abstract screening
978	Bertoni N, Szklo AS. [Electronic nicotine delivery systems in Brazilian state capitals: prevalence, profile of use, and implications for the National Tobacco Control Policy]. Cad Saude Publica. 2021;37: e00261920. doi:10.1590/0102-311X00261920	No	Not meeting inclusion criteria	Title and abstract screening
979	Berube L, Duffy VB, Hayes JE, Hoffman HJ, Rawal S. Associations between chronic cigarette smoking and taste function: Results from the 2013-2014 national health and nutrition examination survey. Physiol Behav. 2021;240: 113554. doi:10.1016/j.physbeh.2021.113554	No	Not meeting inclusion criteria	Title and abstract screening
980	Besaratinia A. COVID-19: a pandemic converged with global tobacco epidemic and widespread vaping-state of the evidence. Carcinogenesis. 2021;42: 1009–1022. doi:10.1093/carcin/bgab061	No	Not meeting inclusion criteria	Title and abstract screening
981	Besaratinia A. From Tobacco Cigarettes to Electronic Cigarettes: The Two Sides of a Nicotine Coin. Front Oral Health. 2021;2: 790634. doi:10.3389/froh.2021.790634	No	Not meeting inclusion criteria	Title and abstract screening
982	Besaratinia A., Caceres A., Tommasi S. DNA Hydroxymethylation in Smoking-Associated Cancers. Int J Mol Sci. 2022;23: 2657. doi:10.3390/ijms23052657	No	Not meeting inclusion criteria	Title and abstract screening
983	Best CS, Brown A, Hunt K. Purchasing of tobacco-related and e-cigarette-related products within prisons before and after implementation of smoke-free prison policy: analysis of prisoner spend data across Scotland, UK. BMJ Open. 2022;12: e058909. doi:10.1136/bmjopen-2021-058909	No	Not meeting inclusion criteria	Title and abstract screening
984	Bestman E.G., Brooks J.K., Mostoufi B., Bashirelahi N. What every dentist needs to know about electronic cigarettes. Gen Dent. 2021;69: 31–35.	No	Not meeting inclusion criteria	Title and abstract screening
985	Betker L, Nagelschmidt K, Leppin N, Knorrenschild JR, Volberg C, Berthold D, et al. The Difficulties in End-of-Life Discussions - Family Inventory (DEOLD-FI): Development and Initial Validation of a Self-Report Questionnaire in a Sample of Terminal Cancer Patients. J Pain Symptom Manage. 2021;62: e130–e138. doi:10.1016/j.jpainsymman.2021.04.022	No	Not meeting inclusion criteria	Title and abstract screening
986	Betts JM, Tiffany ST. Comparing the reward value of cigarettes and food during tobacco abstinence and nonabstinence. Drug Alcohol Depend. 2019;204: 107475. doi:10.1016/j.drugalcdep.2019.04.040	No	Not meeting inclusion criteria	Title and abstract screening
987	Bevins RA, Barrett ST, Huynh YW, Thompson BM, Kwan DA, Murray JE. Experimental analysis of behavior and tobacco regulatory research on nicotine reduction. J Exp Anal Behav. 2018;110: 1–10. doi:10.1002/jeab.439	No	Not meeting inclusion criteria	Title and abstract screening
988	Beznos B, Sayner R, Carpenter DM, Davis SA, Lee C, Loughlin CE, et al. Communication About Adolescent and Caregiver Smoking and Vaping During Pediatric Asthma Visits: Implications for Providers. J Pediatr Health Care. 2021;35: 401–407. doi:10.1016/j.pedhc.2021.02.004	No	Not meeting inclusion criteria	Title and abstract screening
989	Bhad R., Singh Y.C., Kalra D., Sidhu M. Nicotine Replacement Therapy for Tobacco Addiction: What every psychiatrist should know? Indian J Psychiatry. 2023;65: S53.	No	Not meeting inclusion criteria	Title and abstract screening
990	Bhalerao A, Sivandzade F, Archie SR, Cucullo L. Public Health Policies on E-Cigarettes. Curr Cardiol Rep. 2019;21: 111. doi:10.1007/s11886-019-1204-y	No	Not meeting inclusion criteria	Title and abstract screening
991	Bhat TA, Kalathil SG, Goniewicz ML, Hutson A, Thanavala Y. Not all vaping is the same: differential pulmonary effects of vaping cannabidiol versus nicotine. Thorax. 2023;78: 922–932. doi:10.1136/thorax-2022-218743	No	Not meeting inclusion criteria	Title and abstract screening
992	Bhatnagar A, Whitsel LP, Blaha MJ, Huffman MD, Krishan-Sarin S, Maa J, et al. New and Emerging Tobacco Products and the Nicotine Endgame: The Role of Robust Regulation and Comprehensive Tobacco Control and Prevention: A Presidential Advisory From the American Heart Association. Circulation. 2019;139: e937–e958. doi:10.1161/CIR.0000000000000669	No	Not meeting inclusion criteria	Title and abstract screening
993	Bhatnagar A. Editorial Commentary: The cardiovascular cost of vaping. Trends Cardiovasc Med. 2020;30: 141–142. doi:10.1016/j.tcm.2019.05.011	No	Not meeting inclusion criteria	Title and abstract screening
994	Bhatnagar A., Payne T.J., Robertson R.M. Is There A Role for Electronic Cigarettes in Tobacco Cessation? J Am Heart Assoc. 2019;8: e012742. doi:10.1161/JAHA.119.012742	No	Not meeting inclusion criteria	Title and abstract screening
995	Bhutia T.D., Mehendale A., Lad N., Vaishnav P. E-cigarettes: Gateway to tobacco addiction among adolescents. Tob Induc Dis. 2021;19. doi:10.18332/tid/140847	No	Not meeting inclusion criteria	Title and abstract screening
996	Bhutta Z.A., Hauerslev M., Farmer M., Lewis-Watts L. COVID-19, children and non-communicable diseases: Translating evidence into action. Arch Dis Child. 2021;106: 141–142. doi:10.1136/archdischild-2020-319923	No	Not meeting inclusion criteria	Title and abstract screening

997	Bianco CL, Pratt SI, Ferron JC, Brunette MF. Electronic Cigarette Use During a Randomized Trial of Interventions for Smoking Cessation Among Medicaid Beneficiaries with Mental Illness. J Dual Diagn. 2019;15: 184–191. doi:10.1080/15504263.2019.1620400	No	Not meeting inclusion criteria	Title and abstract screening
998	Bianco E., Skipalskyi A., Goma F., Odeh H., Hasegawa K., Zawawi M.A., et al. E-Cigarettes: A New Threat to Cardiovascular Health - A World Heart Federation Policy Brief. Glob Heart. 2021;16: A3. doi:10.5334/gh.1076	No	Not meeting inclusion criteria	Title and abstract screening
999	Bidwell LC, Karoly HC, Hutchison KE, Bryan AD. ADHD symptoms impact smoking outcomes and withdrawal in response to Varenicline treatment for smoking cessation. Drug Alcohol Depend. 2017;179: 18–24. doi:10.1016/j.drugalcdep.2017.06.020	No	Not meeting inclusion criteria	Title and abstract screening
1000	Bierhoff J, Haardörfer R, Windle M, Berg CJ. Psychological Risk Factors for Alcohol, Cannabis, and Various Tobacco Use among Young Adults: A Longitudinal Analysis. Subst Use Misuse. 2019;54: 1365–1375. doi:10.1080/10826084.2019.1581220	No	Not meeting inclusion criteria	Title and abstract screening
1001	BinDhim NF, Althumiri NA, Basyouni MH, AlMousa N, AlJuwaysim MF, Alhakbani A, et al. Exploring the Impact of COVID-19 Response on Population Health in Saudi Arabia: Results from the "Sharik" Health Indicators Surveillance System during 2020. Int J Environ Res Public Health. 2021;18. doi:10.3390/ijerph18105291	No	Not meeting inclusion criteria	Title and abstract screening
1002	Biondi-Zoccai G, Peruzzi M, Frati G. E-Cigarettes, Incentives, and Drugs for Smoking Cessation. N Engl J Med. 2018;379: 991–2. doi:10.1056/NEJMc1809349	No	Not meeting inclusion criteria	Title and abstract screening
1003	Birch B.R. EDITORIAL COMMENT. Urology. 2021;147: 184. doi:10.1016/j.urology.2020.05.090	No	Not meeting inclusion criteria	Title and abstract screening
1004	Birdsey J, Cornelius M, Jamal A, Park-Lee E, Cooper MR, Wang J, et al. Tobacco Product Use Among U.S. Middle and High School Students - National Youth Tobacco Survey, 2023. MMWR Morb Mortal Wkly Rep. 2023;72: 1173–1182. doi:10.15585/mmwr.mm7244a1	No	Not meeting inclusion criteria	Title and abstract screening
1005	Bishop E, East N, Miazzi F, Fiebelkorn S, Breheny D, Gaca M, et al. A contextualised e-cigarette testing strategy shows flavourings do not impact lung toxicity in vitro. Toxicol Lett. 2023;380: 1–11. doi:10.1016/j.toxlet.2023.03.006	No	Not meeting inclusion criteria	Title and abstract screening
1006	Bishop E, Terry A, East N, Breheny D, Gaca M, Thorne D. A 3D in vitro comparison of two undiluted e-cigarette aerosol generating systems. Toxicol Lett. 2022;358: 69–79. doi:10.1016/j.toxlet.2022.01.002	No	Not meeting inclusion criteria	Title and abstract screening
1007	Bista S, Lechner WV, Anderson M, Kenne KN, Kenne DR. Cigarette and e-cigarette use as a function of psychological distress following COVID-19 related university campus closures. Am J Drug Alcohol Abuse. 2023;49: 239–248. doi:10.1080/00952990.2023.2171300	No	Not meeting inclusion criteria	Title and abstract screening
1008	Bjørnelv G, Hagen TP, Forma L, Aas E. Care pathways at end-of-life for cancer decedents: registry based analyses of the living situation, healthcare utilization and costs for all cancer decedents in Norway in 2009-2013 during their last 6 months of life. BMC Health Serv Res. 2022;22: 1–13. doi:10.1186/s12913-022-08526-w	No	Not meeting inclusion criteria	Title and abstract screening
1009	Bjurlin MA, Basak R, Zambrano I, Schatz D, El Shahawy O, Sherman S, et al. Patterns and associations of smoking and electronic cigarette use among survivors of tobacco related and non-tobacco related cancers: A nationally representative cross-sectional analysis. Cancer Epidemiol. 2022;78: 101913. doi:10.1016/j.canep.2021.101913	No	Not meeting inclusion criteria	Title and abstract screening
1010	Bjurlin MA, Basak R, Zambrano I, Schatz D, El Shahawy O, Sherman S, et al. Perceptions of e-cigarette harm among cancer survivors: Findings from a nationally representative survey. Cancer Epidemiol. 2022;78: 102037. doi:10.1016/j.canep.2021.102037	No	Not meeting inclusion criteria	Title and abstract screening
1011	Bjurlin MA, Matulewicz RS, Roberts TR, Dearing BA, Schatz D, Sherman S, et al. Carcinogen Biomarkers in the Urine of Electronic Cigarette Users and Implications for the Development of Bladder Cancer: A Systematic Review. Eur Urol Oncol. 2021;4: 766–783. doi:10.1016/j.euo.2020.02.004	No	Not meeting inclusion criteria	Title and abstract screening
1012	Bjurlin MA, Kamecki H, Gordon T, Krajewski W, Matulewicz RS, Malkiewicz B, et al. Alternative tobacco products use and its impact on urologic health - will the lesser evil still be evil? A commentary and review of literature. Cent Eur J Urol. 2021;74: 152–160. doi:10.5173/ceju.2021.0110	No	Not meeting inclusion criteria	Title and abstract screening
1013	Blacker C.J. Clinical issues to consider for clozapine patients who vape: A case illustration. Focus U S. 2020;18: 55–57. doi:10.1176/appi.focus.20190025	No	Not meeting inclusion criteria	Title and abstract screening
1014	Blackwell C.W., Lopez Castillo H. Use of electronic nicotine delivery systems (ENDS) in lesbian, gay, bisexual, transgender and queer persons: Implications for public health nursing. Public Health Nurs. 2020;37: 569–580. doi:10.1111/phn.12746	No	Not meeting inclusion criteria	Title and abstract screening
1015	Blaha M.J., Ratchford E.V. Electronic cigarettes. Vasc Med U K. 2019;24: 267–269. doi:10.1177/1358863X19837361	No	Not meeting inclusion criteria	Title and abstract screening
1016	Blalock K., Breve F., Varrassi G., Magnusson P., Pergolizzi J. Vaping and subsequent comorbidities potentially associated with increased mortality and more severe illness in covid-19: A narrative review. Signa Vitae. 2021;17: 25–36. doi:10.22514/sv.2021.113	No	Not meeting inclusion criteria	Title and abstract screening
1017	Blank MD, Ozga JE, Romm KF, Douglas A, Alexander L, Doogan NJ, et al. Geographic isolation predicts tobacco product use among youth: A latent class analysis. J Rural Health. 2022;38: 373–381. doi:10.1111/jrh.12583	No	Not meeting inclusion criteria	Title and abstract screening
1018	Blank MD, Pearson J, Cobb CO, Felicione NJ, Hiler MM, Spindle TR, et al. What factors reliably predict electronic cigarette nicotine delivery? Tob Control. 2020;29: 644–651. doi:10.1136/tobaccocontrol-2019-055193	No	Not meeting inclusion criteria	Title and abstract screening
1019	Blinick R., Chaya N., Zalta B., Haramati L.B., Shmukler A. Cracking the Opium Den: Cardiothoracic Manifestations of Drug Abuse. J Thorac Imaging. 2021;36: W16–W31. doi:10.1097/RTI.0000000000000488	No	Not meeting inclusion criteria	Title and abstract screening
1020	Block AC, Schneller LM, Leigh NJ, Heo J, Goniewicz ML, O'Connor RJ. Heavy metals in ENDS: a comparison of open versus closed systems purchased from the USA, England, Canada and Australia. Tob Control. 2023. doi:10.1136/tc-2023-057932	No	Not meeting inclusion criteria	Title and abstract screening
1021	Blondino CT, Clifford JS, Lu J, Prom-Wormley EC. The association between internalizing and externalizing severity with current use of cigarettes, e-cigarettes, and alcohol in adults: Wave 1 of the Population Assessment of Tobacco and Health (PATH) study. Addict Behav. 2021;119: 106890. doi:10.1016/j.addbeh.2021.106890	No	Not meeting inclusion criteria	Title and abstract screening
1022	Bluestein MA, Bejarano G, Tackett AP, Duano JC, Rawls SG, Vandewater EA, et al. E-Cigarette Quit Attempts and Experiences in a Convenience Sample of Adult Users. Int J Environ Res Public Health. 2023;20. doi:10.3390/ijerph20032332	No	Not meeting inclusion criteria	Title and abstract screening
1023	Bluestein MA, Harrell MB, Hebert ET, Chen B, Kuk AE, Spells CE, et al. Associations Between Perceptions of e-Cigarette Harmfulness and Addictiveness and the Age of E-Cigarette Initiation Among the Population Assessment of Tobacco and Health (PATH) Youth. Tob Use Insights. 2022;15: 1179173X221133645. doi:10.1177/1179173X221133645	No	Not meeting inclusion criteria	Title and abstract screening
1024	Blume LF, Lines S. The Role of the School Nurse in Creating a Vape-Free School. NASN Sch Nurse. 2020;35: 166–172. doi:10.1177/1942602X20913261	No	Not meeting inclusion criteria	Title and abstract screening
1025	Boakye E, El Shahawy O, Obisesan O, Dzaye O, Osei AD, Erhabor J, et al. The inverse association of state cannabis vaping prevalence with the e-cigarette or vaping product-use associated lung injury. PLoS One. 2022;17: e0276187. doi:10.1371/journal.pone.0276187	No	Not meeting inclusion criteria	Title and abstract screening

1026	Boakye E, Osuji N, Erhabor J, Obisesan O, Osei AD, Mirbolouk M, et al. Assessment of Patterns in e-Cigarette Use Among Adults in the US, 2017-2020. JAMA Netw Open. 2022;5: e2223266. doi:10.1001/jamanetworkopen.2022.23266	No	Not meeting inclusion criteria	Title and abstract screening
1027	Boakye E, Obisesan OH, Osei AD, Dzaye O, Uddin SMI, Hirsch GA, et al. The Promise and Peril of Vaping. Curr Cardiol Rep. 2020;22: 155. doi:10.1007/s11886-020-01414-x	No	Not meeting inclusion criteria	Title and abstract screening
1028	Boddu S., Bojanowski C.M., Scholten E., Bosompra N.A., Advani I.N., Chen Y., et al. Investigating the effects of different forms of tobacco use on sleep quality and cough: A matter of gender? Am J Respir Crit Care Med. 2019;199. Available: https://www.atsjournals.org/doi/abs/10.1164/ajrccm-conference.2019.199.1_MeetingAbstracts.A1180	No	Not meeting inclusion criteria	Title and abstract screening
1029	Bodor JN, Patel JD, Wakelee HA, Levy BP, Borghaei H, Pellini B, et al. Phase II Randomized Trial of Carboplatin, Pemetrexed, and Bevacizumab With and Without Atezolizumab in Stage IV Nonsquamous Non-Small-Cell Lung Cancer Patients Who Harbor a Sensitizing EGFR Mutation or Have Never Smoked. Clin Lung Cancer. 2023;24: e242–e246. doi:10.1016/j.cllc.2023.05.003	No	Not meeting inclusion criteria	Title and abstract screening
1030	Bogomolov A, Zaikov S, Gogunska I, Tkhorovskyi M. HEATED TOBACCO PRODUCTS: WE STILL NEED TO KNOW A BIT MORE. Wiad Lek. 2022;75: 1771–1775. doi:10.36740/WLek202207129	No	Not meeting inclusion criteria	Title and abstract screening
1031	Bojanowski C.M., Corriden R., Chien J., Crotty Alexander L.E. Electronic cigarette use alters gene expression in circulating neutrophils. Am J Respir Crit Care Med. 2018;197. Available: https://www.atsjournals.org/doi/pdf/10.1164/ajrccm-conference.2018.197.1_MeetingAbstracts.A3563	No	Not meeting inclusion criteria	Title and abstract screening
1032	Bojko M., Clothier J.S., Starnes V.A., Baker C.J. Surgical Resection of a Symptomatic Ascending Aortic Mural Thrombus. Ann Thorac Surg. 2022;114: e279–e282. doi:10.1016/j.athoracsur.2021.12.012	No	Not meeting inclusion criteria	Title and abstract screening
1033	Bold K, O'Malley S, Krishnan-Sarin S, Morean M. E-cigarette Use Patterns, Flavors, and Device Characteristics Associated With Quitting Smoking Among a U.S. sample of Adults Using E-cigarettes in a Smoking Cessation Attempt. Nicotine Tob Res. 2023;25: 954–961. doi:10.1093/ntr/ntac276	No	Not meeting inclusion criteria	Title and abstract screening
1034	Bold K.W., Kong G., Morean M., Gueorguieva R., Camenga D.R., Simon P., et al. Trends in various e-cigarette devices used by high school adolescents from 2017-2019. Drug Alcohol Depend. 2021;219: 108497. doi:10.1016/j.drugalcdep.2020.108497	No	Not meeting inclusion criteria	Title and abstract screening
1035	Bold KW, Buta E, Simon P, Kong G, Morean M, Camenga D, et al. Using latent class analysis to examine susceptibility to various tobacco products among adolescents. Nicotine Tob Res. 2020;22: 2059–2065. doi:10.1093/ntr/ntz216https://dx.doi.org/10.1093/ntr/ntz216	No	Not meeting inclusion criteria	Title and abstract screening
1036	Bold KW, Buta E, Simon P, Gueorguieva R, Jackson A, Suttiratana SC, et al. Examining the potential role of e-cigarettes to reduce health disparities associated with menthol cigarette use: Characterizing e-cigarette use, flavors, and reasons for use among US adults smoking menthol cigarettes. Drug Alcohol Depend. 2022;236: 109475. doi:10.1016/j.drugalcdep.2022.109475	No	Not meeting inclusion criteria	Title and abstract screening
1037	Bold KW, Sussman S, O'Malley SS, Grana R, Foulds J, Fishbein H, et al. Measuring E-cigarette dependence: Initial guidance. Addict Behav. 2018;79: 213–218. doi:10.1016/j.addbeh.2017.11.015	No	Not meeting inclusion criteria	Title and abstract screening
1038	Bolivar H.A., Elliott R.J., Middleton W., Yoon J.H., Okoli C.T.C., Haliwa I., et al. Social Smoking Environment and Associations with Cardiac Rehabilitation Attendance. J Cardiopulm Rehabil Prev. 2021;41: 46–51. doi:10.1097/HCR.0000000000000518	No	Not meeting inclusion criteria	Title and abstract screening
1039	Bonevski B, Manning V, Wynne O, Gartner C, Borland R, Baker AL, et al. QuitNic: A Pilot Randomized Controlled Trial Comparing Nicotine Vaping Products With Nicotine Replacement Therapy for Smoking Cessation Following Residential Detoxification. Nicotine Tob Res. 2021;23: 462–470. doi:10.1093/ntr/ntaa143	No	Not meeting inclusion criteria	Title and abstract screening
1040	Bonilla A, Blair AJ, Alamro SM, Ward RA, Feldman MB, Dutko RA, et al. Recurrent spontaneous pneumothoraces and vaping in an 18-year-old man: a case report and review of the literature. J Med Case Rep. 2019;13: 283. doi:10.1186/s13256-019-2215-4	No	Not meeting inclusion criteria	Title and abstract screening
1041	Bonnier A, Saha S, Shkolnik B, Saha BK. A comparative analysis of acute eosinophilic pneumonia associated with smoking and vaping. Am J Med Sci. 2023;365: 315–317. doi:10.1016/j.amjms.2022.10.003	No	Not meeting inclusion criteria	Title and abstract screening
1042	Booker R. Palliative Radiation Therapy: The Role of Radiation Therapy in Palliative and End-of-Life Care. Clin J Oncol Nurs. 2022;26: 628–635. doi:10.1188/22.CJON.628-635	No	Not meeting inclusion criteria	Title and abstract screening
1043	Boozary LK, Frank-Pearce SG, Alexander AC, Waring JJC, Ehlke SJ, Businelle MS, et al. "Correlates of e-cigarette use among adults initiating smoking cessation treatment": Corrigendum. Drug Alcohol Depend. 2021;228. doi:10.1016/j.drugalcdep.2021.109035https://dx.doi.org/10.1016/j.drugalcdep.2021.109035	No	Not meeting inclusion criteria	Title and abstract screening
1044	Boozary LK, Frank-Pearce SG, Alexander AC, Waring JJC, Ehlke SJ, Businelle MS, et al. Correlates of e-cigarette use among adults initiating smoking cessation treatment. Drug Alcohol Depend. 2021;224: 108724. doi:10.1016/j.drugalcdep.2021.108724	No	Not meeting inclusion criteria	Title and abstract screening
1045	Borchardt B, Kastaun S, Pashutina Y, Viechtbauer W, Kotz D. Motivation to stop smoking in the German population between 2016 - 2021 and associated factors: results from a repeated cross-sectional representative population survey (German Study on Tobacco Use, DEBRA study). BMJ Open. 2023;13: e068198. doi:10.1136/bmjopen-2022-068198	No	Not meeting inclusion criteria	Title and abstract screening
1046	Borchert D.H., Kelm H., Morean M., Tannapfel A. Reporting of pneumothorax in association with vaping devices and electronic cigarettes. BMJ Case Rep. 2021;14: e247844. doi:10.1136/bcr-2021-247844	No	Not meeting inclusion criteria	Title and abstract screening
1047	Borger TN, Puleo GE, Rivera Rivera JN, Montgomery D, Bowling WR, Burris JL. A descriptive study of cervical cancer survivors' persistent smoking behavior and perceived barriers to quitting. Psychol Addict Behav. 2022;36: 109–116. doi:10.1037/adb0000692	No	Not meeting inclusion criteria	Title and abstract screening
1048	Borkar NA, Roos B, Prakash YS, Sathish V, Pabelick CM. Nicotinic α7 acetylcholine receptor (α7nAChR) in human airway smooth muscle. Arch Biochem Biophys. 2021;706: 108897. doi:10.1016/j.abb.2021.108897	No	Not meeting inclusion criteria	Title and abstract screening
1049	Borkar NA, Thompson MA, Bartman CM, Sathish V, Prakash YS, Pabelick CM. Nicotine affects mitochondrial structure and function in human airway smooth muscle cells. Am J Physiol Lung Cell Mol Physiol. 2023;325: L803–L818. doi:10.1152/ajplung.00158.2023	No	Not meeting inclusion criteria	Title and abstract screening
1050	Borkar NA, Thompson MA, Bartman CM, Khalfaoui L, Sine SM, Sathish V, et al. Nicotinic Receptors in Airway Disease. Am J Physiol Lung Cell Mol Physiol. 2023. doi:10.1152/ajplung.00268.2023	No	Not meeting inclusion criteria	Title and abstract screening
1051	Borkar NA, Roos B, Prakash YS, Sathish V, Pabelick CM. Nicotinic alpha7 acetylcholine receptor (alpha7nAChR) in human airway smooth muscle. Arch Biochem Biophys. 2021;706: 108897. doi:10.1016/j.abb.2021.108897	No	Not meeting inclusion criteria	Title and abstract screening
1052	Borland R, Le Grande M, Heckman BW, Fong GT, Bickel WK, Stein JS, et al. The Predictive Utility of Valuing the Future for Smoking Cessation: Findings from the ITC 4 Country Surveys. Int J Env Res Public Health. 2022;19. doi:10.3390/ijerph19020631	No	Not meeting inclusion criteria	Title and abstract screening
1053	Borland R, Murray K, Gravely S, Fong GT, Thompson ME, McNeill A, et al. A new classification system for describing concurrent use of nicotine vaping products alongside cigarettes (so-called 'dual use'): findings from the ITC-4 Country Smoking and Vaping wave 1 Survey. Addiction. 2019;114 Suppl 1: 24–34. doi:10.1111/add.14570	No	Not meeting inclusion criteria	Title and abstract screening
1054	Borrelli B, O'Connor GT. E-Cigarettes to Assist with Smoking Cessation. N Engl J Med. 2019;380: 678–679. doi:10.1056/NEJMe1816406	No	Not meeting inclusion criteria	Title and abstract screening

1055	Bos PMJ, Soeteman-Hernandez LG, Talhout R. Risk assessment of components in tobacco smoke and e-cigarette aerosols: a pragmatic choice of dose metrics. Inhal Toxicol. 2021;33: 81–95. doi:10.1080/08958378.2021.1909678	No	Not meeting inclusion criteria	Title and abstract screening
1056	Bosch de Basea M, Belachew AB, Jankowski M, Meteran H, Dumas O. ERS International Congress 2021: highlights from the Epidemiology and Environment Assembly. ERJ Open Res. 2022;8. doi:10.1183/23120541.00697-2021	No	Not meeting inclusion criteria	Title and abstract screening
1057	BOSHARA P., DALAL B.D. MORE AIR, AIR: A CASE OF SPONTANEOUS PNEUMOMEDIASTINUM ASSOCIATED WITH CROHN'S ILEOCOLITIS. Chest. 2023;164: A3456. doi:10.1016/j.chest.2023.07.2247	No	Not meeting inclusion criteria	Title and abstract screening
1058	BOSHARA P., HANONA P.A.U.L., POUDEL S., JAIYESIMI I., EZEKWUDO D., ALLEN T., et al. AN AGGRESSIVE CASE OF THORACIC UNDIFFERENTIATED SMARCA4-DEFICIENT TUMOR WITH EXTENSIVE PLEURAL INVOLVEMENT. Chest. 2023;164: A4286–A4287. doi:10.1016/j.chest.2023.07.2789	No	Not meeting inclusion criteria	Title and abstract screening
1059	Bostic C., Huber L., Romeo-Stuppy K. A Holistic Approach to Human Rights and the Tobacco Epidemic. Nicotine Tob Res. 2020;22: 1054–1055. doi:10.1093/ntr/ntz104	No	Not meeting inclusion criteria	Title and abstract screening
1060	Boucher L.W., Loh G.A. A CASE of DABBING-INDUCED LIPOID PNEUMONIA. J Investig Med. 2021;70: 180. doi:10.1136/jim-2022-WRMC.133	No	Not meeting inclusion criteria	Title and abstract screening
1061	Boulin M., Cransac-Miet A., Maynadie M., Volot F., Creuzot-Garcher C., Eicher J.-C., et al. COVID-19 Lockdown in Patients with Chronic Diseases: A Cross-Sectional Study. Int J Environ Res Public Health. 2022;19: 3957. doi:10.3390/ijerph19073957	No	Not meeting inclusion criteria	Title and abstract screening
1062	Bourdon J.L., Hancock L.C. Using electronic audience response technology to track e-cigarette habits among college freshmen. Addict Behav. 2019;95: 24–27. doi:10.1016/j.addbeh.2019.02.019	No	Not meeting inclusion criteria	Title and abstract screening
1063	Bovero A, Cotardo F, Lops C, Botto R, Geminiani GC. Is there a relationship between end-of-life cancer patients' dignity-related distress and caregivers' distress? An exploratory study. Palliat Support Care. 2023;21: 578–584. doi:10.1017/S1478951522000840	No	Not meeting inclusion criteria	Title and abstract screening
1064	Bovero A, Digiovanni Y, Botto R, Leombruni P. End-of-life cancer patients' total pain: the necessity to supplement pharmacology with psycho-socio-spiritual treatments. Pain Manag. 2022;12: 895–906. doi:10.2217/pmt-2022-0015	No	Not meeting inclusion criteria	Title and abstract screening
1065	Bovero A, Opezzo M, Botto R, Gottardo F, Torta R. Hope in end-of-life cancer patients: A cross-sectional analysis. Palliat Support Care. 2021;19: 563–569. doi:10.1017/S1478951520001388	No	Not meeting inclusion criteria	Title and abstract screening
1066	Bovero A, Opezzo M, Tesio V. Relationship between demoralization and quality of life in end-of-life cancer patients. Psychooncology. 2023;32: 429–437. doi:10.1002/pon.6095	No	Not meeting inclusion criteria	Title and abstract screening
1067	Bovero A, Vitiello LP, Botto R, Gottardo F, Cito A, Geminiani GC. Demoralization in End-of-Life Cancer Patients' Family Caregivers: A Cross-Sectional Study. Am J Hosp Palliat Med. 2022;39: 332–339. doi:10.1177/10499091211023482	No	Not meeting inclusion criteria	Title and abstract screening
1068	Bowker K, Lewis S, Ussher M, Naughton F, Phillips L, Coleman T, et al. Smoking and vaping patterns during pregnancy and the postpartum: A longitudinal UK cohort survey. Addict Behav. 2021;123: 107050. doi:10.1016/j.addbeh.2021.107050	No	Not meeting inclusion criteria	Title and abstract screening
1069	Bowker K, Orton S, Cooper S, Naughton F, Whitemore R, Lewis S, et al. Views on and experiences of electronic cigarettes: a qualitative study of women who are pregnant or have recently given birth. BMC Pregnancy Childbirth. 2018;18: 233. doi:10.1186/s12884-018-1856-4	No	Not meeting inclusion criteria	Title and abstract screening
1070	Bowker K, Ussher M, Cooper S, Orton S, Coleman T, Campbell KA. Addressing and Overcoming Barriers to E-Cigarette Use for Smoking Cessation in Pregnancy: A Qualitative Study. Int J Env Res Public Health. 2020;17. doi:10.3390/ijerph17134823	No	Not meeting inclusion criteria	Title and abstract screening
1071	Bowling GC, Dimitrakoff JD. PSMA PET in Prostate Cancer—A Biomarker or a Surrogate End Point? JAMA Oncol. 2022;8: 1–1. doi:10.1001/jamaoncol.2021.7991	No	Not meeting inclusion criteria	Title and abstract screening
1072	Boyce-Fappiano D, Liao K, Miller C, Peterson SK, Elting LS, Guadagnolo BA. Greater preferences for death in hospital and mechanical ventilation at the end of life among non-whites recently diagnosed with cancer. Support Care Cancer. 2021;29: 6555–6564. doi:10.1007/s00520-021-06226-5	No	Not meeting inclusion criteria	Title and abstract screening
1073	Boyce-Fappiano D, Liao K, Miller C, Peterson SK, Elting L, Guadagnolo BA. Preferences for More Aggressive End-of-life Pharmacologic Care Among Racial Minorities in a Large Population-Based Cohort of Cancer Patients. J Pain Symptom Manage. 2021;62: 482–491. doi:10.1016/j.jpainsymman.2021.02.001	No	Not meeting inclusion criteria	Title and abstract screening
1074	Boykan R., Walley S. Asthma to EVALI: Tobacco Use Is a Pediatric Problem. Hosp Pediatr. 2021;11: 106–108. doi:10.1542/hpeds.2020-003715	No	Not meeting inclusion criteria	Title and abstract screening
1075	Boyle RG, Stanton CA, Sharma E, Tang Z. Examining quit attempts and successful quitting after recent cigarette tax increases. Prev Med. 2019;118: 226–231. doi:10.1016/j.ypmed.2018.11.008	No	Not meeting inclusion criteria	Title and abstract screening
1076	Boynton MH, Sanzo N, Brothers W, Kresovich A, Sutfin EL, Sheeran P, et al. Perceived effectiveness of objective elements of vaping prevention messages among adolescents. Tob Control. 2022. doi:10.1136/tobaccocontrol-2021-057151	No	Not meeting inclusion criteria	Title and abstract screening
1077	Boynton MH, Sanzo N, Brothers W, Kresovich A, Sutfin EL, Sheeran P, et al. Perceived effectiveness of objective elements of vaping prevention messages among adolescents. Tob Control. 2023;32: e228–e235. doi:10.1136/tobaccocontrol-2021-057151	No	Not meeting inclusion criteria	Title and abstract screening
1078	Braciszewski JM, Vose-O'Neal A, Gamarel KE, Colby SM. Combustible Cigarette Smoking and Alternative Tobacco Use in a Sample of Youth Transitioning from Foster Care. Child Youth Serv Rev. 2019;96: 231–236. doi:10.1016/j.childyouth.2018.11.054	No	Not meeting inclusion criteria	Title and abstract screening
1079	Bracken-Clarke D, Kapoor D, Baird AM, Buchanan PJ, Gately K, Cuffe S, et al. Vaping and lung cancer - A review of current data and recommendations. Lung Cancer Amst Neth. 2021;153: 11–20. doi:10.1016/j.lungcan.2020.12.030	No	Not meeting inclusion criteria	Title and abstract screening
1080	Braillon A. Association Between Cigarette Smoking and COVID-19 Outcomes. JAMA Intern Med. 2021;181: 1137–1138. doi:10.1001/jamainternmed.2021.1920	No	Not meeting inclusion criteria	Title and abstract screening
1081	Braillon A. Tobacco control without a maximum nicotine level in products is a smokescreen. BMJ. 2022;378: o1942. doi:10.1136/bmj.o1942	No	Not meeting inclusion criteria	Title and abstract screening
1082	Braillon A, Lang AE. The International Agency for Research on Cancer and e-cigarette carcinogenicity: time for an evaluation. Eur J Epidemiol. 2023;38: 391. doi:10.1007/s10654-023-00993-7	No	Not meeting inclusion criteria	Title and abstract screening
1083	Bramati PS, Azhar A, Khan R, Tovbin M, Cooper A, Pangemanan I, et al. High Flow Nasal Cannula in Patients With Cancer at the End of Life. J Pain Symptom Manage. 2023;65: e369–e373. doi:10.1016/j.jpainsymman.2022.12.141	No	Not meeting inclusion criteria	Title and abstract screening

1084	Branstetter SA, Nye RT, Muscat JE. Time to first cigarette of the day and 4-(methylnitrosamino)-1-(3-pyridyl)-1-butanol (NNAL) in adult regular and non-daily smokers: (NHANES) 2007-10. Regul Toxicol Pharmacol. 2019;108: 104454. doi:10.1016/j.yrtph.2019.104454	No	Not meeting inclusion criteria	Title and abstract screening
1085	Brasky TM, Hinton A, Doogan NJ, Cooper SE, Nagaraja HN, Xi W, et al. Characteristics of the Tobacco User Adult Cohort in Urban and Rural Ohio. Tob Regul Sci. 2018;4: 614–630. doi:10.18001/TRS.4.1.8	No	Not meeting inclusion criteria	Title and abstract screening
1086	Brath H, Kaser S, Tatschl C, Fischer-See S, Fasching P. [Smoking, heated tobacco products, alcohol and diabetes mellitus (update 2023)]. Wien Klin Wochenschr. 2023;135: 84–90. doi:10.1007/s00508-023-02161-z	No	Not meeting inclusion criteria	Title and abstract screening
1087	Braun IM, Abrams DI, Blansky SE, Pergam SA. Cannabis and the Cancer Patient. J Natl Cancer Inst Monogr. 2021;2021: 68–77. doi:10.1093/jncimonographs/lgab012	No	Not meeting inclusion criteria	Title and abstract screening
1088	Braverman MT, Geldhof GJ, Hoogesteger LA, Johnson JA. Predicting students' noncompliance with a smoke-free university campus policy. Prev Med. 2018;114: 209–216. doi:10.1016/j.ypmed.2018.07.002	No	Not meeting inclusion criteria	Title and abstract screening
1089	Bravo-Gutierrez OA, Falfan-Valencia R, Ramirez-Venegas A, Sansores RH, Ponciano-Rodriguez G, Perez-Rubio G. Lung Damage Caused by Heated Tobacco Products and Electronic Nicotine Delivery Systems: A Systematic Review. Int J Environ Res Public Health. 2021;18. doi:10.3390/ijerph18084079	No	Not meeting inclusion criteria	Title and abstract screening
1090	Braymiller J. Epidemiology of nicotine use among adolescents and young adults in the United States: Findings from the PATH study. Diss Abstr Int Sect B Sci Eng. 2021;82: No-Specified.	No	Not meeting inclusion criteria	Title and abstract screening
1091	Braymiller JL, Riehm KE, Meier M, Krueger EA, Unger JB, Barrington-Trimis JL, et al. Associations of alternative cannabis product use and poly-use with subsequent illicit drug use initiation during adolescence. Psychopharmacol Berl. 2023. doi:10.1007/s00213-023-06330-w	No	Not meeting inclusion criteria	Title and abstract screening
1092	Breland A, Maloney SF, Soule EK, Ramôa C, Barnes A, Lipato T, et al. Abuse liability of electronic cigarettes in men who are experienced electronic cigarette users. Exp Clin Psychopharmacol. 2020;28: 235–244. doi:10.1037/pha0000305	No	Not meeting inclusion criteria	Title and abstract screening
1093	Breland A, Soule E, Lopez A, Ramôa C, El-Hellani A, Eissenberg T. Electronic cigarettes: what are they and what do they do? Ann N Acad Sci. 2017;1394: 5–30. doi:10.1111/nyas.12977	No	Not meeting inclusion criteria	Title and abstract screening
1094	Brennan-Tovey K, Aquino MRJ, Flanagan S, Kaner E, Wearn A, Bigirumurame T, et al. Implementation of the NHS-funded tobacco dependence services in England: a qualitative study to understand the contexts of implementation. Lancet. 2022;400 Suppl 1: S24. doi:10.1016/S0140-6736(22)02234-6	No	Not meeting inclusion criteria	Title and abstract screening
1095	Brewer NT, Jeong M, Hall MG, Baig SA, Mendel JR, Lazard AJ, et al. Impact of e-cigarette health warnings on motivation to vape and smoke. Tob Control. 2019. doi:10.1136/tobaccocontrol-2018-054878	No	Not meeting inclusion criteria	Title and abstract screening
1096	Bricker J.B., Watson N.L., Mull K.E., Sullivan B.M., Heffner J.L. Efficacy of Smartphone Applications for Smoking Cessation: A Randomized Clinical Trial. JAMA Intern Med. 2020;180: 1472–1480. doi:10.1001/jamainternmed.2020.4055	No	Not meeting inclusion criteria	Title and abstract screening
1097	Bricker J, Miao Z, Mull K, Santiago-Torres M, Vock DM. Can a Single Variable Predict Early Dropout From Digital Health Interventions? Comparison of Predictive Models From Two Large Randomized Trials. J Med Internet Res. 2023;25: e43629. doi:10.2196/43629	No	Not meeting inclusion criteria	Title and abstract screening
1098	Brimkulov N, Vinnikov D, Dzhilkiadarova Z, Aralbaeva A. Tobacco use among Kyrgyzstan medical students: an 11-year follow-up cross-sectional study. BMC Public Health. 2017;17: 625. doi:10.1186/s12889-017-4547-6	No	Not meeting inclusion criteria	Title and abstract screening
1099	Bringgold W., Berg K., Smith S., Piper M., Jorenby D. Does dual use of electronic cigarettes and conventional cigarettes confer a pulmonary health advantage? a baseline and 12 month prospective cohort analysis. Am J Respir Crit Care Med. 2019;199. Available: https://www.atsjournals.org/doi/abs/10.1164/ajrccm-conference.2019.199.1_MeetingAbstracts.A4178	No	Not meeting inclusion criteria	Title and abstract screening
1100	Bringgold W., Berg K.M., Piper M.E., Jorenby D.E. Does dual use of electronic cigarettes and conventional cigarettes confer a pulmonary health advantage? a baseline cohortanalysis. J Gen Intern Med. 2018;33: 168–169.	No	Not meeting inclusion criteria	Title and abstract screening
1101	Brinson D, Ward C, Ford C, Begg A. Smokefree and vapefree streets: high levels of support from tourists, residents and businesses, implications for tourist-destination communities in New Zealand. N Z Med J. 2022;135: 73–84.	No	Not meeting inclusion criteria	Title and abstract screening
1102	Britton J. Denicotinised cigarettes. The Lancet. 2018;392: 104–105. doi:10.1016/S0140-6736%2818%2931358-8	No	Not meeting inclusion criteria	Title and abstract screening
1103	Brockenberry L. Racial differences in tobacco use and risk factors among young adults: Roles of expectancies and emotion regulation. Diss Abstr Int Sect B Sci Eng. 2022;83: No-Specified.	No	Not meeting inclusion criteria	Title and abstract screening
1104	Brooks JM, Mermelstein RJ. Negative Affect and Cigarette Cessation in Dual Users of Cigarettes and Electronic Nicotine Delivery Systems. Subst Use Misuse. 2022;57: 1294–1302. doi:10.1080/10826084.2022.2079135	No	Not meeting inclusion criteria	Title and abstract screening
1105	Brose LS, Brown J, Robson D, McNeill A. Mental health, smoking, harm reduction and quit attempts - a population survey in England. BMC Public Health. 2020;20: 1237. doi:10.1186/s12889-020-09308-x	No	Not meeting inclusion criteria	Title and abstract screening
1106	Brose LS, McDermott MS, McNeill A. Heated Tobacco Products and Nicotine Pouches: A Survey of People with Experience of Smoking and/or Vaping in the UK. Int J Env Res Public Health. 2021;18. doi:10.3390/ijerph18168852	No	Not meeting inclusion criteria	Title and abstract screening
1107	Brothers TD, Leaman M, Bonn M, Lewer D, Atkinson J, Fraser J, et al. Evaluation of an emergency safe supply drugs and managed alcohol program in COVID-19 isolation hotel shelters for people experiencing homelessness. Drug Alcohol Depend. 2022;235: 109440. doi:10.1016/j.drugalcdep.2022.109440	No	Not meeting inclusion criteria	Title and abstract screening
1108	Brouwer AF, Jeon J, Jimenez-Mendoza E, Land SR, Holford TR, Friedman AS, et al. Changing patterns of cigarette and ENDS transitions in the USA: a multistate transition analysis of adults in the PATH Study in 2017-2019 vs 2019-2021. medRxiv. 2023. doi:10.1101/2023.10.20.23297320	No	Not meeting inclusion criteria	Title and abstract screening
1109	Brouwer AF, Jeon J, Jimenez-Mendoza E, Land SR, Holford TR, Friedman AS, et al. Changing patterns of cigarette and ENDS transitions in the USA: a multistate transition analysis of youth and adults in the PATH Study in 2015-2017 vs 2017-2019. Tob Control. 2023. doi:10.1136/tc-2022-057905	No	Not meeting inclusion criteria	Title and abstract screening
1110	Brouwer AF, Levy DT, Jeon J, Jimenez-Mendoza E, Sanchez-Romero LM, Mistry R, et al. The Impact of Current Tobacco Product Use Definitions on Estimates of Transitions Between Cigarette and ENDS Use. Nicotine Tob Res. 2022;24: 1756–1762. doi:10.1093/ntr/ntac132	No	Not meeting inclusion criteria	Title and abstract screening
1111	Brown A, O'Donnell R, Eadie D, Ford A, Mitchell D, Hackett A, et al. E-cigarette Use in Prisons With Recently Established Smokefree Policies: A Qualitative Interview Study With People in Custody in Scotland. Nicotine Tob Res. 2021;23: 939–946. doi:10.1093/ntr/ntaa271	No	Not meeting inclusion criteria	Title and abstract screening
1112	Brown A, O'Donnell R, Eadie D, Purves R, Sweeting H, Ford A, et al. Initial Views and Experiences of Vaping in Prisons: A Qualitative Study With People in Custody Preparing for the Imminent Implementation of Scotland's Prison Smokefree Policy. Nicotine Tob Res. 2021;23: 543–549. doi:10.1093/ntr/ntaa088	No	Not meeting inclusion criteria	Title and abstract screening

1113	Brown HA, Roberts RD, Chen TA, Businelle MS, Obasi EM, Kendzor DE, et al. Perceived Disease Risk of Smoking, Barriers to Quitting, and Cessation Intervention Preferences by Sex Amongst Homeless Adult Concurrent Tobacco Product Users and Conventional Cigarette-Only Users. Int J Env Res Public Health. 2022;19. doi:10.3390/ijerph19063629	No	Not meeting inclusion criteria	Title and abstract screening
1114	Brown J. A gateway to more productive research on e-cigarettes? Commentary on a comprehensive framework for evaluating public health impact. Addiction. 2017;112: 21–22. doi:10.1111/add.13449	No	Not meeting inclusion criteria	Title and abstract screening
1115	Brown J, Shahab L, West R. Does the offer of e-cigarettes benefit smoking cessation among unselected smokers? Addiction. 2019;114: 186–187. doi:10.1111/add.14415	No	Not meeting inclusion criteria	Title and abstract screening
1116	Brown P.E., Izawa Y., Balakrishnan K., Fu S.H., Chakma J., Menon G., et al. Mortality Associated with Ambient PM2:5 Exposure in India: Results from the Million Death Study. Environ Health Perspect. 2022;130: 097004–14. doi:10.1289/EHP9538	No	Not meeting inclusion criteria	Title and abstract screening
1117	Brown R.A., Hecht J., Bloom E.L., Minami H., Kahler C.W., Abrantes A.M., et al. Development and preliminary pilot evaluation of a brief tablet computer intervention to motivate tobacco quitline use among smokers in substance use treatment. Am J Addict. 2017;26: 587–594. doi:10.1111/ajad.12559	No	Not meeting inclusion criteria	Title and abstract screening
1118	Brown S., Nwokoro C., Bush A., Lenney W., Vestbo J., Pao C., et al. Another public health catastrophe. The Lancet. 2021;398: 2243. doi:10.1016/S0140-6736%2821%2902730-6	No	Not meeting inclusion criteria	Title and abstract screening
1119	Brown TS, Grewal U, Thotamgari SR, Ananthaneni AK, Burnett C, Vutukuri S, et al. End of Life Care in Hospitalized Patients With Lung Cancer. Int J Radiat Oncol Biol Phys. 2022;112: e18–e18. doi:10.1016/j.ijrobp.2021.10.195	No	Not meeting inclusion criteria	Title and abstract screening
1120	Brown T, Grewal US, Ananthaneni AK, Thotamgari SR, Beedupalli K. Letter to the Editor: Poor Access to Ambulatory Palliative Care and Needless Aggressive Care toward the End of Life among Patients with Lung Cancer. J Palliat Med. 2022;25: 698–699. doi:10.1089/jpm.2022.0020	No	Not meeting inclusion criteria	Title and abstract screening
1121	Bruijnzeel A.W. Shifting Frontiers in Basic Research on Nicotine and Tobacco Products. Nicotine Tob Res. 2020;22: 145–146. doi:10.1093/ntr/ntz190	No	Not meeting inclusion criteria	Title and abstract screening
1122	Bruijnzeel A.W. The Unhealthy Association Between Smoking, Vaping, and Other Drug Use. Nicotine Tob Res. 2022;24: 1139–1140. doi:10.1093/ntr/ntac130	No	Not meeting inclusion criteria	Title and abstract screening
1123	Brunet J, Price J, Srikanthan A, Gillison F, Standage M, Taljaard M, et al. The physicAl aCtivity Counselling for young adult cancEr SurvivorS (ACCESS) trial: a protocol for a parallel, two-arm pilot randomized controlled trial. 2022;17: e0273045. doi:10.1371/journal.pone.0273045	No	Not meeting inclusion criteria	Title and abstract screening
1124	Bruno S, Bazzani A, Marantonio S, Cruz-Sanabria F, Benedetti D, Frumento P, et al. Poor sleep quality and unhealthy lifestyle during the lockdown: an Italian study. Sleep Med. 2022;90: 53–64. doi:10.1016/j.sleep.2022.01.002	No	Not meeting inclusion criteria	Title and abstract screening
1125	Bteddini DS, LeLaurin JH, Chi X, Hall JM, Theis RP, Gurka MJ, et al. Mixed methods evaluation of vaping and tobacco product use prevention interventions among youth in the Florida 4-H program. Addict Behav. 2023;141: 107637. doi:10.1016/j.addbeh.2023.107637	No	Not meeting inclusion criteria	Title and abstract screening
1126	Buchanan T, Lindorff K, Carson-Chahhoud K. E-cigarette regulation: Is it time for a new Framework Convention on Tobacco Control? Respirology. 2023;28: 220–222. doi:10.1111/resp.14466	No	Not meeting inclusion criteria	Title and abstract screening
1127	Buchanan T, White SL, Marshall H, Carson-Chahhoud KV, Magee CA, Kelly PJ. Authors' response to "Vaping nicotine should be part of Australia's tobacco control policy". Aust N Z J Public Health. 2022. doi:10.1111/1753-6405.13248	No	Not meeting inclusion criteria	Title and abstract screening
1128	Bucharskaya A.B., Yanina I.Y., Atsigeida S.V., Genin V.D., Lazareva E.N., Navolokin N.A., et al. Optical clearing and testing of lung tissue using inhalation aerosols: prospects for monitoring the action of viral infections. Biophys Rev. 2022;14: 1005–1022. doi:10.1007/s12551-022-00991-1	No	Not meeting inclusion criteria	Title and abstract screening
1129	Buchting FO, Emory KT, Scout, Kim Y, Fagan P, Vera LE, et al. Transgender Use of Cigarettes, Cigars, and E-Cigarettes in a National Study. Am J Prev Med. 2017;53: e1–e7. doi:10.1016/j.amepre.2016.11.022	No	Not meeting inclusion criteria	Title and abstract screening
1130	Buckell J, Hensher DA, Hess S. Kicking the habit is hard: A hybrid choice model investigation into the role of addiction in smoking behavior. Health Econ. 2021;30: 3–19. doi:10.1002/hec.4173	No	Not meeting inclusion criteria	Title and abstract screening
1131	Buckner JD, Morris PE, Zvolensky MJ. Cannabis use and electronic cigarette use: The role of dual use on use frequency and related problems. Psychiatry Res. 2021;304: 114126. doi:10.1016/j.psychres.2021.114126	No	Not meeting inclusion criteria	Title and abstract screening
1132	Buckner JD, Shepherd J, Stoneking FR, Zvolensky MJ. Dual Electronic and Combustible Cigarette Use: Understanding the Relation of Cannabis Use with E-Cigarette Outcomes. Subst Use Misuse. 2023; 1–7. doi:10.1080/10826084.2023.2275570	No	Not meeting inclusion criteria	Title and abstract screening
1133	Budenz A, Klein A, Prutzman Y. The Relationship Between Trauma Exposure and Adult Tobacco Use: Analysis of the National Epidemiologic Survey on Alcohol and Related Conditions (III). Nicotine Tob Res. 2021;23: 1716–1726. doi:10.1093/ntr/ntab057	No	Not meeting inclusion criteria	Title and abstract screening
1134	Bugge C, Saether EM, Kristiansen IS. Men receive more end-of-life cancer hospital treatment than women: fact or fiction? Acta Oncol. 2021;60: 984–991. doi:10.1080/0284186X.2021.1917000	No	Not meeting inclusion criteria	Title and abstract screening
1135	Bukke VN, Archana M, Villani R, Serviddio G, Cassano T. Pharmacological and Toxicological Effects of Phytocannabinoids and Recreational Synthetic Cannabinoids: Increasing Risk of Public Health. Pharm Basel Switz. 2021;14. doi:10.3390/ph14100965	No	Not meeting inclusion criteria	Title and abstract screening
1136	Bulat E, Komlan AG, Bonaparte C, White R, Jotwani R. Perioperative considerations of patient E-cigarette use for the anesthesiologist. J Clin Anesth. 2022;78: 110619. doi:10.1016/j.jclinane.2021.110619	No	Not meeting inclusion criteria	Title and abstract screening
1137	Bullen C, Verbiest M, Galea-Singer S, Kurdziel T, Laking G, Newcombe D, et al. The effectiveness and safety of combining varenicline with nicotine e-cigarettes for smoking cessation in people with mental illnesses and addictions: study protocol for a randomised-controlled trial. BMC Public Health. 2018;18: 596. doi:10.1186/s12889-018-5351-7	No	Not meeting inclusion criteria	Title and abstract screening
1138	Bullock G.S., Nicholson K.F., Waterman B.R., Niesen E., Salamh P., Thigpen C.A., et al. Health Conditions, Substance Use, Physical Activity, and Quality of Life in Current and Former Baseball Players. Orthop J Sports Med. 2021;9. doi:10.1177/23259671211056645	No	Not meeting inclusion criteria	Title and abstract screening
1139	Buma S, van Klinken M, van der Noort V. A Targeted Discharge Pathway to Reduce Hospital Readmission and Dying in Hospital in Cancer Patients at the End of Life. Semin Oncol Nurs. 2023;39: N.PAG-N.PAG. doi:10.1016/j.soncn.2023.151506	No	Not meeting inclusion criteria	Title and abstract screening
1140	Bunch D. RC Currents. AARC Newsroom. 2023; 11–11.	No	Not meeting inclusion criteria	Title and abstract screening
1141	Burhansstipanov L, Krebs LU, Petereit D, Dignan MB, Ahamed SI, Sargent M, et al. Reality Versus Grant Application Research "Plans". Health Promot Pr. 2018;19: 566–572. doi:10.1177/1524839917700892	No	Not meeting inclusion criteria	Title and abstract screening

1142	Burmeister M.A., Hawthorne A., Weldon A.J., White J. Adverse Neurologic Effects of Electronic Cigarette Use. US Pharm. 2022;47: HS-12.	No	Not meeting inclusion criteria	Title and abstract screening
1143	Burnley A, Bold KW, Kong G, Wu R, Krishnan-Sarin S. E-cigarette use perceptions that differentiate e-cigarette susceptibility and use among high school students. Am J Drug Alcohol Abuse. 2021;47: 238–246. doi:10.1080/00952990.2020.1826501	No	Not meeting inclusion criteria	Title and abstract screening
1144	Burstrom A., Bay A., Berghammer M. What adolescents with congenital heart disease want to know about their heart disease and health including sexual health and contraception. Cardiol Young. 2023;33: S278. doi:10.1017/S1047951123001099	No	Not meeting inclusion criteria	Title and abstract screening
1145	Burt B, Li J. The electronic cigarette epidemic in youth and young adults: A practical review. JAAPA. 2020;33: 17–23. doi:10.1097/01.JAA.0000654384.02068.99	No	Not meeting inclusion criteria	Title and abstract screening
1146	Bush A, Lintowska A, Mazur A, Hadjipanayis A, Grossman Z, Del Torso S, et al. E-Cigarettes as a Growing Threat for Children and Adolescents: Position Statement From the European Academy of Paediatrics. Front Pediatr. 2021;9: 698613. doi:10.3389/fped.2021.698613	No	Not meeting inclusion criteria	Title and abstract screening
1147	Bush A. Long-term consequences of childhood respiratory diseases. Pediatr Pulmonol. 2021;56: S12–S14. doi:10.1002/ppul.25497	No	Not meeting inclusion criteria	Title and abstract screening
1148	Bush A. Transition to Adult Care: What adults should know about Pediatric Respiratory Diseases. Pediatr Pulmonol. 2022;57: S22–S24. doi:10.1002/ppul.25960	No	Not meeting inclusion criteria	Title and abstract screening
1149	Bush A., Bhatt J.M., Carroll W., Child F., Connett G., Doull I., et al. A rational approach to e-cigarettes: Challenging ERS policy on tobacco harm reduction. Eur Respir J. 2020;55: e2000355. doi:10.1183/13993003.00355-2020	No	Not meeting inclusion criteria	Title and abstract screening
1150	Bush A, Ferkol T, Valiulis A, Mazur A, Chkhaidze I, Maglakelidze T, et al. Unfriendly Fire: How the Tobacco Industry is Destroying the Future of Our Children. Acta Medica Litu. 2021;28: 6–18. doi:10.15388/Amed.2020.28.1.6	No	Not meeting inclusion criteria	Title and abstract screening
1151	Butkus R., Rapp K., Cooney T.G., Engel L.S. Envisioning a Better U.S. Health Care System for All: Reducing Barriers to Care and Addressing Social Determinants of Health. Ann Intern Med. 2020;172: S50–S59. doi:10.7326/M19-2410	No	Not meeting inclusion criteria	Title and abstract screening
1152	Button E, Cardona M, Huntley K, Gavin NC, LeBlanc TW, Olsen A, et al. Clinicians' Understanding of Preferences and Values of People with Hematological Malignancies at the End of Life: Concurrent Surveys. J Palliat Med. 2022;25: 1386–1397. doi:10.1089/jpm.2021.0490	No	Not meeting inclusion criteria	Title and abstract screening
1153	Buu A, Cai Z, Li R, Wong SW, Lin HC, Su WC, et al. The association between short-term emotion dynamics and cigarette dependence: A comprehensive examination of dynamic measures. Drug Alcohol Depend. 2021;218: 108341. doi:10.1016/j.drugalcdep.2020.108341	No	Not meeting inclusion criteria	Title and abstract screening
1154	Byrne JM, Mesarwi P, Edmonds KP, Atayee RS. Dexmedetomidine Continuous Infusion for Refractory Cancer Pain at End of Life: A Case Report. J Pain Palliat Care Pharmacother. 2022;36: 200–206. doi:10.1080/15360288.2022.2102705	No	Not meeting inclusion criteria	Title and abstract screening
1155	Cabral P. E-cigarette use and intentions related to psychological distress among cigarette, e-cigarette, and cannabis vape users during the start of the COVID-19 pandemic. BMC Psychol. 2022;10: 201. doi:10.1186/s40359-022-00910-9	No	Not meeting inclusion criteria	Title and abstract screening
1156	Cabriales JA, Richards DK, Cooper TV. Heterogeneity of intermittent smokers in a Hispanic college student sample. Addict Behav. 2019;96: 94–99. doi:10.1016/j.addbeh.2019.04.028	No	Not meeting inclusion criteria	Title and abstract screening
1157	Cadham CJ, Liber AC, Sánchez-Romero LM, Issabakhsh M, Warner KE, Meza R, et al. The actual and anticipated effects of restrictions on flavoured electronic nicotine delivery systems: a scoping review. BMC Public Health. 2022;22: 2128. doi:10.1186/s12889-022-14440-x	No	Not meeting inclusion criteria	Title and abstract screening
1158	Cahn Z, Berg CJ. Commentary on Seyla et al. (2018): Advantages in the consideration of causal mechanisms for studies of gateway e-cigarette use. Addiction. 2018;113: 334–335. doi:10.1111/add.14079	No	Not meeting inclusion criteria	Title and abstract screening
1159	Cai ED, Swetter SM, Sarin KY. Association of multiple primary melanomas with malignancy risk: A population-based analysis of entries from the Surveillance, Epidemiology, and End Results program database during 1973-2014. J Am Acad Dermatol. 2023;88: e211–e219. doi:10.1016/j.jaad.2018.09.027	No	Not meeting inclusion criteria	Title and abstract screening
1160	Cai H., Garcia J.G.N., Wang C. More to Add to E-Cigarette Regulations: Unified Approaches. Chest. 2020;157: 771–773. doi:10.1016/j.chest.2019.11.024	No	Not meeting inclusion criteria	Title and abstract screening
1161	Calabro K, Khalil G, Chen M, Perry C, Prokhorov A. Pilot study to inform young adults about the risks of electronic cigarettes through text messaging. 2019;10. doi:10.1016/j.abrep.2019.100224	No	Not meeting inclusion criteria	Title and abstract screening
1162	Calello D.P., Aldy K., Jefri M., Nguyen T.-A.T., Krotulski A., Logan B., et al. Identification of a novel opioid, N-piperidinyl etonitazene (etonitazepipne), in patients with suspected opioid overdose. Clin Toxicol. 2022;60: 1067–1069. doi:10.1080/15563650.2022.2084406	No	Not meeting inclusion criteria	Title and abstract screening
1163	Calvache JA, Moreno S, Prue G, Reid J, Ahmedzai SH, Arango-Gutierrez A, et al. Knowledge of end-of-life wishes by physicians and family caregivers in cancer patients. BMC Palliat Care. 2021;20: 1–7. doi:10.1186/s12904-021-00823-1	No	Not meeting inclusion criteria	Title and abstract screening
1164	Camacho O, Hedge A, Lowe F, Newland N, Gale N, McEwan M, et al. Statistical analysis plan for "A randomised, controlled study to evaluate the effects of switching from cigarette smoking to using a tobacco heating product on health effect indicators in healthy subjects." 2020;17: 100535. doi:10.1016/j.conctc.2020.100535	No	Not meeting inclusion criteria	Title and abstract screening
1165	Camacho-Rivera M, Islam JY, Rodriguez DL, Vidot DC. Cannabis Use among Cancer Survivors amid the COVID-19 Pandemic: Results from the COVID-19 Cannabis Health Study. Cancers Basel. 2021;13. doi:10.3390/cancers13143495	No	Not meeting inclusion criteria	Title and abstract screening
1166	Cambron C. E-Cigarette Use Is Associated with Increased Psychological Distress among Youth: A Pooled Cross-Sectional Analysis of State-Level Data from 2019 and 2021. Int J Env Res Public Health. 2022;19. doi:10.3390/ijerph191811726	No	Not meeting inclusion criteria	Title and abstract screening
1167	Cameron A, Meng Yip H, Garg M. e-Cigarettes and Oral Cancer: what do we know so far?. Br J Oral Maxillofac Surg. 2023;61: 380–382. doi:10.1016/j.bjoms.2023.03.013	No	Not meeting inclusion criteria	Title and abstract screening
1168	Camoretti-Mercado B, Liao Q, Tian Z, Allen-Gipson D. Editorial: Cigarette Smoke, E-Cigarette/E-Vaping and COVID-19: Risks and Implications in This New Era. Front Physiol. 2021;12: 724910. doi:10.3389/fphys.2021.724910	No	Not meeting inclusion criteria	Title and abstract screening
1169	Campagna D, Caci G. Taking for Granted Conclusions from Studies that Cannot Prove Causality of Respiratory Symptoms and Vaping. Am J Respir Crit Care Med. 2022;206: 1567–1568. doi:10.1164/rccm.202205-0878LE	No	Not meeting inclusion criteria	Title and abstract screening
1170	Campbell B, Yip D, Le T, Gubner N, Guydish J. Relationship between Tobacco Use and Health-Related Quality of Life (HRQoL) among Clients in Substance Use Disorders Treatment. J Psychoact Drugs. 2019;51: 48–57. doi:10.1080/02791072.2018.1555651	No	Not meeting inclusion criteria	Title and abstract screening

1171	Campbell BK, Le T, Kapiteni K, Gubner NR, Guydish J. Correlates of lifetime blunt/spliff use among cigarette smokers in substance use disorders treatment. J Subst Abuse Treat. 2020;116: 108064. doi:10.1016/j.jsat.2020.108064	No	Not meeting inclusion criteria	Title and abstract screening
1172	Campos NS, Bluth T, Hemmes SNT, Librero J, Pozo N, Ferrando C, et al. Intraoperative positive end-expiratory pressure and postoperative pulmonary complications: a patient-level meta-analysis of three randomised clinical trials. BJA Br J Anaesth. 2022;128: 1040–1051. doi:10.1016/j.bja.2022.02.039	No	Not meeting inclusion criteria	Title and abstract screening
1173	Campos-Outcalt D. A review of the latest USPSTF recommendations. J Fam Pract. 2021;70: 189–204. doi:10.12788/jfp.0184	No	Not meeting inclusion criteria	Title and abstract screening
1174	Canchola A, Meletz R, Khandakar RA, Woods M, Lin YH. Temperature dependence of emission product distribution from vaping of vitamin E acetate. PLoS One. 2022;17: e0265365. doi:10.1371/journal.pone.0265365	No	Not meeting inclusion criteria	Title and abstract screening
1175	Cantini L, Mentrasti G, Russo GL, Signorelli D, Pasello G, Rijavec E, et al. Evaluation of COVID-19 impact on DELAYing diagnostic-therapeutic pathways of lung cancer patients in Italy (COVID-DELAY study): fewer cases and higher stages from a real-world scenario. ESMO Open. 2022;7: 100406. doi:10.1016/j.esmoop.2022.100406	No	Not meeting inclusion criteria	Title and abstract screening
1176	Cao D.C., Forrester M. Pediatric e-cigarette exposures treated at emergency departments. Clin Toxicol. 2022;60: 59–60. doi:10.1080/15563650.2022.2107776	No	Not meeting inclusion criteria	Title and abstract screening
1177	Cao X, Coyle JP, Xiong R, Wang Y, Heflich RH, Ren B, et al. Invited review: human air-liquid-interface organotypic airway tissue models derived from primary tracheobronchial epithelial cells-overview and perspectives. In Vitro Cell Dev Biol Anim. 2021;57: 104–132. doi:10.1007/s11626-020-00517-7	No	Not meeting inclusion criteria	Title and abstract screening
1178	Cao Y, Yi H, Zhou J, Cheng Y, Mao Y. Regulations on e-cigarettes: China is taking action. Pulmonology. 2023;29: 359–361. doi:10.1016/j.pulmoe.2023.02.007	No	Not meeting inclusion criteria	Title and abstract screening
1179	Capodanno D, Wijns W. Non-fatal MI as surrogate end point for all-cause or cardiovascular mortality. Nat Rev Cardiol. 2022;19: 149–150. doi:10.1038/s41569-021-00667-5	No	Not meeting inclusion criteria	Title and abstract screening
1180	Caponnetto P, Caruso M, Maglia M, Emma R, Saitta D, Busà B, et al. Non-inferiority trial comparing cigarette consumption, adoption rates, acceptability, tolerability, and tobacco harm reduction potential in smokers switching to Heated Tobacco Products or electronic cigarettes: Study protocol for a randomized controlled t. Contemp Clin Trials Commun. 2020;17: 100518. doi:10.1016/j.conctc.2020.100518	No	Not meeting inclusion criteria	Title and abstract screening
1181	Caponnetto P, Casu M, Crane D, Ross L, Quattropani MC, Polosa R. User evaluation and feasibility test of an app designed for smoking cessation in Italian people who smoke: preliminary findings from an uncontrolled pre-test post-test open study. BMC Psychol. 2023;11: 387. doi:10.1186/s40359-023-01430-w	No	Not meeting inclusion criteria	Title and abstract screening
1182	Caponnetto P, DiPiazza J, Cappello GC, Demma S, Maglia M, Polosa R. Multimodal Smoking Cessation in a Real-Life Setting: Combining Motivational Interviewing With Official Therapy and Reduced Risk Products. Tob Use Insights. 2019;12: 1179173X19878435. doi:10.1177/1179173X19878435	No	Not meeting inclusion criteria	Title and abstract screening
1183	Caponnetto P, DiPiazza J, Signorelli M, Maglia M, Polosa R. Existing and emerging smoking cessation options for people with schizophrenia spectrum disorders. J Addict Dis. 2018;37: 279–290. doi:10.1080/10550887.2019.1679063	No	Not meeting inclusion criteria	Title and abstract screening
1184	Caponnetto P, Maglia M, Cannella MC, Inguscio L, Buonocore M, Scoglio C, et al. Impact of Different e-Cigarette Generation and Models on Cognitive Performances, Craving and Gesture: A Randomized Cross-Over Trial (CogEcig). Front Psychol. 2017;8: 127. doi:10.3389/fpsyg.2017.00127	No	Not meeting inclusion criteria	Title and abstract screening
1185	Caponnetto P, Maglia M, Prosperini G, Busà B, Polosa R. Carbon monoxide levels after inhalation from new generation heated tobacco products. Respir Res. 2018;19: 164. doi:10.1186/s12931-018-0867-z	No	Not meeting inclusion criteria	Title and abstract screening
1186	Caponnetto P, Polosa R. Approved and emerging smoking cessation treatments for people with schizophrenia spectrum disorders: A narrative review. Health Psychol Res. 2020;8: 9237. doi:10.4081/hpr.2020.9237	No	Not meeting inclusion criteria	Title and abstract screening
1187	Caponnetto P, Triscari S, Vitale NM, Polosa R. Letter to the Editors of Substance Abuse: Research and Treatment; Adolescents Who Vape Nicotine and Their Experiences Vaping: A Qualitative Study; Regarding Dubé et al. (2023). Subst Abuse. 2023;17: 11782218231197959. doi:10.1177/11782218231197959	No	Not meeting inclusion criteria	Title and abstract screening
1188	Caponnetto P, Maglia M, Polosa R. Efficacy of smoking cessation with varenicline plus counselling for e-cigarettes users (VAREVAPE): a protocol for a randomized controlled trial. 2019;15. doi:10.1016/j.conctc.2019.100412	No	Not meeting inclusion criteria	Title and abstract screening
1189	Caponnetto P., DIpiazza J., Kim J., Maglia M., Polosa R. A Single-Arm, Open-Label, Pilot, and Feasibility Study of a High Nicotine Strength E-Cigarette Intervention for Smoking Cessation or Reduction for People with Schizophrenia Spectrum Disorders Who Smoke Cigarettes. Nicotine Tob Res. 2021;23: 1113–1122. doi:10.1093/ntr/ntab005	No	Not meeting inclusion criteria	Title and abstract screening
1190	Caponnetto P., Polosa R. A qualitative study of the views about smoking, licensed cessation aids and e-cigarettes in people with schizophrenia spectrum disorders. J Addict Dis. 2021;39: 152–165. doi:10.1080/10550887.2020.1826097	No	Not meeting inclusion criteria	Title and abstract screening
1191	Carboni L, Ponzoni L, Braida D, Sala M, Gotti C, Zoli M. Altered mRNA Levels of Stress-Related Peptides in Mouse Hippocampus and Caudate-Putamen in Withdrawal after Long-Term Intermittent Exposure to Tobacco Smoke or Electronic Cigarette Vapour. Int J Mol Sci. 2021;22. doi:10.3390/ijms22020599	No	Not meeting inclusion criteria	Title and abstract screening
1192	Cardenas A, Lotfipour S. Age- and Sex-Dependent Nicotine Pretreatment Effects on the Enhancement of Methamphetamine Self-administration in Sprague-Dawley Rats. Nicotine Tob Res Off J Soc Res Nicotine Tob. 2022;24: 1186–1192. doi:10.1093/ntr/ntab218	No	Not meeting inclusion criteria	Title and abstract screening
1193	Cardenas AM. Age, genetic, and sex differences in alpha(alpha)6-containing nicotinic receptors and nicotine-induced behaviors. Diss Abstr Int Sect B Sci Eng. 2023;84: No-Specified.	No	Not meeting inclusion criteria	Title and abstract screening
1194	Cardona S, Calixte R, Rivera A, Islam JY, Vidot DC, Camacho-Rivera M. Perceptions and Patterns of Cigarette and E-Cigarette Use among Hispanics: A Heterogeneity Analysis of the 2017-2019 Health Information National Trends Survey. Int J Environ Res Public Health. 2021;18. doi:10.3390/ijerph18126378	No	Not meeting inclusion criteria	Title and abstract screening
1195	Carpenter MJ, Wahlquist AE, Dahne J, Gray KM, Cummings KM, Warren G, et al. Effect of unguided e-cigarette provision on uptake, use, and smoking cessation among adults who smoke in the USA: a naturalistic, randomised, controlled clinical trial. EClinicalMedicine. 2023;63: 102142. doi:10.1016/j.eclinm.2023.102142	No	Not meeting inclusion criteria	Title and abstract screening
1196	Carr E. Targeting Nicotine Addiction. Clin J Oncol Nurs. 2018;22: 243–244. doi:10.1188/18.CJON.243-244	No	Not meeting inclusion criteria	Title and abstract screening
1197	Carre C., Lafnitzegger A. THE ADOLESCENT WHO CRIED CHEST PAIN: A CURIOUS CHEST MASS. J Hosp Med. 2023;18: S821–S822. doi:10.1002/jhm.13090	No	Not meeting inclusion criteria	Title and abstract screening
1198	Carrillo N. Nicotine dependence and tobacco use disorder treatment. Addict Med Case Evid-Based Guide. 2022; 101–111. doi:10.1007/978-3-030-86430-9_10https://dx.doi.org/10.1007/978-3-030-86430-9_10	No	Not meeting inclusion criteria	Title and abstract screening
1199	Carroll AJ, Huffman MD, Wileyto EP, Khan SS, Fox E, Smith JD, et al. Change in cardiovascular health among adults with current or past major depressive disorder enrolled in intensive smoking cessation treatment. J Affect Disord. 2023;333: 527–534. doi:10.1016/j.jad.2023.04.089	No	Not meeting inclusion criteria	Title and abstract screening

1200	Carroll AJ, Kim K, Miele A, Olonoff M, Leone FT, Schnoll RA, et al. Longitudinal associations between smoking and affect among cancer patients using varenicline to quit smoking. Addict Behav. 2019;95: 206–210. doi:10.1016/j.addbeh.2019.04.003	No	Not meeting inclusion criteria	Title and abstract screening
1201	Carroll DM, Cole A. Racial/ethnic group comparisons of quit ratios and prevalences of cessation-related factors among adults who smoke with a quit attempt. Am J Drug Alcohol Abuse. 2022;48: 58–68. doi:10.1080/00952990.2021.1977310	No	Not meeting inclusion criteria	Title and abstract screening
1202	Carroll DM, Lindgren BR, Dermody SS, Denlinger-Apte R, Egbert A, Cassidy RN, et al. Impact of nicotine reduction in cigarettes on smoking behavior and exposure: Are there differences by race/ethnicity, educational attainment, or gender? Drug Alcohol Depend. 2021;225: 108756. doi:10.1016/j.drugalcdep.2021.108756	No	Not meeting inclusion criteria	Title and abstract screening
1203	Carrott P.W. Commentary: Smoking cessation: No one said it was E-asy. J Thorac Cardiovasc Surg. 2022;163: 311. doi:10.1016/j.jtcvs.2020.12.013	No	Not meeting inclusion criteria	Title and abstract screening
1204	Cartmel B, Fucito LM, Bold KW, Neveu S, Li F, Rojewski AM, et al. No Additional Effect of a Personalized Tobacco Treatment Intervention on Smoking Abstinence in Individuals Eligible for Lung Cancer Screening: Brief Report of a Randomized Trial. J Thorac Oncol. 2023. doi:10.1016/j.jtho.2023.11.012	No	Not meeting inclusion criteria	Title and abstract screening
1205	Cartujano-Barrera F, Azogini C, McIntosh S, Bansal-Travers M, Ossip DJ, Cupertino AP. Developing Graphic Messages for Vaping Prevention Among Black and Latino Adolescents: Participatory Research Approach. J Particip Med. 2021;13: e29945. doi:10.2196/29945	No	Not meeting inclusion criteria	Title and abstract screening
1206	Cartujano-Barrera F, Hernández-Torrez R, Cai X, Orfin RH, Azogini C, Chávez-Iñiguez A, et al. Evaluating the Immediate Impact of Graphic Messages for Vaping Prevention among Black and Latino Adolescents: A Randomized Controlled Trial. Int J Env Res Public Health. 2022;19. doi:10.3390/ijerph191610026	No	Not meeting inclusion criteria	Title and abstract screening
1207	Caruso M, Distefano A, Emma R, Di Rosa M, Carota G, Rust S, et al. Role of Cigarette Smoke on Angiotensin-Converting Enzyme-2 Protein Membrane Expression in Bronchial Epithelial Cells Using an Air-Liquid Interface Model. Front Pharmacol. 2021;12: 652102. doi:10.3389/fphar.2021.652102	No	Not meeting inclusion criteria	Title and abstract screening
1208	Case KR, Lazard AJ, Mackert MS, Perry CL. Source Credibility and E-Cigarette Attitudes: Implications for Tobacco Communication. Health Commun. 2018;33: 1059–1067. doi:10.1080/10410236.2017.1331190	No	Not meeting inclusion criteria	Title and abstract screening
1209	Casseus M, Cooney JM, Wackowski OA. Tobacco Use, Dependence, and Age of Initiation among Youths with Cognitive Disability. J Pediatr. 2022;247: 102-108.e8. doi:10.1016/j.jpeds.2022.04.049	No	Not meeting inclusion criteria	Title and abstract screening
1210	Cassidy R.N., Long V., Tidey J.W., Colby S.M. Validation of an e-cigarette purchase task in advanced generation device users. Nicotine Tob Res. 2021;22: 1851–1859. doi:10.1093/NTR/NTAA060	No	Not meeting inclusion criteria	Title and abstract screening
1211	Cassidy RN, Aston ER, Tidey JW, Colby SM. Behavioral economic demand and delay discounting are differentially associated with cigarette dependence and use in adolescents. Addict Behav. 2020;103: 106225. doi:10.1016/j.addbeh.2019.106225	No	Not meeting inclusion criteria	Title and abstract screening
1212	Cassidy RN, Bello MS, Denlinger-Apte R, Goodwin C, Godin J, Strahley AE, et al. The impact of the COVID-19 pandemic on a sample of US young adults who smoke cigarettes: A qualitative analysis. Addict Behav. 2023;141: 107650. doi:10.1016/j.addbeh.2023.107650	No	Not meeting inclusion criteria	Title and abstract screening
1213	Cassidy RN, Colby SM, Tidey JW, Jackson KM, Cioe PA, Krishnan-Sarin S, et al. Adolescent smokers' response to reducing the nicotine content of cigarettes: Acute effects on withdrawal symptoms and subjective evaluations. Drug Alcohol Depend. 2018;188: 153–160. doi:10.1016/j.drugalcdep.2018.04.006	No	Not meeting inclusion criteria	Title and abstract screening
1214	Cassidy RN, Long V, Tidey JW, Colby SM. Validation of an E-cigarette Purchase Task in Advanced Generation Device Users. Nicotine Tob Res. 2020;22: 1851–1859. doi:10.1093/ntr/ntaa060	No	Not meeting inclusion criteria	Title and abstract screening
1215	Cassidy RN, Tidey JW, Colby SM. Exclusive E-Cigarette Users Report Lower Levels of Respiratory Symptoms Relative to Dual E-Cigarette and Cigarette Users. Nicotine Tob Res. 2020;22: S54–S60. doi:10.1093/ntr/ntaa150	No	Not meeting inclusion criteria	Title and abstract screening
1216	Castro EM, Lotfipour S, Leslie FM. Nicotine on the developing brain. Pharmacol Res. 2023;190: 106716. doi:10.1016/j.phrs.2023.106716	No	Not meeting inclusion criteria	Title and abstract screening
1217	Catala-Valentin A, Bernard JN, Caldwell M, Maxson J, Moore SD, Andl CD. E-Cigarette Aerosol Exposure Favors the Growth and Colonization of Oral Streptococcus mutans Compared to Commensal Streptococci. Microbiol Spectr. 2022;10: e0242121. doi:10.1128/spectrum.02421-21	No	Not meeting inclusion criteria	Title and abstract screening
1218	Catala-Valentin AR, Almeda J, Bernard JN, Cole AM, Cole AL, Moore SD, et al. E-Cigarette Aerosols Promote Oral S. aureus Colonization by Delaying an Immune Response and Bacterial Clearing. Cells. 2022;11. doi:10.3390/cells11050773	No	Not meeting inclusion criteria	Title and abstract screening
1219	Cavalcante TM, Szklo AS, Perez CA, Thrasher JF, Szklo M, Ouimet J, et al. Electronic cigarette awareness, use, and perception of harmfulness in Brazil: findings from a country that has strict regulatory requirements. Cad Saude Publica. 2017;33Suppl 3: e00074416. doi:10.1590/0102-311X00074416	No	Not meeting inclusion criteria	Title and abstract screening
1220	Cavallo DA, Krishnan-Sarin S. Nicotine Use Disorders in Adolescents. Pediatr Clin North Am. 2019;66: 1053–1062. doi:10.1016/j.pcl.2019.08.002	No	Not meeting inclusion criteria	Title and abstract screening
1221	Cavazos-Rehg P, Li X, Kasson E, Kaiser N, Borodovsky J, Grucza RA. Investigating the role of familial and peer-related factors on electronic nicotine delivery systems (ENDS) use among U.S. adolescents. J Adolesc. 2021;87: 98–105. doi:10.1016/j.adolescence.2021.01.003	No	Not meeting inclusion criteria	Title and abstract screening
1222	Celebi T.B., Muller J., Terzella M.J. OSTEOPATHIC CONSIDERATIONS IN PNEUMONIA. Osteopath Fam Physician. 2021;13: 14–20. doi:10.33181/13048	No	Not meeting inclusion criteria	Title and abstract screening
1223	Cen T, Mai Y, Jin J, Huang M, Li M, Wang S, et al. Interleukin-41 diminishes cigarette smoke-induced lung inflammation in mice. Int Immunopharmacol. 2023;124: 110794. doi:10.1016/j.intimp.2023.110794	No	Not meeting inclusion criteria	Title and abstract screening
1224	Centner A.M., Bhide P.G., Salazar G. Nicotine in senescence and atherosclerosis. Cells. 2020;9: 1035. doi:10.3390/cells9041035	No	Not meeting inclusion criteria	Title and abstract screening
1225	Centner AM. The role of smoking and vaping in atherosclerosis. Diss Abstr Int Sect B Sci Eng. 2023;84: No-Specified.	No	Not meeting inclusion criteria	Title and abstract screening
1226	Cerni J, Hosseinzadeh H, Mullan J, Westley-Wise V, Chantrill L, Barclay G, et al. Does Geography Play a Role in the Receipt of End-of-Life Care for Advanced Cancer Patients? Evidence from an Australian Local Health District Population-Based Study. J Palliat Med. 2023;26: 1453–1465. doi:10.1089/jpm.2022.0555	No	Not meeting inclusion criteria	Title and abstract screening
1227	Cerni J, Rhee J, Hosseinzadeh H. Challenges and strategies to improve the provision of end-of-life cancer care in rural and regional communities: Perspectives from Australian rural health professionals. Aust J Rural Health. 2023;31: 714–725. doi:10.1111/ajr.13001	No	Not meeting inclusion criteria	Title and abstract screening
1228	Cerrai S, Potente R, Gorini G, Gallus S, Molinaro S. What is the face of new nicotine users? 2012-2018 e-cigarettes and tobacco use among young students in Italy. Int J Drug Policy. 2020;86: 102941. doi:10.1016/j.drugpo.2020.102941	No	Not meeting inclusion criteria	Title and abstract screening

1229	Cesur F, Atasever Z, Ozoran Y. Impact of vitamin D3 supplementation on COVID-19 vaccine response and immunoglobulin G antibodies in deficient women: a randomized controlled trial. 2023;41: 2860-2867. doi:10.1016/j.vaccine.2023.03.046	No	Not meeting inclusion criteria	Title and abstract screening
1230	Cha S, Amato MS, Papandonatos GD, Edwards G, Berdecio I, Jacobs MA, et al. Changes over time in reasons for quitting vaping among treatment-seeking young people from 2019 to 2022. Addict Behav Rep. 2024;19: 100521. doi:10.1016/j.abrep.2023.100521	No	Not meeting inclusion criteria	Title and abstract screening
1231	Chadi N, Belanger RE. Teen vaping: There is no vapour without fire. Paediatr Child Health. 2020;25: 337–339. doi:10.1093/pch/pxz137	No	Not meeting inclusion criteria	Title and abstract screening
1232	Chadi N, Harris SK, Masonbrink A, Gray NJ, Meyers M, Bhave S, et al. Protecting youth from the risks of electronic cigarettes. J Adolesc Health. 2020;66: 127–131. doi:10.1016/j.jadohealth.2019.10.007https://dx.doi.org/10.1016/j.jadohealth.2019.10.007	No	Not meeting inclusion criteria	Title and abstract screening
1233	Chaffee B.W., Couch E.T., Gansky S.A. Trends in characteristics and multi-product use among adolescents who use electronic cigarettes, United States 2011-2015. PLoS ONE. 2017;12: e0177073. doi:10.1371/journal.pone.0177073	No	Not meeting inclusion criteria	Title and abstract screening
1234	Chaffee BW, Couch ET, Popova L, Halpern-Felsher B. Effects of a Reduced Risk Claim on Adolescents' Smokeless Tobacco Perceptions and Willingness to Use. J Adolesc Health Off Publ Soc Adolesc Med. 2023;73: 445–451. doi:10.1016/j.jadohealth.2023.04.025	No	Not meeting inclusion criteria	Title and abstract screening
1235	Chaffee BW, Cheng J, Couch ET, Hoeft KS, Halpern-Felsher B. Adolescents' Substance Use and Physical Activity Before and During the COVID-19 Pandemic. JAMA Pediatr. 2021;175: 715–722. doi:10.1001/jamapediatrics.2021.0541	No	Not meeting inclusion criteria	Title and abstract screening
1236	Chaffee BW, Lauten K, Sharma E, Everard CD, Duffy K, Park-Lee E, et al. Oral Health in the Population Assessment of Tobacco and Health Study. J Dent Res. 2022;101: 1046–1054. doi:10.1177/00220345221086272	No	Not meeting inclusion criteria	Title and abstract screening
1237	Chague F., Boulin M., Eicher J.-C., Bichat F., Saint-Jalmes M., Cransac A., et al. Smoking in Patients With Chronic Cardiovascular Disease During COVID-19 Lockdown. Front Cardiovasc Med. 2022;9: 845439. doi:10.3389/fcvm.2022.845439	No	Not meeting inclusion criteria	Title and abstract screening
1238	Chague F., Cottin Y., Lhuillier I., Guinchard S., Tribouillard L., Bichat F., et al. Sport-related acute myocardial infarction. Contemporary data from IMACS survey. Eur Heart J. 2021;42: 2450. doi:10.1093/eurheartj/ehab724.2450	No	Not meeting inclusion criteria	Title and abstract screening
1239	Chague F., Kouame M.I.M., Lhuillier I., Maza M., Bichat F., Philippe B., et al. Sport-related myocardial infarction: Context of onset, clinical features and one-year follow-up. Results from the IMACS prospective cohort. Arch Cardiovasc Dis Suppl. 2023;15: 128. doi:10.1016/j.acvdsp.2022.10.247	No	Not meeting inclusion criteria	Title and abstract screening
1240	Chaiton M, Dubray J, Kundu A, Schwartz R. Perceived Impact of COVID on Smoking, Vaping, Alcohol and Cannabis Use Among Youth and Youth Adults in Canada. Can J Psychiatry. 2022;67: 407–409. doi:10.1177/07067437211042132	No	Not meeting inclusion criteria	Title and abstract screening
1241	Chaiton M, Fan J, Bondy SJ, Cohen JE, Dubray J, Eissenberg T, et al. E-Cigarette Dependence and Depressive Symptoms Among Youth. Am J Prev Med. 2023. doi:10.1016/j.amepre.2023.09.020	No	Not meeting inclusion criteria	Title and abstract screening
1242	Chakladar J., Shende N., Li W.T., Ongkeko W.M. Pan-cancer analysis of immune-associated genes and pathways dysregulated by tobacco reveals osteopontin as a key mediator of smoking-associated carcinogenesis. Cancer Res. 2022;82. doi:10.1158/1538-7445.AM2022-1337	No	Not meeting inclusion criteria	Title and abstract screening
1243	Chakma J.K., Dhaliwal R.S., Mehrotra R. White paper on electronic nicotine delivery system. Indian J Med Res Suppl. 2019;149: 574–583. doi:10.4103/ijmr.IJMR_957_19	No	Not meeting inclusion criteria	Title and abstract screening
1244	Chakma J.K., Kumar H., Bhargava S., Khanna T. The e-cigarettes ban in India: an important public health decision. Lancet Public Health. 2020;5: e426. doi:10.1016/S2468-2667%2820%2930063-3	No	Not meeting inclusion criteria	Title and abstract screening
1245	Chalker C, Santana-Davila R, Voutsinas JM, Wu Q, Hwang V, Baik CS, et al. High End-of-Life Health Care Utilization in a Contemporary Cohort of Head and Neck Cancer Patients Treated with Immune Checkpoint Inhibitors. J Palliat Med. 2022;25: 614–619. doi:10.1089/jpm.2021.0323	No	Not meeting inclusion criteria	Title and abstract screening
1246	Champion KE, Krebsbach KR, Stallion CL, Dookeran KA. Mediation of race/ethnicity (R/E) disparities in mental health patient reported outcomes (PROS) among elderly female patients with breast cancer (BC) in the Surveillance, Epidemiology, and end results-medicare health outcomes survey (SEER-MHOS) database. J Clin Oncol. 41: e22527–e22527. doi:10.1200/JCO.2023.41.16_suppl.e22527	No	Not meeting inclusion criteria	Title and abstract screening
1247	Chan L, Harris-Roxas B, Freeman B, MacKenzie R, Woodland L, O'Hara BJ. Attitudes towards the "Shisha No Thanks" campaign video: Content analysis of Facebook comments. Tob Induc Dis. 2022;20: 88. doi:10.18332/tid/153543	No	Not meeting inclusion criteria	Title and abstract screening
1248	Chan-Hosokawa A., Deisher C., Labay L.M. Analytical and Clinical Implications of Emerging Cannabinoid Isomers. J Med Toxicol. 2023;19: 124. doi:10.1007/s13181-023-00930-w	No	Not meeting inclusion criteria	Title and abstract screening
1249	Chand BR, Hosseinzadeh H. Association between e-cigarette use and asthma: a systematic review and meta-analysis. J Asthma. 2022;59: 1722–1731. doi:10.1080/02770903.2021.1971703	No	Not meeting inclusion criteria	Title and abstract screening
1250	Chandy M., Obal D., Wu J.C. Elucidating effects of environmental exposure using human-induced pluripotent stem cell disease modeling. EMBO Mol Med. 2022;14: e13260. doi:10.15252/emmm.202013260	No	Not meeting inclusion criteria	Title and abstract screening
1251	Chang HC, Tsai YW, Shiu MN, Wang YT, Chang PY. Elucidating challenges that electronic cigarettes pose to tobacco control in Asia: a population-based national survey in Taiwan. BMJ Open. 2017;7: e014263. doi:10.1136/bmjopen-2016-014263	No	Not meeting inclusion criteria	Title and abstract screening
1252	Chang JT-C. Characterizing the temporal aspects of tobacco use and related diseases. Diss Abstr Int Sect B Sci Eng. 2023;84: No-Specified.	No	Not meeting inclusion criteria	Title and abstract screening
1253	Chang JT, Mayer M, Jackson RA, Rostron BL, Coleman B, Lee T, et al. Characteristics and patterns of cigarette smoking and vaping by past-year smokers who reported using ENDS to help quit smoking in the past year: Findings from the 2018-2019 Tobacco Use Supplement to the Current Population Survey. Nicotine Tob Res. 2022. doi:10.1093/ntr/ntac199	No	Not meeting inclusion criteria	Title and abstract screening
1254	Chang JT, Mayer M, Jackson RA, Rostron BL, Coleman B, Lee T, et al. Characteristics and Patterns of Cigarette Smoking and Vaping By Past-Year Smokers Who Reported Using Electronic Nicotine Delivery System to Help Quit Smoking in the Past Year: Findings From the 2018-2019 Tobacco Use Supplement to the Current Population Su. Nicotine Tob Res. 2023;25: 596–601. doi:10.1093/ntr/ntac199	No	Not meeting inclusion criteria	Title and abstract screening
1255	Chang LH, Couvy-Duchesne B, Liu M, Medland SE, Verhulst B, Benotsch EG, et al. Association between polygenic risk for tobacco or alcohol consumption and liability to licit and illicit substance use in young Australian adults. Drug Alcohol Depend. 2019;197: 271–279. doi:10.1016/j.drugalcdep.2019.01.015	No	Not meeting inclusion criteria	Title and abstract screening
1256	Chang LH, Whitfield JB, Liu M, Medland SE, Hickie IB, Martin NG, et al. Associations between polygenic risk for tobacco and alcohol use and liability to tobacco and alcohol use, and psychiatric disorders in an independent sample of 13,999 Australian adults. Drug Alcohol Depend. 2019;205: 107704. doi:10.1016/j.drugalcdep.2019.107704	No	Not meeting inclusion criteria	Title and abstract screening
1257	Chapman S., Bareham D., Maziak W. The gateway effect of e-cigarettes: Reflections on main criticisms. Nicotine Tob Res. 2019;21: 695–698. doi:10.1093/ntr/nty067	No	Not meeting inclusion criteria	Title and abstract screening

1258	Charat S.T., Cheng S., Emami A. TREATING CHRONIC PAIN AND COMPLEX PERSISTENT OPIOID DEPENDENCE WITH BUPRENORPHINE/NALOXONE IN A PRIMARY CARE INTERDISCIPLINARY CLINIC. J Gen Intern Med. 2023;38: S601. doi:10.1007/s11606-023-08226-z	No	Not meeting inclusion criteria	Title and abstract screening
1259	Chatterjee S., Caporale A., Tao J.Q., Guo W., Johncola A., Strasser A.A., et al. Acute e-cig inhalation impacts vascular health: A study in smoking naive subjects. Am J Physiol - Heart Circ Physiol. 2021;320: H144–H158. doi:10.1152/AJPHEART.00628.2020	No	Not meeting inclusion criteria	Title and abstract screening
1260	Chaturvedi AK, Freedman ND, Abnet CC. The Evolving Epidemiology of Oral Cavity and Oropharyngeal Cancers. Cancer Res. 2022;82: 2821–2823. doi:10.1158/0008-5472.CAN-22-2124	No	Not meeting inclusion criteria	Title and abstract screening
1261	Chaudhary H., Yousaf Z., Nasir U., Waheed T., Syed K. Spontaneous pneumomediastinum mimicking acute pericarditis. Clin Case Rep. 2021;9: e05156. doi:10.1002/ccr3.5156	No	Not meeting inclusion criteria	Title and abstract screening
1262	Chaumont M., Communi D., Tagliatti V., Colet J.-M., Van de Borne P. Serum proteome and high wattage e-cigarette vaping: A randomized crossover study. J Hypertens. 2021;39: e167. doi:10.1097/01.hjh.0000746252.51817.71	No	Not meeting inclusion criteria	Title and abstract screening
1263	Chehab M. Legislation Related to E-Cigarettes in The Gulf Cooperation Council Countries. Asian Pac J Cancer Prev. 2020;21: 3449–3451. doi:10.31557/APJCP.2020.21.12.3449	No	Not meeting inclusion criteria	Title and abstract screening
1264	Chellian R, Behnood-Rod A, Bruijnzeel AW. Development of Dependence in Smokers and Rodents With Voluntary Nicotine Intake: Similarities and Differences. Nicotine Tob Res. 2023;25: 1229–1240. doi:10.1093/ntr/ntac280	No	Not meeting inclusion criteria	Title and abstract screening
1265	Chellian R, Behnood-Rod A, Bruijnzeel DM, Wilson R, Pandy V, Bruijnzeel AW. Rodent models for nicotine withdrawal. J Psychopharmacol. 2021;35: 1169–1187. doi:10.1177/02698811211005629	No	Not meeting inclusion criteria	Title and abstract screening
1266	Chellian R, Behnood-Rod A, Wilson R, Wilks I, Knight P, Febo M, et al. Exposure to smoke from high- but not low-nicotine cigarettes leads to signs of dependence in male rats and potentiates the effects of nicotine in female rats. Pharmacol Biochem Behav. 2020;196: 172998. doi:10.1016/j.pbb.2020.172998	No	Not meeting inclusion criteria	Title and abstract screening
1267	Chellian R., Behnood-Rod A., Wilson R., Kamble S.H., Sharma A., McCurdy C.R., et al. Adolescent nicotine and tobacco smoke exposure enhances nicotine self-administration in female rats. Neuropharmacology. 2020;176: 108243. doi:10.1016/j.neuropharm.2020.108243	No	Not meeting inclusion criteria	Title and abstract screening
1268	Chellian R, Behnood-Rod A, Bruijnzeel AW. Development of dependence in smokers and rodents with voluntary nicotine intake: similarities and differences. Nicotine Tob Res Off J Soc Res Nicotine Tob. 2022. doi:10.1093/ntr/ntac280	No	Not meeting inclusion criteria	Title and abstract screening
1269	Chen A, Krebs NM, Zhu J, Muscat JE. Nicotine metabolite ratio predicts smoking topography: The Pennsylvania Adult Smoking Study. Drug Alcohol Depend. 2018;190: 89–93. doi:10.1016/j.drugalcdep.2018.06.003	No	Not meeting inclusion criteria	Title and abstract screening
1270	Chen C., Anderson C.M., Babb S.D., Frank R., Wong S., Kuiper N.M., et al. Evaluation of the Asian Smokers' Quitline: A Centralized Service for a Dispersed Population. Am J Prev Med. 2021;60: S154–S162. doi:10.1016/j.amepre.2020.01.033	No	Not meeting inclusion criteria	Title and abstract screening
1271	Chen CH, Wen F-H, Chou W-C, Chen J-S, Chang W-C, Hsieh C-H, et al. Associations of prognostic-awareness-transition patterns with end-of-life care in cancer patients' last month. Support Care Cancer. 2022;30: 5975–5989. doi:10.1007/s00520-022-07007-4	No	Not meeting inclusion criteria	Title and abstract screening
1272	Chen H., Chan Y.L., Thorpe A.E., Pollock C.A., Saad S., Oliver B.G. Inhaled or Ingested, Which Is Worse, E-Vaping or High-Fat Diet? Front Immunol. 2022;13: 913044. doi:10.3389/fimmu.2022.913044	No	Not meeting inclusion criteria	Title and abstract screening
1273	Chen H, Tao X, Cao H, Li B, Sun Q, Wang W, et al. Nicotine exposure exacerbates silica-induced pulmonary fibrosis via STAT3-BDNF-TrkB-mediated epithelial-mesenchymal transition in alveolar type II cells. Food Chem Toxicol Int J Publ Br Ind Biol Res Assoc. 2023;175: 113694. doi:10.1016/j.fct.2023.113694	No	Not meeting inclusion criteria	Title and abstract screening
1274	Chen HC, Chen YY, Chao MR, Chang YZ. Validation of a high-throughput method for simultaneous determination of areca nut and tobacco biomarkers in hair using microwave-assisted extraction and isotope dilution liquid chromatography tandem mass spectrometry. J Pharm Biomed Anal. 2022;216: 114775. doi:10.1016/j.jpba.2022.114775	No	Not meeting inclusion criteria	Title and abstract screening
1275	Chen J, Rao VR. A dynamic model of rational addiction with stockpiling and learning: An empirical examination of e-cigarettes. Manag Sci. 2020;66: 5886–5905. doi:10.1287/mnsc.2019.3490https://dx.doi.org/10.1287/mnsc.2019.3490	No	Not meeting inclusion criteria	Title and abstract screening
1276	Chen L, Arens R, Chidambaram AG, Capponi S, Alshawa L, Claeys TA, et al. Vaping Associated Pulmonary Nontuberculous Mycobacteria. Lung. 2021;199: 21–27. doi:10.1007/s00408-020-00414-6	No	Not meeting inclusion criteria	Title and abstract screening
1277	Chen LS, Baker T, Brownson RC, Carney RM, Jorenby D, Hartz S, et al. Smoking Cessation and Electronic Cigarettes in Community Mental Health Centers: Patient and Provider Perspectives. Community Ment Health J. 2017;53: 695–702. doi:10.1007/s10597-016-0065-8	No	Not meeting inclusion criteria	Title and abstract screening
1278	Chen M, Zhao E, Li M, Xu M, Hao S, Gao Y, et al. Kaempferol inhibits non-homologous end joining repair via regulating Ku80 stability in glioma cancer. Phytomedicine. 2023;116: N.PAG-N.PAG. doi:10.1016/j.phymed.2023.154876	No	Not meeting inclusion criteria	Title and abstract screening
1279	Chen Q, Wang S, Lang J-H. Clinical characteristics and prognostic factors of stage IC ovarian clear cell carcinoma: a Surveillance, Epidemiology, and End Results (SEER) analysis. Arch Gynecol Obstet. 2021;304: 521–529. doi:10.1007/s00404-020-05952-1	No	Not meeting inclusion criteria	Title and abstract screening
1280	Chen Q, Huang Q, Liu W, Zhou X. Advanced glycation end products via skin autofluorescence as a new biomarker for major adverse cardiovascular events: A meta-analysis of prospective studies. Nutr Metab Cardiovasc Dis. 2022;32: 1083–1092. doi:10.1016/j.numecd.2022.01.032	No	Not meeting inclusion criteria	Title and abstract screening
1281	Chen R, Pierce JP, Leas EC, White MM, Kealey S, Strong DR, et al. Use of Electronic Cigarettes to Aid Long-Term Smoking Cessation in the United States: Prospective Evidence From the PATH Cohort Study. Am J Epidemiol. 2020;189: 1529–1537. doi:10.1093/aje/kwaa161	No	Not meeting inclusion criteria	Title and abstract screening
1282	Chen R, Pierce JP, Leas EC, Benmarhnia T, Strong DR, White MM, et al. Effectiveness of e-cigarettes as aids for smoking cessation: evidence from the PATH Study cohort, 2017-2019. Tob Control. 2022. doi:10.1136/tobaccocontrol-2021-056901	No	Not meeting inclusion criteria	Title and abstract screening
1283	Chen R, Pierce JP, Leas EC, Benmarhnia T, Strong DR, White MM, et al. Effectiveness of e-cigarettes as aids for smoking cessation: evidence from the PATH Study cohort, 2017-2019. Tob Control. 2023;32: e145–e152. doi:10.1136/tobaccocontrol-2021-056901	No	Not meeting inclusion criteria	Title and abstract screening
1284	Chen S. CHANGING TRENDS IN THE POPULARITY OF E-CIGARETTE USE AMONG ADOLESCENTS IN THE UNITED STATES. J Am Acad Child Adolesc Psychiatry. 2020;59: S259. doi:10.1016/j.jaac.2020.08.447	No	Not meeting inclusion criteria	Title and abstract screening
1285	Chen T, Wu M, Dong Y, Kong B, Cai Y, Hei C, et al. Effect of e-cigarette refill liquid on follicular development and estrogen secretion in rats. Tob Induc Dis. 2022;20: 36. doi:10.18332/tid/146958	No	Not meeting inclusion criteria	Title and abstract screening
1286	Chen W, Bhatia S, Brohl A, Chandra S, Dakhil S, Fecher L, et al. ADAM Trial: a multicenter, randomized, double-blinded, placebo-controlled, phase 3 trial of adjuvant avelumab (anti-PD-L1 antibody) in Merkel cell carcinoma patients with lymph node metastases; NCT03271372. 2022;142: 2837-. doi:10.1016/j.jid.2022.08.007	No	Not meeting inclusion criteria	Title and abstract screening

1287	Chen X, Wang Y. Author response to: "E-cigarettes and the youngest, not a problem in Europe: No data yet". Am J Prev Med. 2018;54: e81. doi:10.1016/j.amepre.2017.12.009https://dx.doi.org/10.1016/j.amepre.2017.12.009	No	Not meeting inclusion criteria	Title and abstract screening
1288	Chen Y-C. Novel insights into nicotine dependence: The hope for new cessation therapeutics. Diss Abstr Int Sect B Sci Eng. 2023;84: No-Specified.	No	Not meeting inclusion criteria	Title and abstract screening
1289	Chen Y-C, Fowler JP, Wang J, Watson CJW, Sherafat Y, Staben A, et al. The Novel CYP2A6 Inhibitor, DLCI-1, Decreases Nicotine Self-Administration in Mice. J Pharmacol Exp Ther. 2020;372: 21–29. doi:10.1124/jpet.119.260653	No	Not meeting inclusion criteria	Title and abstract screening
1290	Chen Y, Weng S. Reappraisal of the T Category for Solitary Intrahepatic Cholangiocarcinoma by Tumor Size in 611 Early-Stage (T1-2N0M0) Patients After Hepatectomy: a Surveillance, Epidemiology, and End Results (SEER) Analysis. J Gastrointest Surg. 2021;25: 1989–1999. doi:10.1007/s11605-020-04833-x	No	Not meeting inclusion criteria	Title and abstract screening
1291	Chen-Sankey J, Bover Manderski MT, Young WJ, Delnevo CD. Examining the Survey Setting Effect on Current E-Cigarette Use Estimates among High School Students in the 2021 National Youth Tobacco Survey. Int J Env Res Public Health. 2022;19. doi:10.3390/ijerph19116468	No	Not meeting inclusion criteria	Title and abstract screening
1292	Chen-Sankey J, Ganz O, Seidenberg A, Choi K. Effect of a "tobacco-free nicotine" claim on intentions and perceptions of Puff Bar e-cigarette use among non-tobacco-using young adults. Tob Control. 2021. doi:10.1136/tobaccocontrol-2021-056957	No	Not meeting inclusion criteria	Title and abstract screening
1293	Chen-Sankey J, Ganz O, Seidenberg A, Choi K. Effect of a "tobacco-free nicotine" claim on intentions and perceptions of Puff Bar e-cigarette use among non-tobacco-using young adults. Tob Control. 2023;32: 501–504. doi:10.1136/tobaccocontrol-2021-056957	No	Not meeting inclusion criteria	Title and abstract screening
1294	Chen-Sankey J, Jeong M, Wackowski OA, Unger JB, Niederdeppe J, Bernat E, et al. Noticing people, discounts and non-tobacco flavours in e-cigarette ads may increase e-cigarette product appeal among non-tobacco-using young adults. Tob Control. 2023;33: 30–37. doi:10.1136/tobaccocontrol-2022-057269	No	Not meeting inclusion criteria	Title and abstract screening
1295	Chen-Sankey J, Unger JB, Bernat E, Niederdeppe J, Bansal-Travers M, Choi K. Price promotion receipt and use progression of any tobacco, cigarettes, e-cigarettes and cigars among US youth between 2016 and 2018. Tob Control. 2023;32: 225–232. doi:10.1136/tobaccocontrol-2021-056667	No	Not meeting inclusion criteria	Title and abstract screening
1296	Chen-Sankey J., Bover-Manderski M.T. Importance of a Balanced Public Health Approach When Assessing Recent Patterns in the Prevalence of Adult e-Cigarette Use in the US. JAMA Netw Open. 2022;5: E2223274. doi:10.1001/jamanetworkopen.2022.23274	No	Not meeting inclusion criteria	Title and abstract screening
1297	Cheney M.K., Dobbs P.D., Dunlap C., Lu Y., Oehlers J., Hodges E. Young Adult JUUL Users' Beliefs About JUUL. J Adolesc Health. 2021;68: 138–145. doi:10.1016/j.jadohealth.2020.05.039	No	Not meeting inclusion criteria	Title and abstract screening
1298	Cheney MK, Song H, Bhochhibhoya S, Lu Y. Chronic disease as a risk factor for cigarette and e-cigarette use from young adulthood to adulthood. Prev Med Rep. 2023;36: 102473. doi:10.1016/j.pmedr.2023.102473	No	Not meeting inclusion criteria	Title and abstract screening
1299	Cheng E.R., Carroll A.E. Vaping and Youth - First, Do No Harm. JAMA Pediatr. 2020;174: 645–646. doi:10.1001/jamapediatrics.2020.0277	No	Not meeting inclusion criteria	Title and abstract screening
1300	Cheng G, Guo J, Carmella SG, Lindgren B, Ikuemonisan J, Niesen B, et al. Increased acrolein-DNA adducts in buccal brushings of e-cigarette users. Carcinogenesis. 2022;43: 437–444. doi:10.1093/carcin/bgac026	No	Not meeting inclusion criteria	Title and abstract screening
1301	Cheng KA, Nichols H, McAdams HP, Henry TS, Washington L. Imaging of Smoking and Vaping Related Diffuse Lung Injury. Radiol Clin North Am. 2022;60: 941–950. doi:10.1016/j.rcl.2022.06.004	No	Not meeting inclusion criteria	Title and abstract screening
1302	Cheng KW, Liu F, Pesko MF, Levy DT, Fong GT, Cummings KM. Impact of vaping restrictions in public places on smoking and vaping in the United States-evidence using a difference-in-differences approach. Addiction. 2022. doi:10.1111/add.16039	No	Not meeting inclusion criteria	Title and abstract screening
1303	Cheng KW, Liu F, Pesko MF, Levy DT, Fong GT, Cummings KM. Impact of vaping restrictions in public places on smoking and vaping in the United States-evidence using a difference-in-differences approach. Addiction. 2023;118: 160–166. doi:10.1111/add.16039	No	Not meeting inclusion criteria	Title and abstract screening
1304	Cheng-Sheng Yu, Yu-Da Chen, Shy-Shin Chang, Jui-Hsiang Tang, Wu JL, Chang-Hsien Lin, et al. Exploring and predicting mortality among patients with end-stage liver disease without cancer: a machine learning approach. Eur J Gastroenterol Hepatol. 2021;33: 1117–1123. doi:10.1097/MEG.0000000000002169	No	Not meeting inclusion criteria	Title and abstract screening
1305	Chesaniuk M, Sokolovsky AW, Ahluwalia JS, Jackson KM, Mermelstein R. Dependence motives of young adult users of electronic nicotine delivery systems. Addict Behav. 2019;95: 1–5. doi:10.1016/j.addbeh.2019.02.014	No	Not meeting inclusion criteria	Title and abstract screening
1306	Chesney TR, Hallet J. Time at Home as a Patient-Centered End Point for Surgical Cancer Treatment-Reply. JAMA Surg. 2021;156: 794–795. doi:10.1001/jamasurg.2021.0646	No	Not meeting inclusion criteria	Title and abstract screening
1307	Chhina MS. Are e-cigarettes a safer alternative to reduce incidences of oral cancer?. Evid Based Dent. 2023. doi:10.1038/s41432-023-00956-7	No	Not meeting inclusion criteria	Title and abstract screening
1308	ChiCTR2100049568. A prospective randomized controlled study on prevention of anastomotic leakage after laparoscopic radical resection of rectal cancer by serosal suture. 2021. Available: https://www.cochranelibrary.com/central/doi/10.1002/central/CN-02408502/full	No	Not meeting inclusion criteria	Title and abstract screening
1309	ChiCTR2200064487. Application of 68Ga/177Lu-TBM-001 in diagnosis and treatment of bone diseases. 2022. Available: https://www.cochranelibrary.com/central/doi/10.1002/central/CN-02560876/full	No	Not meeting inclusion criteria	Title and abstract screening
1310	ChiCTR2300073069. Multicenter, single-dose, randomized, open, parallel-designed bioequivalence study of carrilizumab for injection with old and new processes in healthy male subjects. 2023. Available: https://www.cochranelibrary.com/central/doi/10.1002/central/CN-02579200/full	No	Not meeting inclusion criteria	Title and abstract screening
1311	ChiCTR2300075994. The safety and efficacy of adjuvant liver suspension technique in patients with complex liver segments undergoing laparoscopic hepatectomy--a prospective, randomized controlled study. 2023. Available: https://www.cochranelibrary.com/central/doi/10.1002/central/CN-02600426/full	No	Not meeting inclusion criteria	Title and abstract screening
1312	Chien JY, Gu YC, Liu CH, Tsai HM, Lee CN, Yang AC, et al. Rapid detection of nicotine and benzoic acid in e-liquids with surface-enhanced Raman scattering and artificial intelligence-assisted spectrum interpretation. J Pharm Biomed Anal. 2023;233: 115456. doi:10.1016/j.jpba.2023.115456	No	Not meeting inclusion criteria	Title and abstract screening
1313	Chin A, Zonfrillo MR, Heinly A, Ford SR, Quintos JB, Topor LS. Screening and counseling for nicotine use in youth with diabetes. Pediatr Diabetes. 2022;23: 157–164. doi:10.1111/pedi.13287	No	Not meeting inclusion criteria	Title and abstract screening
1314	Chinwong S., Saharatchataphong K., Kachai C., Chinwong D. Knowledge and attitude towards electronic cigarettes among university student smokers in Thailand. Tob Induc Dis. 2021;19. doi:10.18332/tid/141040	No	Not meeting inclusion criteria	Title and abstract screening
1315	Chittem M, Eliott J, Olver I. Demonstrating the importance of cultural considerations at end of life utilizing the perspective of Indian patients with cancer. Support Care Cancer. 2022;30: 2515–2525. doi:10.1007/s00520-021-06656-1	No	Not meeting inclusion criteria	Title and abstract screening

1316	Chiu Qua DA. Tobacco Cessation Strategies: It Takes a Village. Med. 2017;Spec No: 54–58.	No	Not meeting inclusion criteria	Title and abstract screening
1317	Cho B, Lee S, Pan Y, Sharma M, Holland K. Association of cancer information seeking behavior with cigarette smoking and E-cigarette use among U.S. adults by education attainment level: A multi-year cross-sectional analysis from a nationally representative sample in 2017-2020. Prev Med. 2023;172: 107550. doi:10.1016/j.ypmed.2023.107550	No	Not meeting inclusion criteria	Title and abstract screening
1318	Cho EJ, Hill B, Lehew C, Rahmandar M, Fadavi S. Adolescents' Awareness of E-Cigarette or Vaping Product Use-Associated Lung Injury (EVALI) and E-Cigarette Use Impact on Health. Pediatr Dent. 2023;45: 125–132.	No	Not meeting inclusion criteria	Title and abstract screening
1319	Cho J, Goldenson NI, Stone MD, McConnell R, Barrington-Trimis JL, Chou C-P, et al. Characterizing Polytobacco Use Trajectories and Their Associations With Substance Use and Mental Health Across Mid-Adolescence. Nicotine Tob Res Off J Soc Res Nicotine Tob. 2018;20: S31–S38. doi:10.1093/ntr/ntx270	No	Not meeting inclusion criteria	Title and abstract screening
1320	Cho YJ, Brinkman MC, Hinton A, Nshimiyimana JD, Mehta T, Adeniji A, et al. The sweet spot study-Developing e-liquid product standards for nicotine form and concentration to improve public health: Protocol for a randomized, double-blinded, crossover study. PLoS One. 2023;18: e0291522. doi:10.1371/journal.pone.0291522	No	Not meeting inclusion criteria	Title and abstract screening
1321	Cho YJ, Thrasher JF, Driezen P, Hitchman SC, Reid JL, Hammond D. Trends in exposure to and perceptions of e-cigarette marketing among youth in England, Canada and the United States between 2017 and 2019. Health Educ Res. 2022;36: 657–668. doi:10.1093/her/cyab039	No	Not meeting inclusion criteria	Title and abstract screening
1322	Cho YJ, Thrasher JF, Gravely S, Alberg A, Borland R, Yong HH, et al. Adult smokers' discussions about vaping with health professionals and subsequent behavior change: a cohort study. Addiction. 2022;117: 2933–2942. doi:10.1111/add.15994	No	Not meeting inclusion criteria	Title and abstract screening
1323	Cho YJ, Thrasher JF, Yong HH, Szklo AS, O'Connor RJ, Bansal-Travers M, et al. Path analysis of warning label effects on negative emotions and quit attempts: A longitudinal study of smokers in Australia, Canada, Mexico, and the US. Soc Sci Med. 2018;197: 226–234. doi:10.1016/j.socscimed.2017.10.003	No	Not meeting inclusion criteria	Title and abstract screening
1324	CHOHAN A.A., CHOUDHURY S., T TAWEESEDT P., N UPADHYAY H., FRANCO R.E.N.E., VAKIL A. A SEVERE CASE OF ORGANIZING PNEUMONIA FROM VAPING AND COCAINE USE. Chest. 2022;162: A2138–A2139. doi:10.1016/j.chest.2022.08.1766	No	Not meeting inclusion criteria	Title and abstract screening
1325	Choi J, Jung H-T, Ferrell A, Woo S, Haddad L. Machine Learning-Based Nicotine Addiction Prediction Models for Youth E-Cigarette and Waterpipe (Hookah) Users. J Clin Med. 2021;10. doi:10.3390/jcm10050972	No	Not meeting inclusion criteria	Title and abstract screening
1326	Choi K, Omole T, Wills T, Merianos AL. E-cigarette-inclusive smoke-free policies, excise taxes, tobacco 21 and changes in youth e-cigarette use: 2017-2019. Tob Control. 2022;31: 758–761. doi:10.1136/tobaccocontrol-2020-056260	No	Not meeting inclusion criteria	Title and abstract screening
1327	Choi K, Wills TA, Inoue-Choi M. E-cigarettes for smoking reduction: a piece of the public health puzzle. Lancet Respir Med. 2021;9: 804–805. doi:10.1016/S2213-2600(21)00071-0	No	Not meeting inclusion criteria	Title and abstract screening
1328	Chong WH, Saha B, Ibrahim A, Smith TC. Dyspnea in a 57-Year-Old Man With Recent Viral Illness. Chest. 2021;160: e19–e24. doi:10.1016/j.chest.2021.01.012	No	Not meeting inclusion criteria	Title and abstract screening
1329	Chong WW, Acar ZI, West ML, Wong F. A Scoping Review on the Medical and Recreational Use of Cannabis During the COVID-19 Pandemic. Cannabis Cannabinoid Res. 2022;7: 591–602. doi:10.1089/can.2021.0054	No	Not meeting inclusion criteria	Title and abstract screening
1330	Chou E.Y., Pelz B.J., Chiu A.M., Soung P.J. All that Wheezes is not Asthma or Bronchiolitis. Crit Care Clin. 2022;38: 213–229. doi:10.1016/j.ccc.2021.11.002	No	Not meeting inclusion criteria	Title and abstract screening
1331	Chou SP, Saha TD, Zhang H, Ruan WJ, Huang B, Grant BF, et al. Prevalence, correlates, comorbidity and treatment of electronic nicotine delivery system use in the United States. Drug Alcohol Depend. 2017;178: 296–301. doi:10.1016/j.drugalcdep.2017.05.026	No	Not meeting inclusion criteria	Title and abstract screening
1332	Choulika S, Le Faou AL. [Smoking cessation among HIV smokers: Experience of a French hospital-based smoking cessation service]. Encephale. 2017;43: 110–113. doi:10.1016/j.encep.2015.12.026	No	Not meeting inclusion criteria	Title and abstract screening
1333	Chrea C., Salzberger T., Abetz-Webb L., Afolalu E.F., Cano S., Rose J., et al. DEVELOPMENT OF A FIT-FOR-PURPOSE TOBACCO AND NICOTINE PRODUCTS DEPENDENCE INSTRUMENT. Value Health. 2018;21: S387. doi:10.1016/j.jval.2018.09.2301	No	Not meeting inclusion criteria	Title and abstract screening
1334	Christensen CH, Chang JT, Rostron BL, Hammad HT, van Bemmel DM, Del Valle-Pinero AY, et al. Biomarkers of Inflammation and Oxidative Stress among Adult Former Smoker, Current E-Cigarette Users-Results from Wave 1 PATH Study. Cancer Epidemiol Biomark Prev. 2021;30: 1947–1955. doi:10.1158/1055-9965.EPI-21-0140	No	Not meeting inclusion criteria	Title and abstract screening
1335	Christian WJ, Valvi NR, Walker CJ. Investigating the Relation between Electronic Cigarette Use and Sleep Duration in Kentucky Using the BRFSS, 2016-2017. South Med J. 2023;116: 326–331. doi:10.14423/SMJ.0000000000001529	No	Not meeting inclusion criteria	Title and abstract screening
1336	Chu A, Harris D. Percutaneous Transesophageal Gastrostomy—A Compassionate Intervention for Malignant Bowel Obstruction in Patients Approaching End of Life (CS346). J Pain Symptom Manage. 2023;65: e563–e563. doi:10.1016/j.jpainsymman.2023.02.108	No	Not meeting inclusion criteria	Title and abstract screening
1337	Chu K-H, Matheny S, Furek A, Sidani J, Radio S, Miller E, et al. Identifying student opinion leaders to lead e-cigarette interventions: protocol for a randomized controlled pragmatic trial. Trials. 2021;22: 31. doi:10.1186/s13063-020-04990-z	No	Not meeting inclusion criteria	Title and abstract screening
1338	Chu KH, Hershey TB, Hoffman BL, Wolynn R, Colditz JB, Sidani JE, et al. Puff Bars, Tobacco Policy Evasion, and Nicotine Dependence: Content Analysis of Tweets. J Med Internet Res. 2022;24: e27894. doi:10.2196/27894	No	Not meeting inclusion criteria	Title and abstract screening
1339	Chu KH, Hershey TB, Sidani JE. Collaborative Public Health Strategies to Combat e-Cigarette Regulation Loopholes. JAMA Pediatr. 2021;175: 1102–1104. doi:10.1001/jamapediatrics.2021.2432	No	Not meeting inclusion criteria	Title and abstract screening
1340	Chu M, Deng J, Hu H, Wang R, Li D, Chen Z, et al. Nicotine transport across calu-3 cell monolayer: effect of nicotine salts and flavored e-liquids. Drug Dev Ind Pharm. 2023;49: 628–636. doi:10.1080/03639045.2023.2263791	No	Not meeting inclusion criteria	Title and abstract screening
1341	Chugh A, Arora M, Jain N, Vidyasagaran A, Readshaw A, Sheikh A, et al. The global impact of tobacco control policies on smokeless tobacco use: a systematic review. Lancet Glob Health. 2023;11: e953–e968. doi:10.1016/S2214-109X(23)00205-X	No	Not meeting inclusion criteria	Title and abstract screening
1342	Chun HR, Cheon E, Hwang JE. Systematic review of changed smoking behaviour, smoking cessation and psychological states of smokers according to cigarette type during the COVID-19 pandemic. BMJ Open. 2022;12: e055179. doi:10.1136/bmjopen-2021-055179	No	Not meeting inclusion criteria	Title and abstract screening
1343	Chun J., Yu M., Kim J., Kim A. E-Cigarette, Cigarette, and Dual Use in Korean Adolescents: A Test of Problem Behavior Theory. J Psychoactive Drugs. 2020;52: 27–36. doi:10.1080/02791072.2020.1712501	No	Not meeting inclusion criteria	Title and abstract screening
1344	Chung S, Bengtson CD, Kim MD, Salathe M. CrossTalk opposing view: E-cigarettes expose users to adverse effects of vapours and the potential for nicotine addiction. J Physiol. 2020;598: 3053–3056. doi:10.1113/JP279271	No	Not meeting inclusion criteria	Title and abstract screening

1345	Chung S., Sailland-Tschudi J., Baumlin-Schmid N., Salathe M. Nicotine-induced airway liquid volume loss depends on apical nicotinic acetylcholine receptors and possibly TRPA1. Am J Respir Crit Care Med. 2017;195. doi:10.1164/ajrccm-conference.2017.A73	No	Not meeting inclusion criteria	Title and abstract screening
1346	Chung S, Baumlin N, Dennis JS, Moore R, Salathe SF, Whitney PL, et al. Electronic Cigarette Vapor with Nicotine Causes Airway Mucociliary Dysfunction Preferentially via TRPA1 Receptors. Am J Respir Crit Care Med. 2019;200: 1134–1145. doi:10.1164/rccm.201811-2087OC	No	Not meeting inclusion criteria	Title and abstract screening
1347	Chung T, Sartor C, Hipwell AE, Grosso A, Jiang Y. Person-centered patterns of substance use during the COVID-19 pandemic and their associations with COVID-related impacts on health and personal finances in young Black and White women. Drug Alcohol Depend. 2022;240: 109620. doi:10.1016/j.drugalcdep.2022.109620	No	Not meeting inclusion criteria	Title and abstract screening
1348	Chung-Hall J, Craig LV, Kyriakos CN, Fong GT. U.S. Food and Drug Administration Must Ban Menthol Cigarettes Without Delay: Lessons From Other Countries. Am J Prev Med. 2023;65: 1192–1195. doi:10.1016/j.amepre.2023.08.001	No	Not meeting inclusion criteria	Title and abstract screening
1349	Chung-Hall J, Fong GT, Meng G, Cummings KM, Hyland A, O'Connor RJ, et al. Evaluating the impact of menthol cigarette bans on cessation and smoking behaviours in Canada: longitudinal findings from the Canadian arm of the 2016-2018 ITC Four Country Smoking and Vaping Surveys. Tob Control. 2022;31: 556–563. doi:10.1136/tobaccocontrol-2020-056259	No	Not meeting inclusion criteria	Title and abstract screening
1350	Chung-Hall J, Fong GT, Meng G, Craig LV. Illicit cigarette purchasing after implementation of menthol cigarette bans in Canada: findings from the 2016-2018 ITC Four Country Smoking and Vaping Surveys. Tob Control. 2023. doi:10.1136/tc-2022-057697	No	Not meeting inclusion criteria	Title and abstract screening
1351	Churchill V, Fairman RT, Brown D, Massey ZB, Ashley DL, Popova L. "I Get the Flavors and It Makes Me Love Vaping More": How and Why Youth Users Modify Electronic Nicotine Delivery Systems. Nicotine Tob Res. 2023;25: 1791–1797. doi:10.1093/ntr/ntad104	No	Not meeting inclusion criteria	Title and abstract screening
1352	Cioe PA, Gordon REF, Guthrie KM, Freiberg MS, Kahler CW. Perceived barriers to smoking cessation and perceptions of electronic cigarettes among persons living with HIV. AIDS Care. 2018;30: 1469–1475. doi:10.1080/09540121.2018.1489103	No	Not meeting inclusion criteria	Title and abstract screening
1353	Cioe PA, Mercurio AN, Lechner W, Costantino CC, Tidey JW, Eissenberg T, et al. A pilot study to examine the acceptability and health effects of electronic cigarettes in HIV-positive smokers. Drug Alcohol Depend. 2020;206: 107678. doi:10.1016/j.drugalcdep.2019.107678	No	Not meeting inclusion criteria	Title and abstract screening
1354	Cipriano-Steffens T, Cursio JF, Hlubocky F, Sumner M, Garnigan-Peters D, Powell J, et al. Improving End of Life Cancer Outcomes Through Development and Implementation of a Spiritual Care Advocate Program. Am J Hosp Palliat Med. 2021;38: 1441–1450. doi:10.1177/1049909121995413	No	Not meeting inclusion criteria	Title and abstract screening
1355	Claborn K, Lippard E, Conway F. Longitudinal Correlates of Suicide Ideation in People who use Drugs during the COVID-19 Pandemic. Res Sq. 2022. doi:10.21203/rs.3.rs-1910465/v1	No	Not meeting inclusion criteria	Title and abstract screening
1356	Claire R., Chamberlain C., Davey M.-A., Cooper S.E., Berlin I., Leonardi-Bee J., et al. Pharmacological interventions for promoting smoking cessation during pregnancy. Cochrane Database Syst Rev. 2020;2020: CD010078. doi:10.1002/14651858.CD010078.pub3	No	Not meeting inclusion criteria	Title and abstract screening
1357	Clark DL, Walley SC. Clinical Progress Note: E-cigarette, or Vaping, Product Use-Associated Lung Injury. J Hosp Med. 2021;16: 485–488. doi:10.12788/jhm.3585	No	Not meeting inclusion criteria	Title and abstract screening
1358	Clausen B, Rinck M, Nizio P, Matoska CT, Zappi C, Smits JAJ, et al. Study protocol for approach bias retraining for nicotine addiction among dual combustible and electronic cigarette users. Contemp Clin Trials. 2023;128: 107145. doi:10.1016/j.cct.2023.107145	No	Not meeting inclusion criteria	Title and abstract screening
1359	Clawson AH, Ruppe NM, Nwankwo CN, Blair AL. Profiles of Nicotine and Cannabis Exposure among Young Adults with Asthma. Behav Med. 2022;48: 18–30. doi:10.1080/08964289.2020.1763904	No	Not meeting inclusion criteria	Title and abstract screening
1360	Clegg H, Howle F, Groom K, Moore R, Hryhorskyj L, Grundy S, et al. Understanding the enablers and barriers to implementing smoke-free NHS sites across acute care trusts in Greater Manchester: results of a hospital staff survey. Future Heal J. 2021;8: e676–e682. doi:10.7861/fhj.2020-0243	No	Not meeting inclusion criteria	Title and abstract screening
1361	Clegg H., Howle F., Groom K., Moore R., Grundy S., Tempowski A., et al. Understanding the barriers and enablers to implementing a smoke free site across acute care trusts in greater manchester; Results of a hospital staff survey. Thorax. 2021;76: A64–A65. doi:10.1136/thorax-2020-BTSabstracts.111	No	Not meeting inclusion criteria	Title and abstract screening
1362	Cleirec G, Desmier E, Lacatus C, Lesgourgues S, Braun A, Peloso C, et al. Efficiency of Inhaled Cannabidiol in Cannabis Use Disorder: The Pilot Study Cannavap. Front Psychiatry. 2022;13: 899221. doi:10.3389/fpsyt.2022.899221	No	Not meeting inclusion criteria	Title and abstract screening
1363	Clendennen SL, Chen B, Sumbe A, Harrell MB. Patterns in Mental Health Symptomatology and Cigarette, E-cigarette, and Marijuana Use Among Texas Youth and Young Adults Amid the Coronavirus Disease 2019 Pandemic. Nicotine Tob Res. 2023;25: 266–273. doi:10.1093/ntr/ntac205	No	Not meeting inclusion criteria	Title and abstract screening
1364	Clifford JS, Lu J, Blondino CT, Do EK, Prom-Wormley EC. The Association Between Health Literacy and Tobacco Use: Results from a Nationally Representative Survey. J Community Health. 2022;47: 63–70. doi:10.1007/s10900-021-01019-7	No	Not meeting inclusion criteria	Title and abstract screening
1365	Clifton M.S. Commentary on long-term outcomes of congenital diaphragmatic hernia: A single institution experience. J Pediatr Surg. 2022;57: 570–571. doi:10.1016/j.jpedsurg.2021.09.033	No	Not meeting inclusion criteria	Title and abstract screening
1366	Coban T., Incesoy A.C., Demir B., Karabiber E., Gul O., Coban O., et al. Evaluation of tobacco product-selling websites accessed using search engines. Turk Thorac J. 2020;21: 377–382. doi:10.5152/TurkThoracJ.2019.190124	No	Not meeting inclusion criteria	Title and abstract screening
1367	Cobb CO, Lester RC, Rudy AK, Hoetger C, Scott M, Austin M, et al. Tobacco-use behavior and toxicant exposure among current dual users of electronic cigarettes and tobacco cigarettes. Exp Clin Psychopharmacol. 2021;29: 625–635. doi:10.1037/pha0000417	No	Not meeting inclusion criteria	Title and abstract screening
1368	Coelho SG, Keough MT, Hodgins DC, Shead NW, Parmar PK, Kim HS. Latent profile analyses of addiction and mental health problems in two large samples. Int J Ment Health Addict. 2023; No-Specified. doi:10.1007/s11469-022-01003-9https://dx.doi.org/10.1007/s11469-022-01003-9	No	Not meeting inclusion criteria	Title and abstract screening
1369	Cohen JE, Krishnan-Sarin S, Eissenberg T, Gould TJ, Berman ML, Bhatnagar A, et al. Balancing Risks and Benefits of E-Cigarettes in the Real World. Am J Public Health. 2022;112: e1–e2. doi:10.2105/AJPH.2021.306607	No	Not meeting inclusion criteria	Title and abstract screening
1370	Cohen S, Brennan B, Banerjee M, Kalemkerian GP. Temporal trends in small cell lung cancer: Analysis of the U.S. Surveillance, Epidemiology and End Results (SEER) database. J Clin Oncol. 41: e20641–e20641. doi:10.1200/JCO.2023.41.16_suppl.e20641	No	Not meeting inclusion criteria	Title and abstract screening
1371	Cohn A, Ehlke S, Cobb CO. Relationship of nicotine deprivation and indices of alcohol use behavior to implicit alcohol and cigarette approach cognitions in smokers. Addict Behav. 2017;67: 58–65. doi:10.1016/j.addbeh.2016.12.004	No	Not meeting inclusion criteria	Title and abstract screening
1372	Cohn AM, Blount BC, Hashibe M. Nonmedical Cannabis Use: Patterns and Correlates of Use, Exposure, and Harm, and Cancer Risk. J Natl Cancer Inst Monogr. 2021;2021: 53–67. doi:10.1093/jncimonographs/lgab006	No	Not meeting inclusion criteria	Title and abstract screening
1373	Cohn AM, Chen S. Age groups differences in the prevalence and popularity of individual tobacco product use in young adult and adult marijuana and tobacco co-users and tobacco-only users: Findings from Wave 4 of the population assessment of tobacco and health study. Drug Alcohol Depend. 2022;233: 109278. doi:10.1016/j.drugalcdep.2022.109278	No	Not meeting inclusion criteria	Title and abstract screening

1374	Cohn AM, Ehlke SJ, Cobb CO, Soule EK. Hookah tobacco smoking in a large urban sample of adult cigarette smokers: Links with alcohol and poly-tobacco use. Addict Behav. 2017;68: 1–5. doi:10.1016/j.addbeh.2016.12.012	No	Not meeting inclusion criteria	Title and abstract screening
1375	Cohn AM, Elmasry H, Wild RC, Johnson AL, Abudayyeh H, Kurti A, et al. Birth Outcomes Associated With E-Cigarette and Non-E-Cigarette Tobacco Product Use During Pregnancy: An Examination of PATH Data Waves 1-5. Nicotine Tob Res. 2023;25: 444–452. doi:10.1093/ntr/ntac111	No	Not meeting inclusion criteria	Title and abstract screening
1376	Cohn AM, Ganz O, Dennhardt AA, Murphy JG, Ehlke S, Cha S, et al. Menthol cigarette smoking is associated with greater subjective reward, satisfaction, and "throat hit", but not greater behavioral economic demand. Addict Behav. 2020;101: 106108. doi:10.1016/j.addbeh.2019.106108	No	Not meeting inclusion criteria	Title and abstract screening
1377	Cole AG, Lienemann BA, Sun J, Chang J, Zhu SH. California School Staff Reports of Seeing Students Vaping at School and Disciplinary Actions. J Sch Nurs. 2022; 10598405221127694. doi:10.1177/10598405221127694	No	Not meeting inclusion criteria	Title and abstract screening
1378	Coleman M., Arunogiri S., Ridley K., White S. SMOKING CESSATION RESOURCES FOR PSYCHIATRISTS. Aust N Z J Psychiatry. 2022;56: 36–37. doi:10.1177/00048674221088686	No	Not meeting inclusion criteria	Title and abstract screening
1379	Coleman SRM, Piper ME, Byron MJ, Bold KW. Dual Use of Combustible Cigarettes and E-cigarettes: a Narrative Review of Current Evidence. Curr Addict Rep. 2022;9: 353–362. doi:10.1007/s40429-022-00448-1	No	Not meeting inclusion criteria	Title and abstract screening
1380	Collar N, O'Neill B, Parham K, Perkins S, Pixley AJ, Gentry E. Identifying Best Practices and Gaps in Late-Stage Lung Cancer: From Diagnosis and Staging Through Survivorship and/or End of Life. J Oncol Navig Surviv. 2022;13: 271–277.	No	Not meeting inclusion criteria	Title and abstract screening
1381	Collins SE, Nelson LA, Stanton J, Mayberry N, Ubay T, Taylor EM, et al. Harm reduction treatment for smoking (HaRT-S): findings from a single-arm pilot study with smokers experiencing chronic homelessness. Subst Abus. 2019;40: 229–239. doi:10.1080/08897077.2019.1572049	No	Not meeting inclusion criteria	Title and abstract screening
1382	Coltin H, Rapoport A, Baxter NN, Nagamuthu C, Nathan PC, Pole JD, et al. Locus-of-care disparities in end-of-life care intensity among adolescents and young adults with cancer: A population-based study using the IMPACT cohort. Cancer 0008543X. 2022;128: 326–334. doi:10.1002/cncr.33926	No	Not meeting inclusion criteria	Title and abstract screening
1383	Comiford AL, Rhoades DA, Dvorak JD, Ding K, Mehta T, Spicer P, et al. Use of Potentially Reduced Exposure Tobacco Products Among American Indian Smokeless Tobacco Users: Associations With Cessation Behaviors and Cotinine Levels. Public Health Rep. 2020;135: 141–149. doi:10.1177/0033354919893031	No	Not meeting inclusion criteria	Title and abstract screening
1384	Conklin D.J. How Irritating! Electronic Cigarettes Not "95% Safer" Than Combustible Cigarettes: Recent Mechanistic Insights Into Endothelial Dysfunction. Arterioscler Thromb Vasc Biol. 2022;42: 1351–1354. doi:10.1161/ATVBAHA.122.318468	No	Not meeting inclusion criteria	Title and abstract screening
1385	Conklin D.J., Schick S., Blaha M.J., Carll A., DeFilippis A., Ganz P., et al. Cardiovascular injury induced by tobacco products: Assessment of risk factors and biomarkers of harm. A Tobacco Centers of Regulatory Science compilation. Am J Physiol - Heart Circ Physiol. 2019;316: H801–H827. doi:10.1152/ajpheart.00591.2018	No	Not meeting inclusion criteria	Title and abstract screening
1386	Conte G, Pacino SA, Urso S, Emma R, Pedullà E, Cibella F, et al. Repeatability of dental shade by digital spectrophotometry in current, former, and never smokers. Odontology. 2022;110: 605–618. doi:10.1007/s10266-022-00692-x	No	Not meeting inclusion criteria	Title and abstract screening
1387	Contreras J., Castillo W.C., Olives E.V. Translation and cross-cultural adaptation of the Awareness and Beliefs about Cancer questionnaire into Spanish to be used in Hispanics living in the US. Qual Life Res. 2022;31: S95. doi:10.1007/s12288-022-01605-2	No	Not meeting inclusion criteria	Title and abstract screening
1388	Conway KP, Green VR, Kasza KA, Silveira ML, Borek N, Kimmel HL, et al. Co-occurrence of tobacco product use, substance use, and mental health problems among adults: Findings from Wave 1 (2013-2014) of the Population Assessment of Tobacco and Health (PATH) Study. Drug Alcohol Depend. 2017;177: 104–111. doi:10.1016/j.drugalcdep.2017.03.032	No	Not meeting inclusion criteria	Title and abstract screening
1389	Conway KP, Green VR, Kasza KA, Silveira ML, Borek N, Kimmel HL, et al. Co-occurrence of tobacco product use, substance use, and mental health problems among youth: Findings from wave 1 (2013-2014) of the population assessment of tobacco and health (PATH) study. Addict Behav. 2018;76: 208–217. doi:10.1016/j.addbeh.2017.08.009	No	Not meeting inclusion criteria	Title and abstract screening
1390	Conyers J., Rajah F. Vaping associated lung disease: A case presentation. Pediatr Crit Care Med. 2021;22: 203–204. doi:10.1097/01.pcc.0000739888.26552.e0	No	Not meeting inclusion criteria	Title and abstract screening
1391	Cook RW, Petkov VI, Byun JS, Bailey CN, Rogers JH, Kurley SJ, et al. Long-term outcomes in a population-based cohort of 2,967 uveal melanoma patients clinically tested with the 15-gene expression profile: A collaborative study with the National Cancer Institute (NCI) Surveillance, Epidemiology, and End Results (SEER) Progr. J Clin Oncol. 41: e21574–e21574. doi:10.1200/JCO.2023.41.16_suppl.e21574	No	Not meeting inclusion criteria	Title and abstract screening
1392	Cook S, Hirschtick JL, Patel A, Brouwer A, Jeon J, Levy DT, et al. A longitudinal study of menthol cigarette use and smoking cessation among adult smokers in the US: Assessing the roles of racial disparities and E-cigarette use. Prev Med. 2022;154: 106882. doi:10.1016/j.ypmed.2021.106882	No	Not meeting inclusion criteria	Title and abstract screening
1393	Cook SF, Fleischer NL, Arenberg DA, Meza R. Author Response to Issues for Studies on E-cigarettes and Chronic Obstructive Pulmonary Disorder. Am J Prev Med. 2023;65: 1198–1199. doi:10.1016/j.amepre.2023.09.011	No	Not meeting inclusion criteria	Title and abstract screening
1394	Cook S, Ortiz Chavez S, Zavala-Arciniega L, Hirschtick JL, Fleischer NL. Trends of Single, Dual, and Polytobacco Use Among School-Based Students in the United States: An Analysis of the National Youth Tobacco Survey. Am J Health Promot AJHP. 2023;37: 1078–1090. doi:10.1177/08901171231191557	No	Not meeting inclusion criteria	Title and abstract screening
1395	Cooke I, Sadruddin S, Patil D, Tripathi S, Nabavizadeh R, Joshi S, et al. A Helpful Tool in the Renal Surgery Armamentarium: Dorsal Lumbotomy nephrectomy for Tumor in Patients with End-Stage Renal Disease. Urology. 2021;153: 327–332. doi:10.1016/j.urology.2021.03.008	No	Not meeting inclusion criteria	Title and abstract screening
1396	Cooke ME, Clifford JS, Do EK, Gilman JM, Maes HH, Peterson RE, et al. Polygenic score for cigarette smoking is associated with ever electronic-cigarette use in a college-aged sample. Addiction. 2022;117: 1071–1078. doi:10.1111/add.15716	No	Not meeting inclusion criteria	Title and abstract screening
1397	Cool T, Baena ARY, Forsberg EC. Clearing the Haze: How Does Nicotine Affect Hematopoiesis before and after Birth? Cancers Basel. 2021;14. doi:10.3390/cancers14010184	No	Not meeting inclusion criteria	Title and abstract screening
1398	Cool T., Rodriguez Y Baena A., Forsberg E.C. Clearing the haze: How does nicotine affect hematopoiesis before and after birth? Cancers. 2022;14: 184. doi:10.3390/cancers14010184	No	Not meeting inclusion criteria	Title and abstract screening
1399	Cooper M, Day HR, Ren C, Oniyide O, Corey CG, Ambrose BK, et al. Correlates of tobacco product initiation among youth and young adults between waves 1-4 of the population assessment of tobacco and Health (PATH) study (2013-2018). Addict Behav. 2022;134: 107396. doi:10.1016/j.addbeh.2022.107396	No	Not meeting inclusion criteria	Title and abstract screening
1400	Cooper M, Loukas A, Case KR, Marti CN, Perry CL. A longitudinal study of risk perceptions and e-cigarette initiation among college students: Interactions with smoking status. Drug Alcohol Depend. 2018;186: 257–263. doi:10.1016/j.drugalcdep.2017.11.027	No	Not meeting inclusion criteria	Title and abstract screening
1401	Cooper M, Loukas A, Harrell MB, Perry CL. College students' perceptions of risk and addictiveness of e-cigarettes and cigarettes. J Am Coll Health J ACH. 2017;65: 103–111. doi:10.1080/07448481.2016.1254638	No	Not meeting inclusion criteria	Title and abstract screening
1402	Cooper MR, Case KR, Hébert ET, Vandewater EA, Raese KA, Perry CL, et al. Characterizing ENDS use in young adults with ecological momentary assessment: Results from a pilot study. Addict Behav. 2019;91: 30–36. doi:10.1016/j.addbeh.2018.11.024	No	Not meeting inclusion criteria	Title and abstract screening

1403	Cooper S., Henderson B. Electronic Nicotine Delivery Systems (ENDS) Flavorants Act As Non-Competitive Antagonists of nAChRs and Contribute To Reinforcement-Related Behavior. FASEB J. 2021;35. doi:10.1096/fasebj.2021.35.S1.03550	No	Not meeting inclusion criteria	Title and abstract screening
1404	Cooper SY, Akers AT, Journigan VB, Henderson BJ. Novel Putative Positive Modulators of alpha4beta2 nAChRs Potentiate Nicotine Reward-Related Behavior. Mol Basel Switz. 2021;26. doi:10.3390/molecules26164793	No	Not meeting inclusion criteria	Title and abstract screening
1405	Cooper SY, Henderson BJ. The Impact of Electronic Nicotine Delivery System (ENDS) Flavors on Nicotinic Acetylcholine Receptors and Nicotine Addiction-Related Behaviors. Molecules. 2020;25. doi:10.3390/molecules25184223	No	Not meeting inclusion criteria	Title and abstract screening
1406	Cordoba J. Electronic cigarette cessation program in caja costarricense del seguro social (CCSS)-Costa Rica: An assessment to guide the future development of an educational intervention for CCSS's health practitioners. Diss Abstr Int Sect B Sci Eng. 2024;85: No-Specified.	No	Not meeting inclusion criteria	Title and abstract screening
1407	Coreas S.I., Rodriquez E.J., Rahman S.G., El-Toukhy S., Compton W.M., Blanco C., et al. Smoking susceptibility and tobacco media engagement among youth never smokers. Pediatrics. 2021;147: e2020017921. doi:10.1542/peds.2020-017921	No	Not meeting inclusion criteria	Title and abstract screening
1408	Corn B, Feldman D, Hull J, O'Rourke M, Bakitas M. Dispositional hope as a potential outcome parameter among patients with advanced malignancy: an analysis of the ENABLE database. 2022;128: 401-409. doi:10.1002/cncr.33907	No	Not meeting inclusion criteria	Title and abstract screening
1409	Cornelius ME, Wang TW, Jamal A, Loretan CG, Neff LJ. Tobacco Product Use Among Adults - United States, 2019. MMWR Morb Mortal Wkly Rep. 2020;69: 1736–1742. doi:10.15585/mmwr.mm6946a4	No	Not meeting inclusion criteria	Title and abstract screening
1410	Cornelius ME, Loretan CG, Jamal A, Davis Lynn BC, Mayer M, Alcantara IC, et al. Tobacco Product Use Among Adults - United States, 2021. MMWR Morb Mortal Wkly Rep. 2023;72: 475–483. doi:10.15585/mmwr.mm7218a1	No	Not meeting inclusion criteria	Title and abstract screening
1411	Cornelius ME, Wang TW, Jamal A, Loretan CG, Willis G, Graham-Glover B, et al. State-Specific Prevalence of Adult Tobacco Product Use and Cigarette Smoking Cessation Behaviors, United States, 2018-2019. Prev Chronic Dis. 2023;20: E107. doi:10.5888/pcd20.230132	No	Not meeting inclusion criteria	Title and abstract screening
1412	Cornish EJ, Brose LS, McNeill A. The Use of Tobacco Industry Vaping Products in the UK and Product Characteristics: A Cross-Sectional Survey. Nicotine Tob Res. 2022;24: 1003–1011. doi:10.1093/ntr/ntab253	No	Not meeting inclusion criteria	Title and abstract screening
1413	Corona G, Sansone A, Pallotti F, Ferlin A, Pivonello R, Isidori AM, et al. People smoke for nicotine, but lose sexual and reproductive health for tar: a narrative review on the effect of cigarette smoking on male sexuality and reproduction. J Endocrinol Invest. 2020;43: 1391–1408. doi:10.1007/s40618-020-01257-x	No	Not meeting inclusion criteria	Title and abstract screening
1414	Correa J.B., Tully L.K., Doran N. Expectancies and reasons for use of e-cigarettes among young adults: A longitudinal analysis. Psychol Addict Behav. 2019;33: 730–736. doi:10.1037/adb0000514	No	Not meeting inclusion criteria	Title and abstract screening
1415	Correa PCRP. No controversy: e-cigarettes are not a treatment for tobacco/nicotine cessation. J Bras Pneumol Publicacao Of Soc Bras Pneumol E Tisilogia. 2022;48: e20220283. doi:10.36416/1806-3756/e20220283	No	Not meeting inclusion criteria	Title and abstract screening
1416	Corrêa PCRP. No controversy: e-cigarettes are not a treatment for tobacco/nicotine cessation. J Bras Pneumol. 2022;48: e20220283. doi:10.36416/1806-3756/e20220283	No	Not meeting inclusion criteria	Title and abstract screening
1417	Costa AR, Lunet N, Martins-Branco D, Gomes B, Lopes S. Hospitalizations at the End of Life Among Chronic Obstructive Pulmonary Disease and Lung Cancer Patients: A Nationwide Study. J Pain Symptom Manage. 2021;62: 48–57. doi:10.1016/j.jpainsymman.2020.11.015	No	Not meeting inclusion criteria	Title and abstract screening
1418	Coughlin LN, Wilson SM, Erwin MC, Beckham JC, Calhoun PS. Cigarette smoking rates among veterans: Association with rurality and psychiatric disorders. Addict Behav. 2019;90: 119–123. doi:10.1016/j.addbeh.2018.10.034	No	Not meeting inclusion criteria	Title and abstract screening
1419	Couraud S, Cortot AB, Pivot XB, Touboul C, Lhomel C, Blay JY, et al. Beliefs and behavior regarding e-cigarettes in a large cross-sectional survey. Prev Med Rep. 2018;10: 332–336. doi:10.1016/j.pmedr.2018.04.009	No	Not meeting inclusion criteria	Title and abstract screening
1420	Cousins M.M., Mayo C., Coughlin L.N., Devasia T.P., Allen S.G., Bryant A.K., et al. Cannabis Use in Patients Seen in an Academic Radiation Oncology Department. Int J Radiat Oncol Biol Phys. 2021;111: e493–e494. doi:10.1016/j.ijrobp.2021.07.1363	No	Not meeting inclusion criteria	Title and abstract screening
1421	Cox M., Hughes M. Osteomyelitis Complicating Digital Ulcer Disease. J Clin Rheumatol. 2021;27: E230–E231. doi:10.1097/RHU.0000000000001325	No	Not meeting inclusion criteria	Title and abstract screening
1422	Cox S, Brown J, Kock L, Shahab L. Prevalence and characteristics of ever regular use of non-combustible nicotine for 1 year or more: a population survey in England. Harm Reduct J. 2021;18: 114. doi:10.1186/s12954-021-00562-9	No	Not meeting inclusion criteria	Title and abstract screening
1423	Cox S, Murray J, Ford A, Holmes L, Robson D, Dawkins L. A cross-sectional survey of smoking and cessation support policies in a sample of homeless services in the United Kingdom. BMC Health Serv Res. 2022;22: 635. doi:10.1186/s12913-022-08038-7	No	Not meeting inclusion criteria	Title and abstract screening
1424	Cox S, Ward E, Ross L, Notley C. How a sample of English stop smoking services and vape shops adapted during the early COVID-19 pandemic: a mixed-methods cross-sectional survey. Harm Reduct J. 2021;18: 95. doi:10.1186/s12954-021-00541-0	No	Not meeting inclusion criteria	Title and abstract screening
1425	Cox S, West R, Notley C, Soar K, Hastings J. Toward an ontology of tobacco, nicotine and vaping products. Addiction. 2022. doi:10.1111/add.16010	No	Not meeting inclusion criteria	Title and abstract screening
1426	Cox S, West R, Notley C, Soar K, Hastings J. Toward an ontology of tobacco, nicotine and vaping products. Addiction. 2023;118: 177–188. doi:10.1111/add.16010	No	Not meeting inclusion criteria	Title and abstract screening
1427	Cox S, Bauld L, Brown R, Carlisle M, Ford A, Hajek P, et al. Evaluating the effectiveness of e-cigarettes compared with usual care for smoking cessation when offered to smokers at homeless centres: protocol for a multi-centre cluster-randomized controlled trial in Great Britain. 2022;117: 2096-2107. doi:10.1111/add.15851	No	Not meeting inclusion criteria	Title and abstract screening
1428	Cox S, Frings D, Ahmed R, Dawkins L. Messages matter: the Tobacco Products Directive nicotine addiction health warning versus an alternative relative risk message on smokers' willingness to use and purchase an electronic cigarette. 2018;8: 136-139. doi:10.1016/j.abrep.2018.09.006	No	Not meeting inclusion criteria	Title and abstract screening
1429	Cox S., Notley C. Response to Campbell et al., (2018) - Health risk perceptions and reasons for use of tobacco products among clients in addictions treatment. Addict Behav. 2019;90: 378–379. doi:10.1016/j.addbeh.2018.11.047	No	Not meeting inclusion criteria	Title and abstract screening
1430	Cox S, Jakes S. Nicotine and e-cigarettes: Rethinking addiction in the context of reduced harm. Int J Drug Policy. 2017;44: 84–85. doi:10.1016/j.drugpo.2017.03.009	No	Not meeting inclusion criteria	Title and abstract screening
1431	Cox S, Notley C. Cleaning up the science: The need for an ontology of consensus scientific terms in e-cigarette research. Addiction. 2021;116: 997–998. doi:10.1111/add.15374https://dx.doi.org/10.1111/add.15374	No	Not meeting inclusion criteria	Title and abstract screening

1432	Cozzolino C, Picchio V, Floris E, Pagano F, Saade W, Peruzzi M, et al. Modified risk tobacco products and cardiovascular repair: still very smoky. Curr Stem Cell Res Ther. 2022. doi:10.2174/1574888X17666220802142532	No	Not meeting inclusion criteria	Title and abstract screening
1433	Crane LA, Asdigian NL, Fitzgerald MD. Looking Cool, Doing Tricks, Managing Stress, and Nicotine Addiction: Youth Perspectives on Nicotine Vaping and Implications for Prevention. Am J Health Promot AJHP. 2023;37: 964–974. doi:10.1177/08901171231189560	No	Not meeting inclusion criteria	Title and abstract screening
1434	Cravo A, Bush J, Sharma G, Savioz R, Martin C, Craige S, et al. A randomised, parallel group study to evaluate the safety profile of an electronic vapour product over 12 weeks. 2016;81 Suppl 1: S1-S14. doi:10.1016/j.yrtph.2016.10.003	No	Not meeting inclusion criteria	Title and abstract screening
1435	Crea F. The complex relationship among heart failure, cancer, and lipid lowering, and an update on cardiomyopathies. Eur Heart J. 2021;42: 3029–3032. doi:10.1093/eurheartj/ehab554	No	Not meeting inclusion criteria	Title and abstract screening
1436	Creager M.A., Hamburg N.M. Smoking Cessation Improves Outcomes in Patients with Peripheral Artery Disease. JAMA Cardiol. 2022;7: 15–16. doi:10.1001/jamacardio.2021.3987	No	Not meeting inclusion criteria	Title and abstract screening
1437	Creamer MR, Wang TW, Babb S, Cullen KA, Day H, Willis G, et al. Tobacco Product Use and Cessation Indicators Among Adults - United States, 2018. MMWR Morb Mortal Wkly Rep. 2019;68: 1013–1019. doi:10.15585/mmwr.mm6845a2	No	Not meeting inclusion criteria	Title and abstract screening
1438	Creo A, Sriram S, Vaughan LE, Weaver AL, Lteif A, Kumar S. Risk of substance use disorders among adolescents and emerging adults with type 1 diabetes: A population-based cohort study. Pediatr Diabetes. 2021;22: 1143–1149. doi:10.1111/pedi.13266	No	Not meeting inclusion criteria	Title and abstract screening
1439	Critcher C.R., Siegel M. Cross-Sectional Analyses Can Evaluate the Plausibility of, but Not Validate, Causal Accounts. Am J Prev Med. 2022;62: e141–e143. doi:10.1016/j.amepre.2021.09.009	No	Not meeting inclusion criteria	Title and abstract screening
1440	Critselis E., Panagiotakos D. Impact of Electronic Cigarette use on Cardiovascular Health: Current Evidence, Causal Pathways, and Public Health Implications. Angiology. 2023. doi:10.1177/00033197231161905	No	Not meeting inclusion criteria	Title and abstract screening
1441	Crosby K. How the Food and Drug Administration Convinced Teens to Rethink Their Relationship With Cigarettes. Am J Prev Med. 2019;56: S1–S4. doi:10.1016/j.amepre.2018.10.013	No	Not meeting inclusion criteria	Title and abstract screening
1442	Cross SH, Yeager K, Curseen KA, Zarrabi AJ, Quest T, Kavalieratos D. Social Deprivation and End-of-Life Care Use Among Adults with Cancer (Sch460). J Pain Symptom Manage. 2023;65: e611–e612. doi:10.1016/j.jpainsymman.2023.02.197	No	Not meeting inclusion criteria	Title and abstract screening
1443	Cross SJ, Lotfipour S, Leslie FM. Mechanisms and genetic factors underlying co-use of nicotine and alcohol or other drugs of abuse. Am J Drug Alcohol Abuse. 2017;43: 171–185. doi:10.1080/00952990.2016.1209512	No	Not meeting inclusion criteria	Title and abstract screening
1444	Crue T, Lee GY, Peng JY-C, Schaunaman N, Agraval H, Day BJ, et al. Single cell RNA-sequencing of human precision-cut lung slices: A novel approach to study the effect of vaping and viral infection on lung health. Innate Immun. 2023;29: 61–70. doi:10.1177/17534259231181029	No	Not meeting inclusion criteria	Title and abstract screening
1445	Crume T. Tobacco Use During Pregnancy. Clin Obstet Gynecol. 2019;62: 128–141. doi:10.1097/GRF.0000000000000413	No	Not meeting inclusion criteria	Title and abstract screening
1446	Crummey A., Keogh A., Ulhaq A., Besi E. Novel nicotine pouches: An investigation into staff awareness. Cogent Med. 2021;8. doi:10.1080/2331205X.2021.2002558	No	Not meeting inclusion criteria	Title and abstract screening
1447	Cruz-Vidal DA, Mull ES, Taveras J, Shell R, Hunt GW, Fowler B, et al. EVALI versus MIS-C, one more overlapping diagnosis to consider. Pediatr Pulmonol. 2021;56: 2918–2924. doi:10.1002/ppul.25558	No	Not meeting inclusion criteria	Title and abstract screening
1448	CTRI/2021/08/035750. Part of a muscle(fascia lata)from the thigh during reconstruction helps in preventing drooling of saliva/food particles from the mouth. 2021. Available: https://www.cochranelibrary.com/central/doi/10.1002/central/CN-02328571/full	No	Not meeting inclusion criteria	Title and abstract screening
1449	CTRI/2022/09/045294. Comparing two methods of giving oxygen during anesthesia i.e. High Flow nasal Oxygen or routinely done bag-mask ventilation in patients with difficult mask ventilation. 2022. Available: https://www.cochranelibrary.com/central/doi/10.1002/central/CN-02473079/full	No	Not meeting inclusion criteria	Title and abstract screening
1450	CTRI/2023/02/049533. Comparing current hospital practices with a new fifteen point system to increase patient satisfaction and shorten hospital stay after head and neck cancer operations. 2023. Available: https://www.cochranelibrary.com/central/doi/10.1002/central/CN-02529313/full	No	Not meeting inclusion criteria	Title and abstract screening
1451	CTRI/2023/10/059362. The comparing study on two different methods of creating a stoma in patients who need a permanent colostomy. 2023. Available: https://www.cochranelibrary.com/central/doi/10.1002/central/CN-02625946/full	No	Not meeting inclusion criteria	Title and abstract screening
1452	Cuccia A.F., Patel M., Amato M.S., Stephens D.K., Yoon S.N., Vallone D.M. Quitting e-cigarettes: Quit attempts and quit intentions among youth and young adults. Prev Med Rep. 2021;21: 101287. doi:10.1016/j.pmedr.2020.101287	No	Not meeting inclusion criteria	Title and abstract screening
1453	CUI Lishuan, LI Linlin, SONG Dun, ZHANG Jinhua. A multidisciplinary team-based model of palliative treatment and nursing for a patient with end-stage cancer. Chin J Integr Nurs. 2022;8: 146–149. doi:10.55111/j.issn2709-1961.202206026	No	Not meeting inclusion criteria	Title and abstract screening
1454	Culbreth R.E., Spears C.A., Brandenberger K., Feresin R., Self-Brown S., Goodfellow L.T., et al. Dual use of electronic cigarettes and traditional cigarettes among adults: Psychosocial correlates and associated respiratory symptoms. Respir Care. 2021;66: 951–959. doi:10.4187/RESPCARE.08381	No	Not meeting inclusion criteria	Title and abstract screening
1455	Cullen G, Neely L, McDonald MR, Cousino KM, Drobek C, Przywara MA. The Effect of Aromatherapy in the Management of Cancer-Related Pain at the End of Life: A Pilot Study. Clin J Oncol Nurs. 2023;27: 404–410. doi:10.1188/23.CJON.404-410	No	Not meeting inclusion criteria	Title and abstract screening
1456	Cummings KM, Ballin S, Sweanor D. The past is not the future in tobacco control. Prev Med. 2020;140: 106183. doi:10.1016/j.ypmed.2020.106183	No	Not meeting inclusion criteria	Title and abstract screening
1457	Cummings KM, Fong GT. Predicting the future of smoking in a rapidly evolving nicotine market-place. Addiction. 2019;114: 3–5. doi:10.1111/add.14785https://dx.doi.org/10.1111/add.14785	No	Not meeting inclusion criteria	Title and abstract screening
1458	Cummings K.J., Lai P.S., Redlich C.A. Occupational and Environmental Contributions to Lung Disease. Clin Chest Med. 2020;41: xiii–xv. doi:10.1016/j.ccm.2020.09.001	No	Not meeting inclusion criteria	Title and abstract screening
1459	Cummings K.M., Hammond D. E-cigarettes: striking the right balance. Lancet Public Health. 2020;5: e180–e181. doi:10.1016/S2468-2667%2820%2930004-9	No	Not meeting inclusion criteria	Title and abstract screening
1460	Cummings KM, Polosa R. E-Cigarette and COPD: Unreliable Conclusion About Health Risks. J Gen Intern Med. 2018;33: 784–785. doi:10.1007/s11606-018-4396-8	No	Not meeting inclusion criteria	Title and abstract screening

1461	Cunningham JK, Ritchey J, Arambula Solomon TG. With socioeconomic status controlled, cigarette use is lower among American Indians/Alaska Natives than whites. Drug Alcohol Depend. 2020;211: 107836. doi:10.1016/j.drugalcdep.2020.107836	No	Not meeting inclusion criteria	Title and abstract screening
1462	Currie ER, Johnston EE, Bakitas M, Roeland E, Lindley LC, Gilbertson-White S, et al. Caregiver Reported Quality of End-of-Life Care of Adolescent and Young Adult Decedents With Cancer. J Palliat Care. 2022;37: 87–92. doi:10.1177/08258597211001991	No	Not meeting inclusion criteria	Title and abstract screening
1463	Curry E, Nemeth JM, Wermert A, Conroy S, Shoben A, Ferketich AK, et al. A Descriptive Report of Electronic Cigarette Use After Participation in a Community-Based Tobacco Cessation Trial. Nicotine Tob Res. 2017;20: 135–139. doi:10.1093/ntr/ntx013	No	Not meeting inclusion criteria	Title and abstract screening
1464	Cwalina S.N., McConnell R., Benowitz N.L., Barrington-Trimis J.L. Tobacco-free Nicotine - New Name, Same Scheme? N Engl J Med. 2021;385: 2406–2408. doi:10.1056/NEJMp2111159	No	Not meeting inclusion criteria	Title and abstract screening
1465	Cwalina SN, Majmundar A, Unger JB, Barrington-Trimis JL, Pentz MA. Adolescent menthol cigarette use and risk of nicotine dependence: Findings from the national Population Assessment on Tobacco and Health (PATH) study. Drug Alcohol Depend. 2020;206: 107715. doi:10.1016/j.drugalcdep.2019.107715	No	Not meeting inclusion criteria	Title and abstract screening
1466	Czaplicki L., Kostygina G., Kim Y., Perks S.N., Szczypka G., Emery S.L., et al. Characterising JUUL-related posts on Instagram. Tob Control. 2019. doi:10.1136/tobaccocontrol-2018-054824	No	Not meeting inclusion criteria	Title and abstract screening
1467	Czaplicki L, Kostygina G, Kim Y, Perks SN, Szczypka G, Emery SL, et al. Characterising JUUL-related posts on Instagram. Tob Control. 2020;29: 612–617. doi:10.1136/tobaccocontrol-2018-054824	No	Not meeting inclusion criteria	Title and abstract screening
1468	Czoli C, Luongo G, Mischki T. Characterizing polysubstance use: What do we know about use of cigarettes, vaping products, cannabis, and alcohol among Canadians? Health Rep. 2023;34: 16–22. doi:10.25318/82-003-x202300400002-eng	No	Not meeting inclusion criteria	Title and abstract screening
1469	D'Angelo H, Land SR, Mayne RG. Assessing Electronic Nicotine Delivery Systems Use at NCI-Designated Cancer Centers in the Cancer Moonshot-funded Cancer Center Cessation Initiative. Cancer Prev Res Phila. 2021;14: 763–766. doi:10.1158/1940-6207.CAPR-21-0105	No	Not meeting inclusion criteria	Title and abstract screening
1470	D'Angelo H., Patel M., Rose S.W. Convenience Store Access and E-cigarette Advertising Exposure Is Associated With Future E-cigarette Initiation Among Tobacco-Naive Youth in the PATH Study (2013-2016). J Adolesc Health. 2021;68: 794–800. doi:10.1016/j.jadohealth.2020.08.030	No	Not meeting inclusion criteria	Title and abstract screening
1471	D'Ruiz CD, O'Connell G, Graff DW, Yan XS. Measurement of cardiovascular and pulmonary function endpoints and other physiological effects following partial or complete substitution of cigarettes with electronic cigarettes in adult smokers. Regul Toxicol Pharmacol. 2017;87: 36–53. doi:10.1016/j.yrtph.2017.05.002	No	Not meeting inclusion criteria	Title and abstract screening
1472	Dacic S., Strobel P. Challenges in lung and thoracic pathology. Virchows Arch. 2021;478. doi:10.1007/s00428-021-03067-9	No	Not meeting inclusion criteria	Title and abstract screening
1473	Dagla I, Gikas E, Tsarbopoulos A. Two Fast GC-MS Methods for the Measurement of Nicotine, Propylene Glycol, Vegetable Glycol, Ethylmaltol, Diacetyl, and Acetylpropionyl in Refill Liquids for E-Cigarettes. Molecules. 2023;28. doi:10.3390/molecules28041902	No	Not meeting inclusion criteria	Title and abstract screening
1474	Dagli E., Dilektasli A.G., Pisinger C. Preventing the initiation of tobacco and e-cigarette use among the youth. ERS Monogr. 2021;2021: 287–307. doi:10.1183/2312508X.10003620	No	Not meeting inclusion criteria	Title and abstract screening
1475	Dahdah A, Jaggers RM, Sreejit G, Johnson J, Kanuri B, Murphy AJ, et al. Immunological Insights into Cigarette Smoking-Induced Cardiovascular Disease Risk. Cells. 2022;11. doi:10.3390/cells11203190	No	Not meeting inclusion criteria	Title and abstract screening
1476	Daher M, Al Rifai M, Mahtta D, Krittanawong C, Berman J, Ullah W, et al. Substance Use and Premature Atherosclerotic Cardiovascular Disease (From the CDC Behavioral Risk Factor Surveillance System [BRFSS] Survey). Am J Cardiol. 2021;152: 177–178. doi:10.1016/j.amjcard.2021.04.027	No	Not meeting inclusion criteria	Title and abstract screening
1477	Dai H. Changes in Flavored Tobacco Product Use Among Current Youth Tobacco Users in the United States, 2014-2017. JAMA Pediatr. 2019;173: 282–284. doi:10.1001/jamapediatrics.2018.4595	No	Not meeting inclusion criteria	Title and abstract screening
1478	Dai H. Heated tobacco product use and associated factors among U.S. youth, 2019. Drug Alcohol Depend. 2020;214: 108150. doi:10.1016/j.drugalcdep.2020.108150	No	Not meeting inclusion criteria	Title and abstract screening
1479	Dai H. Prevalence and Factors Associated With Youth Vaping Cessation Intention and Quit Attempts. Pediatrics. 2021;148. doi:10.1542/peds.2021-050164	No	Not meeting inclusion criteria	Title and abstract screening
1480	Dai H, Hao J. Flavored Tobacco Use Among U.S. Adults by Age Group: 2013-2014. Subst Use Misuse. 2019;54: 315–323. doi:10.1080/10826084.2018.1521428	No	Not meeting inclusion criteria	Title and abstract screening
1481	Dai H. Attitudes Toward Tobacco 21 Among US Youth. Pediatrics. 2017;140. doi:10.1542/peds.2017-0570	No	Not meeting inclusion criteria	Title and abstract screening
1482	Dai H. Tobacco Product Use Among Lesbian, Gay, and Bisexual Adolescents. Pediatrics. 2017;139. doi:10.1542/peds.2016-3276	No	Not meeting inclusion criteria	Title and abstract screening
1483	Dai W., Zhao L., Shi J., Carreno J., Kleinman M., Herman D., et al. Effects of Chronic Electronic Cigarette Vapor Exposure and Standard Cigarette Smoke on Myocardial Infarction and No-reflow in a Rat Model. FASEB J. 2021;35. doi:10.1096/fasebj.2021.35.S1.02900	No	Not meeting inclusion criteria	Title and abstract screening
1484	Daiber A, Kuntic M, Oelze M, Hahad O, Munzel T. E-cigarette effects on vascular function in animals and humans. Pflugers Arch. 2023;475: 783–796. doi:10.1007/s00424-023-02813-z	No	Not meeting inclusion criteria	Title and abstract screening
1485	Dalisay F, Okamoto SK, Teneza J, Dalton C, Lizama K, Pokhrel P, et al. Types of Offers of Combustible Cigarettes, E-Cigarettes, and Betel Nut Experienced by Guam Youths. Int J Env Res Public Health. 2023;20. doi:10.3390/ijerph20196832	No	Not meeting inclusion criteria	Title and abstract screening
1486	Damani J. MARIJUANA-ASSOCIATED CYSTIC LUNG DISEASE. Chest. 2021;160: A1172. doi:10.1016/j.chest.2021.07.1075	No	Not meeting inclusion criteria	Title and abstract screening
1487	Damay VA, Setiawan, Lesmana R, Akbar MR, Lukito AA, Tarawan VM, et al. Electronic Cigarette and Atherosclerosis: A Comprehensive Literature Review of Latest Evidences. Int J Vasc Med. 2022;2022: 4136811. doi:10.1155/2022/4136811	No	Not meeting inclusion criteria	Title and abstract screening
1488	Damay VA, Setiawan S, Lesmana R, Akbar MR, Lukito AA. How Electronic Cigarette Affects the Vascular System. J Smok Cessat. 2022;2022: 3216580. doi:10.1155/2022/3216580	No	Not meeting inclusion criteria	Title and abstract screening
1489	Danielsson M., Lammi A., Siitonen S., Pylkkanen L., Ollgren J., Vasankari T. Smoking and snus use among Finnish young males. Tob Induc Dis. 2018;16: 91. doi:10.18332/tid/83805	No	Not meeting inclusion criteria	Title and abstract screening

1490	Daniluk A, Gawlikowska-Sroka A, Stępien-Słodkowska M, Dzięciołowska-Baran E, Michnik K. Electronic Cigarettes and Awareness of Their Health Effects. Adv Exp Med Biol. 2018;1039: 1–8. doi:10.1007/5584_2017_83	No	Not meeting inclusion criteria	Title and abstract screening
1491	Darabseh M.Z., Selfe J., Morse C.I., Aburub A., Degens H. Does aerobic exercise facilitate smoking cessation: A systematic review of randomized controlled trials with meta-analysis. Physiother U K. 2022;114: e87–e88. doi:10.1016/j.physio.2021.12.029	No	Not meeting inclusion criteria	Title and abstract screening
1492	Darquenne C., Elliott A.R., Puliyakote A.S.K., Alexander L.E.C., Theilmann R.J. Early detection of lung abnormalities in asymptomatic e-cigarette vapers using THC products. J Aerosol Med Pulm Drug Deliv. 2021;34: A8. doi:10.1089/jamp.2021.ab02.abstracts	No	Not meeting inclusion criteria	Title and abstract screening
1493	Das M. E-cigarettes and smoking cessation. Lancet Oncol. 2019;20: e136. doi:10.1016/S1470-2045(19)30067-1	No	Not meeting inclusion criteria	Title and abstract screening
1494	Dautzenberg B, Adler M, Garelik D, Loubrieu JF, Mathern G, Peiffer G, et al. Practical guidelines on e-cigarettes for practitioners and others health professionals. A French 2016 expert's statement. Rev Mal Respir. 2017;34: 155–164. doi:10.1016/j.rmr.2017.01.001	No	Not meeting inclusion criteria	Title and abstract screening
1495	Dautzenberg B., Garelik D. Patients with lung cancer: Are electronic cigarettes harmful or useful? Lung Cancer. 2017;105: 42–48. doi:10.1016/j.lungcan.2016.05.011	No	Not meeting inclusion criteria	Title and abstract screening
1496	DAVENPORT L. Urine metabolites could predict end of life in lung cancer. Chest Physician. 2021;16: 1–5.	No	Not meeting inclusion criteria	Title and abstract screening
1497	Davidoff AJ, Canavan ME, Prsic E, Saphire M, Wang S-Y, Presley CJ. End-of-life patterns of symptom management and cancer-directed care among Medicare beneficiaries with lung cancer: a claims-based analysis. Support Care Cancer. 2021;29: 3921–3932. doi:10.1007/s00520-020-05964-2	No	Not meeting inclusion criteria	Title and abstract screening
1498	Davidson S.M., Boldt G., Louie A.V. How can we better help cancer patients quit smoking? Int J Radiat Oncol Biol Phys. 2017;99: E393. doi:10.1016/j.ijrobp.2017.06.1543	No	Not meeting inclusion criteria	Title and abstract screening
1499	Davies NP, Callister ME, Copeland H, Griffiths S, Holtam L, Lambert P, et al. Opportunistic Non-Governmental Organisation Delivery of a Virtual Stop Smoking Service in England during the COVID-19 Lockdown. Int J Env Res Public Health. 2022;19. doi:10.3390/ijerph19137722	No	Not meeting inclusion criteria	Title and abstract screening
1500	Davila-Garcia M., Dehkordi O. CHARACTERIZATION OF MENTHOL AS A NEUROACTIVE COMPOUND. IBRO Neurosci Rep. 2023;15: S49. doi:10.1016/j.ibneur.2023.08.2152	No	Not meeting inclusion criteria	Title and abstract screening
1501	Davis DR, Fucito LM, Kong G, Jackson A, Bold KW, Baldassarri SR, et al. Adapting research protocols in response to e-cigarette, or vaping, product use associated lung injury: A response to CDC recommendations for e-cigarette trials. Nicotine Tob Res. 2021;23: 619–620. doi:10.1093/ntr/ntaa002https://dx.doi.org/10.1093/ntr/ntaa002	No	Not meeting inclusion criteria	Title and abstract screening
1502	Davis J., Qaqish R.M., Pertzborn M., Ararat E. A case of langerhans cell histiocytosis presenting as bilateral pneumothoraces and cystic lung disease. Am J Respir Crit Care Med. 2021;203. doi:10.1164/ajrccm-conference.2021.203.1_MeetingAbstracts.A3265	No	Not meeting inclusion criteria	Title and abstract screening
1503	Davis ME. Enhancing Nurses' Comfort and Capability With End-of-Life Care for Patients With Cancer. Clin J Oncol Nurs. 2022;26: 363–366. doi:10.1188/22.CJON.363-366	No	Not meeting inclusion criteria	Title and abstract screening
1504	Davis MP, Vanenkevort EA, Elder A, Young A, Correa Ordonez ID, Wojtowicz MJ, et al. The Financial Impact of Palliative Care and Aggressive Cancer Care on End-of-Life Health Care Costs. Am J Hosp Palliat Med. 2023;40: 52–60. doi:10.1177/10499091221098062	No	Not meeting inclusion criteria	Title and abstract screening
1505	Dawkins L, Bauld L, Ford A, Robson D, Hajek P, Parrott S, et al. A cluster feasibility trial to explore the uptake and use of e-cigarettes versus usual care offered to smokers attending homeless centres in Great Britain. PLoS One. 2020;15: e0240968. doi:10.1371/journal.pone.0240968	No	Not meeting inclusion criteria	Title and abstract screening
1506	Dawkins L, Ford A, Bauld L, Balaban S, Tyler A, Cox S. A cross sectional survey of smoking characteristics and quitting behaviour from a sample of homeless adults in Great Britain. Addict Behav. 2019;95: 35–40. doi:10.1016/j.addbeh.2019.02.020	No	Not meeting inclusion criteria	Title and abstract screening
1507	Day-Lewis M, Chiel L, Gaffin J, Lee PY, Chandler MT, Son MB, et al. Pediatric EVALI in the Age of COVID-19/MIS-C: Diagnostic Considerations. Hosp Pediatr. 2022;12: e249–e254. doi:10.1542/hpeds.2021-006426	No	Not meeting inclusion criteria	Title and abstract screening
1508	de Aguiar Assis RL, Junho BT, Campos VR. Lower performance of executive functions predicts higher alcohol and tobacco consumption among adolescents. J Bras Psiquiatr. 2019;68: 146–152. doi:10.1590/0047-2085000000240https://dx.doi.org/10.1590/0047-2085000000240	No	Not meeting inclusion criteria	Title and abstract screening
1509	De Aquino J, Gueorguieva R, Eid T, Sofuoglu M. Differential effects of nicotine delivery rate on abuse potential and urges to smoke: a human laboratory study with implications for tobacco regulatory science. 2021;46: 427-428. doi:10.1038/s41386-021-01238-5	No	Not meeting inclusion criteria	Title and abstract screening
1510	De Aquino J.P., MacLean R.R., Gueorguieva R., DeVito E.E., Eid T., Sofuoglu M. Impact of delivery rate on the acute response to intravenous nicotine: A human laboratory study with implications for regulatory science. Addict Biol. 2022;27: e13161. doi:10.1111/adb.13161	No	Not meeting inclusion criteria	Title and abstract screening
1511	De Genna NM, Goldschmidt L, Day NL, Cornelius MD. Prenatal tobacco exposure, maternal postnatal nicotine dependence and adolescent risk for nicotine dependence: Birth cohort study. Neurotoxicol Teratol. 2017;61: 128–132. doi:10.1016/j.ntt.2017.02.004	No	Not meeting inclusion criteria	Title and abstract screening
1512	De Genna NM, Goldschmidt L, Richardson GA, Cornelius MD, Day NL. Trajectories of pre- and postnatal co-use of cannabis and tobacco predict co-use and drug use disorders in adult offspring. Neurotoxicol Teratol. 2018;70: 10–17. doi:10.1016/j.ntt.2018.09.002	No	Not meeting inclusion criteria	Title and abstract screening
1513	De Genna NM, Richardson GA, Goldschmidt L, Day NL, Cornelius MD. Prenatal exposures to tobacco and cannabis: Associations with adult electronic cigarette use. Drug Alcohol Depend. 2018;188: 209–215. doi:10.1016/j.drugalcdep.2018.03.045	No	Not meeting inclusion criteria	Title and abstract screening
1514	de Granda-Orive J.I., Solano-Reina S., de Granda-Beltran A.M., Jimenez-Ruiz C. Asthma and tobacco: dangerous liaisons. Ann Epidemiol. 2017;27: 406–407. doi:10.1016/j.annepidem.2017.05.011	No	Not meeting inclusion criteria	Title and abstract screening
1515	de Granda-Orive JI, Pascual-Lledó JF, Asensio-Sánchez S, Solano-Reina S, García-Rueda M, Martínez-Muñiz MÁ, et al. Is There an Association Between the Degree of Nicotine Dependence and the Motivation to Stop Smoking? Arch Bronconeumol Engl Ed. 2019;55: 139–145. doi:10.1016/j.arbres.2018.08.003	No	Not meeting inclusion criteria	Title and abstract screening
1516	de Haro D., Arranz Alvarez B., Lopez M., Ramirez Cervantes K., Fernandez Sanchez B., Amador M.L. 1738P Impact of digital platforms on exposure to tobacco and new smoking devices: A survey approach. Ann Oncol. 2023;34: S944. doi:10.1016/j.annonc.2023.09.2692	No	Not meeting inclusion criteria	Title and abstract screening
1517	De Jaeger C, Saskia K, Elena V, Carla L, Hani B, Louis B, et al. ASTCOQ02 a natural telomerase activator, lengthens telomeres in humans in a middle-aged population. A randomized, doubleblind, placebo-controlled study. 2022;13: S30-S31. doi:10.1007/s41999-022-00711-8	No	Not meeting inclusion criteria	Title and abstract screening
1518	De La Garza R, Yoon J, Yammine L, Holst M, Salas R. Preliminary evaluation of the effects of electronic cigarettes versus own cigarette on withdrawal, craving, and smoking severity in tobacco-dependent volunteers. 2016;41: S380-. doi:10.1038/npp.2016.241	No	Not meeting inclusion criteria	Title and abstract screening

1519	De Luca SN, Chan SMH, Dobric A, Wang H, Seow HJ, Brassington K, et al. Cigarette smoke-induced pulmonary impairment is associated with social recognition memory impairments and alterations in microglial profiles within the suprachiasmatic nucleus of the hypothalamus. Brain Behav Immun. 2023;109: 292–307. doi:10.1016/j.bbi.2023.02.005	No	Not meeting inclusion criteria	Title and abstract screening
1520	de Medeiros KS, Pacheco BFP, de Oliveira PE, de Gois Nogueira IL, Beserra Diogenes VR, Fernandes FG, et al. Impact of e-cigarettes as cancer risk: A protocol for systematic review and meta-analysis. Medicine (Baltimore). 2023;102: e32233. doi:10.1097/MD.0000000000032233	No	Not meeting inclusion criteria	Title and abstract screening
1521	de Oliveira G.M.M., Mendes M., Dutra O.P., Achutti A., Fernandes M., Azevedo V., et al. 2019: Recommendations for reducing tobacco consumption in Portuguese-speaking countries-positioning of the federation of Portuguese language cardiology societies. Arq Bras Cardiol. 2019;112: 477–486. doi:10.5935/abc.20190071	No	Not meeting inclusion criteria	Title and abstract screening
1522	De Socio G.V., Ricci E., Maggi P., Orofino G., Squillace N., Menzaghi B., et al. Is It Feasible to Impact on Smoking Habits in HIV-Infected Patients? Mission Impossible from the STOPSHIV Project Cohort. J Acquir Immune Defic Syndr. 2020;83: 496–503. doi:10.1097/QAI.0000000000002284	No	Not meeting inclusion criteria	Title and abstract screening
1523	de Vries E, Leal Arenas FA, van der Heide A, Gempeler Rueda FE, Murillo R, Morales O, et al. Medical decisions concerning the end of life for cancer patients in three Colombian hospitals – a survey study. BMC Palliat Care. 2021;20: 1–12. doi:10.1186/s12904-021-00853-9	No	Not meeting inclusion criteria	Title and abstract screening
1524	de Wylson Fernandes Gomes de Mattos D, Thuler LC, da Silva Lima FF, de Camargo B, Ferman S. The do-not-resuscitate-like (DNRL) order, a medical directive for limiting life-sustaining treatment in the end-of-life care of children with cancer: experience of major cancer center in Brazil. Support Care Cancer. 2022;30: 4283–4289. doi:10.1007/s00520-021-06717-5	No	Not meeting inclusion criteria	Title and abstract screening
1525	DeAtley T, Sokolovsky AW, Snell ML, Tidey J. Mediational pathways of tobacco use among adult daily smokers with psychiatric symptoms in the Population Assessment of Tobacco and Health (PATH) survey. Addict Behav. 2022;129: 107249. doi:10.1016/j.addbeh.2022.107249	No	Not meeting inclusion criteria	Title and abstract screening
1526	DeAtley T, Stone MD, Johnson AC, Mercincavage M, Audrain-McGovern J, Strasser AA. Differences in biobehavioral measures of cigarette smoking by depression symptomology. Addict Behav. 2023;146: 107800. doi:10.1016/j.addbeh.2023.107800	No	Not meeting inclusion criteria	Title and abstract screening
1527	DeAtley T, Harrison A, Cassidy R, Kuo C, Higgins ST, Tidey JW. Subjective experiences, contexts, and risk perceptions of very low nicotine content cigarettes and electronic cigarettes among people with depression and anxiety disorders who smoke. Drug Alcohol Depend. 2023;244: 109767. doi:10.1016/j.drugalcdep.2023.109767	No	Not meeting inclusion criteria	Title and abstract screening
1528	Deery C. What are the health impacts of nicotine and tobacco products on young people?. Evid Based Dent. 2023. doi:10.1038/s41432-023-00945-w	No	Not meeting inclusion criteria	Title and abstract screening
1529	DeGuzman A, Lorenson MY, Walker AM. Bittersweet: relevant amounts of the common sweet food additive, glycerol, accelerate the growth of PC3 human prostate cancer xenografts. BMC Res Notes. 2022;15: 101. doi:10.1186/s13104-022-05990-9	No	Not meeting inclusion criteria	Title and abstract screening
1530	DeHart WB, Kaplan BA, Pope DA, Mellis AM, Bickel WK. The experimental tobacco marketplace: Narrative influence on electronic cigarette substitution. Exp Clin Psychopharmacol. 2019;27: 115–124. doi:10.1037/pha0000233	No	Not meeting inclusion criteria	Title and abstract screening
1531	Del Mastro L, Poggio F, Blondeaux E, De Placido S, Giuliano M, Forestieri V, et al. Fluorouracil and dose-dense adjuvant chemotherapy in patients with early-stage breast cancer (GIM2): end-of-study results from a randomised, phase 3 trial. Lancet Oncol. 2022;23 1077-4114 (Print): 1571–1582. doi:10.1016/S1470-2045(22)00632-5	No	Not meeting inclusion criteria	Title and abstract screening
1532	DeLay T.K., Cobbs P., Stallworth J., Bellot S., Hoopes C., Gongora E., et al. Long-haul ECMO as Bridge to Transplant in COVID-19 ARDS. ASAIO J. 2023;69: 95. doi:10.1097/01.mat.0000943764.37209.ee	No	Not meeting inclusion criteria	Title and abstract screening
1533	Dell LG, Page MK, Leigh NJ, Goniewicz ML. Removal of mango-flavoured Juul pods created opportunity for adulterated mango Juul-compatible pods with altered chemical constituents. Tob Control. 2022;31: s230–s233. doi:10.1136/tc-2022-057476	No	Not meeting inclusion criteria	Title and abstract screening
1534	Delles C., Olfert I.M. Electronic cigarettes: How bad are they for your health? Cardiovasc Res. 2021;116: E46–E66. doi:10.1093/CVR/CVAA041	No	Not meeting inclusion criteria	Title and abstract screening
1535	Delnevo CD, Jeong M, Teotia A, Bover Manderski MM, Singh B, Hrywna M, et al. Communication Between US Physicians and Patients Regarding Electronic Cigarette Use. JAMA Netw Open. 2022;5: e226692. doi:10.1001/jamanetworkopen.2022.6692	No	Not meeting inclusion criteria	Title and abstract screening
1536	Denis-Vatant C, Merieux C, Leclerc L, Duc H, Berton C, Jarrige R, et al. [Relationship between vaping and smoking among 15-year-olds in high school. Results of a descriptive cross-sectional and monocentric observational study conducted in the metropolitan area of Saint-Étienne]. Rev Mal Respir. 2019;36: 850–860. doi:10.1016/j.rmr.2019.04.002	No	Not meeting inclusion criteria	Title and abstract screening
1537	Denlinger-Apte R, Suerken CK, Ross JC, Reboussin BA, Spangler J, Wagoner KG, et al. Decreases in smoking and vaping during COVID-19 stay-at-home orders among a cohort of young adults in the United States. Prev Med. 2022;156: 106992. doi:10.1016/j.ypmed.2022.106992	No	Not meeting inclusion criteria	Title and abstract screening
1538	Denlinger-Apte RL, Koopmeiners JS, Tidey JW, Luo X, Smith TT, Pacek LR, et al. Support for a nicotine reduction policy among participants enrolled in a 20-week trial of very low nicotine content cigarettes. Addict Behav. 2021;114: 106727. doi:10.1016/j.addbeh.2020.106727	No	Not meeting inclusion criteria	Title and abstract screening
1539	Denlinger-Apte RL, Lockhart DE, Strahley AE, Cassidy RN, Donny EC, O'Connor RJ, et al. "I think it's a good idea for the people that's young, the kids, but for someone like me it's a bad idea." - Interviews about a U.S. menthol cigarette ban with people who smoke menthol cigarettes. Drug Alcohol Depend. 2022;232: 109293. doi:10.1016/j.drugalcdep.2022.109293	No	Not meeting inclusion criteria	Title and abstract screening
1540	Depoorter V, Vanschoenbeek K, Decoster L, Silversmit G, Debruyne PR, De Groof I, et al. End-of-Life Care in the Last Three Months before Death in Older Patients with Cancer in Belgium: A Large Retrospective Cohort Study Using Data Linkage. Cancers. 2023;15: 3349. doi:10.3390/cancers15133349	No	Not meeting inclusion criteria	Title and abstract screening
1541	Derefinko K.J., Salgado Garcia F.I., Sumrok D.D. Smoking Cessation for Those Pursuing Recovery from Substance Use Disorders. Med Clin North Am. 2018;102: 781–796. doi:10.1016/j.mcna.2018.02.014	No	Not meeting inclusion criteria	Title and abstract screening
1542	Desai RW, Demir K, Tsolakos N, Moir-Savitz TR, Gaworski CL, Weil R, et al. Comparison of the toxicological potential of two JUUL ENDS products to reference cigarette 3R4F and filtered air in a 90-day nose-only inhalation toxicity study. Food Chem Toxicol Int J Publ Br Ind Biol Res Assoc. 2023;179: 113917. doi:10.1016/j.fct.2023.113917	No	Not meeting inclusion criteria	Title and abstract screening
1543	Deschasse G, Charpentier A, Prod'homme C, Genin M, Delecluse C, Gaxatte C, et al. Transition to Comfort Care Only and End-of-Life Trajectories in an Acute Geriatric Unit: A Secondary Analysis of the DAMAGE Cohort. J Am Med Dir Assoc. 2022;23: 1492–1498. doi:10.1016/j.jamda.2022.04.016	No	Not meeting inclusion criteria	Title and abstract screening
1544	Desorgher L, Berthet A, Rossier J, Bochud F, Froidevaux P. Dosimetry in the lungs of α-particles ((210)Po) and β-particles ((210)Pb) present in the tobacco smoke of conventional cigarettes and heated tobacco products. J Env Radioact. 2023;263: 107178. doi:10.1016/j.jenvrad.2023.107178	No	Not meeting inclusion criteria	Title and abstract screening
1545	Dess RT, Jackson WC, Spratt DE. End Point Definitions and Surrogacy in Prostate Cancer: Will Metastasis-Free Survival Become Event-Free Survival With Advances in Molecular Imaging? J Clin Oncol. 2021;39: 2844–2845. doi:10.1200/JCO.21.00376	No	Not meeting inclusion criteria	Title and abstract screening
1546	Dessaix A, Jardine E, Freeman B, Kameron C. Undermining Australian controls on electronic nicotine delivery systems: illicit imports and illegal sales. Tob Control. 2022;31: 689–690. doi:10.1136/tc-2022-057772	No	Not meeting inclusion criteria	Title and abstract screening
1547	Devarajan A., Azadeh N. A Rare Case of Capecitabine Superimposed with Adalimumab: Drug Induced Eosinophilic Pneumonia. Am J Respir Crit Care Med. 2022;205. doi:10.1164/ajrccm-conference.2022.205.1_MeetingAbstracts.A1396	No	Not meeting inclusion criteria	Title and abstract screening

1548	DeVito EE, Buta E, Sofuoglu M. E-cigarette nicotine dose and flavor: Relationship with appeal, choice, and tobacco use amongst veterans with comorbid psychiatric disorders. Addict Behav. 2019;92: 53–57. doi:10.1016/j.addbeh.2018.12.013	No	Not meeting inclusion criteria	Title and abstract screening
1549	DeVito EE, Krishnan-Sarin S. E-cigarettes: Impact of E-Liquid Components and Device Characteristics on Nicotine Exposure. Curr Neuropharmacol. 2018;16: 438–459. doi:10.2174/1570159X15666171016164430	No	Not meeting inclusion criteria	Title and abstract screening
1550	Dewar EO, Dee EC, Arega MA, Ahn C, Sanford NN. Trends in frequency of e-cigarette use among cancer patients and survivors in the United States, 2014-2018. Addict Behav. 2021;119: 106913. doi:10.1016/j.addbeh.2021.106913	No	Not meeting inclusion criteria	Title and abstract screening
1551	DHILLON V., EMRAN J., ALHADID W., SALAZAR LUNA L.U.I.S., THAPA S.S. INCIDENTAL CONGENITAL PULMONARY AIRWAY MALFORMATION IN THE ADULT PATIENT: TO TREAT OR NOT TO TREAT? Chest. 2023;164: A4741. doi:10.1016/j.chest.2023.07.3072	No	Not meeting inclusion criteria	Title and abstract screening
1552	Dhuliawala S., Kathe N., Payakachat N. PNS69 FACTORS ASSOCIATED WITH INITIATION OF E-CIGARETTE AMONG MIDDLE SCHOOL VS. HIGH SCHOOL CHILDREN. Value Health. 2019;22: S297–S298. doi:10.1016/j.jval.2019.04.1932	No	Not meeting inclusion criteria	Title and abstract screening
1553	Di Cicco M, Sepich M, Beni A, Comberiati P, Peroni DG. How E-cigarettes and vaping can affect asthma in children and adolescents. Curr Opin Allergy Clin Immunol. 2022;22: 86–94. doi:10.1097/ACI.0000000000000807	No	Not meeting inclusion criteria	Title and abstract screening
1554	Di Cicco M., Sepich M., Ragazzo V., Peroni D.G., Comberiati P. Potential effects of E-cigarettes and vaping on pediatric asthma. Minerva Pediatr. 2020;72: 372–382. doi:10.23736/S0026-4946.20.05973-3	No	Not meeting inclusion criteria	Title and abstract screening
1555	di Giacomo E, Colmegna F, Pescatore F, Aspesi F, Fotiadou M, Clerici M. The burden of personality disorders on the DSM 5 addiction to tobacco during pregnancy. Compr Psychiatry. 2018;84: 101–105. doi:10.1016/j.comppsych.2018.04.009	No	Not meeting inclusion criteria	Title and abstract screening
1556	Diamanti A, Galiatsatou A, Sarantaki A, Katsaounou P, Varnakioti D, Lykeridou A. Barriers to Smoking Cessation and Characteristics of Pregnant Smokers in Greece. Maedica Bucur. 2021;16: 405–414. doi:10.26574/maedica.2021.16.3.405	No	Not meeting inclusion criteria	Title and abstract screening
1557	Dias e Silva D, Melo Ferreira V, Swerts DB, Eleoterio GN, Takahashi LiT, Nogueira CV, et al. Timing of specialized palliative care integration and its impact on quality of end-of-life care indicators among Latin American cancer patients. J Clin Oncol. 41: e24168–e24168. doi:10.1200/JCO.2023.41.16_suppl.e24168	No	Not meeting inclusion criteria	Title and abstract screening
1558	Diaz D, Luo X, Hatsukami DK, Donny EC, O'Connor RJ. Cigarette filter ventilation, smoking topography, and subjective effects: A mediational analysis. Drug Alcohol Depend. 2022;241: 109683. doi:10.1016/j.drugalcdep.2022.109683	No	Not meeting inclusion criteria	Title and abstract screening
1559	Dierickx S, Matthys O, Deliens L, De Vleminck A, Lapeire L, Hudson P, et al. O.10.2 - Development of an eHealth program to empower people with advanced cancer and their family caregivers: Presenter(s): Vincent Van Goethem, Ghent University/End-of-life Care Research Group, Belgium. Patient Educ Couns. 2023;109: N.PAG-N.PAG. doi:10.1016/j.pec.2022.10.095	No	Not meeting inclusion criteria	Title and abstract screening
1560	Dierker L, Braymiller J, Rose J, Goodwin R, Selya A. Nicotine dependence predicts cannabis use disorder symptoms among adolescents and young adults. Drug Alcohol Depend. 2018;187: 212–220. doi:10.1016/j.drugalcdep.2018.02.037	No	Not meeting inclusion criteria	Title and abstract screening
1561	Dimitriadis K., Narkiewicz K., Leontsinis I., Thomopoulos K., Iliakis P., Mantzouranis E., et al. SYMPATHETIC NERVE ACTIVITY CHANGES FOLLOWING ACUTE EXPOSURES TO ELECTRONIC AND TOBACCO CIGARETTE SMOKING IN HUMANS. J Am Coll Cardiol. 2021;77: 1613. doi:10.1016/S0735-1097%2821%2902970-3	No	Not meeting inclusion criteria	Title and abstract screening
1562	Dinardo P, Rome ES. Vaping: The new wave of nicotine addiction. Cleve Clin J Med. 2019;86: 789–798. doi:10.3949/ccjm.86a.19118	No	Not meeting inclusion criteria	Title and abstract screening
1563	Ding K, Jiang X, Ni J, Zhang C, Li A, Zhou J. JWA inhibits nicotine-induced lung cancer stemness and progression through CHRNA5/AKT-mediated JWA/SP1/CD44 axis. Ecotoxicol Environ Saf. 2023;259: 115043. doi:10.1016/j.ecoenv.2023.115043	No	Not meeting inclusion criteria	Title and abstract screening
1564	Ding R, Ren X, Sun Q, Sun Z, Duan J. An integral perspective of canonical cigarette and e-cigarette-related cardiovascular toxicity based on the adverse outcome pathway framework. J Adv Res. 2023;48: 227–257. doi:10.1016/j.jare.2022.08.012	No	Not meeting inclusion criteria	Title and abstract screening
1565	Ding R., Ren X., Sun Q., Sun Z., Duan J. An integral perspective of canonical cigarette and e-cigarette-related cardiovascular toxicity based on the adverse outcome pathway framework. J Adv Res. 2022. doi:10.1016/j.jare.2022.08.012	No	Not meeting inclusion criteria	Title and abstract screening
1566	Dionne-Odom J, Azuero A, Taylor R, Dosse C, Bechthold A, Currie E, et al. A lay navigator-led, early palliative care intervention for African American and rural family caregivers of individuals with advanced cancer (Project Cornerstone): results of a pilot randomized trial. 2021. doi:10.1002/cncr.34044	No	Not meeting inclusion criteria	Title and abstract screening
1567	Dionne-Odom J, Azuero A, Taylor R, Dosse C, Bechthold A, Currie E, et al. A lay navigator-led, early palliative care intervention for African American and rural family caregivers of individuals with advanced cancer (Project Cornerstone): results of a pilot randomized trial. 2022;128: 1321-1330. doi:10.1002/cncr.34044	No	Not meeting inclusion criteria	Title and abstract screening
1568	DiPiazza J, Caponnetto P, Askin G, Christos P, Maglia MLP, Gautam R, et al. Sensory experiences and cues among E-cigarette users. Harm Reduct J. 2020;17: 75. doi:10.1186/s12954-020-00420-0	No	Not meeting inclusion criteria	Title and abstract screening
1569	DiSilvio B, Baqdunes M, Alhajhusain A, Cheema T. Smoking Addiction and Strategies for Cessation. Crit Care Nurs Q. 2021;44: 33–48. doi:10.1097/CNQ.0000000000000338	No	Not meeting inclusion criteria	Title and abstract screening
1570	Dixit D, Herbst E, Das S. E-Cigarette Use and Perceptions Among Veterans Receiving Outpatient Treatment in Veterans Affairs Substance Use and Mental Health Clinics. Mil Med. 2020. doi:10.1093/milmed/usaa292	No	Not meeting inclusion criteria	Title and abstract screening
1571	Do EK, Prom-Wormley EC, Fuemmeler BF, Dick DM, Kendler KS, Maes HH. Associations Between Initial Subjective Experiences with Tobacco and Self-Reported Recent Use in Young Adulthood. Subst Use Misuse. 2018;53: 2291–2298. doi:10.1080/10826084.2018.1473435	No	Not meeting inclusion criteria	Title and abstract screening
1572	Do EK, Tulsiani S, Vallone DM, Hair EC. Transitions in Frequent to Daily Tobacco and Nicotine Use among Youth and Young Adults. Subst Use Misuse. 2022;57: 1681–1687. doi:10.1080/10826084.2022.2107674	No	Not meeting inclusion criteria	Title and abstract screening
1573	Do EK, Tulsiani S, Edwards G, Cha S, Amato MS, Hair EC. Treatment-seeking young people enrolled in a United States vaping cessation intervention trial report high frequency of use and nicotine dependence. Prev Med Rep. 2023;36: 102533. doi:10.1016/j.pmedr.2023.102533	No	Not meeting inclusion criteria	Title and abstract screening
1574	Dobbs PD, Lu Y, Dunlap CM, Newcombe KV, Baer CM, Hodges E, et al. Young adults' intention to quit using JUUL. Drug Alcohol Depend. 2021;218: 108399. doi:10.1016/j.drugalcdep.2020.108399	No	Not meeting inclusion criteria	Title and abstract screening
1575	Dobbs PD, Hodges EJ, Dunlap CM, Cheney MK. Potential risk factors for cigarette use among a sample of college JUUL users. J Am Coll Health. 2022;70: 1321–1325. doi:10.1080/07448481.2020.1806850	No	Not meeting inclusion criteria	Title and abstract screening
1576	Dogar O, Keding A, Gabe R, Marshall AM, Huque R, Barua D, et al. Cytisine for smoking cessation in patients with tuberculosis: a multicentre, randomised, double-blind, placebo-controlled phase 3 trial. Lancet Glob Health. 2020;8: e1408–e1417. doi:10.1016/S2214-109X(20)30312-0	No	Not meeting inclusion criteria	Title and abstract screening

1577	Domenico L., Deremer C.E., Nichols K.L., Campbell C., Moreau J.R., Childs G.S., et al. Combatting the epidemic of e-cigarette use and vaping among students and transitional-age youth. Curr Psychopharmacol. 2021;10: 5–16. doi:10.2174/2211556009999200613224100	No	Not meeting inclusion criteria	Title and abstract screening
1578	Donaldson EA, Hoffman AC, Zandberg I, Blake KD. Media exposure and tobacco product addiction beliefs: Findings from the 2015 Health Information National Trends Survey (HINTS-FDA 2015). Addict Behav. 2017;72: 106–113. doi:10.1016/j.addbeh.2017.04.001	No	Not meeting inclusion criteria	Title and abstract screening
1579	Donaldson SI, Dormanesh A, Escobedo P, Majmundar A, Kirkpatrick M, Allem JP. The impact of e-cigarette product place in music videos on susceptibility to use e-cigarettes among young adults: An experimental investigation. Addict Behav. 2022;130: 107307. doi:10.1016/j.addbeh.2022.107307	No	Not meeting inclusion criteria	Title and abstract screening
1580	Donaldson SI, Dormanesh A, Perez C, Majmundar A, Allem JP. Association Between Exposure to Tobacco Content on Social Media and Tobacco Use: A Systematic Review and Meta-analysis. JAMA Pediatr. 2022;176: 878–885. doi:10.1001/jamapediatrics.2022.2223	No	Not meeting inclusion criteria	Title and abstract screening
1581	Donaldson SI, Dormanesh A, Perez C, Zaffer MO, Majmundar A, Unger JB, et al. Monitoring the Official YouTube Channels of E-Cigarette Companies: A Thematic Analysis. Health Educ Behav. 2023;50: 677–682. doi:10.1177/10901981221148964	No	Not meeting inclusion criteria	Title and abstract screening
1582	Dong Y., Chenna A. DILEMMA OF VAPING-ASSOCIATED LUNG INJURY IN PATIENT PRESENTING WITH SHORTNESS OF BREATH AND ACUTE KIDNEY INJURY. Chest. 2020;158: A2365. doi:10.1016/j.chest.2020.08.2008	No	Not meeting inclusion criteria	Title and abstract screening
1583	Donny E. Reducing harm by targeting the addictiveness of combusted tobacco products through regulated reductions in nicotine content. Cancer Res. 2017;77. doi:10.1158/1538-7445.AM2017-SY01-03	No	Not meeting inclusion criteria	Title and abstract screening
1584	Donny EC, Walker N, Hatsukami D, Bullen C. Reducing the nicotine content of combusted tobacco products sold in New Zealand. Tob Control. 2017;26: e37–e42. doi:10.1136/tobaccocontrol-2016-053186	No	Not meeting inclusion criteria	Title and abstract screening
1585	Doot R., Young A., Dominguez T., Helili Z., Keyser H., Goodarz J., et al. Blood pool selection for quantifying lung inflammation via [18 F]NOS uptake in nicotine users and healthy humans. J Nucl Med. 2020;61.	No	Not meeting inclusion criteria	Title and abstract screening
1586	Doran N, Brikmanis K, Petersen A, Delucchi K, Al-Delaimy WK, Luczak S, et al. Does e-cigarette use predict cigarette escalation? A longitudinal study of young adult non-daily smokers. Prev Med. 2017;100: 279–284. doi:10.1016/j.ypmed.2017.03.023	No	Not meeting inclusion criteria	Title and abstract screening
1587	Doran N, Correa JB, Myers MG, Tully L. Associations Between Self-Reported and Biological Measures of Nicotine Consumption Among Young Adult Nondaily Cigarette Smokers. Am J Addict. 2020;29: 471–475. doi:10.1111/ajad.13052	No	Not meeting inclusion criteria	Title and abstract screening
1588	Douglas C.E., Henson R., Drope J., Wender R.C. The American Cancer Society public health statement on eliminating combustible tobacco use in the United States. CA Cancer J Clin. 2018;68: 240–245. doi:10.3322/caac.21455	No	Not meeting inclusion criteria	Title and abstract screening
1589	Dowd A.N., Tiffany S.T. Comparison of Tobacco and Electronic Cigarette Reward Value Measured during a Cue-Reactivity Task: An Extension of the Choice Behavior under Cued Conditions Procedure. Nicotine Tob Res. 2019;21: 1394–1400. doi:10.1093/ntr/nty143	No	Not meeting inclusion criteria	Title and abstract screening
1590	Dowd AN. An examination of objective and self-report measures of ad libitum electronic cigarette use: Identifying patterns of puffing behavior and evaluating self-report items. Diss Abstr Int Sect B Sci Eng. 2023;84: No-Specified.	No	Not meeting inclusion criteria	Title and abstract screening
1591	Dowd AN, Thrul J, Czaplicki L, Kennedy RD, Moran MB, Spindle TR. A Cross-Sectional Survey on Oral Nicotine Pouches: Characterizing Use-Motives, Topography, Dependence Levels, and Adverse Events. Nicotine Tob Res Off J Soc Res Nicotine Tob. 2023. doi:10.1093/ntr/ntad179	No	Not meeting inclusion criteria	Title and abstract screening
1592	Drake EK, Weeks LE, van Manen M, Curran J, McKibbon S. The delivery of palliative and end-of-life care to adolescents and young adults living with cancer: a scoping review protocol. JBI Evid Synth. 2021;19: 3384–3393. doi:10.11124/JBIES-20-00444	No	Not meeting inclusion criteria	Title and abstract screening
1593	Drake EK, Weeks LE, van Manen M, Shin HD, Wong H, Taylor D, et al. The Delivery of Palliative and End-of-Life Care to Adolescents and Young Adults Living with Cancer: A Scoping Review. J Adolesc Young Adult Oncol. 2023;12: 611–624. doi:10.1089/jayao.2023.0013	No	Not meeting inclusion criteria	Title and abstract screening
1594	Drazen JM, Morrissey S, Campion EW. The Dangerous Flavors of E-Cigarettes. N Engl J Med. 2019;380: 679–680. doi:10.1056/NEJMe1900484	No	Not meeting inclusion criteria	Title and abstract screening
1595	Dresler C. Current status of smoking cessation program (tobacco control). J Thorac Oncol. 2017;12: S1602.	No	Not meeting inclusion criteria	Title and abstract screening
1596	Dresler C. Heat-Not-Burn Tobacco: Real Risk Reduction or Industry's Next Promise. J Thorac Oncol. 2018;13: S271–S272. doi:10.1016/j.jtho.2018.08.153	No	Not meeting inclusion criteria	Title and abstract screening
1597	Driezen P, Gravely S, Wadsworth E, Smith DM, Loewen R, Hammond D, et al. Increasing Cannabis Use Is Associated With Poorer Cigarette Smoking Cessation Outcomes: Findings From the ITC Four Country Smoking and Vaping Surveys, 2016-2018. Nicotine Tob Res. 2022;24: 53–59. doi:10.1093/ntr/ntab122	No	Not meeting inclusion criteria	Title and abstract screening
1598	Driezen P, Kasza KA, Gravely S, Thompson ME, Fong GT, Michael Cummings K, et al. Was COVID-19 associated with increased cigarette purchasing, consumption, and smoking at home among US smokers in early 2020? Findings from the US arm of the International Tobacco Control (ITC) Four Country Smoking and Vaping Survey. Addict Behav. 2022;129: 107276. doi:10.1016/j.addbeh.2022.107276	No	Not meeting inclusion criteria	Title and abstract screening
1599	DRKS00007930. Acute Effect of „electronic – cigarette"-use on vascular endothelial function. 2015. Available: https://www.cochranelibrary.com/central/doi/10.1002/central/CN-01803479/full	No	Not meeting inclusion criteria	Title and abstract screening
1600	DRKS00011406. Retraining of dysfunctional behavioral tendencies using an Approach-Avoidance Task in tobacco dependence. 2016. Available: https://www.cochranelibrary.com/central/doi/10.1002/central/CN-01858283/full	No	Not meeting inclusion criteria	Title and abstract screening
1601	DRKS00014652. Take control! Evaluation of a novel training to improve self-control in tobacco dependence. 2018. Available: https://www.cochranelibrary.com/central/doi/10.1002/central/CN-01899314/full	No	Not meeting inclusion criteria	Title and abstract screening
1602	DRKS00019221. The efficacy of Cognitive Bias Modification as an add-on in smoking cessation: a randomized-controlled double-blind study. 2019. Available: https://www.cochranelibrary.com/central/doi/10.1002/central/CN-02067411/full	No	Not meeting inclusion criteria	Title and abstract screening
1603	DRKS00023788. „Meine Zeit ohne": prevention and health promotion at vocational schools with a focus on substance use and internet-related disorders. 2021. Available: https://www.cochranelibrary.com/central/doi/10.1002/central/CN-02238900/full	No	Not meeting inclusion criteria	Title and abstract screening
1604	DRKS00024200. International Euro Ewing (iEuroEwing) trial for treatment optimisation in patients with Ewing sarcoma. 2023. Available: https://www.cochranelibrary.com/central/doi/10.1002/central/CN-02572803/full	No	Not meeting inclusion criteria	Title and abstract screening
1605	DRKS00026186. Influence of a music intervention on subjective and objective pain and stress parameters in pain patients. 2022. Available: https://www.cochranelibrary.com/central/doi/10.1002/central/CN-02377828/full	No	Not meeting inclusion criteria	Title and abstract screening

1606	Drobes DJ. Pharmacological treatment of tobacco use disorder. APA Handb Psychopharmacol. 2019; 609–629. doi:10.1037/0000133-027https://dx.doi.org/10.1037/0000133-027	No	Not meeting inclusion criteria	Title and abstract screening
1607	Du P, Bascom R, Fan T, Sinharoy A, Yingst J, Mondal P, et al. Changes in Flavor Preference in a Cohort of Long-Term Electronic Cigarette Users. Ann Am Thorac Soc. 2020;17: 573–581. doi:10.1513/AnnalsATS.201906-472OC	No	Not meeting inclusion criteria	Title and abstract screening
1608	Duan Z, Hamilton-Moseley KR, McNeel TS, Berg CJ, Choi K. Cumulative Exposure to E-Cigarette Coupons and Changes in E-Cigarette Use Among U.S. Adults. Am J Prev Med. 2023. doi:10.1016/j.amepre.2023.09.001	No	Not meeting inclusion criteria	Title and abstract screening
1609	Duan Z, Kasson E, Ruchelli S, Rajamahanty A, Williams R, Sridharan P, et al. Assessment of Online Marketing and Sales Practices Among Recreational Cannabis Retailers in Five U.S. Cities. Cannabis Cannabinoid Res. 2023. doi:10.1089/can.2022.0334	No	Not meeting inclusion criteria	Title and abstract screening
1610	Duan Z, Le D, Ciceron AC, Dickey-Chasins R, Wysota CN, Bar-Zeev Y, et al. "It's like if a vape pen and a cigarette had a baby": a mixed methods study of perceptions and use of IQOS among US young adults. Health Educ Res. 2022;37: 364–377. doi:10.1093/her/cyac019	No	Not meeting inclusion criteria	Title and abstract screening
1611	Duan Z, Levine H, Bar-Zeev Y, Cui Y, LoParco CR, Wang Y, et al. The impacts of electronic cigarette health warning labels on use intentions and perceptions: A cross-sectional study of US and Israeli adults who use tobacco. J Public Health Res. 2023;12: 22799036231214396. doi:10.1177/22799036231214396	No	Not meeting inclusion criteria	Title and abstract screening
1612	Duan Z, Levine H, Romm KF, Bar-Zeev Y, Abroms LC, Griffith L, et al. IQOS Marketing Strategies and Expenditures in the United States From Market Entrance in 2019 to Withdrawal in 2021. Nicotine Tob Res. 2023;25: 1798–1803. doi:10.1093/ntr/ntad096	No	Not meeting inclusion criteria	Title and abstract screening
1613	Duan Z, Romm KF, Henriksen L, Schleicher NC, Johnson TO, Wagener TL, et al. The Impact of Recent Tobacco Regulations and COVID-19 Restrictions and Implications for Future E-Cigarette Retail: Perspectives from Vape and Vape-and-Smoke Shop Merchants. Int J Env Res Public Health. 2022;19. doi:10.3390/ijerph19073855	No	Not meeting inclusion criteria	Title and abstract screening
1614	Duan Z, Wysota CN, Romm KF, Levine H, Bar-Zeev Y, Choi K, et al. Correlates of Perceptions, Use, and Intention to Use Heated Tobacco Products Among US Young Adults in 2020. Nicotine Tob Res. 2022;24: 1968–1977. doi:10.1093/ntr/ntac185	No	Not meeting inclusion criteria	Title and abstract screening
1615	Duarte D.A., Chen-Sankey J.C., Dang K., Orozco L., Jewett B., Choi K. "Isn't there a bunch of side effects?": A focus group study on the beliefs about cessation treatments of non-college educated young adult smokers. J Subst Abuse Treat. 2020;112: 36–41. doi:10.1016/j.jsat.2020.01.013	No	Not meeting inclusion criteria	Title and abstract screening
1616	Dube CE, Pbert L, Nagawa CS, Simone DP, Wijesundara JG, Sadasivam RS. Adolescents Who Vape Nicotine and Their Experiences Vaping: A Qualitative Study. Subst Abuse Res Treat. 2023;17: 11782218231183934. doi:10.1177/11782218231183934	No	Not meeting inclusion criteria	Title and abstract screening
1617	Duchon M, Sousa C, Ludivine D, Lavoue V. How I do Hyperthermic Intraperitoneal Chemotherapy (HIPEC) in ovarian cancer: a 10 steps video ? 2023; 102563. doi:10.1016/j.jogoh.2023.102563	No	Not meeting inclusion criteria	Title and abstract screening
1618	Duell AK, Pankow JF, Peyton DH. Free-Base Nicotine Determination in Electronic Cigarette Liquids by 1H NMR Spectroscopy. Chem Res Toxicol. 2018;31: 431–434. doi:10.1021/acs.chemrestox.8b00097	No	Not meeting inclusion criteria	Title and abstract screening
1619	Duell AK, Pankow JF, Peyton DH. Nicotine in tobacco product aerosols: "It's deja vu all over again". Tob Control. 2020;29: 656–662. doi:10.1136/tobaccocontrol-2019-055275	No	Not meeting inclusion criteria	Title and abstract screening
1620	Dugan J., Jain N., Irons C., Sharma P., Sediqe S., Hejal R. DIFFERENTIATION SYNDROME AFTER GILTERITINIB: A TRICKY MIMICKER OF SEPSIS. Chest. 2021;160: A877. doi:10.1016/j.chest.2021.07.820	No	Not meeting inclusion criteria	Title and abstract screening
1621	Dugas EN, Sylvestre MP, O'Loughlin EK, Brunet J, Kakinami L, Constantin E, et al. Nicotine dependence and sleep quality in young adults. Addict Behav. 2017;65: 154–160. doi:10.1016/j.addbeh.2016.10.020	No	Not meeting inclusion criteria	Title and abstract screening
1622	Dugas EN, Sylvestre MP, Wellman RJ, Winickoff J, Montreuil A, O'Loughlin J. Measures of nicotine dependence enhance interpretation of number of cigarettes smoked in youth smoking surveillance. Drug Alcohol Depend. 2019;204: 107534. doi:10.1016/j.drugalcdep.2019.06.036	No	Not meeting inclusion criteria	Title and abstract screening
1623	Dugas EN, Sylvestre MP, Wellman RJ, O'Loughlin J. Does use of other tobacco products change when cigarette smoking status changes: A descriptive study of young adults. Addict Behav. 2020;107: 106404. doi:10.1016/j.addbeh.2020.106404	No	Not meeting inclusion criteria	Title and abstract screening
1624	Dugas EN, Wellman RJ, Sylvestre MP, Bélanger RE, O'Loughlin J. Who mixes tobacco with cannabis and does mixing relate to nicotine dependence? Addict Behav. 2022;128: 107254. doi:10.1016/j.addbeh.2022.107254	No	Not meeting inclusion criteria	Title and abstract screening
1625	Duggan J.P., Peters A.S., Trachiotis G.D., Antevil J.L. Epidemiology of Coronary Artery Disease. Surg Clin North Am. 2022;102: 499–516. doi:10.1016/j.suc.2022.01.007	No	Not meeting inclusion criteria	Title and abstract screening
1626	Dumas TM, Ellis WE, Van Hedger S, Litt DM, MacDonald M. Lockdown, bottoms up? Changes in adolescent substance use across the COVID-19 pandemic. Addict Behav. 2022;131: 107326. doi:10.1016/j.addbeh.2022.107326	No	Not meeting inclusion criteria	Title and abstract screening
1627	Dunbar MS, Shiffman S, Chandra S. Exposure to workplace smoking bans and continuity of daily smoking patterns on workdays and weekends. Addict Behav. 2018;80: 53–58. doi:10.1016/j.addbeh.2018.01.006	No	Not meeting inclusion criteria	Title and abstract screening
1628	Dunn DS, Johnson AL, Sterling KL, Cohn AM. Differences in reasons for little cigar/cigarillo use across white and black/African American young adult users. Addict Behav. 2021;118: 106884. doi:10.1016/j.addbeh.2021.106884	No	Not meeting inclusion criteria	Title and abstract screening
1629	Duong M, Rangarajan S, Zhang X, Killian K, Mony P, Swaminathan S, et al. Effects of bidi smoking on all-cause mortality and cardiorespiratory outcomes in men from south Asia: an observational community-based substudy of the Prospective Urban Rural Epidemiology Study (PURE). Lancet Glob Health. 2017;5: e168–e176. doi:10.1016/S2214-109X(17)30004-9	No	Not meeting inclusion criteria	Title and abstract screening
1630	Durieux B, Catzen H, Gray T, Tulsky J, Sanders J. "I Think that She Would Have Wanted...": Caregiver Reflections on Goal-Concordant Care for Cancer Patients at the End of Life. J Pain Symptom Manage. 2022;63: 871–872. doi:10.1016/j.jpainsymman.2022.02.064	No	Not meeting inclusion criteria	Title and abstract screening
1631	Durkin SJ, Brennan E, Wakefield MA. Optimising tobacco control campaigns within a changing media landscape and among priority populations. Tob Control. 2022;31: 284–290. doi:10.1136/tobaccocontrol-2021-056558	No	Not meeting inclusion criteria	Title and abstract screening
1632	Durrani K, El Din S-MA, Sun Y, Rule AM, Bressler J. Ethyl maltol enhances copper mediated cytotoxicity in lung epithelial cells. Toxicol Appl Pharmacol. 2021;410: 115354. doi:10.1016/j.taap.2020.115354	No	Not meeting inclusion criteria	Title and abstract screening
1633	Duru Çetinkaya P, Turan A, Deniz PP. Effectiveness of Behavioral and Pharmacological Smoking Cessation Treatment in Patients with Failed Attempt at Quitting with E-cigarettes. Turk Thorac J. 2020;21: 404–408. doi:10.5152/TurkThoracJ.2020.20181	No	Not meeting inclusion criteria	Title and abstract screening
1634	Dusautoir R., Zarcone G., Platel A., Kervoaze G., Laine W., Kluza J., et al. P02-15 Long-term respiratory impact of electronic cigarette. Toxicol Lett. 2021;350: S78. doi:10.1016/S0378-4274%2821%2900429-X	No	Not meeting inclusion criteria	Title and abstract screening

1635	Duszynski TJ, Fadel W, Wools-Kaloustian KK, Dixon BE, Yiannoutsos C, Halverson PK, et al. Association of Health Status and Nicotine Consumption with SARS-CoV-2 positivity rates. BMC Public Health. 2021;21: 1786. doi:10.1186/s12889-021-11867-6	No	Not meeting inclusion criteria	Title and abstract screening
1636	Dvorsky MR, Langberg JM. Cigarette and e-cigarette use and social perceptions over the transition to college: The role of ADHD symptoms. Psychol Addict Behav. 2019;33: 318–330. doi:10.1037/adb0000450	No	Not meeting inclusion criteria	Title and abstract screening
1637	Dyson J, Skinner J, Crick J, Crooks MG. Designing an intervention to help the quitters quit: A qualitative, intervention co-design study. PEC Innov. 2023;2: 100141. doi:10.1016/j.pecinn.2023.100141	No	Not meeting inclusion criteria	Title and abstract screening
1638	E Culbreth R, J Brandenberger K, Battey-Muse CM, Gardenhire DS. 2021 Year in Review: E-Cigarettes, Hookah Use, and Vaping Lung Injuries During the COVID-19 Pandemic. Respir Care. 2022;67: 709–714. doi:10.4187/respcare.09919	No	Not meeting inclusion criteria	Title and abstract screening
1639	Eapen MS, Lu W, Hackett TL, Singhera GK, Thompson IE, McAlinden KD, et al. Dysregulation of endocytic machinery and ACE2 in small airways of smokers and COPD patients can augment their susceptibility to SARS-CoV-2 (COVID-19) infections. Am J Physiol Lung Cell Mol Physiol. 2021;320: L158–L163. doi:10.1152/ajplung.00437.2020	No	Not meeting inclusion criteria	Title and abstract screening
1640	East K, Reid JL, Burkhalter R, Wackowski OA, Thrasher JF, Tattan-Birch H, et al. Exposure to Negative News Stories About Vaping, and Harm Perceptions of Vaping, Among Youth in England, Canada, and the United States Before and After the Outbreak of E-cigarette or Vaping-Associated Lung Injury ('EVALI'). Nicotine Tob Res. 2022;24: 1386–1395. doi:10.1093/ntr/ntac088	No	Not meeting inclusion criteria	Title and abstract screening
1641	East KA, Miller CR, Hitchman SC, McNeill A, Tompkins CNE. "It's not what you'd term normal smoking": a qualitative exploration of language used to describe heated tobacco product use and associated user identity. Addiction. 2022. doi:10.1111/add.16051	No	Not meeting inclusion criteria	Title and abstract screening
1642	East KA, Miller CR, Hitchman SC, McNeill A, Tompkins CNE. "It's not what you'd term normal smoking": a qualitative exploration of language used to describe heated tobacco product use and associated user identity. Addiction. 2023;118: 533–538. doi:10.1111/add.16051	No	Not meeting inclusion criteria	Title and abstract screening
1643	East KA, Reid JL, Burkhalter R, Kock L, Hyland A, Fong GT, et al. Evaluating the Outcomes of the Menthol Cigarette Ban in England by Comparing Menthol Cigarette Smoking Among Youth in England, Canada, and the US, 2018-2020. JAMA Netw Open. 2022;5: e2210029. doi:10.1001/jamanetworkopen.2022.10029	No	Not meeting inclusion criteria	Title and abstract screening
1644	Ebrahimi Kalan M, Bahelah R, Bursac Z, Ben Taleb Z, DiFranza JR, Tleis M, et al. Predictors of nicotine dependence among adolescent waterpipe and cigarette smokers: A 6-year longitudinal analysis. Drug Alcohol Depend. 2020;217: 108346. doi:10.1016/j.drugalcdep.2020.108346	No	Not meeting inclusion criteria	Title and abstract screening
1645	Ebrahimi Kalan M, Brewer NT. Longitudinal transitions in e-cigarette and cigarette use among US adults: prospective cohort study. Lancet Reg Health Am. 2023;22: 100508. doi:10.1016/j.lana.2023.100508	No	Not meeting inclusion criteria	Title and abstract screening
1646	Ebrahimi Kalan M, Bursac Z, Jebai R, Zare S, Li W, Gautam P, et al. State-specific Prevalence and Factors Associated With Current Marijuana, ENDS, and Cigarette use Among US Adults With Asthma. Tob Use Insights. 2022;15: 1179173X221105783. doi:10.1177/1179173X221105783	No	Not meeting inclusion criteria	Title and abstract screening
1647	Ebrahimi Kalan M, Jebai R, Li W, Gautam P, Alemohammad SY, Mortazavizadeh Z, et al. COVID-19 and tobacco products use among US adults, 2021 National Health Interview Survey. Health Sci Rep. 2023;6: e1542. doi:10.1002/hsr2.1542	No	Not meeting inclusion criteria	Title and abstract screening
1648	Ebrahimi Kalan M, Lazard AJ, Sheldon JM, Whitesell C, Hall MG, Ribisl KM, et al. Terms tobacco users employ to describe e-cigarette aerosol. Tob Control. 2022. doi:10.1136/tobaccocontrol-2021-057233	No	Not meeting inclusion criteria	Title and abstract screening
1649	Ebrahimi Kalan M, Lazard AJ, Sheldon JM, Whitesell C, Hall MG, Ribisl KM, et al. Terms tobacco users employ to describe e-cigarette aerosol. Tob Control. 2023;33: 15–20. doi:10.1136/tobaccocontrol-2021-057233	No	Not meeting inclusion criteria	Title and abstract screening
1650	Ebrahimi Kalan M, Ward KD, Ben Taleb Z. Can We Measure Nicotine Dependence in Dual Users of Cigarettes and ENDS? Nicotine Tob Res. 2021;23: 872–873. doi:10.1093/ntr/ntaa263	No	Not meeting inclusion criteria	Title and abstract screening
1651	Ebrahimi Kalan M., Behaleh R., DiFranza J.R., Bursac Z., Ben Taleb Z., Tleis M., et al. Natural Course of Nicotine Dependence Among Adolescent Waterpipe and Cigarette Smokers. J Adolesc Health. 2020;67: 859–867. doi:10.1016/j.jadohealth.2020.04.030	No	Not meeting inclusion criteria	Title and abstract screening
1652	Ebrahimi Kalan M., Bursac Z., Behaleh R., Jebai R., Osibogun O., Gautam P., et al. Nicotine-naive adolescents who live with tobacco products users, 2018 Florida Youth Tobacco Survey. J Addict Dis. 2020;39: 265–269. doi:10.1080/10550887.2020.1856299	No	Not meeting inclusion criteria	Title and abstract screening
1653	Ebrahimi Kalan M, Jebai R, Li W, Gautam P, Osibogun O, Alqahtani MM, et al. High on Hookah: Smoking Marijuana from a Hookah among Adults in the United States, Population Assessment of Tobacco and Health Study, 2015-2019. Subst Use Misuse. 2023;58: 657–665. doi:10.1080/10826084.2023.2177966	No	Not meeting inclusion criteria	Title and abstract screening
1654	Ebrahimi Kalan M, McKelvey K, Ibrahimou B, Trucco EM, Ben Taleb Z. The Road to Vaping: E-cigarette Susceptibility and Curiosity Among U.S. Adolescents Susceptible and Nonsusceptible to Cigarette Smoking. Health Educ Behav Off Publ Soc Public Health Educ. 2020;47: 696–705. doi:10.1177/1090198120943166	No	Not meeting inclusion criteria	Title and abstract screening
1655	Echevarria C, Sinha IP. Heterogeneity in the measurement and reporting of outcomes in studies of electronic cigarette use in adolescents: a systematic analysis of observational studies. Tob Control. 2017;26: 247–253. doi:10.1136/tobaccocontrol-2015-052881	No	Not meeting inclusion criteria	Title and abstract screening
1656	Echeveste Sanchez M, Quadir SG, Whindleton CM, Hoffman JL, Faccidomo SP, Guhr Lee TN, et al. The effects of electronic nicotine vapor on voluntary alcohol consumption in female and male C57BL/6 J mice. Drug Alcohol Depend. 2022;241: 109676. doi:10.1016/j.drugalcdep.2022.109676	No	Not meeting inclusion criteria	Title and abstract screening
1657	Economidou EC, Soteriades ES. Adolescent tobacco use in the current era of multiple products for smoking. J Adolesc Health. 2020;66: 258–259. doi:10.1016/j.jadohealth.2019.10.017https://dx.doi.org/10.1016/j.jadohealth.2019.10.017	No	Not meeting inclusion criteria	Title and abstract screening
1658	Editorial Office. Retraction Notice to "Cancer Prevalence in E-Cigarette Users: A Retrospective Cross-Sectional NHANES Study". World J Oncol. 2022;13: 417. doi:10.14740/wjon1438r	No	Not meeting inclusion criteria	Title and abstract screening
1659	Edwards KC, Kasza KA, Tang Z, Stanton CA, Sharma E, Halenar MJ, et al. Correlates of tobacco product reuptake and relapse among youth and adults in the USA: findings from the PATH Study Waves 1-3 (2013-2016). Tob Control. 2020;29: s216–s226. doi:10.1136/tobaccocontrol-2020-055660	No	Not meeting inclusion criteria	Title and abstract screening
1660	Effah F., Taiwo B., Baines D., Bailey A., Marczylo T. Pulmonary effects of e-liquid flavors: a systematic review. J Toxicol Environ Health - Part B Crit Rev. 2022;25: 343–371. doi:10.1080/10937404.2022.2124563	No	Not meeting inclusion criteria	Title and abstract screening
1661	Egger S, Watts C, Dessaix A, Brooks A, Jenkinson E, Grogan P, et al. Parent's awareness of, and influence on, their 14-17-year-old child's vaping and smoking behaviours; an analysis of 3242 parent-child pairs in Australia. Addict Behav. 2024;150: 107931. doi:10.1016/j.addbeh.2023.107931	No	Not meeting inclusion criteria	Title and abstract screening
1662	Ehlke SJ, Ganz O, Kendzor DE, Cohn AM. Differences between adult sexual minority females and heterosexual females on menthol smoking and other smoking behaviors: Findings from Wave 4 (2016-2018) of the population assessment of tobacco and health study. Addict Behav. 2022;129: 107265. doi:10.1016/j.addbeh.2022.107265	No	Not meeting inclusion criteria	Title and abstract screening
1663	Ehlke SJ, Kendzor DE, Smith MA, Sifat MS, Boozary LK, Cohn AM. Single-use, co-use, and polysubstance use of alcohol, tobacco, and cannabis in sexual minority and heterosexual females. Am J Addict. 2023;32: 66–75. doi:10.1111/ajad.13344	No	Not meeting inclusion criteria	Title and abstract screening

1664	Eidelman AI. Smoking, Vaping, While Breastfeeding in the Era of COVID-19. Breastfeed Med. 2021;16: 765. doi:10.1089/bfm.2021.29192.aie	No	Not meeting inclusion criteria	Title and abstract screening
1665	Eisenberg MJ, Hébert-Losier A, Filion KB. Use of e-Cigarettes for Smoking Cessation-Reply. JAMA. 2021;325: 1006–1007. doi:10.1001/jama.2020.27210	No	Not meeting inclusion criteria	Title and abstract screening
1666	Eisenberg MJ, Hébert-Losier A, Windle SB, Greenspoon T, Brandys T, Fülöp T, et al. Effect of e-Cigarettes Plus Counseling vs Counseling Alone on Smoking Cessation: A Randomized Clinical Trial. JAMA. 2020;324: 1844–1854. doi:10.1001/jama.2020.18889	No	Not meeting inclusion criteria	Title and abstract screening
1667	Eisenhauer EA, Schwartz R, Cunningham R, Hagen L, Fong GT, Callard C, et al. Perspective on Cancer Control: Whither the Tobacco Endgame for Canada? Curr Oncol. 2022;29: 2081–2090. doi:10.3390/curroncol29030168	No	Not meeting inclusion criteria	Title and abstract screening
1668	El Shahawy O., Loney T., Shah T., Sherman S.E., Blaha M.J. Response to Letter Regarding the Article “Association of E-Cigarettes With Erectile Dysfunction: The Population Assessment of Tobacco and Health Study.” Am J Prev Med. 2022;63: e105–e106. doi:10.1016/j.amepre.2022.03.031	No	Not meeting inclusion criteria	Title and abstract screening
1669	El-Awa F., Tageldin M.A., Prasad V., Al-Mulla A., Heydari G., Alebshehy R. Lung health in the Eastern Mediterranean Region: The need to end designated smoking areas in public places. East Mediterr Health J. 2019;25: 297–298. doi:10.26719/2019.25.5.297	No	Not meeting inclusion criteria	Title and abstract screening
1670	El-Hellani A, Hanna E, Sharma M, Blohowiak R, Joseph P, Eid T, et al. Nicotine flux as a powerful tool for regulating nicotine delivery from e-cigarettes: Protocol of two complimentary randomized crossover clinical trials. PLoS One. 2023;18: e0291786. doi:10.1371/journal.pone.0291786	No	Not meeting inclusion criteria	Title and abstract screening
1671	El-Khoury F, El Aarbaoui T, Héron M, Hejblum G, Métadieu B, Le Faou AL, et al. Smoking cessation using preference-based tools among socially disadvantaged smokers: study protocol for a pragmatic, multicentre randomised controlled trial. BMJ Open. 2021;11: e048859. doi:10.1136/bmjopen-2021-048859	No	Not meeting inclusion criteria	Title and abstract screening
1672	El-Mahdy MA, Mahgoup EM, Ewees MG, Eid MS, Abdelghany TM, Zweier JL. Long-term electronic cigarette exposure induces cardiovascular dysfunction similar to tobacco cigarettes: role of nicotine and exposure duration. Am J Physiol Heart Circ Physiol. 2021;320: H2112–H2129. doi:10.1152/ajpheart.00997.2020	No	Not meeting inclusion criteria	Title and abstract screening
1673	Elena Cavarretta E, Sciarretta S, Nocella C, Peruzzi M, Marullo A, Loffredo L, et al. Subjective smoking satisfaction between heat-not-burn, electronic vaping, and traditional tobacco combustion cigarettes: a sub-analysis of the SUR-VAPES 2 trial. 2019;26: S114-. doi:10.1177/2047487319860053	No	Not meeting inclusion criteria	Title and abstract screening
1674	Elhadi M, Khaled A, Msherghi A. Infectious diseases as a cause of death among cancer patients: a trend analysis and population-based study of outcome in the United States based on the Surveillance, Epidemiology, and End Results database. Infect Agent Cancer. 2021;16: 1–11. doi:10.1186/s13027-021-00413-z	No	Not meeting inclusion criteria	Title and abstract screening
1675	Elharrar X, Fortin M, Beguinot E, Dols AM, Greillier L, Martinet Y. [Prohibition of tobacco sales to minors in France and Quebec. Comparison of legislative frameworks, their enforcement, and underage smoking]. Rev Epidemiol Sante Publique. 2019;67: 181–187. doi:10.1016/j.respe.2019.02.001	No	Not meeting inclusion criteria	Title and abstract screening
1676	Elin Smith K. Prevalence and Correlates of Electronic Cigarette Use Among a Clinical Sample of Polysubstance Users in Kentucky: Long Live the Cigarette? Subst Use Misuse. 2019;54: 225–235. doi:10.1080/10826084.2018.1512629	No	Not meeting inclusion criteria	Title and abstract screening
1677	Elmahdi FM, Aljohani RS, Alharbi NA, Yousef SE, Alharbi NM, Afasha RB, et al. A Cytological Study of Oral Human Papillomavirus (HPV) Infection Among Electronic Cigarette Smokers in Al-Madinah Al-Munawara. Cureus. 2023;15: e40421. doi:10.7759/cureus.40421	No	Not meeting inclusion criteria	Title and abstract screening
1678	Elmahdy M., Mahgoup E., Ewees M., Eid M., Abdelghany T., Zweier J. Role of Nicotine and Exposure Duration in the Cardiovascular Toxicity of Electronic Cigarettes and Tobacco Cigarettes in a Long-term Mouse Exposure Model. FASEB J. 2021;35. doi:10.1096/fasebj.2021.35.S1.04618	No	Not meeting inclusion criteria	Title and abstract screening
1679	Elmallah R.D.K., Sbravati N., Hendrix S.T., Greggory Tharp J., Bergin P. What is the effect of smoking on postoperative outcomes after ankle-fracture surgery? A retrospective cohort study. Curr Orthop Pract. 2021;32: 130–133. doi:10.1097/BCO.0000000000000966	No	Not meeting inclusion criteria	Title and abstract screening
1680	Elser H, Vijayaraghavan M, Kasner SE. E-Cigarettes and Stroke Risk-Present Uncertainties and Future Directions. JAMA Neurol. 2023;80: 883–884. doi:10.1001/jamaneurol.2023.2050	No	Not meeting inclusion criteria	Title and abstract screening
1681	Elton-Marshall T, Driezen P, Fong GT, Cummings KM, Persoskie A, Wackowski O, et al. Adult perceptions of the relative harm of tobacco products and subsequent tobacco product use: Longitudinal findings from waves 1 and 2 of the population assessment of tobacco and health (PATH) study. Addict Behav. 2020;106: 106337. doi:10.1016/j.addbeh.2020.106337	No	Not meeting inclusion criteria	Title and abstract screening
1682	Eltorai AE, Choi AR, Eltorai AS. Impact of Electronic Cigarettes on Various Organ Systems. Respir Care. 2019;64: 328–336. doi:10.4187/respcare.06300	No	Not meeting inclusion criteria	Title and abstract screening
1683	Emma R, Caruso M, Campagna D, Pulvirenti R, Li Volti G. The Impact of Tobacco Cigarettes, Vaping Products and Tobacco Heating Products on Oxidative Stress. Antioxid Basel. 2022;11. doi:10.3390/antiox11091829	No	Not meeting inclusion criteria	Title and abstract screening
1684	Emma R, Fuochi V, Distefano A, Partsinevelos K, Rust S, Zadjali F, et al. Cytotoxicity, mutagenicity and genotoxicity of electronic cigarettes emission aerosols compared to cigarette smoke: the REPLICA project. Sci Rep. 2023;13: 17859. doi:10.1038/s41598-023-44626-1	No	Not meeting inclusion criteria	Title and abstract screening
1685	Emma R, Polosa R, Caruso M. High nicotine exposure in rodents is unlikely to inform about its toxicity in humans. Eur Respir J. 2019;54. doi:10.1183/13993003.01073-2018	No	Not meeting inclusion criteria	Title and abstract screening
1686	Emoto S, Fukunaga Y, Nakanishi R, Hirayama K, Nagaoka T, Matsui S, et al. Short- and long-term outcomes of laparoscopic surgery with extracorporeal anastomosis for transverse colon cancer: comparison of triangulating anastomosis with functional end-to-end anastomosis. Surg Endosc. 2022;36: 3261–3269. doi:10.1007/s00464-021-08638-0	No	Not meeting inclusion criteria	Title and abstract screening
1687	Englund L. The afterlives of death: gratitude and gratefulness in end-of-life writing by Jenny Diski and Oliver Sacks. Mortality. 2023;28: 529–542. doi:10.1080/13576275.2022.2031942	No	Not meeting inclusion criteria	Title and abstract screening
1688	Eniola K. E-Cigarette Use Among Adolescents, a Gateway to Nicotine Addiction. J Adolesc Health Off Publ Soc Adolesc Med. 2023;73: 602. doi:10.1016/j.jadohealth.2023.04.021	No	Not meeting inclusion criteria	Title and abstract screening
1689	Enlow PT, Felicione N, Williford DN, Durkin K, Blank MD, Duncan CL. Validation of the Electronic Cigarette Expectancy Scale for Adolescents. Subst Use Misuse. 2022;57: 212–221. doi:10.1080/10826084.2021.2002898	No	Not meeting inclusion criteria	Title and abstract screening
1690	Ensor C., Thatcher S.E., Alsiraj Y.A., Liang J., Cassis L.A. Nicotine infusion augments angiotensin iiinduced abdominal aortic aneurysms in female and male hypercholesterolemic mice. Arterioscler Thromb Vasc Biol. 2020;40. doi:10.1161/atvb.40.suppl-1.443	No	Not meeting inclusion criteria	Title and abstract screening
1691	Entwistle MR, Valle K, Schweizer D, Cisneros R. Electronic cigarette (e-cigarette) use and frequency of asthma symptoms in adult asthmatics in California. J Asthma. 2021;58: 1460–1466. doi:10.1080/02770903.2020.1805751	No	Not meeting inclusion criteria	Title and abstract screening
1692	Enyioha C, Boynton MH, Ranney LM, Byron MJ, Goldstein AO, Kistler CE. Preferences for different features of ENDS products by tobacco product use: a latent class analysis. Subst Abuse Treat Prev Policy. 2022;17: 18. doi:10.1186/s13011-022-00448-4	No	Not meeting inclusion criteria	Title and abstract screening

1693	Enzinger AC, Ghosh K, Keating NL, Cutler DM, Landrum MB, Wright AA. US Trends in Opioid Access Among Patients With Poor Prognosis Cancer Near the End-of-Life. J Clin Oncol. 2021;39: 2948–2958. doi:10.1200/JCO.21.00476	No	Not meeting inclusion criteria	Title and abstract screening
1694	Enzinger AC, Ghosh K, Keating NL, Cutler DM, Clark CR, Florez N, et al. Racial and Ethnic Disparities in Opioid Access and Urine Drug Screening Among Older Patients With Poor-Prognosis Cancer Near the End of Life. J Clin Oncol. 2023;41: 2511–2522. doi:10.1200/JCO.22.01413	No	Not meeting inclusion criteria	Title and abstract screening
1695	Epperson A.E., Wong S., Lambin E.F., Henriksen L., Baiocchi M., Flora J.A., et al. Adolescents' Health Perceptions of Natural American Spirit's On-the-Pack Eco-Friendly Campaign. J Adolesc Health. 2021;68: 604–611. doi:10.1016/j.jadohealth.2020.06.033	No	Not meeting inclusion criteria	Title and abstract screening
1696	Epstein AS, Riley M, Nelson JE, Bernal C, Martin S, Xiao H. Goals of care documentation by medical oncologists and oncology patient end-of-life care outcomes. Cancer 0008543X. 2022;128: 3400–3407. doi:10.1002/cncr.34400	No	Not meeting inclusion criteria	Title and abstract screening
1697	Erhabor J, Boakye E, Obisesan O, Osei AD, Tasdighi E, Mirbolouk H, et al. E-Cigarette Use Among US Adults in the 2021 Behavioral Risk Factor Surveillance System Survey. JAMA Netw Open. 2023;6: e2340859. doi:10.1001/jamanetworkopen.2023.40859	No	Not meeting inclusion criteria	Title and abstract screening
1698	Erinoso O, Oyapero A, Amure M, Osoba M, Osibogun O, Wright K, et al. Electronic cigarette use among adolescents and young adults in Nigeria: Prevalence, associated factors and patterns of use. PLoS One. 2021;16: e0258850. doi:10.1371/journal.pone.0258850	No	Not meeting inclusion criteria	Title and abstract screening
1699	Erinoso O, Oyapero A, Osoba M, Amure M, Osibogun O, Wright K, et al. Association between anxiety, alcohol, poly-tobacco use and waterpipe smoking: A cross-sectional study in Lagos, Nigeria. Niger Postgrad Med J. 2021;28: 117–125. doi:10.4103/npmj.npmj_530_21	No	Not meeting inclusion criteria	Title and abstract screening
1700	Erku DA, Bauld L, Dawkins L, Gartner CE, Steadman KJ, Noar SM, et al. Does the content and source credibility of health and risk messages related to nicotine vaping products have an impact on harm perception and behavioural intentions? A systematic review. Addict Abingdon Engl. 2021;116: 3290–3303. doi:10.1111/add.15473	No	Not meeting inclusion criteria	Title and abstract screening
1701	Erly BK, Prochazka AV. E-cigarettes were more effective than nicotine replacement for smoking cessation at 1 year. Ann Intern Med. 2019;170: JC50. doi:10.7326/ACPJ201905210-050	No	Not meeting inclusion criteria	Title and abstract screening
1702	Erythropel H.C., Garcia Torres D.S., Woodrow J.G., De Winter T.M., Falinski M.M., Anastas P.T., et al. Quantification of Flavorants and Nicotine in Waterpipe Tobacco and Mainstream Smoke and Comparison to E-cigarette Aerosol. Nicotine Tob Res. 2021;23: 600–604. doi:10.1093/ntr/ntaa114	No	Not meeting inclusion criteria	Title and abstract screening
1703	Escobar Y-NH, Morrison CB, Chen Y, Hickman E, Love CA, Rebuli ME, et al. Differential responses to e-cig generated aerosols from humectants and different forms of nicotine in epithelial cells from nonsmokers and smokers. Am J Physiol Lung Cell Mol Physiol. 2021;320: L1064–L1073. doi:10.1152/ajplung.00525.2020	No	Not meeting inclusion criteria	Title and abstract screening
1704	Eshraghian EA, Al-Delaimy WK. A review of constituents identified in e-cigarette liquids and aerosols. Tob Prev Cessat. 2021;7: 10. doi:10.18332/tpc/131111	No	Not meeting inclusion criteria	Title and abstract screening
1705	Espinoza-Derout J, Shao XM, Lao CJ, Hasan KM, Rivera JC, Jordan MC, et al. Electronic Cigarette Use and the Risk of Cardiovascular Diseases. Front Cardiovasc Med. 2022;9: 879726. doi:10.3389/fcvm.2022.879726	No	Not meeting inclusion criteria	Title and abstract screening
1706	Espinoza-Derout J., Shao X.M., Hasan K.M., Rivera J.-C., Lao C., Wilson J., et al. Acipimox Normalizes The Cardiac Phenotype Induced By Electronic Cigarettes. J Endocr Soc. 2022;6: A6. doi:10.1210/jendso/bvac150.011	No	Not meeting inclusion criteria	Title and abstract screening
1707	Espinoza-Derout J., Shao X.M., Molina-Mancio J., Hasan K.M., Mtume N., Sinha-Hikim A.P., et al. Electronic Cigarette Exposure Induces Pro-Inflammatory Changes in Adipose Tissue in Apolipoprotein E (APOE) Knockout Mice. J Endocr Soc. 2020;4: A350. doi:10.1210/jendso/bvaa046.691	No	Not meeting inclusion criteria	Title and abstract screening
1708	Esposito F, Squillante J, Nolasco A, Montuori P, Macrì PG, Cirillo T. Acrylamide levels in smoke from conventional cigarettes and heated tobacco products and exposure assessment in habitual smokers. Env Res. 2022;208: 112659. doi:10.1016/j.envres.2021.112659	No	Not meeting inclusion criteria	Title and abstract screening
1709	Essau CA [Ed], Delfabbro PH [Ed]. Adolescent addiction: Epidemiology, assessment, and treatment. Adolesc Addict Epidemiol Assess Treat. 2020.	No	Not meeting inclusion criteria	Title and abstract screening
1710	Esteban-Lopez M., Perry M.D., Garbinski L.D., Manevski M., Andre M., Ceyhan Y., et al. Health effects and known pathology associated with the use of E-cigarettes. Toxicol Rep. 2022;9: 1357–1368. doi:10.1016/j.toxrep.2022.06.006	No	Not meeting inclusion criteria	Title and abstract screening
1711	Estey D. The role of e-cigarette use in smoking cessation and e-cigarette dependence. Diss Abstr Int Sect B Sci Eng. 2023;84: No-Specified.	No	Not meeting inclusion criteria	Title and abstract screening
1712	Estruch R., Ruilope L.M., Cosentino F. The year in cardiovascular medicine 2020: Epidemiology and prevention. Eur Heart J. 2021;42: 813–821. doi:10.1093/eurheartj/ehaa1062	No	Not meeting inclusion criteria	Title and abstract screening
1713	Etter J-F, Vera Cruz G, Khazaal Y. Predicting smoking cessation, reduction and relapse six months after using the Stop-Tabac app for smartphones: a machine learning analysis. BMC Public Health. 2023;23: 1076. doi:10.1186/s12889-023-15859-6	No	Not meeting inclusion criteria	Title and abstract screening
1714	Etter JF. E-cigarettes and the obsolescence of combustion. Expert Rev Respir Med. 2018;12: 345–347. doi:10.1080/17476348.2018.1453809	No	Not meeting inclusion criteria	Title and abstract screening
1715	Etter JF. Gateway effects and electronic cigarettes. Addiction. 2018;113: 1776–1783. doi:10.1111/add.13924	No	Not meeting inclusion criteria	Title and abstract screening
1716	Etter JF. Are long-term vapers interested in vaping cessation support? Addiction. 2019;114: 1473–1477. doi:10.1111/add.14595	No	Not meeting inclusion criteria	Title and abstract screening
1717	Etter JF, Khazaal Y. The Stop-tabac smartphone application for smoking cessation: a randomized controlled trial. Addiction. 2022;117: 1406–1415. doi:10.1111/add.15738	No	Not meeting inclusion criteria	Title and abstract screening
1718	EUCTR2013-000361-36-GB. Cannabidiol: a novel intervention for cannabis use problems? 2013. Available: https://www.cochranelibrary.com/central/doi/10.1002/central/CN-01815800/full	No	Not meeting inclusion criteria	Title and abstract screening
1719	EUCTR2017-003188-36-FI. Keski-ikäisten pitkään tupakoineiden tupakasta vieroitus sähkötupakalla. 2017. Available: https://www.cochranelibrary.com/central/doi/10.1002/central/CN-01897880/full	No	Not meeting inclusion criteria	Title and abstract screening
1720	EUCTR2020-005641-17-PL. Clinical trial of statins CAncer preventive and Pleiotropic TherApy IN smokers with chronic obstructive pulmonary disease (COPD). 2021. Available: https://www.cochranelibrary.com/central/doi/10.1002/central/CN-02377985/full	No	Not meeting inclusion criteria	Title and abstract screening
1721	Eva TA, Barua N, Chowdhury MdM, Yeasmin S, Rakib A, Islam MR, et al. Perspectives on signaling for biological- and processed food-related advanced glycation end-products and its role in cancer progression. Crit Rev Food Sci Nutr. 2022;62: 2655–2672. doi:10.1080/10408398.2020.1856771	No	Not meeting inclusion criteria	Title and abstract screening

1722	Evans C.M., McCubbrey A.L. Can eosinophils prevent lung injury? ask PHIL. Am J Respir Cell Mol Biol. 2021;64: 523–524. doi:10.1165/rcmb.2021-0083ED	No	Not meeting inclusion criteria	Title and abstract screening
1723	Evans R.E., Herbert S., Owen W., Rao D. Case of e-cigarette or vaping product use-associated lung injury (EVALI) in London, UK. BMJ Case Rep. 2021;14: e240700. doi:10.1136/bcr-2020-240700	No	Not meeting inclusion criteria	Title and abstract screening
1724	Evans WK, Tammemägi MC, Walker MJ, Cameron E, Leung YW, Ashton S, et al. Integrating Smoking Cessation Into Low-Dose Computed Tomography Lung Cancer Screening: Results of the Ontario, Canada Pilot. J Thorac Oncol. 2023;18: 1323–1333. doi:10.1016/j.jtho.2023.07.004	No	Not meeting inclusion criteria	Title and abstract screening
1725	Evans-Polce RJ, Schuler MS, Kcomt L, McCabe VV, McCabe SE. Sexual Identity Differences in Tobacco (Re)Uptake: Testing Mediation by Internalizing and Externalizing Symptoms. Am J Prev Med. 2023;64: 824–833. doi:10.1016/j.amepre.2023.01.017	No	Not meeting inclusion criteria	Title and abstract screening
1726	Evans-Polce RJ, Veliz P, Kcomt L, Boyd CJ, McCabe SE. Nicotine and Tobacco Product Use and Dependence Symptoms Among US Adolescents and Adults: Differences by Age, Sex, and Sexual Identity. Nicotine Tob Res. 2021;23: 2065–2074. doi:10.1093/ntr/ntab127	No	Not meeting inclusion criteria	Title and abstract screening
1727	Eyal N. Rescuing Vapers Versus Rescuing Smokers: The Ethics. Nicotine Tob Res. 2021;23: 26–31. doi:10.1093/ntr/ntaa157	No	Not meeting inclusion criteria	Title and abstract screening
1728	Fadeyi O, Randhawa A, Shankar A, Garabetian C, Singh H, Topacio A. Thromboembolism Triggered by a Combination of Electronic Cigarettes and Oral Contraceptives: A Case Report and Review of Literature. J Investig Med High Impact Case Rep. 2023;11: 23247096231181072. doi:10.1177/23247096231181072	No	Not meeting inclusion criteria	Title and abstract screening
1729	Fadus M.C., Squeglia L.M., Valadez E.A., Tomko R.L., Bryant B.E., Gray K.M. Adolescent Substance Use Disorder Treatment: an Update on Evidence-Based Strategies. Curr Psychiatry Rep. 2019;21: 96. doi:10.1007/s11920-019-1086-0	No	Not meeting inclusion criteria	Title and abstract screening
1730	Fagan P., Guy M., Pokhrel P., Herzog T., Soule E., Maloney S., et al. Nicotine content in flavored electronic cigarette liquids using three unique samples. Tob Induc Dis. 2018;16: 298. doi:10.18332/tid/84629	No	Not meeting inclusion criteria	Title and abstract screening
1731	Fagan P, Pokhrel P, Herzog TA, Guy MC, Sakuma K-LK, Trinidad DR, et al. Warning Statements and Safety Practices Among Manufacturers and Distributors of Electronic Cigarette Liquids in the United States. Nicotine Tob Res Off J Soc Res Nicotine Tob. 2018;20: 970–976. doi:10.1093/ntr/ntx101	No	Not meeting inclusion criteria	Title and abstract screening
1732	Fahey MC, Krukowski RA, Anderson RT, Cohn WF, Porter KJ, Reid T, et al. Reaching adults who smoke cigarettes in rural Appalachia: Rationale, design & analysis plan for a mixed-methods study disseminating pharmacy-delivered cessation treatment. Contemp Clin Trials. 2023;134: 107335. doi:10.1016/j.cct.2023.107335	No	Not meeting inclusion criteria	Title and abstract screening
1733	Fairman RT, Cho YJ, Popova L, Cummings KM, Smith TT, Fong GT, et al. Support for nicotine reduction in cigarettes: findings from the 2016 and 2020 ITC Four Country Smoking and Vaping Surveys. Tob Control. 2023. doi:10.1136/tc-2023-058128	No	Not meeting inclusion criteria	Title and abstract screening
1734	Fairman RT, Churchill V, Garner JB, Brown D, Massey ZB, Ashley DL, et al. It's addiction at this Point": A qualitative examination of youth E-cigarette use trajectory in the United States. Tob Use Insights. 2023;16: 1179173X231185455. doi:10.1177/1179173X231185455	No	Not meeting inclusion criteria	Title and abstract screening
1735	Fait BW, Thompson DC, Mose TN, Jatlow P, Jordt SE, Picciotto MR, et al. Menthol disrupts nicotine's psychostimulant properties in an age and sex-dependent manner in C57BL/6J mice. Behav Brain Res. 2017;334: 72–77. doi:10.1016/j.bbr.2017.07.027	No	Not meeting inclusion criteria	Title and abstract screening
1736	Fallahi P., Ferrari S.M., Elia G., Ragusa F., Paparo S.R., Patrizio A., et al. Cytokines as Targets of Novel Therapies for Graves' Ophthalmopathy. Front Endocrinol. 2021;12: 654473. doi:10.3389/fendo.2021.654473	No	Not meeting inclusion criteria	Title and abstract screening
1737	Fallin-Bennett A, Lisha NE, Ling PM. Other Tobacco Product Use Among Sexual Minority Young Adult Bar Patrons. Am J Prev Med. 2017;53: 327–334. doi:10.1016/j.amepre.2017.03.006	No	Not meeting inclusion criteria	Title and abstract screening
1738	Famiglietti A, Memoli JW, Khaitan PG. Are electronic cigarettes and vaping effective tools for smoking cessation? Limited evidence on surgical outcomes: a narrative review. J Thorac Dis. 2021;13: 384–395. doi:10.21037/jtd-20-2529	No	Not meeting inclusion criteria	Title and abstract screening
1739	Fan T., Yingst J.M., Bascom R., Tuanquin L., Veldheer S., Branstetter S., et al. Feasibility of Patient Navigation-Based Smoking Cessation Program in Cancer Patients. Int J Environ Res Public Health. 2022;19: 4034. doi:10.3390/ijerph19074034	No	Not meeting inclusion criteria	Title and abstract screening
1740	Fangfang Liang, Fuchao Ma, Jincai Zhong, Liang F, Ma F, Zhong J. Prognostic factors of patients after liver cancer surgery: Based on Surveillance, Epidemiology, and End Results database. Medicine (Baltimore). 2021;100: 1–6. doi:10.1097/MD.0000000000026694	No	Not meeting inclusion criteria	Title and abstract screening
1741	Farber H.J. New understanding of the health hazards of electronic cigarettes and vaping. Pediatr Rev. 2020;41: 152–154. doi:10.1542/pir.2019-0269	No	Not meeting inclusion criteria	Title and abstract screening
1742	Farber H.J. The Most Important Learnings from the new Official American Thoracic Society (ATS) Clinical Practice Guidelines: Initiating Pharmacologic Treatment in Tobacco Dependent Adults. Arch Bronconeumol. 2021;57: 737–738. doi:10.1016/j.arbr.2021.04.018	No	Not meeting inclusion criteria	Title and abstract screening
1743	Farber H.J., Folan P. The tobacco industry targets youth. Am J Respir Crit Care Med. 2017;196: 11–12.	No	Not meeting inclusion criteria	Title and abstract screening
1744	Farber H.J., Neptune E., Ewart G. U.S. Food and drug administration regulation of tobacco products time for a course correction. Ann Am Thorac Soc. 2019;16: 44–48. doi:10.1513/AnnalsATS.201809-597PS	No	Not meeting inclusion criteria	Title and abstract screening
1745	Farber H.J., Neptune E.R., Ewart G.W. Corrective statements from the tobacco industry: More evidence for why we need effective tobacco control. Ann Am Thorac Soc. 2018;15: 127–130. doi:10.1513/AnnalsATS.201711-845GH	No	Not meeting inclusion criteria	Title and abstract screening
1746	Farber HJ, Conrado Pacheco Gallego M, Galiatsatos P, Folan P, Lamphere T, Pakhale S. Harms of Electronic Cigarettes: What the Healthcare Provider Needs to Know. Ann Am Thorac Soc. 2021;18: 567–572. doi:10.1513/AnnalsATS.202009-1113CME	No	Not meeting inclusion criteria	Title and abstract screening
1747	Farkas A., Tomisa G., Kis E., Horvath A. Health effects of cigarettes, electronic cigarettes and waterpipes. Orv Hetil. 2021;162. doi:10.1556/650.2021.32079	No	Not meeting inclusion criteria	Title and abstract screening
1748	Farkas G, Kocsis ZS, Székely G, Dobozi M, Kenessey I, Polgár C, et al. Smoking, chromosomal aberrations, and cancer incidence in healthy subjects. Mutat Res Genet Toxicol Env Mutagen. 2021;867: 503373. doi:10.1016/j.mrgentox.2021.503373	No	Not meeting inclusion criteria	Title and abstract screening
1749	Farooqui M, Shoaib S, Afaq H, Quadri S, Zaina F, Baig A, et al. Bidirectionality of Smoking and Depression in Adolescents: a Systemic Review. Trends Psychiatry Psychother. 2022. doi:10.47626/2237-6089-2021-0429	No	Not meeting inclusion criteria	Title and abstract screening
1750	Farrimond H. A typology of vaping: Identifying differing beliefs, motivations for use, identity and political interest amongst e-cigarette users. Int J Drug Policy. 2017;48: 81–90. doi:10.1016/j.drugpo.2017.07.011	No	Not meeting inclusion criteria	Title and abstract screening

1751	Farrimond H, Abraham C. Developing E-cigarette friendly smoking cessation services in England: staff perspectives. Harm Reduct J. 2018;15: 38. doi:10.1186/s12954-018-0244-8	No	Not meeting inclusion criteria	Title and abstract screening
1752	Farsalinos K.E., Yannovits N., Sarri T., Voudris V., Poulas K. Nicotine delivery to the aerosol of a heat-not-burn tobacco product: Comparison with a tobacco cigarette and e-cigarettes. Nicotine Tob Res. 2018;20: 1004–1009. doi:10.1093/ntr/ntx138	No	Not meeting inclusion criteria	Title and abstract screening
1753	Farsalinos KE, Niaura R. E-cigarettes and Smoking Cessation in the United States According to Frequency of E-cigarette Use and Quitting Duration: Analysis of the 2016 and 2017 National Health Interview Surveys. Nicotine Tob Res. 2020;22: 655–662. doi:10.1093/ntr/ntz025	No	Not meeting inclusion criteria	Title and abstract screening
1754	Farsalinos KE, Poulas K, Voudris V, Le Houezec J. E-cigarette use in the European Union: millions of smokers claim e-cigarettes helped them quit...Maziak W., Ben Taleb Z. Eurobarometer survey and e-cigarettes: unsubstantiated claims. Addiction 2017; 112 : 545. Addiction. 2017;112: 545–546. doi:10.1111/add.13722	No	Not meeting inclusion criteria	Title and abstract screening
1755	Farshbaf M, Mojarad-Jabali S, Hemmati S, Khosroushahi AY, Motasadizadeh H, Zarebkohan A, et al. Enhanced BBB and BBTB penetration and improved anti-glioma behavior of Bortezomib through dual-targeting nanostructured lipid carriers. J Control Release. 2022;345: 371–384. doi:10.1016/j.jconrel.2022.03.019	No	Not meeting inclusion criteria	Title and abstract screening
1756	Faryad S., Ali M., Meeker J. Acute Eosinophilic Pneumonia Caused by Cigar Smoking. Am J Respir Crit Care Med. 2022;205. doi:10.1164/ajrccm-conference.2022.205.1_MeetingAbstracts.A1127	No	Not meeting inclusion criteria	Title and abstract screening
1757	Farzal Z., Jaspers I., Rebuli M., Clapp P. The science behind Vaping: Empowering patients to quit. Otolaryngol Head Neck Surg. 2021;165: P51–P52. doi:10.1177/01945998211030910g	No	Not meeting inclusion criteria	Title and abstract screening
1758	Farzal Z., Perry M.F., Yarbrough W.G., Kimple A.J. The Adolescent Vaping Epidemic in the United States - How It Happened and Where We Go from Here. JAMA Otolaryngol - Head Neck Surg. 2019;145: 885–886. doi:10.1001/jamaoto.2019.2410	No	Not meeting inclusion criteria	Title and abstract screening
1759	Faucheux A, Bloomer C, Quattlebaum AM, Khoury L, Olson E, Omlor R, et al. Barriers to Palliative Care in the Immunotherapy Era: End-of-Life Outcomes Among a Retrospective Cohort of Metastatic Lung Cancer Patients (Sch414). J Pain Symptom Manage. 2023;65: e586–e586. doi:10.1016/j.jpainsymman.2023.02.151	No	Not meeting inclusion criteria	Title and abstract screening
1760	Faulcon LM, Rudy S, Limpert J, Wang B, Murphy I. Adverse Experience Reports of Seizures in Youth and Young Adult Electronic Nicotine Delivery Systems Users. J Adolesc Health. 2020;66: 15–17. doi:10.1016/j.jadohealth.2019.10.002	No	Not meeting inclusion criteria	Title and abstract screening
1761	Fayon M. Cigarette Smoke Exposure and Lung Health. Pediatr Pulmonol. 2022;57: S36–S38. doi:10.1002/ppul.25961	No	Not meeting inclusion criteria	Title and abstract screening
1762	Fearon IM. Human abuse liability assessment of e-cigarettes: Why, what and how? Drug Test Anal. 2022. doi:10.1002/dta.3251	No	Not meeting inclusion criteria	Title and abstract screening
1763	Fearon IM. Human abuse liability assessment of e-cigarettes: Why, what and how? Drug Test Anal. 2023;15: 1211–1221. doi:10.1002/dta.3251	No	Not meeting inclusion criteria	Title and abstract screening
1764	Feeney S, Rossetti V, Terrien J. E-Cigarettes-a review of the evidence-harm versus harm reduction. Tob Use Insights. 2022;15: 1179173X221087524. doi:10.1177/1179173X221087524	No	Not meeting inclusion criteria	Title and abstract screening
1765	Feldman R, Stanton M, Suelzer EM. Compiling Evidence for EVALI: A Scoping Review of In Vivo Pulmonary Effects After Inhaling Vitamin E or Vitamin E Acetate. J Med Toxicol. 2021;17: 278–288. doi:10.1007/s13181-021-00823-w	No	Not meeting inclusion criteria	Title and abstract screening
1766	Felicione NJ. Short-term abstinence effects in experienced electronic cigarette users. Diss Abstr Int Sect B Sci Eng. 2020;81: No-Specified.	No	Not meeting inclusion criteria	Title and abstract screening
1767	Felicione NJ, Cummings KM, Gravely S, Hammond D, McNeill A, Borland R, et al. "Don't Know" Responses for Nicotine Vaping Product Features among Adult Vapers: Findings from the 2018 and 2020 ITC Four Country Smoking and Vaping Surveys. Int J Env Res Public Health. 2021;18. doi:10.3390/ijerph18157928	No	Not meeting inclusion criteria	Title and abstract screening
1768	Felicione NJ, Fix BV, McNeill A, Cummings KM, Goniewicz ML, Hammond D, et al. Characteristics and changes over time of nicotine vaping products used by vapers in the 2016 and 2018 ITC Four Country Smoking and Vaping Surveys. Tob Control. 2022;31: e66–e73. doi:10.1136/tobaccocontrol-2020-056239	No	Not meeting inclusion criteria	Title and abstract screening
1769	Felicione NJ, Kaiser L, Leigh NJ, Page MK, Block AC, Schurr BE, et al. Comparing POD and MOD ENDS Users' Product Characteristics, Use Behaviors, and Nicotine Exposure. Nicotine Tob Res. 2022. doi:10.1093/ntr/ntac211	No	Not meeting inclusion criteria	Title and abstract screening
1770	Felicione NJ, Kaiser L, Leigh NJ, Page MK, Block AC, Schurr BE, et al. Comparing POD and MOD ENDS Users' Product Characteristics, Use Behaviors, and Nicotine Exposure. Nicotine Tob Res. 2023;25: 498–504. doi:10.1093/ntr/ntac211	No	Not meeting inclusion criteria	Title and abstract screening
1771	Felicione NJ, Schneller LM, Goniewicz ML, Hyland AJ, Cummings KM, Bansal-Travers M, et al. Oral Nicotine Product Awareness and Use Among People Who Smoke and Vape in the U.S. Am J Prev Med. 2022;63: 611–618. doi:10.1016/j.amepre.2022.04.019	No	Not meeting inclusion criteria	Title and abstract screening
1772	Felix AT, Ntarisa AV. Review of natural radioactivity in tobacco cigarette brands. J Env Radioact. 2023;272: 107348. doi:10.1016/j.jenvrad.2023.107348	No	Not meeting inclusion criteria	Title and abstract screening
1773	Feng J, Sosnoff CS, Bernert JT, Blount BC, Li Y, Del Valle-Pinero AY, et al. Urinary Nicotine Metabolites and Self-Reported Tobacco Use Among Adults in the Population Assessment of Tobacco and Health (PATH) Study, 2013-2014. Nicotine Tob Res. 2022;24: 768–777. doi:10.1093/ntr/ntab206	No	Not meeting inclusion criteria	Title and abstract screening
1774	Feng W, Smith CM, Simpson DA, Gupta GP. Targeting Non-homologous and Alternative End Joining Repair to Enhance Cancer Radiosensitivity. Semin Radiat Oncol. 2022;32: 29–41. doi:10.1016/j.semradonc.2021.09.007	No	Not meeting inclusion criteria	Title and abstract screening
1775	Fenton ATHR, Fletcher KM, Kizildag D, Borstelmann NA, Kessler D, Cronin C, et al. Cancer Caregivers' Prognostic and End-of-Life Communication Needs and Experiences and their Impact. J Pain Symptom Manage. 2023;65: 16–25. doi:10.1016/j.jpainsymman.2022.09.012	No	Not meeting inclusion criteria	Title and abstract screening
1776	Ferkol T.W. Electronic cigarettes use in children and adolescents. Pediatr Pulmonol. 2021;56: S14–S16. doi:10.1002/ppul.25497	No	Not meeting inclusion criteria	Title and abstract screening
1777	Ferkol TW, Farber HJ, La Grutta S, Leone FT, Marshall HM, Neptune E, et al. Electronic cigarette use in youths: a position statement of the Forum of International Respiratory Societies. Eur Respir J. 2018;51. doi:10.1183/13993003.00278-2018	No	Not meeting inclusion criteria	Title and abstract screening
1778	Fernandes TP, Silverstein SM, Almeida NL, Santos NA. Visual impairments in tobacco use disorder. Psychiatry Res. 2019;271: 60–67. doi:10.1016/j.psychres.2018.11.024	No	Not meeting inclusion criteria	Title and abstract screening
1779	Fernández-García D, Ordás B, Fernández-Peña R, Bárcena-Calvo C, Ordoñez C, Amo-Setién FJ, et al. Smoking in nursing students: A prevalence multicenter study. Med Baltim. 2020;99: e19414. doi:10.1097/MD.0000000000019414	No	Not meeting inclusion criteria	Title and abstract screening

1780	Fernández-Ruiz I. A proteomic model shows potential as a surrogate end point for CVD risk. Nat Rev Cardiol. 2022;19: 352–352. doi:10.1038/s41569-022-00716-7	No	Not meeting inclusion criteria	Title and abstract screening
1781	Fernández-Ruiz I. CVD screening trial in older men misses primary end point but suggests benefits. Nat Rev Cardiol. 2022;19: 722–722. doi:10.1038/s41569-022-00781-y	No	Not meeting inclusion criteria	Title and abstract screening
1782	Ferrara P, Antonazzo IC, Polosa R. Response to BNT162b2 mRNA COVID-19 vaccine among healthcare workers in Italy: a 3-month follow-up-Reply. Intern Emerg Med. 2022;17: 313–314. doi:10.1007/s11739-021-02911-9	No	Not meeting inclusion criteria	Title and abstract screening
1783	Ferrara P, Battiato S, Polosa R. Progress and prospects for artificial intelligence in clinical practice: learning from COVID-19. Intern Emerg Med. 2022;17: 1855–1857. doi:10.1007/s11739-022-03080-z	No	Not meeting inclusion criteria	Title and abstract screening
1784	Ferrara P, Gianfredi V, Tomaselli V, Polosa R. The Effect of Smoking on Humoral Response to COVID-19 Vaccines: A Systematic Review of Epidemiological Studies. Vaccines Basel. 2022;10. doi:10.3390/vaccines10020303	No	Not meeting inclusion criteria	Title and abstract screening
1785	Fery C., Desombre A., Quirin T., Badertscher P., Sticherling C., Knecht S., et al. Magnetic Field Measurements of Portable Electronic Devices: The Risk Inside Pockets for Patients With Cardiovascular Implantable Devices. Circ Arrhythm Electrophysiol. 2022;15: E010646. doi:10.1161/CIRCEP.121.010646	No	Not meeting inclusion criteria	Title and abstract screening
1786	Filaudeau E., Sabo A.-N., Meyer E., Monassier L., Kemmel V. P14-08: Cytotoxicity and oxidative stress induced by different berry e-liquid flavors on an alveolar-capillary cell model according to two exposure methods. Toxicol Lett. 2023;384: S169. doi:10.1016/S0378-4274%2823%2900665-3	No	Not meeting inclusion criteria	Title and abstract screening
1787	Filby S, van der Zee K, van Walbeek C. The temporary ban on tobacco sales in South Africa: lessons for endgame strategies. Tob Control. 2022;31: 694–700. doi:10.1136/tobaccocontrol-2020-056209	No	Not meeting inclusion criteria	Title and abstract screening
1788	Filippidis FT, Laverty AA, Mons U, Jimenez-Ruiz C, Vardavas CI. Changes in smoking cessation assistance in the European Union between 2012 and 2017: pharmacotherapy versus counselling versus e-cigarettes. Tob Control. 2019;28: 95–100. doi:10.1136/tobaccocontrol-2017-054117	No	Not meeting inclusion criteria	Title and abstract screening
1789	Finck JM, Bohnet S, Auth K, Tangemann-Munstedt I, Dromann D, Franzen KF. Smoking Behavior and Smoking Cessation Because of and during the COVID-19 Pandemic: A Brief Online Survey 12 Months into the Pandemic and during the Second Wave in Europe. Int J Environ Res Public Health. 2022;19. doi:10.3390/ijerph192416540	No	Not meeting inclusion criteria	Title and abstract screening
1790	Firigato I, López RVM, Curioni OA, De Antonio J, Gattás GF, de Toledo Gonçalves F. Many hands make light work: CNV of GSTM1 effect on the oral carcinoma risk. Cancer Epidemiol. 2022;78: 102150. doi:10.1016/j.canep.2022.102150	No	Not meeting inclusion criteria	Title and abstract screening
1791	FISCHER E., LO T. CHINA WHITE IN ASTHMATIC RECREATIONAL DRUG USERS: DOES IT CONTRIBUTE TO PNEUMATOCELE DEVELOPMENT? Chest. 2022;162: A1397. doi:10.1016/j.chest.2022.08.1178	No	Not meeting inclusion criteria	Title and abstract screening
1792	Fitzpatrick P., Bhardwaj N., Niranjan V., Frazer K., Lyons A., Guerin S., et al. "IT'S A MARATHON AND NOT A SPRINT" - QUALITATIVE FINDINGS FROM PATIENTS WITH CANCER WHO SMOKE/ SMOKED TO INFORM THE DEVELOPMENT OF A SMOKING CESSATION PATHWAY IN CANCER SERVICES. J Epidemiol Community Health. 2023;77: A92. doi:10.1136/jech-2023-SSMabstracts.189	No	Not meeting inclusion criteria	Title and abstract screening
1793	Fix BV, OConnor RJ, Goniewicz ML, Leigh NL, Cummings M, Hitchman SC, et al. Characterisation of vaping liquids used in vaping devices across four countries: results from an analysis of selected vaping liquids reported by users in the 2016 ITC Four Country Smoking and Vaping Survey. Tob Control. 2023;32: 51–59. doi:10.1136/tobaccocontrol-2020-056338	No	Not meeting inclusion criteria	Title and abstract screening
1794	Flannery J.S. Disregarding the impact of nicotine on the developing brain when evaluating costs and benefits of noncombustible nicotine products. Prev Med. 2019;120: 157. doi:10.1016/j.ypmed.2018.11.023	No	Not meeting inclusion criteria	Title and abstract screening
1795	Flint S.W., Hennessy M. Are e-cigarette product advertisements on public transport driving public misunderstanding and potentially increased use? Perspect Public Health. 2020;140: 91–92. doi:10.1177/1757913919884592	No	Not meeting inclusion criteria	Title and abstract screening
1796	Florez MA, Jaoude JA, Patel RR, Kouzy R, Lin TA, De B, et al. Incidence of Primary End Point Changes Among Active Cancer Phase 3 Randomized Clinical Trials. JAMA Netw Open. 2023;6: e2313819–e2313819. doi:10.1001/jamanetworkopen.2023.13819	No	Not meeting inclusion criteria	Title and abstract screening
1797	Folan P, Abramova R, Fardellone C. What Is Menthol? Am J Respir Crit Care Med. 2017;196: P9–P10. doi:10.1164/rccm.1965P9	No	Not meeting inclusion criteria	Title and abstract screening
1798	Folayan MO, Alade O, Adeyemo Y, Sabbagh HJ, Oyapero A, Oziegbe EO, et al. Differences in risk indicators associated with electronic cigarette use and tobacco smoking among adolescents and young people in Nigeria. BMJ Open Respir Res. 2022;9. doi:10.1136/bmjresp-2022-001285	No	Not meeting inclusion criteria	Title and abstract screening
1799	Fong GT, Chung-Hall J, Meng G, Craig LV, Thompson ME, Quah ACK, et al. Impact of Canada's menthol cigarette ban on quitting among menthol smokers: pooled analysis of pre-post evaluation from the ITC Project and the Ontario Menthol Ban Study and projections of impact in the USA. Tob Control. 2022. doi:10.1136/tobaccocontrol-2021-057227	No	Not meeting inclusion criteria	Title and abstract screening
1800	Fong GT, Chung-Hall J, Meng G, Craig LV, Thompson ME, Quah ACK, et al. Impact of Canada's menthol cigarette ban on quitting among menthol smokers: pooled analysis of pre-post evaluation from the ITC Project and the Ontario Menthol Ban Study and projections of impact in the USA. Tob Control. 2023;32: 734–738. doi:10.1136/tobaccocontrol-2021-057227	No	Not meeting inclusion criteria	Title and abstract screening
1801	Fonseca Fuentes X, Kashyap R, Hays JT, Chalmers S, Lama von Buchwald C, Gajic O, et al. VpALI-Vaping-related Acute Lung Injury: A New Killer Around the Block. Mayo Clin Proc. 2019;94: 2534–2545. doi:10.1016/j.mayocp.2019.10.010	No	Not meeting inclusion criteria	Title and abstract screening
1802	Ford A, Uny I, Lowes J, Naughton F, Cooper S, Coleman T, et al. A Qualitative Study of Factors Influencing Adherence among Pregnant Women Taking Part in a Trial of E-Cigarettes for Smoking Cessation. Int J Env Res Public Health. 2021;18. doi:10.3390/ijerph18020430	No	Not meeting inclusion criteria	Title and abstract screening
1803	Ford MA, Lapham GT, Matson TE, Luce C, Oliver MM, Binswanger IA. Comparing Cannabis Use for Pain to Use for Other Reasons in Primary Care Patients. J Am Board Fam Med. 2023. doi:10.3122/jabfm.2023.230163R1	No	Not meeting inclusion criteria	Title and abstract screening
1804	Forest V, Pourchez J. Nano-delivery to the lung - by inhalation or other routes and why nano when micro is largely sufficient? Adv Drug Deliv Rev. 2022;183: 114173. doi:10.1016/j.addr.2022.114173	No	Not meeting inclusion criteria	Title and abstract screening
1805	Formanek P, Salisbury-Afshar E, Afshar M. Helping Patients With ESRD and Earlier Stages of CKD to Quit Smoking. Am J Kidney Dis. 2018;72: 255–266. doi:10.1053/j.ajkd.2018.01.057	No	Not meeting inclusion criteria	Title and abstract screening
1806	Forno E, Abman SH, Singh J, Robbins ME, Selvadurai H, Schumacker PT, et al. Update in Pediatrics 2020. Am J Respir Crit Care Med. 2021;204: 274–284. doi:10.1164/rccm.202103-0605UP	No	Not meeting inclusion criteria	Title and abstract screening
1807	Foronjy R. E-cigarettes and COPD. Eur Clin Respir J. 2018;5: 1–2. doi:10.1080/20018525.2017.1401867	No	Not meeting inclusion criteria	Title and abstract screening
1808	Foulds J, Hobkirk A, Wasserman E, Richie J, Veldheer S, Krebs NM, et al. Estimation of compliance with exclusive smoking of very low nicotine content cigarettes using plasma cotinine. Prev Med. 2018;117: 24–29. doi:10.1016/j.ypmed.2018.04.011	No	Not meeting inclusion criteria	Title and abstract screening

1809	Foulds J., Cobb C.O., Yen M.-S., Veldheer S., Brosnan P., Yingst J., et al. Effect of Electronic Nicotine Delivery Systems on Cigarette Abstinence in Smokers With No Plans to Quit: Exploratory Analysis of a Randomized Placebo-Controlled Trial. Nicotine Tob Res. 2022;24: 955–961. doi:10.1093/ntr/ntab247	No	Not meeting inclusion criteria	Title and abstract screening
1810	Foulkes I., Sharpless N.E. Cancer grand challenges: Embarking on a new era of discovery. Cancer Discov. 2021;11: 23–27. doi:10.1158/2159-8290.CD-20-1657	No	Not meeting inclusion criteria	Title and abstract screening
1811	Fountoulakis P, Theofilis P, Tsalamandris S, Antonopoulos AS, Tsioufis P, Toutouzas K, et al. The cardiovascular consequences of electronic cigarette smoking: a narrative review. Expert Rev Cardiovasc Ther. 2023;21: 651–661. doi:10.1080/14779072.2023.2264179	No	Not meeting inclusion criteria	Title and abstract screening
1812	Fowler CD, Turner JR, Imad Damaj M. Molecular Mechanisms Associated with Nicotine Pharmacology and Dependence. Handb Exp Pharmacol. 2020;258: 373–393. doi:10.1007/164_2019_252	No	Not meeting inclusion criteria	Title and abstract screening
1813	Fox KR, Ferketich AK, Groner JA, Rausch JR, Garg V, Grant VR, et al. The Association of Global and Disease-Related Stress With Susceptibility to and Use of E-Cigarettes and Marijuana Among Adolescents With Congenital Heart Disease. J Pediatr Psychol. 2023;48: 458–467. doi:10.1093/jpepsy/jsad005	No	Not meeting inclusion criteria	Title and abstract screening
1814	Franck C., Filion K.B., Eisenberg M.J. Smoking Cessation in Patients With Acute Coronary Syndrome. Am J Cardiol. 2018;121: 1105–1111. doi:10.1016/j.amjcard.2018.01.017	No	Not meeting inclusion criteria	Title and abstract screening
1815	FRANKI R. Vaping habit may lead to nicotine addiction in teens. Chest Physician. 2019;14: 13–13.	No	Not meeting inclusion criteria	Title and abstract screening
1816	Franzen K, Pankow W, Andreas S. [The e-cigarette - means of smoking cessation?]. Dtsch Med Wochenschr. 2022;147: 1481–1487. doi:10.1055/a-1860-5696	No	Not meeting inclusion criteria	Title and abstract screening
1817	Franzen K, Pankow W, Andreas S. [The e-cigarette - means of smoking cessation?]. Laryngorhinootologie. 2023. doi:10.1055/a-2194-5666	No	Not meeting inclusion criteria	Title and abstract screening
1818	Fraser T, Glover M, Truman P. Government and public health responses to e-cigarettes in New Zealand: vapers' perspectives. Harm Reduct J. 2018;15: 13. doi:10.1186/s12954-018-0219-9	No	Not meeting inclusion criteria	Title and abstract screening
1819	Frazier J., Coblentz T., Bruce J., Nassabeh S., Plants R., Burrage E., et al. Effect of E-liquid Base Solution (Vegetable Glycerin or Propylene Glycol) on Aortic Function in Mice. FASEB J. 2021;35. doi:10.1096/fasebj.2021.35.S1.01638	No	Not meeting inclusion criteria	Title and abstract screening
1820	Freedman J.E., Trivedi C.M. The Adverse Vascular Effects of E-Cigarettes: Smoke Without the Fire. J Am Coll Cardiol. 2019;73: 2738–2739. doi:10.1016/j.jacc.2019.02.072	No	Not meeting inclusion criteria	Title and abstract screening
1821	Freeman B. Reigniting tobacco control: returning Australia to the front of the pack. Public Health Res Pr. 2023;33. doi:10.17061/phrp3312304	No	Not meeting inclusion criteria	Title and abstract screening
1822	Freeman B, Owen K, Rickards S, Brooks A, Clare PJ, Dessaix A. E-cigarette use by people who smoke or have recently quit, New South Wales, 2016-2020. Med J Aust. 2023;218: 131–137. doi:10.5694/mja2.51811	No	Not meeting inclusion criteria	Title and abstract screening
1823	Freeman B, Owen K, Rickards S, Brooks A, Clare PJ, Dessaix A. E-cigarette use by people who smoke or have recently quit, New South Wales, 2016-2020. Med J Aust. 2022. doi:10.5694/mja2.51811	No	Not meeting inclusion criteria	Title and abstract screening
1824	Freitas-Lemos R, Stein JS, Pope DA, Brown J, Feinstein M, Stamborski KM, et al. E-liquid purchase as a function of workplace restriction in the experimental tobacco marketplace. Exp Clin Psychopharmacol. 2022;30: 371–377. doi:10.1037/pha0000444	No	Not meeting inclusion criteria	Title and abstract screening
1825	Freitas-Lemos R, Stein JS, Tegge AN, Kaplan BA, Heckman BW, McNeill A, et al. Illegal Experimental Tobacco Marketplace II: effects of vaping product bans - findings from the 2020 International Tobacco Control Project. Tob Control. 2022;31: s214–s222. doi:10.1136/tc-2022-057515	No	Not meeting inclusion criteria	Title and abstract screening
1826	Freitas-Lemos R, Tegge AN, Athamneh LN, Yeh YH, Craft WH, Stein JS, et al. Is perception reality? Associations among "light" cigarettes and number of cigarettes smoked per day. Drug Alcohol Depend. 2023;244: 109709. doi:10.1016/j.drugalcdep.2022.109709	No	Not meeting inclusion criteria	Title and abstract screening
1827	Freitas-Lemos R, Tegge AN, Stein JS, DeHart WB, Reisinger SA, Shields PG, et al. The experimental tobacco marketplace: Effects of low-ventilated cigarette exposure. Addict Behav. 2022;125: 107160. doi:10.1016/j.addbeh.2021.107160	No	Not meeting inclusion criteria	Title and abstract screening
1828	Freitas-Lemos R, Tegge AN, Tomlinson DC, Yeh YH, Stein JS, Michael Cummings K, et al. Illegal product purchasing in the experimental tobacco marketplace: Effects of menthol cigarette and cigarette ventilation ban. Drug Alcohol Depend. 2023;253: 111015. doi:10.1016/j.drugalcdep.2023.111015	No	Not meeting inclusion criteria	Title and abstract screening
1829	Frey S.M., Burkard T., Meienberg A. Smoking cessation in peripheral peripheral artery disease: "It always pays to stop smoking!" Gefasschirurgie. 2020;25: 172–178. doi:10.1007/s00772-020-00627-2	No	Not meeting inclusion criteria	Title and abstract screening
1830	Frie J.A., Nolan C.J., Murray J.E., Khokhar J.Y. Addiction-Related Outcomes of Nicotine and Alcohol Co-use: New Insights Following the Rise in Vaping. Nicotine Tob Res. 2022;24: 1141–1149. doi:10.1093/ntr/ntab231	No	Not meeting inclusion criteria	Title and abstract screening
1831	Fried N., Morris T., Whitehead A., Lazartigues E., Yue X., Gardner J. Chronic Inhaled Nicotine-Induced Pulmonary Hypertension and Right Ventricular Remodeling are Mediated by Angiotensin-II Type 1 Receptor. FASEB J. 2021;35. doi:10.1096/fasebj.2021.35.S1.01483	No	Not meeting inclusion criteria	Title and abstract screening
1832	Fried ND, Morris TM, Whitehead A, Lazartigues E, Yue X, Gardner JD. Angiotensin II type 1 receptor mediates pulmonary hypertension and right ventricular remodeling induced by inhaled nicotine. Am J Physiol Heart Circ Physiol. 2021;320: H1526–H1534. doi:10.1152/ajpheart.00883.2020	No	Not meeting inclusion criteria	Title and abstract screening
1833	Friedman A.S., Tam J. E-Cigarettes: Matching Risks With Regulations. Am J Prev Med. 2021;60: 146–150. doi:10.1016/j.amepre.2020.07.011	No	Not meeting inclusion criteria	Title and abstract screening
1834	Friedman AS. Association of vaping-related lung injuries with rates of e-cigarette and cannabis use across US states. Addiction. 2021;116: 651–657. doi:10.1111/add.15235https://dx.doi.org/10.1111/add.15235	No	Not meeting inclusion criteria	Title and abstract screening
1835	Friedman AS, Morean ME. State marijuana policies and vaping associated lung injuries in the US. Drug Alcohol Depend. 2021;228: 109086. doi:10.1016/j.drugalcdep.2021.109086	No	Not meeting inclusion criteria	Title and abstract screening
1836	Friedman J, Schooler GR, Kwon JK, Artunduaga M. Pediatric electronic cigarette or vaping product use-associated lung injury (EVALI): updates in the coronavirus disease 2019 (COVID-19) pandemic era. Pediatr Radiol. 2022;52: 2009–2016. doi:10.1007/s00247-022-05454-z	No	Not meeting inclusion criteria	Title and abstract screening
1837	Friedman N, Seltzer J, Harvey H, Ly B, Schneir A. Severe alcohol withdrawal in an adolescent male. Toxicol Rep. 2023;10: 428–430. doi:10.1016/j.toxrep.2023.03.006	No	Not meeting inclusion criteria	Title and abstract screening

1838	Fu R, Shi J, Chaiton M, Leventhal AM, Unger JB, Barrington-Trimis JL. A Machine Learning Approach to Identify Predictors of Frequent Vaping and Vulnerable Californian Youth Subgroups. Nicotine Tob Res. 2022;24: 1028–1036. doi:10.1093/ntr/ntab257	No	Not meeting inclusion criteria	Title and abstract screening
1839	Fucito LM, Malinosky H, Baldassarri SR, Herbst RS. Clearing the Haze: What Do We Still Need to Learn about Electronic Nicotine Delivery Systems?. Cancer Prev Res Phila Pa. 2021;14: 5–10. doi:10.1158/1940-6207.CAPR-20-0394	No	Not meeting inclusion criteria	Title and abstract screening
1840	Fucito LM, Bold KW, Baldassarri SR, LaVigne JP, Ford B, Sather P, et al. Use and perceptions of electronic nicotine delivery systems among patients attending lung cancer screening who smoke. Prev Med Rep. 2021;23: 101444. doi:10.1016/j.pmedr.2021.101444	No	Not meeting inclusion criteria	Title and abstract screening
1841	Fucito LM, Bold KW, Cannon S, Serrantino A, Marrero R, O'Malley SS. Cigarette Smoking in Response to COVID-19: Examining Co-Morbid Medical Conditions and Risk Perceptions. Int J Env Res Public Health. 2022;19. doi:10.3390/ijerph19148239	No	Not meeting inclusion criteria	Title and abstract screening
1842	Fuentes AL, Crotty Alexander LE. Beware, vaping e-cigarettes around children is adversely impacting their lung health. Thorax. 2022;77: 638–639. doi:10.1136/thoraxjnl-2021-218168	No	Not meeting inclusion criteria	Title and abstract screening
1843	Fujita Y., Nakamura J., Zhang Z., Matuda T., Kawanisi M. Alcohol Dehydrogenase-Mediated Activation of Glycidol Leads to the Generation of DNACrosslinking Metabolites. Environ Mol Mutagen. 2022;63: 79–80. doi:10.1002/em.22502	No	Not meeting inclusion criteria	Title and abstract screening
1844	Fulton-Ward T, Middleton G. The impact of genomic context on outcomes of solid cancer patients treated with genotype-matched targeted therapies: a comprehensive review. Ann Oncol. 2023. doi:10.1016/j.annonc.2023.10.124	No	Not meeting inclusion criteria	Title and abstract screening
1845	Funk OL, Nollen NL, Wagener TL, Ahluwalia JS, Mayo MS, Mahmud KMF, et al. Concurrent Choice Assessment of Preference and Substitutability of E-cigarettes and Heated Tobacco Products for Combustible Cigarettes Among African American and White Smokers. Nicotine Tob Res. 2023;25: 1505–1508. doi:10.1093/ntr/ntad052	No	Not meeting inclusion criteria	Title and abstract screening
1846	Funt SA, McHugh DJ, Tsai S, Knezevic A, O'Donnell D, Patil S, et al. Four Cycles of Etoposide plus Cisplatin for Patients with Good-Risk Advanced Germ Cell Tumors. The oncologist. 2021;26: 483–491. doi:10.1002/onco.13719	No	Not meeting inclusion criteria	Title and abstract screening
1847	Furlow B. US researchers call for FDA action to avoid another EVALI outbreak. Lancet Respir Med. 2023;11: e39. doi:10.1016/S2213-2600(23)00045-0	No	Not meeting inclusion criteria	Title and abstract screening
1848	Furlow B. Juul settles youth e-cigarette marketing lawsuits. Lancet Respir Med. 2023;11: e56. doi:10.1016/S2213-2600(23)00179-0	No	Not meeting inclusion criteria	Title and abstract screening
1849	Fuster D, Studer J, Gmel G, Bertholet N. Correlates of the use of electronic devices to vape cannabis in a cohort of young Swiss male reporting current cannabis use. Eur J Public Health. 2021;31: 437–441. doi:10.1093/eurpub/ckaa176	No	Not meeting inclusion criteria	Title and abstract screening
1850	Gaalema DE, Snell LM, Tidey JW, Sigmon SC, Heil SH, Lee DC, et al. Potential effects of nicotine content in cigarettes on use of other substances. Prev Med. 2022; 107290. doi:10.1016/j.ypmed.2022.107290	No	Not meeting inclusion criteria	Title and abstract screening
1851	Gaddey HL, Dakkak M, Jackson NM. Smoking Cessation Interventions. Am Fam Physician. 2022;106: 513–522.	No	Not meeting inclusion criteria	Title and abstract screening
1852	Gades MS, Alcheva A, Riegelman AL, Hatsukami DK. The Role of Nicotine and Flavor in the Abuse Potential and Appeal of Electronic Cigarettes for Adult Current and Former Cigarette and Electronic Cigarette Users: A Systematic Review. Nicotine Tob Res. 2022;24: 1332–1343. doi:10.1093/ntr/ntac073	No	Not meeting inclusion criteria	Title and abstract screening
1853	Gades MS, Hatsukami DK. Response to: Appropriate Policy Implications of the Fact that High Content and Flavored e-Cigarettes have Higher Abuse Liability. Nicotine Tob Res. 2022;24: 1515–1516. doi:10.1093/ntr/ntac120	No	Not meeting inclusion criteria	Title and abstract screening
1854	Gage S.H., Maynard O.M. Smoke-free policies in psychiatric hospitals need resources. Lancet Psychiatry. 2017;4: 509–510. doi:10.1016/S2215-0366%2817%2930241-9	No	Not meeting inclusion criteria	Title and abstract screening
1855	Gaiha S.M., Halpern-Felsher B. Corroborating Adolescent Tobacco Use and Sociodemographic Patterns From Multiple National Surveys. J Adolesc Health. 2021;68: 642–643. doi:10.1016/j.jadohealth.2021.01.015	No	Not meeting inclusion criteria	Title and abstract screening
1856	Gaiha S.M., Halpern-Felsher B. Stemming the tide of youth E-cigarette use: Promising progress in the development and evaluation of E-cigarette prevention and cessation programs. Addict Behav. 2021;120: 106960. doi:10.1016/j.addbeh.2021.106960	No	Not meeting inclusion criteria	Title and abstract screening
1857	Gaiha S.M., Halpern-Felsher B. 126. Adolescent Perceptions of Different e-Cigarette Marketing Characteristics in Retail, Online and Social Media. J Adolesc Health. 2022;70: S67. doi:10.1016/j.jadohealth.2022.01.043	No	Not meeting inclusion criteria	Title and abstract screening
1858	Gaiha S.M., Lempert L.K., McKelvey K., Halpern-Felsher B. 109. Adolescent E-Cigarette Use and Access Before and During the COVID-19 Pandemic. J Adolesc Health. 2021;68: S57. doi:10.1016/j.jadohealth.2020.12.118	No	Not meeting inclusion criteria	Title and abstract screening
1859	Gaiha S.M., Warnock A., Kile S., Brake K., Vong do Rosario C., Oates G.R., et al. Does virtual versus in-person e-cigarette education have a differential impact? Health Educ J. 2022;81: 891–900. doi:10.1177/00178969221119287	No	Not meeting inclusion criteria	Title and abstract screening
1860	Gaiha SM, Duemler A, Silverwood L, Razo A, Halpern-Felsher B, Walley SC. School-based e-cigarette education in Alabama: Impact on knowledge of e-cigarettes, perceptions and intent to try. Addict Behav. 2021;112: 106519. doi:10.1016/j.addbeh.2020.106519	No	Not meeting inclusion criteria	Title and abstract screening
1861	Gaiha SM, Epperson AE, Halpern-Felsher B. Youth perceptions of e-cigarette-related risk of lung issues and association with e-cigarette use. Health Psychol. 2022;41: 417–422. doi:10.1037/hea0001146	No	Not meeting inclusion criteria	Title and abstract screening
1862	Gaiha SM, Halpern-Felsher B. Public Health Considerations for Adolescent Initiation of Electronic Cigarettes. Pediatrics. 2020;145: S175–S180. doi:10.1542/peds.2019-2056E	No	Not meeting inclusion criteria	Title and abstract screening
1863	Gaiha SM, Lempert LK, Halpern-Felsher B. Underage Youth and Young Adult e-Cigarette Use and Access Before and During the Coronavirus Disease 2019 Pandemic. JAMA Netw Open. 2020;3: e2027572. doi:10.1001/jamanetworkopen.2020.27572	No	Not meeting inclusion criteria	Title and abstract screening
1864	Gaikwad RN, Alqifari F, Alnasser M, Bajad P, Jain P, Gondivkar S. Smoking cessation interventions in patients diagnosed with head and neck cancers: A systematic review of randomized controlled trials. Int J Health Sci Qassim. 2023;17: 45–53.	No	Not meeting inclusion criteria	Title and abstract screening
1865	Galderisi A., Ferraro V.A., Caserotti M., Quareni L., Perilongo G., Baraldi E. Protecting youth from the vaping epidemic. Pediatr Allergy Immunol. 2020;31: 66–68. doi:10.1111/pai.13348	No	Not meeting inclusion criteria	Title and abstract screening
1866	Galderisi A, Ferraro VA, Caserotti M, Quareni L, Perilongo G, Baraldi E. Protecting youth from the vaping epidemic. Pediatr Allergy Immunol Off Publ Eur Soc Pediatr Allergy Immunol. 2020;31 Suppl 26: 66–68. doi:10.1111/pai.13348	No	Not meeting inclusion criteria	Title and abstract screening

1867	Gale N, McEwan M, Camacho OM, Hardie G, Proctor CJ, Murphy J. Changes in biomarkers after 180 days of tobacco heating product use: a randomised trial. Intern Emerg Med. 2021;16: 2201–2212. doi:10.1007/s11739-021-02798-6	No	Not meeting inclusion criteria	Title and abstract screening
1868	Gale N, McEwan M, Hardie G, Proctor CJ, Murphy J. Changes in biomarkers of exposure and biomarkers of potential harm after 360 days in smokers who either continue to smoke, switch to a tobacco heating product or quit smoking. Intern Emerg Med. 2022;17: 2017–2030. doi:10.1007/s11739-022-03062-1	No	Not meeting inclusion criteria	Title and abstract screening
1869	Galéra C, Salla J, Montagni I, Hanne-Poujade S, Salamon R, Grondin O, et al. Stress, attention deficit hyperactivity disorder (ADHD) symptoms and tobacco smoking: The i-Share study. Eur Psychiatry. 2017;45: 221–226. doi:10.1016/j.eurpsy.2017.07.007	No	Not meeting inclusion criteria	Title and abstract screening
1870	Gali K, Bokemeyer F, Behrens S, Möhl A, Obi N, Becher H, et al. Changes in cigarette smoking behavior among breast cancer and unaffected women - A prospective study in the MARIE cohort. Cancer Epidemiol. 2022;81: 102282. doi:10.1016/j.canep.2022.102282	No	Not meeting inclusion criteria	Title and abstract screening
1871	Gali K, Kastaun S, Pischke CR, Kotz D. Trends and consumption patterns in the use of e-cigarettes among adolescents and young adults in Germany (the DEBRA study). Addict Behav. 2022;133: 107375. doi:10.1016/j.addbeh.2022.107375	No	Not meeting inclusion criteria	Title and abstract screening
1872	Galiatsatos P., Garfield J., Melzer A.C., Leone F.T., Farber H.J., Ruminjo J.K., et al. Summary for clinicians: An ats clinical practice guideline for initiating pharmacologic treatment in tobacco-dependent adults. Ann Am Thorac Soc. 2021;18: 187–190. doi:10.1513/AnnalsATS.202008-971CME	No	Not meeting inclusion criteria	Title and abstract screening
1873	Galica J, Saunders S, Romkey-Sinasac C, Silva A, Ethier J-L, Giroux J, et al. The needs of gynecological cancer survivors at the end of primary treatment: A scoping review and proposed model to guide clinical discussions. Patient Educ Couns. 2022;105: 1761–1782. doi:10.1016/j.pec.2021.11.020	No	Not meeting inclusion criteria	Title and abstract screening
1874	Galimov A, Meza L, Unger JB, Baezconde-Garbanati L, Cruz TB, Sussman S. Vape Shop Employees: Do They Act as Smoking Cessation Counselors?. Nicotine Tob Res Off J Soc Res Nicotine Tob. 2021;23: 756–759. doi:10.1093/ntr/ntaa218	No	Not meeting inclusion criteria	Title and abstract screening
1875	Gallagher K-P-D, Vargas P-A, Santos-Silva A-R. The use of E-cigarettes as a risk factor for oral potentially malignant disorders and oral cancer: a rapid review of clinical evidence. Med Oral Patol Oral Cirugia Bucal. 2023. doi:10.4317/medoral.26042	No	Not meeting inclusion criteria	Title and abstract screening
1876	Gallegos-Carrillo K, Barrientos-Gutiérrez I, Arillo-Santillán E, Zavala-Arciniega L, Cho YJ, Thrasher JF. Health Professionals' Counseling about Electronic Cigarettes for Smokers and Vapers in a Country That Bans the Sales and Marketing of Electronic Cigarettes. Int J Env Res Public Health. 2020;17. doi:10.3390/ijerph17020442	No	Not meeting inclusion criteria	Title and abstract screening
1877	Gallus S, Borroni E, Liu X, Carrozzi L, Dalla Pietra G, Eslami Varzaneh S, et al. Electronic cigarette use among Italian smokers: patterns, settings, and adverse events. Tumori. 2020; 300891620915784. doi:10.1177/0300891620915784	No	Not meeting inclusion criteria	Title and abstract screening
1878	Gallus S, Bosetti C, Gorini G, Stival C, Boffi R, Lugo A, et al. The Association of Tobacco Smoking, Second-hand Smoke, and Novel Tobacco Products With COVID-19 Severity and Mortality in Italy: Results From the COSMO-IT Study. J Epidemiol. 2023;33: 367–371. doi:10.2188/jea.JE20220321	No	Not meeting inclusion criteria	Title and abstract screening
1879	Gallus S, Lugo A, Liu X, Borroni E, Clancy L, Gorini G, et al. Use and Awareness of Heated Tobacco Products in Europe. J Epidemiol. 2022;32: 139–144. doi:10.2188/jea.JE20200248	No	Not meeting inclusion criteria	Title and abstract screening
1880	Gallus S, Lugo A, Stival C, Cerrai S, Clancy L, Filippidis FT, et al. Electronic cigarette use in 12 European countries. Results from the TackSHS survey. J Epidemiol. 2021. doi:10.2188/jea.JE20210329	No	Not meeting inclusion criteria	Title and abstract screening
1881	Gallus S, Lugo A, Stival C, Cerrai S, Clancy L, Filippidis FT, et al. Electronic Cigarette Use in 12 European Countries: Results From the TackSHS Survey. J Epidemiol. 2023;33: 276–284. doi:10.2188/jea.JE20210329	No	Not meeting inclusion criteria	Title and abstract screening
1882	Gallus S, Stival C, Carreras G, Gorini G, Amerio A, McKee M, et al. Use of electronic cigarettes and heated tobacco products during the Covid-19 pandemic. Sci Rep. 2022;12: 702. doi:10.1038/s41598-021-04438-7	No	Not meeting inclusion criteria	Title and abstract screening
1883	Galor A. Tumor necrosis factor a antibody drops meet primary efficacy end point. Ophthalmol Times. 2021;46: 11–12.	No	Not meeting inclusion criteria	Title and abstract screening
1884	Galper Grossman S. Vape Gods and Judaism-E-cigarettes and Jewish Law. Rambam Maimonides Med J. 2019;10. doi:10.5041/RMMJ.10372	No	Not meeting inclusion criteria	Title and abstract screening
1885	Gambaryan M, Kalinina A, Popovich M, Starovoytov M, Drapkina O, Boytsov S. Electronic cigarettes in Russia: time for an action. Results from Russian Tobacco Control policy evaluation survey. 2019;26: S113-. doi:10.1177/2047487319860053	No	Not meeting inclusion criteria	Title and abstract screening
1886	Gambaryan M, Popovich M, Kalinina A, Starovoytov M, Boytsov S, Drapkina O. Exposure of electronic cigarettes in Russia: results from Russian tobacco control policy evaluation study. 2018;52. doi:10.1183/13993003.congress-2018.OA5222	No	Not meeting inclusion criteria	Title and abstract screening
1887	Gambaryan M.G., Kalinina A.M., Popovich M.V., Starovoytov M.L., Drapkina O.M., Boytsov S.A., et al. The whole truth of electronic cigarettes: The russian reality. Part II. Use of electronic cigarettes in russia: Associations with demographic factors, advertisement and promotion, tobacco smoking and quit smoking attempts. Results from adult population re. Profil Meditsina. 2019;22: 14–27. doi:10.17116/profmed20192206214	No	Not meeting inclusion criteria	Title and abstract screening
1888	Gambichler T, Becker JC, Susok L, Käpynen R, Abu Rached N. Model for End-Stage Liver Disease Correlates with Disease Relapse and Death of Patients with Merkel Cell Carcinoma. Cancers. 2023;15: 3195. doi:10.3390/cancers15123195	No	Not meeting inclusion criteria	Title and abstract screening
1889	Gammon DG, Rogers T, Coats EM, Nonnemaker JM, Spinks JG, Valverde R, et al. Changes in Availability of ENDS: 2019-2020, U.S. Am J Prev Med. 2022;63: 1017–1025. doi:10.1016/j.amepre.2022.07.006	No	Not meeting inclusion criteria	Title and abstract screening
1890	Ganapathy V., Manyanga J., McGuire D., Brobst D., Sadhasivam B., Chinnaiyan M., et al. E-cigarette aerosol exposure increases NF-kB and modulates inflammatory markers in oral epithelial cells. Cancer Res. 2023;83. doi:10.1158/1538-7445.AM2023-4470	No	Not meeting inclusion criteria	Title and abstract screening
1891	Ganaway A, Sunaga Y, Ohta Y, Ohta J, Akay M, Akay YM. Investigating the Modulation of the VTA Neurons in Nicotine-Exposed Pups during Early Maturation Using Optogenetics. Int J Mol Sci. 2023;24. doi:10.3390/ijms24032280	No	Not meeting inclusion criteria	Title and abstract screening
1892	Ganguly K., Thimraj T.A., Nordstrom A., Ramstrom M., Lin E., O'Brien F., et al. Flavor, nicotine content, vaping regime, and lung region matter: Alarm anti-proteases and anti-microbial defensins are important for electronic cigarette related pulmonary response. Am J Respir Crit Care Med. 2020;201. Available: https://www.atsjournals.org/doi/abs/10.1164/ajrccm-conference.2020.201.1_MeetingAbstracts.A2768	No	Not meeting inclusion criteria	Title and abstract screening
1893	Gangwani M.K., Katyal A. Reexpansion pulmonary edema after pneumothorax drainage: An uncommon under-recognized complication. Crit Care Med. 2021;49: 469. doi:10.1097/01.ccm.0000729664.37784.fc	No	Not meeting inclusion criteria	Title and abstract screening
1894	Ganne N, Palraj R, Husted E, Shah I. E-cigarette or vaping product use-associated lung injury (EVALI) masquerading as COVID-19. BMJ Case Rep. 2021;14. doi:10.1136/bcr-2021-243885	No	Not meeting inclusion criteria	Title and abstract screening
1895	Gantiva C, Angel-Sanint L, Velasco-Vivas A. Impact of e-liquid warning labels on young adults' perception of e-cigarettes and intention to use them: an experimental online study. 2021. doi:10.1136/tobaccocontrol-2021-056761	No	Not meeting inclusion criteria	Title and abstract screening

1896	Gantiva C, Angel-Sanint L, Velasco-Vivas A. Impact of e-liquid warning labels on young adults' perception of e-cigarettes and intention to use them: An experimental online study. Tob Control Int J. 2023;32: e247–e250. doi:10.1136/tobaccocontrol-2021-056761	No	Not meeting inclusion criteria	Title and abstract screening
1897	Ganz O, Cohn AM, Goodwin RD, Giovenco DP, Wackowski OA, Talbot EM, et al. Internalizing problems are associated with initiation and past 30-Day use of flavored tobacco products. Addict Behav. 2022;125: 107162. doi:10.1016/j.addbeh.2021.107162	No	Not meeting inclusion criteria	Title and abstract screening
1898	Ganz O, Rimal RN, Johnson AL, Cohn AM, Horn K, Delnevo CD, et al. Tobacco use and the interplay of internalizing, externalizing and substance use problems: A latent class analysis of data from the Population Assessment of Tobacco and Health Study. Drug Alcohol Depend. 2019;205. doi:10.1016/j.drugalcdep.2019.107686https://dx.doi.org/10.1016/j.drugalcdep.2019.107686	No	Not meeting inclusion criteria	Title and abstract screening
1899	Gao B, Ma S, Zhao W, Tian M, Huang Y, Chen L, et al. Calculated cancer risks for polycyclic aromatic hydrocarbon mixtures in mainstream smoke of cigarettes sold in China. Regul Toxicol Pharmacol. 2023;142: 105427. doi:10.1016/j.yrtph.2023.105427	No	Not meeting inclusion criteria	Title and abstract screening
1900	Gao X, Humberstone L, Liu Y. Actual Use Behavior Assessment of a Novel Puff Recording Electronic Nicotine Delivery System: An Observation Study. JMIR Form Res. 2022. doi:10.2196/43175	No	Not meeting inclusion criteria	Title and abstract screening
1901	Garavaglia ML, Bodega F, Porta C, Milzani A, Sironi C, Dalle-Donne I. Molecular Impact of Conventional and Electronic Cigarettes on Pulmonary Surfactant. Int J Mol Sci. 2023;24. doi:10.3390/ijms241411702	No	Not meeting inclusion criteria	Title and abstract screening
1902	Gardner LA, Rowe AL, Newton NC, Aitken T, Stockings E, Thornton L, et al. School-based preventive interventions targeting e-cigarette use among adolescents: a systematic review protocol. BMJ Open. 2022;12: e065509. doi:10.1136/bmjopen-2022-065509	No	Not meeting inclusion criteria	Title and abstract screening
1903	Gardner LD, Liu ST, Xiao H, Anic GM, Kasza KA, Sharma E, et al. Electronic Nicotine Delivery System (ENDS) Device Types and Flavors Used by Youth in the PATH Study, 2016-2019. Int J Env Res Public Health. 2022;19. doi:10.3390/ijerph19095236	No	Not meeting inclusion criteria	Title and abstract screening
1904	Garey L, Olofsson H, Garza T, Shepherd JM, Smit T, Zvolensky MJ. The Role of Anxiety in Smoking Onset, Severity, and Cessation-Related Outcomes: a Review of Recent Literature. Curr Psychiatry Rep. 2020;22: 38. doi:10.1007/s11920-020-01160-5	No	Not meeting inclusion criteria	Title and abstract screening
1905	Garey L, Scott-Sheldon LAJ, Olofsson H, Nelson KM, Japuntich SJ. Electronic Cigarette Cessation among Adolescents and Young Adults. Subst Use Misuse. 2021;56: 1900–1903. doi:10.1080/10826084.2021.1958850	No	Not meeting inclusion criteria	Title and abstract screening
1906	Garey L, Smit T, Bizier A, Redmond BY, Ditre JW, Rogers AH, et al. Pain interference among adult dual combustible and electronic tobacco users in terms of perceived barriers for quitting. Exp Clin Psychopharmacol. 2023. doi:10.1037/pha0000660	No	Not meeting inclusion criteria	Title and abstract screening
1907	Garg I, Vidholia A, Garg A, Singh S, Agahi A, Laroia A, et al. E-cigarette or vaping product use-associated lung injury: A review of clinico-radio-pathological characteristics. Respir Investig. 2022;60: 738–749. doi:10.1016/j.resinv.2022.06.011	No	Not meeting inclusion criteria	Title and abstract screening
1908	Garg N, Patell R, Dodge LE, Miller EJ, Halleck J, Einstein DJ, et al. Patients' perceptions of treatment benefits and correlation with end-of-life quality markers in advanced malignancy. J Clin Oncol. 40: 188–188. doi:10.1200/JCO.2022.40.28_suppl.188	No	Not meeting inclusion criteria	Title and abstract screening
1909	Garg R, Croston MA, Thompson T, McQueen A, Kreuter MW. Correlates of smoking discarded cigarettes in a sample of low-income adults. Addict Behav. 2022;128: 107237. doi:10.1016/j.addbeh.2022.107237	No	Not meeting inclusion criteria	Title and abstract screening
1910	Garnett C, Oldham M, Shahab L, Tattan-Birch H, Cox S. Characterising smoking and smoking cessation attempts by risk of alcohol dependence: A representative, cross-sectional study of adults in England between 2014-2021. Lancet Reg Health Eur. 2022;18: 100418. doi:10.1016/j.lanepe.2022.100418	No	Not meeting inclusion criteria	Title and abstract screening
1911	Garofoli M. Adolescent Substance Abuse. Prim Care. 2020;47: 383–394. doi:10.1016/j.pop.2020.02.013	No	Not meeting inclusion criteria	Title and abstract screening
1912	Garretson M, Gentile D, Brown M, Asgarisabet P, Swain RS, Klink AJ, et al. Representation of age, race and ethnicity in real-world evidence (RWE), randomized clinical trial (RCT), and Surveillance, Epidemiology, and End Results (SEER) cancer patient registry data in advanced renal cell carcinoma (aRCC). J Clin Oncol. 41: e18672–e18672. doi:10.1200/JCO.2023.41.16_suppl.e18672	No	Not meeting inclusion criteria	Title and abstract screening
1913	Garrett PI, Honeycutt SC, Marston C, Allen N, Barraza AG, Dewey M, et al. Nicotine-free vapor inhalation produces behavioral disruptions and anxiety-like behaviors in mice: Effects of puff duration, session length, sex, and flavor. Pharmacol Biochem Behav. 2021;206: 173207. doi:10.1016/j.pbb.2021.173207	No	Not meeting inclusion criteria	Title and abstract screening
1914	Garside J, Shen Q, Westermayer B, van de Ven M, Kroep S, Chirikov V, et al. Association Between Intermediate End Points, Progression-free Survival, and Overall Survival in First-line Advanced or Recurrent Endometrial Cancer. Clin Ther. 2023;45: 983–990. doi:10.1016/j.clinthera.2023.07.025	No	Not meeting inclusion criteria	Title and abstract screening
1915	Gass JC, Morris DH, Winters J, VanderVeen JW, Chermack S. Characteristics and clinical treatment of tobacco smokers enrolled in a VA substance use disorders clinic. J Subst Abuse Treat. 2018;84: 1–8. doi:10.1016/j.jsat.2017.10.006	No	Not meeting inclusion criteria	Title and abstract screening
1916	Gaughan S, Williams M. The holistic management of malignant bowel obstruction in women with advanced ovarian cancer at end of life. Br J Nurs. 2023;32: 550–555. doi:10.12968/bjon.2023.32.12.550	No	Not meeting inclusion criteria	Title and abstract screening
1917	Geci M, Scialdone M, Tishler J. The Dark Side of Cannabidiol: The Unanticipated Social and Clinical Implications of Synthetic Δ(8)-THC. Cannabis Cannabinoid Res. 2023;8: 270–282. doi:10.1089/can.2022.0126	No	Not meeting inclusion criteria	Title and abstract screening
1918	Gelino BW, Reed DD, Spindle TR, Amlung M, Strickland JC. Association of electronic nicotine delivery system (ENDS) and cigarette solo and dual use with alcohol-related consequences among US adults. Addict Behav. 2023;146: 107806. doi:10.1016/j.addbeh.2023.107806	No	Not meeting inclusion criteria	Title and abstract screening
1919	Gentile A, Bianco A, Nordström A, Nordström P. Use of alcohol, drugs, inhalants, and smoking tobacco and the long-term risk of depression in men: A nationwide Swedish cohort study from 1969-2017. Drug Alcohol Depend. 2021;221: 108553. doi:10.1016/j.drugalcdep.2021.108553	No	Not meeting inclusion criteria	Title and abstract screening
1920	Gentile D, Balanean A, Asgarisabet P, Brown M, Swain RS, Klink AJ, et al. Comparison of diversity across real-world evidence, randomized clinical trial, and Surveillance, Epidemiology, and End Results (SEER) data in patients with metastatic breast cancer. J Clin Oncol. 41: e18670–e18670. doi:10.1200/JCO.2023.41.16_suppl.e18670	No	Not meeting inclusion criteria	Title and abstract screening
1921	Gentry S., Forouhi N.G., Notley C. Are electronic cigarettes an effective aid to smoking cessation or reduction among vulnerable groups? A systematic review of quantitative and qualitative evidence. Nicotine Tob Res. 2019;21: 602–616. doi:10.1093/ntr/nty054	No	Not meeting inclusion criteria	Title and abstract screening
1922	Gentry S.V., Ward E., Dawkins L., Holland R., Notley C. Reported patterns of vaping to support long-term abstinence from smoking: A cross-sectional survey of a convenience sample of vapers. Harm Reduct J. 2020;17: 70. doi:10.1186/s12954-020-00418-8	No	Not meeting inclusion criteria	Title and abstract screening
1923	Gentzke AS, Wang TW, Cornelius M, Park-Lee E, Ren C, Sawdey MD, et al. Tobacco Product Use and Associated Factors Among Middle and High School Students - National Youth Tobacco Survey, United States, 2021. MMWR Surveill Summ. 2022;71: 1–29. doi:10.15585/mmwr.ss7105a1	No	Not meeting inclusion criteria	Title and abstract screening
1924	Geringer R. HYPEREOSINOPHILIC SYNDROME: A CASE OF HYPOXIC RESPIRATORY FAILURE. J Gen Intern Med. 2023;38: S589–S590. doi:10.1007/s11606-023-08226-z	No	Not meeting inclusion criteria	Title and abstract screening

1925	Germeroth LJ, Baker NL, Saladin ME. Intolerance for smoking abstinence among nicotine-deprived, treatment-seeking smokers. Addict Behav. 2018;84: 13–19. doi:10.1016/j.addbeh.2018.03.019	No	Not meeting inclusion criteria	Title and abstract screening
1926	Gerresheim G, Schwemmer U. [Inhalative toxic lung failure-E-cigarette or vaping product use-associated lung injury]. Anaesthesiologie. 2023;72: 261–265. doi:10.1007/s00101-022-01246-0	No	Not meeting inclusion criteria	Title and abstract screening
1927	Gessner K.H., Siemens D.R., Bjurlin M.A. Does Smoking Cessation Reduce Bladder Cancer Recurrence? J Bone Jt Surg. 2022;207: 1178–1179. doi:10.1097/JU.0000000000002649	No	Not meeting inclusion criteria	Title and abstract screening
1928	Geste JR, Levin B, Wilks I, Pompilus M, Zhang X, Esser KA, et al. Relationship Between Nicotine Intake and Reward Function in Rats With Intermittent Short Versus Long Access to Nicotine. Nicotine Tob Res. 2020;22: 213–223. doi:10.1093/ntr/ntz052	No	Not meeting inclusion criteria	Title and abstract screening
1929	Getachew B, Payne JB, Vu M, Pillai D, Shah J, Levine H, et al. Perceptions of Alternative Tobacco Products, Anti-tobacco Media, and Tobacco Regulation among Young Adults: A Qualitative Study. Am J Health Behav. 2018;42: 118–130. doi:10.5993/AJHB.42.4.11	No	Not meeting inclusion criteria	Title and abstract screening
1930	Geynisman DM. The End of the Beginning-Lessons From the First 10 Years as an Oncologist. J Clin Oncol. 2021;39: 2516–2517. doi:10.1200/JCO.21.00378	No	Not meeting inclusion criteria	Title and abstract screening
1931	Ghafari G, Ali Y, Tahseen D, Liszewski WJ, West DP, Nardone B. 28481 Risk for nonmelanoma second primary malignancy in malignant melanoma survivors: A nationwide report from the National Cancer Institute Surveillance, Epidemiology, and End Results program. J Am Acad Dermatol. 2021;85: AB192–AB192. doi:10.1016/j.jaad.2021.06.782	No	Not meeting inclusion criteria	Title and abstract screening
1932	Ghamri R.A. Identification of the most effective pharmaceutical products for smoking cessation: A literature review. J Subst Use. 2018;23: 670–674. doi:10.1080/14659891.2018.1489010	No	Not meeting inclusion criteria	Title and abstract screening
1933	Gharzai LA, Jiang R, Jaworski EM, Morales Rivera K, Dess RT, Jackson WC, et al. Meta-Analysis of Candidate Surrogate End Points in Advanced Prostate Cancer. NEJM Evid. 2023;2: 1–11. doi:10.1056/EVIDoa2200195	No	Not meeting inclusion criteria	Title and abstract screening
1934	Ghebreyesus T.A. Progress in beating the tobacco epidemic. The Lancet. 2019;394: 548–549. doi:10.1016/S0140-6736%2819%2931730-1	No	Not meeting inclusion criteria	Title and abstract screening
1935	Ghiggia A, Pierotti V, Tesio V, Bovero A. Personality matters: relationship between personality characteristics, spirituality, demoralization, and perceived quality of life in a sample of end-of-life cancer patients. Support Care Cancer. 2021;29: 7775–7783. doi:10.1007/s00520-021-06363-x	No	Not meeting inclusion criteria	Title and abstract screening
1936	Gholap V, Halquist MS. Historical Perspective of Proactive and Reactive Regulations of E-cigarettes to Combat Nicotine Addiction. J Addict Med. 2020;14: 443–445. doi:10.1097/ADM.0000000000000627	No	Not meeting inclusion criteria	Title and abstract screening
1937	Gholap V.V., Pearcy A.C., Halquist M.S. Potential factors affecting free base nicotine yield in electronic cigarette aerosols. Expert Opin Drug Deliv. 2021;18: 979–989. doi:10.1080/17425247.2021.1890714	No	Not meeting inclusion criteria	Title and abstract screening
1938	Gholap VV, Kosmider L, Golshahi L, Halquist MS. Nicotine forms: why and how do they matter in nicotine delivery from electronic cigarettes?. Expert Opin Drug Deliv. 2020;17: 1727–1736. doi:10.1080/17425247.2020.1814736	No	Not meeting inclusion criteria	Title and abstract screening
1939	Ghosh A, Girish V, Yuan ML, Coakley RD, Wrennall JA, Alexis NE, et al. Combustible and Electronic Cigarette Exposures Increase ACE2 Activity and SARS-CoV-2 Spike Binding. Am J Respir Crit Care Med. 2022;205: 129–133. doi:10.1164/rccm.202106-1377LE	No	Not meeting inclusion criteria	Title and abstract screening
1940	Ghosh A., Ahmad S., Coakley R.D., Sassano M.F., Alexis N.E., Tarran R. Lipid-laden macrophages are not unique to patients with E-cigarette or vaping product use-associated lung injury. Am J Respir Crit Care Med. 2021;203: 1030–1033. doi:10.1164/RCCM.202009-3507LE	No	Not meeting inclusion criteria	Title and abstract screening
1941	Ghosh A, Beyazcicek O, Davis ES, Onyenwoke RU, Tarran R. Cellular effects of nicotine salt-containing e-liquids. J Appl Toxicol JAT. 2021;41: 493–505. doi:10.1002/jat.4060	No	Not meeting inclusion criteria	Title and abstract screening
1942	Ghosh A, Coakley RD, Ghio AJ, Muhlebach MS, Esther CRJ, Alexis NE, et al. Chronic E-Cigarette Use Increases Neutrophil Elastase and Matrix Metalloprotease Levels in the Lung. Am J Respir Crit Care Med. 2019;200: 1392–1401. doi:10.1164/rccm.201903-0615OC	No	Not meeting inclusion criteria	Title and abstract screening
1943	Ghosh T.S., Tolliver R., Reidmohr A., Lynch M. Youth vaping and associated risk behaviors - A snapshot of Colorado. N Engl J Med. 2019;380: 389–390. doi:10.1056/NEJMc1900830	No	Not meeting inclusion criteria	Title and abstract screening
1944	Ghura S, Gross R, Jordan-Sciutto K, Dubroff J, Schnoll R, Collman RG, et al. Bidirectional Associations among Nicotine and Tobacco Smoke, NeuroHIV, and Antiretroviral Therapy. J Neuroimmune Pharmacol. 2020;15: 694–714. doi:10.1007/s11481-019-09897-4	No	Not meeting inclusion criteria	Title and abstract screening
1945	Gianniosis M., Hart S. ANCHORING IN THE TIME OF COVID-19. Chest. 2021;160: A776. doi:10.1016/j.chest.2021.07.732	No	Not meeting inclusion criteria	Title and abstract screening
1946	Gibbs V, Geneen L, Champaneria R, Raval P, Dorée C, Brunskill S, et al. Pharmacological interventions for the prevention of bleeding in people undergoing definitive fixation or joint replacement for hip, pelvic and long bone fractures. Cochrane Database Syst Rev. 2023. doi:10.1002/14651858.CD013499.pub2	No	Not meeting inclusion criteria	Title and abstract screening
1947	Giberson J, Nardone N, Addo N, Khan S, Jacob P, Benowitz N, et al. Nicotine Intake in Adult Pod E-cigarette Users: Impact of User and Device Characteristics. Nicotine Tob Res Off J Soc Res Nicotine Tob. 2023;25: 1489–1495. doi:10.1093/ntr/ntad050	No	Not meeting inclusion criteria	Title and abstract screening
1948	Gibson-Young L, Martinasek M, Tamulevicius N, Fortner M, Alanazi AM. Examining electronic nicotine delivery system use and perception of use among college students with and without asthma across the South. J Am Coll Health. 2022;70: 2026–2032. doi:10.1080/07448481.2020.1842414	No	Not meeting inclusion criteria	Title and abstract screening
1949	Gibson-Young L, Martinasek M, Tamulevicius N, Fortner M, Alanazi AM. Examining electronic nicotine delivery system use and perception of use among college students with and without asthma across the south. J Am Coll Health. 2020; No-Specified. doi:10.1080/07448481.2020.1842414https://dx.doi.org/10.1080/07448481.2020.1842414	No	Not meeting inclusion criteria	Title and abstract screening
1950	Giger R, Schmid M, Schubert A, Nisa L, Mueller S, Schubert M. Benefit of a multiprofessional preoperative information day for head and neck cancer patients after complex surgery. 2021;118: 1-. doi:10.1016/S1368-8375(21)00407-3	No	Not meeting inclusion criteria	Title and abstract screening
1951	Giglio M, Preziosa A, Mele R, Brienza N, Grasso S, Puntillo F. Effects of an Intrathecal Drug Delivery System Connected to a Subcutaneous Port on Pain, Mood and Quality of Life in End Stage Cancer Patients: An Observational Study. Cancer Control J Moffitt Cancer Cent. 2022;29: 1–8. doi:10.1177/10732748221133752	No	Not meeting inclusion criteria	Title and abstract screening
1952	Gilbody S, Peckham E, Bailey D, Arundel C, Heron P, Crosland S, et al. Smoking cessation for people with severe mental illness (SCIMITAR+): a pragmatic randomised controlled trial. Lancet Psychiatry. 2019;6: 379–390. doi:10.1016/S2215-0366(19)30047-1	No	Not meeting inclusion criteria	Title and abstract screening
1953	Gilbody S., Peckham E. What does the rise and rise of electronic nicotine delivery systems mean for mental health services? Lancet Psychiatry. 2020;7: 11–13. doi:10.1016/S2215-0366%2819%2930479-1	No	Not meeting inclusion criteria	Title and abstract screening

1954	Giles ML, Gartner C, Boyd MA. Smoking and HIV: what are the risks and what harm reduction strategies do we have at our disposal? AIDS Res Ther. 2018;15: 26. doi:10.1186/s12981-018-0213-z	No	Not meeting inclusion criteria	Title and abstract screening
1955	Gimm G., Parekh T., Rossheim M.E. Prevalence and risk factors of e-cigarette use among working-age adults with and without disabilities in 2017-2018. Disabil Health J. 2021;14: 101048. doi:10.1016/j.dhjo.2020.101048	No	Not meeting inclusion criteria	Title and abstract screening
1956	Giovacchini CX, Crotty Alexander LE, Que LG. Electronic Cigarettes: A Pro-Con Review of the Current Literature. J Allergy Clin Immunol Pract. 2022;10: 2843–2851. doi:10.1016/j.jaip.2022.07.009	No	Not meeting inclusion criteria	Title and abstract screening
1957	Giovenco DP, Spillane TE, Maggi RM, Lee EY, Philbin MM. Multi-level drivers of tobacco use and purchasing behaviors during COVID-19 “lockdown”: A qualitative study in the United States. Int J Drug Policy. 2021;94: 103175. doi:10.1016/j.drugpo.2021.103175	No	Not meeting inclusion criteria	Title and abstract screening
1958	Giovino GA, Swan GE, Blount B, O’Malley S, Brown DC, Hendershot TP, et al. PhenX: Host: Biobehavioral measures for tobacco regulatory research. Tob Control. 2020;29: s13–s19. doi:10.1136/tobaccocontrol-2019-054975	No	Not meeting inclusion criteria	Title and abstract screening
1959	Gipson CD, Fowler CD. Nicotinic Receptors Underlying Nicotine Dependence: Evidence from Transgenic Mouse Models. Curr Top Behav Neurosci. 2020;45: 101–121. doi:10.1007/7854_2020_134	No	Not meeting inclusion criteria	Title and abstract screening
1960	Giralt A, Iskandar AR, Martin F, Moschini E, Serchi T, Kondylis A, et al. Comparison of the biological impact of aerosol of e-vapor device with MESH R technology and cigarette smoke on human bronchial and alveolar cultures. Toxicol Lett. 2021;337: 98–110. doi:10.1016/j.toxlet.2020.11.006	No	Not meeting inclusion criteria	Title and abstract screening
1961	Girard N, Galland-Girodet S, Avrillon V, Besse B, Duruisseaux M, Cadranel J, et al. Lorlatinib for advanced ROS1+ non-small-cell lung cancer: results of the IFCT-1803 LORLATU study. ESMO Open. 2022;7: 100418. doi:10.1016/j.esmoop.2022.100418	No	Not meeting inclusion criteria	Title and abstract screening
1962	Gittelman M, Southworth H, Carle AC, Anzeljc S, Qian X, Wervey Arnold M, et al. Types of Tobacco Products Used by Caregivers of Newborns in the Primary Care Setting. Clin Pediatr Phila. 2022;61: 535–541. doi:10.1177/00099228221091030	No	Not meeting inclusion criteria	Title and abstract screening
1963	Giulietti F, Filipponi A, Rosettani G, Giordano P, Iacoacci C, Spannella F, et al. Pharmacological Approach to Smoking Cessation: An Updated Review for Daily Clinical Practice. High Blood Press Cardiovasc Prev. 2020;27: 349–362. doi:10.1007/s40292-020-00396-9	No	Not meeting inclusion criteria	Title and abstract screening
1964	Gladstone DJ, Lindsay MP, Douketis J, Smith EE, Dowlatshahi D, Wein T, et al. Canadian Stroke Best Practice Recommendations: Secondary Prevention of Stroke Update 2020. Can J Neurol Sci. 2022;49: 315–337. doi:10.1017/cjn.2021.127	No	Not meeting inclusion criteria	Title and abstract screening
1965	Glantz S.A. Appropriate Policy Implications of the Fact That High Content and Flavored E-Cigarettes Have Higher Abuse Liability. Nicotine Tob Res. 2022;24: 1513–1514. doi:10.1093/ntr/ntac089	No	Not meeting inclusion criteria	Title and abstract screening
1966	Glantz S.A. The Perils of Drawing Strong Conclusions Based on Underpowered Analyses. Am J Prev Med. 2022;62: e137–e139. doi:10.1016/j.amepre.2021.09.010	No	Not meeting inclusion criteria	Title and abstract screening
1967	Glantz S.A., Halpern-Felsher B., Springer M.L. Marijuana, secondhand smoke, and social acceptability. JAMA Intern Med. 2018;178: 13–14. doi:10.1001/jamainternmed.2017.5301	No	Not meeting inclusion criteria	Title and abstract screening
1968	Glantz SA. e-Cigarettes Used by Adolescents to Try to Quit Smoking Are Associated With Less Quitting: A Cross-Sectional Analysis of the National Youth Tobacco Survey. J Adolesc Health. 2023;72: 359–364. doi:10.1016/j.jadohealth.2022.10.011	No	Not meeting inclusion criteria	Title and abstract screening
1969	Glantz SA. e-Cigarettes Used by Adolescents to Try to Quit Smoking Are Associated With Less Quitting: A Cross-Sectional Analysis of the National Youth Tobacco Survey. J Adolesc Health Off Publ Soc Adolesc Med. 2022. doi:10.1016/j.jadohealth.2022.10.011	No	Not meeting inclusion criteria	Title and abstract screening
1970	Glasgow TE, Miller CA, Barsell DJ, Do EK, Fuemmeler BF. Assessing how a tobacco-free campus leads to attitude change and support among students, faculty, and staff. Tob Prev Cessat. 2021;7: 53. doi:10.18332/tpc/138224	No	Not meeting inclusion criteria	Title and abstract screening
1971	Glasser A.M., Vojjala M., Cantrell J., Levy D.T., Giovenco D.P., Abrams D., et al. Patterns of E-cigarette use and subsequent cigarette smoking cessation over 2 Years (2013/2014-2015/2016) in the population assessment of tobacco and health study. Nicotine Tob Res. 2021;23: 669–677. doi:10.1093/ntr/ntaa182	No	Not meeting inclusion criteria	Title and abstract screening
1972	Glasser AM, Collins L, Pearson JL, Abudayyeh H, Niaura RS, Abrams DB, et al. Overview of Electronic Nicotine Delivery Systems: A Systematic Review. Am J Prev Med. 2017;52: e33–e66. doi:10.1016/j.amepre.2016.10.036	No	Not meeting inclusion criteria	Title and abstract screening
1973	Glover M., Breier B.H., Bauld L. Could Vaping be a New Weapon in the Battle of the Bulge? Nicotine Tob Res. 2017;19: 1536–1540. doi:10.1093/ntr/ntw278	No	Not meeting inclusion criteria	Title and abstract screening
1974	Glynn TJ, Hays JT, Kemper K. E-Cigarettes, Harm Reduction, and Tobacco Control: A Path Forward? Mayo Clin Proc. 2021;96: 856–862. doi:10.1016/j.mayocp.2020.11.022	No	Not meeting inclusion criteria	Title and abstract screening
1975	Godbee M., Jakate S., Eswaran S. A Case of Electronic Nicotine Delivery Systems (ENDS) Liver Injury. Hepatology. 2021;73: 1230–1232. doi:10.1002/hep.31475	No	Not meeting inclusion criteria	Title and abstract screening
1976	Godoy R, Callejas FJ, Cruz J, Tornero AI, Tárraga PJ, Rodríguez-Montes JA. [Comparative analysis: Effectiveness of nicotine addiction treatment in people with psychiatric comorbidity]. Semergen. 2018;44: 249–256. doi:10.1016/j.semerg.2017.03.008	No	Not meeting inclusion criteria	Title and abstract screening
1977	Goizueta A.A., Estrada-Y-Martin R.M., Cherian S.V. Lung Cancer in Women: a Review. Curr Pulmonol Rep. 2021;10: 53–61. doi:10.1007/s13665-021-00270-6	No	Not meeting inclusion criteria	Title and abstract screening
1978	Gokul K, Zhang Z, Subramaniam DS. Incidence of palliative care consultations in end-of-life care among patients with pancreatic cancer in the US. J Clin Oncol. 41: e16302–e16302. doi:10.1200/JCO.2023.41.16_suppl.e16302	No	Not meeting inclusion criteria	Title and abstract screening
1979	Golan R, Muthigi A, Ghomeshi A, White J, Saltzman RG, Diaz P, et al. Misconceptions of Vaping Among Young Adults. Cureus. 2023;15: e38202. doi:10.7759/cureus.38202	No	Not meeting inclusion criteria	Title and abstract screening
1980	Gold AK, Otto MW, Hoyt DL, Garey L, Smit T, Zvolensky M. Do Pain-Related Anxiety and Difficulties With Emotion Regulation Impact Abstinence Expectancies or Motivation to Quit E-Cigarette Use? J Stud Alcohol Drugs. 2021;82: 414–421. doi:10.15288/jsad.2021.82.414	No	Not meeting inclusion criteria	Title and abstract screening
1981	Goldberg LR, Gould TJ. Genetic influences impacting nicotine use and abuse during adolescence: Insights from human and rodent studies. Brain Res Bull. 2022;187: 24–38. doi:10.1016/j.brainresbull.2022.06.007	No	Not meeting inclusion criteria	Title and abstract screening
1982	Goldberg SB, Herbst RS. The end of the beginning: progress and next steps in KRAS-mutant non-small-cell lung cancer. Lancet. 2023;401: 706–707. doi:10.1016/S0140-6736(23)00288-X	No	Not meeting inclusion criteria	Title and abstract screening

1983	Goldberg Scott S., Feigelson H., Powers J., Clennin M., Lyons J., Gray M., et al. DEMOGRAPHIC, CLINICAL, AND BEHAVIORAL FACTORS ASSOCIATED WITH E-CIGARETTE USE IN A LARGE COHORT IN THE UNITED STATES. Chest. 2021;160: A2451. doi:10.1016/j.chest.2021.07.2118	No	Not meeting inclusion criteria	Title and abstract screening
1984	Golden S.E., Schweiger L., Melzer A.C., Ono S.S., Datta S., Davis J.M., et al. "It's a decision I have to make": Patient perspectives on smoking and cessation after lung cancer screening decisions. Prev Med Rep. 2022;30: 102014. doi:10.1016/j.pmedr.2022.102014	No	Not meeting inclusion criteria	Title and abstract screening
1985	Golden T., VanFrank B.K., Courtney-Long E. E-cigarette Screening and Clinical Intervention Behaviors Among Pediatric Primary Care Providers, Docstyles 2021. Pediatrics. 2022;149. Available: https://publications.aap.org/pediatrics/article/149/1MeetingAbstractsFebruary2022/740/186223/E-cigarette-Screening-and-Clinical-Intervention	No	Not meeting inclusion criteria	Title and abstract screening
1986	Goldenson N.I., Buchhalter A.R., Augustson E.M., Rubinstein M.L., Henningfield J.E. Abuse liability assessment of the JUUL system in four flavors relative to combustible cigarette, nicotine gum and a comparator electronic nicotine delivery system among adult smokers. Drug Alcohol Depend. 2020;217: 108395. doi:10.1016/j.drugalcdep.2020.108395	No	Not meeting inclusion criteria	Title and abstract screening
1987	Goldenson NI, Buchhalter AR, Augustson EM, Rubinstein ML, Van Hoof D, Henningfield JE. Abuse liability assessment of the JUUL system in two nicotine concentrations compared to combustible cigarette, nicotine gum and comparator electronic nicotine delivery system. Drug Alcohol Depend. 2020;217: 108441. doi:10.1016/j.drugalcdep.2020.108441	No	Not meeting inclusion criteria	Title and abstract screening
1988	Goldenson NI, Khoddam R, Stone MD, Leventhal AM. Associations of ADHD Symptoms With Smoking and Alternative Tobacco Product Use Initiation During Adolescence. J Pediatr Psychol. 2018;43: 613–624. doi:10.1093/jpepsy/jsx153	No	Not meeting inclusion criteria	Title and abstract screening
1989	Goldenson NI, Shiffman S, Hatcher C, Lamichhane D, Gaggar A, Le GM, et al. Switching away from Cigarettes across 12 Months among Adult Smokers Purchasing the JUUL System. Am J Health Behav. 2021;45: 443–463. doi:10.5993/AJHB.45.3.4	No	Not meeting inclusion criteria	Title and abstract screening
1990	Goldenson N, Fearon I, Buchhalter A, Henningfield J. An Open-Label, Randomized, Controlled, Crossover Study to Assess Nicotine Pharmacokinetics and Subjective Effects of the JUUL System with Three Nicotine Concentrations Relative to Combustible Cigarettes in Adult Smokers. 2021;23: 947-955. doi:10.1093/ntr/ntab001	No	Not meeting inclusion criteria	Title and abstract screening
1991	Goldie CL, Nguyen P, Robinson AG, Goldie CE, Kircher CE, Hanna TP. Quality of End-of-Life Care for People with Advanced Non-Small Cell Lung Cancer in Ontario: A Population-Based Study. Curr Oncol. 2021;28: 3297–3315. doi:10.3390/curroncol28050286	No	Not meeting inclusion criteria	Title and abstract screening
1992	Goldsteen P.A., Yoseif C., Dolga A.M., Gosens R. Human pluripotent stem cells for the modelling and treatment of respiratory diseases. Eur Respir Rev. 2021;30: 210042. doi:10.1183/16000617.0042-2021	No	Not meeting inclusion criteria	Title and abstract screening
1993	Golinelli D, Siconolfi D, Shadel WG, Seelam R, Tucker JS. Patterns of alternative tobacco product use among youth experiencing homelessness. Addict Behav. 2019;99: 106088. doi:10.1016/j.addbeh.2019.106088	No	Not meeting inclusion criteria	Title and abstract screening
1994	Gómez Cerezo JF, López Paz JE, Fernández Pardo J. Update on new forms of tobacco use. Clin Investig Arter. 2022;34: 330–338. doi:10.1016/j.arteri.2022.03.004	No	Not meeting inclusion criteria	Title and abstract screening
1995	Gomez M, Jeudy J, Petit A, Brunet J, Saint-Cast Y, Rabarin F, et al. Acute pain after digital crushing and nicotine intoxication. Cigarettes are always a false friend. A prospective study. Orthop Traumatol Surg Res. 2022; 103425. doi:10.1016/j.otsr.2022.103425	No	Not meeting inclusion criteria	Title and abstract screening
1996	Gomez M, Jeudy J, Petit A, Brunet J, Saint-Cast Y, Rabarin F, et al. Acute pain after digital crushing and nicotine intoxication. Cigarettes are always a false friend. A prospective study. Orthop Traumatol Surg Res. 2023;109: 103425. doi:10.1016/j.otsr.2022.103425	No	Not meeting inclusion criteria	Title and abstract screening
1997	Gomez Y, Creamer M, Trivers KF, Anic G, Morse AL, Reissig C, et al. Patterns of tobacco use and nicotine dependence among youth, United States, 2017-2018. Prev Med. 2020;141: 106284. doi:10.1016/j.ypmed.2020.106284	No	Not meeting inclusion criteria	Title and abstract screening
1998	Goniewicz ML. Biomarkers of Electronic Nicotine Delivery Systems (ENDS) use. Addict Neurosci. 2023;6. doi:10.1016/j.addicn.2023.100077	No	Not meeting inclusion criteria	Title and abstract screening
1999	Goniewicz ML, Stanton CA. Impact of flavour restricting policies on non-cigarette tobacco products. Tob Control. 2022;31: s159–s160. doi:10.1136/tc-2022-057785	No	Not meeting inclusion criteria	Title and abstract screening
2000	González Roz A, Secades Villa R, Weidberg S. Evaluating nicotine dependence levels in e-cigarette users. Adicciones. 2017;29: 136–138. doi:10.20882/adicciones.905	No	Not meeting inclusion criteria	Title and abstract screening
2001	Gonzalez-Santiago S, Lopez-Miranda E, Escriva-De-Romani S, Jimenez-Rodriguez B, Antolin-Novoa S, Gonzalez-Cortijo L, et al. Time and motion randomised study of a subcutaneous (SC) pertuzumab and trastuzumab fixed-dose combination (PH FDC) for the treatment of HER2-positive early breast cancer (HER2 EBC): pHaTiMa. 2023;8. doi:10.1016/j.esmoop.2023.101340	No	Not meeting inclusion criteria	Title and abstract screening
2002	Goodman RE, Thordsen SE, Sridhar N, Calderon A, Weil E, Chaudhary LN, et al. CHANGE IN LEFT VENTRICULAR END DIASTOLIC INDEX OR GLOBAL LONGITUDINAL STRAIN MAY PREDICT DEVELOPMENT OF CANCER THERAPY RELATED CARDIAC DYSFUNCTION IN HER2 POSITIVE BREAST CANCER PATIENTS RECEIVING TRASTUZUMAB. J Am Coll Cardiol JACC. 2022;79: 1887–1887. doi:10.1016/S0735-1097(22)02878-9	No	Not meeting inclusion criteria	Title and abstract screening
2003	Goodney PP, Spangler EL, Newhall K, Brooke BS, Schanzer A, Tan TW, et al. Feasibility and pilot efficacy of a brief smoking cessation intervention delivered by vascular surgeons in the Vascular Physician Offer and Report (VAPOR) Trial. J Vasc Surg. 2017;65: 1152-1160.e2. doi:10.1016/j.jvs.2016.10.121	No	Not meeting inclusion criteria	Title and abstract screening
2004	Goodwin R.D. Impact of Cannabis Use on Nicotine and Tobacco Use Outcomes. Nicotine Tob Res. 2021;22: 1257–1259. doi:10.1093/ntr/ntaa096	No	Not meeting inclusion criteria	Title and abstract screening
2005	Goon S., Slotnick M., Leung C. Subjective Social Status and Its Relationship To Diet and Health Behavior Among Students at a Large, Midwestern University. Curr Dev Nutr. 2023;7: 100344. doi:10.1016/j.cdnut.2023.100344	No	Not meeting inclusion criteria	Title and abstract screening
2006	Gorbett DM. Development of a measure assessing perceptions of e-cigarettes: A mixed methods approach. Diss Abstr Int Sect B Sci Eng. 2022;83: No-Specified.	No	Not meeting inclusion criteria	Title and abstract screening
2007	Gordon D. SESSION 1: SUBSTANCE USE IN ADOLESCENCE, PERFORMANCE AND IMAGE-ENHANCING DRUGS (PIEDS) AND NICOTINE DEPENDENCE AND VAPING: SUBSTANCE USE IN ADOLESCENCE. Aust N Z J Psychiatry. 2022;56: 30. doi:10.1177/00048674221088686	No	Not meeting inclusion criteria	Title and abstract screening
2008	Gordon J.S., Bell M.L., Armin J.S., Giacobbi P.R., Nair U.S. A telephone-based guided imagery tobacco cessation intervention: Results of a randomized feasibility trial. Transl Behav Med. 2021;11: 516–529. doi:10.1093/tbm/ibaa052	No	Not meeting inclusion criteria	Title and abstract screening
2009	Gordon LG, Preston P. Healthcare costs attributable to e-cigarette use and subsequent uptake of cigarette smoking by Australians who have never smoked. Aust Health Rev Publ Aust Hosp Assoc. 2023. doi:10.1071/AH23178	No	Not meeting inclusion criteria	Title and abstract screening
2010	Gordon T., Fine J. Cornering the suspects in vaping-associated EVALI. N Engl J Med. 2020;382: 755–756. doi:10.1056/NEJMe2001065	No	Not meeting inclusion criteria	Title and abstract screening
2011	Gordon T, Karey E, Rebuli ME, Escobar Y-NH, Jaspers I, Chen LC. E-Cigarette Toxicology. Annu Rev Pharmacol Toxicol. 2022;62: 301–322. doi:10.1146/annurev-pharmtox-042921-084202	No	Not meeting inclusion criteria	Title and abstract screening

2012	Górski P. E-cigarettes or heat-not-burn tobacco products - advantages or disadvantages for the lungs of smokers. Adv Respir Med. 2019;87: 123–134. doi:10.5603/ARM.2019.0020	No	Not meeting inclusion criteria	Title and abstract screening
2013	Gorukanti A, Delucchi K, Ling P, Fisher-Travis R, Halpern-Felsher B. Adolescents' attitudes towards e-cigarette ingredients, safety, addictive properties, social norms, and regulation. Prev Med. 2017;94: 65–71. doi:10.1016/j.ypmed.2016.10.019	No	Not meeting inclusion criteria	Title and abstract screening
2014	Goswami P. Impact of advance care planning and end-of-life conversations on patients with cancer: An integrative review of literature. J Nurs Scholarsh. 2023;55: 272–290. doi:10.1111/jnu.12804	No	Not meeting inclusion criteria	Title and abstract screening
2015	Gotanda H, Nuckols TK, Lauzon M, Tsugawa Y. Comparison of Advance Care Planning and End-of-Life Care Intensity Between Dementia Versus Cancer Patients. JGIM J Gen Intern Med. 2022;37: 3251–3257. doi:10.1007/s11606-021-07330-2	No	Not meeting inclusion criteria	Title and abstract screening
2016	Gottlieb MA. E-Cigarettes versus Nicotine-Replacement Therapy for Smoking Cessation. N Engl J Med. 2019;380: 1974. doi:10.1056/NEJMc1903758	No	Not meeting inclusion criteria	Title and abstract screening
2017	Gottlieb MA. Regulation of E-Cigarettes in the United States and Its Role in a Youth Epidemic. Child Basel Switz. 2019;6. doi:10.3390/children6030040	No	Not meeting inclusion criteria	Title and abstract screening
2018	Gould GS, Hurst JR, Trofor A, Alison JA, Fox G, Kulkarni MM, et al. Recognising the importance of chronic lung disease: a consensus statement from the Global Alliance for Chronic Diseases (Lung Diseases group). Respir Res. 2023;24: 15. doi:10.1186/s12931-022-02297-y	No	Not meeting inclusion criteria	Title and abstract screening
2019	Gouldthorpe C, Power J, Taylor A, Davies A. Specialist Palliative Care for Patients with Cancer: More Than End-of-Life Care. Cancers. 2023;15: 3551. doi:10.3390/cancers15143551	No	Not meeting inclusion criteria	Title and abstract screening
2020	Goulette M., Schlienz N.J., Case A., Hansen E., Ashare R.L., Goniewicz M., et al. Self-reported knowledge of DELTA9-tetrahydrocannabinol (THC) and cannabidiol (CBD) potency in cannabis products among cancer patients and survivors: Results from a survey of cannabis consumers at an NCIdesignated cancer center. Cancer Res. 2023;83. doi:10.1158/1538-7445.AM2023-6472	No	Not meeting inclusion criteria	Title and abstract screening
2021	Goulette MR, Gravely S, Xu SS, Meng G, Quah ACK, Lee S, et al. Perceptions of harmfulness of heated tobacco and nicotine vaping products compared to cigarettes, and the association of advertising exposure on harm perceptions among adults who smoke in South Korea: Cross-sectional findings from the 2020 ITC Korea Surve. Tob Induc Dis. 2023;21: 121. doi:10.18332/tid/170252	No	Not meeting inclusion criteria	Title and abstract screening
2022	Gouse B.M., Nieves-Archibald A., Trutzer I., Rezvani M., Srinath M., Chang A., et al. Pediatric Malignant Catatonia Associated With Vaporized Cannabis Use: A Case Series. J Acad Consult-Liaison Psychiatry. 2021;62: 445–448. doi:10.1016/j.jaclp.2021.02.004	No	Not meeting inclusion criteria	Title and abstract screening
2023	Graham AL, Amato MS, Cha S, Jacobs MA, Bottcher MM, Papandonatos GD. Effectiveness of a Vaping Cessation Text Message Program Among Young Adult e-Cigarette Users: A Randomized Clinical Trial. JAMA Intern Med. 2021;181: 923–930. doi:10.1001/jamainternmed.2021.1793	No	Not meeting inclusion criteria	Title and abstract screening
2024	Graham AL, Burke MV, Jacobs MA, Cha S, Croghan IT, Schroeder DR, et al. An integrated digital/clinical approach to smoking cessation in lung cancer screening: study protocol for a randomized controlled trial. Trials. 2017;18: 568. doi:10.1186/s13063-017-2312-x	No	Not meeting inclusion criteria	Title and abstract screening
2025	Graham AL, Cha S, Papandonatos GD, Amato MS, Jacobs MA, Abroms LC, et al. E-cigarette and combusted tobacco abstinence among young adults: Secondary analyses from a U.S.-based randomized controlled trial of vaping cessation. Prev Med. 2022; 107119. doi:10.1016/j.ypmed.2022.107119	No	Not meeting inclusion criteria	Title and abstract screening
2026	Graham E., McCaig L., Lau G.S.-K., Tejura A., Cao A., Zuo Y.Y., et al. E-cigarette aerosol exposure of pulmonary surfactant impairs its surface tension reducing function. bioRxiv. 2022. doi:10.1101/2022.08.16.501319	No	Not meeting inclusion criteria	Title and abstract screening
2027	GRAHAM J.M., ALI M.S. ACUTE EOSINOPHILIC PNEUMONIA AFTER COVID-19. Chest. 2023;164: A3195–A3196. doi:10.1016/j.chest.2023.07.2094	No	Not meeting inclusion criteria	Title and abstract screening
2028	Graham R, Bharthi K, Williams J, Sharifi V, Pedram P, Fahim M, et al. Trends in vaping and smoking behavior before and during the COVID-19 pandemic in Canada: Beneficial and potentially detrimental changes. Addict Behav. 2024;149: 107839. doi:10.1016/j.addbeh.2023.107839	No	Not meeting inclusion criteria	Title and abstract screening
2029	Grant JE, Lust K, Fridberg DJ, King AC, Chamberlain SR. E-cigarette use (vaping) is associated with illicit drug use, mental health problems, and impulsivity in university students. Ann Clin Psychiatry. 2019;31: 27–35.	No	Not meeting inclusion criteria	Title and abstract screening
2030	Grassi M., Citla-Sridhar D., Mason K.K., Mack J.M. Disseminated fungal infection and marijuana use in a pediatric cancer patient. J Investig Med. 2022;70: 549–550. doi:10.1136/jim-2022-SRMC.198	No	Not meeting inclusion criteria	Title and abstract screening
2031	Gravely S, Chung-Hall J, Craig LV, Fong GT, Cummings KM, Borland R, et al. Evaluating the impact of plain packaging among Canadian smokers: findings from the 2018 and 2020 ITC Smoking and Vaping Surveys. Tob Control. 2021. doi:10.1136/tobaccocontrol-2021-056635	No	Not meeting inclusion criteria	Title and abstract screening
2032	Gravely S, Chung-Hall J, Craig LV, Fong GT, Cummings KM, Borland R, et al. Evaluating the impact of plain packaging among Canadian smokers: findings from the 2018 and 2020 ITC Smoking and Vaping Surveys. Tob Control. 2023;32: 153–162. doi:10.1136/tobaccocontrol-2021-056635	No	Not meeting inclusion criteria	Title and abstract screening
2033	Gravely S, Craig LV, Cummings KM, Ouimet J, Loewen R, Martin N, et al. Smokers' cognitive and behavioural reactions during the early phase of the COVID-19 pandemic: Findings from the 2020 ITC Four Country Smoking and Vaping Survey. PLoS One. 2021;16: e0252427. doi:10.1371/journal.pone.0252427	No	Not meeting inclusion criteria	Title and abstract screening
2034	Gravely S, Cummings KM, Hammond D, Borland R, McNeill A, East KA, et al. Self-Reported Quit Aids and Assistance Used By Smokers At Their Most Recent Quit Attempt: Findings from the 2020 International Tobacco Control Four Country Smoking and Vaping Survey. Nicotine Tob Res. 2021;23: 1699–1707. doi:10.1093/ntr/ntab068	No	Not meeting inclusion criteria	Title and abstract screening
2035	Gravely S, Driezen P, McClure EA, Hammond D, Michael Cummings K, Chan G, et al. Corrigendum to "Differences between adults who smoke cigarettes daily and do and do not co-use cannabis: Findings from the 2020 ITC four country smoking and vaping survey" [Addict. Behav. 135 (2022) 107434]. Addict Behav. 2022;137: 107529. doi:10.1016/j.addbeh.2022.107529	No	Not meeting inclusion criteria	Title and abstract screening
2036	Gravely S, Driezen P, McClure EA, Hammond D, Michael Cummings K, Chan G, et al. Differences between adults who smoke cigarettes daily and do and do not co-use cannabis: Findings from the 2020 ITC four country smoking and vaping survey. Addict Behav. 2022;135: 107434. doi:10.1016/j.addbeh.2022.107434	No	Not meeting inclusion criteria	Title and abstract screening
2037	Gravely S, Meng G, Hammond D, Hyland A, Michael Cummings K, Borland R, et al. Differences in cigarette smoking quit attempts and cessation between adults who did and did not take up nicotine vaping: Findings from the ITC four country smoking and vaping surveys. Addict Behav. 2022;132: 107339. doi:10.1016/j.addbeh.2022.107339	No	Not meeting inclusion criteria	Title and abstract screening
2038	Gravely S, Meng G, Hammond D, Reid JL, Seo YS, Hyland A, et al. Electronic nicotine delivery systems (ENDS) flavours and devices used by adults before and after the 2020 US FDA ENDS enforcement priority: findings from the 2018 and 2020 US ITC Smoking and Vaping Surveys. Tob Control. 2022;31: s167–s175. doi:10.1136/tobaccocontrol-2022-057445	No	Not meeting inclusion criteria	Title and abstract screening
2039	Gravely S, Meng G, Hammond D, Driezen P, Thrasher JF, Fong GT, et al. Support for pictorial health warning labels on cigarette packages in the United States among adults who currently smoke or quit smoking: Findings from the ITC US Smoking and Vaping Surveys. Tob Induc Dis. 2023;21: 84. doi:10.18332/tid/166001	No	Not meeting inclusion criteria	Title and abstract screening
2040	Gravely S, Smith DM, Liber AC, Cummings KM, East KA, Hammond D, et al. Responses to potential nicotine vaping product flavor restrictions among regular vapers using non-tobacco flavors: Findings from the 2020 ITC Smoking and Vaping Survey in Canada, England and the United States. Addict Behav. 2022;125: 107152. doi:10.1016/j.addbeh.2021.107152	No	Not meeting inclusion criteria	Title and abstract screening

2041	Gravely S, Yong HH, Reid JL, East KA, Gartner CE, Levy DT, et al. Do Current Smokers and Ex-Smokers Who Use Nicotine Vaping Products Daily Versus Weekly Differ on Their Reasons for Vaping? Findings from the 2020 ITC Four Country Smoking and Vaping Survey. Int J Env Res Public Health. 2022;19. doi:10.3390/ijerph192114130	No	Not meeting inclusion criteria	Title and abstract screening
2042	Gravely S, Yong HH, Reid JL, East KA, Liber AC, Michael Cummings K, et al. An examination of quitting smoking as a reason for vaping by the type of nicotine vaping device used most often among adults who smoke and vape: Findings from the Canada, England and the United States 2020 ITC Smoking and Vaping Survey. Prev Med Rep. 2023;33: 102201. doi:10.1016/j.pmedr.2023.102201	No	Not meeting inclusion criteria	Title and abstract screening
2043	Gravely S., Driezen P., McClure E.A., Hammond D., Michael Cummings K., Chan G., et al. Corrigendum to "Differences between adults who smoke cigarettes daily and do and do not co-use cannabis: Findings from the 2020 ITC four country smoking and vaping survey" [Addict. Behav. 135 (2022) 107434] (Addictive Behaviors (2022) 135, (S0306460322002. Addict Behav. 2023;137: 107529. doi:10.1016/j.addbeh.2022.107529	No	Not meeting inclusion criteria	Title and abstract screening
2044	Gravely S, Driezen P, McClure EA, Smith DM, Fong GT. Prevalence of depressive symptoms and cannabis use among adult cigarette smokers in Canada: cross-sectional findings from the 2020 International Tobacco Control Policy Evaluation Project Canada Smoking and Vaping Survey. CMAJ Open. 2023;11: E516–E526. doi:10.9778/cmajo.20220081	No	Not meeting inclusion criteria	Title and abstract screening
2045	Gravely S, Driezen P, McClure EA, Hammond D, Michael Cummings K, Chan G, et al. Corrigendum to "Differences between adults who smoke cigarettes daily and do and do not co-use cannabis: Findings from the 2020 ITC four country smoking and vaping survey" [Addict. Behav. 135 (2022) 107434]. Addict Behav. 2023;137: N.PAG-N.PAG. doi:10.1016/j.addbeh.2022.107529	No	Not meeting inclusion criteria	Title and abstract screening
2046	Gray TF, Plotke R, Heuer L, Topping CE, Nipp RD, Wang AC, et al. Perceptions of prognosis and end-of-life care outcomes in patients with advanced lung and gastrointestinal cancer. Palliat Med. 2023;37: 740–748. doi:10.1177/02692163231155511	No	Not meeting inclusion criteria	Title and abstract screening
2047	Green AC, Driezen P, Noar SM, Hammond D, Fong GT. Impact of adding and removing warning label messages from cigarette packages on adult smokers' awareness about the health harms of smoking: findings from the ITC Canada Survey. Tob Control Int J. 2019;28: e56–e63. doi:10.1136/tobaccocontrol-2018-054885https://dx.doi.org/10.1136/tobaccocontrol-2018-054885	No	Not meeting inclusion criteria	Title and abstract screening
2048	Green D.B., Restrepo C.S., Legasto A.C., Bang T.J., Oh A.S., Vargas D. Imaging of the rare cystic lung diseases. Curr Probl Diagn Radiol. 2022;51: 648–658. doi:10.1067/j.cpradiol.2021.02.003	No	Not meeting inclusion criteria	Title and abstract screening
2049	Green MJ, Maddock J, Di Gessa G, Wielgoszewska B, Parsons S, Griffith GJ, et al. The UK Coronavirus Job Retention Scheme and smoking, alcohol consumption and vaping during the COVID-19 pandemic: evidence from eight longitudinal population surveys. BMC Med. 2022;20: 345. doi:10.1186/s12916-022-02511-0	No	Not meeting inclusion criteria	Title and abstract screening
2050	Green R, Ray LA. Effects of varenicline on subjective craving and relative reinforcing value of cigarettes. Drug Alcohol Depend. 2018;188: 53–59. doi:10.1016/j.drugalcdep.2018.03.037	No	Not meeting inclusion criteria	Title and abstract screening
2051	Greener M. Latest clinical research. Indep Nurse. 2022;2022: 12–13. doi:10.12968/indn.2022.2.12	No	Not meeting inclusion criteria	Title and abstract screening
2052	Gregory A., Xu Z., Pratte K., Berman S., Lichtblau N., Lu R., et al. Prediction of Electronic Nicotine Delivery Systems Use in COPDGene Using Multi-Omic Biomarkers. Am J Respir Crit Care Med. 2022;205. doi:10.1164/ajrccm-conference.2022.205.1_MeetingAbstracts.A3268	No	Not meeting inclusion criteria	Title and abstract screening
2053	Gribben V., Klein J.D. Practical Implications of the US Preventive Services Task Force Recommendations on Adolescents and Tobacco. J Adolesc Health. 2020;67: 328–330. doi:10.1016/j.jadohealth.2020.06.005	No	Not meeting inclusion criteria	Title and abstract screening
2054	Griffin S, DeFoor MG, Bail JR, Prevost S. Commentary on "Increased nicotine vaping due to the COVID-19 pandemic among US young adults: Associations with nicotine dependence, vaping frequency, and reasons for use". Prev Med. 2023;169: 107410. doi:10.1016/j.ypmed.2022.107410	No	Not meeting inclusion criteria	Title and abstract screening
2055	Grigg J. Smoking, nicotine, and COVID-19. Lancet Respir Med. 2022;10: 818–819. doi:10.1016/S2213-2600%2822%2900258-2	No	Not meeting inclusion criteria	Title and abstract screening
2056	Grigsby TJ. Development and psychometric properties of the tobacco and nicotine consequences scale (TANCS) to screen for cigarette and e-cigarette misuse in community settings. Addict Behav. 2019;98: 106058. doi:10.1016/j.addbeh.2019.106058	No	Not meeting inclusion criteria	Title and abstract screening
2057	Grigson P.S. Addiction: A multi-determined chronic disease. Brain Res Bull. 2018;138: 1–4. doi:10.1016/j.brainresbull.2017.11.019	No	Not meeting inclusion criteria	Title and abstract screening
2058	Grill K. E-cigarettes: The long-term liberal perspective. Nicotine Tob Res. 2021;23: 9–13. doi:10.1093/ntr/ntaa085	No	Not meeting inclusion criteria	Title and abstract screening
2059	Grondin CJ, Davis AP, Wiegers JA, Wiegers TC, Sciaky D, Johnson RJ, et al. Predicting molecular mechanisms, pathways, and health outcomes induced by Juul e-cigarette aerosol chemicals using the Comparative Toxicogenomics Database. Curr Res Toxicol. 2021;2: 272–281. doi:10.1016/j.crtox.2021.08.001	No	Not meeting inclusion criteria	Title and abstract screening
2060	Groner J. Health effects of electronic cigarettes. Curr Probl Pediatr Adolesc Health Care. 2022;52: 101202. doi:10.1016/j.cppeds.2022.101202	No	Not meeting inclusion criteria	Title and abstract screening
2061	Grossenbacher F., Cazaubon Y., Medhioub Y., Plenier Y., Duliere L., Marty H., et al. E-liquid sold as CBD e-liquid containing XRL-11: 4 cases reported. A warning signal to health authorities and e-cigarettes users. Clin Toxicol. 2021;59: 550. doi:10.1080/15563650.2021.1906080	No	Not meeting inclusion criteria	Title and abstract screening
2062	Grummon AH, Hall MG, Mitchell CG, Pulido M, Mendel Sheldon J, Noar SM, et al. Reactions to messages about smoking, vaping and COVID-19: two national experiments. Tob Control. 2022;31: 402–410. doi:10.1136/tobaccocontrol-2020-055956	No	Not meeting inclusion criteria	Title and abstract screening
2063	Guadagnolo BA. Preferences for More Aggressive End-of-life Pharmacologic Care Among Racial Minorities in a Large Population-Based Cohort of Cancer Patients. J Pain Symptom Manage. 2022;63: e268–e270. doi:10.1016/j.jpainsymman.2021.11.001	No	Not meeting inclusion criteria	Title and abstract screening
2064	Guarin GE, Dee EC, Robredo JPG, Eala MAB, Medina Jr. MF, Tanco KC. End-of-life care for Filipino patients with cancer. Palliat Support Care. 2023;21: 365–369. doi:10.1017/S1478951522001183	No	Not meeting inclusion criteria	Title and abstract screening
2065	Gubner NR, Guydish J, Humfleet GL, Benowitz NL, Hall SM. Nicotine biomarkers and rate of nicotine metabolism among cigarette smokers taking buprenorphine for opioid dependency. Drug Alcohol Depend. 2017;178: 267–270. doi:10.1016/j.drugalcdep.2017.05.020	No	Not meeting inclusion criteria	Title and abstract screening
2066	Gubner NR, Williams DD, Le T, Garcia W, Vijayaraghavan M, Guydish J. Smoking related outcomes before and after implementation of tobacco-free grounds in residential substance use disorder treatment programs. Drug Alcohol Depend. 2019;197: 8–14. doi:10.1016/j.drugalcdep.2019.01.001	No	Not meeting inclusion criteria	Title and abstract screening
2067	Gubner NR, Williams DD, Pagano A, Campbell BK, Guydish J. Menthol cigarette smoking among individuals in treatment for substance use disorders. Addict Behav. 2018;80: 135–141. doi:10.1016/j.addbeh.2018.01.015	No	Not meeting inclusion criteria	Title and abstract screening
2068	Gucht DV, Adriaens K, Baeyens F. Online Vape Shop Customers Who Use E-Cigarettes Report Abstinence from Smoking and Improved Quality of Life, But a Substantial Minority Still Have Vaping-Related Health Concerns. Int J Env Res Public Health. 2017;14. doi:10.3390/ijerph14070798	No	Not meeting inclusion criteria	Title and abstract screening
2069	Gueorguieva R, Buta E, Morean M, Krishnan-Sarin S. Two-part models for repeatedly measured ordinal data with "don't know" category. Stat Med. 2020;39: 4574–4592. doi:10.1002/sim.8739	No	Not meeting inclusion criteria	Title and abstract screening

2070	Guerrero A.M., Luna Diaz L.V., Balestrini K.C., Calderon Candelario R.A., Holt G.E., Mirsaeidi M., et al. Vaping education improves success rate for replacement of tobacco cigarettes with electronic cigarettes. Am J Respir Crit Care Med. 2019;199. Available: https://www.atsjournals.org/doi/abs/10.1164/ajrccm-conference.2019.199.1_MeetingAbstracts.A3010	No	Not meeting inclusion criteria	Title and abstract screening
2071	Guerrero A.M., Schweitzer M.D., Balestrini K., Luna Diaz L.V., Holt G., Mirsaeidi M., et al. Vaping characteristics of veterans replacing tobacco smoking with electronic cigarettes. Am J Respir Crit Care Med. 2017;195. doi:10.1164/ajrccm-conference.2017.D102	No	Not meeting inclusion criteria	Title and abstract screening
2072	Guerrero-Cignarella A, Luna Diaz LV, Balestrini K, Holt G, Mirsaeidi M, Calderon-Candelario R, et al. Differences in vaping topography in relation to adherence to exclusive electronic cigarette use in veterans. PloS One. 2018;13: e0195896. doi:10.1371/journal.pone.0195896	No	Not meeting inclusion criteria	Title and abstract screening
2073	Guevara A., Wang Z., Caldwell J.L., Tapa S., Ngo L., I-Ju Lee E., et al. B-PO04-010 THE EFFECT OF CHRONIC NICOTINE EXPOSURE ON CARDIAC ELECTROPHYSIOLOGY IN THE RABBIT HEART. Heart Rhythm. 2021;18: S283–S284. doi:10.1016/j.hrthm.2021.06.707	No	Not meeting inclusion criteria	Title and abstract screening
2074	Guevara A.M., Caldwell J., Tapa S., Ngo L., Lee I.-J.E., Wang Z., et al. BS-400-49 THE EFFECT OF CHRONIC NICOTINE EXPOSURE ON CARDIAC ELECTROPHYSIOLOGY IN THE RABBIT HEART. Heart Rhythm. 2022;19: S508–S509. doi:10.1016/j.hrthm.2022.03.1197	No	Not meeting inclusion criteria	Title and abstract screening
2075	Guez G., Ewen R., Shah F., Patel B. BILATERAL SPONTANEOUS PNEUMOTHORAXES ASSOCIATED WITH VAPING. Chest. 2021;160: A2444. doi:10.1016/j.chest.2021.07.2111	No	Not meeting inclusion criteria	Title and abstract screening
2076	Gugala E, Okoh CM, Ghosh S, Moczygemba LR. Pulmonary Health Effects of Electronic Cigarettes: A Scoping Review. Health Promot Pr. 2022;23: 388–396. doi:10.1177/1524839920985506	No	Not meeting inclusion criteria	Title and abstract screening
2077	Guillaumier A, Manning V, Wynne O, Gartner C, Borland R, Baker AL, et al. Electronic nicotine devices to aid smoking cessation by alcohol- and drug-dependent clients: protocol for a pilot randomised controlled trial. Trials. 2018;19: 415. doi:10.1186/s13063-018-2786-1	No	Not meeting inclusion criteria	Title and abstract screening
2078	Guillaumin C, Urban T. [The fight against smoking. The need to consider behavioral dependence]. Rev Pneumol Clin. 2017;73: 294–298. doi:10.1016/j.pneumo.2017.09.005	No	Not meeting inclusion criteria	Title and abstract screening
2079	Gul F, Khan AA, Kazmi SNH, Abbas K, Basit J. Vaping, an emerging public health concern in South Asia: a short communication. Ann Med Surg 2012. 2023;85: 2270–2272. doi:10.1097/MS9.0000000000000297	No	Not meeting inclusion criteria	Title and abstract screening
2080	Gulati G.K., Hinds B.J. Smoking cessation potential of smartphone-assisted behavioral therapy coupled to programmable carbon nanotube membrane nicotine delivery device. Crit Rev Ther Drug Carrier Syst. 2018;35: 495–520. doi:10.1615/CritRevTherDrugCarrierSyst.2018020331	No	Not meeting inclusion criteria	Title and abstract screening
2081	Gullapalli D, Vangara A, Ganti SS, Kommineni SS, Rahmlow T, Moon J. Gilchrist's Hollow Lung. Cureus. 2023;15: e44288. doi:10.7759/cureus.44288	No	Not meeting inclusion criteria	Title and abstract screening
2082	Gultzow T., Smit E.S., Crutzen R., Jolani S., Hoving C., Dirksen C.D. Effects of an Explicit Value Clarification Method With Computer-Tailored Advice on the Effectiveness of a Web-Based Smoking Cessation Decision Aid: Findings From a Randomized Controlled Trial. J Med Internet Res. 2022;24: e34246. doi:10.2196/34246	No	Not meeting inclusion criteria	Title and abstract screening
2083	Gunda V, Chhonker YS, Natesh NS, Raut P, Muniyan S, Wyatt TA, et al. Nuclear factor kappa-B contributes to cigarette smoke tolerance in pancreatic ductal adenocarcinoma through cysteine metabolism. Biomed Pharmacother. 2021;144: 112312. doi:10.1016/j.biopha.2021.112312	No	Not meeting inclusion criteria	Title and abstract screening
2084	Gunge D, Marganski J, Advani I, Boddu S, Chen YJE, Mehta S, et al. Deleterious Association of Inhalant Use on Sleep Quality during the COVID-19 Pandemic. Int J Env Res Public Health. 2021;18. doi:10.3390/ijerph182413203	No	Not meeting inclusion criteria	Title and abstract screening
2085	Guo J, Hecht SS. DNA damage in human oral cells induced by use of e-cigarettes. Drug Test Anal. 2022. doi:10.1002/dta.3375	No	Not meeting inclusion criteria	Title and abstract screening
2086	Guo J, Hecht SS. DNA damage in human oral cells induced by use of e-cigarettes. Drug Test Anal. 2023;15: 1189–1197. doi:10.1002/dta.3375	No	Not meeting inclusion criteria	Title and abstract screening
2087	Guo W, Yu JZ, Chan W. Face Mask as a Versatile Sampling Device for the Assessment of Personal Exposure to 54 Toxic Compounds in Environmental Tobacco Smoke. Chem Res Toxicol. 2023;36: 1140–1150. doi:10.1021/acs.chemrestox.3c00114	No	Not meeting inclusion criteria	Title and abstract screening
2088	Guo X, Chan YC, Gautam T, Zhao R. Autoxidation of glycols used in inhalable daily products: implications for the use of artificial fogs and e-cigarettes. Env Sci Process Impacts. 2023;25: 1657–1669. doi:10.1039/d3em00214d	No	Not meeting inclusion criteria	Title and abstract screening
2089	Gupta AK, Mehrotra R. Increasing use of flavoured tobacco products amongst youth. Indian J Tuberc. 2021;68S: S105–S107. doi:10.1016/j.ijtb.2021.07.020	No	Not meeting inclusion criteria	Title and abstract screening
2090	Gupta AK, Mehrotra R. Safety Concerns for Tobacco-Free Products Containing Synthetic Nicotine. Nicotine Tob Res. 2021;23: 1980–1981. doi:10.1093/ntr/ntab071	No	Not meeting inclusion criteria	Title and abstract screening
2091	Gupta B.M., Ahmed K.K.M., Visakhi P. E-Cigarettes: A scientometric assessment of global publications output during 2001-18. J Young Pharm. 2020;12: 29–36. doi:10.5530/JYP.2020.12.7	No	Not meeting inclusion criteria	Title and abstract screening
2092	Gupta K, Caputo S, Casado C, Jang A, Sweeney PL, Lanka SM, et al. Characterization of ctDNA findings at the end of life in patients with prostate cancer. J Clin Oncol. 2023;41: 230–230. doi:10.1200/JCO.2023.41.6_suppl.230	No	Not meeting inclusion criteria	Title and abstract screening
2093	Gupta PC, Pednekar MS, Narake S, Puntambekar N, Mc Carthy WJ, Mistry R. Awareness and Use of e-cigarettes among Adolescents and Their Adult Caregivers in Two Cities of India. Asian Pac J Cancer Prev APJCP. 2023;24: 2195–2197. doi:10.31557/APJCP.2023.24.7.2195	No	Not meeting inclusion criteria	Title and abstract screening
2094	Gupta R, Lin Y, Luna K, Logue A, Yoon AJ, Haptonstall KP, et al. Electronic and Tobacco Cigarettes Alter Polyunsaturated Fatty Acids and Oxidative Biomarkers. Circ Res. 2021;129: 514–526. doi:10.1161/CIRCRESAHA.120.317828	No	Not meeting inclusion criteria	Title and abstract screening
2095	Gupta R, van Dongen J, Fu Y, Abdellaoui A, Tyndale RF, Velagapudi V, et al. Epigenome-wide association study of serum cotinine in current smokers reveals novel genetically driven loci. Clin Epigenetics. 2019;11: 1. doi:10.1186/s13148-018-0606-9	No	Not meeting inclusion criteria	Title and abstract screening
2096	Gupte HA, Chatterjee N, Mandal G, D'Costa M. Adolescents and E-cigarettes in India: A Qualitative Study of Perceptions and Practices. Asian Pac J Cancer Prev. 2022;23: 2991–2997. doi:10.31557/APJCP.2022.23.9.2991	No	Not meeting inclusion criteria	Title and abstract screening
2097	Gurram N, Thomson G, Wilson N, Hoek J. Electronic cigarette online marketing by New Zealand vendors. N Z Med J. 2019;132: 20–33.	No	Not meeting inclusion criteria	Title and abstract screening
2098	Gutierrez A, Nguyen JD, Creehan KM, Javadi-Paydar M, Grant Y, Taffe MA. Effects of combined THC and heroin vapor inhalation in rats. Psychopharmacol Berl. 2022;239: 1321–1335. doi:10.1007/s00213-021-05904-w	No	Not meeting inclusion criteria	Title and abstract screening

2099	Gutierrez A, Taffe MA. Rats chasing the dragon: A new heroin inhalation method. J Neurosci Methods. 2023;402: 110013. doi:10.1016/j.jneumeth.2023.110013	No	Not meeting inclusion criteria	Title and abstract screening
2100	Gutsche J.T., Grant M.C., Kiefer J.J., Ghadimi K., Lane-Fall M.B., Mazzeffi M.A. The Year in Cardiothoracic Critical Care: Selected Highlights from 2019. J Cardiothorac Vasc Anesth. 2022;36: 45–57. doi:10.1053/j.jvca.2020.09.114	No	Not meeting inclusion criteria	Title and abstract screening
2101	Guttentag A, Tseng TY, Shelley D, Kirchner T. Analyzing Trajectories of Acute Cigarette Reduction Post-Introduction of an E-Cigarette Using Ecological Momentary Assessment Data. Int J Env Res Public Health. 2022;19. doi:10.3390/ijerph19127452	No	Not meeting inclusion criteria	Title and abstract screening
2102	Guy EFS, Knopp JL, Lerios T, Chase JG. Airflow and dynamic circumference of abdomen and thorax for adults at varied continuous positive airway pressure ventilation settings and breath rates. Sci Data. 2023;10: 481. doi:10.1038/s41597-023-02326-5	No	Not meeting inclusion criteria	Title and abstract screening
2103	Guydish J, Kapiteni K, Le T, Campbell B, Pinsker E, Delucchi K. Tobacco use and tobacco services in California substance use treatment programs. Drug Alcohol Depend. 2020;214: 108173. doi:10.1016/j.drugalcdep.2020.108173	No	Not meeting inclusion criteria	Title and abstract screening
2104	Guzman I.A., Kramer M.R., Kancherla V. Association between maternal prepregnancy diabetes mellitus and preconception folic acid supplement use by Hispanic ethnicity: Findings from Georgia, New York City, and Puerto Rico PRAMS 2016-2018. Birth Defects Res. 2022;114: 873–884. doi:10.1002/bdr2.2078	No	Not meeting inclusion criteria	Title and abstract screening
2105	Guzmán Y, Ríos J, Paredes J, Domínguez P, Maurel J, González-Abós C, et al. Time Interval Between the End of Neoadjuvant Therapy and Elective Resection of Locally Advanced Rectal Cancer in the CRONOS Study. JAMA Surg. 2023;158: 910–919. doi:10.1001/jamasurg.2023.2521	No	Not meeting inclusion criteria	Title and abstract screening
2106	Gwon SH, Lee H-J, Brian Ahn H. Transcranial Direct Current Stimulation in Nicotine Use: Nursing Implications for Patient Outcomes. J Addict Nurs. 2023;34: E74–E78. doi:10.1097/JAN.0000000000000542	No	Not meeting inclusion criteria	Title and abstract screening
2107	Ha D., Zeng C., Steiner J.F. Physical Activity, Sedentary, and Smoking Behaviors of a Cohort of Individuals at High Risk for Lung Cancer. Am J Respir Crit Care Med. 2021;203. doi:10.1164/ajrccm-conference.2021.TP136	No	Not meeting inclusion criteria	Title and abstract screening
2108	Ha G., Matsuda B.J. Lipoid Pneumonia as a Long-Term Complication of Laparoscopic Gastric Banding Surgery. Am J Respir Crit Care Med. 2022;205. doi:10.1164/ajrccm-conference.2022.205.1_MeetingAbstracts.A4215	No	Not meeting inclusion criteria	Title and abstract screening
2109	Habil MR, Salazar-Gonzalez RA, Doll MA, Hein DW. Differences in beta-naphthylamine metabolism and toxicity in Chinese hamster ovary cell lines transfected with human CYP1A2 and NAT2*4, NAT2*5B or NAT2*7B N-acetyltransferase 2 haplotypes. Arch Toxicol. 2022;96: 2999–3012. doi:10.1007/s00204-022-03367-2	No	Not meeting inclusion criteria	Title and abstract screening
2110	Habil MR, Salazar-González RA, Doll MA, Hein DW. Differences in β-naphthylamine metabolism and toxicity in Chinese hamster ovary cell lines transfected with human CYP1A2 and NAT2*4, NAT2*5B or NAT2*7B N-acetyltransferase 2 haplotypes. Arch Toxicol. 2022;96: 2999–3012. doi:10.1007/s00204-022-03367-2	No	Not meeting inclusion criteria	Title and abstract screening
2111	Haeny AM, Gueorguieva R, Montgomery L, Bold KW, Fucito LM, Wu R, et al. The effect of varenicline on smoking and drinking outcomes among Black and White adults with alcohol use disorder and co-occurring cigarette smoking: A secondary analysis of two clinical trials. Addict Behav. 2021;122: 106970. doi:10.1016/j.addbeh.2021.106970	No	Not meeting inclusion criteria	Title and abstract screening
2112	Hafner SJ. This is not a pipe - But how harmful is electronic cigarette smoke. Biomed J. 2021;44: 227–234. doi:10.1016/j.bj.2021.05.006	No	Not meeting inclusion criteria	Title and abstract screening
2113	Hahn AW, Ruderman SA, Nance RM, Whitney BW, Eltonsy S, Haidar L, et al. Vaporized Nicotine (E-Cigarette) and Tobacco Smoking Among People With HIV: Use Patterns and Associations With Depression and Panic Symptoms. J Acquir Immune Defic Syndr. 2023;92: 197–203. doi:10.1097/QAI.0000000000003132	No	Not meeting inclusion criteria	Title and abstract screening
2114	Hair E, Bennett M, Williams V, Johnson A, Rath J, Cantrell J, et al. Progression to established patterns of cigarette smoking among young adults. Drug Alcohol Depend. 2017;177: 77–83. doi:10.1016/j.drugalcdep.2017.03.040	No	Not meeting inclusion criteria	Title and abstract screening
2115	Hairi FM, Goh KT, Driezen P, Nordin ASA, Yee A, Tajuddin NAA, et al. Reasons for using e-cigarettes and support for e-cigarette regulations: Findings from the 2020 ITC Malaysia Survey. Tob Induc Dis. 2022;20: 33. doi:10.18332/tid/146364	No	Not meeting inclusion criteria	Title and abstract screening
2116	Hajat C, Stein E, Ramstrom L, Shantikumar S, Polosa R. The health impact of smokeless tobacco products: a systematic review. Harm Reduct J. 2021;18: 123. doi:10.1186/s12954-021-00557-6	No	Not meeting inclusion criteria	Title and abstract screening
2117	Hajat C, Stein E, Selya A, Polosa R. Analysis of common methodological flaws in the highest cited e-cigarette epidemiology research. Intern Emerg Med. 2022;17: 887–909. doi:10.1007/s11739-022-02967-1	No	Not meeting inclusion criteria	Title and abstract screening
2118	Hajat C, Stein E, Shantikumar S, Niaura R, Ferrara P, Polosa R. A scoping review of studies on the health impact of electronic nicotine delivery systems. Intern Emerg Med. 2022;17: 241–268. doi:10.1007/s11739-021-02835-4	No	Not meeting inclusion criteria	Title and abstract screening
2119	Hajek P, Phillips-Waller A, Przulj D, Pesola F, Myers Smith K, Bisal N, et al. A Randomized Trial of E-Cigarettes versus Nicotine-Replacement Therapy. N Engl J Med. 2019;380: 629–637. doi:10.1056/NEJMoa1808779	No	Not meeting inclusion criteria	Title and abstract screening
2120	Hajek P, Przulj D, Pesola F, Griffiths C, Walton R, McRobbie H, et al. Author Correction: Electronic cigarettes versus nicotine patches for smoking cessation in pregnancy: a randomized controlled trial. Nat Med. 2022. doi:10.1038/s41591-022-02099-1	No	Not meeting inclusion criteria	Title and abstract screening
2121	Hajek P, Przulj D, Pesola F, Griffiths C, Walton R, McRobbie H, et al. Electronic cigarettes versus nicotine patches for smoking cessation in pregnancy: a randomized controlled trial. Nat Med. 2022;28: 958–964. doi:10.1038/s41591-022-01808-0	No	Not meeting inclusion criteria	Title and abstract screening
2122	Hajek P, Przulj D, Pesola F, Griffiths C, Walton R, McRobbie H, et al. Author Correction: Electronic cigarettes versus nicotine patches for smoking cessation in pregnancy: a randomized controlled trial. Nat Med. 2023;29: 2957. doi:10.1038/s41591-022-02099-1	No	Not meeting inclusion criteria	Title and abstract screening
2123	Hajek P., Przulj D., Pesola F., Griffiths C., Walton R., McRobbie H., et al. Author Correction: Electronic cigarettes versus nicotine patches for smoking cessation in pregnancy: a randomized controlled trial (Nature Medicine, (2022), 28, 5, (958-964), 10.1038/s41591-022-01808-0). Nat Med. 2022. doi:10.1038/s41591-022-02099-1	No	Not meeting inclusion criteria	Title and abstract screening
2124	Hajipouran Benam K., Kaiser A., Salem C., Alvarenga B., Pagliaro A., Smith K. A Robotic System for Real-Time Analysis of Inhaled Sub-Micron and Microparticles. Am J Respir Crit Care Med. 2022;205. doi:10.1164/ajrccm-conference.2022.205.1_MeetingAbstracts.A3264	No	Not meeting inclusion criteria	Title and abstract screening
2125	Halasz G., Parati G., Piepoli M.F. Editor comment: Focus on cardiovascular prevention. Eur J Prev Cardiol. 2021;28: 1523–1525. doi:10.1093/eurjpc/zwab194	No	Not meeting inclusion criteria	Title and abstract screening
2126	Hall EA, Sauer HE, Habashy C, Anghelescu DL. Methadone for Cancer Pain in Pediatric End-of-Life Care. Am J Hosp Palliat Med. 2021;38: 914–919. doi:10.1177/1049909120963641	No	Not meeting inclusion criteria	Title and abstract screening
2127	Hall W, Gartner C, Bonevski B. Lessons from the public health responses to the US outbreak of vaping-related lung injury. Addiction. 2021;116: 985–993. doi:10.1111/add.15108https://dx.doi.org/10.1111/add.15108	No	Not meeting inclusion criteria	Title and abstract screening

2128	Hall W, Gartner C, Bonevski B. The evali outbreak makes a strong case for better regulation of all vaporizer products. Addiction. 2021;116: 999. doi:10.1111/add.15437https://dx.doi.org/10.1111/add.15437	No	Not meeting inclusion criteria	Title and abstract screening
2129	Hallingberg B, Angel L, Brown R, Copeland L, Gray L, Van Godwin J, et al. Changes in childhood experimentation with, and exposure to, tobacco and e-cigarettes and perceived smoking norms: a repeated cross-sectional study of 10-11 year olds' in Wales. BMC Public Health. 2021;21: 1924. doi:10.1186/s12889-021-12004-z	No	Not meeting inclusion criteria	Title and abstract screening
2130	Halpern D. A NEAR MIS-C. J Hosp Med. 2023;18: S816. doi:10.1002/jhm.13090	No	Not meeting inclusion criteria	Title and abstract screening
2131	Halpern SD, Volpp KG. E-Cigarettes, Incentives, and Drugs for Smoking Cessation. N Engl J Med. 2018;379: 992. doi:10.1056/NEJMc1809349	No	Not meeting inclusion criteria	Title and abstract screening
2132	Halpern-Felsher B, Kim H. Measuring E-cigarette use, dependence, and perceptions: Important principles and considerations to advance tobacco regulatory science. Addict Behav. 2018;79: 201–202. doi:10.1016/j.addbeh.2017.11.014	No	Not meeting inclusion criteria	Title and abstract screening
2133	Halpern-Felsher B. Point-of-sale marketing of heated tobacco products in Israel: Cause for concern. Isr J Health Policy Res. 2019;8: 47. doi:10.1186/s13584-019-0316-6	No	Not meeting inclusion criteria	Title and abstract screening
2134	Halpern-Felsher B., Lazaro A., Ceballos R., Zorrilla M., Zicherman B., Anoshiravani A. 180. The Stanford Vaping Information, Solutions, and Interventions Toolkit (VISIT) for Healthcare Providers. J Adolesc Health. 2022;70: S94. doi:10.1016/j.jadohealth.2022.01.097	No	Not meeting inclusion criteria	Title and abstract screening
2135	Halpern-Felsher B., Walley S.C. A Comparison of Virtual versusIn-person Curriculum on Student Knowledge and Perceptions of E-cigarettes. Pediatrics. 2022;149. Available: https://publications.aap.org/pediatrics/article/149/1MeetingAbstractsFebruary2022/735/186377/A-Comparison-of-Virtual-versus-In-person	No	Not meeting inclusion criteria	Title and abstract screening
2136	Hamad SH, Brinkman MC, Tsai Y-H, Mellouk N, Cross K, Jaspers I, et al. Pilot Study to Detect Genes Involved in DNA Damage and Cancer in Humans: Potential Biomarkers of Exposure to E-Cigarette Aerosols. Genes. 2021;12. doi:10.3390/genes12030448	No	Not meeting inclusion criteria	Title and abstract screening
2137	Hamann SL, Kungskulniti N, Charoenca N, Kasemsup V, Ruangkanchanasetr S, Jongkhajornpong P. Electronic Cigarette Harms: Aggregate Evidence Shows Damage to Biological Systems. Int J Environ Res Public Health. 2023;20. doi:10.3390/ijerph20196808	No	Not meeting inclusion criteria	Title and abstract screening
2138	Hamberger ES, Halpern-Felsher B. Concern over tobacco and marijuana perceptions and use among adolescents and young adults with cystic fibrosis. Addict Behav. 2023;142: 1–8. doi:10.1016/j.addbeh.2023.107669	No	Not meeting inclusion criteria	Title and abstract screening
2139	Hameed A., Malik D. Public Health Practitioners' Knowledge towards Nicotine and Other Cigarette Components on Various Human Diseases in Pakistan: A Contribution to Smoking Cessation Policies. BioMed Res Int. 2022;2022: 7909212. doi:10.1155/2022/7909212	No	Not meeting inclusion criteria	Title and abstract screening
2140	Hamidovic A. E-Cigarette Dependence and Weight-Related Attitudes/Behaviors Associated with Eating Disorders in Adolescent Girls. JACCP J Am Coll Clin Pharm. 2021;4: 1721. doi:10.1002/jac5.1561	No	Not meeting inclusion criteria	Title and abstract screening
2141	Hammad M, ElAffendi M, Ateya AA, Abd El-Latif AA. Efficient Brain Tumor Detection with Lightweight End-to-End Deep Learning Model. Cancers. 2023;15: 2837. doi:10.3390/cancers15102837	No	Not meeting inclusion criteria	Title and abstract screening
2142	Hammer NM, Hansson H, Pedersen LH, Abitz M, Sjøgren P, Schmiegelow K, et al. Intersectoral collaboration in home-based end-of-life pediatric cancer care: A qualitative multiple-case study integrating families' and professionals' experiences. Palliat Med. 2023;37: 149–162. doi:10.1177/02692163221135350	No	Not meeting inclusion criteria	Title and abstract screening
2143	Hammett E., Veldheer S., Yingst J., Hrabovsky S., Foulds J. Characteristics, use patterns and perceptions of electronic cigarette users who were never traditional cigarette smokers. Addict Behav. 2017;65: 92–97. doi:10.1016/j.addbeh.2016.10.007	No	Not meeting inclusion criteria	Title and abstract screening
2144	Hammond D, Reid JL, Burkhalter R, O'Connor RJ, Goniewicz ML, Wackowski OA, et al. Trends in e-cigarette brands, devices and the nicotine profile of products used by youth in England, Canada and the USA: 2017-2019. Tob Control. 2021. doi:10.1136/tobaccocontrol-2020-056371	No	Not meeting inclusion criteria	Title and abstract screening
2145	Hammond D, Reid JL, Burkhalter R, Bansal Travers M, Gravely S, Hyland A, et al. E-Cigarette Flavors, Devices, and Brands Used by Youths Before and After Partial Flavor Restrictions in the United States: Canada, England, and the United States, 2017–2020. Am J Public Health. 2022;112: 1014–1024. doi:10.2105/AJPH.2022.306780	No	Not meeting inclusion criteria	Title and abstract screening
2146	Hammond D, Reid JL, Cole AG, Leatherdale ST. Electronic cigarette use and smoking initiation among youth: a longitudinal cohort study. CMAJ. 2017;189: E1328–E1336. doi:10.1503/cmaj.161002	No	Not meeting inclusion criteria	Title and abstract screening
2147	Hammond D, Wackowski OA, Reid JL, O'Connor RJ. Use of JUUL E-cigarettes Among Youth in the United States. Nicotine Tob Res Off J Soc Res Nicotine Tob. 2020;22: 827–832. doi:10.1093/ntr/nty237	No	Not meeting inclusion criteria	Title and abstract screening
2148	Han B, Aung TW, Volkow ND, Silveira ML, Kimmel HL, Blanco C, et al. Tobacco Use, Nicotine Dependence, and Cessation Methods in US Adults With Psychosis. JAMA Netw Open. 2023;6: e234995. doi:10.1001/jamanetworkopen.2023.4995	No	Not meeting inclusion criteria	Title and abstract screening
2149	Han CH, Chung JH. Factors associated with electronic cigarette use among adolescents asthma in the Republic of Korea. J Asthma. 2021;58: 1451–1459. doi:10.1080/02770903.2020.1802745	No	Not meeting inclusion criteria	Title and abstract screening
2150	Han DH, Seo DC. Financial strain and electronic nicotine delivery systems use among U.S. young adults: A longitudinal panel analysis, 2013-2018. Addict Behav. 2021;114: 106716. doi:10.1016/j.addbeh.2020.106716	No	Not meeting inclusion criteria	Title and abstract screening
2151	Han E. SESSION 1: SUBSTANCE USE IN ADOLESCENCE, PERFORMANCE AND IMAGE-ENHANCING DRUGS (PIEDS) AND NICOTINE DEPENDENCE AND VAPING - PERFORMANCE AND IMAGE-ENHANCING DRUGS (PIEDS). Aust N Z J Psychiatry. 2022;56: 30. doi:10.1177/00048674221088686	No	Not meeting inclusion criteria	Title and abstract screening
2152	Han K-T, Kim W, Kim S. Disparities in healthcare expenditures according to economic status in cancer patients undergoing end-of-life care. BMC Cancer. 2022;22: 1–9. doi:10.1186/s12885-022-09373-y	No	Not meeting inclusion criteria	Title and abstract screening
2153	Han M, Seo D, Kim Y, Seo HG, Cho SI, Lee S, et al. Factors Associated with Quit Intentions among Adult Smokers in South Korea: Findings from the 2020 ITC Korea Survey. Int J Env Res Public Health. 2022;19. doi:10.3390/ijerph191710839	No	Not meeting inclusion criteria	Title and abstract screening
2154	Han SG, Sillé FC, Mihalic JN, Rule AM. The relationship between the use of electronic nicotine delivery systems (ENDS) and effects on pulmonary immune responses-a literature review. Env Res. 2023;221: 115234. doi:10.1016/j.envres.2023.115234	No	Not meeting inclusion criteria	Title and abstract screening
2155	Han Y., Xu Z., Mo Z., Huang H., Wu Z., Jiang X., et al. MiceVAPORDot: A novel automated approach for high-throughput behavioral characterization during E-cigarette exposure in mice. bioRxiv. 2023. doi:10.1101/2023.10.27.564133	No	Not meeting inclusion criteria	Title and abstract screening
2156	Hanafin J., Clancy L. A qualitative study of e-cigarette use among young people in Ireland: Incentives, disincentives, and putative cessation. PLoS ONE. 2020;15: e0244203. doi:10.1371/journal.pone.0244203	No	Not meeting inclusion criteria	Title and abstract screening

2157	Hanafin J., Sunday S., Clancy L. E-cigarettes and smoking in Irish teens: a logistic regression analysis of current (past 30-day) use of e-cigarettes. J Public Health Ger. 2021. doi:10.1007/s10389-021-01610-1	No	Not meeting inclusion criteria	Title and abstract screening
2158	Hanafin J., Sunday S., Clancy L. E-cigarettes in Ireland - prevalence, motivations, and relationship with tobacco. Eur Respir J. 2022;60. doi:10.1183/13993003.congress-2022.2073	No	Not meeting inclusion criteria	Title and abstract screening
2159	Hanafin J., Sunday S., Clancy L. Gender differences in teenage e-cigarette prevalence and predictive variables. Eur Respir J. 2022;60. doi:10.1183/13993003.congress-2022.2071	No	Not meeting inclusion criteria	Title and abstract screening
2160	Hanafin J, Sunday S, Clancy L. Friends and family matter Most: a trend analysis of increasing e-cigarette use among Irish teenagers and socio-demographic, personal, peer and familial associations. BMC Public Health. 2021;21: 1988. doi:10.1186/s12889-021-12113-9	No	Not meeting inclusion criteria	Title and abstract screening
2161	Hanari K, Moody SY, Sugiyama T, Tamiya N. Preferred Place of End-of-Life Care Based on Clinical Scenario: A Cross-Sectional Study of a General Japanese Population. Healthc 2227-9032. 2023;11: 406. doi:10.3390/healthcare11030406	No	Not meeting inclusion criteria	Title and abstract screening
2162	Hancox R.J., Jones S., Baggott C., Chen D., Corna N., Davies C., et al. New Zealand COPD Guidelines: Quick Reference Guide. N Z Med J. 2021;134: 76–110.	No	Not meeting inclusion criteria	Title and abstract screening
2163	Hanewinkel R, Niederberger K, Pedersen A, Unger JB, Galimov A. E-cigarettes and nicotine abstinence: a meta-analysis of randomised controlled trials. Eur Respir Rev. 2022;31. doi:10.1183/16000617.0215-2021	No	Not meeting inclusion criteria	Title and abstract screening
2164	Hanewinkel R, Niederberger K, Pedersen A, Unger JB, Galimov A. Reply to: "Nicotine or tobacco abstinence?". Eur Respir Rev. 2022;31. doi:10.1183/16000617.0158-2022	No	Not meeting inclusion criteria	Title and abstract screening
2165	Hanewinkel R. [E-Cigarettes and Health - The Cons]. Dtsch Med Wochenschr 1946. 2019;144: 1304–1306. doi:10.1055/a-0919-1289	No	Not meeting inclusion criteria	Title and abstract screening
2166	Hanewinkel R. [Electronic cigarettes: harm reduction or harm prolongation?]. Pneumol Stuttg Ger. 2023;77: 233–238. doi:10.1055/a-2034-6214	No	Not meeting inclusion criteria	Title and abstract screening
2167	Hannel T., Wei L., Muhammad-Kah R., Largo E. EPH102 Modeling the Population Health Impact of Nicotine Misperceptions. Value Health. 2023;26: S183. doi:10.1016/j.jval.2023.03.984	No	Not meeting inclusion criteria	Title and abstract screening
2168	Hansen J., Hanewinkel R., Morgenstern M. Electronic cigarette marketing and smoking behaviour in adolescence: A cross-sectional study. ERJ Open Res. 2018;4: 00155–02018. doi:10.1183/23120541.00155-2018	No	Not meeting inclusion criteria	Title and abstract screening
2169	Hansen WB, Beamon ER, Saldana S, Kelly S, Wyrick DL. D.A.R.E./keepin' it REAL elementary curriculum: Substance use outcomes. PLoS One. 2023;18: e0284457. doi:10.1371/journal.pone.0284457	No	Not meeting inclusion criteria	Title and abstract screening
2170	Hansson H, Björk M, Santacroce SJ, Raunkiær M. End-of-life palliative home care for children with cancer: A qualitative study on parents' experiences. Scand J Caring Sci. 2023;37: 917–926. doi:10.1111/scs.13066	No	Not meeting inclusion criteria	Title and abstract screening
2171	Haosheng Tan, Deyuan Fu. Influence of advanced age on the prognosis of triple-negative breast cancer patients: A surveillance, epidemiology, and end results-based study. J Cancer Res Ther. 2023;19: S323–S327. doi:10.4103/jcrt.jcrt_90_21	No	Not meeting inclusion criteria	Title and abstract screening
2172	Hapuarachchi T, Fernando G, Weerasingha S, Ozdemir S, Teo I, Vishwanath P, et al. Disparities in end-of-life outcomes among advanced cancer patients in Sri Lanka: Results from the APPROACH study. Palliat Support Care. 2022;20: 832–838. doi:10.1017/S147895152100167X	No	Not meeting inclusion criteria	Title and abstract screening
2173	Harada HA, Liu J, Shortell JD, Beesley SJ. A 20-Year-Old Man with e-Cigarette or Vaping Product Use-Associated Lung Injury (EVALI) and Thrombotic Coagulopathy. Am J Case Rep. 2021;22: e929915. doi:10.12659/AJCR.929915	No	Not meeting inclusion criteria	Title and abstract screening
2174	Hardie L, McCool J, Freeman B. Online retail promotion of e-cigarettes in New Zealand: A content analysis of e-cigarette retailers in a regulatory void. Health Promot J Aust Off J Aust Assoc Health Promot Prof. 2022;33: 91–98. doi:10.1002/hpja.464	No	Not meeting inclusion criteria	Title and abstract screening
2175	Hardie L, McCool J, Freeman B. E-Cigarette Retailers' Use of Instagram in New Zealand: A Content Analysis. Int J Environ Res Public Health. 2023;20. doi:10.3390/ijerph20031897	No	Not meeting inclusion criteria	Title and abstract screening
2176	Harini G., Leelavathi L. Nicotine replacement therapy for smoking cessation-an overview. Indian J Public Health Res Dev. 2019;10: 3588–3592. doi:10.5958/0976-5506.2019.04144.5	No	Not meeting inclusion criteria	Title and abstract screening
2177	Harlow AF, McConnell RS, Barrington-Trimis JL. Underage E-Cigarette Purchasing and Vaping Progression Among Young Adults. J Adolesc Health. 2023;72: 260–266. doi:10.1016/j.jadohealth.2022.09.018	No	Not meeting inclusion criteria	Title and abstract screening
2178	Harlow AF, Stokes AC, Brooks DR, Benjamin EJ, Leventhal AM, McConnell RS, et al. Prospective association between e-cigarette use frequency patterns and cigarette smoking abstinence among adult cigarette smokers in the United States. Addiction. 2022;117: 3129–3139. doi:10.1111/add.16009	No	Not meeting inclusion criteria	Title and abstract screening
2179	Harlow AF, Vogel EA, Tackett AP, Cho J, Han DH, Wong M, et al. Adolescent Use of Flavored Non-Tobacco Oral Nicotine Products. Pediatrics. 2022;150. doi:10.1542/peds.2022-056586	No	Not meeting inclusion criteria	Title and abstract screening
2180	Harlow AF, McConnell RS, Barrington-Trimis JL. Underage E-Cigarette Purchasing and Vaping Progression Among Young Adults. J Adolesc Health Off Publ Soc Adolesc Med. 2022. doi:10.1016/j.jadohealth.2022.09.018	No	Not meeting inclusion criteria	Title and abstract screening
2181	Harnischfeger N, Rath HM, Alt-Epping B, Brand H, Haller K, Letsch A, et al. Association between oncologists' death anxiety and their end-of-life communication with advanced cancer patients. Psychooncology. 2023;32: 923–932. doi:10.1002/pon.6132	No	Not meeting inclusion criteria	Title and abstract screening
2182	Harrell P.T., Brandon T.H., England K.J., Barnett T.E., Brockenberry L.O., Simmons V.N., et al. Vaping Expectancies: A Qualitative Study among Young Adult Nonusers, Smokers, Vapers, and Dual Users. Subst Abuse Res Treat. 2019;13. doi:10.1177/1178221819866210	No	Not meeting inclusion criteria	Title and abstract screening
2183	Harrell PT, Brandon TH, Stark SE, Simmons VN, Barnett TE, Quinn GP, et al. Measuring vaping-related expectancies in young adults: Psychometric evaluation of the Electronic Nicotine Vaping Outcomes (ENVO) scale. Drug Alcohol Depend. 2023;246: 109861. doi:10.1016/j.drugalcdep.2023.109861	No	Not meeting inclusion criteria	Title and abstract screening
2184	Harrell PT, Naqvi SMH, Plunk AD, Ji M, Martins SS. Patterns of youth tobacco and polytobacco usage: The shift to alternative tobacco products. Am J Drug Alcohol Abuse. 2017;43: 694–702. doi:10.1080/00952990.2016.1225072	No	Not meeting inclusion criteria	Title and abstract screening
2185	Harrington C, Walsh E. Tobacco-free campuses - a pipe dream? A survey of current smoking cessation practice in mental health units in Ireland. BJPsych Bull. 2023; 1–5. doi:10.1192/bjb.2023.50	No	Not meeting inclusion criteria	Title and abstract screening

2186	Harris A.C., Muelken P., Smethells J.R., Yershova K., Stepanov I., Olson T.T., et al. Effects of nicotine-containing and “nicotine-free” e-cigarette refill liquids on intracranial self-stimulation in rats. Drug Alcohol Depend. 2018;185: 1–9. doi:10.1016/j.drugalcdep.2017.11.032	No	Not meeting inclusion criteria	Title and abstract screening
2187	Harris A.C., Smethells J.R., Palumbo M., Goniewicz M., Lesage M.G. Comparison of the relative abuse liability of electronic cigarette aerosol extracts and nicotine alone in adolescent rats: A behavioral economic analysis. Int J Environ Res Public Health. 2020;17: 860. doi:10.3390/ijerph17030860	No	Not meeting inclusion criteria	Title and abstract screening
2188	Harris CC. Tobacco smoking, E-cigarettes, and nicotine harm. Proc Natl Acad Sci U A. 2018;115: 1406–1407. doi:10.1073/pnas.1722636115	No	Not meeting inclusion criteria	Title and abstract screening
2189	Harter P, Mouret-Reynier M-A, Lorusso D, Cropet C, Guerra EM, Wolfrum-Ristau P, et al. Efficacy of subsequent therapies in patients (pts) with advanced ovarian cancer (AOC) in the phase III PAOLA-1/ENGOT-ov25 trial according to whether disease progression occurred during or after the end of olaparib (ola) maintenance. J Clin Oncol. 41: 5550–5550. doi:10.1200/JCO.2023.41.16_suppl.5550	No	Not meeting inclusion criteria	Title and abstract screening
2190	Hartmann-Boyce J, Butler AR, Theodoulou A, Onakpoya IJ, Hajek P, Bullen C, et al. Biomarkers of potential harm in people switching from smoking tobacco to exclusive e-cigarette use, dual use or abstinence: secondary analysis of Cochrane systematic review of trials of e-cigarettes for smoking cessation. Addiction. 2022. doi:10.1111/add.16063	No	Not meeting inclusion criteria	Title and abstract screening
2191	Hartmann-Boyce J, Butler AR, Theodoulou A, Onakpoya IJ, Hajek P, Bullen C, et al. Biomarkers of potential harm in people switching from smoking tobacco to exclusive e-cigarette use, dual use or abstinence: secondary analysis of Cochrane systematic review of trials of e-cigarettes for smoking cessation. Addiction. 2023;118: 539–545. doi:10.1111/add.16063	No	Not meeting inclusion criteria	Title and abstract screening
2192	Hartmann-Boyce J, Lindson N, Butler AR, McRobbie H, Bullen C, Begh R, et al. Electronic cigarettes for smoking cessation. Cochrane Database Syst Rev. 2022;11: CD010216. doi:10.1002/14651858.CD010216.pub7	No	Not meeting inclusion criteria	Title and abstract screening
2193	Hartmann-Boyce J, McRobbie H, Butler AR, Lindson N, Bullen C, Begh R, et al. Electronic cigarettes for smoking cessation. Cochrane Database Syst Rev. 2021;9: CD010216. doi:10.1002/14651858.CD010216.pub6	No	Not meeting inclusion criteria	Title and abstract screening
2194	Hartmann-Boyce J, Theodoulou A, Farley A, Hajek P, Lycett D, Jones LL, et al. Interventions for preventing weight gain after smoking cessation. Cochrane Database Syst Rev. 2021;10: CD006219. doi:10.1002/14651858.CD006219.pub4	No	Not meeting inclusion criteria	Title and abstract screening
2195	Hartmann-Boyce J., Lindson N., Butler A.R., McRobbie H., Bullen C., Begh R., et al. Electronic cigarettes for smoking cessation. Cochrane Database Syst Rev. 2022;2022: CD010216. doi:10.1002/14651858.CD010216.pub7	No	Not meeting inclusion criteria	Title and abstract screening
2196	Hartmann-Boyce J., Theodoulou A., Farley A., Hajek P., Lycett D., Jones L.L., et al. Interventions for preventing weight gain after smoking cessation. Cochrane Database Syst Rev. 2021;2021: CD006219. doi:10.1002/14651858.CD006219.pub4	No	Not meeting inclusion criteria	Title and abstract screening
2197	Hartshorn G, Browning M, Chalil Madathil K, Mau F, Ranganathan S, Todd A, et al. Efficacy of virtual reality assisted guided imagery (VRAGI) in a home setting for pain management in patients with advanced cancer: protocol for a randomised controlled trial. 2022;12. doi:10.1136/bmjopen-2022-064363	No	Not meeting inclusion criteria	Title and abstract screening
2198	Hartwell G, Egan M, Brown J, Pliakas T, Petticrew M. Use of e-Cigarettes and Attendance at Stop Smoking Services: A Population Survey in England. Toxics. 2022;10. doi:10.3390/toxics10100593	No	Not meeting inclusion criteria	Title and abstract screening
2199	Hartwell G, Egan M, Petticrew M. Understanding decisions to use e-cigarettes or behavioural support to quit tobacco: a qualitative study of current and ex-smokers and stop smoking service staff. Addict Abingdon Engl. 2020;115: 518–526. doi:10.1111/add.14844	No	Not meeting inclusion criteria	Title and abstract screening
2200	Hartz SM, Horton AC, Hancock DB, Baker TB, Caporaso NE, Chen LS, et al. Genetic correlation between smoking behaviors and schizophrenia. Schizophr Res. 2018;194: 86–90. doi:10.1016/j.schres.2017.02.022	No	Not meeting inclusion criteria	Title and abstract screening
2201	Harvanko A., Kryscio R., Martin C., Kelly T. Stimulus effects of propylene glycol and vegetable glycerin in electronic cigarette liquids. Drug Alcohol Depend. 2019;194: 326–329. doi:10.1016/j.drugalcdep.2018.08.039	No	Not meeting inclusion criteria	Title and abstract screening
2202	Harvanko A, Koester KA, Helen GS, Olson S, Kim HC, Ling PM. A Mixed-Methods Study on Use of Different Tobacco Products among Younger and Older Adults with Lower and Higher Levels of Nicotine Exposure in California in 2019-2020. Int J Environ Res Public Health. 2022;19. doi:10.3390/ijerph19095563	No	Not meeting inclusion criteria	Title and abstract screening
2203	Hasan M., Bellamkonda L., Vo T. A case of massive hemoptysis secondary to metastatic lung cancer. Am J Med Sci. 2023;365: S256–S257. doi:10.1016/S0002-9629%2823%2900482-2	No	Not meeting inclusion criteria	Title and abstract screening
2204	Hasegawa T, Ito Y, Furukawa Y, Okuyama T, Kojima N, Uchida M, et al. Specialized Palliative Care and Intensity of End-of-Life Care Among Adolescents and Young Adults with Cancer: A Medical Chart Review. J Adolesc Young Adult Oncol. 2023;12: 488–495. doi:10.1089/jayao.2022.0078	No	Not meeting inclusion criteria	Title and abstract screening
2205	Hassan A., Jafry A., Gupta S., Ul Haq A., Moad J., Bhardwaj H. CATAMENIAL HEMOPNEUMOTHORAX: AN UNUSUAL CASE OF ENDOMETRIOSIS OF THE PLEURA. Chest. 2021;160: A1358. doi:10.1016/j.chest.2021.07.1242	No	Not meeting inclusion criteria	Title and abstract screening
2206	Hassoun A., Brady K., Arefi R., Trifonova I., Tsirilakis K. Vaping-Associated Lung Injury During COVID-19 Multisystem Inflammatory Syndrome Outbreak. J Emerg Med. 2021;60: 524–530. doi:10.1016/j.jemermed.2020.12.005	No	Not meeting inclusion criteria	Title and abstract screening
2207	Hatano Y, Morita T, Mori M, Aoyama M, Yoshida S, Amano K, et al. Association between experiences of advanced cancer patients at the end of life and depression in their bereaved caregivers. Psychooncology. 2022;31: 1243–1252. doi:10.1002/pon.5915	No	Not meeting inclusion criteria	Title and abstract screening
2208	Hatsukami D.K. Reducing Nicotine in Cigarettes to Minimally Addictive Levels: A New Frontier for Tobacco Control. JAMA Psychiatry. 2018;75: 987–988. doi:10.1001/jamapsychiatry.2018.1829	No	Not meeting inclusion criteria	Title and abstract screening
2209	Hatsukami DK, Xu D, Ferris Wayne G. Regulatory Approaches and Implementation of Minimally Addictive Combusted Products. Nicotine Tob Res. 2022;24: 453–462. doi:10.1093/ntr/ntab138	No	Not meeting inclusion criteria	Title and abstract screening
2210	Hatsukami DK, Donny EC. The debate about nicotine addiction and the role of medicinal products: Commentary on Zeller. Nicotine Tob Res. 2019;21: 338–339. doi:10.1093/ntr/nty195https://dx.doi.org/10.1093/ntr/nty195	No	Not meeting inclusion criteria	Title and abstract screening
2211	Hawkins K.B., Dunn C.P., Tercyak K.P., Riley J.B. 156. Factors Associated with Disordered Eating Behavior Among Adolescent Girls: Screening and Education. J Adolesc Health. 2022;70: S82. doi:10.1016/j.jadohealth.2022.01.073	No	Not meeting inclusion criteria	Title and abstract screening
2212	Hayashi E, Aoyama M, Fukano F, Takano J, Shimizu Y, Miyashita M. Effects of Bathing in a Tub on Physical and Psychological Symptoms of End-of-Life Cancer Patients: An Observational, Controlled Study. J Hosp Palliat Nurs. 2022;24: 30–39. doi:10.1097/NJH.0000000000000803	No	Not meeting inclusion criteria	Title and abstract screening
2213	Hayashi Y, Sato K, Ogawa M, Taguchi Y, Wakayama H, Nishioka A, et al. Association Among End-Of-Life Discussions, Cancer Patients’ Quality of Life at End of Life, and Bereaved Families’ Mental Health. Am J Hosp Palliat Med. 2022;39: 1071–1081. doi:10.1177/10499091211061713	No	Not meeting inclusion criteria	Title and abstract screening
2214	Hayes JE, Baker AN. Flavor science in the context of research on electronic cigarettes. Front Neurosci. 2022;16: 918082. doi:10.3389/fnins.2022.918082	No	Not meeting inclusion criteria	Title and abstract screening

2215	Hayes K.A., Olson L.T., Brown E.M., Battles H.B., Juster H.R. Medicaid coverage for tobacco dependence treatment: Enrollee awareness and use. Prev Med Rep. 2021;24: 101509. doi:10.1016/j.pmedr.2021.101509	No	Not meeting inclusion criteria	Title and abstract screening
2216	Haziza C, de La Bourdonnaye G, Merlet S, Benzimra M, Ancerewicz J, Donelli A, et al. Assessment of the reduction in levels of exposure to harmful and potentially harmful constituents in Japanese subjects using a novel tobacco heating system compared with conventional cigarettes and smoking abstinence: a randomized controlled study in con. 2016;81: 489-499. doi:10.1016/j.yrtph.2016.09.014	No	Not meeting inclusion criteria	Title and abstract screening
2217	He M, Qing K, Tustison NJ, Beaulac Z, King TW, Huff TB, et al. Characterizing Gas Exchange Physiology in Healthy Young Electronic-Cigarette Users with Hyperpolarized 129Xe MRI: A Pilot Study. Int J Chron Obstruct Pulmon Dis. 2021;16: 3183–3187. doi:10.2147/COPD.S324388	No	Not meeting inclusion criteria	Title and abstract screening
2218	Heath D.M., Koslosky E.J., Bartush K.C., Hogue G.D. Marijuana in Orthopaedics: Effects on Bone Health, Wound-Healing, Surgical Complications, and Pain Management. JBJS Rev. 2022;10: e21.00184. doi:10.2106/JBJS.RVW.21.00184	No	Not meeting inclusion criteria	Title and abstract screening
2219	Hébert ET, Bhushan T, Ra CK, Frank-Pearce S, Alexander AC, Cole AB, et al. Daily use of nicotine replacement medications is related to daily smoking status: An ecological momentary assessment study. Drug Alcohol Depend. 2021;229: 109161. doi:10.1016/j.drugalcdep.2021.109161	No	Not meeting inclusion criteria	Title and abstract screening
2220	Hebert-Losier A., Filion K.B., Windle S.B., Eisenberg M.J. A Randomized Controlled Trial Evaluating the Efficacy of E-Cigarette Use for Smoking Cessation in the General Population: E3 Trial Design. CJC Open. 2020;2: 168–175. doi:10.1016/j.cjco.2020.03.006	No	Not meeting inclusion criteria	Title and abstract screening
2221	Heckman B.W., Dahne J., Germeroth L.J., Mathew A.R., Santa Ana E.J., Johnson R.H., et al. Does Cessation Fatigue Predict Smoking-Cessation Milestones? A Longitudinal Study of Current and Former Smokers. J Consult Clin Psychol. 2018;86: 903–914. doi:10.1037/ccp0000338	No	Not meeting inclusion criteria	Title and abstract screening
2222	Heckman BW, Cummings KM, Stoltman JJK, Dahne J, Borland R, Fong GT, et al. Longer duration of smoking abstinence is associated with waning cessation fatigue. Behav Res Ther. 2019;115: 12–18. doi:10.1016/j.brat.2018.11.011	No	Not meeting inclusion criteria	Title and abstract screening
2223	Heers H, Gschnell M, Schrade S, Urhahn F, Pedrosa Carrasco A, Morin A, et al. End of life care: Preferences of patients with advanced urologic and dermatologic malignancies. J Clin Oncol. 41: e24006–e24006. doi:10.1200/JCO.2023.41.16_suppl.e24006	No	Not meeting inclusion criteria	Title and abstract screening
2224	Hefner K, Valentine G, Sofuoglu M. Electronic cigarettes and mental illness: Reviewing the evidence for help and harm among those with psychiatric and substance use disorders. Am J Addict. 2017;26: 306–315. doi:10.1111/ajad.12504	No	Not meeting inclusion criteria	Title and abstract screening
2225	Hefner KR, Sollazzo A, Mullaney S, Coker KL, Sofuoglu M. E-cigarettes, alcohol use, and mental health: Use and perceptions of e-cigarettes among college students, by alcohol use and mental health status. Addict Behav. 2019;91: 12–20. doi:10.1016/j.addbeh.2018.10.040	No	Not meeting inclusion criteria	Title and abstract screening
2226	Heiden BT, Baker TB, Smock N, Pham G, Chen J, Bierut LJ, et al. Assessment of formal tobacco treatment and smoking cessation in dual users of cigarettes and e-cigarettes. Thorax. 2022. doi:10.1136/thorax-2022-218680	No	Not meeting inclusion criteria	Title and abstract screening
2227	Heiden BT, Eaton DB Jr, Chang SH, Yan Y, Schoen MW, Chen LS, et al. The Impact of Persistent Smoking After Surgery on Long-term Outcomes After Stage I Non-small Cell Lung Cancer Resection. Chest. 2022;161: 1687–1696. doi:10.1016/j.chest.2021.12.634	No	Not meeting inclusion criteria	Title and abstract screening
2228	Heiden BT, Engelhardt KE, Cao C, Meyers BF, Puri V, Cao Y, et al. Association between lung cancer screening and smoking cessation. Cancer Epidemiol. 2022;79: 102194. doi:10.1016/j.canep.2022.102194	No	Not meeting inclusion criteria	Title and abstract screening
2229	Heiden BT, Engelhardt KE, Cao C, Meyers BF, Puri V, Cao Y, et al. Prevalence of cigarette and e-cigarette use among U.S. adults eligible for lung cancer screening based on updated USPSTF guidelines. Cancer Epidemiol. 2022;76: 102079. doi:10.1016/j.canep.2021.102079	No	Not meeting inclusion criteria	Title and abstract screening
2230	Heinly A, Walley S. The nicotine and tobacco epidemic among adolescents: new products are addicting our youth. Curr Opin Pediatr. 2023;35: 513–521. doi:10.1097/MOP.0000000000001271	No	Not meeting inclusion criteria	Title and abstract screening
2231	Heldt N.A., Reichenbach N., McGary H.M., Persidsky Y. Effects of Electronic Nicotine Delivery Systems and Cigarettes on Systemic Circulation and Blood-Brain Barrier: Implications for Cognitive Decline. Am J Pathol. 2021;191: 243–255. doi:10.1016/j.ajpath.2020.11.007	No	Not meeting inclusion criteria	Title and abstract screening
2232	Helgertz S, Kingsbury J. Teens less susceptible to vaping when restricted to tobacco-flavored e-cigarettes: implications for flavored tobacco policies. Nicotine Tob Res Off J Soc Res Nicotine Tob. 2022. doi:10.1093/ntr/ntac272	No	Not meeting inclusion criteria	Title and abstract screening
2233	Hellesen S, Bankston-Lee K, Maytum C, Tong EK. Strategies and lessons learned for smoke and Tobacco-Free policy change on community colleges with community-based support. J Am Coll Health. 2022;70: 2406–2415. doi:10.1080/07448481.2020.1865972	No	Not meeting inclusion criteria	Title and abstract screening
2234	Hellesen S., Bankston-Lee K., Maytum C., Tong E.K. Strategies and lessons learned for smoke and Tobacco-Free policy change on community colleges with community-based support. J Am Coll Health J ACH. 2021; 1–10. doi:10.1080/07448481.2020.1865972	No	Not meeting inclusion criteria	Title and abstract screening
2235	Hemmy Asamsama O, Miller SC, Silvestri MM, Bonanno C, Krondilou K. Impact of implementing a tobacco and recreational nicotine-free policy and enhanced treatments on programmatic and patient-level outcomes within a residential substance use disorder treatment program. J Subst Abuse Treat. 2019;107: 44–49. doi:10.1016/j.jsat.2019.09.004	No	Not meeting inclusion criteria	Title and abstract screening
2236	Henderson B.J., Cooper S.Y. Nicotine formulations impact reinforcement-related behaviors in a mouse model of vapor self-administration. Drug Alcohol Depend. 2021;224: 108732. doi:10.1016/j.drugalcdep.2021.108732	No	Not meeting inclusion criteria	Title and abstract screening
2237	Henderson BJ, Richardson MR, Cooper SY. A high-fat diet has sex-specific effects on nicotine vapor self-administration in mice. Drug Alcohol Depend. 2022;241: 109694. doi:10.1016/j.drugalcdep.2022.109694	No	Not meeting inclusion criteria	Title and abstract screening
2238	Henderson E, Rodriguez Guerrero LA, Continente X, Fernández E, Tigova O, Cortés-Francisco N, et al. Measurement of airborne nicotine, as a marker of secondhand smoke exposure, in homes with residents who smoke in 9 European countries. Env Res. 2023;219: 115118. doi:10.1016/j.envres.2022.115118	No	Not meeting inclusion criteria	Title and abstract screening
2239	Hendlin Y., Vora M., Elias J., Benowitz-Fredericks C., Ling P. Assessing the tobacco harm reduction (THR) debate: A systematic review. Tob Induc Dis. 2018;16: 257–258. doi:10.18332/tid/84665	No	Not meeting inclusion criteria	Title and abstract screening
2240	Henn SL, Martinasek MP, Lange M. Vaping Behavior in Young Adults During the COVID-19 Pandemic. Respir Care. 2023;68: 1493–1501. doi:10.4187/respcare.10629	No	Not meeting inclusion criteria	Title and abstract screening
2241	Hensel EC, Sarles SE, Nuss CJ, Terry JN, Polgampola Ralalage CR, DiFrancesco AG, et al. Effect of Third Party Components on Emissions from a Pod Style Electronic Cigarette. Toxicol Sci Off J Soc Toxicol. 2023. doi:10.1093/toxsci/kfad096	No	Not meeting inclusion criteria	Title and abstract screening
2242	Henstra C, Dekkers BGJ, Olgers TJ, Ter Maaten JC, Touw DJ. Managing intoxications with nicotine-containing e-liquids. Expert Opin Drug Metab Toxicol. 2022;18: 115–121. doi:10.1080/17425255.2022.2058930	No	Not meeting inclusion criteria	Title and abstract screening
2243	Herbach E, O'Rorke MA, Carnahan RM, McDowell BD, Allen B, Grumbach I, et al. Cardiac Adverse Events Associated With Chemo-Radiation Versus Chemotherapy for Resectable Stage III Non-Small-Cell Lung Cancer: A Surveillance, Epidemiology and End Results-Medicare Study. J Am Heart Assoc. 2022;11: 1–14. doi:10.1161/JAHA.122.027288	No	Not meeting inclusion criteria	Title and abstract screening

2244	Herbec A, Brown J, Jackson SE, Kale D, Zatoński M, Garnett C, et al. Perceived risk factors for severe Covid-19 symptoms and their association with health behaviours: Findings from the HEBECO study. Acta Psychol Amst. 2022;222: 103458. doi:10.1016/j.actpsy.2021.103458	No	Not meeting inclusion criteria	Title and abstract screening
2245	Herbec A., Zatonski M., Zatonski W.A., Janik-Koncewicz K., Mons U., Fong G.T., et al. Dependence, plans to quit, quitting self-efficacy and past cessation behaviours among menthol and other flavoured cigarette users in Europe: The EUREST-PLUS ITC Europe Surveys. Tob Induc Dis. 2018;16: A19. doi:10.18332/tid/111356	No	Not meeting inclusion criteria	Title and abstract screening
2246	Herbert G., Woolley G., Roberts D., Domarkas J., Wright J., Wright G., et al. THE QUANTITATIVE ASSESSMENT OF VAPE DEVICES AS NOVEL PULMONARY DRUG DELIVERY SYSTEMS USING FLUORINE-18 RADIOLABELLED DRUG MOLECULES. J Aerosol Med Pulm Drug Deliv. 2022;35: A17. doi:10.1089/jamp.2022.ab01.abstracts	No	Not meeting inclusion criteria	Title and abstract screening
2247	Herbst RS, Hatsukami D, Acton D, Giuliani M, Moushey A, Phillips J, et al. Electronic Nicotine Delivery Systems: An Updated Policy Statement from the American Association for Cancer Research and the American Society of Clinical Oncology. Clin Cancer Res. 2022;28: 4861–4870. doi:10.1158/1078-0432.CCR-22-2429	No	Not meeting inclusion criteria	Title and abstract screening
2248	Herbst RS, Hatsukami D, Acton D, Giuliani M, Moushey A, Phillips J, et al. Electronic Nicotine Delivery Systems: An Updated Policy Statement From the American Association for Cancer Research and the American Society of Clinical Oncology. J Clin Oncol. 2022;40: 4144–4155. doi:10.1200/JCO.22.01749	No	Not meeting inclusion criteria	Title and abstract screening
2249	Hering T. [E-cigarettes: toxicological fiasco or better than not giving up smoking?]. Internist Berl. 2020;61: 634–643. doi:10.1007/s00108-020-00794-0	No	Not meeting inclusion criteria	Title and abstract screening
2250	Heris C, Scully M, Chamberlain C, White V. E-cigarette use and the relationship to smoking among Aboriginal and Torres Strait Islander and non-Indigenous Australian Secondary Students, 2017. Aust N Z J Public Health. 2022. doi:10.1111/1753-6405.13299	No	Not meeting inclusion criteria	Title and abstract screening
2251	Herman M, Tarran R. E-cigarettes, nicotine, the lung and the brain: multi-level cascading pathophysiology. J Physiol. 2020;598: 5063–5071. doi:10.1113/JP278388	No	Not meeting inclusion criteria	Title and abstract screening
2252	Hernandez A, Sonavane M, Smith KR, Seiger J, Migaud ME, Gassman NR. Dihydroxyacetone suppresses mTOR nutrient signaling and induces mitochondrial stress in liver cells. PLoS One. 2022;17: e0278516. doi:10.1371/journal.pone.0278516	No	Not meeting inclusion criteria	Title and abstract screening
2253	Hernandez ML, Burbank AJ, Alexis NE, Rebuli ME, Hickman ED, Jaspers I, et al. Electronic Cigarettes and Their Impact on Allergic Respiratory Diseases: A Work Group Report of the AAAAI Environmental Exposures and Respiratory Health Committee. J Allergy Clin Immunol Pr. 2021;9: 1142–1151. doi:10.1016/j.jaip.2020.12.065	No	Not meeting inclusion criteria	Title and abstract screening
2254	Hernandez-Diaz S., Smith L.H., Dollinger C., Wyszynski D. Risk factors for Severe Coronavirus Disease 2019 (COVID-19) during pregnancy. Paediatr Perinat Epidemiol. 2021;35: 79. doi:10.1111/ppe.12814	No	Not meeting inclusion criteria	Title and abstract screening
2255	Hernandez-Diaz S., Smith L.H., Dollinger C., Rasmussen S.A., Schisterman E.F., Bellocco R., et al. International Registry of Coronavirus Exposure in Pregnancy (IRCEP): Cohort Description and Methodological Considerations. Am J Epidemiol. 2022;191: 967–979. doi:10.1093/aje/kwac046	No	Not meeting inclusion criteria	Title and abstract screening
2256	Hernandez-Perez A, Garcia-Gomez L, Robles-Hernandez R, Thirion-Romero I, Osio-Echanove J, Rodriguez-Llamazares S, et al. Addiction to Tobacco Smoking and Vaping. Rev Investig Clin Organo Hosp Enfermedades Nutr. 2023;75: 158–168. doi:10.24875/RIC.23000117	No	Not meeting inclusion criteria	Title and abstract screening
2257	Herndon P, Jassal JS, Cramer JD. Association between E-cigarette use and oral HPV-16 infection. Oral Oncol. 2022;125: 105676. doi:10.1016/j.oraloncology.2021.105676	No	Not meeting inclusion criteria	Title and abstract screening
2258	Héron M, Le Faou AL, Ibanez G, Métadieu B, Melchior M, El-Khoury Lesueur F. Smoking cessation using preference-based tools: a mixed method pilot study of a novel intervention among smokers with low socioeconomic position. Addict Sci Clin Pr. 2021;16: 43. doi:10.1186/s13722-021-00254-6	No	Not meeting inclusion criteria	Title and abstract screening
2259	Herout KT, Durant EJ, Fong J. Dysphagia as the Predominant Symptom in Posterior Circulation Stroke: A Case Report. Am J Case Rep. 2021;22: e930502. doi:10.12659/AJCR.930502	No	Not meeting inclusion criteria	Title and abstract screening
2260	Herrel LA, Zhu Z, Ryan AM, Hollenbeck BK, Miller DC. Intensity of end-of-life care for dual-eligible beneficiaries with cancer and the impact of delivery system affiliation. Cancer 0008543X. 2021;127: 4628–4635. doi:10.1002/cncr.33874	No	Not meeting inclusion criteria	Title and abstract screening
2261	Herrmann AK, Cowgill B, Guthmann D, Richardson J, Cindy Chang L, Crespi CM, et al. Developing and Evaluating a School-Based Tobacco and E-Cigarette Prevention Program for Deaf and Hard-of-Hearing Youth. Health Promot Pr. 2023; 15248399221151180. doi:10.1177/15248399221151180	No	Not meeting inclusion criteria	Title and abstract screening
2262	Herrmann AK, Ferullo SL, Hernandez M, Barriga VA, Leggis B, Ruiz J, et al. Adapting E-cigarette prevention programming to reach the latinx community. Cancer Causes Control. 2023. doi:10.1007/s10552-023-01796-7	No	Not meeting inclusion criteria	Title and abstract screening
2263	Hersi M., Traversy G., Thombs B.D., Beck A., Skidmore B., Groulx S., et al. Effectiveness of stop smoking interventions among adults: Protocol for an overview of systematic reviews and an updated systematic review. Syst Rev. 2019;8: 28. doi:10.1186/s13643-018-0928-x	No	Not meeting inclusion criteria	Title and abstract screening
2264	Herzig SE, Albers L, Soto D, Lee R, Ramirez C, Rahman T, et al. Pandemic-related life changes and adolescent initiation of cannabis and tobacco/nicotine use. Addict Behav. 2023;144: 107724. doi:10.1016/j.addbeh.2023.107724	No	Not meeting inclusion criteria	Title and abstract screening
2265	Heung Y, Zhukovsky D, Hui D, Lu Z, Andersen C, Bruera E. Quality of End-of-Life Care during the COVID-19 Pandemic at a Comprehensive Cancer Center. Cancers. 2023;15: 2201. doi:10.3390/cancers15082201	No	Not meeting inclusion criteria	Title and abstract screening
2266	Hey Seemann Vestergaard A, Asbjoern Neergaard M, Fokdal LU, Fynbo Christiansen C, Brink Valentin J, Paaske Johnsen S. Utilisation of Hospital-Based Specialist Palliative Care in Patients With Gynaecological Cancer: Temporal Trends, Predictors and Association With High-Intensity End-of-Life Care. Obstet Gynecol Surv. 2023;78: 474–475. doi:10.1097/01.ogx.0000967020.48960.63	No	Not meeting inclusion criteria	Title and abstract screening
2267	Heza L., Olive A., Miller J. Pneumatosis intestinalis and intestinal perforation in a case of multisystem inflammatory syndrome in children. BMJ Case Rep. 2021;14: 241688. doi:10.1136/bcr-2021-241688	No	Not meeting inclusion criteria	Title and abstract screening
2268	Hickling LM, Perez-Iglesias R, McNeill A, Dawkins L, Moxham J, Ruffell T, et al. A pre-post pilot study of electronic cigarettes to reduce smoking in people with severe mental illness. Psychol Med. 2019;49: 1033–1040. doi:10.1017/S0033291718001782	No	Not meeting inclusion criteria	Title and abstract screening
2269	Hickman E., Cobos-Uribe C., Immormino R., Rebuli M.E., Moran T., Alexis N.E., et al. Are Human Monocyte Derived Macrophages a Suitable Surrogate for In Vivo Derived Lung Macrophages in Studies of Inhaled Toxicants? Am J Respir Crit Care Med. 2022;205. doi:10.1164/ajrccm-conference.2022.205.1_MeetingAbstracts.A5789	No	Not meeting inclusion criteria	Title and abstract screening
2270	Higgins GA, Sellers EM. 5-HT2A and 5-HT2C receptors as potential targets for the treatment of nicotine use and dependence. Prog Brain Res. 2021;259: 229–263. doi:10.1016/bs.pbr.2021.01.007	No	Not meeting inclusion criteria	Title and abstract screening
2271	Higgins S.T. Editorial: 5th Special Issue on behavior change, health, and health disparities. Prev Med. 2018;117: 1–4. doi:10.1016/j.ypmed.2018.10.008	No	Not meeting inclusion criteria	Title and abstract screening
2272	Higgins ST, Bergeria CL, Davis DR, Streck JM, Villanti AC, Hughes JR, et al. Response to reduced nicotine content cigarettes among smokers differing in tobacco dependence severity. Prev Med. 2018;117: 15–23. doi:10.1016/j.ypmed.2018.04.010	No	Not meeting inclusion criteria	Title and abstract screening

2273	Higgins ST, DeSarno M, Bunn JY, Gaalema DE, Leventhal AM, Davis DR, et al. Cumulative vulnerabilities as a potential moderator of response to reduced nicotine content cigarettes. Prev Med. 2021;152: 106714. doi:10.1016/j.ypmed.2021.106714	No	Not meeting inclusion criteria	Title and abstract screening
2274	Higgins ST, Erath TG, DeSarno M, Reed DD, Gaalema DE, Sigmon SC, et al. Leveraging the cigarette purchase task to understand relationships between cumulative vulnerabilities, the relative reinforcing effects of smoking, and response to reduced nicotine content cigarettes. Prev Med. 2022; 107206. doi:10.1016/j.ypmed.2022.107206	No	Not meeting inclusion criteria	Title and abstract screening
2275	Higgins ST, Kurti AN, Palmer M, Tidey JW, Cepeda-Benito A, Cooper MR, et al. A review of tobacco regulatory science research on vulnerable populations. Prev Med. 2019;128: 105709. doi:10.1016/j.ypmed.2019.04.024	No	Not meeting inclusion criteria	Title and abstract screening
2276	Higgins ST, Redner R, Arger CA, Kurti AN, Priest JS, Bunn JY. Use of higher-nicotine/tar-yield (regular full-flavor) cigarettes is associated with nicotine dependence and smoking during pregnancy among U.S. women. Prev Med. 2017;104: 57–62. doi:10.1016/j.ypmed.2017.07.029	No	Not meeting inclusion criteria	Title and abstract screening
2277	Higham A, Beech A, Jackson N, Lea S, Singh D. Sputum cell counts in COPD patients who use electronic cigarettes. Eur Respir J. 2022;59. doi:10.1183/13993003.03016-2021	No	Not meeting inclusion criteria	Title and abstract screening
2278	Hikisz P, Jacenik D. The Tobacco Smoke Component, Acrolein, as a Major Culprit in Lung Diseases and Respiratory Cancers: Molecular Mechanisms of Acrolein Cytotoxic Activity. Cells. 2023;12. doi:10.3390/cells12060879	No	Not meeting inclusion criteria	Title and abstract screening
2279	Hill K., Welsh D.A., Richey L., Lin H., Apolzan J., Celestin M., et al. Survey of physician approach to treatment of tobacco dependence. Am J Respir Crit Care Med. 2021;203. doi:10.1164/ajrccm-conference.2021.203.1_MeetingAbstracts.A1649	No	Not meeting inclusion criteria	Title and abstract screening
2280	Hillyer G.C., Mapanga W., Jacobson J.S., Graham A., Mmoledi K., Makhutle R., et al. Attitudes toward tobacco cessation and lung cancer screening in two South African communities. Glob Public Health. 2020;15: 1537–1550. doi:10.1080/17441692.2020.1761425	No	Not meeting inclusion criteria	Title and abstract screening
2281	Hillyer GC, Nazareth M, Lima S, Schmitt KM, Reyes A, Fleck E, et al. E-cigarette Use Among Young Adult Patients: The Opportunity to Intervene on Risky Lifestyle Behaviors to Reduce Cancer Risk. J Community Health. 2022;47: 94–100. doi:10.1007/s10900-021-01027-7	No	Not meeting inclusion criteria	Title and abstract screening
2282	Hindocha C, McClure EA. Unknown population-level harms of cannabis and tobacco co-use: if you don't measure it, you can't manage it. Addiction. 2021;116: 1622–1630. doi:10.1111/add.15290	No	Not meeting inclusion criteria	Title and abstract screening
2283	Hinds JT, Loukas A, Perry CL. Characterizing the polytobacco behaviors of sexual minority young adult college students. Drug Alcohol Depend. 2020;213: 108126. doi:10.1016/j.drugalcdep.2020.108126	No	Not meeting inclusion criteria	Title and abstract screening
2284	Hirai T, Fujiyoshi K, Yamada S, Matsumoto T, Kikuchi J, Ishida K, et al. Advanced Glycation End Products Are Associated with Diabetes Status and Physical Functions in Patients with Cardiovascular Disease. Nutrients. 2022;14: 3032–3032. doi:10.3390/nu14153032	No	Not meeting inclusion criteria	Title and abstract screening
2285	Hiramoto S, Taniyama T, Kikuchi A, Hori T, Yoshioka A, Inoue A. Effects of molecular targeting agents and immune-checkpoint inhibitors in patients with advanced cancer who are near the end of life. Palliat Support Care. 2021;19: 709–714. doi:10.1017/S147895152100002X	No	Not meeting inclusion criteria	Title and abstract screening
2286	Hirata M, Kobayashi K. Experiences with the end-of-life decision-making process in children with cancer, their parents, and healthcare professionals: A systematic review and meta-ethnography. J Pediatr Nurs. 2023;69: e45–e64. doi:10.1016/j.pedn.2022.12.004	No	Not meeting inclusion criteria	Title and abstract screening
2287	Hiratsuka Y, Oishi T, Miyashita M, Morita T, Mack JW, Sato Y, et al. Factors related to specialized palliative care use and aggressive care at end of life in Japanese patients with advanced solid cancers: a cohort study. Support Care Cancer. 2021;29: 7805–7813. doi:10.1007/s00520-021-06364-w	No	Not meeting inclusion criteria	Title and abstract screening
2288	Hirooka K, Okumura Y, Matsumoto S, Fukahori H, Ogawa A. Quality of End-of-Life in Cancer Patients With Dementia: Using A Nationwide Inpatient Database. J Pain Symptom Manage. 2022;64: 1–7. doi:10.1016/j.jpainsymman.2022.03.016	No	Not meeting inclusion criteria	Title and abstract screening
2289	Hirsch J, Schwartz R. Structural Conditions as Cause: Explaining the Rapid Rise in Youth E-Cigarette Use by Re-thinking Models of Addiction. Subst Use Misuse. 2021;56: 1892–1899. doi:10.1080/10826084.2021.1954022	No	Not meeting inclusion criteria	Title and abstract screening
2290	Hirschhorn N. Another perspective on the Foundation for a Smoke-Free World. The Lancet. 2018;391: 25. doi:10.1016/S0140-6736%2817%2933312-3	No	Not meeting inclusion criteria	Title and abstract screening
2291	Hirvonen J, Zanotti-Fregonara P, Gorelick DA, Lyoo CH, Rallis-Frutos D, Morse C, et al. Decreased Cannabinoid CB(1) Receptors in Male Tobacco Smokers Examined With Positron Emission Tomography. Biol Psychiatry. 2018;84: 715–721. doi:10.1016/j.biopsych.2018.07.009	No	Not meeting inclusion criteria	Title and abstract screening
2292	Hiscock R, Augustin NH, Branston JR, Gilmore AB. Longitudinal evaluation of the impact of standardised packaging and minimum excise tax on tobacco sales and industry revenue in the UK. Tob Control. 2021;30: 515–522. doi:10.1136/tobaccocontrol-2019-055387	No	Not meeting inclusion criteria	Title and abstract screening
2293	Ho F, Kow A, Lim WC, Chen MZ, Rais NCM, Ling NMW, et al. Feasibility of a Geriatric Oncology Longitudinal End to End (GOLDEN) Program in a Tertiary Cancer Center in Singapore. Oncologist. 2023;28: e198–e204. doi:10.1093/oncolo/oyac276	No	Not meeting inclusion criteria	Title and abstract screening
2294	Ho JTK, Tyndale RF, Baker TB, Amos CI, Chiu A, Smock N, et al. Racial disparities in intensity of smoke exposure and nicotine intake among low-dependence smokers. Drug Alcohol Depend. 2021;221: 108641. doi:10.1016/j.drugalcdep.2021.108641	No	Not meeting inclusion criteria	Title and abstract screening
2295	Ho KY, Li WHC, Wang MP, Lam KKW, Lam TH, Chan SSC. Comparison of two approaches in achieving smoking abstinence among patients in an outpatient clinic: A Phase 2 randomized controlled trial. Patient Educ Couns. 2018;101: 885–893. doi:10.1016/j.pec.2018.02.003	No	Not meeting inclusion criteria	Title and abstract screening
2296	Ho M.S., Ho M., Librach C. iPSC: BEYOND SMOKE SIGNALS: METABOLOMIC ALTERATIONS AND PARACRINE SIGNALING UNDERLIE VAPING LIQUID MEDIATED DISRUPTION OF HUMAN INDUCED PLURIPOTENT STEM CELLS (HIPSCS) DERIVED ENDOTHELIAL AND SMOOTH MUSCLE CELLS RECIPROCAL CROSS-TALK. Cytotherapy. 2022;24: S150. doi:10.1016/S1465-3249%2822%2900397-8	No	Not meeting inclusion criteria	Title and abstract screening
2297	Hobkirk A.L., Houser K.R., Hoglen B., Bitzer Z.T., Fendrich A., Bordner C.R., et al. Evidence from an fMRI study that dessert-flavored e-cigarettes engage taste-related, but not smoking-related, brain circuitry for female daily smokers. Exp Clin Psychopharmacol. 2021. doi:10.1037/pha0000488	No	Not meeting inclusion criteria	Title and abstract screening
2298	Hobkirk AL, Bitzer Z, Goel R, Sica CT, Livelsberger C, Yingst J, et al. An Electronic Aerosol Delivery System for Functional Magnetic Resonance Imaging. Subst Abuse Res Treat. 2020;14: 1178221820904140. doi:10.1177/1178221820904140	No	Not meeting inclusion criteria	Title and abstract screening
2299	Hobkirk AL, Hoglen B, Sheng T, Kristich A, Yingst JM, Houser KR, et al. Intentions and Attempts to Quit JUUL E-Cigarette Use: The Role of Perceived Harm and Addiction. Prev Chronic Dis. 2022;19: E06. doi:10.5888/pcd19.210255	No	Not meeting inclusion criteria	Title and abstract screening
2300	Hoerger M, Gramling R, Epstein R, Fenton JJ, Mohile SG, Kravitz RL, et al. Patient, caregiver, and oncologist predictions of quality of life in advanced cancer: Accuracy and associations with end-of-life care and caregiver bereavement. Psychooncology. 2022;31: 978–984. doi:10.1002/pon.5887	No	Not meeting inclusion criteria	Title and abstract screening
2301	Hoetger C., Bono R.S., Nicksic N.E., Barnes A.J., Cobb C.O. Influence of electronic cigarette characteristics on susceptibility, perceptions, and abuse liability indices among combustible tobacco cigarette smokers and non-smokers. Int J Environ Res Public Health. 2019;16: 1825. doi:10.3390/ijerph16101825	No	Not meeting inclusion criteria	Title and abstract screening

2302	Hoffman BL, Wolynn R, Barrett E, Manganello JA, Felter EM, Sidani JE, et al. Viewer reactions to EVALI storylines on popular medical dramas: A thematic analysis of Twitter messages. J Health Commun. 2023;28: 282–291. doi:10.1080/10810730.2023.2201814	No	Not meeting inclusion criteria	Title and abstract screening
2303	Hoffmeyer N, Hinton A, Wagener TL, Tackett AP. Correlates of youth Poly-E-Cigarette device use. Addict Behav. 2022;129: 107248. doi:10.1016/j.addbeh.2022.107248	No	Not meeting inclusion criteria	Title and abstract screening
2304	Hogarth L, Mathew AR, Hitsman B. Current major depression is associated with greater sensitivity to the motivational effect of both negative mood induction and abstinence on tobacco-seeking behavior. Drug Alcohol Depend. 2017;176: 1–6. doi:10.1016/j.drugalcdep.2017.02.009	No	Not meeting inclusion criteria	Title and abstract screening
2305	Hohenadel I.A., Siegel E. From vaping and smoking: Risk assessment of new tobacco and nicotine releasing systems. Atemwegs- Lungenkrankh. 2022;48: 105–110. doi:10.5414/ATX2596	No	Not meeting inclusion criteria	Title and abstract screening
2306	Holden S.K., Domen C.H., Sillau S., Liu Y., Leehey M.A. Higher Risk, Higher Reward? Self-Reported Effects of Real-World Cannabis Use in Parkinson's Disease. Mov Disord Clin Pract. 2022;9: 340–350. doi:10.1002/mdc3.13414	No	Not meeting inclusion criteria	Title and abstract screening
2307	Hollis A, Downey E, Standing S, Leahy J, Ebbert K, Ganesh A. A vaping risks education program for school students: Evaluation of the solve mystery toolkit. Prev Med Rep. 2022;28: 101852. doi:10.1016/j.pmedr.2022.101852	No	Not meeting inclusion criteria	Title and abstract screening
2308	Holt A., Anbil A., Combs M., Underwood M., Rudy A., Sawyer A., et al. Impact of vaping ethanol on the roadside field sobriety test in a clinical setting. Toxicol Anal Clin. 2022;34: S75–S76. doi:10.1016/j.toxac.2022.06.105	No	Not meeting inclusion criteria	Title and abstract screening
2309	Holt AK, Anbil A, Combs MM, Sales ER, Boone EL, Poklis JL, et al. The impact of vaping ethanol-containing electronic cigarette liquids on roadside impairment investigations. J Anal Toxicol. 2023;47: 737–745. doi:10.1093/jat/bkad049	No	Not meeting inclusion criteria	Title and abstract screening
2310	Honeycutt L, Huerne K, Miller A, Wennberg E, Filion KB, Grad R, et al. A systematic review of the effects of e-cigarette use on lung function. NPJ Prim Care Respir Med. 2022;32: 45. doi:10.1038/s41533-022-00311-w	No	Not meeting inclusion criteria	Title and abstract screening
2311	Hong T., Wu J., Wijaya D., Xuan Z., Fetterman J.L. JUUL the heartbreaker: Twitter analysis of cardiovascular health perceptions of vaping. Tob Induc Dis. 2021;19: 1–6. doi:10.18332/TID/130961	No	Not meeting inclusion criteria	Title and abstract screening
2312	Hood-Medland EA, Dove MS, Tong EK. Assessment and Counseling Gaps Among Former Smokers Eligible for Lung Cancer Screening in US Adults : A Cross-Sectional Analysis of National Health and Nutrition Examination Surveys (NHANES), 2013-2018. J Gen Intern Med. 2022;37: 2711–2718. doi:10.1007/s11606-022-07542-0	No	Not meeting inclusion criteria	Title and abstract screening
2313	Hooshiar S, Esmaili H, Taherian A, Jafarnejad S. Exercise, Advanced Glycation End Products, and Their Effects on Cardiovascular Disorders: A Narrative Review. Heart Mind. 2022;6: 139–150. doi:10.4103/hm.hm_31_22	No	Not meeting inclusion criteria	Title and abstract screening
2314	Hoover LV, Yu HP, Cummings JR, Ferguson SG, Gearhardt AN. Co-occurrence of food addiction, obesity, problematic substance use, and parental history of problematic alcohol use. Psychol Addict Behav. 2022. doi:10.1037/adb0000870	No	Not meeting inclusion criteria	Title and abstract screening
2315	Hoover LV, Yu HP, Cummings JR, Ferguson SG, Gearhardt AN. Co-occurrence of food addiction, obesity, problematic substance use, and parental history of problematic alcohol use. Psychol Addict Behav. 2023;37: 928–935. doi:10.1037/adb0000870	No	Not meeting inclusion criteria	Title and abstract screening
2316	Hope TA, Calais J. PSMA PET in Prostate Cancer—A Biomarker or a Surrogate End Point?—Reply...Bowling GC, Dimitrakoff JD. PSMA PET in Prostate Cancer—A Biomarker or a Surrogate End Point? JAMA Oncology. 2022;8(4):1. JAMA Oncol. 2022;8: 1–1. doi:10.1001/jamaoncol.2021.7994	No	Not meeting inclusion criteria	Title and abstract screening
2317	Hopkinson N.S. The prominence of e-cigarettes is a symptom of decades of failure to tackle smoking properly. BMJ Online. 2019;364: l647. doi:10.1136/bmj.l647	No	Not meeting inclusion criteria	Title and abstract screening
2318	Horsfall LJ, Clarke CS, Nazareth I, Ambler G. The value of blood-based measures of liver function and urate in lung cancer risk prediction: A cohort study and health economic analysis. Cancer Epidemiol. 2023;84: 102354. doi:10.1016/j.canep.2023.102354	No	Not meeting inclusion criteria	Title and abstract screening
2319	Hoshina Y. E-cigarette or vaping product use-associated lung injury: A great COVID-19 mimicker in young adult. Clin Case Rep. 2021;9: e05016. doi:10.1002/ccr3.5016	No	Not meeting inclusion criteria	Title and abstract screening
2320	Hou Z, Huang S, Li Z. Androgens in prostate cancer: A tale that never ends. Cancer Lett. 2021;516: 1–12. doi:10.1016/j.canlet.2021.04.010	No	Not meeting inclusion criteria	Title and abstract screening
2321	Hryhorskyj L, Howle F, Groom K, Moore R, Clegg H, Shackley D, et al. Patient survey examining the experience of care of a hospital-based opt-out tobacco dependency treatment service (the CURE Project). BMJ Open Respir Res. 2022;9. doi:10.1136/bmjresp-2022-001334	No	Not meeting inclusion criteria	Title and abstract screening
2322	Hsiao Y-C, Matulewicz RS, Sherman SE, Jaspers I, Weitzman ML, Gordon T, et al. Untargeted Metabolomics to Characterize the Urinary Chemical Landscape of E-Cigarette Users. Chem Res Toxicol. 2023;36: 630–642. doi:10.1021/acs.chemrestox.2c00346	No	Not meeting inclusion criteria	Title and abstract screening
2323	Hswen Y, Yom-Tov E. Analysis of a Vaping-Associated Lung Injury Outbreak through Participatory Surveillance and Archival Internet Data. Int J Env Res Public Health. 2021;18. doi:10.3390/ijerph18158203	No	Not meeting inclusion criteria	Title and abstract screening
2324	Hu L, Chu Q, Fan Z, Chen Y. Discussion of advance care planning on end-of-life decisions with lung cancer patients in Wuhan, China: attitude, timing and future directions. Intern Med J. 2021;51: 2111–2118. doi:10.1111/imj.14958	No	Not meeting inclusion criteria	Title and abstract screening
2325	Hu SS, Wang TW, Homa DM, Tsai J, Neff L. Cigarettes, Smokeless Tobacco, and E-Cigarettes: State-Specific Use Patterns Among U.S. Adults, 2017-2018. Am J Prev Med. 2022;62: 930–942. doi:10.1016/j.amepre.2021.12.014	No	Not meeting inclusion criteria	Title and abstract screening
2326	Hu X, Deng K, Ye H, Sun Z, Huang W, Sun Y, et al. Trends in Tumor Site-Specific Survival of Bone Sarcomas from 1980 to 2018: A Surveillance, Epidemiology and End Results-Based Study. Cancers. 2021;13: 5381. doi:10.3390/cancers13215381	No	Not meeting inclusion criteria	Title and abstract screening
2327	Hu Y, Zhao Z, Harmon T, Pentel PR, Ehrich M, Zhang C. Paradox of PEGylation in fabricating hybrid nanoparticle-based nicotine vaccines. Biomaterials. 2018;182: 72–81. doi:10.1016/j.biomaterials.2018.08.015	No	Not meeting inclusion criteria	Title and abstract screening
2328	Huang F, Xu G, Du H. A New Nomogram for Predicting Overall Survival and Assisting Postoperative Adjuvant Treatment Decision-Making in Stage II Oral Tongue Squamous Cell Carcinoma: A Surveillance, Epidemiology and End Results (SEER) Database Analysis. J Oral Maxillofac Surg 02782391. 2021;79: 2147–2154. doi:10.1016/j.joms.2021.04.010	No	Not meeting inclusion criteria	Title and abstract screening
2329	Huang S, Tang O, Zheng X, Li H, Wu Y, Yang L. Effectiveness of smoking cessation on the high-risk population of lung cancer with early screening: a systematic review and meta-analysis of randomized controlled trials until January 2022. Arch Public Health Arch Belg Sante Publique. 2023;81: 101. doi:10.1186/s13690-023-01111-5	No	Not meeting inclusion criteria	Title and abstract screening
2330	Huerne K., Eisenberg M.J. Vaping Cessation Interventions in Former Smokers: A Review. Can J Cardiol. 2023. doi:10.1016/j.cjca.2023.04.020	No	Not meeting inclusion criteria	Title and abstract screening

2331	Huey S, Granitto M. Escape the vape: Health hazards of the latest nicotine craze. Nursing (Lond). 2017;47: 46–51. doi:10.1097/01.NURSE.0000510746.39843.e6	No	Not meeting inclusion criteria	Title and abstract screening
2332	Huey SW, Granitto MH. Smoke screen: The teen vaping epidemic uncovers a new concerning addiction. J Am Assoc Nurse Pract. 2020;32: 293–298. doi:10.1097/JXX.0000000000000234	No	Not meeting inclusion criteria	Title and abstract screening
2333	Hugar LA, Yabes JG, Filippou P, Wulff-Burchfield EM, Lopa SH, Gore J, et al. High-intensity end-of-life care among Medicare beneficiaries with bladder cancer. Urol Oncol. 2021;39: 731.e17-731.e24. doi:10.1016/j.urolonc.2021.02.008	No	Not meeting inclusion criteria	Title and abstract screening
2334	Hughes J, Sykes G, Hughes K, O'Reilly M, Goodwin J, Sutton C, et al. From gateways to multilinear connections: A qualitative longitudinal investigation of the relationships between vaping and smoking among adolescent users. Int J Drug Policy. 2021;97: 103341. doi:10.1016/j.drugpo.2021.103341	No	Not meeting inclusion criteria	Title and abstract screening
2335	Hughes J.R. Better Understanding Harm Reduction. Nicotine Tob Res. 2021;23: 779. doi:10.1093/ntr/ntab021	No	Not meeting inclusion criteria	Title and abstract screening
2336	Hughes JR, Shiffman S, Naud S, Peters EN. Day-to-day variability in self-reported cigarettes per day. Nicotine Tob Res. 2017;19: 1107–1111. doi:10.1093/ntr/ntx057https://dx.doi.org/10.1093/ntr/ntx057	No	Not meeting inclusion criteria	Title and abstract screening
2337	Huh J, Yu S, Galimov A, Meza LR, Galstyan E, Medel D, et al. Hypothetical flavour ban and intention to vape among vape shop customers: the role of flavour preference and e-cigarette dependence. Tob Control. 2021. doi:10.1136/tobaccocontrol-2020-056321	No	Not meeting inclusion criteria	Title and abstract screening
2338	Huizink AC. Trends and associated risks in adolescent substance use: E-cigarette use and nitrous oxide use. Curr Opin Psychol. 2022;45: 101312. doi:10.1016/j.copsyc.2022.101312	No	Not meeting inclusion criteria	Title and abstract screening
2339	Hung M, Spencer A, Hon ES, Licari FW, Cheever VJ, Moffat R, et al. E-cigarette addiction and harm perception: Does initiation flavor choice matter?. BMC Public Health. 2022;22: 1780. doi:10.1186/s12889-022-14166-w	No	Not meeting inclusion criteria	Title and abstract screening
2340	Hurt RT, Ebbert JO, Croghan IT, Schroeder DR, Hurt RD, Hays JT. Varenicline for tobacco-dependence treatment in alcohol-dependent smokers: A randomized controlled trial. Drug Alcohol Depend. 2018;184: 12–17. doi:10.1016/j.drugalcdep.2017.11.017	No	Not meeting inclusion criteria	Title and abstract screening
2341	Hussain H., Sehring M., Aulakh B.S. COVID-19-Associated Coagulopathy: A Case Report of Thrombosis despite Therapeutic Anticoagulation. Case Rep Crit Care. 2020;2020: 8876932. doi:10.1155/2020/8876932	No	Not meeting inclusion criteria	Title and abstract screening
2342	Hussain H., Sehring M., Taneja D. SMOKE EVERYWHERE, FROM LUNGS TO SPINE! Chest. 2020;158: A950. doi:10.1016/j.chest.2020.08.885	No	Not meeting inclusion criteria	Title and abstract screening
2343	Hussain S., Shahid Z., Foroozesh M., Sofi U. E-cigarettes: A novel therapy or a looming catastrophe. Ann Thorac Med. 2021;16: 73–80. doi:10.4103/atm.ATM_190_20	No	Not meeting inclusion criteria	Title and abstract screening
2344	Huynh A, Wisk LE. Application of behavioral economics for understanding health behaviors among adolescents and young adults. Curr Opin Pediatr. 2022;34: 326–333. doi:10.1097/MOP.0000000000001126	No	Not meeting inclusion criteria	Title and abstract screening
2345	Huynh E.M., Ghazala Z., Jagana R., Gokarakonda S. 43.3 Cannabis Vaping in Adolescents: A Review of Prevalence, Comorbidities, Appraisal of Lung Injury, and Long-Term Complications. J Am Acad Child Adolesc Psychiatry. 2021;60: S232. doi:10.1016/j.jaac.2021.09.325	No	Not meeting inclusion criteria	Title and abstract screening
2346	Hwang J. Subjective Changes in Tobacco Product Use among Korean Adults during the COVID-19 Pandemic. Int J Env Res Public Health. 2022;19. doi:10.3390/ijerph19063272	No	Not meeting inclusion criteria	Title and abstract screening
2347	Hwang J, Chun HR, Cheon E. A qualitative study on the impact of COVID-19 on the behavior and attitudes of smokers and non-smokers in South Korea. BMC Public Health. 2021;21: 1972. doi:10.1186/s12889-021-12079-8	No	Not meeting inclusion criteria	Title and abstract screening
2348	Hwang JH, Ryu DH, Park SW. Heated tobacco products: Cigarette complements, not substitutes. Drug Alcohol Depend. 2019;204: 107576. doi:10.1016/j.drugalcdep.2019.107576	No	Not meeting inclusion criteria	Title and abstract screening
2349	Hwang JS, Lee CM, Lee K, Kim CY. Nicotine Dependence Evaluated by Urinary Cotinine and Heaviness of Smoking Index among Smokers, Vapers, and Dual Users: A Cross-Sectional Study Using the Korea National Health and Nutrition Examination Survey Data. Korean J Fam Med. 2021;42: 197–203. doi:10.4082/kjfm.20.0056	No	Not meeting inclusion criteria	Title and abstract screening
2350	Iacobucci G. Vaping: BMA to review effects of "growing public health epidemic". BMJ. 2023;382: 1560. doi:10.1136/bmj.p1560	No	Not meeting inclusion criteria	Title and abstract screening
2351	Ichikura K, Matsuoka S, Chiba H, Ishida H, Fukase Y, Murase H, et al. Health care providers' perspectives on providing end-of-life psychiatric care in cardiology and oncology hospitals: a cross-sectional questionnaire survey. BMC Palliat Care. 2023;22: 1–10. doi:10.1186/s12904-023-01138-z	No	Not meeting inclusion criteria	Title and abstract screening
2352	Ickes M, Hester JW, Wiggins AT, Rayens MK, Hahn EJ, Kavuluru R. Prevalence and reasons for Juul use among college students. J Am Coll Health J ACH. 2020;68: 455–459. doi:10.1080/07448481.2019.1577867	No	Not meeting inclusion criteria	Title and abstract screening
2353	Iida A., Fujiwara Y., Nojima T., Naito H., Nakao A., Mikane T. Cardiac arrest due to liquid nicotine intoxication: a case report. Acute Med Surg. 2021;8: e720. doi:10.1002/ams2.720	No	Not meeting inclusion criteria	Title and abstract screening
2354	Iizuka-Honma H, Mitsumori T, Yoshikawa S, Takizawa H, Noguchi M. Factors Associated With Survival in Patients With End-of-Life Hematological Malignancies Who Received Chemotherapy: A Retrospective Single-Institution Study. Am J Hosp Palliat Med. 2023;40: 812–819. doi:10.1177/10499091221142502	No	Not meeting inclusion criteria	Title and abstract screening
2355	Ikander T, Raunkiær M, Hansen O, Dieperink KB. Nurses' involvement in end-of-life discussions with incurable cancer patients and family caregivers: An integrative review. Palliat Support Care. 2022;20: 570–581. doi:10.1017/S1478951521000596	No	Not meeting inclusion criteria	Title and abstract screening
2356	Ikeda S., Takeda M., Sato K., Miki K., Fukuda K., Shiba N. A case of vasospastic angina with exertional sign. J Cardiol Cases. 2021;24: 199–202. doi:10.1016/j.jccase.2021.04.004	No	Not meeting inclusion criteria	Title and abstract screening
2357	Ikegwuonu T, Hilton S, Smith KE, Buckton CH, Wong M, Weishaar HB. Understanding commercial actors' engagement in policy debates on proposed e-cigarette regulation in Scotland. Tob Control. 2022;31: 511–519. doi:10.1136/tobaccocontrol-2020-056084	No	Not meeting inclusion criteria	Title and abstract screening
2358	Ilakkuvan V, Johnson A, Villanti AC, Evans WD, Turner M. Patterns of Social Media Use and Their Relationship to Health Risks Among Young Adults. J Adolesc Health. 2019;64: 158–164. doi:10.1016/j.jadohealth.2018.06.025	No	Not meeting inclusion criteria	Title and abstract screening
2359	Iles IA, Gillman AS, Klein WMP, Ferrer RA, Kaufman A. Associations between absolute and relative electronic cigarette harm perceptions and information-seeking behaviours among US adult current, former and never smokers. Drug Alcohol Rev. 2022;41: 356–364. doi:10.1111/dar.13368	No	Not meeting inclusion criteria	Title and abstract screening

2360	Imaoka D. Educating staff nurses on e-cigarette and vaping associated lung injury. Diss Abstr Int Sect B Sci Eng. 2022;83: No-Specified.	No	Not meeting inclusion criteria	Title and abstract screening
2361	Imbriani M.E., Catino A., Gasparre V., Calabrese N., Zamparella M., Bafunno D., et al. Tabagism and cessation programs in primary care. Tumori. 2019;105: 185. doi:10.1177/0300891619872589	No	Not meeting inclusion criteria	Title and abstract screening
2362	Indralingam R, Rak K, Richardson A, Crowley-Matoka M, White D, Arnold R, et al. Overcoming End of Life Discussion Hesitancy in Advance Care Planning for Patients with Advanced Cancer and Their Caregivers (RP309). J Pain Symptom Manage. 2022;63: 1072–1072. doi:10.1016/j.jpainsymman.2022.04.025	No	Not meeting inclusion criteria	Title and abstract screening
2363	Ingelfinger JR. Childhood Risk Factors and Prediction of Adult Cardiovascular End Points. N Engl J Med. 2022;386: 1948–1949. doi:10.1056/NEJMe2203743	No	Not meeting inclusion criteria	Title and abstract screening
2364	Insel P.A., Blaschke T.F., Amara S.G., Meyer U.A. Introduction to the Theme “New Insights, Strategies, and Therapeutics for Common Diseases.” Annu Rev Pharmacol Toxicol. 2022;62: 19–24. doi:10.1146/annurev-pharmtox-091421-094627	No	Not meeting inclusion criteria	Title and abstract screening
2365	Introne WJ, Huizing M, Malicdan MCV, O’Brien KJ, Gahl WA. Hermansky-Pudlak Syndrome. 1993.	No	Not meeting inclusion criteria	Title and abstract screening
2366	Ioakeimidis N, Vlachopoulos C, Georgakopoulos C, Abdelrasoul M, Skliros N, Katsi V, et al. Smoking cessation rates with varenicline and electronic cigarettes in relapsed smokers with a history of acute coronary syndrome. 2018;39: 242-. doi:10.1093/eurheartj/ehy565.P1234	No	Not meeting inclusion criteria	Title and abstract screening
2367	Ioakeimidis N., Vlachopoulos C., Abdelrasoul M., Georgakopoulos C., Terentes-Printzios D., Maggas N., et al. Aortic stiffness is significantly increased in low-nicotine dependent smokers after the use of both combustible and electronic cigarettes. Eur Heart J. 2017;38: 709. doi:10.1093/eurheartj/ehx502.P3406	No	Not meeting inclusion criteria	Title and abstract screening
2368	Ioakeimidis N., Vlachopoulos C., Georgakopoulos C., Dima I., Solomou E., Gardikioti V., et al. Two-year therapeutic effectiveness of pharmacotherapy versus electronic cigarettes for smoking cessation: A single-center experience. Eur Heart J. 2019;40: 2627. doi:10.1093/eurheartj/ehz745.0799	No	Not meeting inclusion criteria	Title and abstract screening
2369	Irajpour A, Hashemi M, Taleghani F. Clinical practice guideline for end-of-life care in patients with cancer: a modified ADAPTE process. Support Care Cancer. 2022;30: 2497–2505. doi:10.1007/s00520-021-06558-2	No	Not meeting inclusion criteria	Title and abstract screening
2370	IRCT20150919024080N19. Effect of Aromatherapy in CIPN and Fatigue. 2021. Available: https://www.cochranelibrary.com/central/doi/10.1002/central/CN-02329408/full	No	Not meeting inclusion criteria	Title and abstract screening
2371	Isett KR, Rosenblum S, Barna JA, Hicks D, Gilbert GH, Melkers J. Missed opportunities for detecting alternative nicotine product use in youth: Data from the National Dental Practice-Based Research Network. J Adolesc Health. 2018;63: 587–593. doi:10.1016/j.jadohealth.2018.06.007https://dx.doi.org/10.1016/j.jadohealth.2018.06.007	No	Not meeting inclusion criteria	Title and abstract screening
2372	Iskandar A., Zanetti F., Giralt A., Kondylis A., Sewer A., Ortega-Torres L., et al. An in vitro systems toxicology assessment of novel electronic vapor device by using air-liquid interface buccal, bronchial, small airway, and alveolar culture models. Naunyn Schmiedebergs Arch Pharmacol. 2021;394: S39. doi:10.1007/s00210-021-02066-6	No	Not meeting inclusion criteria	Title and abstract screening
2373	Islam S, Thompson K, Abadi M, Wharton K, Lipperman-Kreda S. “I don’t know whose mouth has been on this”: youth nicotine and cannabis vaping practices in the context of the COVID-19 pandemic. BMC Public Health. 2022;22: 1808. doi:10.1186/s12889-022-14201-w	No	Not meeting inclusion criteria	Title and abstract screening
2374	Islam T, Eckel S, Liu F, Barrington-Trimis J, Harlow AF, Benowitz N, et al. Effects of dual use of e-cigarette and cannabis during adolescence on cigarette use in young adulthood. Tob Control. 2023. doi:10.1136/tc-2022-057542	No	Not meeting inclusion criteria	Title and abstract screening
2375	Ismail A, Berdine G, Nugent K. Subpleural sparing: Clinical, physiological, and radiological implications. Am J Med Sci. 2023;365: 219–225. doi:10.1016/j.amjms.2022.11.002	No	Not meeting inclusion criteria	Title and abstract screening
2376	Ismail A., Berdine G., Nugent K. Subpleural sparing: clinical, physiological, and radiological implications. Am J Med Sci. 2022. doi:10.1016/j.amjms.2022.11.002	No	Not meeting inclusion criteria	Title and abstract screening
2377	ISRCTN12950872. A study to evaluate the safety, tolerability, and processing by the body of single-ascending doses of RO7490677 in healthy participants. 2022. Available: https://www.cochranelibrary.com/central/doi/10.1002/central/CN-02429826/full	No	Not meeting inclusion criteria	Title and abstract screening
2378	ISRCTN13158982. Enhancing dental health advice. 2022. Available: https://www.cochranelibrary.com/central/doi/10.1002/central/CN-02411561/full	No	Not meeting inclusion criteria	Title and abstract screening
2379	ISRCTN14041907. MECHANISMS Study: using game theory to assess the effects of social norms and social networks on adolescent smoking in schools. 2023. Available: https://www.cochranelibrary.com/central/doi/10.1002/central/CN-02524971/full	No	Not meeting inclusion criteria	Title and abstract screening
2380	ISRCTN14643179. Assessing if stopping and starting standard of care medication for later stage melanoma can reduce the body’s resistance to the treatment. 2023. Available: https://www.cochranelibrary.com/central/doi/10.1002/central/CN-02591983/full	No	Not meeting inclusion criteria	Title and abstract screening
2381	ISRCTN16455577. Is it possible to reduce smoking at Danish vocational high schools? 2018. Available: https://www.cochranelibrary.com/central/doi/10.1002/central/CN-01904790/full	No	Not meeting inclusion criteria	Title and abstract screening
2382	ISRCTN17715270. A study to investigate the safety, tolerability and concentration in the blood of nicotine compared between different types of nicotine replacement therapies (NRTs). 2022. Available: https://www.cochranelibrary.com/central/doi/10.1002/central/CN-02452631/full	No	Not meeting inclusion criteria	Title and abstract screening
2383	ISRCTN63825779. Yorkshire Enhanced Stop Smoking (YESS). 2018. Available: https://www.cochranelibrary.com/central/doi/10.1002/central/CN-01907084/full	No	Not meeting inclusion criteria	Title and abstract screening
2384	ISRCTN82413824. Effectiveness of electronic cigarettes compared with combination nicotine replacement therapy for smoking cessation in patients with chronic obstructive pulmonary disease and effect on lung health (ECAL Trial). 2023. Available: https://www.cochranelibrary.com/central/doi/10.1002/central/CN-02603333/full	No	Not meeting inclusion criteria	Title and abstract screening
2385	ISRCTN93764730. FEASIBILITY of IBIS 3. An International Breast Intervention Study investigating prevention of late recurrence in ER+ breast cancer survivors following 5 years of adjuvant treatment. 2014. Available: https://www.cochranelibrary.com/central/doi/10.1002/central/CN-02444299/full	No	Not meeting inclusion criteria	Title and abstract screening
2386	ISRCTN94991276. DETECTION Trial: using blood tests to guide early treatment of relapse in early stage melanoma. 2021. Available: https://www.cochranelibrary.com/central/doi/10.1002/central/CN-02330077/full	No	Not meeting inclusion criteria	Title and abstract screening
2387	Issabakhsh M, Meza R, Li Y, Yuan Z, Sanchez-Romero LM, Levy DT. Public health impact of a US menthol cigarette ban on the non-Hispanic black population: a simulation study. Tob Control. 2022. doi:10.1136/tobaccocontrol-2022-057298	No	Not meeting inclusion criteria	Title and abstract screening
2388	Issabakhsh M, Meza R, Li Y, Yuan Z, Sanchez-Romero LM, Levy DT. Public health impact of a US menthol cigarette ban on the non-Hispanic black population: a simulation study. Tob Control. 2023;33: 126–130. doi:10.1136/tobaccocontrol-2022-057298	No	Not meeting inclusion criteria	Title and abstract screening

2389	Issabakhsh M, Sánchez-Romero LM, Le TTT, Liber AC, Tan J, Li Y, et al. Machine learning application for predicting smoking cessation among US adults: An analysis of waves 1-3 of the PATH study. PLoS One. 2023;18: e0286883. doi:10.1371/journal.pone.0286883	No	Not meeting inclusion criteria	Title and abstract screening
2390	Ito N., Masuda T., Yamaguchi K., Sakamoto S., Horimasu Y., Nakashima T., et al. Pneumatosis Intestinalis following Radiation Esophagitis during Chemoradiotherapy for Lung Cancer: A Case Report. Case Rep Oncol. 2021;14: 1454–1459. doi:10.1159/000518315	No	Not meeting inclusion criteria	Title and abstract screening
2391	Ivzori Erel A, Cohen M. "No place like home?" A qualitative study of the experience of sense of place among cancer patients near the end of life. Health Soc Care Community. 2022;30: e1194–e1201. doi:10.1111/hsc.13526	No	Not meeting inclusion criteria	Title and abstract screening
2392	Jaafar A., Albarazanchi A., Kadhim M.J., Darvin M.E., Vaczi T., Tuchin V.V., et al. Impact of e-cigarette liquid on porcine lung tissue - ex vivo confocal Raman micro-spectroscopy study. J Biophotonics. 2023; e202300336. doi:10.1002/jbio.202300336	No	Not meeting inclusion criteria	Title and abstract screening
2393	Jabba S.V., Diaz A.N., Erythropel H.C., Zimmerman J.B., Jordt S.E. Chemical adducts of flavor aldehydes formed in e-cigarette liquids are cytotoxic and inhibit mitochondrial function in respiratory epithelial cells. Chem Senses. 2021;46. doi:10.1093/chemse/bjab034/6376000	No	Not meeting inclusion criteria	Title and abstract screening
2394	Jabbour SK, Hochster HS. Evolving paradigms in locally advanced rectal cancer: the means justify the ends. JNCI J Natl Cancer Inst. 2023;115: 1439–1441. doi:10.1093/jnci/djad196	No	Not meeting inclusion criteria	Title and abstract screening
2395	Jackson A, Grobman B, Krishnan-Sarin S. Recent findings in the pharmacology of inhaled nicotine: Preclinical and clinical in vivo studies. Neuropharmacology. 2020;176: 108218. doi:10.1016/j.neuropharm.2020.108218	No	Not meeting inclusion criteria	Title and abstract screening
2396	Jackson A., Green B., Erythropel H.C., Kong G., Cavallo D.A., Eid T., et al. Influence of menthol and green apple e-liquids containing different nicotine concentrations among youth e-cigarette users. Exp Clin Psychopharmacol. 2021;29: 355–365. doi:10.1037/pha0000368	No	Not meeting inclusion criteria	Title and abstract screening
2397	Jackson CD, Carter J, Kansagara D. E-Cigarettes Versus Nicotine Replacement Therapy for Smoking Cessation : Hajek P, Phillips-waller A, Przulj D, et al. A Randomized Trial of E-Cigarettes versus Nicotine-Replacement Therapy. N Engl J Med. 2019;380(7):629-637. J Gen Intern Med. 2021;36: 1481–1483. doi:10.1007/s11606-020-06408-7	No	Not meeting inclusion criteria	Title and abstract screening
2398	Jackson D., Stalgaitis C.A., Wagner D.E., Beckerley S., Fernandez P. 63. Who's Vaping? Understanding the Psychographics of Adolescent E-Cigarette Users in an Era of Growing Use. J Adolesc Health. 2020;66: S33. doi:10.1016/j.jadohealth.2019.11.066	No	Not meeting inclusion criteria	Title and abstract screening
2399	Jackson I, Osaghae I, Etuk A, Jackson N. Prevalence and Factors Associated with Electronic Cigarette Use Among Young Adult Cancer Survivors Using Behavioral Risk Factor Surveillance System, 2016-2018. J Adolesc Young Adult Oncol. 2021;10: 588–598. doi:10.1089/jayao.2020.0104	No	Not meeting inclusion criteria	Title and abstract screening
2400	Jackson I, Oboli G, Udoh A. E-cigarette use and health-related quality of life among adult cancer survivors in the United States. Ann Epidemiol. 2021;61: 13–13. doi:10.1016/j.annepidem.2021.05.024	No	Not meeting inclusion criteria	Title and abstract screening
2401	Jackson I., Etuk A., Jackson N., Osaghae I. Effect of nicotine, low nicotine, and e-cigarette beliefs on cigarette and e-cigarette use in the US population and cancer survivors. J Public Health Ger. 2022;30: 861–869. doi:10.1007/s10389-020-01361-5	No	Not meeting inclusion criteria	Title and abstract screening
2402	Jackson MA, Baker AL, Gould GS, Brown AL, Dunlop AJ, McCarter K. Smoking cessation interventions for pregnant women attending treatment for substance use disorders: A systematic review. Addiction. 2022;117: 847–860. doi:10.1111/add.15663	No	Not meeting inclusion criteria	Title and abstract screening
2403	Jackson S, Brown J, Norris E, Livingstone-Banks J, Hayes E, Lindson N. Mindfulness for smoking cessation. Cochrane Database Syst Rev. 2022;4: CD013696. doi:10.1002/14651858.CD013696.pub2	No	Not meeting inclusion criteria	Title and abstract screening
2404	Jackson S.E., Tattan-Birch H., Shahab L., Beard E., Brown J. Have there been sustained impacts of the Covid-19 pandemic on trends in smoking prevalence, uptake, quitting, use of treatment, and relapse? A monthly population study in England, 2017-2022. medRxiv. 2022. doi:10.1101/2022.12.06.22283023	No	Not meeting inclusion criteria	Title and abstract screening
2405	Jackson SE, Kotz D, West R, Brown J. Reply to "Smoke free, but dependent on nicotine" (Karam-Hage 2019). Addict Abingdon Engl. 2019;114: 1887–1888. doi:10.1111/add.14715	No	Not meeting inclusion criteria	Title and abstract screening
2406	Jackson SE, Kotz D, West R, Brown J. Reply to "Smoke free, but dependent on nicotine" (Karam-Hage 2019)...Karam-hage M. Smoke-free, but dependent on nicotine: comment on jackson et al. Addiction 2019; 114: 1887–1890. Addiction. 2019;114: 1887–1888. doi:10.1111/add.14715	No	Not meeting inclusion criteria	Title and abstract screening
2407	Jackson SE, Beard E, Brown J. Smokers' Use of E-Cigarettes in Situations Where Smoking Is not Permitted in England: Quarterly Trends 2011-2020 and Associations With Sociodemographic and Smoking Characteristics. Nicotine Tob Res. 2021;23: 1831–1838. doi:10.1093/ntr/ntab119	No	Not meeting inclusion criteria	Title and abstract screening
2408	Jackson SE, Cox S, Shahab L, Brown J. Prevalence of use and real-world effectiveness of smoking cessation aids during the COVID-19 pandemic: a representative study of smokers in England. Addiction. 2022;117: 2504–2514. doi:10.1111/add.15903	No	Not meeting inclusion criteria	Title and abstract screening
2409	Jackson SE, Cox S, Shahab L, Brown J. Impact of the cost-of-living crisis on the nature of attempts to stop smoking and to reduce alcohol consumption in Great Britain: A representative population survey, 2021-2022. PLoS One. 2023;18: e0286183. doi:10.1371/journal.pone.0286183	No	Not meeting inclusion criteria	Title and abstract screening
2410	Jackson SE, Garnett C, Shahab L, Oldham M, Brown J. Association of the COVID-19 lockdown with smoking, drinking and attempts to quit in England: an analysis of 2019-20 data. Addiction. 2021;116: 1233–1244. doi:10.1111/add.15295	No	Not meeting inclusion criteria	Title and abstract screening
2411	Jackson SE, Kock L, Kotz D, Brown J. Real-world effectiveness of smoking cessation aids: A population survey in England with 12-month follow-up, 2015-2020. Addict Behav. 2022;135: 107442. doi:10.1016/j.addbeh.2022.107442	No	Not meeting inclusion criteria	Title and abstract screening
2412	Jackson SE, Kotz D, West R, Brown J. Moderators of real-world effectiveness of smoking cessation aids: a population study. Addiction. 2019;114: 1627–1638. doi:10.1111/add.14656	No	Not meeting inclusion criteria	Title and abstract screening
2413	Jackson SE, Tattan-Birch H, Brown J. Trends in where people buy their vaping products and differences by user and device characteristics: A population study in England, 2016-23. Addiction. 2023. doi:10.1111/add.16387	No	Not meeting inclusion criteria	Title and abstract screening
2414	Jackson SE, Tattan-Birch H, Shahab L, Brown J. How has Expenditure on Nicotine Products Changed in a Fast-Evolving Marketplace? A Representative Population Survey in England, 2018-2022. Nicotine Tob Res. 2023;25: 1585–1593. doi:10.1093/ntr/ntad074	No	Not meeting inclusion criteria	Title and abstract screening
2415	Jackson WC, Tang M, Schipper MJ, Sandler HM, Zumsteg ZS, Efstathiou JA, et al. Biochemical Failure Is Not a Surrogate End Point for Overall Survival in Recurrent Prostate Cancer: Analysis of NRG Oncology/RTOG 9601. J Clin Oncol. 2022;40: 3172–3179. doi:10.1200/JCO.21.02741	No	Not meeting inclusion criteria	Title and abstract screening
2416	Jacob J, Boczkowska S, Zaluska W, Buraczynska M. Apolipoprotein A5 gene polymorphism (rs662799) and cardiovascular disease in end-stage kidney disease patients. BMC Nephrol. 2022;23: 1–7. doi:10.1186/s12882-022-02925-1	No	Not meeting inclusion criteria	Title and abstract screening
2417	Jacob JS, Hunt RD. Increasing incidence of pediatric mycosis fungoides from 2000 to 2017: A national population-based study of the Surveillance, Epidemiology, and End Results database and Texas Cancer Registry. J Am Acad Dermatol. 2023;88: 185–187. doi:10.1016/j.jaad.2022.03.046	No	Not meeting inclusion criteria	Title and abstract screening

2418	Jacobson K., Martinez J., Larroque S., Jones I.W., Paschke T. Nicotine pharmacokinetics of electronic cigarettes: A pooled data analysis from the literature. Toxicol Rep. 2021;8: 84–95. doi:10.1016/j.toxrep.2020.12.016	No	Not meeting inclusion criteria	Title and abstract screening
2419	Jafar M.N., Alawadi A.I.M. THE ASSOCIATION BETWEEN ORAL BIOLOGICAL STATUS AND VARIOUS SMOKING HABITS. Biochem Cell Arch. 2021;21: 3041–3044.	No	Not meeting inclusion criteria	Title and abstract screening
2420	Jafarzadeh NS, Bello MS, Wong M, Cho J, Leventhal AM. Associations between anxiety symptoms and barriers to smoking cessation among African Americans who smoke cigarettes daily. Drug Alcohol Depend. 2023;245: 109808. doi:10.1016/j.drugalcdep.2023.109808	No	Not meeting inclusion criteria	Title and abstract screening
2421	Jaffe JH. Making the Case for Nicotine Addiction: Select Reminiscences - A Commentary on Kozlowski et al. (2020). J Stud Alcohol Drugs. 2020;81: 738–739.	No	Not meeting inclusion criteria	Title and abstract screening
2422	Jahnel T, Ferguson SG, Partos T, Brose LS. Socioeconomic differences in the motivation to stop using e-cigarettes and attempts to do so. Addict Behav Rep. 2020;11: 100247. doi:10.1016/j.abrep.2020.100247	No	Not meeting inclusion criteria	Title and abstract screening
2423	Jahromi MK, Tehrani AN, Farhadnejad H, Emamat H, Ahmadirad H, Teymoori F, et al. Dietary advanced glycation end products are associated with an increased risk of breast cancer in Iranian adults. BMC Cancer. 2023;23: 1–10. doi:10.1186/s12885-023-11462-5	No	Not meeting inclusion criteria	Title and abstract screening
2424	Jain A, Davis AM. Initiating Pharmacologic Treatment in Tobacco-Dependent Adults. JAMA. 2021;325: 301–302. doi:10.1001/jama.2020.24790	No	Not meeting inclusion criteria	Title and abstract screening
2425	Jain A, Gandhi Z, Desai R, Mansuri U, Rizvi B, Alvarez M, et al. Nationwide Trends in Hospitalizations and Outcomes of Pulmonary Circulation Disorders Among Patients With Cannabis Use Disorder in the United States. Cureus. 2022;14: e22897. doi:10.7759/cureus.22897	No	Not meeting inclusion criteria	Title and abstract screening
2426	Jain A., Minish J., Maharrey P., Patel D. A rare complication of chemotherapy: Gemcitabine-induced thrombotic microangiopathy. Crit Care Med. 2021;49: 486. doi:10.1097/01.ccm.0000729792.62012.1e	No	Not meeting inclusion criteria	Title and abstract screening
2427	Jain V., Rifai M.A., Brinzevich D., Taj M., Saleh M., Krittanawong C., et al. Association of premature atherosclerotic cardiovascular disease with higher risk of cancer: A behavioral risk factor surveillance system study. Eur J Prev Cardiol. 2022;29: 493–501. doi:10.1093/eurjpc/zwab084	No	Not meeting inclusion criteria	Title and abstract screening
2428	Jakharia K., Henderson A.G. Parsonage turner syndrome: A rare cause of dyspnea. Am J Respir Crit Care Med. 2021;203. doi:10.1164/ajrccm-conference.2021.203.1_MeetingAbstracts.A2391	No	Not meeting inclusion criteria	Title and abstract screening
2429	Jakob J, Joss S, Meier AN, Tal K, Schoeni A, Marti J, et al. The price of nicotine dependence: A comparison of the cost of nicotine across products in Switzerland, Germany, USA, Sweden, France and the UK, in 2019. Tob Prev Cessat. 2022;8: 42. doi:10.18332/tpc/156052	No	Not meeting inclusion criteria	Title and abstract screening
2430	James SA. Electronic nicotine delivery systems and smoking reduction, substitution, and cessation. Diss Abstr Int Sect B Sci Eng. 2017;78: No-Specified.	No	Not meeting inclusion criteria	Title and abstract screening
2431	James SA, Cheney MK, Smith KM, Beebe LA. Experiences of women with cervical dysplasia and associated diagnoses using electronic cigarettes for smoking substitution. Health Expect Int J Public Particip Health Care Health Policy. 2019;22: 931–938. doi:10.1111/hex.12897	No	Not meeting inclusion criteria	Title and abstract screening
2432	Jancey J, Leaver T, Wolf K, Freeman B, Chai K, Bialous S, et al. Promotion of E-Cigarettes on TikTok and Regulatory Considerations. Int J Env Res Public Health. 2023;20. doi:10.3390/ijerph20105761	No	Not meeting inclusion criteria	Title and abstract screening
2433	Jancey J, Maycock B, McCausland K, Howat P. E-Cigarettes: Implications for Health Promotion in the Asian Pacific Region. Asia Pac J Public Health. 2018;30: 321–327. doi:10.1177/1010539518762855	No	Not meeting inclusion criteria	Title and abstract screening
2434	Jankowski M, Kaleta D, Zgliczyński WS, Grudziąż-Sękowska J, Wrześniewska-Wal I, Gujski M, et al. Cigarette and E-Cigarette Use and Smoking Cessation Practices among Physicians in Poland. Int J Env Res Public Health. 2019;16. doi:10.3390/ijerph16193595	No	Not meeting inclusion criteria	Title and abstract screening
2435	Jankowski M, Wrześniewska-Wal I, Ostrowska A, Lusawa A, Wierzba W, Pinkas J. Perception of Harmfulness of Various Tobacco Products and E-Cigarettes in Poland: A Nationwide Cross-Sectional Survey. Int J Env Res Public Health. 2021;18. doi:10.3390/ijerph18168793	No	Not meeting inclusion criteria	Title and abstract screening
2436	Jankowski M., Brozek G., Zejda J., Jarosinska A., Idzik A., Majek P. Electronic cigarette in smoking cessation. Eur Respir J. 2017;50. doi:10.1183/1393003.congress-2017.PA1236	No	Not meeting inclusion criteria	Title and abstract screening
2437	Jankowski P., Kawecka-Jaszcz K., Kopec G., Podolec J., Pajak A., Sarnecka A., et al. Polish forum for prevention guidelines on smoking: Update 2017. Kardiol Pol. 2017;75: 409–411. doi:10.5603/KP.2017.0066	No	Not meeting inclusion criteria	Title and abstract screening
2438	Jansen JH, Roumpf S, Welch JL. Reprint of: Vaping associated pulmonary injury (VAPI): Electronic cigarettes are not harmless. Mon. 2022;68: 101414. doi:10.1016/j.disamonth.2022.101414	No	Not meeting inclusion criteria	Title and abstract screening
2439	Jao NC, Gueorguieva R, Hitsman B, Sofuoglu M. Acute effects of inhaled menthol on cognitive effects of intravenous nicotine among young adult cigarette smokers. Addict Behav. 2021;122: 107022. doi:10.1016/j.addbeh.2021.107022	No	Not meeting inclusion criteria	Title and abstract screening
2440	Jaradeh M., Vigneswaran W.T. Epidemiology of lung cancer and the gender differences in risk. J Mens Health. 2022;18: 073. doi:10.31083/j.jomh1803073	No	Not meeting inclusion criteria	Title and abstract screening
2441	Jarrell ZR, Smith MR, He X, Orr M, Jones DP, Go Y-M. Firsthand and Secondhand Exposure Levels of Maltol-Flavored Electronic Nicotine Delivery System Vapors Disrupt Amino Acid Metabolism. Toxicol Sci Off J Soc Toxicol. 2021;182: 70–81. doi:10.1093/toxsci/kfab051	No	Not meeting inclusion criteria	Title and abstract screening
2442	Jasper AE, Sapey E, Thickett DR, Scott A. Understanding potential mechanisms of harm: the drivers of electronic cigarette-induced changes in alveolar macrophages, neutrophils, and lung epithelial cells. Am J Physiol Lung Cell Mol Physiol. 2021;321: L336–L348. doi:10.1152/ajplung.00081.2021	No	Not meeting inclusion criteria	Title and abstract screening
2443	Jatlow P, Valentine G, Gueorguieva R, Nadim H, Wu R, O'Malley SS, et al. Plasma Menthol Glucuronide as a Biomarker of Acute Menthol Inhalation. Tob Regul Sci. 2018;4: 586–591. doi:10.18001/TRS.4.1.5	No	Not meeting inclusion criteria	Title and abstract screening
2444	Javadi-Paydar M, Kerr TM, Harvey EL, Cole M, Taffe MA. Effects of nicotine and THC vapor inhalation administered by an electronic nicotine delivery system (ENDS) in male rats. Drug Alcohol Depend. 2019;198: 54–62. doi:10.1016/j.drugalcdep.2019.01.027	No	Not meeting inclusion criteria	Title and abstract screening
2445	JAVED S.M., MORIDZADEH S.I.N.A., FUENTES F. VAPING-INDUCED SECONDARY ORGANIZING PNEUMONIA VERSUS E-CIGARETTE OR VAPING PRODUCT USE-ASSOCIATED LUNG INJURY (EVALI): DIFFERENT SPECTRUM OF THE SAME ENTITY? Chest. 2023;164: A3287–A3288. doi:10.1016/j.chest.2023.07.2151	No	Not meeting inclusion criteria	Title and abstract screening
2446	Jawad N, Hakeem FF, Sabbah W. Exploring health advice by dental professionals in USA: A secondary data analysis of NHANES (2015-2018). Patient Educ Couns. 2023;119: 108038. doi:10.1016/j.pec.2023.108038	No	Not meeting inclusion criteria	Title and abstract screening

2447	Jayakumar N, Chaiton M, Goodwin R, Schwartz R, O'Connor S, Kaufman P. Co-use and Mixing Tobacco With Cannabis Among Ontario Adults. Nicotine Tob Res. 2021;23: 171–178. doi:10.1093/ntr/ntz238	No	Not meeting inclusion criteria	Title and abstract screening
2448	Jayes L, Waddingham J, Britton J, Murray R. A qualitative study of the implementation and continued delivery of complete and partial smoke-free policies across England's prison estate. Nicotine Tob Res Off J Soc Res Nicotine Tob. 2023. doi:10.1093/ntr/ntac296	No	Not meeting inclusion criteria	Title and abstract screening
2449	Jean-Jacques J, Cook R, Winterstein AG, Goodin A, Brown JD, Jugl S, et al. Priorities for Medical Marijuana Research from the Perspective of Physicians, Dispensary Owners/Staff, and Patients: A Survey Study. Med Cannabis Cannabinoids. 2021;4: 107–113. doi:10.1159/000518105	No	Not meeting inclusion criteria	Title and abstract screening
2450	Jebai R, Osibogun O, Li W, Gautam P, Bursac Z, Ward KD, et al. Temporal Trends in Tobacco Product Use Among US Middle and High School Students: National Youth Tobacco Survey, 2011-2020. Public Health Rep. 2023;138: 483–492. doi:10.1177/00333549221103812	No	Not meeting inclusion criteria	Title and abstract screening
2451	Jebastin Thangaiah J., Booth C.N., Brainard J.A., Elsheikh T.M., Reynolds J.P., Ondrejka S.L., et al. Oil Red O Staining of Pulmonary Macrophages in Bronchoalveolar Lavage Specimens Is Not Specific for Vaping-Associated Lung Injury. Am J Clin Pathol. 2022. doi:10.1093/ajcp/aqac118	No	Not meeting inclusion criteria	Title and abstract screening
2452	Jefee-Bahloul H., Jaafar M. Smoking and trauma in Syrian refugees. Drug Alcohol Depend. 2017;171: e94–e95. doi:10.1016/j.drugalcdep.2016.08.266	No	Not meeting inclusion criteria	Title and abstract screening
2453	Jeklin AT, Alamgeer M, Stirling RG, Maccora J, Kumarahuru R, Paul D, et al. P2.08-02 Burning the Candle at Both Ends-Sleep Quality Before and After Chemotherapy in Lung Cancer Patients - A Systematic Review and Meta-Analysis. J Thorac Oncol. 2022;17: S136–S137. doi:10.1016/j.jtho.2022.07.230	No	Not meeting inclusion criteria	Title and abstract screening
2454	Jenssen BP, Hannan C, Kelly MK, Ylioja T, Schnoll RA, Fiks AG. Inability to Recruit Adolescents for a Vaping Cessation Clinical Trial Within a Large Pediatric Health System. Nicotine Tob Res. 2021;23: 1633–1634. doi:10.1093/ntr/ntab034	No	Not meeting inclusion criteria	Title and abstract screening
2455	Jenssen BP, Hannan C, Kelly MK, Ylioja T, Schnoll RA, Fiks AG. Response to: More Detail Needed for Pilot E-Cigarette Treatment Trial in Youth and Young Adults. Nicotine Tob Res. 2021;23: 1983–1984. doi:10.1093/ntr/ntab103	No	Not meeting inclusion criteria	Title and abstract screening
2456	Jenssen BP, Walley SC, McGrath-Morrow SA. Heat-not-Burn Tobacco Products: Tobacco Industry Claims No Substitute for Science. Pediatrics. 2018;141. doi:10.1542/peds.2017-2383	No	Not meeting inclusion criteria	Title and abstract screening
2457	Jenssen BP, Walley SC, Boykan R, Little Caldwell A, Camenga D. Protecting Children and Adolescents From Tobacco and Nicotine. Pediatrics. 2023;151. doi:10.1542/peds.2023-061805	No	Not meeting inclusion criteria	Title and abstract screening
2458	Jenssen BP, Wilson KM. Tobacco Control and Treatment for the Pediatric Clinician: Practice, Policy, and Research Updates. Acad Pediatr. 2017;17: 233–242. doi:10.1016/j.acap.2016.12.010	No	Not meeting inclusion criteria	Title and abstract screening
2459	Jeon H, Kim S, Lee I. "The communication I had with him back then is still stuck in my mind." Bereaved families of cancer patients' experiences for end-of-life communication. Support Care Cancer. 2023;31: 1–8. doi:10.1007/s00520-023-07753-z	No	Not meeting inclusion criteria	Title and abstract screening
2460	Jeong M, Singh B, Wackowski OA, Mukherjee R, Steinberg MB, Delnevo CD. Content Analysis of E-cigarette News Articles Amidst the 2019 Vaping-Associated Lung Injury (EVALI) Outbreak in the United States. Nicotine Tob Res. 2022;24: 799–803. doi:10.1093/ntr/ntab203	No	Not meeting inclusion criteria	Title and abstract screening
2461	Jeong S.H., Jang B.N., Kim S.H., Jang S.-I., Park E.-C. Investigation of the association between smoking behavior and metabolic syndrome using lipid accumulation product index among south korean adults. Int J Environ Res Public Health. 2021;18: 4151. doi:10.3390/ijerph18084151	No	Not meeting inclusion criteria	Title and abstract screening
2462	Jeongmi Shin, Yejin Kim, Shin Hye Yoo, Jin-Ah Sim, Bhumsuk Keam. Impact of COVID-19 on the End-of-Life Care of Cancer Patients Who Died in a Korean Tertiary Hospital: A Retrospective Study. Korean J Hosp Palliat Care. 2022;25: 150–158. doi:10.14475/jhpc.2022.25.4.150	No	Not meeting inclusion criteria	Title and abstract screening
2463	Jesch E, Kikut AI, Hornik R. Comparing belief in short-term versus long-term consequences of smoking and vaping as predictors of non-use in a 3-year nationally representative survey study of US youth. Tob Control. 2021. doi:10.1136/tobaccocontrol-2021-056886	No	Not meeting inclusion criteria	Title and abstract screening
2464	Jesch E, Kikut AI, Hornik R. Comparing belief in short-term versus long-term consequences of smoking and vaping as predictors of non-use in a 3-year nationally representative survey study of US youth. Tob Control Int J. 2023;32: 435–442. doi:10.1136/tobaccocontrol-2021-056886	No	Not meeting inclusion criteria	Title and abstract screening
2465	Jewitt N, Rapoport A, Gupta A, Srikanthan A, Sutradhar R, Luo J, et al. The Effect of Specialized Palliative Care on End-of-Life Care Intensity in AYAs with Cancer. J Pain Symptom Manage. 2023;65: 222–232. doi:10.1016/j.jpainsymman.2022.11.013	No	Not meeting inclusion criteria	Title and abstract screening
2466	Jha P. The hazards of smoking and the benefits of cessation: A critical summation of the epidemiological evidence in high-income countries. eLife. 2020;9: e49979. doi:10.7554/eLife.49979	No	Not meeting inclusion criteria	Title and abstract screening
2467	Jiang A, Liu N, Zhao R, Liu S, Gao H, Wang J, et al. Construction and Validation of a Novel Nomogram to Predict the Overall Survival of Patients With Combined Small Cell Lung Cancer: A Surveillance, Epidemiology, and End Results Population-Based Study. Cancer Control J Moffitt Cancer Cent. 2021; 1–12. doi:10.1177/10732748211051228	No	Not meeting inclusion criteria	Title and abstract screening
2468	Jiang N, Ho SY, Wang MP, Leung LT, Lam TH. The relationship of waterpipe use with cigarette smoking susceptibility and nicotine dependence: A cross-sectional study among Hong Kong adolescents. Addict Behav. 2017;64: 123–128. doi:10.1016/j.addbeh.2016.08.035	No	Not meeting inclusion criteria	Title and abstract screening
2469	Jiang N, Cleland CM, Wang MP, Kwong A, Lai V, Lam TH. Perceptions and use of e-cigarettes among young adults in Hong Kong. BMC Public Health. 2019;19: 1123. doi:10.1186/s12889-019-7464-z	No	Not meeting inclusion criteria	Title and abstract screening
2470	Jiang N, Xu S, Li L, Cleland CM, Niaura RS. Use of electronic nicotine delivery system (ENDS) devices among U.S. Youth and adults: Findings from the Population Assessment of Tobacco and Health Study Waves 1-5. Addict Behav. 2023;139: 107588. doi:10.1016/j.addbeh.2022.107588	No	Not meeting inclusion criteria	Title and abstract screening
2471	Jilani T., Arevalo G., Haque S., Brumfield E., Spielmann S., Reyes B., et al. Cardiopulmonary implications of hypermobile ehlersdanlos syndrome - a case report. Am J Med Sci. 2023;365: S32. doi:10.1016/S0002-9629%2823%2900066-6	No	Not meeting inclusion criteria	Title and abstract screening
2472	Jilani U.A., Othman Z., Yusuf E., Jilani S.A. PHARMACOLOGICAL AND RECREATIONAL SUBSTANCE ABUSE - AN EMERGING RISK TO CARDIOVASCULAR HEALTH AMONG THE YOUNGER POPULATION. Int J Med Toxicol Leg Med. 2021;24: 81–89. doi:10.5958/0974-4614.2021.00012.7	No	Not meeting inclusion criteria	Title and abstract screening
2473	Jimeno Sánchez J, Chabbar Boudet MC, Morlanes Gracia P, Laita Monreal S, López Perales CR, Cuko G, et al. [Anti-smoking intervention in the acute phase of acute coronary syndrome: Is there additional benefit in patients included in a cardiac rehabilitation program?]. J Heal Qual Res. 2023;38: 105–111. doi:10.1016/j.jhqr.2022.06.005	No	Not meeting inclusion criteria	Title and abstract screening
2474	Jin J. Interventions to Promote Tobacco Cessation. JAMA. 2021;325: 316. doi:10.1001/jama.2020.25605	No	Not meeting inclusion criteria	Title and abstract screening
2475	Jin L., Conklin D.J. A novel evaluation of endothelial dysfunction ex vivo: "Teaching an Old Drug a New Trick." Physiol Rep. 2021;9: e15120. doi:10.14814/phy2.15120	No	Not meeting inclusion criteria	Title and abstract screening

2476	Jin L., Conklin D.J. Ex vivo evaluation of vascular toxicity of tobacco products. Circulation. 2021;144. doi:10.1161/circ.144.suppl-1.13900	No	Not meeting inclusion criteria	Title and abstract screening
2477	Jin L, Lynch J, Richardson A, Lorkiewicz P, Srivastava S, Theis W, et al. Electronic cigarette solvents, pulmonary irritation, and endothelial dysfunction: role of acetaldehyde and formaldehyde. Am J Physiol Heart Circ Physiol. 2021;320: H1510–H1525. doi:10.1152/ajpheart.00878.2020	No	Not meeting inclusion criteria	Title and abstract screening
2478	Jin Y, Xu S, Luo X, Wang Y, Li J, Liang B, et al. A network approach to the symptom-level associations between smoking and posttraumatic stress disorder (PTSD) among young adults exposed to childhood sexual abuse. J Glob Health. 2023;13: 04037. doi:10.7189/jogh.13.04037	No	Not meeting inclusion criteria	Title and abstract screening
2479	Jo M, Lee Y, Kim T. Medical care costs at the end of life among older adults with cancer: a national health insurance data-based cohort study. BMC Palliat Care. 2023;22: 1–9. doi:10.1186/s12904-023-01197-2	No	Not meeting inclusion criteria	Title and abstract screening
2480	Jo S, Kim HC, Lustig N, Chen G, Lee JH. Mixed-effects multilevel analysis followed by canonical correlation analysis is an effective fMRI tool for the investigation of idiosyncrasies. Hum Brain Mapp. 2021;42: 5374–5396. doi:10.1002/hbm.25627	No	Not meeting inclusion criteria	Title and abstract screening
2481	Job MK, Schumacher P, Müller G, Kreyer C. The social and spiritual dimensions of parental experiences on end-of-life care of their child with cancer: A narrative review. Palliat Support Care. 2023;21: 308–319. doi:10.1017/S1478951521001991	No	Not meeting inclusion criteria	Title and abstract screening
2482	Johannesen EJD, Timm H, Róin Á. Caregivers' experiences of end-of-life caregiving to severely ill relatives with cancer dying at home: A qualitative study in the Faroe Islands. Scand J Caring Sci. 2023;37: 788–796. doi:10.1111/scs.13165	No	Not meeting inclusion criteria	Title and abstract screening
2483	Johne S, van der Toorn M, Iskandar AR, Majeed S, Torres LO, Hoeng J, et al. An in vitro evaluation of e-vapor products: The contributions of chemical adulteration, concentration, and device power. Food Chem Toxicol Int J Publ Br Ind Biol Res Assoc. 2023;175: 113708. doi:10.1016/j.fct.2023.113708	No	Not meeting inclusion criteria	Title and abstract screening
2484	Johnson AC, Mercincavage M, Souprountchouk V, Sidhu AK, Villanti AC, Delnevo CD, et al. Construct validity of the Cigarette Ratings Scale and associations with tobacco use and product feature outcomes. Drug Alcohol Depend. 2022;234: 109397. doi:10.1016/j.drugalcdep.2022.109397	No	Not meeting inclusion criteria	Title and abstract screening
2485	Johnson AC, Mercincavage M, Souprountchouk V, Deatley T, Mays D, Strasser AA. Assessing Attention to Tobacco Warnings With a Heatmapping Task. Am J Prev Med. 2023;65: 809–817. doi:10.1016/j.amepre.2023.05.020	No	Not meeting inclusion criteria	Title and abstract screening
2486	Johnson AC, Mercincavage M, Tan ASL, Villanti AC, Delnevo CD, Strasser AA. Effects of reduced nicotine content cigarette advertising with warning labels and social media features on product perceptions among young adults. J Behav Med. 2023;46: 948–959. doi:10.1007/s10865-023-00441-7	No	Not meeting inclusion criteria	Title and abstract screening
2487	Johnson AL, Collins LK, Villanti AC, Pearson JL, Niaura RS. Patterns of Nicotine and Tobacco Product Use in Youth and Young Adults in the United States, 2011-2015. Nicotine Tob Res Off J Soc Res Nicotine Tob. 2018;20: S48–S54. doi:10.1093/ntr/nty018	No	Not meeting inclusion criteria	Title and abstract screening
2488	Johnson DL, Okamoto SK, Rosario MH, Pokhrel P. Tobacco product use and cultural connectedness among Native Hawaiian/Pacific Islander, Asian American, and Filipino American young adults in Hawai'i. J Ethn Subst Abuse. 2022; 1–15. doi:10.1080/15332640.2022.2161082	No	Not meeting inclusion criteria	Title and abstract screening
2489	Johnson-Arbor K. Goodbye JUUL, hello Puff Bars: Trends and toxicity of currently available electronic cigarettes. Am J Emerg Med. 2022;56: 368–369. doi:10.1016/j.ajem.2021.10.014	No	Not meeting inclusion criteria	Title and abstract screening
2490	Johnston E, Bains M, Hunter A, Langley T. The Impact of the COVID-19 Pandemic on Smoking, Vaping, and Smoking Cessation Services in the United Kingdom: A Qualitative Study. Nicotine Tob Res. 2023;25: 339–344. doi:10.1093/ntr/ntac227	No	Not meeting inclusion criteria	Title and abstract screening
2491	Johnston EE, Martinez I, Wolfe J, Asch SM. Quality measures for end-of-life care for children with cancer: A modified Delphi approach. Cancer 0008543X. 2021;127: 2571–2578. doi:10.1002/cncr.33546	No	Not meeting inclusion criteria	Title and abstract screening
2492	Jonas A. Impact of vaping on respiratory health. BMJ. 2022;378: e065997. doi:10.1136/bmj-2021-065997	No	Not meeting inclusion criteria	Title and abstract screening
2493	Jonas A. Lipid-Laden alveolar macrophages and vaping: Lessons from EVALI. EBioMedicine. 2020;60: 103010. doi:10.1016/j.ebiom.2020.103010	No	Not meeting inclusion criteria	Title and abstract screening
2494	Jones CA, Wallace MJ, Bandaru P, Woodbury ED, Mohler PJ, Wold LE. E-cigarettes and arrhythmogenesis: a comprehensive review of pre-clinical studies and their clinical implications. Cardiovasc Res. 2023;119: 2157–2164. doi:10.1093/cvr/cvad113	No	Not meeting inclusion criteria	Title and abstract screening
2495	Jones DM, Guy MC, Soule E, Sakuma KK, Pokhrel P, Orloff M, et al. Characterization of Electronic Cigarette Warning Statements Portrayed in YouTube Videos. Nicotine Tob Res. 2021;23: 1358–1366. doi:10.1093/ntr/ntaa272	No	Not meeting inclusion criteria	Title and abstract screening
2496	Jones G, McIntosh E, Brose L, Klonizakis M. Participant Experiences of a Quit Smoking Attempt Through Either Nicotine Replacement Therapy (NRT) Methods or the Use of an E-cigarette. 2022;16: 272-277. doi:10.1097/ADM.0000000000000881	No	Not meeting inclusion criteria	Title and abstract screening
2497	Jones J, Slayford S, Gray A, Brick K, Prasad K, Proctor C. A cross-category puffing topography, mouth level exposure and consumption study among Italian users of tobacco and nicotine products. Sci Rep. 2020;10: 12. doi:10.1038/s41598-019-55410-5	No	Not meeting inclusion criteria	Title and abstract screening
2498	Jongebloed H., Cole E., Dean E., Ugalde A. GENERAL PRACTICE NURSES SUPPORTING PATIENTS to QUIT SMOKING: A QUALITATIVE STUDY on CURRENT PRACTICE and OPPORTUNITIES for EDUCATION. Support Care Cancer. 2023;31: S47. doi:10.1007/s00520-023-07786-4	No	Not meeting inclusion criteria	Title and abstract screening
2499	Jongenelis M.I., Kameron C., Rudaizky D., Slevin T., Pettigrew S. Perceptions of the harm, addictiveness, and smoking cessation effectiveness of e-cigarettes among Australian young adults. Addict Behav. 2019;90: 217–221. doi:10.1016/j.addbeh.2018.11.004	No	Not meeting inclusion criteria	Title and abstract screening
2500	Jongenelis MI, Jongenelis G, Alexander E, Kennington K, Phillips F, Pettigrew S. A content analysis of the tweets of e-cigarette proponents in Australia. Health Promot J Austr. 2022;33: 445–450. doi:10.1002/hpja.510	No	Not meeting inclusion criteria	Title and abstract screening
2501	Jordt SE, Jabba SV, Silinski P, Berman ML. An electronic cigarette pod system delivering 6-methyl nicotine, a synthetic nicotine analog, marketed in the United States as "PMTA exempt". MedRxiv Prepr Serv Health Sci. 2023. doi:10.1101/2023.11.21.23298778	No	Not meeting inclusion criteria	Title and abstract screening
2502	Jorenby DE, Smith SS, Fiore MC, Baker TB. Nicotine levels, withdrawal symptoms, and smoking reduction success in real world use: A comparison of cigarette smokers and dual users of both cigarettes and E-cigarettes. Drug Alcohol Depend. 2017;170: 93–101. doi:10.1016/j.drugalcdep.2016.10.041	No	Not meeting inclusion criteria	Title and abstract screening
2503	Jose T. 67.4 IDENTIFYING AND MONITORING PATIENTS WHO USE ELECTRONIC VAPING DEVICES: TOOLS FOR CLINICAL PRACTICE. J Am Acad Child Adolesc Psychiatry. 2020;59: S102. doi:10.1016/j.jaac.2020.07.417	No	Not meeting inclusion criteria	Title and abstract screening
2504	Joseph S, Krebs NM, Zhu J, Wert Y, Goel R, Reilly SM, et al. Differences in nicotine dependence, smoke exposure and consumer characteristics between smokers of machine-injected roll-your-own cigarettes and factory-made cigarettes. Drug Alcohol Depend. 2018;187: 109–115. doi:10.1016/j.drugalcdep.2018.01.039	No	Not meeting inclusion criteria	Title and abstract screening

2505	Joshi J, Vora H, Ghosh N, Tankshali R, Jetly D, Trivedi T. Nonhomologous end joining repair pathway molecules as predictive biomarkers for patients with oral squamous cell carcinoma. J Cancer Res Ther. 2021;17: 1031–1038. doi:10.4103/jcrt.JCRT_582_19	No	Not meeting inclusion criteria	Title and abstract screening
2506	Joss S, Moser A, Jakob J, Tal K, Etter JF, Selby K, et al. Counseling in Vape Shops: A Survey of Vape Shop Managers in Switzerland. Int J Env Res Public Health. 2021;18. doi:10.3390/ijerph182010861	No	Not meeting inclusion criteria	Title and abstract screening
2507	Jovanova M., Skurka C., Byrne S., Kalaji M., Greiner Safi A., Porticella N., et al. Should Graphic Warning Labels Proposed for Cigarette Packages Sold in the United States Mention the Food and Drug Administration? Nicotine Tob Res. 2021;23: 402–406. doi:10.1093/ntr/ntaa142	No	Not meeting inclusion criteria	Title and abstract screening
2508	Jude J, Hiller H, Miller J. Melon with a twist: A case of nicotine overdose after ingestion and aspiration of vape liquid. Mil Med. 2021;186: 246–249. doi:10.1093/milmed/usaa331https://dx.doi.org/10.1093/milmed/usaa331	No	Not meeting inclusion criteria	Title and abstract screening
2509	Julião M, Johnston B, Antunes B. Dignity Therapy – Past, present and future journey: Beyond end of life cancer care. Responding to Grassi et al. Psychooncology. 2022;31: 1431–1432. doi:10.1002/pon.5981	No	Not meeting inclusion criteria	Title and abstract screening
2510	Jun J, Fitzpatrick MA, Zain A, Zhang N. Have E-cigarette Risk Perception and Cessation Intent of Young Adult Users Changed During the Pandemic? Am J Health Behav. 2022;46: 304–314. doi:10.5993/AJHB.46.3.9	No	Not meeting inclusion criteria	Title and abstract screening
2511	Jun J, Nan X. Comparative risk assessment and cessation information seeking among smokeless tobacco users. Addict Behav. 2018;80: 14–21. doi:10.1016/j.addbeh.2017.12.031	No	Not meeting inclusion criteria	Title and abstract screening
2512	Jung MY, Matthews AK. Understanding Nurses' Experiences and Perceptions of End-of-Life Care for Cancer Patients in Korea: A Scoping Review. J Palliat Care. 2021;36: 255–264. doi:10.1177/08258597211027021	No	Not meeting inclusion criteria	Title and abstract screening
2513	Just IA, Schoenrath F, Passinger P, Stein J, Kemper D, Knosalla C, et al. Validity of the 6-Minute Walk Test in Patients with End-Stage Lung Diseases Wearing an Oronasal Surgical Mask in Times of the COVID-19 Pandemic. Respiration. 2021;100: 594–599. doi:10.1159/000515606	No	Not meeting inclusion criteria	Title and abstract screening
2514	Kaanders JHAM. Head and neck cancer: high-end technology is no guarantee of high-quality care. Lancet. 2022;399: 2101–2102. doi:10.1016/S0140-6736(22)00468-8	No	Not meeting inclusion criteria	Title and abstract screening
2515	Kadakia KC, Trufan SJ, Jagosky MH, Worrilow WM, Harrison BW, Broyhill KL, et al. Early-onset pancreatic cancer: an institutional series evaluating end-of-life care. Support Care Cancer. 2021;29: 3613–3622. doi:10.1007/s00520-020-05876-1	No	Not meeting inclusion criteria	Title and abstract screening
2516	Kaelin R. Information, content marketing, official communication around E-cigarette "juul"'s introduction into the swiss market. Respiration. 2019;97: 628. doi:10.1159/000499887	No	Not meeting inclusion criteria	Title and abstract screening
2517	Kahe M, Zhang Y. Understanding Gender Differences in Reasons for Using e-Cigarettes Is Important for Intervention. Subst Use Misuse. 2022;57: 2008. doi:10.1080/10826084.2022.2124877	No	Not meeting inclusion criteria	Title and abstract screening
2518	Kahl C. AN UNUSUAL CASE OF STREPTOCOCCAL PHARYNGITIS. J Hosp Med. 2023;18: S438. doi:10.1002/jhm.13090	No	Not meeting inclusion criteria	Title and abstract screening
2519	Kalan ME, Ghobadi H, Taleb ZB, Adham D, Cobb CO, Ward KD, et al. COVID-19 and beliefs about tobacco use: an online cross-sectional study in Iran. Env Sci Pollut Res Int. 2021;28: 40346–40354. doi:10.1007/s11356-020-11038-x	No	Not meeting inclusion criteria	Title and abstract screening
2520	Kalchiem-Dekel O., Fuentes P., Bott M.J., Beattie J.A., Lee R.P., Chawla M., et al. Multiplanar 3D fluoroscopy redefines tool-lesion relationship during robotic-assisted bronchoscopy. Respirology. 2021;26: 120–123. doi:10.1111/resp.13966	No	Not meeting inclusion criteria	Title and abstract screening
2521	Kale D, Brown J, Dawkins L, Goniewicz ML, Leppin C, Tattan-Birch H, et al. Comparing identity, attitudes, and indicators of effectiveness in people who smoke, vape or use heated tobacco products: A cross-sectional study. Addict Behav. 2023;151: 107933. doi:10.1016/j.addbeh.2023.107933	No	Not meeting inclusion criteria	Title and abstract screening
2522	Kale D, Perski O, Herbec A, Beard E, Shahab L. Changes in Cigarette Smoking and Vaping in Response to the COVID-19 Pandemic in the UK: Findings from Baseline and 12-Month Follow up of HEBECO Study. Int J Env Res Public Health. 2022;19. doi:10.3390/ijerph19020630	No	Not meeting inclusion criteria	Title and abstract screening
2523	Kale D, Tattan-Birch H, Brown J, Cox S, Dawkins L, Goniewicz ML, et al. Examining acute psychopharmacological effects of nicotine vaping versus heated tobacco products in a randomised crossover study of product naïve adult smokers. Sci Rep. 2023;13: 22676. doi:10.1038/s41598-023-49602-3	No	Not meeting inclusion criteria	Title and abstract screening
2524	Kaliamurthy S. 2.6 Clinical Interventions for Adolescent Vaping of Nicotine and Cannabis. J Am Acad Child Adolesc Psychiatry. 2022;61: S127. doi:10.1016/j.jaac.2022.07.503	No	Not meeting inclusion criteria	Title and abstract screening
2525	Kaliamurthy S., Camenga D.R. Clinical approach to the treatment of e-cigarette use among adolescents. Curr Probl Pediatr Adolesc Health Care. 2022;52: 101203. doi:10.1016/j.cppeds.2022.101203	No	Not meeting inclusion criteria	Title and abstract screening
2526	Kalke K., Studd H., Scherr C.L. The communication of uncertainty in health: A scoping review. Patient Educ Couns. 2021;104: 1945–1961. doi:10.1016/j.pec.2021.01.034	No	Not meeting inclusion criteria	Title and abstract screening
2527	Kalkhoran S, Kruse GR, Rigotti NA, Rabin J, Ostroff JS, Park ER. Electronic cigarette use patterns and reasons for use among smokers recently diagnosed with cancer. Cancer Med. 2018;7: 3484–3491. doi:10.1002/cam4.1585	No	Not meeting inclusion criteria	Title and abstract screening
2528	Kalkhoran S, Streck JM, Kruse GR, Rigotti NA, Perez GK, Regan S, et al. Longitudinal Electronic Cigarette Use Among Patients Recently Diagnosed With Cancer Enrolled in a Smoking Cessation Trial. Nicotine Tob Res. 2022;24: 970–977. doi:10.1093/ntr/ntac031	No	Not meeting inclusion criteria	Title and abstract screening
2529	Kalkhoran S., Benowitz N.L., Rigotti N.A. Reprint of: Prevention and Treatment of Tobacco Use: JACC Health Promotion Series. J Am Coll Cardiol. 2018;72: 2964–2979. doi:10.1016/j.jacc.2018.10.020	No	Not meeting inclusion criteria	Title and abstract screening
2530	Kalkhoran SM, Levy DE, Rigotti NA. Smoking and E-Cigarette Use Among U.S. Adults During the COVID-19 Pandemic. Am J Prev Med. 2022;62: 341–349. doi:10.1016/j.amepre.2021.08.018	No	Not meeting inclusion criteria	Title and abstract screening
2531	Kallupi M, George O. Nicotine Vapor Method to Induce Nicotine Dependence in Rodents. Curr Protoc Neurosci. 2017;80: 8.41.1-8.41.10. doi:10.1002/cpns.34	No	Not meeting inclusion criteria	Title and abstract screening
2532	Kallupi M., George O. Nicotine vapor method to induce nicotine dependence in rodents. Curr Protoc Neurosci. 2017;2017: 8.41. doi:10.1002/cpns.34	No	Not meeting inclusion criteria	Title and abstract screening
2533	Kamat AM, Apolo AB, Babjuk M, Bivalacqua TJ, Black PC, Buckley R, et al. Definitions, End Points, and Clinical Trial Designs for Bladder Cancer: Recommendations From the Society for Immunotherapy of Cancer and the International Bladder Cancer Group. J Clin Oncol. 2023;41: 5437–5447. doi:10.1200/JCO.23.00307	No	Not meeting inclusion criteria	Title and abstract screening

2534	Kamerow D. Start-up e-cigarette brand aims to "improve smokers' lives." BMJ Online. 2018;362: k2930. doi:10.1136/bmj.k2930	No	Not meeting inclusion criteria	Title and abstract screening
2535	Kan M., Diwadkar A., Shuai H., Himes B.E. SARS-CoV-2 Viral Entry-Related Gene Expression Changes in Response to Cigarette and Glucocorticoid Exposures. Am J Respir Crit Care Med. 2021;203. doi:10.1164/ajrccm-conference.2021.TP105	No	Not meeting inclusion criteria	Title and abstract screening
2536	Kanddy Chin Yee Loo, Yueh Ni Lim, Doreen Kher Lee Kiu, Wee Wee Sim. Gestational trophoblastic neoplasm in a patient with end-stage renal failure (ESRF): the challenges and lessons learnt. BMJ Case Rep. 2022;15: 1–4. doi:10.1136/bcr-2022-249474	No	Not meeting inclusion criteria	Title and abstract screening
2537	Kang BH, Lee DH, Roh MS, Um SJ, Kim I. Acute Eosinophilic Pneumonia after Combined Use of Conventional and Heat-Not-Burn Cigarettes: A Case Report. Med Kaunas. 2022;58. doi:10.3390/medicina58111527	No	Not meeting inclusion criteria	Title and abstract screening
2538	Kang HS, Jung JW, Park HJ, Park DI, Park JS, Park JH, et al. A pilot investigation of e-cigarette use and smoking behaviour among patients with chronic airway disease or respiratory symptoms. Clin Respir J. 2022;16: 17–26. doi:10.1111/crj.13445	No	Not meeting inclusion criteria	Title and abstract screening
2539	Kang N, Kim RK, Kim HJ. Effects of transcranial direct current stimulation on symptoms of nicotine dependence: A systematic review and meta-analysis. Addict Behav. 2019;96: 133–139. doi:10.1016/j.addbeh.2019.05.006	No	Not meeting inclusion criteria	Title and abstract screening
2540	Kangovi S, Mitra N, Turr L, Huo H, Grande D, Long JA. A randomized controlled trial of a community health worker intervention in a population of patients with multiple chronic diseases: Study design and protocol. Contemp Clin Trials. 2017;53: 115–121. doi:10.1016/j.cct.2016.12.009	No	Not meeting inclusion criteria	Title and abstract screening
2541	Kanitra J.J., Thampy C.A., Cullen M.L. A decade's experience of pediatric lung abscess and empyema at a community hospital. Pediatr Pulmonol. 2021;56: 1245–1251. doi:10.1002/ppul.25254	No	Not meeting inclusion criteria	Title and abstract screening
2542	Kano T, Io H, Nakata J, Sasaki Y, Muto M, Shimizu Y, et al. Impact of Transferrin Saturation and Anemia on Radial Artery Calcification in Patients with End-Stage Kidney Disease. Nutrients. 2022;14: 4269-N.PAG. doi:10.3390/nu14204269	No	Not meeting inclusion criteria	Title and abstract screening
2543	Kantrow SP, Jolley SE, Price-Haywood EG, Wang X, Tseng TS, Arnold D, et al. Using the emergency department to investigate smoking in young adults. Ann Epidemiol. 2019;30: 44-49.e1. doi:10.1016/j.annepidem.2018.11.007	No	Not meeting inclusion criteria	Title and abstract screening
2544	Kaplan AG. Cannabis and Lung Health: Does the Bad Outweigh the Good? Pulm Ther. 2021;7: 395–408. doi:10.1007/s41030-021-00171-8	No	Not meeting inclusion criteria	Title and abstract screening
2545	Kaplan B, Marcell AV, Kaplan T, Cohen JE. Association between e-cigarette use and parents' report of attention deficit hyperactivity disorder among US youth. Tob Induc Dis. 2021;19: 44. doi:10.18332/tid/136031	No	Not meeting inclusion criteria	Title and abstract screening
2546	Kaplan B, Koffarnus M, Franck C, Bickel W. Effects of Reduced-Nicotine Cigarettes Across Regulatory Environments in the Experimental Tobacco Marketplace: a Randomized Trial. 2021;23: 1123-1132. doi:10.1093/ntr/ntaa226	No	Not meeting inclusion criteria	Title and abstract screening
2547	Kar A., Thakur S., Rao V.U.S. Electronic cigarette use amongst youth: A threat to public health? Oral Oncol. 2020;104: 104593. doi:10.1016/j.oraloncology.2020.104593	No	Not meeting inclusion criteria	Title and abstract screening
2548	Karam-Hage M. E-cigarettes and the Nicotine Epidemic*. Am J Addict. 2018;27: 650–651. doi:10.1111/ajad.12832	No	Not meeting inclusion criteria	Title and abstract screening
2549	Karam-Hage M. Smoke-free, but dependent on nicotine: comment on Jackson et al. (2019). Addict Abingdon Engl. 2019;114: 1887. doi:10.1111/add.14700	No	Not meeting inclusion criteria	Title and abstract screening
2550	Karam-Hage M, Gonzalez R, Damaj MI. Pharmacotherapy for smoking cessation: overview of medications including over-the-counter nicotine replacement therapy, bupropion, and varenicline. Subst Use Disord. 2021; 221–242.	No	Not meeting inclusion criteria	Title and abstract screening
2551	Karam-Hage M. Smoke-free, but dependent on nicotine: comment on Jackson et al. (2019)...Jackson S, Kotz D, West R et al. Moderators of realworld effectiveness of smoking cessation aids: a population study. Addiction 2019; 114: 1627–38. Addiction. 2019;114: 1887–1887. doi:10.1111/add.14700	No	Not meeting inclusion criteria	Title and abstract screening
2552	Karey E. Ongoing Difficulty of Characterizing Nicotine Product Risks. JAMA Netw Open. 2021;4: e2119888. doi:10.1001/jamanetworkopen.2021.19888	No	Not meeting inclusion criteria	Title and abstract screening
2553	Karim MA, Talluri R, Chido-Amajuoyi OG, Shete S. Awareness of heated tobacco products among US Adults - Health information national trends survey, 2020. Subst Abus. 2022;43: 1023–1034. doi:10.1080/08897077.2022.2060440	No	Not meeting inclusion criteria	Title and abstract screening
2554	Karkin K, İzol V, Kaplan M, Değer M, Akdoğan N, Tansuğ MZ. Demonstration of advanced glycation end product (AGE) expression in bladder cancer tissue in type-2 diabetic and non-diabetic patients and the relationship between AGE accumulation and endoplasmic reticulum stress with bladder cancer. Int J Clin Pract. 2021;75: 1–9. doi:10.1111/ijcp.14526	No	Not meeting inclusion criteria	Title and abstract screening
2555	Kashanchi K.I., Nazemi A.K., Komatsu D.E., Wang E.D. Smoking as a risk factor for complications following arthroscopic rotator cuff repair. JSES Int. 2021;5: 83–87. doi:10.1016/j.jseint.2020.10.002	No	Not meeting inclusion criteria	Title and abstract screening
2556	Kaslow JA, Rosas-Salazar C, Moore PE. E-cigarette and vaping product use-associated lung injury in the pediatric population: A critical review of the current literature. Pediatr Pulmonol. 2021;56: 1857–1867. doi:10.1002/ppul.25384	No	Not meeting inclusion criteria	Title and abstract screening
2557	Kassam A, Gupta A, Rapoport A, Srikanthan A, Sutradhar R, Luo J, et al. Impact of Palliative Care Involvement on End-of-Life Care Patterns Among Adolescents and Young Adults With Cancer: A Population-Based Cohort Study. J Clin Oncol. 2021;39: 2506–2515. doi:10.1200/JCO.20.03698	No	Not meeting inclusion criteria	Title and abstract screening
2558	Kassam A, Gupta A, Rapoport A, Srikanthan A, Sutradhar R, Luo J, et al. Impact of Specialized Versus General Palliative Care on the Intensity of Medical Care at the End of Life in Adolescents and Young Adults with Cancer: A Population-Based Cohort Study. J Pain Symptom Manage. 2022;63: 883–883. doi:10.1016/j.jpainsymman.2022.02.084	No	Not meeting inclusion criteria	Title and abstract screening
2559	Kassner M, Eaton JB, Tang N, Petit JL, Meurice N, Yin HH, et al. High-throughput cell-based assays for identifying antagonists of multiple smoking-associated human nicotinic acetylcholine receptor subtypes. SLAS Discov. 2022;27: 68–76. doi:10.1016/j.slasd.2021.10.001	No	Not meeting inclusion criteria	Title and abstract screening
2560	Kasson E, Singh AK, Huang M, Wu D, Cavazos-Rehg P. Using a mixed methods approach to identify public perception of vaping risks and overall health outcomes on Twitter during the 2019 EVALI outbreak. Int J Med Inf. 2021;155: 104574. doi:10.1016/j.ijmedinf.2021.104574	No	Not meeting inclusion criteria	Title and abstract screening
2561	Kastaun S., Becker S., Kotz D. How effective is the electronic (e-)cigarette for smoking cessation? Gynakologische Prax. 2018;43: 703–713.	No	Not meeting inclusion criteria	Title and abstract screening
2562	Kastaun S., Becker S., Kotz D. How effective is the electronic (e-)cigarette for smoking cessation? Internist Prax. 2018;59: 503–513.	No	Not meeting inclusion criteria	Title and abstract screening

2563	Kastaun S., Becker S., Kotz D. How effective is the electronic (e-)cigarette for smoking cessation? Padiatrische Prax. 2019;91: 347–357.	No	Not meeting inclusion criteria	Title and abstract screening
2564	Kastaun S., Brown J., Kotz D. Association between income and education with quit attempts, use of cessation aids, and short-term success in tobacco smokers: A social gradient analysis from a population-based cross-sectional household survey in Germany (DEBRA study). Addict Behav. 2020;111: 106553. doi:10.1016/j.addbeh.2020.106553	No	Not meeting inclusion criteria	Title and abstract screening
2565	Kastaun S, Kotz D. Brief physician advice for smoking cessation: Results of the DEBRA study. Sucht Z Wiss Prax. 2019;65: 34–41.	No	Not meeting inclusion criteria	Title and abstract screening
2566	Kastner J., Hossain R., White C.S. Epidemiology of Lung Cancer. Semin Roentgenol. 2020;55: 23–40. doi:10.1053/j.ro.2019.10.003	No	Not meeting inclusion criteria	Title and abstract screening
2567	Kasza KA, Coleman B, Sharma E, Conway KP, Cummings KM, Goniewicz ML, et al. Correlates of Transitions in Tobacco Product Use by U.S. Adult Tobacco Users between 2013⁻2014 and 2014⁻2015: Findings from the PATH Study Wave 1 and Wave 2. Int J Env Res Public Health. 2018;15. doi:10.3390/ijerph15112556	No	Not meeting inclusion criteria	Title and abstract screening
2568	Kasza KA, Edwards KC, Anesetti-Rothermel A, Creamer MR, Cummings KM, Niaura RS, et al. E-cigarette use and change in plans to quit cigarette smoking among adult smokers in the United States: Longitudinal findings from the PATH Study 2014-2019. Addict Behav. 2022;124: 107124. doi:10.1016/j.addbeh.2021.107124	No	Not meeting inclusion criteria	Title and abstract screening
2569	Kasza KA, Edwards KC, Kimmel HL, Anesetti-Rothermel A, Cummings KM, Niaura RS, et al. Association of e-Cigarette Use With Discontinuation of Cigarette Smoking Among Adult Smokers Who Were Initially Never Planning to Quit. JAMA Netw Open. 2021;4: e2140880. doi:10.1001/jamanetworkopen.2021.40880	No	Not meeting inclusion criteria	Title and abstract screening
2570	Kasza KA, Edwards KC, Tang Z, Stanton CA, Sharma E, Halenar MJ, et al. Correlates of tobacco product cessation among youth and adults in the USA: findings from the PATH Study Waves 1-3 (2013-2016). Tob Control. 2020;29: s203–s215. doi:10.1136/tobaccocontrol-2019-055255	No	Not meeting inclusion criteria	Title and abstract screening
2571	Kasza KA, Hammond D, Gravely S, O'Connor RJ, Meng G, East K, et al. Associations between nicotine vaping uptake and cigarette smoking cessation vary by smokers' plans to quit: longitudinal findings from the International Tobacco Control Four Country Smoking and Vaping Surveys. Addiction. 2022. doi:10.1111/add.16050	No	Not meeting inclusion criteria	Title and abstract screening
2572	Kasza KA, Hammond D, Gravely S, O'Connor RJ, Meng G, East K, et al. Associations between nicotine vaping uptake and cigarette smoking cessation vary by smokers' plans to quit: longitudinal findings from the International Tobacco Control Four Country Smoking and Vaping Surveys. Addiction. 2023;118: 340–352. doi:10.1111/add.16050	No	Not meeting inclusion criteria	Title and abstract screening
2573	Kasza KA, Hammond D, Reid JL, Rivard C, Hyland A. Youth Use of e-Cigarette Flavor and Device Combinations and Brands Before vs After FDA Enforcement. JAMA Netw Open. 2023;6: e2328805. doi:10.1001/jamanetworkopen.2023.28805	No	Not meeting inclusion criteria	Title and abstract screening
2574	Kasza KA, Rivard C, Seo YS, Reid JL, Gravely S, Fong GT, et al. Use of Electronic Nicotine Delivery Systems or Cigarette Smoking After US Food and Drug Administration-Prioritized Enforcement Against Fruit-Flavored Cartridges. JAMA Netw Open. 2023;6: e2321109. doi:10.1001/jamanetworkopen.2023.21109	No	Not meeting inclusion criteria	Title and abstract screening
2575	Kasza KA, Tang Z, Xiao H, Marshall D, Stanton C, Gross AL, et al. National longitudinal tobacco product cessation rates among US adults from the PATH Study: 2013-2019 (waves 1-5). Tob Control. 2022. doi:10.1136/tc-2022-057323	No	Not meeting inclusion criteria	Title and abstract screening
2576	Kasza KA, Tang Z, Xiao H, Marshall D, Stanton C, Gross A, et al. National longitudinal tobacco product discontinuation rates among US youth from the PATH Study: 2013-2019 (waves 1-5). Tob Control. 2023. doi:10.1136/tc-2022-057729	No	Not meeting inclusion criteria	Title and abstract screening
2577	Katanoda K, Togawa K, Nakamura M. [Is "tobacco harm reduction" possible? Other countries' experiences and perspectives, and how they could inform tobacco control in Japan]. Nihon Koshu Eisei Zasshi Jpn J Public Health. 2023. doi:10.11236/jph.23-076	No	Not meeting inclusion criteria	Title and abstract screening
2578	Kathuria H. Electronic Cigarette Use, Misuse, and Harm. Med Clin North Am. 2022;106: 1081–1092. doi:10.1016/j.mcna.2022.07.009	No	Not meeting inclusion criteria	Title and abstract screening
2579	Kathuria H, Leone FT. COUNTERPOINT: e-Cigarette Use for Harm Reduction in Tobacco Use Disorder? No. Chest. 2021;160: 809–811. doi:10.1016/j.chest.2021.04.044	No	Not meeting inclusion criteria	Title and abstract screening
2580	Kathuria H, Seibert RG, Cobb V, Herbst N, Weinstein ZM, Gowarty M, et al. Perceived barriers to quitting cigarettes among hospitalized smokers with substance use disorders: A mixed methods study. Addict Behav. 2019;95: 41–48. doi:10.1016/j.addbeh.2019.02.017	No	Not meeting inclusion criteria	Title and abstract screening
2581	Kathuria H., Neptune E. Primary and Secondary Prevention of Lung Cancer: Tobacco Treatment. Clin Chest Med. 2020;41: 39–51. doi:10.1016/j.ccm.2019.10.002	No	Not meeting inclusion criteria	Title and abstract screening
2582	Katsaounou P.A. Electronic cigarettes for smoking cessation: an opportunity to readdress smoking cessation treatment. Eur Respir J. 2020;56. doi:10.1183/13993003.00098-2020	No	Not meeting inclusion criteria	Title and abstract screening
2583	Kaur G, Muthumalage T, Rahman I. Mechanisms of toxicity and biomarkers of flavoring and flavor enhancing chemicals in emerging tobacco and non-tobacco products. Toxicol Lett. 2018;288: 143–155. doi:10.1016/j.toxlet.2018.02.025	No	Not meeting inclusion criteria	Title and abstract screening
2584	Kaur R, Gupta S, Tripathi V, Chauhan A, Parashar D, Shankar P, et al. Microbiome based approaches for the degradation of polycyclic aromatic hydrocarbons (PAHs): A current perception. Chemosphere. 2023;341: 139951. doi:10.1016/j.chemosphere.2023.139951	No	Not meeting inclusion criteria	Title and abstract screening
2585	Kaur T, Sharma K, Groban L. Subanesthetic Ketamine Infusion Reducing Symptoms of Depression in a Patient With End-Stage Heart Failure Enrolled in Hospice Care: A Case Report. J Palliat Med. 2023;26: 1435–1438. doi:10.1089/jpm.2022.0430	No	Not meeting inclusion criteria	Title and abstract screening
2586	Kava CM, Soule EK, Seegmiller L, Gold E, Snipes W, Westfield T, et al. "Taking Up a New Problem": Context and Determinants of Pod-Mod Electronic Cigarette Use Among College Students. Qual Health Res. 2021;31: 703–712. doi:10.1177/1049732320971236	No	Not meeting inclusion criteria	Title and abstract screening
2587	Kavousi M., Pisinger C., Barthelemy J.-C., De Smedt D., Koskinas K., Marques-Vidal P., et al. Electronic cigarettes and health with special focus on cardiovascular effects: Position paper of the European Association of Preventive Cardiology (EAPC). Eur J Prev Cardiol. 2021;28: 1552–1566. doi:10.1177/2047487320941993	No	Not meeting inclusion criteria	Title and abstract screening
2588	Kavuluru R, Noh J, Rose SW. Twitter discourse on nicotine as potential prophylactic or therapeutic for COVID-19. Int J Drug Policy. 2022;99: 103470. doi:10.1016/j.drugpo.2021.103470	No	Not meeting inclusion criteria	Title and abstract screening
2589	Kawakami S, Yamato R, Kitamura K, Watanabe Y, Kabasawa K, Takahashi A, et al. Alcohol consumption, smoking, and risk of dementia in community-dwelling Japanese people aged 40-74 years: The Murakami cohort study. Maturitas. 2023;176: 107788. doi:10.1016/j.maturitas.2023.107788	No	Not meeting inclusion criteria	Title and abstract screening
2590	Kcomt L, Evans-Polce RJ, Engstrom CW, Boyd CJ, Veliz PT, West BT, et al. Tobacco Use Among Gender-Varying and Gender-Stable Adolescents and Adults Living in the United States. Nicotine Tob Res. 2022;24: 1498–1503. doi:10.1093/ntr/ntac098	No	Not meeting inclusion criteria	Title and abstract screening
2591	KCT0002760. The safety and efficacy of sural nerve splitting technique. 2018. Available: https://www.cochranelibrary.com/central/doi/10.1002/central/CN-02444383/full	No	Not meeting inclusion criteria	Title and abstract screening

2592	KCT0003262. Evaluation of return-to-work program for cancer survivors. 2018. Available: https://www.cochranelibrary.com/central/doi/10.1002/central/CN-02433804/full	No	Not meeting inclusion criteria	Title and abstract screening
2593	KCT0003756. Comparison of the effect of lung recruitment maneuver on blood pressure according to the posture. 2019. Available: https://www.cochranelibrary.com/central/doi/10.1002/central/CN-02435227/full	No	Not meeting inclusion criteria	Title and abstract screening
2594	KCT0004182. Comparison of neuromuscular blockade recovery coadministered with neostigmine and different dose of calcium gluconate. 2019. Available: https://www.cochranelibrary.com/central/doi/10.1002/central/CN-02433990/full	No	Not meeting inclusion criteria	Title and abstract screening
2595	Keamy-Minor E, McQuoid J, Ling PM. Young adult perceptions of JUUL and other pod electronic cigarette devices in California: a qualitative study. BMJ Open. 2019;9: e026306. doi:10.1136/bmjopen-2018-026306	No	Not meeting inclusion criteria	Title and abstract screening
2596	Keane H., Weier M., Fraser D., Gartner C. "Anytime, anywhere": vaping as social practice. Crit Public Health. 2017;27: 465–476. doi:10.1080/09581596.2016.1250867	No	Not meeting inclusion criteria	Title and abstract screening
2597	Kechter A, Ceasar RC, Simpson KA, Schiff SJ, Dunton GF, Bluthenthal RN, et al. A chocolate cake or a chocolate vape? Young adults describe their relationship with food and weight in the context of nicotine vaping. Appetite. 2022;175: 106075. doi:10.1016/j.appet.2022.106075	No	Not meeting inclusion criteria	Title and abstract screening
2598	Kechter A, Simpson KA, Ceasar RC, Schiff SJ, Yamaguchi N, Bluthenthal RN, et al. Trajectories of Nicotine Use Leading to Dual and Cyclical Tobacco Product Use in Young Adults. Nicotine Tob Res. 2022;24: 986–993. doi:10.1093/ntr/ntab249	No	Not meeting inclusion criteria	Title and abstract screening
2599	Kechter A, Schiff SJ, Simpson KA, Ceasar RC, Braymiller JL, McConnell R, et al. Young adult perspectives on their respiratory health symptoms since vaping. Subst Abuse. 2020; No-Specified. doi:10.1080/08897077.2020.1856290https://dx.doi.org/10.1080/08897077.2020.1856290	No	Not meeting inclusion criteria	Title and abstract screening
2600	Kechter A, Schiff SJ, Simpson KA, Ceasar RC, Braymiller JL, McConnell R, et al. Young adult perspectives on their respiratory health symptoms since vaping. Subst Abuse. 2021;42: 428–432. doi:10.1080/08897077.2020.1856290	No	Not meeting inclusion criteria	Title and abstract screening
2601	Kehl KL, Riely GJ, Lepisto EM, Lavery JA, Warner JL, LeNoue-Newton ML, et al. Correlation Between Surrogate End Points and Overall Survival in a Multi-institutional Clinicogenomic Cohort of Patients With Non–Small Cell Lung or Colorectal Cancer. JAMA Netw Open. 2021;4: e2117547–e2117547. doi:10.1001/jamanetworkopen.2021.17547	No	Not meeting inclusion criteria	Title and abstract screening
2602	Keijsers M., Vega-Corredor M.C., Tomintz M., Hoermann S. Virtual reality technology use in cigarette craving and smoking interventions (i "virtually" quit): Systematic review. J Med Internet Res. 2021;23: e24307. doi:10.2196/24307	No	Not meeting inclusion criteria	Title and abstract screening
2603	Kekere V., Onyeaka H., Muoghalu C., Reng G., Olupona T. 3.104 Prevalence and Correlates of US Adult Opinions on Restricting Exposure of Children to Smoking in Movies: 2020 Health Information National Trends Survey. J Am Acad Child Adolesc Psychiatry. 2022;61: S261–S262. doi:10.1016/j.jaac.2022.09.382	No	Not meeting inclusion criteria	Title and abstract screening
2604	Kelesidis T., Tran E., Nguyen R., Zhang Y., Sosa G., Middlekauff H.R. Association of 1 Vaping Session with Cellular Oxidative Stress in Otherwise Healthy Young People with No History of Smoking or Vaping: A Randomized Clinical Crossover Trial. JAMA Pediatr. 2021;175: 1174–1176. doi:10.1001/jamapediatrics.2021.2351	No	Not meeting inclusion criteria	Title and abstract screening
2605	Kelesidis T., Zhang Y., Tran E., Sosa G., Middlekauff H.R. Increased Expression of Proatherogenic Proteins in Immune Cell Subtypes in Tobacco Cigarette Smokers But Not in Electronic Cigarette Vapers. Can J Cardiol. 2021;37: 1175–1180. doi:10.1016/j.cjca.2021.05.006	No	Not meeting inclusion criteria	Title and abstract screening
2606	Kelesidis T, Zhang Y, Tran E, Sosa G, Middlekauff HR. Expression of Key Inflammatory Proteins Is Increased in Immune Cells From Tobacco Cigarette Smokers But Not Electronic Cigarette Vapers: Implications for Atherosclerosis. J Am Heart Assoc. 2021;10: e019324. doi:10.1161/JAHA.120.019324	No	Not meeting inclusion criteria	Title and abstract screening
2607	Keller-Hamilton B, Curran H, Stevens EM, Slater MD, Lu B, Roberts ME, et al. Associations between Cognitive and Affective Responses to Tobacco Advertisements and Tobacco Use Incidence: A Four-Year Prospective Study among Adolescent Boys. Int J Env Res Public Health. 2021;18. doi:10.3390/ijerph182111666	No	Not meeting inclusion criteria	Title and abstract screening
2608	Keller-Hamilton B, Fioritto M, Klein EG, Brinkman MC, Pennell ML, Nini P, et al. Visual attention to blu's parody warnings and the FDA's warning on e-cigarette advertisements. Addict Behav. 2022;125: 107169. doi:10.1016/j.addbeh.2021.107169	No	Not meeting inclusion criteria	Title and abstract screening
2609	Kendall AD, Hedeker D, Diviak KR, Mermelstein RJ. The Mood Boost from Tobacco Cigarettes is More Erratic with the Additions of Cannabis and Alcohol. Nicotine Tob Res. 2022;24: 1169–1176. doi:10.1093/ntr/ntac001	No	Not meeting inclusion criteria	Title and abstract screening
2610	Kennecke HF, O'Callaghan CJ, Loree JM, Moloo H, Auer R, Jonker DJ, et al. Neoadjuvant Chemotherapy, Excision, and Observation for Early Rectal Cancer: The Phase II NEO Trial (CCTG CO.28) Primary End Point Results. J Clin Oncol. 2023;41: 233–242. doi:10.1200/JCO.22.00184	No	Not meeting inclusion criteria	Title and abstract screening
2611	Kenney AE, Bedoya SZ, Gerhardt CA, Young-Saleme T, Wiener L. End of life communication among caregivers of children with cancer: A qualitative approach to understanding support desired by families. Palliat Support Care. 2021;19: 715–722. doi:10.1017/S1478951521000067	No	Not meeting inclusion criteria	Title and abstract screening
2612	Kenny P, Street DJ, Hall J, Agar M, Phillips J. Valuing End-of-Life Care for Older People with Advanced Cancer: Is Dying at Home Important? Patient. 2021;14: 803–813. doi:10.1007/s40271-021-00517-z	No	Not meeting inclusion criteria	Title and abstract screening
2613	Kent J., Mok G., Austin E. Nicotine toxicity from repeat use of nicotine pouches. Clin Toxicol. 2023;61: 56. doi:10.1080/15563650.2023.2233835	No	Not meeting inclusion criteria	Title and abstract screening
2614	Kenzie ES, Seater M, Wakeland W, Coronado GD, Davis MM. System dynamics modeling for cancer prevention and control: A systematic review. PloS One. 2023;18: e0294912. doi:10.1371/journal.pone.0294912	No	Not meeting inclusion criteria	Title and abstract screening
2615	Keogan S, Alonso T, Sunday S, Tigova O, Fernandez E, Lopez MJ, et al. Lung function changes in patients with chronic obstructive pulmonary disease (COPD) and asthma exposed to secondhand smoke in outdoor areas. J Asthma Off J Assoc Care Asthma. 2021;58: 1169–1175. doi:10.1080/02770903.2020.1766062	No	Not meeting inclusion criteria	Title and abstract screening
2616	Keogan S, Li S, Clancy L. Allen Carr's Easyway to Stop Smoking - A randomised clinical trial. Tob Control. 2019;28: 414–419. doi:10.1136/tobaccocontrol-2018-054243	No	Not meeting inclusion criteria	Title and abstract screening
2617	Kephart L, Rastogi R, Song G, Ursprung WWS, Kingsley M, Bharel M. Implementation and evaluation of the public health emergency response to the 2019 outbreak of e-cigarette and vaping product use-associated lung injury in Massachusetts,USA. Public Health. 2022;204: 25–32. doi:10.1016/j.puhe.2021.12.020	No	Not meeting inclusion criteria	Title and abstract screening
2618	Kertes J, Neumark Y, Grunhaus L, Stein-Reisner O. Comparison of Perceptions and Smoking Cessation Experiences Between Smokers With and Without Serious Mental Illness in a Large Health Maintenance Organization. J Dual Diagn. 2021;17: 284–295. doi:10.1080/15504263.2021.1979348	No	Not meeting inclusion criteria	Title and abstract screening
2619	Khachatoorian C, McWhirter KJ, Luo W, Pankow JF, Talbot P. Tracing the movement of electronic cigarette flavor chemicals and nicotine from refill fluids to aerosol, lungs, exhale, and the environment. Chemosphere. 2022;286: 131494. doi:10.1016/j.chemosphere.2021.131494	No	Not meeting inclusion criteria	Title and abstract screening
2620	Khader H.A. Use and beliefs about e-cigarette among college students in Jordan. Jordan Med J. 2020;54: 89–98.	No	Not meeting inclusion criteria	Title and abstract screening

2621	Khadka S, Awasthi M, Lamichhane RR, Ojha C, Mamudu HM, Lavie CJ, et al. The Cardiovascular Effects of Electronic Cigarettes. Curr Cardiol Rep. 2021;23: 40. doi:10.1007/s11886-021-01469-4	No	Not meeting inclusion criteria	Title and abstract screening
2622	Khaled Z., Dahmash E.Z., Koner J., Al Ani R., Alyami H., Naser A.Y. Assessment of vaping devices as an alternative respiratory drug delivery system. Drug Dev Ind Pharm. 2022. doi:10.1080/03639045.2022.2123926	No	Not meeting inclusion criteria	Title and abstract screening
2623	Khalfaoui L, Mukhtasimova N, Kelley B, Wells N, Teske JJ, Roos BB, et al. Functional α7 nicotinic receptors in human airway smooth muscle increase intracellular calcium concentration and contractility in asthmatics. Am J Physiol Lung Cell Mol Physiol. 2023;325: L17–L29. doi:10.1152/ajplung.00260.2022	No	Not meeting inclusion criteria	Title and abstract screening
2624	KHALID F., KASHYAP R., MOHAN G., TAYYEB M., S LIVORNESE D. E-CIGARETTE OR VAPING PRODUCT USE ASSOCIATED LUNG INJURY: A DIAGNOSTIC DILEMMA. Chest. 2022;162: A1209. doi:10.1016/j.chest.2022.08.967	No	Not meeting inclusion criteria	Title and abstract screening
2625	Khan A., Zhou Y., Young K., Bhele S., Mueller J., Alroumi F. "His-story": The clinical key leading to a diagnosis of evali. Am J Respir Crit Care Med. 2021;203. doi:10.1164/ajrccm-conference.2021.203.1_MeetingAbstracts.A2138	No	Not meeting inclusion criteria	Title and abstract screening
2626	Khan AF, Seow H, Sutradhar R, Peacock S, Chan KK-W, Burge F, et al. Quality of End-of-Life Cancer Care in Canada: A 12-Year Retrospective Analysis of Three Provinces' Administrative Health Care Data Evaluating Changes over Time. Curr Oncol. 2021;28: 4673–4685. doi:10.3390/curroncol28060394	No	Not meeting inclusion criteria	Title and abstract screening
2627	Khan L. Substance use in adolescents: Latest trends. Pediatr Ann. 2019;48: e418–e422. doi:10.3928/19382359-20191018-02	No	Not meeting inclusion criteria	Title and abstract screening
2628	KHAN R., HELLER G., ADRIAN CASIPIT B., AL SONA M., BENZAQUEN S. ACUTE HYPOXIC RESPIRATORY FAILURE DUE TO ORGANIZING PNEUMONIA SECONDARY TO CHRONIC MARIJUANA SMOKING. Chest. 2023;164: A3362–A3363. doi:10.1016/j.chest.2023.07.2191	No	Not meeting inclusion criteria	Title and abstract screening
2629	Khanagar SB, Siddeeqh S, Khinda V, Khinda P, Divakar DD, Jhugroo C. Impact of electronic cigarette smoking on the Saudi population through the analysis of literature: A systematic review. J Oral Maxillofac Pathol JOMFP. 2019;23: 473. doi:10.4103/jomfp.JOMFP_141_19	No	Not meeting inclusion criteria	Title and abstract screening
2630	Khanna D., Lescoat A., Roofeh D., Bernstein E.J., Kazerooni E.A., Roth M.D., et al. Systemic Sclerosis-Associated Interstitial Lung Disease: How to Incorporate Two Food and Drug Administration-Approved Therapies in Clinical Practice. Arthritis Rheumatol. 2022;74: 13–27. doi:10.1002/art.41933	No	Not meeting inclusion criteria	Title and abstract screening
2631	Khanna N, Klyushnenkova E, Gaynor A, Dark M, Melamed J, Bennett M, et al. Integrating a Systematic, Comprehensive E-Cigarette and Vaping Assessment Tool into the Electronic Health Record. J Am Board Fam Med. 2023;36: 405–413. doi:10.3122/jabfm.2022.220410R1	No	Not meeting inclusion criteria	Title and abstract screening
2632	KHASKIA Y., PARFIANOWICZ D., CARLSON J., R. MILLER B. CIGARETTE'S REVENGE: ACUTE EOSINOPHILIC PNEUMONIA INDUCED BY SMOKING. Chest. 2023;164: A3221. doi:10.1016/j.chest.2023.07.2110	No	Not meeting inclusion criteria	Title and abstract screening
2633	Khayat A, Berg CJ, Levine H, Rodnay M, Abroms L, Romm KF, et al. PMI's IQOS and cigarette ads in Israeli media: a content analysis across regulatory periods and target population subgroups. Tob Control. 2022. doi:10.1136/tc-2022-057671	No	Not meeting inclusion criteria	Title and abstract screening
2634	Khayat A, Levine H, Berg CJ, Shauly-Aharonov M, Manor O, Abroms L, et al. IQOS and cigarette advertising across regulatory periods and population groups in Israel: a longitudinal analysis. Tob Control. 2022. doi:10.1136/tc-2022-057585	No	Not meeting inclusion criteria	Title and abstract screening
2635	Khoj L., Zaga V., Amram D.L., Hosein K., Pistone G., Bisconti M., et al. Effects of cannabis smoking on the respiratory system: A state-of-the-art review. Respir Med. 2024;221: 107494. doi:10.1016/j.rmed.2023.107494	No	Not meeting inclusion criteria	Title and abstract screening
2636	Khoj L, Zaga V, Amram DL, Hosein K, Pistone G, Bisconti M, et al. Effects of cannabis smoking on the respiratory system: A state-of-the-art review. Respir Med. 2023;221: 107494. doi:10.1016/j.rmed.2023.107494	No	Not meeting inclusion criteria	Title and abstract screening
2637	Khongtor O., Yunibhand J. Characteristics of dual user smokers who called Thailand National Quitline. Tob Induc Dis. 2021;19. doi:10.18332/tid/141395	No	Not meeting inclusion criteria	Title and abstract screening
2638	Khouja JN, Munafò MR. Commentary on Tattan-Birch et al.: How might the rise in popularity of disposable vapes among young adults impact policy in the United Kingdom? Addiction. 2022. doi:10.1111/add.16067	No	Not meeting inclusion criteria	Title and abstract screening
2639	Khouja JN, Wootton RE, Taylor AE, Davey Smith G, Munafò MR. Association of genetic liability to smoking initiation with e-cigarette use in young adults: A cohort study. PLoS Med. 2021;18: e1003555. doi:10.1371/journal.pmed.1003555	No	Not meeting inclusion criteria	Title and abstract screening
2640	Kiani I.A., Malhi N.K. Adolescent e-cigarette use: A public health crisis. Curr Psychiatry. 2020;19: e5. doi:10.12788/cp.0043	No	Not meeting inclusion criteria	Title and abstract screening
2641	Kilibarda B, Vukovic D, Krstev S. Prevalence and correlates of concurrent use of cigarettes, electronic cigarettes, and waterpipes among Serbian youth. Tob Induc Dis. 2019;17: 66. doi:10.18332/tid/111357	No	Not meeting inclusion criteria	Title and abstract screening
2642	Kim CY, Paek YJ, Seo HG, Cheong YS, Lee CM, Park SM, et al. Dual use of electronic and conventional cigarettes is associated with higher cardiovascular risk factors in Korean men. Sci Rep. 2020;10: 5612. doi:10.1038/s41598-020-62545-3	No	Not meeting inclusion criteria	Title and abstract screening
2643	Kim I, Begay C, Ma HJ, Orozco FR, Rogers CJ, Valente TW, et al. E-Cigarette-Related Health Beliefs Expressed on Twitter Within the U.S. AJPM Focus. 2023;2: 100067. doi:10.1016/j.focus.2023.100067	No	Not meeting inclusion criteria	Title and abstract screening
2644	Kim J, Kambari Y, Taggar A, Quilty LC, Selby P, Caravaggio F, et al. A Measure of Illness Awareness in Individuals With Nicotine Dependence-Nicotine Use Awareness and Insight Scale. Nicotine Tob Res. 2022;24: 536–543. doi:10.1093/ntr/ntab235	No	Not meeting inclusion criteria	Title and abstract screening
2645	Kim J, Lee S. Factors associated with Korean adolescent's e-cigarette use by the severity level of generalized anxiety disorder (GAD-7). J Affect Disord. 2023;340: 129–138. doi:10.1016/j.jad.2023.08.011	No	Not meeting inclusion criteria	Title and abstract screening
2646	Kim J, Khalil G, Moor A. 261. Transforming Influence to Wellness: identifying Adolescentsâ€™ Affect and Preferences During the Preliminary Design of a Social Board Game for Tobacco Prevention. 2020;66: S132-S133. doi:10.1016/j.jadohealth.2019.11.264	No	Not meeting inclusion criteria	Title and abstract screening
2647	Kim J., Khalil G.E., Moor A. 261. Transforming Influence to Wellness: Identifying Adolescents' Affect and Preferences During the Preliminary Design of a Social Board Game for Tobacco Prevention. J Adolesc Health. 2020;66: S132–S133. doi:10.1016/j.jadohealth.2019.11.264	No	Not meeting inclusion criteria	Title and abstract screening
2648	Kim J.H., Chang I.B., Kim Y.H., Min C.Y., Yoo D.M., Choi H.G. Association Between Various Types or Statuses of Smoking and Subjective Cognitive Decline Based on a Community Health Survey of Korean Adults. Front Neurol. 2022;13: 810830. doi:10.3389/fneur.2022.810830	No	Not meeting inclusion criteria	Title and abstract screening
2649	Kim J-H, Yoo SH, Keam B, Heo DS. The impact of palliative care consultation on reducing antibiotic overuse in hospitalized patients with terminal cancer at the end of life: a propensity score-weighting study. J Antimicrob Chemother JAC. 2023;78: 302–308. doi:10.1093/jac/dkac405	No	Not meeting inclusion criteria	Title and abstract screening

2650	Kim J, Keegan TH. Characterizing risky alcohol use, cigarette smoking, e-cigarette use, and physical inactivity among cancer survivors in the USA-a cross-sectional study. J Cancer Surviv Res Pract. 2022. doi:10.1007/s11764-022-01245-5	No	Not meeting inclusion criteria	Title and abstract screening
2651	Kim K, Picciotto MR. Nicotine addiction: More than just dopamine. Curr Opin Neurobiol. 2023;83: 102797. doi:10.1016/j.conb.2023.102797	No	Not meeting inclusion criteria	Title and abstract screening
2652	Kim MH, Kang SI, Cho JR, Lee J, Yang IJ, Suh JW, et al. Objective recovery time with end-to-side versus side-to-side anastomosis after laparoscopic right hemicolectomy for colon cancer: a randomized controlled trial. Surg Endosc. 2022;36: 2499–2506. doi:10.1007/s00464-021-08536-5	No	Not meeting inclusion criteria	Title and abstract screening
2653	Kim RS, Weinberger AH, Chander G, Sulkowski MS, Norton B, Shuter J. Cigarette Smoking in Persons Living with Hepatitis C: The National Health and Nutrition Examination Survey (NHANES), 1999-2014. Am J Med. 2018;131: 669–675. doi:10.1016/j.amjmed.2018.01.011	No	Not meeting inclusion criteria	Title and abstract screening
2654	Kim S. Changes in Multiple and Different Tobacco Product Use Behaviors in Women Before and During Pregnancy: An Analysis of Longitudinal Population Assessment of Tobacco and Health Data. Am J Prev Med. 2020;59: 588–592. doi:10.1016/j.amepre.2020.05.006	No	Not meeting inclusion criteria	Title and abstract screening
2655	Kim S, Jo K. Multiple Tobacco Product Use among Adolescents with Asthma in Korea. Int J Env Res Public Health. 2022;19. doi:10.3390/ijerph19159633	No	Not meeting inclusion criteria	Title and abstract screening
2656	Kim Y. Blood and Tissue Advanced Glycation End Products as Determinants of Cardiometabolic Disorders Focusing on Human Studies. Nutrients. 2023;15: 2002. doi:10.3390/nu15082002	No	Not meeting inclusion criteria	Title and abstract screening
2657	Kimber C, Cox S, Frings D, Albery IP, Dawkins L. Development and testing of relative risk-based health messages for electronic cigarette products. Harm Reduct J. 2021;18: 96. doi:10.1186/s12954-021-00540-1	No	Not meeting inclusion criteria	Title and abstract screening
2658	Kimber C, Frings D, Cox S, Albery I, Dawkins L. The effects of the European e-cigarette health warnings and comparative health messages on non-smokers' and smokers' risk perceptions and behavioural intentions. BMC Public Health. 2018;18: 1259. doi:10.1186/s12889-018-6161-7	No	Not meeting inclusion criteria	Title and abstract screening
2659	Kimber C, Frings D, Cox S, Albery IP, Dawkins L. Communicating the relative health risks of E-cigarettes: An online experimental study exploring the effects of a comparative health message versus the EU nicotine addiction warnings on smokers' and non-smokers' risk perceptions and behavioural intentions. Addict Behav. 2020;101: 106177. doi:10.1016/j.addbeh.2019.106177	No	Not meeting inclusion criteria	Title and abstract screening
2660	Kimber C, Sideropoulos V, Cox S, Frings D, Naughton F, Brown J, et al. E-cigarette support for smoking cessation: Identifying the effectiveness of intervention components in an on-line randomized optimization experiment. Addiction. 2023;118: 2105–2117. doi:10.1111/add.16294	No	Not meeting inclusion criteria	Title and abstract screening
2661	Kimber C, Zaidell L, Hunter S, Cox S, Notley C, Dawkins L. Comparing the Effects of the EU- Versus the US-JUUL Pod in a Sample of UK Smokers: Nicotine Absorption, Satisfaction, and Other Nicotine-Related Subjective Effects. Nicotine Tob Res. 2023;25: 1109–1115. doi:10.1093/ntr/ntac289	No	Not meeting inclusion criteria	Title and abstract screening
2662	Kimber CF, Soar K, Dawkins LE. Changes in puffing topography and subjective effects over a 2-week period in e-cigarette naïve smokers: Effects of device type and nicotine concentrations. Addict Behav. 2021;118: 106909. doi:10.1016/j.addbeh.2021.106909	No	Not meeting inclusion criteria	Title and abstract screening
2663	King B, Borland R, Le Grande M, Diaz D, O'Connor R, East K, et al. Associations between smokers' knowledge of causes of smoking harm and related beliefs and behaviors: Findings from the International Tobacco Control (ITC) Four Country Smoking and Vaping Survey. PLoS One. 2023;18: e0292856. doi:10.1371/journal.pone.0292856	No	Not meeting inclusion criteria	Title and abstract screening
2664	King JL, Reboussin D, Cornacchione Ross J, Wiseman KD, Wagoner KG, Sutfin EL. Polytobacco Use Among a Nationally Representative Sample of Adolescent and Young Adult E-Cigarette Users. J Adolesc Health Off Publ Soc Adolesc Med. 2018;63: 407–412. doi:10.1016/j.jadohealth.2018.04.010	No	Not meeting inclusion criteria	Title and abstract screening
2665	King JL, Reboussin BA, Spangler J, Cornacchione Ross J, Sutfin EL. Tobacco product use and mental health status among young adults. Addict Behav. 2018;77: 67–72. doi:10.1016/j.addbeh.2017.09.012	No	Not meeting inclusion criteria	Title and abstract screening
2666	King W. What Will It Take to End Cancer As We Know It? Oncol Pharm. 2023;16: 1–6.	No	Not meeting inclusion criteria	Title and abstract screening
2667	Kinney W.H., Beckstrom C., Snyder T., Judiscak P., Miller P., Marett J., et al. Electronic-cigarette or vaping associated lung injury (EVALI). Appl Radiol. 2020;49: 42–44.	No	Not meeting inclusion criteria	Title and abstract screening
2668	Kinnucan J, Swaminath A, Sangha M, Zeiger J. Real world dispensary data for IBD patients using medical cannabis. 2021;116: S436-. doi:10.14309/01.ajg.0000777220.73380.41	No	Not meeting inclusion criteria	Title and abstract screening
2669	Kinnunen JM, Ollila H, Minkkinen J, Lindfors PL, Timberlake DS, Rimpela AH. Nicotine matters in predicting subsequent smoking after e-cigarette experimentation: A longitudinal study among Finnish adolescents. Drug Alcohol Depend. 2019;201: 182–187. doi:10.1016/j.drugalcdep.2019.04.019	No	Not meeting inclusion criteria	Title and abstract screening
2670	Kinnunen JM, Rimpela AH, Lindfors PL, Clancy L, Alves J, Hoffmann L, et al. Electronic cigarette use among 14- to 17-year-olds in Europe. Eur J Public Health. 2021;31: 402–408. doi:10.1093/eurpub/ckaa145	No	Not meeting inclusion criteria	Title and abstract screening
2671	Kirby M., Fuentes F., Giri B. Danger in the diffuser: Essential oil inhalation induced hypersensitivity pneumonitis. Am J Respir Crit Care Med. 2021;203. doi:10.1164/ajrccm-conference.2021.203.1_MeetingAbstracts.A2371	No	Not meeting inclusion criteria	Title and abstract screening
2672	Kirby T. Concerns over increased vaping in schoolchildren. Lancet Respir Med. 2019;7: 211–212. doi:10.1016/S2213-2600%2819%2930027-X	No	Not meeting inclusion criteria	Title and abstract screening
2673	Kirk S., Huster M., Elliot E., Powers J. PEDIATRIC ERYTHROCYTOSIS: ETIOLOGIES AND CLINICAL CHARACTERISTICS. Pediatr Blood Cancer. 2023;70. doi:10.1002/pbc.30390	No	Not meeting inclusion criteria	Title and abstract screening
2674	Kirkland SW, Yang EH, Clua MG, Kruhlak M, Villa-Roel C, Elwi A, et al. Comparison of the Management and Short-Term Outcomes between Patients with Advanced Cancer and Other End-of-Life Conditions Presenting to Two Canadian Emergency Departments. J Palliat Med. 2022;25: 915–924. doi:10.1089/jpm.2021.0519	No	Not meeting inclusion criteria	Title and abstract screening
2675	Kirkpatrick MG, Cruz TB, Unger JB, Herrera J, Schiff S, Allem JP. Cartoon-based e-cigarette marketing: Associations with susceptibility to use and perceived expectations of use. Drug Alcohol Depend. 2019;201: 109–114. doi:10.1016/j.drugalcdep.2019.04.018	No	Not meeting inclusion criteria	Title and abstract screening
2676	Kirshenbaum AP, Hughes JR. Reinforcement enhancement by nicotine: A novel abuse-liability assessment of e-cigarettes in young adults. Exp Clin Psychopharmacol. 2021. doi:10.1037/pha0000496	No	Not meeting inclusion criteria	Title and abstract screening
2677	Kishino T, Monden N, Akisada N, Hayashi Y, Nakamura M, Hashimoto K, et al. Comparison of the accuracy of clinical prediction of survival and palliative prognostic index for patients with head and neck squamous cell carcinoma in the end-of-life setting. Auris Nasus Larynx. 2022;49: 133–140. doi:10.1016/j.anl.2021.06.003	No	Not meeting inclusion criteria	Title and abstract screening
2678	Kittaneh AA, Sidhu NK, Tackett AP, Lechner WV. Effects of Negative Emotion on Abstinence Induced Change in Urge to Vape and Measures of Vaping Dependence. Subst Use Misuse. 2021;56: 768–776. doi:10.1080/10826084.2021.1892143	No	Not meeting inclusion criteria	Title and abstract screening

2679	Kitti PM, Anttonen AM, Leskelä R-L, Saarto T. End-of-life care of patients with esophageal or gastric cancer: decision making and the goal of care. Acta Oncol. 2022;61: 1173–1178. doi:10.1080/0284186X.2022.2114379	No	Not meeting inclusion criteria	Title and abstract screening
2680	Kiuchi K, Hasegawa K, Watanabe M, Motegi E, Kosaka N, Fukasawa I. Clinical indicators useful in decision-making about palliative chemotherapy for end-of-life ovarian cancer patients. Arch Gynecol Obstet. 2022;305: 425–430. doi:10.1007/s00404-021-06162-z	No	Not meeting inclusion criteria	Title and abstract screening
2681	Kizhakke Puliyakote AS, Elliott AR, Sa RC, Anderson KM, Crotty Alexander LE, Hopkins SR. Vaping disrupts ventilation-perfusion matching in asymptomatic users. J Appl Physiol Bethesda Md 1985. 2021;130: 308–317. doi:10.1152/japplphysiol.00709.2020	No	Not meeting inclusion criteria	Title and abstract screening
2682	Klawinski D, Hanna I, Breslin NK, Katzenstein HM, Indelicato DJ. Vaping the Venom: Oral Cavity Cancer in a Young Adult With Extensive Electronic Cigarette Use. Pediatrics. 2021;147. doi:10.1542/peds.2020-022301	No	Not meeting inclusion criteria	Title and abstract screening
2683	Klein EG, Czaplicki L, Berman M, Emery S, Schillo B. Visual Attention to the Use of #ad versus #sponsored on e-Cigarette Influencer Posts on Social Media: A Randomized Experiment. J Health Commun. 2020;25: 925–930. doi:10.1080/10810730.2020.1849464	No	Not meeting inclusion criteria	Title and abstract screening
2684	Klein J., Cohen K., Fales A., Pham T., Choi H., Collins B.N., et al. COVID-19 Pandemic and Inner City Youth E-Cigarette Use. Am J Respir Crit Care Med. 2022;205. doi:10.1164/ajrccm-conference.2022.205.1_MeetingAbstracts.A1167	No	Not meeting inclusion criteria	Title and abstract screening
2685	Klein JD. E-Cigarettes: A 1-Way Street to Traditional Smoking and Nicotine Addiction for Youth. Pediatrics. 2018;141. doi:10.1542/peds.2017-2850	No	Not meeting inclusion criteria	Title and abstract screening
2686	Klein JD. Another Study Shows Electronic Cigarettes Harm Lungs: It Is Time for Researchers to Move from the Tobacco Playbook to a Tobacco Endgame. Am J Respir Crit Care Med. 2022;205: 1265–1266. doi:10.1164/rccm.202201-0169ED	No	Not meeting inclusion criteria	Title and abstract screening
2687	Klein MD, Sokol NA, Stroud LR. Electronic Cigarettes: Common Questions and Answers. Am Fam Physician. 2019;100: 227–235.	No	Not meeting inclusion criteria	Title and abstract screening
2688	Klemperer EM. A randomized trial to compare switching to very low nicotine content cigarettes versus reducing cigarettes per day. Diss Abstr Int Sect B Sci Eng. 2018;79: No-Specified.	No	Not meeting inclusion criteria	Title and abstract screening
2689	Klemperer EM, Bunn JY, Palmer AM, Smith TT, Toll BA, Cummings KM, et al. E-cigarette cessation and transitions in combusted tobacco smoking status: Longitudinal findings from the US FDA PATH Study. Addiction. 2023. doi:10.1111/add.16141	No	Not meeting inclusion criteria	Title and abstract screening
2690	Klemperer EM, Luo X, Jensen J, al'Absi M, Cinciripini PM, Robinson JD, et al. Smoking abstinence and cessation-related outcomes one month after an immediate versus gradual reduction in nicotine content of cigarettes. Prev Med. 2022; 107175. doi:10.1016/j.ypmed.2022.107175	No	Not meeting inclusion criteria	Title and abstract screening
2691	Kligerman S. The Immediate Physiologic Effects of Vaping on Pulmonary Perfusion Revealed at MRI. Radiology. 2022;304: 205–207. doi:10.1148/radiol.220325	No	Not meeting inclusion criteria	Title and abstract screening
2692	Kloner RA. Marijuana and electronic cigarettes on cardiac arrhythmias. Heart Rhythm. 2023;20: 87–88. doi:10.1016/j.hrthm.2022.09.022	No	Not meeting inclusion criteria	Title and abstract screening
2693	Kniesburges S, Schlegel P, Peters G, Westphalen C, Jakubaß B, Veltrup R, et al. Effects of surgical masks on aerosol dispersion in professional singing. J Expo Sci Env Epidemiol. 2022;32: 727–734. doi:10.1038/s41370-021-00385-7	No	Not meeting inclusion criteria	Title and abstract screening
2694	Köberlein M, Hermann L, Gantner S, Tur B, Peters G, Westphalen C, et al. Impulse dispersion of aerosols during playing the recorder and evaluation of safety measures. PLoS One. 2022;17: e0266991. doi:10.1371/journal.pone.0266991	No	Not meeting inclusion criteria	Title and abstract screening
2695	Koczkodaj P, Cuchi P, Ciuba A, Gliwska E, Peruga A. Point of Sale Advertising and Promotion of Cigarettes, Electronic Cigarettes, and Heated Tobacco Products in Warsaw, Poland-A Pilot Study. Int J Env Res Public Health. 2021;18. doi:10.3390/ijerph182413002	No	Not meeting inclusion criteria	Title and abstract screening
2696	Koenig JFL, Asendorf T, Simon A, Bleckmann A, Truemper L, Wulf G, et al. "SpezPat"- common advance directives versus disease-centred advance directives: a randomised controlled pilot study on the impact on physicians' understanding of non-small cell lung cancer patients' end-of-life decisions. BMC Palliat Care. 2022;21: 1–10. doi:10.1186/s12904-022-01057-5	No	Not meeting inclusion criteria	Title and abstract screening
2697	Kohn R., Vachani A., Small D., Stephens-Shields A.J., Sheu D., Madden V.L., et al. Comparing Smoking Cessation Interventions among Underserved Patients Referred for Lung Cancer Screening A Pragmatic Trial Protocol. Ann Am Thorac Soc. 2022;19: 303–314. doi:10.1513/AnnalsATS.202104-499SD	No	Not meeting inclusion criteria	Title and abstract screening
2698	Kokila S, Prasad H, Rajmohan M, Kumar Srichinthu K, Mahalakshmi L, Shanmuganathan S, et al. Evaluation of Micronuclei and Cytomorphometric Changes in Patients with Different Tobacco Related Habits Using Exfoliated Buccal Cells. Asian Pac J Cancer Prev APJCP. 2021;22: 1851–1855. doi:10.31557/APJCP.2021.22.6.1851	No	Not meeting inclusion criteria	Title and abstract screening
2699	Kolenda K.-D. Tobacco smoking, electronic inhalation products and tobacco heaters. Health sequelae and potential health risks. Internist Prax. 2019;60: 511–525.	No	Not meeting inclusion criteria	Title and abstract screening
2700	Kolli A.R., Calvino-Martin F., Kuczaj A.K., Wong E.T., Titz B., Xiang Y., et al. Deconvolution of Systemic Pharmacokinetics Predicts Inhaled Aerosol Dosimetry of Nicotine. Eur J Pharm Sci. 2023;180: 106321. doi:10.1016/j.ejps.2022.106321	No	Not meeting inclusion criteria	Title and abstract screening
2701	Kolodner G, DiClemente CC, Miller MM. Nicotine Addiction: A Burning Issue in Addiction Psychiatry. Psychiatr Clin North Am. 2022;45: 451–465. doi:10.1016/j.psc.2022.04.003	No	Not meeting inclusion criteria	Title and abstract screening
2702	Kondracki AJ, Li W, Kalan ME, Ben Taleb Z, Ibrahimou B, Bursac Z. Changes in the National Prevalence of Current E-Cigarette, Cannabis, and Dual Use among Reproductive Age Women (18-44 Years Old) in the United States, 2013-2016. Subst Use Misuse. 2022;57: 833–840. doi:10.1080/10826084.2022.2046092	No	Not meeting inclusion criteria	Title and abstract screening
2703	Kong AY, Gottfredson NC, Ribisl KM, Baggett CD, Delamater PL, Golden SD. Associations of County Tobacco Retailer Availability With U.S. Adult Smoking Behaviors, 2014-2015. Am J Prev Med. 2021;61: e139–e147. doi:10.1016/j.amepre.2021.03.015	No	Not meeting inclusion criteria	Title and abstract screening
2704	Kong G., Bold K.W., Cavallo D.A., Davis D.R., Jackson A., Krishnan-Sarin S. Informing the development of adolescent e-cigarette cessation interventions: A qualitative study. Addict Behav. 2021;114: 106720. doi:10.1016/j.addbeh.2020.106720	No	Not meeting inclusion criteria	Title and abstract screening
2705	Kong G, Krishnan-Sarin S. A call to end the epidemic of adolescent e-cigarette use. Drug Alcohol Depend. 2017;174: 215–221. doi:10.1016/j.drugalcdep.2017.03.001https://dx.doi.org/10.1016/j.drugalcdep.2017.03.001	No	Not meeting inclusion criteria	Title and abstract screening
2706	Konjevod M, Nikolac Perkovic M, Svob Strac D, Uzun S, Nedic Erjavec G, Kozumplik O, et al. Significant association of mu-opioid receptor 1 haplotype with tobacco smoking in healthy control subjects but not in patients with schizophrenia and alcohol dependence. Psychiatry Res. 2020;291: 113278. doi:10.1016/j.psychres.2020.113278	No	Not meeting inclusion criteria	Title and abstract screening
2707	Koo EY, Kozak KJ, Achim V, Wenig BL. E-cigarette use and tobacco harm reduction: Pilot survey study evaluating perspectives of head and neck surgeons. Head Neck. 2022;44: 582–585. doi:10.1002/hed.26949	No	Not meeting inclusion criteria	Title and abstract screening

2708	Kooblall M., Nash D. E - Cigarette - Why do we forget to document in the medical notes? Ir Med J. 2017;110: 521.	No	Not meeting inclusion criteria	Title and abstract screening
2709	Koroukian SM, Douglas SL, Vu L, Fein HL, Gairola R, Warner DF, et al. Aggressive end-of-life care across gradients of cognitive impairment in nursing home patients with metastatic cancer. J Am Geriatr Soc. 2023;71: 3546–3553. doi:10.1111/jgs.18526	No	Not meeting inclusion criteria	Title and abstract screening
2710	Koroukian SM, Douglas SL, Vu L, Fein HL, Gairola R, Warner DF, et al. Incidence of Aggressive End-of-Life Care Among Older Adults With Metastatic Cancer Living in Nursing Homes and Community Settings. JAMA Netw Open. 2023;6: e230394–e230394. doi:10.1001/jamanetworkopen.2023.0394	No	Not meeting inclusion criteria	Title and abstract screening
2711	Kose J., Duquenne P., Hercberg S., Galan P., Touvier M., Fezeu L.K., et al. Sex-specific associations of different risk behaviors with socio-demographic, health status and lifestyle factors. Eur Psychiatry. 2023;66: S381. doi:10.1192/j.eurpsy.2023.825	No	Not meeting inclusion criteria	Title and abstract screening
2712	Kosterman R, Epstein M, Bailey JA, Furlong M, Hawkins JD. The role of electronic cigarette use for quitting or reducing combustible cigarette use in the 30s: Longitudinal changes and moderated relationships. Drug Alcohol Depend. 2021;227: 108940. doi:10.1016/j.drugalcdep.2021.108940	No	Not meeting inclusion criteria	Title and abstract screening
2713	Kotewar SS, Pakhale A, Tiwari R, Reche A, Singi SR. Electronic Nicotine Delivery System: End to Smoking or Just a New Fancy Cigarette. Cureus. 2023;15: e43425. doi:10.7759/cureus.43425	No	Not meeting inclusion criteria	Title and abstract screening
2714	Kotlyar M, Shanley R, Dufresne SR, Corcoran GA, Hatsukami DK. Effect of restricting menthol flavored cigarettes or E-cigarettes on smoking behavior in menthol smokers. Prev Med. 2022; 107243. doi:10.1016/j.ypmed.2022.107243	No	Not meeting inclusion criteria	Title and abstract screening
2715	Kotlyar M, Shanley R, Dufresne SR, Corcoran GA, Hatsukami DK. Effect on Tobacco Use and Subjective Measures of Including E-cigarettes in a Simulated Ban of Menthol in Combustible Cigarettes. Nicotine Tob Res. 2022;24: 1448–1457. doi:10.1093/ntr/ntac107	No	Not meeting inclusion criteria	Title and abstract screening
2716	Kotoulas S-C, Katsaounou P, Riha R, Grigoriou I, Papakosta D, Spyratos D, et al. Electronic Cigarettes and Asthma: What Do We Know So Far?. J Pers Med. 2021;11. doi:10.3390/jpm11080723	No	Not meeting inclusion criteria	Title and abstract screening
2717	Kotz D, Batra A, Kastaun S. Smoking Cessation Attempts and Common Strategies Employed. Dtsch Arztebl Int. 2020;117: 7–13. doi:10.3238/arztebl.2020.0007	No	Not meeting inclusion criteria	Title and abstract screening
2718	Kotz D., Batra A., Kastaun S. Smoking cessation attempts and common strategies employed: A Germany-wide representative survey conducted in 19 waves from 2016 to 2019 (The DEBRA Study) and analyzed by socioeconomic status. Dtsch Arzteblatt Int. 2020;117: 7–13. doi:10.3238/ARZTEBL.2020.0007	No	Not meeting inclusion criteria	Title and abstract screening
2719	Kourea K., Kostelli G., Ikonomidis I. Mid-term effects of electronic cigarette use on vascular function and oxidative stress. Cardiovasc Res. 2021;116: E82. doi:10.1093/CVR/CVAA095	No	Not meeting inclusion criteria	Title and abstract screening
2720	Kousta S. E-cigarettes for smoking cessation. Nat Hum Behav. 2019;3: 322. doi:10.1038/s41562-019-0575-9	No	Not meeting inclusion criteria	Title and abstract screening
2721	Kovach KA, Peterson R, Bharati R, Istas K, Monroe M. Co-creating opportunities to incorporate cessation for electronic nicotine delivery systems in family medicine - a qualitative program evaluation. BMC Fam Pr. 2021;22: 169. doi:10.1186/s12875-021-01520-x	No	Not meeting inclusion criteria	Title and abstract screening
2722	Kovar L, Selzer D, Britz H, Benowitz N, St Helen G, Kohl Y, et al. Comprehensive Parent-Metabolite PBPK/PD Modeling Insights into Nicotine Replacement Therapy Strategies. Clin Pharmacokinet. 2020;59: 1119–1134. doi:10.1007/s40262-020-00880-4	No	Not meeting inclusion criteria	Title and abstract screening
2723	Kowitt SD, Anshari D, Orlan EN, Kim K, Ranney LM, Goldstein AO, et al. Impact of an e-cigarette tax on cigarette and e-cigarette use in a middle-income country: a study from Indonesia using a pre-post design. BMJ Open. 2022;12: e055483. doi:10.1136/bmjopen-2021-055483	No	Not meeting inclusion criteria	Title and abstract screening
2724	Kowitt SD, Cornacchione Ross J, Goldstein AO, Jarman KL, Thrasher JF, Ranney LM. Youth Exposure to Warnings on Cigar, E-Cigarette, and Waterpipe Tobacco Packages. Am J Prev Med. 2021;61: 80–87. doi:10.1016/j.amepre.2021.01.028	No	Not meeting inclusion criteria	Title and abstract screening
2725	Kowitt SD, Mendel Sheldon J, Vereen RN, Kurtzman RT, Gottfredson NC, Hall MG, et al. The Impact of The Real Cost Vaping and Smoking Ads across Tobacco Products. Nicotine Tob Res. 2023;25: 430–437. doi:10.1093/ntr/ntac206	No	Not meeting inclusion criteria	Title and abstract screening
2726	Kowitt SD, Seidenberg AB, Gottfredson O'Shea NC, Ritchie C, Galper EF, Sutfin EL, et al. Synthetic nicotine descriptors: awareness and impact on perceptions of e-cigarettes among US youth. Tob Control. 2023. doi:10.1136/tc-2023-057928	No	Not meeting inclusion criteria	Title and abstract screening
2727	Kowitt SD, Sheldon JM, Vereen RN, Kurtzman RT, Gottfredson NC, Hall MG, et al. The Impact of The Real Cost Vaping and Smoking Ads across Tobacco Products. Nicotine Tob Res. 2022. doi:10.1093/ntr/ntac206	No	Not meeting inclusion criteria	Title and abstract screening
2728	Koyama S, Tabuchi T, Miyashiro I. E-Cigarettes Use Behaviors in Japan: An Online Survey. Int J Env Res Public Health. 2022;19. doi:10.3390/ijerph19020892	No	Not meeting inclusion criteria	Title and abstract screening
2729	Kozak K, George TP. Pharmacotherapy for smoking cessation in schizophrenia: a systematic review. Expert Opin Pharmacother. 2020;21: 581–590. doi:10.1080/14656566.2020.1721466	No	Not meeting inclusion criteria	Title and abstract screening
2730	Kozlowski LT. Minors, Moral Psychology, and the Harm Reduction Debate: The Case of Tobacco and Nicotine. J Health Polit Policy Law. 2017;42: 1099–1112. doi:10.1215/03616878-4193642	No	Not meeting inclusion criteria	Title and abstract screening
2731	Kozlowski LT, Sweanor DT. Young or adult users of multiple tobacco/nicotine products urgently need to be informed of meaningful differences in product risks. Addict Behav. 2018;76: 376–381. doi:10.1016/j.addbeh.2017.01.026	No	Not meeting inclusion criteria	Title and abstract screening
2732	Kozlowski LT. How I Learned That Cigarettes Were Addictive--1970s to 1980s: A Personal History. J Stud Alcohol Drugs. 2020;81: 731–737.	No	Not meeting inclusion criteria	Title and abstract screening
2733	Kraemer J.D., Strasser A.A., Lindblom E.N., Niaura R.S., Mays D. Crowdsourced data collection for public health: A comparison with nationally representative, population tobacco use data. Prev Med. 2017;102: 93–99. doi:10.1016/j.ypmed.2017.07.006	No	Not meeting inclusion criteria	Title and abstract screening
2734	Kranzler HR, Washio Y, Zindel LR, Wileyto EP, Srinivas S, Hand DJ, et al. Placebo-controlled trial of bupropion for smoking cessation in pregnant women. Am J Obstet Gynecol MFM. 2021;3: 100315. doi:10.1016/j.ajogmf.2021.100315	No	Not meeting inclusion criteria	Title and abstract screening
2735	Kreslake JM, Diaz MC, Shinaba M, Vallone DM, Hair EC. Youth and young adult risk perceptions and behaviours in response to an outbreak of e-cigarette/vaping-associated lung injury (EVALI) in the USA. Tob Control. 2022;31: 88–97. doi:10.1136/tobaccocontrol-2020-056090	No	Not meeting inclusion criteria	Title and abstract screening
2736	Kreslake JM, O'Connor KM, Liu M, Vallone DM, Hair E. A resurgence of e-cigarette use among adolescents and young adults late in the COVID-19 pandemic. PLoS One. 2023;18: e0282894. doi:10.1371/journal.pone.0282894	No	Not meeting inclusion criteria	Title and abstract screening

2737	Kresovich A, Noar SM, Gvino E, Prentice-Dunn H, Ribisl KM. A Review of Web-Based Tobacco Control Media Archives for Researchers and Practitioners. J Cancer Educ. 2022;37: 1454–1459. doi:10.1007/s13187-021-01983-7	No	Not meeting inclusion criteria	Title and abstract screening
2738	Kresovich A, Sanzo N, Brothers W, Prentice-Dunn H, Boynton MH, Sutfin EL, et al. What's in the message? An analysis of themes and features used in vaping prevention messages. Addict Behav Rep. 2022;15: 100404. doi:10.1016/j.abrep.2021.100404	No	Not meeting inclusion criteria	Title and abstract screening
2739	Kresovich A, Sanzo N, Brothers W, Prentice-Dunn H, Boynton MH, Sutfin EL, et al. What's in the message? An analysis of themes and features used in vaping prevention messages. Addict Behav Rep. 2021; No-Specified. doi:10.1016/j.abrep.2021.100404https://dx.doi.org/10.1016/j.abrep.2021.100404	No	Not meeting inclusion criteria	Title and abstract screening
2740	Krishnan N, Abroms LC, Berg CJ. Electronic Nicotine Product Cessation and Cigarette Smoking: Analysis of Waves 3 and 4 From the PATH Study. Nicotine Tob Res. 2022;24: 324–332. doi:10.1093/ntr/ntab155	No	Not meeting inclusion criteria	Title and abstract screening
2741	Krishnan N. Applying social cognitive theory to identify predictors of electronic nicotine product use trajectories and cessation. Diss Abstr Int Sect B Sci Eng. 2022;83: No-Specified.	No	Not meeting inclusion criteria	Title and abstract screening
2742	Krishnan N, Berg CJ, Elmi AF, Klemperer EM, Sherman SE, Abroms LC. Trajectories of ENDS and cigarette use among dual users: analysis of waves 1 to 5 of the PATH Study. Tob Control. 2022. doi:10.1136/tc-2022-057405	No	Not meeting inclusion criteria	Title and abstract screening
2743	Krist AH, Davidson KW, Mangione CM, Barry MJ, Cabana M, Caughey AB, et al. Interventions for Tobacco Smoking Cessation in Adults, Including Pregnant Persons: US Preventive Services Task Force Recommendation Statement. JAMA. 2021;325: 265–279. doi:10.1001/jama.2020.25019	No	Not meeting inclusion criteria	Title and abstract screening
2744	Kristjansson A.L., Mann M.J., Sigfusson J., Sarbu E.A., Grubliauskiene J., Daily S.M., et al. Prevalence of e-cigarette use among adolescents in 13 Eastern European towns and cities. Public Health. 2017;147: 66–68. doi:10.1016/j.puhe.2017.02.005	No	Not meeting inclusion criteria	Title and abstract screening
2745	Kroll JL, Jones M, Chen AB, Yang CC, Carmack CL, Cohen L, et al. End-of-Life Care, Symptom Burden, and Quality of Life in Couples Facing Stage IV Lung Cancer: The Role of Patient and Spousal Psychospirituality and Discussions Around Fear of Death and Disease Progression. J Palliat Med. 2023;26: 690–696. doi:10.1089/jpm.2022.0376	No	Not meeting inclusion criteria	Title and abstract screening
2746	Krueger EA, Hong C, Cunningham NJ, Berteau LK, Cordero L, Wu ESC, et al. Prevalence of Nicotine and Tobacco Product Use by Sexual Identity, Gender Identity, and Sex Assigned at Birth Among Emerging Adult Tobacco Users in California, United States. Nicotine Tob Res Off J Soc Res Nicotine Tob. 2023;25: 1378–1385. doi:10.1093/ntr/ntad048	No	Not meeting inclusion criteria	Title and abstract screening
2747	Kruhlak M, Kirkland SW, Clua MG, Villa-Roel C, Elwi A, O'Neill B, et al. An Assessment of the Management of Patients with Advanced End-Stage Illness in the Emergency Department: An Observational Cohort Study. J Palliat Med. 2021;24: 1840–1848. doi:10.1089/jpm.2021.0004	No	Not meeting inclusion criteria	Title and abstract screening
2748	Krusemann E., Boesveldt S., De Graaf K., Talhout R. An overview of the role of flavors in e-cigarette addiction. Tob Induc Dis. 2018;16: 84. doi:10.18332/tid/84095	No	Not meeting inclusion criteria	Title and abstract screening
2749	Krusemann EJZ. The role of flavors in attractiveness of electronic cigarettes. Diss Abstr Int Sect B Sci Eng. 2022;83: No-Specified.	No	Not meeting inclusion criteria	Title and abstract screening
2750	Kruszewski J, Worobiej D, Kolasinska W, Sokolowski RA, Rzad M. Potential benefits and hazards associated with the use of e-cigarettes - a guide for practitioners and current status in Poland. Adv Respir Med. 2021;89: 392–402. doi:10.5603/ARM.a2021.0058	No	Not meeting inclusion criteria	Title and abstract screening
2751	Krysinski A., Russo C., Campagna D., Di Pino A., John S., Belsey J., et al. A multicenter prospective randomized controlled trial investigating the effects of combustion-free nicotine alternatives on cardiovascular risk factors and metabolic parameters in individuals with type 2 diabetes who smoke: the DiaSmokeFree study protocol. Intern Emerg Med. 2023. doi:10.1007/s11739-023-03467-6	No	Not meeting inclusion criteria	Title and abstract screening
2752	Kucera C, Ramalingam A, Srivastava S, Bhatnagar A, Carll AP. Nicotine Formulation Influences the Autonomic and Arrhythmogenic Effects of Electronic Cigarettes. Nicotine Tob Res Off J Soc Res Nicotine Tob. 2023. doi:10.1093/ntr/ntad237	No	Not meeting inclusion criteria	Title and abstract screening
2753	Kuebler WM. (V)aperçu on the harm of nicotine e-cigarettes. Eur Respir J. 2023;61. doi:10.1183/13993003.00886-2023	No	Not meeting inclusion criteria	Title and abstract screening
2754	Kuk AE, Bluestein MA, Chen B, Harrell M, Spells CE, Atem F, et al. The Effect of Perceptions of Hookah Harmfulness and Addictiveness on the Age of Initiation of Hookah Use among Population Assessment of Tobacco and Health (PATH) Youth. Int J Env Res Public Health. 2022;19. doi:10.3390/ijerph19095034	No	Not meeting inclusion criteria	Title and abstract screening
2755	Kumar M, Subramanian K, Tanwar KS, Prabhahar A, Divyaveer S, Sood A, et al. Radioiodine Therapy in Patient with Differentiated Thyroid Cancer and End-Stage Renal Disease on Maintenance Hemodialysis: Case Report with Review of Literature. J Nucl Med Technol. 2021;49: 1–18. doi:10.2967/jnmt.121.261979	No	Not meeting inclusion criteria	Title and abstract screening
2756	Kumar N, Malik S, Malik S, Sahni V, Joshi S. Role of erector spinae plane block in end-of-life care for a patient with advanced abdominal malignancy. Indian J Pain. 2022;36: 108–110. doi:10.4103/ijpn.ijpn_19_22	No	Not meeting inclusion criteria	Title and abstract screening
2757	Kundu A, Kouzoukas E, Zawertailo L, Fougere C, Dragonetti R, Selby P, et al. Scoping review of guidance on cessation interventions for electronic cigarettes and dual electronic and combustible cigarettes use. CMAJ Open. 2023;11: E336–E344. doi:10.9778/cmajo.20210325	No	Not meeting inclusion criteria	Title and abstract screening
2758	Kurti AN, Bunn JY, Villanti AC, Stanton CA, Redner R, Lopez AA, et al. Patterns of Single and Multiple Tobacco Product Use Among US Women of Reproductive Age. Nicotine Tob Res Off J Soc Res Nicotine Tob. 2018;20: S71–S80. doi:10.1093/ntr/nty024	No	Not meeting inclusion criteria	Title and abstract screening
2759	Kurtzman RT, Vereen RN, Mendel Sheldon J, Adams ET, Hall MG, Brewer NT, et al. Adolescents' Understanding of Smoking and Vaping Risk Language: Cognitive Interviews to Inform Scale Development. Nicotine Tob Res. 2022;24: 1741–1747. doi:10.1093/ntr/ntac127	No	Not meeting inclusion criteria	Title and abstract screening
2760	Kusner LL, Misra RS, Lucas R. Editorial: Global excellence in inflammatory diseases: North America 2021. Front Immunol. 2023;14: 1245827. doi:10.3389/fimmu.2023.1245827	No	Not meeting inclusion criteria	Title and abstract screening
2761	Kusonic D, Bijelic K, Kladar N, Bozin B, Torovic L, Srdenovic Conic B. Comparative Health Risk Assessment of Heated Tobacco Products versus Conventional Cigarettes. Subst Use Misuse. 2023;58: 346–353. doi:10.1080/10826084.2022.2161315	No	Not meeting inclusion criteria	Title and abstract screening
2762	Kwan LY, Eaton DL, Andersen SL, Dow-Edwards D, Levin ED, Talpos J, et al. This is your teen brain on drugs: In search of biological factors unique to dependence toxicity in adolescence. Neurotoxicol Teratol. 2020;81: 106916. doi:10.1016/j.ntt.2020.106916	No	Not meeting inclusion criteria	Title and abstract screening
2763	Kwan ML, Haque R, Young-Wolff KC, Lee VS, Roh JM, Ergas IJ, et al. Smoking Behaviors and Prognosis in Patients With Non-Muscle-Invasive Bladder Cancer in the Be-Well Study. JAMA Netw Open. 2022;5: e2244430. doi:10.1001/jamanetworkopen.2022.44430	No	Not meeting inclusion criteria	Title and abstract screening
2764	Kwon E., Adams Z., Seo D.-C. Trajectories and determinants of adolescents' nicotine product use risk among U.S. adolescents in a nationally representative sample of longitudinal cohort. Addict Behav. 2021;116: 106812. doi:10.1016/j.addbeh.2020.106812	No	Not meeting inclusion criteria	Title and abstract screening
2765	Kwon E, Seo D-C, Lin H-C, Chen Z. Predictors of youth e-cigarette use susceptibility in a U.S. nationally representative sample. Addict Behav. 2018;82: 79–85. doi:10.1016/j.addbeh.2018.02.026	No	Not meeting inclusion criteria	Title and abstract screening

2766	Kwon MY, Cho MS. Association of Residence Type on Smoking in South Korean Adolescents during the COVID-19 Pandemic: Findings from a National Survey. Int J Env Res Public Health. 2022;19. doi:10.3390/ijerph191912886	No	Not meeting inclusion criteria	Title and abstract screening
2767	Kyota A, Kanda K, Senuma M, Tsukagoshi N, Futawatari T, Kondo Y. The perception of life and death in patients with end-of-life stage cancer: A systematic review of qualitative research. Eur J Oncol Nurs. 2023;66: N.PAG-N.PAG. doi:10.1016/j.ejon.2023.102354	No	Not meeting inclusion criteria	Title and abstract screening
2768	Kypriotakis G, Robinson JD, Green CE, Cinciripini PM. Patterns of Tobacco Product Use and Correlates Among Adults in the Population Assessment of Tobacco and Health (PATH) Study: A Latent Class Analysis. Nicotine Tob Res Off J Soc Res Nicotine Tob. 2018;20: S81–S87. doi:10.1093/ntr/nty025	No	Not meeting inclusion criteria	Title and abstract screening
2769	Kyriakos CN, Fong GT, de Abreu Perez C, Szklo AS, Driezen P, Quah ACK, et al. Brazilian smokers are ready for the ban on flavour additives in tobacco to be implemented. Prev Med. 2022;160: 107074. doi:10.1016/j.ypmed.2022.107074	No	Not meeting inclusion criteria	Title and abstract screening
2770	L. MYERS A., NGUYEN D., DOLINA M.Y. A CASE OF NON-RESOLVING PNEUMONIA. Chest. 2023;164: A1220–A1221. doi:10.1016/j.chest.2023.07.871	No	Not meeting inclusion criteria	Title and abstract screening
2771	La Rosa G, Vernooij R, Qureshi M, Polosa R, O'Leary R. Clinical testing of the cardiovascular effects of e-cigarette substitution for smoking: a living systematic review. Intern Emerg Med. 2023. doi:10.1007/s11739-022-03161-z	No	Not meeting inclusion criteria	Title and abstract screening
2772	Labro G, Tubach F, Belin L, Dubost JL, Osman D, Muller G, et al. Nicotine patches in patients on mechanical ventilation for severe COVID-19: a randomized, double-blind, placebo-controlled, multicentre trial. Intensive Care Med. 2022;48: 876–887. doi:10.1007/s00134-022-06721-1	No	Not meeting inclusion criteria	Title and abstract screening
2773	Lachhar G., Mahbub E., Suridis E., Guida C., Mohsin S., Melek A., et al. A Rare Case of Sarcoidosis Involving Multiple Tattoos and Organ Systems in an Elderly White Male. Am J Gastroenterol. 2022;117: S2094–S2095. doi:10.14309/01.ajg.0000869812.05930.67	No	Not meeting inclusion criteria	Title and abstract screening
2774	Laestadius L.I., Wahl M.M., Vassey J., Cho Y.I. Compliance with FDA nicotine warning statement provisions in e-liquid promotion posts on instagram. Nicotine Tob Res. 2021;22: 1823–1830. doi:10.1093/NTR/NTAA092	No	Not meeting inclusion criteria	Title and abstract screening
2775	Laestadius LI, Penndorf KE, Seidl M, Cho YI. Assessing the Appeal of Instagram Electronic Cigarette Refill Liquid Promotions and Warnings Among Young Adults: Mixed Methods Focus Group Study. J Med Internet Res. 2019;21: e15441. doi:10.2196/15441	No	Not meeting inclusion criteria	Title and abstract screening
2776	Laestadius LI, Wahl MM, Vassey J, Cho YI. Compliance With FDA Nicotine Warning Statement Provisions in E-liquid Promotion Posts on Instagram. Nicotine Tob Res Off J Soc Res Nicotine Tob. 2020;22: 1823–1830. doi:10.1093/ntr/ntaa092	No	Not meeting inclusion criteria	Title and abstract screening
2777	Lai L, Qiu H. Biological Toxicity of the Compositions in Electronic-Cigarette on Cardiovascular System. J Cardiovasc Transl Res. 2021;14: 371–376. doi:10.1007/s12265-020-10060-1	No	Not meeting inclusion criteria	Title and abstract screening
2778	Lai Y, Li H, Tian L, Ye X, Hu Y. Baseball bat-like gastric tube for end-to-side oesophageal-gastric anastomosis decreased risks of anastomotic leakage after oesophagectomy for oesophageal cancer: A retrospective propensity score matched comparative study with 613 patients. Int J Surg. 2022;98: N.PAG-N.PAG. doi:10.1016/j.ijsu.2022.106227	No	Not meeting inclusion criteria	Title and abstract screening
2779	Lal P, Kapoor R, Singh RJ. Compliance to Online Retail of Electronic Nicotine Delivery Systems (ENDS) in India before and after the 2018 Ban Order of the Government of India. Asian Pac J Cancer Prev. 2021;22: 13–17. doi:10.31557/APJCP.2021.22.S2.13	No	Not meeting inclusion criteria	Title and abstract screening
2780	Lallai V, Chen YC, Roybal MM, Kotha ER, Fowler JP, Staben A, et al. Nicotine e-cigarette vapor inhalation and self-administration in a rodent model: Sex- and nicotine delivery-specific effects on metabolism and behavior. Addict Biol. 2021;26: e13024. doi:10.1111/adb.13024	No	Not meeting inclusion criteria	Title and abstract screening
2781	Lam G.Y., Befus A.D., Damant R.W., Ferrara G., Fuhr D.P., Stickland M.K., et al. Exertional intolerance and dyspnea with preserved lung function: an emerging long COVID phenotype? Respir Res. 2021;22: 222. doi:10.1186/s12931-021-01814-9	No	Not meeting inclusion criteria	Title and abstract screening
2782	Lam K.-K.-W., Ho K.-Y., Wu C.-S.-T., Tong M.-N., Tang L.-N., Mak Y.-W. Exploring Factors Contributing to the Smoking Behaviour among Hong Kong Chinese Young Smokers during COVID-19 Pandemic: A Qualitative Study. Int J Environ Res Public Health. 2022;19: 4145. doi:10.3390/ijerph19074145	No	Not meeting inclusion criteria	Title and abstract screening
2783	Lam MB, Riley KE, Zheng J, Orav EJ, Jha AK, Burke LG. Healthy days at home: A population-based quality measure for cancer patients at the end of life. Cancer 0008543X. 2021;127: 4249–4257. doi:10.1002/cncr.33817	No	Not meeting inclusion criteria	Title and abstract screening
2784	Lam T-C, Chan S-K, Choi C-W, Tsang K-C, Yuen K-K, Soong I, et al. Integrative Palliative Care Service Model Improved End-of-Life Care and Overall Survival of Advanced Cancer Patients in Hong Kong: A Review of Ten-Year Territory-Wide Cohort. J Palliat Med. 2021;24: 1314–1320. doi:10.1089/jpm.2020.0640	No	Not meeting inclusion criteria	Title and abstract screening
2785	Lam TK, Samuels TL, Yan K, Zhang L, Adams J, Stabenau KA, et al. Association of e-Cigarette Exposure with Pediatric Otitis Media Recurrence. Ann Otol Rhinol Laryngol. 2023;132: 1018–1025. doi:10.1177/00034894221129013	No	Not meeting inclusion criteria	Title and abstract screening
2786	Lambart L, Nollen NL, Mayo MS, Funk O, Leavens E, Cruvinel E, et al. The impact of blunt use on smoking abstinence among Black adults: Secondary analysis from randomized controlled smoking cessation clinical trial. Addict Behav. 2024;148: 107877. doi:10.1016/j.addbeh.2023.107877	No	Not meeting inclusion criteria	Title and abstract screening
2787	Lancellotti P., De Pauw M., Claeys M. Cardio-oncology: where do we stand for in Belgium? Acta Cardiol. 2021;76: 204–208. doi:10.1080/00015385.2020.1713521	No	Not meeting inclusion criteria	Title and abstract screening
2788	Land S. ES24.02 The Rationale for FDA (and EMA) Regulatory Review of Tobacco Use in Clinical Trials. J Thorac Oncol. 2021;16: S90–S91. doi:10.1016/j.jtho.2021.01.052	No	Not meeting inclusion criteria	Title and abstract screening
2789	Landry RL, Groom AL, Vu T-HT, Stokes AC, Berry KM, Kesh A, et al. The role of flavors in vaping initiation and satisfaction among U.S. adults. Addict Behav. 2019;99: 106077. doi:10.1016/j.addbeh.2019.106077	No	Not meeting inclusion criteria	Title and abstract screening
2790	Lang AE. E-Cigarettes Could Replace Smoking as a Risk Factor for Oropharyngeal Cancer. JAMA Otolaryngol Head Neck Surg. 2022;148: 384–385. doi:10.1001/jamaoto.2021.4544	No	Not meeting inclusion criteria	Title and abstract screening
2791	Lang AE. The Effect of COVID-19 on Smoking and Vaping Rates. Ann Am Thorac Soc. 2022;19: 1440–1441. doi:10.1513/AnnalsATS.202202-119LE	No	Not meeting inclusion criteria	Title and abstract screening
2792	Lang AE, Yakhkind A. Implementing a Nicotine-Free Policy in the United States Military. Chest. 2022;161: 845–852. doi:10.1016/j.chest.2021.09.020	No	Not meeting inclusion criteria	Title and abstract screening
2793	Langlois S, Zern A, Anderson S, Ashekun O, Ellis S, Graves J, et al. Subjective social status, objective social status, and substance use among individuals with serious mental illnesses. Psychiatry Res. 2020;293: 113352. doi:10.1016/j.psychres.2020.113352	No	Not meeting inclusion criteria	Title and abstract screening
2794	Lanphier E, Bessias S. Stepping Up or Stepping Back: FDA Roles in Producing and Shaping Knowledge of Pediatric Covid-19 Vaccines. Am J Bioeth. 2022;22: 26–28. doi:10.1080/15265161.2022.2110970	No	Not meeting inclusion criteria	Title and abstract screening

2795	Lanspa MJ, Blagev DP, Callahan SJ. Use of e-Cigarettes for Smoking Cessation. JAMA. 2021;325: 1006. doi:10.1001/jama.2020.27207	No	Not meeting inclusion criteria	Title and abstract screening
2796	LANTIGUA TATEM L.U.I.S., RICHARDSON C.C., NGOC VU-TICAR N.H.I., CHILES J.W. PULMONARY MUCORMYCOSIS IN A PATIENT WITH SICKLE CELL CRISIS AND ACUTE CHEST SYNDROME. Chest. 2023;164: A996–A997. doi:10.1016/j.chest.2023.07.734	No	Not meeting inclusion criteria	Title and abstract screening
2797	Lantini R, Sillice MA, Fava JL, Jennings E, Rosen RK, Horowitz SM, et al. Butt Why? Exploring factors associated with cigarette scavenging behaviors among adult smokers enrolling in a clinical trial for smoking cessation. Addict Behav. 2018;78: 200–204. doi:10.1016/j.addbeh.2017.11.037	No	Not meeting inclusion criteria	Title and abstract screening
2798	Lanza HI, Bello MS, Cho J, Barrington-Trimis JL, McConnell R, Braymiller JL, et al. Tobacco and cannabis poly-substance and poly-product use trajectories across adolescence and young adulthood. Prev Med. 2021;148: 106545. doi:10.1016/j.ypmed.2021.106545	No	Not meeting inclusion criteria	Title and abstract screening
2799	Laranjeira C, Querido A. A reflection on: Bovero et al. (2021) “Hope in end-of-life cancer patients: A cross-sectional analysis”. Palliat Support Care. 2021;19: 776–777. doi:10.1017/S1478951521000705	No	Not meeting inclusion criteria	Title and abstract screening
2800	Larcombe A., Chivers E., Berry L., Huxley R., Musk B., Franklin P., et al. E-cigarettes result in partial amelioration of cigarette-smoke-induced disease in mice. Respirology. 2021;26: 36. doi:10.1111/resp.14021	No	Not meeting inclusion criteria	Title and abstract screening
2801	Laronne A, Granek L, Wiener L, Feder-Bubis P, Golan H. “Some things are even worse than telling a child he is going to die”: Pediatric oncology healthcare professionals perspectives on communicating with children about cancer and end of life. Pediatr Blood Cancer. 2022;69: 1–9. doi:10.1002/pbc.29533	No	Not meeting inclusion criteria	Title and abstract screening
2802	Larue F, Tasbih T, Ribeiro PAB, Lavoie KL, Dolan E, Bacon SL. Immediate physiological effects of acute electronic cigarette use in humans: A systematic review and meta-analysis. Respir Med. 2021;190: 106684. doi:10.1016/j.rmed.2021.106684	No	Not meeting inclusion criteria	Title and abstract screening
2803	Lau YK, Okawa S, Meza R, Katanoda K, Tabuchi T. Nicotine dependence of cigarette and heated tobacco users in Japan, 2019: a cross-sectional analysis of the JASTIS Study. Tob Control. 2022;31: e50–e56. doi:10.1136/tobaccocontrol-2020-056237	No	Not meeting inclusion criteria	Title and abstract screening
2804	Lavallée PC, Charles H, Albers GW, Caplan LR, Donnan GA, Ferro JM, et al. Effect of atherosclerosis on 5-year risk of major vascular events in patients with transient ischaemic attack or minor ischaemic stroke: an international prospective cohort study. Lancet Neurol. 2023;22: 320–329. doi:10.1016/S1474-4422(23)00067-4	No	Not meeting inclusion criteria	Title and abstract screening
2805	Laviolette SR. Molecular and neuronal mechanisms underlying the effects of adolescent nicotine exposure on anxiety and mood disorders. Neuropharmacology. 2021;184: 108411. doi:10.1016/j.neuropharm.2020.108411	No	Not meeting inclusion criteria	Title and abstract screening
2806	Layman HM, Thorisdottir IE, Halldorsdottir T, Sigfusdottir ID, Allegrante JP, Kristjansson AL. Substance Use Among Youth During the COVID-19 Pandemic: a Systematic Review. Curr Psychiatry Rep. 2022;24: 307–324. doi:10.1007/s11920-022-01338-z	No	Not meeting inclusion criteria	Title and abstract screening
2807	Lazard AJ, Ebrahimi Kalan M, Nicolla S, Hall MG, Ribisl KM, Sheldon JM, et al. Optimising messages and images for e-cigarette warnings. Tob Control. 2023. doi:10.1136/tc-2022-057859	No	Not meeting inclusion criteria	Title and abstract screening
2808	Lazard AJ, Nicolla S, Darida A, Hall MG. Negative Perceptions of Young People Using E-Cigarettes on Instagram: An Experiment With Adolescents. Nicotine Tob Res. 2021;23: 1962–1966. doi:10.1093/ntr/ntab099	No	Not meeting inclusion criteria	Title and abstract screening
2809	Le Boisselier R., Pornet C., Chretien B., Tournebize J., Batisse A., Martinez M., et al. Vaping drugs in the Northwest (Britany and Normandy areas) of France: Lessons from AddictoVigilance data. Fundam Clin Pharmacol. 2021;35: 62. doi:10.1111/fcp.12671	No	Not meeting inclusion criteria	Title and abstract screening
2810	Le Bozec A., Guedon M., Laurent M., Brugel M., Carlier C., Perrier M., et al. PREVALENCE OF CANNABIDIOL (CBD) CONSUMPTION AND CANCER PATIENTS’ EXPECTATIONS IN ONE ONCOLOGY DAY-HOSPITAL: A CROSS-SECTIONAL STUDY. Eur J Oncol Pharm. 2023;6: 17. doi:10.1097/OP9.0000000000000042	No	Not meeting inclusion criteria	Title and abstract screening
2811	Le D, Ciceron AC, Romm KF, Clausen ME, Abroms LC, Evans WD, et al. E-cigarette cessation interest and quit attempts among young adults reporting exclusive e-cigarette use or dual use with other tobacco products: How can we reach them? Tob Prev Cessat. 2023;9: 33. doi:10.18332/tpc/172416	No	Not meeting inclusion criteria	Title and abstract screening
2812	Le Denmat V, Dewitte JD. [Tobacco and work]. Rev Mal Respir. 2019;36: 1117–1128. doi:10.1016/j.rmr.2019.01.010	No	Not meeting inclusion criteria	Title and abstract screening
2813	Le Foll B., Piper M.E., Fowler C.D., Tonstad S., Bierut L., Lu L., et al. Tobacco and nicotine use. Nat Rev Dis Primer. 2022;8: 19. doi:10.1038/s41572-022-00346-w	No	Not meeting inclusion criteria	Title and abstract screening
2814	Le Grande M, Borland R, Yong HH, Cummings KM, McNeill A, Thompson ME, et al. Predictive Power of Dependence Measures for Quitting Smoking. Findings From the 2016 to 2018 ITC Four Country Smoking and Vaping Surveys. Nicotine Tob Res. 2021;23: 276–285. doi:10.1093/ntr/ntaa108	No	Not meeting inclusion criteria	Title and abstract screening
2815	Le Grande M, Borland R, Yong HH, McNeill A, Fong G, Cummings KM. Age-Related Interactions on Key Theoretical Determinants of Smoking Cessation: Findings from the ITC Four Country Smoking and Vaping Surveys (2016-2020). Nicotine Tob Res. 2022;24: 679–689. doi:10.1093/ntr/ntab230	No	Not meeting inclusion criteria	Title and abstract screening
2816	Le TTT, Issabakhsh M, Li Y, María Sánchez-Romero L, Tan J, Meza R, et al. Are the Relevant Risk Factors Being Adequately Captured in Empirical Studies of Smoking Initiation? A Machine Learning Analysis Based on the Population Assessment of Tobacco and Health Study. Nicotine Tob Res. 2023;25: 1481–1488. doi:10.1093/ntr/ntad066	No	Not meeting inclusion criteria	Title and abstract screening
2817	Leas EC, Moy NH, Nobles AL, Ayers J, Zhu SH, Purushothaman V. Google shopping queries for vaping products, JUUL and IQOS during the E-cigarette, or Vaping, product use Associated Lung Injury (EVALI) outbreak. Tob Control. 2022;31: e74–e77. doi:10.1136/tobaccocontrol-2021-056481	No	Not meeting inclusion criteria	Title and abstract screening
2818	Leas EC, Nobles AL, Caputi TL, Dredze M, Zhu SH, Cohen JE, et al. News coverage of the E-cigarette, or Vaping, product use Associated Lung Injury (EVALI) outbreak and internet searches for vaping cessation. Tob Control. 2021;30: 578–582. doi:10.1136/tobaccocontrol-2020-055755	No	Not meeting inclusion criteria	Title and abstract screening
2819	Leatherdale ST, Bélanger RE, Gansaonré RJ, Cole AG, Haddad S. Youth Vaping During the Early Stages of the COVID-19 Pandemic Period: Adjusted Annual Changes in Vaping Between the Pre-COVID and Initial COVID-Lockdown Waves of the COMPASS Study. Nicotine Tob Res. 2023;25: 193–202. doi:10.1093/ntr/ntac083	No	Not meeting inclusion criteria	Title and abstract screening
2820	Leavens ELS, Carpenter MJ, Smith TT, Nollen NL. Exploratory evaluation of online brief education for JUUL pod-mod use and prevention. Addict Behav. 2021;119: 106942. doi:10.1016/j.addbeh.2021.106942	No	Not meeting inclusion criteria	Title and abstract screening
2821	Leavens ELS, Freedy JR. Youth e-cigarette use: Assessing for, and halting, the hidden habit. J Fam Pr. 2021;70: 342–346. doi:10.12788/jfp.0254	No	Not meeting inclusion criteria	Title and abstract screening
2822	Leavens ELS, Meier E, Brett EI, Stevens EM, Tackett AP, Villanti AC, et al. Polytobacco use and risk perceptions among young adults: The potential role of habituation to risk. Addict Behav. 2019;90: 278–284. doi:10.1016/j.addbeh.2018.11.003	No	Not meeting inclusion criteria	Title and abstract screening
2823	Leavens ELS, Nollen NL, Ahluwalia JS, Mayo MS, Rice M, Brett EI, et al. Changes in dependence, withdrawal, and craving among adult smokers who switch to nicotine salt pod-based e-cigarettes. Addiction. 2022;117: 207–215. doi:10.1111/add.15597	No	Not meeting inclusion criteria	Title and abstract screening

2824	Leavens ELS, Roberts J, Faseru B, Thompson M, Denes-Collar K, Shah H. Development and implementation of the ECHO model in a school setting to address youth electronic cigarette use in Kansas: A protocol. Front Public Health. 2022;10: 1057600. doi:10.3389/fpubh.2022.1057600	No	Not meeting inclusion criteria	Title and abstract screening
2825	Leavens ELS, Stevens EM, Brett EI, Leffingwell TR, Wagener TL. JUUL in school: JUUL electronic cigarette use patterns, reasons for use, and social normative perceptions among college student ever users. Addict Behav. 2019;99: 106047. doi:10.1016/j.addbeh.2019.106047	No	Not meeting inclusion criteria	Title and abstract screening
2826	LeBouf RF, Ranpara A, Ham J, Aldridge M, Fernandez E, Williams K, et al. Chemical Emissions From Heated Vitamin E Acetate-Insights to Respiratory Risks From Electronic Cigarette Liquid Oil Diluents Used in the Aerosolization of Δ(9)-THC-Containing Products. Front Public Health. 2021;9: 765168. doi:10.3389/fpubh.2021.765168	No	Not meeting inclusion criteria	Title and abstract screening
2827	Lechasseur A., Godbout K., Fortin M., Clavel M.-A., Maltais F., Bosse Y., et al. VapALERT study: A prospective, exploratory and adaptative research cohort to investigate the symptomatic and asymptomatic, pulmonary and cardiovascular effects of vaping. Can J Respir Crit Care Sleep Med. 2023;7: 10. doi:10.1080/24745332.2023.2214070	No	Not meeting inclusion criteria	Title and abstract screening
2828	Lechner W.V., Murphy C.M., Colby S.M., Janssen T., Rogers M.L., Jackson K.M. Cognitive risk factors of electronic and combustible cigarette use in adolescents. Addict Behav. 2018;82: 182–188. doi:10.1016/j.addbeh.2018.03.006	No	Not meeting inclusion criteria	Title and abstract screening
2829	Lechner WV, Sidhu NK, Cioe PA, Kahler CW. Effects of time-varying changes in tobacco and alcohol use on depressive symptoms following pharmaco-behavioral treatment for smoking and heavy drinking. Drug Alcohol Depend. 2019;194: 173–177. doi:10.1016/j.drugalcdep.2018.09.030	No	Not meeting inclusion criteria	Title and abstract screening
2830	Lechner W, Meier E, Wiener J, Grant D, Gilmore J, Judah M, et al. The comparative efficacy of first- versus second-generation electronic cigarettes in reducing symptoms of nicotine withdrawal. 2015;110: 862-867. doi:10.1111/add.12870	No	Not meeting inclusion criteria	Title and abstract screening
2831	Lee A., Chang A.A., Lyu J.C., Ling P.M., Hsia S.L. Characterizing Participant Perceptions about Smoking-Cessation Pharmacotherapy and E-Cigarettes from Facebook Smoking-Cessation Support Groups. Int J Environ Res Public Health. 2022;19: 7314. doi:10.3390/ijerph19127314	No	Not meeting inclusion criteria	Title and abstract screening
2832	Lee A, DeGroote NP, Brock KE. Early Versus Late Outpatient Pediatric Palliative Care Consultation and Its Association With End-of-Life Outcomes in Children With Cancer. J Palliat Med. 2023;26: 1466–1473. doi:10.1089/jpm.2023.0063	No	Not meeting inclusion criteria	Title and abstract screening
2833	Lee ARS, Rodriquez EJ, Gallo LC, Giachello AL, Isasi CR, Perreira KM, et al. Acculturation level and change in cigarette consumption behaviors among diverse Hispanics/Latinos: the Hispanic Community Health Study/Study of Latinos. Ann Epidemiol. 2023;84: 33–40. doi:10.1016/j.annepidem.2023.05.005	No	Not meeting inclusion criteria	Title and abstract screening
2834	Lee DN, Liu J, Keller-Hamilton B, Patterson JG, Wedel AV, Vázquez-Otero C, et al. Associations between perceived source credibility, e-cigarettes, and e-cigarette ad perceptions. Prev Med Rep. 2022;28: 101862. doi:10.1016/j.pmedr.2022.101862	No	Not meeting inclusion criteria	Title and abstract screening
2835	Lee DN, Stevens EM, Keller-Hamilton B, Wedel AV, Wagener TL, Patterson JG. Minoritized Sexual Identity and Perceived Effectiveness of Instagram Public Health Messaging about E-cigarettes. J Health Commun. 2022;27: 115–124. doi:10.1080/10810730.2022.2059724	No	Not meeting inclusion criteria	Title and abstract screening
2836	Lee H, Weerakoon SM, Harrell MB, Messiah SE, Rao DR. Neighborhood Characteristics and the Burden of E-Cigarette, or Vaping, Product Use-Associated Lung Injury: An Ecological Comparison Study. Pediatr Allergy Immunol Pulmonol. 2023;36: 16–22. doi:10.1089/ped.2022.0133	No	Not meeting inclusion criteria	Title and abstract screening
2837	Lee H-Y. E-cigarette adoption and use intention among college students: Determinants and warning label effects. Diss Abstr Int Sect B Sci Eng. 2017;77: No-Specified.	No	Not meeting inclusion criteria	Title and abstract screening
2838	Lee J, Olayinka O, Thrul J. Association between pain and e-cigarette use stratified by cigarette smoking status: Results from National Health Interview Survey (NHIS) 2019-2020. Addict Behav. 2023;140: 107625. doi:10.1016/j.addbeh.2023.107625	No	Not meeting inclusion criteria	Title and abstract screening
2839	Lee J, Ouellette RR, Murthy D, Kong G. Content Analysis of YouTube Videos Related to E-Cigarettes and COVID-19. Subst Use Misuse. 2024;59: 143–149. doi:10.1080/10826084.2023.2262027	No	Not meeting inclusion criteria	Title and abstract screening
2840	Lee J., Striley C.W., Custodero C., Rocha H.A., Salloum R.G. Association of pleasant sensations at cigarette smoking initiation with subsequent tobacco product use among U.S. adolescents. Addict Behav. 2019;89: 151–155. doi:10.1016/j.addbeh.2018.10.003	No	Not meeting inclusion criteria	Title and abstract screening
2841	Lee J, Yun J-S, Ko S-H. Advanced Glycation End Products and Their Effect on Vascular Complications in Type 2 Diabetes Mellitus. Nutrients. 2022;14: 3086–3086. doi:10.3390/nu14153086	No	Not meeting inclusion criteria	Title and abstract screening
2842	Lee JGL, Soule EK. Evidence of potential tobacco industry influence in Tobacco Regulatory Science. Tob Control. 2023;32: 400–401. doi:10.1136/tobaccocontrol-2021-056752	No	Not meeting inclusion criteria	Title and abstract screening
2843	Lee J, Cheong J, Markham MJ, Lam J, Warren GW, Salloum RG. Negative affect and the utilization of tobacco treatment among adult smokers with cancer. Psychooncology. 2021;30: 93–102. doi:10.1002/pon.5543	No	Not meeting inclusion criteria	Title and abstract screening
2844	Lee J, Turner K, Xie Z, Kadhim B, Hong Y-R. Association Between Health Information-Seeking Behavior on YouTube and Physical Activity Among U.S. Adults: Results From Health Information Trends Survey 2020. AJPM Focus. 2022;1: 100035. doi:10.1016/j.focus.2022.100035	No	Not meeting inclusion criteria	Title and abstract screening
2845	Lee J. Nicotine Dependence of Electronic Cigarette, Dual Combustible and Electronic Cigarette Users. Korean J Fam Med. 2021;42: 189–190. doi:10.4082/kjfm.42.3E	No	Not meeting inclusion criteria	Title and abstract screening
2846	Lee JX, Phipatanakul W, Gaffin JM. Environment and the development of severe asthma in inner city population. Curr Opin Allergy Clin Immunol. 2023;23: 179–184. doi:10.1097/ACI.0000000000000890	No	Not meeting inclusion criteria	Title and abstract screening
2847	Lee MH, Cool CD, Maloney JP. Histopathological Correlation of Acute on Chronic Eosinophilic Pneumonitis Caused by Vaporized Cannabis Oil Inhalation. Chest. 2021;159: e137–e139. doi:10.1016/j.chest.2020.10.016	No	Not meeting inclusion criteria	Title and abstract screening
2848	Lee P, Fry J. Investigating gateway effects using the PATH study. F1000Res. 2019;8: 264. doi:10.12688/f1000research.18354.2	No	Not meeting inclusion criteria	Title and abstract screening
2849	Lee P. Vaping nic-sickness and siadh. J Gen Intern Med. 2021;36: S369–S370. doi:10.1007/s11606-021-06830-5	No	Not meeting inclusion criteria	Title and abstract screening
2850	Lee P.N., Fry J.S., Gilliland S., Campbell P., Joyce A.R. Estimating the reduction in US mortality if cigarettes were largely replaced by e-cigarettes. Arch Toxicol. 2022;96: 167–176. doi:10.1007/s00204-021-03180-3	No	Not meeting inclusion criteria	Title and abstract screening
2851	Lee PN, Abrams D, Bachand A, Baker G, Black R, Camacho O, et al. Estimating the Population Health Impact of Recently Introduced Modified Risk Tobacco Products: A Comparison of Different Approaches. Nicotine Tob Res. 2021;23: 426–437. doi:10.1093/ntr/ntaa102	No	Not meeting inclusion criteria	Title and abstract screening
2852	Lee PN, Coombs KJ, Afolalu EF. Considerations related to vaping as a possible gateway into cigarette smoking: an analytical review. F1000Res. 2018;7: 1915. doi:10.12688/f1000research.16928.3	No	Not meeting inclusion criteria	Title and abstract screening

2853	Lee R., Vokos C., Kaur M., Austin M. E-cigarette or vaping use associated lung injury (EVALI)-newcopd of the young? Pediatrics. 2021;147: 624–625. doi:10.1542/peds.147.3-MeetingAbstract.625	No	Not meeting inclusion criteria	Title and abstract screening
2854	Lee S, Choi M, Kim D, Shin J, Kim J. Did the COVID-19 Lockdown Reduce Smoking Rate in Adolescents? Int J Env Res Public Health. 2022;20. doi:10.3390/ijerph20010139	No	Not meeting inclusion criteria	Title and abstract screening
2855	Lee S.-H., Ahn S.-H., Cheong Y.-S. Effect of electronic cigarettes on smoking reduction and cessation in Korean Male smokers: A randomized controlled study. J Am Board Fam Med. 2019;32: 567–574. doi:10.3122/jabfm.2019.04.180384	No	Not meeting inclusion criteria	Title and abstract screening
2856	Lee S.H., Han D.-H., Seo D.-C. Toward a better understanding of adult dual use of cigarettes and e-cigarettes based on use intensity and reasons for dual use. Addict Behav. 2023;137: 107517. doi:10.1016/j.addbeh.2022.107517	No	Not meeting inclusion criteria	Title and abstract screening
2857	Lee S.H., Kim S.H. Association of dual use of cigarettes with obstructive sleep apnea assessed by the STOP-Bang score. Tob Induc Dis. 2023;21. doi:10.18332/tid/169727	No	Not meeting inclusion criteria	Title and abstract screening
2858	Lee SC, Maglalang DD, Avila JC, Leavens ELS, Nollen NL, Pulvers K, et al. Change in E-cigarette risk perception and smoking behavior of Black and Latinx individuals who smoke. Drug Alcohol Depend. 2023;245: 109824. doi:10.1016/j.drugalcdep.2023.109824	No	Not meeting inclusion criteria	Title and abstract screening
2859	Lee SJ, Rees VW, Yossefy N, Emmons KM, Tan ASL. Youth and Young Adult Use of Pod-Based Electronic Cigarettes From 2015 to 2019: A Systematic Review. JAMA Pediatr. 2020;174: 714–720. doi:10.1001/jamapediatrics.2020.0259	No	Not meeting inclusion criteria	Title and abstract screening
2860	Lee SA, Sayad E, Yenduri NJS, Wang KY, Guillerman RP, Farber HJ. Improvement in Pulmonary Function Following Discontinuation of Vaping or E-Cigarette Use in Adolescents with EVALI. Pediatr Allergy Immunol Pulmonol. 2021;34: 23–29. doi:10.1089/ped.2020.1270	No	Not meeting inclusion criteria	Title and abstract screening
2861	Lee TH, Kim W. Differences in electronic cigarette use among adolescents in Korea: A nationwide analysis. J Subst Abuse Treat. 2021;131: 108554. doi:10.1016/j.jsat.2021.108554	No	Not meeting inclusion criteria	Title and abstract screening
2862	Lees CS, Seow H, Chan KK, Gayowsky A, Sinnarajah A. Quality of End-of-Life Care in Gastrointestinal Cancers: A 13-Year Population-Based Retrospective Analysis in Ontario, Canada. Curr Oncol. 2022;29: 9163–9171. doi:10.3390/curroncol29120717	No	Not meeting inclusion criteria	Title and abstract screening
2863	Lefever T.W., Thomas B.F., Kovach A.L., Snyder R.W., Wiley J.L. Route of administration effects on nicotine discrimination in female and male mice. Drug Alcohol Depend. 2019;204: 107504. doi:10.1016/j.drugalcdep.2019.06.007	No	Not meeting inclusion criteria	Title and abstract screening
2864	Lehmann K., Kuhn S., Reimer J. Electronic Cigarettes in Germany: Patterns of Use and Perceived Health Improvement. Eur Addict Res. 2017;23: 136–147. doi:10.1159/000475986	No	Not meeting inclusion criteria	Title and abstract screening
2865	Lehrer S, Rheinstein PH. Marijuana smoking and cataract. J Fr Ophtalmol. 2022;45: 267–271. doi:10.1016/j.jfo.2021.12.008	No	Not meeting inclusion criteria	Title and abstract screening
2866	LeLaurin JH, Dallery J, Silver NL, Markham MJ, Theis RP, Chetram DK, et al. An Implementation Trial to Improve Tobacco Treatment for Cancer Patients: Patient Preferences, Treatment Acceptability and Effectiveness. Int J Env Res Public Health. 2020;17. doi:10.3390/ijerph17072280	No	Not meeting inclusion criteria	Title and abstract screening
2867	Leng A, Maitland E, Wang S, Nicholas S, Lan K, Wang J. Preferences for End-of-Life Care Among Patients With Terminal Cancer in China. JAMA Netw Open. 2022;5: e228788–e228788. doi:10.1001/jamanetworkopen.2022.8788	No	Not meeting inclusion criteria	Title and abstract screening
2868	León X, Pujals G, Bulboa C, García J, López M, Quer M. Head and neck squamous cell carcinoma in cigar smokers. Distinctive epidemiological and prognostic characteristics. Acta Otorrinolaringol Esp Engl Ed. 2021;72: 222–229. doi:10.1016/j.otoeng.2020.05.005	No	Not meeting inclusion criteria	Title and abstract screening
2869	Leone FT, Carlsen K-H, Chooljian D, Crotty Alexander LE, Detterbeck FC, Eakin MN, et al. Recommendations for the Appropriate Structure, Communication, and Investigation of Tobacco Harm Reduction Claims. An Official American Thoracic Society Policy Statement. Am J Respir Crit Care Med. 2018;198: e90–e105. doi:10.1164/rccm.201808-1443ST	No	Not meeting inclusion criteria	Title and abstract screening
2870	Leone FT, Evers-Casey S. Tobacco Use Disorder. Med Clin North Am. 2022;106: 99–112. doi:10.1016/j.mcna.2021.08.011	No	Not meeting inclusion criteria	Title and abstract screening
2871	Leone FT, Zhang Y, Evers-Casey S, Evins AE, Eakin MN, Fathi J, et al. Initiating Pharmacologic Treatment in Tobacco-Dependent Adults. An Official American Thoracic Society Clinical Practice Guideline. Am J Respir Crit Care Med. 2020;202: e5–e31. doi:10.1164/rccm.202005-1982ST	No	Not meeting inclusion criteria	Title and abstract screening
2872	Leroy A., Carton L., Gomajee H., Bordet R., Cottencin O. Naltrexone in the treatment of binge eating disorder in a patient with severe alcohol use disorder: a case report. Am J Drug Alcohol Abuse. 2017;43: 618–620. doi:10.1080/00952990.2017.1298117	No	Not meeting inclusion criteria	Title and abstract screening
2873	Leroy V. [Not Available]. Rev Med Suisse. 2023;19: 539. doi:10.53738/REVMED.2023.19.818.539	No	Not meeting inclusion criteria	Title and abstract screening
2874	LESSING A., GUPTA A.N.U.J., PAROYA A., GADA K.D., AHMAD S. A MAN IN HIS 50s WITH HYPOXIC RESPIRATORY FAILURE AND PNEUMONIA: AN ULTRASOUND CASE STUDY. Chest. 2023;164: A4077. doi:10.1016/j.chest.2023.07.2658	No	Not meeting inclusion criteria	Title and abstract screening
2875	Lester H.A., Dougherty D.A. Nicotine Bound to Its Receptors: New Structures for a Vexing Pathopharmacological Problem. Neuron. 2019;104: 431–432. doi:10.1016/j.neuron.2019.10.029	No	Not meeting inclusion criteria	Title and abstract screening
2876	Leung W, Guo C-G, Tsui V, Mak L-Y, Cheung K, Hui C, et al. NARROW BAND OR BLUE LIGHT IMAGING FOR DETECTION OF PROXIMAL COLONIC LESION: a PROSPECTIVE RANDOMIZED TANDEM COLONOSCOPY STUDY. 2021;160: S-137-S-138. doi:10.1016/S0016-5085(21)01081-7	No	Not meeting inclusion criteria	Title and abstract screening
2877	Leventhal A.M., Mason T.B., Kirkpatrick M.G., Anderson M.K., Levine M.D. E-cigarette device power moderates the effects of non-tobacco flavors and nicotine on product appeal in young adults. Addict Behav. 2020;107: 106403. doi:10.1016/j.addbeh.2020.106403	No	Not meeting inclusion criteria	Title and abstract screening
2878	Leventhal A, Cho J, Barrington-Trimis J, Pang R, Schiff S, Kirkpatrick M. Sensory attributes of e-cigarette flavours and nicotine as mediators of interproduct differences in appeal among young adults. Tob Control. 2020;29: 679–686. doi:10.1136/tobaccocontrol-2019-055172	No	Not meeting inclusion criteria	Title and abstract screening
2879	Leventhal AM, Dai H. Prevalence of Flavored e-Cigarette Use Among Subpopulations of Adults in the United States. J Natl Cancer Inst. 2021;113: 418–424. doi:10.1093/jnci/djaa118	No	Not meeting inclusion criteria	Title and abstract screening
2880	Leventhal AM, Cho J, Vogel EA, Tackett AP, Harlow AF, Wong M, et al. Differences in intention to use flavored oral nicotine products among young adult e-cigarette users and non-users. Prev Med Rep. 2022;30: 102027. doi:10.1016/j.pmedr.2022.102027	No	Not meeting inclusion criteria	Title and abstract screening
2881	Leventhal AM, Dai H, Higgins ST. Smoking Cessation Prevalence and Inequalities in the United States: 2014-2019. J Natl Cancer Inst. 2022;114: 381–390. doi:10.1093/jnci/djab208	No	Not meeting inclusion criteria	Title and abstract screening

2882	Leventhal AM, Tackett AP, Whitted L, Jordt SE, Jabba SV. Ice flavours and non-menthol synthetic cooling agents in e-cigarette products: a review. Tob Control. 2022. doi:10.1136/tobaccocontrol-2021-057073	No	Not meeting inclusion criteria	Title and abstract screening
2883	Leventhal AM, Tackett AP, Whitted L, Jordt SE, Jabba SV. Ice flavours and non-menthol synthetic cooling agents in e-cigarette products: a review. Tob Control. 2023;32: 769–777. doi:10.1136/tobaccocontrol-2021-057073	No	Not meeting inclusion criteria	Title and abstract screening
2884	Levin S.N., Venkatesh S., Nelson K.E., Li Y., Aguerre I., Zhu W., et al. Manifestations and impact of the COVID-19 pandemic in neuroinflammatory diseases. Ann Clin Transl Neurol. 2021;8: 918–928. doi:10.1002/acn3.51314	No	Not meeting inclusion criteria	Title and abstract screening
2885	Levoy K, Sullivan SS, Chittams J, Myers RL, Hickman SE, Meghani SH. Don't Throw the Baby Out With the Bathwater: Meta-Analysis of Advance Care Planning and End-of-life Cancer Care. J Pain Symptom Manage. 2023;65: e715–e743. doi:10.1016/j.jpainsymman.2023.02.003	No	Not meeting inclusion criteria	Title and abstract screening
2886	Levy D., Fong E., Wu B., Careskey M., Xoinis K., Harrington J., et al. Sevoflurane to the rescue: A convoluted case of respiratory failure in an asthmatic teenager. Crit Care Med. 2021;49: 494. doi:10.1097/01.ccm.0000729856.48260.2f	No	Not meeting inclusion criteria	Title and abstract screening
2887	Levy DT, Fong GT, Cummings KM, Borland R, Abrams DB, Villanti AC, et al. The need for a comprehensive framework. Addiction. 2017;112: 22–24. doi:10.1111/add.13600https://dx.doi.org/10.1111/add.13600	No	Not meeting inclusion criteria	Title and abstract screening
2888	Levy DT, Cadham CJ, Li Y, Yuan Z, Liber AC, Oh H, et al. A Decision-Theoretic Public Health Framework for Heated Tobacco and Nicotine Vaping Products. Int J Env Res Public Health. 2022;19. doi:10.3390/ijerph192013431	No	Not meeting inclusion criteria	Title and abstract screening
2889	Levy DT, Cadham CJ, Sanchez-Romero LM, Knoll M, Travis N, Yuan Z, et al. An Expert Elicitation on the Effects of a Ban on Menthol Cigarettes and Cigars in the United States. Nicotine Tob Res. 2021;23: 1911–1920. doi:10.1093/ntr/ntab121	No	Not meeting inclusion criteria	Title and abstract screening
2890	Levy DT, Cadham CJ, Yuan Z, Li Y, Gravely S, Cummings KM. Comparison of smoking prevalence in Canada before and after nicotine vaping product access using the SimSmoke model. Can J Public Health. 2023;114: 992–1005. doi:10.17269/s41997-023-00792-3	No	Not meeting inclusion criteria	Title and abstract screening
2891	Levy DT, Gartner C, Liber AC, Sánchez-Romero LM, Yuan Z, Li Y, et al. The Australia Smoking and Vaping Model: The Potential Impact of Increasing Access to Nicotine Vaping Products. Nicotine Tob Res. 2022. doi:10.1093/ntr/ntac210	No	Not meeting inclusion criteria	Title and abstract screening
2892	Levy DT, Gartner C, Liber AC, Sánchez-Romero LM, Yuan Z, Li Y, et al. The Australia Smoking and Vaping Model: The Potential Impact of Increasing Access to Nicotine Vaping Products. Nicotine Tob Res. 2023;25: 486–497. doi:10.1093/ntr/ntac210	No	Not meeting inclusion criteria	Title and abstract screening
2893	Levy DT, Meza R, Yuan Z, Li Y, Cadham C, Sanchez-Romero LM, et al. Public health impact of a US ban on menthol in cigarettes and cigars: a simulation study. Tob Control. 2021. doi:10.1136/tobaccocontrol-2021-056604	No	Not meeting inclusion criteria	Title and abstract screening
2894	Levy DT, Meza R, Yuan Z, Li Y, Cadham C, Sanchez-Romero LM, et al. Public health impact of a US ban on menthol in cigarettes and cigars: a simulation study. Tob Control. 2023;32: e37–e44. doi:10.1136/tobaccocontrol-2021-056604	No	Not meeting inclusion criteria	Title and abstract screening
2895	Levy DT, Thirlway F, Sweanor D, Liber A, Sanchez-Romero LM, Meza R, et al. Do Tobacco Companies Have an Incentive to Promote "Harm Reduction" Products?: The Role of Competition. Nicotine Tob Res. 2023. doi:10.1093/ntr/ntad014	No	Not meeting inclusion criteria	Title and abstract screening
2896	Levy DT, Warner KE, Liber AC, Travis N, Sweanor DT, Meza R, et al. Potential Implications for Tobacco Industry Transformation of the Acquisition of Swedish Match by Philip Morris International. Nicotine Tob Res. 2023;25: 1899–1903. doi:10.1093/ntr/ntad138	No	Not meeting inclusion criteria	Title and abstract screening
2897	Levy S., Evins A.E., Schuster R.M., Green L., Lunstead J., Fuller A., et al. Virtual Group Therapy Programs-The Wave of the Future. J Adolesc Health. 2021;69: 527. doi:10.1016/j.jadohealth.2021.05.018	No	Not meeting inclusion criteria	Title and abstract screening
2898	Levy S., Fuller A., Kelly S., Lunstead J., Weitzman E.R., Straus J.H. A Phone Consultation Call Line to Support SBIRT in Pediatric Primary Care. Front Psychiatry. 2022;13: 882486. doi:10.3389/fpsyt.2022.882486	No	Not meeting inclusion criteria	Title and abstract screening
2899	Lewis-Thames MW, Langston ME, Fuzzell L, Khan S, Moore JX, Han Y. Rural-urban differences e-cigarette ever use, the perception of harm, and e-cigarette information seeking behaviors among U.S. adults in a nationally representative study. Prev Med. 2020;130: 105898. doi:10.1016/j.ypmed.2019.105898	No	Not meeting inclusion criteria	Title and abstract screening
2900	Lewis-Thames MW, Langston ME, Khan S, Han Y, Fuzzell L, Xu S, et al. Racial and Ethnic Differences in Rural-Urban Trends in 5-Year Survival of Patients With Lung, Prostate, Breast, and Colorectal Cancers: 1975-2011 Surveillance, Epidemiology, and End Results (SEER). JAMA Netw Open. 2022;5: e2212246–e2212246. doi:10.1001/jamanetworkopen.2022.12246	No	Not meeting inclusion criteria	Title and abstract screening
2901	Li D, Ossip DJ, Bansal-Travers M, Xie Z. Impact of the FDA flavour enforcement policy on flavoured electronic cigarette use behaviour changes. Tob Control. 2022;31: s176–s183. doi:10.1136/tc-2022-057492	No	Not meeting inclusion criteria	Title and abstract screening
2902	Li J.C., Miller B. Vaping-associated spontaneous pneumomediastinum: A case and review of the literature. Am J Respir Crit Care Med. 2021;203. doi:10.1164/ajrccm-conference.2021.203.1_MeetingAbstracts.A3201	No	Not meeting inclusion criteria	Title and abstract screening
2903	Li J, Huynh L, Cornwell WD, Tang M-S, Simborio H, Huang J, et al. Electronic Cigarettes Induce Mitochondrial DNA Damage and Trigger TLR9 (Toll-Like Receptor 9)-Mediated Atherosclerosis. Arterioscler Thromb Vasc Biol. 2021;41: 839–853. doi:10.1161/ATVBAHA.120.315556	No	Not meeting inclusion criteria	Title and abstract screening
2904	Li J, Yu H, Peng L, Li L, Wang X, Hao J, et al. Novel nomogram predicting cancer-specific survival and overall survival in patients with primary esophageal small-cell carcinoma: A surveillance, epidemiology, and end results-based study. J Cancer Res Ther. 2021;17: 630–637. doi:10.4103/jcrt.JCRT_1612_20	No	Not meeting inclusion criteria	Title and abstract screening
2905	Li L, Borland R, Cummings KM, Fong GT, Gravely S, Smith DM, et al. How Does the Use of Flavored Nicotine Vaping Products Relate to Progression Toward Quitting Smoking? Findings From the 2016 and 2018 ITC 4CV Surveys. Nicotine Tob Res. 2021;23: 1490–1497. doi:10.1093/ntr/ntab033	No	Not meeting inclusion criteria	Title and abstract screening
2906	Li L, Borland R, Cummings KM, Gravely S, Quah ACK, Fong GT, et al. Patterns of Non-Cigarette Tobacco and Nicotine Use Among Current Cigarette Smokers and Recent Quitters: Findings From the 2020 ITC Four Country Smoking and Vaping Survey. Nicotine Tob Res. 2021;23: 1611–1616. doi:10.1093/ntr/ntab040	No	Not meeting inclusion criteria	Title and abstract screening
2907	Li L, Borland R, Cummings KM, Hyland A, Le Grande M, Fong GT, et al. Non-cigarette combustible tobacco use and its associations with subsequent cessation of smoking among daily cigarette smokers: findings from the International Tobacco Control Four Country Smoking and Vaping Surveys (2016-20). Addiction. 2022. doi:10.1111/add.16023	No	Not meeting inclusion criteria	Title and abstract screening
2908	Li L, Borland R, Cummings KM, Hyland A, Le Grande M, Fong GT, et al. Non-cigarette combustible tobacco use and its associations with subsequent cessation of smoking among daily cigarette smokers: findings from the International Tobacco Control Four Country Smoking and Vaping Surveys (2016-20). Addiction. 2023;118: 140–148. doi:10.1111/add.16023	No	Not meeting inclusion criteria	Title and abstract screening
2909	Li L, Borland R, Le Grande M, Gartner C. Future nicotine use preferences of current cigarette smokers: Findings from the 2020 International Tobacco Control Four Country Smoking and Vaping Survey. Drug Alcohol Rev. 2023. doi:10.1111/dar.13791	No	Not meeting inclusion criteria	Title and abstract screening
2910	Li L, Borland R, O'Connor RJ, Fong GT, McNeill A, Driezen P, et al. How Are Self-Reported Physical and Mental Health Conditions Related to Vaping Activities among Smokers and Quitters: Findings from the ITC Four Country Smoking and Vaping Wave 1 Survey. Int J Env Res Public Health. 2019;16. doi:10.3390/ijerph16081412	No	Not meeting inclusion criteria	Title and abstract screening

2911	Li L, Borland R, Yong HH, Gravely S, Fong GT, Cummings KM, et al. Experienced Effects on Well-Being following Smoking Cessation: Findings from the 2020 ITC Four Country Smoking and Vaping Survey. Int J Env Res Public Health. 2022;19. doi:10.3390/ijerph191610037	No	Not meeting inclusion criteria	Title and abstract screening
2912	Li L., Yang C., Zhan S., Wilson K.M., Taioli E., Mazumdar M., et al. Longitudinal Assessment of Association Between Tobacco Use and Tobacco Dependence Among Adults: Latent Class Analysis of the Population Assessment of Tobacco and Health Study Waves 1-4. Nicotine Tob Res Off J Soc Res Nicotine Tob. 2023. doi:10.1093/ntr/ntad114	No	Not meeting inclusion criteria	Title and abstract screening
2913	Li M., Vanegas S., Daunert S., Zingg J.-M. Role of vitamin E and cannabinoids in regulating cellular lipid homeostasis relevant for macrophages foam cells formation. Free Radic Biol Med. 2022;189: 26–27. doi:10.1016/j.freeradbiomed.2022.06.120	No	Not meeting inclusion criteria	Title and abstract screening
2914	Li P, Kong Y, Guo J, Ji X, Han X, Zhang B. Incidence and trends of hepatic cancer among children and adolescents in the United States from 2000 to 2017: Evidence from the Surveillance, Epidemiology, and End Results registry data. Cancer Causes Control. 2023;34: 69–79. doi:10.1007/s10552-022-01640-4	No	Not meeting inclusion criteria	Title and abstract screening
2915	Li T, Xie Q, Li J, Li Z, Xiao J, Liu M, et al. Adjuvant therapy fails to show survival benefit for patients with spindle cell carcinoma: Evidence from the surveillance, epidemiology, and end results database. J Cancer Res Ther. 2021;17: 1172–1178. doi:10.4103/jcrt.JCRT_1701_20	No	Not meeting inclusion criteria	Title and abstract screening
2916	Li Volti G, Polosa R. Clarifying allegations regarding CoEHAR conflicts of interest. Lancet Oncol. 2021;22: e378. doi:10.1016/S1470-2045(21)00477-0	No	Not meeting inclusion criteria	Title and abstract screening
2917	Li Volti G, Polosa R, Caruso M. Assessment of E-cigarette impact on smokers: The importance of experimental conditions relevant to human consumption. Proc Natl Acad Sci U A. 2018;115: E3073–E3074. doi:10.1073/pnas.1801967115	No	Not meeting inclusion criteria	Title and abstract screening
2918	Li W, Vargas-Rivera M, Ebrahimi Kalan M, Ben Taleb Z, Asfar T, Osibogun O, et al. The Effect of Graphic Health Warning Labels Placed on the ENDS Device on Young Adult Users' Experience, Exposure and Intention to Use: A Pilot Study. Health Commun. 2022;37: 842–849. doi:10.1080/10410236.2021.1872158	No	Not meeting inclusion criteria	Title and abstract screening
2919	Li W., Pan J., Wei M., Lv Z., Chen S., Qin Y., et al. Nonocular Influencing Factors for Primary Glaucoma: An Umbrella Review of Meta-Analysis. Ophthalmic Res. 2021;64: 938–950. doi:10.1159/000519247	No	Not meeting inclusion criteria	Title and abstract screening
2920	Li X, Hartwell KJ, Henderson S, Badran BW, Brady KT, George MS. Two weeks of image-guided left dorsolateral prefrontal cortex repetitive transcranial magnetic stimulation improves smoking cessation: A double-blind, sham-controlled, randomized clinical trial. Brain Stimul. 2020;13: 1271–1279. doi:10.1016/j.brs.2020.06.007	No	Not meeting inclusion criteria	Title and abstract screening
2921	Li X, Liu L, Guo L, Xu L, Kuang H, Xu C. Development of a colloidal gold immunochromatographic strip for rapid and sensitive detection of nicotine. J Pharm Biomed Anal. 2023;223: 115132. doi:10.1016/j.jpba.2022.115132	No	Not meeting inclusion criteria	Title and abstract screening
2922	Li X, Zhang Y, Zhang R, Chen F, Shao L, Zhang L. Association Between E-Cigarettes and Asthma in Adolescents: A Systematic Review and Meta-Analysis. Am J Prev Med. 2022;62: 953–960. doi:10.1016/j.amepre.2022.01.015	No	Not meeting inclusion criteria	Title and abstract screening
2923	Li X, Liu L, Guo L, Xu L, Kuang H, Xu C. Development of a colloidal gold immunochromatographic strip for rapid and sensitive detection of nicotine. J Pharm Biomed Anal. 2022;223: 115132. doi:10.1016/j.jpba.2022.115132	No	Not meeting inclusion criteria	Title and abstract screening
2924	Li Y, Dai J, Tran LN, Pinkerton KE, Spindel ER, Nguyen TB. Vaping Aerosols from Vitamin E Acetate and Tetrahydrocannabinol Oil: Chemistry and Composition. Chem Res Toxicol. 2022;35: 1095–1109. doi:10.1021/acs.chemrestox.2c00064	No	Not meeting inclusion criteria	Title and abstract screening
2925	Li Y, Su C, Yan Y, Wang Z, Wei X. A 75-Year-Old Man With Irregular Solid Components Within an Emphysematous Bulla. Chest. 2023;163: e265–e273. doi:10.1016/j.chest.2022.12.043	No	Not meeting inclusion criteria	Title and abstract screening
2926	Li Y, Zhang Y, Walayat A, Fu Y, Liu B, Zhang L, et al. The Regulatory Role of H19/miR-181a/ATG5 Signaling in Perinatal Nicotine Exposure-Induced Development of Neonatal Brain Hypoxic-Ischemic Sensitive Phenotype. Int J Mol Sci. 2022;23. doi:10.3390/ijms23136885	No	Not meeting inclusion criteria	Title and abstract screening
2927	Li Y, Hua R, He J, Zhang H. Survival Contradiction in Stage II, IIIA, And IIIB Colon Cancer: A Surveillance, Epidemiology, and End Result-Based Analysis. Evid-Based Complement Altern Med ECAM. 2022; 1–8. doi:10.1155/2022/4088117	No	Not meeting inclusion criteria	Title and abstract screening
2928	Li-Wei Zhong. A Nursing Experience of Caring for a Patient With Terminal Rectal Cancer at the End of Life in the Intensive Care Unit. Tzu Chi Nurs J. 2022;21: 110–118.	No	Not meeting inclusion criteria	Title and abstract screening
2929	Liang J, Abramson M, Zwar N, Russell G, Holland A, Bonevski B, et al. Diagnosing COPD and supporting smoking cessation in general practice: evidence-practice gaps. 2018;208: 29-34. doi:10.5694/mja17.00664	No	Not meeting inclusion criteria	Title and abstract screening
2930	Liber AC, Cadham C, Cummings M, Levy DT, Pesko M. Poland is not replicating the HTP experience in Japan: a cautionary note. Tob Control. 2023;32: 524–525. doi:10.1136/tobaccocontrol-2021-056887	No	Not meeting inclusion criteria	Title and abstract screening
2931	Liber AC, Cahn Z, Diaz MC, Donovan E, Vallone D, Schillo B. The EVALI outbreak and tobacco sales in the USA, 2014-2020. Tob Control. 2021. doi:10.1136/tobaccocontrol-2021-056807	No	Not meeting inclusion criteria	Title and abstract screening
2932	Liber AC, Cahn Z, Diaz MC, Donovan E, Vallone D, Schillo B. The EVALI outbreak and tobacco sales in the USA, 2014-2020. Tob Control. 2023;32: e166–e172. doi:10.1136/tobaccocontrol-2021-056807	No	Not meeting inclusion criteria	Title and abstract screening
2933	Liber AC, Faraji M, Ranganathan R, Friedman AS. How Complete Are Tobacco Sales Data? Assessing The Comprehensiveness Of US Tobacco Product Retail Sales Data Through Comparisons To Excise Tax Collections. Nicotine Tob Res. 2023. doi:10.1093/ntr/ntad214	No	Not meeting inclusion criteria	Title and abstract screening
2934	Liber AC, Knoll M, Cadham CJ, Issabakhsh M, Oh H, Cook S, et al. The role of flavored electronic nicotine delivery systems in smoking cessation: A systematic review. Drug Alcohol Depend Rep. 2023;7: 100143. doi:10.1016/j.dadr.2023.100143	No	Not meeting inclusion criteria	Title and abstract screening
2935	Liberman J, Wann S. E-Cigarettes-What a Practicing Cardiologist Needs to Know. Am J Cardiol. 2017;119: 681–686. doi:10.1016/j.amjcard.2016.11.011	No	Not meeting inclusion criteria	Title and abstract screening
2936	Lilley J., Kravitz S., Haynes Z., Church T., McKay S., Mertz A. E-cigarette, or vaping, product use associated lung injury and the risks and benefits of a thorough infectious work-up. Respir Med Case Rep. 2021;33: 101465. doi:10.1016/j.rmcr.2021.101465	No	Not meeting inclusion criteria	Title and abstract screening
2937	Lim KH, Heng PP, Nik Mohamed MH, Teh CH, Mohd Yusoff MF, Ling JMY, et al. Prevalence and Factors Associated With Attempts to Quit and Smoking Cessation in Malaysia. Asia Pac J Public Health. 2019;31: 22S-31S. doi:10.1177/1010539519874944	No	Not meeting inclusion criteria	Title and abstract screening
2938	Lim S.-L., Martin L.E., Catley D. Trial-by-Trial Fluctuations in Brain Responses to Stress Predict Subsequent Smoking Decisions That Occur Several Seconds Later. Biol Psychiatry Cogn Neurosci Neuroimaging. 2020;5: 1019–1027. doi:10.1016/j.bpsc.2020.06.012	No	Not meeting inclusion criteria	Title and abstract screening
2939	Limpert J., Murphy I., Konkel K., Rudy S. Possible nicotine-related health effects from electronic nicotine delivery system use by teens: Reports to the food and drug administration. Pediatrics. 2019;144. doi:10.1542/peds.144.2-MeetingAbstract.858	No	Not meeting inclusion criteria	Title and abstract screening

2940	Lin H, Chen M, Yun Q, Zhang L, Chang C. Tobacco dependence affects determinants related to quitting intention and behaviour. Sci Rep. 2021;11: 20202. doi:10.1038/s41598-021-99766-z	No	Not meeting inclusion criteria	Title and abstract screening
2941	Lin HC, Buu A, Su WC. Disposable E-Cigarettes and Associated Health Risks: An Experimental Study. Int J Env Res Public Health. 2022;19. doi:10.3390/ijerph191710633	No	Not meeting inclusion criteria	Title and abstract screening
2942	Lin HX, Zhang Y, Chen MJ, Zheng YT, Yun QP, Zhang LC, et al. The characteristics and patterns of e-cigarette use and its association with cigarette cessation intention in a Chinese smoking population: A mediation analysis. Tob Induc Dis. 2022;20: 16. doi:10.18332/tid/144251	No	Not meeting inclusion criteria	Title and abstract screening
2943	Lin J, Kamamia C, Brown DW, Shao S, McGlynn KA, Nations JA, et al. Comparative study of survival among small cell lung cancer patients in the U.S. military health system and those in the surveillance, epidemiology, and end results (SEER) program. Ann Epidemiol. 2021;64: 132–139. doi:10.1016/j.annepidem.2021.09.010	No	Not meeting inclusion criteria	Title and abstract screening
2944	Lin J, Zhan X, Chen R, Chen T, Jiang M, Li Y, et al. Increased Burden of Second Bladder Cancer and Rectal Cancer in Prostate Cancer Treated With Radiotherapy: Results From Surveillance, Epidemiology, and End Results. Cancer Control J Moffitt Cancer Cent. 2023; 1–10. doi:10.1177/10732748231177544	No	Not meeting inclusion criteria	Title and abstract screening
2945	Lin SH, Chen PS, Chen KC, Chang WH, Wang TY, Yang YK. Transcranial direct current stimulation (tDCS) may reduce the expired CO concentration among opioid users who smoke cigarettes: a randomized sham-controlled study. Psychiatry Res. 2021;299: 113874. doi:10.1016/j.psychres.2021.113874	No	Not meeting inclusion criteria	Title and abstract screening
2946	Lin W, Hobkirk AL, Zhu J, Krebs NM, Hayes JE, Richie JP Jr, et al. Effect of menthol on nicotine reduction: Pooled results from two double-blind randomized controlled trials. Brain Res Bull. 2022;189: 131–138. doi:10.1016/j.brainresbull.2022.08.019	No	Not meeting inclusion criteria	Title and abstract screening
2947	Lin W, Muscat JE. Knowledge and Beliefs Regarding Harm From Specific Tobacco Products: Findings From the H.I.N.T. Survey. Am J Health Promot. 2021; 8901171211026116. doi:10.1177/08901171211026116	No	Not meeting inclusion criteria	Title and abstract screening
2948	Lin W., Antwiler K.D., Mott J.A., Javier E. Cannabis-Induced Atypical Stress Cardiomyopathy: A Case Report. J Addict Med. 2022;16: e289–e290.	No	Not meeting inclusion criteria	Title and abstract screening
2949	Lindley LC, Svynarenko R, Mooney-Doyle K, Mendola A, Naumann WC, Harris R. A National Study of Healthcare Service Patterns at the End of Life Among Children With Cardiac Disease. J Cardiovasc Nurs. 2023;38: 44–51. doi:10.1097/JCN.0000000000000875	No	Not meeting inclusion criteria	Title and abstract screening
2950	Lindskog M, Schultz T, Strang P. Acute healthcare utilization in end-of-life among Swedish brain tumor patients – a population based register study. BMC Palliat Care. 2022;21: 1–10. doi:10.1186/s12904-022-01022-2	No	Not meeting inclusion criteria	Title and abstract screening
2951	Lindson N, Butler AR, Liber A, Levy DT, Barnett P, Theodoulou A, et al. An exploration of flavours in studies of e-cigarettes for smoking cessation: secondary analyses of a systematic review with meta-analyses. Addiction. 2023;118: 634–645. doi:10.1111/add.16091	No	Not meeting inclusion criteria	Title and abstract screening
2952	Lindson N, Theodoulou A, Ordóñez-Mena JM, Fanshawe TR, Sutton AJ, Livingstone-Banks J, et al. Pharmacological and electronic cigarette interventions for smoking cessation in adults: component network meta-analyses. Cochrane Database Syst Rev. 2023;9: CD015226. doi:10.1002/14651858.CD015226.pub2	No	Not meeting inclusion criteria	Title and abstract screening
2953	Lindson-Hawley N., Heath L., Hartmann-Boyce J. Twenty years of the Cochrane Tobacco Addiction Group: Past, present, and future. Nicotine Tob Res. 2018;20: 147–153. doi:10.1093/ntr/ntw274	No	Not meeting inclusion criteria	Title and abstract screening
2954	Ling PM, Glantz SA. Tobacco company strategies to identify and promote the benefits of nicotine. Tob Control. 2019;28: 289–296. doi:10.1136/tobaccocontrol-2018-054300	No	Not meeting inclusion criteria	Title and abstract screening
2955	Ling PM, Hrywna M, Talbot EM, Lewis MJ. Tobacco-Derived Nicotine Pouch Brands and Marketing Messages on Internet and Traditional Media: Content Analysis. JMIR Form Res. 2023;7: e39146. doi:10.2196/39146	No	Not meeting inclusion criteria	Title and abstract screening
2956	Linnansaari A, Ollila H, Pisinger C, Scheffels J, Kinnunen JM, Rimpelä A. Towards Tobacco-Free Generation: implementation of preventive tobacco policies in the Nordic countries. Scand J Public Health. 2022; 14034948221106867. doi:10.1177/14034948221106867	No	Not meeting inclusion criteria	Title and abstract screening
2957	Linnansaari A, Ollila H, Pisinger C, Scheffels J, Kinnunen JM, Rimpelä A. Towards Tobacco-Free Generation: implementation of preventive tobacco policies in the Nordic countries. Scand J Public Health. 2023;51: 1108–1121. doi:10.1177/14034948221106867	No	Not meeting inclusion criteria	Title and abstract screening
2958	Lippert AM. Association Between School-Level Prevalence of Electronic Cigarette Use and Student-Level Use Behaviors, Pre-Use Intentions, and Risk Perceptions: Evidence From the 2014 US National Youth Tobacco Survey. Nicotine Tob Res Off J Soc Res Nicotine Tob. 2018;20: 231–238. doi:10.1093/ntr/ntw395	No	Not meeting inclusion criteria	Title and abstract screening
2959	Little MA, Klesges RC, Bursac Z, Halbert JP, Ebbert J, Talcott GW, et al. Correlates of smoking status in cancer survivors. J Cancer Surviv. 2018;12: 828–834. doi:10.1007/s11764-018-0720-x	No	Not meeting inclusion criteria	Title and abstract screening
2960	Little MA, Pebley K, Reid T, Morris JD, Wiseman KP. Rationale, design, and methods for the development of a youth adapted Brief Tobacco Intervention plus automated text messaging for high school students. Contemp Clin Trials. 2022;119: 106840. doi:10.1016/j.cct.2022.106840	No	Not meeting inclusion criteria	Title and abstract screening
2961	Littman D., Chino F. Availability, reading level, quality, and accessibility of online cancer center smoking cessation materials. J Clin Oncol. 2021;39. doi:10.1200/JCO.2021.39.suppl.e18662	No	Not meeting inclusion criteria	Title and abstract screening
2962	Litton JK, Regan MM, Pusztai L, Rugo HS, Tolaney SM, Garrett-Mayer E, et al. Standardized Definitions for Efficacy End Points in Neoadjuvant Breast Cancer Clinical Trials: NeoSTEEP. J Clin Oncol. 2023;41: 4433–4442. doi:10.1200/JCO.23.00435	No	Not meeting inclusion criteria	Title and abstract screening
2963	Liu C, Yong HH, Gravely S, East K, Kasza K, Gartner C, et al. Gender differences in cigarette smoking cessation attempts among adults who smoke and drink alcohol at high levels: Findings from the 2018-2020 International Tobacco Control Four Country Smoking and Vaping Surveys. Addict Behav. 2023;147: 107817. doi:10.1016/j.addbeh.2023.107817	No	Not meeting inclusion criteria	Title and abstract screening
2964	Liu H, Yu Z, Xu Z, Liu T, Liu W. A scientometric study of tobacco and alcohol use as risk factors for oral cavity health. J Dent Sci. 2023;18: 1883–1888. doi:10.1016/j.jds.2023.05.016	No	Not meeting inclusion criteria	Title and abstract screening
2965	Liu H, Li Q, Zhan Y, Zhang Z, Zeng DD, Leischow SJ. Characterizing Social Media Messages Related to Underage JUUL E-Cigarette Buying and Selling: Cross-Sectional Analysis of Reddit Subreddits. J Med Internet Res. 2020;22: e16962. doi:10.2196/16962	No	Not meeting inclusion criteria	Title and abstract screening
2966	Liu H, Wang R. Associations between the serum magnesium and all-cause or cardiovascular mortality in chronic kidney disease and end-stage renal disease patients: A meta-analysis. Medicine (Baltimore). 2021;100: e27486–e27486. doi:10.1097/MD.0000000000027486	No	Not meeting inclusion criteria	Title and abstract screening
2967	Liu J, Keller-Hamilton B, Patterson JG, Lee DN, Wedel AV, Vázquez-Otero C, et al. How Age and E-cigarette Use Status Interact to Influence E-cigarette Ad Perceptions. Subst Use Misuse. 2023;58: 257–265. doi:10.1080/10826084.2022.2155479	No	Not meeting inclusion criteria	Title and abstract screening
2968	Liu J, Patterson JG, Keller-Hamilton B, Lee DN, Chrzan KR, Stevens EM. Sexual orientation and gender identity differences in perceptions and product appeal in response to e-cigarette advertising. Tob Induc Dis. 2023;21: 111. doi:10.18332/tid/169739	No	Not meeting inclusion criteria	Title and abstract screening

2969	Liu J, Rensch J, Wang J, Jin X, Vansickel A, Edmiston J, et al. Nicotine pharmacokinetics and subjective responses after using nicotine pouches with different nicotine levels compared to combustible cigarettes and moist smokeless tobacco in adult tobacco users. Psychopharmacol Berl. 2022;239: 2863–2873. doi:10.1007/s00213-022-06172-y	No	Not meeting inclusion criteria	Title and abstract screening
2970	Liu J, Reynolds MJ, Hartman L, Gottlieb M, Coogan JM, Winickoff JP. The First State Tobacco Flavor Ban: High School Tobacco Control Needs in a Changing Landscape. J Sch Health. 2022;92: 720–727. doi:10.1111/josh.13171	No	Not meeting inclusion criteria	Title and abstract screening
2971	Liu J, Roberts J, Reynolds MJ, Hanby E, Gundersen DA, Winickoff JP, et al. Barriers and facilitators to address vaping in Massachusetts schools: a mixed-methods study of school-based stakeholders. Transl Behav Med. 2023;13: 589–600. doi:10.1093/tbm/ibad012	No	Not meeting inclusion criteria	Title and abstract screening
2972	Liu J, Tan ASL, Lee J. Vaping of Cannabis, Cannabidiol, and Synthetic Cannabis Among US Sexual Minority Youths. JAMA Netw Open. 2023;6: e2329041. doi:10.1001/jamanetworkopen.2023.29041	No	Not meeting inclusion criteria	Title and abstract screening
2973	Liu J, Tan ASL, Winickoff JP, Rees VW. Correlates of adolescent sole-, dual- and poly-use of cannabis, vaped nicotine, and combusted tobacco. Addict Behav. 2023;146: 107804. doi:10.1016/j.addbeh.2023.107804	No	Not meeting inclusion criteria	Title and abstract screening
2974	Liu J, Winickoff JP, Hanby E, Rees V, Emmons KM, Tan AS. Prevalence and correlates of past 30-day dual-vaping of nicotine and cannabis among adolescents in five New England states. Drug Alcohol Depend. 2023;254: 111055. doi:10.1016/j.drugalcdep.2023.111055	No	Not meeting inclusion criteria	Title and abstract screening
2975	Liu J, Wright C, Elizarova O, Dahne J, Bian J, Tan ASL. Emotional Responses and Perceived Relative Harm Mediate the Effect of Exposure to Misinformation about E-Cigarettes on Twitter and Intention to Purchase E-Cigarettes among Adult Smokers. Int J Env Res Public Health. 2021;18. doi:10.3390/ijerph182312347	No	Not meeting inclusion criteria	Title and abstract screening
2976	Liu J, Wright C, Williams P, Elizarova O, Dahne J, Bian J, et al. Smokers' Likelihood to Engage With Information and Misinformation on Twitter About the Relative Harms of e-Cigarette Use: Results From a Randomized Controlled Trial. JMIR Public Health Surveill. 2021;7: e27183. doi:10.2196/27183	No	Not meeting inclusion criteria	Title and abstract screening
2977	Liu J., Halpern-Felsher B. The Juul Curriculum Is Not the Jewel of Tobacco Prevention Education. J Adolesc Health. 2018;63: 527–528. doi:10.1016/j.jadohealth.2018.08.005	No	Not meeting inclusion criteria	Title and abstract screening
2978	Liu J., Wang J., Wang Y., Shao X., Lutfy K., Friedman T., et al. Nicotine E-Cigarette Vapors Exposure Disturbs 11beta- Hydroxysteroid Dehydrogenase Type 2 Expression in the Kidney May Contribute to E-Cigarette-Induced Blood Pressure Elevation in Mice. J Endocr Soc. 2022;6: A258–A259. doi:10.1210/jendso/bvac150.531	No	Not meeting inclusion criteria	Title and abstract screening
2979	Liu J. Adolescent vaping: multiple substance use and implications for school-based prevention programs. Diss Abstr Int Sect B Sci Eng. 2023;84: No-Specified.	No	Not meeting inclusion criteria	Title and abstract screening
2980	Liu J, Gaiha SM, Halpern-Felsher B. A Breath of Knowledge: Overview of Current Adolescent E-cigarette Prevention and Cessation Programs. Curr Addict Rep. 2020;7: 520–532. doi:10.1007/s40429-020-00345-5	No	Not meeting inclusion criteria	Title and abstract screening
2981	Liu J, Gaiha SM, Halpern-Felsher B. School-based programs to prevent adolescent e-cigarette use: A report card. Curr Probl Pediatr Adolesc Health Care. 2022;52: 101204. doi:10.1016/j.cppeds.2022.101204	No	Not meeting inclusion criteria	Title and abstract screening
2982	Liu J, Wright C, Elizarova O, Dahne J, Bian J, Williams P, et al. Effects of brief exposure to misinformation about e-cigarette harms on Twitter on knowledge and perceptions of e-cigarettes. Digit Health. 2022;8: 20552076221116780. doi:10.1177/20552076221116780	No	Not meeting inclusion criteria	Title and abstract screening
2983	Liu J, Wang M. Development and validation of nomograms predicting cancer-specific survival of vulvar cancer patients: based on the Surveillance, Epidemiology, and End Results Program. Int J Gynecol Obstet. 2022;156: 529–538. doi:10.1002/ijgo.13722	No	Not meeting inclusion criteria	Title and abstract screening
2984	Liu L, Ma L, Chen Z, Geng H, Xi L, McClement S, et al. Dignity at the end of life in traditional Chinese culture: Perspectives of advanced cancer patients and family members. Eur J Oncol Nurs. 2021;54: N.PAG-N.PAG. doi:10.1016/j.ejon.2021.102017	No	Not meeting inclusion criteria	Title and abstract screening
2985	Liu P, Xu L, Chen G, Shi B, Zhang Q, Chen S. Nomograms for predicting survival in patients with micropapillary bladder cancer: a real-world analysis based on the surveillance, epidemiology, and end results database and external validation in a tertiary center. BMC Urol. 2023;23: 1–12. doi:10.1186/s12894-023-01183-z	No	Not meeting inclusion criteria	Title and abstract screening
2986	Liu P-I, Lin M-N, Ho P-S, Tsai J-H, Yang Y-H, Wu K-F, et al. Prediction and potential risk factors for electronic cigarette use behaviors among adolescents: a pilot study in Chiayi, Taiwan. Front Public Health. 2023;11: 1140615. doi:10.3389/fpubh.2023.1140615	No	Not meeting inclusion criteria	Title and abstract screening
2987	Liu S, Yang J. The Role of Temporal Distance Perception in Narrative vs. Non-Narrative Persuasion Related to E-Cigarettes. 2020;25: 543-553. doi:10.1080/10810730.2020.1788678	No	Not meeting inclusion criteria	Title and abstract screening
2988	Liu TJ, Tokita HK, Simon BA. An Enhanced Ambulatory Surgery Experience for Patients with Cancer Through End-to-End Patient Engagement. Adv Anesth. 2022;40: 33–44. doi:10.1016/j.aan.2022.07.001	No	Not meeting inclusion criteria	Title and abstract screening
2989	Liu X, Hadiatullah H, Schnelle-Kreis J, Xu Y, Yue M, Zhang X, et al. Levels and drivers of urban black carbon and health risk assessment during pre- and COVID19 lockdown in Augsburg, Germany. Env Pollut. 2023;316: 120529. doi:10.1016/j.envpol.2022.120529	No	Not meeting inclusion criteria	Title and abstract screening
2990	Liu Y, Greenlund KJ, VanFrank B, Xu F, Lu H, Croft JB. Smoking Cessation Among U.S. Adult Smokers With and Without Chronic Obstructive Pulmonary Disease, 2018. Am J Prev Med. 2022;62: 492–502. doi:10.1016/j.amepre.2021.12.001	No	Not meeting inclusion criteria	Title and abstract screening
2991	Liu Y-L, Wu C-T, Hsu Y-C, Chen M-F, Chen C-S, Shi C-S, et al. Perioperative Complications and Oncologic Outcomes after Radical Cystectomy in End-Stage Renal Disease Patients with Bladder Cancer Obtained Using a Standardized Reporting System. Cancers. 2022;14: N.PAG-N.PAG. doi:10.3390/cancers14143512	No	Not meeting inclusion criteria	Title and abstract screening
2992	Liu Z, Zhang Y, Youn JY, Zhang Y, Makino A, Yuan JX-J, et al. Flavored and Nicotine-Containing E-Cigarettes Induce Impaired Angiogenesis and Diabetic Wound Healing via Increased Endothelial Oxidative Stress and Reduced NO Bioavailability. Antioxid Basel Switz. 2022;11. doi:10.3390/antiox11050904	No	Not meeting inclusion criteria	Title and abstract screening
2993	Livingston JA, Chen CH, Kwon M, Park E. Physical and mental health outcomes associated with adolescent E-cigarette use. J Pediatr Nurs. 2022;64: 1–17. doi:10.1016/j.pedn.2022.01.006	No	Not meeting inclusion criteria	Title and abstract screening
2994	Llanes KD, Ling PM, Guillory J, Vogel EA. Young Adults' Perceptions of and Intentions to Use Nicotine and Cannabis Vaporizers in Response to e-Cigarette or Vaping-Associated Lung Injury Instagram Posts: Experimental Study. J Med Internet Res. 2023;25: e46153. doi:10.2196/46153	No	Not meeting inclusion criteria	Title and abstract screening
2995	Lo H-Y, Lin Y-S, Lin DS-H, Lee J-K, Chen W-J. Association of Statin Therapy With Major Adverse Cardiovascular and Limb Outcomes in Patients With End-stage Kidney Disease and Peripheral Artery Disease Receiving Maintenance Dialysis. JAMA Netw Open. 2022;5: e2229706–e2229706. doi:10.1001/jamanetworkopen.2022.29706	No	Not meeting inclusion criteria	Title and abstract screening
2996	Lo JJ-M, Graves N, Chee JH, Hildon ZJ-L. A systematic review defining non-beneficial and inappropriate end-of-life treatment in patients with non-cancer diagnoses: theoretical development for multi-stakeholder intervention design in acute care settings. BMC Palliat Care. 2022;21: 1–14. doi:10.1186/s12904-022-01071-7	No	Not meeting inclusion criteria	Title and abstract screening
2997	Lochbuehler K, Wileyto EP, Tang KZ, Mercincavage M, Cappella JN, Strasser AA. Do current and former cigarette smokers have an attentional bias for e-cigarette cues? J Psychopharmacol. 2018;32: 316–323. doi:10.1177/0269881117728418	No	Not meeting inclusion criteria	Title and abstract screening

2998	Loffredo L., Carnevale R., Battaglia S., Marti R., Pizzolo S., Bartimoccia S., et al. Impact of chronic use of heat-not-burn cigarettes on oxidative stress, endothelial dysfunction and platelet activation: The SUR-VAPES Chronic Study. Thorax. 2021;76: 618–620. doi:10.1136/thoraxjnl-2020-215900	No	Not meeting inclusion criteria	Title and abstract screening
2999	London GM, Safar ME, Pannier B. The Age–Stiffness Relationships of Elastic and Muscular Arteries in a Control Population and in End-Stage Renal Disease Patients. Kidney Dial. 2023;3: 36–45. doi:10.3390/kidneydial3010003	No	Not meeting inclusion criteria	Title and abstract screening
3000	Long L, Alalwan MA, Keller-Hamilton B, Patterson JG, Roberts ME, Wagener TL, et al. Perceptions of oral nicotine pouches & their marketing among Ohio Appalachia smokers and smokeless tobacco users. PLoS One. 2023;18: e0293597. doi:10.1371/journal.pone.0293597	No	Not meeting inclusion criteria	Title and abstract screening
3001	Long L., Alalwan M.A., Keller-Hamilton B., Slater M.D., Mays D. Correlates of U.S. Young adults' awareness of alcohol use as a behavioral risk factor for cancer. Prev Med Rep. 2022;27: 101765. doi:10.1016/j.pmedr.2022.101765	No	Not meeting inclusion criteria	Title and abstract screening
3002	Loo L.C., Ong K.W., Nidzwal A.K.A., Razali M.H., Naim A., Daud F., et al. Knowledge of chronic kidney disease among undergraduate and postgraduate students in a public university in Klang Valley, Malaysia: A cross sectional study. PLoS ONE. 2022;17: e0274038. doi:10.1371/journal.pone.0274038	No	Not meeting inclusion criteria	Title and abstract screening
3003	Lopez-Olivo MA, Michaud K, Schumacher R, Minnix J, Cinciripini P, Suarez-Almazor ME. Smoking cessation patterns, usefulness of quitting methods, and tobacco cessation motivators and barriers to quit in patients with rheumatoid arthritis. Clin Rheumatol. 2023;42: 2053–2068. doi:10.1007/s10067-023-06593-w	No	Not meeting inclusion criteria	Title and abstract screening
3004	Lopez-Olivo MA, James J, James J, Krause KJ, Roth M, Palos GR, et al. A systematic review and meta-analysis of e-cigarette use among cancer survivors. J Cancer Surviv Res Pract. 2023. doi:10.1007/s11764-023-01357-6	No	Not meeting inclusion criteria	Title and abstract screening
3005	Lorensia A, Pratama AM, Hersandio R. Knowledge and attitudes on smoking cessation of e-cigarettes: a mixed-methods study of pharmacy students in Surabaya, Indonesia. J Prev Med Hyg. 2021;62: E918–E925. doi:10.15167/2421-4248/jpmh2021.62.4.2330	No	Not meeting inclusion criteria	Title and abstract screening
3006	Lorenzo A, Noël F, Lorenzo M, Van Den Broucke J. [The role of spirometry in encouraging smoking cessation in general practice. A pilot study using "lung age"]. Rev Mal Respir. 2017;34: 734–741. doi:10.1016/j.rmr.2016.10.873	No	Not meeting inclusion criteria	Title and abstract screening
3007	Lorza Blasco JJ. Emerging Trends in Nicotine Consumption. Arch Bronconeumol Engl Ed. 2019;55: 71–72. doi:10.1016/j.arbres.2018.03.024	No	Not meeting inclusion criteria	Title and abstract screening
3008	Loud EE, Duong HT, Henderson KC, Reynolds RM, Ashley DL, Thrasher JF, et al. Addicted to smoking or addicted to nicotine? A focus group study on perceptions of nicotine and addiction among US adult current smokers, former smokers, non-smokers and dual users of cigarettes and e-cigarettes. Addict Abingdon Engl. 2022;117: 472–481. doi:10.1111/add.15634	No	Not meeting inclusion criteria	Title and abstract screening
3009	Loukas A, Li X, Wilkinson AV, Marti CN. Longitudinal Examination of ENDS Use Among Young Adult College Students: Associations with Depressive Symptoms and Sensation Seeking. Prev Sci. 2023;24: 1068–1077. doi:10.1007/s11121-023-01572-8	No	Not meeting inclusion criteria	Title and abstract screening
3010	Loukas A, Marti CN, Harrell MB. Electronic nicotine delivery systems use predicts transitions in cigarette smoking among young adults. Drug Alcohol Depend. 2022;231: 109251. doi:10.1016/j.drugalcdep.2021.109251	No	Not meeting inclusion criteria	Title and abstract screening
3011	Love C.A., Kim H.H., Porter N.A., Jaspers I., Clapp P.W. Cannabidiol (CBD) in e-cigarette liquid adducts to cellular proteins induces inflammation and impairs cilia motility in bronchial epithelial cells. J Aerosol Med Pulm Drug Deliv. 2021;34: A6. doi:10.1089/jamp.2021.ab02.abstracts	No	Not meeting inclusion criteria	Title and abstract screening
3012	Love CA, Kim H-YH, Tallman KA, Clapp PW, Porter NA, Jaspers I. Vaping Induced Cannabidiol (CBD) Oxidation Product CBD Quinone Forms Protein Adducts with KEAP1 and Activates KEAP1-Nrf2 Genes. Chem Res Toxicol. 2023;36: 565–569. doi:10.1021/acs.chemrestox.3c00038	No	Not meeting inclusion criteria	Title and abstract screening
3013	Love CA, Schichlein KD, Clapp PW, Jaspers I. Cannabinoid Vaping Products Present Novel Challenges for Assessment of Respiratory Health Effects. Toxicol Sci Off J Soc Toxicol. 2022;188: 1–3. doi:10.1093/toxsci/kfac050	No	Not meeting inclusion criteria	Title and abstract screening
3014	Love M, Gierer S. Electronic Cigarettes and Vaping in Allergic and Asthmatic Disease. Immunol Allergy Clin North Am. 2022;42: 787–800. doi:10.1016/j.iac.2022.06.002	No	Not meeting inclusion criteria	Title and abstract screening
3015	Lu F, Yu M, Chen C, Liu L, Zhao P, Shen B, et al. The Emission of VOCs and CO from Heated Tobacco Products, Electronic Cigarettes, and Conventional Cigarettes, and Their Health Risk. Toxics. 2021;10. doi:10.3390/toxics10010008	No	Not meeting inclusion criteria	Title and abstract screening
3016	Lu J, Lee EWJ. Examining Twitter Discourse on Electronic Cigarette and Tobacco Consumption During National Cancer Prevention Month in 2018: Topic Modeling and Geospatial Analysis. J Med Internet Res. 2021;23: e28042. doi:10.2196/28042	No	Not meeting inclusion criteria	Title and abstract screening
3017	Lu Y, Dobbs PD, Song H, Dunlap C, Cheney MK. Harm perceptions, JUUL dependence, and other tobacco product use among young adults who use JUUL. Addict Behav. 2022;127: 107210. doi:10.1016/j.addbeh.2021.107210	No	Not meeting inclusion criteria	Title and abstract screening
3018	Lucchiari C, Masiero M, Mazzocco K, Veronesi G, Maisonneuve P, Jemos C, et al. Benefits of e-cigarettes in smoking reduction and in pulmonary health among chronic smokers undergoing a lung cancer screening program at 6 months. Addict Behav. 2020;103: 106222. doi:10.1016/j.addbeh.2019.106222	No	Not meeting inclusion criteria	Title and abstract screening
3019	Lucchiari C, Masiero M, Mazzocco K, Veronesi G, Maisonneuve P, Jemos C, et al. Nicotine-Free E-Cigarettes Might Promote Tobacco Smoking Reduction Better Than Nicotine Delivery Devices: results of a Double-Blind Randomized Controlled Trial at 1 Year. 2022;29: 8579-8590. doi:10.3390/curroncol29110676	No	Not meeting inclusion criteria	Title and abstract screening
3020	Lucherini M. Caught in the middle: early career researchers, public health and the emotional production of research. Crit Public Health. 2020;30: 367–372. doi:10.1080/09581596.2018.1550252	No	Not meeting inclusion criteria	Title and abstract screening
3021	Lucherini M., Rooke C., Amos A. "They're thinking, well it's not as bad, I probably won't get addicted to that. But it's still got the nicotine in it, so...": Maturity, control, and socializing: Negotiating identities in relation to smoking and vaping-a qualitative study of young adults. Nicotine Tob Res. 2019;21: 81–87. doi:10.1093/ntr/ntx245	No	Not meeting inclusion criteria	Title and abstract screening
3022	Lugo A, Stival C, Paroni L, Amerio A, Carreras G, Gorini G, et al. The impact of COVID-19 lockdown on gambling habit: A cross-sectional study from Italy. J Behav Addict. 2021;10: 711–721. doi:10.1556/2006.2021.00033	No	Not meeting inclusion criteria	Title and abstract screening
3023	Lugo A., Liu X., Pacifici R., Spizzichino L., Gallus S. Awareness, use, sales and interest of heat-not-burn tobacco products in Italy. Tob Induc Dis. 2018;16: 87–88. doi:10.18332/tid/84144	No	Not meeting inclusion criteria	Title and abstract screening
3024	Lum A, Skelton E, Robinson M, Guillaumier A, Wynne O, Gartner C, et al. Barriers and facilitators to using vaporised nicotine products as smoking cessation aids among people receiving treatment for substance use disorder. Addict Behav. 2022;124: 107097. doi:10.1016/j.addbeh.2021.107097	No	Not meeting inclusion criteria	Title and abstract screening
3025	Lund I, Saebo G. Vaping among Norwegians who smoke or formerly smoked: reasons, patterns of use, and smoking cessation activity. Harm Reduct J. 2023;20: 35. doi:10.1186/s12954-023-00768-z	No	Not meeting inclusion criteria	Title and abstract screening
3026	Lundahl LH, Cannoy C. COVID-19 and Substance Use in Adolescents. Pediatr Clin North Am. 2021;68: 977–990. doi:10.1016/j.pcl.2021.05.005	No	Not meeting inclusion criteria	Title and abstract screening

3027	Luo T., Li M.S., Williams D., Phillippi S., Yu Q., Kantrow S., et al. Using social media for smoking cessation interventions: a systematic review. Perspect Public Health. 2021;141: 50–63. doi:10.1177/1757913920906845	No	Not meeting inclusion criteria	Title and abstract screening
3028	Luo X, Carmella S, Chen M, Jensen J, Wilkens L, Le Marchand L, et al. Urinary Cyanoethyl Mercapturic Acid, a Biomarker of the Smoke Toxicant Acrylonitrile, Clearly Distinguishes Smokers From Nonsmokers. 2020;22: 1744-1747. doi:10.1093/ntr/ntaa080	No	Not meeting inclusion criteria	Title and abstract screening
3029	Luu NM, Phan TH, Oh JK, Myung SK. Exposure to Electronic Cigarette Advertisements and Use of Electronic Cigarettes: A Meta-analysis of Prospective Studies. Nicotine Tob Res. 2022. doi:10.1093/ntr/ntac266	No	Not meeting inclusion criteria	Title and abstract screening
3030	Luu NM, Phan TH, Oh JK, Myung SK. Exposure to Electronic Cigarette Advertisements and Use of Electronic Cigarettes: A Meta-analysis of Prospective Studies. Nicotine Tob Res. 2023;25: 983–990. doi:10.1093/ntr/ntac266	No	Not meeting inclusion criteria	Title and abstract screening
3031	Luxton NA, Shih P, Rahman MA, Adams R, MacKenzie R. Use of electronic cigarettes in the perioperative period: A mixed-method study exploring perceptions of cardiothoracic patients in Australia. Tob Induc Dis. 2018;16: 53. doi:10.18332/tid/98957	No	Not meeting inclusion criteria	Title and abstract screening
3032	Lwin T.S., Mitrakrishnan R.N., Farooq M., Alama M. ST-segment elevationmyocardial infarction with normal coronary arteries secondary to anterior communicating cerebral artery aneurysmal rupture: A case report. Eur Heart J - Case Rep. 2021;5. doi:10.1093/ehjcr/ytab168	No	Not meeting inclusion criteria	Title and abstract screening
3033	Lynch A, Lyon L, Ramalingam ND, Whitehead H, Chang M, Liu R. End-of-life outcomes of patients with advanced cancer enrolled in palliative care. J Clin Oncol. 41: 12030–12030. doi:10.1200/JCO.2023.41.16_suppl.12030	No	Not meeting inclusion criteria	Title and abstract screening
3034	Lynn R.S.R., Galinkin J.L. Cannabis, e-cigarettes and anesthesia. Curr Opin Anaesthesiol. 2020;33: 318–326. doi:10.1097/ACO.0000000000000872	No	Not meeting inclusion criteria	Title and abstract screening
3035	Lyu JC, Huang P, Ling PM. Electronic cigarette related topics with conflicting arguments in Chinese newspapers. Tob Induc Dis. 2022;20: 25. doi:10.18332/tid/145929	No	Not meeting inclusion criteria	Title and abstract screening
3036	Lyu JC, Luli GK, Ling PM. Vaping discussion in the COVID-19 pandemic: An observational study using Twitter data. PLoS One. 2021;16: e0260290. doi:10.1371/journal.pone.0260290	No	Not meeting inclusion criteria	Title and abstract screening
3037	Lyu JC, Sung HY, Yao T, Quah ACK, Jiang Y, Fong GT, et al. Receiving and giving electronic cigarettes as gifts in China: Findings from International Tobacco Control China Survey. Prev Med Rep. 2022;27: 101763. doi:10.1016/j.pmedr.2022.101763	No	Not meeting inclusion criteria	Title and abstract screening
3038	Ma H, Gottfredson O'Shea N, Kieu T, Rohde JA, Hall MG, Brewer NT, et al. Examining the Longitudinal Relationship Between Perceived and Actual Message Effectiveness: A Randomized Trial. Health Commun. 2023; 1–10. doi:10.1080/10410236.2023.2222459	No	Not meeting inclusion criteria	Title and abstract screening
3039	Ma H, Kieu TK, Ribisl KM, Noar SM. Do Vaping Prevention Messages Impact Adolescents and Young Adults? A Meta-Analysis of Experimental Studies. Health Commun. 2023;38: 1709–1722. doi:10.1080/10410236.2023.2185578	No	Not meeting inclusion criteria	Title and abstract screening
3040	Ma H, Gaudiello E, Sheeran P, Sanzo N, Sutfin EL, Noar SM. National Youth tobacco surveys (2014-2019) show increasing beliefs in the harm and relative addiction of e-cigarettes but decreasing associations between those beliefs and e-cigarette use. Addict Behav. 2023;144: 107713. doi:10.1016/j.addbeh.2023.107713	No	Not meeting inclusion criteria	Title and abstract screening
3041	Ma S, Jiang S, Wagener T, Mays D, Chen J, Shang C. The associations between e-liquid characteristics and its pricing: Evidence from online vape shops. PLoS One. 2023;18: e0286258. doi:10.1371/journal.pone.0286258	No	Not meeting inclusion criteria	Title and abstract screening
3042	Ma S, Qiu Z, Yang Q, Bridges JFP, Chen J, Shang C. Expanding the E-Liquid Flavor Wheel: Classification of Emerging E-Liquid Flavors in Online Vape Shops. Int J Env Res Public Health. 2022;19. doi:10.3390/ijerph192113953	No	Not meeting inclusion criteria	Title and abstract screening
3043	Ma T, Wang X, Li L, Sun B, Zhu Y, Xia T. Electronic cigarette aerosols induce oxidative stress-dependent cell death and NF-kappaB mediated acute lung inflammation in mice. Arch Toxicol. 2021;95: 195–205. doi:10.1007/s00204-020-02920-1	No	Not meeting inclusion criteria	Title and abstract screening
3044	Maalej R., Zgueb Y., Aissa A., Ouali U., Nacef F. Adolescents' attitudes towards e-cigarette in Tunisia. Eur Psychiatry. 2021;64: S823. doi:10.1192/j.eurpsy.2021.2174	No	Not meeting inclusion criteria	Title and abstract screening
3045	Maan M, Abuzayeda M, Kaklamanos EG, Jamal M, Dutta M, Moharamzadeh K. Molecular insights into the role of electronic cigarettes in oral carcinogenesis. Crit Rev Toxicol. 2023;53: 1–14. doi:10.1080/10408444.2023.2190764	No	Not meeting inclusion criteria	Title and abstract screening
3046	MacCallum C.A., Lo L.A., Boivin M. "Is medical cannabis safe for my patients?" A practical review of cannabis safety considerations. Eur J Intern Med. 2021;89: 10–18. doi:10.1016/j.ejim.2021.05.002	No	Not meeting inclusion criteria	Title and abstract screening
3047	MacCoun RJ. Multiple gateways. Addiction. 2018;113: 1785–1786. doi:10.1111/add.14196	No	Not meeting inclusion criteria	Title and abstract screening
3048	Macdonald A., Middlekauff H.R. Electronic cigarettes and cardiovascular health: What do we know so far? Vasc Health Risk Manag. 2019;15: 159–174. doi:10.2147/VHRM.S175970	No	Not meeting inclusion criteria	Title and abstract screening
3049	Machiorlatti M, Krebs N, Sun D, Muscat JE. Diurnal variability of cortisol in the Pennsylvania adult smoking study: Exploration of association with nicotine intake. Int J Psychophysiol. 2023;186: 24–32. doi:10.1016/j.ijpsycho.2023.02.001	No	Not meeting inclusion criteria	Title and abstract screening
3050	Maciag MC, Yousuf A, Hauptman M. Impact of Prenatal Exposure to Smoking on Child Health. Clin Obstet Gynecol. 2022;65: 388–396. doi:10.1097/GRF.0000000000000698	No	Not meeting inclusion criteria	Title and abstract screening
3051	MACIAG MC, YOUSUF A, HAUPTMAN M. Impact of Prenatal Exposure to Smoking on Child Health. Clin Obstet Gynecol. 2022;65: 388–396. doi:10.1097/GRF.0000000000000698	No	Not meeting inclusion criteria	Title and abstract screening
3052	Mack JW, Cernik C, Uno H, Xu L, Laurent CA, Fisher L, et al. Discussions About Goals of Care and Advance Care Planning Among Adolescents and Young Adults With Cancer Approaching the End of Life. J Clin Oncol. 2023;41: 4739–4746. doi:10.1200/JCO.23.00641	No	Not meeting inclusion criteria	Title and abstract screening
3053	Mack JW, Fisher L, Kushi L, Chao CR, Vega B, Rodrigues G, et al. Patient, Family, and Clinician Perspectives on End-of-Life Care Quality Domains and Candidate Indicators for Adolescents and Young Adults With Cancer. JAMA Netw Open. 2021;4: e2121888–e2121888. doi:10.1001/jamanetworkopen.2021.21888	No	Not meeting inclusion criteria	Title and abstract screening
3054	Magalhaes M. Vaping Restrictions: Is Priority to the Young Justified? Nicotine Tob Res. 2021;23: 32–35. doi:10.1093/ntr/ntaa175	No	Not meeting inclusion criteria	Title and abstract screening
3055	Maghsoudi-Ganjeh M, Mariano CA, Sattari S, Arora H, Eskandari M. Developing a Lung Model in the Age of COVID-19: A Digital Image Correlation and Inverse Finite Element Analysis Framework. Front Bioeng Biotechnol. 2021;9: 684778. doi:10.3389/fbioe.2021.684778	No	Not meeting inclusion criteria	Title and abstract screening

3056	Maglalang DD, Hu Y, Baslock D, Daus JD, Cano M, Ahluwalia JS. Recency of Cannabis Vaping in Sexual Minorities in Wave 5 of the Population Assessment of Tobacco and Health (PATH) Study. Subst Use Misuse. 2023; 1–7. doi:10.1080/10826084.2023.2262024	No	Not meeting inclusion criteria	Title and abstract screening
3057	Maglalang DD, Hu Y, Baslock D, Daus JD, Cano M, Ahluwalia JS. Recency of Cannabis Vaping in Sexual Minorities in Wave 5 of the Population Assessment of Tobacco and Health (PATH) Study. Subst Use Misuse. 2024;59: 136–142. doi:10.1080/10826084.2023.2262024	No	Not meeting inclusion criteria	Title and abstract screening
3058	Maglia M, Caponnetto P, Polosa R, Russo C, Santisi G. Impact of a soft tip nicotine-free harmless cigarette as part of a smoking cessation program with psychological support and varenicline: an integrated workplace smoking cessation intervention. Health Psychol Res. 2021;9: 24506. doi:10.52965/001c.24506	No	Not meeting inclusion criteria	Title and abstract screening
3059	Mahabee-Gittens E.M., Merianos A.L., Dexheimer J.W., Meyers G.T., Stone L., Tabangin M., et al. Utilization of a clinical decision support tool to reduce child tobacco smoke exposure in the urgent care setting. Pediatr Emerg Care. 2020;36: 527–531. doi:10.1097/PEC.0000000000001646	No	Not meeting inclusion criteria	Title and abstract screening
3060	Maharaj N, Swarath S, Seecheran R, Seecheran V, Seecheran NA. Suspected Cannabis Vaping-Induced Pericardial Effusion. J Investig Med High Impact Case Rep. 2022;10: 23247096221140251. doi:10.1177/23247096221140251	No	Not meeting inclusion criteria	Title and abstract screening
3061	Mahfooz K, Vasavada AM, Joshi A, Pichuthirumalai S, Andani R, Rajotia A, et al. Waterpipe Use and Its Cardiovascular Effects: A Systematic Review and Meta-Analysis of Case-Control, Cross-Sectional, and Non-Randomized Studies. Cureus. 2023;15: e34802. doi:10.7759/cureus.34802	No	Not meeting inclusion criteria	Title and abstract screening
3062	Mahmood SN, Kelemen A. Bronchiectasis, microplastics, and end of life. Chest Physician. 2022;17: 17–17.	No	Not meeting inclusion criteria	Title and abstract screening
3063	Maia-Silva KM, Zamel N, Selby P, Fontes CJF, Santos UP. Tobacco smoking associated with adverse childhood experiences in a Brazilian community university sample: A case-control study. Child Youth Serv Rev. 2021;120. doi:10.1016/j.childyouth.2020.105438https://dx.doi.org/10.1016/j.childyouth.2020.105438	No	Not meeting inclusion criteria	Title and abstract screening
3064	Maier LJ, Ramo DE, Kaur M, Meacham MC, Satre DD. Factors associated with readiness to quit smoking among young adults enrolled in a Facebook-based tobacco and alcohol intervention study. Addict Behav. 2020;111: 106524. doi:10.1016/j.addbeh.2020.106524	No	Not meeting inclusion criteria	Title and abstract screening
3065	Majeed BA, Weaver SR, Gregory KR, Whitney CF, Slovic P, Pechacek TF, et al. Changing Perceptions of Harm of E-Cigarettes Among U.S. Adults, 2012-2015. Am J Prev Med. 2017;52: 331–338. doi:10.1016/j.amepre.2016.08.039	No	Not meeting inclusion criteria	Title and abstract screening
3066	Majeed M., Lewis G., Olang C., Lake S., Gorantla V. The Paradoxical Relationship Between Nicotine and SARS-CoV-2 Infection: A Systematic Review. Int J Res Pharm Sci. 2022;13: 92–101. doi:10.26452/ijrps.v13i1.26	No	Not meeting inclusion criteria	Title and abstract screening
3067	Majmundar A, Allem JP, Cruz TB, Unger JB, Pentz MA. Twitter Surveillance at the Intersection of the Triangulum. Nicotine Tob Res. 2022;24: 118–124. doi:10.1093/ntr/ntab085	No	Not meeting inclusion criteria	Title and abstract screening
3068	Majmundar A, Allem JP, Unger JB, Cruz TB. Vaping and COVID-19: Insights for Public Health and Clinical Care from Twitter. Int J Env Res Public Health. 2021;18. doi:10.3390/ijerph182111231	No	Not meeting inclusion criteria	Title and abstract screening
3069	Majmundar A, Okitondo C, Xue A, Asare S, Bandi P, Nargis N. Nicotine Pouch Sales Trends in the US by Volume and Nicotine Concentration Levels From 2019 to 2022. JAMA Netw Open. 2022;5: e2242235. doi:10.1001/jamanetworkopen.2022.42235	No	Not meeting inclusion criteria	Title and abstract screening
3070	Majmundar A, Pérez C, Huerta M, Unger JB, Allem JP. Describing Memes Referencing Vaping: Thematic Analysis. Subst Use Misuse. 2023;58: 306–310. doi:10.1080/10826084.2022.2161316	No	Not meeting inclusion criteria	Title and abstract screening
3071	Majmundar A, Xue Z, Asare S, Nargis N. Trends in public interest in shopping and point-of-sales of JUUL and Puff Bar 2019-2021. Tob Control. 2022. doi:10.1136/tobaccocontrol-2021-056953	No	Not meeting inclusion criteria	Title and abstract screening
3072	Majmundar A, Xue Z, Asare S, Nargis N. Trends in public interest in shopping and point-of-sales of JUUL and Puff Bar 2019-2021. Tob Control. 2023;32: e236–e242. doi:10.1136/tobaccocontrol-2021-056953	No	Not meeting inclusion criteria	Title and abstract screening
3073	Majmundar A., Chou C.-P., Cruz T.B., Unger J.B. Relationship between social media engagement and e-cigarette policy support. Addict Behav Rep. 2019;9: 100155. doi:10.1016/j.abrep.2018.100155	No	Not meeting inclusion criteria	Title and abstract screening
3074	Majmundar A. Tobacco and marijuana surveillance using Twitter data. Diss Abstr Int Sect B Sci Eng. 2021;82: No-Specified.	No	Not meeting inclusion criteria	Title and abstract screening
3075	Makadia LD, Roper PJ, Andrews JO, Tingen MS. Tobacco Use and Smoke Exposure in Children: New Trends, Harm, and Strategies to Improve Health Outcomes. Curr Allergy Asthma Rep. 2017;17: 55. doi:10.1007/s11882-017-0723-0	No	Not meeting inclusion criteria	Title and abstract screening
3076	Malhotra A.M. What's hot that the other lot got. Thorax. 2019;74: 722–723. doi:10.1136/thoraxjnl-2019-213600	No	Not meeting inclusion criteria	Title and abstract screening
3077	Malhotra C, Bundoc F, Chaudhry I, Teo I, Ozdemir S, Finkelstein E, et al. A prospective cohort study assessing aggressive interventions at the end-of-life among patients with solid metastatic cancer. BMC Palliat Care. 2022;21: 1–10. doi:10.1186/s12904-022-00970-z	No	Not meeting inclusion criteria	Title and abstract screening
3078	Malibary NH, Ezzat MA, Mogharbel AM, Kouzaba KA, Alkadi AA, Malki UH, et al. Factors Affecting Ulcerative Colitis Flare-Ups: Associations With Smoking Habits and Other Patient Characteristics. Cureus. 2021;13: e19834. doi:10.7759/cureus.19834	No	Not meeting inclusion criteria	Title and abstract screening
3079	Malik A, Khan MI, Karbasian H, Nieminen M, Ammad-Ud-Din M, Khan SA. Modeling Public Sentiments About JUUL Flavors on Twitter Through Machine Learning. Nicotine Tob Res Off J Soc Res Nicotine Tob. 2021;23: 1869–1879. doi:10.1093/ntr/ntab098	No	Not meeting inclusion criteria	Title and abstract screening
3080	Malinowska B., Baranowska-kuczko M., Kicman A., Schlicker E. Opportunities, challenges and pitfalls of using cannabidiol as an adjuvant drug in covid-19. Int J Mol Sci. 2021;22: 1–40. doi:10.3390/ijms22041986	No	Not meeting inclusion criteria	Title and abstract screening
3081	Maloney SF, Combs M, Scholtes RL, Underwood M, Kilgalen B, Soule EK, et al. Impacts of COVID-19 on cigarette use, smoking behaviors, and tobacco purchasing behaviors. Drug Alcohol Depend. 2021;229: 109144. doi:10.1016/j.drugalcdep.2021.109144	No	Not meeting inclusion criteria	Title and abstract screening
3082	Maltby S., Frank M.G. Acute rhabdomyolysis: A non-pulmonary vapingrelated toxicity? J Gen Intern Med. 2020;35: S360. doi:10.1007/s11606-020-05890-3	No	Not meeting inclusion criteria	Title and abstract screening
3083	Mamat R, Rashid RA, Shin SM, Ibrahim B, Wahab S, Ahmad A. Prevalence of psilocybin use in vaping and associated factors: a study among amphetamine-type stimulants (ATS) use disorder in Malaysia. J Addict Dis. 2023; 1–13. doi:10.1080/10550887.2023.2240932	No	Not meeting inclusion criteria	Title and abstract screening
3084	Mamudu H., Wang L., Owusu D., Robertson C., Collins C., Littleton M. Prospective study of dual use of e-cigarettes and other tobacco products among school-going youth in rural Appalachian Tennessee. Ann Thorac Med. 2019;14: 127–133. doi:10.4103/atm.ATM_217_18	No	Not meeting inclusion criteria	Title and abstract screening

3085	Mamudu HM, Adzrago D, Dada O, Odame EA, Ahuja M, Awasthi M, et al. Examining Disparities in Current E-Cigarette Use among U.S. Adults before and after the WHO Declaration of the COVID-19 Pandemic in March 2020. Int J Env Res Public Health. 2023;20. doi:10.3390/ijerph20095649	No	Not meeting inclusion criteria	Title and abstract screening
3086	Manandhar A, Haron MH, Ross SA, Klein ML, Elokely KM. Potential Pro-Inflammatory Effect of Vitamin E Analogs through Mitigation of Tetrahydrocannabinol (THC) Binding to the Cannabinoid 2 Receptor. Int J Mol Sci. 2022;23. doi:10.3390/ijms23084291	No	Not meeting inclusion criteria	Title and abstract screening
3087	Mancuso S, Bhalerao A, Cucullo L. Use of Conventional Cigarette Smoking and E-Cigarette Vaping for Experimental Stroke Studies in Mice. Methods Mol Biol. 2023;2616: 441–451. doi:10.1007/978-1-0716-2926-0_31	No	Not meeting inclusion criteria	Title and abstract screening
3088	Mandal A, Singh P, Samaddar A, Singh D, Verma M, Rakesh A, et al. Vaccination of cancer patients against COVID-19: towards the end of a dilemma. Med Oncol. 2021;38: 1–8. doi:10.1007/s12032-021-01540-8	No	Not meeting inclusion criteria	Title and abstract screening
3089	Manglallan KS, Johnson DL, Rosario MH, An KJ, Carson AB, Phillips KT, et al. A Systematic Literature Review of Asian American, Native Hawaiian, and Pacific Islander Youth Electronic Nicotine Delivery Systems (ENDS) Use. Hawaii J Health Soc Welf. 2023;82: 151–157.	No	Not meeting inclusion criteria	Title and abstract screening
3090	Manning K, Garey L, Viana AG, Smit T, Zvolensky MJ. The moderating role of anxiety sensitivity in terms of fatigue severity and e-cigarette use expectancies. J Health Psychol. 2021;26: 2676–2687. doi:10.1177/1359105320926534	No	Not meeting inclusion criteria	Title and abstract screening
3091	Mantey D.S., Clendennen S.I., Sumbe A., Wilkinson A.V., Harrell M.B. Perceived stress and E-cigarette use during emerging adulthood: A longitudinal examination of initiation, progression, and continuation. Prev Med. 2022;160: 107080. doi:10.1016/j.ypmed.2022.107080	No	Not meeting inclusion criteria	Title and abstract screening
3092	Mantey DS, Chido-Amajuoyi OG, Omega-Njemnobi O, Montgomery L. Cigarette smoking frequency, quantity, dependence, and quit intentions during adolescence: Comparison of menthol and non-menthol smokers (National Youth Tobacco Survey 2017-2020). Addict Behav. 2021;121: 106986. doi:10.1016/j.addbeh.2021.106986	No	Not meeting inclusion criteria	Title and abstract screening
3093	Mantey DS, Cooper MR, Loukas A, Perry CL. E-cigarette Use and Cigarette Smoking Cessation among Texas College Students. Am J Health Behav. 2017;41: 750–759. doi:10.5993/AJHB.41.6.9	No	Not meeting inclusion criteria	Title and abstract screening
3094	Mantey DS, Omega-Njemnobi O, Ruiz FA, Chen B, Springer AE, Kelder SH. Remote Versus In-Person Learning During COVID-19: Comparison of E-Cigarette Susceptibility and Ever Use Among a Diverse Cohort of 6th-Grade Students in Texas. Nicotine Tob Res. 2023;25: 254–260. doi:10.1093/ntr/ntac084	No	Not meeting inclusion criteria	Title and abstract screening
3095	Mantey DS, Pasch KE, Loukas A, Perry CL. Exposure to Point-of-Sale Marketing of Cigarettes and E-Cigarettes as Predictors of Smoking Cessation Behaviors. Nicotine Tob Res. 2019;21: 212–219. doi:10.1093/ntr/ntx246	No	Not meeting inclusion criteria	Title and abstract screening
3096	Mantz CA, Yashar CM, Bajaj GK, Sandler HM. Recent Trends in Medicare Payments for Outpatient Cancer Care at the End of Life. Int J Radiat Oncol Biol Phys. 2022;114: S101–S102. doi:10.1016/j.ijrobp.2022.07.525	No	Not meeting inclusion criteria	Title and abstract screening
3097	Mantz CA, Yashar CM, Bajaj GK, Sandler HM. Recent Trends in Medicare Payments for Outpatient Cancer Care at the End of Life. Int J Radiat Oncol Biol Phys. 2023;116: 729–735. doi:10.1016/j.ijrobp.2023.01.005	No	Not meeting inclusion criteria	Title and abstract screening
3098	Manyanga J. Electronic cigarettes: Implications on cancer risk and cisplatin resistance in oral epithelial cells. Diss Abstr Int Sect B Sci Eng. 2021;82: No-Specified.	No	Not meeting inclusion criteria	Title and abstract screening
3099	Manyanga J, Ganapathy V, Bouharati C, Mehta T, Sadhasivam B, Acharya P, et al. Electronic cigarette aerosols alter the expression of cisplatin transporters and increase drug resistance in oral cancer cells. Sci Rep. 2021;11: 1821. doi:10.1038/s41598-021-81148-0	No	Not meeting inclusion criteria	Title and abstract screening
3100	Manz CR, Zhang Y, Chen K, Long Q, Small DS, Evans CN, et al. Long-term Effect of Machine Learning–Triggered Behavioral Nudges on Serious Illness Conversations and End-of-Life Outcomes Among Patients With Cancer: A Randomized Clinical Trial. JAMA Oncol. 2023;9: 414–418. doi:10.1001/jamaoncol.2022.6303	No	Not meeting inclusion criteria	Title and abstract screening
3101	Mao Z, Aglago EK, Zhao Z, Schalkwijk C, Jiao L, Freisling H, et al. Dietary Intake of Advanced Glycation End Products (AGEs) and Mortality among Individuals with Colorectal Cancer. Nutrients. 2021;13: 4435–4435. doi:10.3390/nu13124435	No	Not meeting inclusion criteria	Title and abstract screening
3102	Marbin J., Balk S.J., Gribben V., Groner J. Health disparities in tobacco use and exposure: A structural competency approach. Pediatrics. 2021;147: e2020040253. doi:10.1542/PEDS.2020-040253	No	Not meeting inclusion criteria	Title and abstract screening
3103	Marcham CL, Springston JP. Electronic cigarettes in the indoor environment. Rev Env Health. 2019;34: 105–124. doi:10.1515/reveh-2019-0012	No	Not meeting inclusion criteria	Title and abstract screening
3104	Marongiu F, Bertozzi N, Sibilio A, Tognali D, Mingozzi M, Curcio A. The First Use of Human-Derived ADM in Prepectoral Direct-to-Implant Breast Reconstruction after Skin-Reducing Mastectomy. Aesthetic Plast Surg. 2021;45: 2048–2057. doi:10.1007/s00266-021-02231-5	No	Not meeting inclusion criteria	Title and abstract screening
3105	Marques P, Piqueras L, Sanz M-J. An updated overview of e-cigarette impact on human health. Respir Res. 2021;22: 151. doi:10.1186/s12931-021-01737-5	No	Not meeting inclusion criteria	Title and abstract screening
3106	Marrocco A, Singh D, Christiani DC, Demokritou P. E-Cigarette (E-Cig) Liquid Composition and Operational Voltage Define the In Vitro Toxicity of Δ8Tetrahydrocannabinol/Vitamin E Acetate (Δ8THC/VEA) E-Cig Aerosols. Toxicol Sci. 2022;187: 279–297. doi:10.1093/toxsci/kfac047	No	Not meeting inclusion criteria	Title and abstract screening
3107	Marrocco A, Singh D, Christiani DC, Demokritou P. E-cigarette vaping associated acute lung injury (EVALI): state of science and future research needs. Crit Rev Toxicol. 2022;52: 188–220. doi:10.1080/10408444.2022.2082918	No	Not meeting inclusion criteria	Title and abstract screening
3108	Marti-Aguado D., Clemente-Sanchez A., Bataller R. Cigarette smoking and liver diseases. J Hepatol. 2022;77: 191–205. doi:10.1016/j.jhep.2022.01.016	No	Not meeting inclusion criteria	Title and abstract screening
3109	Martin T, Green M, Whitehead M, Scheett T, Webster M, Hudson G. Six weeks of oral Echinacea purpurea supplementation does not enhance the production of serum erythropoietin or erythropoietic status in recreationally active males with above-average aerobic fitness. 2019;44: 791-795. doi:10.1139/apnm-2018-0783	No	Not meeting inclusion criteria	Title and abstract screening
3110	Martinasek M, Tamulevicius N, Gibson-Young L, McDaniel J, Moss SJ, Pfeffer I, et al. Predictors of Vaping Behavior Change in Young Adults Using the Transtheoretical Model: A Multi-Country Study. Tob Use Insights. 2021;14: 1179173X20988672. doi:10.1177/1179173X20988672	No	Not meeting inclusion criteria	Title and abstract screening
3111	Martínez C, Baena A, Castellano Y, Fu M, Margalef M, Tigova O, et al. Prevalence and determinants of tobacco, e-cigarettes, and cannabis use among nursing students: A multicenter cross-sectional study. Nurse Educ Today. 2019;74: 61–68. doi:10.1016/j.nedt.2018.11.018	No	Not meeting inclusion criteria	Title and abstract screening
3112	Martinez E.B.D.H., Pinedo A.R. NEW PARADIGM OF SMOKING IN YOUNG PEOPLE. Rev Patol Respir. 2022;25: 9–14. doi:10.54701/rpr.2022.01.03	No	Not meeting inclusion criteria	Title and abstract screening
3113	Martinez J.E., Kahana D.D., Ghuman S., Wilson H.P., Wilson J., Kim S.C.J., et al. Unhealthy Lifestyle and Gut Dysbiosis: A Better Understanding of the Effects of Poor Diet and Nicotine on the Intestinal Microbiome. Front Endocrinol. 2021;12: 667066. doi:10.3389/fendo.2021.667066	No	Not meeting inclusion criteria	Title and abstract screening

3114	Martínez Ú, Martínez-Loredo V, Simmons VN, Meltzer LR, Drobes DJ, Brandon KO, et al. Erratum: How Does Smoking and Nicotine Dependence Change After Onset of Vaping? A Retrospective Analysis of Dual Users. Nicotine Tob Res. 2020;22: 864. doi:10.1093/ntr/ntz078	No	Not meeting inclusion criteria	Title and abstract screening
3115	Martinez U, Simmons VN, Brandon KO, Quinn GP, Brandon TH. Examining smoking and vaping behaviors, expectancies, and cessation outcomes between bisexual and heterosexual individuals. Behav Med. 2022; 1–10. doi:10.1080/08964289.2022.2077295	No	Not meeting inclusion criteria	Title and abstract screening
3116	Martinez U, Simmons VN, Brandon KO, Quinn GP, Brandon TH. Examining smoking and vaping behaviors, expectancies, and cessation outcomes between bisexual and heterosexual individuals. Behav Med. 2023;49: 392–401. doi:10.1080/08964289.2022.2077295	No	Not meeting inclusion criteria	Title and abstract screening
3117	Martinez U, Simmons VN, Sutton SK, Drobes DJ, Meltzer LR, Brandon KO, et al. Targeted smoking cessation for dual users of combustible and electronic cigarettes: a randomised controlled trial. Lancet Public Health. 2021;6: e500–e509. doi:10.1016/S2468-2667(20)30307-8	No	Not meeting inclusion criteria	Title and abstract screening
3118	Martinez U., Martinez-Loredo V., Simmons V.N., Meltzer L.R., Drobes D.J., Brandon K.O., et al. Erratum: How does smoking and nicotine dependence change after onset of vaping? A retrospective analysis of dual users (Nicotine and Tobacco Research DOI: 10.1093/ntr/ntz043). Nicotine Tob Res. 2020;22: 864. doi:10.1093/ntr/ntz078	No	Not meeting inclusion criteria	Title and abstract screening
3119	Martinez U, Martinez-Loredo V, Simmons VN, Meltzer LR, Drobes DJ, Brandon KO, et al. "How does smoking and nicotine dependence change after onset of vaping? A retrospective analysis of dual users": Erratum. Nicotine Tob Res. 2020;22: 864. doi:10.1093/ntr/ntz078https://dx.doi.org/10.1093/ntr/ntz078	No	Not meeting inclusion criteria	Title and abstract screening
3120	Martinez-Loredo V, González-Roz A, Dawkins L, Singh D, Murphy JG, MacKillop J. Is E-cigarette Use Associated With Persistence or Discontinuation of Combustible Cigarettes? A 24-Month Longitudinal Investigation in Young Adult Binge Drinkers. Nicotine Tob Res. 2022;24: 962–969. doi:10.1093/ntr/ntac049	No	Not meeting inclusion criteria	Title and abstract screening
3121	Martinez-Morata I., Sanchez T.R., Shimbo D., Navas-Acien A. Electronic Cigarette Use and Blood Pressure Endpoints: a Systematic Review. Curr Hypertens Rep. 2021;23: 2. doi:10.1007/s11906-020-01119-0	No	Not meeting inclusion criteria	Title and abstract screening
3122	Martino S, Ondersma SJ, Forray A, Olmstead TA, Gilstad-Hayden K, Howell HB, et al. A randomized controlled trial of screening and brief interventions for substance misuse in reproductive health. Am J Obstet Gynecol. 2018;218: 322.e1-322.e12. doi:10.1016/j.ajog.2017.12.005	No	Not meeting inclusion criteria	Title and abstract screening
3123	Martins BNFL, Normando AGC, Rodrigues-Fernandes CI, Wagner VP, Kowalski LP, Marques SS, et al. Global frequency and epidemiological profile of electronic cigarette users: a systematic review. Oral Surg Oral Med Oral Pathol Oral Radiol. 2022. doi:10.1016/j.oooo.2022.07.019	No	Not meeting inclusion criteria	Title and abstract screening
3124	Martire K.A., Clare P., Courtney R.J., Bonevski B., Boland V., Borland R., et al. Smoking and finances: Baseline characteristics of low income daily smokers in the FISCALS cohort. Int J Equity Health. 2017;16: 157. doi:10.1186/s12939-017-0643-6	No	Not meeting inclusion criteria	Title and abstract screening
3125	Marynak KL, Moran M. Ad Watch: "Unstoppable" VUSE Alto TV Spot Closely Mirrors "Nicotine Addiction Checks" on TikTok. Tob Control. 2021;30: e60–e61. doi:10.1136/tobaccocontrol-2020-055936	No	Not meeting inclusion criteria	Title and abstract screening
3126	Marynak KL, Robichaud MO, Puryear T, Kennedy RD, Moran MB. #Nicotineaddiction on TikTok: A quantitative content analysis of top-viewed posts. Tob Induc Dis. 2022;20: 69. doi:10.18332/tid/151868	No	Not meeting inclusion criteria	Title and abstract screening
3127	Masaki K, Taketa RM, Nakama MK, Kawamoto CT, Pokhrel P. Relationships Between Depressive Symptoms, Anxiety, Impulsivity and Cigarette and E-cigarette Use Among Young Adults. Hawaii J Health Soc Welf. 2022;81: 51–57.	No	Not meeting inclusion criteria	Title and abstract screening
3128	Mason TB, Tackett AP, Smith CE, Leventhal AM. Tobacco product use for weight control as an eating disorder behavior: Recommendations for future clinical and public health research. Int J Eat Disord. 2022;55: 313–317. doi:10.1002/eat.23651	No	Not meeting inclusion criteria	Title and abstract screening
3129	Mason TB, Tackett AP, Leventhal AM. Indirect Effects of Body Mass Index and Sweet Taste Responsiveness on E-Cigarette Dependence: The Role of E-Cigarette Motives. Subst Use Misuse. 2023; 1–8. doi:10.1080/10826084.2023.2294962	No	Not meeting inclusion criteria	Title and abstract screening
3130	Masone M.C. E-cigarettes and erectile dysfunction. Nat Rev Urol. 2022;19: 67. doi:10.1038/s41585-022-00567-4	No	Not meeting inclusion criteria	Title and abstract screening
3131	Massey M., Barney J. Pulmonary actinomycosis and marijuana vaping. BMJ Case Rep. 2021;14: e240973. doi:10.1136/bcr-2020-240973	No	Not meeting inclusion criteria	Title and abstract screening
3132	Massey ZB, Duong HT, Churchill V, Popova L. Examining reactions to smoking and COVID-19 risk messages: An experimental study with people who smoke. Int J Drug Policy. 2022;102: 103607. doi:10.1016/j.drugpo.2022.103607	No	Not meeting inclusion criteria	Title and abstract screening
3133	Masso Silva J.A., Moshensky A., Brand C., Alhaddad H., Shin J., Almarghalani D., et al. Multi-organ inflammatory effects by the chronic use of the pod-based electronic cigarette juultmin mice. Am J Respir Crit Care Med. 2021;203. doi:10.1164/ajrccm-conference.2021.203.1_MeetingAbstracts.A3100	No	Not meeting inclusion criteria	Title and abstract screening
3134	Masso-Silva J.A., Crotty Alexander L.E. Just When We Thought Nothing Could Be Worse Than Smoking Tobacco, Vaping e-Hookah Proves Us Wrong. Chest. 2022;161: 13–15. doi:10.1016/j.chest.2021.08.042	No	Not meeting inclusion criteria	Title and abstract screening
3135	Masson CL, Le T, Hosakote S, Fokuo JK, Gubner NR, Shingle M, et al. Correlates of e-cigarette use for smoking cessation among clients in residential substance use disorder treatment. Addict Behav. 2021;119: 106947. doi:10.1016/j.addbeh.2021.106947	No	Not meeting inclusion criteria	Title and abstract screening
3136	Mat Salleh R, Baharom N, Siau CS, Chan CMH, Amit N, Sia PY, et al. E-Cigarette Users' Profiles and Their Association with Identified Impacts of COVID-19 on Vaping among Young Adults in Malaysia. Healthc Basel Switz. 2023;11. doi:10.3390/healthcare11030434	No	Not meeting inclusion criteria	Title and abstract screening
3137	Matak L, Mikuš M, Ćorić M, Spagnol G, Matak M, Vujić G. Comparison end-to-end anastomosis with ostomy after secondary surgical cytoreduction for recurrent high-grade serous ovarian cancer: observational single-center study. Arch Gynecol Obstet. 2023;308: 231–237. doi:10.1007/s00404-023-06918-9	No	Not meeting inclusion criteria	Title and abstract screening
3138	Mathew AR, Zhou M. Distress tolerance in relation to cessation history and smoking characteristics among adult daily smokers. Addict Behav. 2020;100: 106124. doi:10.1016/j.addbeh.2019.106124	No	Not meeting inclusion criteria	Title and abstract screening
3139	Mathews M., Lipsky M., Thao B., Su S., Hung M. Differences Between Male and Female E-cigarettes Users' Perception of Health. FASEB J. 2021;35. doi:10.1096/fasebj.2021.35.S1.04899	No	Not meeting inclusion criteria	Title and abstract screening
3140	Mathur Gaiha S, Halpern-Felsher B, Feld AL, Gaber J, Rogers T, Henriksen L. JUUL and other e-cigarettes: Socio-demographic factors associated with use and susceptibility in California. Prev Med Rep. 2021;23: 101457. doi:10.1016/j.pmedr.2021.101457	No	Not meeting inclusion criteria	Title and abstract screening
3141	Matos C.P., Boleo-Tome J.P., Rosa P., Morais A. Tobacco and COVID-19: A position from Sociedade Portuguesa de Pneumologia. Pulmonology. 2021;27: 91–93. doi:10.1016/j.pulmoe.2020.11.002	No	Not meeting inclusion criteria	Title and abstract screening
3142	Matos M.J.R., Rosa M.E.E., Brito V.M., Amaral L.T.W., Beraldo G.L., Fonseca E.K.U.N., et al. Differential diagnoses of acute ground-glass opacity in chest computed tomography: pictorial essay. Einstein Sao Paulo Braz. 2021;19: eRW5772. doi:10.31744/einstein_journal/2021RW5772	No	Not meeting inclusion criteria	Title and abstract screening

3143	Matsuyama Y, Tabuchi T. Heated tobacco product use and combustible cigarette smoking relapse/initiation among former/never smokers in Japan: the JASTIS 2019 study with 1-year follow-up. Tob Control. 2022;31: 520–526. doi:10.1136/tobaccocontrol-2020-056168	No	Not meeting inclusion criteria	Title and abstract screening
3144	Mattapally S., Antony V.B. Computational modeling of aldehyde dehydrogenase 2 (aldh2) a marker for damaged mitochondria in extracellular vesicles of evali. Am J Respir Crit Care Med. 2021;203. doi:10.1164/ajrccm-conference.2021.203.1_MeetingAbstracts.A3626	No	Not meeting inclusion criteria	Title and abstract screening
3145	Mattingly D.T., Mezuk B., Elliott M.R., Fleischer N.L. Discrimination and Tobacco Use Outcomes Among US Adults: Effect Modification by Race/Ethnicity. J Racial Ethn Health Disparities. 2023. doi:10.1007/s40615-023-01527-3	No	Not meeting inclusion criteria	Title and abstract screening
3146	Mattingly DT, Hart JL, Vu T-HT, Walker KL. Appalachian youth e-cigarette harm perceptions and tobacco use. Popul Med. 2021;3. doi:10.18332/popmed/132120	No	Not meeting inclusion criteria	Title and abstract screening
3147	Mattingly DT, Neighbors HW, Mezuk B, Elliott MR, Fleischer NL. Racial/ethnic discrimination and tobacco and cannabis use outcomes among US adults. J Subst Use Addict Treat. 2023;148: 208958. doi:10.1016/j.josat.2023.208958	No	Not meeting inclusion criteria	Title and abstract screening
3148	Mattingly DT, Tompkins LK, Rai J, Sears CG, Walker KL, Hart JL. Tobacco use and harm perceptions among Appalachian youth. Prev Med Rep. 2020;18: 101089. doi:10.1016/j.pmedr.2020.101089	No	Not meeting inclusion criteria	Title and abstract screening
3149	Mattingly DT, Zavala-Arciniega L, Hirschtick JL, Meza R, Levy DT, Fleischer NL. Trends in Exclusive, Dual and Polytobacco Use among U.S. Adults, 2014-2019: Results from Two Nationally Representative Surveys. Int J Env Res Public Health. 2021;18. doi:10.3390/ijerph182413092	No	Not meeting inclusion criteria	Title and abstract screening
3150	Mattsson TO, Pottegård A, Jørgensen TL, Green A, Bliddal M. End-of-life anticancer treatment – a nationwide registry-based study of trends in the use of chemo-, endocrine, immune-, and targeted therapies. Acta Oncol. 2021;60: 961–967. doi:10.1080/0284186X.2021.1890332	No	Not meeting inclusion criteria	Title and abstract screening
3151	Maurer JJ, Sandager-Nielsen K, Schmidt HD. Attenuation of nicotine taking and seeking in rats by the stoichiometry-selective alpha4beta2 nicotinic acetylcholine receptor positive allosteric modulator NS9283. Psychopharmacology (Berl). 2017;234: 475–484. doi:10.1007/s00213-016-4475-7	No	Not meeting inclusion criteria	Title and abstract screening
3152	Mayer M, Shin YE, Baker L, Cordova J, Mayne RG, Reyes-Guzman CM, et al. A Longitudinal Analysis of Respiratory Illness and Tobacco Use Transitions. Am J Prev Med. 2022. doi:10.1016/j.amepre.2022.08.010	No	Not meeting inclusion criteria	Title and abstract screening
3153	Mayer M.E., Kong G., Barrington-Trimis J.L., McConnell R., Leventhal A.M., Krishnan-Sarin S. Blunt and Non-Blunt Cannabis Use and Risk of Subsequent Combustible Tobacco Product Use among Adolescents. Nicotine Tob Res. 2020;22: 1409–1413. doi:10.1093/ntr/ntz225	No	Not meeting inclusion criteria	Title and abstract screening
3154	Mayer M, Shin YE, Baker L, Cordova J, Mayne RG, Reyes-Guzman CM, et al. A longitudinal analysis of respiratory illness and tobacco use transitions. Am J Prev Med. 2023;64: 175–183. doi:10.1016/j.amepre.2022.08.010	No	Not meeting inclusion criteria	Title and abstract screening
3155	Mayet A, Lavagna C. Electronic cigarettes: harm reduction tool or new substance use behavior? Addiction. 2018;113: 1786–1788. doi:10.1111/add.14220	No	Not meeting inclusion criteria	Title and abstract screening
3156	Mayor S. E-cigarettes users have lower carcinogen and toxin levels than smokers, study finds. BMJ Online. 2017;356: j651. doi:10.1136/bmj.j651	No	Not meeting inclusion criteria	Title and abstract screening
3157	Mayorga NA, Garey L, Nizio P, Buckner JD, Zvolensky MJ. The Effects of Cannabis Use: A Test Among Dual Electronic and Combustible Cigarette Users. Am J Addict. 2020;29: 287–294. doi:10.1111/ajad.13021	No	Not meeting inclusion criteria	Title and abstract screening
3158	Mayorga NA, Rogers AH, Smit T, Garey L, Zvolensky MJ. Evaluating the psychometric properties of the e-cigarette barriers to cessation scale. Cogn Behav Ther. 2021;50: 492–508. doi:10.1080/16506073.2021.1901773	No	Not meeting inclusion criteria	Title and abstract screening
3159	Mayorga NA, Smit T, Shepherd JM, Orr MF, Garey L, Zvolensky MJ. Worry and e-cigarette cognition: The moderating role of sex. Addict Behav. 2021;112: 106621. doi:10.1016/j.addbeh.2020.106621	No	Not meeting inclusion criteria	Title and abstract screening
3160	Mays D, Long L, Alalwan MA, Wagener TL, Shang C, Roberts ME, et al. The Effects of Oral Nicotine Pouch Packaging Features on Adult Tobacco Users' and Non-Users' Product Perceptions. Int J Env Res Public Health. 2023;20. doi:10.3390/ijerph20043383	No	Not meeting inclusion criteria	Title and abstract screening
3161	Mays D, Villanti A, Niaura R, Lindblom E, Strasser A. The Effects of Varying Electronic Cigarette Warning Label Design Features On Attention, Recall, and Product Perceptions Among Young Adults. 2019;34: 317-324. doi:10.1080/10410236.2017.1372050	No	Not meeting inclusion criteria	Title and abstract screening
3162	Mazzilli KM, Bover Manderski MT, Delnevo CD, Hrywna M. A Pilot Feasibility Study of an Online Youth Tobacco Survey Administration among High School Students. Int J Env Res Public Health. 2022;19. doi:10.3390/ijerph19169911	No	Not meeting inclusion criteria	Title and abstract screening
3163	McAlinden K., Lu W., Ferdowsi P.V., Myers S., Markos J., Chia C., et al. Electronic cigarette aerosol condensate is cytotoxic and increases ACE2 expression in human airway epithelial cells: implications for COVID-19. Eur Respir J. 2021;58. doi:10.1183/13993003.congress-2021.PA2394	No	Not meeting inclusion criteria	Title and abstract screening
3164	McAlinden K.D., Lu W., Ferdowsi P.V., Myers S., Markos J., Larby J., et al. Electronic Cigarette Aerosol Condensate Increases ACE2 Expression on Human Airway Epithelial Cells: Implications for SARS-CoV-2 (COVID-19). Am J Respir Crit Care Med. 2021;203. doi:10.1164/ajrccm-conference.2021.TP105	No	Not meeting inclusion criteria	Title and abstract screening
3165	McAlinden KD, Lu W, Eapen MS, Sohal SS. Electronic cigarettes: Modern instruments for toxic lung delivery and posing risk for the development of chronic disease. Int J Biochem Cell Biol. 2021;137: 106039. doi:10.1016/j.biocel.2021.106039	No	Not meeting inclusion criteria	Title and abstract screening
3166	McCabe S.E., West B.T., Matthews A.K., Evans-Polce R., Lee J.G.L., Hughes T.L., et al. Sexual Orientation, Tobacco Use, and Tobacco Cessation Treatment-Seeking: Results From a National U.S. Survey. Behav Med. 2021;47: 120–130. doi:10.1080/08964289.2019.1676191	No	Not meeting inclusion criteria	Title and abstract screening
3167	McCabe SE, Matthews AK, Lee JGL, Veliz P, Hughes TL, Boyd CJ. Tobacco Use and Sexual Orientation in a National Cross-sectional Study: Age, Race/Ethnicity, and Sexual Identity-Attraction Differences. Am J Prev Med. 2018;54: 736–745. doi:10.1016/j.amepre.2018.03.009	No	Not meeting inclusion criteria	Title and abstract screening
3168	McCarthy M. US plan gives greater role to electronic cigarettes in tobacco harm reduction. BMJ. 2017;358: j3689. doi:10.1136/bmj.j3689	No	Not meeting inclusion criteria	Title and abstract screening
3169	McCaughan E, Semple CJ, Hanna JR. "Don't forget the children": a qualitative study when a parent is at end of life from cancer. Support Care Cancer. 2021;29: 7695–7702. doi:10.1007/s00520-021-06341-3	No	Not meeting inclusion criteria	Title and abstract screening
3170	McCauley DM, Gaiha SM, Lempert LK, Halpern-Felsher B. Adolescents, Young Adults, and Adults Continue to Use E-Cigarette Devices and Flavors Two Years after FDA Discretionary Enforcement. Int J Env Res Public Health. 2022;19. doi:10.3390/ijerph19148747	No	Not meeting inclusion criteria	Title and abstract screening
3171	McClelland M, McClelland S. Case of a 21-year-old man with persistent lung collapse leading to a pericardectomy linked to vape use. Heart Lung J Crit Care. 2021;50: 262–267. doi:10.1016/j.hrtlng.2020.12.012	No	Not meeting inclusion criteria	Title and abstract screening

3172	McClure EA. Accurate and sufficient measurement of cannabis and tobacco co-use: agreement, consensus and the path forward. Addiction. 2021;116: 1636–1637. doi:10.1111/add.15497	No	Not meeting inclusion criteria	Title and abstract screening
3173	McClure EA, Hamilton L, Schauer GL, Matson TE, Lapham GT. Cannabis and nicotine co-use among primary care patients in a state with legal cannabis access. Addict Behav. 2023;140: 107621. doi:10.1016/j.addbeh.2023.107621	No	Not meeting inclusion criteria	Title and abstract screening
3174	McCormack J., Walker N., McRobbie H., Wright K., Nosa V., Fernandes B., et al. Revised Guidelines for smoking cessation in New Zealand, 2021. N Z Med J. 2022;135: 54–64.	No	Not meeting inclusion criteria	Title and abstract screening
3175	McCracken BA, VanPutte CL, Hildebolt CF. Nicotine-related misperceptions among faculty and students at a Midwestern dental school. J Dent Educ. 2022;86: 161–168. doi:10.1002/jdd.12788	No	Not meeting inclusion criteria	Title and abstract screening
3176	McCrimmon R.J., Palmer K., Alsaleh A.J.O., Lew E., Puttanna A. Cost-Effectiveness of iGlarLixi Versus Premix BIAsp 30 in Patients with Type 2 Diabetes Suboptimally Controlled by Basal Insulin in the UK. Diabetes Ther. 2022;13: 1203–1214. doi:10.1007/s13300-022-01267-3	No	Not meeting inclusion criteria	Title and abstract screening
3177	McDermott CL, Curtis JR, Sun Q, Fedorenko C, Kreizenbeck K, Ramsey SD. Polypharmacy, chemotherapy receipt, and medication-related out-of-pocket costs at end of life among commercially insured adults with advanced cancer. J Oncol Pharm Pract. 2022;28: 836–841. doi:10.1177/10781552211006180	No	Not meeting inclusion criteria	Title and abstract screening
3178	McDermott MS, East KA, Brose LS, McNeill A, Hitchman SC, Partos TR. The effectiveness of using e-cigarettes for quitting smoking compared to other cessation methods among adults in the United Kingdom. Addiction. 2021;116: 2825–2836. doi:10.1111/add.15474	No	Not meeting inclusion criteria	Title and abstract screening
3179	McDonald C.F. E-cigarettes for smoking cessation: Current state of play. Respirology. 2019;24: 722–723. doi:10.1111/resp.13630	No	Not meeting inclusion criteria	Title and abstract screening
3180	McDonald CF. Vaping and the lung: New evidence from the silent zone?. Respirol Carlton Vic. 2021;26: 906–907. doi:10.1111/resp.14132	No	Not meeting inclusion criteria	Title and abstract screening
3181	McDonnell B.P., Dicker P., Regan C. Authors' reply re: Electronic cigarettes and obstetric outcomes: a prospective observational study. BJOG Int J Obstet Gynaecol. 2020;127: 1167. doi:10.1111/1471-0528.16293	No	Not meeting inclusion criteria	Title and abstract screening
3182	McDuff DR, Garvin M, Joy Chang, Thompson D. Substance Misuse in Elite Athletes: Early Detection, Brief Intervention and Referral to Treatment. Clin Sports Med. 2024;43: 127–144. doi:10.1016/j.csm.2023.06.008	No	Not meeting inclusion criteria	Title and abstract screening
3183	McElvaney O.J., Finnerty G., Carroll T.P., Brantly M.L., McElvaney N.G. MZ Alpha-1 Antitrypsin Deficiency. Am J Respir Crit Care Med. 2022;205: P13–P14. doi:10.1164/rccm.2057P13	No	Not meeting inclusion criteria	Title and abstract screening
3184	McEvoy MT, Seashore EL, Smith V, Cheng S, Clay S, Foster J, et al. End-of-Life Outcomes in Children with Relapsed/Refractory Solid Tumors: A Single-Center Study (Sch425). J Pain Symptom Manage. 2023;65: e592–e592. doi:10.1016/j.jpainsymman.2023.02.162	No	Not meeting inclusion criteria	Title and abstract screening
3185	McEwan M, Gale N, Ebajemito JK, Camacho OM, Hardie G, Proctor CJ, et al. A randomized controlled study in healthy participants to explore the exposure continuum when smokers switch to a tobacco heating product or an E-cigarette relative to cessation. Toxicol Rep. 2021;8: 994–1001. doi:10.1016/j.toxrep.2021.05.003	No	Not meeting inclusion criteria	Title and abstract screening
3186	McFadden DD, Bornstein SL, Vassallo R, Salonen BR, Bhuiyan MN, Schroeder DR, et al. Symptoms COVID 19 Positive Vapers Compared to COVID 19 Positive Non-vapers. J Prim Care Community Health. 2022;13: 21501319211062672. doi:10.1177/21501319211062672	No	Not meeting inclusion criteria	Title and abstract screening
3187	McFalls J., Obilom C., Martin K., Shaker K., Phillips T. Articles You Might Have Missed. J Med Toxicol. 2021;17: 437–439. doi:10.1007/s13181-021-00856-1	No	Not meeting inclusion criteria	Title and abstract screening
3188	McGee LU, Parker S, Bulsara S, Escobar B, Kline KN, Jibaja-Weiss ML, et al. Adolescent electronic cigarette counselling: knowledge, attitudes and perceived barriers among clinical staff in a primary care setting. J Eval Clin Pr. 2021;27: 1398–1402. doi:10.1111/jep.13553	No	Not meeting inclusion criteria	Title and abstract screening
3189	McGrath DS, Kim HS, Boothby CA, Romanow NK, Hodgins DC. Patterns and motives for electronic cigarette use in a sample of community-recruited gamblers. Int J Ment Health Addict. 2018;16: 276–283. doi:10.1007/s11469-016-9722-yhttps://dx.doi.org/10.1007/s11469-016-9722-y	No	Not meeting inclusion criteria	Title and abstract screening
3190	McGrath JJ, Racicot S, Okoli CTC, Hammond SK, O'Loughlin J. Airborne Nicotine, Secondhand Smoke, and Precursors to Adolescent Smoking. Pediatrics. 2018;141: S63–S74. doi:10.1542/peds.2017-1026J	No	Not meeting inclusion criteria	Title and abstract screening
3191	McGrath-Morrow SA, Gorzkowski J, Groner JA, Rule AM, Wilson K, Tanski SE, et al. The Effects of Nicotine on Development. Pediatrics. 2020;145. doi:10.1542/peds.2019-1346	No	Not meeting inclusion criteria	Title and abstract screening
3192	McGraw MD, Croft DP, Nacca NE, Rahman I. Reduced plasma phosphatidylethanolamines in e-cigarette, or vaping, product use-associated lung injury (EVALI). Pediatr Pulmonol. 2022;57: 1350–1354. doi:10.1002/ppul.25874	No	Not meeting inclusion criteria	Title and abstract screening
3193	McKelvey K, Baiocchi M, Halpern-Felsher B. Adolescents' and Young Adults' Use and Perceptions of Pod-Based Electronic Cigarettes. JAMA Netw Open. 2018;1: e183535. doi:10.1001/jamanetworkopen.2018.3535	No	Not meeting inclusion criteria	Title and abstract screening
3194	McKenzie CR, Davis J, Dunlop AJ. E-cigarette or vaping product use-associated lung injury in an adolescent. Med J Aust. 2022;216: 374. doi:10.5694/mja2.51462	No	Not meeting inclusion criteria	Title and abstract screening
3195	McKeon G, Scott JG. Smoke and mirrors: Support from psychiatrists for nicotine e-cigarette availability in Australia. Aust N Z J Psychiatry. 2023;57: 169–180. doi:10.1177/00048674221126458	No	Not meeting inclusion criteria	Title and abstract screening
3196	McKeon G, Scott JG. Smoke and mirrors: Support from psychiatrists for nicotine e-cigarette availability in Australia. Aust N Z J Psychiatry. 2022; 48674221126458. doi:10.1177/00048674221126458	No	Not meeting inclusion criteria	Title and abstract screening
3197	McLeish A.C., Hart J.L., Walker K.L. College Student E-Cigarette Users' Knowledge about E-Cigarettes: Ingredients, Health Risks, Device Modifications, and Information Sources. Int J Environ Res Public Health. 2022;19: 1962. doi:10.3390/ijerph19041962	No	Not meeting inclusion criteria	Title and abstract screening
3198	McLeish AC, Smit T, Garey L, Zvolensky MJ. Anxiety Sensitivity and Emotion Dysregulation in Dual and Exclusive E-Cigarette Users. Subst Use Misuse. 2021;56: 1825–1830. doi:10.1080/10826084.2021.1954028	No	Not meeting inclusion criteria	Title and abstract screening
3199	McLeish AC, Hart JL, Walker KL. Long-term impact of the COVID-19 pandemic on use behavior and risk perceptions of college student E-cigarette users. J Am Coll Health J ACH. 2023; 1–5. doi:10.1080/07448481.2023.2194441	No	Not meeting inclusion criteria	Title and abstract screening
3200	McLennan J.D. Wondering about the psychiatrist's role in smoking cessation from inpatients to whole populations. J Can Acad Child Adolesc Psychiatry. 2021;30: 2.	No	Not meeting inclusion criteria	Title and abstract screening

3201	McMahon I, Harris-Lane LM, Donnan J, Bishop L, Harris N. Emerging adult perceptions of higher-risk cannabis consumption behaviours. Harm Reduct J. 2023;20: 127. doi:10.1186/s12954-023-00860-4	No	Not meeting inclusion criteria	Title and abstract screening
3202	McNeill A, Driezen P, Hitchman SC, Cummings KM, Fong GT, Borland R. Indicators of cigarette smoking dependence and relapse in former smokers who vape compared with those who do not: findings from the 2016 International Tobacco Control Four Country Smoking and Vaping Survey. Addiction. 2019;114 Suppl 1: 49–60. doi:10.1111/add.14722	No	Not meeting inclusion criteria	Title and abstract screening
3203	McNeill A, Robson D. A man before his time: Russell's insights into nicotine, smoking, treatment and curbing the smoking problem. Addiction. 2018;113: 759–763. doi:10.1111/add.14043	No	Not meeting inclusion criteria	Title and abstract screening
3204	McNeill A., Driezen P., Hitchman S.C., Cummings K.M., Fong G.T., Borland R. Indicators of cigarette smoking dependence and relapse in former smokers who vape compared with those who do not: findings from the 2016 International Tobacco Control Four Country Smoking and Vaping Survey. Addict Abingdon Engl. 2019;114: 49–60. doi:10.1111/add.14722	No	Not meeting inclusion criteria	Title and abstract screening
3205	McPherson S, Howell D, Lewis J, Barbosa-Leiker C, Bertotti Metoyer P, Roll J. Self-reported smoking effects and comparative value between cigarettes and high dose e-cigarettes in nicotine-dependent cigarette smokers. 2016;27: 301-307. doi:10.1097/FBP.0000000000000226	No	Not meeting inclusion criteria	Title and abstract screening
3206	McQuoid J, Thrul J, Lopez-Paguyo K, Ling PM. Exploring multiple drug use by integrating mobile health and qualitative mapping methods - An individual case study. Int J Drug Policy. 2021;97: 103325. doi:10.1016/j.drugpo.2021.103325	No	Not meeting inclusion criteria	Title and abstract screening
3207	McRobbie H. Modelling the population health effects of e-cigarettes use: Current data can help guide future policy decisions. Nicotine Tob Res. 2017;19: 131–132. doi:10.1093/ntr/ntw387	No	Not meeting inclusion criteria	Title and abstract screening
3208	McRobbie H., Courtney R. SMOKING CESSATION AND ELECTRONIC-CIGARETTES: A SAFETY PERSPECTIVE. Aust N Z J Psychiatry. 2022;56: 17. doi:10.1177/00048674221088686	No	Not meeting inclusion criteria	Title and abstract screening
3209	McRobbie H.J., Phillips-Waller A., El Zerbi C., McNeill A., Hajek P., Pesola F., et al. Nicotine replacement treatment, e-cigarettes and an online behavioural intervention to reduce relapse in recent ex-smokers: A multinational four-arm rct. Health Technol Assess. 2020;24: 1–81. doi:10.3310/hta24680	No	Not meeting inclusion criteria	Title and abstract screening
3210	Meacham MC, Vogel EA, Thrul J, Ramo DE, Satre DD. Addressing cigarette smoking cessation treatment challenges during the COVID-19 pandemic with social media. J Subst Abuse Treat. 2021;129: 108379. doi:10.1016/j.jsat.2021.108379	No	Not meeting inclusion criteria	Title and abstract screening
3211	Mead EL, Duffy V, Oncken C, Litt MD. E-cigarette palatability in smokers as a function of flavorings, nicotine content and propylthiouracil (PROP) taster phenotype. Addict Behav. 2019;91: 37–44. doi:10.1016/j.addbeh.2018.11.014	No	Not meeting inclusion criteria	Title and abstract screening
3212	Mears MJ, Hookfin HL, Bandaru P, Vidal P, Stanford KI, Wold LE. Electronic Nicotine Delivery Systems and Cardiovascular/Cardiometabolic Health. Circ Res. 2023;132: 1168–1180. doi:10.1161/CIRCRESAHA.123.321565	No	Not meeting inclusion criteria	Title and abstract screening
3213	Medeiros GC, Lafer B, Kapczinski F, Miranda-Scippa Â, Almeida KM. Bipolar disorder and tobacco smoking: Categorical and dimensional clinical correlates in subjects from the Brazilian bipolar research network. Compr Psychiatry. 2018;82: 14–21. doi:10.1016/j.comppsych.2017.12.003	No	Not meeting inclusion criteria	Title and abstract screening
3214	Medina S., Grosowsky T. A Collaborative Community Approach to Address the Adolescent Vaping Epidemic. Pediatrics. 2020;146: 489–490. doi:10.1542/peds.146.1_MeetingAbstract.489	No	Not meeting inclusion criteria	Title and abstract screening
3215	Meece L.E., Moran J.P., McConville H.M., Velani S. Pneumonitis Due to "Dabbing" Successfully Managed via Extracorporeal Membrane Oxygenation. Am J Respir Crit Care Med. 2022;205. doi:10.1164/ajrccm-conference.2022.205.1_MeetingAbstracts.A1130	No	Not meeting inclusion criteria	Title and abstract screening
3216	Meehan-Atrash J, Korzun T, Ziegler A. Cannabis Inhalation and Voice Disorders: A Systematic Review. JAMA Otolaryngol Head Neck Surg. 2019;145: 956–964. doi:10.1001/jamaoto.2019.1986	No	Not meeting inclusion criteria	Title and abstract screening
3217	Meehan-Atrash J, Rahman I. Cannabis Vaping: Existing and Emerging Modalities, Chemistry, and Pulmonary Toxicology. Chem Res Toxicol. 2021;34: 2169–2179. doi:10.1021/acs.chemrestox.1c00290	No	Not meeting inclusion criteria	Title and abstract screening
3218	Meehan-Atrash J., Rahman I. Novel Delta-8-Tetrahydrocannabinol Vaporizers Contain Unlabeled Adulterants, Byproducts of Chemical Synthesis, and Heavy Metals. Am J Respir Crit Care Med. 2022;205. doi:10.1164/ajrccm-conference.2022.205.1_MeetingAbstracts.A1148	No	Not meeting inclusion criteria	Title and abstract screening
3219	Meehan-Atrash J, Luo W, McWhirter KJ, Dennis DG, Sarlah D, Jensen RP, et al. The influence of terpenes on the release of volatile organic compounds and active ingredients to cannabis vaping aerosols. RSC Adv. 2021;11: 11714–11723. doi:10.1039/d1ra00934f	No	Not meeting inclusion criteria	Title and abstract screening
3220	Mehta M, Teymouri M, Puthuparampil-Mehta B, Sawh C, Paty P, Kostun ZW, et al. Outcomes of the V-Healthy education and awareness program that empowers high school students to understand and diagnose vascular disease risk factors. J Vasc Surg. 2023;77: 1245–1249. doi:10.1016/j.jvs.2022.12.014	No	Not meeting inclusion criteria	Title and abstract screening
3221	Mehta M., Teymouri M., Puthuparampil-Mehta B., Sawh C., Paty P., Kostun Z.W., et al. Outcomes of the V-Healthy Education & Awareness Program that Empowers High-School Students to Understand and Diagnose Vascular Disease Risk Factors. J Vasc Surg. 2022. doi:10.1016/j.jvs.2022.12.014	No	Not meeting inclusion criteria	Title and abstract screening
3222	Meienberg A., Mayr M., Vischer A., Zellweger M., Burkard T. Nichtrauchen ist clever: A smoking prevention program for schoolchildren and what we learn from the kids. Eur J Prev Cardiol. 2021;28: i280. doi:10.1093/eurjpc/zwab061.290	No	Not meeting inclusion criteria	Title and abstract screening
3223	Meienberg A., Mayr M., Vischer A., Zellweger M.J., Burkard T. Smoking prevention in adolescents: A cross-sectional and qualitative evaluation of a newly implemented prevention program in Switzerland. BMJ Open. 2021;11: e048319. doi:10.1136/bmjopen-2020-048319	No	Not meeting inclusion criteria	Title and abstract screening
3224	Meier E, Tessier KM, Luo X, Dick L, Thomson NM, Hecht SS, et al. Cigarette Smokers Versus Cannabis Smokers Versus Co-users of Cigarettes and Cannabis: A Pilot Study Examining Exposure to Toxicants. Nicotine Tob Res. 2022;24: 125–129. doi:10.1093/ntr/ntab125	No	Not meeting inclusion criteria	Title and abstract screening
3225	Meier E, Wahlquist AE, Heckman BW, Cummings KM, Froeliger B, Carpenter MJ. A Pilot Randomized Crossover Trial of Electronic Cigarette Sampling Among Smokers. Nicotine Tob Res. 2017;19: 176–182. doi:10.1093/ntr/ntw157	No	Not meeting inclusion criteria	Title and abstract screening
3226	Meier E, Burris J, Wahlquist A, Garrett-Mayer E, Gray K, Alberg A, et al. Perceptions of Snus Among US Adult Smokers Given Free Product. 2017;20: 22-29. doi:10.1093/ntr/ntw392	No	Not meeting inclusion criteria	Title and abstract screening
3227	Meier E, Burris J, Wahlquist A, Garrett-Mayer E, Gray K, Alberg A, et al. Perceptions of Snus among US adult smokers given free product. 2018;20: 22-29. doi:10.1093/ntr/ntw392	No	Not meeting inclusion criteria	Title and abstract screening
3228	Meissner H.I., Sharma K., Mandal R.J., Garcia-Cazarin M., Wanke K.L., Moyer J., et al. NIH Tobacco Research and the Emergence of Tobacco Regulatory Science. Nicotine Tob Res. 2022;24: 463–468. doi:10.1093/ntr/ntab205	No	Not meeting inclusion criteria	Title and abstract screening
3229	Meister M.L., Feresin R.G. Blackberry consumption protects against e-cigarette-induced vascular oxidative stress in mice. Food Funct. 2023. doi:10.1039/d3fo02217j	No	Not meeting inclusion criteria	Title and abstract screening

3230	Melero-Ollonarte JL, Lidón-Moyano C, Perez-Ortuño R, Fu M, Ballbè M, Martín-Sánchez JC, et al. Specific biomarker comparison in current smokers, e-cigarette users, and non-smokers. Addict Behav. 2023;140: 107616. doi:10.1016/j.addbeh.2023.107616	No	Not meeting inclusion criteria	Title and abstract screening
3231	Melka A, Chojenta C, Holliday E, Loxton D. E-cigarette use and cigarette smoking initiation among australian women who have never smoked. Drug Alcohol Rev. 2020; No-Specified. doi:10.1111/dar.13131https://dx.doi.org/10.1111/dar.13131	No	Not meeting inclusion criteria	Title and abstract screening
3232	Melka A, Chojenta C, Holliday E, Loxton D. E-cigarette use and cigarette smoking initiation among Australian women who have never smoked. Drug Alcohol Rev. 2021;40: 68–77. doi:10.1111/dar.13131	No	Not meeting inclusion criteria	Title and abstract screening
3233	Meltzer L, Simmons V, Sutton S, Drobes D, Quinn G, Meade C, et al. A randomized controlled trial of a smoking cessation self-help intervention for dual users of tobacco cigarettes and E-cigarettes: intervention development and research design. 2017;60: 56-62. doi:10.1016/j.cct.2017.06.014	No	Not meeting inclusion criteria	Title and abstract screening
3234	Menakuru S, Inzamam Ali M. Beliefs and reality of e-cigarette smoking. BMJ Case Rep. 2018;2018. doi:10.1136/bcr-2018-225683	No	Not meeting inclusion criteria	Title and abstract screening
3235	Mencer N., Sandhu K., Uysal A. Vaping-induced pneumomediastinum and pneumopericardium. Am J Respir Crit Care Med. 2021;203. doi:10.1164/ajrccm-conference.2021.203.1_MeetingAbstracts.A3014	No	Not meeting inclusion criteria	Title and abstract screening
3236	Mendel JR, Hall MG, Baig SA, Jeong M, Brewer NT. Placing Health Warnings on E-Cigarettes: A Standardized Protocol. Int J Environ Res Public Health. 2018;15. doi:10.3390/ijerph15081578	No	Not meeting inclusion criteria	Title and abstract screening
3237	Mendelsohn C.P. Use of Nicotine Replacement Therapy in Young People Entering Custody in New South Wales, Australia. J Paediatr Child Health. 2017;53: 926–927. doi:10.1111/jpc.13611	No	Not meeting inclusion criteria	Title and abstract screening
3238	Mendelsohn C.P. The role of vaping nicotine in psychiatry practice. Australas Psychiatry. 2021;29: 5–6. doi:10.1177/1039856221989096	No	Not meeting inclusion criteria	Title and abstract screening
3239	Mendelsohn C.P., Hall W. Does the gateway theory justify a ban on nicotine vaping in Australia? Int J Drug Policy. 2020;78: 102712. doi:10.1016/j.drugpo.2020.102712	No	Not meeting inclusion criteria	Title and abstract screening
3240	Mendelsohn C. Optimal use of smoking cessation pharmacotherapy. Aust Prescr. 2022;45: 10–14. doi:10.18773/austprescr.2022.001	No	Not meeting inclusion criteria	Title and abstract screening
3241	Mendelsohn C, Hall W, Borland R. Could vaping help lower smoking rates in Australia?. Drug Alcohol Rev. 2020;39: 415–418. doi:10.1111/dar.13039	No	Not meeting inclusion criteria	Title and abstract screening
3242	Mendelsohn CP, Wodak A, Hall W. Nicotine vaping was not the cause of e-cigarette, or vaping, product use-associated lung injury in the United States. Drug Alcohol Rev. 2023;42: 258–261. doi:10.1111/dar.13533	No	Not meeting inclusion criteria	Title and abstract screening
3243	Mendelsohn CP, Hall W. What are the harms of vaping in young people who have never smoked?. Int J Drug Policy. 2023;117: 104064. doi:10.1016/j.drugpo.2023.104064	No	Not meeting inclusion criteria	Title and abstract screening
3244	Mendelsohn CP. Electronic cigarettes in physician practice. Intern Med J. 2018;48: 391–396. doi:10.1111/imj.13761	No	Not meeting inclusion criteria	Title and abstract screening
3245	Mendez D, Warner KE. A Magic Bullet? The Potential Impact of E-Cigarettes on the Toll of Cigarette Smoking. Nicotine Tob Res Off J Soc Res Nicotine Tob. 2021;23: 654–661. doi:10.1093/ntr/ntaa160	No	Not meeting inclusion criteria	Title and abstract screening
3246	Meng X.-C., Guo X.-X., Peng Z.-Y., Wang C., Liu R. Acute Effects of Electronic Cigarettes on Vascular Endothelial Function: A Systematic Review and Meta-analysis of Randomized Controlled Trials. Eur J Prev Cardiol. 2022. doi:10.1093/eurjpc/zwac248	No	Not meeting inclusion criteria	Title and abstract screening
3247	Meng Z, Li Q, Ma Y, Liu C. Transcranial direct current stimulation of the frontal-parietal-temporal brain areas reduces cigarette consumption in abstinent heroin users. J Psychiatr Res. 2022;152: 321–325. doi:10.1016/j.jpsychires.2022.06.045	No	Not meeting inclusion criteria	Title and abstract screening
3248	Mercadante S. The Paradigm Shift from End of Life to Pre-Emptive Palliative Care in Patients with Cancer. Cancers. 2022;14: 3752–3752. doi:10.3390/cancers14153752	No	Not meeting inclusion criteria	Title and abstract screening
3249	Mercincavage M, Lochbuehler K, Wileyto EP, Benowitz NL, Tyndale RF, Lerman C, et al. Association of Reduced Nicotine Content Cigarettes With Smoking Behaviors and Biomarkers of Exposure Among Slow and Fast Nicotine Metabolizers: A Nonrandomized Clinical Trial. JAMA Netw Open. 2018;1: e181346. doi:10.1001/jamanetworkopen.2018.1346	No	Not meeting inclusion criteria	Title and abstract screening
3250	Mercincavage M, Sidhu AK, Waugh L, Kreider C, Souprountchouk V, Delnevo CD, et al. Effects of pictorial warning labels depicting lesser-known and well-known risks of smoking on viewing patterns, recall, and knowledge of smoking harms. Drug Alcohol Depend. 2023;251: 110939. doi:10.1016/j.drugalcdep.2023.110939	No	Not meeting inclusion criteria	Title and abstract screening
3251	Merianos AL, Russell AM, Mahabee-Gittens EM, Barry AE, Yang M, Lin HC. Concurrent use of e-cigarettes and cannabis and associated COVID-19 symptoms, testing, and diagnosis among student e-cigarette users at four U.S. Universities. Addict Behav. 2022;126: 107170. doi:10.1016/j.addbeh.2021.107170	No	Not meeting inclusion criteria	Title and abstract screening
3252	Merianos AL, Jandarov RA, Klein JD, Mahabee-Gittens EM. Characteristics of Daily E-Cigarette Use and Acquisition Means Among a National Sample of Adolescents. Am J Health Promot AJHP. 2019;33: 1115–1122. doi:10.1177/0890117119854051	No	Not meeting inclusion criteria	Title and abstract screening
3253	Merianos AL, Mahabee-Gittens EM, Montemayor BN, Sherman LD, Goidel RK, Bergeron CD, et al. Current tobacco use patterns associated with healthcare utilization among non-Hispanic Black and Hispanic men with chronic conditions. Addict Behav. 2023;143: 107695. doi:10.1016/j.addbeh.2023.107695	No	Not meeting inclusion criteria	Title and abstract screening
3254	Merkel E, Rosenberg AR. Quality measures in end-of-life care among pediatric cancer patients: A lever for optimizing comfort and improving equity. Pediatr Blood Cancer. 2022;69: 1–2. doi:10.1002/pbc.29883	No	Not meeting inclusion criteria	Title and abstract screening
3255	Mermelstein RJ, Hedeker D, Rest EC. Response To: Can We Measure Nicotine Dependence in Dual Users of Cigarettes and ENDS? Nicotine Tob Res. 2021;23: 874–875. doi:10.1093/ntr/ntaa264	No	Not meeting inclusion criteria	Title and abstract screening
3256	Mersha AG, Gould GS, Bovill M, Eftekhari P. Barriers and Facilitators of Adherence to Nicotine Replacement Therapy: A Systematic Review and Analysis Using the Capability, Opportunity, Motivation, and Behaviour (COM-B) Model. Int J Env Res Public Health. 2020;17. doi:10.3390/ijerph17238895	No	Not meeting inclusion criteria	Title and abstract screening
3257	Merz L., Puhan M.A. Taxation options for nicotine and tobacco products in Switzerland - a review of tax policies. Swiss Med Wkly. 2021;151: w30083. doi:10.4414/smw.2021.w30083	No	Not meeting inclusion criteria	Title and abstract screening
3258	Merz W, Magraner J, Gunge D, Advani I, Crotty Alexander LE, Oren E. Electronic cigarette use and perceptions during COVID-19. Respir Med. 2022;200: 106925. doi:10.1016/j.rmed.2022.106925	No	Not meeting inclusion criteria	Title and abstract screening

3259	Mescolo F, Ferrante G, La Grutta S. Effects of E-Cigarette Exposure on Prenatal Life and Childhood Respiratory Health: A Review of Current Evidence. Front Pediatr. 2021;9: 711573. doi:10.3389/fped.2021.711573	No	Not meeting inclusion criteria	Title and abstract screening
3260	Mesdom P, Colle R, Becquemont L, Chappell K, David DJ, Mendez-David I, et al. Tobacco use is associated with low peripheral beta-arrestin 1 levels in major depression: A preliminary report. Drug Alcohol Depend. 2022;240: 109653. doi:10.1016/j.drugalcdep.2022.109653	No	Not meeting inclusion criteria	Title and abstract screening
3261	Metcalf M, Rossie K, Stokes K, Tanner B. Health Care Professionals' Clinical Skills to Address Vaping and e-Cigarette Use by Patients: Needs and Interest Questionnaire Study. JMIR Form Res. 2022;6: e32242. doi:10.2196/32242	No	Not meeting inclusion criteria	Title and abstract screening
3262	Miaw J.L., Hock Y.L.K., Yusoff M.F.M., Ab Rahman J., Ab Rahman N.S., Draman S.B., et al. Low nicotine addiction level, older age, and being female are associated with contemplation and preparation stages of smoking cessation among adult current smokers in Malaysia. Med J Malaysia. 2017;72: 69.	No	Not meeting inclusion criteria	Title and abstract screening
3263	Michalek IM, Koczkodaj P, Durzynska M, Caetano Dos Santos FL, Manczuk M. Tobacco products and electronic cigarettes' legislation violations - observational study in 31 European countries, 2005-2022. Env Res. 2023;233: 116429. doi:10.1016/j.envres.2023.116429	No	Not meeting inclusion criteria	Title and abstract screening
3264	Michaud G.C., Channick C.L., Caplan-Shaw C., Iaccarino J.M., Slatore C.G., Bade B., et al. ATS Core Curriculum 2017: Part IV. Adult pulmonary medicine series editor: Jason T. Poston Part IV Editors: Gaetane C. Michaud, Colleen L. Channick, and Caralee Caplan-Shaw. Ann Am Thorac Soc. 2017;14: S196–S208. doi:10.1513/AnnalsATS.201702-185CME	No	Not meeting inclusion criteria	Title and abstract screening
3265	Middlekauff H.R. Cardiovascular impact of electronic-cigarette use. Trends Cardiovasc Med. 2020;30: 133–140. doi:10.1016/j.tcm.2019.04.006	No	Not meeting inclusion criteria	Title and abstract screening
3266	Middlekauff HR, Cooper ZD, Strauss SB. Drugs of Misuse: Focus on Vascular Dysfunction. Can J Cardiol. 2022;38: 1364–1377. doi:10.1016/j.cjca.2022.04.011	No	Not meeting inclusion criteria	Title and abstract screening
3267	Miech R, Johnston L, O'Malley PM, Bachman JG, Patrick ME. Trends in Adolescent Vaping, 2017-2019. N Engl J Med. 2019;381: 1490–1491. doi:10.1056/NEJMc1910739	No	Not meeting inclusion criteria	Title and abstract screening
3268	Miech R, Patrick ME, Keyes K, O'Malley PM, Johnston L. Adolescent drug use before and during U.S. national COVID-19 social distancing policies. Drug Alcohol Depend. 2021;226: 108822. doi:10.1016/j.drugalcdep.2021.108822	No	Not meeting inclusion criteria	Title and abstract screening
3269	Miech R, Patrick ME, Keyes K. Declines in Adolescent Substance Use After the COVID-19 Pandemic Onset: The Role of Initiation in Grades 7 and 9. J Adolesc Health. 2023;73: 838–844. doi:10.1016/j.jadohealth.2023.05.033	No	Not meeting inclusion criteria	Title and abstract screening
3270	Miech R., Leventhal A.M., O'Malley P.M., Johnston L.D., Barrington-Trimis J.L. Failed Attempts to Quit Combustible Cigarettes and e-Cigarettes among US Adolescents. JAMA. 2022;327: 1179–1181. doi:10.1001/jama.2022.1692	No	Not meeting inclusion criteria	Title and abstract screening
3271	Miguel JP, Dias ADC, Bettini NR, Sousa SABA, Durão MPL, Oliveira LVC, et al. Cigarette smoke exposure causes systemic and autonomic cardiocirculatory changes in rats depending on the daily exposure dose. Life Sci. 2021;277: 119498. doi:10.1016/j.lfs.2021.119498	No	Not meeting inclusion criteria	Title and abstract screening
3272	Míguez MC, Pereira B, Figueiredo B. Tobacco consumption and spontaneous quitting at the first trimester of pregnancy. Addict Behav. 2017;64: 111–117. doi:10.1016/j.addbeh.2016.08.034	No	Not meeting inclusion criteria	Title and abstract screening
3273	Milcarz K, Makowiec-Dąbrowska T, Bak-Romaniszyn L, Kaleta D. Smoking Patterns and Smoking Cessation Willingness-A Study among Beneficiaries of Government Welfare Assistance in Poland. Int J Env Res Public Health. 2017;14. doi:10.3390/ijerph14020131	No	Not meeting inclusion criteria	Title and abstract screening
3274	Milella MS, Sansone A, Basili S, Battaglia G, La Torre G, Ferketich AK, et al. E-learning course improves knowledge in tobacco dependence, electronic nicotine delivery systems and heat-not-burn products in Medical School students. Clin Ter. 2021;172: 427–434. doi:10.7417/CT.2021.2353	No	Not meeting inclusion criteria	Title and abstract screening
3275	Miller A., Krotulski A., Walton S., Dulaney A., Frolichstein A., Dusek H., et al. Kratom cardiotoxicity: A report of reversible brugada pattern and QTC prolongation. Clin Toxicol. 2023;61: 121. doi:10.1080/15563650.2023.2233835	No	Not meeting inclusion criteria	Title and abstract screening
3276	Miller AR, Balino A, Tun Min S, Butt S, Karanth NV, Charakidis M. A single-centre experience of immune checkpoint inhibitors in patients with cancer in the Top End of the Northern Territory, Australia from 2016 to 2021. J Clin Oncol. 41: e18841–e18841. doi:10.1200/JCO.2023.41.16_suppl.e18841	No	Not meeting inclusion criteria	Title and abstract screening
3277	Miller BJ, Meshnick AB, Lushniak BD. Electronic nicotine delivery systems: the need for continued regulatory innovation. Tob Control. 2021. doi:10.1136/tobaccocontrol-2021-056622	No	Not meeting inclusion criteria	Title and abstract screening
3278	Miller C.R., Shi H., Li D., Goniewicz M.L. Cross-sectional associations of smoking and e-cigarette use with self-reported diagnosed hypertension: Findings from wave 3 of the population assessment of tobacco and health study. Toxics. 2021;9: 52. doi:10.3390/toxics9030052	No	Not meeting inclusion criteria	Title and abstract screening
3279	Miller CR, Smith DM, Goniewicz ML. Changes in Nicotine Product Use among Dual Users of Tobacco and Electronic Cigarettes: Findings from the Population Assessment of Tobacco and Health (PATH) Study, 2013-2015. Subst Use Misuse. 2020;55: 909–913. doi:10.1080/10826084.2019.1710211	No	Not meeting inclusion criteria	Title and abstract screening
3280	Miller CR, Sutanto E, Smith DM, Hitchman SC, Gravely S, Yong HH, et al. Awareness, trial and use of heated tobacco products among adult cigarette smokers and e-cigarette users: findings from the 2018 ITC Four Country Smoking and Vaping Survey. Tob Control. 2022;31: 11–18. doi:10.1136/tobaccocontrol-2020-055985	No	Not meeting inclusion criteria	Title and abstract screening
3281	Miller CR, Sutanto E, Smith DM, Hitchman SC, Gravely S, Yong HH, et al. Characterizing Heated Tobacco Product Use Among Adult Cigarette Smokers and Nicotine Vaping Product Users in the 2018 ITC Four Country Smoking & Vaping Survey. Nicotine Tob Res. 2022;24: 493–502. doi:10.1093/ntr/ntab217	No	Not meeting inclusion criteria	Title and abstract screening
3282	Miller CR, Xu SS, Smith DM, Sutanto E, Goniewicz ML, Quah ACK, et al. Assessing use of inhalable nicotine products within complex markets: the dilemma of heated tobacco products. Tob Control. 2022. doi:10.1136/tobaccocontrol-2021-057081	No	Not meeting inclusion criteria	Title and abstract screening
3283	Miller CR, Xu SS, Smith DM, Sutanto E, Goniewicz ML, Quah ACK, et al. Assessing use of inhalable nicotine products within complex markets: the dilemma of heated tobacco products. Tob Control. 2023;33: 103–109. doi:10.1136/tobaccocontrol-2021-057081	No	Not meeting inclusion criteria	Title and abstract screening
3284	Miller J., Khan H., Keduson M.M., Taylor M., Shih A., Goldman J. ECMO in Patients with Unexplained ARDS- Case Definition and Recommendations for Trimethoprim Sulfamethoxazole ARDS. ASAIO J. 2022;68: 17.	No	Not meeting inclusion criteria	Title and abstract screening
3285	Miller J., Khan H., Mino-Kenudson M., Taylor M., Shih A., Goldman J. Definition and Clinical Evaluation for Trimethoprim-Sulfamethoxazole Severe Acute Respiratory Failure. Crit Care Med. 2023. doi:10.1097/CCM.0000000000006002	No	Not meeting inclusion criteria	Title and abstract screening
3286	Miller J.O., Shih A.R., Mino-Kenudson M., Taylor M.S., Goldman J.L. Trimethoprim-sulfamethoxazole-associated fulminant respiratory failure in children and young adults. Am J Respir Crit Care Med. 2021;203: 918–921. doi:10.1164/rccm.202009-3421LE	No	Not meeting inclusion criteria	Title and abstract screening
3287	Miller K, McPherson L, Gewirtz O'Brien J, Svetaz MV. Adolescent Health: Substance Use. FP Essent. 2021;507: 26–32.	No	Not meeting inclusion criteria	Title and abstract screening

3288	Miller R., Varney S. Pediatric exposures to nicotine devices and nicotine solutions reported to United States poison centers: A summary of NPDS cases from 2010-2022. Clin Toxicol. 2023;61: 156–157. doi:10.1080/15563650.2023.2233835	No	Not meeting inclusion criteria	Title and abstract screening
3289	Miller TR, Johnson MB, Dziura JD, Weiss J, Carpenter KM, Grau LE, et al. Cost-Effectiveness of Smoking Cessation Approaches in Emergency Departments. Am J Prev Med. 2023;65: 39–44. doi:10.1016/j.amepre.2023.01.006	No	Not meeting inclusion criteria	Title and abstract screening
3290	Milstred AR, Douglas AE, Romm KF, Blank MD. Evaluation of the Psychometric Properties of Dependence Measures for Exclusive Electronic Cigarette Users. Nicotine Tob Res Off J Soc Res Nicotine Tob. 2022. doi:10.1093/ntr/ntac260	No	Not meeting inclusion criteria	Title and abstract screening
3291	Milstred AR, Douglas AE, Romm KF, Blank MD. Evaluation of the psychometric properties of dependence measures for exclusive electronic cigarette users. Nicotine Tob Res. 2023;25: 563–570. doi:10.1093/ntr/ntac260	No	Not meeting inclusion criteria	Title and abstract screening
3292	Min JY, Levin J, Weinberger AH. Associations of tobacco cigarette use and dependence with substance use disorder treatment completion by sex/gender and race/ethnicity. J Subst Abuse Treat. 2022;140: 108834. doi:10.1016/j.jsat.2022.108834	No	Not meeting inclusion criteria	Title and abstract screening
3293	Mine M., Masaki N., Toya T., Namba T., Nagatomo Y., Takase B., et al. Association between Smoking and Urine Indole Levels Measured by a Commercialized Test. Metabolites. 2022;12: 234. doi:10.3390/metabo12030234	No	Not meeting inclusion criteria	Title and abstract screening
3294	Ming-Shian Lu, Miao-Fen Chen, Yao-Hsu Yang, Chuan-Pin Lee, Chien-Chao Lin, Yuan-Hsi Tseng, et al. Appraisal of lung cancer survival in patients with end-stage renal disease. Arch Med Sci. 2023;19: 86–93. doi:10.5114/aoms.2019.86783	No	Not meeting inclusion criteria	Title and abstract screening
3295	Ming-Yu Cheng, Shu-Chien Liu, Mei-Wen Wang. Nursing Experience of a Young Female Patient with End-Stage Gastric Cancer. Tzu Chi Nurs J. 2022;21: 87–96.	No	Not meeting inclusion criteria	Title and abstract screening
3296	Mirbolouk M, Boakye E, Obisesan O, Osei AD, Dzaye O, Osuji N, et al. E-cigarette use among high school students in the United States prior to the COVID-19 pandemic: Trends, correlates, and sources of acquisition. Prev Med Rep. 2022;29: 101925. doi:10.1016/j.pmedr.2022.101925	No	Not meeting inclusion criteria	Title and abstract screening
3297	Mishra S. Are e-cigarettes beneficial for public health: Hume's guillotine - The debate continues? Indian Heart J. 2017;69: 810–813. doi:10.1016/j.ihj.2017.11.007	No	Not meeting inclusion criteria	Title and abstract screening
3298	Mishra S., Shekunov J., Derscheid D.J., Canterbury E.A., Leung J.G. Delayed Signs and Symptoms of Extended Release Guanfacine Overdose in Two Adolescent Patients: Implications of Monitoring on the Psychiatry Unit. Case Rep Psychiatry. 2022;2022: 2149301. doi:10.1155/2022/2149301	No	Not meeting inclusion criteria	Title and abstract screening
3299	Mistry R, Bondarenko I, Jeon J, Brouwer AF, Mattingly DT, Hirschtick JL, et al. Latent class analysis of use frequencies for multiple tobacco products in US adults. Prev Med. 2021;153: 106762. doi:10.1016/j.ypmed.2021.106762	No	Not meeting inclusion criteria	Title and abstract screening
3300	Mitchell C, Stabellini N, Chen Z, Fu P, Montero AJ. The impact of patient age on palliative care utilization and end-of-life outcomes among patients with metastatic breast cancer. J Clin Oncol. 41: 12034–12034. doi:10.1200/JCO.2023.41.16_suppl.12034	No	Not meeting inclusion criteria	Title and abstract screening
3301	Mitchell H.G., Kromash R.H., Ginley M.K. Moderating factors of co-occurring internet gaming disorder and ends dependency. J Addict Med. 2021;15: E37. doi:10.1097/ADM.0000000000000902	No	Not meeting inclusion criteria	Title and abstract screening
3302	Mitri A., Lin G., Waldman R.A., Grant-Kels J.M. Effects of tobacco and vaping on the skin. Clin Dermatol. 2021;39: 762–771. doi:10.1016/j.clindermatol.2021.05.004	No	Not meeting inclusion criteria	Title and abstract screening
3303	Mitrovich C., Weiss J.E., Ahuja N. Severe necrotizing pneumonia and persistent bacteremia in a teenager with a history of vaping. Pediatrics. 2021;147: 881–883. doi:10.1542/peds.147.3_MeetingAbstract.881	No	Not meeting inclusion criteria	Title and abstract screening
3304	Mitsuko Ushikubo. Nurses' Perceptions Regarding End-of-life Care for Individuals with Non-cancer Diseases on Non-invasive Positive Pressure Ventilation-dependent: A Qualitative and Descriptive Study. Indian J Palliat Care. 2023;29: 175–180. doi:10.25259/IJPC_184_2022	No	Not meeting inclusion criteria	Title and abstract screening
3305	Moazed F., Calfee C.S. The canary in the coal mine is coughing: Electronic cigarettes and respiratory symptoms in adolescents. Am J Respir Crit Care Med. 2017;195: 974–976. doi:10.1164/rccm.201611-2259ED	No	Not meeting inclusion criteria	Title and abstract screening
3306	Modak P, Hutslar C, Polk R, Atkinson E, Fisher L, Macy J, et al. Neural bases of risky decisions involving nicotine vapor versus monetary reward. NeuroImage Clin. 2021;32: 102869. doi:10.1016/j.nicl.2021.102869	No	Not meeting inclusion criteria	Title and abstract screening
3307	Moerke MJ, McMahon LR, Wilkerson JL. More than Smoke and Patches: The Quest for Pharmacotherapies to Treat Tobacco Use Disorder. Pharmacol Rev. 2020;72: 527–557. doi:10.1124/pr.119.018028	No	Not meeting inclusion criteria	Title and abstract screening
3308	Mohamed HSM, Ali IA. Comparative study of pulmonary functions test among different substances abusers. BMC Pulm Med. 2023;23: 452. doi:10.1186/s12890-023-02760-6	No	Not meeting inclusion criteria	Title and abstract screening
3309	Mohamed R, Siddiqui Z, Sutphin R, Kelly KM. Illness representations of vaping associated lung injury (VALI) and E-cigarette use: common sense model of self-regulation driven mixed-method approach. Psychol Health. 2022; 1–21. doi:10.1080/08870446.2021.2024828	No	Not meeting inclusion criteria	Title and abstract screening
3310	Mohamed R, Siddiqui Z, Sutphin R, Kelly KM. Illness representations of vaping associated lung injury (VALI) and E-cigarette use: common sense model of self-regulation driven mixed-method approach. Psychol Health. 2023;38: 1494–1514. doi:10.1080/08870446.2021.2024828	No	Not meeting inclusion criteria	Title and abstract screening
3311	Mohammed HO, Ahmed Alaa El-Din E, Farag AI. Impact of e-cigarettes on colonic mucosa and the role of recovery: involvement of oxidative and inflammatory pathway. Environ Sci Pollut Res Int. 2021;28: 64561–64571. doi:10.1007/s11356-021-15575-x	No	Not meeting inclusion criteria	Title and abstract screening
3312	Mohanty R., Nayak R., Mohanty G., Das A.C., Panda S. Smoking cessation: The aromatherapy way. Indian J Public Health Res Dev. 2019;10: 1071–1075. doi:10.5958/0976-5506.2019.03648.9	No	Not meeting inclusion criteria	Title and abstract screening
3313	Mok Y, Jeon J, Levy DT, Meza R. Associations Between E-cigarette Use and E-cigarette Flavors With Cigarette Smoking Quit Attempts and Quit Success: Evidence From a U.S. Large, Nationally Representative 2018-2019 Survey. Nicotine Tob Res. 2023;25: 541–552. doi:10.1093/ntr/ntac241	No	Not meeting inclusion criteria	Title and abstract screening
3314	Molino A.R., Jerry-Fluker J., Atkinson M.A., Furth S.L., Warady B.A., Ng D.K. The association of alcohol, cigarette, e-cigarette, and marijuana use with disease severity in adolescents and young adults with pediatric chronic kidney disease. Pediatr Nephrol. 2021;36: 2493–2497. doi:10.1007/s00467-021-05044-5	No	Not meeting inclusion criteria	Title and abstract screening
3315	Momokita M, Abe A, Shibata K, Hayashi H, Furuta H, Taniguchi S, et al. Prognostic Nutritional Index in Patients With End-Stage Oral Cancer. Am J Hosp Palliat Med. 2023;40: 396–400. doi:10.1177/10499091221102581	No	Not meeting inclusion criteria	Title and abstract screening
3316	Moncho MEI, Palomar-Abril V, Soria-Comes T. Palliative Care Unit at Home: Impact on Quality of Life in Cancer Patients at the End of Life in a Rural Environment. Am J Hosp Palliat Med. 2022;39: 523–529. doi:10.1177/10499091211038303	No	Not meeting inclusion criteria	Title and abstract screening

3317	Monnig MA, Treloar Padovano H, Sokolovsky AW, DeCost G, Aston ER, Haass-Koffler CL, et al. Association of Substance Use With Behavioral Adherence to Centers for Disease Control and Prevention Guidelines for COVID-19 Mitigation: Cross-sectional Web-Based Survey. JMIR Public Health Surveill. 2021;7: e29319. doi:10.2196/29319	No	Not meeting inclusion criteria	Title and abstract screening
3318	Montalto G.J., Cius E.G., Rahmandar M.H. Cannabinoids and the adolescent patient: A pragmatic guide for pediatric practitioners. Pediatr Ann. 2021;50: e57–e64. doi:10.3928/19382359-20210120-01	No	Not meeting inclusion criteria	Title and abstract screening
3319	Montebello M. SESSION 1: SUBSTANCE USE IN ADOLESCENCE, PERFORMANCE AND IMAGE-ENHANCING DRUGS (PIEDS) AND NICOTINE DEPENDENCE AND VAPING: NICOTINE DEPENDENCE AND VAPING. Aust N Z J Psychiatry. 2022;56: 30. doi:10.1177/00048674221088686	No	Not meeting inclusion criteria	Title and abstract screening
3320	Montemitro C., Ross T.J., Fedota J., Salmeron B.J., di Giannantonio M., Stein E.A. P36. Partial Nicotine Replacement: A First Step Towards Smoking Cessation? Evidence From a Smoking Cessation Protocol. Biol Psychiatry. 2022;91: S102. doi:10.1016/j.biopsych.2022.02.271	No	Not meeting inclusion criteria	Title and abstract screening
3321	Montes de Oca M. Smoking Cessation/Vaccinations. Clin Chest Med. 2020;41: 495–512. doi:10.1016/j.ccm.2020.06.013	No	Not meeting inclusion criteria	Title and abstract screening
3322	Montes de Oca M, Laucho-Contreras ME. Smoking cessation and vaccination. Eur Respir Rev Off J Eur Respir Soc. 2023;32. doi:10.1183/16000617.0187-2022	No	Not meeting inclusion criteria	Title and abstract screening
3323	Montgomery L, Winhusen T, Scodes J, Pavlicova M, Twitty D, Campbell ANC, et al. Reductions in tobacco use in naltrexone, relative to buprenorphine-maintained individuals with opioid use disorder: Secondary analysis from the National Drug Abuse Treatment Clinical Trials Network. J Subst Abuse Treat. 2021;130: 108489. doi:10.1016/j.jsat.2021.108489	No	Not meeting inclusion criteria	Title and abstract screening
3324	Montiel Ishino FA, Villalobos K, Williams F. Substance Use From Social Distancing and Isolation by US Nativity During the Time of COVID-19: Cross-sectional Study. JMIR Public Health Surveill. 2023;9: e38163. doi:10.2196/38163	No	Not meeting inclusion criteria	Title and abstract screening
3325	Moore SP, Osborn C, Gonzalez SK, Quisenberry A, Klein EG, Kaur M, et al. Flavour loyalty may predict cessation or substitution following a cigarillo flavour ban among young adults in the USA. Tob Control Int J. 2022;31: s206–s213.	No	Not meeting inclusion criteria	Title and abstract screening
3326	Morales M., Fahrion A., Watkins S.L. #NicotineAddictionCheck: Puff Bar Culture, Addiction Apathy, and Promotion of E-Cigarettes on TikTok. Int J Environ Res Public Health. 2022;19: 1820. doi:10.3390/ijerph19031820	No	Not meeting inclusion criteria	Title and abstract screening
3327	Morar T., Robertson L. Smoking cessation among people with mental illness: A South African perspective. South Afr Fam Pract. 2022;64: a5489. doi:10.4102/safp.v64i1.5489	No	Not meeting inclusion criteria	Title and abstract screening
3328	Morean M.E., Krishnan-Sarin S., O'Malley S.S. Corrigendum to "Comparing cigarette and e-cigarette dependence and predicting frequency of smoking and e-cigarette use in dual-users of cigarettes and e-cigarettes." [Addict Behaviour. 87(2018), 92-96](S0306460318301886)(10.1016/j.addbeh.2018.06.027). Addict Behav. 2019;97: 132. doi:10.1016/j.addbeh.2019.05.028	No	Not meeting inclusion criteria	Title and abstract screening
3329	Morean M.E., Krishnan-Sarin S., O'Malley S.S. Corrigendum to "Assessing nicotine dependence in adolescent E-cigarette users: The 4-item Patient-Reported Outcomes Measurement Information System (PROMIS) Nicotine Dependence Item Bank for electronic cigarettes" (Drug and Alcohol Dependence (2018) 188 (6. Drug Alcohol Depend. 2020;206: 107602. doi:10.1016/j.drugalcdep.2019.107602	No	Not meeting inclusion criteria	Title and abstract screening
3330	Morean M.E., Krishnan-Sarin S., Sussman S., Foulds J., Fishbein H., Grana R., et al. Erratum: Psychometric evaluation of the Patient-Reported Outcomes Measurement Information System (PROMIS)Nicotine Dependence Item Bankfor use with electronic cigarettes (Nicotine and Tobacco Research (2018) DOI: 10.1093/ntr/ntx271). Nicotine Tob Res. 2020;22: 2123. doi:10.1093/ntr/ntz095	No	Not meeting inclusion criteria	Title and abstract screening
3331	Morean M.E., Wackowski O.A., Eissenberg T., Delnevo C.D., Krishnan-Sarin S., Gueorguieva R. Novel Nicotine Concentration Labels Improve Adolescents' and Young Adults' Understanding of the Nicotine Strength of Electronic Nicotine Delivery System Products. Nicotine Tob Res. 2022;24: 1110–1119. doi:10.1093/ntr/ntac024	No	Not meeting inclusion criteria	Title and abstract screening
3332	Morean ME, Bold KW, Kong G, Gueorguieva R, Camenga DR, Simon P, et al. Adolescents' awareness of the nicotine strength and e-cigarette status of JUUL e-cigarettes. Drug Alcohol Depend. 2019;204: 107512. doi:10.1016/j.drugalcdep.2019.05.032	No	Not meeting inclusion criteria	Title and abstract screening
3333	Morean ME, Krishnan-Sarin S, O'Malley SS. Corrigendum to "Assessing nicotine dependence in adolescent E-cigarette users: The 4-item Patient-Reported Outcomes Measurement Information System (PROMIS) Nicotine Dependence Item Bank for electronic cigarettes" [Drug Alcohol Depend. 188 (2018) 60-63]. Drug Alcohol Depend. 2020;206: 107602. doi:10.1016/j.drugalcdep.2019.107602	No	Not meeting inclusion criteria	Title and abstract screening
3334	Morean ME, Krishnan-Sarin S, Sussman S, Foulds J, Fishbein H, Grana R, et al. Development and psychometric validation of a novel measure of sensory expectancies associated with E-cigarette use. Addict Behav. 2019;91: 208–215. doi:10.1016/j.addbeh.2018.08.031	No	Not meeting inclusion criteria	Title and abstract screening
3335	Morean ME, Krishnan-Sarin S, Sussman S, Foulds J, Fishbein H, Grana R, et al. Psychometric Evaluation of the E-cigarette Dependence Scale. Nicotine Tob Res. 2019;21: 1556–1564. doi:10.1093/ntr/ntx271	No	Not meeting inclusion criteria	Title and abstract screening
3336	Morean ME, Krishnan-Sarin S, Sussman S, Foulds J, Fishbein H, Grana R, et al. Psychometric evaluation of the Patient-Reported Outcomes Measurement Information System (PROMIS)Nicotine Dependence Item Bankfor use with electronic cigarettes. Nicotine Tob Res. 2020;22: 2123. doi:10.1093/ntr/ntz095	No	Not meeting inclusion criteria	Title and abstract screening
3337	Morean ME, L'Insalata A. The Short Form Vaping Consequences Questionnaire: Psychometric Properties of a Measure of Vaping Expectancies for Use With Adult E-cigarette Users. Nicotine Tob Res. 2017;19: 215–221. doi:10.1093/ntr/ntw205	No	Not meeting inclusion criteria	Title and abstract screening
3338	Morean ME, Davis DR, Kong G, Bold KW, Krishnan-Sarin S. Psychometric evaluation of the sensory e-cigarette expectancies scale for use with adolescents. Addict Behav. 2024;148: 107869. doi:10.1016/j.addbeh.2023.107869	No	Not meeting inclusion criteria	Title and abstract screening
3339	Morean ME, Krishnan-Sarin S, O'Malley SS. Corrigendum to "Comparing cigarette and e-cigarette dependence and predicting frequency of smoking and e-cigarette use in dual-users of cigarettes and e-cigarettes." [Addict Behaviour. 87(2018), 92-96]. Addict Behav. 2019;97: 132. doi:10.1016/j.addbeh.2019.05.028	No	Not meeting inclusion criteria	Title and abstract screening
3340	Morean ME, Krishnan-Sarin S, O'Malley SS. "Assessing nicotine dependence in adolescent E-cigarette users: The 4-item Patient-Reported Outcomes Measurement Information System (PROMIS) Nicotine Dependence Item Bank for electronic cigarettes": Corrigendum. Drug Alcohol Depend. 2020;206. doi:10.1016/j.drugalcdep.2019.107602https://dx.doi.org/10.1016/j.drugalcdep.2019.107602	No	Not meeting inclusion criteria	Title and abstract screening
3341	Morean ME, Gueorguieva R, O'Malley S, Krishnan-Sarin S. Including the term "tobacco-free nicotine" in the nicotine addiction warning label mandated by the US Food and Drug Administration alters risk perceptions and use intentions. Tob Control. 2023. doi:10.1136/tc-2023-058133	No	Not meeting inclusion criteria	Title and abstract screening
3342	Morean M, Krishnan-Sarin S, O'Malley SS. Corrigendum to "Comparing cigarette and e-cigarette dependence and predicting frequency of smoking and e-cigarette use in dual-users of cigarettes and e-cigarettes". Addict Behav. 2019;97: 132. doi:10.1016/j.addbeh.2019.05.028https://dx.doi.org/10.1016/j.addbeh.2019.05.028	No	Not meeting inclusion criteria	Title and abstract screening
3343	Morgan JC, Jeong M, Mendel-Sheldon J, Noar SM, Ribisl KM, Brewer NT. The impact of cigarette pack anti-littering messages. Addict Behav. 2022;126: 107184. doi:10.1016/j.addbeh.2021.107184	No	Not meeting inclusion criteria	Title and abstract screening
3344	Morgan JC, Silver N, Cappella JN. How did beliefs and perceptions about e-cigarettes change after national news coverage of the EVALI outbreak? PLoS ONE. 2021;16. doi:10.1371/journal.pone.0250908https://dx.doi.org/10.1371/journal.pone.0250908	No	Not meeting inclusion criteria	Title and abstract screening
3345	Morgan SL, Reed GL, Tidey J, Bunn JY, Harfmann RF, Heil SH, et al. Predictors of Adherence Among Vulnerable Populations of Adults Assigned to Smoke Very Low Nicotine Content Cigarettes. Nicotine Tob Res. 2022;24: 1959–1967. doi:10.1093/ntr/ntac145	No	Not meeting inclusion criteria	Title and abstract screening

3346	Morgenstern M, Nies A, Goecke M, Hanewinkel R. E-Cigarettes and the Use of Conventional Cigarettes. Dtsch Arztebl Int. 2018;115: 243–248. doi:10.3238/arztebl.2018.0243	No	Not meeting inclusion criteria	Title and abstract screening
3347	Morie KP, Wu J, Potenza MN, Krishnan-Sarin S, Mayes LC, Hammond CJ, et al. Daily cannabis use in adolescents who smoke tobacco is associated with altered late-stage feedback processing: A high-density electrical mapping study. J Psychiatr Res. 2021;139: 82–90. doi:10.1016/j.jpsychires.2021.05.022	No	Not meeting inclusion criteria	Title and abstract screening
3348	Morjaria JB, Campagna D, Caci G, O'Leary R, Polosa R. Health impact of e-cigarettes and heated tobacco products in chronic obstructive pulmonary disease: current and emerging evidence. Expert Rev Respir Med. 2022;16: 1213–1226. doi:10.1080/17476348.2023.2167716	No	Not meeting inclusion criteria	Title and abstract screening
3349	Morjaria JB, Campagna D, Caci G, O'Leary R, Polosa R. Health impact of e-cigarettes and heated tobacco products in chronic obstructive pulmonary disease: current and emerging evidence. Expert Rev Respir Med. 2023; 1–14. doi:10.1080/17476348.2023.2167716	No	Not meeting inclusion criteria	Title and abstract screening
3350	Morjaria JB, Mondati E, Polosa R. E-cigarettes in patients with COPD: current perspectives. Int J Chron Obstruct Pulmon Dis. 2017;12: 3203–3210. doi:10.2147/COPD.S135323	No	Not meeting inclusion criteria	Title and abstract screening
3351	Morphett K. Commentary on Notley et al. : Understanding transitions in the use of nicotine and tobacco products-the value of qualitative longitudinal research. Addiction. 2021;116: 606–607. doi:10.1111/add.15307	No	Not meeting inclusion criteria	Title and abstract screening
3352	Morphett K, Carter A, Hall W, Gartner C. Medicalisation, smoking and e-cigarettes: evidence and implications. Tob Control. 2017;26: e134–e139. doi:10.1136/tobaccocontrol-2016-053348	No	Not meeting inclusion criteria	Title and abstract screening
3353	Morphett K, Fraser D, Borland R, Hall W, Walker N, Bullen C, et al. A Pragmatic Randomized Comparative Trial of e-Cigarettes and Other Nicotine Products for Quitting or Long-Term Substitution in Smokers. Nicotine Tob Res. 2022;24: 1079–1088. doi:10.1093/ntr/ntab266	No	Not meeting inclusion criteria	Title and abstract screening
3354	Morphett K, Hyland A, Sellars D, Gartner C. The environmental impact of tobacco products: Time to increase awareness and action. Addiction. 2023;118: 396–398. doi:10.1111/add.16046	No	Not meeting inclusion criteria	Title and abstract screening
3355	Morphett K, Puljević C, Borland R, Carter A, Hall W, Gartner C. Attitudes towards a hypothetical "clean nicotine" product and harm reduction among smokers. Int J Drug Policy. 2021;88: 103020. doi:10.1016/j.drugpo.2020.103020	No	Not meeting inclusion criteria	Title and abstract screening
3356	Morphett K., Gartner C. Informed Choice in the Context of Tobacco Use Disorder. Nicotine Tob Res. 2022;24: 1–2. doi:10.1093/ntr/ntab215	No	Not meeting inclusion criteria	Title and abstract screening
3357	Morris JD, Pebley K, Little MA. Vaping Opioids: Should We Be Worried? Am J Health Promot. 2023;37: 1171–1173. doi:10.1177/08901171231193785	No	Not meeting inclusion criteria	Title and abstract screening
3358	Moskal D., Whitaker H., Bernstein J.F., Maisto S.A., Connors G.J. Evaluation of a Web-based Psychosocial Education and Peer Support Program for Alcohol Use Concerns. Alcohol Treat Q. 2021;39: 366–382. doi:10.1080/07347324.2020.1866473	No	Not meeting inclusion criteria	Title and abstract screening
3359	Mosley D.D., DeVasure J.M., Heires A.J., Romberger D.J., Bailey K.L., Wyatt T.A. Alcohol and juul vapor co-exposure impairs airway epithelial repair functions. Alcohol Clin Exp Res. 2021;45: 219A. doi:10.1111/acer.14628	No	Not meeting inclusion criteria	Title and abstract screening
3360	Moss S. AN ELDERLY ZEBRA IN TEENAGER'S CLOTHING: WERNICKE'S ENCEPHALOPATHY. J Hosp Med. 2023;18: S29–S30. doi:10.1002/jhm.13090	No	Not meeting inclusion criteria	Title and abstract screening
3361	Mossman B, Perry LM, Walsh LE, Gerhart J, Malhotra S, Horswell R, et al. Anxiety, depression, and end-of-life care utilization in adults with metastatic cancer. Psychooncology. 2021;30: 1876–1883. doi:10.1002/pon.5754	No	Not meeting inclusion criteria	Title and abstract screening
3362	Motooka Y, Matsui T, Slaton RM, Umetsu R, Fukuda A, Naganuma M, et al. Adverse events of smoking cessation treatments (nicotine replacement therapy and non-nicotine prescription medication) and electronic cigarettes in the Food and Drug Administration Adverse Event Reporting System, 2004-2016. SAGE Open Med. 2018;6: 2050312118777953. doi:10.1177/2050312118777953	No	Not meeting inclusion criteria	Title and abstract screening
3363	Motschman C.A., Tiffany S.T. Combined smoking and alcohol cues: Effects on craving, drug-seeking, and consumption. Alcohol Clin Exp Res. 2021;45: 1864–1876. doi:10.1111/acer.14662	No	Not meeting inclusion criteria	Title and abstract screening
3364	Mourali M, Drake C. The Challenge of Debunking Health Misinformation in Dynamic Social Media Conversations: online Randomized Study of Public Masking During COVID-19. 2022;24: e34831. doi:10.2196/34831	No	Not meeting inclusion criteria	Title and abstract screening
3365	Moustafa AF, Rodriguez D, Mazur A, Audrain-McGovern J. Adolescent perceptions of E-cigarette use and vaping behavior before and after the EVALI outbreak. Prev Med Int J Devoted Pract Theory. 2021;145. doi:10.1016/j.ypmed.2021.106419https://dx.doi.org/10.1016/j.ypmed.2021.106419	No	Not meeting inclusion criteria	Title and abstract screening
3366	Mowls DS, Boeckman L, Gillaspy SR, Beebe LA. Long-term Quit Rates in Fax-Referred as Compared to Self-Referred Tobacco Quitline Registrants. Am J Prev Med. 2017;52: e115–e121. doi:10.1016/j.amepre.2016.11.006	No	Not meeting inclusion criteria	Title and abstract screening
3367	Moyns E.J., Jagpal P.S., Ferner R.E., Sandilands E.A., Gray L.A., Thanacoody R.H.K., et al. Recreational, inhalational misuse of hyoscine butylbromide (Buscopan) tablets as reported to the UK national poisons information service. Clin Toxicol. 2021;59: 552. doi:10.1080/15563650.2021.1906080	No	Not meeting inclusion criteria	Title and abstract screening
3368	Mueller SD. Using health assessments to impact intent-to-quit tobacco. Diss Abstr Int Sect B Sci Eng. 2021;82: No-Specified.	No	Not meeting inclusion criteria	Title and abstract screening
3369	Muhammad Siddique Qurashi H., Modelewski M., Saleem T., Ramesh N. CHONDRORADIONECROSIS AFTER LARYNGEAL RADIOTHERAPY: A NECESSARY EVIL OR AN AVOIDABLE COMPLICATION. Chest. 2021;160: A1478. doi:10.1016/j.chest.2021.07.1355	No	Not meeting inclusion criteria	Title and abstract screening
3370	Muhumuza D., Coiquaud R., Lechaux-Clement C., Loubrieu V. The pharmacist on the frontline to help smokers quit. Actual Pharm. 2021;60: 22–27. doi:10.1016/j.actpha.2021.08.014	No	Not meeting inclusion criteria	Title and abstract screening
3371	Mukherjee A. Beta hemolytic streptococcus induced septic pulmonary emboli complicated by lemierre's syndrome. Am J Respir Crit Care Med. 2021;203. doi:10.1164/ajrccm-conference.2021.203.1_MeetingAbstracts.A3969	No	Not meeting inclusion criteria	Title and abstract screening
3372	Mukhin A, Zuo Y, Berg H, Morgan J, Rose J, Sai K. Comparison of brain nicotine uptake from electronic cigarettes and traditional combustible cigarettes. 2021;46: 458-459. doi:10.1038/s41386-021-01238-5	No	Not meeting inclusion criteria	Title and abstract screening
3373	Müller T, Shevchenko Y, Gerhardt S, Kiefer F, Vollstädt-Klein S. The influence of perceived stress and self-control on efficacy of repeated transcranial direct current stimulation in non-treatment-seeking smokers. Drug Alcohol Depend. 2021;226: 108861. doi:10.1016/j.drugalcdep.2021.108861	No	Not meeting inclusion criteria	Title and abstract screening
3374	Mulligan KM, Zheng DX, Gallo Marin B, Do MT, Tucker DL, Igbinoba Z, et al. COVID-19 and EVALI: Considerations regarding two concurrent public health crises. Am J Emerg Med. 2022;56: 389–390. doi:10.1016/j.ajem.2021.11.042	No	Not meeting inclusion criteria	Title and abstract screening

3375	Mullins MA, Shahan K, Zaha V, Sulistio M, Terauchi S, Gerber DE, et al. Are we missing the beat in cancer end-of-life care: Prevalence and end-of-life monitoring of implantable cardioverter defibrillators in a population-based sample of older adults diagnosed with breast, colorectal, or pancreatic cancer. J Clin Oncol. 41: e18728–e18728. doi:10.1200/JCO.2023.41.16_suppl.e18728	No	Not meeting inclusion criteria	Title and abstract screening
3376	Mullins MA, Ruterbusch JJ, Clarke P, Uppal S, Wallner LP, Cote ML. Trends and racial disparities in aggressive end-of-life care for a national sample of women with ovarian cancer. Cancer 0008543X. 2021;127: 2229–2237. doi:10.1002/cncr.33488	No	Not meeting inclusion criteria	Title and abstract screening
3377	Mumtaz H, Hameed M, Sangah AB, Zubair A, Hasan M. Association between smoking and non-alcoholic fatty liver disease in Southeast Asia. Front Public Health. 2022;10: 1008878. doi:10.3389/fpubh.2022.1008878	No	Not meeting inclusion criteria	Title and abstract screening
3378	Mun S, Wang R, Ma X, Ananth P. Sociodemographic and hospital-based predictors of intense end-of-life care among children, adolescents, and young adults with hematologic malignancies. Cancer 0008543X. 2021;127: 3817–3824. doi:10.1002/cncr.33764	No	Not meeting inclusion criteria	Title and abstract screening
3379	Munir A., Aadil M., Qadir T., Tong C., Zeshan M. Prevalence and treatment of vaping and e-cigarette use among adolescents: A literature review. Am J Addict. 2021;30: 266. doi:10.1111/ajad.13173	No	Not meeting inclusion criteria	Title and abstract screening
3380	Murff HJ, Greevy RA, Sternlieb S, Gilliam K, King S, Sanghani R, et al. The Fish Oil to Reduce Tobacco Use iN Expectant mothers (FORTUNE) feasibility trial. Am J Obstet Gynecol MFM. 2022;4: 100707. doi:10.1016/j.ajogmf.2022.100707	No	Not meeting inclusion criteria	Title and abstract screening
3381	Murphy C., Kenny C., Hemann B., Haigney M. NICOTINE-INDUCED JUNCTIONAL RHYTHM: A CASE OF VAPING-INDUCED DYSRHYTHMIA. J Am Coll Cardiol. 2021;77: 2060. doi:10.1016/S0735-1097%2821%2903416-1	No	Not meeting inclusion criteria	Title and abstract screening
3382	Murphy CC, Tortolero GA, Gerber DE, Pruitt SL. An Updated Report on the Prevalence of Prior Cancer Among Persons Newly Diagnosed With Cancer in the Surveillance, Epidemiology, and End Results Program. JAMA Oncol. 2023;9: 1147–1150. doi:10.1001/jamaoncol.2023.1723	No	Not meeting inclusion criteria	Title and abstract screening
3383	Murphy CM, Martin RA, Tidey JW, Colby SM, Rohsenow DJ. Smoking outcome expectancies predict smoking during voucher-based treatment for smokers with substance use disorders. J Subst Abuse Treat. 2018;90: 73–78. doi:10.1016/j.jsat.2018.04.013	No	Not meeting inclusion criteria	Title and abstract screening
3384	Murphy K., Ventarola D. Iliofemoral Artery Endofibrosis Associated with Microembolization. J Vasc Surg. 2022;76: e24–e25. doi:10.1016/j.jvs.2022.06.106	No	Not meeting inclusion criteria	Title and abstract screening
3385	Murray R, Brain K, Britton J, Lewis S, Thorley R, Baldwin D, et al. PL03.03 Personalised Smoking Cessation Support in a Lung Cancer Screening Programme: the Yorkshire Enhanced Stop Smoking Study (YESS). 2022;17: S1-. doi:10.1016/j.jtho.2022.07.011	No	Not meeting inclusion criteria	Title and abstract screening
3386	Murray R., Thorley R., Baldwin D., Copeland H., Lewis S., Crosbie P., et al. The effectiveness of offering ongoing and repeated provision of co-located, opt-out smoking cessation support as part of a lung cancer screening programme. Lung Cancer. 2023;178: S70–S71. doi:10.1016/S0169-5002%2823%2900588-3	No	Not meeting inclusion criteria	Title and abstract screening
3387	Murray R.L., Evison M., Callister M.E. Nicotine or tobacco abstinence? Eur Respir Rev. 2022;31: 220089. doi:10.1183/16000617.0089-2022	No	Not meeting inclusion criteria	Title and abstract screening
3388	Mus S, Monzon J, Thrasher JF, Barnoya J. E-cigarette vending machines: a new access channel for youth in Guatemala City. Tob Control. 2023;32: e269–e270. doi:10.1136/tobaccocontrol-2021-057102	No	Not meeting inclusion criteria	Title and abstract screening
3389	Musa N., Ibrahim K.S., Katiman D., Lim C.W., Ismail J.R., Chua N.Y.C., et al. Vaping knowledge, attitude and practice amongst general public in Malaysia aisya. Respirology. 2017;22: 50. doi:10.1111/resp.13206_121	No	Not meeting inclusion criteria	Title and abstract screening
3390	Muthumalage T., McGraw M., Friedman A., Friedman M., Rahman I. Toxicological assessment of e-cigarette or vaping product use associated lung injury (EVALI) cartridges and constituents. FASEB J. 2021;35. doi:10.1096/fasebj.2021.35.S1.02541	No	Not meeting inclusion criteria	Title and abstract screening
3391	Myers L, Ireland MJ, Viljoen B, Goodwin B. Evaluating changes to home bowel cancer screening kits: an end-user perspective study. Cancer Causes Control. 2023;34: 583–594. doi:10.1007/s10552-023-01695-x	No	Not meeting inclusion criteria	Title and abstract screening
3392	Myers MG, MacPherson L. Cigarette smoking and nicotine dependence in adolescence. Oxf Handb Adolesc Subst Abuse. 2019; 113–129.	No	Not meeting inclusion criteria	Title and abstract screening
3393	Myers Smith K, Phillips-Waller A, Pesola F, McRobbie H, Przulj D, Orzol M, et al. E-cigarettes versus nicotine replacement treatment as harm reduction interventions for smokers who find quitting difficult: randomized controlled trial. Addiction. 2022;117: 224–233. doi:10.1111/add.15628	No	Not meeting inclusion criteria	Title and abstract screening
3394	Nabavizadeh P., Liu J., Rao P., Ibrahim S., Han D.D., Derakhshandeh R., et al. Impairment of Endothelial Function by Cigarette Smoke Is Not Caused by a Specific Smoke Constituent, but by Vagal Input From the Airway. Arterioscler Thromb Vasc Biol. 2022;42: 1324–1332. doi:10.1161/ATVBAHA.122.318051	No	Not meeting inclusion criteria	Title and abstract screening
3395	Nabi-Burza E., Drehmer J.E., Hipple Walters B., Rigotti N.A., Ossip D.J., Levy D.E., et al. Treating Parents for Tobacco Use in the Pediatric Setting: The Clinical Effort Against Secondhand Smoke Exposure Cluster Randomized Clinical Trial. JAMA Pediatr. 2019;173: 931–939. doi:10.1001/jamapediatrics.2019.2639	No	Not meeting inclusion criteria	Title and abstract screening
3396	Nacca N, McGraw MD, Croft DP, Kalininskiy A. Weight loss in adolescents presenting with EVALI. Pediatr Pulmonol. 2021;56: 2785–2786. doi:10.1002/ppul.25523	No	Not meeting inclusion criteria	Title and abstract screening
3397	Nadler E, Vasudevan A, Wentworth C, Robert N, Penrod JR, Fiore J, et al. Real-world relationship of early end points to survival end points in patients with resectable non-small-cell lung cancer. Future Oncol. 2023;19: 1785–1800. doi:10.2217/fon-2023-0170	No	Not meeting inclusion criteria	Title and abstract screening
3398	Nagano T., Katsurada M., Yasuda Y., Kobayashi K., Nishimura Y. Current pharmacologic treatments for smoking cessation and new agents undergoing clinical trials. Ther Adv Respir Dis. 2019;13. doi:10.1177/1753466619875925	No	Not meeting inclusion criteria	Title and abstract screening
3399	Nahhas G.J., Wilson D., Talbot V., Cartmell K.B., Warren G.W., Toll B.A., et al. Feasibility of implementing a hospital-based “opt-out” tobacco-cessation service. Nicotine Tob Res. 2017;19: 937–943. doi:10.1093/ntr/ntw312	No	Not meeting inclusion criteria	Title and abstract screening
3400	Nåhls N-S, Leskelä R-L, Saarto T, Hirvonen O, Anttonen A. Effect of palliative care decisions making on hospital service use at end-of-life in patients with malignant brain tumors: a retrospective study. BMC Palliat Care. 2023;22: 1–7. doi:10.1186/s12904-023-01154-z	No	Not meeting inclusion criteria	Title and abstract screening
3401	Naidoo M, Mbonane TP, Mathee A. Risk Factors and Perceptions of E-Cigarette Use Among Selected Users in Johannesburg, South Africa. J Community Health. 2021;46: 405–412. doi:10.1007/s10900-020-00948-z	No	Not meeting inclusion criteria	Title and abstract screening
3402	Naidu V, Zeki AA, Sharma P. Sex differences in the induction of angiotensin converting enzyme 2 (ACE-2) in mouse lungs after e-cigarette vapor exposure and its relevance to COVID-19. J Investig Med Off Publ Am Fed Clin Res. 2021;69: 954–961. doi:10.1136/jim-2020-001768	No	Not meeting inclusion criteria	Title and abstract screening
3403	Nakama C, Tabuchi T. Use of heated tobacco products by people with chronic diseases: The 2019 JASTIS study. PLoS One. 2021;16: e0260154. doi:10.1371/journal.pone.0260154	No	Not meeting inclusion criteria	Title and abstract screening

3404	Nakamura M, Abe M, Ohkura M, Treadow J, Yu CR, Park PW. Efficacy of Varenicline for Cigarette Reduction Before Quitting in Japanese Smokers: A Subpopulation Analysis of the Reduce to Quit Trial. Clin Ther. 2017;39: 863–872. doi:10.1016/j.clinthera.2017.03.007	No	Not meeting inclusion criteria	Title and abstract screening
3405	Nakamura T, Takeyasu Y, Yoshida T, Ohashi K, Ohe Y. End-of-life impact of concurrent diabetes mellitus and adrenal insufficiency as immune-related adverse events in an advanced non-small cell lung cancer patient. Thorac Cancer. 2022;13: 3073–3075. doi:10.1111/1759-7714.14660	No	Not meeting inclusion criteria	Title and abstract screening
3406	Nam J, Krishnan G, Shofer S, Navuluri N. Interventions to improve lung cancer screening among racially and ethnically minoritized groups: A scoping review. Lung Cancer. 2022;176: 46–55. doi:10.1016/j.lungcan.2022.12.016	No	Not meeting inclusion criteria	Title and abstract screening
3407	Nam J, Krishnan G, Shofer S, Navuluri N. Interventions to improve lung cancer screening among racially and ethnically minoritized groups: A scoping review. Lung Cancer. 2023;176: 46–55. doi:10.1016/j.lungcan.2022.12.016	No	Not meeting inclusion criteria	Title and abstract screening
3408	Nam JK, Piper ME, Tong Z, Li R, Yang JJ, Jorenby DE, et al. Dependence motives and use contexts that predicted smoking cessation and vaping cessation: A two-year longitudinal study with 13 waves. Drug Alcohol Depend. 2023;250: 110871. doi:10.1016/j.drugalcdep.2023.110871	No	Not meeting inclusion criteria	Title and abstract screening
3409	NAM Y., MIODOWNIK H.O.P.E., RAMANATHAN R., VISWANATHAN A., DWECK E.Z.R.A. A CASE OF THORACIC ENDOMETRIOSIS PRESENTING WITH TENSION CATAMENIAL HEMOTHORAX. Chest. 2023;164: A3720–A3721. doi:10.1016/j.chest.2023.07.2420	No	Not meeting inclusion criteria	Title and abstract screening
3410	Nantavithya C, Paulino AC, Liao K, Woodhouse KD, McGovern SL, Grosshans DR, et al. Observed-to-expected incidence ratios of second malignant neoplasms after radiation therapy for medulloblastoma: A Surveillance, Epidemiology, and End Results analysis. Cancer 0008543X. 2021;127: 2368–2375. doi:10.1002/cncr.33507	No	Not meeting inclusion criteria	Title and abstract screening
3411	Nardiello C., Morty R.E. World No Tobacco Day 2020. Am J Physiol - Lung Cell Mol Physiol. 2020;318: L1010–L1015. doi:10.1152/AJPLUNG.00110.2020	No	Not meeting inclusion criteria	Title and abstract screening
3412	Naskar S, Jakati PK. "Vaping:" Emergence of a New Paraphernalia. Indian J Psychol Med. 2017;39: 566–572. doi:10.4103/IJPSYM.IJPSYM_142_17	No	Not meeting inclusion criteria	Title and abstract screening
3413	Nasr P, Forsgren M, Balkhed W, Jönsson C, Dahlström N, Simonsson C, et al. A rapid, non-invasive, clinical surveillance for CachExia, sarcopenia, portal hypertension, and hepatocellular carcinoma in end-stage liver disease: the ACCESS-ESLD study protocol. BMC Gastroenterol. 2023;23: 1–13. doi:10.1186/s12876-023-03093-8	No	Not meeting inclusion criteria	Title and abstract screening
3414	Nathan R, Mupamombe CT, Elibol J, Case AA, Smith D, Hyland A, et al. Assessing Efficacy and Use Patterns of Medical Cannabis for Symptom Management in Elderly Cancer Patients. Am J Hosp Palliat Care. 2022; 10499091221110217. doi:10.1177/10499091221110217	No	Not meeting inclusion criteria	Title and abstract screening
3415	Nathan R, Mupamombe CT, Elibol J, Case AA, Smith D, Hyland A, et al. Assessing Efficacy and Use Patterns of Medical Cannabis for Symptom Management in Elderly Cancer Patients. Am J Hosp Palliat Med. 2023;40: 368–373. doi:10.1177/10499091221110217	No	Not meeting inclusion criteria	Title and abstract screening
3416	Navas-Nacher EL, Estrella ML, Giachello AL, Payne TJ, Kandi L, Walker, et al. Perceptions of electronic cigarettes among ethno-culturally diverse Latino adults in four US urban centers. Ethn Health. 2022;27: 1207–1221. doi:10.1080/13557858.2020.1844155	No	Not meeting inclusion criteria	Title and abstract screening
3417	Naveed A, Dang N, Gonzalez P, Choi SH, Mathew A, Wardle M, et al. E-Cigarette Dependence and Weight-Related Attitudes/Behaviors Associated With Eating Disorders in Adolescent Girls. Front Psychiatry. 2021;12: 713094. doi:10.3389/fpsyt.2021.713094	No	Not meeting inclusion criteria	Title and abstract screening
3418	Nawi AM, Ismail R, Ibrahim F, Hassan MR, Manaf MRA, Amit N, et al. Risk and protective factors of drug abuse among adolescents: a systematic review. BMC Public Health. 2021;21: 2088. doi:10.1186/s12889-021-11906-2	No	Not meeting inclusion criteria	Title and abstract screening
3419	Nayeri A, Middlekauff H. Vaping Instead of Cigarette Smoking: A Panacea or Just Another Form of Cardiovascular Risk?. Can J Cardiol. 2021;37: 690–698. doi:10.1016/j.cjca.2020.12.008	No	Not meeting inclusion criteria	Title and abstract screening
3420	NCT00625131. Nicotine Patch Pretreatment for Smoking Cessation in PTSD. 2008. Available: https://www.cochranelibrary.com/central/doi/10.1002/central/CN-02026533/full	No	Not meeting inclusion criteria	Title and abstract screening
3421	NCT00722124. S-Adenosyl-L-Methionine (SAMe) for Smoking Abstinence. 2008. Available: https://www.cochranelibrary.com/central/doi/10.1002/central/CN-01519910/full	No	Not meeting inclusion criteria	Title and abstract screening
3422	NCT01654107. Persistence-Targeted Smoking Cessation. 2012. Available: https://www.cochranelibrary.com/central/doi/10.1002/central/CN-01478407/full	No	Not meeting inclusion criteria	Title and abstract screening
3423	NCT01665066. Pharmacokinetic Profile of Toxic Substances and Nicotine in Electronic Cigarettes. 2012. Available: https://www.cochranelibrary.com/central/doi/10.1002/central/CN-01537058/full	No	Not meeting inclusion criteria	Title and abstract screening
3424	NCT01735487. CogEcig: cognitive Functioning and Electronic Cigarette. 2012. Available: https://www.cochranelibrary.com/central/doi/10.1002/central/CN-01538944/full	No	Not meeting inclusion criteria	Title and abstract screening
3425	NCT01782599. Electronic Cigarettes and Reactivity to Smoking Cues. 2013. Available: https://www.cochranelibrary.com/central/doi/10.1002/central/CN-01540253/full	No	Not meeting inclusion criteria	Title and abstract screening
3426	NCT01925781. e-Cigarettes Versus NRT Gum for Smoking Cessation. 2013. Available: https://www.cochranelibrary.com/central/doi/10.1002/central/CN-01489648/full	No	Not meeting inclusion criteria	Title and abstract screening
3427	NCT02004171. Electronic Cigarettes or Nicotine Inhaler for Smoking Cessation. 2013. Available: https://www.cochranelibrary.com/central/doi/10.1002/central/CN-01479553/full	No	Not meeting inclusion criteria	Title and abstract screening
3428	NCT02102100. The Effects of Menthol as Delivered by an Electronic Cigarette on the Desirability of Nicotine in Tobacco Users. 2013. Available: https://www.cochranelibrary.com/central/doi/10.1002/central/CN-01581303/full	No	Not meeting inclusion criteria	Title and abstract screening
3429	NCT02108626. Electronic Cigarettes in Daily Dependent Smokers. 2014. Available: https://www.cochranelibrary.com/central/doi/10.1002/central/CN-01544895/full	No	Not meeting inclusion criteria	Title and abstract screening
3430	NCT02179034. Flavors and E-cigarette Effects in Adolescent Smokers- STUDY 1. 2014. Available: https://www.cochranelibrary.com/central/doi/10.1002/central/CN-01546876/full	No	Not meeting inclusion criteria	Title and abstract screening
3431	NCT02188511. Effects of Electronic Cigarettes on Lung Biology. 2014. Available: https://www.cochranelibrary.com/central/doi/10.1002/central/CN-01547191/full	No	Not meeting inclusion criteria	Title and abstract screening
3432	NCT02357173. A Trial of E-cigarettes in Current Cigarette Smokers. 2015. Available: https://www.cochranelibrary.com/central/doi/10.1002/central/CN-01551982/full	No	Not meeting inclusion criteria	Title and abstract screening

3433	NCT02385227. Characterization of Biomarkers of Tobacco Exposure, Urge-to-Smoke Following Exclusive and Dual Ad Lib Use of Electronic Cigarettes. 2015. Available: https://www.cochranelibrary.com/central/doi/10.1002/central/CN-02027550/full	No	Not meeting inclusion criteria	Title and abstract screening
3434	NCT02416011. Smoking Cessation Self-Help for Dual Users of Tobacco Cigarettes and E-Cigarettes. 2015. Available: https://www.cochranelibrary.com/central/doi/10.1002/central/CN-01505709/full	No	Not meeting inclusion criteria	Title and abstract screening
3435	NCT02422914. Benefits of Tobacco Free Cigarette. 2015. Available: https://www.cochranelibrary.com/central/doi/10.1002/central/CN-02028151/full	No	Not meeting inclusion criteria	Title and abstract screening
3436	NCT02433015. Neurophysiological and Immunological Effects of the Transition From Combustible to Electronic Cigarettes. 2015. Available: https://www.cochranelibrary.com/central/doi/10.1002/central/CN-01506179/full	No	Not meeting inclusion criteria	Title and abstract screening
3437	NCT02470754. Clinical Pharmacology of Electronic Cigarettes. 2015. Available: https://www.cochranelibrary.com/central/doi/10.1002/central/CN-01584491/full	No	Not meeting inclusion criteria	Title and abstract screening
3438	NCT02487953. Electronic Nicotine Delivery Systems as a Smoking Cessation Treatment. 2015. Available: https://www.cochranelibrary.com/central/doi/10.1002/central/CN-01553359/full	No	Not meeting inclusion criteria	Title and abstract screening
3439	NCT02498145. Short Term Effects of Electronic Cigarettes in Tobacco Dependent Adults. 2015. Available: https://www.cochranelibrary.com/central/doi/10.1002/central/CN-01490983/full	No	Not meeting inclusion criteria	Title and abstract screening
3440	NCT02522156. Looming Vulnerability and Smoking Cessation Attempts. 2015. Available: https://www.cochranelibrary.com/central/doi/10.1002/central/CN-01491469/full	No	Not meeting inclusion criteria	Title and abstract screening
3441	NCT02590393. The Role of Nicotine and Non-Nicotine Alkaloids in E-Cigarette Use and Dependence. 2015. Available: https://www.cochranelibrary.com/central/doi/10.1002/central/CN-01493353/full	No	Not meeting inclusion criteria	Title and abstract screening
3442	NCT02596685. Effects of Electronic Cigarette Use on the Lungs. 2015. Available: https://www.cochranelibrary.com/central/doi/10.1002/central/CN-01493528/full	No	Not meeting inclusion criteria	Title and abstract screening
3443	NCT02628964. Electronic Cigarettes (E-cigarettes) as a Harm Reduction Strategy. 2015. Available: https://www.cochranelibrary.com/central/doi/10.1002/central/CN-01554362/full	No	Not meeting inclusion criteria	Title and abstract screening
3444	NCT02648178. Evaluation of Appeal and Impact of E-Cigarettes Among Chronic Smokers With Smoking-Related Cancers. 2016. Available: https://www.cochranelibrary.com/central/doi/10.1002/central/CN-01584627/full	No	Not meeting inclusion criteria	Title and abstract screening
3445	NCT02724241. E-Cigarettes and SNA. 2016. Available: https://www.cochranelibrary.com/central/doi/10.1002/central/CN-01556961/full	No	Not meeting inclusion criteria	Title and abstract screening
3446	NCT02740595. E-cigarettes and Blood Vessel Function. 2016. Available: https://www.cochranelibrary.com/central/doi/10.1002/central/CN-01557407/full	No	Not meeting inclusion criteria	Title and abstract screening
3447	NCT03035708. Human Laboratory Study of Varenicline for Alcohol Use Disorder. 2017. Available: https://www.cochranelibrary.com/central/doi/10.1002/central/CN-01561616/full	No	Not meeting inclusion criteria	Title and abstract screening
3448	NCT03050853. The Appeal and Impact of E-cigarettes in Smokers With SMI. 2017. Available: https://www.cochranelibrary.com/central/doi/10.1002/central/CN-01561943/full	No	Not meeting inclusion criteria	Title and abstract screening
3449	NCT03060083. Switching to Very Low Nicotine Content Cigarettes vs Reducing Cigarettes Per Day. 2017. Available: https://www.cochranelibrary.com/central/doi/10.1002/central/CN-01562156/full	No	Not meeting inclusion criteria	Title and abstract screening
3450	NCT03061253. E-cigarettes and Cardiovascular Function. 2017. Available: https://www.cochranelibrary.com/central/doi/10.1002/central/CN-02046871/full	No	Not meeting inclusion criteria	Title and abstract screening
3451	NCT03072628. E-cigarettes, Nicotine Inhaler, and Blood Vessel Function. 2017. Available: https://www.cochranelibrary.com/central/doi/10.1002/central/CN-01562429/full	No	Not meeting inclusion criteria	Title and abstract screening
3452	NCT03113136. Examination of Low Wattage and High Wattage E-Cigarettes. 2017. Available: https://www.cochranelibrary.com/central/doi/10.1002/central/CN-01575885/full	No	Not meeting inclusion criteria	Title and abstract screening
3453	NCT03164668. Menthol Flavored E-cigarette Use During a Simulated Ban of Menthol Cigarettes. 2017. Available: https://www.cochranelibrary.com/central/doi/10.1002/central/CN-01575267/full	No	Not meeting inclusion criteria	Title and abstract screening
3454	NCT03168191. Flavors and E-cigarette Effects in Adolescent Smokers- STUDY 2. 2017. Available: https://www.cochranelibrary.com/central/doi/10.1002/central/CN-01575326/full	No	Not meeting inclusion criteria	Title and abstract screening
3455	NCT03185546. REN-Project 2 Cigarette and E-cigarette Nicotine Content and E-liquid Flavors. 2017. Available: https://www.cochranelibrary.com/central/doi/10.1002/central/CN-01593126/full	No	Not meeting inclusion criteria	Title and abstract screening
3456	NCT03194256. Evaluating New Nicotine Standards for Cigarettes - Project 3. 2017. Available: https://www.cochranelibrary.com/central/doi/10.1002/central/CN-01495131/full	No	Not meeting inclusion criteria	Title and abstract screening
3457	NCT03249428. E-Cigarette Inner City RCT. 2017. Available: https://www.cochranelibrary.com/central/doi/10.1002/central/CN-01495731/full	No	Not meeting inclusion criteria	Title and abstract screening
3458	NCT03264313. Deep Transcranial Magnetic Stimulation (dTMS) to Induce Smoking Cessation. 2017. Available: https://www.cochranelibrary.com/central/doi/10.1002/central/CN-01496137/full	No	Not meeting inclusion criteria	Title and abstract screening
3459	NCT03272685. Impact of Very Low Nicotine Content Cigarettes in a Complex Marketplace. 2017. Available: https://www.cochranelibrary.com/central/doi/10.1002/central/CN-01563791/full	No	Not meeting inclusion criteria	Title and abstract screening
3460	NCT03402243. Effect of Banning Menthol Flavorant on Cigarette and E-Cigarette Use. 2018. Available: https://www.cochranelibrary.com/central/doi/10.1002/central/CN-01522091/full	No	Not meeting inclusion criteria	Title and abstract screening
3461	NCT03463837. Identify Biomarkers of Tobacco Exposure and Nicotine Uptake From JUUL 5% in Adult Smokers. 2018. Available: https://www.cochranelibrary.com/central/doi/10.1002/central/CN-01589687/full	No	Not meeting inclusion criteria	Title and abstract screening

3462	NCT03473483. Cigarette Harm Reduction With Scheduled Electronic Cigarette Use. 2018. Available: https://www.cochranelibrary.com/central/doi/10.1002/central/CN-01567397/full	No	Not meeting inclusion criteria	Title and abstract screening
3463	NCT03492463. The Role of Nicotine Dose and Route of Delivery in Affecting Adoption of E-cigarettes and Reducing Exposure to Toxic Combustion Products. 2018. Available: https://www.cochranelibrary.com/central/doi/10.1002/central/CN-01586111/full	No	Not meeting inclusion criteria	Title and abstract screening
3464	NCT03496116. The Influence of Electronic (ECIG) Heater Resistance on ECIG Acute Effects. 2018. Available: https://www.cochranelibrary.com/central/doi/10.1002/central/CN-01568019/full	No	Not meeting inclusion criteria	Title and abstract screening
3465	NCT03593239. Comparing the Pharmacokinetics of Nicotine Salt Based ENDS in Healthy Smokers. 2018. Available: https://www.cochranelibrary.com/central/doi/10.1002/central/CN-01661273/full	No	Not meeting inclusion criteria	Title and abstract screening
3466	NCT03603340. The ESTxENDS Trial- Effects of Using Electronic Nicotine Delivery Systems (ENDS/Vaporizer/E-cig) on Depression. 2018. Available: https://www.cochranelibrary.com/central/doi/10.1002/central/CN-01659337/full	No	Not meeting inclusion criteria	Title and abstract screening
3467	NCT03625986. Does Switching to Nicotine Containing Electronic Cigarettes Reduce Health Risk Markers. 2018. Available: https://www.cochranelibrary.com/central/doi/10.1002/central/CN-01626177/full	No	Not meeting inclusion criteria	Title and abstract screening
3468	NCT03634839. Effects of E-cigarette Flavors on Youth. 2018. Available: https://www.cochranelibrary.com/central/doi/10.1002/central/CN-01626307/full	No	Not meeting inclusion criteria	Title and abstract screening
3469	NCT03635333. Effects of E-Cigarette Flavors on Adults. 2018. Available: https://www.cochranelibrary.com/central/doi/10.1002/central/CN-01662319/full	No	Not meeting inclusion criteria	Title and abstract screening
3470	NCT03682900. Expanding the Click City Tobacco Prevention Program to Include E-cigarettes and Other Novel Tobacco Products. 2018. Available: https://www.cochranelibrary.com/central/doi/10.1002/central/CN-01706501/full	No	Not meeting inclusion criteria	Title and abstract screening
3471	NCT03700112. Clinical Study Comparing 7 ENDS Products and 1 Combustible Cigarette Using 2 Delivery Methods. 2018. Available: https://www.cochranelibrary.com/central/doi/10.1002/central/CN-01663960/full	No	Not meeting inclusion criteria	Title and abstract screening
3472	NCT03719391. United States Pre-Market Tobacco Application Pharmacokinetics. 2018. Available: https://www.cochranelibrary.com/central/doi/10.1002/central/CN-01794612/full	No	Not meeting inclusion criteria	Title and abstract screening
3473	NCT03905928. Establishing the Effect of Flavor on the Addictive Potential of Electronic Cigarettes. 2019. Available: https://www.cochranelibrary.com/central/doi/10.1002/central/CN-01931203/full	No	Not meeting inclusion criteria	Title and abstract screening
3474	NCT04047836. Vaping High vs. Low Nicotine E-Liquid. 2019. Available: https://www.cochranelibrary.com/central/doi/10.1002/central/CN-01966213/full	No	Not meeting inclusion criteria	Title and abstract screening
3475	NCT04058717. Low Nicotine Cigarettes Plus Electronic Cigarettes. 2019. Available: https://www.cochranelibrary.com/central/doi/10.1002/central/CN-01954657/full	No	Not meeting inclusion criteria	Title and abstract screening
3476	NCT04084210. Impact of Alternative Nicotine-Delivery Products on Combustible Cigarette Use. 2019. Available: https://www.cochranelibrary.com/central/doi/10.1002/central/CN-01984171/full	No	Not meeting inclusion criteria	Title and abstract screening
3477	NCT04090879. Low Nicotine Content Cigarettes in Vulnerable Populations: affective Disorders. 2019. Available: https://www.cochranelibrary.com/central/doi/10.1002/central/CN-01984278/full	No	Not meeting inclusion criteria	Title and abstract screening
3478	NCT04092101. Low Nicotine Content Cigarettes in Vulnerable Populations: opioid Use Disorder. 2019. Available: https://www.cochranelibrary.com/central/doi/10.1002/central/CN-01984308/full	No	Not meeting inclusion criteria	Title and abstract screening
3479	NCT04092387. Low Nicotine Content Cigarettes in Vulnerable Populations: women of Reproductive Age. 2019. Available: https://www.cochranelibrary.com/central/doi/10.1002/central/CN-01984316/full	No	Not meeting inclusion criteria	Title and abstract screening
3480	NCT04170907. Pharmacokinetics and Pharmacodynamics of Different Nicotine Salt Concentration Vape System Pods and Free-base Nicotine. 2019. Available: https://www.cochranelibrary.com/central/doi/10.1002/central/CN-02010010/full	No	Not meeting inclusion criteria	Title and abstract screening
3481	NCT04179617. Impact of New Product Standards for JUUL Among Dual JUUL/Combusted Cigarette Users. 2019. Available: https://www.cochranelibrary.com/central/doi/10.1002/central/CN-02078971/full	No	Not meeting inclusion criteria	Title and abstract screening
3482	NCT04188197. Assisting Smokers to Switch to a JUUL E-Cigarette by Devaluing Combustible Cigarettes. 2019. Available: https://www.cochranelibrary.com/central/doi/10.1002/central/CN-02052943/full	No	Not meeting inclusion criteria	Title and abstract screening
3483	NCT04231539. Pharmacokinetics, Subjective Effects, and Abuse Liability of Nicotine Salt-Based Vaping Products With Tobacco or Unflavored E-liquids, SALTVAPE Study. 2020. Available: https://www.cochranelibrary.com/central/doi/10.1002/central/CN-02088157/full	No	Not meeting inclusion criteria	Title and abstract screening
3484	NCT04250727. Switching to Potential Reduced Exposure Products in Adult Smokers. 2020. Available: https://www.cochranelibrary.com/central/doi/10.1002/central/CN-02079952/full	No	Not meeting inclusion criteria	Title and abstract screening
3485	NCT04258852. Understanding Factors That Influence Electronic Cigarette Nicotine Delivery Through PET Imaging of Beta-2 Nicotinic Acetylcholine Receptors. 2020. Available: https://www.cochranelibrary.com/central/doi/10.1002/central/CN-02080167/full	No	Not meeting inclusion criteria	Title and abstract screening
3486	NCT04262817. The Interaction of Flavor With Nicotine Form in Adult Smokers. 2020. Available: https://www.cochranelibrary.com/central/doi/10.1002/central/CN-02080264/full	No	Not meeting inclusion criteria	Title and abstract screening
3487	NCT04286295. Pilot RCT of Cytisine vs Combination NRT in Relapsed Smokers. 2020. Available: https://www.cochranelibrary.com/central/doi/10.1002/central/CN-02088556/full	No	Not meeting inclusion criteria	Title and abstract screening
3488	NCT04429568. THC Crossover Study. 2020. Available: https://www.cochranelibrary.com/central/doi/10.1002/central/CN-02125510/full	No	Not meeting inclusion criteria	Title and abstract screening
3489	NCT04449510. Short-Term Cardiovascular Effects of E-Cigarettes: influence of E-Liquid pH. 2020. Available: https://www.cochranelibrary.com/central/doi/10.1002/central/CN-02133954/full	No	Not meeting inclusion criteria	Title and abstract screening
3490	NCT04521647. Effects of Nicotine in E-cigarettes on Smoking Behaviors. 2020. Available: https://www.cochranelibrary.com/central/doi/10.1002/central/CN-02162996/full	No	Not meeting inclusion criteria	Title and abstract screening

3491	NCT04550026. Cardiovascular Effects of Heated Tobacco Products (HTP). 2020. Available: https://www.cochranelibrary.com/central/doi/10.1002/central/CN-02163688/full	No	Not meeting inclusion criteria	Title and abstract screening
3492	NCT04568395. Acute Effects of TCIG vs ECIG in PLWH. 2020. Available: https://www.cochranelibrary.com/central/doi/10.1002/central/CN-02181560/full	No	Not meeting inclusion criteria	Title and abstract screening
3493	NCT04602494. Varenicline For Vaping Cessation In Non Smoker Vaper Adolescents. 2020. Available: https://www.cochranelibrary.com/central/doi/10.1002/central/CN-02183921/full	No	Not meeting inclusion criteria	Title and abstract screening
3494	NCT04645914. Influence of the Consumption of Conventional and Electric Cigarettes on Skin Circulation. 2020. Available: https://www.cochranelibrary.com/central/doi/10.1002/central/CN-02205944/full	No	Not meeting inclusion criteria	Title and abstract screening
3495	NCT04646668. Comparative Abuse Liability Among African American and White Smokers. 2020. Available: https://www.cochranelibrary.com/central/doi/10.1002/central/CN-02205964/full	No	Not meeting inclusion criteria	Title and abstract screening
3496	NCT04696380. Do Flavors Increase the Addiction Potential of Nicotine? 2021. Available: https://www.cochranelibrary.com/central/doi/10.1002/central/CN-02233677/full	No	Not meeting inclusion criteria	Title and abstract screening
3497	NCT04709471. E-cigarette Nicotine Study. 2021. Available: https://www.cochranelibrary.com/central/doi/10.1002/central/CN-02234000/full	No	Not meeting inclusion criteria	Title and abstract screening
3498	NCT04866277. Experimental Study to Reduce Low Birthweight. 2021. Available: https://www.cochranelibrary.com/central/doi/10.1002/central/CN-02289442/full	No	Not meeting inclusion criteria	Title and abstract screening
3499	NCT04898075. Quit Nicotine: e-Cig Cessation Intervention. 2021. Available: https://www.cochranelibrary.com/central/doi/10.1002/central/CN-02277808/full	No	Not meeting inclusion criteria	Title and abstract screening
3500	NCT04907136. Developing and Testing Health Warning Labels on the ENDS Device. 2021. Available: https://www.cochranelibrary.com/central/doi/10.1002/central/CN-02277993/full	No	Not meeting inclusion criteria	Title and abstract screening
3501	NCT04943822. The Effectiveness of APP Intervention on Home Self-care Ability and Quality of Life of Patients With Intestinal Stoma. 2021. Available: https://www.cochranelibrary.com/central/doi/10.1002/central/CN-02289975/full	No	Not meeting inclusion criteria	Title and abstract screening
3502	NCT04946825. Quit Smoking Study for People Who Use E-Cigarettes. 2021. Available: https://www.cochranelibrary.com/central/doi/10.1002/central/CN-02279348/full	No	Not meeting inclusion criteria	Title and abstract screening
3503	NCT04974580. Research and Innovation to Stop E-cigarette/Vaping in Young Adults. 2021. Available: https://www.cochranelibrary.com/central/doi/10.1002/central/CN-02296946/full	No	Not meeting inclusion criteria	Title and abstract screening
3504	NCT04996940. Comparative Acceptability of Tobacco and Menthol Flavored E-cigarettes. 2021. Available: https://www.cochranelibrary.com/central/doi/10.1002/central/CN-02306989/full	No	Not meeting inclusion criteria	Title and abstract screening
3505	NCT05037656. Testing a School-Based E-cigarette, Tobacco, and Betel (Areca) Nut Use Prevention Curriculum for Guam Youths. 2021. Available: https://www.cochranelibrary.com/central/doi/10.1002/central/CN-02307758/full	No	Not meeting inclusion criteria	Title and abstract screening
3506	NCT05040386. Nurse Coach-Led Early Palliative Care for Older Adults With COPD and Their Care Partners: the Project EPIC RCT. 2021. Available: https://www.cochranelibrary.com/central/doi/10.1002/central/CN-02307827/full	No	Not meeting inclusion criteria	Title and abstract screening
3507	NCT05048836. Healthy Food First. 2021. Available: https://www.cochranelibrary.com/central/doi/10.1002/central/CN-02331652/full	No	Not meeting inclusion criteria	Title and abstract screening
3508	NCT05057078. The Effect of Mindfulness Program on Spiritual Well-BeingBreast Cancer Patients. 2021. Available: https://www.cochranelibrary.com/central/doi/10.1002/central/CN-02331811/full	No	Not meeting inclusion criteria	Title and abstract screening
3509	NCT05110872. Cigarette and E-cigarette Flavors Manipulations in the Experimental Tobacco Marketplace. 2021. Available: https://www.cochranelibrary.com/central/doi/10.1002/central/CN-02352633/full	No	Not meeting inclusion criteria	Title and abstract screening
3510	NCT05112562. Attentional Bias Modification Training for E-cigarette Users. 2021. Available: https://www.cochranelibrary.com/central/doi/10.1002/central/CN-02341591/full	No	Not meeting inclusion criteria	Title and abstract screening
3511	NCT05129592. Can Exposure to Brief Messages Correct Misperceptions? 2021. Available: https://www.cochranelibrary.com/central/doi/10.1002/central/CN-02353074/full	No	Not meeting inclusion criteria	Title and abstract screening
3512	NCT05144542. Risk and Benefits of Electronic Cigarettes to Older Smokers at High Risk for Lung Cancer. 2021. Available: https://www.cochranelibrary.com/central/doi/10.1002/central/CN-02353484/full	No	Not meeting inclusion criteria	Title and abstract screening
3513	NCT05145543. Effect of Topically Administered Levobupivacaine- Fentanyl Versus Levobupivacaine- Dexamethasone Combination in the Control of Pain After Endoscopic Sinus Surgery. 2021. Available: https://www.cochranelibrary.com/central/doi/10.1002/central/CN-02353520/full	No	Not meeting inclusion criteria	Title and abstract screening
3514	NCT05162781. Transferring Speed of Processing Gains to Everyday Cognitive Tasks After Stroke. 2021. Available: https://www.cochranelibrary.com/central/doi/10.1002/central/CN-02353914/full	No	Not meeting inclusion criteria	Title and abstract screening
3515	NCT05205382. Assessing the Effect of Nicotine Reduction on ENDS User's Addiction and Exposures. 2022. Available: https://www.cochranelibrary.com/central/doi/10.1002/central/CN-02367153/full	No	Not meeting inclusion criteria	Title and abstract screening
3516	NCT05206435. Methadone-Maintained Smokers Switching to E-Cigarettes. 2022. Available: https://www.cochranelibrary.com/central/doi/10.1002/central/CN-02367178/full	No	Not meeting inclusion criteria	Title and abstract screening
3517	NCT05210998. Respiratory Muscle Training in Obstructive Sleep Apnea Syndrome. 2022. Available: https://www.cochranelibrary.com/central/doi/10.1002/central/CN-02367261/full	No	Not meeting inclusion criteria	Title and abstract screening
3518	NCT05211882. ENABLE (Educate, Nurture, Advise, Before Life Ends) Intervention for Heart Failure (HF) Patients and Their Caregivers in Singapore. 2022. Available: https://www.cochranelibrary.com/central/doi/10.1002/central/CN-02367284/full	No	Not meeting inclusion criteria	Title and abstract screening
3519	NCT05212220. Effectiveness of Ecological Momentary Assessment Based Smoking Cessation Intervention. 2022. Available: https://www.cochranelibrary.com/central/doi/10.1002/central/CN-02367288/full	No	Not meeting inclusion criteria	Title and abstract screening

3520	NCT05248321. Precise Delivery of Tranexamic Acid to Enhance Endoscopic Hemostasis for Peptic Ulcer Bleeding. 2022. Available: https://www.cochranelibrary.com/central/doi/10.1002/central/CN-02381713/full	No	Not meeting inclusion criteria	Title and abstract screening
3521	NCT05257629. Aggressive Smoking Cessation Trial (ASAP). 2022. Available: https://www.cochranelibrary.com/central/doi/10.1002/central/CN-02381963/full	No	Not meeting inclusion criteria	Title and abstract screening
3522	NCT05260827. The Effects of Heated Tobacco Products Use on Lung Function and Volatile Organic Compounds in Exhaled Air. 2022. Available: https://www.cochranelibrary.com/central/doi/10.1002/central/CN-02382045/full	No	Not meeting inclusion criteria	Title and abstract screening
3523	NCT05278065. E-cigarettes for Harm Reduction in Adults With Asthma. 2022. Available: https://www.cochranelibrary.com/central/doi/10.1002/central/CN-02382419/full	No	Not meeting inclusion criteria	Title and abstract screening
3524	NCT05327439. Using ANDS to Reduce Harm for Low SES Cigarette Smokers. 2022. Available: https://www.cochranelibrary.com/central/doi/10.1002/central/CN-02388329/full	No	Not meeting inclusion criteria	Title and abstract screening
3525	NCT05338801. Effect of Menthol on ENDS Users' Addiction and Exposure. 2022. Available: https://www.cochranelibrary.com/central/doi/10.1002/central/CN-02398286/full	No	Not meeting inclusion criteria	Title and abstract screening
3526	NCT05342194. Toripalimab Plus Lenvatinib and Gemcitabine-based Chemotherapy in 1L Treatment of Advanced ICC: a Phase III Study. 2022. Available: https://www.cochranelibrary.com/central/doi/10.1002/central/CN-02398368/full	No	Not meeting inclusion criteria	Title and abstract screening
3527	NCT05344261. Effect of Prophylactic TMR and RPNI on Neuroma and Phantom Limb Pain. 2022. Available: https://www.cochranelibrary.com/central/doi/10.1002/central/CN-02398431/full	No	Not meeting inclusion criteria	Title and abstract screening
3528	NCT05364463. Effect of Reactive Hyperemia and Ultrasound-guided Puncture on the Success Rate of Radial Artery Cannulation (RadialHUS). 2022. Available: https://www.cochranelibrary.com/central/doi/10.1002/central/CN-02398842/full	No	Not meeting inclusion criteria	Title and abstract screening
3529	NCT05366790. A Brief Digital Screening Tool to Address Tobacco and E-cigarette Use in Pediatric Medical Care. 2022. Available: https://www.cochranelibrary.com/central/doi/10.1002/central/CN-02398882/full	No	Not meeting inclusion criteria	Title and abstract screening
3530	NCT05367492. Varenicline for Nicotine Vaping Cessation in Adolescents. 2022. Available: https://www.cochranelibrary.com/central/doi/10.1002/central/CN-02395792/full	No	Not meeting inclusion criteria	Title and abstract screening
3531	NCT05379712. Nutritional Supplementation in Head and Neck Cancers. 2022. Available: https://www.cochranelibrary.com/central/doi/10.1002/central/CN-02397285/full	No	Not meeting inclusion criteria	Title and abstract screening
3532	NCT05421962. Analgosedation for Elective Colonoscopy. 2022. Available: https://www.cochranelibrary.com/central/doi/10.1002/central/CN-02422993/full	No	Not meeting inclusion criteria	Title and abstract screening
3533	NCT05430334. Assess the Influence of Nicotine Flux and Nicotine Form on Subjective Effects Related to Dependency. 2022. Available: https://www.cochranelibrary.com/central/doi/10.1002/central/CN-02423237/full	No	Not meeting inclusion criteria	Title and abstract screening
3534	NCT05432830. Sex Differences in E-cig Perception: study 1. 2022. Available: https://www.cochranelibrary.com/central/doi/10.1002/central/CN-02413260/full	No	Not meeting inclusion criteria	Title and abstract screening
3535	NCT05451641. Blood Flow Restriction Resistance Training Intervention on Vascular Function. 2022. Available: https://www.cochranelibrary.com/central/doi/10.1002/central/CN-02423711/full	No	Not meeting inclusion criteria	Title and abstract screening
3536	NCT05473585. Impact of New Standards for Tobacco Products Among Dual E-Cigarette/Combusted Cigarette Users - Project 2. 2022. Available: https://www.cochranelibrary.com/central/doi/10.1002/central/CN-02431241/full	No	Not meeting inclusion criteria	Title and abstract screening
3537	NCT05510154. COPD E-cigarette Topography Training. 2022. Available: https://www.cochranelibrary.com/central/doi/10.1002/central/CN-02456792/full	No	Not meeting inclusion criteria	Title and abstract screening
3538	NCT05516277. Insomnia Treatment and Cardiometabolic Health in Older Adults With Posttraumatic Stress Disorder. 2022. Available: https://www.cochranelibrary.com/central/doi/10.1002/central/CN-02456923/full	No	Not meeting inclusion criteria	Title and abstract screening
3539	NCT05525078. Quit or Switch: e-cigarette Study. 2022. Available: https://www.cochranelibrary.com/central/doi/10.1002/central/CN-02457112/full	No	Not meeting inclusion criteria	Title and abstract screening
3540	NCT05541497. Varenicline for Treatment of E-cigarette Dependence. 2022. Available: https://www.cochranelibrary.com/central/doi/10.1002/central/CN-02460793/full	No	Not meeting inclusion criteria	Title and abstract screening
3541	NCT05545761. The Effect of Music Therapy on Sleep Quality and Depression in Menopausal Women. 2022. Available: https://www.cochranelibrary.com/central/doi/10.1002/central/CN-02475022/full	No	Not meeting inclusion criteria	Title and abstract screening
3542	NCT05575349. Effects of Mandala Coloring on Anxiety and Quality of Life of Women in the Climacteric Period. 2022. Available: https://www.cochranelibrary.com/central/doi/10.1002/central/CN-02471415/full	No	Not meeting inclusion criteria	Title and abstract screening
3543	NCT05582642. Respiratory Muscles After Inspiratory Muscle Training After COVID-19. 2022. Available: https://www.cochranelibrary.com/central/doi/10.1002/central/CN-02475275/full	No	Not meeting inclusion criteria	Title and abstract screening
3544	NCT05658471. Pharmacokinetics and Pharmacodynamics of Nicotine With Use of Standardized Research Electronic Cigarette (SREC). 2022. Available: https://www.cochranelibrary.com/central/doi/10.1002/central/CN-02507517/full	No	Not meeting inclusion criteria	Title and abstract screening
3545	NCT05664867. Implementation of Population Breast Cancer Genetic Services in Federally Qualified Health Centers (FQHC). 2022. Available: https://www.cochranelibrary.com/central/doi/10.1002/central/CN-02509872/full	No	Not meeting inclusion criteria	Title and abstract screening
3546	NCT05706610. A Pilot Feasibility Trial of a Tailored Intervention to Improve Adherence in Adolescents and Young Adults With Cancer. 2023. Available: https://www.cochranelibrary.com/central/doi/10.1002/central/CN-02518139/full	No	Not meeting inclusion criteria	Title and abstract screening
3547	NCT05743530. Effect of Perioperative Neurostimulation in Patients Undergoing Whipple Operation (Pancreaticoduodenectomy) on Acute Postsurgical Inflammatory Response. 2023. Available: https://www.cochranelibrary.com/central/doi/10.1002/central/CN-02526969/full	No	Not meeting inclusion criteria	Title and abstract screening
3548	NCT05753397. ITP Block: single or Multiple Injection? 2023. Available: https://www.cochranelibrary.com/central/doi/10.1002/central/CN-02528786/full	No	Not meeting inclusion criteria	Title and abstract screening

3549	NCT05761353. Endermologie Versus Negative Pressure Therapy on Postmastectomy Lymphedema. 2023. Available: https://www.cochranelibrary.com/central/doi/10.1002/central/CN-02531353/full	No	Not meeting inclusion criteria	Title and abstract screening
3550	NCT05763836. Comparison of the Analgesic Effects of Scalp Nerve Block and Intravenous Ibuprofen Applications Under the Guidance of Nociception Level Index (NoL) in Patients Undergoing Elective Supratentorial Craniotomy. 2023. Available: https://www.cochranelibrary.com/central/doi/10.1002/central/CN-02531419/full	No	Not meeting inclusion criteria	Title and abstract screening
3551	NCT05772845. Vaping and Smoking Withdrawal Project. 2023. Available: https://www.cochranelibrary.com/central/doi/10.1002/central/CN-02535948/full	No	Not meeting inclusion criteria	Title and abstract screening
3552	NCT05819346. Digital Lifestyle Intervention for Lung Cancer Survivors After Inpatient Rehabilitation. 2023. Available: https://www.cochranelibrary.com/central/doi/10.1002/central/CN-02555425/full	No	Not meeting inclusion criteria	Title and abstract screening
3553	NCT05829070. Improving Health Insurance Literacy Among Young Adult Cancer Survivors. 2023. Available: https://www.cochranelibrary.com/central/doi/10.1002/central/CN-02559672/full	No	Not meeting inclusion criteria	Title and abstract screening
3554	NCT05836051. Testing the Effect of ENDS Flavors on Neurotransmission. 2023. Available: https://www.cochranelibrary.com/central/doi/10.1002/central/CN-02554102/full	No	Not meeting inclusion criteria	Title and abstract screening
3555	NCT05849051. Little Cigar and Cigarillo Warnings Among US Adults Who Use LCCs. 2023. Available: https://www.cochranelibrary.com/central/doi/10.1002/central/CN-02559987/full	No	Not meeting inclusion criteria	Title and abstract screening
3556	NCT05874661. FOA on Sit to Stand in Individuals Post Stroke. 2023. Available: https://www.cochranelibrary.com/central/doi/10.1002/central/CN-02564823/full	No	Not meeting inclusion criteria	Title and abstract screening
3557	NCT05897242. A Smartphone Application (ACT on Vaping) for Vaping Cessation in Young Adults. 2023. Available: https://www.cochranelibrary.com/central/doi/10.1002/central/CN-02568084/full	No	Not meeting inclusion criteria	Title and abstract screening
3558	NCT05911243. Feasibility of Auricular Acupressure for Appetite and Weight in Patients With Stage II-IV Gastric, Esophageal, and Pancreatic Cancer. 2023. Available: https://www.cochranelibrary.com/central/doi/10.1002/central/CN-02573503/full	No	Not meeting inclusion criteria	Title and abstract screening
3559	NCT05919381. Gentuximab Combined With Paclitaxel Compared With Placebo Combined With Paclitaxel for Gastric Adenocarcinoma. 2023. Available: https://www.cochranelibrary.com/central/doi/10.1002/central/CN-02577724/full	No	Not meeting inclusion criteria	Title and abstract screening
3560	NCT05932745. Effects of Novel E-cigarette Constituents on Adults TCORS 3.0. 2023. Available: https://www.cochranelibrary.com/central/doi/10.1002/central/CN-02578768/full	No	Not meeting inclusion criteria	Title and abstract screening
3561	NCT05938530. Sirolimus Drug-coated Balloon Versus Stent Graft. 2023. Available: https://www.cochranelibrary.com/central/doi/10.1002/central/CN-02580838/full	No	Not meeting inclusion criteria	Title and abstract screening
3562	NCT05946707. Effects of Oxygen Supply After Lung Isolation in Thoracic Surgery. 2023. Available: https://www.cochranelibrary.com/central/doi/10.1002/central/CN-02582746/full	No	Not meeting inclusion criteria	Title and abstract screening
3563	NCT05962229. Comparative Pharmacokinetics and Pharmacodynamics of Synthetic Nicotine. 2023. Available: https://www.cochranelibrary.com/central/doi/10.1002/central/CN-02585142/full	No	Not meeting inclusion criteria	Title and abstract screening
3564	NCT05994209. Testing the Feasibility and Acceptability of Social Media and Digital Therapeutics to Decrease Vaping Behaviors. 2023. Available: https://www.cochranelibrary.com/central/doi/10.1002/central/CN-02590047/full	No	Not meeting inclusion criteria	Title and abstract screening
3565	NCT05999383. Understanding the Clinical Pharmacology of Marijuana-Tobacco Co-administration. 2023. Available: https://www.cochranelibrary.com/central/doi/10.1002/central/CN-02592209/full	No	Not meeting inclusion criteria	Title and abstract screening
3566	NCT06021613. The MARY-JANE Cannabis and Heart Rhythm Trial. 2023. Available: https://www.cochranelibrary.com/central/doi/10.1002/central/CN-02595509/full	No	Not meeting inclusion criteria	Title and abstract screening
3567	NCT06027723. Nicotine and Pavlovian Bias. 2023. Available: https://www.cochranelibrary.com/central/doi/10.1002/central/CN-02595633/full	No	Not meeting inclusion criteria	Title and abstract screening
3568	NCT06077240. Effects of E-cigs vs Pouches on Cigarette Smoking and Addiction. 2023. Available: https://www.cochranelibrary.com/central/doi/10.1002/central/CN-02603488/full	No	Not meeting inclusion criteria	Title and abstract screening
3569	NCT06082349. The N-LVA Study: RCT Comparing LVA vs. Sham Surgery in Cancer-related Lymphedema. 2023. Available: https://www.cochranelibrary.com/central/doi/10.1002/central/CN-02603270/full	No	Not meeting inclusion criteria	Title and abstract screening
3570	NCT06087328. CAN-DOSE Study: cessation With Augmented Nicotine for Dual Use Of Smoking and E-cigarettes. 2023. Available: https://www.cochranelibrary.com/central/doi/10.1002/central/CN-02606204/full	No	Not meeting inclusion criteria	Title and abstract screening
3571	NCT06098235. Oriental Intervention for Enhanced Neurocognitive Health (Orient) Diet in Populations With High Risk of Stroke (ENDS). 2023. Available: https://www.cochranelibrary.com/central/doi/10.1002/central/CN-02608026/full	No	Not meeting inclusion criteria	Title and abstract screening
3572	NCT06131723. UCLA Health Patient Health Tickler Email. 2023. Available: https://www.cochranelibrary.com/central/doi/10.1002/central/CN-02626737/full	No	Not meeting inclusion criteria	Title and abstract screening
3573	NCT06136832. Pneumonia Vaccine Education Intervention Study. 2023. Available: https://www.cochranelibrary.com/central/doi/10.1002/central/CN-02626823/full	No	Not meeting inclusion criteria	Title and abstract screening
3574	NCT06149494. RCT of Vapendavir in Patients With COPD and Human Rhinovirus/Enterovirus Upper Respiratory Infection. 2023. Available: https://www.cochranelibrary.com/central/doi/10.1002/central/CN-02631227/full	No	Not meeting inclusion criteria	Title and abstract screening
3575	Neale J, Parkin S, Hermann L, Metrebian N, Roberts E, Robson D, et al. Substance use and homelessness: A longitudinal interview study conducted during COVID-19 with implications for policy and practice. Int J Drug Policy. 2022;108: 103818. doi:10.1016/j.drugpo.2022.103818	No	Not meeting inclusion criteria	Title and abstract screening
3576	Neczypor EW, Mears MJ, Ghosh A, Sassano MF, Gumina RJ, Wold LE, et al. E-Cigarettes and Cardiopulmonary Health: Review for Clinicians. Circulation. 2022;145: 219–232. doi:10.1161/CIRCULATIONAHA.121.056777	No	Not meeting inclusion criteria	Title and abstract screening
3577	Needle JS, Friebert S, Thompkins JD, Grossoehme DH, Baker JN, Jiang J, et al. Effect of the Family-Centered Advance Care Planning for Teens with Cancer Intervention on Sustainability of Congruence About End-of-Life Treatment Preferences: A Randomized Clinical Trial. JAMA Netw Open. 2022;5: e2220696–e2220696. doi:10.1001/jamanetworkopen.2022.20696	No	Not meeting inclusion criteria	Title and abstract screening

3578	Neisler J., Reitzel L.R., Garey L., Kenzdor D.E., Hebert E.T., Vijayaraghavan M., et al. Concurrent nicotine and tobacco product use among homeless smokers and associations with cigarette dependence and other factors related to quitting. Drug Alcohol Depend. 2018;185: 133–140. doi:10.1016/j.drugalcdep.2017.12.012	No	Not meeting inclusion criteria	Title and abstract screening
3579	Nemeh H., Coba V., Chulkov M., Gupta A., Yeldo N., Chamogeorgakis T., et al. Lung Transplantation for the Treatment of Vaping-Induced, Irreversible, End-Stage Lung Injury. Ann Thorac Surg. 2021;111: e353–e355. doi:10.1016/j.athoracsur.2020.07.097	No	Not meeting inclusion criteria	Title and abstract screening
3580	NERY T., BISPO N., MARTINS S.M. EXPLORATORY EPIDEMIOLOGIC OVERVIEW OF TOBACCO USE, ELECTRONIC CIGARETTE USE, AND MORTALITY BY COPD IN BRAZIL: IMPORTANCE OF USE OF SURVEYS WITH OPEN DATA. Chest. 2022;162: A2639. doi:10.1016/j.chest.2022.08.2156	No	Not meeting inclusion criteria	Title and abstract screening
3581	Nestor LJ, McCabe E, Jones J, Clancy L, Garavan H. Shared and divergent neural reactivity to non-drug operant response outcomes in current smokers and ex-smokers. Brain Res. 2018;1680: 54–61. doi:10.1016/j.brainres.2017.12.003	No	Not meeting inclusion criteria	Title and abstract screening
3582	Nettboy S., Amen J., Cocoros A., Varela-Margolles D., Khanna A., Mazzei T., et al. WHEN HOOFBEATS DO MEAN ZEBRA: EVALI WITH PNEUMATOCELE DURING PEAK COVID-19. Chest. 2021;160: A819–A820. doi:10.1016/j.chest.2021.07.771	No	Not meeting inclusion criteria	Title and abstract screening
3583	Neuberger M. Tobacco, Nicotine and Health. Med Kaunas Lith. 2021;57. doi:10.3390/medicina57080740	No	Not meeting inclusion criteria	Title and abstract screening
3584	Neums L, Koestler DC, Xia Q, Hu J, Patel S, Bell-Glenn S, et al. Assessing equivalent and inverse change in genes between diverse experiments. Front Bioinform. 2022;2: 893032. doi:10.3389/fbinf.2022.893032	No	Not meeting inclusion criteria	Title and abstract screening
3585	Newcombe KV, Dobbs PD, Oehlers JS, Dunlap CM, Cheney MK. College Students' Reasons for Using JUULs. Am J Health Promot. 2021;35: 835–840. doi:10.1177/0890117121992292	No	Not meeting inclusion criteria	Title and abstract screening
3586	Ng D.Q., Miller K.A., Thomas S.M., Freyer D.R., Milam J., Chan A. Adverse lifestyle behaviors among childhood cancer survivors with cognitive impairment: The Project Forward Cohort. J Clin Oncol. 2021;39. doi:10.1200/JCO.2020.39.28_suppl.138	No	Not meeting inclusion criteria	Title and abstract screening
3587	Ng DQ, Ritt-Olson A, Freyer DR, Miller KA, Thomas SM, Milam J, et al. Substance Use Among Young Adult Survivors of Childhood Cancer With Cognitive Impairment: An Analysis of the Project Forward Cohort. JCO Oncol Pr. 2023;19: e345–e354. doi:10.1200/OP.22.00458	No	Not meeting inclusion criteria	Title and abstract screening
3588	Ng Fat L., Scholes S., Mindell J.S. MOTIVATION TO QUIT SMOKING AND CHANGES IN CIGARETTE CONSUMPTION, AMONG SMOKERS WHO USE E-CIGARETTES, FINDINGS FROM THE HEALTH SURVEY FOR ENGLAND. J Epidemiol Community Health. 2017;71: A33. doi:10.1136/jech-2017-SSMAbstracts.65	No	Not meeting inclusion criteria	Title and abstract screening
3589	Ng G, Attwells S, Zawertailo L. The development and validation of an electronic nicotine delivery system (ENDS) image cue stimulus set. Drug Alcohol Depend. 2022;236: 109496. doi:10.1016/j.drugalcdep.2022.109496	No	Not meeting inclusion criteria	Title and abstract screening
3590	Ng G., Attwells S., Selby P., Zawertailo L. Effectiveness of Non-Nicotinic E-Cigarettes to Reduce Cue- and Abstinence-Induced Cigarette Craving in Non-Treatment Seeking Daily Dependent Smokers. Psychopharmacology (Berl). 2021;238: 1461–1472. doi:10.1007/s00213-021-05772-4	No	Not meeting inclusion criteria	Title and abstract screening
3591	Ng GWY. E-cigarettes: Biomarkers of exposure and the development and validation of a research toolkit. Diss Abstr Int Sect B Sci Eng. 2022;83: No-Specified.	No	Not meeting inclusion criteria	Title and abstract screening
3592	Ngene-Kinoti RM, Kyalo M, Odero J. NURSES' KNOWLEDGE AND ATTITUDE REGARDING END-OF-LIFE CARE AMONG ADVANCED CANCER PATIENTS IN KENYATTA NATIONAL HOSPITAL. East Afr Med J. 2022;99: 4488–4496.	No	Not meeting inclusion criteria	Title and abstract screening
3593	Nguyen HV, Mital S. Effects of e-cigarette use on mental health among youths: quasi-experimental evidence from Canada. Addiction. 2022;117: 2673–2682. doi:10.1111/add.15943	No	Not meeting inclusion criteria	Title and abstract screening
3594	Nguyen M., Santos C. Sarcoidosis, the great mimicker; An unusual presentation. Respirology. 2023;28: 200–201. doi:10.1111/resp.14460	No	Not meeting inclusion criteria	Title and abstract screening
3595	Nguyen N, Kapiteni K, Straus ER, Guydish J. Factors associated with dual and polytobacco use among people in residential substance use disorder treatment. Am J Addict. 2021;30: 496–504. doi:10.1111/ajad.13206	No	Not meeting inclusion criteria	Title and abstract screening
3596	Nguyen N, Peyser ND, Olgin JE, Pletcher MJ, Beatty AL, Modrow MF, et al. Associations between tobacco and cannabis use and anxiety and depression among adults in the United States: Findings from the COVID-19 citizen science study. PLoS One. 2023;18: e0289058. doi:10.1371/journal.pone.0289058	No	Not meeting inclusion criteria	Title and abstract screening
3597	Nguyen N, Thrul J, Neilands TB, Ling PM. Associations Between Product Type and Intensity of Tobacco and Cannabis Co-use on the Same Day Among Young Adult Smokers: Smartphone-Based Daily-Diary Study. JMIR Mhealth Uhealth. 2023;11: e40736. doi:10.2196/40736	No	Not meeting inclusion criteria	Title and abstract screening
3598	Nguyen N, Mathur Gaiha S, Halpern-Felsher B. Self-reported changes in cannabis vaping among US adolescents and young adults early in the COVID-19 pandemic. Prev Med Rep. 2021;24: 101654. doi:10.1016/j.pmedr.2021.101654	No	Not meeting inclusion criteria	Title and abstract screening
3599	Nguyen S., Harb A., Luna R., Arriaga K., Marshall J., Abraham A., et al. 043 Electronic Nicotine Delivery Systems Elevate Oxidative Stress and Accelerate Fibrosis Within the Mouse Corpora Cavernosa. J Sex Med. 2021;18: S24. doi:10.1016/j.jsxm.2021.01.112	No	Not meeting inclusion criteria	Title and abstract screening
3600	Nguyen T, Li GE, Chen H, Cranfield CG, McGrath KC, Gorrie CA. Neurological effects in the offspring after switching from tobacco cigarettes to e-cigarettes during pregnancy in a mouse model. Toxicol Sci Off J Soc Toxicol. 2019. doi:10.1093/toxsci/kfz194	No	Not meeting inclusion criteria	Title and abstract screening
3601	Nguyen Zarndt A, Donaldson EA, Bernat JK, Henrie JA, Portnoy DB. Adult Use of and Transitions From Nicotine and Non-nicotine-Containing E-cigarettes: Data From the Population Assessment of Tobacco and Health (PATH) Study, 2013-2016. Nicotine Tob Res. 2020;22: 1225–1229. doi:10.1093/ntr/ntz215	No	Not meeting inclusion criteria	Title and abstract screening
3602	Nicholson T, Davis L, Davis ET, Newton Ede M, Scott A, Jones SW. e-Cigarette Vapour Condensate Reduces Viability and Impairs Function of Human Osteoblasts, in Part, via a Nicotine Dependent Mechanism. Toxics. 2022;10. doi:10.3390/toxics10090506	No	Not meeting inclusion criteria	Title and abstract screening
3603	Nickels A.S., Warner D.O., Jenkins S.M., Tilburt J., Hays J.T. Pulmonologists' and primary care physicians' responses to an adult patient with asthma who inquires about using electronic cigarettes as a smoking cessation tool. Ann Am Thorac Soc. 2017;14: 466–468. doi:10.1513/AnnalsATS.201610-828LE	No	Not meeting inclusion criteria	Title and abstract screening
3604	Nickels AS, Warner DO, Jenkins SM, Tilburt J, Hays JT. Beliefs, Practices, and Self-efficacy of US Physicians Regarding Smoking Cessation and Electronic Cigarettes: A National Survey. Nicotine Tob Res. 2017;19: 197–207. doi:10.1093/ntr/ntw194	No	Not meeting inclusion criteria	Title and abstract screening
3605	Nicksic NE. E-cigarette school policies, advertising, and use among Texas adolescents. Diss Abstr Int Sect B Sci Eng. 2017;77: No-Specified.	No	Not meeting inclusion criteria	Title and abstract screening
3606	Nicolaou A., Moore A., Wamamili B., Walls T., Pattemore P. E-cigarette use patterns, brand preference and knowledge about vaping among teenagers (13-16 years) and parents of children attending Christchurch Hospital. N Z Med J. 2022;135: 94–101.	No	Not meeting inclusion criteria	Title and abstract screening

3607	Nieder C, Aanes SG, Haukland EC. Palliative non-small cell lung cancer treatment and end-of-life care stratified by sex and childlessness: an important interplay in unmarried patients? Support Care Cancer. 2022;30: 5527–5532. doi:10.1007/s00520-022-06987-7	No	Not meeting inclusion criteria	Title and abstract screening
3608	Niederbacher N., Bermudez L.G., Gonzalez D.M., Bernal C., Garcia F., Leon D., et al. Electronic cigarettes: Genetic and epigenetic impact (Review). Int J Epigenetics. 2021;1: 2. doi:10.3892/ije.2021.2	No	Not meeting inclusion criteria	Title and abstract screening
3609	Nierengarten MB. First person profile: Himisha Beltran, MD: By all accounts, Dr Beltran is living her wish and achieving her goal of marrying research to clinical work to, in the end, better the lives of people with cancer: By all accounts, Dr Beltran is living her wish a. Cancer 0008543X. 2022;128: 4038–4038. doi:10.1002/cncr.34536	No	Not meeting inclusion criteria	Title and abstract screening
3610	Nierengarten MB. Palliative care training can help patients with advanced cancer at the end of life. Cancer 0008543X. 2023;129: 2439–2440. doi:10.1002/cncr.34949	No	Not meeting inclusion criteria	Title and abstract screening
3611	Nierengarten MB. Systemic therapies at the end of life vary by patient and practice: Whether or not patients with cancer at the end of life received systemic therapies, including immunotherapies, depended partly on their race, insurance type, and the practice at which the. Cancer 0008543X. 2023;129: 3354–3355. doi:10.1002/cncr.35053	No	Not meeting inclusion criteria	Title and abstract screening
3612	Nierengarten MB. Electronic nicotine delivery system reduces exposure to tobacco-related carcinogens. Cancer. 2021;127: 3280–3281. doi:10.1002/cncr.33863	No	Not meeting inclusion criteria	Title and abstract screening
3613	Nieslony F., Nieslony D., Mado H. Electronic cigarettes and pregnancy-what do we currently know? Electron J Gen Med. 2021;19: em339. doi:10.29333/ejgm/11410	No	Not meeting inclusion criteria	Title and abstract screening
3614	Nighbor TD, Browning KO, Reed EN, Oliver AC, DeSarno MJ, Kurti AN, et al. Using an experimental tobacco marketplace to pilot test the substitutability of JUUL e-cigarettes and other alternative nicotine and tobacco products for conventional cigarettes among vulnerable populations. Prev Med. 2022; 107122. doi:10.1016/j.ypmed.2022.107122	No	Not meeting inclusion criteria	Title and abstract screening
3615	Nikčević AV, Alma L, Marino C, Kolubinski D, Yılmaz-Samancı AE, Caselli G, et al. Modelling the contribution of negative affect, outcome expectancies and metacognitions to cigarette use and nicotine dependence. Addict Behav. 2017;74: 82–89. doi:10.1016/j.addbeh.2017.06.002	No	Not meeting inclusion criteria	Title and abstract screening
3616	Nishimura M, Asai K, Tabuchi T, Toyokura E, Kawai T, Miyamoto A, et al. Association of combustible cigarettes and heated tobacco products use with SARS-CoV-2 infection and severe COVID-19 in Japan: a JASTIS 2022 cross-sectional study. Sci Rep. 2023;13: 1120. doi:10.1038/s41598-023-28006-3	No	Not meeting inclusion criteria	Title and abstract screening
3617	Niu S, Colon GR, Molberg K, Chen H, Carrick K, Yan S, et al. Significance of Oil-Red-O positive macrophages in bronchoalveolar lavage in diagnosing E-cigarettes or vaping product use-associated lung injury: A case series. Diagn Cytopathol. 2021;49: 876–884. doi:10.1002/dc.24760	No	Not meeting inclusion criteria	Title and abstract screening
3618	Noar SM, Gottfredson N, Vereen RN, Kurtzman R, Sheldon JM, Adams E, et al. Development of the UNC Perceived Message Effectiveness Scale for Youth. Tob Control. 2021. doi:10.1136/tobaccocontrol-2021-056929	No	Not meeting inclusion criteria	Title and abstract screening
3619	Noar SM, Gottfredson N, Vereen RN, Kurtzman R, Sheldon JM, Adams E, et al. Development of the UNC Perceived Message Effectiveness Scale for Youth. Tob Control. 2023;32: 553–558. doi:10.1136/tobaccocontrol-2021-056929	No	Not meeting inclusion criteria	Title and abstract screening
3620	Noar SM, Gottfredson NC, Kieu T, Rohde JA, Hall MG, Ma H, et al. Impact of Vaping Prevention Advertisements on US Adolescents: A Randomized Clinical Trial. JAMA Netw Open. 2022;5: e2236370. doi:10.1001/jamanetworkopen.2022.36370	No	Not meeting inclusion criteria	Title and abstract screening
3621	Noar SM, Ma H, Kieu T, Ribisl KM. Can Extant Vaping Prevention Message Experiments Tell Us Something About What Works? A Response to O'Keefe. Health Commun. 2023;38: 1727–1730. doi:10.1080/10410236.2023.2212195	No	Not meeting inclusion criteria	Title and abstract screening
3622	Noda Y, So R, Sonoda M, Tabuchi T, Nomura A. The Usefulness of a Smartphone App-Based Smoking Cessation Program for Conventional Cigarette Users, Heated Tobacco Product Users, and Dual Users: Retrospective Study. J Med Internet Res. 2023;25: e42776. doi:10.2196/42776	No	Not meeting inclusion criteria	Title and abstract screening
3623	Noel JK, Rosenthal SR, Skierkowski-Foster D, Borden SK. Effect of COVID-19 Lockdown on Substance Use Among Middle School and High School Students: A Natural Experiment. Public Health Rep. 2023;138: 349–356. doi:10.1177/00333549221138856	No	Not meeting inclusion criteria	Title and abstract screening
3624	Nollen NL, Leavens ELS, Ahluwalia JS, Rice M, Mayo MS, Pulvers K. Menthol versus non-menthol flavouring and switching to e-cigarettes in black and Latinx adult menthol combustible cigarette smokers: secondary analyses from a randomised clinical trial. Tob Control. 2022. doi:10.1136/tobaccocontrol-2021-057180	No	Not meeting inclusion criteria	Title and abstract screening
3625	Nollen NL, Leavens ELS, Ahluwalia JS, Rice M, Mayo MS, Pulvers K. Menthol versus non-menthol flavouring and switching to e-cigarettes in black and Latinx adult menthol combustible cigarette smokers: secondary analyses from a randomised clinical trial. Tob Control. 2023;32: 786–789. doi:10.1136/tobaccocontrol-2021-057180	No	Not meeting inclusion criteria	Title and abstract screening
3626	Nomura A, Ikeda T, Fujimoto T, Morita Y, Taniguchi C, Ishizawa T, et al. Outcomes of a telemedicine smoking cessation programme for heated tobacco product users in Japan: a retrospective cohort study. BMJ Open. 2022;12: e063489. doi:10.1136/bmjopen-2022-063489	No	Not meeting inclusion criteria	Title and abstract screening
3627	Nordin A.S.A., Mohamad A.S., Quah A.C.K., Hairi F.M., Yee A., Tajuddin N.A.A., et al. Methods of the 2020 (Wave 1) International Tobacco Control (ITC) Malaysia survey. Tob Induc Dis. 2022;20: 20. doi:10.18332/tid/146568	No	Not meeting inclusion criteria	Title and abstract screening
3628	Norling L.V., Halade G.V. Helpful inflammation turned harmful in non-communicable diseases. Curr Opin Pharmacol. 2022;67: 102317. doi:10.1016/j.coph.2022.102317	No	Not meeting inclusion criteria	Title and abstract screening
3629	Norman FF, Diaz J, Martin-Davila P, Tato M, Miguel LG, Gil E, et al. Bilateral pulmonary infiltrates in a traveler from Saudi Arabia with probable electronic or vaping associated lung injury (EVALI). J Travel Med. 2023. doi:10.1093/jtm/taad133	No	Not meeting inclusion criteria	Title and abstract screening
3630	Norman R.H. Letter to the Editor. ATLA Altern Lab Anim. 2018;46: 375–376. doi:10.1177/026119291804600607	No	Not meeting inclusion criteria	Title and abstract screening
3631	Nothelle S, Chamberlain AM, Jacobson D, Green AR, Boyd CM, Rocca WA, et al. Prevalence of co-occurring serious illness diagnoses and association with health care utilization at the end of life. J Am Geriatr Soc. 2022;70: 2621–2629. doi:10.1111/jgs.17881	No	Not meeting inclusion criteria	Title and abstract screening
3632	Notley C. Commentary on Loud et al.: Reconsidering nicotine dependence in adults. Addiction. 2022;117: 482–483. doi:10.1111/add.15701	No	Not meeting inclusion criteria	Title and abstract screening
3633	Notley C, Butler AR, Lindson N, Bullen C, Theodoulou A, Begh R, et al. The Cochrane review of electronic cigarettes for smoking cessation: remaining focused on the evidence. Eur Respir J. 2021;58. doi:10.1183/13993003.02117-2021	No	Not meeting inclusion criteria	Title and abstract screening
3634	Notley C, Ward E, Dawkins L, Holland R. User pathways of e-cigarette use to support long term tobacco smoking relapse prevention: a qualitative analysis. Addiction. 2021;116: 596–605. doi:10.1111/add.15226	No	Not meeting inclusion criteria	Title and abstract screening
3635	Notley C, West R, Soar K, Hastings J, Cox S. Toward an ontology of identity-related constructs in addiction, with examples from nicotine and tobacco research. Addiction. 2022. doi:10.1111/add.16079	No	Not meeting inclusion criteria	Title and abstract screening

3636	Notley C, West R, Soar K, Hastings J, Cox S. Toward an ontology of identity-related constructs in addiction, with examples from nicotine and tobacco research. Addiction. 2023;118: 548–557. doi:10.1111/add.16079	No	Not meeting inclusion criteria	Title and abstract screening
3637	Notley C., Brown T.J., Bauld L., Hardeman W., Holland R., Naughton F., et al. Development of a complex intervention for the maintenance of postpartum smoking abstinence: process for defining evidence-based intervention. Int J Environ Res Public Health. 2019;16: 1968. doi:10.3390/ijerph16111968	No	Not meeting inclusion criteria	Title and abstract screening
3638	Novais A.M.L., Castello Branco R.C. Association between e-cigarette and atherosclerosis: A review. Atherosclerosis. 2022;355: 6. doi:10.1016/j.atherosclerosis.2022.06.245	No	Not meeting inclusion criteria	Title and abstract screening
3639	Novotny TE, van Schalkwyk MCI. How Should Physicians in Low- and Middle-Income Countries Regard Electronic Nicotine Delivery Systems to Facilitate Smoking Cessation?. AMA J Ethics. 2020;22: E82-92. doi:10.1001/amajethics.2020.82	No	Not meeting inclusion criteria	Title and abstract screening
3640	Nowak A., Pawliczak R. Comparison of the harmfulness of smoking tobacco cigarettes versus heat-not-burn tobacco in patients with asthma. Alergol Pol - Pol J Allergol. 2021;8: 72–76. doi:10.5114/pja.2021.106686	No	Not meeting inclusion criteria	Title and abstract screening
3641	Nowak A., Pawliczak R. Cigarette smoking and oxidative stress. Alergol Pol - Pol J Allergol. 2022;9: 89–98. doi:10.5114/pja.2022.116285	No	Not meeting inclusion criteria	Title and abstract screening
3642	Noyes M, Herbert A, Moloney S, Irving H, Bradford N. Location of end-of-life care of children with cancer: A systematic review of parent experiences. Pediatr Blood Cancer. 2022;69: 1–12. doi:10.1002/pbc.29621	No	Not meeting inclusion criteria	Title and abstract screening
3643	NTR5113. BLENDED SMOKING CESSATION TREATMENT. 2015. Available: https://www.cochranelibrary.com/central/doi/10.1002/central/CN-01877487/full	No	Not meeting inclusion criteria	Title and abstract screening
3644	Nursey F, Gillett K. Intrathecal drug delivery for cancer pain at the end of life: a case study. Br J Nurs. 2023;32: S16–S22. doi:10.12968/bjon.2023.32.5.S16	No	Not meeting inclusion criteria	Title and abstract screening
3645	Nuryunarsih D, Lewis S, Langley T. Health Risks of Kretek Cigarettes: A Systematic Review. Nicotine Tob Res. 2021;23: 1274–1282. doi:10.1093/ntr/ntab016	No	Not meeting inclusion criteria	Title and abstract screening
3646	Nyakundi C.K., Fullerton D.G. Tobacco addiction in young people: the paediatrician's role. Paediatr Child Health U K. 2022;32: 266–272. doi:10.1016/j.paed.2022.04.005	No	Not meeting inclusion criteria	Title and abstract screening
3647	Nyakutsikwa B, Britton J, Bogdanovica I, Boobis A, Langley T. Characterising vaping products in the United Kingdom: an analysis of Tobacco Products Directive notification data. Addiction. 2021;116: 2521–2528. doi:10.1111/add.15463	No	Not meeting inclusion criteria	Title and abstract screening
3648	Nyman AL, Jivani S, Jazwa A, Heath E, Redmon PB, Sinha B, et al. Student tobacco use, secondhand smoke exposure, and policy beliefs before and after implementation of a tobacco-free campus policy: Analysis of five U.S. college and university campuses. Prev Med. 2022;163: 107238. doi:10.1016/j.ypmed.2022.107238	No	Not meeting inclusion criteria	Title and abstract screening
3649	O'Brien E.K., Baig S.A., Persoskie A. Developing and Validating Measures of Absolute and Relative E-Cigarette Product Risk Perceptions: Single Items Can Be Surprisingly Comprehensive. Nicotine Tob Res. 2022;24: 316–323. doi:10.1093/ntr/ntab156	No	Not meeting inclusion criteria	Title and abstract screening
3650	O'Brien EK, Roditis M, Persoskie A, Margolis KA. Youths' Perceptions of Nicotine Harm and Associations With Product Use. Nicotine Tob Res Off J Soc Res Nicotine Tob. 2023;25: 1302–1309. doi:10.1093/ntr/ntad028	No	Not meeting inclusion criteria	Title and abstract screening
3651	O'Callaghan M, Boyle N, Fabre A, Keane MP, McCarthy C. Vaping-Associated Lung Injury: A Review. Med Kaunas. 2022;58. doi:10.3390/medicina58030412	No	Not meeting inclusion criteria	Title and abstract screening
3652	O'Connor R, Rousu MC, Bansal-Travers M, Vogl L, Corrigan JR. Using Experimental Auctions to Examine Demand for E-Cigarettes. Nicotine Tob Res. 2017;19: 767–772. doi:10.1093/ntr/ntw257	No	Not meeting inclusion criteria	Title and abstract screening
3653	O'Connor R, Schneller LM, Felicione NJ, Talhout R, Goniewicz ML, Ashley DL. Evolution of tobacco products: recent history and future directions. Tob Control. 2022;31: 175–182. doi:10.1136/tobaccocontrol-2021-056544	No	Not meeting inclusion criteria	Title and abstract screening
3654	O'Connor R.J., Fix B.V., McNeill A., Goniewicz M.L., Bansal-Travers M., Heckman B.W., et al. Characteristics of nicotine vaping products used by participants in the 2016 ITC Four Country Smoking and Vaping Survey. Addict Abingdon Engl. 2019;114: 15–23. doi:10.1111/add.14571	No	Not meeting inclusion criteria	Title and abstract screening
3655	O'Connor RJ, Fix BV, McNeill A, Goniewicz ML, Bansal-Travers M, Heckman BW, et al. Characteristics of nicotine vaping products used by participants in the 2016 ITC Four Country Smoking and Vaping Survey. Addiction. 2019;114 Suppl 1: 15–23. doi:10.1111/add.14571	No	Not meeting inclusion criteria	Title and abstract screening
3656	O'Donnell R, Brown A, Eadie D, Mitchell D, Bauld L, Demou E, et al. Challenges associated with e-cigarette use by people in custody in Scottish prisons: a qualitative interview study with prison staff. BMJ Open. 2022;12: e051009. doi:10.1136/bmjopen-2021-051009	No	Not meeting inclusion criteria	Title and abstract screening
3657	O'Dowd A. Vaping is causing rise in asthma and wheezing episodes in children, MPs hear. BMJ. 2023;381: 1503. doi:10.1136/bmj.p1503	No	Not meeting inclusion criteria	Title and abstract screening
3658	O'Gara E, Sharma E, Boyle RG, Taylor KA. Exploring exclusive and poly-tobacco use among adult cigarette smokers in Minnesota. Am J Health Behav. 2017;41: 84–91. doi:10.5993/AJHB.41.1.9https://dx.doi.org/10.5993/AJHB.41.1.9	No	Not meeting inclusion criteria	Title and abstract screening
3659	O'Hanlon CE, Giannitrapani KF, Lindvall C, Gamboa RC, Canning M, Asch SM, et al. Patient and Caregiver Prioritization of Palliative and End-of-Life Cancer Care Quality Measures. JGIM J Gen Intern Med. 2022;37: 1429–1435. doi:10.1007/s11606-021-07041-8	No	Not meeting inclusion criteria	Title and abstract screening
3660	O'Hanlon CE, Lindvall C, Giannitrapani KF, Garrido M, Ritchie C, Asch S, et al. Expert Stakeholder Prioritization of Process Quality Measures to Achieve Patient- and Family-Centered Palliative and End-of-Life Cancer Care. J Palliat Med. 2021;24: 1321–1333. doi:10.1089/jpm.2020.0633	No	Not meeting inclusion criteria	Title and abstract screening
3661	O'Leary R, Polosa R, Li Volti G. Critical appraisal of the European Union Scientific Committee on Health, Environmental and Emerging Risks (SCHEER) Preliminary Opinion on electronic cigarettes. Harm Reduct J. 2021;18: 31. doi:10.1186/s12954-021-00476-6	No	Not meeting inclusion criteria	Title and abstract screening
3662	O'Leary R, Qureshi MA, La Rosa GRM, Vernooij RWM, Odimegwu DC, Bertino G, et al. Respiratory and Cardiovascular Health Effects of e-Cigarette Substitution: Protocol for Two Living Systematic Reviews. JMIR Res Protoc. 2021;10: e29084. doi:10.2196/29084	No	Not meeting inclusion criteria	Title and abstract screening
3663	O'Leary R, Polosa R. e-Cigarette Use and the Cessation of Tobacco Cigarette Smoking: Protocol for an Umbrella Review. JMIR Res Protoc. 2023;12: e47711. doi:10.2196/47711	No	Not meeting inclusion criteria	Title and abstract screening
3664	O'Mahony S, Kittelson S, Barker PC, Delgado Guay MO, Yao Y, Handzo GF, et al. Association of Race with End-of-Life Treatment Preferences in Older Adults with Cancer Receiving Outpatient Palliative Care. J Palliat Med. 2021;24: 1174–1182. doi:10.1089/jpm.2020.0542	No	Not meeting inclusion criteria	Title and abstract screening

3665	O'Toole D. Rapid recommendations Updates from 2020 guidelines: part 2. Can Fam Physician. 2022;68: 36–38. doi:10.46747/cfp.680136	No	Not meeting inclusion criteria	Title and abstract screening
3666	Oberle C.D., Marcell H.S., Noebel N.A. Orthorexia nervosa and substance use for the purposes of weight control, conformity, and emotional coping. Eat Weight Disord. 2022;27: 553–561. doi:10.1007/s40519-021-01190-9	No	Not meeting inclusion criteria	Title and abstract screening
3667	Oberoi S., Protudjer J.L.P., Rapoport A., Rassekh S.R., Crooks B., Siden H., et al. Perspectives of pediatric oncologists and palliative care physicians on the therapeutic use of cannabis in children with cancer. Cancer Rep. 2022;5: e1551. doi:10.1002/cnr2.1551	No	Not meeting inclusion criteria	Title and abstract screening
3668	Obri MS, Youssef RM, Alluri S, Vemulapalli K, Ichkhanian Y, Todter EN, et al. Disparities in Referrals to End-of-Life Care in Eligible Hepatocellular Carcinoma Patients. Dig Dis Sci. 2023;68: 3277–3282. doi:10.1007/s10620-023-07992-4	No	Not meeting inclusion criteria	Title and abstract screening
3669	Odani S, Armour BS, Graffunder CM, Willis G, Hartman AM, Agaku IT. State-Specific Prevalence of Tobacco Product Use Among Adults - United States, 2014-2015. MMWR Morb Mortal Wkly Rep. 2018;67: 97–102. doi:10.15585/mmwr.mm6703a3	No	Not meeting inclusion criteria	Title and abstract screening
3670	Odani S, Tabuchi T. Prevalence of heated tobacco product use in Japan: the 2020 JASTIS study. Tob Control. 2022;31: e64–e65. doi:10.1136/tobaccocontrol-2020-056257	No	Not meeting inclusion criteria	Title and abstract screening
3671	Odutola M.K., van Leeuwen M.T., Turner J., Bruinsma F., Seymour J.F., Prince H.M., et al. Associations between Smoking and Alcohol and Follicular Lymphoma Incidence and Survival: A Family-Based Case-Control Study in Australia. Cancers. 2022;14: 2710. doi:10.3390/cancers14112710	No	Not meeting inclusion criteria	Title and abstract screening
3672	Oh H, Cook S, Siegel L, Liber A, Levy DT, Fleischer NL. The Longitudinal Association Between Cigarette Coupon Receipt and Short-term Smoking Cessation Among US Adults. Nicotine Tob Res. 2023;25: 738–745. doi:10.1093/ntr/ntac258	No	Not meeting inclusion criteria	Title and abstract screening
3673	Oh H, Im B, Seo W. Comparisons of the stages and psychosocial factors of smoking cessation and coping strategies for smoking cessation in college student smokers: Conventional cigarette smokers compared to dual smokers of conventional and e-cigarettes. Jpn J Nurs Sci. 2019;16: 345–354. doi:10.1111/jjns.12241	No	Not meeting inclusion criteria	Title and abstract screening
3674	Oh H, Smith L, Jacob L, Du J, Shin JI, Zhou S, et al. Food Insecurity and Substance Use Among Young Adult College Students in the United States. J Addict Med. 2023;17: 163–168. doi:10.1097/ADM.0000000000001062	No	Not meeting inclusion criteria	Title and abstract screening
3675	Ohara H, Ito S, Takanami Y. Binary classification of users of electronic cigarettes and smokeless tobacco through biomarkers to assess similarity with current and former smokers: machine learning applied to the population assessment of tobacco and health study. BMC Public Health. 2023;23: 589. doi:10.1186/s12889-023-15511-3	No	Not meeting inclusion criteria	Title and abstract screening
3676	Ohashi K, Hayashida A, Nozawa A, Matsumura K, Ito S. Human vasculature-on-a-chip with macrophage-mediated endothelial activation: The biological effect of aerosol from heated tobacco products on monocyte adhesion. Toxicol Vitro Int J Publ Assoc BIBRA. 2023;89: 105582. doi:10.1016/j.tiv.2023.105582	No	Not meeting inclusion criteria	Title and abstract screening
3677	Okamura T, Shimada C, Ito M, Ito K. Analysis of challenges faced by care managers in providing end-of-life care for older people with terminally ill cancer and dementia. Psychogeriatrics. 2021;21: 854–855. doi:10.1111/psyg.12745	No	Not meeting inclusion criteria	Title and abstract screening
3678	Okonkwo CA. Factors associated with electronic cigarette use among adolescents in Texas. Diss Abstr Int Sect B Sci Eng. 2021;82: No-Specified.	No	Not meeting inclusion criteria	Title and abstract screening
3679	Okubo R, Tabuchi T. Smoking and drinking among patients with mental disorders: Evidence from a nationally representative Japanese survey. J Affect Disord. 2021;279: 443–450. doi:10.1016/j.jad.2020.10.037	No	Not meeting inclusion criteria	Title and abstract screening
3680	Okumura K, Jyothula S, Kaleekal T, Dhand A. 1-Year Outcomes of Lung Transplantation for Coronavirus Disease 2019–Associated End-Stage Lung Disease in the United States. Clin Infect Dis. 2023;76: 2140–2147. doi:10.1093/cid/ciad072	No	Not meeting inclusion criteria	Title and abstract screening
3681	Olenik A, Mospan CM. Smoking cessation: Identifying readiness to quit and designing a plan. JAAPA. 2017;30: 13–19. doi:10.1097/01.JAA.0000520530.80388.2f	No	Not meeting inclusion criteria	Title and abstract screening
3682	Olfson M, Wall MM, Liu SM, Sultan RS, Blanco C. E-cigarette Use Among Young Adults in the U.S. Am J Prev Med. 2019;56: 655–663. doi:10.1016/j.amepre.2018.12.004	No	Not meeting inclusion criteria	Title and abstract screening
3683	Olga Sribna O., Kvasha O., Rafael D. Influence of electronic nicotine delivery systems on the risk of development of cardiovascular diseases: Evaluation of tolerance to physical activity. Eur J Prev Cardiol. 2019;26: S112–S113. doi:10.1177/2047487319860053	No	Not meeting inclusion criteria	Title and abstract screening
3684	Oliveira L.R., Ferreira R.M., Pinheiro M.R., Silva H.F., Tuchin V.V., Oliveira L.M. Broadband spectral verification of optical clearing reversibility in lung tissue. J Biophotonics. 2022; e202200185. doi:10.1002/jbio.202200185	No	Not meeting inclusion criteria	Title and abstract screening
3685	Oliver JA, Foulds J. Association Between Cigarette Smoking Frequency and Tobacco Use Disorder in U.S. Adults. Am J Prev Med. 2021;60: 726–728. doi:10.1016/j.amepre.2020.10.019	No	Not meeting inclusion criteria	Title and abstract screening
3686	Oliver JA, Pacek LR, Locey EN, Fish LM, Hendricks PS, Pollak KI. Lack of utility of cigarettes per day cutoffs for clinical and laboratory smoking research. Addict Behav. 2019;98: 106066. doi:10.1016/j.addbeh.2019.106066	No	Not meeting inclusion criteria	Title and abstract screening
3687	Olmstead TA, Yonkers KA, Ondersma SJ, Forray A, Gilstad-Hayden K, Martino S. Cost-effectiveness of electronic- and clinician-delivered screening, brief intervention and referral to treatment for women in reproductive health centers. Addiction. 2019;114: 1659–1669. doi:10.1111/add.14668	No	Not meeting inclusion criteria	Title and abstract screening
3688	Olonoff M, Niaura R, Hitsman B. "Electronic Cigarettes" Are Not Cigarettes, and Why That Matters. Nicotine Tob Res. 2019;21: 1441–1444. doi:10.1093/ntr/nty205	No	Not meeting inclusion criteria	Title and abstract screening
3689	Olonoff M, Niaura RS, Ciecierski CC, Ciolino JD, Hitsman B. Electronic nicotine delivery systems: use, knowledge, and attitudes among diverse college students. J Am Coll Health. 2022;70: 2159–2167. doi:10.1080/07448481.2020.1846044	No	Not meeting inclusion criteria	Title and abstract screening
3690	Omaiye EE, Luo W, McWhirter KJ, Pankow JF, Talbot P. Flavour chemicals, synthetic coolants and pulegone in popular mint-flavoured and menthol-flavoured e-cigarettes. Tob Control. 2022;31: e3–e9. doi:10.1136/tobaccocontrol-2021-056582	No	Not meeting inclusion criteria	Title and abstract screening
3691	Omaiye EE, Cordova I, Davis B, Talbot P. Counterfeit Electronic Cigarette Products with Mislabeled Nicotine Concentrations. Tob Regul Sci. 2017;3: 347–357. doi:10.18001/TRS.3.3.10	No	Not meeting inclusion criteria	Title and abstract screening
3692	Omaiye EE, Luo W, McWhirter KJ, Pankow JF, Talbot P. Disposable Puff Bar Electronic Cigarettes: Chemical Composition and Toxicity of E-liquids and a Synthetic Coolant. Chem Res Toxicol. 2022;35: 1344–1358. doi:10.1021/acs.chemrestox.1c00423	No	Not meeting inclusion criteria	Title and abstract screening
3693	Oncken C, Ricci KA, Kuo CL, Dornelas E, Kranzler HR, Sankey HZ. Correlates of Electronic Cigarettes Use Before and During Pregnancy. Nicotine Tob Res. 2017;19: 585–590. doi:10.1093/ntr/ntw225	No	Not meeting inclusion criteria	Title and abstract screening

3694	Onda M., Kuwanoe T., Hashimoto A., Horiguchi M., Domichi M., Sakane N. Pharmacist-Delivered Smoking Cessation Program in Community Pharmacy (The FINE Program) in Japan-The Development of a Training Course and a Feasibility Study. J Pharm Pract. 2021;34: 625–630. doi:10.1177/0897190019889745	No	Not meeting inclusion criteria	Title and abstract screening
3695	Onor I.O., Stirling D.L., Williams S.R., Bediako D., Borghol A., Harris M.B., et al. Clinical effects of cigarette smoking: Epidemiologic impact and review of pharmacotherapy options. Int J Environ Res Public Health. 2017;14: 1147. doi:10.3390/ijerph14101147	No	Not meeting inclusion criteria	Title and abstract screening
3696	Onyije FM, Dolatkhah R, Olsson A, Bouaoun L, Deltour I, Erdmann F, et al. Risk factors for childhood brain tumours: A systematic review and meta-analysis of observational studies from 1976 to 2022. Cancer Epidemiol. 2023;88: 102510. doi:10.1016/j.canep.2023.102510	No	Not meeting inclusion criteria	Title and abstract screening
3697	Oren E., Magraner J., Gunge D., Merz W., Advani I.N., Crotty Alexander L.E. Assessing electronic cigarette use and perceptions during the COVID-19 pandemic in the united states. Am J Respir Crit Care Med. 2021;203. doi:10.1164/ajrccm-conference.2021.203.1_MeetingAbstracts.A1510	No	Not meeting inclusion criteria	Title and abstract screening
3698	Orlovic M, Droney J, Vickerstaff V, Rosling J, Bearne A, Powell M, et al. Accuracy of clinical predictions of prognosis at the end-of-life: evidence from routinely collected data in urgent care records. BMC Palliat Care. 2023;22: 1–11. doi:10.1186/s12904-023-01155-y	No	Not meeting inclusion criteria	Title and abstract screening
3699	Ortega F, Calero M, Rico N, Martín-Lara MA. COVID-19 personal protective equipment (PPE) contamination in coastal areas of Granada, Spain. Mar Pollut Bull. 2023;191: 114908. doi:10.1016/j.marpolbul.2023.114908	No	Not meeting inclusion criteria	Title and abstract screening
3700	Orton S., Taylor L., Laing L., Lewis S., Ussher M., Coleman T., et al. Are E-cigarettes associated with postpartum return to smoking? Secondary analyses of a UK pregnancy longitudinal cohort. BMJ Open. 2022;12: e061028. doi:10.1136/bmjopen-2022-061028	No	Not meeting inclusion criteria	Title and abstract screening
3701	Osaghae I, Chido-Amajuoyi OG, Khalifa BAA, Talluri R, Shete S. Cannabis Use among Cancer Survivors: Use Pattern, Product Type, and Timing of Use. Cancers. 2023;15: 5822. doi:10.3390/cancers15245822	No	Not meeting inclusion criteria	Title and abstract screening
3702	Osborn CC, Suratkal JP, Pike Moore SN, Koopman Gonzalez S, Sterling KL, Quisenberry AJ, et al. Dissonance in Young Adult Cigarillo Users' Categorization of Concept Flavored and Unflavored Products. Int J Env Res Public Health. 2022;19. doi:10.3390/ijerph19127219	No	Not meeting inclusion criteria	Title and abstract screening
3703	Osibogun O, Bursac Z, Mckee M, Li T, Maziak W. Cessation outcomes in adult dual users of e-cigarettes and cigarettes: the Population Assessment of Tobacco and Health cohort study, USA, 2013-2016. Int J Public Health. 2020;65: 923–936. doi:10.1007/s00038-020-01436-w	No	Not meeting inclusion criteria	Title and abstract screening
3704	Osibogun O., Bursac Z., Maziak W. Longitudinal transition outcomes among adult dual users of e-cigarettes and cigarettes with the intention to quit in the United States: PATH Study (2013-2018). Prev Med Rep. 2022;26: 101750. doi:10.1016/j.pmedr.2022.101750	No	Not meeting inclusion criteria	Title and abstract screening
3705	Osinski K., Ross H., Clarke L., Dear J., Veiraiah A. A case of ingestion of two vape cartridges. Clin Toxicol. 2021;59: 674–675. doi:10.1080/15563650.2020.1834575	No	Not meeting inclusion criteria	Title and abstract screening
3706	Oudit G.Y., Sivilotti M.L.A., Eisenberg M.J. Cardiovascular Disease and Drugs of Abuse and Abuse Potential. Can J Cardiol. 2022;38: 1329–1330. doi:10.1016/j.cjca.2022.07.014	No	Not meeting inclusion criteria	Title and abstract screening
3707	Overbeek DL, Kass AP, Chiel LE, Boyer EW, Casey AMH. A review of toxic effects of electronic cigarettes/vaping in adolescents and young adults. Crit Rev Toxicol. 2020;50: 531–538. doi:10.1080/10408444.2020.1794443	No	Not meeting inclusion criteria	Title and abstract screening
3708	Owotomo O, Stritzel H, McCabe SE, Boyd CJ, Maslowsky J. Smoking Intention and Progression From E-Cigarette Use to Cigarette Smoking. Pediatrics. 2020;146. doi:10.1542/peds.2020-002881	No	Not meeting inclusion criteria	Title and abstract screening
3709	Owotomo O, Maslowsky J, Loukas A. Perceptions of the Harm and Addictiveness of Conventional Cigarette Smoking Among Adolescent E-Cigarette Users. J Adolesc Health Off Publ Soc Adolesc Med. 2018;62: 87–93. doi:10.1016/j.jadohealth.2017.08.007	No	Not meeting inclusion criteria	Title and abstract screening
3710	Owotomo O, Walley S. The youth e-cigarette epidemic: updates and review of devices, epidemiology and regulation. Curr Probl Pediatr Adolesc Health Care. 2022;52: 101200. doi:10.1016/j.cppeds.2022.101200	No	Not meeting inclusion criteria	Title and abstract screening
3711	Ozga JE, Felicione NJ, Douglas A, Childers M, Blank MD. Electronic Cigarette Terminology: Where Does One Generation End and the Next Begin? Nicotine Tob Res. 2022;24: 421–424. doi:10.1093/ntr/ntab164	No	Not meeting inclusion criteria	Title and abstract screening
3712	Ozga JE, Stroup AM, Abadi MH, Cheney MK, Majmundar A, Garrison KA, et al. E-cigarette Marketing Expenditures in the United States From 2016 to 2021: Targeted Media Outlets Geared Toward People Who Are at Increased Risk for Tobacco Use. Nicotine Tob Res. 2023;25: 581–585. doi:10.1093/ntr/ntac209	No	Not meeting inclusion criteria	Title and abstract screening
3713	Ozgunay SE, Karasu D, Dulger S, Yilmaz C, Tabur Z. [Relationship between cigarette smoking and the carbon monoxide concentration in the exhaled breath with perioperative respiratory complications]. Braz J Anesth. 2018;68: 462–471. doi:10.1016/j.bjan.2018.02.006	No	Not meeting inclusion criteria	Title and abstract screening
3714	Pabelick C., Thompson M., Sine S., Prakash Y.S. Targeting nicotinic receptors for asthma. Am J Respir Crit Care Med. 2017;195. doi:10.1164/ajrccm-conference.2017.A77	No	Not meeting inclusion criteria	Title and abstract screening
3715	Pacek L.R., Villanti A.C., Joseph McClernon F. Not Quite the Rule, but No Longer the Exception: Multiple Tobacco Product Use and Implications for Treatment, Research, and Regulation. Nicotine Tob Res. 2020;22: 2114–2117. doi:10.1093/ntr/ntz221	No	Not meeting inclusion criteria	Title and abstract screening
3716	Pacek LR, Rass O, Johnson MW. Knowledge about nicotine among HIV-positive smokers: Implications for tobacco regulatory science policy. Addict Behav. 2017;65: 81–86. doi:10.1016/j.addbeh.2016.10.008	No	Not meeting inclusion criteria	Title and abstract screening
3717	Paciência I, Cavaleiro Rufo J, Moreira A. Environmental inequality: Air pollution and asthma in children. Pediatr Allergy Immunol. 2022;33. doi:10.1111/pai.13818	No	Not meeting inclusion criteria	Title and abstract screening
3718	Pacula RL. The need to more effectively regulate end markets: A primary public health lesson of the U.S. vaping associated lung injury outbreak. Addiction. 2021;116: 994–995. doi:10.1111/add.15179https://dx.doi.org/10.1111/add.15179	No	Not meeting inclusion criteria	Title and abstract screening
3719	Paek J, Son S, Choi YJ. E-cigarette and cigarette use among cancer survivors versus general population: a case-control study in Korea. J Cancer Surviv. 2022;16: 741–750. doi:10.1007/s11764-021-01067-x	No	Not meeting inclusion criteria	Title and abstract screening
3720	Pagano A., Gubner N.R., Le T., Yip D., Williams D., Delucchi K., et al. Differences in tobacco use prevalence, behaviors, and cessation services by race/ethnicity: A survey of persons in addiction treatment. J Subst Abuse Treat. 2018;94: 9–17. doi:10.1016/j.jsat.2018.08.003	No	Not meeting inclusion criteria	Title and abstract screening
3721	Page MK, Block AC, Santiago AL, Leigh NJ, Kaiser LM, Martin CD, et al. Changes in product labelling practices and the use of flavouring chemical additives in vaping products after enactment of statewide flavour legislation. Tob Control. 2022;31: s223–s229. doi:10.1136/tc-2022-057469	No	Not meeting inclusion criteria	Title and abstract screening
3722	Page MK, Goniewicz ML. New Analytical Method for Quantifying Flavoring Chemicals of Potential Respiratory Health Risk Concerns in e-Cigarette Liquids. Front Chem. 2021;9: 763940. doi:10.3389/fchem.2021.763940	No	Not meeting inclusion criteria	Title and abstract screening

3723	Pajai DD, Paul P, Reche A. Pharmacotherapy in Tobacco Cessation: A Narrative Review. Cureus. 2023;15: e35086. doi:10.7759/cureus.35086	No	Not meeting inclusion criteria	Title and abstract screening
3724	Palma C., Lee K., Gomez A., Katragadda R., Honsberg A. Large Bilateral Pneumothoraces in a Young Male with Covid-19 Pneumonia. Am J Respir Crit Care Med. 2022;205. doi:10.1164/ajrccm-conference.2022.205.1_MeetingAbstracts.A4560	No	Not meeting inclusion criteria	Title and abstract screening
3725	Palmer AM, Brandon TH. How do electronic cigarettes affect cravings to smoke or vape? Parsing the influences of nicotine and expectancies using the balanced-placebo design. J Consult Clin Psychol. 2018;86: 486–491. doi:10.1037/ccp0000303	No	Not meeting inclusion criteria	Title and abstract screening
3726	Palmer AM, Carpenter MJ, Rojewski AM, Haire K, Baker NL, Toll BA. Nicotine replacement therapy for vaping cessation among mono and dual users: A mixed methods preliminary study. Addict Behav. 2023;139: 107579. doi:10.1016/j.addbeh.2022.107579	No	Not meeting inclusion criteria	Title and abstract screening
3727	Palmer AM, Price SN, Foster MG, Sanford BT, Fucito LM, Toll BA. Urgent Need for Novel Investigations of Treatments to Quit E-cigarettes: Findings from a Systematic Review. Cancer Prev Res Phila. 2022;15: 569–580. doi:10.1158/1940-6207.CAPR-22-0172	No	Not meeting inclusion criteria	Title and abstract screening
3728	Palmer AM, Rojewski AM, Carpenter MJ, Klemperer EM, Baker NL, Sanford BT, et al. Interest in quitting e-cigarette use by device type and smoking history in US adults. Tob Control. 2023. doi:10.1136/tc-2022-057710	No	Not meeting inclusion criteria	Title and abstract screening
3729	Palmer AM, Toll BA, Carpenter MJ, Donny EC, Hatsukami DK, Rojewski AM, et al. Reappraising Choice in Addiction: Novel Conceptualizations and Treatments for Tobacco Use Disorder. Nicotine Tob Res. 2022;24: 3–9. doi:10.1093/ntr/ntab148	No	Not meeting inclusion criteria	Title and abstract screening
3730	Palmer AM, Tomko RL, Squeglia LM, Gray KM, Carpenter MJ, Smith TT, et al. A pilot feasibility study of a behavioral intervention for nicotine vaping cessation among young adults delivered via telehealth. Drug Alcohol Depend. 2022;232: 109311. doi:10.1016/j.drugalcdep.2022.109311	No	Not meeting inclusion criteria	Title and abstract screening
3731	Palmer A, Brandon T. Nicotine or expectancies? Using the balanced-placebo design to test immediate outcomes of vaping. 2019;97: 90-96. doi:10.1016/j.addbeh.2019.04.026	No	Not meeting inclusion criteria	Title and abstract screening
3732	Panagiotakos DB, Georgoulis M, Kapetanstrataki M, Behrakis P. Prevalence, patterns and determinants of electronic cigarette and heated tobacco product use in Greece: a cross-sectional survey. Hell J Cardiol. 2023. doi:10.1016/j.hjc.2023.01.002	No	Not meeting inclusion criteria	Title and abstract screening
3733	Pandarathodiyil AK, Ramanathan A, Garg R, Doss JG, Abd Rahman FB, Ghani WMN, et al. Lactate Dehydrogenase Levels in the Saliva of Cigarette and E-Cigarette Smokers (Vapers): A Comparative Analysis. Asian Pac J Cancer Prev. 2021;22: 3227–3235. doi:10.31557/APJCP.2021.22.10.3227	No	Not meeting inclusion criteria	Title and abstract screening
3734	Pang E., Stern M. Effective ways of supporting patients to stop smoking. Prescriber. 2017;28: 14–20. doi:10.1002/psb.1536	No	Not meeting inclusion criteria	Title and abstract screening
3735	Pang R.D., Goldenson N.I., Kirkpatrick M., Barrington-Trimis J.L., Cho J., Leventhal A.M. Sex differences in the appeal of flavored e-cigarettes among young adult e-cigarette users. Psychol Addict Behav. 2020;34: 303–307. doi:10.1037/adb0000548	No	Not meeting inclusion criteria	Title and abstract screening
3736	Pang RD, Andrabi N, Leventhal AM. Premenstrual symptoms and factors implicated in smoking cessation among woman smokers. Exp Clin Psychopharmacol. 2017;25: 235–241. doi:10.1037/pha0000132	No	Not meeting inclusion criteria	Title and abstract screening
3737	Pang Y, Li M, Li F, Lei J, Zhang T. Preliminary study on the E-liquid and aerosol on the neurobehavior of C. elegans. Environ Int. 2023;179: 108180. doi:10.1016/j.envint.2023.108180	No	Not meeting inclusion criteria	Title and abstract screening
3738	Pankow W, Andreas S, Rupp A, Pfeifer M. [Smoking Cessation with E-Cigarettes? - Ad Hoc Statement of the German Respiratory Society (DGP)]. Pneumologie. 2021;75: 31–32. doi:10.1055/a-1323-6045	No	Not meeting inclusion criteria	Title and abstract screening
3739	Papadakis S, Katsaounou P, Kyriakos CN, Balmford J, Tzavara C, Girvalaki C, et al. Quitting behaviours and cessation methods used in eight European Countries in 2018: findings from the EUREST-PLUS ITC Europe Surveys. Eur J Public Health. 2020;30: iii26–iii33. doi:10.1093/eurpub/ckaa082	No	Not meeting inclusion criteria	Title and abstract screening
3740	Parekh T., Pemmasani S., Desai R. Marijuana Use Among Young Adults (18-44 Years of Age) and Risk of Stroke: A Behavioral Risk Factor Surveillance System Survey Analysis. Stroke. 2020;51: 308–310. doi:10.1161/STROKEAHA.119.027828	No	Not meeting inclusion criteria	Title and abstract screening
3741	Parikh NS, Navi BB, Merkler AE, Kamel H. Electronic Cigarette Use and Cigarette-Smoking Cessation Attempts Among Stroke Survivors in the US. JAMA Neurol. 2021;78: 759–760. doi:10.1001/jamaneurol.2021.0636	No	Not meeting inclusion criteria	Title and abstract screening
3742	Parikh R., Belok S.H., O'Donnell C., Wakeman C.B., Mishuris R.G., Potter J., et al. Implementation of a multi-disciplinary team focused on providing comprehensive care for patients suffering from vaping-related illness. Am J Respir Crit Care Med. 2020;201. Available: https://www.atsjournals.org/doi/abs/10.1164/ajrccm-conference.2020.201.1_MeetingAbstracts.A1077	No	Not meeting inclusion criteria	Title and abstract screening
3743	Parikh RB, Liu M, Li E, Li R, Chen J. Trajectories of mortality risk among patients with cancer and associated end-of-life utilization. NPJ Digit Med. 2021;4: 1–5. doi:10.1038/s41746-021-00477-6	No	Not meeting inclusion criteria	Title and abstract screening
3744	Parikh RB, Chen J, Heintz J, LaPergola M, Bilker W, Patel MS, et al. End-of-life spending analysis of randomized trial of machine learning nudges to prompt serious illness communication among patients with cancer. J Clin Oncol. 41: 6515–6515. doi:10.1200/JCO.2023.41.16_suppl.6515	No	Not meeting inclusion criteria	Title and abstract screening
3745	Park E, Kwon M, Chacko T, Zhou Y, Chen C, Goniewicz ML, et al. Instruments to measure e-cigarette related constructs: a systematic review. BMC Public Health. 2022;22: 1135. doi:10.1186/s12889-022-13510-4	No	Not meeting inclusion criteria	Title and abstract screening
3746	Park EY, Park E, Jeong BY, Park J, Lee DH, Benowitz NL, et al. Comparison of smoking cessation rates of Quitline users in Korea between smokers of ultra-low nicotine yield cigarettes and other types of cigarette: a prospective study. Addiction. 2020;115: 1745–1753. doi:10.1111/add.15021	No	Not meeting inclusion criteria	Title and abstract screening
3747	Park H. Flavored tobacco product use and its influence on quitting behaviors in vulnerable U.S. adult populations. Diss Abstr Int Sect Humanit Soc Sci. 2024;85: No-Specified.	No	Not meeting inclusion criteria	Title and abstract screening
3748	Park J, Kim HJ, Shin SH, Park E, Oh JK, Park EY, et al. Perceptions of Heated Tobacco Products (HTPs) and Intention to Quit Among Adult Tobacco Users in Korea. J Epidemiol. 2022;32: 357–362. doi:10.2188/jea.JE20200213	No	Not meeting inclusion criteria	Title and abstract screening
3749	Park JA, Crotty Alexander LE, Christiani DC. Vaping and Lung Inflammation and Injury. Annu Rev Physiol. 2022;84: 611–629. doi:10.1146/annurev-physiol-061121-040014	No	Not meeting inclusion criteria	Title and abstract screening
3750	Park K., Masso-Silva J., Alhaddad H., Advani I., Gunge D., Pham J., et al. Induction of Inflammation in the Brain by Daily JUUL Aerosol Inhalation. FASEB J. 2021;35. doi:10.1096/fasebj.2021.35.S1.04368	No	Not meeting inclusion criteria	Title and abstract screening
3751	Park M.-B., Choi J.-K. Differences between the effects of conventional cigarettes, e-cigarettes and dual product use on urine cotinine levels. Tob Induc Dis. 2019;17: 12. doi:10.18332/tid/100527	No	Not meeting inclusion criteria	Title and abstract screening

3752	Park S., Choi C.-M., Hwang S.-S., Choi Y.-L., Kim H.Y., Kim Y.-C., et al. Lung Cancer in Korea. J Thorac Oncol. 2021;16: 1988–1993. doi:10.1016/j.jtho.2021.09.007	No	Not meeting inclusion criteria	Title and abstract screening
3753	Park S.J., Walser T.C., Tran L.M., Perdomo C., Wang T., Hong L.-S., et al. The role of e-cigarette exposure on pulmonary epithelial cell transformation. Cancer Prev Res (Phila Pa). 2015;8. doi:10.1158/1940-6215.PREV-14-B22	No	Not meeting inclusion criteria	Title and abstract screening
3754	Park SH, Lee L, Shearston JA, Weitzman M. Patterns of electronic cigarette use and level of psychological distress. PLoS One. 2017;12: e0173625. doi:10.1371/journal.pone.0173625	No	Not meeting inclusion criteria	Title and abstract screening
3755	Park-Lee E, Gentzke AS, Ren C, Cooper M, Sawdey MD, Hu SS, et al. Impact of Survey Setting on Current Tobacco Product Use: National Youth Tobacco Survey, 2021. J Adolesc Health. 2023;72: 365–374. doi:10.1016/j.jadohealth.2022.10.012	No	Not meeting inclusion criteria	Title and abstract screening
3756	Park-Lee E, Ren C, Cooper M, Cornelius M, Jamal A, Cullen KA. Tobacco Product Use Among Middle and High School Students - United States, 2022. MMWR Morb Mortal Wkly Rep. 2022;71: 1429–1435. doi:10.15585/mmwr.mm7145a1	No	Not meeting inclusion criteria	Title and abstract screening
3757	Parker M.A., Pearson J.L., Villanti A.C. Limited utility of detailed e-cigarette use measures: An analysis of NESARC-III. Addict Behav. 2019;97: 56–62. doi:10.1016/j.addbeh.2019.05.001	No	Not meeting inclusion criteria	Title and abstract screening
3758	Parker MA, Byers JE, Villanti AC. Effect of brief nicotine corrective messaging on nicotine beliefs in persons who use opioids. Exp Clin Psychopharmacol. 2022;30: 1008–1015. doi:10.1037/pha0000497	No	Not meeting inclusion criteria	Title and abstract screening
3759	Parker M, Byers J, Villanti A. Effect of brief nicotine corrective messaging on nicotine beliefs in persons who use opioids. 2021. doi:10.1037/pha0000497	No	Not meeting inclusion criteria	Title and abstract screening
3760	Parks MJ, Fleischer NL, Patrick ME. Response to "commentary on 'increased nicotine vaping due to the COVID-19 pandemic among US young adults: Associations with nicotine dependence, vaping frequency, and reasons for use'". Prev Med. 2023;169: 107439. doi:10.1016/j.ypmed.2023.107439	No	Not meeting inclusion criteria	Title and abstract screening
3761	Pashutina Y, Kotz D, Kastaun S. Attempts to quit smoking, use of smoking cessation methods, and associated characteristics among COPD patients. NPJ Prim Care Respir Med. 2022;32: 50. doi:10.1038/s41533-022-00316-5	No	Not meeting inclusion criteria	Title and abstract screening
3762	Pasquereau A, Guignard R, Andler R, Nguyen-Thanh V. Electronic cigarettes, quit attempts and smoking cessation: a 6-month follow-up. Addiction. 2017;112: 1620–1628. doi:10.1111/add.13869	No	Not meeting inclusion criteria	Title and abstract screening
3763	Patanavanich R, Aekplakorn W, Glantz SA, Kalayasiri R. Use of E-Cigarettes and Associated Factors among Youth in Thailand. Asian Pac J Cancer Prev. 2021;22: 2199–2207. doi:10.31557/APJCP.2021.22.7.2199	No	Not meeting inclusion criteria	Title and abstract screening
3764	Patanavanich R., Glantz S.A. The Theoretical Problems Do Not Materially Affect the Results of Our Meta-Analysis of Smoking and COVID-19 Disease Progression. Nicotine Tob Res. 2021;23: 882–883. doi:10.1093/ntr/ntaa250	No	Not meeting inclusion criteria	Title and abstract screening
3765	Pate S., Miller R., halabiya M. MYCOPLASMA GORDONAE: A RARE CAUSE OF ARDS IN THE IMMUNOCOMPROMISED. Chest. 2021;160: A306. doi:10.1016/j.chest.2021.07.312	No	Not meeting inclusion criteria	Title and abstract screening
3766	Patel A, Hirschtick JL, Cook S, Usidame B, Mistry R, Levy DT, et al. Sociodemographic Patterns of Exclusive and Dual Use of ENDS and Menthol/Non-Menthol Cigarettes among US Youth (Ages 15-17) Using Two Nationally Representative Surveys (2013-2017). Int J Env Res Public Health. 2021;18. doi:10.3390/ijerph18157781	No	Not meeting inclusion criteria	Title and abstract screening
3767	Patel M, Cuccia AF, Czaplicki L, Donovan EM, Simard B, Pitzer L, et al. Smokers' behavioral intentions in response to a low-nicotine cigarette policy. Drug Alcohol Depend. 2019;205: 107645. doi:10.1016/j.drugalcdep.2019.107645	No	Not meeting inclusion criteria	Title and abstract screening
3768	Patel MS, Patel SB, Steinberg MB. Smoking Cessation. Ann Intern Med. 2021;174: ITC177–ITC192. doi:10.7326/AITC202112210	No	Not meeting inclusion criteria	Title and abstract screening
3769	PATEL N., MCCANN T., NAMDARIZANDI V., HAYNER C. EVALI-RELATED PULMONARY TOXICITY. Chest. 2022;161: A373. doi:10.1016/j.chest.2021.12.403	No	Not meeting inclusion criteria	Title and abstract screening
3770	Patel P., Ferkol T. An Adolescent with Respiratory Distress. J Allergy Clin Immunol Pract. 2021;9: 4502–4504. doi:10.1016/j.jaip.2021.09.043	No	Not meeting inclusion criteria	Title and abstract screening
3771	Patel U., Tavrawala K., Mohammad Z., Krishnakumar A.N., Varghese A., Ashwini M.S., et al. Substance Use Disorders and Prevalence of Stroke Amongst the US Population. Circulation. 2022;146. doi:10.1161/circ.146.suppl_1.13921	No	Not meeting inclusion criteria	Title and abstract screening
3772	Patnode CD, Henderson JT, Coppola EL, Melnikow J, Durbin S, Thomas RG. Interventions for Tobacco Cessation in Adults, Including Pregnant Persons: Updated Evidence Report and Systematic Review for the US Preventive Services Task Force. JAMA. 2021;325: 280–298. doi:10.1001/jama.2020.23541	No	Not meeting inclusion criteria	Title and abstract screening
3773	Patrick ME, Parks MJ, Fairlie AM, Kreski NT, Keyes KM, Miech R. Using Substances to Cope With the COVID-19 Pandemic: U.S. National Data at Age 19 Years. J Adolesc Health. 2022;70: 340–344. doi:10.1016/j.jadohealth.2021.11.006	No	Not meeting inclusion criteria	Title and abstract screening
3774	Patten T, De Biasi M. History repeats itself: Role of characterizing flavors on nicotine use and abuse. Neuropharmacology. 2020;177: 108162. doi:10.1016/j.neuropharm.2020.108162	No	Not meeting inclusion criteria	Title and abstract screening
3775	Patten T., Dreier A., Halberstadter K., De Biasi M. Effects of a fruit-flavorant on nicotine consumption and preference throughout adolescence and young adulthood. Neuropsychopharmacology. 2019;44: 378. doi:10.1038/s41386-019-0546-x	No	Not meeting inclusion criteria	Title and abstract screening
3776	Patten T., Dreier A., Herman R.J., Kimball B.A., De Biasi M. Exposure to fruit-flavoring during adolescence increases nicotine consumption and promotes dose escalation. Neuropharmacology. 2021;195: 108672. doi:10.1016/j.neuropharm.2021.108672	No	Not meeting inclusion criteria	Title and abstract screening
3777	Patterson F, Grandner MA, Lozano A, Satti A, Ma G. Transitioning from adequate to inadequate sleep duration associated with higher smoking rate and greater nicotine dependence in a population sample. Addict Behav. 2018;77: 47–50. doi:10.1016/j.addbeh.2017.09.011	No	Not meeting inclusion criteria	Title and abstract screening
3778	Patterson JG, Hinton A, Cooper SE, Wewers ME. Differences in Quit Attempts, Successful Quits, Methods, and Motivations in a Longitudinal Cohort of Adult Tobacco Users by Sexual Orientation. Nicotine Tob Res. 2021;23: 1952–1957. doi:10.1093/ntr/ntab116	No	Not meeting inclusion criteria	Title and abstract screening
3779	Patterson JG, Keller-Hamilton B, Wedel A, Vázquez-Otero C, Liu J, Lee D, et al. Absolute and relative e-cigarette harm perceptions among young adult lesbian and bisexual women and nonbinary people assigned female at birth. Addict Behav. 2023;146: 107788. doi:10.1016/j.addbeh.2023.107788	No	Not meeting inclusion criteria	Title and abstract screening
3780	Patterson JG, Keller-Hamilton B, Wedel AV, Wagener TL, Stevens EM. Responses to e-cigarette health messages among young adult sexual minoritized women and nonbinary people assigned female at birth: Assessing the influence of message theme and format. Drug Alcohol Depend. 2022;231: 109249. doi:10.1016/j.drugalcdep.2021.109249	No	Not meeting inclusion criteria	Title and abstract screening

3781	Patterson JG, LaPolt DT, Miranda AR, Zettler PJ, Berman M, Roberts ME, et al. Switching stories: user testimonials on juul.com continue to contradict JUUL's switch ≠ cessation narrative. Tob Control. 2021;30: e37–e40. doi:10.1136/tobaccocontrol-2020-055816	No	Not meeting inclusion criteria	Title and abstract screening
3782	Patwardhan S. Confidence in nicotine for tobacco harm reduction-Bridging the policy-practice gap. Drug Test Anal. 2023;15: 1205–1210. doi:10.1002/dta.3413	No	Not meeting inclusion criteria	Title and abstract screening
3783	Patwardhan S. Confidence in nicotine for tobacco harm reduction - Bridging the policy-practice gap. Drug Test Anal. 2022. doi:10.1002/dta.3413	No	Not meeting inclusion criteria	Title and abstract screening
3784	Paumgartten F. A critical appraisal of the harm reduction argument for heat-not-burn tobacco products. Rev Panam Salud PublicaPan Am J Public Health. 2018;42: e161. doi:10.26633/RPSP.2018.161	No	Not meeting inclusion criteria	Title and abstract screening
3785	Paumgartten F. Heat-not-burn and electronic cigarettes: Truths and untruths about harm reduction. Rev Assoc Med Bras. 2018;64: 104–105. doi:10.1590/1806-9282.64.02.104	No	Not meeting inclusion criteria	Title and abstract screening
3786	Pavic M, Wolfer A, Li Q, Gircys A, Juritz S, Wicki A, et al. Regulatory Challenges: Is a Surrogate End Point Instead of Overall Survival Enough for Regulatory Approval of (Neo)Adjuvant Cancer Treatment? The Swissmedic Perspective. J Clin Oncol. 2023;41: 4973–4975. doi:10.1200/JCO.23.01442	No	Not meeting inclusion criteria	Title and abstract screening
3787	Pavlacic J.M., Schulenberg S.E., Pan C., Payne T.J., Williams D.C., Mann J.R. Psychological correlates of smoker's identity in adults reporting mental health diagnoses. J Subst Use. 2021;26: 558–565. doi:10.1080/14659891.2020.1862324	No	Not meeting inclusion criteria	Title and abstract screening
3788	Payne R. Use and abuse of psychoactive products. Prescriber. 2018;29: 4. doi:10.1002/psb.1661	No	Not meeting inclusion criteria	Title and abstract screening
3789	Pazdro-Zastawny K, Dorobisz K, Bobak-Sarnowska E, Zatonski T. Prevalence and Associated Factors of Cigarette Smoking Among Medical Students in Wroclaw, Poland. Risk Manag Healthc Policy. 2022;15: 509–519. doi:10.2147/RMHP.S337529	No	Not meeting inclusion criteria	Title and abstract screening
3790	Peasley-Miklus C, Klemperer E, Hughes J, Villanti A, Krishnan-Sarin S, DeSarno M, et al. The interactive effects of JUUL flavor and nicotine concentration on addiction potential. 2022. doi:10.1037/pha0000591	No	Not meeting inclusion criteria	Title and abstract screening
3791	Pebley K, Krukowski RA, Mallawaarachchi I, Wayne Talcott G, Klesges RC, Little MA. Dual and polytobacco use after a period of enforced tobacco cessation. Addict Behav. 2021;123: 107077. doi:10.1016/j.addbeh.2021.107077	No	Not meeting inclusion criteria	Title and abstract screening
3792	Peckens S., Adelman M.M., Ashcraft A.M., Xiang J., Sheppard B., King D.E. Improving Chronic Obstructive Pulmonary Disease (COPD) Symptoms Using a Team-Based Approach. J Am Board Fam Med. 2020;33: 978–985. doi:10.3122/jabfm.2020.06.200176	No	Not meeting inclusion criteria	Title and abstract screening
3793	Peckham E., Allgar V., Crosland S., Heron P., Johnston G., Newbronner E., et al. Investigating smoking and nicotine dependence among people with severe mental illness during the COVID-19 pandemic: Analysis of linked data from a UK Closing the Gap cohort. BJPsych Open. 2021;7: e86. doi:10.1192/bjo.2021.45	No	Not meeting inclusion criteria	Title and abstract screening
3794	Pednekar P., Amoah K., homer R., Ryu C., Lutchmansingh D. BULLOUS LUNG DISEASE FOLLOWING COVID-19 INFECTION. Chest. 2021;160: A356. doi:10.1016/j.chest.2021.07.358	No	Not meeting inclusion criteria	Title and abstract screening
3795	Pei-Yu ChenTao, Hui-Ling Yeh. Critical Care for a Patient with End-Stage Esophageal Cancer Complicated by Tracheoesophageal Fistula. Tzu Chi Nurs J. 2023;22: 77–86.	No	Not meeting inclusion criteria	Title and abstract screening
3796	Peiffer G, Underner M, Perriot J. [COPD and smoking cessation: Patients' expectations and responses of health professionals]. Rev Pneumol Clin. 2018;74: 375–390. doi:10.1016/j.pneumo.2018.10.001	No	Not meeting inclusion criteria	Title and abstract screening
3797	PEITRZYK K., MCCANN T., MALLICK S., PATEL N. UNILATERAL GROUND GLASS OPACITIES IN ELECTRONIC VAPING-ASSOCIATED LUNG INJURY. Chest. 2022;161: A374. doi:10.1016/j.chest.2021.12.404	No	Not meeting inclusion criteria	Title and abstract screening
3798	Pelcovits A, Olszewski AJ, Decker D, Guyer D, Leblanc TW, Egan P. Impact of Early Palliative Care on End-of-Life Outcomes in Hematologic Malignancies. J Palliat Med. 2022;25: 556–561. doi:10.1089/jpm.2021.0193	No	Not meeting inclusion criteria	Title and abstract screening
3799	Pellevoisin C., Markus J., Kaluzhny Y., Letasiova S., Klausner M., Ayehunie S. OS01-05 Human-relevant 3D models for in vitro inhalation toxicology studies. Toxicol Lett. 2023;384: S60. doi:10.1016/S0378-4274%2823%2900415-0	No	Not meeting inclusion criteria	Title and abstract screening
3800	Peluso M.J., Daniel Kelly J., Lu S., Goldberg S.A., Davidson M.C., Mathur S., et al. Persistence, Magnitude, and Patterns of Postacute Symptoms and Quality of Life Following Onset of SARS-CoV-2 Infection: Cohort Description and Approaches for Measurement. Open Forum Infect Dis. 2022;9: ofab640. doi:10.1093/ofid/ofab640	No	Not meeting inclusion criteria	Title and abstract screening
3801	Peng AR, Schnoll R, Hawk LW Jr, Cinciripini P, George TP, Lerman C, et al. Predicting smoking abstinence with biological and self-report measures of adherence to varenicline: Impact on pharmacogenetic trial outcomes. Drug Alcohol Depend. 2018;190: 72–81. doi:10.1016/j.drugalcdep.2018.04.035	No	Not meeting inclusion criteria	Title and abstract screening
3802	Peng G, Xi Y, Bellini C, Pham K, Zhuang ZW, Yan Q, et al. Nicotine dose-dependent epigenomic-wide DNA methylation changes in the mice with long-term electronic cigarette exposure. Am J Cancer Res. 2022;12: 3679–3692.	No	Not meeting inclusion criteria	Title and abstract screening
3803	Peng Y, Liu F, Qiao Y, Wang P, Du H, Si C, et al. Genetically Modified Circulating Levels of Advanced Glycation End-Products and Their Soluble Receptor (AGEs-RAGE Axis) with Risk and Mortality of Breast Cancer. Cancers. 2022;14: 6124. doi:10.3390/cancers14246124	No	Not meeting inclusion criteria	Title and abstract screening
3804	Pennell NA. Strategies and End Points in the Development of Novel Immunotherapy Trials for Patients With Unresectable, Locally Advanced Non–Small-Cell Lung Cancer. J Clin Oncol. 2022;40: 3353–3356. doi:10.1200/JCO.22.00827	No	Not meeting inclusion criteria	Title and abstract screening
3805	Penzes M., Joo T., Urban R. Perceived harm of heated tobacco products, e-cigarettes, and nicotine replacement therapy compared with conventional cigarettes among ever and current heated tobacco users. Addict Behav Rep. 2022;15: 100432. doi:10.1016/j.abrep.2022.100432	No	Not meeting inclusion criteria	Title and abstract screening
3806	Penzes M, Solimini R, Dominguez FMR, Joo T, Vardavas CI, Behrakis P. Recommendations for treating electronic cigarette and heated tobacco product dependence. Tob Prev Cessat. 2021;7: 67. doi:10.18332/tpc/142961	No	Not meeting inclusion criteria	Title and abstract screening
3807	Pepper J.K., Farrelly M.C., Watson K.A. Adolescents' understanding and use of nicotine in e-cigarettes. Addict Behav. 2018;82: 109–113. doi:10.1016/j.addbeh.2018.02.015	No	Not meeting inclusion criteria	Title and abstract screening
3808	Pepper JK, Zitney LV, Preiss A, Bukowski T, Szczypka G, Kim AE. Can social media monitoring help identify the next EVALI? An examination of Reddit posts about vitamin E acetate and Dank Vapes. Drug Alcohol Depend. 2022;230. doi:10.1016/j.drugalcdep.2021.109193https://dx.doi.org/10.1016/j.drugalcdep.2021.109193	No	Not meeting inclusion criteria	Title and abstract screening
3809	Peraza N, Bello MS, Schiff SJ, Cho J, Zhang Y, Callahan C, et al. Drug and alcohol dependence acute effects of pod-style e-cigarettes in vaping-naïve smokers. Drug Alcohol Depend. 2021;228: 109083. doi:10.1016/j.drugalcdep.2021.109083	No	Not meeting inclusion criteria	Title and abstract screening

3810	Pereira D, Dos Santos Borrego A, Garcia Alves Â. [The Health Effects and Anesthetic Management for Patients Using E-Cigarettes and Heat-Not-Burn Tobacco Products: A Narrative Review]. Acta Med Port. 2022;35: 663–668. doi:10.20344/amp.16904	No	Not meeting inclusion criteria	Title and abstract screening
3811	Perez M.F., Atuegwu N.C., Mortensen E.M., Oncken C. The inflammatory biomarker YKL-40 is elevated in the serum, but not the sputum, of E-cigarette users. Exp Lung Res. 2020;47: 55–66. doi:10.1080/01902148.2020.1847216	No	Not meeting inclusion criteria	Title and abstract screening
3812	Perez M.F., Crotty Alexander L.E. Reply: E-Cigarette, or vaping, product use-associated lung injury: A response to perez and crotty alexander. Ann Am Thorac Soc. 2020;17: 908. doi:10.1513/AnnalsATS.202003-260LE	No	Not meeting inclusion criteria	Title and abstract screening
3813	Perez MN, Madden RE, Zukin HN, Velazquez-Martin B, Barakat LP. Psychosocial needs of pediatric cancer patients and their caregivers at end of treatment: Why psychosocial screening remains important. Pediatr Blood Cancer. 2023;70: 1–9. doi:10.1002/pbc.30540	No	Not meeting inclusion criteria	Title and abstract screening
3814	Pérez-Camargo DA, Allende-Pérez SR, Rivera-Franco MM, Urbalejo-Ceniceros VI, Sevilla-González M, Arzate-Mireles CE, et al. Clinical effects of hydration, supplementary vitamins, and trace elements during end-of-life care for cancer patients. Nutr Hosp. 2023;40: 626–632. doi:10.20960/nh.04446	No	Not meeting inclusion criteria	Title and abstract screening
3815	Pérez-Martín H, Lidón-Moyano C, González-Marrón A, Fu M, Pérez-Ortuño R, Ballbè M, et al. Variation in Nicotine Metabolization According to Biological Factors and Type of Nicotine Consumer. Healthc Basel. 2023;11. doi:10.3390/healthcare11020179	No	Not meeting inclusion criteria	Title and abstract screening
3816	Perez-Padilla R., Selman M. Preface. Rev Invest Clin. 2019;71: 5–6.	No	Not meeting inclusion criteria	Title and abstract screening
3817	Pérez-Rubio G, López-Flores LA, García-Carmona S, García-Gómez L, Noé-Díaz V, Ambrocio-Ortiz E, et al. Genetic variants as risk factors for cigarette smoking at an early age and relapse to smoking cessation treatment: A pilot study. Gene. 2019;694: 93–96. doi:10.1016/j.gene.2019.01.036	No	Not meeting inclusion criteria	Title and abstract screening
3818	Pérez-Rubio G, López-Flores LA, Ramírez-Venegas A, Noé-Díaz V, García-Gómez L, Ambrocio-Ortiz E, et al. Genetic polymorphisms in CYP2A6 are associated with a risk of cigarette smoking and predispose to smoking at younger ages. Gene. 2017;628: 205–210. doi:10.1016/j.gene.2017.07.051	No	Not meeting inclusion criteria	Title and abstract screening
3819	Pergadia ML, Newcomer JW, Gilbert DG. Depression and Nicotine Withdrawal Associations with Combustible and Electronic Cigarette Use. Int J Env Res Public Health. 2020;17. doi:10.3390/ijerph17249334	No	Not meeting inclusion criteria	Title and abstract screening
3820	Pericot-Valverde I, Elliott RJ, Miller ME, Tidey JW, Gaalema DE. Posttraumatic stress disorder and tobacco use: A systematic review and meta-analysis. Addict Behav. 2018;84: 238–247. doi:10.1016/j.addbeh.2018.04.024	No	Not meeting inclusion criteria	Title and abstract screening
3821	Pericot-Valverde I, Yoon JH, Gaalema DE. Single- and cross-commodity delay discounting of money and e-cigarette liquid in experienced e-cigarette users. Drug Alcohol Depend. 2020;206: 107740. doi:10.1016/j.drugalcdep.2019.107740	No	Not meeting inclusion criteria	Title and abstract screening
3822	Pericot-Valverde I., Fernandez-Artamendi S., Gonzalez-Roz A., Lopez-Nunez C., Weidberg S.E., Secades-Villa R. Validation of a purchase task for assessing the relative reinforcing efficacy of e-cigarettes. Drug Alcohol Depend. 2017;171: e163. doi:10.1016/j.drugalcdep.2016.08.448	No	Not meeting inclusion criteria	Title and abstract screening
3823	Perkins K.A., Karelitz J.L., Boldry M.C. Nicotine acutely enhances reinforcement from non-drug rewards in humans. Front Psychiatry. 2017;8: 65. doi:10.3389/fpsyt.2017.00065	No	Not meeting inclusion criteria	Title and abstract screening
3824	Perkins KA, Karelitz JL. A Procedure to Standardize Puff Topography During Evaluations of Acute Tobacco or Electronic Cigarette Exposure. Nicotine Tob Res. 2020;22: 689–698. doi:10.1093/ntr/nty261	No	Not meeting inclusion criteria	Title and abstract screening
3825	Perkins KA, Karelitz JL, Michael VC. Effects of nicotine versus placebo e-cigarette use on symptom relief during initial tobacco abstinence. Exp Clin Psychopharmacol. 2017;25: 249–254. doi:10.1037/pha0000134	No	Not meeting inclusion criteria	Title and abstract screening
3826	Perkins KA, Kunkle N, Karelitz JL. Preliminary test of cigarette nicotine discrimination threshold in non-dependent versus dependent smokers. Drug Alcohol Depend. 2017;175: 36–41. doi:10.1016/j.drugalcdep.2017.01.033	No	Not meeting inclusion criteria	Title and abstract screening
3827	Perkins K, Herb T, Karelitz J. Discrimination of nicotine content in electronic cigarettes. 2019;91: 106-111. doi:10.1016/j.addbeh.2018.05.027	No	Not meeting inclusion criteria	Title and abstract screening
3828	Perkins K, Karelitz J, Boldry M. Reinforcement enhancing effects of nicotine via patch and nasal spray. 2018;21: 778-783. doi:10.1093/ntr/nty038	No	Not meeting inclusion criteria	Title and abstract screening
3829	Perl O., Shuster A., Heflin M., Na S., Kidwai A., Booker N., et al. Nicotine-Related Beliefs Induce Dose-Dependent Responses in the Human Brain. bioRxiv. 2022. doi:10.1101/2022.07.15.500226	No	Not meeting inclusion criteria	Title and abstract screening
3830	Perman-Howe PR, Horton M, Robson D, McDermott MS, McNeill A, Brose LS. Harm perceptions of nicotine-containing products and associated sources of information in UK adults with and without mental ill health: A cross-sectional survey. Addiction. 2022;117: 715–729. doi:10.1111/add.15657	No	Not meeting inclusion criteria	Title and abstract screening
3831	Perri RL, Perrotta D. Transcranial direct current stimulation of the prefrontal cortex reduces cigarette craving in not motivated to quit smokers: A randomized, sham-controlled study. Addict Behav. 2021;120: 106956. doi:10.1016/j.addbeh.2021.106956	No	Not meeting inclusion criteria	Title and abstract screening
3832	Perry C.L., Creamer M.R., Chaffee B.W., Unger J.B., Sutfin E.L., Kong G., et al. Research on Youth and Young Adult Tobacco Use, 2013-2018, from the Food and Drug Administration-National Institutes of Health Tobacco Centers of Regulatory Science. Nicotine Tob Res. 2020;22: 1063–1076. doi:10.1093/ntr/ntz059	No	Not meeting inclusion criteria	Title and abstract screening
3833	Persaud N, Thorpe K, Bedard M, Hwang S, Pinto A, Jüni P, et al. Cash transfer during the COVID-19 pandemic: a multicentre, randomised controlled trial. 2021;9. doi:10.1136/fmch-2021-001452	No	Not meeting inclusion criteria	Title and abstract screening
3834	Peruga A, Martínez C, Fu M, Ballbè M, Tigova O, Carnicer-Pont D, et al. [Current use of electronic cigarettes among never smoker high school students]. Gac Sanit. 2022;36: 433–438. doi:10.1016/j.gaceta.2022.01.004	No	Not meeting inclusion criteria	Title and abstract screening
3835	Peruga A, Martínez C, Fu M, Ballbè M, Tigova O, Carnicer-Pont D, et al. [Electronic cigarette use among high school students during the COVID-19 pandemic]. Gac Sanit. 2023;37: 102324. doi:10.1016/j.gaceta.2023.102324	No	Not meeting inclusion criteria	Title and abstract screening
3836	Peruga A, Rodríguez Lozano F, López MJ, Córdoba García R, Nerín I, Sureda X, et al. [Tobacco heated products: a new challenge in tobacco control]. Gac Sanit. 2022;36: 57–59. doi:10.1016/j.gaceta.2020.12.033	No	Not meeting inclusion criteria	Title and abstract screening
3837	Pesko MF. How Data Security Concerns Can Hinder Natural Experiment Research: Background and Potential Solutions. J Natl Cancer Inst Monogr. 2022;2022: 89–94. doi:10.1093/jncimonographs/lgac007	No	Not meeting inclusion criteria	Title and abstract screening
3838	Pesola F, Phillips-Waller A, Beard E, Shahab L, Sweanor D, Jarvis M, et al. Effects of reduced-risk nicotine-delivery products on smoking prevalence and cigarette sales: an observational study. Public Health Res Southampt Engl. 2023;11: 1–39. doi:10.3310/RPDN7327	No	Not meeting inclusion criteria	Title and abstract screening

3839	Peter J., Krause K., Staubach P., Wu M.A., Davis M. Chronic Urticaria and Recurrent Angioedema: Clues to the Mimics. J Allergy Clin Immunol Pract. 2021;9: 2220–2228. doi:10.1016/j.jaip.2021.03.043	No	Not meeting inclusion criteria	Title and abstract screening
3840	Peters E.N., Herrmann E.S., Cohn A.M., Coleman-Cowger V.H., Smith C., Koszowski B., et al. How Will Alcohol Research be Impacted by Future Reduction in Nicotine Content in Cigarettes? Alcohol Clin Exp Res. 2018;42: 2090–2093. doi:10.1111/acer.13869	No	Not meeting inclusion criteria	Title and abstract screening
3841	Peters M.J. Commitment to quit is essential for tobacco harm reduction. Respirology. 2021;26: 638–640. doi:10.1111/resp.14085	No	Not meeting inclusion criteria	Title and abstract screening
3842	Peters S, Van Hout E, Jansen L, van den Akker M. Psychosocial Well-Being of Older Spouses During End-of-Life Caregiving for Their Partners With Cancer: A Phenomenological Study. J Gerontol Nurs. 2022;48: 33–39. doi:10.3928/00989134-20220506-02	No	Not meeting inclusion criteria	Title and abstract screening
3843	Peterson EB, Pitzer L, Zhao X. Disparities in Nicotine Addictiveness and Cancer Harm Perceptions Among U.S. Adults: A Trend Analysis Using the Health Information National Trends Survey. Nicotine Tob Res. 2023;25: 639–647. doi:10.1093/ntr/ntac245	No	Not meeting inclusion criteria	Title and abstract screening
3844	Peterson LA, Hecht SS. Tobacco, e-cigarettes, and child health. Curr Opin Pediatr. 2017;29: 225–230. doi:10.1097/MOP.0000000000000456	No	Not meeting inclusion criteria	Title and abstract screening
3845	Petrache I, Gupta A, Hume PS, Rivera T, Schweitzer KS, Chu HW. Pathogenesis of E-Cigarette Vaping Product Use-Associated Lung Injury (EVALI). Compr Physiol. 2023;13: 4617–4630. doi:10.1002/cphy.c220022	No	Not meeting inclusion criteria	Title and abstract screening
3846	Petrella F. Electronic cigarettes, vaping-related lung injury and lung cancer: where do we stand? Eur J Cancer Prev. 2021;30: 293–296. doi:10.1097/CEJ.0000000000000630	No	Not meeting inclusion criteria	Title and abstract screening
3847	Petrella F, Rizzo S, Masiero M, Marzorati C, Casiraghi M, Bertolaccini L, et al. Clinical impact of vaping on cardiopulmonary function and lung cancer development: an update. Eur J Cancer Prev Off J Eur Cancer Prev Organ ECP. 2023;32: 584–589. doi:10.1097/CEJ.0000000000000797	No	Not meeting inclusion criteria	Title and abstract screening
3848	Petrillo LA, El-Jawahri A, Gallagher ER, Jackson VA, Temel JS, Greer JA. Patient-Reported and End-of-Life Outcomes Among Adults With Lung Cancer Receiving Targeted Therapy in a Clinical Trial of Early Integrated Palliative Care: A Secondary Analysis. J Pain Symptom Manage. 2021;62: e65–e74. doi:10.1016/j.jpainsymman.2021.02.010	No	Not meeting inclusion criteria	Title and abstract screening
3849	Petruzella S, Bochner BH, Kenney J, Whiting K, Sadeghi K, Benfante N, et al. Examining the Accuracy of Self-Reported Smoking-Related Exposure among Recently Diagnosed Nonmuscle Invasive Bladder Cancer Patients. J Urol. 2021;205: 1321–1325. doi:10.1097/JU.0000000000001571	No	Not meeting inclusion criteria	Title and abstract screening
3850	Petteway RJ. Black & Mild: A Tutorial, After the CDC. Health Promot Pr. 2022;23: 549–551. doi:10.1177/15248399221099441	No	Not meeting inclusion criteria	Title and abstract screening
3851	Pettigrew S, Santos JA, Pinho-Gomes AC, Li Y, Jones A. Exposure to e-cigarette advertising and young people's use of e-cigarettes: A four-country study. Tob Induc Dis. 2023;21: 141. doi:10.18332/tid/172414	No	Not meeting inclusion criteria	Title and abstract screening
3852	Petty-Saphon N., Kavanagh P. Towards a Tobacco Free Ireland-scaling up and strengthening quit smoking behaviour at population level. Ir J Med Sci. 2020;189: 3–10. doi:10.1007/s11845-019-02083-8	No	Not meeting inclusion criteria	Title and abstract screening
3853	Pfeffer D, Wigginton B, Gartner C, Morphett K. Smokers' Understandings of Addiction to Nicotine and Tobacco: A Systematic Review and Interpretive Synthesis of Quantitative and Qualitative Research. Nicotine Tob Res. 2018;20: 1038–1046. doi:10.1093/ntr/ntx186	No	Not meeting inclusion criteria	Title and abstract screening
3854	Pfeifer P, Auer R, Baggio S, Moggi F. A Nationwide Study of Inpatient Case Rate Incidence of Cannabis-Related Diagnoses in Switzerland. Int J Public Health. 2022;67: 1605554. doi:10.3389/ijph.2022.1605554	No	Not meeting inclusion criteria	Title and abstract screening
3855	Pg Suhaimi A.M.A., Abdul Rahman H., Ong S.K., Koh D. Predictors of non-communicable diseases screening behaviours among adult population in Brunei Darussalam: a retrospective study. J Public Health Ger. 2021;29: 1303–1312. doi:10.1007/s10389-020-01240-z	No	Not meeting inclusion criteria	Title and abstract screening
3856	Pham K, DeFina S, Wang H. E-Cigarettes Promote Macrophage-Tumor Cells Crosstalk: Focus on Breast Carcinoma Progression and Lung Metastasis. Explor Res Hypothesis Med. 2021;6: 60–66. doi:10.14218/erhm.2021.00002	No	Not meeting inclusion criteria	Title and abstract screening
3857	PHAM L.I.S.A., TOTH S., MOORE D.R. PULMONARY VENO-OCCLUSIVE DISEASE SECONDARY TO VASCULITIS. Chest. 2023;164: A6037–A6038. doi:10.1016/j.chest.2023.07.3892	No	Not meeting inclusion criteria	Title and abstract screening
3858	Phan PT, Vu GV, Ngo CQ, Pham QLT, Doan LPT, Nguyen THT, et al. Tobacco smoking and nicotine dependence among patients with respiratory diseases in Vietnam: Status and correlated factors. J Subst Abuse Treat. 2022;135: 108562. doi:10.1016/j.jsat.2021.108562	No	Not meeting inclusion criteria	Title and abstract screening
3859	Phetphum C, Prajongjeep A, Keeratisiroj O, Thawatchaijareonying K. Communication intervention to improve perceived threat of smoking-related COVID-19 and intentions to quit smoking during the COVID-19 pandemic in Thailand. Tob Induc Dis. 2022;20: 66. doi:10.18332/tid/150363	No	Not meeting inclusion criteria	Title and abstract screening
3860	Philip HE, Hein DM, Sanford NN. Racial disparities in e-cigarette use among conventionally smoking cancer survivors in the united states, 2014-2018. Subst Use Misuse. 2021; No-Specified. doi:10.1080/10826084.2021.2019786https://dx.doi.org/10.1080/10826084.2021.2019786	No	Not meeting inclusion criteria	Title and abstract screening
3861	Philip HE, Hein DM, Sanford NN. Racial Disparities in E-Cigarette Use among Conventionally Smoking Cancer Survivors in the United States, 2014-2018. Subst Use Misuse. 2022;57: 660–663. doi:10.1080/10826084.2021.2019786	No	Not meeting inclusion criteria	Title and abstract screening
3862	Phillips E, Wang TW, Husten CG, Corey CG, Apelberg BJ, Jamal A, et al. Tobacco Product Use Among Adults - United States, 2015. MMWR Morb Mortal Wkly Rep. 2017;66: 1209–1215. doi:10.15585/mmwr.mm6644a2	No	Not meeting inclusion criteria	Title and abstract screening
3863	Phillips E.M., Frates E.P., Park D.J. Lifestyle Medicine. Phys Med Rehabil Clin N Am. 2020;31: 515–526. doi:10.1016/j.pmr.2020.07.006	No	Not meeting inclusion criteria	Title and abstract screening
3864	Phillips KT, Okamoto SK, Johnson DL, Rosario MH, Manglallan KS, Pokhrel P. Correlates of tobacco use among Asian and Pacific Islander youth and young adults in the U.S.: A systematic review of the literature. Exp Clin Psychopharmacol. 2021;29: 440–455. doi:10.1037/pha0000511	No	Not meeting inclusion criteria	Title and abstract screening
3865	Phillips L., Campbell K.A., Coleman T., Ussher M., Cooper S., Lewis S., et al. Barriers and facilitators to staying smoke-free after having a baby, a qualitative study: Women's views on support needed to prevent returning to smoking postpartum. Int J Environ Res Public Health. 2021;18: 11358. doi:10.3390/ijerph182111358	No	Not meeting inclusion criteria	Title and abstract screening
3866	Phillips Z., Wilhite J.A., Hardowar K., Fisher H., Altshuler L., Holmes I., et al. CAN YOU HEAR AND SEE ME NOW? A TELEMEDICINE QUALITY IMPROVEMENT PROJECT IN A ARGE, URBAN SAFETY NET HEALTH SYSTEM. J Gen Intern Med. 2022;37: S135. doi:10.1007/s11606-022-07653-8	No	Not meeting inclusion criteria	Title and abstract screening
3867	Phillips-Waller A, Przulj D, Pesola F, Smith KM, Hajek P. Nicotine Delivery and User Ratings of IQOS Heated Tobacco System Compared With Cigarettes, Juul, and Refillable E-Cigarettes. Nicotine Tob Res. 2021;23: 1889–1894. doi:10.1093/ntr/ntab094	No	Not meeting inclusion criteria	Title and abstract screening

3868	Phua ZJ, MacInnis RJ, Hodge AM, Lynch BM, Hopper JL, Smith-Warner SA, et al. Pre-diagnostic cigarette smoking and risk of second primary cancer: The Melbourne Collaborative Cohort Study. Cancer Epidemiol. 2023;85: 102406. doi:10.1016/j.canep.2023.102406	No	Not meeting inclusion criteria	Title and abstract screening
3869	Picchio V., Pagano F., Carnevale R., D'Amico A., Cozzolino C., Floris E., et al. Exposure to Serum From Exclusive Heated Tobacco Product Smokers Induces mTOR Activation and Fibrotic Features in Human Cardiac Fibroblasts. Circulation. 2023;148. doi:10.1161/circ.148.suppl_1.15697	No	Not meeting inclusion criteria	Title and abstract screening
3870	Pichelstorfer L., Winkler-Heil R., Boy M., Hofmann W. Aerosol dynamics simulations of the anatomical variability of e-cigarette particle and vapor deposition in a stochastic lung. J Aerosol Sci. 2021;158: 105706. doi:10.1016/j.jaerosci.2020.105706	No	Not meeting inclusion criteria	Title and abstract screening
3871	Pierce J.P., Messer K., Leas E.C., Kealey S., White M.M., Benmarhnia T. A Source of Bias in Studies of E-Cigarettes and Smoking Cessation. Nicotine Tob Res. 2020;22: 861–862. doi:10.1093/ntr/ntz143	No	Not meeting inclusion criteria	Title and abstract screening
3872	Pierce JP, Luo M, McMenamin SB, Stone MD, Leas EC, Strong D, et al. Declines in cigarette smoking among US adolescents and young adults: indications of independence from e-cigarette vaping surge. Tob Control. 2023. doi:10.1136/tc-2022-057907	No	Not meeting inclusion criteria	Title and abstract screening
3873	Pierce JP, Benmarhnia T, Chen R, White M, Abrams DB, Ambrose BK, et al. Role of e-cigarettes and pharmacotherapy during attempts to quit cigarette smoking: The PATH Study 2013-16. PLoS One. 2020;15: e0237938. doi:10.1371/journal.pone.0237938	No	Not meeting inclusion criteria	Title and abstract screening
3874	Pierce JP, Chen R, Kealey S, Leas EC, White MM, Stone MD, et al. Incidence of Cigarette Smoking Relapse Among Individuals Who Switched to e-Cigarettes or Other Tobacco Products. JAMA Netw Open. 2021;4: e2128810. doi:10.1001/jamanetworkopen.2021.28810	No	Not meeting inclusion criteria	Title and abstract screening
3875	Pierce JP, Leas EC, Strong DR. Biochemical Validation of Dependence on JUUL and Other E-Cigarettes Among Youth. Pediatrics. 2023;151. doi:10.1542/peds.2022-059158	No	Not meeting inclusion criteria	Title and abstract screening
3876	Pierce JP, Zhang J, Crotty Alexander LE, Leas EC, Kealey S, White MM, et al. Daily E-cigarette Use and the Surge in JUUL Sales: 2017-2019. Pediatrics. 2022;149. doi:10.1542/peds.2021-055379	No	Not meeting inclusion criteria	Title and abstract screening
3877	Pignataro RM, Daramola C. Becoming a tobacco-free campus: A survey of student attitudes, opinions and behaviors. Tob Prev Cessat. 2020;6: 54. doi:10.18332/tpc/125915	No	Not meeting inclusion criteria	Title and abstract screening
3878	Pike JR, Tan N, Miller S, Cappelli C, Xie B, Stacy AW. The Effect of E-cigarette Commercials on Youth Smoking: A Prospective Study. Am J Health Behav. 2019;43: 1103–1118. doi:10.5993/AJHB.43.6.8	No	Not meeting inclusion criteria	Title and abstract screening
3879	Pike JR, Shono Y, Tan N, Xie B, Stacy AW. Retail outlets prompt associative memories linked to the repeated use of nicotine and tobacco products among alternative high school students in California. Addict Behav. 2019;99: 106067. doi:10.1016/j.addbeh.2019.106067	No	Not meeting inclusion criteria	Title and abstract screening
3880	Pilati S.F.M., Pilati P.V.F. A new in vitro study demonstrates that electronic cigarettes promote cellular carcinogenic characteristics. Evid Based Dent. 2023. doi:10.1038/s41432-023-00942-z	No	Not meeting inclusion criteria	Title and abstract screening
3881	Pilati SFM, Pilati PVF. A new in vitro study demonstrates that electronic cigarettes promote cellular carcinogenic characteristics. Evid Based Dent. 2023. doi:10.1038/s41432-023-00942-z	No	Not meeting inclusion criteria	Title and abstract screening
3882	Pina J.A., Namba M.D., Leyrer-Jackson J.M., Cabrera-Brown G., Gipson C.D. Social Influences on Nicotine-Related Behaviors. Int Rev Neurobiol. 2018;140: 1–32. doi:10.1016/bs.irn.2018.07.001	No	Not meeting inclusion criteria	Title and abstract screening
3883	Pincus J., Sandoval V., Dick B., Sanekommu G., Rajasekaran R., Ramasamy R., et al. E-Cigarette-Associated Endothelial Damage: A Potential Mechanism for Erectile Dysfunction. Sex Med Rev. 2022;10: 168–173. doi:10.1016/j.sxmr.2021.01.003	No	Not meeting inclusion criteria	Title and abstract screening
3884	Pingree G., DeShazo J., Schuman T. Impact of dual electronic and conventional cigarette use on diagnosis and surgery for chronic rhinosinusitis: A multicenter study. Int Forum Allergy Rhinol. 2023. doi:10.1002/alr.23281	No	Not meeting inclusion criteria	Title and abstract screening
3885	Pinho-Gomes A.-C., Alvin Santos J., Jones A., Raj Thout S., Pettigrew S. Attitudes and behaviours regarding e-cigarettes in people aged 15-30 years in the UK: a cross-sectional study. Lancet Lond Engl. 2023;402: S76. doi:10.1016/S0140-6736%2823%2902110-4	No	Not meeting inclusion criteria	Title and abstract screening
3886	Pinho-Gomes A-C, Alvin Santos J, Jones A, Raj Thout S, Pettigrew S. Attitudes and behaviours regarding e-cigarettes in people aged 15-30 years in the UK: a cross-sectional study. Lancet Lond Engl. 2023;402 Suppl 1: S76. doi:10.1016/S0140-6736(23)02110-4	No	Not meeting inclusion criteria	Title and abstract screening
3887	Pinho-Gomes A-C, Santos JA, Jones A, Thout SR, Pettigrew S. E-cigarette attitudes and behaviours amongst 15-30-year-olds in the UK. J Public Health Oxf Engl. 2023. doi:10.1093/pubmed/fdad138	No	Not meeting inclusion criteria	Title and abstract screening
3888	Pino LE, Triana I, Perez C, Piotrostanalzki A, Ruiz-Patino A, Lopes G, et al. Electronic nicotine delivery systems (ECs) and COVID-19: the perfect storm for young consumers. Clin Transl Oncol Off Publ Fed Span Oncol Soc Natl Cancer Inst Mex. 2021;23: 5–9. doi:10.1007/s12094-020-02391-x	No	Not meeting inclusion criteria	Title and abstract screening
3889	Pipe A.L. Vaping, Smoking Cessation, and Harm Reduction? Look Before You Leap. Can J Cardiol. 2021;37: 683–685. doi:10.1016/j.cjca.2021.02.002	No	Not meeting inclusion criteria	Title and abstract screening
3890	Pipe AL, Mir H. E-Cigarettes Reexamined: Product Toxicity. Can J Cardiol. 2022;38: 1395–1405. doi:10.1016/j.cjca.2022.08.001	No	Not meeting inclusion criteria	Title and abstract screening
3891	Piper M.E., Bullen C., Krishnan-Sarin S., Rigotti N.A., Steinberg M.L., Streck J.M., et al. Defining and Measuring Abstinence in Clinical Trials of Smoking Cessation Interventions: An Updated Review. Nicotine Tob Res. 2020;22: 1098–1106. doi:10.1093/ntr/ntz110	No	Not meeting inclusion criteria	Title and abstract screening
3892	Piper ME, Baker TB, Benowitz NL, Kobinsky KH, Jorenby DE. Dual Users Compared to Smokers: Demographics, Dependence, and Biomarkers. Nicotine Tob Res. 2019;21: 1279–1284. doi:10.1093/ntr/nty231	No	Not meeting inclusion criteria	Title and abstract screening
3893	Piper ME, Baker TB, Benowitz NL, Jorenby DE. Changes in Use Patterns Over 1 Year Among Smokers and Dual Users of Combustible and Electronic Cigarettes. Nicotine Tob Res. 2020;22: 672–680. doi:10.1093/ntr/ntz065	No	Not meeting inclusion criteria	Title and abstract screening
3894	Piper ME, Baker TB, Zwaga D, Bolt DM, Kobinsky KH, Jorenby DE. Understanding contexts of smoking and vaping among dual users: analysis of ecological momentary assessment data. Addiction. 2022;117: 1416–1426. doi:10.1111/add.15747	No	Not meeting inclusion criteria	Title and abstract screening
3895	Piper ME, Baker TB, Benowitz NL, Jorenby DE. "Changes in use patterns over 1 year among smokers and dual users of combustible and electronic cigarettes": Corrigendum. Nicotine Tob Res. 2020;22: 1934. doi:10.1093/ntr/ntz164https://dx.doi.org/10.1093/ntr/ntz164	No	Not meeting inclusion criteria	Title and abstract screening
3896	Pitlick M.M., Lang D.K., Meehan A.M., McCoy C.P. EVALI: A Mimicker of COVID-19. Mayo Clin Proc Innov Qual Outcomes. 2021;5: 682–687. doi:10.1016/j.mayocpiqo.2021.03.002	No	Not meeting inclusion criteria	Title and abstract screening

3897	Pitzer CR, Aboaziza EA, O'Reilly JM, Mandler WK, Olfert IM. Nicotine and Microvascular Responses in Skeletal Muscle from Acute Exposure to Cigarettes and Vaping. Int J Mol Sci. 2023;24. doi:10.3390/ijms241210208	No	Not meeting inclusion criteria	Title and abstract screening
3898	Plawecki A.M., Keller C.E., Mayerhoff R.M. Glycogenic Acanthosis: An Unusual Cause of Vocal Fold Leukoplakia. Laryngoscope. 2022;132: 1641–1643. doi:10.1002/lary.29972	No	Not meeting inclusion criteria	Title and abstract screening
3899	Plurphanswat N, Rodu B. Why can't smokers quit? Longitudinal study of smokers in the US using the Population Assessment of Tobacco and Health (PATH) waves 1 to 5. Addict Behav Rep. 2023;18: 100517. doi:10.1016/j.abrep.2023.100517	No	Not meeting inclusion criteria	Title and abstract screening
3900	Podgorski III E, Driver L, Gulati A, Abdi S. Catheter-based Techniques for Terminal Cancer Pain: A Review of Nonneuraxial Interventions with Clinical Implications for End-of-Life Pain Management. Pain Physician. 2021;24: E1137–E1146.	No	Not meeting inclusion criteria	Title and abstract screening
3901	Pokhrel P, Elwir T, Mettias H, Kawamoto CT, Oli N, Okamoto SK. The Effects of E-Cigarette Use on Alcohol and Marijuana Abuse Symptoms in an Ethnically Diverse Sample of Young Adults. Int J Env Res Public Health. 2021;18. doi:10.3390/ijerph182413159	No	Not meeting inclusion criteria	Title and abstract screening
3902	Pokhrel P, Kawamoto CT, Pagano I, Herzog TA. Trajectories of e-cigarette advertising exposure, e-cigarette use and cigarette smoking in a sample of young adults from Hawaii. Addiction. 2022;117: 2015–2026. doi:10.1111/add.15815	No	Not meeting inclusion criteria	Title and abstract screening
3903	Pokhrel P, Lam TH, Pagano I, Kawamoto CT, Herzog TA. Young adult e-cigarette use outcome expectancies: Validity of a revised scale and a short scale. Addict Behav. 2018;78: 193–199. doi:10.1016/j.addbeh.2017.11.019	No	Not meeting inclusion criteria	Title and abstract screening
3904	Pokhrel P, Lipperman-Kreda S, Wills TA, Keawe'aimoku Kaholokula J, Kawamoto CT, Amin S, et al. Ethnicity, Coronavirus Disease-Related Stress, and E-cigarette Use and Cigarette Smoking Among Young Adults: A Longitudinal Study. Nicotine Tob Res. 2023;25: 1676–1686. doi:10.1093/ntr/ntad095	No	Not meeting inclusion criteria	Title and abstract screening
3905	Pokhrel P, Phillips KT, Tam H, Kawamoto CT, Nakama M, Kaholokula JK. Ethnicity and the mechanisms of point-of-sale e-cigarette marketing's influence on behavior: A longitudinal study. Psychol Addict Behav. 2022;36: 452–465. doi:10.1037/adb0000784	No	Not meeting inclusion criteria	Title and abstract screening
3906	Pokhrel P, Kawamoto CT, Mettias H, Elwir T, Herzog T. Predictors of Discontinued E-Cigarette Use at One-Year Follow-Up in a Sample of Young Adults. Int J Environ Res Public Health. 2023;20. doi:10.3390/ijerph20064770	No	Not meeting inclusion criteria	Title and abstract screening
3907	Polosa R. Examining the evidence for the health impact of combustion-free products: progress and prospects for tobacco harm reversal and reduction. Intern Emerg Med. 2021;16: 2043–2046. doi:10.1007/s11739-021-02837-2	No	Not meeting inclusion criteria	Title and abstract screening
3908	Polosa R, Cibella F, Caponnetto P, Maglia M, Prosperini U, Russo C, et al. Health impact of E-cigarettes: a prospective 3.5-year study of regular daily users who have never smoked. Sci Rep. 2017;7: 13825. doi:10.1038/s41598-017-14043-2	No	Not meeting inclusion criteria	Title and abstract screening
3909	Polosa R, Farsalinos K, Prisco D. A double-edged sword: e-cigarettes, and other electronic nicotine delivery systems (ENDS): reply. Intern Emerg Med. 2020;15: 1119–1121. doi:10.1007/s11739-019-02228-8	No	Not meeting inclusion criteria	Title and abstract screening
3910	Polosa R, Morjaria JB, Prosperini U, Russo C, Pennisi A, Puleo R, et al. Health effects in COPD smokers who switch to electronic cigarettes: a retrospective-prospective 3-year follow-up. Int J Chron Obstruct Pulmon Dis. 2018;13: 2533–2542. doi:10.2147/COPD.S161138	No	Not meeting inclusion criteria	Title and abstract screening
3911	Polosa R, Morjaria JB, Prosperini U, Busà B, Pennisi A, Malerba M, et al. COPD smokers who switched to e-cigarettes: health outcomes at 5-year follow up. Ther Adv Chronic Dis. 2020;11: 2040622320961617. doi:10.1177/2040622320961617	No	Not meeting inclusion criteria	Title and abstract screening
3912	Polosa R, Spicuzza L, Palmucci S. Molecular Imaging of Pulmonary Inflammation: Claiming That Vaping Is More Harmful Than Smoking Is Unsupported. J Nucl Med. 2023;64: 994–995. doi:10.2967/jnumed.123.265533	No	Not meeting inclusion criteria	Title and abstract screening
3913	Polosa R, Tomaselli V, Ferrara P, Romeo AC, Rust S, Saitta D, et al. Seroepidemiological Survey on the Impact of Smoking on SARS-CoV-2 Infection and COVID-19 Outcomes: Protocol for the Troina Study. JMIR Res Protoc. 2021;10: e32285. doi:10.2196/32285	No	Not meeting inclusion criteria	Title and abstract screening
3914	Polosa R. Tobacco harm reduction and e-cigarettes. Toxicol Lett. 2017;280: S59. doi:10.1016/j.toxlet.2017.07.148	No	Not meeting inclusion criteria	Title and abstract screening
3915	Polosa R., Casale T.B., Tashkin D.P. A Close Look at Vaping in Adolescents and Young Adults in the United States. J Allergy Clin Immunol Pract. 2022;10: 2831–2842. doi:10.1016/j.jaip.2022.06.005	No	Not meeting inclusion criteria	Title and abstract screening
3916	Pomerleau OF, Benowitz NL, Stitzer ML, Henningfield JE, Hatsukami DK, Corrigall WA, et al. Srnt As An Outgrowth Of The 1988 Surgeon General's Report On Nicotine Addiction: Reflections Of The Early Presidents. Nicotine Tob Res Off J Soc Res Nicotine Tob. 2023. doi:10.1093/ntr/ntad151	No	Not meeting inclusion criteria	Title and abstract screening
3917	Ponte-Negretti CI, Wyss FS, Piskorz D, Santos RD, Villar R, Lorenzatti A, et al. Latin American Consensus on management of residual cardiometabolic risk. A consensus paper prepared by the Latin American Academy for the Study of Lipids and Cardiometabolic Risk (ALALIP) endorsed by the Inter-American Society of Cardiology (IASC), the In. Arch Cardiol Mex. 2022;92: 99–112. doi:10.24875/ACM.21000005	No	Not meeting inclusion criteria	Title and abstract screening
3918	Ponticelli D, Losa L, Campagna D, Magliuolo R, Vitale A, Cacciapuoti D, et al. Smoking habits predict adverse effects after mRNA COVID-19 vaccine: Empirical evidence from a pilot study. Public Health. 2023;219: 18–21. doi:10.1016/j.puhe.2023.03.013	No	Not meeting inclusion criteria	Title and abstract screening
3919	Ponticelli D, Madotto F, Conti S, Antonazzo IC, Vitale A, Della Ragione G, et al. Response to BNT162b2 mRNA COVID-19 vaccine among healthcare workers in Italy: a 3-month follow-up. Intern Emerg Med. 2022;17: 481–486. doi:10.1007/s11739-021-02857-y	No	Not meeting inclusion criteria	Title and abstract screening
3920	Ponugoti A., Ngo H., Stinnett S.S., Vajzovic L. Evaluation of chronic effects of e-cigarette aerosol inhalation on macular perfusion using OCT-Angiography. Invest Ophthalmol Vis Sci. 2021;62. Available: https://iovs.arvojournals.org/article.aspx?articleid=2773431	No	Not meeting inclusion criteria	Title and abstract screening
3921	Pop A.M., Coros R., Stoica A.M., Monea M. Reply to Takeshita, W.M.; Ribeiro, D.A. Comment on "Pop et al. Early Diagnosis of Oral Mucosal Alterations in Smokers and E-Cigarette Users Based on Micronuclei Count: A Cross-Sectional Study among Dental Students. Int. J. Environ. Res. Public Health 2021. Int J Environ Res Public Health. 2022;19: 3845. doi:10.3390/ijerph19073845	No	Not meeting inclusion criteria	Title and abstract screening
3922	Popova L. Carpe covid: using COVID-19 to communicate about harms of tobacco products. Tob Control. 2022;31: 397–398. doi:10.1136/tobaccocontrol-2020-056276	No	Not meeting inclusion criteria	Title and abstract screening
3923	Popova L, Henderson K, Kute N, Singh-Looney M, Ashley DL, Reynolds RM, et al. "I'm Bored and I'm Stressed": A Qualitative Study of Exclusive Smokers, ENDS Users, and Transitioning Smokers or ENDS Users in the Time of COVID-19. Nicotine Tob Res. 2023;25: 185–192. doi:10.1093/ntr/ntab199	No	Not meeting inclusion criteria	Title and abstract screening
3924	Popova L, McDonald EA, Sidhu S, Barry R, Richers Maruyama TA, Sheon NM, et al. Perceived harms and benefits of tobacco, marijuana, and electronic vaporizers among young adults in Colorado: implications for health education and research. Addiction. 2017;112: 1821–1829. doi:10.1111/add.13854	No	Not meeting inclusion criteria	Title and abstract screening
3925	Popova L., Majeed B., Owusu D., Spears C.A., Ashley D.L. Who are the smokers who never plan to quit and what do they think about the risks of using tobacco products? Addict Behav. 2018;87: 62–68. doi:10.1016/j.addbeh.2018.06.024	No	Not meeting inclusion criteria	Title and abstract screening

3926	Popova L, Fairman RT, Akani B, Dixon K, Weaver SR. "Don't do vape, bro!" A qualitative study of youth's and parents' reactions to e-cigarette prevention advertisements. Addict Behav. 2021;112: 106565. doi:10.1016/j.addbeh.2020.106565	No	Not meeting inclusion criteria	Title and abstract screening
3927	Posner H, Romm KF, Henriksen L, Bernat D, Berg CJ. Reactions to Sales Restrictions on Flavored Vape Products or All Vape Products Among Young Adults in the United States. Nicotine Tob Res. 2022;24: 333–341. doi:10.1093/ntr/ntab154	No	Not meeting inclusion criteria	Title and abstract screening
3928	Pougnet R, Lucas D, Pougnet L, Chapalain D, Fortin C, Loddé B, et al. [Smoking, vaping among hospital staff during the Covid-19 pandemic: Appraisal of the "tobacco-free hospital, campus" campaign in Brest]. Rev Mal Respir. 2022;39: 413–419. doi:10.1016/j.rmr.2022.01.020	No	Not meeting inclusion criteria	Title and abstract screening
3929	Power GM, Tyrrell J, Frayling TM, Smith GD, Richardson TG, Davey Smith G. Mendelian Randomization Analyses Suggest Childhood Body Size Indirectly Influences End Points From Across the Cardiovascular Disease Spectrum Through Adult Body Size. J Am Heart Assoc. 2021;10: 1–8. doi:10.1161/JAHA.121.021503	No	Not meeting inclusion criteria	Title and abstract screening
3930	Powers J.M., Heckman B.W., LaRowe L.R., Ditre J.W. Smokers with pain are more likely to report use of e-cigarettes and other nicotine products. Exp Clin Psychopharmacol. 2020;28: 601–608. doi:10.1037/pha0000335	No	Not meeting inclusion criteria	Title and abstract screening
3931	Powers JM, Maisto SA, Zvolensky MJ, Heckman BW, Ditre JW. Longitudinal associations between pain and use of cigarettes and e-cigarettes in the Population Assessment of Tobacco and Health (PATH) Study. Nicotine Tob Res. 2023;25: 404–411. doi:10.1093/ntr/ntac197	No	Not meeting inclusion criteria	Title and abstract screening
3932	Powers JM, Maisto SA, Zvolensky MJ, Heckman BW, Ditre JW. Longitudinal Associations Between Pain and Use of Cigarettes and E-Cigarettes in the Population Assessment of Tobacco and Health (PATH) Study. Nicotine Tob Res Off J Soc Res Nicotine Tob. 2022. doi:10.1093/ntr/ntac197	No	Not meeting inclusion criteria	Title and abstract screening
3933	Powers JM, LaRowe LR, Garey L, Zvolensky MJ, Ditre JW. Pain intensity, e-cigarette dependence, and cessation-related outcomes: The moderating role of pain-related anxiety. Addict Behav. 2020;111: 106548. doi:10.1016/j.addbeh.2020.106548	No	Not meeting inclusion criteria	Title and abstract screening
3934	Powers JM, LaRowe LR, Lape EC, Zvolensky MJ, Ditre JW. Anxiety sensitivity, pain severity and co-use of cigarettes and e-cigarettes among adults with chronic pain. J Behav Med. 2021;44: 392–401. doi:10.1007/s10865-021-00210-4	No	Not meeting inclusion criteria	Title and abstract screening
3935	Prabha S., Nadkarni S., Agarwal V., Horton T. 2 Methyl-AP-237 Overdose: A Case Report. J Addict Med. 2022;16: e316.	No	Not meeting inclusion criteria	Title and abstract screening
3936	Pradère P, Ruppert AM, Peiffer G, Perriot J, Adler M, Underner M. [Inhaled marijuana and the lung, a toxic cocktail?]. Rev Mal Respir. 2022;39: 708–718. doi:10.1016/j.rmr.2022.08.007	No	Not meeting inclusion criteria	Title and abstract screening
3937	Prasad KN, Bondy SC. Electronic cigarette aerosol increases the risk of organ dysfunction by enhancing oxidative stress and inflammation. Drug Chem Toxicol. 2022;45: 2561–2567. doi:10.1080/01480545.2021.1972680	No	Not meeting inclusion criteria	Title and abstract screening
3938	Prater LC, O'Rourke B, Schnell P, Xu W, Li Y, Gustin J, et al. Examining the Association of Billed Advance Care Planning With End-of-Life Hospital Admissions Among Advanced Cancer Patients in Hospice. Am J Hosp Palliat Med. 2022;39: 504–510. doi:10.1177/10499091211039449	No	Not meeting inclusion criteria	Title and abstract screening
3939	Pratiti R., Mukherjee D. Epidemiology and adverse consequences of hookah/waterpipe use: A systematic review. Cardiovasc Hematol Agents Med Chem. 2019;17: 82–93. doi:10.2174/1871525717666190904151856	No	Not meeting inclusion criteria	Title and abstract screening
3940	Pratt SI, Ferron JC, Brunette MF, Santos M, Sargent J, Xie H. E-Cigarette Provision to Promote Switching in Cigarette Smokers With Serious Mental Illness-A Randomized Trial. Nicotine Tob Res. 2022;24: 1405–1412. doi:10.1093/ntr/ntac082	No	Not meeting inclusion criteria	Title and abstract screening
3941	Presant C.A., Ashing K., Yeung S., Macalintal J., Gascon B., Sandoval A., et al. Empowering tobacco using cancer patient initiation of tobacco cessation by a personal pathway to success program during preoperative patient counseling: A feasibility study. Cancer Res. 2022;82. doi:10.1158/1538-7445.AM2022-LB553	No	Not meeting inclusion criteria	Title and abstract screening
3942	Presant C.A., Ashing K., Yeung S., Macalintal J., Gascon B., Sandoval A., et al. Enhancing minority patient participation in tobacco cessation by a personal pathway to success program. Cancer Epidemiol Biomarkers Prev. 2023;32. doi:10.1158/1538-7755.DISP22-A095	No	Not meeting inclusion criteria	Title and abstract screening
3943	Presant CA, Macalintal J, Ashing KT, Yeung S, Tiep B, West H, et al. Tragic Triad of Tobacco Dependence, Cancer, and COVID-19 Pandemic: An Urgent Call for Attention by Health Care Systems and Professionals. JCO Oncol Pr. 2022;18: 99–105. doi:10.1200/OP.21.00625	No	Not meeting inclusion criteria	Title and abstract screening
3944	Presley CJ, Kaur K, Han L, Soulos PR, Zhu W, Corneau E, et al. Aggressive End-of-Life Care in the Veterans Health Administration versus Fee-for-Service Medicare among Patients with Advanced Lung Cancer. J Palliat Med. 2022;25: 932–939. doi:10.1089/jpm.2021.0436	No	Not meeting inclusion criteria	Title and abstract screening
3945	Preteroti M, Wilson ET, Eidelman DH, Baglole CJ. Modulation of pulmonary immune function by inhaled cannabis products and consequences for lung disease. Respir Res. 2023;24: 95. doi:10.1186/s12931-023-02399-1	No	Not meeting inclusion criteria	Title and abstract screening
3946	Prieto Fernandez A, Palomo Antequera B, Del Castillo Arango K, Blanco Guindel M, Nava Tomas ME, Mesa Alvarez AM. Inhalational lung diseases. Radiologia. 2022;64 Suppl 3: 290–300. doi:10.1016/j.rxeng.2022.10.007	No	Not meeting inclusion criteria	Title and abstract screening
3947	Prigerson HG, Neugut AI. You Get (offered) What You (can) Pay for: Explaining Disparities in End-of-Life Cancer Care. J Clin Oncol. 2023;41: 4721–4723. doi:10.1200/JCO.23.00608	No	Not meeting inclusion criteria	Title and abstract screening
3948	Prijic Z, Igic R. Cigarette smoking and medical students. J BUON Off J Balk Union Oncol. 2021;26: 1709–1718.	No	Not meeting inclusion criteria	Title and abstract screening
3949	Printz C. US Food and Drug Administration considers comments on proposed nicotine product regulations: Some criticize the agency for not moving fast enough to stop youth e-cigarette use. Cancer. 2018;124: 3959–3960. doi:10.1002/cncr.31792	No	Not meeting inclusion criteria	Title and abstract screening
3950	Printz C. Fighting the teen vaping epidemic: With rates of adolescent vaping on the rise, experts caution that new federal rules targeting e-cigarettes may not be strong enough. Cancer. 2020;126: 1147–1148. doi:10.1002/cncr.32779	No	Not meeting inclusion criteria	Title and abstract screening
3951	Prochaska JJ, Benowitz NL. Current advances in research in treatment and recovery: Nicotine addiction. Sci Adv. 2019;5: eaay9763. doi:10.1126/sciadv.aay9763	No	Not meeting inclusion criteria	Title and abstract screening
3952	Prochaska JJ, Brown-Johnson C, Baiocchi M, Lazaro AS, Chieng A, Stinson S, et al. Treating tobacco dependence to aid re-employment among job-seekers: A randomized controlled trial. Prev Med. 2020;141: 106259. doi:10.1016/j.ypmed.2020.106259	No	Not meeting inclusion criteria	Title and abstract screening
3953	Prochaska JJ, Vogel EA, Benowitz N. Nicotine delivery and cigarette equivalents from vaping a JUULpod. Tob Control. 2022;31: e88–e93. doi:10.1136/tobaccocontrol-2020-056367	No	Not meeting inclusion criteria	Title and abstract screening
3954	Prokhorov AV, Calabro KS, Arya A, Russell S, Czerniak KW, Botello GC, et al. Mobile Text Messaging for Tobacco Risk Communication Among Young Adult Community College Students: Randomized Trial of Project Debunk. JMIR Mhealth Uhealth. 2021;9: e25618. doi:10.2196/25618	No	Not meeting inclusion criteria	Title and abstract screening

3955	Przewozniak K., Koczkodaj P., Ciuba A. The patterns and trends in e-cigarette consumption and use in Poland: One of the biggest markets in Europe. Tob Induc Dis. 2019;17. doi:10.18332/tid/112072	No	Not meeting inclusion criteria	Title and abstract screening
3956	Przewozniak K., Przepiorka I., Cedzynska M. Are Electronic Nicotine Delivery Systems (ENDS) an effective aid in smoking cessation?: A critical look. Tob Induc Dis. 2019;17. doi:10.18332/tid/111266	No	Not meeting inclusion criteria	Title and abstract screening
3957	Przulj D, Hajek P, Phillips-Waller A. E-Cigarettes versus Nicotine-Replacement Therapy for Smoking Cessation. Reply. N Engl J Med. 2019;380: 1974–1975. doi:10.1056/NEJMc1903758	No	Not meeting inclusion criteria	Title and abstract screening
3958	Przulj D, Pesola F, Myers Smith K, McRobbie H, Coleman T, Lewis S, et al. Helping pregnant smokers quit: a multi-centre randomised controlled trial of electronic cigarettes versus nicotine replacement therapy. Health Technol Assess. 2023;27: 1–53. doi:10.3310/AGTH6901	No	Not meeting inclusion criteria	Title and abstract screening
3959	Psonka Y, Vannimenus C. [Perception of ENDS among French high school students in Nord and Pas-de-Calais]. Rev Mal Respir. 2023. doi:10.1016/j.rmr.2023.08.001	No	Not meeting inclusion criteria	Title and abstract screening
3960	Pulvers K, Correa JB, Krebs P, El Shahawy O, Marez C, Doran N, et al. JUUL E-Cigarette Quit Attempts and Cessation Perceptions in College Student JUUL E-Cigarette Users. Am J Health Promot. 2021;35: 624–632. doi:10.1177/0890117120982408	No	Not meeting inclusion criteria	Title and abstract screening
3961	Pulvers K, Emami AS, Nollen NL, Romero DR, Strong DR, Benowitz NL, et al. Tobacco Consumption and Toxicant Exposure of Cigarette Smokers Using Electronic Cigarettes. Nicotine Tob Res. 2018;20: 206–214. doi:10.1093/ntr/ntw333	No	Not meeting inclusion criteria	Title and abstract screening
3962	Pulvers K, Rice M, Ahluwalia JS, Arnold MJ, Marez C, Nollen NL. "It is the One Thing that has Worked": facilitators and barriers to switching to nicotine salt pod system e-cigarettes among African American and Latinx people who smoke: a content analysis. Harm Reduct J. 2021;18: 98. doi:10.1186/s12954-021-00543-y	No	Not meeting inclusion criteria	Title and abstract screening
3963	Purani H, Friedrichsen S, Allen AM. Sleep quality in cigarette smokers: Associations with smoking-related outcomes and exercise. Addict Behav. 2019;90: 71–76. doi:10.1016/j.addbeh.2018.10.023	No	Not meeting inclusion criteria	Title and abstract screening
3964	Puri C., Zulfiqar S., Qureshi A., Hyun Rhee J. VAPING-INDUCED DIFFUSE ALVEOLAR HEMORRHAGE. Chest. 2021;160: A696. doi:10.1016/j.chest.2021.07.660	No	Not meeting inclusion criteria	Title and abstract screening
3965	Qanash S, Alemam S, Mahdi E, Softah J, Touman AA, Alsulami A. Electronic cigarette among health science students in Saudi Arabia. Ann Thorac Med. 2019;14: 56–62. doi:10.4103/atm.ATM_76_18	No	Not meeting inclusion criteria	Title and abstract screening
3966	Qasim H, Alarabi AB, Alzoubi KH, Karim ZA, Alshbool FZ, Khasawneh FT. The effects of hookah/waterpipe smoking on general health and the cardiovascular system. Environ Health Prev Med. 2019;24: 58. doi:10.1186/s12199-019-0811-y	No	Not meeting inclusion criteria	Title and abstract screening
3967	Qi H, Chang X, Wang K, Xu Q, Liu M, Han B. Comparative analyses of transcriptome sequencing and carcinogenic exposure toxicity of nicotine and 6-methyl nicotine in human bronchial epithelial cells. Toxicol Vitro Int J Publ Assoc BIBRA. 2023;93: 105661. doi:10.1016/j.tiv.2023.105661	No	Not meeting inclusion criteria	Title and abstract screening
3968	Qiu H, Zhang H, Han DD, Derakhshandeh R, Wang X, Goyal N, et al. Increased vulnerability to atrial and ventricular arrhythmias caused by different types of inhaled tobacco or marijuana products. Heart Rhythm. 2023;20: 76–86. doi:10.1016/j.hrthm.2022.09.021	No	Not meeting inclusion criteria	Title and abstract screening
3969	Qiu Y, Chen H, Dai Y, Bao B, Tian L, Chen Y. Nontherapeutic Risk Factors of Different Grouped Stage IIIC Breast Cancer Patients' Mortality: A Study of the US Surveillance, Epidemiology, and End Results Database. Breast J. 2022;2022: 1–9. doi:10.1155/2022/6705052	No	Not meeting inclusion criteria	Title and abstract screening
3970	Quan Ng D., Milam J., Ritt-Olson A., Freyer D.R., Miller K.A., Thomas S.M., et al. ASSOCIATIONS BETWEEN DEPRESSION, COGNITIVE TOXICITY, AND SUBSTANCE USE IN CHILDHOOD CANCER SURVIVORS: A MEDIATION ANALYSIS. Support Care Cancer. 2022;30: S149. doi:10.1007/s00520-022-07099-y	No	Not meeting inclusion criteria	Title and abstract screening
3971	Qureshi MA, Vernooij RWM, La Rosa GRM, Polosa R, O'Leary R. Respiratory health effects of e-cigarette substitution for tobacco cigarettes: a systematic review. Harm Reduct J. 2023;20: 143. doi:10.1186/s12954-023-00877-9	No	Not meeting inclusion criteria	Title and abstract screening
3972	Qwaider YZ, Sell NM, Stafford CE, Kunitake H, Ricciardi R, Bordeianou LG, et al. The time Interval Between the End of Radiotherapy and Surgery Does Not Affect Outcomes in Rectal Cancer. Am Surg. 2023;89: 831–836. doi:10.1177/00031348211047215	No	Not meeting inclusion criteria	Title and abstract screening
3973	Rabade-Castedo C., de Granda-Orive J.I., Gonzalez-Barcala F.J. Incremento de la prevalencia del tabaquismo: ?causas y actuacion? Arch Bronconeumol. 2019;55: 557–558. doi:10.1016/j.arbr.2019.06.006	No	Not meeting inclusion criteria	Title and abstract screening
3974	Rabenstein A., Ruther T. Use of e-cigarettes - Opportunities and risks. Atemwegs- Lungenkrankh. 2019;45: 350–355. doi:10.5414/ATX02399	No	Not meeting inclusion criteria	Title and abstract screening
3975	Rabinow L.A., Ipp L., Avery J., Green C.M. 180. A Missing Patch in Training: Pediatric Residents' Attitudes, Practices, Training and Confidence Around Prescribing Nicotine Replacement Therapy. J Adolesc Health. 2023;72: S100–S101. doi:10.1016/j.jadohealth.2022.11.202	No	Not meeting inclusion criteria	Title and abstract screening
3976	Rades D, Segedin B, Schild SE, Lomidze D, Veninga T, Cacicedo J. Identifying patients with malignant spinal cord compression (MSCC) near end of life who can benefit from palliative radiotherapy. Radiat Oncol. 2022;17: 1–8. doi:10.1186/s13014-022-02117-z	No	Not meeting inclusion criteria	Title and abstract screening
3977	Raghu VK, Walia AS, Zinzuwadia AN, Goiffon RJ, Shepard JO, Aerts HJWL, et al. Validation of a Deep Learning-Based Model to Predict Lung Cancer Risk Using Chest Radiographs and Electronic Medical Record Data. JAMA Netw Open. 2022;5: e2248793. doi:10.1001/jamanetworkopen.2022.48793	No	Not meeting inclusion criteria	Title and abstract screening
3978	Raghunathan N.J., Brens J., Vemuri S., Li Q.S., Mao J.J., Korenstein D. In the weeds: a retrospective study of patient interest in and experience with cannabis at a cancer center. Support Care Cancer. 2022;30: 7491–7497. doi:10.1007/s00520-022-07170-8	No	Not meeting inclusion criteria	Title and abstract screening
3979	Raheja V., Amjad M., Ochieng P. Stubborn Valley Fever in the Electric City. Am J Respir Crit Care Med. 2022;205. doi:10.1164/ajrccm-conference.2022.205.1_MeetingAbstracts.A4537	No	Not meeting inclusion criteria	Title and abstract screening
3980	Rahman A, Alqaisi S, Alzakhari R, Saith S. Characterization and Summarization of the Impact of Electronic Cigarettes on the Cardiovascular System: A Systematic Review and Meta-Analysis. Cureus. 2023;15: e39528. doi:10.7759/cureus.39528	No	Not meeting inclusion criteria	Title and abstract screening
3981	Rahman M.A., Joseph B., Nimmi N. Electronic Cigarettes or Vaping: Are There Any Differences in the Profiles, Use and Perceptions between a Developed and a Developing Country? Int J Environ Res Public Health. 2022;19: 1673. doi:10.3390/ijerph19031673	No	Not meeting inclusion criteria	Title and abstract screening
3982	Rahman MA, Cai L, Tawfik SA, Tucker S, Burton A, Perera G, et al. Nicotine Sensors for Wearable Battery-Free Monitoring of Vaping. ACS Sens. 2022;7: 82–88. doi:10.1021/acssensors.1c01633	No	Not meeting inclusion criteria	Title and abstract screening
3983	Rai S, Hormozdyaran S, Burns J, Amodio JB, Quizon AI. Diagnosis of EVALI in Adolescents During the COVID-19 Pandemic: A Case Series. Hosp Pediatr. 2022;12: 538–543. doi:10.1542/hpeds.2021-006110	No	Not meeting inclusion criteria	Title and abstract screening

3984	Raitasalo K, Bye EK, Pisinger C, Scheffels J, Tokle R, Kinnunen JM, et al. Single, Dual, and Triple Use of Cigarettes, e-Cigarettes, and Snus among Adolescents in the Nordic Countries. Int J Env Res Public Health. 2022;19. doi:10.3390/ijerph19020683	No	Not meeting inclusion criteria	Title and abstract screening
3985	Raja A., Chhaya D.G., Veerappan A., Awada C., Seshan S.V., Zelikoff J.T., et al. Effects of Electronic Cigarette Aerosol Exposure on Kidney Health in Diabetic/Obese Mice. J Am Soc Nephrol. 2023;34: 834.	No	Not meeting inclusion criteria	Title and abstract screening
3986	Raja A., Zelikoff J.T., Jaimes E.A. A contemporary review of nephrotoxicity and e-cigarette use. Curr Opin Toxicol. 2022;31: 100361. doi:10.1016/j.cotox.2022.100361	No	Not meeting inclusion criteria	Title and abstract screening
3987	Raja J., Khouzam A., Khouzam N., Khouzam R.N. Smoke and Heart Should Stay Apart: A Look at E Cigarettes and Other Alternatives to Conventional Cigarettes, and Their Impact on Cardiovascular Health. Curr Probl Cardiol. 2021;46: 100640. doi:10.1016/j.cpcardiol.2020.100640	No	Not meeting inclusion criteria	Title and abstract screening
3988	Raja J., Khouzam A., Khouzam N., Khouzam R. Smoke and heart should stay apart: E cigarettes and other alternatives to conventional cigarettes' impact on cardiovascular health. J Investig Med. 2021;69: 498. doi:10.1136/jim-2021-SRMC.203	No	Not meeting inclusion criteria	Title and abstract screening
3989	Ramakrishnan P, Ashok A, Hebbar PB. COVID-19: A policy window for E-cigarette regulation. Indian J Public Health. 2021;65: 324–325. doi:10.4103/ijph.IJPH_235_21	No	Not meeting inclusion criteria	Title and abstract screening
3990	Ramalingam S. PL03.03 The Disparity of Lung Cancer Prevention, Diagnosis and Treatment Around the World...What Is the Role of IASLC. J Thorac Oncol. 2019;14: S9–S10. doi:10.1016/j.jtho.2019.08.064	No	Not meeting inclusion criteria	Title and abstract screening
3991	Ramamurthi D, Chau C, Jackler RK. Exploitation of the COVID-19 pandemic by e-cigarette marketers. Tob Control. 2021;30: e56–e59. doi:10.1136/tobaccocontrol-2020-055855	No	Not meeting inclusion criteria	Title and abstract screening
3992	Ramclam A, Taing M, Kyburz B, Williams T, Casey K, Correa-Fernández V, et al. An epidemic and a pandemic collide: Assessing the feasibility of tobacco treatment among vulnerable groups at COVID-19 protective lodging. Fam Syst Health. 2022;40: 120–125. doi:10.1037/fsh0000658	No	Not meeting inclusion criteria	Title and abstract screening
3993	Ramenzoni LL, Schneider A, Fox SC, Meyer M, Meboldt M, Attin T, et al. Cytotoxic and Inflammatory Effects of Electronic and Traditional Cigarettes on Oral Gingival Cells Using a Novel Automated Smoking Instrument: An In Vitro Study. Toxics. 2022;10. doi:10.3390/toxics10040179	No	Not meeting inclusion criteria	Title and abstract screening
3994	Ramírez-Cervantes KL, García-Rodríguez E. [Patterns of electronic cigarette and tobacco heating products use in Spain.]. Rev Esp Salud Publica. 2021;95.	No	Not meeting inclusion criteria	Title and abstract screening
3995	Ramôa CP, Eissenberg T, Sahingur SE. Increasing popularity of waterpipe tobacco smoking and electronic cigarette use: Implications for oral healthcare. J Periodontal Res. 2017;52: 813–823. doi:10.1111/jre.12458	No	Not meeting inclusion criteria	Title and abstract screening
3996	Ramôa C, Hiler M, Spindle T, Lopez A, Karaoghlanian N, Lipato T, et al. Electronic cigarette nicotine delivery can exceed that of combustible cigarettes: a preliminary report. 2016;25: e6-9. doi:10.1136/tobaccocontrol-2015-052447	No	Not meeting inclusion criteria	Title and abstract screening
3997	Ramos-Fernández MR, Ortiz-Ortiz KJ, Torres-Cintrón CR, Tortolero-Luna G. Patterns of End-of-Life Care as Measured by Emergency Room Visits Among Cancer Patients in Puerto Rico. Am J Hosp Palliat Med. 2022;39: 72–78. doi:10.1177/10499091211025743	No	Not meeting inclusion criteria	Title and abstract screening
3998	Rampal L. COVID-19 pandemic update. Med J Malaysia. 2020;75: 195–198.	No	Not meeting inclusion criteria	Title and abstract screening
3999	Rams A, Kowitt S, Ritchie C, Sutfin EL, Sheeran P, Noar SM. `Awareness and perceptions of FDA's JUUL Marketing Denial Order:A national study of US adolescents. Nicotine Tob Res. 2023. doi:10.1093/ntr/ntad191	No	Not meeting inclusion criteria	Title and abstract screening
4000	RANDHAWA R., SHAH T. PATIENT WITH EHLERS-DANLOS SYNDROME AND A RARE OBSTRUCTIVE PHYSIOLOGY ON PULMONARY FUNCTION STUDIES. Chest. 2022;162: A2197. doi:10.1016/j.chest.2022.08.1816	No	Not meeting inclusion criteria	Title and abstract screening
4001	Ranjit A, Latvala A, Kinnunen TH, Kaprio J, Korhonen T. Depressive symptoms predict smoking cessation in a 20-year longitudinal study of adult twins. Addict Behav. 2020;108: 106427. doi:10.1016/j.addbeh.2020.106427	No	Not meeting inclusion criteria	Title and abstract screening
4002	Ranjit A, McCutchan G, Brain K, Poole R. "That's the whole thing about vaping, it's custom tasty goodness": a meta-ethnography of young adults' perceptions and experiences of e-cigarette use. Subst Abuse Treat Prev Policy. 2021;16: 85. doi:10.1186/s13011-021-00416-4	No	Not meeting inclusion criteria	Title and abstract screening
4003	Ranpara A, Stefaniak AB, Williams K, Fernandez E, LeBouf RF. Modeled Respiratory Tract Deposition of Aerosolized Oil Diluents Used in DELTA9-THC-Based Electronic Cigarette Liquid Products. Front Public Health. 2021;9: 744166. doi:10.3389/fpubh.2021.744166	No	Not meeting inclusion criteria	Title and abstract screening
4004	Rao I, Lydall G. Psilocybin for the treatment of substance use disorders. Adv Psychedelic Med State---Art Ther Appl. 2019; 127–147.	No	Not meeting inclusion criteria	Title and abstract screening
4005	Rapoport A, Gupta S. Children and adolescents with hematologic cancers deserve better end-of-life care. Cancer 0008543X. 2021;127: 3724–3726. doi:10.1002/cncr.33763	No	Not meeting inclusion criteria	Title and abstract screening
4006	Rapp JL, Alpert N, Wilson KM, Flores RM, Taioli E. Changes in E-Cigarette Perceptions Over Time: A National Youth Tobacco Survey Analysis. Am J Prev Med. 2021;61: 174–181. doi:10.1016/j.amepre.2021.03.006	No	Not meeting inclusion criteria	Title and abstract screening
4007	Rashidi A, Garimella PS, Al-Asaad A, Kharadjian T, Torres MN, Thakkar J. Anemia Management in the Cancer Patient With CKD and End-Stage Kidney Disease. Adv Chronic Kidney Dis. 2022;29: 180–180. doi:10.1053/j.ackd.2022.03.005	No	Not meeting inclusion criteria	Title and abstract screening
4008	Rasmussen M, Larsson M, Gilljam H, Adami J, Wärjerstam S, Post A, et al. Effectiveness of tobacco cessation interventions for different groups of tobacco users in Sweden: a study protocol for a national prospective cohort study. BMJ Open. 2022;12: e053090. doi:10.1136/bmjopen-2021-053090	No	Not meeting inclusion criteria	Title and abstract screening
4009	Raspe M, Bals R, Hering T, Pankow W, Rupp A, Rustler C, et al. [COVID-19 and Smoking - A Position Paper by the DGP Taskforce for Smoking Cessation]. Pneumologie. 2021;75: 846–855. doi:10.1055/a-1503-1744	No	Not meeting inclusion criteria	Title and abstract screening
4010	Rattanawong P., Firouzbakht T., Sorajja D. VAPING CARBOXY-TETRAHYDROCANNABINOL UNMASKS BRUGADA PATTERN AND INDUCES VENTRICULAR FIBRILLATION IN BRUGADA SYNDROME. J Am Coll Cardiol. 2021;77: 2066. doi:10.1016/S0735-1097%2821%2903422-7	No	Not meeting inclusion criteria	Title and abstract screening
4011	Rauschert C, Möckl J, Seitz NN, Wilms N, Olderbak S, Kraus L. The Use of Psychoactive Substances in Germany. Dtsch Arztebl Int. 2022;119: 527–534. doi:10.3238/arztebl.m2022.0244	No	Not meeting inclusion criteria	Title and abstract screening
4012	Ray A., Mohapatra D. Life threatening effects of oral habits. Indian J Public Health Res Dev. 2019;10: 1067–1070. doi:10.5958/0976-5506.2019.03647.7	No	Not meeting inclusion criteria	Title and abstract screening

4013	Raymond B.H., Collette-Merrill K., Harrison R.G., Jarvis S., Rasmussen R.J. The nicotine content of a sample of E-cigarette liquid manufactured in the United States. J Addict Med. 2018;12: 127–131. doi:10.1097/ADM.0000000000000376	No	Not meeting inclusion criteria	Title and abstract screening
4014	Rayner RE, Makena P, Prasad GL, Cormet-Boyaka E. Cigarette smoke preparations, not electronic nicotine delivery system preparations, induce features of lung disease in a 3D lung repeat-dose model. Am J Physiol Lung Cell Mol Physiol. 2021;320: L276–L287. doi:10.1152/ajplung.00452.2020	No	Not meeting inclusion criteria	Title and abstract screening
4015	RBR-8ht5nqq. Tele Rehabilitation for Women with Urinary Incontinence after Gynecological Pelvic Cancer Treatment. 2022. Available: https://www.cochranelibrary.com/central/doi/10.1002/central/CN-02379183/full	No	Not meeting inclusion criteria	Title and abstract screening
4016	RBR-97rb5wk. Physiotherapy for Women After Gynecologic Pelvic Cancer Treatment. 2021. Available: https://www.cochranelibrary.com/central/doi/10.1002/central/CN-02379187/full	No	Not meeting inclusion criteria	Title and abstract screening
4017	Reblin M, Iacob E, Tay DL, Li H, Thomas Hebdon MC, Beck A, et al. Family Caregiver Reports of Their Own and Patient Symptoms in Cancer Home Hospice Approaching End-of-Life. Am J Hosp Palliat Med. 2023;40: 508–516. doi:10.1177/10499091221108119	No	Not meeting inclusion criteria	Title and abstract screening
4018	Rebuli ME. Phosphatidylethanolamines as biomarkers of e-cigarette or vaping product use-associated lung injury. Pediatr Pulmonol. 2022;57: 1792–1794. doi:10.1002/ppul.25951	No	Not meeting inclusion criteria	Title and abstract screening
4019	Rebuli ME, Rose JJ, Noël A, Croft DP, Benowitz NL, Cohen AH, et al. The E-cigarette or Vaping Product Use-Associated Lung Injury Epidemic: Pathogenesis, Management, and Future Directions: An Official American Thoracic Society Workshop Report. Ann Am Thorac Soc. 2023;20: 1–17. doi:10.1513/AnnalsATS.202209-796ST	No	Not meeting inclusion criteria	Title and abstract screening
4020	Recchia A, Rizzi B, Favero A, Nobili A, Pasina L. Prevalence of Delirium in End-of-Life Palliative Care Patients: An Observational Study. Med Princ Pract. 2022;31: 118–124. doi:10.1159/000521994	No	Not meeting inclusion criteria	Title and abstract screening
4021	Reddy DR, Muthu M, Cuenca J, Wegner R, Crommett J, Gutierrez C, et al. 536: END-OF-LIFE CARE AND CLINICAL CHARACTERISTICS OF NONSURVIVOR CANCER PATIENTS WITH COVID-19. Crit Care Med. 2022;50: 259–259. doi:10.1097/01.ccm.0000808468.43737.d4	No	Not meeting inclusion criteria	Title and abstract screening
4022	Reddy V, Wurtz M, Patel SH, McCarthy M, Raval AP. Oral contraceptives and stroke: Foes or friends. Front Neuroendocr. 2022;67: 101016. doi:10.1016/j.yfrne.2022.101016	No	Not meeting inclusion criteria	Title and abstract screening
4023	Redfield R.R., Hahn S.M., Sharpless N.E. Redoubling efforts to help americans quit smoking - Federal initiatives to tackle the country's longest-running epidemic. N Engl J Med. 2020;383: 1606–1609. doi:10.1056/NEJMp2003255	No	Not meeting inclusion criteria	Title and abstract screening
4024	Redmond BY, Salwa A, Bricker JB, Buckner JD, Garey L, Zvolensky MJ. Personalized feedback intervention for individuals with low distress tolerance who smoke cigarettes: A randomized controlled trial of a digital intervention. J Subst Use Addict Treat. 2023;155: 209163. doi:10.1016/j.josat.2023.209163	No	Not meeting inclusion criteria	Title and abstract screening
4025	Ree AH, Mælandsmo GM, Flatmark K, Russnes HG, Gómez Castañeda M, Aas E. Cost-effectiveness of molecularly matched off-label therapies for end-stage cancer – the MetAction precision medicine study. Acta Oncol. 2022;61: 955–962. doi:10.1080/0284186X.2022.2098053	No	Not meeting inclusion criteria	Title and abstract screening
4026	Reed T., Gordon D., Dyal B.W., Powell-Roach K., Ezenwa M.O., Johnson-Mallard V., et al. Demographic factors and rural residence history associated with smoking history. Cancer Epidemiol Biomarkers Prev. 2022;31. doi:10.1158/1538-7755.DISP21-PO-012	No	Not meeting inclusion criteria	Title and abstract screening
4027	Reeve GS, Rostami MR, Reich RF, Behrman DA, Leopold PL, Crystal RG, et al. Oral epithelium response of electronic cigarette users to electronic cigarette. J Oral Pathol Med. 2023;52: 431–439. doi:10.1111/jop.13380	No	Not meeting inclusion criteria	Title and abstract screening
4028	Regner MF, Tregellas J, Kluger B, Wylie K, Gowin JL, Tanabe J. The insula in nicotine use disorder: Functional neuroimaging and implications for neuromodulation. Neurosci Biobehav Rev. 2019;103: 414–424. doi:10.1016/j.neubiorev.2019.06.002	No	Not meeting inclusion criteria	Title and abstract screening
4029	Rehni AK, Cho S, Zhang Z, Khushal P, Raval AP, Koch S, et al. Red Cell Microparticles Suppress Hematoma Growth Following Intracerebral Hemorrhage in Chronic Nicotine-Exposed Rats. Int J Mol Sci. 2022;23. doi:10.3390/ijms232315167	No	Not meeting inclusion criteria	Title and abstract screening
4030	Reid JL, Burkhalter R, Kasza K, Seo YS, East K, Hyland A, et al. Minimum legal age laws and perceived access to cigarettes, e-cigarettes, and other substances among youth in Canada, England, and the United States: 2017-2021. Int J Drug Policy. 2023;115: 104003. doi:10.1016/j.drugpo.2023.104003	No	Not meeting inclusion criteria	Title and abstract screening
4031	Reidel B, Abdelwahab S, Wrennall JA, Clapp PW, Beers JL, Jackson KD, et al. Vaping additives cannabinoid oil and vitamin E acetate adhere to and damage the human airway epithelium. J Appl Toxicol. 2023;43: 680–693. doi:10.1002/jat.4415	No	Not meeting inclusion criteria	Title and abstract screening
4032	Reidel B., Abdelwahab S.H., Wrennall J.A., Murray J.L., Jackson K.D., Tarran R., et al. Cannabinoid Oil and Vitamin e Acetate Containing Vapors Accumulate on the Human Airway Epithelium and Cause Severe Cellular Damage. Am J Respir Crit Care Med. 2021;203. doi:10.1164/ajrccm-conference.2021.TP109	No	Not meeting inclusion criteria	Title and abstract screening
4033	Reigle J, Secic D, Biesiada J, Wetzel C, Shamsaei B, Chu J, et al. Tobacco smoking induces metabolic reprogramming of renal cell carcinoma. J Clin Invest. 2021;131. doi:10.1172/JCI140522	No	Not meeting inclusion criteria	Title and abstract screening
4034	Reinhold B, Fischbein R, Bhamidipalli SS, Bryant J, Kenne DR. Associations of attitudes towards electronic cigarettes with advertisement exposure and social determinants: a cross sectional study. Tob Induc Dis. 2017;15: 13. doi:10.1186/s12971-017-0118-y	No	Not meeting inclusion criteria	Title and abstract screening
4035	Ren M., Lotfipour S. Nicotine gateway effects on adolescent substance use. West J Emerg Med. 2019;20: 696–709. doi:10.5811/westjem.2019.7.41661	No	Not meeting inclusion criteria	Title and abstract screening
4036	Ren M., Lotfipour S., Leslie F. Unique effects of nicotine across the lifespan. Pharmacol Biochem Behav. 2022;214: 173343. doi:10.1016/j.pbb.2022.173343	No	Not meeting inclusion criteria	Title and abstract screening
4037	Ren X, Lin L, Sun Q, Li T, Sun M, Sun Z, et al. Metabolomics-based safety evaluation of acute exposure to electronic cigarettes in mice. Sci Total Env. 2022;839: 156392. doi:10.1016/j.scitotenv.2022.156392	No	Not meeting inclusion criteria	Title and abstract screening
4038	Renda B, Andrade AK, Frie JA, Sgarbossa CL, Murray JE, Khokhar JY. High-dose adolescent nicotine exposure permits spontaneous nicotine self-administration in adult male rats. Drug Alcohol Depend. 2020;215: 108215. doi:10.1016/j.drugalcdep.2020.108215	No	Not meeting inclusion criteria	Title and abstract screening
4039	Renda B, Andrade AK, Stone APS, El Azali R, Sharivker M, Khokhar JY, et al. Adolescent nicotine and footshock exposure augments adult nicotine self-administration and drug-seeking without affecting baseline anxiety-like behaviour or stress responsivity in male rats. Psychopharmacol Berl. 2021;238: 1687–1701. doi:10.1007/s00213-021-05803-0	No	Not meeting inclusion criteria	Title and abstract screening
4040	Rengo J., Savage P., Gaalema D., Ades P. Challenges associated with delivering smoking cessation counseling in cardiac rehabilitation. J Cardiopulm Rehabil Prev. 2021;41: E21. doi:10.1097/HCR.0000000000000653	No	Not meeting inclusion criteria	Title and abstract screening
4041	Rensch J, Liu J, Wang J, Vansickel A, Edmiston J, Sarkar M. Nicotine pharmacokinetics and subjective response among adult smokers using different flavors of on!® nicotine pouches compared to combustible cigarettes. Psychopharmacol Berl. 2021;238: 3325–3334. doi:10.1007/s00213-021-05948-y	No	Not meeting inclusion criteria	Title and abstract screening

4042	Reyes-Guzman CM, Patel M, Wang TW, Corcy N, Chomenko D, Slotman B, et al. Disparities in Smokefree and Vapefree Home Rules and Smokefree Policy Attitudes Based on Housing Type and Cigarette Smoking Status, United States, 2019. Int J Env Res Public Health. 2023;20. doi:10.3390/ijerph20146356	No	Not meeting inclusion criteria	Title and abstract screening
4043	Reyfman P.A., Sugar E., Hazucha H., Hixon J., Reynolds C., Bose S., et al. Study protocol for a national cohort of adults focused on respiratory health: The American Lung Association Lung Health Cohort (ALA-LHC) Study. BMJ Open. 2021;11: e053342. doi:10.1136/bmjopen-2021-053342	No	Not meeting inclusion criteria	Title and abstract screening
4044	Reynales-Shigematsu LM, Barnoya J, Cavalcante T, Aburto TC, Romieu I, Stern MC, et al. Latin America and the Caribbean Code Against Cancer 1st edition: Tobacco and nicotine-related products, secondhand smoke, and alcohol and cancer. Cancer Epidemiol. 2023;86 Suppl 1: 102413. doi:10.1016/j.canep.2023.102413	No	Not meeting inclusion criteria	Title and abstract screening
4045	Reynolds C, Staples H. Hemoptysis in an adolescent with EVALI. Pediatr Pulmonol. 2022;57: 2875–2876. doi:10.1002/ppul.26090	No	Not meeting inclusion criteria	Title and abstract screening
4046	Reynolds CME, Hanafin J, Sunday S, McAvoy H, Clancy L. Odds of gambling among adolescents: a secondary analysis of the cross-sectional European School Survey Project on Alcohol and Other Drugs. Lancet. 2023;402 Suppl 1: S79. doi:10.1016/S0140-6736(23)02118-9	No	Not meeting inclusion criteria	Title and abstract screening
4047	Reynolds LM, Zamora C, Lee UJ, Stokes AC, Benjamin EJ, Bhatnagar A, et al. Tobacco Use Prevalence and Transitions From 2013 to 2018 Among Adults With a History of Cardiovascular Disease. J Am Heart Assoc. 2021;10: e021118. doi:10.1161/JAHA.121.021118	No	Not meeting inclusion criteria	Title and abstract screening
4048	Reynolds RM, Popova L, Ashley DL, Henderson KC, Ntansah CA, Yang B, et al. Messaging about very low nicotine cigarettes (VLNCs) to influence policy attitudes, harm perceptions and smoking motivations: a discrete choice experiment. Tob Control. 2022. doi:10.1136/tc-2022-057577	No	Not meeting inclusion criteria	Title and abstract screening
4049	Rezk-Hanna M, Warda US, Stokes AC, Fetterman J, Li J, Macey PM, et al. Associations of Smokeless Tobacco Use With Cardiovascular Disease Risk: Insights From the Population Assessment of Tobacco and Health Study. Nicotine Tob Res. 2022;24: 1063–1070. doi:10.1093/ntr/ntab258	No	Not meeting inclusion criteria	Title and abstract screening
4050	Rezk-Hanna M, Gupta R, Nettle C, Dobrin D, Cheng C, Means A, et al. Differential Effects of Electronic Hookah Vaping and Traditional Combustible Hookah Smoking on Oxidation, Inflammation, and Arterial Stiffness. 2022;161: 208-218. doi:10.1016/j.chest.2021.07.027	No	Not meeting inclusion criteria	Title and abstract screening
4051	Rezk-Hanna M., Cheng C.W., Adolfo A., Brecht M.L. Electronic hookah vaping reduces plasma concentrations of nitrate/nitrite and increases systemic oxidative stress. Eur Respir J. 2022;60: 1956. doi:10.1093/eurheartj/ehac544.1956	No	Not meeting inclusion criteria	Title and abstract screening
4052	Rezk-Hanna M., Gupta R., Nettle C.O., Dobrin D., Cheng C.-W., Means A., et al. Differential Effects of Electronic Hookah Vaping and Traditional Combustible Hookah Smoking on Oxidation, Inflammation and Arterial Stiffness. Chest. 2021. doi:10.1016/j.chest.2021.07.027	No	Not meeting inclusion criteria	Title and abstract screening
4053	Rezk-Hanna M, Seals DR, Rossman MJ, Gupta R, Nettle CO, Means A, et al. Ascorbic Acid Prevents Vascular Endothelial Dysfunction Induced by Electronic Hookah (Waterpipe) Vaping. J Am Heart Assoc. 2021;10: e019271. doi:10.1161/JAHA.120.019271	No	Not meeting inclusion criteria	Title and abstract screening
4054	Rhoads S, Auth R, Chambers A, Blundin M, Mahoney L, McLaughlin S, et al. Perceptions and Use of E-cigarettes among Young Adults with Cystic Fibrosis: An Observational Study. R Med J 2013. 2023;106: 58–63.	No	Not meeting inclusion criteria	Title and abstract screening
4055	Ribisl KM, Hatsukami DK, Huang J, Williams RS, Donny EC. Strategies to Reduce Illicit Trade of Regular Nicotine Tobacco Products After Introduction of a Low-Nicotine Tobacco Product Standard. Am J Public Health. 2019;109: 1007–1014. doi:10.2105/AJPH.2019.305067	No	Not meeting inclusion criteria	Title and abstract screening
4056	Rice M, Nollen NL, Ahluwalia JS, Benowitz N, Woodcock A, Pulvers K. Effects of Marijuana Use on Smokers Switching to E-Cigarettes in a Randomized Clinical Trial. Nicotine Tob Res. 2022;24: 994–1002. doi:10.1093/ntr/ntac008	No	Not meeting inclusion criteria	Title and abstract screening
4057	Richter L, Pugh BS, Smith PH, Ball SA. The co-occurrence of nicotine and other substance use and addiction among youth and adults in the United States: implications for research, practice, and policy. Am J Drug Alcohol Abuse. 2017;43: 132–145. doi:10.1080/00952990.2016.1193511	No	Not meeting inclusion criteria	Title and abstract screening
4058	Richters A, Yildirim H, Booth CM, Vera Badillo FE, Kiemeney LALM, Aben KKH. Changes to Primary End Points in Randomized Clinical Trials on Immune Checkpoint Inhibitors in Urothelial, Renal Cell, and Lung Cancers: A Systematic Review. JAMA Oncol. 2023;9: 1144–1147. doi:10.1001/jamaoncol.2023.1374	No	Not meeting inclusion criteria	Title and abstract screening
4059	Riggle J., Pitts L.R., Postigo M. Bilateral endobronchial valves: An option for severe bilateral persistent air leak. Am J Respir Crit Care Med. 2021;203. doi:10.1164/ajrccm-conference.2021.203.1_MeetingAbstracts.A2193	No	Not meeting inclusion criteria	Title and abstract screening
4060	Rigotti N.A. Monitoring the rapidly changing landscape of e-cigarettes. Ann Intern Med. 2018;169: 494–495. doi:10.7326/M18-2176	No	Not meeting inclusion criteria	Title and abstract screening
4061	Rigotti N.A., Chang Y., Davis E.M., Regan S., Levy D.E., Ylioja T., et al. Comparative Effectiveness of Postdischarge Smoking Cessation Interventions for Hospital Patients: The Helping HAND 4 Randomized Clinical Trial. JAMA Intern Med. 2022;182: 814–824. doi:10.1001/jamainternmed.2022.2300	No	Not meeting inclusion criteria	Title and abstract screening
4062	Rigotti N.A., Chang Y., Tindle H.A., Kalkhoran S.M., Levy D.E., Regan S., et al. Association of E-cigarette use with smoking cessation among smokers who plan to quit after a hospitalization a prospective study. Ann Intern Med. 2018;168: 613–620. doi:10.7326/M17-2048	No	Not meeting inclusion criteria	Title and abstract screening
4063	Rigotti NA. Randomized Trials of e-Cigarettes for Smoking Cessation. JAMA. 2020;324: 1835–1837. doi:10.1001/jama.2020.18967	No	Not meeting inclusion criteria	Title and abstract screening
4064	Rigotti NA, Chang Y, Regan S, Lee S, Kelley JHK, Davis E, et al. Cigarette Smoking and Risk Perceptions During the COVID-19 Pandemic Reported by Recently Hospitalized Participants in a Smoking Cessation Trial. J Gen Intern Med. 2021;36: 3786–3793. doi:10.1007/s11606-021-06913-3	No	Not meeting inclusion criteria	Title and abstract screening
4065	Rigotti NA, Kruse GR, Livingstone-Banks J, Hartmann-Boyce J. Treatment of Tobacco Smoking: A Review. JAMA. 2022;327: 566–577. doi:10.1001/jama.2022.0395	No	Not meeting inclusion criteria	Title and abstract screening
4066	Rinaldi S, Pieper E, Schulz T, Zimmermann R, Luch A, Laux P, et al. Oral nicotine pouches with an aftertaste? Part 2: in vitro toxicity in human gingival fibroblasts. Arch Toxicol. 2023;97: 2343–2356. doi:10.1007/s00204-023-03554-9	No	Not meeting inclusion criteria	Title and abstract screening
4067	Risi S. On the Origins of the Electronic Cigarette: British American Tobacco's Project Ariel (1962-1967). Am J Public Health. 2017;107: 1060–1067. doi:10.2105/AJPH.2017.303806	No	Not meeting inclusion criteria	Title and abstract screening
4068	Rivard C, Brown A, Kasza K, Bansal-Travers M, Hyland A. Home Tobacco Use Policies and Exposure to Secondhand Tobacco Smoke: Findings from Waves 1 through 4 of the Population Assessment of Tobacco and Health (PATH) Study. Int J Env Res Public Health. 2021;18. doi:10.3390/ijerph18189719	No	Not meeting inclusion criteria	Title and abstract screening
4069	Rizzo A, Mollica V, Massari F. Randomized Clinical Trials in the Era of Precision Oncology—The Role of End Points, Industry Funding, and Medical Writing Integrity...Del Paggio JC, Berry JS, Hopman WM, et al. Evolution of the randomized clinical trial in the era of precision oncology. J. JAMA Oncol. 2021;7: 1577–1577. doi:10.1001/jamaoncol.2021.3335	No	Not meeting inclusion criteria	Title and abstract screening
4070	Robausch M, Grössmann N, Wild C. Cancer care near the end-of-life in Austria: A retrospective data analysis. Eur J Cancer Care (Engl). 2021;30: 1–7. doi:10.1111/ecc.13423	No	Not meeting inclusion criteria	Title and abstract screening

4071	Roberts E, Copeland C, Robson D, McNeill A. Drug-related deaths associated with vaping product use in the United Kingdom. Addiction. 2021;116: 2908–2911. doi:10.1111/add.15468	No	Not meeting inclusion criteria	Title and abstract screening
4072	Roberts E, Taylor E, Cox S, Brose L, McNeill A, Robson D. Pattern and prevalence of vaping nicotine and non-nicotine drugs in the United Kingdom: a cross-sectional study. BMJ Open. 2023;13: e066826. doi:10.1136/bmjopen-2022-066826	No	Not meeting inclusion criteria	Title and abstract screening
4073	Roberts M.E., Keller-Hamilton B., Hinton A., Browning C.R., Slater M.D., Xi W., et al. The magnitude and impact of tobacco marketing exposure in adolescents' day-to-day lives: An ecological momentary assessment (EMA) study. Addict Behav. 2019;88: 144–149. doi:10.1016/j.addbeh.2018.08.035	No	Not meeting inclusion criteria	Title and abstract screening
4074	Roberts ME, Banse R, Ebbeler C, Ferketich AK. Spousal concordance in the use of alternative tobacco products: A multi-country investigation. Drug Alcohol Depend. 2017;171: 16–19. doi:10.1016/j.drugalcdep.2016.11.017	No	Not meeting inclusion criteria	Title and abstract screening
4075	Roberts ME, Keller-Hamilton B, Ferketich AK, Berman ML. Juul and the upsurge of e-cigarette use among college undergraduates. J Am Coll Health. 2022;70: 9–12. doi:10.1080/07448481.2020.1726355	No	Not meeting inclusion criteria	Title and abstract screening
4076	Roberts ME, Tackett AP, Singer JM, Wagner DD, Lu B, Wagener TL, et al. Dual Use of E-Cigarettes and Cannabis Among Young People in America: A New Public Health Hurdle? J Stud Alcohol Drugs. 2022;83: 768–770. doi:10.15288/jsad.2022.83.768	No	Not meeting inclusion criteria	Title and abstract screening
4077	Roberts ME, Keller-Hamilton B, Ferketich AK, Berman ML. Juul and the upsurge of e-cigarette use among college undergraduates. J Am Coll Health. 2020; No-Specified. doi:10.1080/07448481.2020.1726355https://dx.doi.org/10.1080/07448481.2020.1726355	No	Not meeting inclusion criteria	Title and abstract screening
4078	Roberts W, Moore KE, Peltier MR, Verplaetse TL, Oberleitner L, Hacker R, et al. Electronic Cigarette Use and Risk of Harmful Alcohol Consumption in the U.S. Population. Alcohol Clin Exp Res. 2018;42: 2385–2393. doi:10.1111/acer.13889	No	Not meeting inclusion criteria	Title and abstract screening
4079	Roberts W, Ralevski E, Verplaetse TL, McKee SA, Petrakis IL. Tobacco use during a clinical trial of mecamylamine for alcohol dependence: Medication effects on smoking and associations with reductions in drinking. J Subst Abuse Treat. 2018;94: 91–96. doi:10.1016/j.jsat.2018.08.015	No	Not meeting inclusion criteria	Title and abstract screening
4080	Roberts W., McKee S.A. Nicotine metabolism speed predicts hazardous drinking in tobacco users: Findings from the population assessment of tobacco and health. Alcohol Clin Exp Res. 2021;45: 140A. doi:10.1111/acer.14628	No	Not meeting inclusion criteria	Title and abstract screening
4081	Robertson C, Watanabe SM, Sinnarajah A, Potapov A, Faily V, Tarumi Y, et al. Association between Consultation by a Comprehensive Integrated Palliative Care Program and Quality of End-of-Life Care in Patients with Advanced Cancer in Edmonton, Canada. Curr Oncol. 2023;30: 897–907. doi:10.3390/curroncol30010068	No	Not meeting inclusion criteria	Title and abstract screening
4082	Robinson J. E-cigarettes as effective as nicotine replacementtherapy for smoking cessation, says draft guidance. Pharm J. 2021;306. doi:10.1211/PJ.2021.1.92931	No	Not meeting inclusion criteria	Title and abstract screening
4083	Robinson JD, Kypriotakis G, Karam-Hage M, Cui Y, Beneventi D, Blalock JA, et al. Brief report: Characterization of electronic cigarette use among patients of a comprehensive cancer center. Am J Addict. 2023. doi:10.1111/ajad.13486	No	Not meeting inclusion criteria	Title and abstract screening
4084	Robinson PD, Jayasuriya G, Haggie S, Uluer AZ, Gaffin JM, Fleming L. Issues affecting young people with asthma through the transition period to adult care. Paediatr Respir Rev. 2022;41: 30–39. doi:10.1016/j.prrv.2021.09.005	No	Not meeting inclusion criteria	Title and abstract screening
4085	Robinson RJ, Eddingsaas NC, DiFrancesco AG, Jayasekera S, Hensel EC Jr. A framework to investigate the impact of topography and product characteristics on electronic cigarette emissions. PLoS One. 2018;13: e0206341. doi:10.1371/journal.pone.0206341	No	Not meeting inclusion criteria	Title and abstract screening
4086	Robinson TJ, Wilson LE, Marcom PK, Troester M, Lynch CF, Hernandez BY, et al. Analysis of Sociodemographic, Clinical, and Genomic Factors Associated With Breast Cancer Mortality in the Linked Surveillance, Epidemiology, and End Results and Medicare Database. JAMA Netw Open. 2021;4: e2131020–e2131020. doi:10.1001/jamanetworkopen.2021.31020	No	Not meeting inclusion criteria	Title and abstract screening
4087	Roche DJO, Bujarski S, Green R, Hartwell EE, Leventhal AM, Ray LA. Alcohol, tobacco, and marijuana consumption is associated with increased odds of same-day substance co- and tri-use. Drug Alcohol Depend. 2019;200: 40–49. doi:10.1016/j.drugalcdep.2019.02.035	No	Not meeting inclusion criteria	Title and abstract screening
4088	Roditis ML, Dineva A, Smith A, Walker M, Delahanty J, D'lorio E, et al. Reactions to electronic nicotine delivery system (ENDS) prevention messages: results from qualitative research used to inform FDA's first youth ENDS prevention campaign. Tob Control. 2020;29: 510–515. doi:10.1136/tobaccocontrol-2019-055104	No	Not meeting inclusion criteria	Title and abstract screening
4089	Rodrigo G, Jaccard G, Tafin Djoko D, Korneliou A, Esposito M, Belushkin M. Cancer potencies and margin of exposure used for comparative risk assessment of heated tobacco products and electronic cigarettes aerosols with cigarette smoke. Arch Toxicol. 2021;95: 283–298. doi:10.1007/s00204-020-02924-x	No	Not meeting inclusion criteria	Title and abstract screening
4090	Rodriguez GM, Kapphahn K, Patel MI. Disparities in intensity of care at the end-of-life for patients with gastrointestinal cancer. J Clin Oncol. 41: 6558–6558. doi:10.1200/JCO.2023.41.16_suppl.6558	No	Not meeting inclusion criteria	Title and abstract screening
4091	Rodríguez-Bolaños R, Arillo-Santillán E, Guzmán-Rodríguez C, Barrientos-Gutiérrez I, Gallegos-Carrillo K, Titus A, et al. Similarities and Differences in Substance Use Patterns Among Lesbian, Gay, Bisexual, and Heterosexual Mexican Adult Smokers. LGBT Health. 2021;8: 545–553. doi:10.1089/lgbt.2020.0457	No	Not meeting inclusion criteria	Title and abstract screening
4092	Rodriguez-Vega A., Dutra-Tavares A.C., Souza T.P., Semeao K.A., Filgueiras C.C., Ribeiro-Carvalho A., et al. Nicotine Exposure in a Phencyclidine-Induced Mice Model of Schizophrenia: Sex-Selective Medial Prefrontal Cortex Protein Markers of the Combined Insults in Adolescent Mice. Int J Mol Sci. 2023;24: 14634. doi:10.3390/ijms241914634	No	Not meeting inclusion criteria	Title and abstract screening
4093	Rodu B, Plurphanswat N. Cross-sectional e-cigarette studies are unreliable without timing of exposure and disease diagnosis. Intern Emerg Med. 2023;18: 319–323. doi:10.1007/s11739-022-03141-3	No	Not meeting inclusion criteria	Title and abstract screening
4094	Rogers SW, Myers EJ, Gahring LC. Age-Associated Tooth Loss and Oral Microbial Dysbiosis in a Mouse Genetic Model of Chronic Nicotine Exposure. Front Immunol. 2020;11: 575200. doi:10.3389/fimmu.2020.575200	No	Not meeting inclusion criteria	Title and abstract screening
4095	Rohde JA, Noar SM, Horvitz C, Lazard AJ, Cornacchione Ross J, Sutfin EL. The Role of Knowledge and Risk Beliefs in Adolescent E-Cigarette Use: A Pilot Study. Int J Env Res Public Health. 2018;15. doi:10.3390/ijerph15040830	No	Not meeting inclusion criteria	Title and abstract screening
4096	Rohde JA, Noar SM, Prentice-Dunn H, Kresovich A, Hall MG. Comparison of Message and Effects Perceptions for The Real Cost E-Cigarette Prevention Ads. Health Commun. 2021;36: 1222–1230. doi:10.1080/10410236.2020.1749353	No	Not meeting inclusion criteria	Title and abstract screening
4097	Rohde JA, Noar SM, Sheldon JM, Hall MG, Kieu T, Brewer NT. Identifying Promising Themes for Adolescent Vaping Warnings: A National Experiment. Nicotine Tob Res. 2022;24: 1379–1385. doi:10.1093/ntr/ntac093	No	Not meeting inclusion criteria	Title and abstract screening
4098	Rohde JA, Noar SM, Mendel JR, Hall MG, Baig SA, Ribisl KM, et al. E-Cigarette Health Harm Awareness and Discouragement: Implications for Health Communication. Nicotine Tob Res Off J Soc Res Nicotine Tob. 2020;22: 1131–1138. doi:10.1093/ntr/ntz194	No	Not meeting inclusion criteria	Title and abstract screening
4099	Rohsenow DJ, Martin RA, Tidey JW, Colby SM, Monti PM. Treating Smokers in Substance Treatment With Contingent Vouchers, Nicotine Replacement and Brief Advice Adapted for Sobriety Settings. J Subst Abuse Treat. 2017;72: 72–79. doi:10.1016/j.jsat.2016.08.012	No	Not meeting inclusion criteria	Title and abstract screening

4100	Rohsenow DJ, Tidey JW, Martin RA, Colby SM, Eissenberg T. Effects of six weeks of electronic cigarette use on smoking rate, CO, cigarette dependence, and motivation to quit smoking: A pilot study. Addict Behav. 2018;80: 65–70. doi:10.1016/j.addbeh.2018.01.012	No	Not meeting inclusion criteria	Title and abstract screening
4101	Romano I, Costello MJ, Ropp C, Li Y, Sousa S, Bruce D, et al. Evaluating the short-term impact of a tobacco-free policy in an inpatient addiction treatment setting. J Subst Abuse Treat. 2019;107: 50–59. doi:10.1016/j.jsat.2019.09.007	No	Not meeting inclusion criteria	Title and abstract screening
4102	Romano I, Patte KA, de Groh M, Jiang Y, Leatherdale ST. Perceptions of and adherence to early COVID-19-related restrictions and associations with substance use among youth in Canada. Health Promot Chronic Prev Can. 2022;42: 479–489. doi:10.24095/hpcdp.42.11/12.03	No	Not meeting inclusion criteria	Title and abstract screening
4103	Romm KF, Cohn AM, Wang Y, Williams R, Berg CJ. Disparities in trajectories of cigarette and E-cigarette use across sexual orientation groups of young adult men and women in the US. Addict Behav. 2023;145: 107763. doi:10.1016/j.addbeh.2023.107763	No	Not meeting inclusion criteria	Title and abstract screening
4104	Romm KF, Cohn AM, Wang Y, Berg CJ. Psychosocial predictors of trajectories of dual cigarette and e-cigarette use among young adults in the US. Addict Behav. 2023;141: 107658. doi:10.1016/j.addbeh.2023.107658	No	Not meeting inclusion criteria	Title and abstract screening
4105	Romm KF, Dopke C, Price OA, Pannell A, Williams R, Berg CJ. Parental influences on tobacco use and likelihood of future use among sexual minority young adult men and women in the United States. Am J Addict. 2023;32: 450–459. doi:10.1111/ajad.13418	No	Not meeting inclusion criteria	Title and abstract screening
4106	Romm KF, Henriksen L, Huang J, Le D, Clausen M, Duan Z, et al. Impact of existing and potential e-cigarette flavor restrictions on e-cigarette use among young adult e-cigarette users in 6 US metropolitan areas. Prev Med Rep. 2022;28: 101901. doi:10.1016/j.pmedr.2022.101901	No	Not meeting inclusion criteria	Title and abstract screening
4107	Romm KF, Huebner DM, Pratt-Chapman ML, Rodriguez-Diaz CE, Wang Y, Ma Y, et al. Disparities in traditional and alternative tobacco product use across sexual orientation groups of young adult men and women in the US. Subst Abus. 2022;43: 815–824. doi:10.1080/08897077.2021.2010259	No	Not meeting inclusion criteria	Title and abstract screening
4108	Romm KF, Patterson B, Arem H, Price OA, Wang Y, Berg CJ. Cross-Sectional Retrospective Assessments versus Longitudinal Prospective Assessments of Substance Use Change among Young Adults during COVID-19: Magnitude and Correlates of Discordant Findings. Subst Use Misuse. 2022;57: 484–489. doi:10.1080/10826084.2021.2012696	No	Not meeting inclusion criteria	Title and abstract screening
4109	Romm KF, Patterson B, Crawford ND, Posner H, West CD, Wedding D, et al. Changes in young adult substance use during COVID-19 as a function of ACEs, depression, prior substance use and resilience. Subst Abus. 2022;43: 212–221. doi:10.1080/08897077.2021.1930629	No	Not meeting inclusion criteria	Title and abstract screening
4110	Romm KF, Patterson B, Wang Y, Wysota CN, Bar-Zeev Y, Levine H, et al. Drivers and Passengers in Vehicles Driven Under the Influence of Alcohol or Marijuana: Behavior Profiles and Risk Factors Among Young Adults in a Longitudinal Study. J Stud Alcohol Drugs. 2022;83: 342–351. doi:10.15288/jsad.2022.83.342	No	Not meeting inclusion criteria	Title and abstract screening
4111	Romm KF, Turiano NA, Milstred AR, Bray BC, Dino G, Doogan N, et al. Socioecological Predictors of Change in Adolescent Tobacco Use Across Waves 1-4 of the Population Assessment of Tobacco and Health Study. J Adolesc Health. 2022. doi:10.1016/j.jadohealth.2022.09.026	No	Not meeting inclusion criteria	Title and abstract screening
4112	Romm KF, Turiano NA, Milstred AR, Bray BC, Dino G, Doogan N, et al. Socioecological Predictors of Change in Adolescent Tobacco Use Across Waves 1-4 of the Population Assessment of Tobacco and Health Study. J Adolesc Health. 2023;72: 375–382. doi:10.1016/j.jadohealth.2022.09.026	No	Not meeting inclusion criteria	Title and abstract screening
4113	Romm KF, Wang Y, Duan Z, Bennett B, Fuss C, Ma Y, et al. Psychosocial predictors of longitudinal changes in tobacco and cannabis use among young adults. Addict Behav. 2022;129: 107264. doi:10.1016/j.addbeh.2022.107264	No	Not meeting inclusion criteria	Title and abstract screening
4114	Romm KF, Wang Y, Ma Y, Wysota CN, Blank MD, Huebner DM, et al. The reciprocal relationships of social norms and risk perceptions to cigarette, e-cigarette, and cannabis use: Cross-lagged panel analyses among US young adults in a longitudinal study. Drug Alcohol Depend. 2022;238: 109570. doi:10.1016/j.drugalcdep.2022.109570	No	Not meeting inclusion criteria	Title and abstract screening
4115	Romm KF, Wang Y, Schleicher NC, Pannel A, Williams R, Berg CJ, et al. Rates of Age Verification for Cigarette and E-cigarette Purchases as a Function of State T21 Laws Before and After Implementation of the Federal T21 Law in the United States. Nicotine Tob Res. 2023;25: 1386–1390. doi:10.1093/ntr/ntad044	No	Not meeting inclusion criteria	Title and abstract screening
4116	Ronchetti J., Terriau A. Help me quit smoking but don't make me sick! The controversial effects of electronic cigarettes on tobacco smokers. Soc Sci Med. 2021;274: 113770. doi:10.1016/j.socscimed.2021.113770	No	Not meeting inclusion criteria	Title and abstract screening
4117	Rosa WE, McDarby M, Buller H, Ferrell BR. Palliative Care Clinician Perspectives on Person-Centered End-of-Life Communication for Racially and Culturally Minoritized Persons with Cancer. Cancers. 2023;15: 4076. doi:10.3390/cancers15164076	No	Not meeting inclusion criteria	Title and abstract screening
4118	Rosario MH, Johnson DL, Manglallan KS, Phillips KT, Pokhrel P, Okamoto SK. Tobacco use among native Hawaiian and Pacific Islander youth in the U.S. and USAPI: a systematic review of the literature. J Ethn Subst Abuse. 2021; 1–16. doi:10.1080/15332640.2021.1965934	No	Not meeting inclusion criteria	Title and abstract screening
4119	Rosario MH, Johnson DL, Manglallan KS, Phillips KT, Pokhrel P, Okamoto SK. Tobacco use among native Hawaiian and Pacific Islander youth in the U.S. and USAPI: a systematic review of the literature. J Ethn Subst Abuse. 2023;22: 500–515. doi:10.1080/15332640.2021.1965934	No	Not meeting inclusion criteria	Title and abstract screening
4120	Rose JE, Behm FM, Botts TL, Botts DR, Willette PN, Vocci F, et al. Novel rapid-acting sublingual nicotine tablet as a cigarette substitution strategy. Psychopharmacol Berl. 2022;239: 2853–2862. doi:10.1007/s00213-022-06171-z	No	Not meeting inclusion criteria	Title and abstract screening
4121	Rose JE, Frisbee S, Campbell D, Salley A, Claerhout S, Davis JM. Smoking reduction using electronic nicotine delivery systems in combination with nicotine skin patches. Psychopharmacol Berl. 2023;240: 1901–1909. doi:10.1007/s00213-023-06401-y	No	Not meeting inclusion criteria	Title and abstract screening
4122	Rose JJ, Rebuli ME, Noël A, Croft DP. Clearing Some of the Haze around E-cigarette or Vaping Product Use-Associated Lung Injury (EVALI). Ann Am Thorac Soc. 2022;19: 1805–1807. doi:10.1513/AnnalsATS.202208-685ED	No	Not meeting inclusion criteria	Title and abstract screening
4123	Rose SW, Anesetti-Rothermel A, Westneat S, van de Venne J, Folger S, Rahman B, et al. Inequitable Distribution of FTP Marketing by Neighborhood Characteristics: Further Evidence for Targeted Marketing. Nicotine Tob Res. 2022;24: 484–492. doi:10.1093/ntr/ntab222	No	Not meeting inclusion criteria	Title and abstract screening
4124	Rosen MR, Lambert KA, Nakatani MM, Ibrahim S, Troung T, Feng K, et al. Quality for all: Clinical trial enrollment and end-of-life care in solid and hematologic malignancies. J Clin Oncol. 41: 12028–12028. doi:10.1200/JCO.2023.41.16_suppl.12028	No	Not meeting inclusion criteria	Title and abstract screening
4125	Rosen RL, Ramasubramani RS, Benowitz NL, Gandhi KK, Williams JM. Caffeine levels and dietary intake in smokers with schizophrenia and bipolar disorder. Psychiatry Res. 2023;319: 114989. doi:10.1016/j.psychres.2022.114989	No	Not meeting inclusion criteria	Title and abstract screening
4126	Rosen RL, Steinberg ML. Factors associated with past-year attempts to quit e-cigarettes among current users: Findings from the Population Assessment of Tobacco and Health Wave 4 (2017-2018). Drug Alcohol Depend. 2021;227: 108973. doi:10.1016/j.drugalcdep.2021.108973	No	Not meeting inclusion criteria	Title and abstract screening
4127	Rosenthal H, Chow N, Mehta S, Pham D, Milanaik R. Puff bars: a dangerous trend in adolescent disposable e-cigarette use. Curr Opin Pediatr. 2022;34: 288–294. doi:10.1097/MOP.0000000000001123	No	Not meeting inclusion criteria	Title and abstract screening
4128	Rosenthal SR, Wensley IA, Noel JK. Eliminating Disparities in Young Adult Tobacco Use: The Need for Integrated Behavioral Healthcare. R Med J 2013. 2022;105: 38–42.	No	Not meeting inclusion criteria	Title and abstract screening

4129	Rosewich M. Effects of active and passive smoking on the health of children and adolescents. Pneumologe. 2020;17: 51–56. doi:10.1007/s10405-019-00298-1	No	Not meeting inclusion criteria	Title and abstract screening
4130	Ross CS, Pae V, Henehan ER, Zhang TC, Siegel MB. 154. Vaping Flavors Associated With Vaping Dependence Symptoms Among Adolescents: A Pilot Study Using Vape Factor Fast Find (VF3)...SAHM Annual Meeting, Psychological Well-Being: International Transcultural Perspectives, March 6-9, 2019, Washington, DC, U. J Adolesc Health. 2019;64: S79–S79. doi:10.1016/j.jadohealth.2018.10.170	No	Not meeting inclusion criteria	Title and abstract screening
4131	Ross KY. Susceptibility to e-cigarette use among U.S. middle school students. Diss Abstr Int Sect B Sci Eng. 2023;84: No-Specified.	No	Not meeting inclusion criteria	Title and abstract screening
4132	Ross L, Neergaard MA, Petersen MA, Groenvold M. The quality of end of life care for Danish cancer patients who have received specialized palliative: a national survey using the Danish version of VOICES-SF. Support Care Cancer. 2022;30: 3593–3602. doi:10.1007/s00520-021-06756-y	No	Not meeting inclusion criteria	Title and abstract screening
4133	Ross L, Neergaard MA, Petersen MA, Groenvold M. The quality of end-of-life care for Danish cancer patients who have received non-specialized palliative care: a national survey using the Danish version of VOICES-SF. Support Care Cancer. 2022;30: 9507–9516. doi:10.1007/s00520-022-07302-0	No	Not meeting inclusion criteria	Title and abstract screening
4134	Rossheim ME, LoParco CR, Walker A, Livingston MD, Trangenstein PJ, Olsson S, et al. Delta-8 THC Retail Availability, Price, and Minimum Purchase Age. Cannabis Cannabinoid Res. 2022. doi:10.1089/can.2022.0079	No	Not meeting inclusion criteria	Title and abstract screening
4135	Rotermann M, Gilmour H. Correlates of vaping among adolescents in Canada. Health Rep. 2022;33: 24–35. doi:10.25318/82-003-x202200700003-eng	No	Not meeting inclusion criteria	Title and abstract screening
4136	Rowa-Dewar N, Rooke C, Amos A. Using e-cigarettes in the home to reduce smoking and secondhand smoke: disadvantaged parents' accounts. Health Educ Res. 2017;32: 12–21. doi:10.1093/her/cyw052	No	Not meeting inclusion criteria	Title and abstract screening
4137	Rowse B, Witt M, Hoek J. The rise and rise of specialist vape stores: will proposed changes protect rangatahi? N Z Med J. 2023;136: 122–126.	No	Not meeting inclusion criteria	Title and abstract screening
4138	Rubenstein D, Pacek LR, McClernon FJ. Multiple Tobacco Product Use Conceptual Framework: A 2021 Update on Evidence. Nicotine Tob Res. 2022;24: 1208–1217. doi:10.1093/ntr/ntac032	No	Not meeting inclusion criteria	Title and abstract screening
4139	Rubenstein D, Sokolovsky AW, Aston ER, Nollen NL, Schmid CH, Rice M, et al. Predictors of smoking reduction among African American and Latinx smokers in a randomized controlled trial of JUUL e-cigarettes. Addict Behav. 2021;122: 107037. doi:10.1016/j.addbeh.2021.107037	No	Not meeting inclusion criteria	Title and abstract screening
4140	Rubenstein D, Sweitzer MM, Scroggs LB, Denlinger-Apte RL, Kozink R, McClernon FJ. Menthol preference moderates the relationship between cigarette nicotine content choice and reactions in young adults who smoke infrequently. Drug Alcohol Depend. 2022;238: 109580. doi:10.1016/j.drugalcdep.2022.109580	No	Not meeting inclusion criteria	Title and abstract screening
4141	Rubin J, Chiu ML, Mino-Kenudson M, Sharma A, Witkin AS, Moschovis PP, et al. ARDS With Pneumothorax in a Young Adult. Chest. 2022;161: e111–e116. doi:10.1016/j.chest.2021.09.006	No	Not meeting inclusion criteria	Title and abstract screening
4142	Rubinson NG, Fong GT, Gravely S, Quah ACK, Bitan M, Lev Ari S, et al. The association between risk perceptions, anxiety, and self-reported changes in tobacco and nicotine product use due to COVID-19 in May-June 2020 in Israel. BMC Public Health. 2023;23: 759. doi:10.1186/s12889-023-15351-1	No	Not meeting inclusion criteria	Title and abstract screening
4143	Ruder K. Vaping's Cardiopulmonary Associations in Adolescents Examined. JAMA. 2022;328: 608–609. doi:10.1001/jama.2022.12266	No	Not meeting inclusion criteria	Title and abstract screening
4144	Ruedisueli I., Arastoo S., Gupta P.K., Gornbein J., Middlekauff H. Neural-hematopoietic-inflammatory axis in nonsmokers, electronic cigarette users, and tobacco smokers. Physiol Rep. 2022;10: e15412. doi:10.14814/phy2.15412	No	Not meeting inclusion criteria	Title and abstract screening
4145	Ruglass LM, Root JC, Dambreville N, Shevorykin A, Haque N, Sun V, et al. Smoking policies in the home have less influence on cigarettes per day and nicotine dependence level among African American than White smokers: A cross-sectional analysis. J Natl Med Assoc. 2019;111: 606–615. doi:10.1016/j.jnma.2019.07.002	No	Not meeting inclusion criteria	Title and abstract screening
4146	Ruhl J, Adamo P, Negoita S. Cancer Registry. The National Cancer Institute's Surveillance, Epidemiology, and End Results Program: 50 Years of Turning Data Into Discovery. Rec Gt Val Publ Co Inc. 2023;35: 26–27.	No	Not meeting inclusion criteria	Title and abstract screening
4147	Ruppert A.-M., Urban T. Smoking cessation in lung cancer: Methods and results; role of e-cigarette. Rev Mal Respir Actual. 2020;12: 2S14-2S21. doi:10.1016/S1877-1203%2820%2930079-3	No	Not meeting inclusion criteria	Title and abstract screening
4148	Ruppert A.-M., Urban T. Rev Mal Respir Actual. 2021;13: 2S8-2S11. doi:10.1016/S1877-1203%2821%2900090-2	No	Not meeting inclusion criteria	Title and abstract screening
4149	Ruppert A.-M., Urban T. Rev Mal Respir Actual. 2021;13: 2S8-2S11. doi:10.1016/S1877-1203%2821%2900090-2	No	Not meeting inclusion criteria	Title and abstract screening
4150	Ruppert A.M., Urban T. Le sevrage tabagique dans le cancer bronchique: les methodes et les resultats; place de la e-cigarette. Rev Mal Respir Actual. 2019;11: 177–183. doi:10.1016/S1877-1203%2819%2930084-9	No	Not meeting inclusion criteria	Title and abstract screening
4151	Russell C., Katsampouris E., McKeganey N. Harm and Addiction Perceptions of the JUUL E-Cigarette among Adolescents. Nicotine Tob Res. 2020;22: 713–721. doi:10.1093/ntr/ntz183	No	Not meeting inclusion criteria	Title and abstract screening
4152	Russell KW, Katz MG, Phillips RC, Kelley-Quon LI, Acker SN, Shahi N, et al. Adolescent Vaping-Associated Trauma in the Western United States. J Surg Res. 2022;276: 251–255. doi:10.1016/j.jss.2022.02.026	No	Not meeting inclusion criteria	Title and abstract screening
4153	Russell L., Sahil S., Cheng A.-L., Reisz C. 25525 High-risk behaviors in teenagers: Legislative impact on indoor tanning injuries. J Am Acad Dermatol. 2021;85: AB60. doi:10.1016/j.jaad.2021.06.267	No	Not meeting inclusion criteria	Title and abstract screening
4154	Russo C, Caponnetto P, Cibella F, Maglia M, Alamo A, Campagna D, et al. A double blind randomized controlled trial investigating efficacy and safety of varenicline for smoking cessation in patients with type 2 diabetes: study protocol. Intern Emerg Med. 2021;16: 1823–1839. doi:10.1007/s11739-021-02684-1	No	Not meeting inclusion criteria	Title and abstract screening
4155	Russo C, Cibella F, Mondati E, Caponnetto P, Frazzetto E, Caruso M, et al. Lack of Substantial Post-Cessation Weight Increase in Electronic Cigarettes Users. Int J Env Res Public Health. 2018;15. doi:10.3390/ijerph15040581	No	Not meeting inclusion criteria	Title and abstract screening
4156	Russo C, Walicka M, Caponnetto P, Cibella F, Maglia M, Alamo A, et al. Efficacy and Safety of Varenicline for Smoking Cessation in Patients With Type 2 Diabetes: A Randomized Clinical Trial. JAMA Netw Open. 2022;5: e2217709. doi:10.1001/jamanetworkopen.2022.17709	No	Not meeting inclusion criteria	Title and abstract screening
4157	Rusy DA, Honkanen A, Landrigan-Ossar MF, Chatterjee D, Schwartz LI, Lalwani K, et al. Vaping and E-Cigarette Use in Children and Adolescents: Implications on Perioperative Care From the American Society of Anesthesiologists Committee on Pediatric Anesthesia, Society for Pediatric Anesthesia, and American Academy of Pediatrics Section on An. Anesth Analg. 2021;133: 562–568. doi:10.1213/ANE.0000000000005519	No	Not meeting inclusion criteria	Title and abstract screening

4158	Ruszkiewicz J.A., Zhang Z., Goncalves F.M., Tizabi Y., Zelikoff J.T., Aschner M. Neurotoxicity of e-cigarettes. Food Chem Toxicol. 2020;138: 111245. doi:10.1016/j.fct.2020.111245	No	Not meeting inclusion criteria	Title and abstract screening
4159	Rüther T, Kahnert K, Mader M, Rabenstein A, Falkai P, Fischer E, et al. Reduction of bronchial response to mannitol after partial switch from conventional tobacco to electronic cigarette consumption. Respir Med. 2021;178: 106324. doi:10.1016/j.rmed.2021.106324	No	Not meeting inclusion criteria	Title and abstract screening
4160	Rutherford BN, Sun T, Lim CCW, Chung J, Cheng B, Davidson L, et al. Changes in Viewer Engagement and Accessibility of Popular Vaping Videos on TikTok: A 12-Month Prospective Study. Int J Env Res Public Health. 2022;19. doi:10.3390/ijerph19031141	No	Not meeting inclusion criteria	Title and abstract screening
4161	Ryan D., Miller K., Capaldi C., Pasquarello C., Yang Q., Hirose H. Massive hemoptysis bridged with VV ECMO: A case report. Front Cardiovasc Med. 2022;9: 997990. doi:10.3389/fcvm.2022.997990	No	Not meeting inclusion criteria	Title and abstract screening
4162	Ryu S, Price SK. Embodied storytelling and meaning-making at the end of life: VoicingHan avatar life-review for palliative care in cancer patients. Arts Health Int J Res Policy Pract. 2022;14: 326–340. doi:10.1080/17533015.2021.1942939	No	Not meeting inclusion criteria	Title and abstract screening
4163	Saba NF, Mody MD, Rocco JW, Haddad RI, Yom SS. Head and neck cancer: high-end technology is no guarantee of high-quality care - Authors' reply. Lancet. 2022;399: 2102–2102. doi:10.1016/S0140-6736(22)00426-3	No	Not meeting inclusion criteria	Title and abstract screening
4164	Sabbagh HJ, Abdelaziz W, Quritum M, AlKhateeb NA, Abourdan J, Qureshi N, et al. Cigarettes' use and capabilities-opportunities-motivation-for-behavior model: a multi-country survey of adolescents and young adults. Front Public Health. 2022;10: 875801. doi:10.3389/fpubh.2022.875801	No	Not meeting inclusion criteria	Title and abstract screening
4165	Saeed S., Shahroz I.M., Khan S.U., Agha A.A. Voice against tobacco: A call for integrated action for effective change. Pak J Med Sci. 2022;38: 436–437. doi:10.12669/pjms.38.ICON-2022.5788	No	Not meeting inclusion criteria	Title and abstract screening
4166	Sagud M., Mihaljevic Peles A., Pivac N. Smoking in schizophrenia: Recent findings about an old problem. Curr Opin Psychiatry. 2019;32: 402–408. doi:10.1097/YCO.0000000000000529	No	Not meeting inclusion criteria	Title and abstract screening
4167	Saha S.P., Banks M.A., Whayne T.F. Managing cardiovascular risk factors without medications: What is the evidence? Cardiovasc Hematol Agents Med Chem. 2021;19: 8–16. doi:10.2174/1871525718666200518093418	No	Not meeting inclusion criteria	Title and abstract screening
4168	Sahingoz Erdal G, Yaman M, Servi EY, Ugur H, Kasapoglu P, Cikot M, et al. Measurement of Advanced Glycation End Products Could Be Used as an Indicator of Unhealthy Nutrition for Colorectal Cancer Risk. Nutr Cancer. 2022;74: 896–902. doi:10.1080/01635581.2021.1938148	No	Not meeting inclusion criteria	Title and abstract screening
4169	Sahoo P.K., Priyadarshini S.R. Electronic nicotine delivery system. Indian J Public Health Res Dev. 2019;10: 951–955. doi:10.5958/0976-5506.2019.03619.2	No	Not meeting inclusion criteria	Title and abstract screening
4170	Sahu K.K., Mishra A.K., Lal A., Siddiqui A.D., George S.V. Cocaine-induced methemoglobinemia. World J Emerg Med. 2021;12: 81–82. doi:10.5847/WJEM.J.1920-8642.2021.01.015	No	Not meeting inclusion criteria	Title and abstract screening
4171	Sahu R, Shah K, Malviya R, Paliwal D, Sagar S, Singh S, et al. E-Cigarettes and Associated Health Risks: An Update on Cancer Potential. Adv Respir Med. 2023;91: 516–531. doi:10.3390/arm91060038	No	Not meeting inclusion criteria	Title and abstract screening
4172	Saitta D, Chowdhury A, Ferro GA, Nalis FG, Polosa R. A Risk Assessment Matrix for Public Health Principles: The Case for E-Cigarettes. Int J Env Res Public Health. 2017;14. doi:10.3390/ijerph14040363	No	Not meeting inclusion criteria	Title and abstract screening
4173	Sakaguchi C, Nagata Y, Kikuchi A, Takeshige Y, Minami N. Differences in Levels of Biomarkers of Potential Harm Among Users of a Heat-Not-Burn Tobacco Product, Cigarette Smokers, and Never-Smokers in Japan: A Post-Marketing Observational Study. Nicotine Tob Res Off J Soc Res Nicotine Tob. 2021;23: 1143–1152. doi:10.1093/ntr/ntab014	No	Not meeting inclusion criteria	Title and abstract screening
4174	Sala M, Gotti C. Electronic nicotine delivery systems (ENDS): A convenient means of smoking?. Pharmacol Res. 2023;195: 106885. doi:10.1016/j.phrs.2023.106885	No	Not meeting inclusion criteria	Title and abstract screening
4175	Salazar MM, DeCook LJ, Butterfield RJ, Zhang N, Sen A, Wu KL, et al. End-of-Life Care in Patients Undergoing Allogeneic Hematopoietic Cell Transplantation. J Palliat Med. 2022;25: 97–105. doi:10.1089/jpm.2021.0093	No	Not meeting inclusion criteria	Title and abstract screening
4176	Salem H, Kazak A, Andersen E, Belmonte F, Johansen C, Schmiegelow K, et al. Home-based cognitive behavioural therapy for families of young children with cancer (FAMOS): a nationwide randomised controlled trial. 2021;68: e28853. doi:10.1002/pbc.28853	No	Not meeting inclusion criteria	Title and abstract screening
4177	Salloum RG, Lee J, Porter M, Dallery J, McDaniel AM, Bian J, et al. Evidence-based tobacco treatment utilization among dual users of cigarettes and E-cigarettes. Prev Med. 2018;114: 193–199. doi:10.1016/j.ypmed.2018.07.010	No	Not meeting inclusion criteria	Title and abstract screening
4178	Salloum RG, LeLaurin JH, Lee JH, Lafata JE, Williams M, Wang Y, et al. Primary Care Physician Perspectives on Recommending E-cigarettes to Smokers: a Best-Worst Discrete Choice Experiment. J Gen Intern Med. 2021;36: 3353–3360. doi:10.1007/s11606-021-06615-w	No	Not meeting inclusion criteria	Title and abstract screening
4179	Salloum RG, Tan ASL, Thompson L. What Parents Need to Know About Teen Vaping and What They Can Do About It. JAMA Pediatr. 2021;175: 440. doi:10.1001/jamapediatrics.2020.6689	No	Not meeting inclusion criteria	Title and abstract screening
4180	SALOMON A., GARNER C., HAYES J.A. UP IN SMOKE: AN UNLIKELY CAUSE OF LUNG INJURY IN A MILITARY VETERAN. Chest. 2023;164: A5147–A5148. doi:10.1016/j.chest.2023.07.3334	No	Not meeting inclusion criteria	Title and abstract screening
4181	Saloojee Y, Mathee A. COVID-19 and a temporary ban on tobacco sales in South Africa: impact on smoking cessation. Tob Control. 2022;31: e207–e210. doi:10.1136/tobaccocontrol-2020-056293	No	Not meeting inclusion criteria	Title and abstract screening
4182	Salt V., Osborne C. Mental health, smoking and poverty: Benefits of supporting smokers to quit. BJPsych Bull. 2020;44: 213–218. doi:10.1192/bjb.2020.88	No	Not meeting inclusion criteria	Title and abstract screening
4183	Salvatori P. The rationale of ethanol inhalation for disinfection of the respiratory tract in SARS-CoV-2-positive asymptomatic subjects. Pan Afr Med J. 2021;40: 201. doi:10.11604/pamj.2021.40.201.31211	No	Not meeting inclusion criteria	Title and abstract screening
4184	Sampson L., Ettman C.K., Abdalla S.M., Colyer E., Dukes K., Lane K.J., et al. Financial hardship and health risk behavior during COVID-19 in a large US national sample of women. SSM - Popul Health. 2021;13: 100734. doi:10.1016/j.ssmph.2021.100734	No	Not meeting inclusion criteria	Title and abstract screening
4185	Samuel-Ryals CA, Mbah OM, Hinton SP, Cross SH, Reeve BB, Dusetzina SB. Evaluating the Contribution of Patient-Provider Communication and Cancer Diagnosis to Racial Disparities in End-of-Life Care Among Medicare Beneficiaries. JGIM J Gen Intern Med. 2021;36: 3311–3320. doi:10.1007/s11606-021-06778-6	No	Not meeting inclusion criteria	Title and abstract screening
4186	Sanchez J, Tabuyo A, Morales AT, Pitteloud MJ, Kshetry A, Schlumbrecht M. Palliative Medicine Referral Patterns and End-of-Life Interventions Among Patients with Advanced or Metastatic Gynecologic Cancer in a University Cancer Center (QI422). J Pain Symptom Manage. 2022;63: 897–897. doi:10.1016/j.jpainsymman.2022.02.109	No	Not meeting inclusion criteria	Title and abstract screening

4187	Sanchez S, Kaufman P, Pelletier H, Baskerville B, Feng P, O'Connor S, et al. Is vaping cessation like smoking cessation? A qualitative study exploring the responses of youth and young adults who vape e-cigarettes. Addict Behav. 2021;113: 106687. doi:10.1016/j.addbeh.2020.106687	No	Not meeting inclusion criteria	Title and abstract screening
4188	Sanchez S, Kundu A, Limanto E, Selby P, Baskerville NB, Chaiton M. Smartphone Apps for Vaping Cessation: Quality Assessment and Content Analysis. JMIR Mhealth Uhealth. 2022;10: e31309. doi:10.2196/31309	No	Not meeting inclusion criteria	Title and abstract screening
4189	Sanchez S, Deck A, Baskerville NB, Chaiton M. Supporting Youth Vaping Cessation With the Crush the Crave Smartphone App: Protocol for a Randomized Controlled Trial. JMIR Res Protoc. 2023;12: e42956. doi:10.2196/42956	No	Not meeting inclusion criteria	Title and abstract screening
4190	Sanchez-Roige S, Cox NJ, Johnson EO, Hancock DB, Davis LK. Alcohol and cigarette smoking consumption as genetic proxies for alcohol misuse and nicotine dependence. Drug Alcohol Depend. 2021;221: 108612. doi:10.1016/j.drugalcdep.2021.108612	No	Not meeting inclusion criteria	Title and abstract screening
4191	Sánchez-Romero LM, Liber AC, Li Y, Yuan Z, Tam J, Travis N, et al. The smoking and vaping model, A user-friendly model for examining the country-specific impact of nicotine VAPING product use: application to Germany. BMC Public Health. 2023;23: 2299. doi:10.1186/s12889-023-17152-y	No	Not meeting inclusion criteria	Title and abstract screening
4192	Sanford BT, Brownstein NC, Baker NL, Palmer AM, Smith TT, Rojewski AM, et al. Shift From Smoking Cigarettes to Vaping Nicotine in Young Adults. JAMA Intern Med. 2023. doi:10.1001/jamainternmed.2023.5239	No	Not meeting inclusion criteria	Title and abstract screening
4193	Sanford BT, Rojewski AM, Palmer AM, Baker NL, Carpenter MJ, Smith TT, et al. E-Cigarette Screening in Primary Care. Am J Prev Med. 2023;65: 517–520. doi:10.1016/j.amepre.2023.02.030	No	Not meeting inclusion criteria	Title and abstract screening
4194	Sanford BT, Toll BA, Palmer AM, Foster MG, Cummings KM, Stansell S, et al. Tobacco Treatment Outcomes for Hospital Patients With and Without Mental Health Diagnoses. Front Psychiatry. 2022;13: 853001. doi:10.3389/fpsyt.2022.853001	No	Not meeting inclusion criteria	Title and abstract screening
4195	Sanford N.N., Mahal B.A. The Use of e-Cigarettes in Patients with Cancer - A Double Shipwreck - In Reply. JAMA Oncol. 2019;5: 1372. doi:10.1001/jamaoncol.2019.2396	No	Not meeting inclusion criteria	Title and abstract screening
4196	SANGHVI A., NANAVATY D., BANSAL K., SHIREEN KANAMGODE S., SINGH S., NARAYAN G., et al. VAPING IS ASSOCIATED WITH PULMONARY EMBOLISM: A NATIONWIDE ANALYSIS. Chest. 2023;164: A6380. doi:10.1016/j.chest.2023.07.4111	No	Not meeting inclusion criteria	Title and abstract screening
4197	Santana LADM, Andrade JRG, Souza ECM, Nogueira DPS, Nunes FS, Trento CL, et al. Use of electronic cigarette and the risk of developing potentially malignant disorders: What are we really supposed to know? J Stomatol Oral Maxillofac Surg. 2023;124: 101357. doi:10.1016/j.jormas.2022.101357	No	Not meeting inclusion criteria	Title and abstract screening
4198	Santana LA da M, Andrade JRG, Souza E de CM, Nogueira DP de S, Nunes F de S, Trento CL, et al. Use of electronic cigarette and the risk of developing potentially malignant disorders: What are we really supposed to know?. J Stomatol Oral Maxillofac Surg. 2022; 101357. doi:10.1016/j.jormas.2022.101357	No	Not meeting inclusion criteria	Title and abstract screening
4199	Santi A.D., Emamian A., Appu M., Siongco A., Laningham F., Tcheng W.Y., et al. What's lungs got to do, got to do with it? A 13-year-old male with vomiting and abdominal pain. Pediatrics. 2021;147: 893–894. doi:10.1542/peds.147.3_MeetingAbstract.893	No	Not meeting inclusion criteria	Title and abstract screening
4200	Santiago-Torres M, Mull KE, Sullivan BM, Bricker JB. Relative Efficacy of an Acceptance and Commitment Therapy-Based Smartphone App with a Standard US Clinical Practice Guidelines-Based App for Smoking Cessation in Dual Users of Combustible and Electronic Cigarettes: Secondary Findings from a Randomized Tri. Subst Use Misuse. 2023; 1–10. doi:10.1080/10826084.2023.2293732	No	Not meeting inclusion criteria	Title and abstract screening
4201	Santiago-Torres M, Mull KE, Sullivan BM, Bricker JB. Use of e-Cigarettes in Cigarette Smoking Cessation: Secondary Analysis of a Randomized Controlled Trial. JMIR Mhealth Uhealth. 2023;11: e48896. doi:10.2196/48896	No	Not meeting inclusion criteria	Title and abstract screening
4202	Santiago-Torres M., Mull K.E., Sullivan B.M., Kendzor D.E., Bricker J.B. Efficacy and utilization of smartphone applications for smoking cessation among low-income adults: Secondary analysis of the iCanQuit randomized trial. Drug Alcohol Depend. 2022;231: 109258. doi:10.1016/j.drugalcdep.2021.109258	No	Not meeting inclusion criteria	Title and abstract screening
4203	Santiago-Torres M., Mull K.E., Sullivan B.M., Zvolensky M.J., Kahler C.W., Bricker J.B. Efficacy of smartphone applications for smoking cessation in heavy-drinking adults: Secondary analysis of the iCanQuit randomized trial. Addict Behav. 2022;132: 107377. doi:10.1016/j.addbeh.2022.107377	No	Not meeting inclusion criteria	Title and abstract screening
4204	Santiago-Torres M, Mull KE, Sullivan BM, Rigotti NA, Bricker JB. Acceptance and Commitment Therapy-Based Smartphone Applications for Cessation of Tobacco Use among Adults with High Nicotine Dependence: Results from the iCanQuit Randomized Trial. Subst Use Misuse. 2023;58: 354–364. doi:10.1080/10826084.2022.2161317	No	Not meeting inclusion criteria	Title and abstract screening
4205	Santos B, Flumignan R, Civile V, Atallah Á, Nakano L. Prophylactic anticoagulants for non-hospitalised people with COVID-19. Cochrane Database Syst Rev. 2023. doi:10.1002/14651858.CD015102.pub2	No	Not meeting inclusion criteria	Title and abstract screening
4206	Santos PMG, Mathis NJ, Lapen K, Lobaugh S, Yerramilli D, Bekelman JE, et al. Assessment of Guideline-Nonconcordant Radiotherapy in Medicare Beneficiaries With Metastatic Cancer Near the End of Life, 2015-2017. JAMA Health Forum. 2022;3: 1–4. doi:10.1001/jamahealthforum.2021.4468	No	Not meeting inclusion criteria	Title and abstract screening
4207	Santoso H., Chalidyanto D., Laksono A.D. The prevalence of cancer in indonesia: An ecological analysis. Indian J Forensic Med Toxicol. 2021;15: 3170–3176. doi:10.37506/ijfmt.v15i3.15791	No	Not meeting inclusion criteria	Title and abstract screening
4208	Sapru A., Sommerset J., Karmy-Jones R. Is There a Role for the Vascular Laboratory in Supporting Home-Based Vascular Rehabilitation Programs? J Vasc Ultrasound. 2022;46: 61–66. doi:10.1177/15443167221076128	No	Not meeting inclusion criteria	Title and abstract screening
4209	Saqib HWU, Farooqi HA. The non-small-cell lung cancer crisis: a worldwide need for radiomics-enhanced early detection solutions. Eur J Cardio-Thorac Surg Off J Eur Assoc Cardio-Thorac Surg. 2023;64. doi:10.1093/ejcts/ezad367	No	Not meeting inclusion criteria	Title and abstract screening
4210	Sarel E., Hoppenstein D.R., Lahav M., Ifrach N., Fanadka F., Fredman B.D. Respiratory failure induced by vaping-associated pulmonary injury: Case report of a new entity. Isr Med Assoc J. 2021;23: 59–60.	No	Not meeting inclusion criteria	Title and abstract screening
4211	Sargent JD, Edwards KC, Emond J, Tanski S, Taylor KA, Pierce JP, et al. Author Response to E-cigarettes and Respiratory Disorder: The Broader Research Context. Nicotine Tob Res. 2023;25: 1217–1218. doi:10.1093/ntr/ntad036	No	Not meeting inclusion criteria	Title and abstract screening
4212	Sargi J., Heaton J., Adarkwah O., Parhar G., Zaman K., Mesiha N., et al. A case of exogenous lipoid pneumonia in a critically ill patient. Am J Respir Crit Care Med. 2021;203. doi:10.1164/ajrccm-conference.2021.203.1_MeetingAbstracts.A2161	No	Not meeting inclusion criteria	Title and abstract screening
4213	Sarin A., Seth S. Voice of Tobacco Victims (VoTV) campaign sensitized more than 21000+ policymakers & enforcers in India on tobacco control in 9 years. Tob Induc Dis. 2021;19. doi:10.18332/tid/140855	No	Not meeting inclusion criteria	Title and abstract screening
4214	Sarin A., Singhavi H., Patil A., Mehrotra R., Seth S., Janardhanan R. Descriptive investigation of government events and public interest analysis using Google trends between pictorial health warnings policy implementation and electronic nicotine delivery systems ban in India. Medico-Leg Update. 2021;21: 210–217.	No	Not meeting inclusion criteria	Title and abstract screening
4215	Satchell T, Diaz MC, Stephens D, Bertrand A, Schillo BA, Whitsel LP. The impact of two state-level approaches to restricting the sale of flavored tobacco products. BMC Public Health. 2022;22: 1799. doi:10.1186/s12889-022-14172-y	No	Not meeting inclusion criteria	Title and abstract screening

4216	Satish M., Basavaraj R., Begum N., Kumar A.S. COMPARISON OF BLOOD PRESSURE IN SMOKELESS TOBACCO USERS AND NON TOBACCO USERS. J Cardiovasc Dis Res. 2021;12: 383–391. doi:10.31838/jcdr.2021.12.06.49	No	Not meeting inclusion criteria	Title and abstract screening
4217	Sato A, Ishigami A. Effects of heated tobacco product aerosol extracts on DNA methylation and gene transcription in lung epithelial cells. Toxicol Appl Pharmacol. 2023;475: 116637. doi:10.1016/j.taap.2023.116637	No	Not meeting inclusion criteria	Title and abstract screening
4218	Sato A, Fujimori M, Shirai Y, Umezawa S, Mori M, Jinno S, et al. Assessing the need for a question prompt list that encourages end-of-life discussions between patients with advanced cancer and their physicians: A focus group interview study - ERRATUM. Palliat Support Care. 2022;20: 618–620. doi:10.1017/S1478951522000153	No	Not meeting inclusion criteria	Title and abstract screening
4219	Sato A, Fujimori M, Shirai Y, Umezawa S, Mori M, Jinno S, et al. Assessing the need for a question prompt list that encourages end-of-life discussions between patients with advanced cancer and their physicians: A focus group interview study. Palliat Support Care. 2022;20: 564–569. doi:10.1017/S1478951521001796	No	Not meeting inclusion criteria	Title and abstract screening
4220	Sato F, Nakamura Y, Kayaba K, Ishikawa S. Stroke Risk Due to Smoking Characterized by Sex Differences in Japan: The Jichi Medical School Cohort Study. J Stroke Cerebrovasc Dis. 2022;31: 106203. doi:10.1016/j.jstrokecerebrovasdis.2021.106203	No	Not meeting inclusion criteria	Title and abstract screening
4221	Savill KMZ, Gentile D, Asgarisabet P, Brown M, Chopra D, John WS, et al. Differences in racial and sex at birth representativeness between randomized controlled trial (RCT) and real-world evidence (RWE) or Surveillance, Epidemiology, and End Results (SEER) datasets in advanced non-small cell lung cancer (aNSCLC). J Clin Oncol. 41: e18654–e18654. doi:10.1200/JCO.2023.41.16_suppl.e18654	No	Not meeting inclusion criteria	Title and abstract screening
4222	Savin MJ, Frank-Pearce SG, Pulvers K, Vidrine DJ. The association between lifetime polytobacco use and intention to quit among HIV-positive cigarette smokers. Drug Alcohol Depend. 2018;191: 152–158. doi:10.1016/j.drugalcdep.2018.05.007	No	Not meeting inclusion criteria	Title and abstract screening
4223	Savitz D.A. Invited Commentary: The Epidemiology of Electronic Cigarettes and Reproductive Health Begins. Am J Epidemiol. 2021;190: 362–364. doi:10.1093/aje/kwaa066	No	Not meeting inclusion criteria	Title and abstract screening
4224	Sawyer LE, Brandon TH. Unintended Consequences: Testing the Effects of Adolescent-Targeted Anti-Vaping Media upon Adult Smokers. Nicotine Tob Res. 2022. doi:10.1093/ntr/ntac277	No	Not meeting inclusion criteria	Title and abstract screening
4225	Sawyer LE, Brandon TH. Unintended Consequences: Testing the Effects of Adolescent-Targeted Anti-vaping Media Upon Adult Smokers. Nicotine Tob Res. 2023;25: 967–974. doi:10.1093/ntr/ntac277	No	Not meeting inclusion criteria	Title and abstract screening
4226	Sayed IM, Tindle C, Fonseca AG, Ghosh P, Das S. Functional assays with human patient-derived enteroid monolayers to assess the human gut barrier. STAR Protoc. 2021;2: 100680. doi:10.1016/j.xpro.2021.100680	No	Not meeting inclusion criteria	Title and abstract screening
4227	Saz-Lara A, Martínez-Vizcaíno V, Sequí-Domínguez I, Álvarez-Bueno C, Notario-Pacheco B, Cavero-Redondo I. The effect of smoking and smoking cessation on arterial stiffness: a systematic review and meta-analysis. Eur J Cardiovasc Nurs. 2022;21: 297–306. doi:10.1093/eurjcn/zvab102	No	Not meeting inclusion criteria	Title and abstract screening
4228	Schaal CM, Bora-Singhal N, Kumar DM, Chellappan SP. Regulation of Sox2 and stemness by nicotine and electronic-cigarettes in non-small cell lung cancer. Mol Cancer. 2018;17: 149. doi:10.1186/s12943-018-0901-2	No	Not meeting inclusion criteria	Title and abstract screening
4229	Schaller K., Kahnert S., Mons U. E-cigarette use in Germany from 2015 to 2017. Tob Induc Dis. 2018;16: 96–97. doi:10.18332/tid/84010	No	Not meeting inclusion criteria	Title and abstract screening
4230	Scharf P., Rizzetto F., Xavier L.F., Farsky S.H.P. Xenobiotics Delivered by Electronic Nicotine Delivery Systems: Potential Cellular and Molecular Mechanisms on the Pathogenesis of Chronic Kidney Disease. Int J Mol Sci. 2022;23: 10293. doi:10.3390/ijms231810293	No	Not meeting inclusion criteria	Title and abstract screening
4231	Scharf P., Sandri S., Borges P.P., de Oliveira T.F., Aschner M., Farsky S.H.P. P13-12: Heated tobacco and cigarette smoke exposure impairs macrophage responsiveness and functions. Toxicol Lett. 2023;384: S163. doi:10.1016/S0378-4274%2823%2900652-5	No	Not meeting inclusion criteria	Title and abstract screening
4232	Schauer GL, Rosenberry ZR, Peters EN. Marijuana and tobacco co-administration in blunts, spliffs, and mulled cigarettes: A systematic literature review. Addict Behav. 2017;64: 200–211. doi:10.1016/j.addbeh.2016.09.001	No	Not meeting inclusion criteria	Title and abstract screening
4233	Scheibein F, McGirr K, Morrison A, Roche W, Wells JSG. An exploratory non-randomized study of a 3-month electronic nicotine delivery system (ENDS) intervention with people accessing a homeless supported temporary accommodation service (STA) in Ireland. Harm Reduct J. 2020;17: 73. doi:10.1186/s12954-020-00406-y	No	Not meeting inclusion criteria	Title and abstract screening
4234	Scheibein F, McGirr K, Morrison A, Roche W, Wells JSG. "An exploratory non-randomized study of a 3-month electronic nicotine delivery system (ENDS) intervention with people accessing a homeless supported temporary accommodation service (STA) in Ireland": Correction. Harm Reduct J. 2021;18. doi:10.1186/s12954-021-00549-6https://dx.doi.org/10.1186/s12954-021-00549-6	No	Not meeting inclusion criteria	Title and abstract screening
4235	Scheier LM, Komarc M. Are E-cigarette Users a Unique Group of Smokers? Latent Class Analysis of the National Youth Tobacco Survey. J Drug Educ. 2020;49: 87–114. doi:10.1177/0047237920980483	No	Not meeting inclusion criteria	Title and abstract screening
4236	Schellack N., Schellack G., Bronkhorst E. Smoking cessation in review. SA Pharm J. 2020;87: 43–46.	No	Not meeting inclusion criteria	Title and abstract screening
4237	Scherman A., Tolosa J.E., McEvoy C. Smoking cessation in pregnancy: a continuing challenge in the United States. Ther Adv Drug Saf. 2018;9: 457–474. doi:10.1177/2042098618775366	No	Not meeting inclusion criteria	Title and abstract screening
4238	Schiavon S, Hodgin K, Sellers A, Hendricks PS, Gaggar A, Scarinci I, et al. Differences among cigarette-only smokers compared to dual users of cigarettes and little cigars/cigarillos in the criminal justice population. Addict Behav. 2019;90: 236–240. doi:10.1016/j.addbeh.2018.11.001	No	Not meeting inclusion criteria	Title and abstract screening
4239	Schichlein KD, Love CA, Conolly MP, Kurz JL, Hickman ED, Clapp PW, et al. Vaping product exposure system (VaPES): a novel in vitro aerosol deposition system. Inhal Toxicol. 2023;35: 324–332. doi:10.1080/08958378.2023.2289021	No	Not meeting inclusion criteria	Title and abstract screening
4240	Schimpf M.D., Ulmer T.E., Hiller H.M., Barbuto A.F. The use of blue lotus (Nymphaea Caerulea) in vaping devices: A case series. J Med Toxicol. 2021;17: 135. doi:10.1007/s13181-021-00832-9	No	Not meeting inclusion criteria	Title and abstract screening
4241	Schmidt S. Vaper, beware: The unique toxicological profile of electronic cigarettes. Environ Health Perspect. 2020;128: 052001. doi:10.1289/EHP6628	No	Not meeting inclusion criteria	Title and abstract screening
4242	Schmitz JM, Stotts AL, Yoon JH, Northrup TF, Villarreal YR, Yammine L, et al. Naltrexone plus bupropion reduces cigarette smoking in individuals with methamphetamine use disorder: A secondary analysis from the CTN ADAPT-2 trial. J Subst Use Addict Treat. 2023;151: 208987. doi:10.1016/j.josat.2023.208987	No	Not meeting inclusion criteria	Title and abstract screening
4243	Schneller LM, Lindgren BR, Shields PG, Hatsukami DK, O'Connor RJ. Strong preference for mint snus flavor among research participants. Addict Behav Rep. 2017;6: 51–55. doi:10.1016/j.abrep.2017.07.004https://dx.doi.org/10.1016/j.abrep.2017.07.004	No	Not meeting inclusion criteria	Title and abstract screening
4244	Schneller LM, Felicione NJ, Hammond D, Goniewicz ML, O'Connor RJ. Tobacco-Free Oral Nicotine Product Use Among Youth in the U.S., 2019-2021. AJPM Focus. 2023;2: 100061. doi:10.1016/j.focus.2022.100061	No	Not meeting inclusion criteria	Title and abstract screening

4245	Schneller LM, Kasza KA, Hammond D, Bansal-Travers M, O'Connor R, Hyland A. E-cigarette and tobacco product use among NYS youth before and after a state-wide vaping flavour restriction policy, 2020-2021. Tob Control. 2022;31: s161–s166. doi:10.1136/tobaccocontrol-2022-057450	No	Not meeting inclusion criteria	Title and abstract screening
4246	Schuler MS, Wong EC, Ramchand R. Military service branch differences in alcohol use, tobacco use, prescription drug misuse, and mental health conditions. Drug Alcohol Depend. 2022;235: 109461. doi:10.1016/j.drugalcdep.2022.109461	No	Not meeting inclusion criteria	Title and abstract screening
4247	Schulz JA, West JC, Hall JP, Villanti AC. Disparities in Tobacco Use by Disability and Type: Findings From the 2019 National Health Interview Survey. Am J Prev Med. 2022;63: 552–563. doi:10.1016/j.amepre.2022.05.004	No	Not meeting inclusion criteria	Title and abstract screening
4248	Schulz JA, Parker MA, Villanti AC. Trends in cigarette smoking prevalence and status among U.S. adults with disabilities, 2015-2019. Drug Alcohol Depend. 2023;243: 109738. doi:10.1016/j.drugalcdep.2022.109738	No	Not meeting inclusion criteria	Title and abstract screening
4249	Schuster RM, Cather C, Pachas GN, Nielsen L, Iroegbulem V, Dufour J, et al. A randomized controlled trial of varenicline and brief behavioral counseling delivered by lay counselors for adolescent vaping cessation: Study protocol. Front Psychiatry. 2023;14: 1083791. doi:10.3389/fpsyt.2023.1083791	No	Not meeting inclusion criteria	Title and abstract screening
4250	Schuster RM, Cather C, Pachas GN, Zhang H, Cieslak KM, Hoeppner SS, et al. Predictors of tobacco abstinence in outpatient smokers with schizophrenia or bipolar disorder treated with varenicline and cognitive behavioral smoking cessation therapy. Addict Behav. 2017;71: 89–95. doi:10.1016/j.addbeh.2017.02.028	No	Not meeting inclusion criteria	Title and abstract screening
4251	Schwarz ES. Inhalants. Crit Care Clin. 2021;37: 687–702. doi:10.1016/j.ccc.2021.03.014	No	Not meeting inclusion criteria	Title and abstract screening
4252	Schwotzer D., Gigliotti A., Irshad H., Dye W., McDonald J. Phytol, not propylene glycol, causes severe pulmonary injury after inhalation dosing in Sprague-Dawley rats. Inhal Toxicol. 2021;33: 33–40. doi:10.1080/08958378.2020.1867260	No	Not meeting inclusion criteria	Title and abstract screening
4253	Scott A, Lugg ST, Aldridge K, Lewis KE, Bowden A, Mahida RY, et al. Pro-inflammatory effects of e-cigarette vapour condensate on human alveolar macrophages. Thorax. 2018;73: 1161–1169. doi:10.1136/thoraxjnl-2018-211663	No	Not meeting inclusion criteria	Title and abstract screening
4254	Scott J.G., McKeon G. SHOULD PSYCHIATRISTS SUPPORT INCREASED REGULATED AVAILABILITY OF E-CIGARETTES AS A HARM-REDUCTION MEASURE? Aust N Z J Psychiatry. 2022;56: 18. doi:10.1177/00048674221088686	No	Not meeting inclusion criteria	Title and abstract screening
4255	Scott JG, Jhetam S, Chen R, Daglish M. Should psychiatrists support the availability of nicotine e-cigarettes in Australia? Australas Psychiatry. 2019;27: 392–395. doi:10.1177/1039856219859271	No	Not meeting inclusion criteria	Title and abstract screening
4256	Scullion J. Chronic obstructive pulmonary disease: all things inhaled and more. Indep Nurse. 2022;2022: 14–16. doi:10.12968/indn.2022.14	No	Not meeting inclusion criteria	Title and abstract screening
4257	Scully M, Greenhalgh E, Bain E, Wakefield M, Durkin S, White V. E-cigarette use and other risk factors associated with tobacco smoking susceptibility among Australian adolescents. Aust N Z J Public Health. 2023;47: 100076. doi:10.1016/j.anzjph.2023.100076	No	Not meeting inclusion criteria	Title and abstract screening
4258	Scully M, Wakefield M, Scollo M, Durkin S, White V. Prevalence and correlates of flavour capsule cigarette use among Australian adolescents. Health Promot J Austr. 2022;33: 920–925. doi:10.1002/hpja.570	No	Not meeting inclusion criteria	Title and abstract screening
4259	Seal B., Sharma A., Thronton M., Liu C., Hauptmann E., Ali M., et al. Impact of Donor Vaping or Electronic Cigarette Use on Early Outcomes after Lung Transplantation- A Single Center's Experience. J Heart Lung Transplant. 2023;42: S401–S402. doi:10.1016/j.healun.2023.02.1040	No	Not meeting inclusion criteria	Title and abstract screening
4260	See JHJ, Yong TH, Poh SLK, Lum YC. Smoker motivations and predictors of smoking cessation: lessons from an inpatient smoking cessation programme. Singap Med J. 2019;60: 583–589. doi:10.11622/smedj.2019148	No	Not meeting inclusion criteria	Title and abstract screening
4261	Seftel A.D. Male/Female Sexual Function/Dysfunction, Andrology. J Urol. 2022;207: 1137. doi:10.1097/JU.0000000000002479	No	Not meeting inclusion criteria	Title and abstract screening
4262	Seidenberg A.B., Ayo-Yusuf O.A., Rees V.W. Characteristics of "American snus" and Swedish snus products for sale in Massachusetts, USA. Nicotine Tob Res. 2018;20: 262–266. doi:10.1093/ntr/ntw334	No	Not meeting inclusion criteria	Title and abstract screening
4263	Seiler-Ramadas R, Sandner I, Haider S, Grabovac I, Dorner TE. Health effects of electronic cigarette (e-cigarette) use on organ systems and its implications for public health. Wien Klin Wochenschr. 2021;133: 1020–1027. doi:10.1007/s00508-020-01711-z	No	Not meeting inclusion criteria	Title and abstract screening
4264	Selby DL, Harrison AA, Fozard TE, Kolokotroni KZ. Dissociating wanting and anticipated liking from consummatory liking in smokers with different levels of nicotine dependence. Addict Behav. 2020;102: 106185. doi:10.1016/j.addbeh.2019.106185	No	Not meeting inclusion criteria	Title and abstract screening
4265	Selva Kumar D, Peterson M, Zhang C, Fagan P, Nahvi S. The impact of menthol cigarette use on quit attempts and abstinence among smokers with opioid use disorder. Addict Behav. 2021;118: 106880. doi:10.1016/j.addbeh.2021.106880	No	Not meeting inclusion criteria	Title and abstract screening
4266	Selya AS, Rose JS, Dierker L, Hedeker D, Mermelstein RJ. "Commentary on Selya et al. (2018): Advantages in the consideration of causal mechanisms for studies of gateway e-cigarette use": Erratum. Addiction. 2018;113: 1367. doi:10.1111/add.14253https://dx.doi.org/10.1111/add.14253	No	Not meeting inclusion criteria	Title and abstract screening
4267	Selya AS, Dierker L, Rose JS, Hedeker D, Mermelstein RJ. The Role of Nicotine Dependence in E-Cigarettes' Potential for Smoking Reduction. Nicotine Tob Res. 2018;20: 1272–1277. doi:10.1093/ntr/ntx160	No	Not meeting inclusion criteria	Title and abstract screening
4268	Selya AS, Rose JS, Dierker L, Hedeker D, Mermelstein RJ. Evaluating the mutual pathways among electronic cigarette use, conventional smoking and nicotine dependence. Addiction. 2018;113: 325–333. doi:10.1111/add.14013	No	Not meeting inclusion criteria	Title and abstract screening
4269	Senior K. NHS England ends treatment break rule in bowel cancer. Lancet Oncol. 2023;24 1077-4114 (Print): 842–842. doi:10.1016/S1470-2045(23)00330-3	No	Not meeting inclusion criteria	Title and abstract screening
4270	Seo HG, Xu SS, Li G, Gravely S, Quah ACK, Lee S, et al. Reasons for Initiation and Regular Use of Heated Tobacco Products among Current and Former Smokers in South Korea: Findings from the 2020 ITC Korea Survey. Int J Env Res Public Health. 2023;20. doi:10.3390/ijerph20064963	No	Not meeting inclusion criteria	Title and abstract screening
4271	Seo YS, Chang YP. Racial and Ethnic Differences in E-Cigarette and Cigarette Use Among Adolescents. J Immigr Minor Health. 2022;24: 713–720. doi:10.1007/s10903-021-01229-0	No	Not meeting inclusion criteria	Title and abstract screening
4272	Seri A., Rattanawong P., Firouzbakht T., Sorajja D. Vaping tetrahydrocannabinol unmasks Brugada pattern and induces ventricular fibrillation in Brugada syndrome: A case report. Eur Heart J - Case Rep. 2022;6: ytac200. doi:10.1093/ehjcr/ytac200	No	Not meeting inclusion criteria	Title and abstract screening
4273	Seth S., Sarin A. Communities Against Tobacco (CAT): An important initiative in tobacco control in India. Tob Induc Dis. 2021;19. doi:10.18332/tid/140985	No	Not meeting inclusion criteria	Title and abstract screening

4274	Sethi S., Ju X., Antonsson A., Canfell K., Smith M.A., Garvey G., et al. Oral HPV Infection among Indigenous Australians; Incidence, Persistence, and Clearance at 12-Month Follow-up. Cancer Epidemiol Biomarkers Prev. 2022;31: 604–613. doi:10.1158/1055-9965.EPI-21-1056	No	Not meeting inclusion criteria	Title and abstract screening
4275	Seyler T, Blount BC, Wang L. HPLC-MS/MS Method for Measuring Cotinine and Trans-3'-Hydroxycotinine in Bronchoalveolar Lavage Fluid of Patients with e-Cigarette, or Vaping, Product Use-Associated Lung Injury (EVALI). J Anal Toxicol. 2023. doi:10.1093/jat/bkad077	No	Not meeting inclusion criteria	Title and abstract screening
4276	Shabestari A.N., Tamehri Zadeh S.S., Zahmatkesh P., Baghdadabad L.Z., Mirzaei A., Mashhadi R., et al. The impact of conventional smoking versus electronic cigarette on the expression of VEGF, PEMPA1, and PTEN in rat prostate. Prostate Int. 2022. doi:10.1016/j.prnil.2022.11.002	No	Not meeting inclusion criteria	Title and abstract screening
4277	Shabestari AN, Tamehri Zadeh SS, Zahmatkesh P, Baghdadabad LZ, Mirzaei A, Mashhadi R, et al. The impact of conventional smoking versus electronic cigarette on the expression of VEGF, PEMPA1, and PTEN in rat prostate. Prostate Int. 2023;11: 76–82. doi:10.1016/j.prnil.2022.11.002	No	Not meeting inclusion criteria	Title and abstract screening
4278	Shafie-Khorassani F, Piper ME, Jorenby DE, Baker TB, Benowitz NL, Hayes-Birchler T, et al. Associations of demographics, dependence, and biomarkers with transitions in tobacco product use in a cohort of cigarette users and dual users of cigarettes and e-cigarettes. Nicotine Tob Res Off J Soc Res Nicotine Tob. 2022. doi:10.1093/ntr/ntac207	No	Not meeting inclusion criteria	Title and abstract screening
4279	Shah C, Singh P, Matin S, Farrow J, Magon R, Zia A, et al. A physician associate-led clinic for people with severe mental illness in the United Kingdom. JAAPA. 2021;34: 1–6. doi:10.1097/01.JAA.0000758220.38067.49	No	Not meeting inclusion criteria	Title and abstract screening
4280	Shah K., Murthy A., Kennedy C. E-cigarette and vaping: Overview of product use related lung injury and its implications for medical & public health providers in the age of respiratory illness. Am J Addict. 2021;30: 252–253. doi:10.1111/ajad.13173	No	Not meeting inclusion criteria	Title and abstract screening
4281	Shah P.A., Cunningham C.O., Brisbane M.T., Deluca J.P., Nahvi S. Use of Smoking Cessation Methods among Patients Receiving Office-based Buprenorphine Maintenance Treatment. J Addict Med. 2017;11: 494–497. doi:10.1097/ADM.0000000000000356	No	Not meeting inclusion criteria	Title and abstract screening
4282	Shah S., Weber G., Nathan N. Beyond the Smoke Screen: Vaping in Adolescents. Anesth Analg. 2021;133: 561. doi:10.1213/ANE.0000000000005684	No	Not meeting inclusion criteria	Title and abstract screening
4283	Shah S.I., Javier J.R., Brumberg H.L. The vapes of wrath: advocating to protect children from electronic nicotine systems in the age of flavored vapes. Pediatr Res. 2020;87: 972–975. doi:10.1038/s41390-020-0872-z	No	Not meeting inclusion criteria	Title and abstract screening
4284	Shah V.D., Mohammad A., Nandkeolyar S., Stoletniy L., Contractor T. Torsades de Pointes due to Excessive Marijuana Use in a Susceptible Patient. Case Rep Cardiol. 2021;2021: 6621496. doi:10.1155/2021/6621496	No	Not meeting inclusion criteria	Title and abstract screening
4285	Shah W, O'Driscoll F, Ahmed S, Faul J. Documentation of electronic nicotine delivery systems (ENDS) in medically admitted patients in connolly hospital Dublin. Fiona O'driscoll, Shahzad Ahmed, Waheed Shah professor John Faul, Connolly hospital Dublin, Dublin. 2018;52. doi:10.1183/13993003.congress-2018.PA4548	No	Not meeting inclusion criteria	Title and abstract screening
4286	Shahab L, Brown J, Boelen L, Beard E, West R, Munafò MR. Unpacking the Gateway Hypothesis of E-Cigarette Use: The Need for Triangulation of Individual- and Population-Level Data. Nicotine Tob Res. 2022;24: 1315–1318. doi:10.1093/ntr/ntac035	No	Not meeting inclusion criteria	Title and abstract screening
4287	Shahandeh N, Chowdhary H, Middlekauff HR. Vaping and cardiac disease. Heart. 2021;107: 1530–1535. doi:10.1136/heartjnl-2020-318150	No	Not meeting inclusion criteria	Title and abstract screening
4288	Shaheen R.S., Alsaif F.M., Alghofaily G.A., Alhumaid N.S., Almusallam R.Z., Alharthi R.A. The prevalence and extent of physiological and pathological gingival pigmentation in patients visiting Riyadh Elm University clinics. Pak J Med Health Sci. 2021;15: 3039–3043. doi:10.53350/pjmhs2115103039	No	Not meeting inclusion criteria	Title and abstract screening
4289	Shahwan S, Abdin E, Shafie S, Chang S, Sambasivam R, Zhang Y, et al. Prevalence and correlates of smoking and nicotine dependence: results of a nationwide cross-sectional survey among Singapore residents. BMJ Open. 2019;9: e032198. doi:10.1136/bmjopen-2019-032198	No	Not meeting inclusion criteria	Title and abstract screening
4290	Shalata W, Abo Abod M, Golosky M, Boehm Cohen L, Kassirer M, Kamenev I, et al. Cannabis Vaping-induced Lung Injury. Isr Med Assoc J. 2023;25: 360–361.	No	Not meeting inclusion criteria	Title and abstract screening
4291	Shang C, Chaloupka FJ. The Trend of Voluntary Warnings in Electronic Nicotine Delivery System Magazine Advertisements. Int J Env Res Public Health. 2017;14. doi:10.3390/ijerph14010062	No	Not meeting inclusion criteria	Title and abstract screening
4292	Shang C. How to regulate vaping products to advance cancer prevention: Evidence from online stores. J Clin Oncol. 2022;40. doi:10.1200/JCO.2022.40.16_suppl.e22502	No	Not meeting inclusion criteria	Title and abstract screening
4293	Shangase ZP, Tsoka-Gwegweni JM, Egbe CO. Barriers to Smoking Cessation Among Drug-Resistant Tuberculosis Patients in South Africa. Ann Glob Health. 2017;83: 501–508. doi:10.1016/j.aogh.2017.05.007	No	Not meeting inclusion criteria	Title and abstract screening
4294	Shao XM, Liu S, Lee ES, Fung D, Pei H, Liang J, et al. Chronic intermittent nicotine delivery via lung alveolar region-targeted aerosol technology produces circadian pharmacokinetics in rats resembling human smokers. J Appl Physiol Bethesda Md 1985. 2018;125: 1555–1562. doi:10.1152/japplphysiol.00357.2018	No	Not meeting inclusion criteria	Title and abstract screening
4295	Shao Y, Zou J, Xie Z, Mayne RG, Ossip DJ, Rahman I, et al. Perceptions of Oral Nicotine Pouches on Reddit: Observational Study. J Med Internet Res. 2022;24: e37071. doi:10.2196/37071	No	Not meeting inclusion criteria	Title and abstract screening
4296	Sharfstein J.M. How Do You Solve a Problem Like Juul? Milbank Q. 2018;96: 417–420. doi:10.1111/1468-0009.12334	No	Not meeting inclusion criteria	Title and abstract screening
4297	Shariat S.F. Electronic Cigarettes: A Wolf in Sheep's Clothing. Eur Urol. 2017;71: 924–925. doi:10.1016/j.eururo.2017.01.014	No	Not meeting inclusion criteria	Title and abstract screening
4298	Sharifi H. Health care professional and cigarette cessation volunteers knowledge, attitude and practice on e-cigarettes. Tob Induc Dis. 2018;16: 94. doi:10.18332/tid/83894	No	Not meeting inclusion criteria	Title and abstract screening
4299	Sharifi H., Ghanei M., Jamaati H., Masjedi M.R., Emami H., Hessami Z., et al. Knowledge, attitude and practice of e-cigarettes among healthcare professionals and smoking cessation volunteers. Minerva Pneumol. 2019;58: 64–69. doi:10.23736/S0026-4954.19.01849-2	No	Not meeting inclusion criteria	Title and abstract screening
4300	Sharifi-Zahabi E, Sharafabad FH, Abdollahzad H, Malekahmadi M, Rad NB. Circulating Advanced Glycation End Products and Their Soluble Receptors in Relation to All-Cause and Cardiovascular Mortality: A Systematic Review and Meta-analysis of Prospective Observational Studies. Adv Nutr. 2021;12: 2157–2171. doi:10.1093/advances/nmab072	No	Not meeting inclusion criteria	Title and abstract screening
4301	Sharma A, Gupta I, Venkatesh U, Singh AK, Golamari R, Arya P. E-cigarettes and myocardial infarction: A systematic review and meta-analysis. Int J Cardiol. 2023;371: 65–70. doi:10.1016/j.ijcard.2022.09.007	No	Not meeting inclusion criteria	Title and abstract screening
4302	Sharma A., Gupta I., Uv V., Golamari R. E-Cigarettes and myocardial Infarction: A systemic review and meta-analysis. Eur J Prev Cardiol. 2021;28: i72. doi:10.1093/eurjpc/zwab061.047	No	Not meeting inclusion criteria	Title and abstract screening

4303	Sharma A., Gupta I., Venkatesh U., Singh A.K., Golamari R., Arya P. E-cigarettes and myocardial infarction: A systematic review and meta-analysis. Int J Cardiol. 2022. doi:10.1016/j.ijcard.2022.09.007	No	Not meeting inclusion criteria	Title and abstract screening
4304	Sharma E, Yang DH, Stroud LR. Variations in Electronic Nicotine Delivery System (ENDS) device types and association with cigarette quit attempts. Prev Med. 2021;148: 106588. doi:10.1016/j.ypmed.2021.106588	No	Not meeting inclusion criteria	Title and abstract screening
4305	Sharma H, Ruikar M. Electronic cigarettes: Ally or an enemy in combating tobacco-use-associated diseases - An integrative review. Indian J Dent Res Off Publ Indian Soc Dent Res. 2023;34: 216–222. doi:10.4103/ijdr.ijdr_890_21	No	Not meeting inclusion criteria	Title and abstract screening
4306	Sharma MK, Suman LN, Srivastava K, Suma N, Vishwakarma A. Psychometric properties of Fagerstrom Test of Nicotine Dependence: A systematic review. Ind Psychiatry J. 2021;30: 207–216. doi:10.4103/ipj.ipj_51_21	No	Not meeting inclusion criteria	Title and abstract screening
4307	Sharma P, Sheikh T, Williams C. Electronic Vaping Product Use Among Adolescents in the Era of the COVID-19 Pandemic: An Updated Scientific Review for Clinicians. WMJ. 2021;120: 205–208.	No	Not meeting inclusion criteria	Title and abstract screening
4308	Sharma R., Dow-Fleisner S., Struik L. Vaping-Specific Regulations as Primary Interventions for Nicotine-Related Cancers: A Scoping Review of Policy at the Federal (Canada), Provincial (British Columbia), and Regional (Okanagan) Levels. Curr Oncol. 2023;30: 6927–6928. doi:10.3390/curroncol30070503	No	Not meeting inclusion criteria	Title and abstract screening
4309	Sharma R., Gartner C.E., Castle D.J., Mendelsohn C.P. Should we encourage smokers with severe mental illness to switch to electronic cigarettes? Aust N Z J Psychiatry. 2017;51: 663–664. doi:10.1177/0004867417697823	No	Not meeting inclusion criteria	Title and abstract screening
4310	Sharma R., Struik L. Quitting Nicotine Use: A Qualitative Comparison of Cessation Experiences of Smokers, Vapers, and Dual Users. Curr Oncol. 2023;30: 6930–6931. doi:10.3390/curroncol30070503	No	Not meeting inclusion criteria	Title and abstract screening
4311	Sharma R., Wigginton B., Meurk C., Ford P., Gartner C.E. Motivations and limitations associated with vaping among people with mental illness: A qualitative analysis of reddit discussions. Int J Environ Res Public Health. 2017;14: 7. doi:10.3390/ijerph14010007	No	Not meeting inclusion criteria	Title and abstract screening
4312	Sharma RH, Rodberg D, Struik LL. Experiences of nicotine users motivated to quit during the COVID-19 pandemic: a secondary qualitative analysis. BMJ Open. 2023;13: e070906. doi:10.1136/bmjopen-2022-070906	No	Not meeting inclusion criteria	Title and abstract screening
4313	Sharma S, Nozohouri S, Vaidya B, Abbruscato T. Repurposing metformin to treat age-related neurodegenerative disorders and ischemic stroke. Life Sci. 2021;274: 119343. doi:10.1016/j.lfs.2021.119343	No	Not meeting inclusion criteria	Title and abstract screening
4314	Sheehan S, Hanna JR, Drury A, McCance T, Semple CJ, O'Neill C. A Systematic Review of Educational Interventions to Equip Health and Social Care Professionals to Promote End-of-Life Supportive Care when a Parent with Dependent Children is Dying with Cancer. Semin Oncol Nurs. 2023;39: N.PAG-N.PAG. doi:10.1016/j.soncn.2023.151474	No	Not meeting inclusion criteria	Title and abstract screening
4315	Sheehan S, Hanna JR, Drury A, McCance T, Semple CJ, O'Neill C. Corrigendum to "A systematic review of educational interventions to equip health and social care professionals to promote end of life supportive care when a parent with dependent children is dying with cancer" [Seminars in Oncology Nursing 39/5 2023 15147. Semin Oncol Nurs. 2023;39: N.PAG-N.PAG. doi:10.1016/j.soncn.2023.151474	No	Not meeting inclusion criteria	Title and abstract screening
4316	Sheffer CE, Stein JS, Petrucci C, Mahoney MC, Johnson S, Giesie P, et al. Tobacco Dependence Treatment in Oncology: Initial Patient Clinical Characteristics and Outcomes from Roswell Park Comprehensive Cancer Center. Int J Env Res Public Health. 2020;17. doi:10.3390/ijerph17113907	No	Not meeting inclusion criteria	Title and abstract screening
4317	Sheffer CE, Shevorykin A, Foulds J, Carl E, Mahoney MC, Bensch L, et al. The Roswell eND scale: Brief, valid assessment of nicotine dependence adults seeking to discontinue e-cigarette use. Drug Alcohol Depend. 2022;243: 109708. doi:10.1016/j.drugalcdep.2022.109708	No	Not meeting inclusion criteria	Title and abstract screening
4318	Sheffer CE, Shevorykin A, Foulds J, Carl E, Mahoney MC, Bensch L, et al. The Roswell eND scale: Brief, valid assessment of nicotine dependence adults seeking to discontinue e-cigarette use. Drug Alcohol Depend. 2023;243: 109708. doi:10.1016/j.drugalcdep.2022.109708	No	Not meeting inclusion criteria	Title and abstract screening
4319	Shehata SA, Toraih EA, Ismail EA, Hagras AM, Elmorsy E, Fawzy MS. Vaping, Environmental Toxicants Exposure, and Lung Cancer Risk. Cancers. 2023;15. doi:10.3390/cancers15184525	No	Not meeting inclusion criteria	Title and abstract screening
4320	Shelton C.M., Black H., Proctor J., Hagemann T.M. A Comprehensive Review of Vaping Use in Pediatric Patients and Recent Changes in Regulatory Laws. J Pediatr Pharmacol Ther. 2022;27: 109–119. doi:10.5863/1551-6776-27.2.109	No	Not meeting inclusion criteria	Title and abstract screening
4321	Shen C., Rafton S., Thakkar R., Ebel B., Rave L., Ulvenes A., et al. Advocacy in Action: Passing Tobacco 21 Legislation in Washington State. Pediatrics. 2020;146: 488–489. doi:10.1542/peds.146.1_MeetingAbstract.488	No	Not meeting inclusion criteria	Title and abstract screening
4322	Shen C, Tannenbaum D, Horn R, Rogers J, Eng C, Zhou S, et al. Overall Survival in Phase 3 Clinical Trials and the Surveillance, Epidemiology, and End Results Database in Patients With Metastatic Colorectal Cancer, 1986-2016: A Systematic Review. JAMA Netw Open. 2022;5: e2213588–e2213588. doi:10.1001/jamanetworkopen.2022.13588	No	Not meeting inclusion criteria	Title and abstract screening
4323	Shepard A., Yearde O., Sanchez J., Amador R., Xirinachs G., Hernandez E., et al. Potential carcinogenic effects of electronic nicotine delivery systems through epigenetic reprogramming in oral, brain, and lung stem cells. Cancer Res. 2023;83. doi:10.1158/1538-7445.AM2023-6584	No	Not meeting inclusion criteria	Title and abstract screening
4324	Shephard A, Dolek S, Barrett SP. Investigating predictors of problematic alcohol, cannabis, and nicotine use among legal users of all three substances. Front Psychiatry. 2023;14: 1110415. doi:10.3389/fpsyt.2023.1110415	No	Not meeting inclusion criteria	Title and abstract screening
4325	Sherafat Y., Bautista M., Fowler C.D. Multidimensional Intersection of Nicotine, Gene Expression, and Behavior. Front Behav Neurosci. 2021;15: 649129. doi:10.3389/fnbeh.2021.649129	No	Not meeting inclusion criteria	Title and abstract screening
4326	Sherman J, Dyar C, McDaniel J, Funderburg NT, Rose KM, Gorr M, et al. Sexual minorities are at elevated risk of cardiovascular disease from a younger age than heterosexuals. J Behav Med. 2022;45: 571–579. doi:10.1007/s10865-021-00269-z	No	Not meeting inclusion criteria	Title and abstract screening
4327	Sherow B, Gacutan J, Tait H, Johnston EL, Clark GF. Land use and COVID-19 lockdowns influence debris composition and abundance in stormwater drains. Sci Total Env. 2023;871: 161908. doi:10.1016/j.scitotenv.2023.161908	No	Not meeting inclusion criteria	Title and abstract screening
4328	Sheth AR, Grewal US, Thotamgari SR, Shi R. Outcomes in patients with primary malignant cardiac tumors: A surveillance, epidemiology, and end results program (SEER) database analysis. J Card Surg. 2021;36: 3463–3465. doi:10.1111/jocs.15725	No	Not meeting inclusion criteria	Title and abstract screening
4329	Shi M, Zhai G. Models for Predicting Early Death in Patients With Stage IV Esophageal Cancer: A Surveillance, Epidemiology, and End Results-Based Cohort Study. Cancer Control J Moffitt Cancer Cent. 2022;29: 1–11. doi:10.1177/10732748211072976	No	Not meeting inclusion criteria	Title and abstract screening
4330	Shi R, Feldman R, Liu J, Clark PI. The Dilemma of Correcting Nicotine Misperceptions: Nicotine Replacement Therapy versus Electronic Cigarettes. Health Commun. 2021;36: 1856–1866. doi:10.1080/10410236.2020.1800288	No	Not meeting inclusion criteria	Title and abstract screening
4331	Shiffman S, Augustson EM. Introduction to the Special Issue on JUUL Use. Am J Health Behav. 2021;45: 397–401. doi:10.5993/AJHB.45.3.1	No	Not meeting inclusion criteria	Title and abstract screening

4332	Shiffman S, Scholl SM, Mao JM. Very-low-nicotine-content cigarettes and dependence among non-daily smokers. Drug Alcohol Depend. 2019;197: 1–7. doi:10.1016/j.drugalcdep.2018.12.021	No	Not meeting inclusion criteria	Title and abstract screening
4333	Shih C.-S., Etter J.-F. Public attitudes towards e-cigarette regulations and policies in Taiwan. J Public Health Res. 2020;9: 458–462. doi:10.4081/jphr.2020.1698	No	Not meeting inclusion criteria	Title and abstract screening
4334	Shimosato M, Sakane N. Oral dryness and moisture degree at the lingual but not buccal mucosa predict prognosis in end-of-life cancer patients. Support Care Cancer. 2021;29: 6289–6296. doi:10.1007/s00520-021-06212-x	No	Not meeting inclusion criteria	Title and abstract screening
4335	Shin DW, Kim YI, Kim SJ, Kim JS, Chong S, Park YS, et al. Lung cancer specialist physicians' attitudes towards e-cigarettes: A nationwide survey. PLoS One. 2017;12: e0172568. doi:10.1371/journal.pone.0172568	No	Not meeting inclusion criteria	Title and abstract screening
4336	Shin SH. Preventing E-cigarette use among high-risk adolescents: A trauma-informed prevention approach. Addict Behav. 2021;115: 106795. doi:10.1016/j.addbeh.2020.106795	No	Not meeting inclusion criteria	Title and abstract screening
4337	Shin SH, Conley D, Ksinan Jiskrova G, Wills TA. Adverse Childhood Experiences and E-Cigarette Use During Young Adulthood. Am J Addict. 2019;28: 303–310. doi:10.1111/ajad.12890	No	Not meeting inclusion criteria	Title and abstract screening
4338	Shin YM, Hunt DP, Akwe J. An Epidemic Supplanted by a Pandemic: Vaping-Related Illness and COVID-19. South Med J. 2022;115: 8–12. doi:10.14423/SMJ.0000000000001344	No	Not meeting inclusion criteria	Title and abstract screening
4339	Shipman JG, Onyenwoke RU, Sivaraman V. Calcium-Dependent Pulmonary Inflammation and Pharmacological Interventions and Mediators. Biol Basel. 2021;10. doi:10.3390/biology10101053	No	Not meeting inclusion criteria	Title and abstract screening
4340	Shirazian S, Starakiewicz P, Latcha S. Cancer Screening in End-Stage Kidney Disease. Adv Chronic Kidney Dis. 2021;28: 502–502. doi:10.1053/j.ackd.2021.09.006	No	Not meeting inclusion criteria	Title and abstract screening
4341	Shitole SG, Lazar JM, Taub CC, Furlani AC, Konkle-Parker DJ, Dionne-Odom J, et al. Human Immunodeficiency Virus and Cardiac End-Organ Damage in Women: Findings From an Echocardiographic Study Across the United States. Clin Infect Dis. 2023;76: 210–219. doi:10.1093/cid/ciac795	No	Not meeting inclusion criteria	Title and abstract screening
4342	Shittu AAT, Kumar BP, Okafor U, Berkelhamer SK, Goniewicz ML, Wen X. Changes in e-cigarette and cigarette use during pregnancy and their association with small-for-gestational-age birth. Am J Obstet Gynecol. 2022;226: 730.e1-730.e10. doi:10.1016/j.ajog.2021.11.1354	No	Not meeting inclusion criteria	Title and abstract screening
4343	Shmulewitz D, Greenstein E, Stohl M, Fink DS, Roncone S, Walsh C, et al. Validity of the DSM-5 tobacco use disorder diagnostics in adults with problematic substance use. Drug Alcohol Depend. 2022;234: 109411. doi:10.1016/j.drugalcdep.2022.109411	No	Not meeting inclusion criteria	Title and abstract screening
4344	Shore BJ, Flaugh R, Shannon BA, Curran P, Hogue G. Preoperative Considerations for Teenagers Undergoing Orthopaedic Surgery: VTE Prevention, Mental Health Assessment, Vaping, and Drug Addiction. J Pediatr Orthop. 2021;41: S64–S69. doi:10.1097/BPO.0000000000001764	No	Not meeting inclusion criteria	Title and abstract screening
4345	Short M., Cole A.G. Factors associated with e-cigarette escalation among high school students: A review of the literature. Int J Environ Res Public Health. 2021;18: 10067. doi:10.3390/ijerph181910067	No	Not meeting inclusion criteria	Title and abstract screening
4346	Shroff S.M., Gunjal S., Sreerramareddy C.T. P1.01-08 A Content Analysis E-cigarette Retailer Websites and Nicotine Addiction among E-cigarette Users-Mixed Methods Study from Malaysia. J Thorac Oncol. 2023;18: S185. doi:10.1016/j.jtho.2023.09.286	No	Not meeting inclusion criteria	Title and abstract screening
4347	Shuter J., Reddy K.P., Hyle E.P., Stanton C.A., Rigotti N.A. Harm reduction for smokers living with HIV. Lancet HIV. 2021;8: e652–e658. doi:10.1016/S2352-3018%2821%2900156-9	No	Not meeting inclusion criteria	Title and abstract screening
4348	Shuter J, Yang A, Kim RS, Brownstein JS. Interest in Tobacco Treatment Delivered During Rideshare Travel. Mayo Clin Proc Innov Qual Outcomes. 2019;3: 461–465. doi:10.1016/j.mayocpiqo.2019.07.005	No	Not meeting inclusion criteria	Title and abstract screening
4349	Sidani JE, Hoffman BL, Colditz JB, Melcher E, Taneja SB, Shensa A, et al. E-Cigarette-Related Nicotine Misinformation on Social Media. Subst Use Misuse. 2022;57: 588–594. doi:10.1080/10826084.2022.2026963	No	Not meeting inclusion criteria	Title and abstract screening
4350	Sidani JE, Colditz JB, Barrett EL, Shensa A, Chu KH, James AE, et al. I wake up and hit the JUUL: Analyzing Twitter for JUUL nicotine effects and dependence. Drug Alcohol Depend. 2019;204: 107500. doi:10.1016/j.drugalcdep.2019.06.005	No	Not meeting inclusion criteria	Title and abstract screening
4351	Siddiqi TJ, Rashid AM, Siddiqi AK, Anwer A, Usman MS, Sakhi H, et al. Association of Electronic Cigarette Exposure on Cardiovascular Health: A Systematic Review and Meta-Analysis. Curr Probl Cardiol. 2023;48: 101748. doi:10.1016/j.cpcardiol.2023.101748	No	Not meeting inclusion criteria	Title and abstract screening
4352	Sidhu NK, Lechner WV, Cwalina SN, Whitted L, Smiley SL, Barrington-Trimis JL, et al. Adolescent and Young Adult Response to Hypothetical E-Liquid Flavor Restrictions. J Stud Alcohol Drugs. 2023;84: 303–308. doi:10.15288/jsad.21-00466	No	Not meeting inclusion criteria	Title and abstract screening
4353	Siegel J, Patel SH, Mankaliye B, Raval AP. Impact of Electronic Cigarette Vaping on Cerebral Ischemia: What We Know So Far. Transl Stroke Res. 2022;13: 923–938. doi:10.1007/s12975-022-01011-w	No	Not meeting inclusion criteria	Title and abstract screening
4354	Siegel S.D., Lerman C., Flitter A., Schnoll R.A. The use of the nicotine metabolite ratio as a biomarker to personalize smoking cessation treatment: Current evidence and future directions. Cancer Prev Res (Phila Pa). 2020;13: 261–272. doi:10.1158/1940-6207.CAPR-19-0259	No	Not meeting inclusion criteria	Title and abstract screening
4355	Sieluk J, Yang L, Haiderali A, Huang M, Hirshfield KM. Systemic therapy, survival and end-of-life costs for metastatic triple-negative breast cancer: retrospective SEER-Medicare study of women age ≥65 years. Future Oncol. 2021;17: 2581–2592. doi:10.2217/fon-2021-0019	No	Not meeting inclusion criteria	Title and abstract screening
4356	Sikic A, Frie JA, Khokhar JY, Murray JE. Sex Differences in the Behavioural Outcomes of Prenatal Nicotine and Tobacco Exposure. Front Neurosci. 2022;16: 921429. doi:10.3389/fnins.2022.921429	No	Not meeting inclusion criteria	Title and abstract screening
4357	Sikka G., Schreiber R., Galiatsatos P. Management of electronic cigarette addiction in youth and young adults: A brief case series. Am J Respir Crit Care Med. 2021;203. doi:10.1164/ajrccm-conference.2021.203.1_MeetingAbstracts.A3010	No	Not meeting inclusion criteria	Title and abstract screening
4358	Sikka G, Oluyinka M, Schreiber R, Galiatsatos P. Electronic Cigarette Cessation in Youth and Young Adults: A Case Series. Tob Use Insights. 2021;14: 1179173X211026676. doi:10.1177/1179173X211026676	No	Not meeting inclusion criteria	Title and abstract screening
4359	Silva ALOD, Moreira JC. Why electronic cigarettes are a public health threat? Cad Saude Publica. 2019;35: e00246818. doi:10.1590/0102-311X00246818	No	Not meeting inclusion criteria	Title and abstract screening
4360	Silva CP, Maggs JL, Kelly BC, Vuolo M, Staff J. Associations Between E-Cigarettes and Subsequent Cocaine Use in Adolescence: An Analysis of the UK Millennium Cohort Study. Nicotine Tob Res Off J Soc Res Nicotine Tob. 2022. doi:10.1093/ntr/ntac219	No	Not meeting inclusion criteria	Title and abstract screening

4361	Silva CP, Maggs JL, Kelly BC, Vuolo M, Staff J. Associations between e-cigarettes and subsequent cocaine use in adolescence: An analysis of the UK Millennium Cohort Study. Nicotine Tob Res. 2023;25: 514–523. doi:10.1093/ntr/ntac219	No	Not meeting inclusion criteria	Title and abstract screening
4362	Silva L, Bezzo FB, van Ham M. Covid-19 restrictions: An opportunity to highlight the effect of neighbourhood deprivation on individuals' health-related behaviours. Soc Sci Med. 2023;325: 115917. doi:10.1016/j.socscimed.2023.115917	No	Not meeting inclusion criteria	Title and abstract screening
4363	Silveira ML, Conway KP, Green VR, Kasza KA, Sargent JD, Borek N, et al. Longitudinal associations between youth tobacco and substance use in waves 1 and 2 of the Population Assessment of Tobacco and Health (PATH) Study. Drug Alcohol Depend. 2018;191: 25–36. doi:10.1016/j.drugalcdep.2018.06.018	No	Not meeting inclusion criteria	Title and abstract screening
4364	Silveira ML, Everard CD, Sharma E, Lauten K, Alexandridis AA, Duffy K, et al. Tobacco Use and Incidence of Adverse Oral Health Outcomes Among US Adults in the Population Assessment of Tobacco and Health Study. JAMA Netw Open. 2022;5: e2245909. doi:10.1001/jamanetworkopen.2022.45909	No	Not meeting inclusion criteria	Title and abstract screening
4365	Silver N, Kierstead E, Kostygina G, Tran H, Briggs J, Emery S, et al. The Influence of Provaping "Gatewatchers" on the Dissemination of COVID-19 Misinformation on Twitter: Analysis of Twitter Discourse Regarding Nicotine and the COVID-19 Pandemic. J Med Internet Res. 2022;24: e40331. doi:10.2196/40331	No	Not meeting inclusion criteria	Title and abstract screening
4366	Silver N, Kierstead E, Tran B, Sparrock L, Vallone D, Schillo B. Belief and Recall of Nicotine as Therapeutic for COVID-19 May Undermine E-Cigarette Quitting Behavior. Health Educ Behav. 2022;49: 929–933. doi:10.1177/10901981221109127	No	Not meeting inclusion criteria	Title and abstract screening
4367	Silver NA, Kierstead EC, Briggs J, Schillo B. Charming e-cigarette users with distorted science: a survey examining social media platform use, nicotine-related misinformation and attitudes towards the tobacco industry. BMJ Open. 2022;12: e057027. doi:10.1136/bmjopen-2021-057027	No	Not meeting inclusion criteria	Title and abstract screening
4368	Simeone S., Rasheed A. Post-COVID-19 Fibrosis Presenting with Usual Interstitial Pattern. Am J Respir Crit Care Med. 2022;205. doi:10.1164/ajrccm-conference.2022.205.1_MeetingAbstracts.A4211	No	Not meeting inclusion criteria	Title and abstract screening
4369	Simms L., Moreau M., Trelles Sticken E., Wieczorek R., Pour S.J., Chapman F., et al. P14-13: Use of Quantitative In Vitro to In Vivo Extrapolation (QIVIVE) for the assessment of Non-Combustible Next Generation products. Toxicol Lett. 2023;384: S171. doi:10.1016/S0378-4274%2823%2900670-7	No	Not meeting inclusion criteria	Title and abstract screening
4370	Simon P, Jiang Y, Buta E, Sartor CE, Krishnan-Sarin S, Gueorguieva R. Longitudinal Trajectories of Multiple Nicotine Product Use Among Youths in the Population Assessment of Tobacco and Health Study. JAMA Netw Open. 2022;5: e223549. doi:10.1001/jamanetworkopen.2022.3549	No	Not meeting inclusion criteria	Title and abstract screening
4371	Simonavicius E, McNeill A, Arnott D, Brose LS. What factors are associated with current smokers using or stopping e-cigarette use? Drug Alcohol Depend. 2017;173: 139–143. doi:10.1016/j.drugalcdep.2017.01.002	No	Not meeting inclusion criteria	Title and abstract screening
4372	Simpson KA, Kechter A, Schiff SJ, Braymiller JL, Yamaguchi N, Ceasar RC, et al. Characterizing symptoms of e-cigarette dependence: a qualitative study of young adults. BMC Public Health. 2021;21: 959. doi:10.1186/s12889-021-10945-z	No	Not meeting inclusion criteria	Title and abstract screening
4373	Sindelar J.L. Regulating vaping - Policies, possibilities, and perils. N Engl J Med. 2020;382: e54. doi:10.1056/NEJMp1917065	No	Not meeting inclusion criteria	Title and abstract screening
4374	SINDU D., MOIN A., MOHAMED H.E., WALIA R., ARJUNA A. HIDDEN EXPLANT CANCERS IN LUNG TRANSPLANT RECIPIENTS. Chest. 2023;164: A6464–A6465. doi:10.1016/j.chest.2023.07.4168	No	Not meeting inclusion criteria	Title and abstract screening
4375	Singer JM, Keller-Hamilton B, Roberts ME, Klein EG, Ferketich AK. Geographic Differences in JUUL Use and Risk Perceptions. Subst Use Misuse. 2022;57: 1918–1922. doi:10.1080/10826084.2022.2120360	No	Not meeting inclusion criteria	Title and abstract screening
4376	Singh A. A commentary on adolescent electronic cigarette use and nicotine addiction. Pediatr Pulmonol. 2021;56: 3580–3585. doi:10.1002/ppul.25676	No	Not meeting inclusion criteria	Title and abstract screening
4377	Singh A. Adolescent e-cigarette or vaping product use-associated lung injury: A case series and review of the literature. Pediatr Pulmonol. 2022;57: 1076–1084. doi:10.1002/ppul.25832	No	Not meeting inclusion criteria	Title and abstract screening
4378	Singh A., Chaturvedi P. Misleading evidence of electronic cigarettes efficacy for tobacco cessation. Indian J Med Paediatr Oncol. 2020;41: 319–320. doi:10.4103/ijmpo.ijmpo_89_20	No	Not meeting inclusion criteria	Title and abstract screening
4379	Singh AV, Maharjan RS, Kromer C, Laux P, Luch A, Vats T, et al. Advances in Smoking Related In Vitro Inhalation Toxicology: A Perspective Case of Challenges and Opportunities from Progresses in Lung-on-Chip Technologies. Chem Res Toxicol. 2021;34: 1984–2002. doi:10.1021/acs.chemrestox.1c00219	No	Not meeting inclusion criteria	Title and abstract screening
4380	Singh B., Hrywna M., Wackowski O.A., Delnevo C.D., Jane Lewis M., Steinberg M.B. Knowledge, recommendation, and beliefs of e-cigarettes among physicians involved in tobacco cessation: A qualitative study. Prev Med Rep. 2017;8: 25–29. doi:10.1016/j.pmedr.2017.07.012	No	Not meeting inclusion criteria	Title and abstract screening
4381	Singh B. Communication of e-cigarettes among providers and their adolescent patients: A mixed method study. Diss Abstr Int Sect B Sci Eng. 2022;83: No-Specified.	No	Not meeting inclusion criteria	Title and abstract screening
4382	Singh I, Valavil Punnapuzha V, Mitsakakis N, Fu R, Chaiton M. A Machine Learning Approach Reveals Distinct Predictors of Vaping Dependence for Adolescent Daily and Non-Daily Vapers in the COVID-19 Era. Healthc Basel Switz. 2023;11. doi:10.3390/healthcare11101465	No	Not meeting inclusion criteria	Title and abstract screening
4383	Singh PN, Moses O, Shih W, Hubbard M. Cohort profile for the Loma Linda University Health BREATHE programme: a model to study continuously incentivised employee smoking cessation. BMJ Open. 2022;12: e053303. doi:10.1136/bmjopen-2021-053303	No	Not meeting inclusion criteria	Title and abstract screening
4384	Singh S, Molina E, Perraillon M, Fischer SM. Post-Acute Care Outcomes of Cancer Patients <65 Reveal Disparities in Care Near the End of Life. J Palliat Med. 2023;26: 1081–1089. doi:10.1089/jpm.2022.0190	No	Not meeting inclusion criteria	Title and abstract screening
4385	Singhal S, Degano C, Berenbaum E, Keller-Olaman S. Does Vaping Increase the Risk of COVID-19 Transmission and Make Individuals Who Vape Susceptible to Infection and Prone to Severe Illness? A Review. J Can Dent Assoc. 2022;88: m1.	No	Not meeting inclusion criteria	Title and abstract screening
4386	Siqueira LM. Nicotine and Tobacco as Substances of Abuse in Children and Adolescents. Pediatrics. 2017;139. doi:10.1542/peds.2016-3436	No	Not meeting inclusion criteria	Title and abstract screening
4387	Siqueira LM, COMMITTEE ON SUBSTANCE USE AND PREVENTION. Nicotine and Tobacco as Substances of Abuse in Children and Adolescents. Pediatrics. 2017;139. doi:10.1542/peds.2016-3436	No	Not meeting inclusion criteria	Title and abstract screening
4388	Sirigaddi K., Greenspan R., Khan A., Mann M. A CASE OF VAPING-RELATED LUNG INJURY MIMICKING COVID-19. Chest. 2021;160: A2446. doi:10.1016/j.chest.2021.07.2113	No	Not meeting inclusion criteria	Title and abstract screening
4389	Siripongvutikorn Y, Tabuchi T, Okawa S. Workplace smoke-free policies that allow heated tobacco products and electronic cigarettes use are associated with use of both these products and conventional tobacco smoking: the 2018 JASTIS study. Tob Control. 2021;30: 147–154. doi:10.1136/tobaccocontrol-2019-055465	No	Not meeting inclusion criteria	Title and abstract screening

4390	Sisk BA, Keenan MA, Schulz GL, Bakitas M, Currie ER, Gilbertson-White S, et al. Bereaved Caregivers Perspectives of Negative Communication Experiences Near the End of Life for Adolescents and Young Adults with Cancer. J Adolesc Young Adult Oncol. 2022;11: 498–505. doi:10.1089/jayao.2021.0154	No	Not meeting inclusion criteria	Title and abstract screening
4391	Sissung TM, Figg WD. Commentary: Electronic Nicotine Delivery Systems: Seeing Through the Clouds. Fam Community Health. 2022;45: 202–205. doi:10.1097/FCH.0000000000000324	No	Not meeting inclusion criteria	Title and abstract screening
4392	Sitas F, Short G, Brooke M, Griffiths K. Smoking and vaping on death notification forms in Australia: Improving the reporting of preventable risk factors. Health Promot J Austr. 2023;34: 723–724. doi:10.1002/hpja.703	No	Not meeting inclusion criteria	Title and abstract screening
4393	Siudak Z., Krawczyk-Ozog A., Twarda I., Franczak I., Rajtar-Salwa R., Bartus S., et al. "Heart without smoke" educational campaign - The role of patient education in secondary prevention of cardiovascular disease. Kardiol Pol. 2018;76: 125–129. doi:10.5603/KP.a2017.0167	No	Not meeting inclusion criteria	Title and abstract screening
4394	Sivabalah K., Crane D., Coyne J., Hancock M., Neville S., Crossfield A., et al. AN INNOVATIVE DIGITAL APPROACH TO SUPPORT NHS STAFF TO STOP SMOKING ACROSS AN INTEGRATED CARE SYSTEM. Thorax. 2023;78: A54–A55. doi:10.1136/thorax-2023-BTSabstracts.80	No	Not meeting inclusion criteria	Title and abstract screening
4395	Skelton E, Guillaumier A, Tzelepis F, Walsberger S, Paul CL, Dunlop AJ, et al. Alcohol and other drug health-care providers and their client's perceptions of e-cigarette use, safety and harm reduction. Drug Alcohol Rev. 2021;40: 998–1002. doi:10.1111/dar.13276	No	Not meeting inclusion criteria	Title and abstract screening
4396	Skelton E, Lum A, Cooper LE, Barnett E, Smith J, Everson A, et al. Addressing smoking in sheltered homelessness with intensive smoking treatment (ASSIST project): A pilot feasibility study of varenicline, combination nicotine replacement therapy and motivational interviewing. Addict Behav. 2022;124: 107074. doi:10.1016/j.addbeh.2021.107074	No	Not meeting inclusion criteria	Title and abstract screening
4397	Skelton E, Lum A, Robinson M, Dunlop A, Guillaumier A, Baker A, et al. A pilot randomised controlled trial of abrupt versus gradual smoking cessation in combination with vaporised nicotine products for people receiving alcohol and other drug treatment. Addict Behav. 2022;131: 107328. doi:10.1016/j.addbeh.2022.107328	No	Not meeting inclusion criteria	Title and abstract screening
4398	Skelton E, Silberberg L, Guillaumier A, Dunlop AJ, Wilkinson RB, Bonevski B. Electronic cigarettes: Ever use, current use and attitudes among alcohol and other drug clients. Drug Alcohol Rev. 2020;39: 7–11. doi:10.1111/dar.13005	No	Not meeting inclusion criteria	Title and abstract screening
4399	Skjodt N., Wang B., Li D., Fiselier A., Rodriguez-Juarez R., Kovalchuk O., et al. Modelling the molecular effects of cannabis extracts on human airway epithelial tissue. Eur Respir J. 2021;58. doi:10.1183/13993003.congress-2021.PA2042	No	Not meeting inclusion criteria	Title and abstract screening
4400	Sklenarik S., Lehane M., Bouchard S., Potenza M., Astur R. Using virtual reality to reduce problematic gambling in young adults. J Behav Addict. 2022;11: 77. doi:10.1556/2006.2022.00700	No	Not meeting inclusion criteria	Title and abstract screening
4401	Skvortsova E.S., Mamchenko M.M. Smoking electronic cigarettes as a medical and social problem. Profil Meditsina. 2021;24: 89–94. doi:10.17116/PROFMED20212408189	No	Not meeting inclusion criteria	Title and abstract screening
4402	Sledge GWJr. Musings of a Cancer Doctor: When Plagues End. Oncol Times. 2022;44: 17–18. doi:10.1097/01.COT.0000824700.30925.93	No	Not meeting inclusion criteria	Title and abstract screening
4403	Sleiman M., Tercyak K., Yockel M.R. Electronic nicotine delivery system (ENDS) use among LatinX youth in low-resource communities: Implications for policy and regulation. Cancer Res. 2023;83. doi:10.1158/1538-7445.AM2023-LB052	No	Not meeting inclusion criteria	Title and abstract screening
4404	Sly J.R., Miller S.J., Li Y., Bolutayo K., Jandorf L. Low-dose computed tomography lung cancer screening as a teachable moment for smoking cessation among African American smokers: A feasibility study. J Psychosoc Oncol. 2018;36: 784–792. doi:10.1080/07347332.2018.1499693	No	Not meeting inclusion criteria	Title and abstract screening
4405	Smadhi H., Khalfallah I., Megdiche M.L. What is the doctors' attitude towards tobacco? Eur Respir J. 2017;50. doi:10.1183/1393003.congress-2017.PA1262	No	Not meeting inclusion criteria	Title and abstract screening
4406	Smeds MR, Privratsky A, Thrush CR, Barone CP, Escobar GA, Ali AT, et al. Nicotine Dependence and Willingness to Quit Smoking in Vascular Surgery Patients. Ann Vasc Surg. 2017;45: 144–153. doi:10.1016/j.avsg.2017.06.049	No	Not meeting inclusion criteria	Title and abstract screening
4407	Smethells JR, Burroughs D, Saykao A, Pentel PR, Rezvani AH, LeSage MG. The reinforcement threshold and elasticity of demand for nicotine in an adolescent rat model of depression. Drug Alcohol Depend. 2021;219: 108433. doi:10.1016/j.drugalcdep.2020.108433	No	Not meeting inclusion criteria	Title and abstract screening
4408	Smets J., Baeyens F., Chaumont M., Adriaens K., Van Gucht D. When less is more: Vaping low-nicotine vs. high-nicotine E-liquid is compensated by increased wattage and higher liquid consumption. Int J Environ Res Public Health. 2019;16: 723. doi:10.3390/ijerph16050723	No	Not meeting inclusion criteria	Title and abstract screening
4409	Smiley SL, Shin H, Rose SW, Rodriguez YL, Barahona R, Baezconde-Garbanati L. Perceptions of E-cigarettes and Flavor Restrictions among Tobacco Retailers in Los Angeles. Am J Health Behav. 2020;44: 893–901. doi:10.5993/AJHB.44.6.12	No	Not meeting inclusion criteria	Title and abstract screening
4410	Smit T, Garey L, Langdon KJ, Ditre JW, Rogers AH, Orr MF, et al. Differential effect of sex on pain severity and smoking behavior and processes. Addict Behav. 2019;90: 229–235. doi:10.1016/j.addbeh.2018.11.007	No	Not meeting inclusion criteria	Title and abstract screening
4411	Smit T, Olofsson H, Nizio P, Garey L, Zvolensky MJ. Pain severity and e-cigarette health literacy: the moderating role of sex. Subst Abuse. 2019;13: 1178221819897070. doi:10.1177/1178221819897070	No	Not meeting inclusion criteria	Title and abstract screening
4412	Smith C.A., McNeill A., Kock L., Ahmed Z., Shahab L. Mental health professionals' perceptions, judgements and decision-making practices regarding the use of electronic cigarettes as a tobacco harm reduction intervention in mental healthcare: A qualitative focus group study. Addict Behav Rep. 2019;10: 100184. doi:10.1016/j.abrep.2019.100184	No	Not meeting inclusion criteria	Title and abstract screening
4413	Smith CE. New e-cigarettes and metabolic outcomes. Diss Abstr Int Sect B Sci Eng. 2022;83: No-Specified.	No	Not meeting inclusion criteria	Title and abstract screening
4414	Smith CG, Chen Q, Huang B, Miller RW, DeSimone CP, Dietrich 3rd CS, et al. The Log Odds of Positive Lymph Nodes Predict Survival of Advanced-Stage Endometrial Cancer: A Retrospective Analysis of 3230 Patients in the Surveillance, Epidemiology, and End Results Database. J Gynecol Surg. 2022;38: 278–286. doi:10.1089/gyn.2021.0128	No	Not meeting inclusion criteria	Title and abstract screening
4415	Smith D.M., Gawron M., Balwicki L., Sobczak A., Matynia M., Goniewicz M.L. Exclusive versus dual use of tobacco and electronic cigarettes among adolescents in Poland, 2010-2016. Addict Behav. 2019;90: 341–348. doi:10.1016/j.addbeh.2018.11.035	No	Not meeting inclusion criteria	Title and abstract screening
4416	Smith DM, Hyland A, Kozlowski L, O'Connor RJ, Collins RL. Use of Inhaled Nicotine and Cannabis Products among Adults Who Vape Both Substances. Subst Use Misuse. 2022;57: 432–441. doi:10.1080/10826084.2021.2019773	No	Not meeting inclusion criteria	Title and abstract screening
4417	Smith DM, Kozlowski L, O'Connor RJ, Hyland A, Collins RL. Reasons for individual and concurrent use of vaped nicotine and cannabis: their similarities, differences, and association with product use. J Cannabis Res. 2021;3: 39. doi:10.1186/s42238-021-00097-7	No	Not meeting inclusion criteria	Title and abstract screening
4418	Smith H, Lucherini M, Amos A, Hill S. The emerging norms of e-cigarette use among adolescents: A meta-ethnography of qualitative evidence. Int J Drug Policy. 2021;94: 103227. doi:10.1016/j.drugpo.2021.103227	No	Not meeting inclusion criteria	Title and abstract screening

4419	Smith J., Togawa K., Dresler C., Hawari F., Zain Z.M., Stewart B., et al. Smoking cessation after a cancer diagnosis: Commentary on special supplement in Cancer Epidemiology. Cancer Epidemiol. 2022;79: 102210. doi:10.1016/j.canep.2022.102210	No	Not meeting inclusion criteria	Title and abstract screening
4420	Smith L.M., Boehm L., Strang L.V., DeMarre C., Marcyjanik D. Targeted Education for School Staff on Electronic Nicotine Delivery Systems: A Nurse Led Intervention. J Sch Nurs Off Publ Natl Assoc Sch Nurses. 2021; 10598405211043127. doi:10.1177/10598405211043127	No	Not meeting inclusion criteria	Title and abstract screening
4421	Smith LC. Immunopharmacology and behavioral pharmacology of opioids and nicotine. Diss Abstr Int Sect B Sci Eng. 2022;83: No-Specified.	No	Not meeting inclusion criteria	Title and abstract screening
4422	Smith LC, George O. Advances in smoking cessation pharmacotherapy: Non-nicotinic approaches in animal models. Neuropharmacology. 2020;178: 108225. doi:10.1016/j.neuropharm.2020.108225	No	Not meeting inclusion criteria	Title and abstract screening
4423	Smith M.J., Hilton S. Adolescents' views on user-generated content and influencer marketing of e-cigarettes on social media: a focus group study. The Lancet. 2022;400: S77. doi:10.1016/S0140-6736%2822%2902287-5	No	Not meeting inclusion criteria	Title and abstract screening
4424	Smith MJ, Buckton C, Patterson C, Hilton S. User-generated content and influencer marketing involving e-cigarettes on social media: a scoping review and content analysis of YouTube and Instagram. BMC Public Health. 2023;23: 530. doi:10.1186/s12889-023-15389-1	No	Not meeting inclusion criteria	Title and abstract screening
4425	Smith MR, Jarrell ZR, Orr M, Liu KH, Go Y-M, Jones DP. Metabolome-wide association study of flavorant vanillin exposure in bronchial epithelial cells reveals disease-related perturbations in metabolism. Environ Int. 2021;147: 106323. doi:10.1016/j.envint.2020.106323	No	Not meeting inclusion criteria	Title and abstract screening
4426	Smith MJ, Hilton S. Adolescents' views on user-generated content and influencer marketing of e-cigarettes on social media: a focus group study. Lancet. 2022;400 Suppl 1: S77. doi:10.1016/S0140-6736(22)02287-5	No	Not meeting inclusion criteria	Title and abstract screening
4427	Smith ML, Herbert A, Hughes A, Northstone K, Howe LD. Socioeconomic position and adverse childhood experiences as risk factors for health-related behaviour change and employment adversity during the COVID-19 pandemic: insights from a prospective cohort study in the UK. BMC Public Health. 2022;22: 1820. doi:10.1186/s12889-022-14184-8	No	Not meeting inclusion criteria	Title and abstract screening
4428	Smith N., Malarkey S., Valera P., Alzate-Duque L., Baquerizo H. A health and justice lab to address cancer related health disparities and social determinants of health in marginalized communities: The Cancer Health Justice Lab. Cancer Res. 2021;81. doi:10.1158/1538-7445.AM2021-2554	No	Not meeting inclusion criteria	Title and abstract screening
4429	Smith P., Daniel R., Murray R.L., Moore G., Nelson A., Brain K. Psychosocial determinants of quit motivation in older smokers from deprived backgrounds: A cross-sectional survey. BMJ Open. 2021;11: e044815. doi:10.1136/bmjopen-2020-044815	No	Not meeting inclusion criteria	Title and abstract screening
4430	Smith PH, Ward RM, Bartoszek LA, Branscum PW. College students' patterns of electronic nicotine delivery system use and other substance use. J Am Coll Health. 2022;70: 1882–1888. doi:10.1080/07448481.2020.1841210	No	Not meeting inclusion criteria	Title and abstract screening
4431	Smith T.T., Koopmeiners J.S., Tessier K.M., Davis E.M., Conklin C.A., Denlinger-Apte R.L., et al. Randomized Trial of Low-Nicotine Cigarettes and Transdermal Nicotine. Am J Prev Med. 2019;57: 515–524. doi:10.1016/j.amepre.2019.05.010	No	Not meeting inclusion criteria	Title and abstract screening
4432	Smith TT, Hatsukami DK, Benowitz NL, Colby SM, McClernon FJ, Strasser AA, et al. Whether to push or pull? Nicotine reduction and non-combusted alternatives - Two strategies for reducing smoking and improving public health. Prev Med. 2018;117: 8–14. doi:10.1016/j.ypmed.2018.03.021	No	Not meeting inclusion criteria	Title and abstract screening
4433	Smith TT, Nahhas GJ, Borland R, Cho YJ, Chung-Hall J, Fairman RT, et al. Which tobacco control policies do smokers support? Findings from the International Tobacco Control Four Country Smoking and Vaping Survey. Prev Med. 2021;149: 106600. doi:10.1016/j.ypmed.2021.106600	No	Not meeting inclusion criteria	Title and abstract screening
4434	Smith TT, Rupprecht LE, Denlinger-Apte RL, Weeks JJ, Panas RS, Donny EC, et al. Animal Research on Nicotine Reduction: Current Evidence and Research Gaps. Nicotine Tob Res. 2017;19: 1005–1015. doi:10.1093/ntr/ntx077	No	Not meeting inclusion criteria	Title and abstract screening
4435	Smith TT, Wahlquist AE, Heckman BW, Cummings KM, Carpenter MJ. Impact of E-cigarette Sampling on Cigarette Dependence and Reinforcement Value. Nicotine Tob Res. 2020;22: 297–301. doi:10.1093/ntr/nty258	No	Not meeting inclusion criteria	Title and abstract screening
4436	Smith V., Ullah F., Wall G. Survival after intentional ingestion of highly concentrated, bulk nicotine solution. Clin Toxicol. 2021;59: 1281. doi:10.1080/15563650.2021.1921789	No	Not meeting inclusion criteria	Title and abstract screening
4437	Snell L.M., Colby S.M., Deatley T., Cassidy R., Tidey J.W. Associations Between Nicotine Knowledge and Smoking Cessation Behaviors Among US Adults Who Smoke. Nicotine Tob Res. 2022;24: 855–863. doi:10.1093/ntr/ntab246	No	Not meeting inclusion criteria	Title and abstract screening
4438	Snell M, Harless D, Shin S, Cunningham P, Barnes A. A longitudinal assessment of nicotine dependence, mental health, and attempts to quit Smoking: Evidence from waves 1-4 of the Population Assessment of Tobacco and Health (PATH) study. Addict Behav. 2021;115: 106787. doi:10.1016/j.addbeh.2020.106787	No	Not meeting inclusion criteria	Title and abstract screening
4439	Snelling S, Yong HH, Kasza K, Borland R. Does alcohol consumption elevate smoking relapse risk of people who used to smoke? Differences by duration of smoking abstinence. J Subst Use Addict Treat. 2023;159: 209260. doi:10.1016/j.josat.2023.209260	No	Not meeting inclusion criteria	Title and abstract screening
4440	Snoderly HT, Nurkiewicz TR, Bowdridge EC, Bennewitz MF. E-Cigarette Use: Device Market, Study Design, and Emerging Evidence of Biological Consequences. Int J Mol Sci. 2021;22. doi:10.3390/ijms222212452	No	Not meeting inclusion criteria	Title and abstract screening
4441	Snow D. Nicotine Addiction and E-Cigarettes. J Addict Nurs. 2020;31: 77–78. doi:10.1097/JAN.0000000000000331	No	Not meeting inclusion criteria	Title and abstract screening
4442	Snowden E., Godfrey A. DABBING LUNG INJURY MANAGED BY VV ECMO. Crit Care Med. 2023;51: 456. doi:10.1097/01.ccm.0000909436.86276.83	No	Not meeting inclusion criteria	Title and abstract screening
4443	Soares LO, Da Silva Celestino C, Jardim BG, Werneck JT, Picciani BLS, Da Costa Fontes KBF, et al. THE USE OF ELECTRONIC CIGARETTES AS A POTENTIAL ETIOLOGIC FACTOR OF SQUAMOUS CELL CARCINOMA. Oral Surg Oral Med Oral Pathol Oral Radiol. 2022;134: e228–e228. doi:10.1016/j.oooo.2022.01.728	No	Not meeting inclusion criteria	Title and abstract screening
4444	Sobieski E, Yingst J, Foulds J. Quitting electronic cigarettes: Factors associated with quitting and quit attempts in long-term users. Addict Behav. 2022;127: 107220. doi:10.1016/j.addbeh.2021.107220	No	Not meeting inclusion criteria	Title and abstract screening
4445	Sobrero AF, Pastorino A, Zalcberg JR. You're Cured Till You're Not: Should Disease-Free Survival Be Used as a Regulatory or Clinical End Point for Adjuvant Therapy of Cancer? J Clin Oncol. 2022;40: 4044–4047. doi:10.1200/JCO.22.01531	No	Not meeting inclusion criteria	Title and abstract screening
4446	Society for Adolescent Health and Medicine. Protecting Youth From the Risks of Electronic Cigarettes. J Adolesc Health Off Publ Soc Adolesc Med. 2020;66: 127–131. doi:10.1016/j.jadohealth.2019.10.007	No	Not meeting inclusion criteria	Title and abstract screening
4447	Sohal S.S. Therapeutic Modalities for Asthma, COPD, and Pathogenesis of COVID-19: Insights from the Special Issue. J Clin Med. 2022;11: 4525. doi:10.3390/jcm11154525	No	Not meeting inclusion criteria	Title and abstract screening

4448	Sokolov M, Petrov B, Maslyankov S, Angelov K, Atanasova M, Tzoneva D, et al. Technical considerations depending on the level of vascular ligation in laparoscopic rectal resection. 2021. doi:10.1007/s00464-021-08479-x	No	Not meeting inclusion criteria	Title and abstract screening
4449	Solanki K., Patel R., Sopontammarak B., Elabbassi A.M. A Curious Case of a Colossal Carcinoid Tumor. Am J Respir Crit Care Med. 2022;205. doi:10.1164/ajrccm-conference.2022.205.1_MeetingAbstracts.A4404	No	Not meeting inclusion criteria	Title and abstract screening
4450	Solares C.A. Interdisciplinary Teamwork within the Medical Profession: The Way of the Future. Am J Rhinol Allergy. 2021;35: 148–151. doi:10.1177/1945892421992580	No	Not meeting inclusion criteria	Title and abstract screening
4451	Solecki S., Adegite E., Turchi R. Clearing the air: Adolescent smoking trends. Curr Opin Pediatr. 2019;31: 670–674. doi:10.1097/MOP.0000000000000810	No	Not meeting inclusion criteria	Title and abstract screening
4452	Solidum JGN, Solomon RG, Silverio CE, Dee EC. The Philippine vape bill: how politics affects health. Lancet Respir Med. 2022;10: e86. doi:10.1016/S2213-2600(22)00292-2	No	Not meeting inclusion criteria	Title and abstract screening
4453	Solimini R, Stack C, Clifford K, Bencze L, Ruiz Dominguez FM, Vardavas CI, et al. Reporting system of suspected adverse effects from electronic cigarettes and refill liquids in Europe. Tob Prev Cessat. 2021;7: 71. doi:10.18332/tpc/143331	No	Not meeting inclusion criteria	Title and abstract screening
4454	Solingapuram Sai KK, Rose JE, Mukhin AG. Effect of Electronic Cigarette Liquid pH on Retention of 11C-Nicotine in a Respiratory Tract Model. Nicotine Tob Res Off J Soc Res Nicotine Tob. 2023;25: 1406–1410. doi:10.1093/ntr/ntad039	No	Not meeting inclusion criteria	Title and abstract screening
4455	Solingapuram Sai KK, Zuo Y, Rose JE, Garg PK, Garg S, Nazih R, et al. Rapid Brain Nicotine Uptake from Electronic Cigarettes. J Nucl Med. 2020;61: 928–930. doi:10.2967/jnumed.119.230748	No	Not meeting inclusion criteria	Title and abstract screening
4456	Soneji S, Barrington-Trimis JL, Wills TA, Leventhal AM, Unger JB, Gibson LA, et al. Association Between Initial Use of e-Cigarettes and Subsequent Cigarette Smoking Among Adolescents and Young Adults: A Systematic Review and Meta-analysis. JAMA Pediatr. 2017;171: 788–797. doi:10.1001/jamapediatrics.2017.1488	No	Not meeting inclusion criteria	Title and abstract screening
4457	Song A, Zhang Z, Liu Z. Psychoactive Comfort Products or Snacks: How Chinese Young Adults Perceive the Potentially Addictive Nature of E-Cigarettes. Healthc Basel Switz. 2023;11. doi:10.3390/healthcare11101440	No	Not meeting inclusion criteria	Title and abstract screening
4458	Song Y, Li X, Li C, Xu S, Liu Y, Wu X. What Are the Effects of Electronic Cigarettes on Lung Function Compared to Non-Electronic Cigarettes? A Systematic Analysis. Int J Public Health. 2022;67: 1604989. doi:10.3389/ijph.2022.1604989	No	Not meeting inclusion criteria	Title and abstract screening
4459	Sóñora G, Reynales-Shigematsu LM, Barnoya J, Llorente B, Szklo AS, Thrasher JF. Achievements, challenges, priorities and needs to address the current tobacco epidemic in Latin America. Tob Control. 2022;31: 138–141. doi:10.1136/tobaccocontrol-2021-057007	No	Not meeting inclusion criteria	Title and abstract screening
4460	Sontag J, Manderski M, Hammond D, Wackowski O. US young adults' perceived effectiveness of draft pictorial e-cigarette warning labels. 2019. doi:10.1136/tobaccocontrol-2018-054802	No	Not meeting inclusion criteria	Title and abstract screening
4461	Sontag JM, Wackowski OA, Hammond D. Baseline assessment of noticing e-cigarette health warnings among youth and young adults in the United States, Canada and England, and associations with harm perceptions, nicotine awareness and warning recall. Prev Med Rep. 2019;16: 100966. doi:10.1016/j.pmedr.2019.100966	No	Not meeting inclusion criteria	Title and abstract screening
4462	Soo J, Easwaran M, Erickson-DiRenzo E. Impact of Electronic Cigarettes on the Upper Aerodigestive Tract: A Comprehensive Review for Otolaryngology Providers. OTO Open. 2023;7: e25. doi:10.1002/oto2.25	No	Not meeting inclusion criteria	Title and abstract screening
4463	Sood SB, Weatherly AJ, Smith AH, Murphy MA, Conrad SJ, Bichell DP. Vaping Contributing to Postoperative Acute Respiratory Distress Syndrome. Ann Thorac Surg. 2021;112: e169–e171. doi:10.1016/j.athoracsur.2021.01.021	No	Not meeting inclusion criteria	Title and abstract screening
4464	Sorgen LJ, Ferrer RA, Klein WMP, Kaufman AR. Smoking self-concept moderates the effects of self-affirmation on smoking-related beliefs and behavioral intentions. Psychol Health. 2022;37: 964–984. doi:10.1080/08870446.2021.1912346	No	Not meeting inclusion criteria	Title and abstract screening
4465	Sosnoff CS, Caron K, Akins JR, Dortch K, Hunter RE, Pine BN, et al. Serum Concentrations of Cotinine and Trans-3'-Hydroxycotinine in US Adults: Results From Wave 1 (2013-2014) of the Population Assessment of Tobacco and Health Study. Nicotine Tob Res. 2022;24: 736–744. doi:10.1093/ntr/ntab240	No	Not meeting inclusion criteria	Title and abstract screening
4466	Soule E, Bansal-Travers M, Grana R, McIntosh S, Price S, Unger JB, et al. Electronic cigarette use intensity measurement challenges and regulatory implications. Tob Control. 2023;32: 124–129. doi:10.1136/tobaccocontrol-2021-056483	No	Not meeting inclusion criteria	Title and abstract screening
4467	Soule EK, Mayne S, Snipes W, Do EK, Theall T, Höchsmann C, et al. Electronic cigarette nicotine flux, nicotine yield, and particulate matter emissions: impact of device and liquid heterogeneity. Nicotine Tob Res. 2022. doi:10.1093/ntr/ntac196	No	Not meeting inclusion criteria	Title and abstract screening
4468	Soule EK, Mayne S, Snipes W, Do EK, Theall T, Höchsmann C, et al. Electronic Cigarette Nicotine Flux, Nicotine Yield, and Particulate Matter Emissions: Impact of Device and Liquid Heterogeneity. Nicotine Tob Res. 2023;25: 412–420. doi:10.1093/ntr/ntac196	No	Not meeting inclusion criteria	Title and abstract screening
4469	Soulet S, Constans L, Quinty V. Physical and chemical characterizations of a reference e-cigarette used in animal testing. Sci Rep. 2023;13: 16624. doi:10.1038/s41598-023-43733-3	No	Not meeting inclusion criteria	Title and abstract screening
4470	Spaedy A., Doumas S., Solhkhah R. Substance Use Disorders in Vulnerable Children. Pediatr Clin North Am. 2020;67: 373–385. doi:10.1016/j.pcl.2019.11.002	No	Not meeting inclusion criteria	Title and abstract screening
4471	Sparrock LS, Phan L, Chen-Sankey J, Hacker K, Ajith A, Jewett B, et al. Heated Tobacco Products: Awareness, Beliefs, Use and Susceptibility among US Adult Current Tobacco Users, 2021. Int J Environ Res Public Health. 2023;20. doi:10.3390/ijerph20032016	No	Not meeting inclusion criteria	Title and abstract screening
4472	Spatuzzi R, Vespa A, Fabbietti P, Ricciuti M, Rosati G, Guariniello L, et al. Elderly Helping Other Elderly: A Comparative Study of Family Caregiver Burden Between Patients With Dementia or Cancer at the End of Life. J Soc Work End--Life Palliat Care. 2022;18: 96–108. doi:10.1080/15524256.2022.2042459	No	Not meeting inclusion criteria	Title and abstract screening
4473	Spears CA, Jones DM, Cottrell-Daniels C, Elahi H, Strosnider C, Luong J, et al. "When I Don't Have a Cigarette It's Helpful, but It Really Don't Satisfy:" Qualitative Study of Electronic Nicotine Delivery Systems (ENDS) Use among Low-Income Smokers. Int J Env Res Public Health. 2022;19. doi:10.3390/ijerph19031157	No	Not meeting inclusion criteria	Title and abstract screening
4474	Spears CA, Jones DM, Weaver SR, Pechacek TF, Eriksen MP. Motives and perceptions regarding electronic nicotine delivery systems (ENDS) use among adults with mental health conditions. Addict Behav. 2018;80: 102–109. doi:10.1016/j.addbeh.2018.01.014	No	Not meeting inclusion criteria	Title and abstract screening
4475	Spoladore R, Daus F, Pezzini S, Milani M, Limonta A, Savonitto S. The point on the electronic cigarette more than 10 years after its introduction. Eur Heart J Suppl. 2022;24: I148–I152. doi:10.1093/eurheartjsupp/suac105	No	Not meeting inclusion criteria	Title and abstract screening
4476	Spoladore R, Daus F, Pezzini S, Napoli F, Cante L, Savonitto S. [The fact about e-cigarettes and cardiovascular risk]. G Ital Cardiol 2006. 2023;24: 466–474. doi:10.1714/4041.40206	No	Not meeting inclusion criteria	Title and abstract screening

4477	Spoons J., Treat S., McNabney M., Horner J., Smith N. Evali masquerading as cannabinoid hyperemesis syndrome. Am J Respir Crit Care Med. 2021;203. doi:10.1164/ajrccm-conference.2021.203.1_MeetingAbstracts.A2142	No	Not meeting inclusion criteria	Title and abstract screening
4478	Springfeld C, Büchler MW, Neoptolemos JP. Interpreting Primary End Points in Randomized Trials of Pancreatic Cancer. J Clin Oncol. 2023;41: 4182–4183. doi:10.1200/JCO.23.00779	No	Not meeting inclusion criteria	Title and abstract screening
4479	Squeglia LM, Fadus MC, McClure EA, Tomko RL, Gray KM. Pharmacological Treatment of Youth Substance Use Disorders. J Child Adolesc Psychopharmacol. 2019;29: 559–572. doi:10.1089/cap.2019.0009	No	Not meeting inclusion criteria	Title and abstract screening
4480	Sreedharan S, Mian M, Robertson RA, Rhodes A. Radiological findings of e-cigarette or vaping product use associated lung injury: A systematic review. Heart Lung. 2021;50: 736–741. doi:10.1016/j.hrtlng.2021.05.004	No	Not meeting inclusion criteria	Title and abstract screening
4481	Sribna O., Kvasha O. Electronic nicotine delivery systems (ENDS) usage and cardiovascular diseases risk. Eur J Prev Cardiol. 2018;25: S84.	No	Not meeting inclusion criteria	Title and abstract screening
4482	Sridharan K, Paul E, Stirling RG, Li C. Impacts of multidisciplinary meeting case discussion on palliative care referral and end-of-life care in lung cancer: a retrospective observational study. Intern Med J. 2021;51: 1450–1456. doi:10.1111/imj.15215	No	Not meeting inclusion criteria	Title and abstract screening
4483	St Claire AW, Schillo BA, Lien RK, Keller PA, O'Gara E, D'Silva J, et al. Changing patterns in E-cigarette use among Minnesota adults between 2014 and 2018. Prev Med Rep. 2019;16: 101014. doi:10.1016/j.pmedr.2019.101014	No	Not meeting inclusion criteria	Title and abstract screening
4484	St Claire S, Gouda H, Schotte K, Fayokun R, Fu D, Varghese C, et al. Lung health, tobacco, and related products: gaps, challenges, new threats, and suggested research. Am J Physiol Lung Cell Mol Physiol. 2020;318: L1004–L1007. doi:10.1152/ajplung.00101.2020	No	Not meeting inclusion criteria	Title and abstract screening
4485	St Helen G, Benowitz NL, Ahluwalia JS, Tyndale RF, Addo N, Gregorich SE, et al. Black Light Smokers: How Nicotine Intake and Carcinogen Exposure Differ Across Various Biobehavioral Factors. J Natl Med Assoc. 2019;111: 509–520. doi:10.1016/j.jnma.2019.04.004	No	Not meeting inclusion criteria	Title and abstract screening
4486	Staal Y, Havermans A, van Nierop L, Visser W, Wijnhoven S, Bil W, et al. Conceptual model for the evaluation of attractiveness, addictiveness and toxicity of tobacco and related products: The example of JUUL e-cigarettes. Regul Toxicol Pharmacol. 2021;127: 105077. doi:10.1016/j.yrtph.2021.105077	No	Not meeting inclusion criteria	Title and abstract screening
4487	Staal Y.C.M., Li Y., Gerber L.-S., Fokkens P., Cremers H., Cassee F.R., et al. Neuromodulatory and neurotoxic effects of e-cigarette vapor using a realistic exposure method. Inhal Toxicol. 2022. doi:10.1080/08958378.2022.2118911	No	Not meeting inclusion criteria	Title and abstract screening
4488	Stacey S.K. E-cigarettes: What you need to know. Osteopath Fam Physician. 2018;10: 35.	No	Not meeting inclusion criteria	Title and abstract screening
4489	Stanton CA, Pasch KE, Pericot-Valverde I, Cruz-Cano R, Moran MB, Abadi MH, et al. Longitudinal associations between U.S. youth exposure to E-cigarette marketing and E-cigarette use harm perception and behavior change. Prev Med. 2022;164: 107266. doi:10.1016/j.ypmed.2022.107266	No	Not meeting inclusion criteria	Title and abstract screening
4490	Stanton CA, Tang Z, Sharma E, Seaman E, Gardner LD, Silveira ML, et al. Predictors of E-cigarette and Cigarette Use Trajectory Classes from Early Adolescence to Emerging Adulthood Across Four Years (2013-2017) of the PATH Study. Nicotine Tob Res. 2023;25: 421–429. doi:10.1093/ntr/ntac119	No	Not meeting inclusion criteria	Title and abstract screening
4491	Stead M, Ford A, Angus K, MacKintosh AM, Purves R, Mitchell D. E-Cigarette Advertising in the UK: A Content Analysis of Traditional and Social Media Advertising to Observe Compliance with Current Regulations. Nicotine Tob Res. 2021;23: 1839–1847. doi:10.1093/ntr/ntab075	No	Not meeting inclusion criteria	Title and abstract screening
4492	Stefaniak AB, LeBouf RF, Ranpara AC, Leonard SS. Toxicology of flavoring- and cannabis-containing e-liquids used in electronic delivery systems. Pharmacol Ther. 2021;224: 107838. doi:10.1016/j.pharmthera.2021.107838	No	Not meeting inclusion criteria	Title and abstract screening
4493	Stegmann M. THE EFFECT OF PRIORITISING TREATMENT GOALS ON DECISION SELF-EFFICACY AMONG OLDER PATIENTS WITH CANCER IN A PALLIATIVE SETTING (OPTIONSTUDY). 2019;9: A46-A47. doi:10.1136/spcare-2019-ACPICONGRESSABS.143	No	Not meeting inclusion criteria	Title and abstract screening
4494	Stein J.S., Koffarnus M.N., Stepanov I., Hatsukami D.K., Bickel W.K. Cigarette and e-liquid demand and substitution in e-cigarette-naive smokers. Exp Clin Psychopharmacol. 2018;26: 233–243. doi:10.1037/pha0000192	No	Not meeting inclusion criteria	Title and abstract screening
4495	Stein JH, Korcarz CE. E-Cigarettes versus Nicotine-Replacement Therapy for Smoking Cessation. N Engl J Med. 2019;380: 1973–1974. doi:10.1056/NEJMc1903758	No	Not meeting inclusion criteria	Title and abstract screening
4496	Steinberg M.L., Rosen R.L., Billingsley B., Shah D., Bender M., Shargo K., et al. Tobacco/nicotine use among individuals using cannabis for therapeutic purposes. Am J Addict. 2022;31: 486–493. doi:10.1111/ajad.13323	No	Not meeting inclusion criteria	Title and abstract screening
4497	Steiner V, Joubert L, Shlonsky A, Morris A. Hospital Parenting Support for Adults with Incurable End-Stage Cancer: Multidisciplinary Health Professional Perspectives. Health Soc Work. 2021;46: 289–298. doi:10.1093/hsw/hlab024	No	Not meeting inclusion criteria	Title and abstract screening
4498	Steliga M. Nicotine dependence and cessation in lung cancer patients. J Thorac Oncol. 2017;12: S1691.	No	Not meeting inclusion criteria	Title and abstract screening
4499	Steliga MA. Health hazards of electronic cigarettes and their utility in smoking cessation. J Thorac Cardiovasc Surg. 2022;163: 307–310. doi:10.1016/j.jtcvs.2020.11.180	No	Not meeting inclusion criteria	Title and abstract screening
4500	Stevens EM, Keller-Hamilton B, Mays D, Unger JB, Wackowski OA, West JC, et al. Optimizing Images for an E-Cigarette Messaging Campaign: Liking and Perceived Effectiveness. Int J Env Res Public Health. 2021;18. doi:10.3390/ijerph182412989	No	Not meeting inclusion criteria	Title and abstract screening
4501	Stevens ER, Lei L, Cleland CM, Vojjala M, El-Shahawy O, Berger KI, et al. Electronic cigarettes as a harm reduction strategy among patients with COPD: protocol for an open-label two arm randomized controlled pilot trial. Addict Sci Clin Pr. 2022;17: 2. doi:10.1186/s13722-021-00284-0	No	Not meeting inclusion criteria	Title and abstract screening
4502	Stitt D., Kumar N. Drugs of Abuse and the Nervous System. Contin Lifelong Learn Neurol. 2020;26: 765–784. doi:10.1212/CON.0000000000000857	No	Not meeting inclusion criteria	Title and abstract screening
4503	Stokes A.C. Declines in electronic cigarette use among US youth in the era of Covid-19-A critical opportunity to stop youth vaping in its tracks. JAMA Netw Open. 2020;3: e20228221. doi:10.1001/jamanetworkopen.2020.28221	No	Not meeting inclusion criteria	Title and abstract screening
4504	Stokes AC, Xie W, Wilson AE, Yang H, Orimoloye OA, Harlow AF, et al. Association of Cigarette and Electronic Cigarette Use Patterns With Levels of Inflammatory and Oxidative Stress Biomarkers Among US Adults: Population Assessment of Tobacco and Health Study. Circulation. 2021;143: 869–871. doi:10.1161/CIRCULATIONAHA.120.051551	No	Not meeting inclusion criteria	Title and abstract screening
4505	Stone A. Biden Cancer Moonshot Relaunch Will "End Cancer as We Know It". Voice. 2022;37: 17–17.	No	Not meeting inclusion criteria	Title and abstract screening

4506	Stone E, Evison M, van Zyl-Smit RN, Andarini S, Shah S, Vandeleur M. Recreational vaping ban in Australia-policy failure or masterstroke? Lancet. 2023. doi:10.1016/S0140-6736(23)02298-5	No	Not meeting inclusion criteria	Title and abstract screening
4507	Stone E. ES13.03 Foundation for a Smoke Free Future: Funding Opportunities or Smoke Screen for the Tobacco Industry? J Thorac Oncol. 2019;14: S47. doi:10.1016/j.jtho.2019.08.128	No	Not meeting inclusion criteria	Title and abstract screening
4508	Stone E., Peters M. Young low and middle-income country (LMIC) smokers- implications for global tobacco control. Transl Lung Cancer Res. 2017;6: S44–S46. doi:10.21037/tlcr.2017.10.11	No	Not meeting inclusion criteria	Title and abstract screening
4509	Stone E.T., Deitchman A., Das N. Its not easy as ild. Am J Respir Crit Care Med. 2021;203. doi:10.1164/ajrccm-conference.2021.203.1_MeetingAbstracts.A2035	No	Not meeting inclusion criteria	Title and abstract screening
4510	Stone H., Donahue A., Fleisher S. Seizure, abdominal pain, and urine pink in an adolescent female-what's the link? J Investig Med. 2022;70: 603. doi:10.1136/jim-2022-SRMC.314	No	Not meeting inclusion criteria	Title and abstract screening
4511	Stone MD, Braymiller JL, Strong DR, Cwalina SN, Dimofte CV, Barrington-Trimis JL. Differentiating Reasons for Young Adult E-Cigarette Use Using Maximum Difference Choice Models. Nicotine Tob Res Off J Soc Res Nicotine Tob. 2023. doi:10.1093/ntr/ntad017	No	Not meeting inclusion criteria	Title and abstract screening
4512	Stöver H. [Diversification of smoking cessation programmes-the role of e-cigarettes]. Bundesgesundheitsblatt Gesundheitsforschung Gesundheitsschutz. 2021;64: 1473–1479. doi:10.1007/s00103-021-03435-5	No	Not meeting inclusion criteria	Title and abstract screening
4513	Stower H. E-cigarettes to help smoking cessation. Nat Med. 2019;25: 358. doi:10.1038/s41591-019-0395-5	No	Not meeting inclusion criteria	Title and abstract screening
4514	Streck JM, Kalkhoran S, Bearnot B, Gupta PS, Kalagher KM, Regan S, et al. Perceived risk, attitudes, and behavior of cigarette smokers and nicotine vapers receiving buprenorphine treatment for opioid use disorder during the COVID-19 pandemic. Drug Alcohol Depend. 2021;218: 108438. doi:10.1016/j.drugalcdep.2020.108438	No	Not meeting inclusion criteria	Title and abstract screening
4515	Streck JM, Regan S, Bearnot B, Gupta PS, Kalkhoran S, Kalagher KM, et al. Prevalence of Cannabis Use and Cannabis Route of Administration among Massachusetts Adults in Buprenorphine Treatment for Opioid Use Disorder. Subst Use Misuse. 2022;57: 1104–1110. doi:10.1080/10826084.2022.2063899	No	Not meeting inclusion criteria	Title and abstract screening
4516	Streck JM, Regan S, Neil J, Kalkhoran S, Gupta PS, Bearnot B, et al. Interest in Electronic Cigarettes for Smoking Cessation Among Adults With Opioid Use Disorder in Buprenorphine Treatment: A Mixed-Methods Investigation. Nicotine Tob Res. 2022;24: 1134–1138. doi:10.1093/ntr/ntab259	No	Not meeting inclusion criteria	Title and abstract screening
4517	Streck JM, Lee J-W, Walter AW, Rosen RL, Gareen IF, Kircher SM, et al. Cigarette and Alternative Tobacco Product Use among Adult Cancer Survivors Enrolled in 9 ECOG-ACRIN Clinical Trials. Cancer Epidemiol Biomark Prev Publ Am Assoc Cancer Res Cosponsored Am Soc Prev Oncol. 2023;32: 1552–1557. doi:10.1158/1055-9965.EPI-23-0420	No	Not meeting inclusion criteria	Title and abstract screening
4518	Strickland JC, Vsevolozhskaya OA, Stoops WW. E-Cigarette Demand: Impact of Commodity Definitions and Test-Retest Reliability. Nicotine Tob Res. 2021;23: 557–565. doi:10.1093/ntr/ntaa139	No	Not meeting inclusion criteria	Title and abstract screening
4519	Striley CW, Nutley SK, Hoeflich CC. E-cigarettes and non-suicidal self-injury: Prevalence of risk behavior and variation by substance inhaled. Front Psychiatry. 2022;13: 911136. doi:10.3389/fpsyt.2022.911136	No	Not meeting inclusion criteria	Title and abstract screening
4520	Strombotne K, Buckell J, Sindelar JL. Do JUUL and e-cigarette flavours change risk perceptions of adolescents? Evidence from a national survey. Tob Control. 2021;30: 199–205. doi:10.1136/tobaccocontrol-2019-055394	No	Not meeting inclusion criteria	Title and abstract screening
4521	Strong D.R., Leas E., Elton-Marshall T., Wackowski O.A., Travers M., Bansal-Travers M., et al. Harm perceptions and tobacco use initiation among youth in Wave 1 and 2 of the Population Assessment of Tobacco and Health (PATH) Study. Prev Med. 2019;123: 185–191. doi:10.1016/j.ypmed.2019.03.017	No	Not meeting inclusion criteria	Title and abstract screening
4522	Strong D.R., Messer K., White M., Shi Y., Noble M., Portnoy D.B., et al. Youth perception of harm and addictiveness of tobacco products: Findings from the Population Assessment of Tobacco and Health Study (Wave 1). Addict Behav. 2019;92: 128–135. doi:10.1016/j.addbeh.2018.12.005	No	Not meeting inclusion criteria	Title and abstract screening
4523	Strong DR, Pierce JP, White M, Stone MD, Abrams DB, Glasser AM, et al. Changes in Tobacco Dependence and Association With Onset and Progression of Use by Product Type From Waves 1 to 3 of the Population Assessment of Tobacco and Health (PATH) Study. Nicotine Tob Res Off J Soc Res Nicotine Tob. 2023;25: 1781–1790. doi:10.1093/ntr/ntad107	No	Not meeting inclusion criteria	Title and abstract screening
4524	Strong DR, Leas E, Noble M, White M, Glasser A, Taylor K, et al. Validation of the Wave 1 and Wave 2 Population Assessment of Tobacco and Health (PATH) Study Indicators of Tobacco Dependence Using Biomarkers of Nicotine Exposure Across Tobacco Products. Nicotine Tob Res. 2022;24: 10–19. doi:10.1093/ntr/ntab162	No	Not meeting inclusion criteria	Title and abstract screening
4525	Strong DR, Myers MG, Pulvers K, Noble M, Brikmanis K, Doran N. Marijuana use among US tobacco users: Findings from wave 1 of the population assessment of tobacco health (PATH) study. Drug Alcohol Depend. 2018;186: 16–22. doi:10.1016/j.drugalcdep.2017.12.044	No	Not meeting inclusion criteria	Title and abstract screening
4526	Stroud I, Gutman LM. Longitudinal changes in the mental health of UK young male and female adults during the COVID-19 pandemic. Psychiatry Res. 2021;303: 114074. doi:10.1016/j.psychres.2021.114074	No	Not meeting inclusion criteria	Title and abstract screening
4527	Stroud L.R., McCallum M., Borba K. 25.1 TOBACCO USE DURING PREGNANCY: IMPACT ON OFFSPRING NEUROBEHAVIORAL DEVELOPMENT. J Am Acad Child Adolesc Psychiatry. 2019;58: S337. doi:10.1016/j.jaac.2019.07.798	No	Not meeting inclusion criteria	Title and abstract screening
4528	Stroup A.M., Branstetter S.A. Comparing Immediate Physiological Responses and Future Intentions of Use between Combustible and Electronic Waterpipe Users. Addict Behav. 2021;115: 106741. doi:10.1016/j.addbeh.2020.106741	No	Not meeting inclusion criteria	Title and abstract screening
4529	Struik L, Yang Y. e-Cigarette Cessation: Content Analysis of a Quit Vaping Community on Reddit. J Med Internet Res. 2021;23: e28303. doi:10.2196/28303	No	Not meeting inclusion criteria	Title and abstract screening
4530	Struik LL, O'Loughlin EK, Riglea T, O'Loughlin JL. Predictors of past-year e-cigarette use among young adults. Prev Med Rep. 2022;29: 101965. doi:10.1016/j.pmedr.2022.101965	No	Not meeting inclusion criteria	Title and abstract screening
4531	Studts J, Carter L, Feldman J, Donaldson D, Pantelas J, Ostroff J, et al. P2.08-07 The Campaign to End Lung Cancer Stigma: The ACS National Lung Cancer Roundtable Efforts to Confront and Extinguish Lung Cancer Stigma. J Thorac Oncol. 2022;17: S141–S141. doi:10.1016/j.jtho.2022.07.235	No	Not meeting inclusion criteria	Title and abstract screening
4532	Su A, Albarran Garcia D, Li M, Ogbonna C, Tsang T, Tsang A, et al. Clinical course and treatment interventions for adolescents who vaped during the COVID-19 pandemic. BMJ Case Rep. 2023;16. doi:10.1136/bcr-2023-255844	No	Not meeting inclusion criteria	Title and abstract screening
4533	Su L, Liu J, Yue Q, Zhang S, Zhao C, Sun X, et al. Evaluation of the effects of e-cigarette aerosol extracts and tobacco cigarette smoke extracts on human gingival epithelial cells. Toxicol Vitro. 2023; 105605. doi:10.1016/j.tiv.2023.105605	No	Not meeting inclusion criteria	Title and abstract screening
4534	Su W-C, Lee J, Zhang K, Wong S-W, Buu A. Estimation of Health Risks Caused by Metals Contained in E-Cigarette Aerosol through Passive Vaping. Toxics. 2023;11. doi:10.3390/toxics11080684	No	Not meeting inclusion criteria	Title and abstract screening

4535	Su W-C, Wong S-W, Buu A. Deposition of E-cigarette aerosol in human airways through passive vaping. Indoor Air. 2021;31: 348–356. doi:10.1111/ina.12754	No	Not meeting inclusion criteria	Title and abstract screening
4536	Suchanecka A, Chmielowiec J, Chmielowiec K, Trybek G, Jaroń A, Czarny W, et al. Serotonin Receptor HTR3A Gene Polymorphisms rs1985242 and rs1062613, E-Cigarette Use and Personality. Int J Env Res Public Health. 2022;19. doi:10.3390/ijerph19084746	No	Not meeting inclusion criteria	Title and abstract screening
4537	Sudano I., Barthelmes J., Kubli B. Smoking, smoking cessation and cardiovascular risk. Kardiovaskulare Med. 2018;21: 274–277.	No	Not meeting inclusion criteria	Title and abstract screening
4538	Sudarshan K.B., Hori Y., Swid M.A., Karavos A.C., Wooten C., Armour J.A., et al. A novel metric linking stellate ganglion neuronal population dynamics to cardiopulmonary physiology. Am J Physiol - Heart Circ Physiol. 2021;321: H369–H381. doi:10.1152/AJPHEART.00206.2021	No	Not meeting inclusion criteria	Title and abstract screening
4539	Sugiyo D., Limato R., Handari S.D. Tobacco Use and the Risk of Suffering from COVID-19. Open Access Maced J Med Sci. 2021;9: 111–117. doi:10.3889/oamjms.2021.5757	No	Not meeting inclusion criteria	Title and abstract screening
4540	Sullivan L., Sell R. EVERY SYSTEM IN LUPUS ERYTHEMATOSUS. Chest. 2021;160: A2071. doi:10.1016/j.chest.2021.07.1828	No	Not meeting inclusion criteria	Title and abstract screening
4541	Sultan AS, Jessri M, Farah CS. Electronic nicotine delivery systems: Oral health implications and oral cancer risk. J Oral Pathol Med Off Publ Int Assoc Oral Pathol Am Acad Oral Pathol. 2021;50: 316–322. doi:10.1111/jop.12810	No	Not meeting inclusion criteria	Title and abstract screening
4542	Sumner W., Farsalinos K. Lessons and guidance from the special issue on electronic cigarette use and public health. Int J Environ Res Public Health. 2018;15: 1338. doi:10.3390/ijerph15071338	No	Not meeting inclusion criteria	Title and abstract screening
4543	Sumner W, Kim SS, Vitzthum L, Moore K, Atwood T, Murphy J, et al. End of treatment cone-beam computed tomography (CBCT) is predictive of radiation response and overall survival in oropharyngeal squamous cell carcinoma. Radiat Oncol. 2021;16: 1–9. doi:10.1186/s13014-021-01871-w	No	Not meeting inclusion criteria	Title and abstract screening
4544	Sun J., Kovalszki A. HYPERSENSITIVITY PNEUMONITIS OR EVALI? A CASE OF ACUTE RESPIRATORY FAILURE IN AN ADOLESCENT. Ann Allergy Asthma Immunol. 2021;127: S88. doi:10.1016/j.anai.2021.08.275	No	Not meeting inclusion criteria	Title and abstract screening
4545	Sun T, Lim CCW, Chung J, Cheng B, Davidson L, Tisdale C, et al. Vaping on TikTok: a systematic thematic analysis. Tob Control. 2021. doi:10.1136/tobaccocontrol-2021-056619	No	Not meeting inclusion criteria	Title and abstract screening
4546	Sun Y.-W., Chen K.-M., Atkins H., Aliaga C., Gordon T., Guttenplan J.B., et al. Effects of E-Cigarette Aerosols with Varying Levels of Nicotine on Biomarkers of Oxidative Stress and Inflammation in Mice. Chem Res Toxicol. 2021;34: 1161–1168. doi:10.1021/acs.chemrestox.1c00033	No	Not meeting inclusion criteria	Title and abstract screening
4547	Sund L.J., Dargan P.I., Wood D.M. The emerging cloud: a survey of vapers, their health, and the associated burden on the UK health system. Clin Toxicol. 2023;61: 39–40. doi:10.1080/15563650.2023.2192024	No	Not meeting inclusion criteria	Title and abstract screening
4548	Sund LJ, Dargan PI, Archer JRH, Wood DM. E-cigarette or vaping-associated lung injury (EVALI): a review of international case reports from outside the United States of America. Clin Toxicol Phila. 2023;61: 91–97. doi:10.1080/15563650.2022.2160342	No	Not meeting inclusion criteria	Title and abstract screening
4549	Sund LJ, Dargan PI, Archer JRH, Blundell MS, Wood DM. The Emerging Cloud: a survey of vapers, their health and utilization of healthcare within the UK. QJM. 2023;116: 993–1001. doi:10.1093/qjmed/hcad210	No	Not meeting inclusion criteria	Title and abstract screening
4550	Sund LJ, Wood DM, Archer JRH, Blundell MS, Dargan PI. The unseen cloud: a survey of vaping practices and the acquisition of vaping products within the UK. QJM. 2023;116: 99–106. doi:10.1093/qjmed/hcac220	No	Not meeting inclusion criteria	Title and abstract screening
4551	Sung J, Shrestha SS, Kim Y, Emery S, Wang X. Changes in Sales of Tobacco and Nicotine Replacement Therapy Products Before and During the COVID-19 Pandemic. Prev Chronic Dis. 2023;20: E71. doi:10.5888/pcd20.220406	No	Not meeting inclusion criteria	Title and abstract screening
4552	Sussan TE, Shahzad FG, Tabassum E, Cohen JE, Wise RA, Blaha MJ, et al. Electronic cigarette use behaviors and motivations among smokers and non-smokers. BMC Public Health. 2017;17: 686. doi:10.1186/s12889-017-4671-3	No	Not meeting inclusion criteria	Title and abstract screening
4553	Sussman MA. VAPIng into ARDS: Acute Respiratory Distress Syndrome and Cardiopulmonary Failure. Pharmacol Ther. 2022;232: 108006. doi:10.1016/j.pharmthera.2021.108006	No	Not meeting inclusion criteria	Title and abstract screening
4554	Sussman RA, Golberstein E, Polosa R. Aerial Transmission of the SARS-CoV-2 Virus through Environmental E-Cigarette Aerosols: Implications for Public Policies. Int J Env Res Public Health. 2021;18. doi:10.3390/ijerph18041437	No	Not meeting inclusion criteria	Title and abstract screening
4555	Sussman RA, Golberstein E, Polosa R. Analytic modeling and risk assessment of aerial transmission of SARS-CoV-2 virus through vaping expirations in shared micro-environments. Env Sci Pollut Res Int. 2022;29: 83020–83044. doi:10.1007/s11356-022-20499-1	No	Not meeting inclusion criteria	Title and abstract screening
4556	Sussman S, Galimov A, Meza L, Huh J, Baezconde-Garbanati L, Pokhrel P. Peer Crowd Identification of Young and Early Middle Adulthood Customers at Vape Shops. J Drug Educ. 2021;50: 98–107. doi:10.1177/00472379221106365	No	Not meeting inclusion criteria	Title and abstract screening
4557	Sutfin E.L., Denlinger-Apte R.L., Ross J.C., Wagoner K.G., Suerken C.K., Spangler J., et al. Corrigendum to "Longitudinal latent class analysis of tobacco use and correlates among young adults over a 10-year period" [Drug Alcohol Depend. 236 (2022) 109474] (Drug and Alcohol Dependence (2022) 236, (S0376871622002113), (10.1016/j.drugalcdep.2022.10. Drug Alcohol Depend. 2022;239: 109593. doi:10.1016/j.drugalcdep.2022.109593	No	Not meeting inclusion criteria	Title and abstract screening
4558	Sutherland MT, Stein EA. Functional Neurocircuits and Neuroimaging Biomarkers of Tobacco Use Disorder. Trends Mol Med. 2018;24: 129–143. doi:10.1016/j.molmed.2017.12.002	No	Not meeting inclusion criteria	Title and abstract screening
4559	Sutton SK, Brandon KO, Harrell PT, Martínez Ú, Simmons VN, Gore LR, et al. Identifying prospective subpopulations of combustible and electronic cigarette dual users in the United States via finite mixture modeling. Addiction. 2022;117: 2493–2503. doi:10.1111/add.15906	No	Not meeting inclusion criteria	Title and abstract screening
4560	Suzuki Y, Dohmae S, Ohyama K, Chiba T, Nakagami S, Miyagi E, et al. Real-world data on home end-of-life care for older adults with cancer: A retrospective claims data analysis. Aging Med. 2023;6: 163–169. doi:10.1002/agm2.12246	No	Not meeting inclusion criteria	Title and abstract screening
4561	Svenson M, Green J, Maynard OM. Tackling Smoker Misperceptions About E-cigarettes Using Expert Videos. Nicotine Tob Res. 2021;23: 1848–1854. doi:10.1093/ntr/ntab104	No	Not meeting inclusion criteria	Title and abstract screening
4562	Sverre E., Peersen K., Perk J., Husebye E., Gullestad L., Dammen T., et al. Challenges in coronary heart disease prevention-experiences from a long-term follow-up study in Norway. Scand Cardiovasc J. 2021;55: 73–81. doi:10.1080/14017431.2020.1852308	No	Not meeting inclusion criteria	Title and abstract screening
4563	Swartz MD, DeSantis SM, Yaseen A, Brito FA, Valerio-Shewmaker MA, Messiah SE, et al. Antibody Duration After Infection From SARS-CoV-2 in the Texas Coronavirus Antibody Response Survey. J Infect Dis. 2023;227: 193–201. doi:10.1093/infdis/jiac167	No	Not meeting inclusion criteria	Title and abstract screening

4564	Sweet L, Brasky TM, Cooper S, Doogan N, Hinton A, Klein EG, et al. Quitting Behaviors Among Dual Cigarette and E-Cigarette Users and Cigarette Smokers Enrolled in the Tobacco User Adult Cohort. Nicotine Tob Res. 2019;21: 278–284. doi:10.1093/ntr/nty222	No	Not meeting inclusion criteria	Title and abstract screening
4565	Swetha T.V., Duraisamy R., Kumar M.P.S. Knowledge, attitude, and practices regarding smoking habits among school students in Chennai city. Drug Invent Today. 2018;10: 2033–2037.	No	Not meeting inclusion criteria	Title and abstract screening
4566	Sylvestre M-P, Dinkou GDT, Naja M, Riglea T, Pelekanakis A, Belanger M, et al. A longitudinal study of change in substance use from before to during the COVID-19 pandemic in young adults. Lancet Reg Health Am. 2022;8: 100168. doi:10.1016/j.lana.2021.100168	No	Not meeting inclusion criteria	Title and abstract screening
4567	Szilcz M, Wastesson JW, Morin L, Calderón-Larrañaga A, Lambe M, Johnell K. Potential overtreatment in end-of-life care in adults 65 years or older dying from cancer: applying quality indicators on nationwide registries. Acta Oncol. 2022;61: 1437–1445. doi:10.1080/0284186X.2022.2153621	No	Not meeting inclusion criteria	Title and abstract screening
4568	Szostak J., Wong E.T., Titz B., Lee T., Wong S.K., Low T., et al. A 6-month systems toxicology inhalation study in ApoE-/- mice demonstrates reduced cardiovascular effects of E-vapor aerosols compared with cigarette smoke. Am J Physiol - Heart Circ Physiol. 2020;318: H604–H631. doi:10.1152/ajpheart.00613.2019	No	Not meeting inclusion criteria	Title and abstract screening
4569	Szparaga M, Swiercz R, Stepnik M. Review of data on chemical content in an aerosol resulting from heating a tobacco or a solution used in e-cigarettes and in the smoke generated from the reference cigarettes. Toxicol Mech Methods. 2021;31: 323–333. doi:10.1080/15376516.2021.1884922	No	Not meeting inclusion criteria	Title and abstract screening
4570	Szymanski FM, Semczuk-Kaczmarek K, Kuna P. "Health outcomes in COPD smokers using heated tobacco products: a 3-year follow-up: comment". Intern Emerg Med. 2021;16: 2331–2333. doi:10.1007/s11739-021-02753-5	No	Not meeting inclusion criteria	Title and abstract screening
4571	Tabangcura KR, Taketa R, Kawamoto CT, Amin S, Sussman S, Okamoto SK, et al. Peer Crowds and Tobacco Product Use in Hawai'i: A Qualitative Study. Int J Env Res Public Health. 2023;20. doi:10.3390/ijerph20021029	No	Not meeting inclusion criteria	Title and abstract screening
4572	Tabibnia G, Ghahremani DG, Pochon JF, Diaz MP, London ED. Negative affect and craving during abstinence from smoking are both linked to default mode network connectivity. Drug Alcohol Depend. 2023;249: 109919. doi:10.1016/j.drugalcdep.2023.109919	No	Not meeting inclusion criteria	Title and abstract screening
4573	Tabuchi T. ES24.03 Current Status of Smoking Cessation with Heated Tobacco Products Use in Japan. J Thorac Oncol. 2021;16: S91. doi:10.1016/j.jtho.2021.01.053	No	Not meeting inclusion criteria	Title and abstract screening
4574	Tabuyo-Martin A, Torres-Morales A, Pitteloud MJ, Kshetry A, Oltmann C, Pearson JM, et al. Palliative Medicine Referral and End-of-Life Interventions Among Racial and Ethnic Minority Patients With Advanced or Recurrent Gynecologic Cancer. Cancer Control J Moffitt Cancer Cent. 2023; 1–9. doi:10.1177/10732748231157191	No	Not meeting inclusion criteria	Title and abstract screening
4575	Tackett AP, Han DH, Peraza N, Whaley RC, Mason T, Cahn R, et al. Effects of "Ice" flavoured e-cigarettes with synthetic cooling agent WS-23 or menthol on user-reported appeal and sensory attributes. Tob Control. 2023. doi:10.1136/tc-2023-058125	No	Not meeting inclusion criteria	Title and abstract screening
4576	Tackett AP, Wong M, Cho J, Harlow AF, Vogel EA, Han DH, et al. Willingness to Use Commercial Nicotine Gums, Lozenges, and Gummies Among Nontobacco Using Adolescents in Southern California. J Adolesc Health. 2023;72: 277–286. doi:10.1016/j.jadohealth.2022.09.027	No	Not meeting inclusion criteria	Title and abstract screening
4577	Taira T, Nozawa H, Kawai K, Sasaki K, Murono K, Emoto S, et al. Prognoses in Pathologically Confirmed T1 Lower Rectal Cancer Patients with or without Preoperative Therapy: An Analysis Using the Surveillance, Epidemiology, and End Results Database. Oncology. 2022;100: 82–88. doi:10.1159/000521033	No	Not meeting inclusion criteria	Title and abstract screening
4578	Tal O, Ben Shem E, Peled O, Elyashiv O, Levy T. Age Disparities in End of Life Symptom Management Among Patients with Epithelial Ovarian Cancer. J Palliat Care. 2023;38: 184–191. doi:10.1177/08258597221083418	No	Not meeting inclusion criteria	Title and abstract screening
4579	Talbot EM, Giovenco DP, Grana R, Hrywna M, Ganz O. Cross-promotion of nicotine pouches by leading cigarette brands. Tob Control. 2023;32: 528–529. doi:10.1136/tobaccocontrol-2021-056899	No	Not meeting inclusion criteria	Title and abstract screening
4580	Talih S, Hanna E, Salman R, Salam S, El-Hage R, Karaoghlanian N, et al. Influence of nicotine form and nicotine flux on puffing behavior and mouth-level exposure to nicotine from electronic nicotine delivery systems. Drug Alcohol Depend. 2023;254: 111052. doi:10.1016/j.drugalcdep.2023.111052	No	Not meeting inclusion criteria	Title and abstract screening
4581	Talih S, Salman R, Karam E, El-Hourani M, El-Hage R, Karaoghlanian N, et al. Hot Wires and Film Boiling: Another Look at Carbonyl Formation in Electronic Cigarettes. Chem Res Toxicol. 2020. doi:10.1021/acs.chemrestox.0c00196	No	Not meeting inclusion criteria	Title and abstract screening
4582	Tam J, Jaffri MA, Mok Y, Jeon J, Szklo AS, Souza MC, et al. Patterns of Birth Cohort–Specific Smoking Histories in Brazil. Am J Prev Med. 2023;64: S63–S71. doi:10.1016/j.amepre.2022.12.002	No	Not meeting inclusion criteria	Title and abstract screening
4583	Tam J, Jimenez Mendoza E, Buckell J, Sindelar J, Meza R. Responses to real-world and hypothetical e-cigarette flavor bans among US young adults who use flavored e-cigarettes. Nicotine Tob Res. 2023. doi:10.1093/ntr/ntad258	No	Not meeting inclusion criteria	Title and abstract screening
4584	Tam J, Jimenez Mendoza E, Buckell J, Sindelar J, Meza R. Responses to real-world and hypothetical menthol flavor bans among US young adults who smoke menthol cigarettes. Nicotine Tob Res. 2023. doi:10.1093/ntr/ntad259	No	Not meeting inclusion criteria	Title and abstract screening
4585	Tam J, Warner KE. Authors' response to letter to the editor by O'Loughlin and Wellman concerning "Students' cigarette smoking and the perceived nicotine content of their e-cigarettes". Am J Prev Med. 2018;55: 933. doi:10.1016/j.amepre.2018.08.011https://dx.doi.org/10.1016/j.amepre.2018.08.011	No	Not meeting inclusion criteria	Title and abstract screening
4586	Tami-Maury I., Garcia H., Onigbogi M., Ismael J., Manrique J., Vazquez V., et al. Smoking cessation knowledge and perceptions of cancer care providers at six Latin American cancer institutions. Rev Panam Salud PublicaPan Am J Public Health. 2022;46: e121. doi:10.26633/RPSP.2022.121	No	Not meeting inclusion criteria	Title and abstract screening
4587	Tan AS, Chen JT, Keen R, Scout N, Gordon B, Applegate J, et al. Culturally tailored anti-smoking messages: A randomized trial with U.S. sexual minority young women. Am J Prev Med. 2023. doi:10.1016/j.amepre.2023.12.001	No	Not meeting inclusion criteria	Title and abstract screening
4588	Tan H.T., Chai B.C., Lui Y.S. Effects of COVID-19 on Substance Use in Singapore. Subst Abuse Res Treat. 2021;15. doi:10.1177/11782218211030533	No	Not meeting inclusion criteria	Title and abstract screening
4589	Tan X., Vrana K., Ding Z.-M. Cotinine: Pharmacologically Active Metabolite of Nicotine and Neural Mechanisms for Its Actions. Front Behav Neurosci. 2021;15: 758252. doi:10.3389/fnbeh.2021.758252	No	Not meeting inclusion criteria	Title and abstract screening
4590	Tanaka A, Nagata C, Goto M, Adachi K. Nurse intervention process for the thoughts and concerns of people with cancer at the end of their life: A structural equation modeling approach. Nurs Health Sci. 2023;25: 150–160. doi:10.1111/nhs.13013	No	Not meeting inclusion criteria	Title and abstract screening
4591	Tang M-S, Tang Y-L. Can electronic-cigarette vaping cause cancer?. J Cancer Biol. 2021;2: 68–70. doi:10.46439/cancerbiology.2.027	No	Not meeting inclusion criteria	Title and abstract screening
4592	Tang MS, Lee HW, Weng MW, Wang HT, Hu Y, Chen LC, et al. DNA damage, DNA repair and carcinogenicity: Tobacco smoke versus electronic cigarette aerosol. Mutat Res Rev Mutat Res. 2022;789: 108409. doi:10.1016/j.mrrev.2021.108409	No	Not meeting inclusion criteria	Title and abstract screening

4593	Tang VM, Goud R, Zawertailo L, Selby P, Coroiu A, Sloan ME, et al. Repetitive transcranial magnetic stimulation for smoking cessation: Next steps for translation and implementation into clinical practice. Psychiatry Res. 2023;326: 115340. doi:10.1016/j.psychres.2023.115340	No	Not meeting inclusion criteria	Title and abstract screening
4594	Tang X., Benowitz N., Gundel L., Hang B., Havel C.M., Hoh E., et al. Thirdhand Exposures to Tobacco-Specific Nitrosamines through Inhalation, Dust Ingestion, Dermal Uptake, and Epidermal Chemistry. Environ Sci Technol. 2022;56: 12506–12516. doi:10.1021/acs.est.2c02559	No	Not meeting inclusion criteria	Title and abstract screening
4595	Taniyama T, Tokutani R, Hiramoto S. Risk factors of sudden unexpected death in patients with advanced cancer near the end of life. Palliat Support Care. 2022;20: 818–822. doi:10.1017/S1478951521001632	No	Not meeting inclusion criteria	Title and abstract screening
4596	Tanner NT, Thomas NA, Ward R, Rojewski A, Gebregziabher M, Toll BA, et al. Association of Cigarette Type and Nicotine Dependence in Patients Presenting for Lung Cancer Screening. Chest. 2020;158: 2184–2191. doi:10.1016/j.chest.2020.05.608	No	Not meeting inclusion criteria	Title and abstract screening
4597	Tannock IF, Pond GR, Booth CM. Biased Evaluation in Cancer Drug Trials—How Use of Progression-Free Survival as the Primary End Point Can Mislead. JAMA Oncol. 2022;8: 679–680. doi:10.1001/jamaoncol.2021.8206	No	Not meeting inclusion criteria	Title and abstract screening
4598	Tannous S, Darlot F, Cador M, Caille S. Flavor additives facilitate oral self-administration of nicotine solution in mice. Psychopharmacol Berl. 2021;238: 2235–2247. doi:10.1007/s00213-021-05848-1	No	Not meeting inclusion criteria	Title and abstract screening
4599	Tansey W, Li K, Zhang H, Linderman SW, Rabadan R, Blei DM, et al. Dose-response modeling in high-throughput cancer drug screenings: an end-to-end approach. Biostatistics. 2022;23: 643–665. doi:10.1093/biostatistics/kxaa047	No	Not meeting inclusion criteria	Title and abstract screening
4600	Tanski S, Halenar MJ, Edwards KC, Emond J, Woloshin S, Brunette M, et al. Tobacco Product Use and Functionally Important Respiratory Symptoms Among US Adolescents/Young Adults. Acad Pediatr. 2022;22: 1006–1016. doi:10.1016/j.acap.2022.03.001	No	Not meeting inclusion criteria	Title and abstract screening
4601	Tarren JR, Bartlett SE. Alcohol and nicotine interactions: pre-clinical models of dependence. Am J Drug Alcohol Abuse. 2017;43: 146–154. doi:10.1080/00952990.2016.1197232	No	Not meeting inclusion criteria	Title and abstract screening
4602	Tashkin DP, Tan WC. Inhaled Marijuana and the Lung. J Allergy Clin Immunol Pr. 2022;10: 2822–2829. doi:10.1016/j.jaip.2022.05.009	No	Not meeting inclusion criteria	Title and abstract screening
4603	Tattan-Birch H, Brown J, Jackson SE. 'Give 'em the vape, sell "em the pods": razor-and-blades methods of pod e-cigarette pricing. Tob Control. 2022;31: 773–774. doi:10.1136/tobaccocontrol-2020-056354	No	Not meeting inclusion criteria	Title and abstract screening
4604	Tattan-Birch H, Hartmann-Boyce J, Kock L, Simonavicius E, Brose L, Jackson S, et al. Heated tobacco products for smoking cessation and reducing smoking prevalence. Cochrane Database Syst Rev. 2022;1: CD013790. doi:10.1002/14651858.CD013790.pub2	No	Not meeting inclusion criteria	Title and abstract screening
4605	Tattan-Birch H, Jackson SE, Dockrell M, Brown J. Tobacco-free Nicotine Pouch Use in Great Britain: A Representative Population Survey 2020-2021. Nicotine Tob Res. 2022;24: 1509–1512. doi:10.1093/ntr/ntac099	No	Not meeting inclusion criteria	Title and abstract screening
4606	Tattan-Birch H, Jackson SE, Kock L, Dockrell M, Brown J. Rapid growth in disposable e-cigarette vaping among young adults in Great Britain from 2021 to 2022: a repeat cross-sectional survey. Addiction. 2022. doi:10.1111/add.16044	No	Not meeting inclusion criteria	Title and abstract screening
4607	Tattan-Birch H, Kock L, Brown J, Beard E, Bauld L, West R, et al. E-cigarettes to Augment Stop Smoking In-person Support and Treatment With Varenicline (E-ASSIST): A Pragmatic Randomized Controlled Trial. Nicotine Tob Res. 2023;25: 395–403. doi:10.1093/ntr/ntac149	No	Not meeting inclusion criteria	Title and abstract screening
4608	Tattan-Birch H, Shahab L. The psychobiology of nicotine vaping: Impact on addiction, cognition, mood, anxiety and appetite. Psychobiol Issues Subst Use Misuse. 2021; 265–288.	No	Not meeting inclusion criteria	Title and abstract screening
4609	Taylor E, Aleyan S, East K, Michael Cummings K, Thrasher JF, Fong GT, et al. Associations Between Noticing Nicotine Vaping Product Health Warning Labels, Harm Perceptions, and Use Among Adult Vapers, Current and Former Smokers. Findings From the 2018 ITC Four Country Smoking and Vaping Survey. Nicotine Tob Res. 2022;24: 1020–1027. doi:10.1093/ntr/ntab256	No	Not meeting inclusion criteria	Title and abstract screening
4610	Taylor E, Arnott D, Cheeseman H, Hammond D, Reid JL, McNeill A, et al. Association of Fully Branded and Standardized e-Cigarette Packaging With Interest in Trying Products Among Youths and Adults in Great Britain. JAMA Netw Open. 2023;6: e231799. doi:10.1001/jamanetworkopen.2023.1799	No	Not meeting inclusion criteria	Title and abstract screening
4611	Taylor E, Brose LS, McNeill A, Brown J, Kock L, Robson D. Associations between smoking and vaping prevalence, product use characteristics, and mental health diagnoses in Great Britain: a population survey. BMC Med. 2023;21: 211. doi:10.1186/s12916-023-02890-y	No	Not meeting inclusion criteria	Title and abstract screening
4612	Taylor EV, East KA, McNeill A, Cummings M, Thrasher J, Fong GT, et al. Changes in responses to nicotine vaping product warnings and leaflets in England compared with Canada, the US and Australia: findings from the 2016-2018 ITC Four Country Smoking and Vaping Surveys. Tob Control. 2022;31: 107–111. doi:10.1136/tobaccocontrol-2020-055739	No	Not meeting inclusion criteria	Title and abstract screening
4613	Taylor MR, Carrasco K, Carrasco A, Basu A. Tobacco and ADHD: A Role of MAO-Inhibition in Nicotine Dependence and Alleviation of ADHD Symptoms. Front Neurosci. 2022;16: 845646. doi:10.3389/fnins.2022.845646	No	Not meeting inclusion criteria	Title and abstract screening
4614	Tchaconas A., Truglio J. Diffuse alveolar hemorrhage as a complication of antiphospholipid syndrome. J Gen Intern Med. 2021;36: S324–S325. doi:10.1007/s11606-021-06830-5	No	Not meeting inclusion criteria	Title and abstract screening
4615	Tehrani MW, Newmeyer MN, Rule AM, Prasse C. Characterizing the Chemical Landscape in Commercial E-Cigarette Liquids and Aerosols by Liquid Chromatography-High-Resolution Mass Spectrometry. Chem Res Toxicol. 2021;34: 2216–2226. doi:10.1021/acs.chemrestox.1c00253	No	Not meeting inclusion criteria	Title and abstract screening
4616	Terentes-Printzios D., Ioakeimidis N., Vlachopoulos C., Abdelrassoul M., Georgakopoulos C., Gourgouli I., et al. Electronic cigarette smoking exerts an unfavourable effect on aortic hemodynamics and wave reflections. J Hypertens. 2018;36: e2–e3.	No	Not meeting inclusion criteria	Title and abstract screening
4617	Terry MB, Colditz GA. Epidemiology and Risk Factors for Breast Cancer: 21st Century Advances, Gaps to Address through Interdisciplinary Science. Cold Spring Harb Perspect Med. 2023;13. doi:10.1101/cshperspect.a041317	No	Not meeting inclusion criteria	Title and abstract screening
4618	Thandra KC, Barsouk A, Saginala K, Aluru JS, Barsouk A. Epidemiology of lung cancer. Contemp Oncol Poznan Pol. 2021;25: 45–52. doi:10.5114/wo.2021.103829	No	Not meeting inclusion criteria	Title and abstract screening
4619	The Lancet. Nicotine addiction, reduction, and smoking cessation. The Lancet. 2017;390: 716. doi:10.1016/S0140-6736%2817%2932240-7	No	Not meeting inclusion criteria	Title and abstract screening
4620	The Lancet. Philip Morris International: money over morality? The Lancet. 2019;394: 709. doi:10.1016/S0140-6736%2819%2931998-1	No	Not meeting inclusion criteria	Title and abstract screening
4621	The Lancet Oncology. E-cigarettes-new product, old tricks. Lancet Oncol. 2018;19: 1543. doi:10.1016/S1470-2045%2818%2930853-2	No	Not meeting inclusion criteria	Title and abstract screening

4622	The Lancet Oncology. Endocrine disruptors-the lessons (not) learned. Lancet Oncol. 2021;22: 1483. doi:10.1016/S1470-2045%2821%2900597-0	No	Not meeting inclusion criteria	Title and abstract screening
4623	The Lancet Respiratory Medicine. Evolution of e-cigarettes: vigilance is needed to protect adolescent health. Lancet Respir Med. 2020;8: 217. doi:10.1016/S2213-2600%2820%2930075-8	No	Not meeting inclusion criteria	Title and abstract screening
4624	The Lancet Respiratory Medicine. Pandemic fans the flames of smoking and health inequity. Lancet Respir Med. 2021;9: 435. doi:10.1016/S2213-2600%2821%2900180-6	No	Not meeting inclusion criteria	Title and abstract screening
4625	Theng Y.M., Wahab S., Wahab N.A.A., Sidi H., Das S. Schizophrenia and nicotine dependence: What psychopharmacological treatment options are available for the Duo Perturbationes? Curr Drug Targets. 2019;20: 173–181. doi:10.2174/1389450118666171017163741	No	Not meeting inclusion criteria	Title and abstract screening
4626	Thirion-Romero I, Perez-Padilla R, Zabert G, Barrientos-Gutierrez I. RESPIRATORY IMPACT OF ELECTRONIC CIGARETTES AND “LOW-RISK” TOBACCO. Rev Investig Clin Organo Hosp Enfermedades Nutr. 2019;71: 17–27. doi:10.24875/RIC.18002616	No	Not meeting inclusion criteria	Title and abstract screening
4627	Thirlway F. Nicotine addiction as a moral problem: Barriers to e-cigarette use for smoking cessation in two working-class areas in Northern England. Soc Sci Med. 2019;238: 112498. doi:10.1016/j.socscimed.2019.112498	No	Not meeting inclusion criteria	Title and abstract screening
4628	Thirlway F. Subculture wars: The struggle for the vape industry. Br J Sociol. 2022. doi:10.1111/1468-4446.12981	No	Not meeting inclusion criteria	Title and abstract screening
4629	Thirlway F. Subculture wars: The struggle for the vape industry. Br J Sociol. 2023;74: 3–16. doi:10.1111/1468-4446.12981	No	Not meeting inclusion criteria	Title and abstract screening
4630	Thomas DR, Fina LH, Adamson JP, Sawyer C, Jones A, Nnoaham K, et al. Social, demographic and behavioural determinants of SARS-CoV-2 infection: a case-control study carried out during mass community testing of asymptomatic individuals in South Wales, December 2020. Epidemiol Infect. 2022;150: e115. doi:10.1017/S0950268822000620	No	Not meeting inclusion criteria	Title and abstract screening
4631	Thomas J.L., Velazquez I.D. The Losing Battles in the Winning Campaign Against Cardiovascular Disease. Cardiovasc Revasc Med. 2021;26: 32–33. doi:10.1016/j.carrev.2021.02.005	No	Not meeting inclusion criteria	Title and abstract screening
4632	Thomas JE, Pasch KE, Nathan Marti C, Loukas A. Depressive symptoms prospectively increase risk for new onset cigarette and ENDS dependence symptoms. Addict Behav. 2024;148: 107870. doi:10.1016/j.addbeh.2023.107870	No	Not meeting inclusion criteria	Title and abstract screening
4633	Thomas KH, Dalili MN, López-López JA, Keeney E, Phillippo D, Munafò MR, et al. Smoking cessation medicines and e-cigarettes: a systematic review, network meta-analysis and cost-effectiveness analysis. Health Technol Assess. 2021;25: 1–224. doi:10.3310/hta25590	No	Not meeting inclusion criteria	Title and abstract screening
4634	Thomas KH, Dalili MN, López-López JA, Keeney E, Phillippo DM, Munafò MR, et al. Comparative clinical effectiveness and safety of tobacco cessation pharmacotherapies and electronic cigarettes: a systematic review and network meta-analysis of randomized controlled trials. Addiction. 2022;117: 861–876. doi:10.1111/add.15675	No	Not meeting inclusion criteria	Title and abstract screening
4635	Thomas R, Parker LS, Shiffman S. The Ethics of Tobacco Harm Reduction: An Analysis of E-Cigarette Availability From the Perspectives of Utilitarianism, Bioethics, and Public Health Ethics. Nicotine Tob Res Off J Soc Res Nicotine Tob. 2021;23: 3–8. doi:10.1093/ntr/ntaa198	No	Not meeting inclusion criteria	Title and abstract screening
4636	Thongsutt T, Yusote C, Jubprang S, Sasisuwan A, Poonchuay N, Chanawong A, et al. Factors Associated with Knowledge and Attitude towards E-Cigarettes among Undergraduate Students in Thailand: A Cross-Sectional Study. Asian Pac J Cancer Prev. 2023;24: 559–567. doi:10.31557/APJCP.2023.24.2.559	No	Not meeting inclusion criteria	Title and abstract screening
4637	Thorisdottir IE, Agustsson G, Oskarsdottir SY, Kristjansson AL, Asgeirsdottir BB, Sigfusdottir ID, et al. Effect of the COVID-19 pandemic on adolescent mental health and substance use up to March, 2022, in Iceland: a repeated, cross-sectional, population-based study. Lancet Child Adolesc Health. 2023;7: 347–357. doi:10.1016/S2352-4642(23)00022-6	No	Not meeting inclusion criteria	Title and abstract screening
4638	Thorisdottir IE, Asgeirsdottir BB, Kristjansson AL, Valdimarsdottir HB, Jonsdottir Tolgyes EM, Sigfusson J, et al. Depressive symptoms, mental wellbeing, and substance use among adolescents before and during the COVID-19 pandemic in Iceland: a longitudinal, population-based study. Lancet Psychiatry. 2021;8: 663–672. doi:10.1016/S2215-0366(21)00156-5	No	Not meeting inclusion criteria	Title and abstract screening
4639	Thorndike AN. E-Cigarette Use by Young Adult Nonsmokers: Next-Generation Nicotine Dependence? Ann Intern Med. 2019;170: 70–71. doi:10.7326/M18-2581	No	Not meeting inclusion criteria	Title and abstract screening
4640	Tidey JW, Davis DR, Miller ME, Pericot-Valverde I, Denlinger-Apte RL, Gaalema DE. Modeling nicotine regulation: A review of studies in smokers with mental health conditions. Prev Med. 2018;117: 30–37. doi:10.1016/j.ypmed.2018.07.003	No	Not meeting inclusion criteria	Title and abstract screening
4641	Tidey JW, Snell LM, Colby SM, Cassidy RN, Denlinger-Apte RL. Effects of very low nicotine content cigarettes on smoking across vulnerable populations. Prev Med. 2022; 107099. doi:10.1016/j.ypmed.2022.107099	No	Not meeting inclusion criteria	Title and abstract screening
4642	Tiede A, Baumann S, Krolo F, Gaertner B, John U, Freyer-Adam J. Health behaviors of general hospital patients with at-risk alcohol use: Change over two years after discharge. Gen Hosp Psychiatry. 2023;81: 76–81. doi:10.1016/j.genhosppsych.2023.02.002	No	Not meeting inclusion criteria	Title and abstract screening
4643	Tiefenthaler CM, Hirst JM, Edmonds KP, Atayee RS. Chlorpromazine’s Potential Role in Palliating Distressing Symptoms Associated With Hyperactive Delirium in Patients at End of Life. J Palliat Med. 2023;26: 826–830. doi:10.1089/jpm.2022.0308	No	Not meeting inclusion criteria	Title and abstract screening
4644	Tielbeek JJ, Vink JM, Polderman TJC, Popma A, Posthuma D, Verweij KJH. Genetic correlation of antisocial behaviour with alcohol, nicotine, and cannabis use. Drug Alcohol Depend. 2018;187: 296–299. doi:10.1016/j.drugalcdep.2018.03.020	No	Not meeting inclusion criteria	Title and abstract screening
4645	Tijdink J., Van Noorden M. Are e-cigarettes effective in the treatment of nicotine dependence? Tijdschr Voor Psychiatr. 2019;61: 427.	No	Not meeting inclusion criteria	Title and abstract screening
4646	Tildy BE, McNeill A, East K, Gravely S, Fong GT, Cummings KM, et al. Self-reported depression and anxiety and healthcare professional interactions regarding smoking cessation and nicotine vaping: Findings from 2018 International Tobacco Control Four Country Smoking and Vaping (ITC 4CV) Survey. Tob Prev Cessat. 2023;9: 26. doi:10.18332/tpc/168288	No	Not meeting inclusion criteria	Title and abstract screening
4647	Timmermans EJ, Veldhuizen EM, Snijder MB, Huisman M, Kunst AE. Neighbourhood safety and smoking in population subgroups: The HELIUS study. Prev Med. 2018;112: 111–118. doi:10.1016/j.ypmed.2018.04.012	No	Not meeting inclusion criteria	Title and abstract screening
4648	Tirelli U, Chirumbolo S. In Italy Banning Smoking Has Not Worked. How Crucial Are New Nicotine-Delivery Devices in Reducing Tobacco-Related Health Risk? Nicotine Tob Res. 2023;25: 1909–1911. doi:10.1093/ntr/ntad128	No	Not meeting inclusion criteria	Title and abstract screening
4649	Tituana N.Y., Clavijo C.G., Espinoza E.F., Tituana V.A. E-cigarette use-associated lung injury (EVALI). Pneumologie. 2023. doi:10.1055/a-2161-0105	No	Not meeting inclusion criteria	Title and abstract screening
4650	Tokle R. Varieties in vaper identitys in a Norwegian context. Tob Induc Dis. 2018;16: 90–91. doi:10.18332/tid/83800	No	Not meeting inclusion criteria	Title and abstract screening

4651	Tokle R. "Vaping and fidget-spinners": A qualitative, longitudinal study of e-cigarettes in adolescence. Int J Drug Policy. 2020;82: 102791. doi:10.1016/j.drugpo.2020.102791	No	Not meeting inclusion criteria	Title and abstract screening
4652	Tokle R, Brunborg GS, Vedoy TF. Adolescents' Use of Nicotine-Free and Nicotine E-Cigarettes: A Longitudinal Study of Vaping Transitions and Vaper Characteristics. Nicotine Tob Res Off J Soc Res Nicotine Tob. 2022;24: 400–407. doi:10.1093/ntr/ntab192	No	Not meeting inclusion criteria	Title and abstract screening
4653	Tokle R, Pedersen W. "Cloud chasers" and "substitutes": e-cigarettes, vaping subcultures and vaper identities. Sociol Health Illn. 2019;41: 917–932. doi:10.1111/1467-9566.12854	No	Not meeting inclusion criteria	Title and abstract screening
4654	Tolaney SM, Garrett-Mayer E, White J, Blinder VS, Foster JC, Amiri-Kordestani L, et al. Updated Standardized Definitions for Efficacy End Points (STEEP) in Adjuvant Breast Cancer Clinical Trials: STEEP Version 2.0. J Clin Oncol. 2021;39: 2720–2731. doi:10.1200/JCO.20.03613	No	Not meeting inclusion criteria	Title and abstract screening
4655	Tomaselli V, Ferrara P, Cantone GG, Romeo AC, Rust S, Saitta D, et al. The effect of laboratory-verified smoking on SARS-CoV-2 infection: results from the Troina sero-epidemiological survey. Intern Emerg Med. 2022;17: 1617–1630. doi:10.1007/s11739-022-02975-1	No	Not meeting inclusion criteria	Title and abstract screening
4656	Tomek S.E., Olive M.F. Preface. Int Rev Neurobiol. 2018;140: xi–xv. doi:10.1016/S0074-7742%2818%2930098-9	No	Not meeting inclusion criteria	Title and abstract screening
4657	Tomioka H, Wada T, Yamazoe M, Yoshizumi Y, Nishio C, Ishimoto G. Ten-year experience of smoking cessation in a single center in Japan. Respir Investig. 2019;57: 380–387. doi:10.1016/j.resinv.2019.01.007	No	Not meeting inclusion criteria	Title and abstract screening
4658	Tong EK, Zhu SH, Anderson CM, Avdalovic MV, Amin AN, Diamant AL, et al. Implementation, Maintenance, and Outcomes of an Electronic Referral to a Tobacco Quitline Across Five Health Systems. Nicotine Tob Res. 2023;25: 1135–1144. doi:10.1093/ntr/ntad008	No	Not meeting inclusion criteria	Title and abstract screening
4659	Tonstad S. E-cigarettes for smokers trying to quit. Eur Respir J. 2020;56: 2002934. doi:10.1183/13993003.02934-2020	No	Not meeting inclusion criteria	Title and abstract screening
4660	Tonstad S., Arons C., Rollema H., Berlin I., Hajek P., Fagerstrom K., et al. Varenicline: mode of action, efficacy, safety and accumulated experience salient for clinical populations. Curr Med Res Opin. 2020;36: 713–730. doi:10.1080/03007995.2020.1729708	No	Not meeting inclusion criteria	Title and abstract screening
4661	Toquet S., Cousson J., Choiselle N., Gozalo C., Giusti D., Bani-Sadr F., et al. Alveolar hemorrhage due to marijuana smoking using water pipe made with plastic bottle: case report and narrative review of the literature. Inhal Toxicol. 2021;33: 168–176. doi:10.1080/08958378.2021.1939465	No	Not meeting inclusion criteria	Title and abstract screening
4662	Torrens A., Ruiz C.M., Martinez M.X., Tagne A.M., Roy P., Grimes D., et al. Nasal accumulation and metabolism of DELTA9-tetrahydrocannabinol following aerosol ('vaping') administration in an adolescent rat model. Pharmacol Res. 2023;187: 106600. doi:10.1016/j.phrs.2022.106600	No	Not meeting inclusion criteria	Title and abstract screening
4663	Torres VA. Latent profiles of young adults based on e-cigarette outcome expectancies. Diss Abstr Int Sect B Sci Eng. 2023;84: No-Specified.	No	Not meeting inclusion criteria	Title and abstract screening
4664	Tosakoon S, Lawrence WR, Shiels MS, Jackson SS. Sex differences in cancer incidence rates by race and ethnicity: Results from the surveillance, epidemiology, and end results (SEER) Registry (2000-2019). J Clin Oncol. 41: 10547–10547. doi:10.1200/JCO.2023.41.16_suppl.10547	No	Not meeting inclusion criteria	Title and abstract screening
4665	Tosun NL, Allen SS, Eberly LE, Yao M, Stoops WW, Strickland JC, et al. Association of exercise with smoking-related symptomatology, smoking behavior and impulsivity in men and women. Drug Alcohol Depend. 2018;192: 29–37. doi:10.1016/j.drugalcdep.2018.07.022	No	Not meeting inclusion criteria	Title and abstract screening
4666	Tournebize J., Gibaja V., Grossenbacher F., Plenier Y., Medhioub Y., Batisse A., et al. Youth's hospitalizations supposedly related to e-cigarette use in the northeast of France: What's happened? Fundam Clin Pharmacol. 2021;35: 60–61. doi:10.1111/fcp.12671	No	Not meeting inclusion criteria	Title and abstract screening
4667	Tran DD, Davis JP, Ring C, Wang J, Fitzke RE, Leventhal AM, et al. Associations between depression, stress, and e-cigarette use among OEF/OIF veterans. Mil Psychol. 2023;35: 245–251. doi:10.1080/08995605.2022.2114278	No	Not meeting inclusion criteria	Title and abstract screening
4668	Tran DD, Prince MA, Fitzke RE, Ring C, Barrington-Trimis J, Pedersen ER. Preliminary development of the protective behavioral strategies for vaping scale. Addict Behav. 2023;144: 107728. doi:10.1016/j.addbeh.2023.107728	No	Not meeting inclusion criteria	Title and abstract screening
4669	Tran DD, Morrell HER. E-Cigarette Use: The Effects of Psychological Vulnerabilities, Perceptions, and Intentions to Use E-Cigarettes. Psychol Rep. 2023; 332941231161277. doi:10.1177/00332941231161277	No	Not meeting inclusion criteria	Title and abstract screening
4670	Tran Luy M, Airagnes G, Matta J, Toubiana L, Allagbe I, Limosin F, et al. Sociodemographic and Clinical Characteristics of Vapers Using E-Cigarettes Exclusively: The French Vapoquid Study. Subst Use Misuse. 2021;56: 2035–2043. doi:10.1080/10826084.2021.1967987	No	Not meeting inclusion criteria	Title and abstract screening
4671	Tran P., Jayawardene W., DeSalle M. E-cigarettes users' quit methods and interventions that may facilitate quitting. J Addict Med. 2021;15: E17. doi:10.1097/ADM.0000000000000902	No	Not meeting inclusion criteria	Title and abstract screening
4672	Tran T, Ickes MJ, Hester JW, Kavuluru R. Identifying current Juul users among emerging adults through Twitter feeds. Int J Med Inf. 2021;146: 104350. doi:10.1016/j.ijmedinf.2020.104350	No	Not meeting inclusion criteria	Title and abstract screening
4673	Trapl ES, Koopman Gonzalez SJ, Fryer CS. Adolescent dual-product users: Acquisition and situational use of cigarettes and cigars. Drug Alcohol Depend. 2018;188: 356–363. doi:10.1016/j.drugalcdep.2018.04.007	No	Not meeting inclusion criteria	Title and abstract screening
4674	Travaglini LE, Li L, Brown CH, Bennett ME. Predictors of smoking cessation group treatment engagement among veterans with serious mental illness. Addict Behav. 2017;75: 103–107. doi:10.1016/j.addbeh.2017.07.005	No	Not meeting inclusion criteria	Title and abstract screening
4675	Travis N, Knoll M, Cadham CJ, Cook S, Warner KE, Fleischer NL, et al. Health Effects of Electronic Cigarettes: An Umbrella Review and Methodological Considerations. Int J Env Res Public Health. 2022;19. doi:10.3390/ijerph19159054	No	Not meeting inclusion criteria	Title and abstract screening
4676	Travis N, Levy DT, McDaniel PA, Henriksen L. Tobacco retail availability and cigarette and e-cigarette use among youth and adults: a scoping review. Tob Control. 2022;31: e175–e188. doi:10.1136/tobaccocontrol-2020-056376	No	Not meeting inclusion criteria	Title and abstract screening
4677	Treloar Padovano H, Merrill JE, Colby SM, Kahler CW, Gwaltney CJ. Affective and Situational Precipitants of Smoking Lapses Among Adolescents. Nicotine Tob Res. 2020;22: 492–497. doi:10.1093/ntr/ntz002	No	Not meeting inclusion criteria	Title and abstract screening
4678	Tremblay B, Turk MT, Cooper MR, Zoucha R. Knowledge, attitudes, and perceptions of young adults about electronic nicotine delivery systems in the United States: An integrative review. J Cardiovasc Nurs. 2022;37: 86–97. doi:10.1097/JCN.0000000000000731https://dx.doi.org/10.1097/JCN.0000000000000731	No	Not meeting inclusion criteria	Title and abstract screening
4679	Trickett JS, Seaberg PH. 21-Year-Old Man With Fever, Night Sweats, Productive Cough, and Diarrhea. Mayo Clin Proc. 2021;96: 2486–2491. doi:10.1016/j.mayocp.2021.01.038	No	Not meeting inclusion criteria	Title and abstract screening

4680	Trigg J, Ela O, Bowden J, Bartram A, Cenko C, Bonevski B. Vaping harms awareness messaging: exploring young South Australians' responses to vaping prevention campaign materials. Health Promot Int. 2023;38. doi:10.1093/heapro/daad145	No	Not meeting inclusion criteria	Title and abstract screening
4681	Trivers KF, Watson CV, Neff LJ, Jones CM, Hacker K. Tetrahydrocannabinol (THC)-containing e-cigarette, or vaping, product use behaviors among adults after the onset of the 2019 outbreak of e-cigarette, or vaping, product use-associated lung injury (EVALI). Addict Behav. 2021;121: 106990. doi:10.1016/j.addbeh.2021.106990	No	Not meeting inclusion criteria	Title and abstract screening
4682	Troiano G, Nardi A. Instagrammers' attitude towards human papillomavirus (hpv) vaccine: A review. J Hum Behav Soc Environ. 2021; No-Specified. doi:10.1080/10911359.2021.1885554https://dx.doi.org/10.1080/10911359.2021.1885554	No	Not meeting inclusion criteria	Title and abstract screening
4683	Trueland J. Giving bad news and picking up the pieces: tips for cancer nurses: From initial diagnosis to end of life care plans, cancer nurses are likely to take a leading role in difficult conversations and emotional support for patients. Cancer Nurs Pract. 2022;21: 18–19. doi:10.7748/cnp.21.3.18.s10	No	Not meeting inclusion criteria	Title and abstract screening
4684	Truman P, Glover M, Fraser T. An Online Survey of New Zealand Vapers. Int J Env Res Public Health. 2018;15. doi:10.3390/ijerph15020222	No	Not meeting inclusion criteria	Title and abstract screening
4685	Truman P, Stanfill S, Heydari A, Silver E, Fowles J. Monoamine oxidase inhibitory activity of flavoured e-cigarette liquids. Neurotoxicology. 2019;75: 123–128. doi:10.1016/j.neuro.2019.09.010	No	Not meeting inclusion criteria	Title and abstract screening
4686	Truman P, Gilmour M, Robinson G. Acceptability of electronic cigarettes as an option to replace tobacco smoking for alcoholics admitted to hospital for detoxification. N Z Med J. 2018;131: 22–28.	No	Not meeting inclusion criteria	Title and abstract screening
4687	Trumbo CW. Influence of Risk Perception on Attitudes and Norms Regarding Electronic Cigarettes. Risk Anal Off Publ Soc Risk Anal. 2018;38: 906–916. doi:10.1111/risa.12918	No	Not meeting inclusion criteria	Title and abstract screening
4688	Tsai KYF, Hirschi Budge KM, Lepre AP, Rhees MS, Ajdaharian J, Geiler J, et al. Cell invasion, RAGE expression, and inflammation in oral squamous cell carcinoma (OSCC) cells exposed to e-cigarette flavoring. Clin Exp Dent Res. 2020;6: 618–625. doi:10.1002/cre2.314	No	Not meeting inclusion criteria	Title and abstract screening
4689	Tsao C.W., Aday A.W., Almarzooq Z.I., Alonso A., Beaton A.Z., Bittencourt M.S., et al. Heart Disease and Stroke Statistics-2022 Update: A Report from the American Heart Association. Circulation. 2022;145: E153–E639. doi:10.1161/CIR.0000000000001052	No	Not meeting inclusion criteria	Title and abstract screening
4690	Tsentemeidou A, Fyrmpas G, Stavrakas M, Vlachtsis K, Sotiriou E, Poutoglidis A, et al. Human Papillomavirus Vaccine to End Oropharyngeal Cancer. A Systematic Review and Meta-Analysis. Sex Transm Dis. 2021;48: 700–707. doi:10.1097/OLQ.0000000000001405	No	Not meeting inclusion criteria	Title and abstract screening
4691	Tsikrika S., Raftopoulou S., Nanou V., Kakavas S., Tzagkaraki A., Pouriki S., et al. Tobacco smoking and severity of COVID-19: Experience from ICU department in a referral hospital during the third pandemic wave. Eur Respir J. 2022;60. doi:10.1183/13993003.congress-2022.3128	No	Not meeting inclusion criteria	Title and abstract screening
4692	Tsou HH, Wang PH, Ting TH, Ping YH, Liu TY, Cheng HW, et al. Effect of heated tobacco products and traditional cigarettes on pulmonary toxicity and SARS-CoV-2-induced lung injury. Toxicology. 2022;479: 153318. doi:10.1016/j.tox.2022.153318	No	Not meeting inclusion criteria	Title and abstract screening
4693	Tsuguo Iwatani, Shinichi Noto, Koichiro Tsugawa. Changes in Health State Utility Values in Japanese Patients with End-Stage Breast Cancer. Curr Oncol. 2021;28: 4203–4212. doi:10.3390/curroncol28050356	No	Not meeting inclusion criteria	Title and abstract screening
4694	Tu Y-R, Tu K-H, Lee C-C, Fan P-C, Yen C-L, Wu VC-C, et al. Supplementation with Folic Acid and Cardiovascular Outcomes in End-Stage Kidney Disease: A Multi-Institution Cohort Study. Nutrients. 2022;14: 4162. doi:10.3390/nu14194162	No	Not meeting inclusion criteria	Title and abstract screening
4695	Tuchman AE. Advertising and demand for addictive goods: The effects of e-cigarette advertising. Mark Sci. 2019;38: 994–1022.	No	Not meeting inclusion criteria	Title and abstract screening
4696	Tuerlinckx Noguez P, Manfrin Muniz R, Vestena Zillmer JG. Narratives about self-care by people at the end of their lives. Av En Enfermeria. 2022;40: 63–76. doi:10.15446/av.enferm.v40n1.90912	No	Not meeting inclusion criteria	Title and abstract screening
4697	Turkistani YA, Dahlawi M, Bukhari RI, Aldabbagh M, Turkistani YA, Malosh A. Electronic Cigarette Prevalence and Knowledge Among Medical Students in Saudi Arabia and Bahrain: A Cross-National Study. Cureus. 2023;15: e45583. doi:10.7759/cureus.45583	No	Not meeting inclusion criteria	Title and abstract screening
4698	Uhl GR, Koob GF, Cable J. The neurobiology of addiction. Ann N Y Acad Sci. 2019;1451: 5–28. doi:10.1111/nyas.13989	No	Not meeting inclusion criteria	Title and abstract screening
4699	Umutlu S, Kocataş S. The effects of planned education given to parents on smoking in the home environment and passive exposure of children aged 0-5: A quasi-experimental pre-post intervention study from Turkey. J Pediatr Nurs. 2023;72: e228–e237. doi:10.1016/j.pedn.2023.07.023	No	Not meeting inclusion criteria	Title and abstract screening
4700	Unal M., Yalcin B.M., Selcuk M.Y. Are electronic cigarettes saviors or new culprits? J Exp Clin Med Turk. 2017;34: 15–19. doi:10.5835/jecm.omu.34.01.004	No	Not meeting inclusion criteria	Title and abstract screening
4701	Underner M, Peiffer G, Perriot J, Jaafari N. [COVID-19 and changes in electronic cigarette use]. Rev Mal Respir. 2021;38: 859–862. doi:10.1016/j.rmr.2021.05.009	No	Not meeting inclusion criteria	Title and abstract screening
4702	Underner M, Perriot J, Brousse G, de Chazeron I, Schmitt A, Peiffer G, et al. [Stopping and reducing smoking in patients with schizophrenia]. Encephale. 2019;45: 345–356. doi:10.1016/j.encep.2019.04.067	No	Not meeting inclusion criteria	Title and abstract screening
4703	Underner M., Perriot J., Peiffer G., Harika-Germaneau G., Jaafari N. Why stopping smoking is difficult in patients suffering from schizophrenia? How better to take care of them? Rev Med Liege. 2019;74: 23–27.	No	Not meeting inclusion criteria	Title and abstract screening
4704	Unger J.B., Urman R., Cruz T.B., Majmundar A., Barrington-Trimis J., Pentz M.A., et al. Talking about tobacco on Twitter is associated with tobacco product use. Prev Med. 2018;114: 54–56. doi:10.1016/j.ypmed.2018.06.006	No	Not meeting inclusion criteria	Title and abstract screening
4705	Unger JB, Falcon A. E-cigarette use among Hispanics: Reducing risk or recruiting new tobacco users?. Addict Behav. 2022;125: 107149. doi:10.1016/j.addbeh.2021.107149	No	Not meeting inclusion criteria	Title and abstract screening
4706	Unger JB, Rogers C, Barrington-Trimis J, Majmundar A, Sussman S, Allem J-P, et al. "I'm using cigarettes to quit JUUL": An analysis of Twitter posts about JUUL cessation. Addict Behav Rep. 2020;12: 100286. doi:10.1016/j.abrep.2020.100286	No	Not meeting inclusion criteria	Title and abstract screening
4707	Unger S, Golden SE, Melzer AC, Tanner N, Deepak J, Delorit M, et al. Study design for a proactive teachable moment tobacco treatment intervention among patients with pulmonary nodules. Contemp Clin Trials. 2022;121: 106908. doi:10.1016/j.cct.2022.106908	No	Not meeting inclusion criteria	Title and abstract screening
4708	Upadhyay S, Rahman M, Johanson G, Palmberg L, Ganguly K. Heated Tobacco Products: Insights into Composition and Toxicity. Toxics. 2023;11. doi:10.3390/toxics11080667	No	Not meeting inclusion criteria	Title and abstract screening

4709	Uranga C, Rosario J, Phung J, Tran Le, Banh D. IMPLEMENTATION OF END-OF-LIFE CARE TRAINING AT A COMPREHENSIVE CANCER CENTER...47th Annual Oncology Nursing Society Congress, April 27–May 1, 2022, Anaheim, CA. Oncol Nurs Forum. 2022;49: E95–E96. doi:10.1188/22.ONF.E2	No	Not meeting inclusion criteria	Title and abstract screening
4710	Urano T, Shiraki M, Imai T, Iinuma N, Kuroda T, Tanaka S, et al. Association of advanced glycation end-products levels with vascular events in postmenopausal women. Geriatr Gerontol Int. 2021;21: 651–656. doi:10.1111/ggi.14218	No	Not meeting inclusion criteria	Title and abstract screening
4711	US Preventive Services Task Force, Owens DK, Davidson KW, Krist AH, Barry MJ, Cabana M, et al. Primary Care Interventions for Prevention and Cessation of Tobacco Use in Children and Adolescents: US Preventive Services Task Force Recommendation Statement. JAMA. 2020;323: 1590–1598. doi:10.1001/jama.2020.4679	No	Not meeting inclusion criteria	Title and abstract screening
4712	Usidame B, Hirschtick J, Zavala-Arciniega L, Mattingly DT, Patel A, Meza R, et al. Exclusive and dual menthol/non-menthol cigarette use with ENDS among adults, 2013-2019. Prev Med Rep. 2021;24: 101566. doi:10.1016/j.pmedr.2021.101566	No	Not meeting inclusion criteria	Title and abstract screening
4713	Usidame B, Hirschtick JL, Mattingly DT, Patel A, Patrick ME, Fleischer NL. Sociodemographic Patterns of Exclusive and Dual Combustible Tobacco and E-Cigarette Use among US Adolescents-A Nationally Representative Study (2017-2020). Int J Env Res Public Health. 2022;19. doi:10.3390/ijerph19052965	No	Not meeting inclusion criteria	Title and abstract screening
4714	Usman S, Haseeb S, Khan MJ, Weber A. A 65-Year-Old Man With Concerns of Hemoptysis After Recent Motor Vehicle Accident and Blunt Trauma to Chest. Chest. 2023;164: e173–e176. doi:10.1016/j.chest.2023.06.034	No	Not meeting inclusion criteria	Title and abstract screening
4715	Uysal F.E., Gurgun A., Hassoy H., Guduk Z., Ciray N., Ergin I., et al. Evaluation of Smoking and Other Tobacco Product Habits of Ege University Faculty of Health Sciences Students: A cross-sectional Survey Study. Eur Respir J. 2020;56. doi:10.1183/13993003.congress-2020.1368	No	Not meeting inclusion criteria	Title and abstract screening
4716	Uysal M.A., Turan A. Can the fagerstrom test for nicotine dependence be used to evaluate the nicotine dependence in electronic cigarette users? Turk Thorac J. 2019;20: S110. doi:10.5152/TurkThoracJ.2019.110	No	Not meeting inclusion criteria	Title and abstract screening
4717	Vaithilingam S., Venkata A.N., Meena N.K. A 41-Year-Old Man Presenting With Shortness of Breath, Nausea, Vomiting, and Diarrhea. Chest. 2021;159: e87–e91. doi:10.1016/j.chest.2020.09.096	No	Not meeting inclusion criteria	Title and abstract screening
4718	Valera P, Acuna N, Vento I. The Preliminary Efficacy and Feasibility of Group-Based Smoking Cessation Treatment Program for Incarcerated Smokers. Am J Mens Health. 2020;14: 1557988320943357. doi:10.1177/1557988320943357	No	Not meeting inclusion criteria	Title and abstract screening
4719	Valera P, Owens M, Malarkey S, Acuna N. Exploring Tobacco and E-Cigarette Use among Queer Adults during the Early Days of the COVID-19 Pandemic. Int J Env Res Public Health. 2021;18. doi:10.3390/ijerph182412919	No	Not meeting inclusion criteria	Title and abstract screening
4720	Vallone DM, Cuccia AF, Briggs J, Xiao H, Schillo BA, Hair EC. Electronic Cigarette and JUUL Use Among Adolescents and Young Adults. JAMA Pediatr. 2020;174: 277–286. doi:10.1001/jamapediatrics.2019.5436	No	Not meeting inclusion criteria	Title and abstract screening
4721	Valter R, Guyonvarch O, Allagbe I, Le Faou AL. Factors associated with smoking cessation in exclusive smokers and dual users of e-cigarette and conventional cigarettes from CDTnet registry. Prev Med. 2023;173: 107585. doi:10.1016/j.ypmed.2023.107585	No	Not meeting inclusion criteria	Title and abstract screening
4722	van Amsterdam J, Vorspan F, Snijder MB, van den Brink W, Schene AH, Stronks K, et al. Use of the Fagerström test to assess differences in the degree of nicotine dependence in smokers from five ethnic groups: The HELIUS study. Drug Alcohol Depend. 2019;194: 197–204. doi:10.1016/j.drugalcdep.2018.10.011	No	Not meeting inclusion criteria	Title and abstract screening
4723	van Bavel N, Lai P, Loebenberg R, Prenner EJ. Vaping additives negatively impact the stability and lateral film organization of lung surfactant model systems. Nanomed. 2022;17: 827–843. doi:10.2217/nnm-2021-0398	No	Not meeting inclusion criteria	Title and abstract screening
4724	van der Heijden HS, Schirmbeck F, McGuire P, Valmaggia LR, Kempton MJ, van der Gaag M, et al. Association between tobacco use and symptomatology in individuals at ultra-high risk to develop a psychosis: A longitudinal study. Schizophr Res. 2021;236: 48–53. doi:10.1016/j.schres.2021.08.006	No	Not meeting inclusion criteria	Title and abstract screening
4725	van der Toorn M, Koshibu K, Schlage WK, Majeed S, Pospisil P, Hoeng J, et al. Comparison of monoamine oxidase inhibition by cigarettes and modified risk tobacco products. Toxicol Rep. 2019;6: 1206–1215. doi:10.1016/j.toxrep.2019.11.008	No	Not meeting inclusion criteria	Title and abstract screening
4726	van der Werff HFL, Michelet TH, Fredheim OM, Steine S. "Do not resuscitate" order and end-of-life treatment in a cohort of deceased in a Norwegian University Hospital. Acta Anaesthesiol Scand. 2022;66: 1009–1015. doi:10.1111/aas.14104	No	Not meeting inclusion criteria	Title and abstract screening
4727	Van Heel M., Van Gucht D., Vanbrabant K., Baeyens F. The importance of conditioned stimuli in cigarette and e-cigarette craving reduction by e-cigarettes. Int J Environ Res Public Health. 2017;14: 193. doi:10.3390/ijerph14020193	No	Not meeting inclusion criteria	Title and abstract screening
4728	van Klinken M, van Leeuwenhoek A. Palliative and End-of-Life Care for LGBT+ Cancer Patients: Scoping the Problems and Identifying the Solutions. Semin Oncol Nurs. 2023;39: N.PAG-N.PAG. doi:10.1016/j.soncn.2023.151500	No	Not meeting inclusion criteria	Title and abstract screening
4729	van Mourik D.-J.A., Nagelhout G.E., van den Putte B., Hummel K., Willemsen M.C., de Vries H. Did e-cigarette users notice the new european union's e-cigarette legislation? Findings from the 2015-2017 international tobacco control (ITC) Netherlands survey. Int J Environ Res Public Health. 2019;16: 2917. doi:10.3390/ijerph16162917	No	Not meeting inclusion criteria	Title and abstract screening
4730	Van Raemdonck D, Decaluwe H, De Leyn P, Nackaerts K. Commentary: Do we need to search for a patient's cigarettes or just count his or her wrinkles when counseling for lung cancer resection? J Thorac Cardiovasc Surg. 2021;162: 1387–1388. doi:10.1016/j.jtcvs.2020.12.050	No	Not meeting inclusion criteria	Title and abstract screening
4731	Van Schayck O.C.P., Williams S., Barchilon V., Baxter N., Jawad M., Katsaounou P.A., et al. Treating tobacco dependence: Guidance for primary care on life-saving interventions. Position statement of the IPCRG. Npj Prim Care Respir Med. 2017;27: 38. doi:10.1038/s41533-017-0039-5	No	Not meeting inclusion criteria	Title and abstract screening
4732	van Trier T.J., Mohammadnia N., Snaterse M., Peters R.J.G., Jorstad H.T., Bax W.A. Lifestyle management to prevent atherosclerotic cardiovascular disease: evidence and challenges. Neth Heart J. 2022;30: 3–14. doi:10.1007/s12471-021-01642-y	No	Not meeting inclusion criteria	Title and abstract screening
4733	Vanderkam P., Bonneau A., Kinouani S., Dzeraviashka P., Castera P., Besnier M., et al. Duration of the effectiveness of nicotine electronic cigarettes on smoking cessation and reduction: Systematic review and meta-analysis. Front Psychiatry. 2022;13: 915946. doi:10.3389/fpsyt.2022.915946	No	Not meeting inclusion criteria	Title and abstract screening
4734	Vanfrank B., Presley-Cantrell L. A Comprehensive Approach to Increase Adult Tobacco Cessation. JAMA - J Am Med Assoc. 2021;325: 232–233. doi:10.1001/jama.2020.23608	No	Not meeting inclusion criteria	Title and abstract screening
4735	Vaquero-Lozano P., Arroyo-Cozar M., Solano-Reina S., De Granda-Orive J.I., Jimenez-Ruiz C. Tobacco. Rev Patol Respir. 2018;21: 25–27.	No	Not meeting inclusion criteria	Title and abstract screening
4736	Vardavas CI, Girvalaki C, Odani S, Nikitara K, de Vries I, van Riel A, et al. Profile of incidental exposures to e-cigarette liquids in Europe, 2018-2019. Hum Exp Toxicol. 2021;40: 1045–1050. doi:10.1177/0960327120975828	No	Not meeting inclusion criteria	Title and abstract screening
4737	Vardy JL, Liew A, Warby A, Elder A, Keshet I, Devine R, et al. On the receiving end: have patient perceptions of the side-effects of cancer chemotherapy changed since the twentieth century? Support Care Cancer. 2022;30: 3503–3512. doi:10.1007/s00520-022-06804-1	No	Not meeting inclusion criteria	Title and abstract screening

4738	Vasileiadou S, Ekerljung L, Bjerg A, Goksör E. Asthma increased in young adults from 2008-2016 despite stable allergic rhinitis and reduced smoking. 2021;16: e0253322. doi:10.1371/journal.pone.0253322	No	Not meeting inclusion criteria	Title and abstract screening
4739	Vasiljevic Z, Scarpone M, Bergami M, Yoon J, van der Schaar M, Krljanac G, et al. Smoking and sex differences in first manifestation of cardiovascular disease. Atherosclerosis. 2021;330: 43–51. doi:10.1016/j.atherosclerosis.2021.06.909	No	Not meeting inclusion criteria	Title and abstract screening
4740	Vázquez-Nava F, Vázquez-Rodríguez EM, Vázquez-Rodríguez CF, Castillo Ruiz O, Peinado Herreros J. Epidemiological profile of smoking and nicotine addiction among asthmatic adolescents. Public Health. 2017;149: 49–56. doi:10.1016/j.puhe.2017.04.012	No	Not meeting inclusion criteria	Title and abstract screening
4741	Veldhuizen S, Behal A, Zawertailo L, Selby P. Adequacy of nicotine replacement and success quitting tobacco in clinical populations: An observational study. Drug Alcohol Depend. 2023;244: 109796. doi:10.1016/j.drugalcdep.2023.109796	No	Not meeting inclusion criteria	Title and abstract screening
4742	Veliz P, Eisman A, McCabe SE, Evans-Polce R, McCabe VV, Boyd CJ. E-Cigarette Use, Polytobacco Use, and Longitudinal Changes in Tobacco and Substance Use Disorder Symptoms Among U.S. Adolescents. J Adolesc Health. 2020;66: 18–26. doi:10.1016/j.jadohealth.2019.05.026	No	Not meeting inclusion criteria	Title and abstract screening
4743	Veliz P.T., Boyd C.J., Evans-Polce R.J., McCabe S.E. Increases in the Prevalence of Frequent E-Cigarette Use Among Adolescents. Am J Prev Med. 2020;59: 146–147. doi:10.1016/j.amepre.2020.02.004	No	Not meeting inclusion criteria	Title and abstract screening
4744	Vella V, Lappano R, Bonavita E, Maggiolini M, Clarke RB, Belfiore A, et al. Insulin/IGF Axis and the Receptor for Advanced Glycation End Products: Role in Meta-inflammation and Potential in Cancer Therapy. Endocr Rev. 2023;44: 693–723. doi:10.1210/endrev/bnad005	No	Not meeting inclusion criteria	Title and abstract screening
4745	Venditto L, Ferrante G, Caccin A, Franchini G, Zaffanello M, Tenero L, et al. Lung abscess as a complication of Lemierre Syndrome in adolescents: a single center case reports and review of the literature. Ital J Pediatr. 2023;49: 96. doi:10.1186/s13052-023-01499-4	No	Not meeting inclusion criteria	Title and abstract screening
4746	Vereen RN, Krajewski TJ, Wu EY, Zhang JH, Sanzo N, Noar SM. Aided recall of The Real Cost e-cigarette prevention advertisements among a nationally representative sample of adolescents. Prev Med Rep. 2022;28: 101864. doi:10.1016/j.pmedr.2022.101864	No	Not meeting inclusion criteria	Title and abstract screening
4747	Verhaegen A., Van Gaal L. Vaping and Cardiovascular Health: the Case for Health Policy Action. Curr Cardiovasc Risk Rep. 2019;13: 37. doi:10.1007/s12170-019-0634-9	No	Not meeting inclusion criteria	Title and abstract screening
4748	Verma A, Anand K, Bhargava M, Kolluri A, Kumar M, Palve DH. Comparative Evaluation of Salivary Biomarker Levels in e-Cigarette Smokers and Conventional Smokers. J Pharm Bioallied Sci. 2021;13: S1642–S1645. doi:10.4103/jpbs.jpbs_393_21	No	Not meeting inclusion criteria	Title and abstract screening
4749	Versace F, Kypriotakis G, Pluta D. Neuroaffective reactivity profiles are associated with vulnerability to e-cigarette use. Drug Alcohol Depend. 2023;247: 109871. doi:10.1016/j.drugalcdep.2023.109871	No	Not meeting inclusion criteria	Title and abstract screening
4750	Versace F., Stevens E.M., Robinson J.D., Cui Y., Deweese M.M., Engelmann J.M., et al. Brain responses to cigarette-related and emotional images in smokers during smoking cessation: No effect of varenicline or bupropion on the late positive potential. Nicotine Tob Res. 2019;21: 234–240. doi:10.1093/ntr/ntx264	No	Not meeting inclusion criteria	Title and abstract screening
4751	Versella MV, Leyro TM. Electronic cigarettes and nicotine harm-reduction. Curr Opin Psychol. 2019;30: 29–34. doi:10.1016/j.copsyc.2019.01.010	No	Not meeting inclusion criteria	Title and abstract screening
4752	Vevang KR, Zhang L, Grill AE, Hatsukami DK, Meier E, Nomura SO, et al. Furan Metabolites Are Elevated in Users of Various Tobacco Products and Cannabis. Chem Res Toxicol. 2023;36: 157–161. doi:10.1021/acs.chemrestox.2c00412	No	Not meeting inclusion criteria	Title and abstract screening
4753	Vickerman KA, Carpenter KM, Miles LN, Hsu JM, Watt KA, Brandon TH, et al. Treatment development, implementation, and participant baseline characteristics: A randomized pilot study of a tailored quitline intervention for individuals who smoke and vape. Contemp Clin Trials Commun. 2021;24: 100845. doi:10.1016/j.conctc.2021.100845	No	Not meeting inclusion criteria	Title and abstract screening
4754	Vickerman KA, Carpenter KM, Miles LN, Hsu JM, Watt KA, Brandon TH, et al. A Randomized Pilot of a Tailored Smoking Cessation Quitline Intervention for Individuals Who Smoke and Vape. Nicotine Tob Res. 2022;24: 1811–1820. doi:10.1093/ntr/ntac129	No	Not meeting inclusion criteria	Title and abstract screening
4755	Vijayaraghavan M, Elser H, Frazer K, Lindson N, Apollonio D. Interventions to reduce tobacco use in people experiencing homelessness. Cochrane Database Syst Rev. 2020;12: CD013413. doi:10.1002/14651858.CD013413.pub2	No	Not meeting inclusion criteria	Title and abstract screening
4756	Vijayaraghavan M, Hurst S, Pierce JP. A Qualitative Examination of Smoke-Free Policies and Electronic Cigarettes Among Sheltered Homeless Adults. Am J Health Promot. 2017;31: 243–250. doi:10.4278/ajhp.150318-QUAL-781	No	Not meeting inclusion criteria	Title and abstract screening
4757	Vijayaraghavan M., Elser H., Frazer K., Lindson N., Apollonio D. Interventions to reduce tobacco use in people experiencing homelessness. Cochrane Database Syst Rev. 2020;2020: CD013413. doi:10.1002/14651858.CD013413.pub2	No	Not meeting inclusion criteria	Title and abstract screening
4758	Villanti A.C., Byron M.J., Mercincavage M., Pacek L.R. Misperceptions of Nicotine and Nicotine Reduction: The Importance of Public Education to Maximize the Benefits of a Nicotine Reduction Standard. Nicotine Tob Res. 2019;21: S88–S90. doi:10.1093/ntr/ntz103	No	Not meeting inclusion criteria	Title and abstract screening
4759	Villanti A.C., Naud S., West J.C., Pearson J.L., Wackowski O.A., Niaura R.S., et al. Prevalence and correlates of nicotine and nicotine product perceptions in U.S. young adults, 2016. Addict Behav. 2019;98: 106020. doi:10.1016/j.addbeh.2019.06.009	No	Not meeting inclusion criteria	Title and abstract screening
4760	Villanti AC, Johnson AL, Ambrose BK, Cummings KM, Stanton CA, Rose SW, et al. Flavored Tobacco Product Use in Youth and Adults: Findings From the First Wave of the PATH Study (2013-2014). Am J Prev Med. 2017;53: 139–151. doi:10.1016/j.amepre.2017.01.026	No	Not meeting inclusion criteria	Title and abstract screening
4761	Villanti AC, Johnson AL, Halenar MJ, Sharma E, Cummings KM, Stanton CA, et al. Menthol and Mint Cigarettes and Cigars: Initiation and Progression in Youth, Young Adults and Adults in Waves 1-4 of the PATH Study, 2013-2017. Nicotine Tob Res. 2021;23: 1318–1326. doi:10.1093/ntr/ntaa224	No	Not meeting inclusion criteria	Title and abstract screening
4762	Villanti AC, Niaura RS, Pearson JL, Glasser AM, Abrams DB. Fostering transparency in e-cigarette research synthesis: the utility and limitations of methodological hierarchies. Addiction. 2018;113: 409–410. doi:10.1111/add.14143	No	Not meeting inclusion criteria	Title and abstract screening
4763	Villanti AC, Peasley-Miklus C, Cha S, Schulz J, Klemperer EM, LePine SE, et al. Tailored text message and web intervention for smoking cessation in U.S. socioeconomically-disadvantaged young adults: A randomized controlled trial. Prev Med. 2022; 107209. doi:10.1016/j.ypmed.2022.107209	No	Not meeting inclusion criteria	Title and abstract screening
4764	Villanti AC, Wackowski OA, LePine SE, West JC, Stevens EM, Unger JB, et al. Effects of Vaping Prevention Messages on Electronic Vapor Product Beliefs, Perceived Harms, and Behavioral Intentions among Young Adults: A Randomized Controlled Trial. Int J Env Res Public Health. 2022;19. doi:10.3390/ijerph192114182	No	Not meeting inclusion criteria	Title and abstract screening
4765	Villanti AC, West JC, Mays D, Donny EC, Cappella JN, Strasser AA. Impact of Brief Nicotine Messaging on Nicotine-Related Beliefs in a U.S. Sample. Am J Prev Med. 2019;57: e135–e142. doi:10.1016/j.amepre.2019.05.015	No	Not meeting inclusion criteria	Title and abstract screening
4766	Villanti A, Rath J, Williams V, Pearson J, Richardson A, Abrams D, et al. Impact of Exposure to Electronic Cigarette Advertising on Susceptibility and Trial of Electronic Cigarettes and Cigarettes in US Young Adults: a Randomized Controlled Trial. 2016;18: 1331-1339. doi:10.1093/ntr/ntv235	No	Not meeting inclusion criteria	Title and abstract screening

4767	Villanti AC, Naud S, West JC, Pearson JL, Wackowski OA, Hair E, et al. Latent Classes of Nicotine Beliefs Correlate with Perceived Susceptibility and Severity of Nicotine and Tobacco Products in US young adults. Nicotine Tob Res Off J Soc Res Nicotine Tob. 2019;21: S91–S100. doi:10.1093/ntr/ntz156	No	Not meeting inclusion criteria	Title and abstract screening
4768	Villanti AC, Peasley-Miklus C, Mercincavage M, Mays D, Donny EC, Cappella JN, et al. Effect of nicotine corrective messaging on nicotine-related beliefs in US adults: a randomised controlled trial. Tob Control. 2023. doi:10.1136/tc-2023-058252	No	Not meeting inclusion criteria	Title and abstract screening
4769	Villar P.G., Alhasan F., Lippmann S. Vaping: Safer Than Smoking? South Med J. 2020;113: 146. doi:10.14423/SMJ.0000000000001069	No	Not meeting inclusion criteria	Title and abstract screening
4770	Vincent D, Potts J, Durbin J, Moore J, Eley S. Electronic nicotine delivery systems, adolescent use, and nicotine addiction. Nurse Pract. 2019;44: 10–13. doi:10.1097/01.NPR.0000580804.82227.ce	No	Not meeting inclusion criteria	Title and abstract screening
4771	Vinchurkar S., Jain N., Punamiya V. Exploratory analysis of demographic factors and the temporal evolution of COVID-19 in India. J Epidemiol Glob Health. 2021;11: 10–14. doi:10.2991/JEGH.K.200921.001	No	Not meeting inclusion criteria	Title and abstract screening
4772	Vinci C, Sutton SK, Yang MJ, Baban S, Sauls R, Witkiewitz K, et al. Pilot randomized controlled trial of mindfulness-based relapse prevention vs cognitive behavioral therapy for smoking and alcohol use. Drug Alcohol Depend. 2023;244: 109768. doi:10.1016/j.drugalcdep.2023.109768	No	Not meeting inclusion criteria	Title and abstract screening
4773	Virgili F., Nenna R., Ben David S., Mancino E., Di Mattia G., Matera L., et al. E-cigarettes and youth: an unresolved Public Health concern. Ital J Pediatr. 2022;48: 97. doi:10.1186/s13052-022-01286-7	No	Not meeting inclusion criteria	Title and abstract screening
4774	Vishal Rao US, Arakeri G, Ravishankar S, Kar A, Thakur S, Li RJ, et al. The E-cigarette ban in India-A step in the right direction?. J Oral Pathol Med Off Publ Int Assoc Oral Pathol Am Acad Oral Pathol. 2020;49: 617–620. doi:10.1111/jop.13012	No	Not meeting inclusion criteria	Title and abstract screening
4775	Visseren F.L.J., MacH F., Crawford C., Davos C.H., DI Angelantonio E., Franco O.H., et al. 2021 ESC Guidelines on cardiovascular disease prevention in clinical practice. Eur Heart J. 2021;42: 3227–3337. doi:10.1093/eurheartj/ehab484	No	Not meeting inclusion criteria	Title and abstract screening
4776	Vitzthum K, Batra A, Pankow W, Raspe M, Rupp A, Andreas S. [Tobacco Cessation - The Underused Potential in Clinical Routine]. Pneumologie. 2022;76: 126–138. doi:10.1055/a-1524-4478	No	Not meeting inclusion criteria	Title and abstract screening
4777	Vitzthum K, Drazetic A, Markstein A, Rohde M, Pankow W, Mache S. Evaluation of long-term quitters: who stays smoke free forever?. Wien Med Wochenschr 1946. 2021;171: 330–334. doi:10.1007/s10354-020-00797-4	No	Not meeting inclusion criteria	Title and abstract screening
4778	Vivarelli F, Canistro D, Cirillo S, Elias RJ, Granata S, Mussoni M, et al. Unburned Tobacco Cigarette Smoke Alters Rat Ultrastructural Lung Airways and DNA. Nicotine Tob Res. 2021;23: 2127–2134. doi:10.1093/ntr/ntab108	No	Not meeting inclusion criteria	Title and abstract screening
4779	Vivarelli F, Granata S, Rullo L, Mussoni M, Candeletti S, Romualdi P, et al. On the toxicity of e-cigarettes consumption: Focus on pathological cellular mechanisms. Pharmacol Res. 2022;182: 106315. doi:10.1016/j.phrs.2022.106315	No	Not meeting inclusion criteria	Title and abstract screening
4780	Vivian Hui, Brazee R, Rosenzweig M, Young Ji Lee. The Association Between Hormone Receptor Status and End-of-Life Care Among Patients With Metastatic Breast Cancer. Clin J Oncol Nurs. 2022;26: 198–204. doi:10.1188/22.CJON.198-203	No	Not meeting inclusion criteria	Title and abstract screening
4781	Vlahos R. The vape has gone to your head. Brain Behav Immun. 2020;89: 5–6. doi:10.1016/j.bbi.2020.07.047	No	Not meeting inclusion criteria	Title and abstract screening
4782	Vlahos R. E-vaping and high-fat diet consumption: It's all a hazy memory. Brain Behav Immun. 2021;95: 23–24. doi:10.1016/j.bbi.2021.04.009	No	Not meeting inclusion criteria	Title and abstract screening
4783	Vogel B., Acevedo M., Appelman Y., Bairey Merz C.N., Chieffo A., Figtree G.A., et al. The Lancet women and cardiovascular disease Commission: reducing the global burden by 2030. The Lancet. 2021;397: 2385–2438. doi:10.1016/S0140-6736%2821%2900684-X	No	Not meeting inclusion criteria	Title and abstract screening
4784	Vogel EA, Barrington-Trimis JL, Harlow AF, Wong M, Cho J, Han DH, et al. Prevalence of and disparities in adolescents' susceptibility to novel oral nicotine products marketed as "tobacco-free". Prev Med. 2023;166: 107387. doi:10.1016/j.ypmed.2022.107387	No	Not meeting inclusion criteria	Title and abstract screening
4785	Vogel EA, Barrington-Trimis JL, Kechter A, Tackett AP, Liu F, Sussman S, et al. Differences in Young Adults' Perceptions of and Willingness to Use Nicotine Pouches by Tobacco Use Status. Int J Env Res Public Health. 2022;19. doi:10.3390/ijerph19052685	No	Not meeting inclusion criteria	Title and abstract screening
4786	Vogel EA, Benowitz NL, Skan J, Schnellbaecher M, Prochaska JJ. Correlates of the nicotine metabolite ratio in Alaska Native people who smoke cigarettes. Exp Clin Psychopharmacol. 2022;30: 359–364. doi:10.1037/pha0000461	No	Not meeting inclusion criteria	Title and abstract screening
4787	Vogel EA, Henriksen L, Schleicher NC, Prochaska JJ. Perceived Susceptibility to and Seriousness of COVID-19: Associations of Risk Perceptions with Changes in Smoking Behavior. Int J Env Res Public Health. 2021;18. doi:10.3390/ijerph18147621	No	Not meeting inclusion criteria	Title and abstract screening
4788	Vogel EA, Unger JB, Vassey J, Barrington-Trimis JL. Effects of a nicotine warning label and vaping cessation resources on young adults' perceptions of pro-vaping instagram influencer posts. Addict Behav. 2024;149: 107888. doi:10.1016/j.addbeh.2023.107888	No	Not meeting inclusion criteria	Title and abstract screening
4789	Vogel EA, Rebuli ME, Wong M, Leventhal A, Monterosso J, Tackett AP. Feasibility and acceptability of remote procedures to study tobacco product use and respiratory health: an observational study. BMJ Open. 2022;12: e065962. doi:10.1136/bmjopen-2022-065962	No	Not meeting inclusion criteria	Title and abstract screening
4790	Vogel M, Strasser H, Thorens G, Zullino D, Humair J-P, Simon O, et al. [Addictions]. Rev Med Suisse. 2020;16: 8–11.	No	Not meeting inclusion criteria	Title and abstract screening
4791	Vollstädt-Klein S, Grundinger N, Görig T, Szafran D, Althaus A, Mons U, et al. Study protocol: evaluation of the addictive potential of e-cigarettes (EVAPE): neurobiological, sociological, and epidemiological perspectives. BMC Psychol. 2021;9: 181. doi:10.1186/s40359-021-00682-8	No	Not meeting inclusion criteria	Title and abstract screening
4792	Voos N, Goniewicz ML, Eissenberg T. What is the nicotine delivery profile of electronic cigarettes? Expert Opin Drug Deliv. 2019;16: 1193–1203. doi:10.1080/17425247.2019.1665647	No	Not meeting inclusion criteria	Title and abstract screening
4793	Vrinten C, Parnham JC, Radó MK, Filippidis FT, Creese H, Hopkinson NS, et al. Patterns of cigarette and e-cigarette use among UK adolescents: a latent class analysis of the Millennium Cohort Study. Eur J Public Health. 2023;33: 857–863. doi:10.1093/eurpub/ckad124	No	Not meeting inclusion criteria	Title and abstract screening
4794	Vrinten C., Parnham J.C., Rado M.K., Filippidis F.T., Vamos E., Laverty A.A. Associations of social media use with smoking and e-cigarettes: a national longitudinal study. Lancet Lond Engl. 2023;402: S91. doi:10.1016/S0140-6736%2823%2902125-6	No	Not meeting inclusion criteria	Title and abstract screening
4795	Vrinten C, Parnham JC, Rado MK, Filippidis FT, Vamos E, Laverty AA. Associations of social media use with smoking and e-cigarettes: a national longitudinal study. Lancet Lond Engl. 2023;402 Suppl 1: S91. doi:10.1016/S0140-6736(23)02125-6	No	Not meeting inclusion criteria	Title and abstract screening

4796	Vu T.-H.T., Hart J.L., Groom A., Landry R.L., Walker K.L., Giachello A.L., et al. Age differences in electronic nicotine delivery systems (ENDS) usage motivations and behaviors, perceived health benefit, and intention to quit. Addict Behav. 2019;98: 106054. doi:10.1016/j.addbeh.2019.106054	No	Not meeting inclusion criteria	Title and abstract screening
4797	Vu TT, Dario JP, Mateu-Gelabert P, Levine D, Punter MA, Borrell LN, et al. Substance Use Patterns and Their Association with Depression and Social Factors During COVID-19 Among Harlem Residents in New York City. J Community Health. 2023;48: 937–944. doi:10.1007/s10900-023-01253-1	No	Not meeting inclusion criteria	Title and abstract screening
4798	Vukkadala N, Fardeen T, Ramchandran K, Divi V. End-of-Life Practice Patterns in Head and Neck Cancer. Laryngoscope. 2021;131: 1769–1773. doi:10.1002/lary.29423	No	Not meeting inclusion criteria	Title and abstract screening
4799	Vuong JT, Ruedisueli I, Beaudin CS, Middlekauff HR. Electronic Cigarettes: an Overlooked Tool to Alleviate Disparities in Tobacco Use Disorder Among People with Mental Health and Substance Use Disorders. J Gen Intern Med. 2023;38: 1970–1974. doi:10.1007/s11606-023-08137-z	No	Not meeting inclusion criteria	Title and abstract screening
4800	Wackowski OA, Bover Manderski MT, Gratale SK, Weiger CV, O'Connor RJ. Perceptions about levels of harmful chemicals in e-cigarettes relative to cigarettes, and associations with relative e-cigarette harm perceptions, e-cigarette use and interest. Addiction. 2023;118: 1881–1891. doi:10.1111/add.16258	No	Not meeting inclusion criteria	Title and abstract screening
4801	Wackowski OA, Gratale SK, Jeong M, Delnevo CD, Steinberg MB, O'Connor RJ. Over 1 year later: smokers' EVALI awareness, knowledge and perceived impact on e-cigarette interest. Tob Control. 2022. doi:10.1136/tobaccocontrol-2021-057190	No	Not meeting inclusion criteria	Title and abstract screening
4802	Wackowski OA, O'Connor RJ, Diaz D, Rashid M, Lewis MJ, Greene K. "95% less harmful"? Exploring reactions to quantitative modified risk claims for snus and e-cigarettes. Tob Control. 2022;31: 730–736. doi:10.1136/tobaccocontrol-2020-056303	No	Not meeting inclusion criteria	Title and abstract screening
4803	Wackowski OA, Sontag JM, Hammond D, O'Connor RJ, Ohman-Strickland PA, Strasser AA, et al. The Impact of E-Cigarette Warnings, Warning Themes and Inclusion of Relative Harm Statements on Young Adults' E-Cigarette Perceptions and Use Intentions. Int J Env Res Public Health. 2019;16. doi:10.3390/ijerph16020184	No	Not meeting inclusion criteria	Title and abstract screening
4804	Wackowski O, Sontag J, Hammond D, O'connor R, Ohman-Strickland P, Strasser A, et al. The impact of E-cigarette warnings, warning themes and inclusion of relative harm statements on young adultsâ€™ e-cigarette perceptions and use intentions. 2019;16. doi:10.3390/ijerph16020184	No	Not meeting inclusion criteria	Title and abstract screening
4805	Wackowski OA, Gratale SK, Jeong M, Delnevo CD, Steinberg MB, O'Connor RJ. Over 1 year later: Smokers' EVALI awareness, knowledge and perceived impact on e-cigarette interest. Tob Control Int J. 2023;32: e255–e259.	No	Not meeting inclusion criteria	Title and abstract screening
4806	Wackowski OA, Hammond D, O'Connor RJ, Strasser AA, Delnevo CD. Considerations and Future Research Directions for E-Cigarette Warnings-Findings from Expert Interviews. Int J Environ Res Public Health. 2017;14. doi:10.3390/ijerph14070781	No	Not meeting inclusion criteria	Title and abstract screening
4807	Wada T, Adachi Y, Murakami S, Ito Y, Itazawa T, Tsuchida A, et al. Maternal exposure to smoking and infant's wheeze and asthma: Japan Environment and Children's Study. Allergol Int. 2021;70: 445–451. doi:10.1016/j.alit.2021.04.008	No	Not meeting inclusion criteria	Title and abstract screening
4808	Wagener TL, Avery JA, Leavens ELS, Simmons WK. Associated Changes in E-cigarette Puff Duration and Cigarettes Smoked per Day. Nicotine Tob Res. 2021;23: 760–764. doi:10.1093/ntr/ntaa211	No	Not meeting inclusion criteria	Title and abstract screening
4809	Waks AG, Tarantino P, Freedman RA, Lin NU, Tayob N, Vallejo CT, et al. Outcomes according to treatment received for small node-negative HER2+ breast tumors in the Surveillance, Epidemiology, and End Results (SEER) database, 2010-2019. J Clin Oncol. 41: 517–517. doi:10.1200/JCO.2023.41.16_suppl.517	No	Not meeting inclusion criteria	Title and abstract screening
4810	Walayat A, Li Y, Zhang Y, Fu Y, Liu B, Shao XM, et al. Fetal e-cigarette exposure programs a neonatal brain hypoxic-ischemic sensitive phenotype via altering DNA methylation patterns and autophagy signaling pathway. Am J Physiol Regul Integr Comp Physiol. 2021;321: R791–R801. doi:10.1152/ajpregu.00207.2021	No	Not meeting inclusion criteria	Title and abstract screening
4811	WALBRIDGE G., MOREIRA SANCHES PERACI M., R. GORGY N., ZAKHARY J.O.H.N. E-CIGARETTE OR VAPING USE-ASSOCIATED LUNG INJURY (EVALI): EFFECTS OF VAPING OR COVID-19? Chest. 2022;162: A312. doi:10.1016/j.chest.2022.08.241	No	Not meeting inclusion criteria	Title and abstract screening
4812	Walele T, Bush J, Koch A, Savioz R, Martin C, O'Connell G. Evaluation of the safety profile of an electronic vapour product used for two years by smokers in a real-life setting. Regul Toxicol Pharmacol. 2018;92: 226–238. doi:10.1016/j.yrtph.2017.12.010	No	Not meeting inclusion criteria	Title and abstract screening
4813	Walia JY, Harocopos GJ, Hong AR. High-risk human papillomavirus-associated corneal/conjunctival intraepithelial neoplasia in a young patient. Am J Ophthalmol Case Rep. 2023;32: 101942. doi:10.1016/j.ajoc.2023.101942	No	Not meeting inclusion criteria	Title and abstract screening
4814	Walicka M, Russo C, Baxter M, John I, Caci G, Polosa R. Impact of stopping smoking on metabolic parameters in diabetes mellitus: A scoping review. World J Diabetes. 2022;13: 422–433. doi:10.4239/wjd.v13.i6.422	No	Not meeting inclusion criteria	Title and abstract screening
4815	Walker MW, Navarro M, Roditis M, Dineva AN. Adolescent risk perceptions of ENDS use: Room for change in tobacco education. Prev Med Rep. 2022;26: 101719. doi:10.1016/j.pmedr.2022.101719	No	Not meeting inclusion criteria	Title and abstract screening
4816	Walker N, Parag V, Wong SF, Youdan B, Broughton B, Bullen C, et al. Use of e-cigarettes and smoked tobacco in youth aged 14-15 years in New Zealand: findings from repeated cross-sectional studies (2014-19). Lancet Public Health. 2020;5: e204–e212. doi:10.1016/S2468-2667(19)30241-5	No	Not meeting inclusion criteria	Title and abstract screening
4817	Walker N., Parag V., Verbiest M., Laking G., Laugesen M., Bullen C. Nicotine patches with e-cigarettes for smoking cessation: Twitter discussion from a respirology journal club - Authors' reply. Lancet Respir Med. 2020;8: e9. doi:10.1016/S2213-2600%2820%2930073-4	No	Not meeting inclusion criteria	Title and abstract screening
4818	Wall A., Roslin S., Borg B., McDermott S., Walele T., Nahde T., et al. E-Cigarette Aerosol Deposition and Disposition of [11C]Nicotine Using Positron Emission Tomography: A Comparison of Nicotine Uptake in Lungs and Brain Using Two Different Nicotine Formulations. Pharmaceuticals. 2022;15: 367. doi:10.3390/ph15030367	No	Not meeting inclusion criteria	Title and abstract screening
4819	Wallace A.M., Foronjy R.E. Electronic cigarettes: Not evidence-based cessation. Transl Lung Cancer Res. 2019;8: S7–S10. doi:10.21037/tlcr.2019.03.08	No	Not meeting inclusion criteria	Title and abstract screening
4820	Walley S.C., Jenssen B., Wilson K. The intersection of tobacco and marijuana use in adolescents and young adults. Pediatrics. 2019;144: e20193025. doi:10.1542/peds.2019-3025	No	Not meeting inclusion criteria	Title and abstract screening
4821	Walley SC, Wilson KM, Winickoff JP, Groner J. A Public Health Crisis: Electronic Cigarettes, Vape, and JUUL. Pediatrics. 2019;143. doi:10.1542/peds.2018-2741	No	Not meeting inclusion criteria	Title and abstract screening
4822	Walsh CA, Good J, Ismaiel A, Yarborough S, Shen MJ. Development and refinement of a novel end-of-life planning website for patients with advanced cancer: a mixed methods approach. Support Care Cancer. 2023;31: 1–12. doi:10.1007/s00520-023-08153-z	No	Not meeting inclusion criteria	Title and abstract screening
4823	Walters KJ, Gray KM, Gex KS, McClure EA. The role of emotion differentiation in the association between momentary affect and tobacco/nicotine craving in young adults. Nicotine Tob Res Off J Soc Res Nicotine Tob. 2023. doi:10.1093/ntr/ntad001	No	Not meeting inclusion criteria	Title and abstract screening
4824	Wanar A, Morrison TM, Standish KR, Colson ER, Drainoni ML, Colvin BN, et al. Understanding cigarette smoking among mothers with opioid use disorder. J Subst Use Addict Treat. 2023;150: 209065. doi:10.1016/j.josat.2023.209065	No	Not meeting inclusion criteria	Title and abstract screening

4825	Wanchoo K, Abrams M, Merchant RM, Ungar L, Guntuku SC. Reddit language indicates changes associated with diet, physical activity, substance use, and smoking during COVID-19. PLoS One. 2023;18: e0280337. doi:10.1371/journal.pone.0280337	No	Not meeting inclusion criteria	Title and abstract screening
4826	Wang H, Chen H, Huang L, Li X, Wang L, Li S, et al. In vitro toxicological evaluation of a tobacco heating product THP COO and 3R4F research reference cigarette on human lung cancer cells. Toxicol Vitro. 2021;74: 105173. doi:10.1016/j.tiv.2021.105173	No	Not meeting inclusion criteria	Title and abstract screening
4827	Wang J, Tang J, Liu X, He D. A web-based prognostic nomogram for the cancer specific survival of elderly patients with T1-T3N0M0 renal pelvic transitional cell carcinoma based on the surveillance, epidemiology, and end results database. BMC Urol. 2022;22: 1–11. doi:10.1186/s12894-022-01028-1	No	Not meeting inclusion criteria	Title and abstract screening
4828	Wang K, Liu Y, Ouedraogo Y, Wang N, Xie X, Xu C, et al. Principal component analysis of early alcohol, drug and tobacco use with major depressive disorder in US adults. J Psychiatr Res. 2018;100: 113–120. doi:10.1016/j.jpsychires.2018.02.022	No	Not meeting inclusion criteria	Title and abstract screening
4829	Wang L, Wu M, Li R, Xu X, Zhu C, Feng X. MVI-Mind: A Novel Deep-Learning Strategy Using Computed Tomography (CT)-Based Radiomics for End-to-End High Efficiency Prediction of Microvascular Invasion in Hepatocellular Carcinoma. Cancers. 2022;14: 2956-N.PAG. doi:10.3390/cancers14122956	No	Not meeting inclusion criteria	Title and abstract screening
4830	Wang L, Du L, Xiong X, Lin Y, Zhu J, Yao Z, et al. Repurposing dextromethorphan and metformin for treating nicotine-induced cancer by directly targeting CHRNA7 to inhibit JAK2/STAT3/SOX2 signaling. Oncogene. 2021;40: 1974–1987. doi:10.1038/s41388-021-01682-z	No	Not meeting inclusion criteria	Title and abstract screening
4831	Wang MP, Li WH, Wu Y, Lam TH, Chan SS. Electronic cigarette use is not associated with quitting of conventional cigarettes in youth smokers. Pediatr Res. 2017;82: 14–18. doi:10.1038/pr.2017.80	No	Not meeting inclusion criteria	Title and abstract screening
4832	Wang Q., Hsu M.L., Wen C., Dowlati A., Zhang Y., Jiang C., et al. 1698MO E-cigarette use among us ever-smoking cancer survivors and its implications on lung cancer screening. Ann Oncol. 2023;34: S928. doi:10.1016/j.annonc.2023.09.2652	No	Not meeting inclusion criteria	Title and abstract screening
4833	Wang Q., Sundar I., Blum J.L., Ratner J.R., Lucas J.H., Chuang T., et al. Prenatal e-cig aerosol exposure leads to extracellular matrix remodeling and dysregulated myogenesis in offspring mice with sex- dependent manner. Am J Respir Crit Care Med. 2020;201. Available: https://www.atsjournals.org/doi/abs/10.1164/ajrccm-conference.2020.201.1_MeetingAbstracts.A4603	No	Not meeting inclusion criteria	Title and abstract screening
4834	Wang Q, Alpert N, Zhang Y, Tran JH, Jiang C, Wang X, et al. EP07.01-002 Surgery in Stage I-III Malignant Pleural Mesothelioma: A Surveillance, Epidemiology, and End Results (SEER)- Medicare Analysis 1995-2015. J Thorac Oncol. 2022;17: S312–S313. doi:10.1016/j.jtho.2022.07.534	No	Not meeting inclusion criteria	Title and abstract screening
4835	Wang Q, Du W, Wang H, Geng P, Sun Y, Zhang J, et al. Nicotine's effect on cognition, a friend or foe?. Prog Neuropsychopharmacol Biol Psychiatry. 2023;124: 110723. doi:10.1016/j.pnpbp.2023.110723	No	Not meeting inclusion criteria	Title and abstract screening
4836	Wang R., Hall J.M., Salloum R.G., Kates F., Cogle C.R., Bruijnzeel A.W., et al. Prevalence of Underreported Nicotine Exposure Among US Non-Smoking Adults: A Comparison of Self-Reported Exposure and Serum Cotinine Levels from NHANES 2013-2020. Nicotine Tob Res Off J Soc Res Nicotine Tob. 2023. doi:10.1093/ntr/ntad165	No	Not meeting inclusion criteria	Title and abstract screening
4837	Wang S, Shafique S, Xiao D, Barker K, Wang K, Xie X. Clusters of substance use and mental health variables with emergency room visits in U.S. adults: The 2020 National Survey on Drug Use and Health. J Affect Disord. 2023;339: 683–690. doi:10.1016/j.jad.2023.07.017	No	Not meeting inclusion criteria	Title and abstract screening
4838	Wang TW, Asman K, Gentzke AS, Cullen KA, Holder-Hayes E, Reyes-Guzman C, et al. Tobacco Product Use Among Adults - United States, 2017. MMWR Morb Mortal Wkly Rep. 2018;67: 1225–1232. doi:10.15585/mmwr.mm6744a2	No	Not meeting inclusion criteria	Title and abstract screening
4839	Wang TW, Walton K, Reyes-Guzman C, Cullen KA, Jamal A. State-Specific Prevalence of Tobacco Product Use Among US Women, Tobacco Use Supplement to the Current Population Survey, 2018-2019. Prev Chronic Dis. 2021;18: E36. doi:10.5888/pcd18.200547	No	Not meeting inclusion criteria	Title and abstract screening
4840	Wang X, Kim Y, Trivers KF, Tynan MA, Shrestha SS, Emery S, et al. Changes in Sales of E-Cigarettes, Cigarettes, and Nicotine Replacement Therapy Products Before, During, and After the EVALI Outbreak. Prev Chronic Dis. 2022;19: E86. doi:10.5888/pcd19.220087	No	Not meeting inclusion criteria	Title and abstract screening
4841	Wang X, McCaw ZR, Tian L, Stinchcombe TE, Vokes E, Ludmir EB, et al. Using a Clinically Interpretable End Point Composed of Multiple Outcomes to Evaluate Totality of Treatment Effect in Comparative Oncology Studies. JAMA Netw Open. 2023;6: e2319055–e2319055. doi:10.1001/jamanetworkopen.2023.19055	No	Not meeting inclusion criteria	Title and abstract screening
4842	Wang X, Wang Q, Wei D, Chang X. Association Between Soluble Receptor for Advanced Glycation End Product and Endogenous Secretory Soluble Receptor for Advanced Glycation End Product Levels and Carotid Atherosclerosis in Diabetes: A Systematic Review and Meta-Analysis. Can J Diabetes. 2021;45: 634–640. doi:10.1016/j.jcjd.2021.01.004	No	Not meeting inclusion criteria	Title and abstract screening
4843	Wang X, Ghimire R, Shrestha SS, Borowiecki M, Emery S, Trivers KF. Trends in Nicotine Strength in Electronic Cigarettes Sold in the United States by Flavor, Product Type, and Manufacturer, 2017-2022. Nicotine Tob Res Off J Soc Res Nicotine Tob. 2023;25: 1355–1360. doi:10.1093/ntr/ntad033	No	Not meeting inclusion criteria	Title and abstract screening
4844	Wang Y, Duan Z, Romm KF, Ma Y, Douglas Evans W, Bennett B, et al. Bidirectional associations between depressive symptoms and cigarette, e-cigarette, cannabis, and alcohol use: Cross-lagged panel analyses among young adults before and during COVID-19. Addict Behav. 2022;134: 107422. doi:10.1016/j.addbeh.2022.107422	No	Not meeting inclusion criteria	Title and abstract screening
4845	Wang Y, Duan Z, Weaver SR, Popova L, Spears CA, Ashley DL, et al. Consumption of JUUL vs. Other E-Cigarette Brands among U.S. E-Cigarette Users: Evidence from Wave 5 of the PATH Study. Int J Env Res Public Health. 2022;19. doi:10.3390/ijerph191710837	No	Not meeting inclusion criteria	Title and abstract screening
4846	Wang Y, Xu S, Zhang X, Zhang Y, Feng Y, Chen R. Effects of Tobacco Versus Electronic Cigarette Usage on Nonsuicidal Self-Injury and Suicidality Among Chinese Youth: Cross-Sectional Self-Report Survey Study. JMIR Public Health Surveill. 2023;9: e47058. doi:10.2196/47058	No	Not meeting inclusion criteria	Title and abstract screening
4847	Wang Y, Xu YA, Wu J, Kim HM, Fetterman JL, Hong T, et al. Moralization of E-cigarette Use and Regulation: A Mixed-Method Computational Analysis of Opinion Polarization. Health Commun. 2023;38: 1666–1676. doi:10.1080/10410236.2022.2027640	No	Not meeting inclusion criteria	Title and abstract screening
4848	Wang Y, LoParco CR, Cui Y, Duan Z, Bar-Zeev Y, Levine H, et al. Profiles of tobacco product use and related consumer characteristics in the US and Israel: A multiple-group latent class analysis. Glob Public Health. 2023;18: 2267652. doi:10.1080/17441692.2023.2267652	No	Not meeting inclusion criteria	Title and abstract screening
4849	Wang Y-H, Enguidanos S. Comparing Variations in Advance Directives Timing among Older Adults with End-Stage Renal Disease versus Cancer. Am J Hosp Palliat Med. 2023;40: 147–152. doi:10.1177/10499091221097676	No	Not meeting inclusion criteria	Title and abstract screening
4850	Wang Z. Colonic J-pouch versus side-to-end anastomosis for rectal cancer: a systematic review and meta-analysis of randomized controlled trials. BMC Surg. 2021;21: 1–12. doi:10.1186/s12893-021-01313-0	No	Not meeting inclusion criteria	Title and abstract screening
4851	Wang-Schweig M, Jason LA, Stevens E, Chaparro J. Tobacco Use among Recovery Home Residents: Vapers Less Confident to Quit. Am J Health Behav. 2019;43: 1064–1074. doi:10.5993/AJHB.43.6.5	No	Not meeting inclusion criteria	Title and abstract screening
4852	Ward AM, Yaman R, Ebbert JO. Electronic nicotine delivery system design and aerosol toxicants: A systematic review. PLoS One. 2020;15: e0234189. doi:10.1371/journal.pone.0234189	No	Not meeting inclusion criteria	Title and abstract screening
4853	Ward M, May P, Normand C, Kenny RA, Nolan A. Comparing Underlying and Contributory Cause of Death in Registry Data With End-of-Life Proxy Interviews: Findings From The Irish Longitudinal Study on Ageing (TILDA). J Appl Gerontol. 2021;40: 1189–1196. doi:10.1177/0733464820935295	No	Not meeting inclusion criteria	Title and abstract screening

4854	Wark PAB, Pathinayake PS, Eapen MS, Sohal SS. Asthma, COPD and SARS-CoV-2 infection (COVID-19): potential mechanistic insights. Eur Respir J. 2021;58. doi:10.1183/13993003.00920-2021	No	Not meeting inclusion criteria	Title and abstract screening
4855	Warlick C, Richter KP, Catley D, Gajewski BJ, Martin LE, Mussulman LM. Two brief valid measures of therapeutic alliance in counseling for tobacco dependence. J Subst Abuse Treat. 2018;86: 60–64. doi:10.1016/j.jsat.2017.12.010	No	Not meeting inclusion criteria	Title and abstract screening
4856	Warner KE, Schroeder SA. FDA's Innovative Plan to Address the Enormous Toll of Smoking. JAMA. 2017;318: 1755–1756. doi:10.1001/jama.2017.14336	No	Not meeting inclusion criteria	Title and abstract screening
4857	Warner KE, Mendez D. Response to letter from Ward-Peterson and Maziak, "for smoking cessation, e-cigarette flavors aren't required". Nicotine Tob Res. 2019;21: 134–135. doi:10.1093/ntr/nty219https://dx.doi.org/10.1093/ntr/nty219	No	Not meeting inclusion criteria	Title and abstract screening
4858	Wasfi RA, Bang F, de Groh M, Champagne A, Han A, Lang JJ, et al. Chronic health effects associated with electronic cigarette use: A systematic review. Front Public Health. 2022;10: 959622. doi:10.3389/fpubh.2022.959622	No	Not meeting inclusion criteria	Title and abstract screening
4859	Wasio A., Sanchez Fernandez L.L., Saintil M., Anis K., Singh H. A Case of Status Asthmaticus Refractory to Conventional Treatment Strategies. Am J Respir Crit Care Med. 2021;203. doi:10.1164/ajrccm-conference.2021.TP120	No	Not meeting inclusion criteria	Title and abstract screening
4860	Wasserman E., Perosevic N. A REMINDER OF THE EXISTENCE OF EVALI IN THE TIME OF COVID-19. Chest. 2021;160: A1687. doi:10.1016/j.chest.2021.07.1535	No	Not meeting inclusion criteria	Title and abstract screening
4861	Watabe Y, Ujiie H, Matsuno Y, Fukui H, Fujiwara-Kuroda A, Kato T, et al. Rare Diagnosis of a Multilobular Pulmonary Mass. Chest. 2021;160: e63–e67. doi:10.1016/j.chest.2021.01.034	No	Not meeting inclusion criteria	Title and abstract screening
4862	Watanabe Y, Tanaka S, Hasebe K, Hiratsuka Y, Yoshida T, Kusano J, et al. Long-Term Outcomes of End-Flexible-Rigidscopic Transoral Surgery for Pharyngolaryngeal Cancer. Laryngoscope. 2023;133: 1415–1424. doi:10.1002/lary.30316	No	Not meeting inclusion criteria	Title and abstract screening
4863	Waters EA, Ball L, Gehlert S. "I don't believe it." Acceptance and skepticism of genetic health information among African-American and White smokers. Soc Sci Med. 2017;184: 153–160. doi:10.1016/j.socscimed.2017.04.053	No	Not meeting inclusion criteria	Title and abstract screening
4864	Watkins S.L., Thrul J., Max W., Ling P.M. Cold Turkey and Hot Vapes? A National Study of Young Adult Cigarette Cessation Strategies. Nicotine Tob Res. 2020;22: 638–646. doi:10.1093/ntr/nty270	No	Not meeting inclusion criteria	Title and abstract screening
4865	Watkins S.L., Thrul J., Max W., Ling P.M. Real-world effectiveness of smoking cessation strategies for young and older adults: Findings from a nationally representative cohort. Nicotine Tob Res. 2020;22: 1560–1568. doi:10.1093/ntr/ntz223	No	Not meeting inclusion criteria	Title and abstract screening
4866	Watkins SL, Pieper F, Chaffee BW, Yerger VB, Ling PM, Max W. Flavored Tobacco Product Use Among Young Adults by Race and Ethnicity: Evidence From the Population Assessment of Tobacco and Health Study. J Adolesc Health. 2022;71: 226–232. doi:10.1016/j.jadohealth.2022.02.013	No	Not meeting inclusion criteria	Title and abstract screening
4867	Watson ML, McKnight ER, Groner JA, Manos BE, Ebersole AM, Bonny AE. Patterns and perceptions of nicotine use among U.S. adolescents and young adults receiving medication treatment for opioid use disorder. Prev Med Rep. 2023;35: 102303. doi:10.1016/j.pmedr.2023.102303	No	Not meeting inclusion criteria	Title and abstract screening
4868	Watson NL, Mull KE, Bricker JB. The association between frequency of e-cigarette use and long-term smoking cessation outcomes among treatment-seeking smokers receiving a behavioral intervention. Drug Alcohol Depend. 2021;218: 108394. doi:10.1016/j.drugalcdep.2020.108394	No	Not meeting inclusion criteria	Title and abstract screening
4869	Watt E., Gardner-Medwin J., Bush A., Langley R.J. E-CIGARETTE ADDICTION IN ADOLESCENTS - HOW DO WE GET THEM TO STOP? Thorax. 2023;78: A141–A142. doi:10.1136/thorax-2023-BTSabstracts.211	No	Not meeting inclusion criteria	Title and abstract screening
4870	Watts C, Burton S, Freeman B. Creating a market for IQOS: analysis of Philip Morris' strategy to introduce heated tobacco products to the Australian consumer market. Tob Control. 2022;31: 458–463. doi:10.1136/tobaccocontrol-2020-056057	No	Not meeting inclusion criteria	Title and abstract screening
4871	Watts C, Egger S, Dessaix A, Brooks A, Jenkinson E, Grogan P, et al. Vaping product access and use among 14-17-year-olds in New South Wales: a cross-sectional study. Aust N Z J Public Health. 2022. doi:10.1111/1753-6405.13316	No	Not meeting inclusion criteria	Title and abstract screening
4872	Watts C, Jones M, Lindorff K, Freeman B. How tobacco companies use the revolving door between government and industry to influence policymaking: an Australian case study. Public Health Res Pr. 2023. doi:10.17061/phrp33122305	No	Not meeting inclusion criteria	Title and abstract screening
4873	Wawryk-Gawda E, Chylińska-Wrzos P, K Zarobkiewicz M, Chłapek K, Jodłowska-Jędrych B. Lung histomorphological alterations in rats exposed to cigarette smoke and electronic cigarette vapour. Exp Ther Med. 2020;19: 2826–2832. doi:10.3892/etm.2020.8530	No	Not meeting inclusion criteria	Title and abstract screening
4874	Weaver S.R., Heath J.W., Ashley D.L., Huang J., Pechacek T.F., Eriksen M.P. What are the reasons that smokers reject ENDS? A national probability survey of U.S. Adult smokers, 2017-2018. Drug Alcohol Depend. 2020;211: 107855. doi:10.1016/j.drugalcdep.2020.107855	No	Not meeting inclusion criteria	Title and abstract screening
4875	Webb Hooper M, Kolar SK. Racial/ethnic differences in electronic cigarette knowledge, social norms, and risk perceptions among current and former smokers. Addict Behav. 2017;67: 86–91. doi:10.1016/j.addbeh.2016.12.013	No	Not meeting inclusion criteria	Title and abstract screening
4876	Webb J., Peerbux S., Smittenaar P., Siddiqui S., Sherwani Y., Ahmed M., et al. Preliminary outcomes of a digital therapeutic intervention for smoking cessation in adult smokers: Randomized controlled trial. JMIR Ment Health. 2020;7: e22833. doi:10.2196/22833	No	Not meeting inclusion criteria	Title and abstract screening
4877	Wee LH, Galvan JAA, Patil SS, Madhavan P, Mahalingam D, Yeong CH, et al. Understanding Factors Associated with Motivation to Quit Vaping among Vapers in the Federal Territory of Kuala Lumpur, Malaysia. Healthc Basel Switz. 2023;11. doi:10.3390/healthcare11141980	No	Not meeting inclusion criteria	Title and abstract screening
4878	Ween MP, Moshensky A, Thredgold L, Bastian NA, Hamon R, Badiei A, et al. E-cigarettes and health risks: more to the flavor than just the name. Am J Physiol Lung Cell Mol Physiol. 2021;320: L600–L614. doi:10.1152/ajplung.00370.2020	No	Not meeting inclusion criteria	Title and abstract screening
4879	Ween M.P., Chapman D.G., Larcombe A.N. What doctors should consider before prescribing e-liquids for e-cigarettes. Med J Aust. 2022;216: 14–16. doi:10.5694/mja2.51351	No	Not meeting inclusion criteria	Title and abstract screening
4880	Weiger C, Chen-Sankey J, Jeong M, Delnevo C, Wackowski O. Awareness and beliefs about FDA e-cigarette regulation in the premarket application review era. Addict Behav. 2023;144: 107748. doi:10.1016/j.addbeh.2023.107748	No	Not meeting inclusion criteria	Title and abstract screening
4881	Weiger C, Moran MB, Kennedy RD, Limaye R, Cohen J. Beliefs and Characteristics Associated With Believing Nicotine Causes Cancer: A Descriptive Analysis to Inform Corrective Message Content and Priority Audiences. Nicotine Tob Res. 2022;24: 1264–1272. doi:10.1093/ntr/ntac060	No	Not meeting inclusion criteria	Title and abstract screening
4882	Weiger C, Cohen JE, David Kennedy R, Moran MB. Testing messaging strategies to correct beliefs about nicotine and relative harm perceptions of non-cigarette tobacco products compared to cigarettes: A 2 x 2 factorial experiment of factsheets. Addict Behav. 2024;150: 107915. doi:10.1016/j.addbeh.2023.107915	No	Not meeting inclusion criteria	Title and abstract screening

4883	Weisman S. COVID Mimics at the Height of the Pandemic. J Thorac Imaging. 2021;36: W134. doi:10.1097/RTI.0000000000000619	No	Not meeting inclusion criteria	Title and abstract screening
4884	Weiss M., Danese S., Ruiz K.A., Kjelstrom S., Buckley M., Leitenberger A., et al. A Survey of Breast Cancer Patients' Use of Cannabis During Radiation Therapy. Int J Radiat Oncol Biol Phys. 2021;111: e165–e166. doi:10.1016/j.ijrobp.2021.07.642	No	Not meeting inclusion criteria	Title and abstract screening
4885	Wekon-Kemeni C., Santhanam P., Halani P., Bradford L., Loughlin C.E. A gut feeling: Abdominal symptoms as an initial presentation of EVALI. Pediatrics. 2021;147: e20193834. doi:10.1542/PEDS.2019-3834	No	Not meeting inclusion criteria	Title and abstract screening
4886	Wellman RJ, O'Loughlin EK, Sylvestre M-P, Dugas EN, O'Loughlin JL. Factors associated with cannabis use in early adolescence. Health Promot Chronic Dis Prev Can Res Policy Pract. 2023;43: 14–26. doi:10.24095/hpcdp.43.1.02	No	Not meeting inclusion criteria	Title and abstract screening
4887	Wells SY, LoSavio ST, Patel TA, Evans MK, Beckham JC, Calhoun P, et al. Contingency management and cognitive behavior therapy for smoking cessation among veterans with posttraumatic stress disorder: Design and methodology of a randomized clinical trial. Contemp Clin Trials. 2022;119: 106839. doi:10.1016/j.cct.2022.106839	No	Not meeting inclusion criteria	Title and abstract screening
4888	Wen F-H, Chou W-C, Hou M-M, Su P-J, Shen W-C, Chen J-S, et al. Depressive-Symptom Trajectories From End-of-Life Caregiving Through the First 2 Bereavement Years for Family Caregivers of Advanced Cancer Patients. J Pain Symptom Manage. 2021;62: 699–708. doi:10.1016/j.jpainsymman.2021.03.018	No	Not meeting inclusion criteria	Title and abstract screening
4889	Wen F-H, Chou W-C, Hou M-M, Su P-J, Shen W-C, Chen J-S, et al. Caregivers' Death-Preparedness States Impact Caregiving Outcomes and Patients' End-of-Life Care. J Pain Symptom Manage. 2022;63: 199–209. doi:10.1016/j.jpainsymman.2021.09.012	No	Not meeting inclusion criteria	Title and abstract screening
4890	Wen H, Xie C, Wang F, Wu Y, Yu C. Trends in Disease Burden Attributable to Tobacco in China, 1990-2017: Findings From the Global Burden of Disease Study 2017. Front Public Health. 2020;8: 237. doi:10.3389/fpubh.2020.00237	No	Not meeting inclusion criteria	Title and abstract screening
4891	Wen Q, Chan KH, Shi K, Lv J, Guo Y, Pei P, et al. Tobacco smoking and solid fuels for cooking and risk of liver cancer: A prospective cohort study of 0.5 million Chinese adults. Int J Cancer. 2022;151: 181–190. doi:10.1002/ijc.33977	No	Not meeting inclusion criteria	Title and abstract screening
4892	Wen Q, Wang X, Lv J, Guo Y, Pei P, Yang L, et al. Association between involuntary smoking and risk of cervical cancer in Chinese female never smokers: A prospective cohort study. Env Res. 2022;212: 113371. doi:10.1016/j.envres.2022.113371	No	Not meeting inclusion criteria	Title and abstract screening
4893	Wen X, Chung MV, Liszewski KA, Todoro LD, Giancarlo EM, Zhang W, et al. Cigarette Smoking Abstinence Among Pregnant Individuals Using E-Cigarettes or Nicotine Replacement Therapy. JAMA Netw Open. 2023;6: e2330249. doi:10.1001/jamanetworkopen.2023.30249	No	Not meeting inclusion criteria	Title and abstract screening
4894	Wen X, Liu L, Moe AA, Ormond IK, Shuren CC, Scott IN, et al. Use of E-Cigarettes and Cigarettes During Late Pregnancy Among Adolescents. JAMA Netw Open. 2023;6: e2347407. doi:10.1001/jamanetworkopen.2023.47407	No	Not meeting inclusion criteria	Title and abstract screening
4895	Wen Z, Wang J, Tu B, Liu Y, Yang Y, Hou L, et al. Radiofrequency ablation combined with toripalimab for recurrent hepatocellular carcinoma: a prospective controlled trial. 2023;12: 20311-20320. doi:10.1002/cam4.6602	No	Not meeting inclusion criteria	Title and abstract screening
4896	Weng X, Shen C, Van Scoy LJ, Boltz M, Joshi M, Wang L. End-of-Life Costs of Cancer Patients With Alzheimer's Disease and Related Dementias in the U.S. J Pain Symptom Manage. 2022;64: 449–460. doi:10.1016/j.jpainsymman.2022.07.014	No	Not meeting inclusion criteria	Title and abstract screening
4897	Wenzel K.R., Fishman M., Dease M., Soo A., Kolodner G. Addressing Tobacco Use Disorder in SUD Treatment: Talking about Tobacco. J Addict Med. 2023;17: e321.	No	Not meeting inclusion criteria	Title and abstract screening
4898	Weser V, Duncan L, Sands B, Schartmann A, Jacobo S, Francois B, et al. Evaluation of a virtual reality E-cigarette prevention game for adolescents. 2021;122. doi:10.1016/j.addbeh.2021.107027	No	Not meeting inclusion criteria	Title and abstract screening
4899	West R, Cox S. The 1988 US Surgeon General's report Nicotine Addiction: how well has it stood up to three more decades of research? Addiction. 2022;117: 2346–2350. doi:10.1111/add.15754	No	Not meeting inclusion criteria	Title and abstract screening
4900	Westgeest HM, Kuppen MCP, van den Eertwegh FAJM, van Oort IM, Coenen JLLM, van Moorselaar JRJA, et al. High-Intensity Care in the End-of-Life Phase of Castration-Resistant Prostate Cancer Patients: Results from the Dutch CAPRI-Registry. J Palliat Med. 2021;24: 1789–1797. doi:10.1089/jpm.2020.0800	No	Not meeting inclusion criteria	Title and abstract screening
4901	Westmaas J.L., Strollo S.E., Newton C.C., Carter B.D., Diver W.R., Flanders W.D., et al. Association between smoking cannabis and quitting cigarettes in a large american cancer society cohort. Cancer Epidemiol Biomarkers Prev. 2021;30: 1956–1964. doi:10.1158/1055-9965.EPI-20-1810	No	Not meeting inclusion criteria	Title and abstract screening
4902	Westmaas JL, Masters M, Bandi P, Majmundar A, Asare S, Diver WR. COVID-19 and Tweets About Quitting Cigarette Smoking: Topic Model Analysis of Twitter Posts 2018-2020. JMIR Infodemiology. 2022;2: e36215. doi:10.2196/36215	No	Not meeting inclusion criteria	Title and abstract screening
4903	Westphalen C, Kniesburges S, Veltrup R, Gantner S, Peters G, Benthaus T, et al. Sources of Aerosol Dispersion During Singing and Potential Safety Procedures for Singers. J Voice. 2023;37: 504–514. doi:10.1016/j.jvoice.2021.03.013	No	Not meeting inclusion criteria	Title and abstract screening
4904	Westwood A.T.R., Vanker A., Gray D. Tobacco, nicotine and e-cigarettes: Protecting children in south africa. SAJCH South Afr J Child Health. 2019;13: 4–5. doi:10.7196/SAJCH.2019.v13i1.1639	No	Not meeting inclusion criteria	Title and abstract screening
4905	Wetherill RR, Dubroff J. Reply: Molecular Imaging of Pulmonary Inflammation: Claiming That Vaping Is More Harmful Than Smoking Is Unsupported. J Nucl Med. 2023;64: 995. doi:10.2967/jnumed.123.265570	No	Not meeting inclusion criteria	Title and abstract screening
4906	Wharton MK, Islam S, Abadi MH, Pokhrel P, Lipperman-Kreda S. COVID-19 Restrictions and Adolescent Cigarette and E-cigarette Use in California. Am J Prev Med. 2022. doi:10.1016/j.amepre.2022.09.014	No	Not meeting inclusion criteria	Title and abstract screening
4907	Wharton MK, Islam S, Abadi MH, Pokhrel P, Lipperman-Kreda S. COVID-19 Restrictions and Adolescent Cigarette and E-cigarette Use in California. Am J Prev Med. 2023;64: 385–392. doi:10.1016/j.amepre.2022.09.014	No	Not meeting inclusion criteria	Title and abstract screening
4908	Whetstone V. A PROPOSED STUDY OF THE EFFECTS OF EARLY END OF LIFE DISCUSSIONS ON QUALITY OF LIFE IN PATIENTS WITH TERMINAL LUNG CANCER...47th Annual Oncology Nursing Society Congress, April 27–May 1, 2022, Anaheim, CA. Oncol Nurs Forum. 2022;49: E49–E50. doi:10.1188/22.ONF.E2	No	Not meeting inclusion criteria	Title and abstract screening
4909	Whitaker M, Elliott J, Chadeau-Hyam M, Riley S, Darzi A, Cooke G, et al. Persistent COVID-19 symptoms in a community study of 606,434 people in England. Nat Commun. 2022;13: 1957. doi:10.1038/s41467-022-29521-z	No	Not meeting inclusion criteria	Title and abstract screening
4910	White A.V., Wambui D.W., Pokhrel L.R. Risk assessment of inhaled diacetyl from electronic cigarette use among teens and adults. Sci Total Environ. 2021;772: 145486. doi:10.1016/j.scitotenv.2021.145486	No	Not meeting inclusion criteria	Title and abstract screening
4911	White AM, Li D, Snell LM, O'Connor R, Hoetger C, Croft D, et al. Perceptions of Tobacco Product-Specific COVID-19 Risk and Changes in Tobacco Use Behaviors Among Smokers, E-Cigarette Users, and Dual Users. Nicotine Tob Res. 2021;23: 1617–1622. doi:10.1093/ntr/ntab053	No	Not meeting inclusion criteria	Title and abstract screening

4912	White C.J., White B.S., Wadhwa S.R., Aouad A. Acute Respiratory Distress Syndrome During the COVID-19 Era. Infect Dis Clin Pract. 2022;30: e1102. doi:10.1097/IPC.0000000000001102	No	Not meeting inclusion criteria	Title and abstract screening
4913	White CM, Hatsukami DK, Donny EC. Reducing the relative value of cigarettes: Considerations for nicotine and non-nicotine factors. Neuropharmacology. 2020;175: 108200. doi:10.1016/j.neuropharm.2020.108200	No	Not meeting inclusion criteria	Title and abstract screening
4914	White CM, Tessier KM, Koopmeiners JS, Denlinger-Apte RL, Cobb CO, Lane T, et al. Preliminary evidence on cigarette nicotine reduction with concurrent access to an e-cigarette: Manipulating cigarette nicotine content, e-liquid nicotine content, and e-liquid flavor availability. Prev Med. 2022; 107213. doi:10.1016/j.ypmed.2022.107213	No	Not meeting inclusion criteria	Title and abstract screening
4915	White D., Bush A., Smyth A.R., Bhatt J.M. Why and how should children be protected from the deluge of vaping related media and marketing overexposure? Breathe. 2023;19: 230141. doi:10.1183/20734735.0141-2023	No	Not meeting inclusion criteria	Title and abstract screening
4916	Whitehead AK, Erwin AP, Yue X. Nicotine and vascular dysfunction. Acta Physiol Oxf Engl. 2021;231: e13631. doi:10.1111/apha.13631	No	Not meeting inclusion criteria	Title and abstract screening
4917	Whitehead AK, Meyers MC, Taylor CM, Luo M, Dowd SE, Yue X, et al. Sex-Dependent Effects of Inhaled Nicotine on the Gut Microbiome. Nicotine Tob Res Off J Soc Res Nicotine Tob. 2022;24: 1363–1370. doi:10.1093/ntr/ntac064	No	Not meeting inclusion criteria	Title and abstract screening
4918	Wick KD, Matthay MA. Environmental Factors. Crit Care Clin. 2021;37: 717–732. doi:10.1016/j.ccc.2021.05.002	No	Not meeting inclusion criteria	Title and abstract screening
4919	Wickham RJ, Henderson BJ, Jackson AB, Kabbani N. Editorial: Pharmacological and behavioral effects of added flavorants on tobacco addiction. Front Neurosci. 2022;16: 1100476. doi:10.3389/fnins.2022.1100476	No	Not meeting inclusion criteria	Title and abstract screening
4920	Wiegert EVM, da Costa Rosa KS, dos Santos RTF, dos Santos DA, de Freitas R, de Oliveira LC. The use of nutrition support near the end of life for hospitalized patients with advanced cancer at a reference center: Two realities. Nutr Clin Pract. 2022;37: 425–434. doi:10.1002/ncp.10737	No	Not meeting inclusion criteria	Title and abstract screening
4921	Wieghorst A., Roessler K.K., Hendricks O., Andersen T.E. The effect of medical cannabis on cognitive functions: a systematic review. Syst Rev. 2022;11: 210. doi:10.1186/s13643-022-02073-5	No	Not meeting inclusion criteria	Title and abstract screening
4922	Wiens T, Taylor J, Cole C, Saravia S, Peterson J, Lunda M, et al. Lessons Learned From the E-cigarette, or Vaping, Product Use-Associated Lung Injury (EVALI) Outbreak Response, Minnesota, 2019-2020. Public Health Rep. 2022;137: 1053–1060. doi:10.1177/00333549211051394	No	Not meeting inclusion criteria	Title and abstract screening
4923	Wiers R.W., Grasman R.P.P.P. Editorial special issue addictive behaviors, networks, complexity and addictive behaviors. Addict Behav. 2022;132: 107369. doi:10.1016/j.addbeh.2022.107369	No	Not meeting inclusion criteria	Title and abstract screening
4924	WIG R., RATHBUN J.O.H.N., W BLOOM J.O.H.N., TAMMER EL-AINI A. PLASTIC PROBLEMS: OCEANS AND TRACHEOBRONCHIAL TREES. Chest. 2022;162: A1562–A1563. doi:10.1016/j.chest.2022.08.1314	No	Not meeting inclusion criteria	Title and abstract screening
4925	Wigginton B., Morphett K., Gartner C. Differential access to health care and support? A qualitative analysis of how Australian smokers conceptualise and respond to stigma. Crit Public Health. 2017;27: 577–590. doi:10.1080/09581596.2016.1266298	No	Not meeting inclusion criteria	Title and abstract screening
4926	Wigginton B, Morphett K, Gartner C. Is it the nicotine? Australian smokers' accounts of nicotine addiction. Addict Res Theory. 2017;25: 293–301. doi:10.1080/16066359.2016.1269892https://dx.doi.org/10.1080/16066359.2016.1269892	No	Not meeting inclusion criteria	Title and abstract screening
4927	Willett J., Achenbach S., Pinto F.J., Poppas A., Elkind M.S.V. The tobacco endgame - Eradicating a worsening epidemic: A Joint Opinion from the American College of Cardiology, American Heart Association, European Society of Cardiology, and the World Heart Federation. Eur Heart J. 2021;42: 3044–3048. doi:10.1093/eurheartj/ehab245	No	Not meeting inclusion criteria	Title and abstract screening
4928	Willett J., Achenbach S., Pinto F.J., Poppas A., Elkind M.S.V. The Tobacco Endgame: Eradicating a Worsening Epidemic A Joint Opinion From the American Heart Association, World Heart Federation, American College of Cardiology, and the European Society of Cardiology. J Am Coll Cardiol. 2021;78: 77–81. doi:10.1016/j.jacc.2021.04.005	No	Not meeting inclusion criteria	Title and abstract screening
4929	Williams MA, Reddy G, Quinn MJ, Millikan Bell A. Toxicological assessment of electronic cigarette vaping: an emerging threat to force health, readiness and resilience in the U.S. Army. Drug Chem Toxicol. 2022;45: 2049–2085. doi:10.1080/01480545.2021.1905657	No	Not meeting inclusion criteria	Title and abstract screening
4930	Wills AG, Hopfer C. Phenotypic and genetic relationship between BMI and cigarette smoking in a sample of UK adults. Addict Behav. 2019;89: 98–103. doi:10.1016/j.addbeh.2018.09.025	No	Not meeting inclusion criteria	Title and abstract screening
4931	Wills T.A., Sargent J.D. Do E-cigarettes reduce smoking? Prev Med. 2017;100: 285–286. doi:10.1016/j.ypmed.2017.04.030	No	Not meeting inclusion criteria	Title and abstract screening
4932	Wills TA, Choi K, Perez MF. E-cigarettes and Respiratory Disorder: The Broader Context. Nicotine Tob Res. 2023;25: 1215–1216. doi:10.1093/ntr/ntad029	No	Not meeting inclusion criteria	Title and abstract screening
4933	Wills TA, Kaholokula JK, Pokhrel P, Pagano I. Ethnic differences in respiratory disease for Native Hawaiians and Pacific Islanders: Analysis of mediation processes in two community samples. PLoS One. 2023;18: e0290794. doi:10.1371/journal.pone.0290794	No	Not meeting inclusion criteria	Title and abstract screening
4934	Wills TA, Leventhal AM, Sargent JD, Pagano I. Concurrent Use of E-cigarettes, Combustible Cigarettes, and Marijuana. Pediatrics. 2021;148. doi:10.1542/peds.2021-050749	No	Not meeting inclusion criteria	Title and abstract screening
4935	Wills TA, Xie W, Stokes AC. Issues for Studies on E-cigarettes and Chronic Obstructive Pulmonary Disorder. Am J Prev Med. 2023;65: 1196–1197. doi:10.1016/j.amepre.2023.09.009	No	Not meeting inclusion criteria	Title and abstract screening
4936	Wilson AC, Jungbauer WN, Hussain FT, Lindgren BR, Lassig AAD. Characterization of Baseline Temperature Characteristics Using Thermography in The Clinical Setting. J Surg Res. 2022;272: 26–36. doi:10.1016/j.jss.2021.11.006	No	Not meeting inclusion criteria	Title and abstract screening
4937	Wilson C, Tellez Freitas CM, Awan KH, Ajdaharian J, Geiler J, Thirucenthilvelan P. Adverse effects of E-cigarettes on head, neck, and oral cells: A systematic review. J Oral Pathol Med. 2022;51: 113–125. doi:10.1111/jop.13273	No	Not meeting inclusion criteria	Title and abstract screening
4938	Wilson C.M., Lucado A.M., Wendland D.M., Taylor D.W.M., Black B. Health promotion, wellness and prevention practice in oncologic physical therapy. Rehabil Oncol. 2021;39: E31–E32. doi:10.1097/01.REO.0000000000000254	No	Not meeting inclusion criteria	Title and abstract screening
4939	Wilson C.M., Lucado A.M., Wendland D.M., Taylor D.W.M., Black B. Health Promotion, Wellness, and Prevention Practice in Oncologic Physical Therapy: A Survey Study. Rehabil Oncol. 2021;39: E51–E57. doi:10.1097/01.REO.0000000000000244	No	Not meeting inclusion criteria	Title and abstract screening
4940	Wilson G.L., Keenan J., Grogan S., Porcellato L., Powell S., Gee I. An investigation of factors encouraging and deterring EC use: a thematic analysis of accounts from UK adults. Psychol Health. 2021; 1–17. doi:10.1080/08870446.2021.1952583	No	Not meeting inclusion criteria	Title and abstract screening

4941	Wilson GL, Keenan J, Grogan S, Porcellato L, Powell S, Gee I. An investigation of factors encouraging and deterring EC use: a thematic analysis of accounts from UK adults. Psychol Health. 2022;37: 1379–1395. doi:10.1080/08870446.2021.1952583	No	Not meeting inclusion criteria	Title and abstract screening
4942	Wilson N, Summers JA, Ait Ouakrim D, Hoek J, Edwards R, Blakely T. Improving on estimates of the potential relative harm to health from using modern ENDS (vaping) compared to tobacco smoking. BMC Public Health. 2021;21: 2038. doi:10.1186/s12889-021-12103-x	No	Not meeting inclusion criteria	Title and abstract screening
4943	Winer J.M., Yule A.M., Hadland S.E., Bagley S.M. Addressing adolescent substance use with a public health prevention framework: the case for harm reduction. Ann Med. 2022;54: 2123–2136. doi:10.1080/07853890.2022.2104922	No	Not meeting inclusion criteria	Title and abstract screening
4944	Winhusen T. Introduction to Special Issue on Tobacco and Other Substance Use Disorders: Links and Implications. Am J Drug Alcohol Abuse. 2017;43: 129–131. doi:10.1080/00952990.2016.1257635	No	Not meeting inclusion criteria	Title and abstract screening
4945	Wiseman KP, Margolis KA, Bernat JK, Grana RA. The association between perceived e-cigarette and nicotine addictiveness, information-seeking, and e-cigarette trial among U.S. adults. Prev Med. 2019;118: 66–72. doi:10.1016/j.ypmed.2018.10.003	No	Not meeting inclusion criteria	Title and abstract screening
4946	Wojtecka A, Kalinowska-Beszczynska O, Tyranska-Fobke A, Kaleta D, Wojnarowska M, Robakowska M, et al. Adolescents' Perceptions and Attitudes towards Traditional and Electronic Cigarettes-Results of Focus Group Interviews. Int J Environ Res Public Health. 2023;20. doi:10.3390/ijerph20021438	No	Not meeting inclusion criteria	Title and abstract screening
4947	Wold L.E., Tarran R., Crotty Alexander L.E., Hamburg N.M., Kheradmand F., St. Helen G., et al. Cardiopulmonary Consequences of Vaping in Adolescents: A Scientific Statement from the American Heart Association. Circ Res. 2022;131: E70–E82. doi:10.1161/RES.0000000000000544	No	Not meeting inclusion criteria	Title and abstract screening
4948	Wolfenden L., Stockings E., Yoong S.L. Regulating e-cigarettes in Australia: Implications for tobacco use by young people. Med J Aust. 2018;208: 8-9.e1. doi:10.5694/mja17.00787	No	Not meeting inclusion criteria	Title and abstract screening
4949	Won J, Clark H. Palatal keratosis associated with reverse (or "backwards") smoking (PKARS). N Z Med J. 2022;135: 104–107.	No	Not meeting inclusion criteria	Title and abstract screening
4950	Wong ET, Szostak J, Titz B, Lee T, Wong SK, Lavrynenko O, et al. A 6-month inhalation toxicology study in Apoe-/- mice demonstrates substantially lower effects of e-vapor aerosol compared with cigarette smoke in the respiratory tract. Arch Toxicol. 2021;95: 1805–1829. doi:10.1007/s00204-021-03020-4	No	Not meeting inclusion criteria	Title and abstract screening
4951	Wong JA, Pratt SI, Ferron JC, Gowarty M, Brunette MF. Characteristics of and reasons for electronic cigarette use among adult smokers with schizophrenia/schizoaffective disorder. Ann Clin Psychiatry. 2022;34: 89–96. doi:10.12788/acp.0050	No	Not meeting inclusion criteria	Title and abstract screening
4952	Wood LA, Agbonlahor O, Tomlinson MM, Kerstiens S, Vincent K, McLeish AC, et al. Readability of online e-cigarette cessation information. Tob Induc Dis. 2022;20: 53. doi:10.18332/tid/149906	No	Not meeting inclusion criteria	Title and abstract screening
4953	Wopperer S., Safren L., Masson R., Malik A., Barnett C. Recurrent Vasospastic Angina Caused by Electronic Cigarette Usage. Circulation. 2020;142. doi:10.1161/circ.142.suppl_3.16001	No	Not meeting inclusion criteria	Title and abstract screening
4954	Worley MJ, Isgro M, Heffner JL, Lee SY, Daniel BE, Anthenelli RM. Predictors of reduced smoking quantity among recovering alcohol dependent men in a smoking cessation trial. Addict Behav. 2018;84: 263–270. doi:10.1016/j.addbeh.2018.05.004	No	Not meeting inclusion criteria	Title and abstract screening
4955	Wright C, Williams P, Elizarova O, Dahne J, Bian J, Zhao Y, et al. Effects of brief exposure to misinformation about e-cigarette harms on twitter: a randomised controlled experiment. BMJ Open. 2021;11: e045445. doi:10.1136/bmjopen-2020-045445	No	Not meeting inclusion criteria	Title and abstract screening
4956	Wu D, Kasson E, Singh AK, Ren Y, Kaiser N, Huang M, et al. Topics and Sentiment Surrounding Vaping on Twitter and Reddit During the 2019 e-Cigarette and Vaping Use-Associated Lung Injury Outbreak: Comparative Study. J Med Internet Res. 2022;24: e39460. doi:10.2196/39460	No	Not meeting inclusion criteria	Title and abstract screening
4957	Wu D, Yang Y, Jiang M, Yao R. Competing risk of the specific mortality among Asian-American patients with prostate cancer: a surveillance, epidemiology, and end results analysis. BMC Urol. 2022;22: 1–10. doi:10.1186/s12894-022-00992-y	No	Not meeting inclusion criteria	Title and abstract screening
4958	Wu J, Gao Y, Li D, Gao N. Emission and Gas/Particle Partitioning Characteristics of Nicotine in Aerosols for Electronic Cigarettes. Chem Res Toxicol. 2022;35: 890–897. doi:10.1021/acs.chemrestox.2c00076	No	Not meeting inclusion criteria	Title and abstract screening
4959	Wu L, Zhao W, Chen T, Yang Y. Surgical choice for patients with stage I non-small-cell lung cancer ≤2 cm: an analysis from surveillance, epidemiology, and end results database. J Cardiothorac Surg. 2021;16: 1–10. doi:10.1186/s13019-021-01568-x	No	Not meeting inclusion criteria	Title and abstract screening
4960	Wu M., Sharma P., Rajderkar D. Childhood interstitial lung disease: A case-based review of the imaging findings. Ann Thorac Med. 2021;16: 64–72. doi:10.4103/atm.ATM_384_20	No	Not meeting inclusion criteria	Title and abstract screening
4961	Wu T, Xu K, Liu C, Li Y, Li M. Interleukin-37 ameliorates cigarette smoke-induced lung inflammation in mice. Biomed Pharmacother. 2022;155: 113684. doi:10.1016/j.biopha.2022.113684	No	Not meeting inclusion criteria	Title and abstract screening
4962	Wu YS, Wang MP, Ho SY, Cheung YT, Kwong A, Lai V, et al. Positive perceptions of electronic cigarettes relative to combustible cigarettes are associated with weaker support for endgame policies on combustible cigarettes: A population-based cross-sectional study in Hong Kong. Tob Induc Dis. 2019;17: 61. doi:10.18332/tid/110697	No	Not meeting inclusion criteria	Title and abstract screening
4963	Wulan WR, Kusuma D, Nurjanah N, Aprianti A, Ahsan A. Is Exposure to Social Media Advertising and Promotion Associated with E-cigarette Use? Evidence from Indonesia. Asian Pac J Cancer Prev. 2022;23: 1257–1262. doi:10.31557/APJCP.2022.23.4.1257	No	Not meeting inclusion criteria	Title and abstract screening
4964	Wyatt K, Bastaki H, Davies N. Delivering end-of-life care for patients with cancer at home: Interviews exploring the views and experiences of general practitioners. Health Soc Care Community. 2022;30: e126–e137. doi:10.1111/hsc.13419	No	Not meeting inclusion criteria	Title and abstract screening
4965	Wyatt T.A., DeVasure J.M., Ochoa C., Woodard J.C., Knoell D.L., Bailey K.L. Comparative effects of JUUL, eCig, and cigarette smoke on wound repair in alcohol-exposed bronchial epithelial cells. Am J Respir Crit Care Med. 2019;199. Available: https://www.atsjournals.org/doi/abs/10.1164/ajrccm-conference.2019.199.1_MeetingAbstracts.A4175	No	Not meeting inclusion criteria	Title and abstract screening
4966	Wyatt T.A., Mosley D.D., DeVasure J.M., Bailey K.L. JUUL and alcohol decrease airway epithelial wound repair. Alcohol Clin Exp Res. 2020;44: 48A. doi:10.1111/acer.14356	No	Not meeting inclusion criteria	Title and abstract screening
4967	Wylie BJ, Hauptman M, Hacker MR, Hawkins SS. Understanding Rising Electronic Cigarette Use. Obstet Gynecol. 2021;137: 521–527. doi:10.1097/AOG.0000000000004282	No	Not meeting inclusion criteria	Title and abstract screening
4968	Wysota CN, Le D, Clausen ME, Ciceron AC, Fuss C, Bennett B, et al. Young adults' knowledge, perceptions and use of cannabidiol products: a mixed-methods study. Health Educ Res. 2022;37: 379–392. doi:10.1093/her/cyac030	No	Not meeting inclusion criteria	Title and abstract screening
4969	Xia N., Morteza A., Yang F., Cao H., Wang A. Review of the role of cigarette smoking in diabetic foot. J Diabetes Investig. 2019;10: 202–215. doi:10.1111/jdi.12952	No	Not meeting inclusion criteria	Title and abstract screening

4970	Xia S, Zhu HT, Zhang XY, Shi YJ, Li XT, Sun YS. An End-to-End Auto-Prediction Model Response to Neoadjuvant Chemoradiotherapy for Rectal Cancer Based on Multimodal Segmentation and Multipath Lightweight Convolution. Int J Radiat Oncol Biol Phys. 2022;114: e153–e153. doi:10.1016/j.ijrobp.2022.07.1011	No	Not meeting inclusion criteria	Title and abstract screening
4971	Xian J. An "evali-ultion" of understanding, a case of a 34 year old male with e-cigarette and vaping related lung injury. Am J Respir Crit Care Med. 2021;203. doi:10.1164/ajrccm-conference.2021.203.1_MeetingAbstracts.A3016	No	Not meeting inclusion criteria	Title and abstract screening
4972	Xiang W, Wu C, Wu H, Fang S, Liu N, Yu H. Survival Comparisons between Breast Conservation Surgery and Mastectomy Followed by Postoperative Radiotherapy in Stage I–III Breast Cancer Patients: Analysis of the Surveillance, Epidemiology, and End Results (Seer) Program Database. Curr Oncol. 2022;29: 5731–5747. doi:10.3390/curroncol29080452	No	Not meeting inclusion criteria	Title and abstract screening
4973	Xiao X, Gao B, Pang S, Wang Z, Jiang W, Wang W, et al. Tumor size as a significant prognostic factor in T1 gastric cancer: a Surveillance, Epidemiology, and End Results (SEER) database analysis. BMC Gastroenterol. 2023;23: 1–10. doi:10.1186/s12876-023-02737-z	No	Not meeting inclusion criteria	Title and abstract screening
4974	Xie W, Kathuria H, Galiatsatos P, Blaha MJ, Hamburg NM, Robertson RM, et al. Association of Electronic Cigarette Use With Incident Respiratory Conditions Among US Adults From 2013 to 2018. JAMA Netw Open. 2020;3: e2020816. doi:10.1001/jamanetworkopen.2020.20816	No	Not meeting inclusion criteria	Title and abstract screening
4975	Xie Z, Cartujano-Barrera F, Cupertino P, Li D. Cross-Sectional Associations of Self-Reported Social/Emotional Support and Life Satisfaction with Smoking and Vaping Status in Adults. Int J Env Res Public Health. 2022;19. doi:10.3390/ijerph191710722	No	Not meeting inclusion criteria	Title and abstract screening
4976	Xu C, Wang S, Su BB, Ozuna K, Mao C, Dai Z, et al. Associations of adolescent substance use and depressive symptoms with adult major depressive disorder in the United States: NSDUH 2016-2019. J Affect Disord. 2024;344: 397–406. doi:10.1016/j.jad.2023.10.066	No	Not meeting inclusion criteria	Title and abstract screening
4977	Xu F, Zhao F, Feng X, Li C, Han D, Zheng S, et al. Nomogram for predicting cancer-specific survival in undifferentiated pleomorphic sarcoma: A Surveillance, Epidemiology, and End Results -based study. Cancer Control J Moffitt Cancer Cent. 2021; 1–9. doi:10.1177/10732748211036775	No	Not meeting inclusion criteria	Title and abstract screening
4978	Xu G, Snetselaar LG, Strathearn L, Ryckman K, Nothwehr F, Torner J. Association of Attention-Deficit/Hyperactivity Disorder With E-Cigarette Use. Am J Prev Med. 2021;60: 488–496. doi:10.1016/j.amepre.2020.11.010	No	Not meeting inclusion criteria	Title and abstract screening
4979	Xu J, Liu W, Liu X, Zhou X, Li G. Alcohol drinking, smoking, and cutaneous melanoma risk: Mendelian randomization analysis. Gac Sanit. 2023;37: 102351. doi:10.1016/j.gaceta.2023.102351	No	Not meeting inclusion criteria	Title and abstract screening
4980	Xu T, Qin Y, Ou X, Zhao X, Wang P, Wang M, et al. End-of-life communication experiences within families of people with advanced cancer in China: A qualitative study. Int J Nurs Stud. 2022;132: N.PAG-N.PAG. doi:10.1016/j.ijnurstu.2022.104261	No	Not meeting inclusion criteria	Title and abstract screening
4981	Xu Y, Gu Z, Zhang Y, He M, Gerber BS, Sadasivam RS, et al. Global trends in smoking cessation research from 2002 to 2021: A bibliometric and visual analysis. Prev Med Rep. 2022;30: 101991. doi:10.1016/j.pmedr.2022.101991	No	Not meeting inclusion criteria	Title and abstract screening
4982	Xu Z, Wang J, Cai H, Qi F, Zou Q. Second primary malignancies in oral tongue cancer: A Surveillance, Epidemiology, and End Result–based analysis evaluating the basic characteristics, survival outcomes, and predictive factors. Precis Med Sci. 2022;11: 4–13. doi:10.1002/prm2.12056	No	Not meeting inclusion criteria	Title and abstract screening
4983	Yach D, Scherer G. Applications of biomarkers of exposure and biological effects in users of new generation tobacco and nicotine products: Tentative proposals. Drug Test Anal. 2023;15: 1127–1132. doi:10.1002/dta.3567	No	Not meeting inclusion criteria	Title and abstract screening
4984	Yachimski P, Lucas A, Dusetzina SB. Trends in performance of ERCP among patients with surgically unresectable pancreatic cancer: a Survival, Epidemiology and End Results–Medicare database study. Gastrointest Endosc. 2021;94: 752–752. doi:10.1016/j.gie.2021.03.998	No	Not meeting inclusion criteria	Title and abstract screening
4985	Yamada S, Suga Y, Morishita E, Asakura H. Effect of Anticoagulant/Antifibrinolytic Combination Therapy on Enhanced Fibrinolytic-Type Disseminated Intravascular Coagulation in End-of-Life Stage Solid Tumor Patients. J Palliat Med. 2023;26: 307–311. doi:10.1089/jpm.2022.0249	No	Not meeting inclusion criteria	Title and abstract screening
4986	Yamamoto S, Tanaka A, Ohmagari N, Yamaguchi K, Ishitsuka K, Morisaki N, et al. Use of heated tobacco products, moderate alcohol drinking, and anti-SARS-CoV-2 IgG antibody titers after BNT162b2 vaccination among Japanese healthcare workers. Prev Med. 2022;161: 107123. doi:10.1016/j.ypmed.2022.107123	No	Not meeting inclusion criteria	Title and abstract screening
4987	Yamazaki T, Miyamoto S, Matsubara T, Yamagata H, Kobo H, Otani M, et al. Development of a Japanese Version of the Quality of Life at the End of Life-Cancer Scale. J Pain Symptom Manage. 2023;66: e189–e195. doi:10.1016/j.jpainsymman.2023.04.023	No	Not meeting inclusion criteria	Title and abstract screening
4988	Yan D, Wang Z, Laestadius L, Mosalpuria K, Wilson FA, Yan A, et al. A systematic review for the impacts of global approaches to regulating electronic nicotine products. J Glob Health. 2023;13: 04076. doi:10.7189/jogh.13.04076	No	Not meeting inclusion criteria	Title and abstract screening
4989	Yan H, Liu X, Yin L, Han H, Jin Y, Zhu X, et al. Effects of endoscopic therapy and surgical resection on long-term survival outcomes in patients with duodenal gastrointestinal stromal tumors: a surveillance, epidemiology, and end result program analysis. Surg Endosc. 2022;36: 8030–8038. doi:10.1007/s00464-022-09231-9	No	Not meeting inclusion criteria	Title and abstract screening
4990	Yan X, Salloum RG, Leong MC, Khalil GE, Lee JH, Lou XY. Racial and Ethnic Differences in Initiation of Menthol Tobacco Smoking and Subsequent Tobacco Use in the Population Assessment of Tobacco and Health Study, Waves 1-4 (2013-2018). Nicotine Tob Res. 2023;25: 1440–1446. doi:10.1093/ntr/ntad055	No	Not meeting inclusion criteria	Title and abstract screening
4991	Yang B, Barbati J, Choi Y. Will e-cigarette modified risk messages with a nicotine warning polarize smokers' beliefs about the efficacy of switching completely to e-cigarettes in reducing smoking-related risks? 2021;18. doi:10.3390/ijerph18116094	No	Not meeting inclusion criteria	Title and abstract screening
4992	Yang B, Owusu D, Popova L. Effects of a Nicotine Fact Sheet on Perceived Risk of Nicotine and E-Cigarettes and Intentions to Seek Information About and Use E-Cigarettes. 2019;17. doi:10.3390/ijerph17010131	No	Not meeting inclusion criteria	Title and abstract screening
4993	Yang B, Popova L. Communicating risk differences between electronic and combusted cigarettes: the role of the FDA-mandated addiction warning and a nicotine fact sheet. 2020;29: 663-671. doi:10.1136/tobaccocontrol-2019-055204	No	Not meeting inclusion criteria	Title and abstract screening
4994	Yang B., Owusu D., Popova L. Effects of a nicotine fact sheet on perceived risk of nicotine and e-cigarettes and intentions to seek information about and use e-cigarettes. Int J Environ Res Public Health. 2020;17: 131. doi:10.3390/ijerph17010131	No	Not meeting inclusion criteria	Title and abstract screening
4995	Yang D.C., Gu S., Li J.-M., Hsu S.-W., Chen S.-J., Chang W.-H., et al. Targeting the AXL receptor in combating smoking-related pulmonary fibrosis. Am J Respir Cell Mol Biol. 2021;64: 734–746. doi:10.1165/rcmb.2020-0303OC	No	Not meeting inclusion criteria	Title and abstract screening
4996	Yang H, Ma J. How the COVID-19 pandemic impacts tobacco addiction: Changes in smoking behavior and associations with well-being. Addict Behav. 2021;119: 106917. doi:10.1016/j.addbeh.2021.106917	No	Not meeting inclusion criteria	Title and abstract screening
4997	Yang JJ, Ou T-S, Lin H-C, Kyung Nam J, Piper ME, Buu A. Retrospective and Real-time Measures of the Quantity of E-cigarette Use: An Ecological Momentary Assessment Study. Nicotine Tob Res Off J Soc Res Nicotine Tob. 2023;25: 1667–1675. doi:10.1093/ntr/ntad094	No	Not meeting inclusion criteria	Title and abstract screening
4998	Yang JH, Strodl E, Wu CA, Yin XN, Wen GM, Sun DL, et al. Association between environmental tobacco smoke exposure in early life and autistic-like behaviors in Chinese preschoolers. J Psychosom Res. 2021;152: 110680. doi:10.1016/j.jpsychores.2021.110680	No	Not meeting inclusion criteria	Title and abstract screening

4999	Yang JJ, Lin HC, Ou TS, Tong Z, Li R, Piper ME, et al. The situational contexts and subjective effects of co-use of electronic cigarettes and alcohol among college students: An ecological momentary assessment (EMA) study. Drug Alcohol Depend. 2022;239: 109594. doi:10.1016/j.drugalcdep.2022.109594	No	Not meeting inclusion criteria	Title and abstract screening
5000	Yang L, Bao Z, Zhang L, Lei X. Position management on pulmonary function and bronchopulmonary dysplasia in premature infants: study protocol for a randomised controlled trial. 2022;12: e062291. doi:10.1136/bmjopen-2022-062291	No	Not meeting inclusion criteria	Title and abstract screening
5001	Yang MJ, Sutton SK, Hernandez LM, Jones SR, Wetter DW, Kumar S, et al. A Just-In-Time Adaptive intervention (JITAI) for smoking cessation: Feasibility and acceptability findings. Addict Behav. 2023;136: 107467. doi:10.1016/j.addbeh.2022.107467	No	Not meeting inclusion criteria	Title and abstract screening
5002	Yang QZC. Time at Home as a Patient-Centered End Point for Surgical Cancer Treatment. JAMA Surg. 2021;156: 794–794. doi:10.1001/jamasurg.2021.0634	No	Not meeting inclusion criteria	Title and abstract screening
5003	Yang S., Nugent R., Nugent K. Likelihood of Adolescents Trying Conventional or Electronic Cigarettes Varies with Their Use and Perception of Harm and Addictiveness of Other Tobacco Products, Including Cigars. South Med J. 2022;115: 665–673. doi:10.14423/SMJ.0000000000001445	No	Not meeting inclusion criteria	Title and abstract screening
5004	Yang XY, Kelly BC, Pawson M, Vuolo M. Vaping in a Time of Pandemics: Risk Perception and Motivations for Electronic Cigarette Use. Nicotine Tob Res. 2022. doi:10.1093/ntr/ntac050	No	Not meeting inclusion criteria	Title and abstract screening
5005	Yang XY, Kelly BC, Pawson M, Vuolo M. Vaping in a Time of Pandemics: Risk Perception and Motivations for Electronic Cigarette Use. Nicotine Tob Res. 2023;25: 237–246. doi:10.1093/ntr/ntac050	No	Not meeting inclusion criteria	Title and abstract screening
5006	Yang Y, Dobalian A, Ward KD. COVID-19 Vaccine Hesitancy and Its Determinants Among Adults with a History of Tobacco or Marijuana Use. J Community Health. 2021;46: 1090–1098. doi:10.1007/s10900-021-00993-2	No	Not meeting inclusion criteria	Title and abstract screening
5007	Yao J, Liang X, Liu Y, Li S, Zheng M. Trends in Incidence and Prognostic Factors of Two Subtypes of Primary Liver Cancers: A Surveillance, Epidemiology, and End Results-Based Population Study. Cancer Control J Moffitt Cancer Cent. 2022;29: 1–14. doi:10.1177/10732748211051548	No	Not meeting inclusion criteria	Title and abstract screening
5008	Yao Y., Liu K., Zhang J., Iso H., Lam T.H. Tobacco smoking and older people amid the COVID-19 pandemic: an elephant in the room. Age Ageing. 2021;50: 1865–1867. doi:10.1093/ageing/afab170	No	Not meeting inclusion criteria	Title and abstract screening
5009	Yapar D, Yapar A, Tokgöz MA, Bilge U. Decision tree-based data mining approach for the evaluation of survival in primary malignant bone tumors: A surveillance, epidemiology and end results database study. J Orthop Surg 10225536. 2023;31: 1–13. doi:10.1177/10225536231189780	No	Not meeting inclusion criteria	Title and abstract screening
5010	Yassine A, El Hage R, El-Hellani A, Salman R, Talih S, Eissenberg T, et al. Did JUUL alter the content of menthol pods in response to US FDA flavour enforcement policy?. Tob Control. 2022;31: s234–s237. doi:10.1136/tc-2022-057506	No	Not meeting inclusion criteria	Title and abstract screening
5011	Ye Z, Ma B, Maitland E, Nicholas S, Wang J, Leng A. Structuring healthcare advance directives: Evidence from Chinese end-of-life cancer patients' treatment preferences. Health Expect. 2023;26: 1648–1657. doi:10.1111/hex.13769	No	Not meeting inclusion criteria	Title and abstract screening
5012	Yerramilli D, Johnstone CA. Radiation Therapy at the End of-Life: Quality of Life and Financial Toxicity Considerations. Semin Radiat Oncol. 2023;33: 203–210. doi:10.1016/j.semradonc.2022.11.002	No	Not meeting inclusion criteria	Title and abstract screening
5013	Yeung C.A. Is the use of tobacco products, especially electronic nicotine delivery systems (ENDS), associated with the incidence of oral health outcomes among US adults? Evid Based Dent. 2023. doi:10.1038/s41432-023-00949-6	No	Not meeting inclusion criteria	Title and abstract screening
5014	Yingst JM, Lester C, Livelsberger C, Allen SI, Hammett E, Veldheer S, et al. Pilot randomized controlled trial evaluating the effect of random nicotine delivery on cigarettes per day and smoke exposure. Brain Res Bull. 2022;188: 30–37. doi:10.1016/j.brainresbull.2022.07.017	No	Not meeting inclusion criteria	Title and abstract screening
5015	Yingst JM, Lester C, Veldheer S, Allen SI, Du P, Foulds J. E-cigarette users commonly stealth vape in places where e-cigarette use is prohibited. Tob Control. 2019;28: 493–497. doi:10.1136/tobaccocontrol-2018-054432	No	Not meeting inclusion criteria	Title and abstract screening
5016	Ylioja T, Cochran G, Chang Y, Tindle HA, Rigotti NA. Postdischarge smoking cessation in subgroups of hospitalized smokers: A latent class analysis. Subst Abuse. 2017;38: 493–497. doi:10.1080/08897077.2017.1355870https://dx.doi.org/10.1080/08897077.2017.1355870	No	Not meeting inclusion criteria	Title and abstract screening
5017	Yong HH, Chow R, East K, Thrasher JF, Hitchman SC, Borland R, et al. Do social norms for cigarette smoking and nicotine vaping product use predict trying nicotine vaping products and attempts to quit cigarette smoking amongst adult smokers? Findings from the 2016-2020 ITC Four Country Smoking and Vaping Surveys. Nicotine Tob Res. 2022. doi:10.1093/ntr/ntac212	No	Not meeting inclusion criteria	Title and abstract screening
5018	Yong HH, Chow R, East K, Thrasher JF, Hitchman SC, Borland R, et al. Do Social Norms for Cigarette Smoking and Nicotine Vaping Product Use Predict Trying Nicotine Vaping Products and Attempts to Quit Cigarette Smoking Amongst Adult Smokers? Findings From the 2016-2020 International Tobacco Control Four Country Smoking and. Nicotine Tob Res. 2023;25: 505–513. doi:10.1093/ntr/ntac212	No	Not meeting inclusion criteria	Title and abstract screening
5019	Yong HH, Gravely S, Borland R, Gartner C, Michael Cummings K, East K, et al. Do Smokers' Perceptions of the Harmfulness of Nicotine Replacement Therapy and Nicotine Vaping Products as Compared to Cigarettes Influence Their Use as an Aid for Smoking Cessation? Findings from the ITC Four Country Smoking and Vaping Surveys. Nicotine Tob Res. 2022;24: 1413–1421. doi:10.1093/ntr/ntac087	No	Not meeting inclusion criteria	Title and abstract screening
5020	Yong HH, Karmakar C, Motin MA, Borland R, Cummings KM, Gravely S, et al. Individual and Conjoint Factors Associated With Beliefs About the Harmfulness of Nicotine Replacement Therapies Relative to Combustible Cigarettes Among People Who Smoke: Findings From the 2020 ITC Four Country Smoking and Vaping Survey. Nicotine Tob Res. 2023;25: 1594–1602. doi:10.1093/ntr/ntad075	No	Not meeting inclusion criteria	Title and abstract screening
5021	Yoon M-J, Cha H, Ahn J, Lee D, Jeong H-S, Koo HS, et al. Dysfunctional activity of classical DNA end-joining renders acquired resistance to carboplatin in human ovarian cancer cells. Cancer Lett. 2021;520: 267–280. doi:10.1016/j.canlet.2021.08.003	No	Not meeting inclusion criteria	Title and abstract screening
5022	Yoon S-H, Song M-K, Kim DI, Lee J-K, Jung J-W, Lee JW, et al. Comparative study of lung toxicity of E-cigarette ingredients to investigate E-cigarette or vaping product associated lung injury. J Hazard Mater. 2023;445: 130454. doi:10.1016/j.jhazmat.2022.130454	No	Not meeting inclusion criteria	Title and abstract screening
5023	Yoshioka S, Katayama H. Actual Situations and Factors Related to Nursing Activities in Supporting the Transition to Homecare Settings for End-Stage Cancer Patients in General Wards in Japan. Am J Hosp Palliat Med. 2021;38: 750–757. doi:10.1177/1049909120959787	No	Not meeting inclusion criteria	Title and abstract screening
5024	Young L.J., Antwi-Boasiako S., Ferrall J., Wold L.E., Mohler P.J., El Refaey M. Genetic and non-genetic risk factors associated with atrial fibrillation. Life Sci. 2022;299: 120529. doi:10.1016/j.lfs.2022.120529	No	Not meeting inclusion criteria	Title and abstract screening
5025	Young WJ, Ganz O, Jeong M, Wackowski OA, Delnevo CD. Perceptions of Game cigarillo packaging among young adult tobacco users: The effect of package color and the "natural leaf" descriptor. Addict Behav. 2022;132: 107334. doi:10.1016/j.addbeh.2022.107334	No	Not meeting inclusion criteria	Title and abstract screening
5026	Young-Wolff KC, Adams SR, Sterling SA, Tan ASL, Salloum RG, Torre K, et al. Nicotine and cannabis vaping among adolescents in treatment for substance use disorders. J Subst Abuse Treat. 2021;125: 108304. doi:10.1016/j.jsat.2021.108304	No	Not meeting inclusion criteria	Title and abstract screening
5027	Youssef M, Marzouk T, Abdelsalam H, Malmstrom H, Barmak AB, Fraser D, et al. The effect of electronic cigarette use on peri-implant conditions in men: a systematic review and meta-analysis. Oral Surg Oral Med Oral Pathol Oral Radiol. 2022. doi:10.1016/j.oooo.2022.08.010	No	Not meeting inclusion criteria	Title and abstract screening

5028	Yousufzai SJ, Cole AG, Nonoyama M, Barakat C. Changes in Cannabis Consumption Among Emerging Adults in Relation to Policy and Public Health Developments. Subst Use Misuse. 2022;57: 730–741. doi:10.1080/10826084.2022.2034873	No	Not meeting inclusion criteria	Title and abstract screening
5029	Yousufzai SJ, Cole AG, Nonoyama M, Barakat C. Changes in Quantity Measures of Various Forms of Cannabis Consumption among Emerging Adults in Canada in Relation to Policy and Public Health Developments. Int J Env Res Public Health. 2023;20. doi:10.3390/ijerph20136213	No	Not meeting inclusion criteria	Title and abstract screening
5030	Yu B, Lin F, Duan J, Ning H. The influence of marital status on survival in patients with nasopharyngeal carcinoma: A surveillance, epidemiology, and end results database analysis. Medicine (Baltimore). 2022;101: e30516–e30516. doi:10.1097/MD.0000000000030516	No	Not meeting inclusion criteria	Title and abstract screening
5031	Yu C., Harb A., Liu J., Sakurai R., Rehan V. MOLECULAR AND PHENOTYPIC CHARACTERIZATION OF THE PERINATAL ELECTRONIC-CIGARETTE EXPOSURE-INDUCED EFFECTS ON BONE MARROW-DERIVED MESENCHYMAL STEM CELLS. J Investig Med. 2023;71: 82. doi:10.1177/10815589221142328	No	Not meeting inclusion criteria	Title and abstract screening
5032	Yu H, Xie S, Zheng X, Zhao Q, Xia X, Ming W-K, et al. Prognosis of the Keratinizing Squamous Cell Carcinoma of the Tongue Based on Surveillance, Epidemiology, and End Results Database. Int J Clin Pract. 2023; 1–11. doi:10.1155/2023/3016994	No	Not meeting inclusion criteria	Title and abstract screening
5033	Yu JB. The Cost of Cancer Care at the End of Life: Implications for Centers for Medicare and Medicaid Services and for Radiation Oncology. Int J Radiat Oncol Biol Phys. 2023;116: 736–738. doi:10.1016/j.ijrobp.2023.02.012	No	Not meeting inclusion criteria	Title and abstract screening
5034	Yu Mi Oh, Yoon Na Kang, Soo Jung Han, Jeong Hye Kim. Decision and Practice of End-of-Life Care in Lung Disease Patients with Physicians Orders for Life Sustaining Treatment. J Hosp Palliat Care. 2023;26: 7–17. doi:10.14475/jhpc.2023.26.1.7	No	Not meeting inclusion criteria	Title and abstract screening
5035	Yu X., Gross E. Acetaldehyde in e-cigarette aerosol and cigarette smoke exacerbate pulmonary microvascular endothelial dysfunction in ALDH2*2 mice. FASEB J. 2021;35. doi:10.1096/fasebj.2021.35.S1.03866	No	Not meeting inclusion criteria	Title and abstract screening
5036	Yu X., Xiao F., Gross E. Acetaldehyde within e-cigarette aerosol and cigarette smoke triggers intracellular calcium release in primary adult cardiomyocytes that is exacerbated in ALDH2*2 mice. FASEB J. 2021;35. doi:10.1096/fasebj.2021.35.S1.03807	No	Not meeting inclusion criteria	Title and abstract screening
5037	Yumeen S, Mirza FN, Mirza HN, Zogg CK, Leventhal JS, Cohen JM. Malignant blue nevus: Characterization of US epidemiology and prognostic factors of a rare neoplasm with aggressive clinical course using the Surveillance, Epidemiology, and End Results Program database. J Am Acad Dermatol. 2023;89: 843–846. doi:10.1016/j.jaad.2023.05.090	No	Not meeting inclusion criteria	Title and abstract screening
5038	Yumrukuz Senel M., Karadogan D., Vardaloglu I., Develi E., Celik S., Hizal M., et al. ERS International Virtual Congress 2021: Highlights from the Turkish Thoracic Society Early Career Members. Turk Thorac J. 2022;23: 173–184. doi:10.5152/TurkThoracJ.2022.21289	No	Not meeting inclusion criteria	Title and abstract screening
5039	YUMRUKUZ SENEL M, DURU AKCALI S, KURT B. Electronic Cigarette-Related Attitudes and Behaviors of Patients Presenting to the Pulmonology Clinic. Balikesir Health Sci J. 2022;11: 242–247. doi:10.53424/balikesirsbd.978358	No	Not meeting inclusion criteria	Title and abstract screening
5040	Yun HW, Jung KJ, Jee SH, Kimm H. Overall quit in triple users of conventional cigarette, e-cigarette and heated tobacco product among healthy adults: a Korea Medical Institute health check-up study. BMJ Open. 2022;12: e051865. doi:10.1136/bmjopen-2021-051865	No	Not meeting inclusion criteria	Title and abstract screening
5041	ZAHID I., BELARDO M., JAM HOOKS B. CANNABIS AS AN EVOLVING CAUSE OF PULMONARY LANGERHANS CELL HISTIOCYTOSIS. Chest. 2023;164: A4782–A4783. doi:10.1016/j.chest.2023.07.3099	No	Not meeting inclusion criteria	Title and abstract screening
5042	Zahirsha Z, Barnes J, Simpson MC, Osazuwa-Peters N. 25553 Suicide risk among melanoma and nonepithelial skin cancer patients: A Surveillance, Epidemiology, and End Results analysis. J Am Acad Dermatol. 2021;85: AB8–AB8. doi:10.1016/j.jaad.2021.06.056	No	Not meeting inclusion criteria	Title and abstract screening
5043	Zarcone G., Lenski M., Martinez T., Garcon G., Allorge D., Nesslany F., et al. P07-04 Comparison of toxicity and transcriptomic effects of emissions from heated tobacco products, electronic cigarettes and tobacco cigarettes in human bronchial epithelial cells. Toxicol Lett. 2021;350: S124–S125. doi:10.1016/S0378-4274%2821%2900540-3	No	Not meeting inclusion criteria	Title and abstract screening
5044	Zavala-Arciniega L, Barrientos-Gutiérrez I, Arillo-Santillán E, Gallegos-Carrillo K, Rodríguez-Bolaños R, F Thrasher J. Profile and patterns of dual use of e-cigarettes and combustible cigarettes among Mexican adults. Salud Publica Mex. 2021;63: 641–652. doi:10.21149/12365	No	Not meeting inclusion criteria	Title and abstract screening
5045	Zavala-Arciniega L, Gutiérrez-Torres DS, Reynales-Shigematsu LM, Barrientos-Gutiérrez I, Fleischer NL, Meza R, et al. [Prevalence, proportion and correlates of flavorcapsule cigarette use in Mexico: results fromthe Ensanut 2018-19]. Salud Publica Mex. 2020;62: 820–828. doi:10.21149/11566	No	Not meeting inclusion criteria	Title and abstract screening
5046	Zavala-Arciniega L, Meza R, Hirschtick JL, Fleischer NL. Disparities in Cigarette, E-cigarette, Cigar, and Smokeless Tobacco Use at the Intersection of Multiple Social Identities in the US Adult Population. Results From the Tobacco Use Supplement to the Current Population Survey 2018-2019 Survey. Nicotine Tob Res. 2023;25: 908–917. doi:10.1093/ntr/ntac261	No	Not meeting inclusion criteria	Title and abstract screening
5047	Zawertailo L, Pavlov D, Ivanova A, Ng G, Baliunas D, Selby P. Concurrent E-Cigarette Use During Tobacco Dependence Treatment in Primary Care Settings: Association With Smoking Cessation at Three and Six Months. Nicotine Tob Res. 2017;19: 183–189. doi:10.1093/ntr/ntw218	No	Not meeting inclusion criteria	Title and abstract screening
5048	Zawertailo L, Voci S, Selby P. The Cigarette Dependence Scale and Heaviness of Smoking Index as predictors of smoking cessation after 10weeks of nicotine replacement therapy and at 6-month follow-up. Addict Behav. 2018;78: 223–227. doi:10.1016/j.addbeh.2017.11.036	No	Not meeting inclusion criteria	Title and abstract screening
5049	Zawertailo L, Kouzoukas E, Fougere C, Dragonetti R, Veldhuizen S, Selby P. Clinical guidance for e-cigarette (vaping) cessation: Results from a modified Delphi panel approach. Prev Med Rep. 2023;35: 102372. doi:10.1016/j.pmedr.2023.102372	No	Not meeting inclusion criteria	Title and abstract screening
5050	Zelicof Paul A, Rutherford KA, Abuso SM. Emergency department management of pediatric acute asthma: an evidence-based review. Pediatr Emerg Med Pr. 2023;20: 1–28.	No	Not meeting inclusion criteria	Title and abstract screening
5051	Zelle SL, Gates KM, Fiez JA, Sayette MA, Wilson SJ. The first day is always the hardest: Functional connectivity during cue exposure and the ability to resist smoking in the initial hours of a quit attempt. Neuroimage. 2017;151: 24–32. doi:10.1016/j.neuroimage.2016.03.015	No	Not meeting inclusion criteria	Title and abstract screening
5052	Zeller M., Sales-Wuillemin E., Guinchard S., Chappe J., Chague F., Ayari H., et al. Psychosocial and behavioral characteristics of still smokers at 6 months after acute cerebro or cardiovascular events: Preliminary findings from INEV@L, a prospective pilot study. Eur Heart J. 2021;42: 2595. doi:10.1093/eurheartj/ehab724.2595	No	Not meeting inclusion criteria	Title and abstract screening
5053	Zemni I, Zemni K, Gara A, Maatouk A, Ben Fredj M, Abroug H, et al. Associated factors with adherence to preventive behaviors related to COVID-19 among medical students in the university of Monastir, Tunisia. PLoS One. 2023;18: e0280921. doi:10.1371/journal.pone.0280921	No	Not meeting inclusion criteria	Title and abstract screening
5054	Zeng Z., Chen W., Crotty Alexander L.E., Jacobson J.R., Dudek S.M., Natarajan V., et al. Endothelial Knockdown of the Tumor Suppressor, WWOX, Increases Severity of Ventilator-Induced Lung Injury. Am J Respir Crit Care Med. 2021;203. doi:10.1164/ajrccm-conference.2021.TP113	No	Not meeting inclusion criteria	Title and abstract screening
5055	Zeng Z, Chen W, Moshensky A, Shakir Z, Khan R, Crotty Alexander LE, et al. Cigarette Smoke and Nicotine-Containing Electronic-Cigarette Vapor Downregulate Lung WWOX Expression, Which Is Associated with Increased Severity of Murine Acute Respiratory Distress Syndrome. Am J Respir Cell Mol Biol. 2021;64: 89–99. doi:10.1165/rcmb.2020-0145OC	No	Not meeting inclusion criteria	Title and abstract screening
5056	Zgliczynski W.S., Jankowski M., Rostkowska O., Gujski M., Wierzba W., Pinkas J. Knowledge and beliefs of e-cigarettes among physicians in Poland. Med Sci Monit. 2019;25: 6322–6330. doi:10.12659/MSM.916920	No	Not meeting inclusion criteria	Title and abstract screening

5057	Zhai D, van Stiphout R, Schiavone G, De Raedt W, Van Hoof C. Characterizing and Modeling Smoking Behavior Using Automatic Smoking Event Detection and Mobile Surveys in Naturalistic Environments: Observational Study. JMIR Mhealth Uhealth. 2022;10: e28159. doi:10.2196/28159	No	Not meeting inclusion criteria	Title and abstract screening
5058	Zhang AC, Rasul R, Golden A, Feuerstein MA. Incidence and mortality trends of metastatic prostate cancer: Surveillance, Epidemiology, and End Results database analysis. Can Urol Assoc J. 2021;15: E637–E643. doi:10.5489/cuaj.7173	No	Not meeting inclusion criteria	Title and abstract screening
5059	Zhang J, Zhang J. Prognostic factors and survival prediction of resected non-small cell lung cancer with ipsilateral pulmonary metastases: a study based on the Surveillance, Epidemiology, and End Results (SEER) database. BMC Pulm Med. 2023;23: 1–11. doi:10.1186/s12890-023-02722-y	No	Not meeting inclusion criteria	Title and abstract screening
5060	Zhang K. Editorial: Application of New Technologies in the Treatment of Substance Use Disorders. Front Psychiatry. 2021;12: 626357. doi:10.3389/fpsyt.2021.626357	No	Not meeting inclusion criteria	Title and abstract screening
5061	Zhang K., Tartarone A., Perez-Rios M., Novello S., Mariniello A., Roviello G., et al. Smoking burden, MPOWER, future tobacco control and real-world challenges in China: reflections on the WHO report on the global tobacco epidemic 2021. Transl Lung Cancer Res. 2022;11: 117–121. doi:10.21037/tlcr-22-27	No	Not meeting inclusion criteria	Title and abstract screening
5062	Zhang L, Gentzke A, Trivers KF, VanFrank B. Tobacco Cessation Behaviors Among U.S. Middle and High School Students, 2020. J Adolesc Health Off Publ Soc Adolesc Med. 2022;70: 147–154. doi:10.1016/j.jadohealth.2021.07.011	No	Not meeting inclusion criteria	Title and abstract screening
5063	Zhang Q, Fan X, Yue Y, Zheng R. Electronic cigarettes: Emerging trends and research hotspots. Tob Induc Dis. 2020;18: 16. doi:10.18332/tid/118719	No	Not meeting inclusion criteria	Title and abstract screening
5064	Zhang Q, Wen C. The risk profile of electronic nicotine delivery systems, compared to traditional cigarettes, on oral disease: a review. Front Public Health. 2023;11: 1146949. doi:10.3389/fpubh.2023.1146949	No	Not meeting inclusion criteria	Title and abstract screening
5065	Zhang X, Sun Y, Cheung DYT, Wang MP, Wu Y, Chak KY, et al. Changes in Tobacco Use in the Early Phase of Coronavirus Disease 2019 Pandemic in Hong Kong: A Qualitative Study. Nicotine Tob Res. 2023;25: 309–317. doi:10.1093/ntr/ntac147	No	Not meeting inclusion criteria	Title and abstract screening
5066	Zhang Y, Archie SR, Ghanwatkar Y, Sharma S, Nozohouri S, Burks E, et al. Potential role of astrocyte angiotensin converting enzyme 2 in the neural transmission of COVID-19 and a neuroinflammatory state induced by smoking and vaping. Fluids Barriers CNS. 2022;19: 46. doi:10.1186/s12987-022-00339-7	No	Not meeting inclusion criteria	Title and abstract screening
5067	Zhang Y, Upson D. E-Cigarettes versus Nicotine-Replacement Therapy for Smoking Cessation. N Engl J Med. 2019;380: 1973. doi:10.1056/NEJMc1903758	No	Not meeting inclusion criteria	Title and abstract screening
5068	Zhang Y., Archie S.R., Ghanwatkar Y., Sharma S., Nozohouri S., Burks E., et al. Correction: Potential role of astrocyte angiotensin converting enzyme 2 in the neural transmission of COVID-19 and a neuroinflammatory state induced by smoking and vaping (Fluids and Barriers of the CNS, (2022), 19, 1, (46), 10.1186/s12987-022-00339-7). Fluids Barriers CNS. 2022;19: 91. doi:10.1186/s12987-022-00388-y	No	Not meeting inclusion criteria	Title and abstract screening
5069	Zhang Y.-Y., Tang W.-T., Zhang H., Wang J., Bai X.-X., Liao Y.-H., et al. Barriers and Facilitators for Smoking Cessation in Chinese Smokers with Chronic Obstructive Pulmonary Disease: A Qualitative Study. Int J COPD. 2022;17: 1107–1120. doi:10.2147/COPD.S356935	No	Not meeting inclusion criteria	Title and abstract screening
5070	Zhang Y-Y, Bu F-L, Dong F, Wang J-H, Zhu S-J, Zhang X-W, et al. The effect of e-cigarettes on smoking cessation and cigarette smoking initiation: An evidence-based rapid review and meta-analysis. Tob Induc Dis. 2021;19: 04. doi:10.18332/tid/131624	No	Not meeting inclusion criteria	Title and abstract screening
5071	Zhang Z., Jiao Z., Blaha M.J., Osei A., Sidhaye V., Ramanathan M., et al. The Association Between E-Cigarette Use and Prediabetes: Results From the Behavioral Risk Factor Surveillance System, 2016-2018. Am J Prev Med. 2022;62: 872–877. doi:10.1016/j.amepre.2021.12.009	No	Not meeting inclusion criteria	Title and abstract screening
5072	Zhao D., Zuo Y., Urmi D.S., Liu Y., Zheng P., Fan W., et al. Perception of e-cigarette use among adult users in china: A mixed-method study. Int J Environ Res Public Health. 2020;17: 2754. doi:10.3390/ijerph17082754	No	Not meeting inclusion criteria	Title and abstract screening
5073	Zhao D, Abdullah AS, Wen T, Chen X, Xiao X, Pan Z, et al. Perceptions of e-cigarettes among smokers and non-smokers in households with children in rural China: A cross-sectional study. Tob Induc Dis. 2021;19: 25. doi:10.18332/tid/133264	No	Not meeting inclusion criteria	Title and abstract screening
5074	Zhao J, Zhang Y, Sisler JD, Shaffer J, Leonard SS, Morris AM, et al. Assessment of reactive oxygen species generated by electronic cigarettes using acellular and cellular approaches. J Hazard Mater. 2018;344: 549–557. doi:10.1016/j.jhazmat.2017.10.057	No	Not meeting inclusion criteria	Title and abstract screening
5075	Zhao K., Li J., Zhou P., Xu L., Yang M. Is electronic cigarette use a risk factor for stroke? A systematic review and meta-analysis. Tob Induc Dis. 2022;20: 101. doi:10.18332/tid/154364	No	Not meeting inclusion criteria	Title and abstract screening
5076	Zhao S, Zhang X, Wang J, Lin J, Cao D, Zhu M. Carcinogenic and non-carcinogenic health risk assessment of organic compounds and heavy metals in electronic cigarettes. Sci Rep. 2023;13: 16046. doi:10.1038/s41598-023-43112-y	No	Not meeting inclusion criteria	Title and abstract screening
5077	Zhong L, Qin L, Ding X, Ma L, Wang Y, Liu M, et al. The regulatory effect of fermented black barley on the gut microbiota and metabolic dysbiosis in mice exposed to cigarette smoke. Food Res Int. 2022;157: 111465. doi:10.1016/j.foodres.2022.111465	No	Not meeting inclusion criteria	Title and abstract screening
5078	Zhou Y, Irshad H, Dye WW, Wu G, Tellez CS, Belinsky SA. Voltage and e-liquid composition affect nicotine deposition within the oral cavity and carbonyl formation. Tob Control. 2021;30: 485–491. doi:10.1136/tobaccocontrol-2020-055619	No	Not meeting inclusion criteria	Title and abstract screening
5079	Zhu D, Zhao G, Wang X. Association of smoking and smoking cessation with overall and cause-specific mortality. Am J Prev Med. 2021;60: 504–512. doi:10.1016/j.amepre.2020.11.003https://dx.doi.org/10.1016/j.amepre.2020.11.003	No	Not meeting inclusion criteria	Title and abstract screening
5080	Zhu H., Lewis D.J. 40792 A national database study on the relationship between electronic cigarette use and common skin cancers. J Am Acad Dermatol. 2023;89: AB111. doi:10.1016/j.jaad.2023.07.448	No	Not meeting inclusion criteria	Title and abstract screening
5081	Zhu J, Wu Y, Xiao W, Li Y. Survival Predictors of Resectable Gallbladder Carcinoma: An Analysis of the Surveillance, Epidemiology, and End Results Database. Am Surg. 2023;89: 1629–1637. doi:10.1177/00031348221074238	No	Not meeting inclusion criteria	Title and abstract screening
5082	Zhu M, Rogers NG, Jahad JV, Herman MA. Sex Differences in the Impact of Electronic Nicotine Vapor on Corticotropin-Releasing Factor Receptor 1 Neurons in the Mouse Ventral Tegmental Area. J Neurosci Off J Soc Neurosci. 2023;43: 3081–3093. doi:10.1523/JNEUROSCI.2087-22.2023	No	Not meeting inclusion criteria	Title and abstract screening
5083	Zhu SH, Ong J, Wong S, Cole A, Zhuang YL, Shi Y. Early adoption of heated tobacco products resembles that of e-cigarettes. Tob Control. 2022;31: e35–e40. doi:10.1136/tobaccocontrol-2020-056089	No	Not meeting inclusion criteria	Title and abstract screening
5084	Zhu SH, Zhuang YL, Wong S, Cummins SE, Tedeschi GJ. E-cigarette use and associated changes in population smoking cessation: evidence from US current population surveys. BMJ. 2017;358: j3262. doi:10.1136/bmj.j3262	No	Not meeting inclusion criteria	Title and abstract screening
5085	ZHU Yalin, CHAI Shouxia, LIU Yikui, WU Lun. Research progress on end-of-life communication in terminal cancer patients. Chin Nurs Res. 2023;37: 98–104. doi:10.12102/j.issn.1009-6493.2023.01.016	No	Not meeting inclusion criteria	Title and abstract screening

5086	Zickgraf HF, Barrada JR. Orthorexia nervosa vs. healthy orthorexia: relationships with disordered eating, eating behavior, and healthy lifestyle choices. Eat Weight Disord. 2022;27: 1313–1325. doi:10.1007/s40519-021-01263-9	No	Not meeting inclusion criteria	Title and abstract screening
5087	Ziedonis D, Das S, Larkin C. Tobacco use disorder and treatment: new challenges and opportunities. Dialogues Clin Neurosci. 2017;19: 271–280.	No	Not meeting inclusion criteria	Title and abstract screening
5088	Ziegler A, Korzun T, Leclerc AA, Gillespie AI. Cannabis Consumption Among Patients With a Voice Disorder: A Multicenter E-Survey Study. J Voice. 2021. doi:10.1016/j.jvoice.2021.02.012	No	Not meeting inclusion criteria	Title and abstract screening
5089	Ziegler A, Korzun T, Leclerc AA, Gillespie AI. Cannabis Consumption Among Patients With a Voice Disorder: A Multicenter E-Survey Study. J Voice. 2023;37: 467.e9-467.e18. doi:10.1016/j.jvoice.2021.02.012	No	Not meeting inclusion criteria	Title and abstract screening
5090	Zielonka TM. A debate: Can we recommend electronic cigarettes to our patients? Opinion 1. Adv Respir Med. 2017;85: 35–39. doi:10.5603/ARM.2017.0007	No	Not meeting inclusion criteria	Title and abstract screening
5091	Zins Z., Troyer M. A CHRONIC CAUSE OF ACUTE CHEST PAIN. J Gen Intern Med. 2023;38: S385–S386. doi:10.1007/s11606-023-08226-z	No	Not meeting inclusion criteria	Title and abstract screening
5092	Znyk M, Wężyk-Caba I, Kaleta D. The Frequency of Tobacco Smoking and E-Cigarettes Use among Primary Health Care Patients-The Association between Anti-Tobacco Interventions and Smoking in Poland. Int J Env Res Public Health. 2022;19. doi:10.3390/ijerph191811584	No	Not meeting inclusion criteria	Title and abstract screening
5093	Znyk M, Jurewicz J, Kaleta D. Exposure to Heated Tobacco Products and Adverse Health Effects, a Systematic Review. Int J Environ Res Public Health. 2021;18. doi:10.3390/ijerph18126651	No	Not meeting inclusion criteria	Title and abstract screening
5094	Zulfiqar H, Rahman O. Vaping Associated Pulmonary Injury. 2022.	No	Not meeting inclusion criteria	Title and abstract screening
5095	Zulfiqar H, Sankari A, Rahman O. Vaping-Associated Pulmonary Injury. 2023.	No	Not meeting inclusion criteria	Title and abstract screening
5096	Zuo N, Ma L, Liu T, Hu W, Luo Y, Meng H, et al. Human papillomavirus associated XPF deficiency increases alternative end joining and cisplatin sensitivity in head and neck squamous cell carcinoma. Oral Oncol. 2023;140: N.PAG-N.PAG. doi:10.1016/j.oraloncology.2023.106367	No	Not meeting inclusion criteria	Title and abstract screening
5097	Zuo Y, Garg PK, Nazih R, Garg S, Rose JE, Murugesan T, et al. A programmable smoke delivery device for PET imaging with cigarettes containing (11)C-nicotine. J Neurosci Methods. 2017;283: 55–61. doi:10.1016/j.jneumeth.2017.03.016	No	Not meeting inclusion criteria	Title and abstract screening
5098	Zvolensky MJ, Manning K, Garey L, Mayorga NA, Peraza N. Fatigue severity and electronic cigarette beliefs and use behavior. Addict Behav. 2019;97: 1–6. doi:10.1016/j.addbeh.2019.05.014	No	Not meeting inclusion criteria	Title and abstract screening
5099	Zvolensky MJ, D'Souza J, Garey L, Alfano CA, Mayorga NA, Peraza N, et al. Subjective sleep quality and electronic cigarette dependence, perceived risks of use, and perceptions about quitting electronic cigarettes. Addict Behav. 2020;102: 106199. doi:10.1016/j.addbeh.2019.106199	No	Not meeting inclusion criteria	Title and abstract screening
5100	Zvolensky MJ, Garey L, Mayorga NA, Peraza N, Rogers AH, Ditre JW, et al. Pain Severity and Anxiety Sensitivity Interplay Among Exclusive and Dual Electronic Cigarette Users. J Stud Alcohol Drugs. 2019;80: 211–219.	No	Not meeting inclusion criteria	Title and abstract screening
5101	Zvolensky MJ, Manning K, Garey L, Alfano CA, Mayorga NA, Peraza N. Emotion dysregulation, fatigue, and electronic cigarette expectancies. Cogn Behav Ther. 2021;50: 138–153. doi:10.1080/16506073.2020.1819868	No	Not meeting inclusion criteria	Title and abstract screening
5102	Zvolensky MJ, Rodríguez-Cano R, Paulus DJ, Kotov R, Bromet E, Gonzalez A, et al. Respiratory problems and anxiety sensitivity in smoking lapse among treatment seeking smokers. Addict Behav. 2017;75: 25–29. doi:10.1016/j.addbeh.2017.06.015	No	Not meeting inclusion criteria	Title and abstract screening
5103	Zvolensky MJ, Shepherd JM, Garey L, Case K, Gallagher MW. The influence of neuroticism in terms of E-cigarette dependence and beliefs about use and quitting among dual users of combustible and electronic cigarettes. Addict Behav. 2020;107: 106396. doi:10.1016/j.addbeh.2020.106396	No	Not meeting inclusion criteria	Title and abstract screening
5104	Zvorsky I, Nighbor TD, Kurti AN, DeSarno M, Naudé G, Reed DD, et al. Sensitivity of hypothetical purchase task indices when studying substance use: A systematic literature review. Prev Med. 2019;128: 105789. doi:10.1016/j.ypmed.2019.105789	No	Not meeting inclusion criteria	Title and abstract screening
5105	Zwar N.A. Smoking cessation. Aust J Gen Pract. 2020;49: 474–481. doi:10.31128/AJGP-03-20-5287	No	Not meeting inclusion criteria	Title and abstract screening
5106	Zwar NA. Smoking cessation. Aust J Gen Pract. 2020;49: 474–481. doi:10.31128/AJGP-03-20-5287	No	Not meeting inclusion criteria	Title and abstract screening
5107	E-cigarettes Less Addictive but Not Harmless. AARC Times. 2017;41: 52–52.	No	Not meeting inclusion criteria	Title and abstract screening
5108	Erratum...Cahn Z., Berg C. J. Commentary on Seyla et al. (2018): Advantages in the consideration of causal mechanisms for studies of gateway e-cigarette use. Addiction 2018; 113: 334-5. Addiction. 2018;113: 1367–1367. doi:10.1111/add.14253	No	Not meeting inclusion criteria	Title and abstract screening
5109	Drugs for smoking cessation. Med Lett Drugs Ther. 2019;61: 105–110.	No	Not meeting inclusion criteria	Title and abstract screening
5110	E-cigarettes Best Other Cessation Tools. 2019;9: OF3-. doi:10.1158/2159-8290.CD-NB2019-018	No	Not meeting inclusion criteria	Title and abstract screening
5111	American Academy of Hospice and Palliative Medicine State of the Science in Hospice and Palliative Care. 2020;60: A1-A12. doi:10.1016/s0885-3924(20)30399-7	No	Not meeting inclusion criteria	Title and abstract screening
5112	A Reminder to Doctors to Bring Up End-of-Life Issues With Their Dying Cancer Patients. J Med Pract Manage. 2021;37: 51–52.	No	Not meeting inclusion criteria	Title and abstract screening
5113	Help End Children's Cancers. Parents. 2021;96: 22–22.	No	Not meeting inclusion criteria	Title and abstract screening
5114	P1-50: Radiological findings of EVALI: A systematic review. Respirology. 2021;26 Suppl 3: 92. doi:10.1111/resp.14150_50	No	Not meeting inclusion criteria	Title and abstract screening

5115	5. Facilitating Behavior Change and Well-being to Improve Health Outcomes: Standards of Medical Care in Diabetes-2022. Diabetes Care. 2022;45: S60–S82. doi:10.2337/dc22-S005	No	Not meeting inclusion criteria	Title and abstract screening
5116	Commentary on Loud et al.: Reconsidering nicotine dependence in adults...Loud EE, Duong HT, Henderson KC, et al. Addicted to smoking or addicted to nicotine? A focus group study on perceptions of nicotine and addiction among US adult current smokers, form. Addiction. 2022;117: 482–483. doi:10.1111/add.15701	No	Not meeting inclusion criteria	Title and abstract screening
5117	Erratum: Adjuvant therapy fails to show survival benefit for patients with spindle cell carcinoma: Evidence from the surveillance, epidemiology, and end results database. J Cancer Res Ther. 2022;18: 1450–1450. doi:10.4103/0973-1482.357038	No	Not meeting inclusion criteria	Title and abstract screening
5118	Errors in Author Contributions and Abstract...Lewis-Thames MW, Langston ME, Khan S, et al. Racial and ethnic differences in rural-urban trends in 5-year survival of patients with lung, prostate, breast, and colorectal cancers: 1975-2011 Surveillance, Epid. JAMA Netw Open. 2022;5: e2221167–e2221167. doi:10.1001/jamanetworkopen.2022.21167	No	Not meeting inclusion criteria	Title and abstract screening
5119	Why we need to end the stigma around cancer. Cancer Nurs Pract. 2022;21: 5–5. doi:10.7748/cnp.21.4.5.s1	No	Not meeting inclusion criteria	Title and abstract screening
5120	Correction to: The Association of E-cigarette Flavors With Satisfaction, Enjoyment, and Trying to Quit or Stay Abstinent From Smoking Among Regular Adult Vapers From Canada and the United States: Findings From the 2018 ITC Four Country Smoking and Vaping. Nicotine Tob Res. 2023;25: 1062. doi:10.1093/ntr/ntac273	No	Not meeting inclusion criteria	Title and abstract screening
5121	Error in Discussion...Wen X, Xia T, Li R, et al. Trends in electronic cigarette use among US adults with a history of cardiovascular disease. JAMA Network Open. 2023;6(8):e2328962. JAMA Netw Open. 2023;6: e2334742–e2334742. doi:10.1001/jamanetworkopen.2023.34742	No	Not meeting inclusion criteria	Title and abstract screening
5122	Improving End-of-Life Conversations for People With Cancer. Oncol Times. 2023;45: 19–19. doi:10.1097/01.COT.0000935932.75074.1d	No	Not meeting inclusion criteria	Title and abstract screening
5123	Older Patients of Color Less Likely to Receive Opioids for Cancer Pain at End of Life. Oncol Pharm. 2023;16: 7–7.	No	Not meeting inclusion criteria	Title and abstract screening
5124	ONCOLOGY SELF-ASSESSMENT: PAIN AND SYMPTOM MANAGEMENT, AND PALLIATIVE AND END-OF-LIFE CARE. ASCO Connect. 2023; 36–36.	No	Not meeting inclusion criteria	Title and abstract screening
5125	THE GOOD NEWS ABOUT CANCER. Mens Health. 2023;38: 70–77.	No	Not meeting inclusion criteria	Title and abstract screening

© 2025 Kundu A. et al.
